# Identification of differential gene expression related to reproduction in the sporophytes of *Saccharina japonica*

**DOI:** 10.3389/fpls.2024.1417582

**Published:** 2024-08-06

**Authors:** Toshiki Uji, Takuya Kandori, Hiroyuki Mizuta

**Affiliations:** Laboratory of Aquaculture Genetics and Genomics, Division of Marine Life Science, Faculty of Fisheries Sciences, Hokkaido University, Hakodate, Japan

**Keywords:** brown algae, gene expression, reproduction, *Saccharina japonica*, sorus

## Abstract

*Saccharina japonica*, a significant brown macroalga in the Pacific Ocean, serves as a food source and industrial material. In aquaculture, collecting mature sporophytes for seedling production is essential but challenging due to environmental changes. In this study, transcriptomic analysis of vegetative and sorus tissues was done to identify differentially expressed genes (DEGs) and enhance our understanding of sorus formation regulation in *S. japonica*. KEGG pathway and Gene Otology (GO) analysis revealed that upregulated DEGs were involved in folate biosynthesis, riboflavin metabolism, and amino acid biosynthesis. In addition, the upregulation of genes associated with cell wall remodeling, such as mannuronan C-5-epimerases, vanadium-dependent haloperoxidases, and NADPH oxidase, was observed in sorus parts. Meanwhile, downregulated DEGs in sorus portions included genes related to chloroplast function. These findings will help us understand the regulatory mechanisms behind sorus formation in *S. japonica* and extracellular matrix remodeling in brown algae.

## 1 Introduction

*Saccharina japonica*, an ecologically significant brown macroalga, is a major component of productive beds on the northwest coast of the Pacific Ocean (Xu et al., 2015). It is one of the most economically important seaweeds in aquaculture, extensively utilized as a food source and raw industrial material (Tseng, 2001). In Japan, *S. japonica* is an essential ingredient for making dashi (soup stock) as “kombu” in Japanese cuisine.

In *S. japonica* aquaculture, high-quality wild mature sporophytes are collected from the periphery of aquaculture sites in autumn to produce seedlings. However, due to recent marine environment changes, it has become increasingly difficult to collect mature *S. japonica* sporophytes that release active zoospores (Yotsukura et al., 2022). Certain hybrid cultivars of *S. japonica*, despite having excellent agronomic traits, also exhibit late maturation (Liu et al., 2023). This hinders the hybrid cultivars from producing spores at the time of seedling production, limiting their practical application. Thus, understanding sporogenesis regulation is crucial for sustainable aquaculture in *S. japonica*.

Previous studies investigated the physiological alterations during sorus formation, such as decreased photosynthesis and increased respiration (Nimura and Mizuta, 2001). An exogenous application of abscisic acid (ABA) to sporophyte disks of *S. japonica* promoted sorus formation, and the amount of ABA in sorus tissue was significantly increased compared to that in vegetative tissue (Nimura and Mizuta, 2002). Sorus also accumulates high levels of phenolics, silicon, and iodoperoxidase (IPO) activity compared to its vegetative parts to increase its defensive capacity (Mizuta and Yasui, 2010; Mizuta and Yasui, 2012) (See review (Uji and Mizuta, 2022)). In addition to physiological studies, systematic analysis identified the genes encoding enzymes for the biosynthesis of cell wall carbohydrates (including alginate, fucoidan, and cellulose) and cytoplasm storage carbohydrates (mannitol, laminarin, and trehalose) during the sporophyte development (Zhang et al., 2020). However, molecular biological findings that complement physiological findings in the sporogenesis of *S. japonica* are still lacking.

In this study, a comparative transcriptomic analysis was performed on vegetative and sorus tissues of *S. japonica* sporophytes to investigate the differentially expressed genes (DEGs) during reproduction. The findings of this study will enhance our understanding of the regulatory mechanisms, especially cell wall remodeling involved in sorus formation of *S. japonica*.

## 2 Materials and methods

### 2.1 Algal materials

Mature *S. japonica* sporophytes cultured in a farming area off the coast of Minamikayabe, Hakodate City, Hokkaido, Japan, were collected in October 2023 and transported in a cool box with refrigerants to our laboratory. After 1 h, the disks (3 cm in diameter) formed sorus (mature) and nonsorus portions that did not contain meristematic regions (vegetative), which were cut by a cork borer from the marginal parts sited at 100–200 cm from the stipe-blade transition along each sporophyte (~3 m long). The release of the zoospores from sorus portions was confirmed. The disks were wiped with a paper towel and washed with sterilized seawater to remove the attached organisms. The disks were cultured in 800 mL of sterile vitamin-free Provasoli’s enriched seawater (Provasoli, 1968) at 10°C under 10–20 μmol photons m^−2^ s^−1^ (12 h light/12 h dark cycle) to reduce the effect of dissection on gene expression. After 1 week, the disks were harvested at 10:00 am, immediately frozen with liquid nitrogen, and stored at −80°C until RNA extraction.

### 2.2 RNA extraction

Total RNA extraction was conducted using a combination of a CTAB-based method and the RNeasy Plant Mini Kit (Qiagen, Hilden, Germany), following the protocol by (Heinrich et al., 2012) with minor modifications. Frozen sporophytes were ground in liquid nitrogen with a mortar and pestle and were transferred to tubes. Then, 1 ml of extraction buffer (composed of 2% CTAB, 1 M NaCl, 100 mM Tris pH 8, 50 mM EDTA, pH 8, 3% Polyvinylpolypyrrolidone) and 40 µl of 1M DTT were added and thoroughly mixed. The resulting mixture was incubated at 45°C for 10 mins. Then, one volume of chloroform: isoamylalcohol (24:1) was vigorously mixed for 10 mins. The tubes were centrifuged for 20 min at 20°C and 12,000 g. Subsequently, 600 µl of the aqueous phase was carefully transferred into a new tube. An additional step involved the gentle addition and mixing of 0.3 volumes of 100% EtOH by inverting the tube. A second chloroform extraction was performed by adding one volume of chloroform: isoamylalcohol (24:1). Following centrifugation, 500 µl of the resulting supernatant was transferred to a new tube. Total RNA extraction was accomplished using an RNeasy Plant Mini Kit, following the manufacturer’s instructions. The extracted RNA was purified using a TURBO DNA-free kit (Invitrogen/Life Technologies, Carlsbad, CA) to obtain DNA-free RNA. RNA samples’ quantity and integrity were assessed using a NanoDrop™ 2000 Spectrophotometer (Thermo Fisher Scientific, Waltham, MA) and an Agilent 2100 Bioanalyzer (Agilent Technologies, Santa Clara, CA).

### 2.3 RNA sequencing analysis

Six libraries of complementary DNA (two conditions: vegetative and mature × three replicates) for *S. japonica* were constructed and subsequently sequenced using an Illumina NovaSeq 6000 instrument at Rhelixa Inc. The obtained reads were trimmed for low-quality reads and adapter sequences using fastp (Chen et al., 2018). After trimming, STAR (Dobin et al., 2013) was used to map high-quality reads to an in-house gene model of *S. japonica*, which was constructed with a reference genome (ASM882872v1) using BRAKER 2.1.6 (Brůna et al., 2021). The normalized expression of each gene was calculated using RSEM (Li and Dewey, 2011) as transcripts per million. DEGs between vegetative and mature were identified using edgeR (Robinson et al., 2010) on the false discovery rate significance score < 0.05 and |log_2_ fold change| > 1.

### 2.4 Functional annotation of RNA-Seq data

The obtained DEGs were annotated as follows. First, gene coding regions of *S. japonica* were inferred by Braker (Brůna et al., 2021) to use the genome sequences for functional annotation. The eukaryotic protein database OrthoDB v10 (Kriventseva et al., 2019; Zdobnov et al., 2021) was used to predict *S. japonica* gene coding regions. The protein sequences of the obtained gene coding regions were functionally annotated using EnTAP (Hart et al., 2020) and InterProScan (Jones et al., 2014).

To assess the biological significance of the DEGs, Gene Ontology and Kyoto Encyclopedia of Genes and Genomes (KEGG) pathway enrichment analyses were conducted. GO terms were assigned to all genes using eggNOG-mapper v2 online (http://eggnog-mapper.embl.de/) (Huerta-Cepas et al., 2019; Cantalapiedra et al., 2021) with the default parameters, except that the min_hit_e-value was set to 0.05. The topGO (Alexa and Rahnenfuhrer, 2023) R package was used for GO enrichment analysis, and GO terms with P < 0.05 were considered significantly enriched in the DEGs. A KOBAS 3.0 software (Bu et al., 2021) was used for KEGG annotation and enrichment analysis based on the KEGG PATHWAY database (https://www.genome.jp/kegg/pathway.html) (Kanehisa and Goto, 2000). Pathways with a corrected p-value (q-value) < 0.05 were defined as significantly enriched pathways for DEGs.

### 2.5 Quantitative PCR

First-strand cDNA was synthesized from 0.5 µg of total RNA (same RNA used for RNA-seq) using a PrimeScript II 1st strand cDNA Synthesis Kit (TaKaRa Bio, Shiga, Japan). The cDNA was diluted 10-fold for qPCR analysis, and 1.0 µl of the diluted cDNA was used as a template in a 20 μL reaction volume using KOD SYBR^®^ qPCR Mix (TOYOBO, Osaka, Japan), following the manufacturer’s instructions. Real-time PCR was performed with a LightCycler^®^ 96 System (Roche Diagnostics, Basel, Switzerland) under the following conditions: 2 min at 98°C followed by 40 cycles of 10 s at 98°C, 10 s at 55°C, and 30 s at 68°C. The mRNA levels were calculated using the 2^−△△Ct^ method and normalized to levels of the 18S ribosomal RNA gene. The relative expression level was calculated as a ratio of the mRNA level to the transcription level of vegetative samples. All experiments were performed in triplicate. **Supplementary Table S1** lists the primers used in this study.

## 3 Results and discussion

### 3.1 Identification of DEGs related to reproduction

Using RNA-seq, the transcripts in *S. japonica* were compared between sorus (mature) and nonsorus portions (vegetative) to identify candidate genes regulating sorus formation. Raw data generated by sequencing ranged from 18.0–24.2 million reads per sample. After filtering, 17.6–23.9 million clean reads were obtained, and the mapping rate was 76.9–86.0%. A summary of the obtained RNA sequencing datasets and mapping rates of clean reads is shown in **Table 1**.

**Table 1:** Summary of transcriptome analysis in *Saccharina japonica*.

Sample name	Raw reads	Clean reads	GC content (%)	Mapping rate (%)
Vegetative-1	20,894,576	20,601,412	53.8	86.0
Vegetative-2	18,062,424	17,633,548	54.4	85.0
Vegeattive-3	19,272,408	18,832,922	54.8	84.4
Mature-1	22,475,716	22,086,784	54.9	84.8
Mature-2	24,258,320	23,923,762	55.8	83.0
Mature-3	23,512,470	23,204,488	55.4	76.9

**Figure 1:**
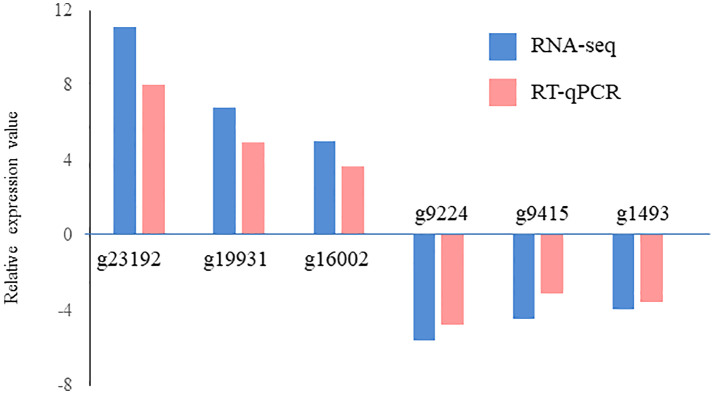
Validation of RNA sequencing (RNA-seq) data using reverse transcription-quantitative polymerase chain reaction (RT-qPCR). Six representative genes were selected to validate the RNA-seq data by RT-qPCR. The pink bars represent the mean log2-fold change obtained by RT-qPCR, and the blue bars represent the RNA-seq data. Results are presented as relative expression compared to that in vegetative tissues. The primers used for RT-qPCR are listed in **Supplementary Table S1**.

A total of 629 DEGs were obtained between mature and vegetative, including 354 upregulated and 275 downregulated genes in sorus portions (**Supplementary Table S2**). Six DEGs (three each for upregulated and downregulated genes) were selected for qPCR analysis to validate the accuracy of the RNA-seq data. As shown in **Figure 1**, expression levels of the selected genes were similar in the RT-qPCR and RNA-seq analyses, indicating that the RNA-seq results were reliable. Shown in **Tables 2**, **3** are the representative genes found to be differentially expressed in mature sporophytes.

**Table 2:** Selected upregulated genes with possible roles in the sporogenesis of *Saccharina japonica*.

Contig ID	Functional categories	Description	Fold Change
g14012	remodeling of alginates	mannuronan C-5-epimerase	11.9
g26842	extracellular matrix	spondin domain-containing protein	10.5
g1659	iodide oxidation	vanadium-dependent iodoperoxidase	9.51
g3247	oxidative burst	respiratory burst oxidase	6.43
g17300	cell wall integrity	WSC domain-containing protein	5.36
g10745	antioxidant activity	superoxide dismutase	4.46
g277	bromide oxidation	vanadium-dependent bromoperoxidase	3.54
g11685	lipid oxidation	lipoxygenase	1.92

**Table 3:** Selected downregulated genes with possible roles in the sporogenesis of *Saccharina japonica*.

Contig ID	Functional categories	Description	Fold Change
g113	photosystem I assembly	Tab2	9.58
g26812	chloroplast inner membrane	Tic20	3.08
g3799	plastoglobules	fibrillin family protein	2.42
g25037	photosynthesis	light harvesting complex protein	2.42
g25030	photosynthesis	light harvesting complex protein	2.29

### 3.2 KEGG enrichment and GO analysis

**Figure 2:**
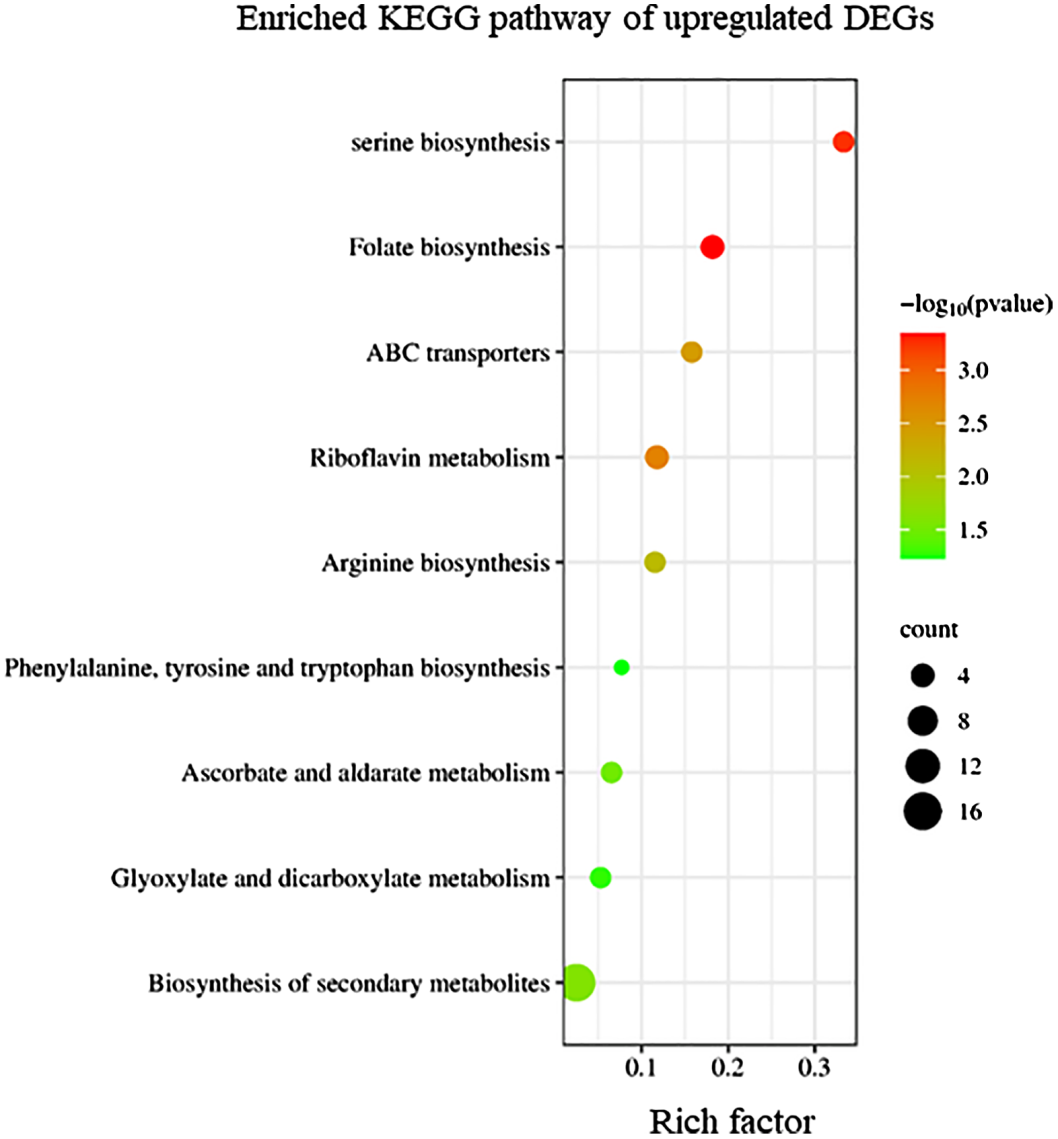
Kyoto Encyclopedia of Genes and Genomes (KEGG) enrichment analysis of the upregulated differentially expressed genes (DEGs). The degree of enrichment increased as the rich factor increased. Larger dots indicate higher numbers of differential genes enriched by the pathway.

KEGG enrichment analysis indicated that upregulated DEGs in sorus portions could be categorized into several pathways, including folate biosynthesis; riboflavin metabolism; and amino acid biosynthesis, such as serine (**Figure 2**). Folates are indispensable components of metabolism in all living organisms; they play as donors and acceptors of one-carbon groups in one-carbon transfer reactions that participate in the formation of numerous important biomolecules, such as nucleic acids, pantothenate (vitamin B5), and amino acids (Gorelova et al., 2017). The strong antioxidant properties of folates can be considered a key factor in elucidating their role in enhancing plant tolerance to diverse abiotic stresses and preventing oxidative damage (Alsamadany et al., 2022). Folates also regulate cellular and molecular events that affect plant growth and development, including cell division (Gorelova et al., 2017). In folate metabolism, serine is crucial for the regulation of methyl group transfer by providing tetrahydrofolate metabolism with C1 units (Ros et al., 2014). Previous studies suggest that serine is involved in the biosynthesis of several biomolecules required for cell proliferation (Ros et al., 2014). Riboflavin (vitamin B2) is a vital component required for fundamental metabolism and a precursor of the coenzymes, FAD and FMN (Jiadkong et al., 2024). Riboflavin metabolism includes antioxidant activity, cell signaling, and coenzyme function (Jiadkong et al., 2024). During sorus formation, upregulation of folate and amino acid biosynthesis, and riboflavin metabolism may play an important role in cell proliferation for sporulation and antioxidant system for reactive oxygen species (ROS) homeostasis.

**Figure 3:**
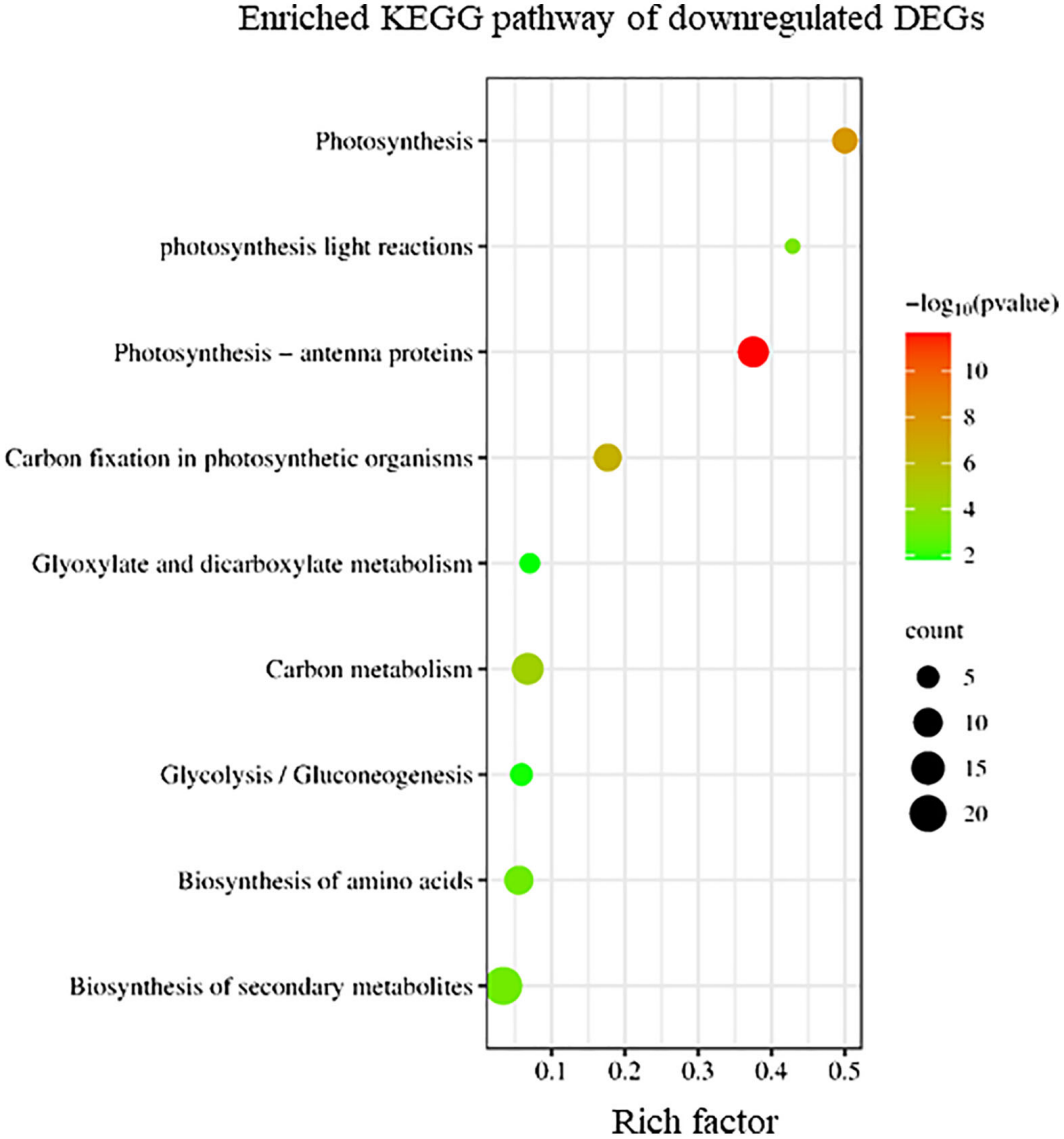
Kyoto Encyclopedia of Genes and Genomes (KEGG) enrichment analysis of the downregulated differentially expressed genes (DEGs). The degree of enrichment increased as the rich factor increased. Larger dots indicate higher numbers of differential genes enriched by the pathway.

Conversely, KEGG enrichment analysis revealed that downregulated DEGs in sorus portions were categorized into pathways, including photosynthesis-antenna proteins, carbon fixation, and secondary metabolite biosynthesis (**Figure 3**).

**Figure 4:**
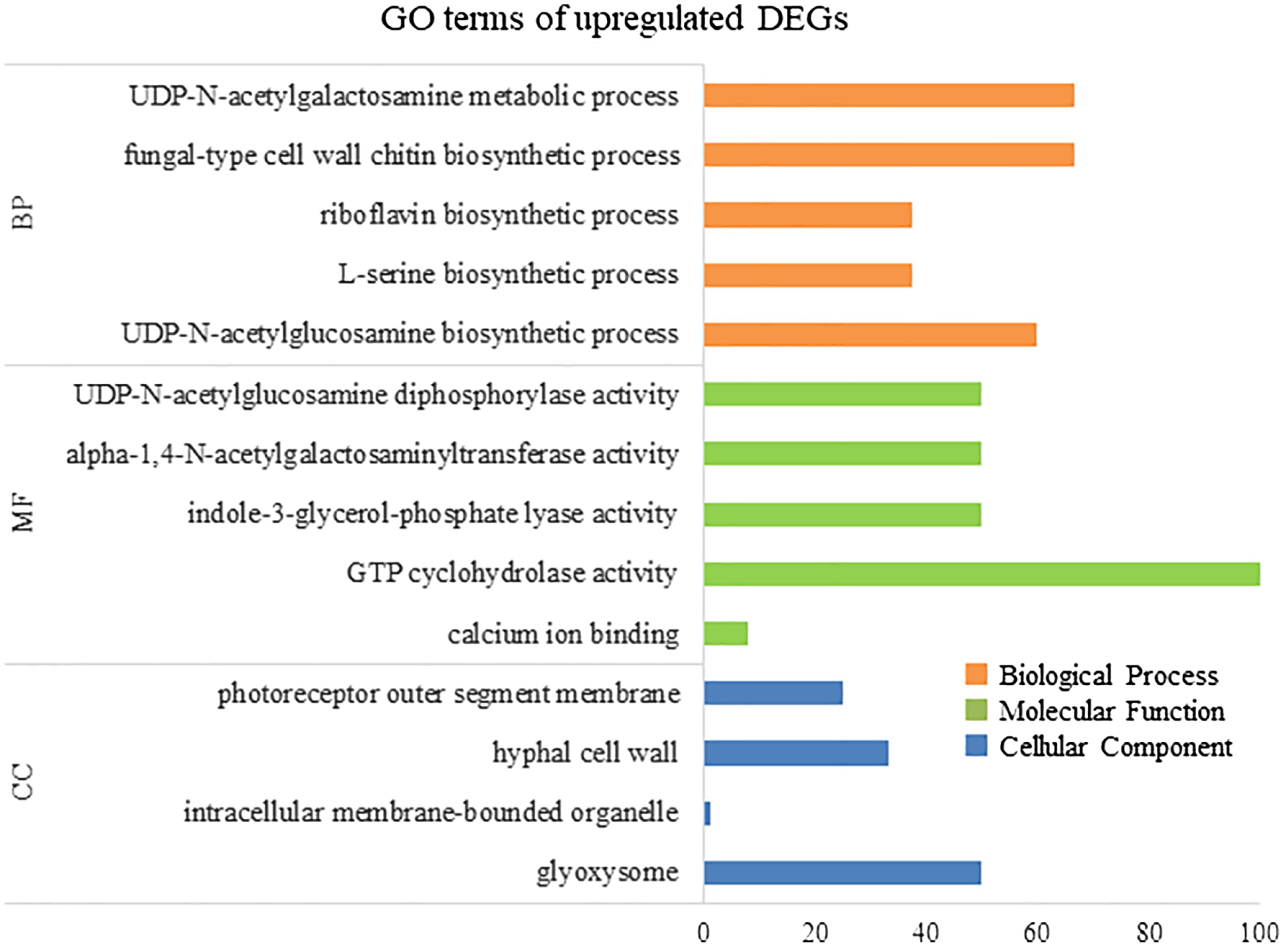
Numbers of enriched Gene Otology (GO) terms for upregulated differentially expressed genes (DEGs). GO terms are presented for three main categories: biological processes, molecular functions, and cellular components. Each GO term is listed in ascending order of p-value.

**Figure 5:**
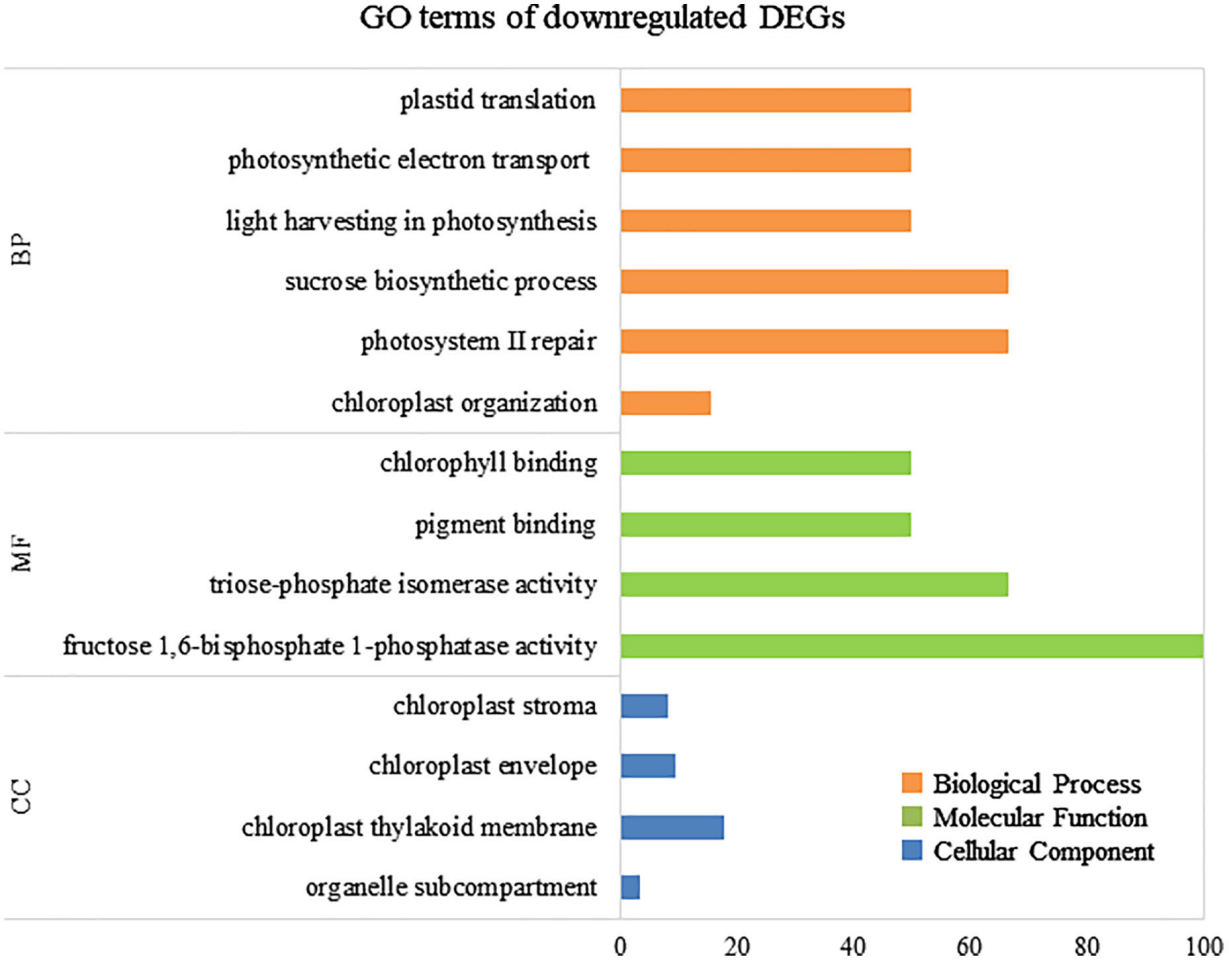
Numbers of enriched Gene Otology (GO) terms for the downregulated differentially expressed genes (DEGs). GO terms are presented for three main categories: biological processes, molecular functions, and cellular components. Each GO term is listed in ascending order of p-value.

To elucidate the biological processes, molecular functions, and cellular components associated with DEGs, GO analysis was performed using eggNOG-mapper and topGO. In upregulated DEGs, GO terms related to serine biosynthesis, riboflavin biosynthesis, and cell wall metabolism were enriched (**Figure 4**). In downregulated DEGs, GO terms related to photosynthesis and chloroplast components were enriched (**Figure 5**). Consequently, KEGG enrichment analysis and GO analysis revealed that upregulated genes were associated with vitamin and amino acid biosynthesis and cell wall metabolism. In contrast, photosynthesis-related genes were downregulated.

### 3.3 Upregulation of ECM remodeling

**Figure 6:**
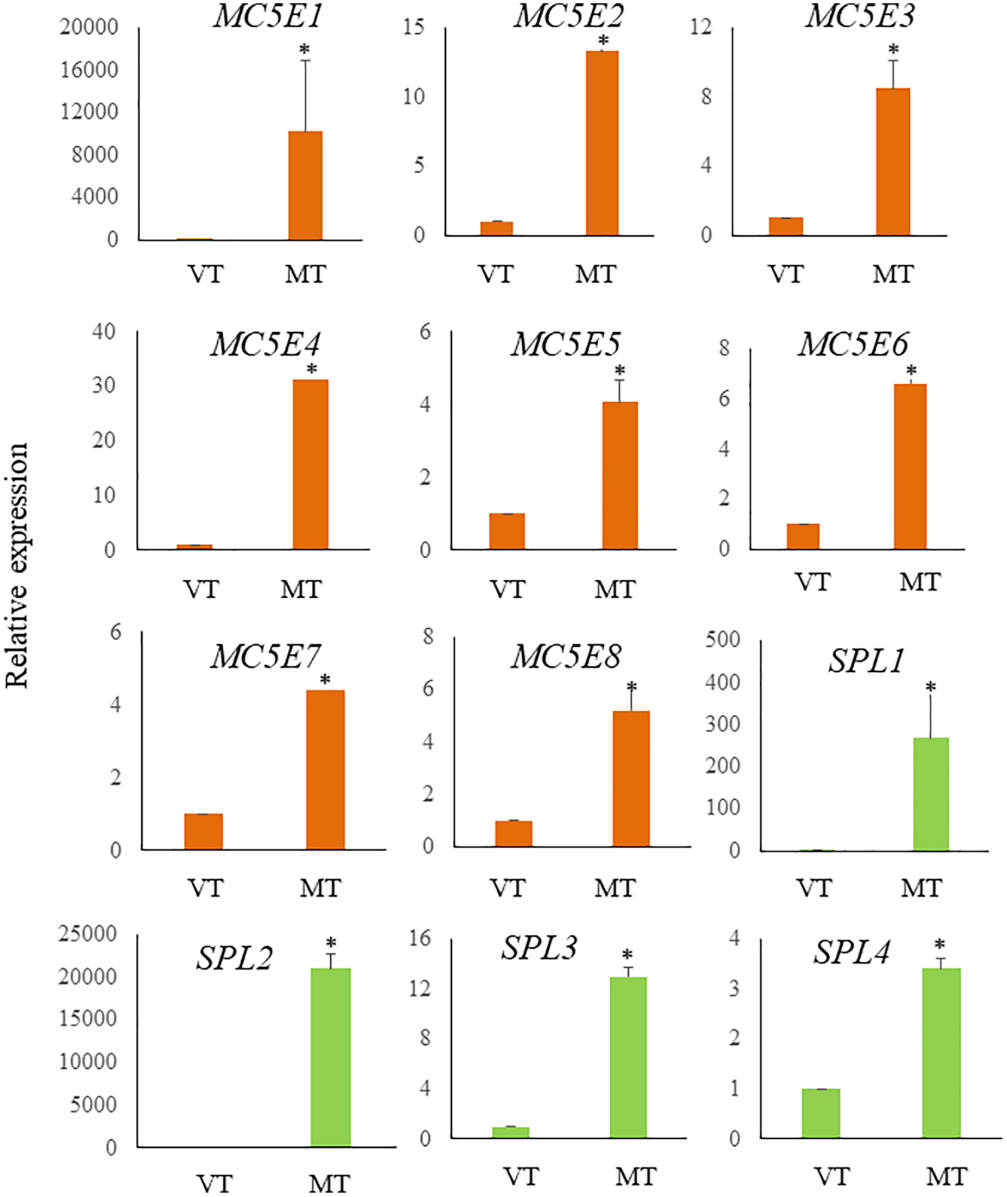
Relative expression levels of ECM related genes in *S. japonica* during sorus formation. RNA samples were prepared from vegetative tissue (VT) and mature tissue formed zoosporangia (MT). Expression levels were assessed using the 18SrRNA gene for normalization. Results are presented as relative expression and compared with that in VT. Data are presented as means ± standard deviations (n = 3). Asterisks indicate significant differences at p < 0.05 between VT and MT (The Mann–Whitney U test).

DEG analysis revealed abundant transcripts in the sorus parts, including genes associated with the extracellular matrix (ECM), such as cell wall remodeling (**Table 2**; **Figure 6**). The ECM is a complex supramolecular network that imparts both rigidity and flexibility to multicellular tissues (Hynes, 2009). Beyond its structural role, it regulates development and protects cells from biotic and abiotic stresses (Kim et al., 2011). The ECMs of macroalgae, commonly referred to as the cell wall, consist of complex assemblages of cellulose, various hemicelluloses, and unique sulfated polysaccharides (Kloareg et al., 2021). The primary cell wall components in brown algae are anionic polysaccharides, specifically alginates and fucose-containing sulfated polysaccharides (Chi et al., 2018). Alginate plays a more prominent role in supporting cell structure than fucoidan (Kloareg et al., 2021).

Alginate is composed of two hexuronic acids: D-mannuronic acid (M) and L-guluronic acid (G). These are arranged in unbranched homopolymeric regions of M and G blocks, interspaced with a random arrangement of both monomers (MG blocks) (Heyraud et al., 1996). Alginate with a low M/G ratio exhibits superior mechanical properties compared to that with a high M/G ratio. Alginate with a high G-content also has a higher antibacterial activity than its low G-content counterpart (Ci et al., 2021). In brown algae, the composition of alginic acid varies with season, region, tissue, and age (Haug et al., 1974; McKee et al., 1992).

Previous research has shown a biosynthetic pathway for alginate in brown algae (Nyvall et al., 2003). The final step involves the epimerization of D-mannuronic residues into L-guluronic residues within the polymer chain, a reaction catalyzed by mannuronan C-5-epimerases (MC5E). Considering both their chemical structure and biological function, which is to provide strength and flexibility to the algal tissue, alginates can be regarded as functional analogs of the pectins found in higher plants. Consequently, mannuronan C-5-epimerases are likely functionally analogous to plant pectin methylesterases in controlling the cell wall matrix texture (Nyvall et al., 2003). In RNA-seq analysis, we found upregulated 8 DEGs encoding enzymes of MC5E (**Figure 6**), and this result showed similarity to previous results (Zhang et al., 2020). G-rich alginate in brown seaweed serves as a skeleton to provide stiffness or elasticity according to the environment, similar to pectins and cellulose in terrestrial plants (Nyvall et al., 2003). The upregulation of various MC5Es in the sorus parts is likely necessary to specifically customize the relative contents and distributions of G blocks, M blocks, and MG blocks in alginate chains.

In addition to MC5Es, cell wall remodeling-related genes were identified as sorus-preferential genes. DEGs encoding vanadium-dependent haloperoxidases (vHPOs) (g1659), (g277), NADPH oxidase (g3247), and superoxide dismutase (SOD) (g10745) were found to be upregulated in the sorus parts (**Table 2**). *Saccharina japonica* has 89 vHPOs, consisting of 21 bromoperoxidases (vBPOs) and 68 IPOs (Liu et al., 2019), emphasizing their role in processes such as chemical defense. vHPOs also play a role in oxidative cross-linking that facilitates the formation of phenolic polymers and their complexation with alginates, contributing to cell wall rigidification (Tarakhovskaya et al., 2015). Previous studies showed that phenolic substances, called phlorotannins, oxidized by vHPOs undergo self-assembly and form a macromolecular cluster with alginate, wherein the phenolic substances are encapsulated within the gel network of alginate (Bitton et al., 2006). The dynamics of vHPO activity during *Fucus vesiculosus* embryogenesis were highly synchronized with H_2_O_2_ content changes. Because H_2_O_2_ serves as a vHPO substrate, elevated ROS levels may serve as a prerequisite for an increased enzyme activity (Lemesheva et al., 2020). Genes involved in vHPO activity, including NADPH oxidase and SOD, were also found in upregulated DEGs of mature *S. japonica* sporophytes. NADPH oxidase, also known as respiratory burst oxidase homolog, is a well-studied enzymatic ROS-producing system (Marino et al., 2012). SODs catalyze the dismutation of O_2_^−^ produced by NADPH oxidase into O_2_ and H_2_O_2_ (Sagi and Fluhr, 2006). In previous studies, substantial intracellular ROS production was observed in *S. japonica* sorus, especially zoosporangium and paraphyzes. The high abundance of transcripts encoding NADPH oxidase and SOD in the sorus may contribute to the control of ROS levels for cell wall remodeling in the sorus.

The cell wall undergoes remodeling in a tightly regulated and polarized manner, a process primarily controlled by the cell wall integrity (CWI) signaling pathway (Levin, 2011). CWI signaling activation regulates the production of various carbohydrate cell wall polymers, as well as their polarized delivery to the site of cell wall remodeling. Proteins with a cell wall integrity and stress response component (WSC) domain were first described as cell surface sensors involved in detecting and transmitting cell wall status to the CWI signaling pathway in *Saccharomyces cerevisiae (* Verna et al., 1997). *S. cerevisiae* Wsc1 accumulates to sites of enhanced mechanical stress through reduced lateral diffusivity, mediated by the binding of its extracellular WSC domain to cell wall polysaccharides (Neeli-Venkata et al., 2021). RNA-seq analysis showed an upregulated DEG (g17300) containing a WSC domain in the sorus parts. Although there is a lack of information on the WSC domain in macroalgae, g17300 may be an important player in cell wall remodeling during *S. japonica* sorus formation.

Four upregulated DEGs were found containing a spondin domain as genes related to the ECM. The spondin family, which includes F-spondin and Mindin, comprises molecules attached to ECM (Feinstein et al., 1999). The F-spondin molecule consists of approximately 800 amino acids containing domains homologous to reelin, FS domain, and multiple TSR repeats (Higashijima et al., 1997), while Mindin contains an FS domain and one TSR domain (Umemiya et al., 1997). Structural studies suggest that the FS domain, exhibiting a homologous structure similar to that of the C2 domain, functions as a membrane-targeting module through Ca^2+^-dependent mechanisms (Tan and Lawler, 2011). There is little information on the spondin family in algae, but four genes that contain only the FS domain (named NySPL1–4) were identified from the red alga *Pyropia yezoensis* genome (Uji et al., 2022). NySPLs have similar secondary structures to that of FS domains from animals, and their transcripts increased in mature thalli treated with 1-Aminocyclopropane-1-carboxylic acid (ACC), which is a gametogenesis inducer. Similarly, the spondin domain from *S. japonica* only contains the FS domain (SjSPL1–4), and their transcripts increased in the sorus parts. Thus, FS domain-containing proteins play a crucial role in the formation of reproductive cells in red and brown macroalgae.

ECM plays crucial roles in protecting cells from biotic and abiotic stresses. The present RNA-seq analysis showed an upregulated DEG (g11685) encoding lipoxygenase (LOX) in the sorus parts. In plants and algae, the LOX pathway has been proposed to play a key role in their defense via major oxylipins. Oxylipins derived from the oxidation of polyunsaturated fatty acids induce the establishment of resistance in the kelp *Laminaria digitata* against infection by its brown algal endophyte *Laminariocolax tomentosoides (* Küpper et al., 2009). In higher plants, oxylipins from the LOX pathway function in cell wall modifications required for root development and pathogen arrest (Vellosillo et al., 2007). The link between the LOX pathway and cell wall modification in brown algae should be investigated.

### 3.4 Downregulation of chloroplast function

**Figure 7:**
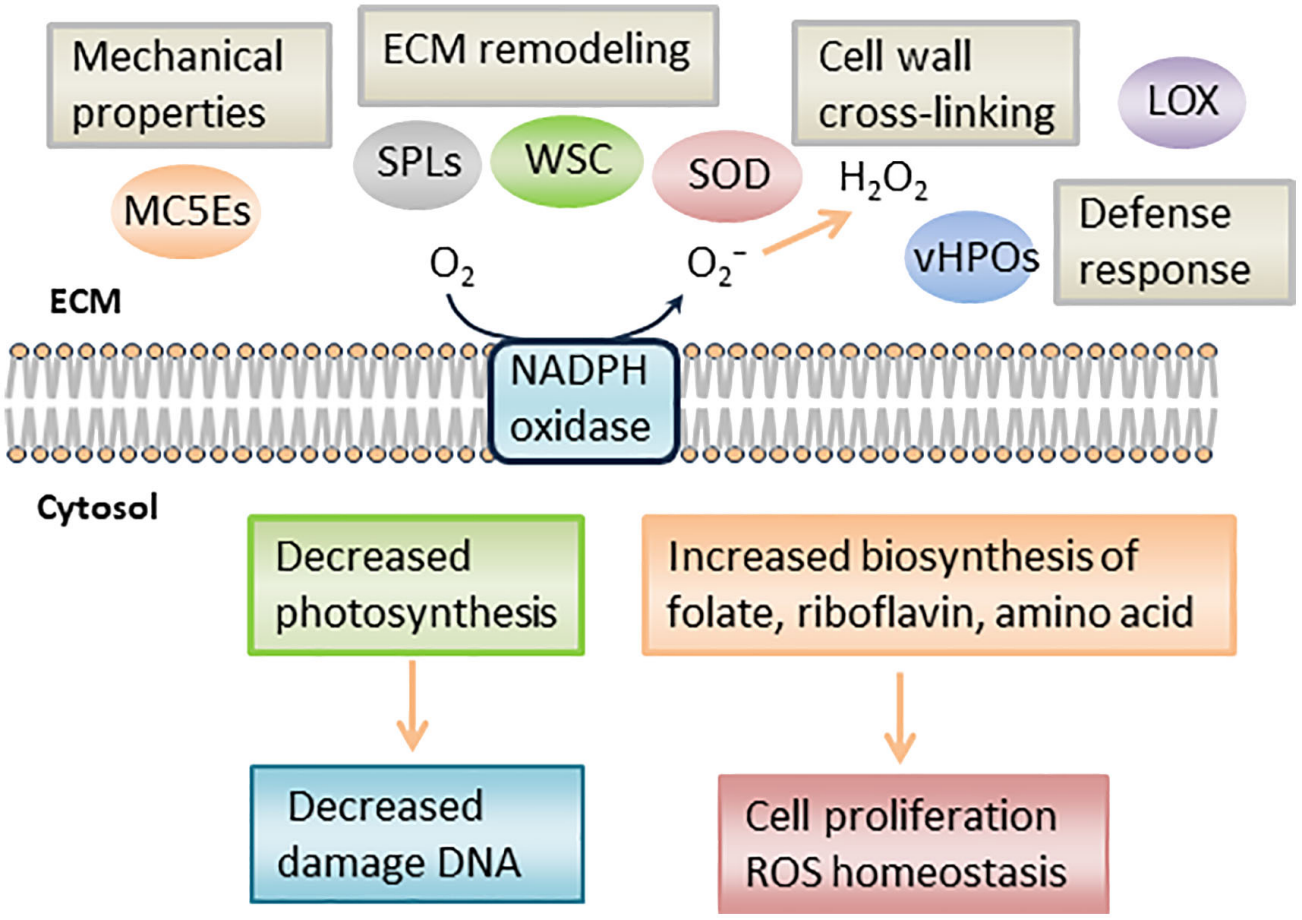
Schematic representation of putative mechanism during *S. japonica* sorus formation. LOX, lipoxygenase; SOD, superoxide dismutase; vHPO, vanadium-dependent haloperoxidase; SPL, spondin-like, WSC, cell wall integrity and stress response component; MC5E, mannuronan C5 epimerase.

In RNA-seq analysis, we found downregulated DEGs associated with chloroplast function, such as Tab2 family RNA-binding protein, Tic20, and light-harvesting complex protein (**Table 3**). In *Chlamydomonas reinhardtii*, Tab2 was identified as an RNA-binding protein required for the translation of the photosystem I subunit, and *Arabidopsis* Tab2 is involved in the signaling pathway of light-controlled synthesis of photosystem proteins during early plant development (Dauvillée et al., 2003; Barneche et al., 2006). Tic20 is a crucial component of the protein-conducting channel within the inner membrane preprotein translocon. In *S. japonica*, Tic20 is specifically positioned in the innermost membrane of the chloroplast, indicating its involvement in facilitating protein transport within the chloroplast (Chen et al., 2019). Light-harvesting complex proteins are known to be involved both in collecting light energy for driving the primary photochemical reactions of photosynthesis (Rochaix and Bassi, 2019). The reduced expression of genes related to chloroplast function is consistent with previous findings that the photosynthetic activity of *S. japonica* sporophyte was lower in the fertile parts than in the sterile parts (Nimura and Mizuta, 2001). One possible cost of photosynthesis in reproductive structures is the increased damage to DNA by the necessary exposure of reproductive structures to photosynthetically active, and hence (in nature) UV-B, radiation, and the increased potential for the production of ROS (Raven and Griffiths, 2015). Finally, we propose a possible model of the mechanisms of sorus development in *S. japonica* (**Figure 7**).

## Conclusion

The transcriptomic analysis suggests the upregulation of genes associated with vitamins and amino acid biosynthesis, ECM remodeling, and cell wall metabolism in *S. japonica* sorus portions, with downregulation of photosynthesis-related genes. Regarding ECM remodeling, DEGs associated with the composition of alginic acid and cell wall cross-linking were upregulated during sorus development. Candidate genes involved in ECM remodeling signaling pathways were also identified. Thus, this study on sorus formation mechanisms in *S. japonica* is important for a sustainable kelp production and to better understand the regulatory mechanisms of ECM remodeling in brown algae.

## Data availability statement

The data presented in the study are deposited in DDBJ under the BioProject Accession number: PRJDB17633.

## Author contributions

TU: Writing – review & editing, Writing – original draft. TK: Writing – review & editing, Writing – original draft. HM: Writing – review & editing, Writing – original draft.
