# Supplementary material for: Identification of differential gene expression related to reproduction in the sporophytes of Saccharina japonica
Source: Front Plant Sci. 2024 Aug 6;15:1417582. doi: 10.3389/fpls.2024.1417582 (PMC11333212; doi:10.3389/fpls.2024.1417582)
Supplement: Supplementary file 1 [file DataSheet_1.docx]

**Supplementary Fig.1**

**The sequence of upregulated DEGs**

>g256.t1

ATGGCGATGCGAATACCCAAGCCTTGGAACCAACAGCCAGCGTGCGCAATGGTCCGGGAT

CCGGAGTGGCCCTTCGTGTACAGCAAGTACCGCACACAAGAGGTGGAAGCGAAGGGCAGG

ACGAACACGAACCTCCAGGATGAACGGCACGAATGCGAAATGCGCGGGGTGAGAAGCAAG

TGCCACCTGTGCGAGGACATCGCTTGCGGGGACGGTCTGGATTCGTCGTGCGCTTTCCAG

CTGCAGGGGGTGAACGTATCTCTCCTGGCCACGAACGTTCCGGCGACCAGCGACACTCCC

TGGGCGGTCGGGGGAAGACATCGGAATGACTTCGGAAACGCCTCCGTCTCCAGGGTGTGC

GTCATGCTCAGCGGGGGGGCGTGTCTGGAGCCTTTCGgcGATGACTACTCCAACTgcacc

gtgagatgttgggGCGACGGATTTTCTGGTGCGGACGCGTCCCCAGTAAATTTCGTCACA

TCCAGGTCCGCGGTTGAAGAGAGCGGGCAGTTCACCGCGACGGTGGCTTGGGAGTACCCT

AAGGCGCTCACGTCGTCTTACGGCCCGCTGGAGCCGACCCCCACCTTCAACCCCCAGGGG

TACGTCTTTACTCTCGCCGGGGGATCGGGCGTGGCGGGTTACGCGGATGGAAGCGGGGCG

GCGGCTCTGTTCAACGACCcgcagGATGTTGCCGTCGATGCTGATGGCAACGTGTACGTC

GCTGACACTGGAAACCACCGAATACGCCGCATCTCTCCCGAGGGAGATGTCACCACCGTG

GCAGGCAGTGGCGAAGAGGGTTCCGACGACGGAAAAGCTGCTGCGGCGTCTTTCTCGTTT

CCCGGAGGCATTGCTCTCTACTACGATTCCTCGGGCGGTCTCGTCCTCTATGTGGccgac

accaacaaccaccgTCTCCGGAAAATCTCAGGGGACGTTAGCAGCGGTGCGGGCACTGTC

ACCTGCCATGCTGGGAGGTGCGGGAATGGAACGGAGACGTGGACGCGAACGACGATGCCA

GACACTCCCGAGCCTGGTTTCGCTGACGGGGACGGGATATTCGCGAGGTTCGATGGTCCT

GCGGGACTGGCCGCTGCGGAAGACGGCACCCTCTTCGTGGCGGACACCAACAATCACCTG

ATAAGAATGGTCCTCGCGAACTCTACCGTGTTCACGCTGGCCGGAGGGCTGGTGGGAGCG

GAGGCGAGCTCGGGCGGGGAAGAGAGCTGCCCGTCCCCTTGTTTGCGGGGCGTGGCTGGT

CACATCGACGGCAATCTCACAGAGGCCAGGTTCAACTACCCGGCAgacgtgtcactggga

cccaaCGGAACGCTTTTTGTGGCCGATTTGCACAGCCTAAGGCGAATATCCATGCCGGAA

GACCCGACCACCGTGCTCGGCATCGGCTTTGACGGAAGGGTTTCAACTGCAGCAGGCGGT

GCAGGGCCTGGAGAATCAGATGGAACGGGCCCCGAGACTCGTTTTGCCAGGCCGTCGGGC

GTTACCTCCACGGCCGATGGCGCCGCCTACATCTCGGACGCGGCCTCTTGCCGACTCCGG

AGAGCGGCTCCAACGGTGTCGTTCGCCGCGGCTCCGGCCACCTGCAACACAACCCTCGCC

CAGGTCTTGAGGCCGTCTGGCTGCTCGTCGTACGAGCCCCCGCACGGCGGGGACGGCCTG

ACCCTGTCCCCGCTCTCGGGAAACGTGTGGTACAACTCCTGGCGCAACGTTTCCGAGTTT

TTGATTGAagatggcggcggcgatgatgatgatgatgatggtgcgtCGGCTatggcggcg

gccgtggcagcagcggcgacagccGCGGCTGCAGGAGGTGGCACcgccgagtactcgtac

aacgaaGGCTCTGAATTCACCGCCGGGCGCGTTACCCATCAGTGCGTGGGGTTTCCACCA

CCTTTCGGATTCGACCGCGCCGACGACCCATTCGACTCCCTCACGGTGGACGACGGAATC

GCGGGTGTCTTCGAAGACACCGGGGTGGGCACCACCATACGGCTAGCCTGCCCGACCAGC

TGTCTcgaggcggcggcgaggggagGTCAAGCTGGAGAGGTTCGCGGGTCTCCAGACTTT

TACACGGATGACAGCGCCGTGTGTATGGCCGCGGTGCATGCGGGAGTTTtggccgccacc

acccccgcgtCGCCTCGCCCGCCCGCTTGGTCGTCATCCTCAGAGGGAACCCTCAACAAC

GACGGGTCCGATGCAGTCGTGGTCGTGATCGCGAGGTTGCTGCCGGCGAACGCCTCCGTA

GCCGGTCAGCCGACGCACTTGGGCTACGAAGCCAACAATGTTTCGACTGGCGACGCGCCG

GGCGACTGGGCGAGGGGTTTCGCTTTGGAGGCGGCCTTACCCAGTGAACTCACGGGGCAG

ACGGTGGCGGGGAAGCCCGCCGGGGCTCTGGGAGAAGGGTGCGGGGCGGTTAGCGACGGG

CAGCCCCCGCAGGAAGCGGTGTTTGGGAGGCCCCAGGGGATTGACGCGTGGCGATCGACA

AACCTTACTGATAAGAAACGGCGGGACCTGCGGACCGGCCCGACAGCTGCCTCGCCCCGA

TGGTTGGTCGGCCACGACTGTTCCGAACCGTCGATGCACCGAGGGACTGTGGCGGTTCCG

CAGCTCTGCGTGGGCCCCGAGGAGTGCGACTGCATCCCTGGTTACACAGGAAACCCCACC

TGTCTAGACGCCCTCTGCGTCCAATCGTGCGAGCACGGCGGGGCTTGCGCGGCTCCCGAC

ACCTGCGAATGCGCGGGAGGGTGGTTCGGGCCCAACTGCACGGTTCCGGTCTGTTCGCAG

ACCTGCGGAAACGGCGGCAATTGCACCGCACCGGGCTCTTGCTCGTGTCCGTCGGAgtgg

tcggggggagggggcaccgtggcggcggcggtggcgacggaggGGTCGGAGGAGGGGGAC

GACTGCCGCGTGCCCGTGTGCGAGATAGAGTGTCTGAACGGCGGGTGGTGCGTGGCGCCG

GGGACTTGCGCCTGCCCGCCGCAGTGGAGTGGTCATGACTGTGGCATGCCTGTGTGCATG

CAGGGTTTCTTCGTGCCGAGCTACAACGAATCCGGTTACGATCATGGCGAGCGCCCGTGG

TGGTCGAAGCGATACGTCCCCTGCGATGTTGCCGGCTGGTGCAATGCCACGAACGGGTTT

GACTGTTCGCAGgcATCGAGGGATTTCGAGGCGATCTCCATCGACTGGGGAGCCTACCAC

AGATCGGAGACCGGCCGGACGGAGATTCCGGACCGCTGCACCACGGTAGAGCTGGAGAAG

GACGTCGTGTCTCCGTTCTCCTACCTTCGGGCTGACAACGGCTCCACTCCGTACGCGAGG

CAAgcATCGAGGGATTTCGAGGCGATCTCCATCGACTGGGGAGCCTACCACAGATCGGAG

ACCGGCCGGACGGAGATTCCGGACCGCTGCACCACGGTAGAGCTGGAGAAGGACGTCGTG

TCTCCGTTCTCCTACCTTCGGGCTGACAACGGCTCCACTCCGTACAGTCCTTTCCACCCT

TACGAGTGGACTGCAGACCCGCGGCACCCCTGGTCGGCTTATAACGACACTGCGCCGGGA

AAAACGGGGCCTTTCCTCTGGGAAGAGGATCGTCAGGTGGCATTGGTGGAGTACAGCAAC

GTCACAGAGGGGCGCTACGTCTGCGCAAACGGCGGCAACTGCACGTCTCCGGGGGTGTGC

GAGTGCGCGGCAGGGTGGTCGGGGTTCGACTGCCGGACTCCCATCTGCtttcagGGCTAC

TACGAGCCCGAGCAGACTCGGTTCGTGTCGGGCAACGGAGACTCGGACGACATCGACGTC

TTCGAACCCTTTTTCGACGAGACCGAAACCGCCGCCTACCGCTTGACGTGGCCGTACTCC

AACCCCAACTACACGGCCGTCTGGGAGAGGTTCGACAACAAGAGCCACGTCGCCACGTAC

ACCGTGGAAGAAGGAAACGCCCGGTATCTCGGACCGTCGGACTGGTCGGATAGCGGGGAC

GAGAGGACTCTCACCTCTCAAGGGGGATACTACTGCTCCGTCAGGGCTAACACGGAGTAC

GAGAACATCGATTTCGTGCTGGACGCGCCCAACTACTGGTCCAGATACATGGACACCAAG

GTGGAGGGCGACGGTGAGACGTACACCTTCTGGGAGAACATGCTGTGGCTTCCGCTTCAC

ATCAAAAGCGCCAGGCTGGAGCTCACCGACGACGACGGTAACTTCTACGTGTACACCGAG

GTCGGGTTCATGCGCGGCGGAATCTGGCACGCGACCGGCGAGCCTTGGCAAAAGGGCGTG

TGCATCGTGCAATTCAACAGGAGCTGCCCGGCGGGGAAAGCGCAGGCTGTGGACCTTCAA

TCCATAGCCCTCGGTGAAACTGGCACCGGGGTCTTCGTCCAGGACACGGACCTGAGCTTC

AGACCACGCGTGACGTACACCCAATGGAAGGAAGAAGGCTCTGGGAGGTGGGAGgaggag

ggaggcgaGTGCGTAGACCAAGTCCTGAGAGGGTGTTACAACAACGGAACCTGCGTCGCC

CCGGACACCTGTGAATGCGCGGAGGGATGGACAGGCTACGACTGTTCGCAGCCGGTGTGT

CCCACCCCCTGCCAACACAACGGCAACTGCACCTTGCCAGACGTGTGCACGTGCGCGCAA

GGGTGGACGGGGGAGTACTGCACGGAGGCGATTTGTGCGCAGGAGTGCAACAATGGCGGG

ACTTGCGTAGCTCCGGACGTGTGCCAATGCGCGCAGTGGGATAGCGACTTCGTGGACGGT

AGGGAGGGCGGCGGGAGGCCGCTGTTCCGAAAGCCGAACGGAGACCCGCAGCAGACGGGG

TGGACGGGGTACGACTGCTCCGTGCCGATATGCGTGCAGGCCGAGGAGTTCGTTTTGAAC

GTGGAAGACGACGCCTCCAGCGATTACAAACCCCTGGGCGGGCACGGCTTCGACGGCACC

CTGGCTTGCGGGAGGGTAAGATGTCCCGAGTACGACGCCACGTACGTCACCAATctcggt

accagTTTCCAGACCGGGTGCGGTAACGACCCGCTCGACAGCGGCTGCTGTTACTgggac

tcctcctcctcctcgtggaCGTGCGAGTTCTGCCAAGAGTTCGAAGAGTTCGATTATAGT

TTCGTGTGCACGGGTAGCATATCGTCCGAGGTCTACACTGACATCGACGACGTCCCCTCG

AGGTTCCTCGGACCAGACGGCGAGGTTTCTCTGTGCGGGCCGGACCTCAGCCCCCTTCCC

CCGGCGGTGAACGCCGATCCCATCAGAGACGACTGGAAATACAGCAGCCACAACCTCCAA

AGCAACGCCACGTCGCCGAGGTTCTTGTGCCAGCGACTCCAATGGGTGCAGGGCGACTAC

ATCGACGACGCTGGGCTGGGCTCGGGAAGCGGGTTTGGCACCGACTCTGGCCTCGCTTCC

GGTCGCCATGTGcgctacaactacaacaactacaccaagGTGAACGACGACACCTGGACC

CATGGAGTTGTCACCCCTGGGGAGGGCGTTTACCGATGTCACAACGGGGGCAGCTGCCTA

GCTCCCGACACATGCTCCTGTCCGGACGGGTGGGACGGCTACGACTGCGAAACACCACTG

TGCAGGCACTTGCAGCCGTCGGCCGTGGTGTCGAGCTGCCAGAACGGGGGGATATGCGCG

GCTAAGGATGACTGCACGTGCGTCACGGCGGACAGCGTTTTGTGGCAGGGGCACTTGCAG

CCGTCGGCCGTGGTGTCGAGCTGCCAGAACGGGGGGATATGCGCGGCTAAGGATGACTGC

ACGTGCGTCACGGCGGACAGCGTTTTGTGGCAGgttgtCGCGGACGATTTGATGCGAGTG

CAGCTGAACACAATGGACGAGGAGAGAGTGGTCGCCTTCGAATCGGACCCATGCGCGATG

ACGGAGCGGTACGCCGCCGTGGAGCACGAGGGGGCCCAATACTACAGGGGGAATTGCACT

GCGCCGGACCAGTGCACATGCGCTTGCTGGGCGCACTTCGACGTAGACGACTGCGAAACG

TTCCAGGTCAACTGCGACGGGCCCTGgcagGACCCCCTGGTGCAGTACAGGGACGTCCTT

CCCGTCACCTACGCTTTCGGGACCGCCTCTTGCTGGAGCGGCTACGAGGGGATACAGTCA

GACGACGACCGGTTCTTGACTTGCCACTACAACATCTTCGAGCCCAGCTGGCTGCAGCGC

AACTCCATCCCCTTGATCattggGGGGGCCTTCTTTGCGTTTTTCGGGTCCATCGTGTAC

GCAGTGGTTCGTCGTCGACTGAAGCGAAGGTTCCTCCAGGCCAAGATAGAGAGGCGACGG

TCTCGACGCAGCTCGGAGATGTCTCAGCTGTCTCAGCAGCCCCACGCCTTTGCCCACCGA

GGCTAA

>g271.t1

ATGTTTCAGCGGACGCCTGGAGACGGTGATACCGCTGCGGTCCCTGTCGTCGTCGAAGTT

TGCGGCCGACTGATTTCTTGCTGCGCTGCAAGTCGGGGTGGGCTTGGCGCTCTATTGCCG

GCGGTGCTGGTTGCAACCACTTTCGTCAGGTTGACTGCCCTGCTGGCGGCATTCCCTCCC

TGCCTCGGCACCGGGGACTCCAAGGAGCTCCAACCAACGCACCCTCTGCTGAGCGGCAGT

GTGTGCAGGGTCCGGGACACCCTCGATTTTCTCGACCCGGTGGCGCGCGCGAAGGCGACC

CTCCTGAACCGCCTGGCCATCGCCAAAGATGAGTTCTCGGTTGGACCCACTTGCCACATC

AACAACGGTGACGAGGAAAACGTCCCCCTATTCGCCGGGCAGTTCCACAAGACACTCCCG

CACGACAAGTTCGGACAGGTGGACGAGTCCGCATACAAGAAACTGCAGGAGTGTGTGTTC

ACGAGCGACATCAATGTGTGTGACGACGTTCCCTCTGGCGCGGGCCGAAGTGGTGGCGCA

AAGCTGACCAACCCGCTCGGGGGCACCGCGCACCAGGTCGATGGTGCCGACAGCGACAAT

GTCTTCATCACCACGCCCGACAGTCTCCTCTCCGAAAGGCTGGCGGCGCAGCAGACAGAG

GTTTACTGGATGGCCCTGGCTAGGGACGTCCCCTTCTCGCAGTTCGACACCAACGATCTC

ATCAGGCTCGCAGCAGAGAACCTTGAGGGACTACCGGCGTTCGAGGGACTTAACATCCCC

AGGAGCAAGGACGGCAAGATTGACACTGTCACTGATCTGTTCCGGACGACCTGGCCGGGT

GTGACCACTGGTCCCGTCGTTTCTCAGTTCCTGTTGTCGGACTTCGAAATCGACTCTATC

GTGGTGGAGCCGAAGGCTGTCCCTCTCGCGAAAGGGATGGACTACATGACATCTTTCCAG

TCATGGCTCGATGTGCAGAACGGTGCTTCCAAAGTGGACACCGTGTTCGACAAAGAGAAC

CCCCGTTTCATCCGAAACGGCCGAGACCTCGCCACTGTCGCCTTCCGGGACATCCTCTAC

ACGGAGGCATTCCGCGCTGCACTCATTTTGTTCAAGCAAGGTGCGCTTGGGGGCTCTGTC

GGCCCCTACGCGGATTCCGCGCGTCAGCAAGGGTTTGCCACCTTCGGCGCTCCGCACATC

CTCACCGCCATGGCCTCGGCCAGCTCTTCCACACGCAATGCGTGGTACGCTAAGTGGCAA

GTCCACCGCATGTTGAGGCCCGAGGCGTACGGTGGCCTGGTGCACAACACGCTCATGAAG

AACGTCATCACCCCGCTGCCCGACTCCCTCCTGAAGAACACGGAGCTCCTGAACCGAGTG

GAAGTGCACAACCAGGCCATGAACACAAGAGGCGAGAAGACCTTCCTGCTCCCCATGGCG

GTAGCAGAGGGCTCTCCCACGCACCCCGCCTACCCTAGTGGACACGCCATCAACGTGGGT

GGTTACATCACCGCTCTCAAGGCGTTCCTCGGGTTCGAGGGCGGGAAGAGGTGCTTCCCC

TACCCGGTCATCTCTAACGACGAGGGTACAGAGAGGATCCCCTATGTACCGACAGGAAGG

GAAATTGTGGGCAAATGCACCAACGAGTACGGACGGGAAGTCGACGGACTGACCTACGAG

GGAGAACTCAACAAGGTAAGCTCCAACGTCCTCTTGGGAAGGTCGCACATCGGTGTTCAC

TGGAGGATGGACGGTGTTTACGGGGCGCTCATGGGAGAGACTAGCGCGGTCCGTCGCCTG

CAGCAGGAACTTCCTGGTCTCTCGGAGGCCCGCATTGTGGACGACGCAGGGCGGCACGAC

ATCCCCCCGGCGACGTACAACTTCCGCCTTTACAGCGGCAAGATGCTCGAGCTGTACGGT

AGAAACTTGTACAAGCTGGATGGCAAGCTTTGCAAGGGGGCCTTCACCGGCGACGACTTC

TGCGACGAGGTGCACGACAGCAGCTTCGAATCGTTTGAAGATGCTATCCAGCAGCATGCC

ACCTTCTCGATCCACACCGAGCTGTGA

>g272.t1

ATGAAGGTTTTCGCCGGCTTTGCGGGCCTGCTGGGGGCATTTCCTCTCTGTCTCGCCGCT

GAGGATCTCTACTATAAGAACGAGTACCACCCGCTGCTGAGCGGGAGCGTATGCAGGGTC

CGGGACTCCCTCGATTTCTTGGACCCGGCAGAGCGCAGGAAGATAACTCTTGACTACCGC

ATGGCCATCGCCGGGTCTGAGATATCGGTTGGACCAACTTGCCACATTACTAATGGCGAC

GAGGAAAACATCCCGCTATTTGCTGGGCAGTACCACAAGACGCTCCCGCACGACAAGTTC

GGACAGGTGGACCCGGACGCCTACAAGAAGCTGCTGAGATGTGTGTTTTCGACTGACATC

AACGTGTGCGAAGACGTGCCCTCCGGGGCCGGAAGGAAGGCCGGAGCAAAGCTGACCAAC

CCGATTGGGGGCACCCCTCACCAGGTCGACGGAGCTGATAGCGACAATGTCTTCATCGCG

ACCCCGGACAGTTTACTCTCCGAAAGGCTGGCCGCGCAGCAGATAGAGCTGTACTGGATG

GCCCTAGCTAGGGACATCCCCTTCTCGCAGTTCGGCACTAATGACGTCACCAGGGCCGCA

GCGGAGAACCTGCAGGCCCTCCCGGCGTTCAGGGGACTTAACATAGTTAGGAGCGAGGGC

GGGAAGATCGACCCTGTCACCGATCTATTCCGTACAGACTGGCCAGGTGTGACCACCGGG

CCCGTGGTATCCCAGTTCATGTTGTCAGACTTCCTGATCGATTCTATCGTGGTGGCGCCG

AAAGCAGACCCCCTCGTCCCAGGGGTGGACTTCATGACCGCCTTCCAGCCGTGGCTCGAC

GTGCAGAACGGTGTTTCGAAAGAGGAGACTCTGGTGGACACGGAGAATCCTCGCTTCGTC

CGGAACGGCCGAGACCTGGCCACTCTGGCCTTCAAGGACCAGCTGTACACGGAGGCCTTC

CGTGCCGCGCTCATATTGTTCACGCAGGGAGCGCTTGGGGGTTCCGTCGGGCCCTACACT

GATTCCGTGCGCCAGCAGGGATTTTCCACATACGGCGAGTCGCACATCCTCACCGCCATG

GGCGCGGCCAACGCTTGCACGCGCCATGCGTGGTACGCAAAGTGGCAAGTCCACCGCGTG

CTAAGGCCGGAGGCATACGGCGGCCTCGTACACAACACGCTGATGAAGGACGTCATCACG

CCGCTGCCGGCTTCTTTGCTGGACAACACGGAGCTGCTTAACCGAGTGGTGGCGCACAAC

AAGAAAATGAACCCAGATGGCGAGAAGACCTTCCTCCTCCCCATGGCGGTAGCACAGGGC

TCTCCCACGCACCCGGCGTACCCCAGCGGTCACGCTATCAATGTCGGTGCTTACATCACC

ACGCTCAAGGCGTTCCTCGGGTTCGAGCTGGGGCAGAGATGTTTCCCCAACCCCGTCATC

TCGAACGACGAGGGGACAGAGAGGATCCCCTTCGTGCCAACAGGAAGGGAGATCGTGGGC

AAGTGTATCGACGAAAATGGCAAGAAGGTCGACGGTCTCACGTACGAAGGAGAGCTCAAC

AAGGTCAGCTCGAACGTGATCTTGGGAAGGTCGCACATCGGAATTCACTACAGGATGGAC

GGAGTCAACGGCGCGCTCGTGGGGGAGACTAGCGCGGTCCGCCGTTTGCAGCAGGAACTG

CCCGGCCTCCCGGAGTCTCGTGTTCCAGGTTGCAAGCAGACTGCCGGATGCGATCGTAGG

GCCGACATCCCCCCGGCGACTTACAAGTTCCGCCTGTACAGCGGCAAGATGCTCGAGCTG

TTTGGCGCGAAACTCTACAAGCTTGGCGACCAGCTTTGCGAGGGGGCTTTCACCGGAGAT

GACTTCTGTGATCCGGTGAGGGAGGACGACTGCGACTCGTTTGAAGACCTTGTCAAGAAG

CATGCAACCTTCTCGGTCCGCACCGAGCTCTGA

>g277.t1

ATGAATTCCGCTGATTTCGAGactttcgtgGGCTTGACGGCACTGCTAGCTGCGTTTCCT

CCCTGCCTCGGTGCTGGTGACAGCACGGAAAGCCGCGCAACACAACCTACCAGCGGGAGC

GTGTGCACGGTCCGGGACTCCCTGGACTTCCTCGACCCGGTGGCGCGCGCCGAGGTGACC

CGAAGGAACCGACTAGCCATCGCCGAGGACGAGTTCAACGTTGGACCGACGTGCCACCTC

ACCAATGGAGACGAGGACAAcgaagcgctgctgctcgcTGGACAGTACCACAAGACCCTC

CCGCACGACAAGTATGGGCAGGTGAACAAGAACGATTACCAGAAGCTGCTGGACTGTGTG

CGCACCAGCGACATCAACGTGTGTGAAAATGTGCCGGCTGGGGGCAGCGCGAAGCTCACC

AACCCGCTCGGGGGCACGGCTCACCAGGTCGACGGTGCCGACAGCGACAACATCTTCATC

GCCACGCCCGACAGTCTCCTCTCCGAACGACTGGCCGCGCAGCAAACCGAGGTGTACTGG

ATGGCCCTGGCCAGGGACATCCCCTTCTCGCAGTTCGCCAACAATGATCTCACCAGGCTC

GCCGCAGAGAACCTTGGGAACAACGAGGCATTCCACGGGCTGAACATCCCCAGGACCGAA

GTTGGAAACAGCGGAAGTGATGAGACGAGGGTCGATCCCGTCACCGATCTGTTCCGCACG

GACTGGCCGGGTGTGACCACCGGCCCCATTGTTTCTCAGTTCCTGCTTTCGGACTTCGAG

TACGACTCTATTGACGTGGAGCCGAAGGCGGTCCCTCTCGCCCCTGGGGTGGACTACATG

ACGTCATTCCAGCCGTGGCTCGATGTCCAGAACGGTGCTTCCGACGTGGACACGGTGTTC

GACAAACCAAACCCTCGCTTCATCCGAAACGGCCGAGACCTGGCGTCTATCGCGTTCAGA

GACCTCCTCTACACGGAGGCCTTCCGAGCCGCGCTCATTTTGTTCAAGCACCGTGCACTT

GGGGGTACCGTCGGCCCCTACGCTGGTTCCAACCGGCAGCAGGGGTTCGCTACCTTCGGC

GAGCCGCACATCCTCACCGCCATGGCCGCGGCTAGCTCTTCTACGCGCCATGCGTGGTAC

GCTAAGTGGCAAGTCCACCGCGTGCTGAGGCCCGAGGCGTACGGTGGCCTGGTCCACAAC

ACGCTCAAGGGGAACGTCAATATTCCGCTGCCGGACTCACTCCTGAGGAACACGGAGCTT

CTCAACCGAGTGGAGTCGCACAACCAGGAGATGAACGGAGGCACGGAGAAGACCTTCCTG

CTCCCCATGGCGGTGGCTGAGGGCTCTCCCACGCATCCAGCGTACCCCAGCGGGCATGCC

ATCAACGTCGGCAGCTACATCACCACGCTTAAGGCGTTCCTCGGATTGGAGATGGGGCAG

AGGTGCTTCCCCGAACCCGTCATCTCGAACGACGAAGGAACAAAGAGGATCGCCTACGTG

CCGACCGGAAGGGAAATTGTGGGCCAGTGCATCGACGAGAACGGCAAAGAGGTCGACGGC

CTCACGTACGAGGGGGAGCTCAACAAGGTCAGCGCCAACGTGCTCTTGGGAAGGTCGCAC

CTCGGTGTTCACTACAGGATGGACGGCGTTAACGGCGCGATGATGGGAGAGACTAGCGCC

GTCCGCCGCCTGCAGCAGGAACTGCCTGGTCTCTCGGAGGCCCGCATTGTGGATGGCGCA

GGGGAGACCGACATCCCACCGGCCACCTACAGCTTCCGCCTTTACGGTGGCAAGACGCTC

GAGCTGTACGGCAGGAACCTGTACAGGCTGGATGGCAAGCTGTGCCAGGGGGCCTACACC

GGCGACGACTTCTGCAACGAGGTACAGGAGGGAGCGTCGGACTCCGCTGAAGACATCGTG

CAGAAACATGCCACCTTCTCGATCCACACCGAGCTTTGA

>g278.t1

ATGATACTATTTATAACGACCGTTGGCCGCCTGAAGACTTTCGTGGGCTTGACGGCGCTG

CTGGCTGCGTTTCCCCCCTGCCTCGGCGCTGGGGACTCTAACGAAACCCAAGCGAACAAG

TCTCCGGCCAGCGGGGACGTGTGCAACGTCCGGGACTCCCTGGACTTCCTCGACCCGGTG

CAGCGCGCGGAGGTTACCCGAGCGAACCGACTGGCCATCGCCGAGGACGAGTTCAAGGTC

GGGCCGACGTGCCACCTTACCAATGGAGACGAGGACAACGAGACACTGCTGCTCGCCGGA

CAGTACCACAAGACCCTCCCGCACAACAAATACGGGCAGGTGAACAAGGACGACTACCAA

AAGCTGCTGGACTGTGTCCGCACCAGCGACATCAACGTGTGCGATAATGTGCCGGCTGGG

GGCAGCGCTAAACTGACCAACCCGCTCGGGGGCACGGCTCACCAGGTCGACGGTGCCGAC

AGCGACAACGTCTTCATCGCCACGCCCGACAGCCTGCTCTCCGAACGGCTGGCCGCCCAG

CAGACCGAGGTGTACTGGATGGCCCTGGCCAGGGACATCCCCTTCTCGCAATTCGCCACC

GACGACCTCACCAGGCTCGCCGCAGAGAACCTTGGGAACAACGAGGCATTTAACGGGCTG

AACATCCCCAGGACCAAAGTTGGAAACGGCGACGACAACGAGACAAAGGTCGACCCCGCC

ACTAATCTGTTCCGCACGGACTGGCCGGGTGTGACCACCGGGCCCATGGTGTCTCAGTTC

TTGCTGTCCGACTTCGAGTACGACTCTATCGACGTGGAGCCGAAGGCGGCACCTCTCGCC

CCTGGGGTGGACTACATGACGTCATTCCAGCCGTGGCTCGATGTCCAGAATGGAGCTTCC

GACGTGGACACTATTATCGACAAGGAGAACCCCCGCTTCATCCGAAACGGCCGAGACCTG

GCCTCTATCGCCTTCAGAGACCTCCTCTACACGGAGGCCTTCCGTGCCGCGCTCATCTTG

TTCAAGCACGGTGCCCTTGGGGGCTCCGTCGGTCCCTACGCCGTGTCCAACCGCCAGGTC

GGGTTCGCTACCTTCGGCGAGCCGCACATCCTCACCGCGATGGCCGCGGCTAGCTCTTCT

ACGCGCCATGCGTGGTACGCTAAGTGGCAAGTCCACCGCGTGCTGAGGCCCGAGGCGTAC

GGTGGCCTGGTCCACAACACCCTCATGAAGGACGTCATCACACCGCTGCCGGACTCCATC

CTGAACAACCTGGACCTCCTCCGAAAGTTGGGCCCCCACAACGAGAAGATGAACGGAGGC

AAGGAGAAGACCTTCCTGCTCCCCATGGCGGTAGCCGAGGGCTCTCCCACGCACCCCGCG

TACCCCAGCGGCCATGCCATCAACGTCGGCAGCTACATCACCACGCTTAAGGCGTTCGTT

GGATTCGAGATGGGTCAGAGGTGCTTCCCCGAACCCGTAATCTCGAACGACGAAGGAACA

AAGAGGATCTCCTACGTGCCGTCAGGGAGGGAGATTGTGGGTCAGTGCATCGACGAGGAC

GGCAAAGAGGTCGACGGCCTCACATACGAGGGGGAGCTCAACAAGGTCAGTGCCAACGTG

CTCCTGGGCAGGTCGCACCTCGGTGTTCACTACAGGATGGACGGTGTCAACGGCGCGATG

ATGGGAGAGACTAGCGCCATCCGCCGCTTGCAGCAGGAACTGCCCGGACTCTCGGAGGCA

CGCGTTGTGGATGACGCAGGGGAGACGGCCATCCCCCCGGCAACATACAGCTTCCGCCTT

TACAGCGGCAAGATGCTCGAGCTGTACGGCAGGAACTTGTACAAGCTGGACGGCAAGCTG

TGCCAGGGGGCCTACACCGGCGACGACTTCTGCAACGAGGTGACGGCGGACGGGTCCAGC

TCCTTTGAAGACCTTGTCCAAGAGCATGCTTCGTTCTCGCTCCGCGCCGAGCTATAA

>g332.t1

AtgagcgacggcagcagcgcaTCCACGTCAAGGCGACGAGAGGGCGCTCGATCTGAAAAA

GACCCCGGCGTCCCTACATCGAAGGCGAAAGATACCCTCGAGGCGCAAGATACCACCACC

GCCCAGGGTAGCACGGGCGCCCGAGAGGGCGCTATCAACGCCAGCGCCAAGCGCCACAGC

CGCAGCGAAGGACGACGACGGCGCAGCAGCGACGGTGGCGACAACATCGTGGGCGACACC

GCCCGAGCAAAAAGCAGTGTCGATGACAACCGCCGAAAACTGGAGGGCATTTTTGGGGAC

AGCAGCGGCGAAGATTCCCGTGACGATGATAGCTCCGAAGAGGAAGAAGCAGCGGGAGAG

CACACAGCTTCTCCATCGCCAAGGGAACGTGGAGGGAGACGTCGGTCTTCGGGATCGCCT

GGGTCAGGGCCCCGAATGCGGGGATCGCCTGACAGGTCGCGAAGCGAGGAGTTGTCGCCG

AGACGCAAAGTTGTCTCGAGGGAACGCCAGCGACCCAGCAAGCGTCAGGAGGATGTGTCG

CCACCTCGCCGCGGAAGGCGACATGTTGGCTCCGCTGGCCCTCCCTCGCTCAGTCAGACG

CAACATGCGGCAAGCTCGCTTGCCGTGATGGGTGCGGGCGGCTCGGGGTCTAGGAGGCCG

AGGACGAGGGCGGAGCTGAAGGAGCAGGGGAGGATGAGAGAGTGGGCCAGCTTTTGCCGG

CGCAAGATATACCTCTCTTTGGACGATCTCTCGAGGGAGGAGCGGCCGCGGGTGGCGAGG

AAAGGCGCAGTTACCCCTTTCTCTCTTCGACTAATGaagGTGGAAGGTTGGCAGGTGCCT

TCGGTTTTGGTGGAGCGCGCGAGCGACGCCGGCCACGGGCTGCGATTTTCGTTGAGCGTG

AGCTTCTACCACGCGGGATCGAAGCGGTTCTACGGAGATACCTTCATGGGAGAATCGCTT

GATGAAGAGGACGAGGAGCGAGTGGAGATCGTTTCGGAAAAGCGTCAGCAACCCTCCTCG

GCCTCGCGTAAGCAGAAGAACCATCGGTCAAAGGCAAACAACGGTCGCGGCAAGCAAGGC

AATCGAAGGCGTCGAGGAAGAGGCGCCAGGGGAGACGAGGACGaaaatgacgacgacgat

gaggaGCAGGAGATGGAGGTTGGGACCAGGCACGAGGAACTTGTCTACTGGTATACCCGA

TTTGAGGATCCGAACTGCATCGCGGTGGTCGAACTGGTGGCTACGATCATGGACACCGTC

AACGGgatacagGTGGGGCAGTACGGTTGTGGCTGGACGTTCATCCAGTTTTTCGGGCCC

AACGAGCCGGAGGCAGTCCAGCACAGGGATGTCTACCGAGGCTCCCCGCGAAACCTCCTT

TTCTTTGAGCAGGGCGACTGGGGGAGCGTGGGAGAGACGGTTATTCCTGGGTGCTCCCTG

TGGTTCACCCTCTCCACGTGGGAAAACTTGATGAAAGCTAGACATTTGTTCCGTCCCGAC

GAAATCGTCTCCGCCACGGATGTGCTTCCGGGCATGCAGACGAAACAAATCGCCATTCCG

GACAAACCTGCAAAAATTGGTCCTTTCTTGGGCCTTCGGGTCGAGGAAGAAGCCGCCCCT

CCGTCACGCCGTGGAAGGCTTtccgaggggagggggcgctGGAGCTTGCTTCCAGCCAAG

CCTCGCCTCGCGGTGTGCGTTGAGTTTCGGCTTTCGGCGCTCCGCGTGATCATTCCCGGG

AGGGAGGGGTACGAGCGAGACCTGTTAAAGAGCCTAGGGGAGGACATCCCAGACCAGGAG

ATCGTGAAAACCACGACACACCAGCACACCTTGGGGTTCTCAAGCACGCAGCGAGAAGTC

GTAACCTCCTCGGCGGGGGGCGCTGGTGGCGggaaaggggaagggaggggggggggtgga

aaccgGGCCAAGATAGTTGATAGGAGGGCAAAAATCGGGTTCCACAACGGCCACACGCTG

GTGCTGCCACACGAGTGGGTGGAAACTCACTTGGAGGAGACTAACGAAGGGGACGTGCTG

ATGCTGCCGGAGGAGGTGTCGTCCCTCAATGTTGATGGTTACGTGACGCATCCGTTCTTC

GCCCTTGTGGTCCTCATCGAGTACACTATCAGAACGAGGCCAAAACATTCGGCGggtcga

gggggcggggggaggggcaagaAAGGAGATCCATTGAGCCAGGCACAAGCGGAGCTCAGC

CATGCCGCTCTACCTGTGGTTGTAGGAATGCAGGTCTTTCTTCCGCACGATGGCAAGAGG

CTTCGGCTTCGCAACACTCCTCGCGACGAAGAGAGCATGGACATTGGGCTGAGGTTGGCG

GCCGATAAAAACGTTCGTCTCATCACTACCGATCTGGTGTACACAAACAAAGACCACCTC

CTGGACGAGGCTCATGGTGGCGCGAGTGATGGCGAGGACGGTCGAGGCCCTAAAATGGTC

TTCTTCGATCTCAAGGCGTACGATTCACACGGGAAGATGCTGGAAGACGAAACTCCTGCA

AGCGGTGACGAAAGTCCGCTGGAGGGTGGCTGGGAACCGCCGAAAGAGACAATCGTAGAA

AGCAGCGACAGTGCGACATCATCAAGTACCGACGACAGTACTAGCTCAGAATCTTCGGAA

GAAAGCTCTGACACTGAGAGCGATGCTccgccggcggcgaagccgaAGAGAACACCCCCA

CGACGTCCGAGGGTCGCAAAGCCAAAACCATCCAAGGTCAAGCACAAGCGACCAGCCCGA

CAAGAGTCGACCAGCGACGGGTCGTCTTCGGAAAGCACCAACCCTTCGCTGGTACAAGGC

GATAGCGATGCGAGTTTCCTGCGTCGCCCGAAGCCTACCCGAAAACGACACGTGCACGTG

CAGGCGACGGAGAAGGGGTGGGACGGCTGGAGAAAAAACGTCAAGGTCCCCCGACGGAGA

GAGTCGCTGATGGGCCAAACCTTACAAGCGCCTCTGCACGCTGAAGCGTCCGCTCAGACG

GCTATCACGTCTGACCGCAAACCGGACTTGGGGTTCTATAGACCAGAagctgtggtggtg

ggggggttcgCGCCGCTCGCTGAGGGTTTATCGAGGGCGAGCCGTACGAGGCTAAGTCGC

CATGGGTTCACCGACGTGATGAAGGATACGGTAGACTCGTACGCCGAGACAGGCGGTAAC

ACCGCAGGACGGCAGCAGATACCTATCGACCTTGACCTGGAAGCAACAGACCCCTTGGCC

AAGCACGAGATCACCCTCCAGTTTGCGGCATTTAGAGCGCCACCTTGCGCGGGTCGGAGA

AAAAGCAGCGGGTCGAGGGAAGAGGgcgagcgaggaggaggaggggaggacggCGACGCA

TCAATGCCTACTTCGTTTTATTTCACGTACCAGTTTTATACGTGTCTGCCCACGAGAACA

GAGAGAATGCTCCTGAGGCCAGACGGACAAAGGGATAGGCTCTACCAGgcggaaggggga

ggggcgacTTGCTTGAATATATTGGTTCGGGAGGGGCGATACGGGAGGGACGAGCCTTCT

CTGGGGTTGCGGCACAGCATCGATACGACGGTGATGCAGCCTTTCGAAGCCCAAGCTTTT

GCGACCTACATGGCGGGAAGCACCCTCTTCGTCGATGTGTGGGACGCGGACGCCTTGATG

CACGTGGGAACGCTGGCCCTGCCCCTGAGAATGCTGATGCGGCAGCAGAAGGGCGTAGTG

AAGACGGCCATGGAGTACGAGGTGGTCGCCAGCACAGCCGCCGCGGAGGACTGCGGGAGC

GGAGGTGGCGTGGCCGTAAAACACGGGACGGTCGGGAAAGGTCCAGTCGTGGGCATGGTT

CAGGTACGGGCGCGCCCTCTGTCGGACACCAACCCagaaatttccaaatatatGGAGGCG

GTTGTTGGTCCCAGTGCCGGGGTTGGAGGCATTGCCAGCCTGGCGCGGAGAGGGAGGGGT

GACGTCGACGCTGACAGCATGGGGTATGACGAGTTAATGTTGTTGGTAAGGCGGTTCCGT

GGGTCTGCTAAGGGCGCTGTTTGGTATTCTGGCCCCCTGTTGAAGCTCCTCGACATTCCT

GGGCACAAGCAGCTTGAGCAAATTTTGGTCAAGGCAGTGGAGCGATCGGAGAGGTCTGGA

TTGGGCTTGGCCGAGGCCTTTCgggaaatggacccgcacaggACGGGCGAGATCAGCACG

CATGACCTCGAAGAGCTTTTCTGGGCAATGCTGTCGCCTGGAGAAATGGCCAGAAATCAC

CTCAAGACAGCGGTGAAGAGAATCGACCCTGGAGAGACAGGAAAAGTAGGCCTGCGAGAA

CTCGTCACCTTTATCCACACGAGGCAAGGGGGCGGGAAGGGCAACGCCGGGAAAACTGCA

GAGACGGGTCTGCGAAGGGCACTCGCGAGGGCGGAGCTTGGTGGAAACTCCGTGGAGGAG

GCGTTCTTCCGCGTTGACGAGAGAGGGGAGGACTCCGTCAGCCACGCGGAGTTTTGGAAG

GCTGTGCGTAGCTTGGGGGACGTCCCGGGCATGGTGAAAGCGGACCTCGATCCACTTCTC

CGCCGGTTTGACACAGCTGGTGATGGAAGAGTTTCGCTCTCCGCTCTCATGCATTGGGCT

GGAAGGAACTTTTTGCCGTGTGCTGGCGTAGAGAACGTGGCGCGAAAGAAGATTTTGTCC

GCGGAAGCCACAGCATCACGCCAAGGACAGAAGGTTTCCATTCAGGAAGCATTCTCGGCG

GCTGGTAACCTCGCAGGCGCCCTGAAGGCACTGGGGAATGTGCACCTGACGCCCAGAGAG

ATGTCGGTCTTGCGCAGGCGTTTCGAGAAGGGCACACGAGAAGGACTGGATGTCCCTGCC

ACGTTGCTGTTCTTCGGCCGCGACGAATTCCAGTCTATTTCTCCGACCTCGGCTGACCCc

ccaaacaacgacaacagtcaCAAGAACGAGATGGAAACGGAGGAAGAGTTGCGGCTAAAA

CGCCGGCAAGCGCAAGAAGAAGAGGAACGATCAGCGTCAGATATAGAGCGCAAGCTGAAG

AGCATCGTTCTGAAAGCCGAGAGCATGGGCACGTCCTTGGCTGAAGTATTCGGGGTGTTT

GATAAGGACGGCTCGGGGTTCATCACCGCCGCGGAGCTGGAGGAAGGACTGCGAGAGCTC

CGGGTTTTCGATGCCGTTCCGAAGGACCAGGTGCTGTCCCTGGTTAGAAAGCTCAAGAAG

AGCTCTGGCGGCAACAGTATCGCAGCTGACAATGAGCCATCCTCAAATGAAAAGCGCGTG

GCCAAAGAGGGAGcagacgacgacggcggcggtctTCTCGTCAGCGCCGAGGAATTCGTG

CGGTTCGTGGGGGGCGAGTACGAGGCGGCCGAGGCTGCCCAGGGTCGCCTGCGAAAGGTC

CTGACGCTAGCGGAGGAGAAAGAGGGTGTCACCTTGGAGTCCGCCTTTGGGGCTCTTGAC

AGGAACGGCACTGGTAGCATCTCCGTTGCTGACCTGGAAGAGGGTCTCCGACAGCTAAAT

GTGTTTGATGGGATGTCGAAGGAGCAAGCGCGTTTGGCGACGAGGCGTTTTGATCACAAC

GGGGACGGTACCGTCAGCCTTCCTGAGTTCCTGGCTTTCGTGGGGAGGCCGTACGCTGCG

AACGACAGGCCGTTGGAGGCAAAACTCAGGAGGGTTTTGCTCAAGGCCGAGTCGATGGGG

GTTTCCATGGAGGAAGCCTTCCAGCACTTCGACAAGGATGGCTGCGGCTCTATAACCACT

CAGGGTTTCTCAACAGGCCTGCGGGAGATGGGCGTCTTCCAGGAGTTTTCTGAGGAGGAG

GTTGAGCAGGTGGTATCCAGCTTCGGCGCCGACGGGGACGGTGCCGTCAGTCTGCCAAAA

TTTCTCCGTTTCCTGGGGAAGGAGTACGGAAGAGGCGGAAAgcggggcggtggtgggggc

ACAGGCCGCGGCTTGGCTCAGAGGCTTCGACTTATCCTGAAAAAGGCGCACGAGCTTGGA

ACTCCCCTGTCGGCGTCTTTCGAAGGCTTCGGAGCTGACGAGAGCGGCCGGGTGTCGGCC

CAGCAGCTCCACTCAGCACTTAAAGAAATTGGGCAATTCCGCTGGACCACGGTGGGAGAA

GTGAAGAGCTTTGTTCGCCTCCTACACGACGACGACGTGGTCGACCCCCCCACCAGTTCC

CCGGAAGGTACAACCTCGCCAAAATCCCGGGCCCCCACACAGGACATGGTGCCCGCTAGG

GAGATGTTCTTGACCCTGCCAGCCTTGGAAGCCTTCGTGGAAGGCGGTGACGCCTTTCTC

GAGAAGAAAGAACGCGCCGCGCTGGCTCGACAGCACAGCCGCGATGCGTCATCTCTCGAC

CACAACCGATTGACGCAGACGGAACCCACGGCAAGCAAAGGAGAGGGTGCATCTGCGGGG

GGTGAAGATATGCGACGTCGCGGTATTTCGCGCGCGAGCTCGTGGACGGCTGCGGAAGGC

TCGGAGCCTAGCGCGGGGGGTGGAAGCGCGACCGAGGACGGGGAAGAAAAGGAAAAGGCA

CTGCTAGAGCAATTGATGGCGGTTCTCGCTCTGGTGCCTCACGGGGAAAGCAAACaagcc

agcggcggcggcgatggacaCGGTGTGCGGGGACACTTGGAGTCGTTCGACGTGGATAAC

GACGGTGTCCTGTCTGTAGAAGAATTCATAGCCGCTCTTCGCAGCCTTGGCGCAAGAGGA

GGGAAATTTCGCGGACAGACCGGCGTCGAGACGCTCGTGTCCCGATTCCGCGACGGCCGC

ACGAGCACCGCAGGCGCCCAGAACGGTGCCAGTATCGTTAAAATCGCGTGGTGGTTTGAT

GAGCTATCGAAAGCGGGGGGGAAAGCGCACGACGCGATCGATCGCATGCGCGGCAACATT

GACAAAGGTGGGGGCTCGCGGAGTGCGGAAGAGCGCGAACACGGCCTTAGGAAAGATAAT

TCCGCGGCTGGAGAAACGTTACGGAGAGCGGTACGGCTAGCGGAGGCGAAAGGCACAACC

TTGGAAAGGACGTTTGCTCGTCTGgacgaggatggggacgggTTTATCACGCTTAGGCAG

TTGCTCCGGGGTTTGGATCAGCTGGGCGTGTTTGAACAGGCTAGTCGAGACGACGTTCTC

GACGCCCTGGATGTTCTCGATGCCGAAGCTCGCGCCAGCCGGCCTCGGAAGGGGCGGCAA

GAAGAGGAGTCGAAAGATGGTGGAGAGGTCAACAGCACTGGGGTGGATTTGGTGGCGTTC

ATCAGGCTCATGCGCCAGAAGCCAATTCCCGCACCAGCAACAGAGGCAGGAGAGGAAAAG

GGTAACGAGGACGGAGTATCGCAGATCAAGAGAGATGAGGCGCCGTACGAGTTCTCGCTC

GATCCGGACACCAAAGCAGCGGAGAAAAAGCTGCGCAGGGTTGTCGCGAAGCAGGCAAGA

CTCGGCGTAGACGTCGAAGGCGTTTTCCGTCGGTACGACCCGGAGCAGACCGGATCCGTG

CTTCGGTCCGACTTCGTGCAGGCGGTGATGCAGCTGGGTATAGGGCTCCTGGAGAGCTCT

GCTTCGAGGCACCCGAACAACTTTTCAAACGCAACCGGTCACGCCGCTGATCCCGTAAGA

AGGAGGCAGCTGGGCCAGCTGGCGCGGGCAAAGGGTCCAGTAGAGCGACGACTCATGAGA

ATGCGTCAGAGCAGGCGAAGTCTCTTCGACGGGGGGGGAGCAGCGCGCAGCGGGGAGACG

AAGGGGGATGATGGGAATGCTGCGGCATTCGAGGAAGGAGACGAGAGTCTGGCACTCATA

CAGTGGTATCGTGaagggcagaagaagtccatgGTGCGGCACATCCTTGCTTCGTCTCTC

ACCACCGAGTACAACTTATTCTTCGCCTTCGCCAGCCCTTTGTTCTTTGAGCACCCCCTT

CGGAACCCGTTCAACCACGAAGAACGATTCAGGATCGACCTGGACGACCACCAGCTCAGG

GTGGTGACCGGTACCACGGAGTGGGCATACCTACGAAGGAACATTTCTCCGTGCGTGGGA

GAAATCGGCGACACTCCCCTCGAGTCCGAATTTTTCGACATCGACCCTCAGCGAGGCGTC

CAGGTAACCCTCATGGCCCACGAAGTCGTCTTCATCCCCTTCGCCTTCCTCTCCCTTGAG

CCCCGCCGCCCGGTATCCCTTACATCTACATCTCCTCGAAAAACGCGTCcttctggaggg

ggggggggggcaggggacgggGGTAGAGGGGACAACGCGGCTGGCGAGGGGGGTGCGGGG

ATGGCGGAGCGGTCTGTGGCTGTGGCGTTTGTGTCGGCGTCTCACGGACACGTTGTCAGC

GTGGTTCAGGTACACTTGCAACGGCGACCTTTTGTGGTCAACAGGACGTTCCGATTCTTC

CAAAACGAGGGGGAGATCCTGAAAAGATCGATCCAGCTGCTCCCCGGTGGGGAACCTAGA

CCTAGCTTCTCCAAACGAGTGAAAGCATCGACCCGTGTGGGCGGAGTTTCTCGAGACCCG

CTTGGCCTGTCCGACGTCGGCAGCGCAAAGTACATCCACTGCGTCGAGGAGGCGTCTCCG

GATCGAGACGATGGTGATGGCGGGAGTGGGGTGGTGATTGAGTGGGCCCGGCGGGCTGTG

GTGGAAAGTGAAGCTAGGGGTTGTGCCGACGTCCAGGAGGTGCTCGTGAAGTGCAGGGTG

GGGCAGTTCCCCTCCCAGAAAAGCTTCTACTTGCTTGTTTACAACGACCATTTTCAAGGC

TCGCTGTACGAAACGTGGCACGTCATCGTTCAAGCCAGATTGCGGATGGACGTACACTCT

CTGCTTGGACAACAGAGCCCCGCTGACCTCGTCGTTCGAGGCGACCGCTACACCCGTAAG

GTCAAGGCATTCTCGTCGGCCCCTGCCGAGGTGTCCTTCGAACCAGCGAACGCCTTTCAA

CTCGTTCCTGGGGCTTACAACCGGGTGGGAGTGCGTTTCCGGCCGCTCTCAGTCGGAAGC

CGGAAGATCCACGTGCACCTTGTCGACATGGATTCGATGGAGATCGTCGGGGCCTGGCTa

gcgacagcgacggcgatgccTCCCGTGGTCACCAAATCTTATGAGGTGGACCTTCCCCGA

GACCGCGCTTGTCACAAGCGCATCGCGTACGCCAATCCCTGGAGCTCGACAAGGCTCTTC

CGTCTGGCTTCTAGCGACCCGAAGATCCTCCGTCCGCGTTATGAGAGCCTCGAGGTGTCA

GGGGGTGGGACAGGGTTCCTCCGCTTGTGGTTTGCAGCCCCGGAGGGTGTCAGCGCAAGG

AAGGAGGCATTTTTATTCGTCAACGACAGCGAGGGGCAGAACGAAGAATGCCTCCTAATC

CGCATGCGTGGGGTTTAG

>g440.t1

ATGGAGCAGCTCTTGGAAATAGCCGGTCGTGACATGGATGGGTCATCGGCCGACGGGAGA

GTGATCTTCGGCGAGGAACGAGTCTTGGAAGCCATGGAAGACCTGGAAGACttggaagac

gaggaggaggacgaagaagaCGAGGAGGTAGAAGTCGACGAGGAGGACTtggacgaggac

gacgaggaggaggaggaggaggaggaggatcgcCCGGTGAAACGGCAACGAGGAGCGGAC

GCTCTCTCCCATCTCCAGCAAAATACGATTGCCCCTCGCGGTGGCGGggccgccggcggc

ggcagcagcgccggCGCCCGCGACGCCGAAGGGAGTGGCGCTGGCAGCGCCCTCTCAGGG

AGCGCTGGTGACGGCAGcgacgacagcgacgacgacgacaacgacgaccaaGAGAAAGAG

ATAATCGAGATTCTATCCGACGAAGAGGATGCGCATTCATCGCACGGCCAAACTCCCGCG

AGCTTCGGCCGAGAACCACTCCACCGATCCCGATCAACGACCAGACCGGATTTCGGGGCC

CATTCAGAAGGCGGGCGCGGCTCCCACCAGGGAAGCAGGGACCGCTCTGGCCTCAGCAGT

GACAGACCTGCCTGGAGCTTGAGATATCATGCCGAGCACGGCGGCGCGAGGGATTCACCG

GGCGGCGCTAAGCCGCCGCTGCCCGATTTTCGTTGCAGAGTCCCCCCACGCGATTCTCTT

CCgtcactacaacagcagcaacaccagcaggaccagcagaagcagccacATTTGCCGATG

TCATGGCCCGAACCCTCTAGATCTCGcacaagcggcggcggcgacagcggcggcggcggc

agcggcggcagttttGTTCCGCCGTGGATGGCTCGGAGTTGCTCCCCTCGCAACTCTCCC

GGCCGAGGGGTCGGGGGGGAGGACGGGAAGCCCTCGGCCGAGGACAAGATCGGGGCGGCT

CGGGCGGAGTCGCCCCTGACTTCGTGGGCGGGTGAGGTGGCGGTTTTAGCAGCGGGAGGG

GCGGCGGAAGTGGAAGTGGAAGCGGAAACGGAAGTGGAGACGGCAAAAgtgtcggctgct

gctgctgctgctgctacatctgTGACATCTAACTTGGGAAAACGAAACTCTCCGAgtgat

gatgttgctgctgttggtggcggtggtgatggtgctggtggtgcgacgATCACTCCaact

gtggtggtggcgggggcgggggcggcggcggcagcagcgaccaCGACGACTGAATTCCCG

CTTAACTTGGAGTGCGCTATCTGCTTGAGCCCTATCGCTGTGGCCGCGCTGTTCGCCTGC

GGGCATGGATCGTGCTGGGAGTGCGCTCACGACTGGTGCTCTAGGAATACGAGCCCCAGC

ATGGACTGCCCCACCTGCCACGTGTCTATACCGAGGAGCGACTTCCGACGGTGCATCGTC

TTGGATGAGAATACGAGCCCCAGCATGGACTGCCCCACCTGCCACGTGTCTATACCGAGG

AGCGACTTCCGACGGTGCATCGTCTTGGATGAGGTGGTAGAGCGGGCGGTGGCTGCAGCT

GGCCTCGACGATGAGGAGTGGCGGTCTCGACTAGAGCGCGGGCAGATGCTAGCAAGGGAG

GCGGAGGCCGCCAGACGCGGAGCGAGGGATGCGCAGCGGAGCGCAGAGGAGGCAGTGGTG

GAGCAGGCGGCGGAGATTCAGCGGCTTAGGGAAGAGGCCAGGTCTCGCGCGGTCGGCCGA

TGGCGCCGACCGTACGAGGGCCCCCCGCCGGCCATGACCGACTACGGCTTCCGCGTGATG

AGCGACCTAGACCCAGCATCCCAGAGATCACTGGAGGGTTTCACCCGCCTCCGAGGCCGT

AGAGACGGGGGGGTCGAAGCGGCACGGTCCCGGGCAGGCTCGGCTGCTTCGGAAGCTGCc

gtcgttgccaccgccgcagTCCTAAGCATGCCCacttcggggggagggggagggtatgGC

GCAGTGAGGTCGAGAACTCAGGCGCAGTCGGGGTCGTCGGCAGCTTCGGCGCTTGGGGTT

AATGGGGAGGACCCATGGTTTTCGCTGAGGTCGAGCCAGCCCAGCAGGCACGCTGGCGTG

GCGATGCCGGGCGGCGGTGGACGCATCGGGCGGGAGGTATTCGGGAGGGGCAACGCTTCG

GGGGGAAGAAAGGCTGGATCAGTCAGCAGGGGCGGTCCTCAAATCGGCGCACGAGCGAGA

CGGCTCCAACTGGCGATCCGCCTACTTCGGACCCGGCCAGGGTCCTACGCGCATGCCCCG

CACCGCCCCTGGGGATGGGCACCAACAGTCTTCGGGGGGGCGCGCGCGCAACTCTACCCT

GTCGTCAATCCACACCATGATGGCCGTGCCATCCGGGCCGCTTGCGGTGGAGCCCATGAG

GGGGGCTGTGGCGCCGGGCAGGCGTCCCGCAGCGCAGGCTCCTCCGGCGCGCTCCAGCCG

CAAGAGGCTGAGCTTGAGAGGCtttttgggagagggggggggggggcgcggcggcaACAC

GCACCTCTTGCCGCGAGGGCGTCGGATCCTACTCCCGCTGCGAGGGGGGGTAGCGCCGGC

GGGGACCCCCCGTTCGGTTACAGAGGTTGTCAGCGCGCGAGAAACTCTTCGCACGTTGGT

GGCCACGCGCGTACTGGGGGGACTAGGTGTTGGCGCgggggtgcggcggcggcggcggcg

gcgggagcttCGGGGCTGCGATTCGACCCCGATTTCGACCTGGAACCGCTGGACATGATT

CCCCGTCACGATGACCTGGGCGCTCGGCCCGAGTGGGATGACGTCCTGGCGGCGGTTTCT

TTGCCGCACCAAGCAGCGGCGCCCCGCGATAGTCTGGCGCAAGGAGATTCTCCGGACAGA

ACAAGGGACTGA

>g455.t1

ATGGCCTCTCCGCAAGCCGATGGGGTCCTGCCCTCGCCACCCCAAAACGGGTCCAACTCA

GTCGCCAACTACGTCGGGCTTGGCATGAAGCGCAGACGGAGCGTGCCAAGCTTGAGTGCG

GAAGAGGCACGACGTGCTGTGCCAGACGACGACGAAACTCGACTCGACAAGATGCAGAAG

GCCTGCACAACGTTGCTGGAGTGCCTTGGGGAGGATGTGAGCAGGGAGGGGCTGGTCAAG

ACCCCCAAGAGGATGGCGGAGGCTCTGCTGGCGTGCACACGAGGGTACAGCCAGAGCGTG

GAGGACATCGTGAACGAGGCTGTGTTTGAGGAGGATCACCACGAGATGATCCTTGTGAAG

GACATCGAGATTCACAGCTTGTGCGAGCACCACATGGTACCGTTCTCCGGCAAGGTCCAC

ATCGCTTACATTCCAAGGTCAAAGGTAATCGGGCTGAGCAAGCTCGCGCGTATCGCGGAC

ATGTTCTCACGGCGTCTGCAGGTGCAGGAGCGACTGACGCGCCAGATCGCCGAGGCCATA

CGAGAGGCGGTCAACCCGCTCGGGGTCGGGGTGGTCATCGAGGCCACGCATATGTGCATG

GTCATGCGAGGCGTGCAGAAACAGGGGGCGATCACCATGACGAGCAGCGTTAACGGTTGC

TTCCAGGCGGACTCTAGGACGAGGGCAGAATTTTTCAGCCTGATCGGCCTCAGCCGTTAG

>g752.t1

ATGCCTGCTGGTGAGAACGATCGACGGTTGCTACAGGGCGCGTCCGGCGGAAACTTCGAC

TTAGTGAAGGCCGCTCTTCAAGCTGGAGCCAGCGTTGATGGACCTCCAGAGCAACCCTGT

GCGCCGATCGTAGCTGCAACCATCGCCGATCATGCCGGTATCATCGAACTTTTGCTCGAG

CACGGAGCAGACCCCGACAGGCCTGTCACGGAAGAGCTGCCACATCCAACTTCGGACATG

GCTGATGCCGTTGCTGGTGAGCGGGCCCTGCACATAGCCACCAGGAAGGGGAACATCGAA

ATCGTTCGCTTGCTGCTGGAGCAAGCCCGTGCCAACCCCAACGCCACGGACGACAAAGGC

GGCACCCCACTCTTGGCGGCTTGCGACAGCTCGAATATAGAGGTAGAAGTGGTGCGGTTG

CTGCTCGAAGCGGGCGCCGACCCGACCTTGGCAGAAAAAAACGGGATCGTCCCTCTGCAC

GTTGTTGCCTACCTCGGTTACGTGGACTTGGTCGGCACGTTGCACTCCAGAGCACCTGCC

ACGTTGAACCGCTCCGCTTCCAAAGGCCAAACACCGCTCTTCCTGGCGTGCACCGGAGGC

CATGAGAGCGTCGTGTCTCAGCTGTTGTCGCTCGGGGCGATGCAGCCGGTGCCCGcggac

gacagcaacagcatgtTGCCGCTTACGACCGCCGTGTCCAAAGGCTTCCTGGGCGTGGTG

CGGGTCCTGATCAACGAAAGAGGGATAAGGGCCATCGGGGGCTGGACAGCCGCCTTGCCC

AGTGCGCTGTGCACGGCCGTTCAAGCCCACCAGGCGAGAATCCTCCGGCTGCTGCTCGCG

GCGAGCGGGGAGGACAGGCGGTCAGAGTGGGCCAACACAAACGTCAAGGGCAGGCACCTG

CTTCACTATGCCGCTGGGTACTGCTGTCACGCTGCGGTGGGCGTTCTCctcgaggcgggg

gcgggcgagGCGGCGCGTGACTCGGAGGGGCGTATCCCTCGAGACGTCGTTGGGGTGGAT

ATCGGTCGAGGTGACGGGGTTCGGATAGATCGGGGGAAGGAAGACGACATCCGCCGGATG

CTGCAGCTTGCCCCAGCCTACCGAGCTCGGTCGTGGGCGTGGGTCTCTGATGAAGATGCG

GacgccggtggtggcggtggcggcgctacTAGTAGTGCCGGCATCTCGTCTCCACCAGCA

GTGCCGAAGGCCCCCCTTACCGGTTTACGGATCTTCCGGCCGAAGGAGAAAAACAGTAAT

GATAGTAGCAAGCGCTTCGTGGCGCTTGTCGGCAGGTGA

>g818.t1

ATGAGGGAAGCGGCCACCCCTCTCACGTCCATGAGGAGGGCCACCGGCAAGTCGTCCAGG

GTCCCATTGTTCCCACGGACTCAGCGTTTTTACAACGTGAACAAGCGCCACTGGGGCAGC

AACCTCGAGCTCGCTCGGACCTGCGACTGGCGGCTCCGCACGCCACCCTTCGAGATGTGG

ACGGACTCTCTCGTCCGAGCTACAGACGCGTCCCAGAGACCCTTCACGAGAACCCTCCTC

GCTAGGAACGAGGGTTCTGCGGCATCGTGCTTGGAGGGGGAAGAAGACGCGCCCGGCTTT

gacctcctgctgctgtgctggaggCCCGGGGCAGCGTCGGTCATCCATGACCACCCCCAG

GCTGGCTGCTGGGTCAAGgttttGAAGGGGGAGATAAAGGAGACGCGGTACACCCTGGCA

TCCCCTCCCGGGCACAGCCTTAgcgcctcctcgtcctcctcttccttcatCGCCGACATG

GGCCACCAACTCcaccacgacgaccaccaccaccaccccaccacccacccccaccctctt

aGCGGCGACAGAAGCGGTAGCTGTGGCGTAGGTGTAGGTGTGGGTGTCGGCGCCGGAGCT

GGctgtggtggtagcggtagcggtagcggtggtggtggtggtggtggtggtggtggtggt

gatggtggcggtcgTCTGCTTGAAACGTCGAGCGTGTATTGCCCCCCCGACTCGGTGACT

TACATCGAGGACTCCATGGGGTACCACAAGATGGAGAACGCCAGCTTGACGGAGGAGTGC

ATCTCGCTGCACCTTTATTCTCCGGGCATCTCGGAGTGTACGGCGTGGGCGGACGAGTAT

TCCTTGAGTAACGCCATGAAGGTTTCCATGTGCCTCGACTCAAGCTTCTCACCGGACAAG

GGGCCGTACAGATTCGGCCCCCCTCTGCACTAG

>g873.t1

ATGCAGGAAATCGAGGCGCCGTTGCCTCGCCTGCGAGttacgacggcggcagcagcggcg

gcggcgggggcggcgggggcggcggtgacggTAGCGAGAGAAGTCGCGGCGTTCGCCCGA

AGACACGGAGCGTGTTCTAGCCAGAAGGACCAACATAATAACAGCAGTGGCCACCGCGTC

TCCGCTGCGTCCTTGTTGGTCTCGGGAGGTCTGCTTCTAGGCTTGCTGTTGGGTTCGGAA

GAAGAGGAAGCGCAGTGCGAGCCGGTCGGAACCCAGACAGCGCAAGTTTCTTCGCGTGCA

AGAAAAGCTAAGGCCATGGATAAGATGTCGCCGGAGAGGCAAGAGCTTAGGCGTGTCGCG

CTCGAGCATCTGACCAAGTCCATGCTAGCGAACGAAGCTGCAAGAGCCGACGATGACGGC

GTGAGGACAGACCTCGACGGCAgcgatagcagtagcagtagcggtagcggtagcaacgga

ggtagcggcagcggtggtggtggtggtagcggcggcggcggtgtcccAGCGATAATGAGA

AACGGCGGCGGGAAAAAAGAtggcgatgacgacgacaaTGGCGACCTTCCAACCAACGTA

TACGACCTCGACCGGGTCCTGACCAAGACCCCGCTGGTCCAAGCCGGAAACGCCTTCCAT

GACACCCTGAacgAGGACGACAGGATAAGATGTCCGTACGTGCTGAACAAGCCTGGACCG

GGGGTCGAGGGCATGGAGGCCGTCGTGAGCTTCGGGGAGTCGGTGGCGGGGCATCCTAAG

ATCGTGCACGGTGGGATCACCGCGCTAGCTTTCGACAACTTGCTGGGGATGGCCCTTTTC

TTGCAAGGCGCCACTTCCGTGTTCACAGCTTTCCTTAAGGTGGACTACCAGGCTCCGCTA

CCGTGCCTGACGACAGCTGTGGTGGACGTGACGATCGACAGGAAGGAGGGACGAAAACTG

TTCGTTACCGGAAGACTGAAGAGCCTAGACGGGGCCGTGCAGTACGCGTCGGCTGAGGCT

CTGTTCATAAAGCCCAGAGAATGA

>g1001.t1

ATGGAGGTGGGGTGCTAcaaggacaccagcagcagaatTTTCCAGAAGTACACAAAGGAG

GAGGACCTGGACACCACCTTTTGTGCGGACTACTGCAAAGACTACCCGTACTACGGGCTG

CAAAACGGTAACGAGTGCTGGTGCGGCGAGTCAGGCAGCGACTTCGACCGCCACGGGGAG

GCGACCAACTGCGACACCGCCTGCTCCGGCAACGACAAGATAAACTGCGGTGGACGATAC

GCCATGCAGGTCTTCAGCCAAGTCGTGGGGCTCGGGTGTGTGAAGGATGCCAAGGACCGG

GTGTTCACCGACAAAACTACTTCTTCAGACATGTCACCTGAGTTTTGCCAATCGCACTGC

GCGAGCTTGGGCAAGTTCTACTACGGTACCCAGTACGGAGTGGAGTGCTGGTGCGGGGAG

GAGGGCACGGACTACGACAAGTACGGCTCGGCCACTTGCGACATGCCTTGCTCTGGCGAC

TCCGCGAACTACTGCGGTGGGAGATACGCCATGAACGTCTACCTCACCGGCGACGccacc

accccccctgtGGAGGACTACGCGTGCTACGTCGACAGCAAGGACGACCGCGTGGGGCAC

AGCATGATCACCAACGTGAAGGGCATGACTCACGAGGTGTGCACGGCTTACTGCGAAGAC

CTTGGAGCCTCATGGGCTCTGGTTCAGTACGGTTACGAGTGCTTCTGTGCGGACGAGGGC

ACCGAGGTCGACCGCCTGGGCTCTACGACGTGCGACCACGAATGCACCGGCGACAAGTCG

CAGACATGCGGTGGCCGAGACAGGTTCAACGCCATCCCCATCAGCGGTACTGTCACCCCG

CCGAGCACGTACCCGTGTTACGCTGACGACGTCGGCGACCGCGTGGGGCAGAACATGAAG

GTCGACGCGAAGATGACCTACGAGTACTGCACGGACCACTGCAACGAGTACGATGCTGCC

TGGGCGATGGTCCAGTACGGAGTGGAGTGCTTCTGCGGGTACGAGGATACCGACATCGAC

CGCTTGGGCTCTGCCACGTGCGACATGGACTGCGCCGGCGACGATTCGCAGACATGCGGC

GGCAGGAACGCCTTCAACGCCATCGCGCTCGGTGGGACAGGCGTCGAGCCCTCGCCCGGC

ACACCGACCCAGAGTCCCGTCACTGTGAACCCTGGCCCGCCGGTGCAAACGACATCGCCC

ACCAAGATCGAAACCATCCCCAACTCGTGCTCTGGGGAGAAGTCGGCAATCCGCTTCTCG

GCAGGCAGCGGTATCGGAGAGACCGGGCGCATGTACCTGGAGGACGGCGGCTGCGGCACG

ATGACTGACCTCTACAAGACGCGCGTGGAGAAAGACGGCTGGGAGCCCAAGGGTCCCATC

TACAtcctcgacgaaaacaacgagGCCATGATGCACCCCACCGAGGTGACCGGCAGGTGG

CTCATCACCTCCGAGCTGCACGTGCTCCGCGGTTCCGTATTCTACTGCCGTGGTAAGGGA

AGTGCGGCAGGCGACGGAGACTGCGACGAGCTCCGCATCCAGTCCACCGGCCCCACCGAC

TACCACGAGATCCGCGGGCACGGAGGGTCACTGTACTTTGAGAACACCGTGGTTACCTCT

TGGGACACCCCCAACGGCGCGGTGCAGAAAGTGTACGAGGGCGGGCGGTCGTACTTGAAC

TGCGTCACCGAGTACGAGAGCGATGAGACCTGCGAAGGGCAGCCGAAGAACGACTACGGC

TGGTGCCGCATGGACATCATCAACTCGGAGATGGGGTACATGGGGTGGTTCGATTCCGAG

TCGTACGGGCTGACCTGGAAGGTGCGCGGGTTCTGCCAAGACAAGAGCAACCCGGAAGTG

TTCGACACCAGCAATGTCTTCGGAGACATCATCGACTCCGACATCCACCACATGTACTac

ggcatgtactcgtacgggcACCAGGGGGGCGTCTGGACCAACAACAAAATGCACGACAAC

TACCAGTACGGCTTTGACCCCCACGACGACTCTGACTACCTCACCATCGCCAACAACGTG

GTCTACAACAACGTGAACCACGGTATCATCGCCTCGAAGCGCTGCAACAACGTTGACATC

TACGACAACGAGGTGTACGACGGTGGGGCTGAGGCCGCGGGTATCTTCCTCCACAGGAGC

TCCGACAGCGCCAGGGTTTACAACAACTACATCCACGACATGCAGAACGCCGGGATTGCC

ATTCTGGAGTCGTTCGACGCTGATATCTACAAGAACGAAATCAGGGATGTAAGGTTCGGC

ATCCGCATCAGCCTCGGCGGCGGGAACAACGACGTGCACGACAACACCTTCGACAACTGC

TCCGATTACGGTTTGTACACGTACAAGGGGTCCGATGCGCCGGAGGAGACAGACGACGGC

CGTCcgggggaaaacatgttcgTCGACAACGAGATCACCAACACCGAGGTCGGGGTGAAG

ATCAAGGAGGGAGACAACACCGTCATCACCGGCAACACCTTCACGGGCACGGTAGAGCTC

GAGTTCTTCGACGCGAAGGCCACCTCGTGGTCGAACAACAAACTCCCAGATGGCGTCTGT

GTGGACATCATCACAAGCGACAAAGGGGTAGACATCGACGAGAGCACCTTCGCATCCAGC

AGCGGCTTGCCCACCGCCAGCTGCTAA

>g1165.t1

ATGAGGGGCCGGAGCGTGAACACCCGAGGGTTGGTGGTCAAGACCGGGGAGGGAGGCGCG

GGTAGCGGTTCGAGGGAAGCCGGGGACAACAGCAGCATGGGTCGGTTAGCGTGTTTGTGC

ACTGCGGTAGCCTCCctgttgctgatgctggtggCGCTTGTGTTGCAGCTGGTGGAGGGG

AACTGGTTTCCGCTGCTGGCGAATGCTGCGGCGTTAGTTCTCCAGGCGGTATGCGGGGCA

ATACTGTGGAGGACCTCGACACAGAAAACTTGTCCGGGCTTAAGCTGGTCGCCGGGAGAC

CCGAAGGCGCACGGCGGCCCCGCGCACTCTTCTCCCTCGGCCAGCCCCCGCCAGCTTCAC

CATAACCGCTCGGAAGGAAACTTAAGCTGGACCGATGTAGAGAGCAAGCATACCGGATTG

GGAGTGGCTGCAATGGAGCAAGGTGCAGGCGGCGGGAGCTATAACCCTAAATCGGGTGGA

GTCAGGTCCACTCGTAAAATTTTGGAGACGAGTCAAGCTCGATCAACGAGGCGGGTCGCC

GCGGGACCGGAGGGAGAGGACTCTTCCGAAAAGCAGTTCGGGCTGGAGACTCCGCTGCAG

CAGGTGGCACGGATACTAGAATTTgtgttagacgagtactcgccgGATGGCCGGACACGC

GACAAGCTGGAAACGGTTCTCGACATCATCAGTCTCCCCGAGGACCTGAACACGGTGACA

ATCCAAAGCATCATGAACGCGGACCTAGATTCGTCGACAACCGAGTGGATAGAGGCCCAA

CTGTCTTCGGGGGCTATGGGGGTTGAGAGCTCGAAGAGTGGAAAGGACTGGGGGCGATTG

AAGGCCGGGCTTAGTGGAGTGGTGAACAGGCTAGCGGTGGAGCTCTCCGGGGACCACCGG

GACACGAGCGAGGCTGACACGGGCATGACGTCGGAGCAGCTCCGACGGCAGAAAGAGGTT

GGGCGAGCATACAATATGGAAAAAGCCGCGTCAACTCGGTCCATGCGGCGGAATACCAAC

ATGAACGTCAGCCAGCTCATGACGCTGCCGGGAGCGAGGGTGGCCTCGACGACTTTGTAC

GAGGTAGACGAGTGCAGCCAGGAGTCTGCCGAGGAGGAAAACACCGAACAGCAAGCGCTT

TTGGAGCTGATGCCAGTGTGGCCGGGGATCGAGCCAGAAGTCCAAAAGTCGCTGGTGGAT

TTATTAACCGCGAAGCACTTCTTAGACTGGGGGTTCGACGTTTTCAAGCTCGAGGAACTG

TCCGGGGGGCACGCCCTCTGGTACACCGGTATGCTTACGCTATACCACTACAACTTCGTC

GGCGTTTTCGGCATCAACCCTGCGAATTTGTCGAGATTCTTGAtgcacatcgagcaaaac

tattGCTTCGATCCGGCGGCGCCTAACCCGTACCACACTTCTTTACATGCGGCGGATGTC

ACCTTAACCGTGGCCCACTTTTGCGAAAACTCGATAGTTTCTTCGGGGATCAAGGCTTTG

CAGGGATTCGCCCTGCTCGTGTCAGCGATCGTACACGACTACCGACACCGAGGGGTGAAC

AACGGCTTCTTGACAAAGTCGAAGGACAGCTTAGCGATCACGTACAACGATGCGTCTGTG

CTGGAGAACTTCCACATCGCCGAGGCCTTCAAGATTATCTACGACGACCGCACGGACATC

CTCAGCGGCCTCACCATCGACGACATGCGAAATTTTCGTCAATTCGTCATTAAGATAATC

TTGGCGACCGATCTAGCTCACGGGTTCGAGTACGTGGCGAGGTTTAAAGCCTCCACCAAC

ACGAAGGGGGGACGGAGACCGAGCATCGTGCCGCACATGGCGAGGGACGGAGCGAAAGGC

TCGGGAGTTCCAGGGTTCGGGGCAGGCGGGGGGACGTCTGCCGCGAAGGGGGGGGCACAG

GGCAAGGACCCGCTGCAGGCGCAAATCCTGCTGATGCAAATGGTTTTGAAGGTGGCGGAC

GTGGCCCATCCGATGAGGCCGTGGGATCTGCATGCGAGATGGACGAAGCTCATCACGGAG

GAGTTCCACCGACAAGGCGACCTTGAGGCGGAGCAAGGGTTCCCCATCTCCCCGCTCTGC

GGGCGAGAAGGGCACAACCAGGCCAAGAGCCAGTGCGACTTCGTGAACTTTGTGGTTCGC

CCGTGTGCGACGGTGTTTTCCGAATTTTGCAAGGATAAAGTGTGGGTGAACACCCTTGAG

AGCAACTACCGTAGGTGA

>g1303.t1

atgaagCGCAACACTTCTTCGACGACGAGCCTGACGGACATGGGCGTGAAGGACAGCGGC

GACAACAAGCTCAACATCATCGAGACGGGCGAGGAGGGGACGCTCGACTACCGCCTCCGC

TACGCCATGGAGGAGCACGCGCACagCGAGCAAGTAATCTCTGTGTGGCACGACATCCCT

CTCTACGTCCGCGACGAAGACGACAAGGCGACCGGTAACCTCAACTTCGTGTGCGAGATC

CCCCGCTGCTCCCGCAAGAAATTCGAGATCTCCACCACGGAGGCCGGCAACCCGATCAAG

CAGGACACCAAGAAGGGGCTGCTCAGGGAGTTCAAGAAGGGCGACATATTTTTCAACTAC

GGGTGCTTCCCGCGAACTTGGGAGGACCCTGACCACGTCCACCCCGACACGGGTTACCAG

GGGGACAACGACCCTCTTGACGTGTGCGAGATCGGTCTCCGCATCGTTGCCACGGGAGAC

GTGAGGCAGGTCAAGGTGCTTGGTGTGCTGGCCATGATCGATGAAGACGAGACGGACTGG

AAGGTGATCGTCATTGACAAGGAAGACCGGTGGGCGCCGGAGCTGAACGATGTGGAGGAC

GTGGAGCGTCTCCTGCCCGGCGTGGTCTCTGCCATTCGCGAGTGGTTCCGCACGTACAAG

GTACCCGACGGCAAGCCGCTAAACAAATTCGCGCTGGACGAGAAATGCATGGGACGATCA

TACGCGATGAAGGTTGTCTCGGAGTGCCACATCTCGTGGCGGAACCTTGTGGGCAAGGCC

GAGCTGGGGGGGGAGATCAAGCACAACCTCTCCGTCTCGAGGCTGAGCGACCTCGCCTCC

GACATCGAGCCGGAGGCCATGATCGATGCCTTCGCGGACGATGCGGATGacgatgaggag

gggggggcggaggcgggagaggaggaTGGTGTCTACCCCACCTCTCTCATCGCGTGA

>g1499.t1

ATGGCAATGATTCTGGGAACCGCAGGTCTACCAGCATCAGACATGAGCGTGCGCATAACC

GCCCACACGAACCGAATGGAATGGGCGCCATCTCCGTCTCCCTCGGTCTGGAGGAAGCGC

GTGGCCCTGGAAGGTCCTCAAGAGTCTGGCCGTGTGACGTCCGTGGTCCGGTACGACGCC

AACAGCGAGTTCAGGGAGCACGGCCACCCCGACGGCGAGGAAATCCTCGTGCTTGAGGGG

TCTTGGCAAGATGCAAACGGCGATCACCCAGCTGGCACGTACATGCTGAACCCGGAGGGT

TTCCATCATGCGCCCTCCTCCAAAGACGGCTGTGTGCTCTGGGTCAAGCTCCGCCAGCAC

GGTGGTCCCTCCCGGGTGCACTCCCGCCTTGACACGAAGAACATGCCGTGGGCAGACACG

GACAGGCCGGGGGTGTCAGTCAAGGCCCTGTACTCTCACCCTGACCACGACGAGTCCATG

CGCATCGAGAAGTGGGAAGCCGGCGCCAAGGTGGAACACCCGTGCTCGAAGCTCACCGAG

ATGCTGATCATCGAAGGAGAGGCACACGACGGAAGTGAGGCCTTCGAACAGCGGAGCTGG

CTTAGCGTGCCCGCGGGGGGCACGGTTTCGATGACCACGGCCACCGGGTGCGAGGTGTTC

GTGAAGGAGGGGGAGTGGCGCCCCCCTGCCGACCAGGCCTGA

>g1620.t1

ATGCAGGAGAACGCGTTTTTGAGCGACTATTTCGCATGCCTTGGGCTTCTTCCCCTCGCG

AGCGCAAGCATGGTTCGGCAGGCGATGGTCAATATCATGCTTGGTTCCATGCCTCAACAG

CGTCAAGAAAGGTCTCCTGGCAAAGCAGGTGGCCTCGTGGGGAGAGAGCGGGGTGGTGTT

CCGCCGCAGGGCCAGGATGGGCGTTGGGGTCACGTGTTGCCGTGTCAAGATGGGACGGAC

TCTCCTTCCAAGTGCATCTTCTGGTGTGCGATCGCTCTCGGGGCGCTCGTGCAGGGTTAC

TCCGCGGCATTTGTGGCGGGGTACCTGAAGCTAGCGCGCGATTCTCTGGCGGACTGCTTC

GACGGAACCACCGTCGAAACGGCCAGGGCCTACCTCGTCATGGCCGTCGTGTACAGATTT

CTGGGGGATCAGGAGAGTAACCAGGAGCATCTCGCCTTCGCGAGCAGCATCATGGAGCGA

CTGCCCCAGGAGGCGACCCCTTCGGCGAAATCATTCGTGAATGAGAGGGACCTCTGGGAC

TTCTATTTCTCTGCGGGTAAATGCATGCACAAGGCGTTCTTGGAGGACGTTCGAAGCATG

GACGATTCGGCGAACACCACGGGAGAGGCCGGGGCGGCACACGATACGGTGATGGAGGGA

GAACCGGGAGATTGTCAAGAGCAAGAAACATACGGGAGGATAAAGAGGAAGAGGGGCTTG

GAGGGAGGCAGCCTCGGGGCGCCGCTGCGGTCGAGTAGCGTTGTCGGCGAACCAGCCAAG

CGCCGGGGTCGACGGGATCCCCCCCCTGCTGGAGCGGCTATGAAGGCCTGTGTCGAGGAT

ACCTTGCCGGAAATGACGAGTCTCTTAGCGGCAATCGAAAGGTCGGGCAAGCGTTCAGGA

GTGGGCACGTTTCTCCTCCACGGCTGCATCGGTTACATGAAGGCCGTGAAGGGCGAGATG

CGCGGCTCCTTCGAGTCGATGATAGTCTGCGCCGGGATGCTGATGCGCTTTCCCGGGATA

TGCCGCTACAGGCCGCACCTAGCGCACTCCGTGCTGACAGCCGCAGCCTCAAGCATGCGC

CGGGACGTGTACGACCCGGTTAGGGAGATCTACAACTCGATACTGCCTTGGAATGGCACG

CCTGCACCGCCGATGGAAGACTGGGGCTTGAAGAATTCCATCTGCAACCATGTATTCTGT

AGGTCTTTGCTGGCCGAAACCAACAACATGGACTTTGCGTCCAACAGCTTGGTTTCTGCA

AGACCTGTCACCCCTCCGGTCCCCGCCGCACCTTTGTCCGACCTCCATACGATTTCGGTC

ATCGGAGACAATCACTGA

>g1658.t1

ATGCAACTCGATGCGGTAGACGAGATGACGGAAGCGTTGAAAGGCACTCAAGTCAACGAG

GTGAATGCTGAAGAGATCGAAGACAGCGATGAGGATGCGACGAGGCACGGGGGCGGTGCC

CCCCCGTCCTACGTGGAACTTTCGTCGCACTTcgtttctctggagagcgcagcggaggcG

ATCGCCTGGGCCAACATCGAGTTCGAGGTGTCGGACGCGATCTTCCACAGCAACAACGGA

GAGCGGACGTCCGCAGCCGCGTTTCACAAGACCCTGCCGCACGACGAACTGGGACAAGTT

GACCCCGTGGCGTTCGACGCCATGGTCCAGTGCGTTGCCACGGCCGACTTTGACACGTGC

GAAGACGTGCCGGCTGGGGGACCCGCCTCCCTCGCCAACCCTCTCGGGGGCATAGCTACG

GATATGGCCGGTCCCGCTAGTTCTGCCCTTCCCATTCCACCTGTGCCGGTCCTCAGCTCT

GAAGAACTGGCTTCTCAGCTGGCTGAGGTTTACTGGATGGCCTTGGCGAGGGACGTCCCG

TTTTCTAAGTACGGGGATGACGATATCACGGTCACCGCAGCAGCCAACCTGGCTGGCATG

CCAGGGTTTGCCCAGATGCCCGGGGTAGCCGTGGGGGAGGACGGCGCGCCTGACCCCCTC

ACCCAGCTCTTCCGGAGCACCGCCTTCGGGGTCGAGACAGGACCGTTCATATCCCAGCTT

CTCGTGCAGGACTTCAGCCTGGACTCAATCACGGTCACGCCTAAGCAGACCACGCTCGCC

CCGGGCGTTGACTACATGACGGCCTACGACCAGTGGCTTTTCATTCAGAACGGCGGGTTC

GATCCCGAGCCGGAGATAGAAGACCCGATTACGCGGTTCCTCCGTAATGCCCGCGACGTA

GCAAGGATGGTGGCCGTCGACACCATCTACACGTAA

>g1659.t1

ATGATCCTCTTGGAGATCGATGCCATCAGCCGCCCCGGAGCCAACGGCCCCTACAGCACC

GAGACTCGCCAGGAAGGCTTCGTCTCCTACGGCACCTCCCACCTCATGAAGCTCATCGGC

ACCGGGGAGATGGCCCAGAGGTCTTCGTGGTACCAGAAGTGGAACGTGCACATGTTCCCA

CGCCCAGAGGCCCTTGCAGGTACACTCCACAATGTTTTGGTTGGCGACCTGAACGTGGAT

TTCGACCCATCGATCATCAACAACACGGAGTTGCTAGACAGGGTGGCTATTCACAACGAG

GCGCAGAACGGGGTGGGGCCCGAGGGACGTACCTTCCTCATGCCCCAGAACGGCGGGTTC

GATCCCGAGCCGGAGATAGAAGACCCGATTACGCGGTTCCTCCGAAACGCCCGCGACGTA

GCAAGGATGGTGGCCGTCGACACCATCTACACCGAGGCGTTTCGAGGCGCAATGATTCTC

TTGGAGATCGACGCTATCAGCCGCCCCGGAGCGAACGGCCCCTACAGCACCGAGACTCGC

CAGGAAGGATTCGTCTCCTACGGCACCTCCCACCTCATGAAGCTCATCGGCACCGGGGAG

ATGGCCCAGAGGTCTTCGTGGTACCAGAAGTGGAACGTGCACATGTTCCCACGCCCAGAG

GCCCTTGCAGGTACACTCCACAATGTTTTGGTTGGCGACCTGAACGTGGATTTCGACCCA

TCGATCATCAACAACACGGAGTTGCTAGACAGGGTGGCTATTCACAACGAGGCGCAGAAC

GGGGTGGGGCCCGAGGGACGTACCTTCCTCATGCCCCAGGCGGCGAGGAATGGGTCGCCA

GCGCACCCTTCCTATCCTTCTGGCCACGCCGTCCAGAATGGGGCCTTCGCAACCGTGCTG

AAGgcTCTTGTCGGCCTGGACCGAGGCGCCACGTGCTTCGAGCGGCCCGTCTACCCCGAC

CCCGACGGGCAGGAGCTTCTGTTCTTCACCGGCGACGAGTGCCTTACAATCGCGGGAGAG

ATCAACAAGATGGCTGTCAACGTCGCGTTCGGCAGGCAGATGCTAGGGATTCACTACAGG

TTCGACGGCCAGGAAGGGCTGACCCTCGGGGAGATGGTCGCTGTGCGGCTGCTGCACCAG

GAGCTCATATCGCTCCCGGAGAACGAGCCCTACGAGTTCCGTCTCATGTTTGGCCAGATC

ATCAGGCTCGAATCCGATGGGTCGTTCTTCGTGGACGGAGTTCGATGCACCGAGGAGGCG

TACATTGGGGCCGACAAGTGCCTTCAGTAA

>g1661.t1

ATGAGTACATGTTTGTCCACTCTCTTCCGTCGTGCGACGTGCCAACAGACACCAGAGGGC

CCTTATGGAGGCACCGCCGCGACGGTCCCCGGCGTGATCGAATCTGAAGAATTTGACTAC

GGCGGAGAAGGCGTGGGATACTCGGACTCGACGTTCGTCAACATTCAACAGGATTTCAGG

CTGAACGAGTCCGTCGACGTCAATGCCATGAGCGGAGGAGGATACAATGTCGGGAAAATC

ACCGCCGGCGAATACCTCCGGTACACCGTGCACGTTACGGAAGATGtCGACCAGTTCTAC

TTCAACTTTGCGGTGGCATCAAACGAAACTACAGGCTCTTTTCGCATTGTCTCCGGCGGC

GACGGCTGCGACGACTACGACACAGACCTCAGCGGGGTCGTGACGGTGCCGAATACTGGG

GGGTGGGAAGACTTCGACGATTTAGCAGTAACCGGAGAAATTGGCGCTGGTCTTGACGCT

GGTGAGGCTTACATCTGGTTTTGCGCACTTACCGAGAACTTCAACATCGACTACTTCTCG

ATGGCGACATCTCCTGCGCCGGAAGGGGCCTACCGAGGCGACCCTGCGGTAGTCCCAGGC

CTCATAGAGGCAGAGGATTTTGACAATGGTGGAGAAGGCGTGGGATACTCAGACTCGACT

GCCGTCAACATTCAACAGGATTATAGACTGGACGAAGCTGTCGACGTCAATGCCATGAGC

ACAGGAGGATACAATGTCGGCAAAATTACCGCTGGAGAATATCTCCGGTACACAGTGGTC

GTTAAGGAAGACGTCGACGCCTTCTTCTTCAACTTCGTGGTATCATCGAACGAAACTACT

GGCTCTTTCCGAATTGTCTCCGGCGGCGCCGGGTGCCACGACTATACCACTGACCTCAGC

GGGGTCGTGATGGTACCGAACACAGGGGGATGGGATTATTTCGAAGACGTAGCAGTATCG

GGGGAAGGTGATGGTGGACTCGTCGTTGGTTCTACCTACATCTGGTTCTGCGCTCTCACC

GAGAACTTCAACATCGACTCCTTCACGATGACGACAACCCCTGCACAGGAAGGAGCCTAC

GGAGGTGTCCCTGCAACAGTTCCAGGCCTCATCGAGGCGGAGGAGTTTGACTACGGCGGA

GAAGGCGTGGGGTACTCAGACTCGACGATCGCCAACATTCAACAGGACTTTAGACTGGAC

GAAGCCGTGGACATCAATGCCTTGGAGGGAGGGGGTTACAATGTCGGCAAAATTACCGCC

GGCGAATATCTCCGGTACACCGTGGACGTTACGGCAGACGTCGACTCCTTCTACTTCAAC

TTCGTGTTGTCATCAAACGAAACTACCGGCTCTTTCCGAATTGTCTCCGGCGGCACCGGG

TGCGACGACTATACCACGGATCTCAGCGGGGTCGTGATGGTGCCGAATACCGGAGGATGG

GAAGACTTCGATAGTGTATCAGTACGCGGGGAAGGGGATGGTGGTCTTCAGCCGGGGCTA

ACCTACATCTGGTTCTGCGCACTCACCGAGAACTTCAACATCGACTCATTCACGATGACC

ACATCGCCTGCACCGGAAGGGGCCTTTGAAGGAGTCCCTGCCACCGTCCCCGGCCGGATC

GAGGCTGAAGAGTTTGATACCGGTGGTGAAGGCGTTGGTTACTCAGACTCGACCACCAAC

AACATTCCACAGGAATTTAGACTGGACGAGGCCGTCGACGTCCACGCTTCGGCCGGAGGT

GGCTACAATGTCGGCAACATCACCTCCGGGGAATACCTCCGATACACAGTGGACGTCATG

GCCAACGTCGACGAGTTTTACTTCGGCTTCATGGTGTCTTCAGACGAAGACACGGGCTCG

TTCCGCATAGTCTCCGGCGGCACCGGGTGCGCCGACTTTACGACCGATCTCAGCGGGATC

GTGACGGTGCCGAACACCGGAGGACTGCAACAGTACGAGGATGTGGCAGTCTCTGGCGGG

GGCAACGGTGGCCTTACCGCTGGAACGGCCTACGTATGGCTCTGCGTACTTTCCGAGACG

TTCAGCATCGACTCCTTCACTATGGACACGGTGCCATCTCTCCCCTTTGGCGGCACCCCT

GCAACCGTTCCGGGTCTGATCGAGGCGGAGGAGTACGACATGGGCGGTCAAGGGGTGGCG

TACAATGACACGACCATTGAAAACAGTGGCAACGCGTTCCGGACGGACGAAAGCGTTGAC

GTTGGGAGCACCGATGATGGCGGTTACTTTGTTGGCTGGGTGAAAGAGGGCGAATTCCTT

CACTACACAGTGGACGTTTCGGAAGACGTTGACGCGTTTATCTTTACTTTCCTGGTGTCG

GCCCCGGTGAGTAGCCCTGGCGCGTTCCGCATCGTCACTGGTGGTACTGATTGCGACGAC

TTCACTACCGATCTCAGCGGCCTCGTGTCGGTTCCCGGGGAAGGGGGCTCTGTCTACAAT

GAAGTCATAACCTACGACGCAGAAGCTGGCGGTCTGTCTGCCGGCGTATCTGTGATTCGG

CTGTGCGTTGTCACTGAATTCTTCAACATCGACTCCTTCAGCATGACAGAGCTCATGGAT

CGTTAA

>g1958.t1

ATGCGAAGGCTGAGCAATCGCCATGCCAACCGCCGTCTCTGCATCGCTTTCCTCCTAGTG

CTAGCAGTAGTGGCCTTCTTGCGCTCATGGCGTCAAGTCGCGCTCGAGCTCCCATCTTCC

AATGGTGACGTCTCAATCTCCACCAGCGGAGGCGAAATTGAAAGCTCGGCGTGCGAATGG

CAACAGGGCGGACCGCAGCTCTACATGCTGCAGCACACGAAGAAGCACCCGAAAGCTTCC

CACTGGTTCCACATAGCCGAGTGCTTCACCGGCCGTCGCGCGGACTTGAAGGAAACCATC

GCCGGCATTAATGGGACCGTCTATCTGCAGGTGTTGGACTCTGGCTGGATGGCGCACTTG

TCGCCCATGAGCCGACTCCTCATCATGGCGGGCCTGACGAGCATCGGCACCGACGTTGTC

CACTTCATCGACGCCGACCGAGCCATCTTCTCCTACCCCTCCGAACAACagcctccttct

cttcctgaaaaccaacaccaccagcagagtATCAACGCCGCCAGCACCACCCCGGCCCAC

CTACGTGCCGCCGAAAACGGTCGAGATACTGAAgacggaaaaaaagaagacgaaaccGTC

GGTAGAGATATGGAGGCCGAACTGGGGCAGCCACCCCCGGGGCAGGTGGGACTCTTGCAG

TTCAACTCGGCCGGCCGCGGAACCGAGTTCATTTTGTCGCTCGACCAATCTCTCCCCGAG

CGATTAACGCTTTCCGCAGAAGTGGAAACCCTCgacggggagggtgggggggaagaATCG

GCGGCTGTTTCTGGGGGGGCGGGaagcgggagcgggggagggggggggggggggggaggg

ggagggggtgggggggggcaggaaggggGCCGGGATGCCTCGGGGTCGGTGTGTGTGAAG

TACCTGTCGACGGTCGGGACTGCGTCGGTCCAGAGGATCGACTGGTTTCCTCGTCGCGAG

GATGCGTTGTCGATGCGGAGGGCGATTGATGTGTATTGTCCCATCCCGTGGCTGAGGGAT

GGTGGTGGCATGGGGAGCGTTAGAGGCGAGGATGGGGCGgtggcgggaggcgggggggag

ggggcggaggagggagggcaTGAGCGGTACAGGCGAAGAATAGAAGCAGCGGGCGacggt

ggtcgggggggggcaggggagggggggcgcgttcGGCGTCTGCTGCTTTATCAGCGTGAC

CAAAACAGGAAGATGGTGCACGTGGAACAGGTCACCGAGAAGCTGCGCGAAAGGCTGGGA

GCGGGCTGGGTCGTGGAGGAGATGACGCACGATGACTCCAGACACCCGTGCGAGTTGGTG

GAGGAGCTGGCCAGCGTGGATGTGCTCCTGACGGCGCACGGGTTtcagTCGATGCTGGGC

CTTTTCATGCGGCCGGGCTCTCTCCTCTTCGAGGTGTTCCCGCACAAGTACTTCAAGGCG

GGGTACGCGCCCATGGCGGAGGGGCTGGGCGTGCGACACGCCTACTCGGAAAGCCGGGCT

CGGCTCCCGATGATGGGCTTCTCGCACCCCAGCACCGAGACCTGTATGAACTGGTACCTG

TGCCGCTGGTACGCTCGCTCTGCGAATGTGGAGTTCGACGACGAAAGTTTGGAGCGCCTG

GTCAGCTTGGCGTTGCACCCCAACGGCGGGGGGAGGTTTTAA

>g1976.t1

ATGAAGAAAGAAGAGCGGCCGGCCgagatgatgacgacgacgaagaaggtagaggcggcg

gcggccgcaccGCCGCCGCACAAGCGGAAGTGCTCTACGCACTTGGAGGAGCTGGAAAAA

ATGCTTGAGATATCGTTGATGCCGGCTTCTTCTCCTTATTCTTCGCGAAAGACGTCATTC

GCAAACTGCATCAGTGCCGCCAGGAGGCCCAGCTGTGAAGGACTCTCGACAACGGCATCC

CCGCGTCACGGAGCACACGCcgtagtcgcagcagcagcagcagcagcagcagctgcgtcg

GCGGGGCGGTCGGACCGCCACGGATCATACGTGGACCAGGGAGAGGGTGCGGCGGGCATG

ACGGaggaaacagaagaaaaaaatcgaggaggaggaggagccgagGGGACAGGTGATGgc

aatgacgatgatgatgatgatggtgagtaTGATGTTGACGAAGTGAGTCTCTTGCGCGAG

CTGACGCGGCAGCAGGAGGAAGACCTACAGATGGCGGCGATGATCGGCCAAAAGCTCCTC

GATACACAGGAAGAGCTCCACGCGGAGCTAGAGgcGGTGCGAGAGGAGAAATGGTTGGTG

AACTGCGAGAAGGACGCGCTCAAGAGAGATTTAGAGCGGGCGACGCAACGCTGCGAGCAA

ATGCAGAGCGAGGCGACGCTCCAGGTGGCCCCAGgcgatCCGCCGGAAGAATTCGACCTG

TGGTCTccagcggcagccgcggcagccgcAGCTGCCGCAGCTTCAGCttcagcttcagca

gcagcagcagcaacagcagcccctGTCCCAGGCGTATCTCCGTCCATCTCCCCCCCGAAC

ATGGCTTACTCCGGcttcgccaccgccgcggcgGACGAGGTCACCCGGTTGCACCAGCTG

GTGGAAGACTTAGAGCTGGCCAACGGCACCCTAAACGAGAAGCTGATCGAGTCCCACCTC

GAAACGAAAGCGTTGAGACGCGCCTCGGACGAATGGCGCGTCGGGCAACTCGTGCTGGAC

CAGAAAGCCATGGTTGTCGACGACAAGACATCCGAGGCGCAGGAACGCCAGTCTGTTGGT

GCGGCGGCACCGGAGGGAAGCGGGCTAGACAAAAAACCGTGGGTGGACGGCATGTCGAGC

GCCTTCGGGTGGCTGGGCCAGGGAGACCAGCAGCGGGATAGGCCTGGGGAGTCTCTGCGT

ACAGATGAAACGTCGAAAGAAGGCGGCCAAGAGGAAGAACTATCTCTGAGAGGCCCAGGG

CAAGGTCTTACGGCGCACTGCAGTTCATCCGTGGGCGGCGATCGACTAGAAGCCGGTCGG

GCAAGCGCGGGAGCGTCTGCGGATGCTCGAGGGGGAGAAACGAGCCAGTCGGCGCGCGAC

CCGGGATGTGAACCGGACCAAGAAACGTCTACACGCCCGTCGCAGGAGAATATTTACGCG

TCGGCAGTATCGCCGTCGTCGCCATCGAAAGTGCTCAGGCGGTTGCCGTCAATTACCGAA

GGACCGGATGAGCAGGCCGAACTGATCGATGCCCTACGCGGACAGGTCGACCAACTCCGA

CTCGAGGCGCAAAGAGCGGGGTCGGTTACATCCTTACCGGGCGCGAGAAGGGGGACGAGG

AGTGCAGAGCTCCAAGACTGCAGACGACGGGGTTCCTCTAGACACTCGTCAgacgaggac

agcagcagcgacaacagcagcaacgacgacagcggcaacgccaacgccaacggtaGCGGT

GGGCTTGAAACGTGTCCGCCACCGACTAGTAGCCTGAACACAGCCGGAGAGGAGAGGAGC

AGCCCTGTCGGCGACACCAACATCAGCATCGACGACGGCCAGACCATACGGATGCTTCAA

GCGCGCGTGCGAGACCTGTCGGCCGAGCTTATGACGGCGAATCTCGAGCTACTGAGACTC

CGCGAGCAAGCCCGGACGGACCAGGACGACATGACCCGACTGCAGAGAGAGAACTTGAGC

TCCCATCGTTTGGGACTGAACAGCGCGATggtggccgcggcggccgcgcgCGCCTCCGCT

ACAGCGGCTTCATCCGGCGCCCGACCCGATTGTCAACGGTGCGGCTACAGCCCCCGTGCC

GATGAGGACAGCAGTTGCCTCAACCATGACGGATTGCGCTGGATTCATGGGTTGTGGCTG

AGGCTCGCGAATGGACTCGGTGGTGAGCAGAGGAGGAACGGGGAAACGGAGAGATTTCGC

GGGGAGCGGTGGAGGTATGGTTCCGCGGGATACGAgtgggggcgcggggagggCAACAGA

CTCCGGTTCGACGTCGACGAAAGAGCGTCTCTActtcaacaagaagaagaagaagtaaaA

CATACTTATTGA

>g2010.t1

ATGGCGCCTGGAGTGACAAGGAACCCGGCggggctactgctgtgccgaccgCCTGTCCAT

GAGGAACAGTCCAATGAGGTGGTGGAGCGGCAGTCCAATCGCGTAATGCGCCTCTACGAG

AAAAGCGTGATTTACTGGCGCTCCACCCCGAGGGCGGACCTTCCCGCGTCTGTGCCCTGG

CACATGATTCCCTTGAGGCCCAGGCACCACAGGCAGCTCCAGGAAGGAGAGGTCGAGAGG

AGCTACACGGGAGgcggagggaaggagagggcgGACTACGAGCCGGCCGCGCTCTACTTC

TTGTCCCGGCGCTTCTTGACGGAGCCCAAAGTCACCGCGAATCGACGCGTCGTCGTGGTC

GGAGCGTCCTCCACGGCCACCGCCTGCCTGGAAGGGCTGGTATTCACCCCGTACCTCAAC

TTCACCAGCCTTACCCTCGTCTCTCCCGACGGGATCCCTCCGCCGAATGACGCCCCCCAA

ACGGAAACCCCGGGCGCTTAcgctagcggcggcggtgaaggAATTCACGGGAAGGCAGGG

GTGGCAACGACAGGCGGCGAGACGAAGACTCCGGCATCGTCAATGGCGGAGGGCGCTGGC

GCTGACCACGGGCCTGACATGCGCCGCGACAGCGGGTGCCGGGCGCGATTAACTCCCCAC

GATGAAGACGCTCCCGACGTCAACCGGTTCGCTAGGTTGAGCCTGGAGCGTCACGTTCGA

ATCGTCAGGTACAAGGCCCACCGAGTGGCCCAAGCTTTCGAGCTGGCCCACATCATCCGG

CTCACCAGCAAGAGCCCTTTGGTTCTACTCCTGGACGACACGTCTCGCCGCTTAGGGTTG

TGGGCCCCCGGTTTGAACCCTTCCGCCCCCGACGTTCCGGGCTTcgtctccctcgactct

ccatTCCTCCACGCCGACGTCGAGGAGAACCTTGCCGGCATCCCTCCAGACGCGAGGGTG

GTGGTGTACGCGGGCGCCCTCAAAGGTTTGTGCGCGGCGCATGGGCTCATCGATCGGGGG

GTAGACCCCGGAAGGATCACCCTGGTTCGTCCTCTTTCGCCCGGGATAAGCCAAGCGGCA

CGAAAGGCGCCGCCGGTGTATGGAAACGCCTCACAAGGGTCGTGGTCTCTGGGGGACGCG

TCCGTCGACATGGCGATGGCGGAGGCAGTCGCTGCGGCCGGGATCAATGACGTCGGCTAC

CGCTTGCTCGAAGGTGTACGCTTGGACGCCGAGGGAGGCGTCGCGGCTGCAGTCTTCGCC

GATTTAGGGGCAGGTACCGGTGGGAATTCCGAAGGTGATGCACAAGACGGGCGAAGGCAC

CGGCCTGTGTCGGCCGCTGCAGGGGCCGCGGCAGGGGGGGAGTATACCCGGGGGGGCACG

GGGGAGAACCTTGACGATGACACTGAGGGGGCAAGCCAGGGGGATGCTCTCACGTGTGGG

ATGCTGCTTTGCGGGGACACGCCGAACGTCGACCCTGACGTGTTCCGCGCGGTCAACAAC

AGCGGTCTCGTGTACGACGGGCGCCTCGTAGTCGACCCGATGTTCAGGACCTCTGACCCG

ACGATACTGGCCGGAGGGACGCTCACGAAGTACTCCCGGGTGCACGGGGCCGGCGCGCCC

AAACACGAGAACTACAACGCAAGAGAGGTCGGCACGCATCTCGCCACCTGCGTGCTACAT

CAAACCGACCCCCTCTCGCTCGTCGATGGGAGCGTCAGCGACTCCTCGCGGTACACCGAC

AGCGCTTCGCTGGAAggtgcgggcggcggcggcggcggcggcaatccCTCTGGTCCCGGC

TCCCGGGGCTTCGGTGTCCAGCCTCTACCTCTCCCGAAGTTCGTACAACCTCGATGCGTT

AGCGGGATGTTCCCTGGTGGGTTGCGGTACGTTCGAGCAACGCTACCGCCAACGGATGAG

TCGTGGAAGACTGTCGGTAGTGACGGACAAAGCGACAGAGGAGGCGAAGAGGAGGATGCA

GCGGGGGAGGGAGACAGCGGCAGAGCCGACGAGAGGGGTGCGATAAAGTGTTTCACAACC

GGAGAAGTCGTCTttaacagcggcagcggcagcggcagcggcagaggtGGCGACACGAAG

GAGGAAAACGCCAAGGGGGTCGGCGGATTCTGCTTGGTGAGGGCGGACCCGCTGGACCGG

TTGTGCGACATAATTTATTGTGGCCAAGGGGCCGTGGAGGCGACAAACTTGAGCAAGCTT

GTGGGAGTACAGCTGGGCTACCTTCAGGGCATGGAAGCTGCGTTCGAGCGCGGAGACGTT

TCTGACTGGGTAGAATTCTTTCGGCAAGGCGCCTTAACACCTCTATGCCACGACCGTTTC

CCAAAGCTCACGGAAGAACTGAGGGAGACCTTGTTGAACGGGGACGAGGCAGCCGCGAAC

CTTCTCTCGGTTCTtgacaagGGAATCAACGAGGGCAAGGCCGACGAGTGGTTAGCGTTA

ACGACGCGCAACGTAGTCGGACCAGGCGCCCGCGAGCTACCACCGTCCACGCGAAAAACC

GTCGAAGGTCGGGCCCTGGAATGGGTGCGACGGAATCGTGGAATGCTCCCGCGATTGGCG

CTGCCTCCGCCTGCCGCACACGCCGCAGGGGGAGGGGTTGGCGGTAGTAGCGGGATGGCG

GGCGGTAAGGCCAGGACGTAG

>g2021.t1

ATGATTCGAGCCTTAAGGACATCATGGGTGAGATGTGACGACGAAAATTACTCGGAGGAG

TTTCGAGATGCATGTGTGGGAAATTCGGCGGTCAATCGCGTTAGCTTCGTCGACGCGGCG

TTCTTCCTACTGATGGCGCTGTGCGCGTGCTGCTCGCCGGCGTTCAACAACGGCTTCTGG

GGGTTCAAGTCGATGGTGTGGGCCTTGCTCCTCGTGGCGTCGATCTTCATCTCgaacgag

tttttcgacggtTACGTCTGGGTGGCGAGGGTCGGGGCCTTCATCTTCACGATCATGCAG

CAGATCGTGCTCATCGATCTCGCCTACAGGGTCAACGATGGCTTGGTCGACATGGCTAAC

GCCAGCGGGGACGGGTCCGACGAAGGGAAGCCGTATCTGGTGGCCTTGATTGCCGGTAGC

GGCTTTCTTTTCGCGGGGTCGCTGTCCGGCATCGGGGTGATGTTCCACTACTTCAGCGGC

TGTTCTGCGAACGAGCTGGTCATCTCGCTGACGCTCATCCTGGCCGTGGCGGTGACGCTT

CTTCAGCTTTCCGGCGGCGAGGGGAACCTTCTCGCGTCCGCTGTGGTGACCGCCTACGCG

ACCTTCCTCTGCTTTTCGGCGGTTTCGAAAACGCCGACTTCCGACTGCAACCCCTTCGTG

GGGGAGTCGAACCTCACCGTCGTGCTGATTGGTCTAGGGCTTACCCTGCTGTCTCTCATC

TGGATAACTTTGAACGCGGCAAGGACCGTGACTTCTCTGCTCGGTGGGAACGCCGACCAG

GTCCAGGAGGCTTCCGGGCCAGTCCAGCCGGGGGGCTCCCTCGAGTCGGGCGACAAAAGC

CGTCCCTTCGTGCAGGATGCTGTGCCCGTGGGGGACGGCATGGGGGAACCCGCTCCGGCC

AGGCCCGCCGCCGGCGCTAGTGCCGCGGGGCAAGGCGCCGTTCAAGACGATGGCGGTTTG

GGCTGGAGGTTCAACGTAGTTATGGTACTGATCTCCATGTTCTACGCCATGATGCTGACG

AACTGGGGGGATATTAACGTCGACGGCGAATCCAGCGACCCCAAGAACGGGTGGACGGCC

ATGTGGCTTACCACCACGGGGCAGTGGGTCTGCTTCATCATCTACGGCTGGACGCTCATT

GCACCCCGCATTTTCCCCGACAGAGACTtttcttaa

>g2304.t1

ATGATGATCTCGCCCGCGACGACGAACAAGAACCTGCAGTTTCAGGGCGACTTTGAAAAA

GAGTTTTGGCCGTGCTCGCTTCACAGCGCGTTCGAAGGTCACCATGCGTTCAAAGCAGTC

TTCCTGGAAGAAGAGCGTTCGAGTGCCAAGTTTCCTCGGCCTCGTGCATCTGGCGGATCT

GGCGGGTCGGAGAAGATATTCCCCGGGAAAATCGTTTCGCTGTCTGAAGACGGCGCCGCG

GCTACTACGCCGAGGGTCACGGGGAAGTGCGCTGACACCgctgaagctgttgttgctcct

gccACCGGTGCCGATTTCGCGCTGACCCGTGCTTCCAAAGGCTTGGTTTCCGTAATCATG

ACTGCGTACAACAGGCACTGCGATTTAGTGCTCAGCCCGGACGATGTGTGGCTGACCATC

CTTGCCCAGTTTTGTGCTTACGTCAACAAGAACGCGGAGGGGTTGCGGAACAGGATCGTC

CAACACGAAGGCAAAACGGAACTCAAGGCGTACTGCAACGGGACGCTGGAAACCGCGGAC

TACGCGTCCATGATCCGGGATCTATTGGTGGAGATCAGGAGTCATATCAAGTCGCCCGAG

CTCGCCGACTGGTTTCGGCCGGGATTCTCAACCACGACGGAAAAAGACGAGGTCTGCGCA

GCGGCGACCGCCATGGCGAGCTTACAGGCGTATTTCGAATTTACGATGCATCTGAGGTGC

GGCATCCCCTCGGTGACTCTGCTGGGCACCGTCGCGGACTGGAAGCTACTGCGGGAGAAG

ATCGAGCGCCTGCGTGACTTCGAGGTGCAAGGAAACCCGGAAGGAAACGTGATGGAACTG

TGGGTGGGTTACCTGCGGAAGGTGTGCGACGGGTTCGTGGAGTCAGCGGAGCACCCGGAC

AGCGCCAAAACCCTCGAGTTCTGGGACAAGGTTGTCTCTCATCGTGGCGGGGGTTCAGGA

TCGAGGTACATCACCGGCTGGTTGACCGCTTTCACGTGCTTTGACCAGAAAGGGAGGTTT

CTCGGCAAATGCAAGGACGATGGCCGGGACATCTGGATGGACATGCCCGTCGGGGAGAAA

AAGAGCGAGGGCGGAGATTTCCCGTTGATTGACCTTGACGATATTTGCCACAACGTGGTC

TCATGCCCTGTGAAGATCGACGACAATGGGGTTGAGTATGACGGCACGCTGTTCGTGGGC

CAGGTGGCGTTCGAGGCCGAGCAGGGCGGGGGGAAGGATTACCCCACTGTCCGCCCGCGC

AACGActggtgcatggccgtagcggcGAAGGAGTGA

>g2308.t1

ATGATGATCTCGCCCGCGACGACGAACAAGAACCTGCACTTTGAGGGCGAGTTTGAAGAG

GAGTTTTGGCCGTGCTCGCTTCACAGCGCGTTCGAATGTCACCATGCGTTCGAAGCAGTA

TTCCTGGAAGAAGAGCCTTCGAGTGACGAGTTTCCTCGGCGTTGTGCATCTTGCGGAGGA

TGTGGCGGGTCGAAGAAGAAATTCCCCGGGAAAATCGTTTCGCTGTCTAAAGATGGCGCC

GCGGCTACGCCGAGGGTCACGGGGATTTGCGCTGACACTGCTGAAGCTGTTGCTCCTGGC

GCCGGTGCCGATTTCGCGCTGACGCGTGCCTCCAAAGGCTTGGTTTCCGTAATCATGACT

GCGTACAACAGGCACTGCAATTTGGTACTCAGCCCGGACGATGTGTGGTTGACCATCCTG

GCCCAGTTTTGTGCATACGTCAACAAGAACGCGGAGGGGTTGCGGAACAGGGTCGTCCAA

CACGAAGGCAAGAAAGAGCTCAAGGCGTACTGCATCGGGTCGCTGGAAACCGCGGACTAC

GCGTCCATGATCCGGGATCTGTTAGTGGAGATCAGGAATAACATCAAGTCGCCCGAGCTC

GCCGACTGGTTCCGGCCGGGGTTCTCAACTACGACGGAAAAAGACGAGGTCTGCGCAGCG

GCGACCGCGATGGCGAGCTTGCAGGCGTATTTTGAATATACGATGCATATGTTATGCGGA

ATCCCCTCGGTGACTATGCTGGGAACCGTCGCGGACTGGAAGTTGCTGCGGGAGAAGATC

GAGCGCCTGCTTGAGTTCGAGGTGCAAGAAAACCCGGAAGGCAACGTGATGGAGCTGTGG

GTGGGTTATCTGCGGAAGGTGTGCGATGGGTTCGTGGAGTCAGCGGAGCACCCGGACAGC

GCCAAAACCCTCGAGTTCTGGGACAAGGTTGTCTGTCATCGTGGCGGGGGTTCAGGATCG

AGGAGCATCACCGGCTGGTTGACCGCTTTCACGTGCTTTGACCAGAAAGGGAGGTTCCTC

GGGAAATGCAAGGACGACGGCCGGGACGTCTGGATGAACATGCGCGTCGGGGAGAACAAG

AGCGAGGGCGGAGATTTCCCGTTGATTGACGTTGACGATATTTGCCACAACGTGGTCTCG

TGCCCCGTGAAGATCGACGACAATGGGGTTGAGTATGACGGCACGCTGTTCGTGGGCCAG

GTGGCGTTCGAGGCCGAGCAGGGCGGGGGGAAGGATTACCCCACTGTCCGCCCGCGCAAC

GActggtgcatggccgtagcggcGAAGGAGTGA

>g2624.t1

ATGATAGCGTCACGCGTGTCGGTTTCAGCATTCCTCGCGGTGGCCATCACGGCAGTAGTT

CTGCTCCCGACAGCAGCACATGCTGGTGGCAGGGATGTCCGCATCACGTATGCGGAGGAA

GACGGCACGGTTAAGACGAACGAGTTCGACCTCCTGGTGGTGGCCTGCGACCCAGGAGCC

CTATCTAACGTTTTGCACGGGAAAGCGGCCCTAGAAGAGCGGGTGGAGACCGCCCTCGAG

AGGTACACGCTAGGGACGTCGCTGTGGGATGTGCGACGAAAAGAGGGGGAGGGCAACACG

TACACGATACGAATGTCGCCTGACCACCTGTCTGCCGGCGACGGACACGTGTATTTAATC

CGGGACGAGCCGCGGGCACGCACGGCCCGCAGCAGCCGGCACACTCTAGTCACGGCGTAC

CAATTCGCGAACACATCTATAAGCGAGGACGAGTTCACAAGCAAAGCGACGGCCGCGATG

GATAGCAGCTCTGGCGAGTACGACTGGCTGGGCCTCGAGCCGACGACGAAGCTGCTAGAG

GTGGAGACGGCATCGGACTACTTCCCACACTTCTCTCAGGAGGATATTCAGGCGGGACTT

CCGTGGGCTGTTTTGGACGCCCAGGGGACCCACAACACCATCTACGTGTCGAGCTTCGGG

TCGTTCGAGTCCGTCCTagccgtgtacgagtacgggAACATGCTCGAAAACACCACACAG

GTCCAGAGCGCGTTCGAGGCCGCGAGGCTTGCCAGCACCAAACCACGCGTGGCGGTTATC

GGGGCTGGGCCGTCAGGGCTGCTGTTCGCTAGCCAGCAGCTAAAGAGAGCAGGGCTGGAA

GACATCACCATCTTCGAGAAGCAAGATAAGTGGGGCGGCAAAACGCGCACGCACTACTAC

AAGGCTCCGGGTTTCCCCGAGGAGGCGAAGGTCGCGGCGGAAATGGGCACCTGTTACCTC

AGCAACACGTACAAAGCTTTGTCCGAGATGCTGGTGACCAAATACGGCCTGAAGAAACCC

GCCCGCATCAACTACGGCGCAGAAGACCCCGGACCACGAGCCATCTTCAACACGTCCAGC

GAAGAGTTCCTGCGTTACCCAATCTGGATTCTGGAGAAACCCGACGGCACACAGATGTCA

AAGGAGGAGTTTGCTGCCGGGATAGCCACCTACATCGGCGCTCACTACTCCCTCGCTGGT

TGGAGCATGGACATCGGCGTGCCCATCCCGCCCAGCCAGCCGCAGGATCTTCCTGATATG

ACATTCCTTCAATATCTGGAGTCTATTGGAATCGGGCACCTCAAGAACGCCTTCTTGTAC

GGCCACTCAGCCCAAGGATACGGCGACATCAGTGAGATCCCGGCGTACTACGGGCTTATC

TGGGTTGCGCCCGACGGGTTTACGGCTCAACTAGACGTCGCGGCATCCATCTCCGAGCAA

TTGGCGGCGGCAGAGGAAGACGCAGAAGCTAGCCTCCGCCTGGCGGAAGGCGCCTCGATA

TTGAGGCACGGCGGCAGGGTTGCTCGAACGTTCAAGGAGAGCGCCGTGCGGATGCTAGAA

AGCGGTGTGCGTCCTAGCGTCGACCAGGCCACGCGTTTGGCAACAAGGTGCTACGAGGAC

GTCCTGGCAGAGGGTGAGGACGAGGAAGTTTCACAAAATGCCGACGACGCCGAAGCGGCG

TATCTTATTCAGGAAGGCTGGGGGGAAGTTTGGGACAGGATCGTGCACCAAGACCAGCTG

CGCAACAAGATCAAGCTTGGGGTTAACGTCATGAAGATTGAACGCAAAGGCCTTGGGGAT

TACGTCTAA

>g2723.t1

ATGACAGCAGACGGTACCGACTATATCCTCATCATCCTCGAAGGCATGGACGGACCGTAC

ACGTTCATGGGTGCCAATGACGGCAAGCAACTCCCGAATGACGTGCAGCCCTTTACTCTC

CCGAACGAAGACCATGCCGAGGGATCgagcgacgaggaggacgaggacgaaacggagggG

GACAACCCCGGGCGACTTCGTGGATTGGACGACGTAGCTATGCTTGGCGATTATGGTTTC

TATGCTGGCTATGTAGCGAGCGCGTTCACGCTCGGCCGTTTCGTAAGCTGTTATGCTTTA

GGTTACATCACAGATTCCGCCGGGAGGAAACCTGTGATCGTCGGGGGGTTGCTGTCGATC

ACCGTGTGCTCCTTGGCGTTCGGGTTCTCACCTACGTTTGGTTTCGCCATTGCATCGAGA

CTCGTTCTCGGCCTGACGAATGGGATGGCCCCAGCCCTGAGAACCGCGATGCGAGAGGTT

TGCGGACAGGAACACGTACTTCAAGGAATGACCTACATCGCTGGAGCTAAATCAGTCAGC

TTGGTTTTGGGCACCGGGATTGGCGGTCTCCTGGCCCAGCCGACTGTGAACTACCCCAGC

CTTTTTCCTGCAGACGGCCTATTTGGCAAGTACCCGTTCCTGCTCCCCAACTTGGTAGGG

GCGGTTGTggcccttctgctgctgccgatcgTTGTGTTGTTCGTTCCCGAGACAAAACAT

TTCGATGagcagcgtgctgctgaaaATGGTCCTGTCAGGTGTGATGACATCGAGGTGGCC

GAGCCAACTCAACAATCGACAGCTTCGTCTGACCTGGATACGCTCACGGATCACCCCATC

GACAGCAAGGGCACGGACACAATCAAGGGACACCGCGATGGGGGGCGGTACGCCAAGATC

GACGAGGAACAGCCCGGCACGCGGCCGCCCGGTGGACTACTGGCAGTGCCTCACGTGAAG

ATTGTTCTCTTTCTCGTGTGCGTCGTGCAGGCGCTGCTGATCGGGTTTGAGGAGGCTTAC

CCTCTATGGGCGCTCAGTACCCCTGATGTGGGAGGATTGGGCTGGGACAGCGTCGAGATA

GGCAAGGTTTTCCTCGCGGCTGGCGTGATCGTAGCAGTGCTACAGCTCTTTGTAGTTCCT

CGCCTCATCAAGGTGCTCGGACTCACGGTTTGGCAGCGGATTGGCTGTCTTCTGGCCATA

CCTGCCTTCATCGCCGTCCCCTTCTCCAGGAGCATCAGCTGGAATGACGGTAGCTTGTTT

ATCGTTTCGGTCGCGAGTACATCTCTCGTGTATTGCTGCATGGCCATGGTTAACCTAGCC

TTGGCTACTTCTTCGACTACTCTGGTTCGCGCAGCTATGCGTGGCAAGCTTGCAGGGCTG

TTCAACATGGCCGAGAACCTCGGCCGTTTCAGCGGGCCAATCGGCTTTGCCACCATGTTC

GCGTGGTCCGTGTCTCCGTCCGCTTCCAGCTGGGTAGGTCACCGGTTCGTGTTTCTTTTT

GCGGCCTCTTCCATGTCTGTCGTGGCTGTCCTCGCGTGGGGGGCGATCACTCACAAGAAC

ATGACGCCCGAAGAAGAATGCACAAGCGACGTTGTCGATGAGGACCGGGTAGGGATTGAG

CTTTTGAGCCCAAGTAGTTCCCACTGA

>g2836.t1

ATGCCTACTCTTATCGAGCAAATCGGCACCGGGGTCATCACCGGGGACAAGGTGATGGAG

CTCTTCGCCGCGGCGAAGAAGATCGGTTTCGCCATCCCCGCGGTGAACGTCACCTCGTCC

TCCACCGCAAACGCTGTcatggaggctgccgcatcggTCAACTCGCCCATCGTCAttcag

GTGTCGAACGGCGGCGCCGCTTTCTTCGCCGGCAAGAGCATCAACAACAAGGAGCCCAAG

GAGCAGGCCTCCATCGCCGGGGCAGTGGCCATGGCCCTGCACGTGCGACAGATGTCCAAG

TTTTACGGCATCCCGGTGGTGCTGCACTCCGACCACTGCGCGAAAAAGCTCCTGCCGTGG

TTCGACGGCATGCTCGAGGCGGACGAGGAGTACTTCGCGAAGAACGGGGAGCCTTTGTTC

TCATCGCACATGCTCGATCTCTCGGAGGAGGTGGACGAGGAGAACATCAGCACCTGCAAG

GCTTACCTCGAGCGTATGGACAAGATGAAGATCTGGCTGGAGATGGAGATCGGTATCACG

GGCGGCGAGGAAGACGGTGTGGACAACACTGGCATGGACCAGAACAAGCTTTACACCCAG

CccgagcagATCTGGGCTGTACAGGAAGCCCTGGGGTCCGTCACCCCCAACTTCAGCATC

GCCGCTGCCTTCGGAAACGTCCACGGCGTGTACAAGCCGGGAAACGTCAAGCTGAACCCC

GGCATCCTCGCGACCCACCAGGAGTTCGTGAAGGAGAAGCTTGGAGAGGGCGCGGACGAG

CGGCCGTGCATTTTCGTGTTCCACGGGGGGTCGGGATCGTCCGAGGAGGACATCCAAATC

GCCGTGAAGGCTGGCGTGGTGAAGATGAACGTCGACACTGACACACAGTGGACGTACTGG

GAGGGCATCAAGAacttttaccagGACAAGGAGGGCTACCTGCAGGGGCAGATTGGCAAC

CCGGACGGGGATGACAAGCCCAACAAGAAGTTCTACGACCCGCGTGTGTGGGTCCGCAAG

GCGGAGGAGAGCATGCGCGACCGCGTGAAGAAGGCGTGCGAGGACCTCGGCAACATCAAC

ACGCTCTCCAAGGAGGTCTAA

>g3244.t1

ATGCCGACGTTTGCCGACACGAAGAATGCTGACGGCAATCGCTCAGCAAGAAGCGCGGCG

ATAGGCCCGGCCACGGTGCAAGGCTTTAGCAAGGACCTGGAGGGCCAGGGCGCCCTTGCT

AGAAGCGGACGTGGTGTCGTTGCAGGTACCACCATCATGGATGCGCAGACAGACACCGAC

GACCTTGACGCGGACCTGGGGCCGGACGACGTCGAGGCGCACATGTCGGTGTTTACATTC

CTTAAAATCGTGACTTTCTGTTCCGCGCTGTACATGTCGACGAACCCGTGGTTCTTGTTC

TACGACCCCGACGAACAAGAGATCTGCCCAACCACGGTCCTTTGCGCAACAACCTTCGTG

GAAATGGGCCTCCTGACGGGGTCACGCATATCTGCGGGAGTGCTGTTCGCGAGCCTTACG

ATCGCCATCTTCTCCAAGTGCTACGCCACGCGAGCGTTCCTGCACAACTCCTGGCTAGGC

GCCGTGCTCGACTTTGAGCCAACTCACGATATCCACACCTACTTCGGGTTCCTTTCCCTC

GGAGCTGCCGCGGAGCACGCCATCCTGCACATTGTCCGGTTCATCTATGCAGGCAACTCG

CACCTCATATACGAGACGGACACCGGTCGCTCCGGCTTGGTGGGGTGCGTTCTGATGCTT

CCCATTGTACTGCCTATGAAGTACGAGTGGATCAAGAGGAGGATGTCCTTCCAGACCAGG

AAATACCTGCACATGACGTTTCTGCCGCTGATGATCGCACTGTGCTTCCACAGCGTCGTA

TTCCGCTACGTGGGATCCATCCTCATCGTGTGGTATGTCGTCGACCGGCTGTACTTCACC

ACCAAACAGGGTTACACGTTCAAGGCTGGGTCGTACATCTACGTCAACTCGCCTACGATC

AGTCGTAGCGAATGGCATCCGTTCTCGATCATCCAGGTGCCCGGCAAAGTGCCCAGGGCT

GCTTTCTACGTCgaggcgGTGGGAGACTGGACGCAAGACCTGTTCAGCCTCGGCCTGGAG

CAGCCTCGCCTTCCGCTGTGGATCACCGCCGCGCAACCCAGCATCATGGAGAAGTCCATC

TACTTCGACAACATCGTCCTGGTATGCACTGGGGCTGGGATCACACCCGCTGTATCTATC

ATCGCGAGGTTCGCCAAGAGGAAGAACATCCACCTTTTGTGGATGTCGAGGGACGGTGGT

CTGGTAGCTCTGTTCGAAAAACAGCTCCGCCAGGTGAACAGCAAGGTGCACCTTACCGGT

AAGCAGTCCGAGGAGACCAAGACCGCCCTGCAAAGGCTTCTCCGGTCATCTAACAAGCAC

GTGAGCGACCATGAGCGAGATGTGCTGAAGTAG

>g3245.t1

ATGCACGGCAAGCAGCGCGTGATGAGGAAGGCGAGCCTCAGGTCCCTCGAGATGAGCCAC

TCCTCCAACTCTAAGCCGTCGAGACATTCAACGATCGAGGAAGACAGTGACCATGCGATC

GACGAAATGAAGACCGCTCACGGTGACACTGCTGTCAACTTCCAACTGCCTGCACAGCGG

GAGGCGGTTATCTCGGATGCTATCGGACTCTCGACGGCCTCGGTTCGACGTCTTCGCCGT

ATCAACTCCATCAGCAACAAACCGACCGATCCCGTGACTGTGAGTTTCGGACGCCCGGAC

ATCGGCGCATACCTCAAGGAAACGTTCACCACGCAGACGGAGATGCTGGACGAAAGCGGC

ATGGACAGATCTAGCGCGATGATCGATGGCTCACCCATTACGCCTGTTTCTACCGTGTCC

CTGCTGAATGCGCGCGATGCTTACCAGGAGTCGAAGCGCCTGGACGCCGACCAGAAGTTC

TCGAAGCAGACGCTCATGAAGGCGGACACCAAAGCCGTGGAAGCTTGGCAACAGCAACGA

GGCAACGGGGCCAACAACAAGGCAAACGCCGGCTCCAAGTCTCCCACCAACGCCGGACCT

AGCCCCATGTCCCAGTGGCTCGTGCTCTACTGTGGTGCCAACCCCAAGGTCGAGGAGGCC

ATCGCCACAACCTGCGACGAGCTCCACGTCAACTGGCAGAAGGAATACTTCGGGGAATGG

TGA

>g3247.t1

ATGCGAAGTGCCGCTGTGGGCCCGCCCGCAGCAACTCACCGTAACACCATCGACCTGGAG

AGCCAGCCGGCTGTTGCTAAAAGGGTTTCTGGGGCCGTTACGGCCACCGCCGTTCcggaa

gggggaaaaaagggtGACGCCGATGCCGACGAAAGACAGGACGGTGTTGATATGCAGATA

TCTGTGCTAACCTTCATGAAAATCGTGACGGTTCTTTCAGCGCTGTACATGGCCGTGAAC

CCGTGGTTCTTGATGTACGACGCCGACACGGGAGACATCTGCCCCTACACGGTGCTCTGC

GCAACAAGCACCGTGGAAATAATTCTCCTAATGGGGTCGCGCCTTTCTGCAGGGGCACTA

CTGGCGAGCCTCGCGATATCCATCCTCTCCAAGTGCTACGCCACGCGTTCGTTCTTGCAC

CACTCCTGGATCGGCGCCGTGCTAGACTTCGAACCATCTCATACTGCCCACACCTATTTC

GGGTATTTATCCCTTGTGGCGGCCCTGGAGCACGGCGCACTGCACTGTGCTCGCTTCATC

TACGCGAAACAGGCGAATCTCATCTACCAGACAGCTACAGGCCGTTCGGGCGTCATCGGG

TGCCTTTTCCTGCTGCCCATAGTGCTGCCTATGAAAACGGAATACCTCAGAACCAAGATA

TCGTTCCAAGTCAGGAAGGTCCTGCATCTACTGTACATTCCATTGACAGTGGCCCTATGC

TTCCACAGCACCACCTTCCGCTACTTCGGGTCTATCCTCGTCGTGTGGGCTATCCTCGAC

CGCGCGTACTTCACCACCAAACAGACCTTTCTTATTGCGCACCCTGTTTTTCAAGCTGTC

GGGCGCGGAACGAGGGTGATTCTGGACCTGCCTTCGGGATATACATTTAAGGCAGGATCA

TACATTTACGTCAATTCACCAATGATCAGCCGGAGTGAGTGGCACCCGTTTTCAATCATA

CAAGTGCCCGGCGAGGTTTCCAGGGCAACGTTCTACGCAGAGGCGGTAGGAGACTGGACC

CAGGCACTGTTTCACCAAGGCCTTGAACAGCCTCGCCTTCCGCTGTGGATAACCGCGGCA

CAACCCAGCCTGATGGAGAAGTCCATCTACTACGACAACGTTGTGCTGGTGTGCACCGGG

GCCGGCATTACACCCGCTGTATCAATCATTGAGAGGTTTACCAAGCGAAAGAACATCCAC

CTGCTATGGATGTCGAGAGACAGCAGCATGGTGGCGCTATTCGAAAAACAGCTCCGGGAG

GTGAAAAGCACGGTTCACATTACTGGGAAGCAGACCAAAGAGACCAAGAACGCTCTCCAA

CGGCTGCTGCGTTCTTCGAACAAAACCACGAGCTCCATCCAGAGAGATGCACCGCATCCG

TGCTACTGCCACCTCCACGGCACGGTCGCGACGTACAACAAGAGCTTTGGGTCTATCCCA

CCGTTCGAGTCCTTCGCGTCTCTTTCACCGAGAATCAGggcGGATAATACTAGCGACACG

TCCGACGATGAAACGAAGATTGATCACCGCGACACGGAAGACCCAGACTTCCTTCCGCCT

AAGGAACAGCTTTTTGTTGATGCAAGAAGACCCTCGACAGACTCGCGGGGTCAGCGGTGC

TTCAGTAACTCGACAACTATGGCAACCACGGGTCCGGTGACACTGGTGTTTGGACGCCCG

AACATCGGTAAATACCTAACCGAGGCGTTCGCCATGCATGCAGAACGTGGAGAGCTGCCT

TGCACCGATGAGGAAATCAACCAGGGCCTATGTGACGCAATGGCGGACGGAGTTTCCTTG

GGAAGGTGTTCCGAGGAACACGAGGGGTTGAAGGATATGGAGAGATCGAGGCGCCTGGAC

ACCGAGCAGGAAACCTCCAAGGAGACGTTGATGAAGGCGGATGCGAAAGTCGTCAAAGTG

GTCCAACAGCAGCGCCGCGGGAGTCGGGTCATCGCCACCCACGACGACACCCAAGACAAG

GTCGTGGCCAACAAGAACAATCGAGACAACAACTGCACATTTCCATCCAGTTCCGGCTGC

CCGCTCGTTCTCTACTGCGGTGCCAACCCTAAGGTTGAGGAGGCCATCGCCAAAACGTGC

GACGAGTTCGGCGTCAAATGGCAGAAGGAGTACTTCGGGGCTTGGTGA

>g3474.t1

ATGTTCGCTGCATCAGCCTCGGTGTTGCTTCTTCTGCTCTCGATCCTATGTGCAACAACA

GCCGGGGCCGACTCAGCGGGGAGCACTGCAAGTGGCGGTGGCATtcgcagtagtacgagt

agaaggGCGGCGGGAGAAGGGCCGATGGTTGAGATTTATGGACCTGCCGAGGGGCACAGG

GCGTCCGCAGATGGGACCTCTCCGGCTAGTGGAGCAAGCAAGGGTGGGGAGTTCACGGAA

AAGGAGGCGGGAGGCTCAGGGGAGGCGGCCCCGAGCGTTTCGTCTCTGGGCATGACTATC

CGCAACGAGTACAAGGAAATCTCGGGCTCTCCATGGCCCTACGTCGCCGAGCCCTTCAAA

AAGTCCACCCTTCGCGCCACCTCGACGCTGGGAAACCTCGGGGCGGACTTCTTTACGTGG

ACGTTCGCGGATGGCAGGGTGTTGGAAGGAAGGGAGGTGACGCACATGTTCAAGGAGACC

GGAGTGCAGTCCGTCTCCCTCTCGCAAACGGTGGTGTCCACGGGAGAAACGTCGACGATG

ACGGCCACCGTTATGGTCAAGTACGTGCGACGAGAGATACGGCAGCTGACGCCGCAAGAC

AGGGAGGCGTTTCTTGATGCCATGGAGACCATCTACCGGGTGTCCCCAGCCGCCGGGGAA

AGCCGGTACGGATCTGAATACAAGGGCATCGGCTATTTCGTCGGCATGCACCTTGAGGGG

GCCGGTGTCACGGACTGCGACCACTGGCACGACGACGCAGGCATCATGACCCACCACGTC

GGCTTCACCCTTTTGTTCGAGCAAGCGCTGCAGGTCATCAACCCCGCCGTCTCCATCCCT

TACTGGGAGTACACCATCGACGCATCTAAGGGGCTGGACTCGTACGGGGAGTCCATGGTG

TTCGACGACGACTGGCTGGGAGCCGCTTCTCCCGACAACGACTTCCACACCGTCGCCAAG

GGCCGCTGGGCGTTTCTACCCGTTAAGCAGGAAGCGTGGTCAAATGTCCACAACCCGTAC

GGCCTCCTTCGCGCTCCTTGGAATACCGACCCGACGCCTTACGTTACCCGCCACAACCTC

ACTAATGGCGAGTCTGATACGGACATGGTGACCTGCGACGTGTACCAGAGGTGCTTCGAA

ACGGACAGCCTCGCGGGCATGAACAACTGCCTGAACGGAGGAACGCACGGTCCGGTTCAC

ATCATCACCGGCGGCGAGTGGGACGACCCCGAAGAAGACCTCATCACGGCTCTCGGGATC

AACGCAATGGTTCCGCTGGTGACAAAGTACTTGTGGCGGAAGGGATACCTGAGGCTGCCC

GAGCACTGCACAGAGGATGAGCACGGCATCGGCCGCTTTTCGACCTGTCGTGCTAGCTGC

CCCGTCGAGCTGTACGAGTCCCGCGGGATGACGCCGTACGACGTTCTCATGGACACCATG

GCGCTGTACTGGGCCGCCCCGTTCACCGGCGGAGTCATCAAATACCTTTCCGATGAGGAC

CGATTCGCGATCGCCGGACACGAGGACGACGAAGTGTACCAGCAGACCGTGTGGAGGAAA

CTACTCTTGGCTCTCTGCGATCCCGGCCATGTGGGGGACATGTACACCTCTTCCGCGCCG

TACGACCCTCTCTTCTGGGTCATCCACCCGGCGGCGGAGCGCTTGCTCTCTTGGAGGAGA

AAGGTGGCAGAGGATGGGGTGGACGGTTACGGCTTCAGCGAGGTTTGGGGCTACACGCAC

GGACACGTCGTGGGAGAAACGGGCATGGTGTGCAACTGGGCGGACGTCCGAGAGGGCTCC

CTCGATATGCCCACCTGCACCAAAGGCATCTGTGGTGGCCACGCGGCGGTGGACGTGCTT

CCCTTCAAAGTCAAGGTCAAAGGAGAATACATTACGATGTCGAACATCCAGTGGCTGGCG

TTCATCTATCCCGATAATGAAGACCTCCCGTACATGTACAACGAATACCGCTGGGATCAC

TGCGCAGCGTCGGCTTTCTCCCCACCTGTGCATGGCCTCTCCACACGCCTCCCCGTCCCC

CTTGCAACTCCCATCTTGGGTGCTCTTGTTCACAGCGCGGACGGGAACTCGATGGGAACC

GTGACCTAA

>g3513.t1

ATGAAGACCTCCTTCGTTGGGCTCGGACTAGCGGCCGTCGCGGCCTCGTCAGTCAATGCC

GTCAAGTACGACGTCGCGACGTGCGCCGAGCTCGCGGACATCGATGACGCCACCGTTACC

TCCCTCAGCATCACCAGCGAGTACTTCGAGTGCGACTCGTACACTCGGTTCCAGGTGCAC

AACGACATGACAATCCAGTCTGACAGGGCCGGCGTGGTCTTCATCAACTTCGCGCTGGAA

GTGCTGGGCGACCTCGTCGTGGAGCCGGACGTGACATTCAGGGACGTCACCGAACAGGAG

ACGGACGGTGGCGTGCTGCGCGTGAGAAAGGGCTCGTCGGCAACCTTCCTAGGGGAGACT

CGCTTTTTGGAGAACAGCATAATCTCTCCCGAGCTCCCCTCCCATTCTTCTGGAGACAGC

GACGACAGCACGGACTACACGGTGATAAGAAGCGGCGGAGCCATCTACAACGAGGGTGAG

CTGGTATTCGAAGGTCTCGTGAAATTCGCGGAGAACAGGGCGCTCGCACCAGAGACGACC

CACAACGTTGGGAGGGGTGGTGCcatctacaacgccgggaccgGGAGCATCTTGTTCAAC

GGGCCGATCAGCATGCGCTGGAACATCGCAGCTGGCTGGGGTTCCCACGCCGGGAAAGGC

GGCGCCATCTACAACgccggcgaggttatcgttggTGACACATCCTCCTTCGCGGGGAAC

ACTGCTGCGgaGGACGGCGCCGCCATTTTCATTCACCCAACAGGCACCATGACCTTCCTT

GGGTTCGCGACTTTCAAAGAAAACAAGTGCTACGAGAACAAAGGGGGCGCAATCTACAAC

CTCGGCACGTTGAACCTGTTGGGCGGCAGCCTGTTCGAGGGCAACGTCGCTGCCGGCTCC

GGCGAAGGCGGCTCCGGCGGTGGAATCTACAACGGAGGAGAAGGCACCGTCTCATTCGGC

GGCGAGAACACGTTCGACGACAACTGGGGCTTCAAGGGTGGCGCCATCTACGCCGAGCCC

GAGGCATCCATCTCACCCCCTGAGGACGGTGGCAGTCTCGTTTTCGAGAACATCCGGGGC

ACGAACTGCCCCGGCATCTACTACGAACCGGTCGACAGCGCCGACGCAGTCTGCGGTCCG

TAA

>g3518.t1

ATGAAGACAGCCTTCTTTGGGCTCGGACTAGCGGTCATTGCGGCCTCGGTCAATGGCATG

GACTACGAGGTCGCGTCGTGCGCCGAGCTTGCCGACATCGACGACACCACCGTCACCTCc

ctcaccatcaccagcagccCCTTCCTGTGCGACTCGTACACGCGCTTCCGAGTCCGTAAC

GACATGGTACTCAAGTCTGACTCGGCTGTGGAGTTTTCGAACTTCGCGCTGAAGGTGCTG

GGTACCCTCACCGTTGAGCCCGACGTGACATTCCAGGACGTCACGGAACAGATGACTCAC

GGCGGTGCGTTGTACGTGGAGAGAGATTCGTCGGCAACCTTCTTGGGGGCGGCTAGTTTC

ATCGGCAACAGCGTAATCTCTGTCGACTTCCCCCCGATTCAGTCCGCGGGCGGTGGCTTC

AGAAACAACTATCTCCCGCGGAACGGTGGGGCCGTCTTCAACAAGGGTGATCTAGTGTTC

GAAGGCGATGCGCTATTTGTGAGCAACGAGGCGGTCACCACGGACGATAACAACAAGGGG

CGAGGAGGTGCTATCTACAACGCCAGGGTCGGCACGATCACGTTCAACGGACAGCTCACC

GCGATCGACAACCGATCGGATGGATACGACGAGGGGGAGGGCGGCGCTATCTTCAACTCG

GGCGCGATTACCGTTGAGGGCACGTCATTGTTCACCGACAACGACTCCTCGGACGGAGGC

GCCATCTACAATCGCCCACAGGGTACCATGACATTCAACGCTTTTGCGAATTTCACTGAA

AACTCCTGCTTCGATAGAAATGGCGGGGCAATCTTCAGCACCGGCTCCATGAACCTGATG

GGCGGTAGCTTGTTCCTGAGGAACTTCGCTTCCAGCAATGAGGACGGAGGCGACGGCGGT

GGGATCTTCAACGGAATTGGAGGCTCTCTATCGTTCGGTGGCACCAATACCTTCCAAGGC

AACCTGGCCTTCACTGGGGGGGCCATCTACCATGAGGAAGGAAGCTCCGTCTCAGGCGGC

GAGCTCATCTTCGAGAGCAACCGGGCAGATAGGTGCCCCGACATCTACACCCGGGGCGCC

GACCCAGTGTGCAGTCCGTGa

>g3669.t1

ATGGAGTTGAGAATGTCTCCCAAGCAGCCTCTCATTACGAAGGTCGCTTTTCAGGGCGAG

GCGGGCGCTTACTCTGAGAAGTCGCTGCGTGATCTTCTAGGCAACAACGTCGTGGCCGTT

GGCCAGGAATCGTTCGAAGATGCTTTCAAGGCGGTTGCCCGACGGGAGGTGGACTACGCG

GTGATCCCCATCGAGAACTCCCTGGGGGGCTCCATCCACGCCAACTACGACCTGCTGCTA

AGATACGAGGTGAGCGCTTGCCGGGCCGAGGCGGCCGCGGTGGTCGaagcaagcaagcac

ctcTTCAACTCGTCCTTCGGCGGCGCCGTCCACGCGACACTAACTATGGCGCCGCTCTAT

GGCGACCCGCCCGTAGCGATACGGACGGGATGGCTTCTCGGCTGCCCCGTCTCCCGCGGC

GAAAGCTTCGGGGGGCGGGCGCGgatgaACGTGGAGAAGGTGGCCACGTACGACACCGCC

GGCAGCGCCAAGATGATCTCGGAGGGCCAAATGGAAGGGTGCGCGGCCATCGCGTCCGAC

CTCGCCGCCGAGGCATGGGGTATGGACGTGGTCGCGTCCAACATCGAGGACGACAGCGTG

AACTTCACCAGGTTCCTGCTCCTTGGACGGCAGCCGGTGAGCGCTTTCCTGTCGCCGGAT

GTTCCTTCCAAGACCAGCATTGTCTTCACTCTGCCCAACACGGCAGGGGCTCTGTACAAG

GCCCTGGCGTGTTTCAGCTTGAGagagATCGACTTCTCGAAGATCGAGTCGCGACCGACC

TCCGCGCAGCTCCTGCAGTACCTCCGTTTCCAGCAGGCCTCGAACGGCAGCATGCATTCT

CCGACGGAAGAGGGGAACCTGGCGGGCAGCGGCGAAGCGCGCCGCTTCCAGTACTGCTTT

TACCTGGACTTCCTGGCAGGAGAACTGGACGACACCGCTCAGTCGGCCCTGTCTCACCTC

CGAGAGCTGTCGCCGTTCTCCAGGGTGCTGGGGTCGTACGCACGGGGCAGCACGCTCGTG

GGGCCGATCGGCGAGACCCTGGCGGCGCTCTCCAGGGGAAGCCATAGCTTGGTGAGCACG

AGCACGAGTAGGGAAGCgGACGCCGTGAACAAACTCGGCGCGGCcgacgcggccgccgcc

gccggactCACGGTCCCGCTCCGACAGATGGGCAAGCGCGGCGACAGGGCGAGCAAGCGG

CTCAAGATCGGCGTGATCGGCTTCGGCAAGTTCGGGCAGTTCATATCAAGGAAGTTCGTG

GCGGACCACGACGTGGTGGCCATGGGGCGGGGGGACCACACGGTCGCCGCCAACGAGATA

GGGGTAAGGTTCTACCCGCAGTTCGAGTCCTCGGACTTCTTCGCCAACGACCTGGACGTG

GTGGTGTTCGCCGTGAGCATCCTGTCCTTCGAAGAGGTGCTGAAGAGCATCCCTTCCAAG

TTCCTCAGCGGCAAGCTCATCGTGGACGTGCTGTCGGTGAAGATGCACGCCAAGCAGACC

ATGATCGACGTCCTTCCCGCCGACGCGGACGTCTTGTGCACCCACCCGATGTTCGGGCCT

GAGTcggggggggatgggtggggCGGCTTCCCCATCGTCTACGACCGCGTGCGCACCACC

GACCATGCCCGCACGGCGGACTACCTGTCCATCTGGGAAGGGGAGAGGTGCAAAATGGTC

GAGATGTCCTGCGAGCTGCACGACAAGTACGCCGCCAACACGCAGTTCATCACCCACCTC

ATGGGCCGCATCCTGGGGAAGCAGGGGCTCTCGAGGACGCCGATCGACACGCAGGGGTTC

TCTAGCGCCCTGCGTCTCATGGAGACGACCTGCGCGGACAGCTTTGAGCTGTTCTACGGC

CTCTTCCGCTACAACCCCCACAGCCATTCCCAGCTGAAGAAGCTCCGCGAGTCTTTCGCG

GAGGTGGAGCGGCAGctGGCGGCCAAGGAAGCGTACCTCGCGGCCAAGGCAGAGATCGCC

GACGACGACCGCCGCCGAATCCTGGCGGAGTTCCGCACCCTCATCCAGGaggctgccacc

accgccgcgcaGAGCGCCACCGAGAAGGTGGCCGCGGACACTGCCGCCCAtgctgcggcc

gccaccgcGGCTGCCGTTTCCGCCGCCGAGAGCGCCGTCCTTGCGGCCAGCGCACAAGAC

AGGATGGTAGCGAaagcctcggcggcggcggaggcggtaggGGGCAAGGGTGGGGTTTCG

AACGGGCATGgcttgtcggcggggggggcggcaaaAAGCGCGGGCGTTGACAAAAAGGAG

GCAGGGCGAtga

>g3975.t1

ATGAAacaaccggcggcggcggcggcggcggcggcgacagcagcagcagcggcgatgtcA

CCGAAAGAACCAGCACCGCCTCCGAAGCGCCAGCGACCCTACAAGCGGGGGGGCGAGGTC

CTCTTCATCGCGGAGGGCATCGCGCAGGCGGCCGTGGGTCTCGTCCGCGACCCTGTCCGA

GGAGCGCAAGCCGACGGCGCGAAGGGCCTGGTCAGGGGGCTTGGCTCAGGCGTGTTTGGC

GTGGTGGCGAAGCCAGCCAGAGGCGTAGCGAGGGCCGGCGCAAACGCGTACACCGGGGTT

AGGATAGGTGTGACTCGAGCCGGCCGCTTGGTTGGCGcaggtggcggcagcagcggcggc

ggcggaggaggcagcggcagcggtcgcAGCTGGAGCGCCTTTTCCAGTGCCAAGGAGCCG

AGAGCCAACAATAACTCCCGCGGGTCCCCGGAGGGAAATGCCTCGCAAGGTGGACCTCGC

TTGCCGGCACCTAAAGGACCAGGCCGAGGCCaacgggggggtgggcgggcggcagagctg

gctactgctgtggaaGGCCTCGGTGTCAGCGCCGGGGCGGAAGGGGAAggtgtggcggca

gcagcagcagcagcgacgacggcggcggctgcagcggcggcgatggcagcagcgaatgcg

gcggagggggaggggggaggggggggggaggacgaggGAGAAGAAGCAACGCGTGCGACG

CTCGAAGGAGCGGCCGCTGTCGCCTGTGACCGCCGGGAGAAGCAGCCGATCGTCTCGGGA

AGCTCGCTGCTTACCGAAGGTGCCGAAGGCGCCGACGGTGGCGGGGTGAGGGTGGGCgcg

agggcgggcgggcgtgggggcgggggcgcggcgGTCGTCGGAAACGATACGTTCTTCCGT

ACGTTGGTGGACGCGCTGACGGAGGCGTTGTTATCGAAGGCGGCGCGGGTTGGCCGGGGA

GAGGGGGAGCCGGAGACCCGGAGCACTTTTACCACGCGGATGGTGCTGCCATCGGGGCTA

CCGAATTGCTTGCGGGTGGTGCTTGCCGTTCAAAAGGCCGTGATGGAAGGAGGGGCGAGA

GGAGGGCGTTTCGCGTGCTGGCGTGTTCTGTCGTGCGGTCCGTTGGGTCTGGCCGTGGCC

GCGGTGGGCGGCGCACGATACTGCCGACGGCCCTTGCTTCCCTTGTGGACTGTCGCCGTG

TACGACTGCCTCAGGAGaGCCCTAGACCACCGCGAAGGAAGGGAGATGCGTCGCCTGTCC

CTACGGCCCGGCGAGTCGGTCGTGCTTGCTCAGGCGCTGGCGCTATGGGTCGCGGATGCC

TGCGTCTACACCTTCCAGGAGCTGTGGACTCCCGAATCGACGGCCTGGGGGACAATGTTT

CctcgagccccgccgagatcGAGCGGCATGGTGGTGGTTCAGACCGGACTCCTCGGGACC

CTTGCGGTGGGGCTCGCCCTGGCCGGAGCTCGTACCGTGGCACCTGCAGGGTGGGCTTCC

GACTTTCGCGGGCACGCCCTATCctacgccgtcgccgccgccggcacagCCGCGTGGCTC

TCAAGCTGGACCGCCCCGCTCCTGGGGTCAAACCCGGTGTCTTGGGTGGTACTTTTCACC

CTGGGACACTCTGACCTCCTATCGGCCCGGACCCAGGGTCGCGGCGGGCGTGGCAgtaac

ggcagcggcggcgcggcgcAATTACCACCGAATGACAATGCTTCTTCGCTGGCGGGGCGG

GCGTGGTGGGAGCTGGTGACGTTAGCGGGAGAAGTTGGAGGCGTTTTAGAGCGGGGGGGT

GTTGGGAATCGTCCTCTGCTGGTGGCGGGGTGGGTGGCggcgctggcggtggtggtgccc

tTGGCCCCGAGGCTGTCGAGGCGGTTTTCGCTGAGCAAGACGGCCTCGAGGAAGCTTTTT

CATGCCCTGGCGACGGCGATGTTTTTGCCCGCGATCGTTCTCGAGCTGGAGTTTTTGTCT

CTCGCTTTCGGCGCGGCCCTGGGAGTTCTGCTTGCCCTAGAGTTCTTGCGGTGCTTGGGA

TGTCCTCCAGTGGCTGCCGCGCTAGATGCTTACTACGGAAGCTTTTTGGATGCGAGGGAT

GGGGGGTCTTTGGTGGGGCTCGCGGGCAACCGCTTGGGTGTTCTTCCCTACGCAGGCGTT

ATTGTTCTCGGTGTCGGGGACGCGATGGGTGCCGTGGTCGGTTCTAGCGTAGGGCGTCTG

CGCTGGCCAGGCTCGCGCCGAACCCTCGAAGGGTCGGCCTCCGTCTTCCTGAGCTCCCTG

GCAACCCTCGTACTCGCGTCTTGCGTCTTGGTGCAGGGACAGGGTGTCGGTGGCCGTGAC

TgggagggcgtggggggggaggggtgggggcgtgCCGCCGCTAGGCTAGCGTGGCCGGTG

GCGCTTGCGAGCCTGATGGAGGCGTTCACCAGTCAGGTCGACAACCTTATTCTTCCGTGT

GTGCTCTTCAGTTGCTTGGCTCTGCAGCGTGAACAGTAG

>g4196.t1

ATGTGCTCGGAGGAGCTGCGAATTAGGTGCGCAGTGCAAGGATCTCGCAGCGACGCTGGT

GGGAGCACGGAGGCGGACCCTTGGCCTAGCCGGCGCTACCGATGGCCCGTACTAGGGGGG

GAGGCGTGGTCGGCGGTGCGTCTTCTCGATCACGCCGAATTGTCGGCAAGCCAAGGGTTT

CGCTTTTTGGTCTTCATCGTGCCGCCGGAGCAAGACGGTGTAGCGGCGACCATTTGTGGA

CGACGCGAGAGAGAGGCACCAGGGGAAGGGCACCCCTCGCCCCGGGCGCCCCCTCCAAGT

TCAAATTCACCGCGCGGTCAGGATGCGATCGCGGATGTTCTCGGCGCGAGAAGGGGCACC

GACCGAGGTGCCAGCGACAGACAAGGGCTGGCTTCTCTGTCCGAAGGAGGCGGCCAAGCG

CTGCTGCACAGAGGGGGTGCCCCGATTATGGGAGCCGGCAGGTCTTTTGCCACGGGGATG

ACAACCGGAGTAGACGGGGACGACGTCCTGGAGGATATTTTCGGGCTCTTCGACGATACT

ATTGGCGACGAGCCCGTCGTGAAGACGAGCTCGGTTGGAAGGGGCAGGCTCGATACCAGC

GGGCGCTCAACCGGCAGCACAACCCACAGAAAAAATCGTCCGCTTGCGGCTGTGCGGAAA

TCTCCGCTTTCCCTGCCGAAGACCCCTGTGTGGCGCATGCTCTACATTGATCCGGACGTG

TCTGATCCCAAGCTTGTGGCGGGGCTGCTGGAAAAGACAGCTTCGCGAGCGTTGCGGTGT

GGCTGCGTATTCGACGTGGCGACGGACCTCGTGGAAGCCCTAGCGGATGTGCGAGGAGAC

ACAATGTGGGTATATCGGGGGGTGTTTATCAACGAGTCCTTGTACCGCCACAAGAAGCTG

GACCACAGCAGATCGGGGGATAGGTCGATTCAGGGCAAGCATTTGTCGCAGTTCTTGCGG

CAGGTCGGAATGTCGGGGACACGCATCGTTCTCCTTGTGGCTGGCGATGTTCACTTCGAC

ACCGCGGACGCCGGGGCTCATGGCTTAAGCGCCGTGCTGCGAAAACCGTTCACGAAGGAC

GGGTTTTGCGACATTTTGCGCGGCATGTACCTCAGCGGCGATCCACCACCTGCAGCCCCT

GCGCCAACGGGGGAACCCGACGGAGCTCAACGGAATTCTGCTGCATCCCACCCCTGCCCA

ACGAACGAGCCTCAGCGCTCACCTCCGTTTCTCGCCCGGCACCAGAGCTTCGCAAAAGGC

TCGATCCCACCAGAAGACAGGCCAAAGGTGGCAGGTGCTGTTCGCGCGACAGGCCCAGCC

GCAACAACGCCCGCGGCAGGGCCAATGGCGATGCACGGCGGCGGGCATGTCCAGCCACCG

TCCACCTATCCAGCGTGCTTGGGGTTCACCCCGTTCCCGCACTCAATGATCAATAGCTAC

CACTTGGTGTCGGGCTTGCTTCGACCGGGACAGGGAGGCCCACGGAGGAAGTACACCAAG

ATTGCCCCGAAGGAAGAGGACGAGGCCGCTGCCGTGCCCGCCCAAGCGTTGCGTGCGTCG

AGTCCTGATCCTCCTGCAATGGCCACGGTGATAACTACATCCGTCGCCACGAATCAGGCT

GGACACGGGGTATTGCCATGCGCGGGGGGTCGAACTGGCGGAGGTCTCGCTGGTTCTCGA

GATGAGCCTGCGGCACAAGAGTTTCCGGCGACATCTTCACCGGAAGAAGCGGCTTCGGGT

GTAGATAGCACTACTAGTAGGGGTGCCGTGGGCGGTGGGAGAAGTCCTACCGCGGGCGCC

GCTGGTGCAATGGGTAGTAGTAGGCTCGGTTGA

>g4438.t1

ATGGGAGTCACCGTGGAaacgacgagggagggagacaAGCAGACGTACCCTCGACCAGGA

GACAGCGTTTCGATGGACTACACTGGGACGCTGCTCGACGGGACGGTTTTCGACAGCTCC

ATCCGGAAGGGGAGGAGGTTCAATTGCAGGATTGGTGTAGGTCAAGTTATCCAGGGATGG

GATGAGGGCGTGCCGAAGATGTCCCTCGGAGAGAAGGCCGTCCTCCGTTGCACTTCCGAC

TACGCGTACGGCCCTCAGGGTGCCGGTGGGGTTATTCCGCCTAACGCAGATCTTGTTTTC

GAGGTGGAATTGCACTCCATCAACGGTGTCGGGAAGTGA

>g4455.t1

ATGGTTAAAGATGCATCATGGCTGTTTGTGGCCGCCTCGAACAAGCGGGACAACCCGAGT

ACTTCCTTGGTACTCTCGAACGACGTCTACGCGTACCCAGACACGTGGCTATGCCTGTAC

AACGACTTCGGCTGCGACGCACAAGACCTGGAGGAGGAGTGCCTGGATACCTCTGTGATG

ACAGAAGGAGGGCAATCGAGTGCCATTTTCTATCCTGGCGGAGAGTTTGAGCAAAACGTC

TCTGCCGTCGGCAAGCAGACTGAAGACAATGGCTGGTGCATAGAGTTCAAGACGTCCGAC

GTGGACGTTTTCCTTGGGGAAGAGCGGCAAGCTAGCAACTACTTGGACTACTTCCTCTTG

GATGCGTACTGGTATCCGGGTGGCAGCGCAGGCGCGTCCACTACGTGCGTTTCCGACGGG

GAGACATGGAAGCCTCACAAGGAATGGGTGTACATATTTCTGCACGATCCCGAAACCGGT

GTAACGTCGACCGGCATCCAGATGTCCTACAGCTGCATCACAAGTGCATCGGACAGCCAC

GTCTTCACATACATGGGCATTGGACTCACCGAGCAACGTAAACTCGGGAACAAGCATGCT

GGATTGTACAGAGCTCTGTCGGTAACGTCGTCGATAGAGAAGGACGAGGTGAAGGCGAAC

ATAACGCACCCTTACGCCCACTTGGCGTTCCAATTGCAGCAAGAGCCGAACTCGTACCAG

ATAGTCACCGAGATCGACCCCTTCGACATCGCCGAGATGCTTGGCAACATCGGCGGCTTC

TGGGATCTCATCTTGATTTTGTGGCCGATATTCTTCGTGGCTGCCACTCGCCAGGAACCT

CACCTGAAGCCCCGCGACTTCAAGAAGCCGGTGGCTATTTTAGCGGGGAAACCCAGCCTT

GCGGGAGTAACGGCCAGCCTCCCCAGACCACTGAAACGACGCATGTCAACCGGTGGCCAG

ACCGGGAGGACCATCGACATCGCGGAACACCAGATTGAGCGGCCGTACTGGGAACCTCCC

TCGCCGGTGATCGAGGTACCTGCCCCACGCCCGAGGCCGAATGACGTGGTTTCAAAGCAT

CAACGACGGGCGGTCGCTTTGTGGATGCTCTTGATCTTCGGAATCTTAGCATATGCCTCT

TGGCTGTTCGCCGCGGCCATGGAGAAGAGGGAAAACCCCAGCACTTCCTTCGTGCTCTCG

AACACCGTCTACACGTATCCGGACGTGTGGGTGTGCTTGTATAACTCATTCGGCTGCGAC

GAACAAGACGTGGAGAGGCAGTGCGTCGATTCGGCCGTACGCACAGATGGCGGCAGCGCG

ACAGCAACTTTCCATCAGGGCGGGGAGTTTGAGCAAACGATCACCGCAGTTGGCGATGTG

ACCGATACGAAAGGCTGGTGCTTGCAGTACAAGACGTCGGAGGTGAcacggtttttgggt

gaggaacGGGATGCCAGCGAAGACCTGGACTACATTAAGTTAAAAGCATCCTGGTACCCG

GGTGGCGGTCCCGGAAATTCTACCACTTGCGTTCCAGACGGGGACGAATGGACGTCCCAC

AGTGAGTGGGTGTACATATTTCTGGTCGACCCGGTTACAAACGCACCGTCAACCGGGATC

CAGACTTCGTACAGCTGCGTCACCAGCGAATCCAACAGCCACACCTTCACATACATGGGC

ATCGGACTCAACGAGCGGCAAAATATCGACGACGACTACGGTGTGTCGTACAAGGCTCTC

TCGGTGACATCGTCTGTTCATAAGGATGCGGTGAATGCGGACATCAGCGACCCTTACGCC

TTCTTGGACGTGCAAATACAGCAGGAGCCAGATTCGTGGGAGATCGTCACAGAAATTGAC

CCGCTCAATGTCGGAGAGATACTCGGCAGCATCGGAGGCTTTTGGGACCTGATCTTGGTG

CTGTGGCCAATATTCTTCGTGGCCGCTACACGTCAGGACCCTCACTTGAAGCCTCGCGAT

TTCAGGAGGTCTGCTGAAACCGTGGCGGGGGTTACCGGGTTTACGGGGGTAACGGCCACC

CTTGCCGGGCCGCTGAAACGCACCATGGCGCGCGGCGACAAGGGCGGGAGAGAAATTGAT

GTCGATGCCACGGACCGGCAAATCGAACGGCCGTACTGGGTGGACACACCATCAGACACC

CAACCTGTTCAACAGCTGGTGAGTGCGCTGAAACGCTTAGATTTGACGTGCCCGGGAAGC

AAGCGTCGCCGCCTGATAAATGCCCTTGTTTCGGCCTGCTGA

>g4456.t1

ATGTCGACGATTTGGGCACTGCTAAAGGTCAGGCATGCCGACAAGAGCAATGTGGGCGCT

GCTGTTGGGGTTTTCTATGTTCTGGACAGGAGGCCAGATGAGGTGGTTTCAGAACATCAG

AAGCGGGCCGTCGCCCTGTGGATGATCTTGATTTTCGGCATTATATCATATACGGTATGG

ATGTTTGTCGATGCCTCCAAGAAGCAGAACAACCCGAGTACTTCCTTCGTGCTCTCGAAC

GAGGTGTACGCGTTTCCAGACACGTGGGTGTGCCTGTACGACACATTCGGCTGCGACACG

CAAGATCTGGAGGAGGAGTGCCTGGACTCCTCCATGATGACCGAAGGGGGGCAATCAAGC

GCGATTTTCTATCCTGGTGGGGAGTATGAGCAAGCGGTGTCGGCCGCTGGCAAGCTGACC

GAGATGAACGGCTGGTGCATAGAGTTCAAGACGTCAGAAGTAAAGCTTTTTCTGGGGGAA

GAGCGGCAAGCTAGCCACTACTTGGACTACTTCCTCTTGGACATGTACTGGTATCCAGGT

GGCAGCGCGGGCACGTCCACTACCTGCGTTCCCGACGGAGAGCGGTGGAAGTCTCACAAG

GAATGGGTGTATGTATTTCTTCACGATCCTGAAACCGATGCCACGTCACCCGGGGTCCAG

ATTTCCTACAGCTGCATCACAAGCGAATCGGACAGCCACATCTTCACATACATGGGCATC

GGACTCACCGAGCAACATAAACTCGAGAGTAATTACGCCGGATTGTACAAGGCTGTGTCG

GTAACGCCGTCCATATACAAAGATGAGGTAAAGGCAAACATAACGCACCCTTACGCCCAC

TTGGCGATCCAATTACAGCAAGAGCCGAACTCGTACCAGATTGTCACCGAGATCGACCCC

TTCGACATCGCGGAGATGCTTGGCAACATCGGCGGCTTTTGGGATCTGATCCTGATTTTG

TGGCCGATATTCTTTATAGCCGCCACTCGACAGGAACCTTATCTCAAGCCGCGCAGGTTT

AAGAAGTCCGCAGCGAAAGTGCTGGAGAAATCCAATCACCTTGTTCGACAAACGGCCAGC

CTTCCCAGGCCGCTGAAACGAAGCACGCCAGCCGAGGGTCAGAAAGAGCGACGCATCGAC

ACCGCAGAGCGGCAACTTGAGCGGCCGTACTGGGAACCTCCCTCCCCGGTGATCGCGTTA

CCTGCTCGACGCCCAGTGCGTGGTTTACGGGGTTGGTGGCGCCGCCCCTGGATGTCGTCG

TCGCCGACAGATGACGGAATGGCCACAAGAGGCATAGGAAATGTGGTGTAG

>g4504.t1

ATGAGTAAAGGTCAAAATGACGAGCCTTGCGCCTTCGCTAAGCTCTGGGGGCAGCTGGAC

GGTTTACCTTTTCACTGCTACATGACGTCCCTCCCCTCGACTCTTGGGCGAGGATCAATA

GCAACACAGGGATCGCGGAAGCCGGCGGGGTTCATCGACTTGGGTCAATCGAAAGCTCTG

TCCAGAGAGCACGCCACGATCAAATGGGTGCCGGAGCAAAAGACGTACCAGATCACGTGC

CTGAGCAAGAATGGCATGGTCGTGTCGGGGTGCTATCACGCCAAGGGCGGTGTCGAGAAT

CTGGAGTCGCGAACACCAATCAAGCTGGGGCCAGCGTCGATGTACTTTCTCCTGCCGGAG

AAGGTCTCGCAGCGACCATCGGCTCCGGCCCCGGCCCCGGCGACGGTCTTATCGGTGCCT

CACCAACCGACTCCGGTTCCTGGGCAACCCCGGGCGTCGCCGGGGGCGGTGAAAGCGTCG

CCGGCTCCGGTAGTAGCCGCCACAGCGGTTTCGTTCGAAGGCATGATCCGCGGAGCCTTC

AGGAGTCCAGCGTTggcagccgccgccggcgccgcgggGTTATCGAGCGGAGACGTGCAT

AACTGGATTATGCAGAACTACCCGGAGTGGAATCAAGAAGGAGCGAGGATGAGAACGCTG

GCCACCGGCATCGACAGCGCCCTGAAGAAAAGCTTTTCGTCaagtgggggggACCGGTGG

ACAGACCCATCTCTCGTGCCGAGCGCCAAGCGACTCAAGCTCGCGAACGACGCCCCAGCG

GCAGCGCCACCCGCCCAAGGCGCTGCCGCTTCGGCGGCTTCTCAGTGGGCTGCAAATATG

GCAggaagcggcgacggcggtcgCCAGGTGAACGGGGGGGGCGTCGCTTCTGCTGTCGAC

AACAGACCGGGGGTGGGGGACGATGATGGTATCGATGTCGGGATGGATGGTTGA

>g4845.t1

ATGGCCCCGGGCACGATGCAAGCTGCTAAGTTTGCTGCTGCGCTTCTGTGTGCGATCGCC

CCTCGCTCCAACGCGGTAGACGTCTCCAGCTGCGAAGAATTTGCCGCGGTTGACCGCAAG

ACTGAGACGGAGGTGACCATCACGAATGCAGACTTCACGTGCACCGAGTACACGCGCTTG

TCCATCAAGTCTCCCAGCATGGTGCTCAAGTCTTCTGTGGGTGCCGTGACTTTCTCCAAC

CTGGCGTTCAGCGTCTACGGAGCGCTCACGGTCGAACCGGACGTCGAATTCACAGGCATC

GAACTCGTGGAAAAGAACGGCGGTGCCTTGAAGCTGAACCTCCTGTCGAGCGCGGTCTTC

GAAGGCACGGCAAAGTTTACCTCCGTCTCGATCCAGCCAACGTACCTCGGCCTCGACGGG

GAGCTCAAACACAAGGGCGCTGCCATCCACAACAAGGGAGAGCTGGTGTTCATGAAAGAT

GCGACCTTCACTGGCTGCCTAAACGACAGGGACGAGTACGGCAGTGGCCCTGGAGGAGCG

GTGTCGAACCACGTAAAGGGCAGCATCAACTTCATGGGCAACCTCGTCATGGTGGACAAC

GAGGCTAACGATTACTTCGGCGATGCTCTGGGTGGATCAATCTACAACCGGGGAGACATC

GTCGTGGATGGAGACGCTGAGTTTGTCGGCAGCAAAGGCGGGTCCGGAGGTACTATTTTC

CAGGAGAAGCATGCGGCATTCAGCGTCTACGGGTTTGCGACGTTCAACGAAAGCAAGTGC

TTTGATACGACCGGCGGAGCGATCGCCAACATCGGCGGGGACATGTTTTTCTACGGTGGC

AGCCTTTTCTACGACAACTTTGCCGAAAACTCCGGGGACGGTGGGTACGGCGGGGCGATC

TACAACTCCTTCGGCGCCAACATCGTTATGGAAGGCCCTACGACGTTCTTAGCGAACAGC

GCCAACTGGGGCGGCGCAATCATGAACGTAGAGACCCAGAACGGTCGCTACGCAGACGAC

GACGCGGAAGAGGTTGAGGTCCCGACCATTAGCTACCCCGATGACACGGTTTTCATCAAC

AACAGCGCTCAGCTCTGCCCCGACTTCGCTATCGGCTTCGACAACGACATCTGCGGACCC

TAA

>g5219.t1

ATGGTGACCACCACCATCTCCGCTGCTTTCGGGGCCGTTACCACGGTGATCCTGGCCAAG

ATTCAGATGGGCTACTGGGACGGGGGGTCGGCCAACAACGGGCTCCTCGCGGGGCTCGTG

GGAATCACCGCCGGCTGCTCCGTCGTCGAGCCCGAGGGGGCGTTCGTCATCGGCGTCGTG

TCGGGCTTCGTGTACACCTACTCCTCTAAGGCCCTGGTGATGCTCAAGACGATGATACTG

CCTCTGATTGGTGAAATCGACCACGAACGGTCAGTGGCGGCATTCGTTAGCGCGTATCTG

AACACTCAGATTGACGACGTCGTGGACGCCATCCCCGTTCACATGTTCTGCGGCGCGTGG

GGAGTGTTCGCCGCCTCGCTCTTCGCTACCAAGGACAACTACGCGGCCGCGTACTACTCA

GACCGCGCCGAAAAGTGCGCCGGGGCGTTTTACGGGGGCGACGGAAGCGCGGTGGCAGCG

AACGTTGTGTTCATCCTGGCTATTGTCGGCTGGGTGGCCAGCACTTGCGGTGTTCTGTTC

GTCGCTCtcaagctcaccgtcgggatgCGGGTCACCAAGGAGATGGAGCAAATCGGCATG

GACGACTCGAAGCACGGGGGCCAGACCTATCCCGAGATGATCAAGGCCATGGCGTGA

>g5248.t1

ATGATGGCCTTCCCCAAGGTTCGGAGTATCGTAGCGATCGCCGCCGTCCTGGTTCAGCTG

CTGGACCAAACGGCAAAGGTGGGCGCTTGCTCGTGCGTATCTCAAGAGAGGGACCTGTGC

GACTACATCGATACCGCAGACGTCGTGCTGCGTGCGAAGGCGCTTTCAAGGTCGGAGCAG

AACGACATCAACGACCCGATCACCTACACTGTCCTCACCACCACACTCTACAAGGCCGAA

CCCAACGTGCGCTACGCCCAGGAGATATCCTTCGTCACGGGGGGCAACAGCGCTCTCTGC

GGGGTCTTTCTGAACATCGATGAGGAGTACCTCATTGGTCTATACTGGAGCGAATCTGGC

TTGGGCACCGACCAGGGGCCCCAGCTGTCGGTTGGCTCGTGCGACCTGGTGCGGGTCTGG

AGCGATGTGACGACGGAAGACATGGACACTCTAGACGCTGGGTGCGGCAACGTCGACCCG

TGCGGTGGCTGTGGCGATTCTCAGGAGTGCACTCTATACCCTCCCACGGGTGAGTACTAC

TGCTCCGGCGTCTGCACCCCGAGCTCATGTGGCGACGAAGAAACGTGTAGCTTGGAGGAT

TTGGTGTGCGTTCGTGCCCCCTGCCCGCGCATGGTTACCTGCACCGACCCTTGCGGTGGC

TGCACTGAATCCCAGGAATGTCTCATGTACCAGCGGAACAGCAAATTTTACTGCTCGGAC

AACTGCGCCAACAGCCCTTGCGCGGACTACGAGACATGCACCACTAAACCCAGAAAATGG

TGCCGCCACTCGGGCTTTGACGGCTGCCCCTCCAGGCGCCGCTGCAAGGCTAACTACTGA

>g5250.t1

ATGGCTTTCCACGAGCTTCTGGGCATCCTGGCCTTTGGCGCCGTCCTGCTGTTAGACCAG

GCGACAAAGGTGGAATCTTGCTCGTGTTCGCCTAACTCGGGGGGGCTGTGCGGCTATGTG

GACACATCACATGTTGTGCTGCATGCAACCGTGCTGTCCAGGTCCGAGCAGGTGGACAGC

AGCGACGACGAGACGTACACGGGAGAAAACAGGGTGGTGGCCATCAACAACGACGATGTG

ACGTACACGGTAGCGGTAAACACCCTCTACAAGGAGGAACCCAACGTGGCGTACGGCCAG

AAGCTGAACATCGTCACCGCGGCGAACGGCGCTTTGTGCGGTATTTCGCTGAAGCTACAC

GAGGAGTACCTCATCGGTCTTTACCGGACTGGAGGCAGCGTTTTCAACCCCGAGCAAGAG

AGTCAGCTAAATGCCAATCTCTGCAGGCTGGTGCGGGTCTGGTCCTCAGTCACCGATGAG

GAGATGGCGCTTCTAGAAGCCGGCTGCGGCGATGACCCATGCGATGGAAGATGCAGCGAG

TTTCAGGTATATAATCTTGAGAAGCGACTGATGAGGGTATAG

>g5253.t1

ATGAGCATCCTGGCATTGGGCGTCGTTCTGCTGCTAGATCAAGCGACGAATGTGGAAGCT

TGCTCGTGTTTCATTAACTCGGGGGGGCTGTGCGACGATGTGGACGCTTCAGGTGTTGTG

CTGCATGCAACCGCGCTTTCCAGGTCGCAGCAAGTGGACATCAACGACGATGTGACGTAC

ACAGTGGCAGTAAACACCCTCTACAAGGTGGAACCCGACGTGACGTACGGCCAGCAGCTG

GACATCGTCACTGGTGGGAACAGCGCCAGCTGCGGGGTGACACTGGAGCTTGGCGAAGAA

TACCTTCTCGGTCTACACCGGAGTGGAGCCAACATTTTCAAACCGGAGCAGGAGGGTCAG

CTAAGGGTTGGTCTCTGCGACCTGGTGCGGCTCTGGAGCTCAGTCACCGATGAAGAGGTG

GCGCTTCTAGAAGCCGGCTGCGGCGACGACCCATGCGATGGAAGATGCAGCGAGTTTCAG

GAGTGTCTGTTCTACTCTGACAGTTCCACGGCGGAGTACTACTGCGCCGACGTGTGCGAC

CCGAGCCCATGCGATGAAGGAGAGATATGTATCTTGGAGGATGTGGACTGTTTTCGGGCC

CCCTGCCCGCCCCTTGCTGAGTGTACCGGTTCCGGCGATTGA

>g5281.t1

ATGCCAGCACAGGTTGCACAAGCAGACCACCTCCTAGACGGCCAGGGTTCgtcgccacta

cagcagcagcaggaggagcagcaacaggagcagcaggagcagctccagaagcaccagcag

cagcaggagcagcaccaggagcagcaccggcaggaggaggaggaggaggaggaggaggag

gacgaggaacaGCAGGAGGTGGTgcttgctgcagcagcatcagaTCAGGAGGATGGtgat

cagcagcatcagcaagaaGGCAACGACACGCAGCAGGATGGCGAAAGCATGGCATCAACA

GCAGCGCCGGTAGAGGAGACGGATACTCACGGCAACGACAGCAAGCAAGGCTTTGGCCAA

GTTCCGGAATCGGCCGTGAAGTTCCTCGTGCCCAGCTACCTCGCAGGGATCTTGATTGGC

AAGAAAGGGGCCACGATCAAAGATatCCAGCAAAAGTCTTCGTGCGTCGTTCGAATCGCG

ACAAGCGGGATGTACTTTCCCGGGACGATGGAGAGAGCGGTGCTTGTAGCCGGGGGCAGC

GAGGGGGTGAAGAAAGCGTGCAGCCTCATCCTCCACACGATCCACgtccccaacaacaac

atatcgGGTTGCGCTCAGCTCGTCAGCCAGGAGAAGGCGGACACCATCGAACTCACGCAG

CGCATGCTCATTCCACTGACGGCCGGTGGGTTGGTGATCGGGCGCAAGGGAGCCAACATC

TCGGCTCTCCTTGAGGCCTCGGGAGCGAAGGTGACTCTCGGGCAGAAGGCCGAGATCAGG

GTGCACGAGAGGATGGTCACGGTGCAGGGAAAACTGACGGCAGCGAAcaagGCCGTCGAG

ATGCTCGTGGACAGGCTGCTGGAGGAGCCAGTCCTCTCCCGCTTCTCGAACCTGAGCGTC

AACTACCGAGGTCACGGCAGCAACTACGCCAAggcggctgctgccgccggcTCCGACACC

ACCCCGACCGACGCGCTCGTAAAACCCACGGACGTGCTCTCCGCAAGCGGTCCTGGTGGA

GGGGGTATCCCGCCGCCAGTCGGCGCGTTTCAGGGTCCCGTTGCGGGTGCCGCCGGCGGC

ACCGTCGTTGTTGTCGGAGACGGTAGCGCTACGTCGAATTCCCCGACACAGTCGGCAGGG

GCTGACAGGGGCAGCCTTCGGATTGTACCGCCTTCGACGTTGCCTGCGCCTGTGATGCCC

GTCAACAGAGGATCGCCGATATCCCCGGCGACGATGCCCATGCCATCCATGGGCCCCGCT

GGACCGATGATGCCCAACCCAGACCACCTGCAAGCCATGACCCCCATGGTGCCGCTCACG

CACTCCCCTCCCATGCATGGGATGGGGATGGCGTACGGCACGCCCCCTCCCACCATCTTC

CAAGCCCCGCCCGGCGCCGTGTTCCACCCGGGGATGCAGTCTCCCATCATGGCCGGCGCT

ATCCCCGCGGTACCGGGAGGTTGCGACGGGTCGCCCGGAGGCTTCATGGCcatgcccccc

ccgtcccagcAGGCGATGTTCCAGTTCGAGCCGATGAGCaacccggtggcggcggcgata

gcgGCTTGCCCGCCGCTGACGACGGTTACCGTGGGCATTCCGGACAACATGATCGGGGCG

ATCCTGGGCCCCGGAGGGGCGACGATCAGCGAGCTGCAGTCGACCACCGGGGCGAGGATT

ATCGTGAGCCAGCGGGACGAATTCTTGCCCGGCACGGAGAACCGTATCCTCACGATCTCG

GGTGCGCCGATGGCGACTCAGCTGATGATATTCACCTCGCGGTTATATGGATCGAGTGAG

CGTGGTGACCCCGACGAAGACATCGACGACGCGGCCAAGGAGATCGTGTACCGCAACTAC

GTGGACATCTCCGTGGCGGTTTCATCGCCGAACGGACTGGTCGTTCCCGTCCTTCGCAAC

GTCGAGGACATGGGGTTTCGCGGGGCCCCTCGACCATCCGCCCGGCGTGCTACCACCCCG

CCGCGAGCCACATGGTCACGACCACCTCCGGTCGTCCTTGTGgcctccgctgctgccgcc

tcgcctttcccgacggtacccatccCGACCGAGCGCGACCGCGACGAGCCTTTGGGAAAC

CTTCTTTTTAAGACTACCGCCCCCGACTGA

>g5320.t1

ATGCGCTACTTTGCTCTTCTGGCCACCATTTACGCCGCCACCGTTGGCATTTCGTCCGCC

CTTGTTGACGCGCGCCAAAGGACCTACGTGGACGTGGAATCCGCTGGTCATGACCGGAGG

ACGTCCGGCAACGGCTGCAGCCCATACGGCTGTGTACCCGAAAACACTCGAGACAAGAGT

CGCGCGAACAACTCTCGCTGGTCTTGCAAGGGAGACCTAGTCGAGTACCCGGGCGACGGC

TGCTGGATCCACTACTACTTTGACGAGCCCCAAGACATTGTCGAGATGCGCATCGCGTTT

TACAAGGGGACCAAGGAAACCCGGACGCTCAACGTGTACGACAACGGTAACTTTCACAGC

CAGATCGAGTCCAGCGGCGAGACGGAAGGATACCAGACCTTCTACCTTGACACCAACGAA

ACGGCCGATCTGAACCTCTACCTCGACGACTACGAGTCCAACCCGGACATGTGGCTTAGC

ATCAAGGAGGTGCGTCCAAAACCGTGA

>g5402.t1

ATGTCGCTGTCCTTCCTCTTCTGTAAGCCATGGCATCCGGCCACCACCTGGAACCAGAAG

AAAATCTGGGAGGCCGAGCAGAAGGACGCCAAGGATAAGAAGGACGAAGCGGCCGCCAAC

AAGGAGCTCAAGAGAGACAGCGACAGACGCTTCTATGAGCACCTGGCCGGGGGCAAGGAG

CAGGACCCGTCCTCGACTGCTCTCAACTTTATGTACGCTCCGCCTCCCGGGTTCCAGCAG

CCCCAGGAAGAGGAGCTCAACGATGATGACGAAGCTGTCAAGAAATTCAAAGCTCTCGCG

AAGATGAAATCAGACCCGAATTTCTCGGCTGCAACGAGCTGCTCGGAGCTAGAGAAGCTG

GTGGGGAGGCGAAAGAACCCGGGCGTGTCCCTCGCGGAACAGCAGGAACGGTTCCCGTTT

CTCAAGGACGCGCCGGTGGAACACGCCTACGCGGCAGGGGTGAAGGTAAACTTCAAGCCG

TTCAACAATGTGGTTCGGCACGTCAGGTGCATGCGGTGCGAGGAGTGGGGACACCGCAGC

GGGGACCGAGAGTGCAAGCGCATAAACGATAACCCGAACGATTACGAGCGCCGCCTCCGC

GAGGACCCGATGTCGCACCTGGTGTCCGCTTCCGATGGAGGCCTCGGGGACGCaggggcc

gcgggggggggcgacgctcTCGGAGCGAGTTCTGCGGATGGTAACCCGAACGAGGACGCG

GACTCGGCGTTCGTGAACAGTCTCTCGTCGAAAAAGAAGCGTCGCCTTTTGAGGATGTTG

CAAAAATTGGaggcgggagagagggggggggcggggaaggaggaggaagaggaggaggag

agggggaagaggagaagaAAGAAAGGACGTAAGAGCAAGAAAAAGAAGAGTACTAAAGAA

GATGGGGGCAGTAAGActaggcggggcggggggggtggtgacgacGAAAGGCGGAAAAAG

GAACAGGAACCGGAGGAGAAAATCGAGGAAGTCCCCAGCGGTGGTCGAAATAGTGGTAAG

GCAAGAAAGAGGAAACGGGAAGAACCGCCGCACCCGTCTAAGGTCGTATCCGAAACCGTC

GCGAAACTGTCGCCGCCGGAAGACGGGGGAGCCGACGACGAGGTTAAAGAAAGACACgct

gaaaagaaaaagaagacgcGGCGAGGGGTTGACCACAGGAGCTCCCGTAGGAAGGGCAGT

AGTCATCGCCCCGAGAAGGATAAGAAGAGTAGTCGGAAAgggacggggaaggggggcgaa

AAGAGAGGGGCAGAGGGCGTTGAGAGGAAGAGCGTGGAGCGCGAGAGAGGGAGAAGGGCA

GGGGGTGGTGATGTCAGTGGAAGTTCTAGCAGTTGA

>g5690.t1

ATGTTTTCTGACAATAATCCGCCACGATCGTCCTCGACCTTGCGTTTCGATTACTTGTTG

TGCAGGGCTACACCCGGGGCAGCGACAATTTCGGGCGGCTGGTTCTCTGGGTGGAATTCG

GGTGAAAACTACAAGATGGTCGCTAAGGTGAAGCACGCCTCGTTTAAGCCCATCACTGCC

AACGTCGATGTTGACCTGGATAGCTTCAAGCAGCCCTGGCTCCGATATGTGGCCCACTAC

TTCCTGCAAATTCCTATGTATTTTATCAAGTTCCCCTTTGCGGTCTTGCTGGGGCTCTGG

AGTGCCCTCTTCTGGCGTACGCCTACGGCTGAAGATGTGGTGACGTTCATCGAGGGCACC

AGTCTAGTCACCGTCCTTCGTCGTGACCGCGCATCCGAGCGCGCTGCACTAGCCTCTTCA

GGAGAAACCGACCGCGCGCAACAGCGCGCGTGCGAGGAGCGCCACTACTACATTCGCGTC

GACAAGTGCATGCTGGAACACGTCAGTCGTGAACGCGGCAGCAAGTACAACGAAGGCACA

GTCACCCCCATCAAGTCGTGGAGCATGAAGTACACCAAGACCAGGTGTGGCGGAGAAGAC

GGCAAGTGGGCCGTGAAGATCACGTCGTTCCTTGTCAACGAAGAGCCGGTGACCGACCCG

GAGAGCCAGGTCTCCTTCCTCCACTTCTACTGGACAACGGCTGCTCACACCAAATGCCAC

GCGCTCGGAAACGCGCTCGTCGAGCGTATCTTGTCGGACGAAGACCTCAAGACCAAGCTC

ATGGAGTCGACGTGGACGACAACTTGGCTGCACCACGGGCTTCTCCACGGTACCCTGGGC

CCCCTGACCACCACCAGGACCGAGGGAAACAAGTGGATGGGGCTGGGATCACCGACGTCT

CGGAACTCTCTTGTGGCGGAATCGCGCAACATGTCGGCTCTTGGCGGCGCGGACCACGGT

CGGGCCGACCGATGGAGTGAGCTGGAGCTCCCTTTGGCTGACTTCATGATGGGGGCACGA

CACGTTCTCCGCAGGGCGATGCGAGACAACGGCATCCCACTCGACATGCAAGAGGGCATC

TTCCTGTCCTGCATTATCCACGCGATAGATCACGTCCTCGGCTCGCGGCTTCTGTGGGGC

ATCAAGTACAAGATCGACTTCAACAGCGAGATGACCACTTGGACTGACCACTGCGAAAGC

GCCAGCTGGAGTTTCATGTGGATGGCTCCGACTCTGCACCCGTTCTACCACAACAAAATA

TGGTGCACTTCCAGGCCGTTCTACAAGCAGCTCTACCGAGGGCTGAAGGAGATTGACACC

TTGAAAATCGCCGACGAAATCACCGCAAGCATCATGTATTGA

>g5714.t1

ATGGCCCTACGACAAGTCGGCGCTGCGCAGATGCGAACGGCATTACGATTGGCGCGGACA

CACCGACAACAGACACAGCCGGGTGGGTGTGCGGCGGTGCACAACGCCTTTGCGCCTACT

TTTtgtgccaacagcagcagcagcagcagtaaaagaaTTAGCGGCAGCGCTGGGCGGAGA

TCGGCGAGCACGAGCAGCGGGTCTGAGGTGACGTCGTCAGGAAAGAGGCCCCTTAGCAGT

AATGCCAGTGCCAACCCGGAAGTGGTTTCGGCGAGCGTTACGGACATGACGAGCAAGTTC

GGTGACCCGTCTTTCTGGGAGGGGGAGTACTCCACGCAGTACGACAGCCAGGAAGAGCTG

CAGCCGTTCGAGTGGTTTCTGCCCTACGACGGGGGTCTTCGACAACACCTTCTTCCTTTC

TTGGAGTCGATGACCTGCAGTCGCCTGCTCCATGTGGGATGCGGCACGAGCGAGGTCGGA

CCCAAGCTCGCCACGGAGCCGGACCTGGAGCTCCACGTGACCGACGCGGACAGCAGCCCT

AGCGCCGTTCGGATCATGAAGCGGCGCCACGCGAGTCTCAATAACTACACCTGCCACCAA

ACGGACGTGCTCAACCTTCCCTTCCCGGAGGAAAGCTTCGACGCCGTCGTGGACAAGGGC

ACGCTGGACGCGCTGTTGTGTCGCAACGTGGAGGACGCGCAGCAGATGGCAACGGAGATG

CACCGGGTTCTCACCAAGGGTGGCGTTTTTGTGCAGATAACGACGGAAGACCCCGAGGCT

CGATTGGAGCTGTTGACAAAACCTTTGATCGAGCAGCACGAGCCGTGGTCGCGCTCTTTG

TTCAAGGAGATCGGGGAGGGGGCCAACGCGACCTACTTCATGTACGTCCTCGTCAAGTAG

>g5806.t1

ATGGCTCCTGTCAACGAGGCCCCACCCCCTTATGAAAAATATGGATCGGTCGACGACTCC

CAGATTCCGACGTCTTACAGCAGGTTGCTCCCAAGGTCGTCAAGGTGCAGTAGCACCAAC

TACCCAGCCGTGGGGGAACAGCATCACAACCTGCGAGACCCTGATGCTGAGAGACGTGTT

CCGTGGAgggttgctgttgccgttgtgtGCGGCTTTGCGTTCGTGGCGGTATGCAGCGTA

CTGTGGGGTCGGAGCAAAGCCAGTCAGAGCACCGCAGCAACTCTGGGGTTTTTCGAGGTC

GGAGCTGACGCCGGTGGCATTGCTGCTGCCGACAtgcgacctattcccgacctctcatct

gctgctgctgctatcgcagCCTTGGAGTTCACGGCACTCAATTTCTACCACATTCGGGAC

GGGAAGCCTGGCCAGGACTACCCTTGGCTGAAGAACATCAAGCTGATCGAGCCACACCGC

GACACGACTCTCGCAGTCGTGGGGGCGCGCAAAGGGTTTGATTATCGCTGGGAAGTGCGC

GCAGGCAGCAGCTCAGGCGGAGTAGGGGAAGTGCACGCTATGGCAACAGGGGCGGTGACC

ATTGTGGTCCTCACCCAGCTCGACGAGAATGTCGTCGTGCTGGAGGAGGTCGATGGGGAC

GGCAACGTGACGAACCGGCTGGAAGAGACAGTGATGGTGAAGTACGTGCGCAGAGAGATA

AGGACTTTGACGGAGGATGAGCGAGAGGAGCTGCTGGATGCGATGTTTCAACTGTGGGCG

GTACGGGTGGACGGTGGCGATGGGAAAGGGATTTACGGGGACGACTATGCTGACGTGTAC

GCCATGAACCGGGTTCACTTCATGGCCACAACGAACGACACCTGCGATCATTTCCACGAC

GGACTCGGCTTCCTGGTGACCCACTCGCTCATCACCAACACCTTCGAGTTCAGCCTGCAG

CTTGTGAACCCCAAGCTGACCGTCCCCTACTGGGATTTCACGATCGAGACGACTGATGCC

GCTGGTGTTGCGTACGACTCGACACTGCCGTACACCAGAACGGAGCTGTTGAGCCCCTCG

TGGTTCGGAACCGCTGATGGCGGCGACAAGATGGTCAAGGACGGTCGGTGGGCGTACACG

GAAATCGCCAAGGACAAGAAGGACAACCCAGGCAAGATCGAGACGGACATCTACGGCAGG

CTACGCTCCCCGTGGACAACCAACGACAGATCGTACTTGTCCCGGGGGCTGGGCGAAGTG

TGCCAAACCAGCGCCGACGAATACTTAACCTGGCCCGACTGCAAGATCCACTACGAGCTC

ACCACAACCAACAGCGATTTCTACTCCTGGGTGTGGGATAGCATGGGCAGTCCGCACGGG

TCAATCCATCTCTGGCTGGGAGGTGCCTTGGACTGCGACCCCATGTACAACAAGATCGGC

AGCCTCGTGGGCTCCGAGATCGCCGAGGCTCTAGCCTACTTGTCGGTCGGCCACCGCAGG

TTATTGTTCTGCGGAGGGACTTGGAGCTGCGTCGGAGAAAAGGCTAGTGTTGACGTTAAG

CCTGAAGAGCTTCTCTCGAGCGGGACCTGCGGTTGCCACGGCTTCGACCTCACTAAAGGA

GACGACTACAAGCTTGTTCTCCCCCAGATCGCTTACCTCGAGTACTTCATCGGCGAATTC

AGTGAGGACACCCAGCGACAGGTCGTCGAGACGCTTTGCTCTGGGGTGGTGGGCCTTGGT

GACATGGATCAATCGAGCTCTTCACTGGATCCGATGTTCTGGCCGGTACATCCAACGATG

GAGCGGCTGTGGCAGTTCGCGGTGGTGACGGGCCAGGTGACGAGGTTCACCTGGCCTGAC

AACGACGTTGAAATCATCTTCCCCGACGGCACGAGCAAGACCGAGTACACGAGCAGCTAc

tacgaggcgtgctacggccACCACGGCAGTGACATATTCCCGTATGGCCTGCTAGACTTG

GACACCAACGGCTTCGAGGTCAAGACGGGAATCCGCGCTAACCCCGTCATCGGGAACAAC

CTCTCCAACCGCGAGGTGCTCGGGGCGTTAGACCCTCGGTCCAACTCCCTGGCTTACGTG

TACGACACGTTTAAGTGGGACCACTGCTTGCCCGAGGGATACGACTTCGATGATGCCTGG

GGCGAAAGCGCTCCCTCGGCTCGGAAGACCTTTTTTGAGAGGGACGGGCCTCTGTTGGCC

GTGTACACGAGCTTCAAGAGGATGATGGCCGAGGTCATGAGTGAGGAAGGAGATCGTTTG

AAGGAGGCGGGTGAAGGAAAATAA

>g5973.t1

ATGCAGCGGCGCGTGGCTCGACGAGATGATGGGAAGAAGAGTGACGGCGGCGATGCAGTT

GGTTCGTCCGCTAACCCGCTTCGCCGACTGAAAAAACTCGATATCTACTCGAGACCAAAG

AAGGAGTTCCAGCGCGGCACTGTGCACGGAGCGGTGGTTACCCTTGtgctggcagcggcg

gtggtggcgctcACCTGGCGAGAGCTAGCGTTCTCCATGAAAACGGAGACCGTGGAGCAT

TTGTTCGTGAACTCCACCATCAGCCCCACGGTGAACGTCACGTTCGATCTCGTATTTGCG

CGCATTCCGTGCGCCATGATCTCGCTCGACGCGGAGGACGCGGCGGGCATTCCTCAGCCG

GACTTGAGGCACGACGTCACCAGGACCAGGCTGGACTTGGAAGGCCGTCCCCTCGACAAG

GGGGAGAAACACGCGATGGGCAACACGCTTAAGGAGGTGATAGAGAAAGTGCAGGGCGAG

CACGATGACGCCGACTTCGCTGCGGAAGAAAAAGCGAGGAGCGGGagcaaggagggggga

gggggtggggatgaCTGCAACTGTTTCGGAGCCGGATTTGAGGGGGAGTGCTGCCGAACG

TGCGAAGATGTCAGGGCTGCCTACCGACGGAAAGGCTGGAAGTTCGAAGCATCCGCGGTG

CCGTCGTGTAGCGGCGAGGCAAGCCTAGCAACCTCAACAGCAGGCGTTACccagaagggg

aaggggaaggtgcAGGCGGGGGCCGTAGAAAGCGAGGGGTGTCAGCTGGCGGGATCACTG

GAGGTTTCGAGGACGGAGGGCAATTTTCATTTCGCACCCGGGTACCGACTCCACCGCCAC

GCCAGCGACATGTCCTTCATGGACAGAATACAGGTGGCGCTGGAGTCTTTCAACACGACC

CACACGATCAATACCCTGACCTTCGGGgaccagcccccccccggacACGCCTCCGCTAAG

CACGAAGCCGCTTCCACTCTGCTGGAGGGGCACCACAAAACTGTGCAGGACACACACGCc

atgcaccagTACTTCCTGCAGCTGGTTCCGACGGTGTACCGGTTCACCGACGGGTCGACG

TTTCATAGCAACCAGTACTCGGCGACCGAGCACCTCAAGCACGTGCATTCAGGAACATCT

TCAGGCCTTCCCGGGGTGTACTTCCACTACGAGGTATCGCCAGTCCAAGCACTCGTAGAG

GAAACGAAAAAGGGGTTTCTATCGTTTTTGACGGGGGCGTGCGGGGTGGTGGGCGGGGTT

TACACCATCCTAGGCCTCGTCAACGCGGGGATCGACGGCTTGCGCTCTGACAAGGGTTTG

CACTGGTCGAGATAG

>g5998.t1

ATGGCGGCTGCCGCCCCCAACAGCGACGGAGATATTATTCTCGAGCCCCAGGGCGACCAC

CACAGCTGGTACTCGCTGAAGCCTCTGACCGAGGCCGGGCAATGGTCGGCCACGCCCGGC

GCGTACTTCAAGAAGCTCAAGGAGGCCAACGGCGGCGCTCCCGTCTACAAGGCGCACCCC

GGGCTGGCGTGCATCGCCATCACCGATCATGCGGGCGGGAAATGGTTTTTTGAACAACCT

GACACTGTGTTGGACAGACAGGATGGCCCGTACTTCGGGCCGCTCAAGTGCACGAAGGAG

TACGTGGGGGACTCGCTCCCGGCCCTAGTGACGAACGTTCCGGAGTCCCACGCGGCGGTA

CGGGCGTACACCAACTCCTTCTTGAGGGAGAGGCTTGGCGCCACGCAAACGGCGCTCACG

CACGCCTCCGACAACTTCTACAAAAACCTCAAGACGAACGGCCTGGGCGACTACCCCACG

GTGTATGCCTTCTTCCTCCAGCAGGCGTACGCCTTCATGCTCGAGTGGGTGTTCGggacg

ggggaggagggcggccagcccctccctcccttcaaaGACTTCATCAACGTCAACCCTAGC

GACGTCAGCGTGCTGCTGAAGCTGGAGATCGACACTCCCATAGCCAACGCGGCGGCCGTC

GTGGCTCAGACGCTGGCGGGCGGGGTGTCGTCGTCGGAGAAGGCTTCCATCGAGGTGCTC

CTGGACTCCATCCGCTCCTCTAAGATGTGGCCCAGGTTCATGGAGATGCTGGAGGAGAGC

AATGTGTCCATCAAGACCCCCGAGCGGTCGTTCATGTTCAACACCGGCTTCCAGTCGTCC

TCCGCGCTCGCGAAGAACATGGAGTACGCCGTCGGTGTCCTCACCGCCCAGCCCGACTTC

GCGGAGGAGCTTCGGAAGGAGCTCGACGGGCAGGCAAGCAAGTCAGCAAGCATTTTGTAA

>g6008.t1

ATGATATCCAGAAATTTTCTTGCGTTCCTCCCACCTCTCCACAGCTATTTTTCCTCCCCT

CCCGATTGTCCCGCATCTCGCGTCAAACACTTGGTTGCTGACTCAGGTAACAACGTGGCC

CATCCCACGTGGGGCAGTGTTGGACAGAGACAGCTCCGCAGCGTGACGGGGGCAAGCTAC

ATCGATCGGGTATCGGCACCTCCTGGGGAAGATCGCCCGACGCCAAGGGAGGTCTTGTCG

GAGGTTTTCCTTTCGACGCCACCGTCGAGCTCGACAACCAGCGCCCTATTCGTGGGGTGG

GGATTGCTCGTTGGGTACGATCTTTTCTTGAATCAGGACAACGTGTCAGAGCCGCTGGAC

ATCGCGTGCGACGATGGGTCGCTCGCTGACGGAGTGTGCCCCTGGGGATCATTGTCGGGG

CCGCTATCTTTCAACAGGTCACAGGCCGAGCTTGACGCCGAGGGCGACGGAACCAGAAGT

CCGATCAACTACGCGACAGCTTACATAGACCTGGACTGGCTGTACGGCAGGGACGATAAA

TCTGCGGCAGCTCTCCGAACACTGCATGCGGGCAACCTGAACCTTACCGATGATGAACTG

CCGCATCTACTGCCCGATGGAACCTGGCTCGTTGCAGACCAACGGCCAACAAGGATGCCT

GTAACATTCGCCCTCTTGACGCTTCTTCTGAGGGAGCACAATCGATGCTGCAACGAAATG

GCCCCAGCGTGGAACGCCAACACGGATGAGGATGCGTACCAGGTGTGCCGGCAGTGGACG

ATCGCTGTATTCCAGCACATATCGGAGAACGAGTTTTCCGAGCGCCTAGTAGGTGGGAGC

GCAAGGTACCTTGGCTCAGCCGCTCACAACTTTTTCTTCGACGACGCCAACGAAAACAGC

ACAGTGGATGCTCGTCGGCAGGCGCATCGGTTGAGCGCCATGGAAATCATTGTGCGGTCA

CGCTATCTGGCTCGAATGAGCGGATCCTACGATGCGAGAACCAACGCCGGCGCGGATGTC

GTGTTTTCTACTGTCGCCAACCAGGCGTTTTACTCGGCCCTACCGTCGATCGTGGACTTA

CGGGACGACAGTTACGGCATCATCGACGAGGACGGGATCAAGCTGACGGCAGCGAGCGCG

GATGTGCCTGGTTTGATCAAGAGAATCGGCGGGATCGAGTCCATCGTCAGGGGGGCCGCG

TACTCACGCGCACAAACTGTGGGTGTTTCTCATGTGCACAAGGCTTTCCCGTCATCGCTC

CTGTTCAACTTGCCGATCATAGCTATCCAGAGGGGGCGGGATCACGGTATCCCGTCCTAC

AATGATGTGCGCCAGGCATGCGGACTTTCGCGGGTAGCGACCTTCCAGGACATCACATCG

GACGATGCATTACAAGGGCGCCTGGTGGATGCCTACGGAGACGTGGAACTGCTGGATGCC

TACACGGGAGCACTGGCGGAAACTGAGGACGGGAGCGGGCTGTTTGCCGGACCCTTGCTG

CGGGCCGTGTTTTTGGAGCAAATGTACAGGGCTATCGTGGGAGACCGTCACTACCACTCG

CACAACTCGCATGACGACGATGCCAAGCTCTCCACGTTCAGCGACCTCGTTCTGGACAAC

TCGTTGATCTCGTCCATACCAGTGGATACCTTCACGGCTACCGATACGGTGGCATCGCCG

TCCTCCTCTTCGACCAAGTTCAAAGAGGTCTATCTATCGGACGGGTTTAAGATCGCATGG

AACGGGTCGGAAGAAACCAATAATACACTGATGCACATGACGATTTCGGCAAGGGGCATA

GGTCTGCAGGGAATGATCGCCGTCGGGTTTGGAGGGGACGACATGGCACACACCAACGAC

TTCGTCATATGCACCGTTTCCTCGGAGAGCTCGGCAAAGTGCACAGACCACTCGGGGATC

AAAGGGCATGGAAGGCCTCCTGGTGATGTCAAAGGTGTTCCCGAGCTCGACGTCGTGGAC

GTTACCTGCGACGGAGAATGGACGAGCGTGACGTTCGCCCGCTTTGGCTTGGGAAACGAC

GATTCCGACTACCCCCTCGCTGACGACATTGCATCTGACCGGGACACACCTGTCGTCTAC

GCGTGGCGTTTGGGAGAGGGCATTGGCATACACTCCAAAAATCAGCGCGGGAGTGTCCTC

CTGAACTTCGCAGACGGCACGTTGACTCCTATCTGCAGCGGCTCCAGCGATTACTACGCG

CTCCATggcgctctgctgctgtttgcttggTTAGTTGTCGCGCCATACGGCATCTATCAG

GCCAGGTACCGCAAAGGTAGAACGACACTGATCGGCAACCTTTGGTGGGAAGCGCACGAA

GATTGTATGATTGTCTGTGCTGAGGCGTTGCTTCCTCTTGCCATCACGGCCATCTTTGCC

ACGGGTGGCCCACACAGCTCAGGTCACGCCAAGTGGGGATTCTACATGATTGGAGCGATT

GTTCTCCAGGTTTTGACCGGATGGGCTCGTGCCAGGGCGCTTTCGGGCAAGAGCAACAAC

TTTTCACCGTTCCACAGGGCGAACAAGCACTTCCATATCTATTCCGGGTGGTTTGCCTAC

ATTGCCGGGCTTGCGCAGTGTTACCGTGGACTCACGCTCGTCGCGGGTTCGGATAAGCTC

ATCTTCTCCGCGGCTGAGATTATTTTTTCGTTAGGTAGGTTCGAAATCGTCACGACCATC

CTGTTTCCGATATGGATTGCGCTTGTGCCTCTCGTCTTCATCATCCTGGAAGTGCGGAAG

CAATTCACGAGGTATTTCAAGAAGGGGGCAGCCAATCTGTGCGGTATGGTGGAGCTTGTC

AACGAGGAATTCTCCGACGAGGCTCTGAAGAAGGAGGTAGAGGACCGGCTTATGCCTAGG

ACGGAAGAGCTACCCCTCTACACAACCCACGAGTTCAACGCCAAGGTCTTGAACGGGCGT

TCATGGGTTATCGTTGACGGAGCGGTCCTCGACGTTGGCTCTTTCGTCAAGCGGCATCCC

GGCGGCGCTCGTGTGCTCATTAATGTCATGGGTACAGATGTGACGTCGGAGATCATAGGA

GAAGACGCTTCCATCGGTAACTCCAGCATGACATTCGACCCGCACACGCACTCCGATATT

GCCCTGGAGATCGCGAGAAGCCTCGTTGTGGGATACATCGAGGAGGAAGACGACTTAGAG

ACGGATAGcagcgacgacgatgatgataatggcgGGGCTAGCATCGGCGCTGATCTCGGG

TTCCTCAACCGGTCGTTCCTACATGCTCGCCGGACGATATCGTTGTCCGGCTCCGACAGC

GAGGGCAACGATCGCCGCAGCTCGTCCCGCATCTTGCACCGAAGCCGCCGTCTCGCACGT

ACATCTCTCGGAAGGAAGAAGTCAAGCCACTCCGGTCGCCTTGCATCGTGGTCTGGCCCG

ACCTCCAGCGGCGACGAGGTGGATAGCAGAAAGTCATTCGAAGGGGGACGCAGATCGTCG

AGTTACAGAGCACAAGGCAGACGTACCAGCGGTGGGAGCAGCGGGAGCGGAAGTGGTAGC

GACAGTTTCGCCGGTGACGAACGGAGGAAGTCATTCAGGGGCAAGGGTAGCACCGAGGGC

GAACATTGGAAGCAGTTGAGTGTCTCCGACGGCAAGATGAGGAAGATGCATGGAGTGCAT

GGAGGCATCGCACCGGTAGGAGCGGCGGTGACACTAAGCGCAAAGGCAGCGGCGATCGTG

AACGCAACATCGCCACCGGCAACGGCGACGGCGCCAGAGCTGCCGACGAGGGGGCAGAAT

GAAACGGAGGTGGAGGGAAAAAGTGACGATGTACCGTCGGTTCCGGTGGATACCTCAACG

GCTGAGCTCACGCCTGTCAAGTCCGTATCGCCTATGTCGAACCAGCCACGGAAACGTTCG

TCGGCGCGCTCAATCTCAATCAATAGGCCAGTGGAGTTGGAGCATCCTGTGGTTATGCGC

ACTTCGACCTCGTTCAATACCAAGCCGGAGGTGACGAATAAGAAACCGAAGGGCAACCCC

GTCCGGCGCATATTAGGGCGAATAGGCTCGCAGTTTGACGCCATCAGCGGAGCCAGCGGC

GACGAAAGCGATATCATACAGGAAGACAGGCCGTTCAAAGCCATGCGAAGGTTGTGGGAG

AAGAAGAACTTGCTGGATTTCTTCCACGTTtgcccactactgctgcacgagaaGATGGGC

GACGGAGGCGACCGTCCGGTCTACAAGTTCATCTTCGCATGTCCAGGAAGGGCGGCGGCA

ATGACTGTCGTGCAGCGGTCCTACAACGCGTTTGCCGTGCGAGTGCAGGGTGAAGAGGGG

ACCGAGCGCCGGGTTGTGCCTGCGAGCCAGACAGACGAAGGCGAACTGTGCATAGAGATG

AGGATCAGGCTTTACCCGGATGGACTCATGAGTAGCCTGTTGCTGCAGCTGATCAAGAAC

TCTGACAATCCCGCAGTGCAGCTGCAGGGGCCCTTTCTGCAAGATAGGCTGATCCCACCG

CCGGCCCACCGCAACGTCGTCATGATCGCAGCCGGAACTGGCATCAACCCAATGGTGCAA

ATGATCCGAGACTACATAAACCCCGATAGCCACAGCCACGGTGTTCTACTCGGGGCACAC

TCGCGGCTAGTGTTGCTCTGGCAAAACAACGAAGAAGGAGACCTATTCTGCGCCGACGAG

CTCACGACACTGCAGGCTGAAGCAAAGGGCCTACTAGAGGTGACGGTTATTCTCAGCGGC

GATAAAACACGACGCAACATGCCGGGTATTGCGTTCCGTCGTGCGAAGGCGAGGCTCATG

AAAAAAGGTGGTAAGGCGGGCTTGGCCAACAGGGGTGCAGGTTATAGAGGGGACACCGAT

GGCGGGACCGGTGACCAAGCTTCGGCAAGAGCGACATCATCGGGGGCAGATGCGAGCAGT

GAGTTGAAACAAGAAGACAAAGACAAGCTTGGCGACGAGCTCATGAGAGGCGCACAATTG

TACCGTCACAAGATCGCGAGAGCACTGTCGCGCGTTTCGGAGGAAGATAGCATGAGCCTC

GGGAGCAGCTTCGTtaacagcagtcgaacatttgGCAGAGACTTGGAATCCggaggcgac

ggcggcagctTCCGTAGCCCCATGGTCCTCTGCAAAGGTGTGGAAGAGAAAGCACCTACT

GGagtcggtggtggcggtggcaacaAGGAACTGCCGTGGTCACCAATCGGCGGAGGCAGG

GCAGGAGGGAGCGGGGGCGACAGTGACCGTACCATACAAACGTTGAATGAACTGCGGCAA

ACGCGGAGTGACCCCCGTCGACACAAGGAGGCTTCTCAAAGCACGGGTCGTTCAGATAGT

GCACAAGAACCAAGCATTGCCACCCGTGTCGCTGGTTGCTCACGgaatttgtgggggggg

ggggaggacggcgggggcagcggcggcgaggaCAGCGGCGACGACTACGGGAGCCCAATC

CCACTTCGGCACAGTCGTGGTGATGTGCGTAGCACTTGCTCTTCTCCGACGCTATCTCGT

CAAGAGGCCACCCGAGACCTCCGTGACGCTAGCAGTGACAGGTCTGGTTGTGTGTCGGAG

GGCGCCAGGACTTACAATGGCAGCAGCTGCGAGAAGGCCACGCGTGGTTCTATCCACCTC

ATGGGCCCGTTCAGTCGAGAGAATGTTGGCCGAGGCCGCTGGCTTCAAAGCGTCCGTAAG

AAGGAAAGTAGCAGCCAAGCCAATaggggcagtggtggcggtggtggtgggaagcTTGTT

CGGCCGACGCTTGATCAGTCGAGTAGTTCCAAGATCGACATCGTTGCGTACAACCCAGGC

GAAGAGGTACAGTCCGACGCCACTACCGTCGCCGGCAGCTGCGGACGCCCGTCACGCTCG

CACCGCCGCGGGAGTTCGGAAAATCCCTCCTCGCCGTCGGGTGTTAACACTCACTCCAAT

TCAGCGCCTACAATAACGAGACACGAATCGTCTAGGAACAGCTCGTCGCGCGACATGGCG

GCTGAACATATGCCTGGATCGTCCCTCGAAGATTCGGGGAGTGGGCTACAGCACACGACG

ATTTCCACAGCCGTAGTAGAGAAGGCCATCGGTCCGGAGGTGATGAGAGCTATAGCCTCT

CTCAAGGCTAGTGAGGTCGCTGGTACGGTTCAGGGGGAATCGACTGACGCTGGAGATGGC

TCGCGCTCTGCCCTGAACACCCTGATCGGCACTGATACCACCGGAGGAGCGCTTCAGGTC

GTCGTGTCGGGCCCAGCAGGCTTCGTGTTCTATGTTGAGGGCCTTCTCGCGGAGATGAGC

ATCCCTCCTCAAGCAGTAGTGCTGCTCGACTAG

>g6154.t1

ATGGCGACGACAATGCTTCGAACGGCGGCTATCATGAGCTTCCTGGCGGTCAAGGCAGCC

GATGCGGCGGACACGGCTTCCCCGAACAAGGCGATCAGCAAGCCCGCGTGCGACTCCAGC

AAAGCCGTCTCGATCCGATACTCTTCGTCATCGGCGAGGCTGTACCTGGAGTCGGGGGCC

GGCTCAACGCGAGGCGGCTGCATGACCCTCACCGAAATCTGGGCAGACCTGGACGGCAGC

CCTCCCCTGTACGCGGTGGACCCGGACAGTGGCGACGTGAGCAGCACCGCGACAGGCACG

TGGCTTCTCACGGAGACCCTGTACGTTGAAGACGGCATCACTCTCCAGGTCCACGGTACC

TCCGCCGGGGGTGATGCCGACGAGTTGCGCTTGTTGAGCACGAGCGACACGTTCATCAAT

CTTCGTGCCCACGGCGGAAGCCTCGACTTCGTCAGCACGAAGGTGTTCGCCTGGGATACC

TCTAACAACGCCGTCGACGAAGACGAGGACGATGGCCGGTCGTACATCAGCGCCATCTCG

GAGATTATCACCGACAGCAGCGAGACCTGCGCTGGCAATGCCAAGAACACGATGGGAGAA

GCGCGCATGGACATCGAGGACAGCGAAATGGGCTACCTTGGATTCGCCGACGGCGAGAGC

TACGGACTCACATGGAAGGTGCGCGGGTTCTGCACGGACACGAGTAACCCGGAATTGTTC

GACGAGGTCAACGTCTACGGCAACATCTACGACTCGGACATCCACAACAACCACTTCGGC

GTCTACACGTACGGCCACCAGCAGggagactggaggaggaacaagatgcaCCACAACACC

GTCTACGGcttcgacccccacgacgacagcgacttcctgaccatccacgacaacgagGTC

TACGACAACGGGAACCACGGGatcatcgcctccaagcgcTGCAACGGCGTGTCCATCCAG

GGGAACGAGGTGTACGGTGGCGGTGACGAGTCCGCCGGTATATTCCTGCACCGGAGCAGC

GACGACGCCACTGTCAAAGGCAACTACGTCCACGACAACGGCGACGCCGGAATGGCGCTT

ATggagagcttcaacgctgaaGTGTCCGACAACCGGTTCGAGGACAACAAGTACGGCATC

AGGCTTTCCGTGGGATGCGCCGACAACGTCTTCTCCAAGAACGACGTCAGCGGCTCCAGC

AAGTACAACATTTTCACGTACACGGGGACTGATGCGCCGTGGGTGGCGGACTCGGGAAGG

CCGCAGGACAACGTCTTCTCGGGCAACACCATCACCGGAGGCGACGAGAGCATCAAGCTC

ACGACCGCCGACGGCACCGAGTTCATCGACAACACCTTCGTGAACGCCGCCACTGTCCGC

TTCGAGGACTGCCAGGACACCCTCATGTCGGGCAACACCGGCCTGGAAGATGCCGAAATG

AAGGTCACCGACGGCTCTTGTTTCATGGCGGGTTCGGACGCCGCCTACACCCCCGTTTGC

TAA

>g6156.t1

AtggcgacaacgacgacaaggCTTCGAGCGGCTGCCTTAATGAGCCTTCTGGCGGCGGCG

AAGGTAGCCGATGCCGCAGACACGGCCTCACCGAACGACCCCATCGAAAAACCCTCGTGT

GACTCCAGCAAAGCCGTCTCCATCCGATACTCGTCATCATCGGAGCGACTGTACCTTGAG

TCCGCGGACGGTAGCACTCGAGGCGGTTGCATGACCCTCACCGAAATCTGGGAAGACCTG

GACGGCAGCGCTCCCCTGTACGCGGTGGATCCAGACAGTGGCGACGTGAGCGATTCCACG

ACAGGCACGTGGCTTCTCACGGATACTCTGTATGTTGAAGACGGCATCACTCTACAGGTT

TACGGTACCTCCGCCGGAGGCGATGCGGACGAGTTGCGCTTGCTAAGCACGAGCGACACG

TTCATCAATCTGCGTGCCCACGGCGGAAGCCTCGACTTCATGAGCACCAAGGTGTTCGCC

TGGGATACCTCTAGCAACTCCGTCGACGAAGACGAGGACGATGGCCGGTCGTACATCAGC

GCCATCTCGGAGATCGTCACCGACACCAGCGAGGACTGCGAGGGAAACGCCAAGGACACG

ATGGGAGAGGCGCGCATGGACATCGAAGACAGCGAGATGGCCTACCTGGGGTTCCACGAC

GGCGAAAGCTACGGGCTAACATGGAAGGTGCGAGGGTTCTGCACGGACACGAGTAACCCG

GAGGTGTTCGACGAGGTCAACGTCTACGGCAACATCTACGACTCGGACATCCACCACAAC

CACTTCGGCGTGTACACGTACGGCCACCAGCAGggagactggaggaggaacaagatgcaC

GACAACACGGCGTACGGCTTtgacccccacgacgacagcgacttcctgaccatccacgac

aacgagGTCTACAACAACGGAAACCacggcatcatcgcctccaagcgATGTAACGGGGTG

TCCATCCAGGGGAACGAGGTATACGGCGGGGGAGAGGAAGCCGTCGGCATATTCCTGCAC

CGGAGCAGCGACGACGCCACTGTCAAAGACAACTACGTGCACGACAACGGCGACGCTGGC

CTGGCGATGATGGAGAGCTTCGACGCCGATGTGTCCGACAACACCTTTGAGAACAACAAG

TACGGCATCAGGCTGTCCGTGGGATGCGCCGACAACATCTTCTCCGACAACGTCGTCAGC

AGCTCCAGCAAGTACAACATTTACACATACACGGGGTCGGATGAGCCGTGGGTAGCGGAT

TCAGGGAGGCCGCAGGACAACACCTTCTCGGACAACACCATCACCGGAGGCGACGAGAGC

ATCAAGCTCACCACTGCCGACGGCACCGAGTTTATCGACAACACCTTCGAGGATGCCGAC

ATTGTCCGCTTCGAGGACTGCCAGGACACCCTCATGTCGGGAAACACCGGCCTGGAAGAC

GTCGATCTGAAGGTCACCGACGGGTCGTGCTTCGACGATGGCTCGGACTCCGCCTACACC

CCCACTTGCTAA

>g6160.t1

ATGCTGACAATGCTTCGGACGTTTGCGGTCATGACCCTGCTGGCAGCGAGGGGAGCCGAC

GCGGCAAACACGGCTTCGAAGAACAAGGCCATCGCGAAACCGTCGTGCAAGTCTAGCACC

AAGGTGTCCATCCGGTACTCCTCGACGTCCAAGCGACTCTACCTGGAGTCGGCAGACGGC

AAGACTCGAGGCGGCTGCGTCACGCTCGAGGACATATGGAGAGACCTGGACGGCGGCGCT

CCTCTGTACGCCGTGAAATCCAGCAGCGGCGACGTGAGCAGCACCATCACAGGGACGTGG

CTGCTCACCGAGAGCTTGTACGTTGAAGACGGCATCACTCTGCAGGTTCGAGGGCTCTGC

AGGGACCTGAGCAACCTCGAAGTGTTCGACGAGGTCAACGTGTACGGGAACATCTACGAC

TCGGACATCCACCACAACTACTTCGGAGTCTACACGTACGGCCACGAGCAGggagactgg

aggaggaacaagatgcaCCACAACTCGGAGTACGGCTTtgacccccacgacgacagcgac

ttcctgaccatccacgacaacgagGTCTACAACAACGGAAACCacggcatcatcgcctcc

aagcgcTGCAACGGAGTGTCCATTCAGGGGAACGAGGTGTACAACGGCGCTGCCAAGTCG

GTGGGCATATTCCTGCACAGAAGCAGCGACGACGCCATTGTCAAGGACAACTACGTCCAC

GACAACGGCGACGCCGGGATGGCGCTGATGGAGACCTTCAACGCCGACGTGTCCAACAAC

AAGTTCGAGGACAACAAGTACGGCATCAGGATGTCCGTGGGGTGCGCCGACAACGTGTTC

TCTAAGAACGACATCACCGGTTCTTCCAAGTACAACGTTTACACGTACCAGGGATCCGAT

GCCCCGTGGGTGGTCGATTCCGGGCGGTCGCAGGACAACGTCTTCTCGGACAACAACATA

AGCGGCGGCCAGGAGACCATCAAGCTCACCATGGCGGACGGCACCGAGTTCATCGACAAC

ACCTTCAAGAGCGCTAAGACGATACGCTTCGAGGACTGCCAGGGAACCCTTATGTCGGGC

AACAGTGGTCTCGGCGGTGCTTCGCTGAAAGTCACCGACGGGTCTTGTTTCGACAAGAAG

TCCGACAGTGCCTACAAGCCTGTTTGCTAA

>g6161.t1

ATGCTGACAATGCTTCGGACGTTTGCGGTCATGACCTTGCTGGCAGCGAGGGGAGCCGAC

GCGGCAAACACGGCTTCGAAGAACAAGGCCATCGCGAAACCGTCGTGCAAGTCTAGCACC

AAGGTGTCCATCCGGTACTCCAAGACGTCGAAGCGGCTCTACCTGGAGTCGGCAGACGGC

AACACTCGAGGCGGCTGCGTCACGCTCGAGGACATCTGGAGAGACCTGGACGGCGGCGCC

CCTCTGTACGCCGTGAAATCCAGCAGCGGCGACGTGAGCAGCACCATCACAGGGACGTGG

CTGCTCACCGAGAGCTTGTACGTTGAAGACGGCATCACTCTGCAGGTTCGAGGGCTCTGC

AAGGACCTGAGCAACCTCGAAGTATTCGACAAGGTCAACGTCTACGGCAACATCTACGAC

TCGGACATCCACCACAACTACTTCGGAGTGTACACCTACGGCCACGAGAAGggagactgg

aggaggaacaagatgcaCCACAACTCGGAGTACGGcttcgacccccacgacgacagcgac

ttcctgaccatccacgacaacgagGTCTACAACAACGGGAACCacggcatcatcgcctcc

aagcgcTGCAACGGAGTGTCCATCCAGGGGAACGAGGTGTACAACGGCGCTGCCAAGTCG

GTGGGCATATTCCTGCACAGAAGCAGCGACGACGCCATTGTCAAGGACAACTACGTCCAC

GACAACGGCGACGCCGGGATGGCGCTGATGGAGACCTTCAACGCCGACGTGTCCAACAAC

AAGTTCGAGGACAACAAGTACGGCATCAGGATGTCCGTGGGGTGCGCCGACAACGTGTTC

TCTAAGAACGACATCACCGGTTCTTCCAAGTACAACGTTTACACGTACCAGGGATCCGAT

GCCCCGTGGGTGGTCGATTCCGGGCGGTCGCAGGACAACGTCTTCTCGGACAACAACATA

AGCGGCGGCCAGGAGACCATCAAGCTCACCATGGCGGACGGCACCGAGTTCATCGACAAC

ACCTTCAAGAGCGCAAAGACGATACGCTTCGAGGACTGCCAGGGAACCCTTATGTCCGGC

AACAGTGGCCTCGGCAGTGCTTCGCTGAAAGTCACCGACGGGTCTTGTTTCGACAAGAAG

TCCGACAGTGCCTACAAGCCTGTTTGCTAA

>g6320.t1

ATGGCTTCTACCCTAGCGGCGACAACCACGTCTTCGCGGCTGTACTACCCATTCAGAGCA

CTAGGAGCTGTGACTGATGGCTTGCCTTTTGTGCTGAACAGGAGAGGGGATGAGTGCTTC

CTCGCCGTGTCCATCGACCGAGCATTTCAGATCTACCGATGTGACCATCTCCGCGTGGTG

CTGGTATCTCCACCGATGAGCAAGAAGATCGCTTGCTTGGAGTCCTGGGGCAACGAGCAC

ACCTTCGTGGGCTCCGGGGGTGATGTGCTGGCCTGGCGACGTCTATCCTGTCTGGGGACA

GTGGGGTCACACCCTGGCACGGTGCGCTTCCTACTTTGCCTTGGGGACGTACTGGTGTCT

CTGTGCGACCAAGGACGGCTCAAGGCTTGGGACCTCAAGCGCCGAAAATTCGCTCGCACA

GACCAAGAGAGggaagacgacaaggaggagggggaggggggggagggtcgtgAGGCCGGC

GCTATTTGTGACGCTGCTTTGGAAGGAGGTTTCGTACCCACTTCTCTAGCGCACCCACCG

ACGTACCTCAACAAGGTCGTCGTGGGTTCGGAGGGCGGCGCGTTACAGCTGTGGAACGTG

CGGACCGGGAGGAGGGTGCACTCGTTCGGGAGCATTGACTCCCAAGGTGCGGCGGTTTTG

TGCATCGAGCCTGCCCCGGCGCTGGATGTGGCAGCCGTTGGCCTCTCGGATGGTCGCGTT

CAGGTGCTGAATCTGCGGACGGACGAGCTGCTGATGACATTTAAGCAGGAGGTTGCCGTT

ACGAGTCTCTCGTTCCGAACAGACGCTACCGCGGTTGAGCTGCCCCTGCTGGcatcgacg

ggggggggtggtcgcgtTTGCCTGTGGGACCTGAAAGAGCGCCGCCTTCACCACACCATG

GCCGCCCACGACGGAGGTATCTCGAAGCTGCAGTTCTTGCCCAGGGAGCCTGTGCTGGTG

ACGAGTGGCACGGACAACTGCATCAAGATGTGGGTGTTCGACTCGCCCGATGGCACAGGC

CGGCTTCTGAGAAGCAGGGAGGGTCATCGTGCACCCCCCCGTGCCATTCGGTACTACGGC

AACACCACACTTGCAAGCATGGCGGAGGGGGCTGATGCGACGGCGCTATGCGTGCTCTCG

GCCGGCACGGACCGCTCCTTCCGGGTGTTCCACACGGTCAGGGACTGCTTGAGCCAGGAG

CTGTCGCAGAAACCGCTTGTCAAGGTGGCTACCCGACATCGAGTGACGACTCAAGATGAC

TTCAAGCTTCGGCCGGTGCTGTCGTTTGCCGCTAATGAGACTAGGGCTAGAGACTGGGCC

AACATCATCACCTGCCACGAGGAGGATAGCAACGCCTACGTCTGGAGCTATGCCAAGCGC

GCCGTCGGAACGCACGTGCTGAGGCAAAAACACTGGCCAGGAAACGCCATGATGCACCCT

CCTGACCCTAGCACCTTCGCCACATCTGTTGCCATAAGCGGCTGCGGTAACTATGGCCTG

GTGGGGACGCGGGGCGGTCACGTCTTCCGGTACAACATGCAGTCGGGGCAACCGAGGGGG

TCGTATCCTCAGTGTGCTACTCCGTCGCCCAAGGCCGTCAAGGCTCTGGCCAACGTGGTG

AAGCCTGGTTCGATCGCTAAGATCACCGCCGACGGGTTTTCGAGCAAGCGGATTGCCGCT

ACAGAGGCGGCAGCTGCCAGGGTGAGGGCGGGAGCGAAGGGGGGGTGGCAGGGACACTCT

GGAGCCGTCTCCGGCATTGCCGTTGACGCGGTGAACCAGACCATGGTGTCCGTTGGGGTA

GACGGGCTTCTCGTGTTCTGGGCCTTCAGGGAAAAGAGGGCCGACGGAGCTATCGCCGTG

GGGTCCGGGGTGTCGAAGCTTGAGCTGGTACGCGATACGGACCTGGTAGCCCTGGCGTGC

GACGACAAGGTGGTGAGGCTGTATGATCTCGCCACGAGAAAGCTCGTGAGGAGGCTAGAG

GGACACAGTCAGCACCTCACCGACATGTGCTTTACCCCGGACGCGAGGCGCCTGGTCACA

GCATCGATGGACCACACCATAAGGATATGGGATCTTCCGACGGGTAGGATGGTGGATTGG

ATGTGGTTCAAGAAGGCGGTAACCGGCGTAACCGTGTCTCCCACTGGGGAGTTCATGTGC

ACCTCGCACCACGGGAGGGTGGGCCTGAGCGTGTGGGCGGACCAGagCTTCTTCCAGCCG

GTGTACTTGGACAAGATCCCGTCGAAGCCCTTCAAGATGGACGACCCGGCGCCTCTGGTT

GAggacagCGCTGCTTTGGAGGTCGACGACAAGGACAGCACAGCCGCCCTCCACGGACAG

TATCGACAGGACAAGAGGGCCTTCAGGGGGGCCAAGGcctcggctgggggggggcgcgac

gaGACCTGGCAGTCCGAAGCGGAGCAGGCGGAGCAAGACGACGAGACCCGCGCCATCACC

CTCAGCTCCCTGCCCCGCGGGTACTGGGCCACCCTGTTCAATCTCGAGGTGGTTAAGGCC

CGAAATCGTCCGATTGAGCCCCCCAAGAAGCCGGAAGCGGCGCCTTTCTTTTTGGCCACG

GTACACCGCGAGGGGGAAGTCGCTCCTTCTTTCGCGGACATGGCTAACGCTAGCCCAAAC

CCTAGCGCACCTAAGGtgccggtggggggggcggcggcggaggcagcgttggtaggtggg

ggtgagggtgtcCCTACCAGTGCTGACTTTCCttcggggggaggaggagcatgGTCAGAT

GACGACGATGAGGACGATgtcggggagggagaggaagggggcaAAAGCCGACCGGGAGGG

gatgaagggggaggggaggcgggcgCGGGAGGGGTCAAGCGGAAGATGGCTGAGGTGGCG

gcaggagcgggggcgggggtggcggctgCCCctgacgcgcccccccccgcccggaggtCG

AGGATCCTGAAGGAGATCAAAACCTCGGCCATAATGGGGGCTTCGGGACCTTCCCGGTGC

AAGCTCGCTGACCTGCTGCTACACTGCGAAGAAACCTACTCCCGAAGATCGGGCGACGAC

atggacgaagaggaggagggggaggggggggggggggcgggcgttgAAAGAGTGGGGGAG

GCAAGCGGCGGAGGTTTGACGCCGTGA

>g6641.t1

ATGGGGAGAAGAACGTTGCTGCTGGGCGCTACTGCTGGTGCCGGCCTCTTCCTTGGCATC

GTAGCTGGCTCGAGCGACGACTTGAATGCCCCGCGGCCACCATACTACAAGAAATGGCGA

CAAAACCCCAAGGTGGTTGTGTTCGGCGACTCCACTTCCGATGCAGGCAGGAGGTTTAAC

GCCCCGGCGAGCTTCGACTTCGACGACATCGGGAAGTTTCCGTTCACAAAATTGTTCGAA

GAGCCGGACAGTGATACAATGTTCAGAGCGTACCTCCCGGATCCTGGGTCCGTCACCAAC

GGCAAGTCGTGGCCGGGGTGGCTCAATGTGCCCGAGGAGCTCAACTACGCCACGAGTTCC

GGCACCGCCACCGACGCGTTCCGCTCTCGCGAGTCCTGCACGGGGTACACTGGCGAAGGA

GAAGCCTTCCCCACAGCGACCTTGAGCGAGCAAGTGACCCGGTACTTCAACGATGTCCTG

GATCACACAAATGATACCCTTGATTACACGCACATCATCAATATCGGTAACAACGACTTC

GGCGGGCTGGTCTCGGCAGCAGAACGCTACGCCGCCGGAGGGGCTGGCTACGTCGACCCC

GTCGCTTTCGAAAACGTCTTCGAAGTCGTCGACGGAGTCCCAACACTTACTTTTGCACCC

TTCATGAGGGAAGTCACACATGCGTGGCAGGAGGGAATCGACAGGCTGCTCGAGCGCGGG

GTGACTGGAAGGATCCTGCTCGCCAACCTTGTGTCGACAGACTTCTTCTCGGGTTCCGAC

TCCAGCGGACTGGAGCTGGCGGACACCATCGACGATGTGTTCACCCAGTTGCGGCTGGAG

GTTGCCTCGATAGCCGAGGCCAACCCTCAAGTCCGGGTACTCGACTTCTCCAACGTGACC

TTGGCTCTGTTCACACAGCCTGAACTCTTCCAGAGCCTCGGCTTTACGACGTTGACGGTA

CCTTGTCTGACCTTCGAGTTCGCCATAGATTCAACGGCCGAGATTATGGGGACTCAGGCT

CAACGTGCTGCAGGGTGCCAGGAGGAGTGCGCCCTTTGCGCCGACCTGGCTTCGCCCTGT

CAGAACTGCTACCAGGGAAACCCGTCCGCCACGGTCTGCGATACACCGGAGACGTACATA

TTCTGGGACACCCTCCATCTGACCACGGAGGTCCACAAGATCCTTGCAGAAGCTACTAGG

CAGTGCGCCAAAGACTTCCCGAACTACGACCGCCCCCTCGTGGCCTTGCTATGCCCGGAG

GGTTCGTGA

>g6678.t1

ATGGCAAGGGACGGGCAGTCACCGCCGCCGGGCCAGGCTTCCTATGGCGCAGTCGCGGGG

AGTCCCGACGGCGAGGACGCTCCGACCTTGCGAACGCGGTATGATCACGGCGTCGAGTCT

GGATTCGAGGACCgaagcaacagcaggaacaacaGTGTTCAGCCGCTACTGTCCAGCCGT

GTTGCCTCACCAATGGCGTCGCCCAGCCGCGCTTCCGAGAGAGAGAGCTGGTCTTGGTGG

CGAGTGGGAACGATtgcggtagcagcaacagcgctgTCCGTCGCCTTCAGCATGTCCAAC

CTTGGCCgtgcttcggcggcggcggacagCGCGATGTTGGGCGCAACAAGTAACGGAGGA

AGCGTTGACGCTGGCAAGCCATCACAGCTTgcccagcagccgcagcagcagcagcgtgcc

gggccgaccgtcatggagacggaggcggcggcagaggtGGCGGCGACCTCGGAGGAGCTT

TCATTCGTGGCGATGAACGACTACACCCGACGCGGGGATATTGTCGGCTTGGGTTACTCG

TGGCTCGAGGGGGGGATGTTGGTGGAGCCATATCGTGAAACAACGCTCGAGGTTGTGTCT

CCCCGAGAGGGGATGACATACTCTTGGGTTGTCACGGGGGtcgcgggggcggggagaggg

gggcaggtTACACTTGGCGAGTACGTGGGGGAGGTGGTGGAAGTTATGTTCTTGAAGGAC

CCCCTGTACACGATCGTCCTCGAagagagaagcagcagcagcggtgttgctgttgctggg

ggtgctggtgctgctggtggtggggtAACTAGGAGCACATCGGTGGACGTATTCTGCAGA

TACGTGCGGAGAGAAATCAGGTCGCTCTTCGACGACGAGCGAAATGAGATGTTCGACGCC

ATGaagGTGCTGTGGGACGTGAAAACGGAAGAGGGGAAAGTGCTGTACGGAGAGCACTAC

CTCGACGTGTGGACGATCGACATGTTCCACCAGGTCGGGGCGTCGGACATCAACTGCGAC

CACCTCCACGACGGAATCGGATTCGGCATCACCCACTCGATGGTGACCATGATGTTCGAG

GGCAGCCTCCAGCTCGTCAACCCCAAGCTGAGCCTCCCGTACTGGGACTTCACCATCGAG

GGGGCCAAGATTGAAACCGATTTCGACGGGGACTACAGCAGGCTCAGAGACGCGTCGGAG

CTCTGGTCGCCAGAGTGGTTCGGAGGTGTCGACCCGGAAGACtttcagGTGAAGGACGGG

AGGTGGGGCTATATGGAGGTGCCCATCGTCGCCCAGGAGCACCGTGACATGCTCGGGGCC

GACGTCTACGGGCGCATGCGCTCGCCGTGGAACGTGAACTCGCGGGAGTATCTGACGCGC

GGCCTGGGGGAGATGTGCGGCGTGGAAAGCACTGATTTCTACACCTGGCCTGGTTGCTCC

AGCCACTACGACTTGCAAGACGTCTTCACGTCGTACTACGGTTACACTTGGTGGTCGCAG

TACGGACCCCACGGGCCTGTCCACGTCTGGGTTGGCGGAAACGTCGACTGCAAGAAAGAC

ATGGACACGGTAATGGACATCGTGGGTGAGGATTGGCGGCTGTCTCTGTCGGGCTACCTG

TTCGTGATGCGCAAGGGCATGTTCCGCGACCACCTGTTCAAGTGCGAGGGGTACGCCGAG

CCGGACGTGACCGAGGAGGATATCATGAAGTCGGGGCAGTGCGGGTGTCTCGACCTGAAC

CTCGAAGAGGGCGACGACTACATCCAGGTGTACGAGGAATTTGCGGAGGGTTACGACTTC

GGGTTCTTGACGGAGGCACAGAAGAAGACGCTCGCCGTCCAGTACTGCAACGGCCAAGTG

GGCATGGGAGAGTCGTCCCAGTCTAGCTCTCCGTTGGACCCGACGTTCTGGTCGATGCAT

CCCACCATGGAACGCCTCCTTGTCTTCAAGCTCATGTCCGGCACGTTCACAGACTTCTCG

TGGCCCGATGAACCCGGCACGTGGACGGGCATGGACGGAATAGAGTACGACGTCGAAATA

TCAACTGAGCAAGACGAGTGCTACGGCCACAGGGGCAGCGACGTCTTCCCGTACACCATC

CTCGCCGACCTGATCACGCCCGTGTTTACGTTCTACGGAGAGACGGGCCCCAACACCATC

TCCAACCGACAGATGATCAAGATGTTCGACCCCAGCGTGAACGCGCTGCCCTTCATCTAC

GACAACTTCGAGTGGCCCCACTGCGCGGCGCAGGGGTACGACTTCACCGACTTCTATAAC

CCGACCACCGGCAGGTCCGGCCCGAAGGGCATGGCTGCGACTCCGGAGGAGAAGTGGAAG

CCCGGGCGCGTCCACGGCTACGGGCTGTTCGAGAAGGGCCAGAACCACAGGTCTCCGATG

TTCGCCGCCGCGAACCGCGCCGCCGCGGATGCTGCGGCGTCTAAGAAGGCTGAGCTTGCG

GGCAGGCTTGCGGGCAGGCTTGCGGGCAGGCTTGCGGGCAGGCTTGCGGGCAGGCTTGCG

GGCAGGCTTGCGGGCAGGCTTGCGGGCAGGCTTGCGGGCAGGCTTGCGGGCAGGCTTGCG

GGCAGGCTTGCGGGCAGGCTTGCGGGCAGGCTTGCGGGCAGGCTTGCGGGCAGGCTTGCG

GGCAGGCTTGCGGGCAGGCTTGCGGGCAGGCTTGCGGGCAGGCTTGCGGGcaggcttgct

tgcttggctaTAGCTGGATGGCTGGAGCTGCTCGATCGGTCGGtccgttga

>g7003.t1

ATGAAGGAGGAGAAAGGTCTGGCCAGAAAGCTCGCGAGCTCTGTGGTGGCAGTCATCGCC

GCAGGGCTATGCCTGGTGTTGATCAACTACGCAGTGTTCATCACCGAGAGCATCCCGGAC

TGGGGCTCAGAGACGAAGGATGCCATGGTCGAGCTGGAGCAGGCCAACATGGCAAGGTTG

GCCTCGGACAAGGCTGAGCACACGGGGTACGTTTTTGGTCTGATGAAGGATGCCTTGCAG

CAGCTCCAGGGCTTCGCCGAGCAAGCGCTCCCCCTGGCGGGTTCTGAGACGATGGAGGTC

GACCGCTACTTGATGAGCTTCCCGGGCCTGGAGCAGGAAACGCCCGACACGAACCACAGC

GTTTGGTATGTCCCCGGGCTCGACCCAGGGACCTCTCCCGCCGTCGGTAGCTCGCTCGAT

GGCCTTCTTAACCGCACGGCACTGATGGACGTGCTGTTTCGGGGAATACCGCGGGAAGGG

TTGATGTACCTGGCCGCCCTGGATGACCCGTCGCTGATCGACGACCTGGACTCGTCGGTC

GTCCTGCAAATGTACCCTGCGTACGACTTGCTCGAGGCGGGCCTTACGACATTCAACGAG

GATTGTGACGACCGCTTCGACACCGGCGACAACGACTTCTCGCTCTCCTACTTCGATCCT

CGATGCCGGGTGTGGTTTCAGGACGCCCGCGCAGACAGCCGCAACGGCGAGCCCATATTC

ACCGATCCGTACGCCTCCGCGGGCACCGGGGCGCTCACCATCACGCCCGCAGCTCCGGTC

TACGCCACCGACGGCGCCACGCTGCTCGGCGTCGTCGGCATCGACATGGACTTCGCGCTC

ATCGAGGCGTCCATCCTCGGCCTGCGCATCATGGGCGACGAGGGGTACGCCTACCTCCTA

ACCCCGACGGGGGGAGAGGTTGCCGCCCACCCGCGGCTGGACGTGCTCGACGGCATCCAG

AATATCGCCGACCTCGAGCCGGGCGTTGACAAGGATGAGTTCGGCCTTCTCGTGGAGCGC

ATGACGAAGGATTGCGCGGGATCGGCGAGCTACCAGAAGAGCGGCGAGACTTGGCTGGTT

TCGTGGGAACACGAGACAGTCAGCCGCTCCGGCGCCGACGCCGCTACGGATAGTGGTGGT

GGGTCGAGCTTCTCGTCTTGCTCAACGGGAGGGTTCATCGTCGCGGTGACCGTCAGCGAG

GGCACCCTGTTGGGGGTTTTTTCCAAAACAGATTCAGAGATCCGAAGCATTGTGATTGTC

GCTAGCgtcgtggtggtgctgttgatgACGGTCACGGTCTGCCTCACGGTGGGGGTTGCG

CGTTCCGTGTCCACCACCCTGACGCGGCCCGTCAACCAGCTCGTGGACATTGTGAAAGGC

CTCAACAACCTGGATTTTTCTGAACAGGACCCCGGGGTGTGGATGCTGAACGACATCAAC

TGCCCGGAGGTGGAAGAACTAATGGAGGCGTTCAAGAGCATGACCACGATAATACACTTC

GGAAACCAGACCCTAGGGAGCGGAGACTTGGACGCCGCGCACAAGAACTACTGCGACGCG

CTCTTCTTGTTTACGAAGCTCAACAACGATCGTGGGATCAGCATCGTCCGCAACAACCTG

GGGAATGTGTGCACCTTGCAAGCGCGAGCTCTCACAACGCGCGCCAAGGTTGAGCTGAAA

CAAAACAAGGGGGGTGCCGCCCGCGCCGACGCGTTGATGCGCGAGGCCGACCAAAAATAT

GAAGACGCCGTCACAAACTTCCGGCTGGCGATAGAAGACTCCGAAATGGCTGCCCCCACG

GCAAGAGGCAGCGGGGACAGCGAATCTCCGCACCCTTTTCATGCTCTGTCCACAACAGTC

CCGAACGACGCCGCTGAGcagaacgaggggggggggagagagcagAAAGGGATAGCTCTG

AAACCCGCTCTCCATGACATCGACGTTGTCGAGGCCCAGCAGACGACATGCAGCGAACCG

GGAGACCCCGACTCGACCGCATCACTGGCACTGCAACTCGCGAACCGTAAGTTCAACCTC

GCGCTGTGTCTGGCCGCCCAGGCGACTGGTGGCGGTGAAACTCGGAACGACAAAGCGGTG

GGCGAAGCTTGCGAATTGATGGCGGAGTGCGAGGCCCTGGTTGCCGAGAGAAACGACGCC

CTTGGCACCGACCGCCAGGTAGAGTACCTCCTCGCCCGCGCCGCCCTCGAATGCACGCGG

CCTGGTCGACACAAACAGACCTCCGAGGTGCTGGAACGTGCCGATAGGATCATCACCGCG

GCACCCAGTCCGCCAGCCACTGCCCCAGTTGCCACCACCGTgaccgcctccaccgcctcc

gccgcctccgccgccaccgctacccctCCGGGCGTGCTGCGGCAGCGACTGCTAGTCGCG

CGGGGGGAAAGCATGCTCGCCGCGGGAGAACACCAACTCGCTGTTGAGTACTGGGCCGAG

GCGATAGTCGGTTGTGGCGAGCTCATGGATGTCGGCGCTGTGACGTCCTCGTTGGTGAAC

CTCAGAGAGCGGGTGGCCTACTTGCGAGCCGGTGAGGGGTGCCAATTTTCGGCTGATCTG

GTTCGAAGCTTAGGTTTCTCGAGCGAAGAGGGAAAAGAGGACAAGGACGGTAGTTCGGTT

TCGGAGGCAGATTTGGTGGAGGCTATTGACTTGCAGGTGAAGAAGCTGGGCGGCGGTGCA

CTTTGGTCGACCGAGTTTGATGCTGACTTGCGCTCTGTCGCGGACTGCACAGGACCGGTC

GAGGTCGATTTGTGCTTCGTCATGGACTGCACTGGGTCGATGGCCACATGGATCCACAAG

ACGAGGCAAAAGCTTTTCGACATCATCGAGCAGACCAAGAGGGAGGCTGAAAACCTCAAG

CTGCGGGTGGCTTTTGTCGGCTATCGCGATTATGGCGGCAGGGTGAAACAGTACGAAGGT

CCCTACGACTTTCACTCTGAGGAGGAGCTTCCCGAACTCGAGAGGAAGTTGAGAGATATC

AAGGCTGGAGGAGGGTCTGGTTACGTGGCCGATATTGCCGGAGGGTTGAAGCGTGCCACT

GAATTGTCGTGGCGCAGCCCCGTGCGTCTGTGCATCCTGTTCGCCGACGCCCCATGCCAC

GGAAGCATTTACCACGACTTTCATGACGCGTACCCCAAAGGCTGCCCCGAAGGTGTAGAT

CCGGCGAGAATGATCTGCAACTTGCAGCAACTAGGCGCGGACTTCTACTTCATTCGAATT

TGCCGGGGAACCGATAAAATGGTGAACCTGTTGAAGGAAACCGTGCCGGTCATGGCGGCC

AAAGACGCTAAAAAAGCGGGTAAGAAGAAGGGGGGCGGCGCGGGCAGATCGACAGCGGCG

GCAAAAAAGAAGAACCACAAGGCCAAGTTTGTGGTGCACAACCTTGAGGCGACGGAAAAC

AGGTTTTTGGAGGTGGTGGTCAACAGCGTGACGGTTAGCGCCGGGCTCAACCTCCGGGTC

GAGGGATACTCGTGA

>g7053.t1

ATGGCGACTTCAATGATTAGCAGAGTGGGGCTAGGGCTCCTGATGATTTCTTCTTGCGCT

CGAGCTATGGCCTACGATCCCGATTTGGGTTCGCCCTTTGGGGCGACTCCCGTTGAGATA

CCCGGTGTGATCCAGGCGGAAGAGTACGACAAGGGTGGAGAAGGCGTGGCATATCATGAC

ACCACCCCTGGCAACAGACGCGGGGCATTCCGGCCAGACGAGGATGTGGACATTTTTGCC

TTGAAAACCGGGTACGCAGTCGGTCACGTCCACCCCGGCGAATACCTGCGATTCACCGTG

AATCTCGACAATCCAGTCAACGAGTTCAGCTTCAACTTCATGATGGCTTCCACGACCAAC

GTTGGCACGTTCCGCATCGTCGCCGGCGGCACTGGGTGCGACGACTATACGACCGACCTC

AGCGGCCTCGTTGAGGTGCCCGCTCAACTCAACTACCGTGGCTACGAAGTATCCGGCCAA

GGAACTGGGGGGCTCGACAAAGGCGTGCAATTCATCTGGATGTGCGTGGTTTCCAGCTTC

TTCGACTTAGACTCGTTCACTATGACAGACATCTCTGCGCCGCCATCGCCGATTCCAACT

CTGGCGCCGACCGCAGCCCCAGTGACTACCATGGAACCTCTTGGTAATGGCGCGGCCTAC

GGAGGTATCCCTGTGGTGGTTCCCGGCATTCTTGAGGCTGAAGAATTCGACCTGGGTGGA

GAAGGCGTGGGTTATCATGACACCACTCCGGGAAACAAGAAAGGGGAGTTCCGCCCTAAC

GAGGACGTGGACATCGCTGTCTTGCCCAACGGTGGATACAACGTTGGGTACATCGAGGCT

GGCGAATACCTTCGATTCACGATAAACTGCACCAAAAAGATTGATCTGGTGTACTTCAGC

TTCAGGGTGGCTTCAGAAGACGGCTTTGGCTCGTTCCGTGTGGTTTCCGGTGGCGAAGGC

TGCCAAGACTATAAGACCGATCTCAGTGGCCTAGTTTCGGTGCCGTCTTCGGGAGGAAAT

CCTCGCTACCAAGACGTGGAAGTCGCCGGCCACGGTACAGGTGGCCTCCGCGCTCACGTT

TCCTACATCTGGCTGTGTGCGGAATCCAAAGGCTTCAACATCGACTCCTTCACTATGTCG

AAAGCCAATACCTGGAACCCACAAACTACCCCAGCCGCTTCAGACGTTTCGGTAGTCGAT

GCCTTCGACAATGTCCCTGCGACTATCCCCGGCATTATTCAGGCGGAAGAGTTCGACACA

GGTGGTGAAGGCGTGGGTTACTCAGACGCCACTCCTGGAAATGAGAAAGGGGTGTTTCGC

CCTAACGAGGACGTGGACATCAACGTCTTGAGCGACGGTAGTTACATCGTCGGCTACGTC

GCTCCTGGCGAATACCTTCGCTACACCGTGACAGTCACCAAAAAAGTTGATAATGTCGTG

TTCGACTTCCGAGTGGCTTCGGCTGATGGCCTTGGCTCGTTCCGTGTGGTCTCTGGTGGT

ACTGGGTGCGACGACTATAAGGCCGACCTCAGCGGCATCGTTTCAGTGCCGGCCACTGGA

GGAAATCTTCGCTTCATTAATTTGATAGtGTCCGGCAAAGGTACCGGTGGCCTTCGTGCT

CAGCCATCCCATGTTCGGCTCTGCGTGCTGACGAAATCCTTCAACATCGACTATTTTTCT

ATGTCGAAGGAATCCACCAAAACCTAA

>g7112.t1

ATGCCTCGAAGTGCTCATGCACCCCCGTCCCGGCGCTCGGATGGGTCATCCGAGAACGCA

CGGCGCGTGTCGGAGGACGGCGCAGGAGAGGAGAAGTGCACCACGGTTGCCATTCGCCTG

AGGCCCCTGAACGAGAGGGAGACCGCGGGAAAGCAAGGCAGGATTTGGCGATGCGTTCCT

ACCCACAACTCCGTCACCCAGACTTCACCCGAAGGCAACCCACTTCCGGTCGGAAAGGGC

AACACGTTCTTCACATACGACCGCATCTTCGACGAGGACAGCTCCACCCAGGAGGTCTAC

GAGGGCGTCGCGCAGGAGATCGTGCACTCCGTGGTTCGCGGTATGAACGGCACCATCTTC

GCGTACGGGCAGACGTCTTCTGGCAagacgttcaccatgcagggGGACGGGGAGAACACC

AAGACTCCCGGCATTGTGCAGATGGCCGCGCGCGATGTGTTCGACCTCATGGGCGAGTCG

CAAAACCGCGTGTTTCTGATGCGGGTGTCGTACCTTGAGATCTACCAGGAGGAGATCAGG

GACCTGCTCAACCCGGGAAGCGCCCAGAGGCTGCAGGTCCGCGAAGACCCGCGGAAGGGC

GTCTACATCGACGCGCACGAGGAAGTCGTCTCGAACTTCAGCACCGTGCTCAACCTCCTG

AGCGCGGGGGAGAAGCAGCGGCACGTGGGCTGCACCGAGATGAACAGCCGCTCCAGCCGC

TCGCACACGCTCTTCCGCCTCGTGGTGGAGAGCCAGGAGCGCTACGTGGACGGCGTACAC

GCTTCGCCGGAAGATGTCGACCCCGCCGTGCTGGTGGCCACTCTTAACCTCGTCGATCTC

GCGGGCTCCGAAAGCGTTCGCCACACGGGCGCAACCGGCATCCGGCAGAAGGAAGGGGGG

AACATTAACCGTAGCCTCCTGACCCTGAGCCGCGTTATCCAGACGCTGAGTCAGGGCGGC

AACGCGCACGTGAACTACCGCGACAGCAAGCTCACCAGGATTCTGCAGCCCAGCCTGTCG

GGCAACGCGGGCATGGCCATCATCTGCTGCGCGACCGCGGCCGAGGGCTTCCTGGAGGAG

ACCCGCAGCACGTTCCAGTTCGCCGCCCGCGCGAAGGAGATCAAGACCAGGGCCACGGTG

AACGAGGTGCTGGACGACAAGGCGCAGCTTCGGCGCATGTCGCAGGAGCTCGCGCAGCTG

AAGCGGCAGCACGCGGAGGGGGGCAGCGGCAGCGCGCTTGTGGAGACGCTGCAGGCCGAG

AAAGCGGAGCAGGCGGCCAAGATCGAGCGGCTGAAGAAGCTCCTCATCAACACCGCGCCC

GCCGAGGTGGACGACGTCGAGCGACACCACATGGCCGTCTCGCCGAGGTACCGCCGCAGC

AAGCGCTCGCGCGAGACCTGGTGCCCCGGGGAGGGCGCCCTCGCGCTGGGCTTGAACTTG

CTTGAGCCTAACTCGCGCATCGCCACCATCGAGGAGGAAGGCCTGGAGCACCTGAGGAAG

CGCAGGTCCAGCGCTGGCCAGTCGACGCCGTCGTCAAAGTCGCGGCCGTCGACGTCCTCT

CCGTGCACGCCCCACGACGCTCGCCGTGTTACCCCGCCGACGCCGGGGATGACCCCGGCG

CGCGCTCGCGCTCACCAGGAGGTGGCCGAGATGCGCGAGCGCTTGGAGGCGCTTCAGGCG

GAGAGGGACGCGAAGGCGGCCGAAGCTGAGGCTGCCTTGGCGGGGATGGCTCGCGTCGAG

CAGCTCGAGCAGGAAGTTTCGCGCCGAGACGCCGAGGTTTTGTCGCTGAAGGAGAAGTTG

GAGGCATCTTCGTCGTCCGCAGGTCAGTCGTCTGTTGTTGGGACCGAGGAGGTGCGGGGT

CGGGTGGAGGAGATCACGGcgcagctggaggaggcggtgaAGGCGAAGGAGGACCTGGAG

GCGGAGCTGACGGAGTTTGGAGAGTACACGGACATGAAAGACGAGGAGATGGTCGACGTG

AAAAAGAACATGGCCGCCATGGAGGAGGAGCTGCGGCAGAAGGAGGCAGAGCTTGCCGCG

ATCCGCGCGGCCGAAGAGAAAGAAGAAGCAGACAACGCCTTGCACGTGGCGGAACTCCAG

GGCAGGGTCCGTCAGCTAGAAAACGCCATCAAGGAGCGCGACGAGGCTATCGACGGCATG

GATGTCGCGGGCGCGGACTCGACACAGAAGGAGGAAGAGCTCGAGACGCTGAAGGCCGCC

CTGAGTGCCTGTCAGGCTGAGCTCGCTGAACACGTCACCCGCGCGGATGGCGCTGAGGAG

GAACTCGAAAGGCTGGTCCTAGAACTTTCTTCCACCAAAGCCCGTCTCGCCGAAGAGCGC

GAGGCCGCCGAGCAGGCCGCCCATGAGGCCTCGGAGGCTGTGCTCGCCGCGAGAGAGGCC

ATCGAGAACGCTTCCCGTCGTGAGAACGAGGCTGCGGTGGCATCGCGCGAGCAGATCGAG

GCGCTGGAGATGGAGAGAGAGGAACTCCTTGAGCAGGAGGCGAAGCTACGGGCACTGAAT

GACGATGCGGAAGCTATATTGGCCGAGAACGAGGCTCGCCTGTCTTCCATTGAGGATGGG

GGTGCGGAGACCGCGCTCGCTTCGGAagccgccgcggcggcgtcgagcgcggAGATCGAA

GTCGCCAGGAAGGCGGTCGAGGCCTCGCAAGCCGAGGTCGCCGAGCGTGACCTTGCCCTC

GCGGAGATGCAGGCGCGGGTTGAGGGGCTGGAGGGGGGTCTCGCCGGCGCGAAAGCGGAG

CAGGGGCGACTGGACGTTCAGgttagggaggagaaagagcgGGCTGACGCGTCGCTTGCT

GAAGTTCTGGCCGGCCAGCGGCGGGCGGAAGATGCGCTTAGCGTTGCCTGCCAGTCGCAG

CTTGAGTTGAGGTGCCGTCTGGCGGATGCCATgctggaggtggaggaggcggaggctgcG

CGTGCTGACATGGCTGCGGCGACCAGCGCCGCCAAGTTCCAGGAAGGGGAGGAAATTGCC

GTGCTTGGGGCTCGGGTGGCTGAGGTTGAGTCGCTCCTGGAGGCCGCCAAGAGCGCGGAA

GCTCTGGCGTCGAGCGCGGCGGAGCAGGCGAatgtcgctgccgctgcggccgctCAGGAG

GCTGACGAGGCGAAGCAGCAGCTCGCTGCGGGCCAGGCCGCCGACGGAGTGAAGGATGCC

AAGATTGCAGAGCTCGCCGATGTCATCGAACGCCTTGAAGCGGACGGAGAAACGCGGTCG

GGGGTGGAGCAGGAGTTCGACGTTGCGCGCCAGCAGATGGACGCCCTCCGCGCggagaag

caggaaggggatgcgGCGGTGGTCCAGGCGCAGGCAGACAAGGAATATTTGGTGGCCCAA

GTGCAGGAGCTCACGGAGAAGCTCGCTGCTGGCGTTGCCGAGGCGGAAGCCGCGCGGGAC

GAGCTTGCGGCGGCGACTGCGAAGGCCTCGGAAGCGGACGCGAACGCGTCCCAAGAGATA

GCCCGCCTTCGTGAAGCAGCGGATGTTTCTTCCGAAGAGGTACCGGCTGTTCAGGCGCAG

CTTGAGGCGGCGAAGGCGGAGGCCGTGTCGCTCGCTGAGGCAGTGGAGGCGCTGGAGGCT

CAGCTGTTGTCCGCCGAAGTTGCCGCGTCAGAGAGGGTGTCTGCGAACGCTGCTCGCGTC

GCTGACGTCGAGGGTCAGCTGAAGGCGGCGGTGGAGTCTTACGAGGGCAGGTTGGCGTCC

GTCCAGGAGGCGGCGGTGGAGAAGGAGAGTGCGGATGCTGTTCGCATTGCTGAGGTGGAG

GGCCAGCTGAAGATGGCCGTGGAGACTGCTGCGACGGAGAGGGATGCATTGGTTGCTGCT

CGTGCCGCTGAGACCGAGAGCCAGATTGAAGCGGCGGCGGGATCTCTCGAGGCCCAGCTG

GCGTCTGCACGGGCGGCCGCGTCCGAGAGGGAGGCTGCGGACGCTGCTCGCATCGCTGAG

CTGAAGGGTCAGATGGAAGCGGCGGCCGAGTCGTTCGAGGCCCGGCTTGCGGCTGCGGAG

ACTGCCGCATCGGAGAAGGAGGCTGCGCATGCTGCTCGCATCTCCGAGATTGACGCGCAG

ACGGAGGCGGCGATCGAGTCGTTCGAAGCCCAGATGGCTGCGTTCCAGAGTGCTACCTCG

GAAGAGCAGGCTGCGGATGCTGCTCACATTGTCGAGATGGAGGCCCAGATGAAAGCGGCG

GTGGAATCTTTCGAGGAGCAGTTGGCGGCTGTGGGGACTGCCGCGTCGGAGAAGCAGGCC

GCGGACGCTACTCGCATTGCGGAGGTGGAAGGCCAGATGAAGGAAGCGGTGGAGTCTTTG

GAGGCTCGGCTAGTGGCAGCAGAGAGTGCTGCATCTGGGACAGAGGCAGTTGGTGCCGCT

CGTATCGCTGAGCTGGAAGCTCAGATTGAGACGGCCGCTGCTTCTTTCAGAGAGGAGTTG

GTAGTGGCAGAGGCTTCTGCTTCGGAGAAGGAGGCTGCGGGTGCTGCTCGTGTCGCCGAC

ATTGAGACCGAGATGGCTGGAGTGGTTGAGTCGCTGGAAGCCAAGTTGGCGGCGGCACAT

ACTGCCGCTTCGGAGAAAGAAGCTGCCGATGCTGCTCGCATCGCCGAGATTGAGAGCGAG

ATGCAAGCGGCAACCGAGTCTTTCGAGGCTAGACTGGCGTCCTTCCAAGGTGCTATCTCT

GAGAGGGAATCGTCGGACTCTGCTTCCATCGCTGAGGTGGAGAGCCAGATGAAGGCGGCG

GCAGCTTCTTTCGAAGAACAGTTGGCGGCTGCTGGGACTGCCGCGTCGGAGAGGGAGGCC

GCGGATGCTGCTCGCATTGCGGAGCTGGAAGGCCAGATGAAGGAGGCGGTGAGGTCTTTC

GAGGCCCGGCTGGCATTCGCTGAGAGCGCTGCGTCCGAGAGAACGGCTGCAGATGCTGCT

CGAGTTGGTGAGGTGGAGAGTCAGATGAAAGCGGCGGCGGAGTCGTTCGAAAGCCAGTTG

GCGGCTGCGGCCACCGTCGCCTCGGAGAGGGAAGCCGCGGACGCTGCTCGCATCGCCGAG

ATCGAGGGAGAAATGAAGGCCGTGGCTGATCAGCTGCCGGAGGCTTTGGCGTCTGTCGAG

AAGCTCACCTCGGAAGTAAACGATTTGGCCGTGAAGGCGGCGCTGGTGGAAGGGCTTACG

GCGGAACTCGAGGCCGCGCGCGAGGAGGCTGCTAGCACGGCCGCCGCCCGCCACACCGAG

GAAGGTACCGCTCGCGCCGAGCTCGAACGCGCGCTCGCTTCTGTGGCGGCGAAAGAGGAG

GAAATCGCCGCTCTGCGGGAGCAGGAGTCCGAGCAGGTGCGCGAGGCGCTATTGGGTGCC

GCTGAGTCGGCCGCGGCTTTGGCGCAGAACGAGACGAAAATCGCGGAGATGAAGCTGAAG

ATGGAGGCGAATGAGTCGGTCGAGAACGAGCTGACGGAGGCCCTCGAATACGCGGAAAGT

CTGTTGAAGGAGGGGCAGGAACTGACAGAGCAGATCTTGGAGAAGGATGCCAAGATTTCG

GTGCAGGAGAGCACGATCGCGGAACTGCGGGAgcagATTGAGGCCGGAAAAGCTGAGCTA

GCGGCGCTGCAGGCCTCGGCGCCATCCCCTTCCCGTTCAACCTGGCAGGCCATAACCGAC

GGCCTGGACGGAGAAGCCGAGGACGGCTCGGAGGTGCGACGGCTCCGGGTGGAGCTCGAC

AAGAAGGGCAGCGAGATGGACACTCTCCTCAAGGAAGCCGCCGCCGTGCGCGCGGAACTC

AAGGAGATGAAGGGTATGTTCAACGCTGCcgaggctggggctggggcggcGGGCGCCGAC

GAGGACTCGCTCCGCAAGCGCCTGTCCCAGCTGATGTGCGAGAATGGGCTGCTGAAGGGC

GAGAAGGATAGGCTCGAGGCCCTTgccgcgtcggcggcggcggagtcCAAGTCGAGGGCT

TCGCGCTCGGCGGTCGAGATCGAGGGTCTCGAGCGCGAGGTCGGGGGGCTGCAGGAGCGC

GCCGAGAAGGCTTCGCGTGGTGCTGCCGAAGGGGACCGGGCAAAGGCGGCGGTCGGGGCG

ATGGACGCCAAGCTTTCGGAGCTTCGTCAGGAGCGTACCGACCTCCAGAATGCTGCCCAG

AGGTCCCTCGATCGCTGCTCGGAGGCCGAAGCGGAGGTCGCTACCCTGCGGGCCCGCCTT

GCCGAGGCCGAAGAGCGCAGCGCGAACCGCGGTGCGATCGTCGCGCTCGAGACGAAGGTG

GACGCGCTCGAGGAAGAGGTGCGCCGGGCCGGCGCGTCCAGGGTGGAAATGGAGGGAGAC

GCAAGGGAGAGCTTGAACAGGGCGAAGCGGCTGCAGGGAGAGGTGGACATGATGAGGGCC

GAGTACGAGATCGTTTCGGAGGAGGCTTCGGGTCTGCGCGGGAGCCTCGCCGGTGCCGAG

GCAAagctcgaggaggcggaggcgcaAGCGGAGGCACTGGCGGCGTCGAACCGTTCTTCG

GACCGTTCCGAGCAGCTGGAAGCCGACATCGCCCGGCTGCTCGAAGAGCGTGCCGAGCTC

CAAGCCGCCCAGCAAGAGACCCTCCGCTCCGCGGAGCAGGCGCGCACCCAGCTGCAAGAG

GAGCACGACGAAGCTGTTCGCGCGCTCGAGGCGGCAATATCGGAGGCTATGGACGCCGTC

GAGATGAAGGAGGCGGAGTTGGCGGAGCTGTCCCAGAAGCGCGATGAGCTGTGCGCGGAG

GTTAGGCGTGCCGGCGACGAGGCTAAGGTGGCGGCGGACAGGCTGGCCGCGGCGGAGGGA

TGTGTGAGGGAGTTGGAGCAGAGGCTTACGGAGGCAGATGAGGAGAGCAAGGACACCGAG

GGCCAGCTCCGCCACCGCCTTAACGCTCAGGTGGCGGAGACCGAAAACCTCCGGCAGGAG

CTGGTGTCCTCCCGCGACCGCTCGGagcaggcggaggaggcggtgcgCGCGTCCGAGCAG

CGCGCCAAAGAGGGCGCCGACAAGAAGACCAAAGGCCTCCTGGCGGAAATGGAAACCCTT

TGCCAAGAAAAGCTGCTCGCGGAGAACAAGGCTGATGAGATGGAGGCGGAGCTTGATCGA

CTGCGGGGGGAAGTGGCCGGTGCGGAGCAGAGGGCGGCGCGGGACCAGAGCATGATCATC

CGTGCATCGGAAGAGCGCATGACGGTGTTCGAGGAGgCTGCTGCGGCTAAAGACAGCGCC

CTCGCCGACGTCCGGGCTGCCCTGAAGACGGCTCGTGGCGCAGAgaaggcggcgacggca

gctGTGGCGGAGGTGACGGAGCAGgccaatgctgccgccgccgaggtCGCCGAGCTCTCG

AGGCAGATCGTGGAGGGCAAGAAGGCCATCGCCCAGAAAGACTCGCGCATTAAGCGACTG

GAAGACGTTCGCCTCACCAACCACCAGGTGGAGAAGCTTCAGGCGATGAAAACCTCGGCC

CGCAAGACCGCCGCCGAGAACAGGGAGTTGAAGCAGAGGTTGGCGGTGCTTGAGGAGGGG

CTGACTCAGACGGGCGGTGGttccggtagcggtagcggatCATCGTCGGGTGCGGGGCAA

GCCGCGGCTGACCTAGCGGCCGCCACCGAACGCATTGCGGAGCTGCAGGGGGCGAAGAAC

AACCTCATGGTGAAGCTGCGCGAGTACGGGAAGCGCGTGCACGAGCTGGAGAAGGAGCAC

ACGCGGGTGCGCGCGGCGGTGGAGCAGAAGGGCGTGTTGGCGCCGGAGGGGCGCGACCTC

GGCGACGCAGTGCTCGAGATGGCGGAGCGCGTCGCCGGGTCGGATGCGTCCATGATGAGC

AGCTTCTCGGCCGGCGAGGGGTCTGCgatcgccgcggccgccgccgccgccgatgctgcG

GAGAGGCACCACGCTGAGCTCCAGGAGATGAGCGCGGCTCTGCGCAAGGGAGAGGAGGAT

CGATCGGCCCTGAAGGAGCAGATGATCGCTGGAGTGGGCAGGTTCAGAGAGCTGGAGGCG

AGAGAGGCCCGCGCGAGGGAAGCGTTGGAGGCTGCGGAGTTGGAGAAGATGGAGGCCGTG

AATGCGGCGGTGAAGGCCAAGGAGAAGGACCACGAGCGTCAGATTAAGTTCCTTCAGGAG

GAGAACGTGACTCTGTTCCAAGAGAACACGGACGTGAGAACCAAGGCCGACACTCACAAG

GCCGAGCTGGATGCCATCAAGGAGAAGCTGTCCACCGTGAAGGCCACGCCTGCGCCCGCG

TCTGCGACCAAAAAGCGGCCTTGCGCTGACAGGACCAATGTCCCCATCGAGGCCGCGCCC

GTCGGCAAGCCCGCCGCCGCGGGTcaacccaccgccgccgccgccagacgCGCGCGTTCC

CGCGCTCTGCTCGAGAGCCCCAGCATTGTTGACGAGGAAGGCGCCGGGGAGTGCACTCAG

TCGTAG

>g7186.t1

ATGTCCGATTCAGTGCCGACAAAGACGGCCATTACGGCAGGCATCAGCCTCGCCGTAGCT

GGCCTTGCGGTCGGGCTTGCGGGTCCCAAGTTGGCCGAGAAGATCATCAGCAAGATGAAG

CCTGGGGGCAAGTTCACTGGGATGAATAAGCCGACAGCAGGCGCGCGGACGGTGAAGGAG

CTGCAGCGCGGCGACCACCCCATCCAGCTCTACTCCTTGGCGACACCGAACGGACAGAAG

GTCACCGTGGCGCTCGAGGAGATGGGGCTTAAATACGATGCCTGGTACACCGACATCATG

GCGGGGGACCAATTCACCTCAGGATTTGTCGAAATCAACCCCAACTCCAAGATCCCGGCT

CTAGTGCACCGTGACGCTCCAGGTGGCAAGCCTCTTCATCTGTTCGAGACAGGCAACATC

CTGCTGTACCTTGCCGAGCTCACCGGCAAGTTCCTCCCGACCGACCCTGCCAAGAAGGCT

GAGTGCCTCAACTGGCTCTTCTTCAACTTCGGTCCTACTGTCACGTCGGACAGCGCCGTA

TCGGTGAAGATCGACTACGCTGTGACCAGGTACTCCACCGAAGTACAGCGCGCGATGGAC

GTGCTTGACAAGCAGCTGGAGAGTAAAAGCTACCTCTGCGGGGAGGAGTGCACTATCGCC

GACATGGCTTGGCTCCCATGGGTCCGGTACTCCACCGAAGTACAGCGCGCGATGGACGTG

CTTGACAAGCAGCTGGAGAGTAAAAGCTACCTCTGCGGGGAGGAGTGCACTATCGCCGAC

ATGGCTTGGCTCCCATGGGTCCGgtGCCTGGACGTGTTCTACAAGGCGTACGAGACGCTT

GAGCTGGCGAGCTACAAGAACGTCGCGGCCTGGCGGGAAAGGATGGAGGCGCGGCCTGCG

ACAGCCAAGGGCCTGAAAATCAACTCCGACGCCGACGGGGGAATCTACCGAAACTACAGC

TCCTAA

>g7760.t1

ATGtccgacagcagcggcagcaccgctTCTGACAGCTCCGAGCCGGGCAGCCCATTCGAG

GCAGCGTTGATGGGCGTGGTGCTTCTCGTCATGTTCGGCTGCCTTTTCACCGGGTGGAAG

ACGCCGGAGACTGTGgtgttccTATGCCTGGTGCTCGTCTGGAACGTCGGCCTGGTCGGC

ACCACGGAGGCATTATCGGGTTTCAGCAACGCGGGGATGCTGGCGGTGGGCGCCCTCTTC

GTGGTCATCAAGGGCGTAGAAAAGTCCCAGCTGGCGGACAAGGCGGCGCGCCAGGTCTTC

GGCCTGAGGACATCTCTCTcggccggcctcgcccggatgaTGTGCCTCTGCTTCGTGCTG

TCGGCGTTCCTGAACAACACCCCCGTGGTGGCCCTGCTCATCCCCATCACCAGGGACTGG

GCGCGCGCGAGAGGCTTCTCCCCCTCCACCTTTCTCATCCCGCTGTCGTACTCGTGCATC

ATGGGCGGCCTCCTCACCGTCATCGGGACTTCGACCAACCTGGTGGTGCAGGGGCTGGTG

CTGGTCGAGGCGAAGACCGACCCCAGCATCGAGAGCATCGGGTTCTTCGAGCCCGCGTAC

GTAGGCGTTCCCCTCGGGGTGATTGGGATGGCGTACCTCGTGGTGTTCGCCCCTAGAGTC

CTGCCGTCCTGCGGCGGACTCTTTCGGTACGTCCGGGACCGCGCGAAAGAGCTGCTGACC

GAGGTCCAAGTGATGGACGATTTTCCGTACATCGGCGAGCCGGCGGGGCTCGTGCTGGTC

AGGCTGGGCCTTCCCCAGGACACCCTCATCAAGATTCGACGGAGAATTCCGTCCGGCATC

CTGCGCCAGATGAGCGCAAGTCTCGAGGGAAACGCGAGCCTGCTCCTGCCTCCCGGGGCG

CCACcgacaggcggcggcggcggtaccggTATCGATGCCGACGCCCTTATGGAGGCCAAC

CAGAGGACGCTCAGGGCGGAGGGGATGTACAGCTACCGCCAGAGAGCCGAGTGCCTGTGG

GGCACGACGGAGGACATGGATTCCGCGGCGGAAGAGAAGAGCCTCACCCcggccccccgc

gccccgcgccGCTCGGGAAGCTTCATCCGGAGAAAGCGCTGGGCGAGCGACGCCGACGCG

ACTGACCTCGCGACGGCCAAAAATAGGGGCCGAGCCGCGACCGTGGGGAGCGCCGCGGTA

GTGCCTGAAAAATGTATCGCCTATGCTAACGCTAAAGTTGACGCTAACGGCAACGCTACC

GCTAGTGTTAATGGCGCGGGTGGTGATAAGGGCGATGGTATAGACAGCGGCACGGCCAAC

GCCGCCTCGGTAAAAATCTCGGACGGAGAAACCTACACGGACATATTTCCGGTTTCGCCC

GCCGAGCCAGTCCAAGCGGGCGACGTCCTCTTCCTCAGTTGCGCCCAGGCGACCATGATC

GACTTCCAGTCGGTCACGGTCAGCCAGGGGCTCAAGGGGCTAAAGTTTCTGGACGTGAGC

GCGCTCGACCTCCCCGGGCACGGGACGGAGTTCTTCGAGATCGTGCTCTCGGACCACAAC

CACTTCGTGGGCCGCTCTGCCGACCGTGACAATTCGGAGTTCGCCGCTTACTACGGATGC

AGCGTGGTCGCGGTCCGCCGAAGGGGCCGCTCGGGGGCCGTCGcgccctccgccgcctcc

gtgCCGGCTACCGCCTCGCGCGGCTGTGGCGGTGGCGCCGACGCCaccggtggtagtggt

ggcggcggcggtgatggcgaAGGCTGGACGCCCTCTGCCGCGCCGTTTAAAGACTCCTCG

CGGGTAACGGACGCCGCAGAGGTTTTCATAGAAGAAGGACTTTGTGCCGAGCCCGCACTC

GACCCCAGCAGTGGACCCCCGACACCGCTCTCGCTGGCTTCGGGTAAAACGTCGACAAAG

TTGCCGGCGGCGGCTGGGCGGTCCGATGTGACGTCACCGCCGAGAGGGACTCGGTCAGCC

GTGCGCGCGATGCGCTCCCCCCTGCGTTCTTTGCGGAAAGAAGTGGACGCTCAGGACGGG

GACGACGTGACTGGCCTGTCGTTCAAGGCGGGggatgtggtgctggtgctggcgaaGGAG

GAGTTCATGGAGAAGTACGCACCGTCGAAGGATTTTTTTCTGTTGACGAAGGTGGGGTCG

GTGCCGAAGCCCGTGCGGTACTACGACTACCTCCCGCTCCTGGCGTTCTTGGGCATGCTA

TGCTGGGTCCTGTTCGACCAAGGCACCGATATGGTGCAAGCGGCATTTGCGGCCGGTGCC

GTCCTAATTTTGGGGGGCTGGGTGGACGCGAAGAAGACAGTAGGTTACATCGACTGGTCC

CTCCTGCTCTTGATTGGGAGCGCGCTAGGGCTCAGCAAGGGCATCGTCAACAGCGGGCTG

GCCGGCTATGTGGGAAGGGCGATTCGCGACTCGGGGATAAGCCCCGACGCCAGCTTGTTC

GTCCTGTACGCCTTCACAATGACGTGCACGGAGCTGATCACgaacaacgccgccgccgct

cttgcCACCCCCATCGCCTTCAACATCGCGCAAGAGCTAGATGTAAGCTACAAACCTTTC

ATTATAACGGTGATGCTAgcagcctcctcctccttcatCACCCCGATAGGCTACCAGACC

AACACCCTCGTATGGGGCCCCGGAGGCTACCGCTTCACCGACTTCATGAAGATAGGGACC

CCGCTGTCATTGATCTACCTCGTCATCGGCTGCCTCCTGGTGCCTAGAGTGTTCCCCTTT

TGA

>g7804.t1

ATGAGGAGACCGGGTAGCCGCCCGCGGTTCGTACCAGGAGGGCTCGTGCTAGCTCTGGCC

ACGGCGAGGAAGAGCGTAACGCGGGCTTTCAACTTCGCCGGGTGCAACAGTGTCCACGGA

CAGCGGGGCCATGCGGGCAGCGGCAAGCGGTTGTGGGCGGCGGCGCAATCGTCTACTGCA

ACCCCACCGGAATCACCGCTGCCCCCGAAGGACGGTGATCTTGTGTCGTTCTTCTTGCTT

CGGCACGGTCAGACCAACTTCAACGCCATAGGGCGTATCCAGGGAACGTTGGACTCGTCG

ATCCTAACCGAGCAGGGTATCTTGCAAGCCTTGGAGGCCGGGAAGACGCTTGCATCGACG

GTTGACCTTGACCTGGGCCCCACCGTGATAGTGTCGCCCATGGGCAGGGCGCAGCAGACG

CTCGCCTGCGTGCGGGAGGAGCTGTGCAAGGGGGAGAGGGACTTCGAGACGGTCGAGATT

GTTCCCAACATCAGGGAGATCGAGCTGTTCGAGTGGCAAGGAGAGCTGAAGGAAGACATT

ATGAAGAACAGTCCTGAGGAGTACTCGGCGTGGGTGGCGGATCCGTCCAACTTCAACTTT

TCGGGAAGGTACCCGGCGAGAGACCTGTGGGAGAGGGCGGAAAAGGCTTGGGAGGAGATG

CGCTCGCTCGAGTCGATCCAAGGGCAACATCGGACCCTTATCGTCGCTCACAATGCCATC

AACCAAGCTTTGCTCTGGACGGCGCTCGGCTGCGATACGTCGTACTTCAGAAAGCTTTCC

TGGCCCAACTGCGCGGTTTTGGAGTTGCAGTGGAGGAGGGGTGCGGCGCGGGCCGAGCGC

TATCGCTGGGTCATCCCCGAGCCGTCGCCGTTTGTTGAAGCTTCGGACGCGGCTCGTCTG

CTCAAGACGAGCGCGGATGACACGGTTTACACCCTGTGA

>g7887.t1

ATGTTCGCTGCGAGTTCGTCGGGTGGGATGCCACCCACCAACGGCATGGCGGGGAGCCTT

CAGGCGAAAAAAGTAACAGCGTCACCGCGTGAATCGCATTCGTCGTCGGTGAACTTTTCG

ACGACCACGACCGCGAGAACTGGTCCGCCTGCCACGCTAGGTGGCGATGCTGGCATCAAC

GGCGTGACCACGGCTTTCGCTAGTATGGGTTTACCACAAGTGGGAGCATCATCGACCCCG

CAGCCACTTCggtcgctgcagcagcagcagcatcaacagcaggaCGAGGAGCAgaggcga

cggcagcagcagaaagagcagcgtcggcagcagatgcagcagacacaggagaagaagcag

cagaagcagcagaagaggcaggagaagcagcagcagaagcagcagcagaggcagcaggag

cagcagcagcagaagcagctccagcaacagctgcaggagaagcaggagcagcagcaacag

ctgcagcaacagctgcagcagctgcagcaccagctgttgcagcagaagcagcagctgctg

cggcagaagcaggagctggagcaggagcagcaggagcaggagcagcagcggcagcagcag

cagcagccacagcagcagcaacaggcacagcagcacaagcacaagcagcagaagcaacgg

gAGACACAGTCGCGGACGCCGTTCAGCGCGAAACCGATTGACCTCCGCCTCCAACTACAG

ACGTCTAAGTTGGCCTTTATCACAAGCAAGCGGAACGGAGACCTTCTCCGAGCGGGTTTC

ACGCTGAGGCGGGAGACCGGCCACGGCACCGTCCAAGAGTTTTTGGACAAGACCCGGATC

GACCGCAAGCCATACGGCGGGCGCGGCTTATTCGGCTTCCCCCCTACCCGCGGGAGTTCA

GTAGGCAGTAGCGTTTTCCGCGCTTGGACTGGCAAAGACAGTCTAGAGAACTCAAGCGGC

TCGACCAACGCAGACGACATCGTCGAGGAGGAAGACGACAGCgagcgcggcggcggtggc

ggcgctgcCAGAACCAGAAGCGCGGATCAGCTTGACGCCGACTACGAAGAATGGCGAGAC

ATGACGTTCGCGTTTAACGAGGTGATGGGGTGGGTGTTCCTGCACCACGCGACGGAGGCC

GGGGTCGAGCTGGTCCACGAGCTCATAAGGACAACCCACGGCCAAGAGATGGGCATTTTT

TCGTCAGACTCGAGGGCCACCGATACGGTCGAAGGCTTTGCGTCGGCGCTGGAATCGTCC

AGGGATGTCAGCGTGATCATTAAGGGGGCCATCGTGCACATTTGCGGGACCTTGCAACAG

GTCCAAAACGTTTCCAAGGCCATCGAAGCGATCGCTAACGGCACTAGTCGGATGTACGCT

ATGGCCGATTGGTACTGGAGAGGAGAACCGGAGAGTGCCGGGCGCTGCCCGTGGTATCCC

ATGTCGAGCGAAGCAGATGCCGCTCTTGAGGCAGCTTACAACTCATACAAGGAGGGCGGC

AGCTTCGGTTCCGCCTTGCAATGCTACGAAGTTGGAGGCCTTTCGTACGCGGCAAACTTC

AAGGTCATGGCGCAGATCAACAAGATCACGGGGCACGTTCGCCCAATCAAGCGGATGGTC

ACCGACCGGCAAGGCTGCCCCCTGCCCCGATGCCCGAAGGAAAACATGCCCATTACATGC

TTTCGCGAGGGCACCAACTACAGTCTCGTCACCGTCACCAAGGAAGAGGACATAATGGTC

AGAGAAGCCGTGGAATTCACGTACACGGACAGCCCGGTGCTTCGGGTGGAGCACGTCTGG

AACTCTTTCTTGTGGGGACACTACGCGCAGAACGCCAAGTCTGTGGGGAACGAGTGCCTG

ATGTTTCACGGCGCTCCGGCGGATTCGCTGAACCAGATATGCAGGACGGGTTTCGACCGG

AGGCTGGCGGGATCGAAGCACGGAGCGATACTACTGGCGAAGGTCCTTGTCGGCGAGTTC

ACGGTCGGGAACAGCACCATGGTAAAGCCGCCGAAGATGTctggccccgctggaggcgac

CGGGAGTACGACACCACCTCCGacgacccccactccccgcAACTCATCGTGAGTCGACCG

ACCGCGCTGCTGTACGCGCAGGCGCCTGCGTTGTTGTACCAGCAATCAATCGCCTACGTA

CACCTTTACTTGTAG

>g8090.t1

ATGTTCGTGAGCGTAATTACCCCCCGGTCCTTGCATGTTCGGCTATCCGACGTCCTCCCC

TCCGCAGATGTCGGGTACATCGTGTGGGACACTGTCGATTCGCGGAAACACCCCGCGGCA

AGTATCGAGCTGCAGCAAAGTCCCTTTAAGTTCCCTGATCTGGCGGTCTGCGCGACTTTC

CCCAGCGGATGTATGTCAGAGAGCACAAACTGCCTTGAGGGCCAGGATTTTCTTCTGTGG

AGTCAGAAGGGCTTCCTGGAAGACCACGACGATTTTGATGTGGCAAACTATCCGGACGTG

GCGAGAGTCAGGGAGACGTTCCCCCATTGCGCCGTGATGCCGCTATCGCAGCTGACTATA

AACGAGACGGCGGTGAACAACGGCGAAATCAAGGAGTTCAGCGCAAGGATTATCCTAGCT

TGGGATGAGGACCCGGACGGGGGTATCGACATCACGAGAAGCTTCATCTTCGAAGAGTAC

GTCAGTGTTCATTTTATTGACATCGCGCAACATGTGGACGATATTGATGGGACGGTCACT

AGTGCCAAGATCCCCTACGAGATAGCCACTCCCACAACGAACGCGTGGTTCACTTCAACC

AGCAACCATATGGTGATAAGCTTGTCCGAGTACTCCGGGATCACGAAGAACGGCGCCAGA

AAGGAGCGTGAGCAGACTTATTCGCAGACCACGACAACCGGAAAGGAACGCTGGTACTGG

ACGGGTGATTCGTATACGTCCCCGATCGCTCTCCTGCAACTTGAGGTCGCCATCACTAAG

TTCGAGTACACCAGAGTCAAGGACGTCGATCCTGTCGACGTGTGGGCGATTATCGGCGCC

ATTGGAGGCGTTTGGCagttcgTCGTGGTGGCGTTCggggtgttcttcatatttTCGGAG

AAGCAGCCACCGGACAAGAAGATGCGCGACTTCAACGACACGTTTGTCACGCCAGCCGCC

AAGATCAACAAACGCTTGTCCAGCATCACCGCCACCTCATTAAGGCCATCACAACAGGAC

ATTGAAATTGACGCAGGAGACGAGGACCTGCCGCCCGGGTGGGTAAAGAAGCAGCGCAAA

TCCGGTAGCGTGTACTACTTCAACACGATGACAGGCTCCACAAGGGCCACCCCCCCCAAC

GGAAGCGGAGAACCAACCGAAAGCGCGCCCGCTGCACCCCGACCTTCGAACGGAGCCCGC

ATGCTGTTCCGgGTGGCACAAGACCCCCTAGACTCGGGGACCGGCGGTGTGCAAAGCTCT

ACAAACGGAACGACTGATGGCGGGAGCCTGCCGCCGGGCTGGCAACAGAGGACAAACGAG

GACGGCAGAAGCTATTATGTGGACACGGAGAACCGAACGACGCAGTGGAATCCGCCTTCG

TGGCGGGTTAGTTCCAGCAGCGGCAAGGTTCAGCCCGTCGACGCTCCCTTGAGGCGCGTA

CGCCGGGGCTCGGCCACGGACATACCTCAGCCTACCGCGCCTCCCACCATCATTCCTCCG

ATCACCACGTCCGCAACGGGGGGCATCGTTAACGAGCCCCTGCCTCCGAACTGGGAAACT

CGCACCACTGGAAATGGGAAGACATGTGGCACAAGACCCCCTAGACTCGGGGACCGGCGG

TGTGCAAAGCTCTACAAACGGAACGATTATGTGGACACGGAGAACCGAACGACGCAGTGG

AATCCGCCTTCGTGGCGGGTTAGTTCCAGCAGCGGCAAGGTTCAGCCCGTCGACGCTCCC

TTGAGGCGCGTACGCCGGGGCTCGGCCACGGACATACCTCAGCCTACCGCGCCTCCCACC

ATCATTCCTCCGATCACCACGTCCGCAACGGGGGGCATCGTTAACGAGCCCCTGCCTCCG

AACTGGGAAACTCGCACCACTGGAAATGGGAAGACATACTACGCCAACACCGTTACAAAG

AAGACCCAGGCGTCGGAGGCGCTGCCTCCGCCGGGGCGTCGTCTCCCTCGTCCGCTGTTG

CACCCGCTCCAGACGCTCGAGTTCTCCGGTTGGGCCGACGCTAGCGTTTCCGGCCGTGGG

ACGAGTCCTGTCGCGGCTGCGCTGTCGCGCTCGGAGAGGTGTTAA

>g8117.t1

ATGATGGCCCGCTGCCTCGTATCTCAGGTGGTGGTTCTATTGCTCACCAGCTGGAAGTCA

GCCGCCACGCCATGCAACCTGTCACAAATTACCGTGCCGACAAGAGTGGAAGACACGGAA

AATGCCTATCGGCTGGCGGAGGCCGTGGACTGCTCGGGTGGGTGGTTTGACGTGGAGTGG

GTCGGCTCTGTCAAGATCGAAACAACTATCCGTGTGCTCGACGGTACCACCCTAAGCGTT

ACTGGTGCGTCAGATGGATCTTCTTTTATCGACGGTTCCAATGAGACATCATTGTTTGAC

GTCATCGGCGGGAACCTGCACTTGTCGAAACTCACGATGGTGAACGGCACTGCTGAGTTT

GGCGGCTCGATCAATGCGACAGACTCTGTTGTGACGACGTCCAGCTGCACCTTCGTGGGC

AACGAGGCGGACTACGGTGGGGCCGTGCATCTCTTCGACTCCACGTTCGAGGCTATGGAT

ACCACATTCTTCCGCAATTTTGCCTCATTCGACGGCGGAGCGATTGTGGCGATATACTCG

AACGTAGCTGTGGGCCAGGGTGGGCGTTTCGAGGACAATGGCGCGTACTATGGAGGTGCT

ATCTACTCGGAGGGCTCTTCGATACACGTAACCGATTATGCATCCTTCTTGGGCAATGCT

GCTTATGTTGGCGGAGGCGCGTACCTCTGGGCCTCCGAGTTCGAGGCAAACAACACGACA

TACACGGACAACAATGCTACAGGCTATGGCGGTGCGATCTGCGCGGGAAATTCGAACGTA

ACGATTGGAGATGGCGCGCGATTTCTGAATAACAGCGCGACGAAGCAAGGTGGCGCTGTG

CTGGTGTATGAAAATTGTTCTCTACTTCTGTTGGACGCGGTGGAGTTCGAACGAAATATA

GCAACCTTCGGAGGAGCTTTATTCGTGGAGGTTTCCGTGCTGTACATGGACGGCTTTGCC

TCCTTCACGAACAACGTTGCGGTCGACTCTGGCGGAGCTGTGTACGGTGTCCTGGCCGAC

ATCACCATCACTGGGACCGCTTTGTGGGAGTCAAACATGGCTGTTTATGGGGCGGGTATG

CGGCTGTATCACTACTGTACGCTCAACGTCACCGGTGGCGTTACATTCCTCAGCAATGCT

GCATTCCAAGGCGCGGGGATGTGGGCATCAGAACGCTCGAACGTGGCCATATCCGGGTCG

TCTACTTTCGAGTCCAACTTTGCACATGGCCACGGTGGTGCACTGGTTATATATGACTCC

ACGGTGGAATTCACGGCCGGCAGCACTGCCAACTTTAACAACAACAGTGCTGtggaagcc

ggggggggggtcctcctCTTCACAGCGGGTGAAATCACTGTTGCAGGCAGCGCGACGTTT

GCCAACAACAGTGCTGTGCTTGGAGGGGGAATTGCGGCTTCGGAAGGCTCATCCGTGACC

GTCACAGGCAATGTTGTATTGGCGGATAATTTGGCGTCTACGGCTGGTGGGGCTATTTAT

CTCGACGCGCCAACTAAAGTGTATGTCAATGGCACGCGTTTTCGGGGAAACAAAGCGGTG

CAGAGTGGTGGGGCTATGCTAATATTTTCGGCAGGAATACGATGGGATTGGGCGATTGTC

TCACACTGTGTTTTCGAACACAACAGGGCTAGCGACGCCGGTGGGGCATTGTTCATTGGC

GGAGGGTTCGTTTCTATAAACGAATCTCAGTTTATCGCCAACAGTGCCGAACATGCTGGC

GGTGCGGTGATGGTGTCGGGTATCGTAGGGTTTGAAAACTGCGTCCTGGTGGACAATGAG

GCTCGTCTGGGACCAGCAATCTACAACGCTGTAACGGTATCGCTGGAATCCACCGACGTT

TGCGACAATCAACTCCTCTGTGACGACAGCAGTTTTCTTGACTGGAAAAATGGCTCTGCG

TACGATGTCGCCTGCGAAGCCTGCCCTGCGGAGGGGAACAACTGCACTATGCCATATTAC

GACAGGGTTCAGCTTTGCGAGTCCGTCCTTGAGCACACCACTAGCCTTGCTGCGGATGGG

ACGCTGGAAACGCTTGATCTAGAGCCGGGATACTGGCGGAGCTCTAACACGAGCAGAGAC

ATCCGAGAGTGTTATGAGGCCGACGCGTGCATTGGGGGGGTGCAGGGTGTATGCGCATCT

GGGTACGAGGGGCCCTTTTGTGCTGTGTGTGCGGACGGACATACGGCTGGACTAGGGTTT

ACTTGTATTGAATGCAGCGGGGTGCGCCGAAAGGCGACAGTAGCCGTGGGGATTGTTCTC

CTAGCTGCGGCTGCCATCATGGTGGCGGTCAGCATCAGTTTCCTGGGTTCTCCAGCTGCC

GAACCGACAACAGGGCTGCACGCCTCTTGCAGAAGGTTTCGCAAAGCAATATCGCGTCCG

AAATGCGCAAAGGCTACCCAGGCTCTCAAGATCGTCATCGTGTCATGGCAAATCGTCACC

CAGTTCGCAACTGTCGCGAGCATCACGTACCCCGCAGTGTATGCGGCTTTCCTCAGGTAT

GTCGACGTGTTCAACCTGAATGTTGCCTGGATGCTTTCGGCGGGGTGCCTGATTAAAAGC

AATTTTTACCACAGCCTCATCGTGTCCACCATAGGACCCTTAGCGATTGCGGCCTGTGTG

CTGGTTTCGCACTCAGTCAGCTGCCGAAGATGTCCTGCACACGACAGAGAAGCACGCGCC

AAAATCGATCATCGGCACGCATCGGTTCTGTTTTGGATGTCGTTGCTGGTCTACGCTTCA

GTATCTTCTTCTGTTTTTCACACGTTTGCCTGCGACGAACTCGACACCGGGAGCTCCTTT

CTCCGCTCTGATCACAGTTTACAGTGCTACACCACAACGCACAAGATTTTCATGGCTTAC

GCTGGCGTAATGGTCGCGGTGTTTCCGCTCGGAATCCCTTTATGCTACACTCTTGTTCTC

TATCGGAATCGAGACTATTTGAAGTTCGCGGTTGACTCTGAGACCATCACGACTGACGTA

GCAAGTTTTAGAGAATTGTGGGAGCCCTACCGACCGCAGGTGTACTTTTACGAGGTCGTG

GAGTGTCTACGCCGCGTGGTGCTCTCCGGGGTTGTTGTGTTTATTTTTCCCAATACAGCT

GGACAAATCGCCACGATCCTTTTGCTAGCAATGTTTACGGCCGCTGTGTTCATGGCCCTG

GACCCTTACGTTAGCCGCTGGCATACGTTGCTGGCCAGAATGAGCCATGCAGTCGTTATT

TTGAGTCTGTATGTGGCGCTGCTGCAAAAGATTGACGATGTGAATGAGGACAAGGCAAGC

CAGCACGTGTTCGCGTGTGTCCTTGTAGCGACGAACTTCGGTATGATTTTGGCAGTCGGG

GTTGAGGCCTATGGCATGTTTTCGGTCGCTGTGTTAGAGGTGCCCGAGTCTACAGCAAGC

TCCGCTCCCAATAGGACCGGGACACACGTGGAAAACTCGTGTGAATCTTCCACGCGAGTT

GTACCTTTGACACAGTCCCAGCCCACCAACACAATATGCGTGGGAACGGCGTAG

>g8320.t1

ATGCGGCGGCTACATACCGCTATCCAGCGGTACGCTTGGGGGAAGGGTGGGTCCGACAGC

ATGGTGGCGCAGCTCAAAAAGACGGAAGACGCAGATTTCAACGTCGATGAAGAGGAAACC

TACGCAGAGCTTTGGATGGGAACGCATCCGAACGGCCCCAGCCGAGTTATGCGGGATGGG

AACGAGGGGGCACACGAGTTACATGGGCTGTATGGTTCTCCGGAGGGACCGGGGATGTTT

TTGATTGAGCTACTGGAAACCTACCCGCACTACCTGGGGGATCAGGAGAATGTCGGAGAC

TTGCCATTTATGTTCAAGGTCCTGTCCATTAATAAGGCGCTCAGCATTCAGGCGCACCCC

GACAAAAAGCTGGCTGAGCGTCTTTACGCCACAAGGCCCGACCTCTACAAGGACGACAAC

CACAAGCCAGAGATGGCCGTAGCGCTGTCGGATTTCGAGGGCCTCTGTGGCTTCCGGCCA

TTCCGGGAGATGAGTGAGTCAAAAGCCATAGACGCCCACCACGTAATAATTATTTCGGCC

ATgtgcgTTAACAACCTCGAGGCATTCCCTGAGCTCCGAGCTGCGATCGGGGTCGAAGCC

ACTGCTCAGGCGTTATCGTGTACATCAGGCGTCAAGTCGGACGAGGAAGAAGCCCTTCGG

TCTTTGTTCCGAGCGTTCGTTACCGCCAACACGGACGTCGTTACCGCCCAGGTCGCTGCT

TTGGTGGAGCGGCTGGAGTTTGAGCGCTTGCCGGTCTCGGAAGTGTCCGGCGATCAAAGC

CTTTGCGGCAGCGGCGAGGAGCACAGGCACTCCCCGGACTCGTGCGGAGACGACAGCAGT

TACGACGGCTTCAACTACGCTGGTCAACGGAACGGCAGCCTGCCGGGCGTCTGTATCACT

GACCACATCCTCGGAAGAAGAAACAGCCGAAAGCAACGCCATACCGACCGCACGATCAGC

GAAGGCGGCGACTGCGGCGACGTTGCGGTGCTGGAAATTCACCGGGAAGAGCTGAGCGAG

ATCGACAGGAAGATAACCTTGGCGGACGGCGGCAGCGATGATACGGTGGATGGAGTGACG

GACCAGGAAGACCTGCACACTGAAGACGACTGCTCTCGCCCCCAAAGCACTAGCGGCAGT

AGTTGCCACGGGAGTGTCAAGGGCAGTGCTCTATgggacaaacagcagcagcaacagcag

cagcagcagcagcctgttgTGGGGAAGCAAAATTTGGGAACGGCCTGTGTGCCAAGGGGC

CGAGGAGCAGCCTCTAGGCTTTTGCGTCGAGGGTGGTCTTCCTCGGCGAAGTCGTTTGTC

GACATCAAGGGGGACCGCAGCGCTAGAGAGGACCACGCGCTGAGGGAATTGATCTTGCGG

CTGTCGGAGGAGTACCCCGGGGACATCGGAATCATGATGCCTCTGCTTCTTAACTACCTC

AGAATGGGGAAGGGGGAGTCTTTCTTCATGGCTGCTAACGAGCCCCACGCGTACCTCAAG

GGAGACATTTTAGAGGTGATGGCTCGGTCGGACAACGTGGTGCGTGTCGCGCTGACTCCG

AAGCACCGCGACGTGCCGCTGTTGTGCGAGATTCTTACGTATCGCATGGGGGCCCCTCCC

ATTGTGCAGCCAGTCGTCGTGGACGATTTCTGCTGCAGGTATACGCCCCCAATCAAGGAC

TTCGAGATGCTCGACATAGAGATCCCACCCCACCAAGAGTACTCCACCGAGCCGGCATCG

GTGGCGGCGTTAGCCATAGTGCTCAACGGTACCGGCTTCGCTTGCGATGGAACCAGCCGC

CAGAAACTCTGTGGCGGGGTTGCCTTCTTTGTCCCAGCAAGAACACCGATTCTTTACACC

TCCACGGGGACCGAAGGCCTGTGGCTCTGCTTGGCAAGGTCTAACCTCAACCATGGGAGC

GGCGAGGAGAGCTAG

>g8334.t1

ATGCGTACATCGTCACCTTTCCTTCTCTCCTCCGCCCCGTGCTCTTTGCCCTTTCGACTA

CACTTGTTGCACATACTTGACATTGATCAGGTGGAAGGTTTCTGGGAAGACGCTTTTACG

GGGATATGCCATTTTCGTGCCCGGTGGCTGCTGCGCGCGTCGGAAGTCCCAGGAGACGTC

GCCGAAGACGAGGTGGTGCTCACTACAGTATCAGACGCCGTCGACGCCACATGCATTTTG

GGACTAGCCCCGATAGGCTTACCCACCCCCGATCTCCAGACGGAGCCGCTCGCGCCCGTC

GAAGCTGAAGCGCCCGCTTCTGATCGAGTGCTGCGAGCTAGGATAAGCAAAGGGAAGGGC

CGGAAGAGGGCGAAGAACGCCATCCTGCTCCTGTCGAGAAGTTTTGATCCCTTCGCGGGT

GTCTTTGGCGAACTGCCCGCCGAACACCCCGCGTTGGTGCAACTCCGCAAGCGAGAGGGG

GCCAAGACTGCCCATGGCAACACCGCGCGGGAGTCCGAGAAAGGAGCTAAAGGGGGGCAT

CAGCGACGCGGCCCGGGACGACCGAGGAAGGTCATTACGCCCCGCTTTAGCGTGCGCGGC

TCTCCCCGAAAGTCGTCGGCTGTTGACAGGGCCGGCGCCGAGCGCAAGGGGCTTCATGGT

GCAAGGTTGGCGGTCAGGCCGAGCCGAGCTTCTTCCAGGGAACGCAGCGAAAGcctcagc

agcaacggcagcagcagcagcagtggcagcagcatcatcgttggAGATAACAGCGGGAGC

AGCgtcggcagcatcagcagctgcggcagcagcagcaacagcaacagcggtttCACGAGC

GGAGACAGCGAAGGCTACTACGACGAGCTAGACATTTGTACTAACGCTTCCCCGTCGGCA

GGCCGAGGGCGCAGGAAAGAGGCAGTGGTAGCAGCGGGGGCAAAGCCAGACGCGAGGCCG

GCGGCGAAACTGAATACGACACTGGAGGCGAAACCGGAGGAAAATCCGGTGGCGCCCGTG

CCAGCTGTGCAGTATCCGGAACGCCCCCGGCGCCTGGCAATGCCCCCTGGTGTGTTCCCT

GGACCTGCTAGCGAAACTGAATCCGGCAGCGACGTCGGGAAACCGGCAGACGCGGCGAAT

GCTTCCCCCCTGACGGGTCGGATGCCGCTACTGCGCGGAGGGCCCCTAGTACGTGCGGCG

CATCAGGGGGGAGATTTGGGGGATGGTGAGCTCTCTCCCATGGCGGAGTACCGCCACCGC

GAGACAGATGTCGGGGAGGAGCACCAGGCTGACATCCCACCGTTGCTGACGGAGGCCCAG

CGCGCGGCGGAGAAGGAACAGACCAGCGAACAGAAGATGGGCGGCACGCTTGTGTGGAGC

AGTATTCGGGACTGGGACGCTCAAGACAAGGAGCGCCTGGAGGACTACCTGGAGAAGGCA

AGGCATGCCACGCATCTAAGGCGGCTCTTGCCGGGCGTGCCCGCGGTGGTGTTCATCGGT

AACGGCGGCGATAAGCGACGCAAGCGCAAGTCGGCGTGGGCGGTCATGGCCGGAGGAAAG

CCCGAGGACCCGGAGAAGATCTGTGTCGTGTGCGCAGGGAGGGAAGTTCTGGAAGTGTCC

TTGGGCAACGTTCAACCGGTGCAGATTGAGGAACACGCGCTCGAAGCGTTCATGCAACAC

GGCAGTCTCACAGATACCCGGGCGGCGCTCAGTATCCTTCAGTCGCGTGTCGACGACTCC

GGCCCGCTTGCCCCGTGGACAGCCCCTCAGATCAAGGCTGTGTCAGAAGCTCTTGACAGA

CACTACGACTTCGACCGGTACTCTCGATCCAGAGGGCACGACCGGCATCGGGATGAGCAC

ACGGACGTGGCATCTCTGAGCACGTCTGTCCCGGGAAAAACGCCTTCCCAGGTGCTATCC

TTCTACTACAGGTACATGGCATCGGGCGACGCTCTTACGGACATCATGGGTGGTGTCGAG

ACCCAGGTAAAAAAGCGAAGCGAGAGGAAGATCACCCGCCCGAggacccctccacccccg

cgcCCGAGCCCGCCCCCGAACCTCAGGCACCGCTCATCCCTCCGGTGCCCCCCGCGGCTG

GCGAAACATCAAGACTCCGACAGTAGTAACGGCGGCTCGTCTCGGCTTCCTGGCAGACGG

GCGCAcgggagcgagcgagcggggCCTGGCAGGGGTGGCAACAAGGCTGCCCTGGATAAT

GGTAACGGGGGTAGGGGAGGCGGCGGTAGCAGAGTTATCGAGAGGCGGGTGGTAGAAGAG

CGCAGGCCGATGCCGGAAGAAGAAGGTCCGCGCGGTGCCGAGCAAGGGCAGGACGGCGAT

GGCGGTTTCGATCGACGCCGCGGCAATGAACGCAACGGTGACCGTTTGGACGGCGCCAGT

GGCGGGAGATCAGTGCCTTGGGCTCACGAAGGAGACTCGTCGGGCGCAAGGGGGGCGCAA

GGGCTCAAGAGGTCCCTCGATGAGCGTGGATTGGGGGATGTGAGGAGGGGCTTGGACCGG

GAGCCACAGCGTCCTCGTCGGCTGGAGGCGCCGCGGGCGGCAATTCCTGTGGACGCCGTC

ATTGTCGAGGTGTCGGATGATGATGAAGCACCAGCTTTCGCACCCCAACGAAGCATGCAC

AGGAACGAACAGCGTCGGAATGAACGAGGCCCGCTCGAGGACAGGGACCGAGACTACGGC

GCCCGGCCTAGGGGCAGAGACTTGAGGGAACCCATTCCGCAGTCTCTCGTGCAGTACCAA

CCCCGGACCGGGAGACCCCACCCAAACCACGGGTCTGTGTACGACCCGCGTTACGACGAC

CGCCTTCACGACCACCGCATGCCCGACAATCGGCGCAACGACGGCCGAAGCAACAATCAG

GTGTACCGTGACGATGGCAGAGGTTACCCCAACGACCAGCGTTACGGTAACGGCCGGCAT

CACGCGATCGACGGCCCGTCACGCCCCGCGGGCCGTGGGGTTGCCGACAGAGGCCTTGGC

GTTGGAGGGGGCGCTTCGATGGAGCGCCGTCGAGCGCAATCCACACCCAGCTACGACGGG

CAATGGAGAGGCGAGGAGAGCCGTGTTGCCTCCGCGATCGGCCCGGAACGAGATGGTCTA

CAAATGGGAGCTTTTTCACCCATTGAGGGTTGCTGGCTGCTGATCGACCGGGCGAGGAAA

ACCAACTTACCTGAGGACCGGGTCAACTATCTGCAGGGGCTGCTTGAGAAACACGAAACG

CAGAACATGACGTTTAACATGCTGGTGGACCGCACGGAAAGCTGCCTGTATGACCGAGCA

GACATTCAGGCGGAATTTGTCAGGCTCTTCTCACAAATGGATTTGCCTCCGCCACGACGA

CCTTTCCGCGCGGCGCCGAGACATGCCGACTGCACGAACGGCAGCGCGTACGGTGTCGGT

GCTCGTTCGGCACCGCGACGAGCACCCGAGTACCCTTTGGAACCGCGTTCCGGAGCGGTG

CGTGACGAGCGGATGGAGCGCGTGGTTGCGGCTGTCACCGTTTCCAACGCTATCGTGGGG

GCACGGGACAGGGAGCGCTTGGGCCCGGAGCCGCAGACGGTGGCATTCGCCTCCGATACG

CGGATGCTAGGTGTGGGTACTTCGATGCGAGGGTCCGGCGAGTTTGCAGCGCCGGTCCCG

GAAGGATATCCAGAGCAAGGCAGCAACTCTTCCAATACGATAAGGGCAGGTCCCCGATCA

GCTCACTCTGACTCGGACCAAGTCACAGGACCGTCGCCCACTGGAACCCTGGCCCGCACA

CCACCTCTACGACTCACCGACGGCGCGACCGGTTCCTCTCAGACCGCACCAGGATCTTCC

GCGGTTTCCTCCTCTGACAACCCTTTGTCAGGGCAAACACCGTCGTGGGTCGGAGCAGAC

AGAAACGGGGGAAACCTGGCTCCTGCCCCTGCCCGTGGCCAATTCCCCATCGCTGCGCCC

TCGACGGGCTGGGATCCTCGGCAGGCAGAGATTGGCTACCATGGTAACCGATCTGCGGAT

GCCGGGGAACACTTGCCCGCTGCACAAGCGGGGCGTTCCTCGCCTGGTGTCGCGCGTACT

CCCCCCGCAGCCGCAGCCTTTGGAAGCGCCGAGCAATGGGACGGGGTCGAACTCGGGAGG

GGGGAGTACGGACGAGGTGTAAGTGGGCGCCCCTAA

>g8362.t1

ATGAGGTCTGCGGTGGCAACAATCGCGGCTCTTCTCTGCTCCGCCTCGGCGAGCGCCTTC

GTCGTACCGGCGGGCCGACCGAACGTAGCTCGCCAGACGGGCGCGAGCGCGGCGAAGCGA

GCAACGTTGGCCCGAAGTGGCCTCGCGATGTCTTCCGTCGCATCGCCGCCGACCCTGATC

CCGGCAGCCACACAGGAGAAGCCGAACGTACAGGCGCTCGGATCGCACGACGAGTACTTG

GCGGCGATGGCACAGAACCAGGACAAGCTGGTGGTAGTGAAATTCTTCGACCAGTTCTGC

CGAGCGTGCGACGAGATCCGACCGCGATTCGAAGAGATGTCGCGATCTCAGTCCTCGGAG

GACGCAGCTTTCTTCGAGCTTGAGTTCTCTACCTCGAAGGACCTGTGCAAGCAGCTCGGC

ATCAAGCGGCTACCCACTGTGCAGATCTACGACGGCAGCAACGgccgGGTGTCCGACCTT

CCCGCGGGACCGAGCCGATTCGCCCAGATCGAGGAACGTTTCGAGGAACTGAGCTCGGCC

AAGGCGAGCGAGAAGGCCACCGCGGAGTAG

>g8408.t1

ATGGACCCGAGGCAAAAGATGACCTCAGCGTTCGGGCTACGAGCGATTGACGACCGCATG

GAGGGAGACATGGCGCGCATCAAGCAGGCAGCCACGATTGCAACGGGCCTGACCGGAGAA

CTCACCCCCAGCGCCTCGGAGTGCGGAGATGACAACAACGCCGCTTCCAGCTCGGAGGGA

GAGCTCACACGCACGCTGTCCGCCCTGCAACGTCTTTCCGAGGAGGAGCGAACCAACTTG

AGACGGAAACTGGGGCAGCAGCTGCAGGCTTCTCGGGAACCGGACCACGATAAGTCCAAG

GCCCAGGATGATGTCATTGCCGTGGCGGCCGACTCCCCGTACCGCGTTGTTCTGCAAAAG

GCTGTCGTCGAGGGAGATCTCCAGGCCGCGTCGGCCGCGCTCCTATCTGGCGCCGATCCC

AACGAACGTGACGATCTGCGCCACAGCTCGCTGCACTTTGCGGCTGCGAAAGGAGACATC

GGTTGCGTGAGGGAGCTCATGAGTTCTGGGGCCCGGGCGAACGTCGCCAACAATGTTGGA

TGGAGCCCGCTGCACTACGCCGTATTGGGCGGGCATGTGGCCGCGGTCGAAGCGCTGCTG

TGCGCGGGAGCATACCCGTGCTTCCGTGATAACCACCTGATCTCGCCCTTGCATCTCGCT

TCCACTCAAGCTGGAGACAGGGTTACTTTTCAGCGCATTGCGGAGCTGCTCGGTCCCTCC

GCCCTGCTAGCACGAGATGAgCTGGAGCAAACACCTCTTCATGTAGCATCCTCCTGTGGT

AACCGCGGCGCGGTGGCCGTTCTCCTGGACCTTAACGCCGACCCCGAGGCAACCGACCGC

AAAGGCCGGACACCGGGGCAGTCGTTCCTGCGGCAGGTGTCGAAGCGCGCGCGCGCCGAC

ATCCGCGCCTCTCTGGCCGACGCCGTCGAACGTCGGACCAACGCGGCGGAGGCCACCGCG

GCCGCTGCGAGTTTCGGGGATGGTTCCGGctcgtcccctgtttttgttaTGTCGGCGTTG

GCGTCGTCGTTGTCTGGCGCGAGTACTTCCAGCTCGAACTCGAGTTCGTCCGGGGCGggt

gctagtggtggtggtgctatggCGTCTTCCGTGTTGTCTGCGATGTCCGCGTCCGGCGAC

CGTTCTTCTCTCGCGAcggcagcggctgcggcggctgcctCCACGAAGGTGGTGATGAGA

AGGGCGGGGTCGACGAGCAGCTACGAGGAAGCAGCTCCGGTCAAGAGGCCTTCGATGCTC

AGCCTGCTGCCGGCGTGGGGGGGGAACGGGCGTGGGGCGGGAGGAGCCAGCGAAAAGGGA

GGGGAAACCTCGTTCCGAACCGCCTTTGCCGTGTtgtag

>g8768.t1

ATGGCATCTTGCCTCTCCCACAGATTTATCTTCGGGGTGCAGGGCAGCCGTGCAAACGCT

ATTGCATTTTCCGATCAAAGTACGATCGTCTACGTAGCAGGACACAACATCGTTATCTAC

AGCATACTTGACGGGAAACAGCGCTTCATCCATGGCGCAGAGTCATCAGAGTCCATCACG

GCCATGACACTTTGCCCTTCGGCACGCTTCGTCGCGCTTGCTGAGAATGGCGACAAACCC

ACGGTCTCTGTGTATGACCTTCGGACGCTTCGAATACGGAAGACACTGCAAGCCGACGGA

GACAAAAGCCAATGTGTAAGCATGGCTTTTAGTCATGACAGTAATCTACTACTTACACAG

GGTGGGGCACCGGAGTGGACCCTCGTGTTGTGGAACTGGGCGAAGGCTCGCCAACTTGCG

AAGATTCGAACATCTGAGACTCTCcctgtctaccagGTCAGCTTCAGCCCTGTGGATACC

TCACTGGCTTGTGTTTCTGGAAACTCAACTTTTCACTTTTATCGCGTGGCCGAAGGGGAC

TTACGACCGATGACAGCTCCACGAGTCAAGGAGCACAATTTTCTGTCCCATTCTTGGCTA

AAGCAGCCTGAAGATCACCTTATTCTGGGCACAGAAACCGGTCAGCTCGTTCTGTTCAGA

TCGGGGGACTTCGTGTGCTACCTGGTCTGTGCGCCTGGTGGCAACACCAAGGTCACAGCG

CTGCTTTCGTTTTCACAGGGTTTCATCGCGGGATGTTCCGACGGGGGGCTGCGTTTGTAC

ATGATGGACCCCGCGGAGATATCAAATCCCTCTAAAATGTTCGAGTGCAAGCAAACATGG

CGCGTGGACACCACGGCTGATGTGGTGAGCCTCGCCTTGTGCCCGAGTGAAGACAGACTG

TGTGCGGTGACATCTGACAACCAGCTGTATGAGGTGAAGATTAACAGCCAGCACAAAGAT

AATGGTATGAATCCGGTCATATCGATGTTTCACGGGCCGGGCCTGAACTCCGGAGGCATC

ACGGGTATGGACACGTGTGTTCGCAAGCCACTCGTGGCAACCTGTGGGATGGATCGCACA

GTCCGAGTATGGAACATCGTTGAGCAGCGACTGGACCTGTGGAAGGCTTTTCAGGAAGAG

CCGTTTAGCCTTGCGATGCACCCGTCAGGGTTGCATCTAGTGGTGGGCTTCGCCGACAAG

CTTCGGTTGATGAACCTCCTGATGGATGACGTCAGGACTTGCCATGAAATTGCCATCAAG

CAGTGCCGAGAAGTCAAGTTCAGCAATGGAGGAAACCTGTTCGCAGCAGTGAACGGAAAT

GTCATAACAATTTTTGACTTCAACACTTACAACAAGATTGCGGATCTTCGTGGCCACAAC

AGCAAGGTTCGACACCTACACTGGGGGGGTCAAGACCAGACCTTAGTGAGTTGTGGTCAA

GATGGAGCGGTCTACCAGTGGGATGTCGATGAAGCCAAACGGCTGGGCGAGTTCGTTCAG

AAAGGAACAGCTTACAGCTGTGCTCTCAGTACGAAAGACTCGGTGTTTACGGTGGGCAAC

GACCGCATGCTAAAAGAGCTGGAGATACCTGATTTCCAAGTGATCAAGGAGCTCAATGCT

GGAGCTACGCTCGGGCAGATAACACTATCGAACTCTGAGCATATGATGTTCGCCGGCACT

TCTGAGAGCGGCAAGCCGGGTTGCGTGCGAGCCTACAACTTTCCGCTCACTGGAGACTAC

CTTGAGTACGCCTGTGTCGGCACGCCTATTACGCGCATGTGCATGACGCATGACGACCAT

TTTTTGTTGGCGGCGGATGAGGAGAGCTGCCTGTTTGTGTTTGACGTGCGAGACCGGCAA

GATCGAGGGCAACCAGGCTCAAAACTGGGAGTTGGCGAGCTACAATCCCTTGCGGCGTCA

GAGGAGATTCTCGTGACAAGAAGCAACCTCGAGGACAAAAACACCCTCATGATAGAGCTC

AAGAACAAGGTGGATGAGCTGGCCCTCCACAACGAGTACCAGCTCCGACTGAAGGATATG

AACTACTCGGAGAAGATTAAGGAGATCACCGAGAAATTCACCCAAGATCTGGAGCAAAGC

AAGAACAAGTTTGACTTACTGAGGGAAGAAAAAAGCGACCTGGAGATGGAGTACGAAGCG

CGCCTGAGAGAAATGGACGAGAAGCACCAGCACGAGCTCCAAGAGCTCGAAAACGCTTAC

CAGCAAACGATTATGGGTGAAGTCGAGCGATATCAGGCGCTCATACAAGAGAGAAATATG

CAGCAGGCTCGATGGgacgaacaacagcagctccTTGTCACAACACACGGAAGGTACGTT

GCTGAATTGACAGAGGAATTCGAGCAGAAACTCGGCGAAGATCGCCAGCTGCGTCTTCAG

TtgcacgaagaaaaaacagaacTCGACAGAGAGTTTACTGAAACGAAGCACCAGGTGGAG

GACGACATTGACACAGAAATCGACAACCTGCGGAATCGCTTCGAAAACCAGCTCTCCGCC

GAGCGCGAGGCAACTCTCCGATATAAGGGGGAGAATGGCATAATGAAGAAGAAGTTCACG

GTCTTAACAAAGGATATTGAAGATCAAAAGGAGGAGATAAGGGGGCTGCACGAGAAGGAA

AAGGACCTCCAGCAGAAGATTAAAGGGTTGGAACGAGAGATCGCAGCTCACAAACGAGAG

ATCAAGTACCGAGATGATACCATCGgagaaaaagagaagaagaTCTATGAGCTCAAGAAA

AAGAACCAAGAGCTGGAGAAATTCAAGTTTGTGCTCGACTTTAAGATCAAGGAGTTGAAG

CGCCAGATTGAGCCTCGCGAGACGGAAATTGGCAGCATGAAGGAACAGATAAAGGAGATG

GATAGAGAGCTGGAGCAGTTCCACAACAGCAACGCTCAACTCGATTTACTTATCGGGGAG

CTCCGCGAAAAGCTAGACGAGATGCAGGCGCAAAACATAGACCAGCGGAAACGGATTGCA

GACCAAGAAACTTCTCGCGGGCGCCTTCAGAAAGAACTACACCAGTGCGTCCAGTACATC

CAAGACCCGTCAGCATTACGAGCGCATGTCACGGCGATGTATAAATCCAATGTCAACGTT

GACCTACCGAGAAGCGAAATGGATGCGAACGTGATCCACGAGTACCACCGACACAAGGAG

TACTTGGAGTCCAGCCTGCGCTACTTGCATCACAAGTTTGTCGAAGATGTCAACGGCCAC

CGTACGGATAACATCAAGGTGATGCAAGATAACATGTTGCTGGTGAAGGAAATCAACATG

CAACGTGGGCACAACAAGGCGGCTAAGAGGGTCCTGGAGGCGCAGgTTAACATGCTAAAA

CGATTTGGTACTTCTTCGAGTCACGGAGGCACCACTAATGCAACACCGCAACTTGCGATG

GCTGTCACTGCTGACCGACGTGCAAGCAGTCAAGAGGAATCAGCAGCCATAATTGAGAGC

AACAAGGCTAGGATTGCTGACCTGAGGACGCTGGTTGCTGATCTAGAAGGCAGGGTGGTG

TCCAATCGGCCATGCAGCCGAGAGATTCTACCACCTATGGAtggcaccactgctgtattc

taG

>g9123.t1

ATGGCGCGCACGAGGCGAAAGGGTGCCTTGCGCATCGCCGTGTTTACCCTCTGCGTGCAG

AGGGCACTGGCCTGGATGCCACAGGTGCTCACAGACCACCGTGTCCGCGTTGCCCACGCC

ACAACAACAGGGAGGAGAAAACGTCAACAACCGGAGCATCAACCTCTAgttgcatcagca

gcagcagcgggggcagcggcaccaccggaggcagcagcagcagcagcagcagcagcagcg

cctgcgGGCGGTGCACAGCCTACGGGAGAGGCTGTCACCGCCAGCCAGCAAAGCGGAGAG

GCTActgttgccaccaccaccgctaccaacaccaacaccaacaccaacaccgccgctaAG

GCCGGCGGCACCGTCGGCGGGAACAAACTCCAGTCCGTCGACTTCACCACCGCGCTCCTG

CTGAGCCGGGAGCTCGAGCGAACGGTGGTCCCGGCCCGGGTCGAAAACGCTTACCAGCTG

GACGCCCACAACCTCGCTTTCGGCCTGAGGACGCTCGAAGGAAACCTGTGGCTCCACGTT

TGCTGGCACCCGAAGGGGGCCAGGTGTCACGTCGGCGCGACCCCGCCCAGAGAGAAGGAG

CAGAAGGCCTACACCTTCTCGCAGACCCTGCGGTCGCTGGTGCGAGGGCTCAACATCGTG

GGGGTGGGGCTGGCGCGACCGTTTGAGCGCGTGGTTCGCCTCGACCTCGCCCCCCGGCTG

GACGTGCCGACAACGTTCCGGCTGTACGTGGAAATCATGGCCAGCCGAAGCAATGTGGTG

CTGGTCGCGGTTGACTCGGTGGGGGTCGAGACGATAGCGGCGTGCGCTTATCAGGTCAGC

CCGAGCAAGTCCGTCCGCCCCCTTTCCACAGGCCAAAGCTACGAGCTACCCCCAGCGATG

GCGGCAAGGCGGCCTACGCTGGAAGAGTCGTTCGAGGACTTTGTGGAAGGGGTGGGGCGG

GTCCCCACCCAGGCGCTCAAGAAGGCCCTCGTCAGCGTCTACCAGGGCACGAGCCCGGTG

CTGGTCAGCGTTATCGCCTCCGGCGTGCCCGGCCTGACCCCAGCGACGAAAGTCGGAGAC

ATCTCCCCGGAGCAGTGGCGCGCGCTGTACTCGGGCCCCTGGAGGCGGTGGCTCCAAGTG

GTCGGTGACGGAGGATCCCCACCCGCCCAGGCCACCAACGGGTTTCCAGATGCCCACGAC

AGCAGTGTCGGTGATGTCGGTGATGTCGGCGATGTCGGTGATGTCGGTGATGTCGGttgc

ggtgacggtggtggaggtggtgacgATGTCGCCGGTATCGCCGGCGGTAACGGCGGCGAT

GACCAATCACCGAGCCGTGGGGACGCGCGGGTCGATTCTTCTTCAGACTTGGTGTCGGTG

ACGCCATGGATCGCGGAAGACGGGGCGTCTTACTACCCCACCACCCTGGCAGAGGAGGCT

GCGGctcagcggcggggggggggggggggggggggggggggggagggggggggggacagc

ggCGGGCGGCGGCTCTTCGGGAGCCTCGGGGAGATGATGGAGACGTACTACAGGGGGGCC

CAGAGCAACGACGAGTTCGACGGGCTGAAGAGGAGGTGCCTGACCCGCGTCACCGCCACC

CTCGTCAAGCTGCGGGAGCGGGCGGACGAGTTCGAAGGGCAGCTGGCCGCGGCGCAGGAG

GACAGGGTAAGCAGGGGGTGTTCACACGGCCGGTGGCACTGGCGACTCACGGTGTCGACG

CTCACGATAGCGGCGGTCGCTGCGGCCCTCTCTCTGCTTGCACCCGACCGTCACGATGGC

GGAACTACTTTGCCTCCTCGGGAACGACGGTCGGCGTGTTCTTGA

>g9262.t1

ATGGCCATGGCAACTTCGCTGAATCGCCCGGAGATAAGAACCGGTGGTCTTCTCCAGAGG

TACGAGCGCCCGTACGGAAAGGCAGGCCAGGAATGTGAAGACTGCTCGCGGTGCGGCCTT

AGTAAAGAGGATTGCATCGATGGATTTTGCCGCCCCGGTGATGGCGTCGAGTGCGCTGCG

CTAGgtgccGCCTGCAGCAACAGTGTCCCCTGCTGCTCTACTGAAGAGGACTGCATCGTG

CCCCCAGAAATGGACACAGCCGAAAATTTCCTCAACAACGGGTTCTGTGAGGTTGCGGAG

TCTCGCACAGGCGAATCTTGCTCGACAGCGGACAACTGCAGGTTCGAGTACGACCTCTGC

GTGGACGGCACCTGCAAGGTTAACCCCAGCGTGCAGGAAGACGCCTCAGTGAAGACCACC

GTATCGGTCAGCGCTAACGCATACGACACTCGCTTGGCGGACAACAACGGCTGCGGTAGC

ACGGGCTGCCTACCGGATCTCACGCGTGACAGCAACACGAAGACAGAGTCTAGGTGGTCG

TGCAGCAAGAGCCTGGGCGAGGGTAACTGCTACATCGAGTACACCTTCGACGAACCCCAG

GACGTGATCAGCATGAACATCGCCTTCCACAAGGGTGACGAGCGCACACGAAGGGTAAAG

GTACTGGGTGACGGCGCCAGGCTCGGCACTTTCACATCCAGCGGCGACACGCTCGACTTC

GAAAACTGGACCCTAAACGCCAAAGATGTTTCCTCTATCAAGCTCGTCGCCCGCGGTCTG

CGGTACAAGGACTGGCTCAGCATCACCGAGGCAAGCCAACAGTTTTAG

>g9272.t1

ATGATTCTGTTTGGCGACATGGACTTCTTCACCTCCGACTCGGTCAGGAACAAGTCTGGC

CAGCGCCTGTGGCTGAAATTTATGTCCCTTCTGGAGGCTGGGGGGCACGTCTACCGCGAC

GGACTGCCGGCGGTCTGCGAGACCCACAAGACGCGCGTCGACTTGTCGTCTCCGGAGGCG

TTCGACCAGAACGTTCCTGACGGCGGCTGCCGGGTCATGTGCGGGGCGACGCTAGCTTGC

GACGGAGGCCACCCCTGCCCCCGgagGTGCCACCCTGGAGACGACCAAGCCCACAAAAAC

GCCGACTGCGGAGTAACTGTGGTAGACGTCTGCCGCAAAGGCCATACCTCCAAGCGCCGG

TGCACGAAAGACCCCGCGGGGCTCCCCTGCAAGCCGTGCGAGCGCGAGGCCGACGCGATC

GCCCGCGAGGTCGCTCGCTTGGCCGAGTCCGAGCGCCAGCGAGAGCTGGAGCGCGAAGCG

GCCTCTGCACGGCTTGCGGAAGCACGGCGCAACGCCGTGCTGGAGCGGGAGAGGCTGACC

CACGAGATGGGCCTGTTTCGGATGGAGCGGGAGACGCAGCGGGAGGAGCTCGACGCCGAG

AGGACGAGGACTTCGCAGCAGGGCGCGCACGCAGAGCTGGGACACAATCGCGCCGCCGCC

TCTAAGCGCGGCGTCAACGcgacaggcggcggcggcggcggcagggacagcggcggcggc

ggcaaggcaTCATCGGGTGTCGCCGACAGCACGGGGTCGGCCGTGTCATCGTCGCGGCAG

CCGACCGGCATCCTGAAAGGGAAGGACAAGAAGAGCGGCAAGGCACGCGGCGACGAAGTC

AAGACTGCCTCGGcaggcgacaacagcaacagcatcaacgcTACTAGCACGAAGGGGGAA

GGGTCTCAGCGAGACGAAAAGGGAGGCGTCATGTGCATGAAGGGCTCCTCCACCCTCCTT

CTTAtcgcgacggcagcggcggcggggaacGCCGGCGGCATCATCGACGCCCTGAAGGCA

ATACCGCCGAGCGACCTGGACAAGACTTCTCACGAGCTGGCGGTGGCTCTGGGAGATGCG

GCGTACCAGTGGTTCCCCCCGTCCAGCGGAGGCGAGCCCGTCCTCGCGCGTCCCCCGGCC

GCGCGCACCGCCCAAGCGCTGGGGCTTATGGGTAAGGGGGAGTGGGTCAAGGCCCGGGCC

ACACTCGCGGCAGCCCTCAAGGACACGGCCGAGGACTCTCACCAGACGTCGTCAACATCA

TCAAAGAGGGACCCGTCCGCAGtctacgctctcgcgctttgcgACCACCACCTCGGTGGC

GGGGCGGAAGCTGCTCGACAGCTCTCCGAGCTTGCGGCCGCGGAGCGGGGGTTTTGGCCC

GGCCCGCCCGACGGCCACCTGCCTCCCGACGCGCGGGCTTTCCCGCTCGTGGCGCTCGTG

CACGCCCACCTTGAAGCCAGTACGCCACCTAGTATAGATCCTCCCTCAACAGCTCCTTCC

AGAGCTGGAGCTAGAGCTGGAACTGGTGATGGGAATGGAGCAGGAGCCGGAGCTGGAGCT

GGGGAAGACACTCTCGATGAGGGTAATGCCGGAGATGACCCCAAGCTGCGAGCGTGCCTG

CTTGCCGTTTCCTTCCTCCGCGCGCCCGCGCACAACCGCAAGGCTGGTAAGGTCGATTCT

GTGGGATGGACAGAGGCGGCCGAGGCGGTGGTAAGGTCGACGGGCGCGGCGCTGTCTCGA

GCTCTGTGGGGTCCCGACGCCGCCGCGGACAACGATAGTGACGGTTTGAATGAAAAccat

ggtggcggtggtgggaagggggcgggggacaACAAGGTAAGGGCGCAGTGGCGGGCGCTG

CAGTCGAAGTGGGGCGTTTCGAGCAGGGCCATGGACGACCTGCTGAACATGTCGGGGCTC

GACGTGATCAAGGCCGACTTCTTGAGCGTGGTGAAGCTGGCCGTGATAGACAGGGAGCGC

GGCTTCGACCTCTCCGCCAGGTCGTTCAACGTCCGCCTTGAAGGGAACCCGGGCACAGGT

AAGACAACCGTGGGCAGGTTGTACTACCGCCTTTTGAAGGACATCGGAGTGTTCGCCTCC

GCCGAGCGGCGGGCGCAAGACCTGCAGGACGCGGCCAACGCCGCGGCAAGGAAGAAAGCC

GCCGACAACGAAAAGCTGAGGGTCAAGAACGAGCGCAAGGCCTTCCAAGCCGCCGGCCTT

CCTTACAACAACCCGCCACCAGCAACGGCGGGAGGCGCcagtagtactgctactactgcg

gtCGGCACCCTCGCTGCTAACTCCGGGTCAGCGGCGGGTGCAGGCGCGGCAACTTCAAAA

GAGGAGGGTTTCGTGGAGACGACCGGTGCCGCCCTGGCCGACTGCGGCACAAAGGGCCTT

GAGGGCATGTTGGCTAAGATCAAGAGCGCTGGCGGAGGCGTGTTGTTTGTTGAtgagGCT

TACACTCTTGAGCCCCAGGGAACCGCAGGAGGCCAGGGGAAACAGGTGCTAAACTTCCTG

CTGGCCGAGATCGAGAACCGCCGCGGGGAGCTGGTGGTGGCCTTCGCCGGATACGCCAAA

AACATGGAAACTCTCTTCGAGTTCAACGAGGGACTGCCCAGCCGTTTCCCCAAGACCTTC

AAATTCGAGGACTACAGCGACGAGCTCTTGCTAGAGATTTTCTTGGGCCTGATGGAGCGC

AACCAAGGGTTGGAGACCTTGCGGCTGGCCCCGGGCGCGGGGGAGGGCGCCCGGTGGGCG

AAGGTTGCTATCGCTCGCCTTGGGCGTAAACGGAGTATGCGCGGCTTTGGGAACGCCCGG

GCCGTTCGCGCGCTGTTCGACAAGGTCATGCAGCGCCAGGCGGACCGCCTCAGCTCGGGA

GGTGAGGCTGCCGATGACGCCGATCCGTACGAGCTCGTGAAGTCTGACCTGCTGGGCGTG

TCCATCTCGAGCCTCGAGGACTCGGCGGATTGGAGGAAGCTCCGGGACATGATCGGGCTG

GCCAGTGTGAAGGAGGCGGTGCACGCGCTAGCTGAGCTGGTGAAGACCAACCTTGTACTC

GAGGAGGCGGGAAAGCCGCCCAGGGGAGTCGCGCTTAACCGGTGCATGATCGGGAACCCG

GGCACGGGCAAGACGACGGTGGCCAAGCTCTACGCTGGTATTCTGACAGACCTCGGCCTC

CTATCGAAGGGCGAGGTGGTGCTCAAGACTGCGTCGGACTTCGTTGGATCCGTCATCGGC

GAGAGCGAGACCAAAACCCGTGCCATTCTGAAAGCGTCCGAGGGCTGTGTTCTTGTCATC

GACGAAGCTTATTCTCTTGGTCCTGGCTCGACGTCGTCGACGGACCCGTACCGGCTGGCG

GTCGTAGACACCTTGGTGGAGCACGTGCAGAACGTCCCCGGCGAGGATCGGTGCGTGTTG

CTGCTGGGGTATCGCGCCGAGATGGAGGAGTTCATGAGGAAGACGAACCCAGGACTTGCC

CGCCGGTTTGCGATGGACAACGCTTTCCTCTTTGAGGACTACACAGATGAGGATCTTCTG

GCCATTCTTCGTGGCAAGCTGAAGAGCGAGGAGCTGATGGCGGGGGTGGAGTCCCTCATG

GCGGCCGCTGCCGTCCTGAGGCAGAGGCGTCTGACGGCTTCGCACTTCGGAAACGGTGGG

GAGGTGGCCAACCTGCTGAGCGAGGCCAAGCTCCGCAAGGAGAAACGCCGGGGCGATGGC

TCCATAGCATCCAGGCTCGACCCGGAGATCGTGCCTCGTGACTTCGACCCGGGGTACGGC

GATGCACCCCCTGATAGCGCGACGCTCGAGGAAGACTTGTTCGGGGATCTCATCGGCTGC

GACGCCATCAAGATTCAGCTGCGGAGGATCAGGTCCACGTTTGTACACGCTGAGAAGGTC

GGCCGCGACCCTCGCGAGACGTTGAACCTCAACTTTAGGTTCACAGGTGCCCCGGGTACG

GGCAAGACCACGATTGCCCAACGACTCGGTCGCATGTTCAAGCAACTAGGCCTTCTCCGT

TCGGACGAGGTGGTGTCTTGCTCTCCGTCCGACTTTACCACTGGCTACGTCGGCCAGGCC

GCCATCAAGACGAAGGAGATGATGGACAAGGCTATCGGGAAGGTCCTCTTCGTCGACGAG

GCGTACGGCCTCAACCCCCACCAAACCGGGTCACACGGGGCAACCTTTATGCAGGAGGCT

GTAGACCAGCTGGTTCAGTGTCTCACGGACGACAAGTACAAGGGAAACATGGTCGTCGTC

GTGGCTGGGTACGCCCGTGACATCGACGGCCTCATGCAGGCCAATCCGGGACTTGCCAGC

CGCTTCCCCGAGACGCTGCACTTTCCAAGCTTCGACGTGCAGGACTCCAGCCGCTTGCTC

GAGACTGGGCTGAAGAAGGGGTTCAACACGGATCTCGCGCCCGACGCTGTGGCCGCGCTA

CCCGGGTTGCTACAGCCTCTGGTACAGGCCCCTCGCTTCGGCAACGGCCGCACCATCACC

GACCTGGCAAAGCGGATCTTCACGGAGATCGGGATGAGGATGGGCGACGGGGGTGATGGG

GGCATCGACAGGCAGACCAGTGGCCGCCGGAGCGACCCAAGGGCTTCAGTCGACGACGTT

CGCCGTTCTGTGGACGCCGTGCTAGCTCAGATGGCCGAGGGGGGTGCCGGTGAAACGCCT

ACTGCTCAAGGGgctaacggcggcggcggcagcggcagcgcatCCCCTGTTGCTTCATTT

GGCGCTAGCAGCGAGGCACCCAAGACGACGAGGGTCACggccacgacgacaacagcGACA

TCGACTTCGTGTCCGGCGGAGATGACTTGCGAGGAAATCTTCACGGGGGCAGGAGAGGGG

AAGGAAGGCGGCGAAGAATGGTCGGACCCCTCGAACCCGTTCTTAGACGATAAGGACAGA

CGAACCCTGCAGAGGGCGTGTGAGATCGCGGGCATTCCCGATGACAGTCCCAAACTCAGC

CAAGATGTCGAGGACAACGAGGACCTCCGGCGCGCTCTCCTCCTGCCCTCGTCGAACGGA

GGCGTGGGTCTCTCTGCCGAGCACGCGGAGGCGTTTCTGCAGAGGGTGGGCACGGACAGA

GCGGCGATGTCGCACCTCGAGAGGGAGGCCCTGGATGCCGCGGCGAAGCGCGTGTCCGAC

GAGGCCGACGCCAAGCGTGCGGTGGAAGAGGCGGGCGCCGAGATCGACGAGGCGGAGTCC

TCCGTAGGATTCGACGACGACCTACTCGCCCTTCTGAGGTTGGCGAAACGTGCCAAGGAG

GAGGCTCTGAGGAAGCTCGAGGAGAAGAGGAGAGTGGAGCTAGCTAAGGAAGCGGCGACG

CAGACCGCTATTCGGCGTATGGGAGTGTGCGTAGCAGGGTACCAGTGGCACCGGACGAGA

AGCGGCTGGAGGTGCGCAGGAGGCACTCACTTTCTGTCAGAAAATGCCGTCGCCGCCGAG

ATAGCTCGTGGAAGCTCATCGTAG

>g9333.t1

ATGATGAAGTCTTGGGCTTTGCTTGCACTGGCGACGTCGGCGGTATTTTCCACGGCCAGA

GCTGCATGCGTGGACATCGAGCGGTATCTCGACATACCCTCGCCACTCACGGGCGACGTG

GAGATCAACCTGGATGGGGTAGACACCATCGAATGTGGAGTGACCGGCGAACAGGCCGAA

ACTAGCGCCGACACGGTGGAGACTGTCACTGTCGACGGTGGGACCCTCACGCTCAAGAGC

TCCAACAATGTCCGGTTTGTGAACGTCGGCTTCACCGTTAGGTCTGGGGCCAACATCGTG

TTTGACATGCCCAAGACATTGTTCGGGCCCAACACCGAAGGGGGCAAAGACGTATCGGGT

TCGAGCTTTGCTACGAGAGTTGAGGAGGGCGCGGGCGCTACTTTCCTGGGGCAAGTTCTA

GCGAAGGAGGTGACCAACGTGTTCGCTCTCTTCCGGAACGACGGCACCATGGAGTTTAAG

GAACGTGTCGTGTTTCAGGACAACGGAAACGTCTTTCCGGACAACACCGGGGTCGTGAAG

TTCAGTGGCGATGCCACCTTCAAGGACAACCGCTTCCTCGCCCTCGAAAACCGGGACTCG

GCATACGTTATCTTCCGAGGAGATGCAACGTTCCGGAACACCAACTGCCGAGAGGCCGGT

GCCGTTGTCAACGAGGGAGACATCACGTTTGAAGGAAAGGCGTACTTCAACGAAAACGAG

AACCGTCGGTCCGACGGCGGCGGTGTGATCAACAGACGTGGGACAATCGTGTTCAACGAA

GCCGTCCAGTTCAACAATAACACTGCAGGCTTCATCGGCGGGGGCATTGTCGTGAGCGGA

GGAGAAGTCGTCTTCAGCAAGGCGGTTACTTTCGACGGCAACACCGCCGAATCGGGCTCC

GCCTTCGCCATCAGTGACGATGGCTCGCTCACGTTCAAGAAGCCTGATCAAGTTATTTCC

AGGAACGCCGGCCTCGACGAATTTTCGGACGAATTCAGCTGCACTTTTGGATTGGTCTTG

GACGAAGCGACTCTGATTGGCTTCACCGGCGATGATGTCTGCATCCCTGCTGGTGATGTG

GCTTAG

>g9341.t1

ATGACGAAGGCTTGGGCTATGCTCGCGCTGGCGACGTCGGGTCTCTTCTCCACGGccagC

GCTGCATGCGTGGCCATCGACCGGTACCTCGACATACCCTCGCCACTGACGGGCGACATG

GAGATTAACCTTGATGGCGTGGACGCCATCGAATGTGGGGAGATCGATGGCTTCAGCGAA

GGCACGTTGGAGACTGTCACTGTCGACGGTGGAACTCTCACGCTCAAGAGCTCCGGCAAT

GTCCGGTTTGTGAGCTTGGGCTTCAGGGTGGAGACCGGGGCCAACATCGTGTTTGACATG

CCCAAGACGATTTTCGGACCCATCACCGATGCCTTTGACACCACAGACTTTCGAGGCTTT

ACCGTGCGAGTTGAGCAGGGCGCGAGCGCTACGTTCCTGGGGCAAGTTCTAGCGGAGGGG

GTTACCAACGTATCCGGCCTTTTCCGGAACGAGGGCAGCATGGAGTTTGAGAAACCCGTG

GTGTTCCAGGACAACCAGAACGTCTGGAGGGGCAACACTGGGGTCTTGATCTTCAAGGAT

GATGCTACCTTCAAAGACAACGCCTTCCTTGCTCTCGACAACGAGGGCTCAGCATTCGTC

AGTTTTGCGAAAGAGGCTACCTTTGATAACAATGCTTTCGCCTTCGACGGAGCTGATGGC

TGCAGCGTGGCGAACCTGGATACTTCGACGATCATCTTTGGAGGAGACGCAACTTTCCAG

AACAACAACTGCGATGAGGGCGTGGCCGTTTTCAACGAGGGAACCATGACGTTTGGAGGA

AAGGCGCGCTTCAATGACAACGAGAACTCCCgtgccgacggcggcggcgtgaCCAACCGC

GGTGGGACATTGGTGTTCAACAAAGCCGTGCAGTTCAACAACAACATTGCAGGAGGCTTC

GGTGGAGGCATTCACGTGGAAGGAGGCGATGTCACCTTCAGCAAGGGCGTGACGTTTGAC

GGAAACGCCGCCCAATCGGGCTCAGCCTTCGCCATCAGGGCTGGTGGCTCCCTCACATTC

GAGAAACCGGAAAATGTCATCTCCAGGAATGCCGTCGTCGACGGAAATTCGGGCAGTGGC

TGCACCTTTGGAGAGGTCGCGGACGGAGCGACTCTGATTGGCTTCACCGGCGATGACGTC

TGCATCCCTGCTGGCGATGCGGCTTAG

>g9375.t1

ATGTCAACGGCTCTGGGAAAGGTCGCATTTCTCGGGATGCTTGCTCTCAATCGAGCTGCT

GCAAGAGACACGAAATCGCCGAACAAACCTATCCCCAAGCCGGAGTGCGATGCGAGCATT

GCTGTGTCTATTCGGTATTCTTCCGTGTCTGCGCGGCTGTACCTCGAGTCGGCGACTGGC

AAGACTCGTGGCGGCTGTGTGACTCTGGAACAAATATGGGAACATCAAAGAGGCGGGGCG

CCGTTGTATGCAGTCGACCCCAAGAGCGGCGACATCAGCGACAGCCCGACAGGGACTTGG

TTGCTCACCGAGGAACTGTTCGTAGGAGACGGCATCACGCTGATGGTGCATGGTTCTTCG

GCCGGTGGCGATGCTGACGAACTCCGTCTCCTTAGTACGAGCGACACCTTCATCAACCTC

CGCGCTCACGGTGGGAGCTTAGATTTCCTGGGCACCAAGGTGTtcgcgtgggatacttcc

aacAATGCCCCCGACAAAGACGAAGAGGATGGTCGGTCGTACATCAGCGCCGTCTCGGAG

GTCATCACCGACGACAGCCAGACGTGCAACGGCAGAGCTAAGAAGAACATGGGAGAGGCG

CGGATGGATATCGAAGACAGCGAAATGGGGTACCTTGGATACCACGGCAGCGAGAGCTAT

GGGCTTACTTGGAAGGTCCGTGGGTTCTGCGTGGACAAGAGTAACCCCGAACTCTTCGAC

GATGTCAATGTCTACGGCAACATCTACGATTCGGACATACACCACAACAACTTCGGGGTC

TACACGTACGGCCACCAGCAAggagactggaggaggaacaagatgcaCGACAACTCGGGG

TACGGCTTCGACCcgcacgacgacagcgacttccTGACGATCCACGACAACGAGGTCTAC

GACAACGGGTACCACGGCATCATCGCTTCCAAGCGTTGCAACGGGGTTTCTATTCAGGGA

AACGAGGTCTACGGCGGAGCGTCGTCGTCCGCGGGCATATTCCTTCACCGGAGCAGCGAC

GACGCCGTTGTCAAAGGTAACTACGTTCACGACAACGGCGACGCTGGCCTTGCGATGATG

GAGTCGTTCAACGCTGACGTGTCCGACAACATATTCGAGAACAACAAGTACGGGGTCAGG

TTTTCCGTCGGATGCGGACAAAACGTGTTCTCCAATAACGTCATCAGCGGCTCCTCCAGG

TACAACACCTACTCGTACCTTGGATCAGACGCGCCCGATGTGGCTGATTCTGGTCGGTCC

CAAGACAACGTATTCCAGGAGAACACCATCATCGGGGGTGCGGAGAGCATCAAGCTTATC

GACGCCGACGGCACCCAGTTCCTCGACAATGCATTCGAGGATGCTAAAACTATCCGTTTC

AATGACGCTAGAAACACTCTCATGTCGGGAAACACTGGCCTTAACAGCAGCAAGCTCAAG

GTTGCCAACGGGGCTTCGTTTGACGCCCGTTCTGACAGTGGATTCTTGCCAATCGCCTAA

>g9381.t1

ATGAGGATTCCACCACCGTGGGCCGGCGGGGTCGCCGGTTGCCTTCTTGTGTCGTTTGCA

TCTCGCATATCAGCCATCACGGTCGAGTTGTCAGAGACTGTGACGGTCATTGAAGGGAGC

AGGTACTTCTTCGGCAGGGGTAGCATCGACGACGACGGCTGCGGTCTGGCAGGGCTTTGC

AATACAGCGTTAACAAGGGATGGTTCCTTCGACCTCGAGTCGCGCGACGGCTGCACCGAG

CCAGTCGACTCTGAACAGCGGGCCGATGATCCGGGAGACTGCCGCCTTGACTTCGCGCTC

TCGCCGTCATCCGATCAGGCAGCGAAGATCGCGTCAATCAAGATAGCCTTCCACCCTGAC

AGCGTGACCAAGATCAAGCTGTTCATCAGACAAGACGGTTTCGAGCCCCAGGTGGTGGAG

TGGGAGGGTGTGCCGGGGTCGGTCGAGTTGCAGGAGGTGACCGGCCTGGAGGGGGCTGCG

GGTGCGACGCTCTTCATCGGGGGCCTCGCGCCTGGTGAATACCTCGGAATCCTGGAAGTT

GAGATCTATGTGGAGGTGGACGAGGTGGAACCCGTTGGGGCTTCGGCGGGCACTCGCGCC

ACCATCTCGGCAACGGCTACCACGGACACCGCCTCCGCCATGAACACCTTGGACGGCGAT

GCTTCCGATGCCTCCTCGTGGAGCTGCTCGTCGGGAGAAGTGTGCGAGATCACCTACGAC

CTTGAGACCGTGGAATCGCTGGAGCAGCTGCGGATCGCCTTCGCAGAAAGTACGGTTGCT

GggggggagttccacatctcgACCGCTGGCGAAAATGGCGCTTTTTCGCCCGTACGCACC

GGCCTTGAGGCCGACGCGGGTGGGCGCCCCCTGGGCAGCGACGGTTTGCAAACCTTCGGA

GGCGTGCGCGCCCTGGCACGGTACGTGAGGATTGAGGCGGTCCCCGCGGAAGGCGGTAGC

ATTGGCATCAACGAGGTGGACTTTCGCGTGGGCGTTGCGGCTCCCGCGCGCCCTGTGGCC

GAGAAGGCGTGGCTGAAGCCGACCGGGATGCTTCCGCTCGGCAACGACCCGGGGTCTAGC

TTCGTACCCATGTACGACGTGAGGGAGGCTGCCGCCGGCGGCTGCGACTCTCCTGCACAC

TTCGGCGGCTGCCACGTGTACTACATCAAGGACAGAGACATGTCGGACGACTCGAGGTGG

ACCTGCGGGCCGGCGGCGGCCGGGGGCATCATGGGTGGGCTAGACAAGTGCACCATCCAG

TTCAATCTTAACGAGATGCGATACGTTCGCCAGATTCagcttgCATTCCACCTGGGCGAT

GAGCAGCATGACGAGTTCAGCGTCGAGGCGTACATCAAGGAGACCGGCTGGGCGACAGTC

ATACCCTCCGCGATCACTTCCGGAGACACCGCTGGCTACCAGACGTTCGACCTTAGCGTC

CACACGGGCGTGATCCGGCTTGTGCCCAAGTTCCAGCGGTACTACCAGTGGATCAACGTC

AAGGAGGCAAGTGTCATGGAGCGAGATTTCTGCACCGCCGACATGTTCATGCCTCGCACG

CGCCGCTCGCTCGACCCAGCCTACTTGGAGATGTCGCTCTTCCTATGA

>g9383.t1

ATGGTCATCCTGGAGAGGAGGAAAAACGACTTCGTGGAAGGGACGGTGCCCGTGTTCACG

AGGGAGAATTCGTTCGAAAATGAGGTCGTCACCAAGTTTTCAAGAAATTTCAAGTTCGAT

ATCGATCAGCCGGGAGATGACCTCTCGTTCAGAGTTCCGCTCGCCACGATCACGGCCCTG

CGGATGCGGTTCCCGGCCAACCGGCTCTTCGTCTTCGAACTCACCGCGTTCGTCTTCGAC

GCCGAAGCAGATGACTTCAATGAGATGAGGCAGACGTTCACGAGTGCTGGCGGGGGCAAG

GTGTGGGAGACGTTCACCCTCTCGGAGCCGGCAGACCTTTCACGCTTTGAGCTCGTCGCA

GTAGAAGGACCCGACTTCGGCGACGACTTCTACCCGACTCTACGGGTGGCAGACTTCCAG

CTCGTGGGGGAGATTGTCAAGATTCCCGGGCACTTCGAAGTCGCCGCGACGACGCAGTTC

GAGTGGAACGTCATCCCCGACATCATCGGCGACGGAGTTTCGGAGCAGCAGGAGATCATG

ACGGCCATCTGCGAGAATAAGGGGTCGACCTTCGACGGCACGGACTGCGTCGGAGAGCTG

GACGACTCCCTCGTGACGTTTAACTTCGTTCGAGGGGACTACTATTTCGACGGGCCCATC

CTCCTCAAGAATGGGGTGACGCTCGTTGGAGGCTTCTCCGACGACTTTCCGAACTGGGCG

TTCTTCCTCGCGTACGACGGGCCCAACTCGGACAACTCCGCGGAGGAGGCTTTGATCGTC

ATCGATGGCGTTACTGACGCAGAAGTGATGTACATTTCCGTGGCTGGCACGGAAGAAGAA

GGCGTGGCCGTCGGACCATTAGGCAGCCTGTGCCTCGATGTCAAGGACTCCCAGGACATC

CGGTTCACCTTCCTGAGGATGAGGGAGGCTCGTTCTGGCGCGGCCCGATTCGCTGACTCC

AGGAACATCACCTGGGATGTGGCCTTCAATGGAGAGTACGAGACGGGGAACTTTCTCGAG

CTCACCCGCGTGGACGATTTCAGATTCGTCGGCTTCCCCGAGATGTCGGGGCTCCTGATC

GACACCTGCAGCAACATTGAGTTCGACGGCATCTTGGGCAGCGGTGAGCTCGACGACTCC

AGCTTCGATGCTCCTACCGGCGGAGAGCAGGTGGCCAACGTGGTCATCACTGGTAATTCG

TCCGGCATCGTCTTCAGGGAATTCATCGTGGGACCCGGCGCTCGAGCAGAGCCACGCATC

GTGATGGAGTCGACGGAACCCCTTACgctcgaggcggcggcggaaatTACGGATGCCGTC

AGCGGAGACTGCATAATTCAGGTCCCCGAGggaacaacaaacgacctcaTTGTCCAAGTC

GAGCCCGAGGTGACCTTGGCGAAGTCAGGCGACTGCTGGGTCCTAGTTTAG

>g9579.t1

ATGACGGAGCTCGCGCAGTTACTGGGCGTTCAGAATCTGCAGGAGTCCATGTTACGGTCC

TTGGAAAGGAGCACGAAGTTGTTTCGGAACAGCCCCGACTTCGCGATACCCGAGGTCCGC

TTCAGTCAAATAGAGGACGGGCGCTTCCCGTACTACGTTACACGGCAGGTGAAGAGGCGA

GGAGTTCTCGTCGTGAGAGGCGTTGTGCCTTCCGACACGGCCCTCCGGTGGCAGGACTCT

TTGCTGGAGTACCTGGAGACCAACGGAGTCAGCGGACCACCTGGGGCGAGAGACGTGTTT

TGGTCGAGAGCGCAGGCGCAAGCGCGGGAGAACCTGGGCGTGCGGCAGGTGCTGGCGGCC

CTCAACCGGCTCTGGAAGCCGGACCCGTCCACCCCTGTCGACCTCACCAAGAGTCTCAGG

AGCACGAAGTTGTTTCGGAACAGCCCCGACTTCGCGATACCCGAGGTCCGCTTCAGTCAA

ATAGAGGACGGGCGCTTCCCGTACTACGTTACACGGCAGGTGAAGAGGCGAGGAGTTCTC

GTCGTGAGAGGCGTTGTGCCTTCCGACACGGCCCTCCGGTGGCAGGACTCTTTGCTGGAG

TACCTGGAGACCAACGGAGTCAGCGGACCACCTGGGGCGAGAGACGTGTTTTGGTCGAGA

GCGCAGGCGCAAGCGCGGGAGAACCTGGGCGTGCGGCAGGTGCTGGCGGCCCTCAACCGG

CTCTGGAAGCCGGACCCGTCCACCCCTGTCGACCTCACCAAGAGTCTCAGTTTTTGCGAC

CGCGCGTTCGTTGGAAACCCCACTTCTTCCAGCTTGATGGGGCGACACGCGGCGGTCATG

GCGGGTGGGCCCGGCGCGTCTCAGCGTTGGCTTAGCCCTCACCACAGCCAGGCGTACCGC

GACATTTTTGGGGGGCGGTGGGAAGACTGGGACCCGTTCATGGCCGGCCCGAGAATGAGG

GCGGCGGAGGcgtcgtcttcgtcttcgtgGGCAGGGGATGATTCGGCTGCGACCCCGGGG

GGGCTGTTGGAGCGAAGCCCTGAGGAGATGGCAATCTTGACGGAGGGGCAGGACGAGTTT

GATGAATACACCACGCCGATGCTCAAGGAGAAGCTGAAGGCGTACCGCGACATTTTTGGG

GGGCGGTGGGAAGACTGGGACCCGTTCATGGCCGGCCCGAGAATGAGGGCGGCGGAGGcg

tcgtcttcgtcttcgtgGGCAGGGGATGATTCGGCTGCGACCCCGGGGGGGCTGGTGGGA

GgcgtccccggggggggggaaacgccgCAGCCGTTTCGACCGTTTCAAGGATACCTTTCT

TTTGGCCGTTGGGGCGAGGTTGGCAATGTTCACGTTGTGCCGACGATGAAGGCGGCCACC

GCTTTCACTCTGCTGCGGCCGTTCATGGACGATGCCTTCGAGGCGGACGCGGGGGACGCG

GACTGGCTGTGCGGCGCGGGTCAGGGCCAGGAGCTGCCGCTGTCGCCCAAGTGGCATCAA

CCTATCCTGGACGCTCTTGTGAGACTTCCGCCGCTCCTGCCTGGGGACGCGGTGTTTTGG

CACGCGGATACCATCCACTGCGACGCCGACGCGGTGGTGCCTCACGAAGGCGGCGGGGCA

GAAGGCGTTTTTGTCCGGGGAGGGACAGGGGACAGCAGCGGTGCAAGAGGAGATCCATTG

GCCACTCCGAAATTCGGCGGCGTACACCTATCCTCCCTGCCCGTTTGCGAGGCGAACTCG

ACGTACGTTAAGGCGCAACGAGAGGCGTTCGCTGAAGGCGTTCACCCCCCGGGGTTCTCA

CCCGTGGCAGACGGTggcgggaagggggaaggggggtttgTGGCGCGGGCTTCGAGggaa

gagttgacgagggagggCAGGCGAGCGATGGGTCTCCGGCGGTATACTAAGGAACATTAC

AACCCCGCTCTGGAGGTGCACCACAGGTTGCTTGAGATCGCGTACAAGTGGTAG

>g9601.t1

ATGCCGACCAAGTCCTGGGCGCTGGGCGCTCTCCTGGCGCTGGCTACATTTTCAGCAGCC

AgGTCAGAATGCGACGACATCGATACGTATTTCGGAGACGTGAATTCCGTGAGCGAGATC

GACCTGGGCGACGTGACGAGCGTGGAGTGCCCCGCGAACGACGAAGGCGGGGAGCTGGAT

GAGGTCCTCATCGAGAGCGGCAAGACCCTCACCATCAAGTCCACCCAAGACTACGTGCGG

TTCGTCAATCTCCGGTTCACAGTTGAAGAAGGGGCGGAGCTGTACTTCGATATGCCTGAA

ACCCGGTTCGGTCCCAACAGTGGGGCCAGAGAGGGTGGATCAGGATTCATGCTCGAAGTC

CTTGCCGGCGGTACCGCCATGTTCTTGGGAGATTTCCATGGAACCGAGGTGGACAACATC

CGTTCCATGTTCTACAACTCCGGCACAATGGACTTCAAGGGCGACACACTCTtcgacaac

aacggcaacgtGTTCCGGAGCAACCTCGGCACCCTAAAGTTCCGTGGGCACAGCGTCTTC

AAGGACAACGCCTGGCTGGCCATCGACAACGAGGGCGACGATGCTTTCGTCAGGTTCTCG

AAGACTGCGACCTTCGACAACAATGCAGGCGGTTTCGATGGCGCCTCGGGCTGTGGAGTC

TCCAACAGAGATGGCACTGCTATCTTCCGTGATGACGTTGTCTTCTCTAACCACCGCTGC

TCTGAGGGCGCGGCCATCTTCAACGGGGGCAAAATGACTTTCTACGGAAAAGCCTACttc

aacgacaacatcaacttCATGGACTGGGGCGGAGGCGCTCGCAACATCGACGGAAACCTC

CTCTTCAAGGGTGCCGTTCAGTTCAACAGAAACGAGGCTGAATACGGAGGAGGCATTGCT

GTGACCGGCGGCGACGTCACGTTCAGGAGGGCGGTCAAGTTCGACACCAACGGAGCTGAT

TTCAGCGGCGGCGCCTTCGCCCTCACCTTCGGCGGCCCGTACGGCACCCCCGATGAAGAG

ATCGGCCCTGGAGTGATGACGTTCCGCAAGCCAGACGTGGTCAGGATCGCGGACAACTAT

ATCGTCGACAGCCAGTACAACGACGAGCCGGTGACTGGGTGCACTCTGGGCTATGTCCAG

GAAGGCACCACGCTGGTTGGTTTCGACTTCGAGGACGTCTGCATGGAGGACACGGTGGCT

TGA

>g9692.t1

ATGAGCACGGCAAATGTGGCGGTGTTTCTGGCCCTCTTGCCGATCACGATTGCCTTGAGC

ATATTACCGGATTTTCCACTCAGCAATAAACAGCTCAAATCCCGTGCCGGGTATTACACG

GACGGAGGGAAGTACCTGGGGGGCATACCGAGGTTGGAAACCGAGATCGAGCTTTACGAG

TGCACCGACGGAAACGCGACCGTTTGCATGAGCTGGACAGCGACAGAGTCGTCTTCGGAC

GAGCACGAGGTAGGCACGTGCTCTTGCCAGTCCGTGAAGAACGACGAGTACTGCGACGCG

TGGACCTGCAGTCAGGTCGAAGTAGACAACGCAGCCACATGCACCGACAGCAGTGAGGAT

AACTGCTTCTTCGAGAGGGAGGTAGAGAGCACGCGGTGCTCTTGTGATGTCGAAGACGAA

GAATCGTCAGGAAAGTTCTGCGCGTCTTGGGCGTGCGTAGAGACCGACTCCGACGGCATT

CAAGAGTTCGAAGATTATCAGTGTGTGAGGGTGTCGCCTTCCGGTCAGTACTGCGAGGCA

TGGACAGGGGACATTGAGAGTGTGGAAGAGGTGGAGACGTCGGCCTGTGAGTGCGTCGAA

GAGTGGGACGGTGACGGTTTCTGCTCCTTCTGGGAGTGCCGGGAACGTGGCTTAGATAAG

TGTTCTCGTTCTATGTACAGTTGGTGCAATCTTGGCGTGTCCGTTGGAGTGGGTGGCGTC

TTTGGCTTCTTCGGCGCGATACTGGCCGCGCTCGGGTTTGCCCATATAACCGAATACGAC

GAAGTATATATGCTCTGGATTGTGTTGGGATTTCTCTGGATGGCTGGTTGGTCTGCCGGC

GTTGTCATTTGGGGGGGACAAGATGGAGCAATGATATCGGGTATTTGGTGGGGAACTACA

GTTGGATATGCCATACTATGTGGGCGTTGGAGGTCTAGTTAA

>g9751.t1

ATGGGCATGGACGACAAGCTGTCGGCGCCTCCGGACTTCGATGGACCCGTGAGGAATCGC

AAATGTACGGACATCATCTTCTTGGTGGCCATCGTCGCCATGTGGATAGCCATGACTGCC

GTGGGCATTGCCAGCGTTCGAGAGGGCGATGTGCAGAAGCTGCTGTCGCCTACAGACCAT

GACGGAAACCTTTGCGGTTGGGATACGGGCTTTGAGGACCTGCCGAACTTGTACTACGTC

AACACTGAGGGTTCGGGGATATGCGTGGAAAGTTGCCCAGATGTAACGGACTTAAGCGAG

GTGCTGTGCCGTTACGATCGAGATGAGGATGTCTCCGACATCGATGCCACAACGGAGGGG

TACTGCCTACCCCAGCTTGAGAGCGTGTCAGtggtaAACTATTGCGTTTTCACCGACAGC

TCTCGGTGGGACGATGTAACCTCGTCGGACCTAGAAGAGTACCTGTCGAGGTTTATCGCC

GACGTGGTTGAGGCTAGAGCCTACGtcttcgggttcgggttcggagTTGCCAtggtggtg

gcgtttttgtATATCGGGCTGCTGCAGATTCCGTTCCTGGTGTCCTTCTTGGTGTGGTCC

TGCGTGCTGCTGATTCTGGTGGCGCTAGTGGTGCTCGCCATTGGCCTCTGGGTGACGGCC

GGGGAGTGGGATGACGAGGGCACTAAGGATGATGAAGTTGTGTACGGCACCTACGTTGTC

TCCGTCATCGCGATGGTGCTAGCGGTTTTGTGGGTTTGCCTGTTCTGCTTCTTGGCGAAG

AGGATATCTCTCGCTATCGGGGTGATCAAGGAAGCCGGGCGAGCGATAGCTGCTATGCCG

CTCATTGCGGTGTGGCCTGTGCTTCAGCTGGCGGGGTCGATCGCGTTCATGGCTATCTGG

ACGTACTACGCTGCTTACACCGCCAGCCTGGGCGAGATCACGACCGAGACCGCAGAAAGC

GCAGGTGGACTCGACATCTCTTACAAGGTATACTCGTTCGACGAAGATGTGGAGTACCGC

GGTTGGTTCCTGATCTTCTGCTTGTTCTGGTCGCTGAACTTCATCACGGCCATGGGGCAG

ATCGTGATCGCTATGTCGGTCGCCCAGTGGTACTTCGCGAGGAACAAAAACTTGGTGGGA

TCGTTGACCGTGGTGTCCTCGGTGGGCAGGACCTTTATCTTCCACGCCGGGACTGCTGCC

TTCGGCGGGCTCATCGTCGCCATCGTGgagatgATCCAAGCGATCTTGTCGTACATCCAG

AAGAAGGCCAAGGGTACGGGTAACAAGCCCCTTCAGTACATCGCGTGCTGCTGCGCGTGC

TGCTTCTGGTGCCTGGAGAAGTGCATCCGCTTCATCAACAAGAACGCATACGTGCAGACC

GCGATTTTCTCTACGAACTTCTGCACCTCGGCGAGGAAGGCGTTCTTCCTCATTGCCCGA

AACATCGCGCGAGTTGCCGCCGTGTCCATCCTGGGGGGGTTCGTCCTCACCATCATGACG

CTTTTCGTGACCGCCGTGACCACCGTCTGCGCGTATTTCGCGATGGAATCCAACATCCGG

TCGAAGCTGAACTCCATCGTGGGGCCCACGATAATGACGGCGGTGCTGGCGTACTACACG

GGCAAGATGATCACCTCCGTGTACGGGAtggccatcaccaccatcctgCAGTGCTTCGTG

GCGGACGAGGAGCTGTTCCCGCCGTCCCAGCAGTTCGCCGAGAACGACCTCAAGAGCTGG

CTTTTCGTGACCGCCGTGACCACCGTCTGCGCGTATTTCGCGATGGAATCCAACATCCGG

TCGAAGCTGAACTCCATCGTGGGGCCCACGATAATGACGGCGGTGCTGGCGTACTACACG

GGCAAGATGATCACCTCGTGTACGGGAtggccatcaccaccatcctgCATGTGCTTCGTG

GCGGATCGAGGAGCTGTTCCCGCCGTCCCAGCAGTTCGCCGAGAACGACCTCAAGAGCTG

GGTGACCGACATCGGCACAGACTCCAGGCTCACTCGAGCCCCCCGCCTGCGAGGCACGGG

GCTCAGAGCTACGCTGTCTCGTAG

>g9930.t1

ATGGCTTCGATGCCGAATCTAGCAGGGGGGTTGCTGTCGGACTTTCTAGTCAAGGGCGCT

GGCGGCGCAGCCTTTGCCCTCTACAACGCCGACACCTGGCGATGGTGGCACTACCCCGCC

TGGCTGTCGCTCATCCTGCTAGGTTTAGAGCTCGTCGCGGCTCTGGTCCACGTCTTGGGA

CGTGCGTCTGGAGCTGAACTCATCCGACCACGCGGGAAGCACCTGGACCAGTTTGAGCTG

CTAGACAACCTTTTCATCACTTTTAACCGGTGCAGCACTGCGGTCTTCACCTACCACGCC

ATACAATACCTCTGGTACTCCCCGTCGGGAGGCCGGGTCAACTGGGACCTCTCGGGGCTT

GGACTCGCGACCGGTCTCGGCGCGTTCGTCGCTCTGTACGTGGTGTACGACCTGTTCTAC

ACGGTCTTCCACCGTGCGCTGCACGTACGCGGGCTGTACAGGCACATccacaagcaccac

caccgacaGAAGGCCCCTTCCAGAGGCAACGCGGACGCCGTCAACGTCCACCCGTTCGAG

TTCCTCTGCGGGGAGTACAACCACCTTCTGGCATTGCACCTGGTGTCCAGGTTCATCGTC

CCCGTGCACGTGGTGTCCGCCGGGGTGTTCATAGTGGTCGGCGGCTTTTTGGCGTCGCTG

AACCATACTCGGTTCGACGTCCGCATCCCTCTTGTCTACGAGGTTCGATATCACGACATC

CACCACTGGTACCCCGAGTGCAACTACGGCCAGTACACCATGCTGTGGGACTGGCTCATG

GGGAGCCTAAAGCCGTACCCTCAAGAGGATCAAGACGGGGATACCAAAACCGCAGCAACA

GCGAATGGCACCAACGGAGCGCGGGGTGCCACCGCTAGCAAGCAGAAGGCGGGTTGA

>g10001.t1

GGGAGCCTGGTGGCAACGCTACCCGCAGTCCTGGCGGACCTCTCGGGCAGTCTGGGGGAG

AAGGTGCGGAGGAATCTTCCAGGGTCAGAACTGGTGGGCAGTATATGGGAGGGAGTACGA

AGCCTCCGAGACGAGGGCGGAGTCACTGAAGAGGAGATGGGTGAAGTAGTGCCGCGGGGC

TGGAGGGATGGGGCATTCTACCCTGAGGAGGTCAGCGCATCCGGGAATTCGGATAGAGAG

GTGTTAGCAGCACACGACGCTGAGACCACCAACTCGCACAGAGCGCAGACTAAACTCGGA

AAACAGGTCAACCGGGTGCGGTGTGACAGGTATGTCACATCTCTGGACGGACTCCCCACC

CACGCACGCCCGCCGGAGGAGAATGGCCCTTTCGGGGAGAGGGAGACTCGCGAATGCGCA

AAAGCTAGGCAGCGGAGCCAGTCAGGACCAGGAGCTGCCGCGTGGCTGCGAGCAAGGCCG

GTGGACGCCCAACGTGTCATACCAGCCCAGGAGTTCCTGTACGCAGGGCGGCGACATCTG

GGGATCGAAGAGCACTTGGCGGCGACGTGCCCTGCATGTGGTACAGTGGAGGCCAACACG

CGACACGCTCGTTTGTGCCACCGCGCGGGCGCGCAGCCGGCATGGACTCGCTGCTTCAGT

GCCACatccgaggggggaggggaagggcttGACGTAGAGGGGGCCAAGGGGGCTCTCCGC

AGGGCACAGGCGGTTTGCTTCGACGTGGACAGCACCGTCATCGCCGAAGAGGGCATCGAC

GTGCTGGCCGACTTTTGCGGAGCAGCAGAAGCCGTCGCCGACCTCACTAGCAGAGCAATG

GGCGGGTCTATGCCCTTCCAGGACGCCCTGAAGGCTAGGCTGGACCTCATGACTCCATCC

AGGGGGGTGGTCGTTCGCTGTCTAGAGGAGCACCCGCCACGGCTATCACCCGGTATTTCG

GAGCTGGTTTCTCTGTTGCACAGCAGAGGGGTTGCGGTTTACCTAGTGTCTGGAGGCTTT

CGGCAGatGATTGAGCCTGTGGCGGACCAGCTGTCCATCCCTCGAGGCAACATATTCGCG

AACTCTATTCAGTTCGACGGCGTTACCGGGGAGTACACCGGCTTCGACGCGGAGGAACCT

ACATCTAGGGACGGCGGGAAGCCCAAGGTTATCGGCCTTCTGTCTAAGGAGTTCCAATAC

GACTGCGTCGTCATGGTAGGGGACGGGGCAACCGACATGCAGGCGAAGCCTCCGGCGCAG

GCGTTCATCGGGTATGGGGGAGTAACGATCCGCGAGGCGGTGCGAAAGGGCGCTGACTGG

TTCGTCACAGACTTCGGGCCCCTGATGGAGGTCCTAGGAGAAAGCGACAGCGCACAGCGA

GGCGAGAAGCGACAAGACCCgcaatag

>g10002.t1

ATGTCAGGGGTCCAGTTGCATTGGCGCCCCTTCCTGTGGCACACGTCGCAGCTCTCGTTG

ATTTTCGCGGGCACCTTTACGTACAAAAGCGCGCGGAAGAGAGTGTGTGGCGGCGGTATT

TCGGAGCTGGTGTCTCTGTTGCACAGCAGAGGGGTTGCGGTTTACCTAGTGTCTGGAGGC

TTCCGGCAGatgaTCGAGCCTGTGGCGGACCAGCTGTCCATCCCTCGAGGCAACATATTC

GCGAACTCCATTCAGTTCGACGGCGTTACCGGGGAGTACACCGGCTTCGACGCGGACGAG

CCTACATCTAGGGACGGCGGGAAGCCCAAGGTTATCGGCCTTCTGTCTAAGGAGTTCCAA

TACGACTGCGTCGTCATGGTAGGGGACGGGGCAACCGACATGCAGGCAAAGCCTCCGGCG

CAGGCGTTCATCGGGTATGGGGGAGTAACGATCCGCGAGGCGGTGCGAAAGGGCGCTGAC

TGGTTCGTCACAGACTTCGGGCCCCTGATGGAGGTCCTAGGAGAAAGCGACAGCGCACAG

CGAGGCGAGAAGCGATAA

>g10150.t1

ATGATCATGCGACGCTGCTCGAGCGAGCCGGCCGCCTCTCGCCGGCGACCCCTCTCCCAG

GGAGAGTGCTCCCGCGCGCGAGGTACAACAACCGGGGGTAGCCGACGGTGTACTCTCTCG

GCGGCGCAGACCTCGCCCTCTTTTTTCGGACGCGCCTGTGCCGACACCGACGGCCCCTCT

GCCCCGGGAGGGACCACGCTGACCGCCCCGCGACCATGGACGgcgtctcagcagcagcag

cagcagcagcagcactgctgctccTCTGAGCTTCgacgccactacagcagcactctCTCC

TGTCGCTCTGAGACGACAATGGCAGCTGTAGTACCCGGCGCTGGTGATGCTGTTGTGGAC

ACATGGCCGCCTCGACACGTTCGACGGCACAGCCAGACCTTGGAGCGGGTTGAACCGGGA

GGGGGCTCAGCACCGGAGGCCCAacaagcggaggaggaggagatggtcGTTCGAGGTGAA

GCGCTTAGTACCACTCCGGCTTCGGGACCACCACCTGCGCTTCCGGTTGTTGTAGCGGAT

GAGCAGCTGCCTGCTGTCCCCGGACGAGGTGGAGGGCctgtgcagcagcaacgcctcttg

ggcggggaggggcacTTCGCGACGATCATGCAGGATCTGCAGCAGCTGAGATGTAGCATG

TCGGAGCCGATGGCCGCGGCGACAATCCCGTCTTCGTCGACCCCTGAAGACGAGGAGCCC

TCCAAGGCCGATCTCGAGACCGTTTCCAAGACCGCTCCCAAGACCGTTCCTGGGACCTGC

GTGGTGTGCTTCTCAGACGGCGAAGACGGTGTCTTGGACGCCGATTGGCTTCACCAGGAG

TTGGTGTCCGCGGTGGCTGTttcggagggggtggagggggaggggacgggggtcgGGGTG

GCGTCGATGAGCGTCGGGAAAGGCACTGGGTGCCCGGCTGCTTCGTGTGTCTCTTGCCTC

AAGACGTACTTCGAGGGCATCGTCACCTCGGGATTTGACGGCGCCTGCCCGCGAATGCGA

TGCATGTGTTGCCCTCGCACGGTGTGTGAGCCGGCATGGGCACCGCTCGTGGACACCAGC

GTGCTCGAAACGTTCAGAGATCGGGCGACGACCCTGCTCTCTCTCCAGTGCGGTTCCTGC

CACAGCCGAGGGAGCGCCATGGTCGTCCCACCGCCCGATCACTCGTACAGcTCGGCGCTG

AACGCCTTGATGACCGACATCGCCACCAACGTCACCAAATCTCGCCATAACAACGACAAG

ACAGCCACCAcggaagggaagaaagggcccgACCCTACCGACACCAGCGCTAACGAGGCG

TTTCGCTCAGCGTTGGCTCGGTACGCGTCCGGAGAGGAGGCGGACGTGGTGAAGTTTTAT

GATGAGGTGGAGGCGGTTATCTCCCTGGCGCTGGTAGCCGGGGAGGGTCAGGACGAGGCC

CGGTACGCGCGTATGGTGTCCGTTCTGAGCTGTGTGGAAGACAGCGAGCGCCGGGCCAGC

CTCCACATCCGCTTCCTCCGCTTCCACCCCATGATGGAGACGACGTGCTGCAAAGCCTTG

CACTGCTTCCGATGCCGCGTCATGGACGGCCACCCCGGGCTAACCTGCGAGCAGTACGAG

GCGAGCAGGGGGCACCAAGACGTCTGCCAGTGCCCTGGATGTGGAGTGTACGTTGTCAAG

GGCGACGGGTGCAACAGCATCACGTGTGTCTGGTGCGACAGCATCACGTGCGTCTGCGGG

GAAGGGTTCAGCTGGACCTCGTTGATGCGGGGCAGGAACGTCGAGGCGGCGGGGGCCTTC

CGCGCCGCTCACCCCGGTGACCCCGGCCGAGCCGCCGCCGAGGTCGTCCGGCGCGGCCTG

CCTGAGGACGATgcgcagactgctgctgctgctgccactacggacggaaacgctgctgct

gccgacgaaGACGAATTGCTGGATGCTGCGGCTTCTATTGACGACgagctacaacagcaa

cagcgacagcggcaggaggaggaggaggaggaggaggcggagcaaCTCGACGACTCCCCA

GACGTCAACCTCCCCTCGACCAACGACCACACCACCCTCTCGGATGGCTTCAGCCCGCTG

CTGGACGATGGTGAGGATCTGTGGCACGAGGACACGAGCCACCAGAACCAGGGGTTGGGG

GAGAGCCTATCCAATGAGGTTCGAGCCCTCGCGGACGAGAACGCCTCATCagcgaccacc

cggggacgcgaGGTTCAAGGCCCAACGGACACATCAGGACGAGATGCTGCAGTGCCGTTG

GAATCGGAGCCCGAAACGACACCCGTGCCGATGCCGGTAACGCCTGCGGAGGAGTTGCGC

CTGGCGAATCTGTTCGCGGAGATTTATCCGGGAGAGGTGTGCCTGGCGCGGTCGATGATG

TTCGAGGAGGAGCACCCTCAGTTCACGGCGCAGTGGGCCACTGCGCGGCTCGAGGGGAGG

TCCTGGGGGGACAGCATGCCGGACCCCCTGGACTACTTTGGCCGGTCCGGCCCCGTCTCC

GCTGAGAACGTAAGCGAGGCCGAGCGACGCTGGAGAGAGGACTCCGCACAAAACTGGAGG

GCGTGCAACGTGtcAGCTGTGGACGAGGCATTGCGCCAAGACGCGAACGCACGTGCGCGC

GTAGTCGAGGCTCTCTACGGCGACGACCGCAGCGAGAGGAGCACCCTCCTTCCCTACGTC

GCGGCGGAACACGCCCGCGCCGCCCTCCGCATCGTCCGCCCCGCTCGGGCGGAAGACGCC

TTGTTCTCTTGGCTCGGGATAGAAGAACCCGAGGAGGAGGACACCGCTAGCGCtagcacc

aacatcaacaccaacacccacgccAGCGGCCACGCTAGTGATGGTGGAGACCTAGTAGAG

GGACGGGGCGTTAATGGAGGCGGTAGatggggaggggacggggacaACCTATCCCCTCCC

Tcgcaagacaacaacaacgacaacggcaacgtaggcgaggaaaaagaggaggaggaggag

gaggaggaggaggaggacgtgtGGGAGTCTCCGTTCGCGCAAGCGATCATGGAGGTGGAG

GAGCCCCCTCCCCTACCGGCGCCGGGGGCCATGTGGATACCGATGCCCCATAGGCGGTTT

AACCCCCCGCCTGTGACGTGCGCGCCGGACCCCTCGGTGAGGCGGGTGTCGCTCTTGTCG

GAGTTAGCTTCGATGTCGACGGCGACGTGGCGGCTGGAGCGGGACGCTAGGGAGTGGGTG

GCGGGATCGACAGCGCGGCAGGAGAAGCTCCGAAGCCTCATGGGTCGGGAGGAAGGCCGG

CTGTCAGAAGCATGGGTGGCGCTCTGGGGGCGACCCTACGGCGGCCAGTACCAACACCAA

GGCGAGCAGGCTGCTGTGCGGGCAGCGGCAAGGCGCTGCGTGTTTCTGGAGGCCTACCCG

GAGCAAGCGTGCCCTGAGCAAGGCCCATCGATGAAGGCGTTCGTGCGCCGAAACTACGGA

GCCGTCGAGCAAGCCCGAGCGGAAGCCGCGGCCCAGCGCGCCGCCGCGTGGGAGATGATG

CACGACCACCAGCCTGagggtggtgatgttggtggtggccgtggtggtgagggtggtgaa

GGTGGGGGAGAGGGTGATGGTGAGGATGGTGAGGGCTGGGACTGGGGGTGGGTTTGGATT

TGGGCTTGGGTTTGGATTTGGGCTTTGGAGTGGCTTACGGGGTgggatgttgttggtggt

ggcgatgttAACGGCAGCGGTGAGGAAGAGAGTAGCGCGAGTGGTTCCGCCGCCACGGCA

GCCGCCGCAGCTTGCCCCTACCCCACCTCTAAATTCGAGCGCGCGCACGCCGCCTATACC

TCGTACCTCCAAGAACAAAGCCGCCTTCgtggctcctcctcctcctcctcctcctcctgc

actTCACGCTGCACCACGGACGCGGACAACAGCTACTTGGACCGCTTGGCCGAAGCGTGG

GAGAAGACAAACCGGCCCCGAATCGCCGCCAAGCGATTCGAGGACGCTCACTTGGTTGGG

GAAGGGGGTGCGTCAaaccctgcagcagcagcagcagcggcggcgacggcggcggttgtG

GCTCTGGTCATTGTGCCCATGCTGGTAGAGCGGACGGTGTTGATGTGCGATTGCGAGCTG

GGTCTCATCGCAGACGACGCGGCGGAGTGGGCCAACCTGAACGCGGGTAGGTTCGAGAGG

GAGTTCGAGAGgatggaggcggcggcagcggcggcggcgggatgtgatgcgggggggggc

gggggcggggcgggacggatGCTTGCGGGGTGCGCGTGCGAAACGGATGGCGCCTGCAGC

CGCCTGGTGGCCTGCCCCGTGGCTACCCGCCGGCTGCTAAAGGTATCCAATGTGGTCTGC

AGCTGCGATTCCGAAGACGCGTTTGATGACGACGACGGTAGCTACCACTTGGGAATGTTT

TGGTGA

>g10227.t1

ATGGGAAAGTCTTGGGCGCTCTGCGCTTTGATGGCGCTGGGCTCGCTGTCTTCGGCCAGG

TCCCTCTGCGATGACGTGCAAACGTACTTAGACATACCAGTACCGCTCGGTGGTGACATG

GAGGTCGAGCTCGACGTGACGAGCCTCGAATGCCCACCGAACGGAGAACTCCCTGACGAG

CTGGACGAGGTGATAGTTCAAGGCGGGACCCTCACGATCAGGGGTACCCAGAGCTCTCTT

CAGTTTGTGAATTTCCGGTTCACGGTCGAGGACGGGGCTTCACTCGTGTTCGACCTCGAC

ACCGTGCTCTTCGGTCCCAACTCAGGGTACGAAGAGAATGCTCAGGGGTACATGATCGAT

GTCAAAGAAGGCGGGAGTGTTACCTTCCTCGGTGCCTTCGAAGCGAACGCGGTGCGGAAC

GTCAAGTCCACCTTTAACAACGCCGGGAACATGGAGTTCAAGGGCACCGCCCTGTTCGAC

GGAAATGCTAACGTTTTCCAGTTCAACAGAGGCGTTGTAAaATTCCGCGAAGTGACGACC

TTCACGGGCACCTGGTACAACGCCATTTACAATCTGGACGCTGGATATGTCAGGTTTTCC

AAGAAGGCTTTGTTCACGAACAACAACAGGTTTTTCGTGGGATCGAGCGCCTGCGCCATC

CACAACCCCACGTTGGACGGCCGGATCATCTTCCGTGGTGACGCAACCTTCGCCGAGCAC

AGCTGCGATGGTCCTGGGGCGATCAGCTCTTCTGGGACAGTCATATTCTATGGGAAGGCG

TCCTTCATTGACAACCAGAGCGACAGCAACGGTGGAGCCGTGCGGAACCGCGTGGGATCC

ATGAGCTTCAAGGCAGCAGTACAATTCGTCGGCAACACGGGGAACTCTGGTGGCGCTCTC

GCAGTGGAAGGCGGCGATGTCACGTTCGACAGGGCCGTGCTGTTCGATGGAAACGTGGCG

AAAGAAACAGGGGGCGCCTTCATCGCCACGTCCGGCAGCGTATTCGACGGCGGAGAGGCA

ATCGCCAGCGGTGACGCGCTGTTGACGTTCAGGAAGTCCAGCGTTGTTAGGGTCAGGAAC

AACTCTATCGCTCCGGAAGAATTCGGCGACGAGGTGCTTGCGCCCCTGTGCACGGTTGGC

TACGTGGAAAATGGTTCCACCGTGGTCGGCTACCCCGAAGACGACATCTGCGTCGAAGTC

TAA

>g10321.t1

ATGCAGGTGTATGGGTGGGATACAAGGGGCATGATCGTGTTCGACGACGTTGCGTGCACA

AACAACACGGCGTTGAATAACGGCGGCAGCTTCTACGCTTTAGGAAGGGCCGCTGCCAGC

AACGGAACGGTTATGCACGACAACGTGGCACGAAATGGAGGCTGTATATACGCGGACGAA

AGAAGCGAAATCGACGTATATGGCGGAGACTTCATGTGCCGAGGCACCGAAAGAGCCGGG

TTTCTTTTCGCCGCGCACGAGGCGCGTGTGAAGATCACCGGGGGCCTATTTGCGAAAAAC

GTGGCGGTCAGGAGAGGCGGCGTGCTGTACTGCGAGGGACCGAGGTCATCGGTAACGATC

GAGGGGGGAACCTTCAGGGACAACAGGGGTCTGGAAGTGGGAGGCGCGATCGTGGCGTGG

GGCGATTCCACTGTCGTGAACATTACTGGGGGGCAATTCACCAATAACACCGCAAAATTC

TACGGCGGCTTCGCTTTTCTAGAGGAAAAGGCTAGCCTCAATTGCAAGGGAGCCGCCATC

AAAGGAAACGCCGCCGGCGACCAAGGCGGGGGGATCTATGCACGAGAAGCCACGACGATA

ACCTCCAGCTGCGACTTGATAGCCAACGATTCTCCCCAGGGAGCCGCCCTCTACTTGACT

TACGTGGAGAGCGCTACATTCGAAAACCACGAAGTCGCCGCCAACGTGGCTTCGGGCGGC

AGCGTGGTGTACCTGGCCGCGAGCTCCGTCGTCGCCAAAGGCGTGACGTTCGAAGCCAGC

GTTGACCATCAGGACGACTCGTCCAACCGCGCGATACAGACGGTCGGCAACACGACGTTG

AGAGCGGAGCAGTGCGCGTTCGAGGACTGGGCGGGCGACACGGTTATCTATCACACGAAC

TGGATGAATGGTACGCTTGTCCTTGACAGCTGTGAATTTAAAAGGAGCTCCGCAACCATG

GTGGTCACCTCGCCCAACTCAGCCGCCGAGATCCGTAACGCTGTTGTCGACGACCTCACG

TTCGCGAACGCCAACGTCGGCCCGCTCAATAGCTCGATTTCGCTTGTCGACCGGGCTTTG

GACTGCGGCGATCCAGGCGCGTGCGGAGCAGGAGCGTGCGTGAACAGTGCACTCGGGGTG

CTGTGCGAGTGCATCGAGGGCGGTGAATGTCtggacggcggcggggggctATCGCTCAGT

CTCAAAACGTCACCGGAGCCAGAGACCTTCAGCCCCGATCTTGTGTCGTATACACTCGAG

GTCTCCTCAGCGGAGGGTGGGACCACGTACTCCATTTGGGACCTGAAGGCCGAGGAGCAT

GCCTTGACCTTGGACATACTTCCATCCAACGGGGTATTGCCTCCTGGAGGTATCGCCACA

GTCACAGTTAGAGGTACCTCCATGAACCAAGACGTCCAGGGGAATCTGACCAGCAGTTTC

GTCGTGACATCCGCCAGTGGCGCCGGCTTGGACTCGACAAACGGAGTCAAACTGGCGGTT

AGCTCGACCTTCTACTTTTGCTACGCTCACGAGTACGCCATGCCGCGGGATGATAACGAC

GGTGCTGACGTCCCATGCGAACAATGCGCTACCGTCGACGGCGAGGAAGGCGTAGACTGC

AACAACCCAGGAGTGACGTTGGCCTCGCTGCCCATTCGGAAAGGATACTGGCGGTCAAGC

CGAAAATCGTTGAAAGTCCAACAATGCTTGCACTCGGAGGCGTGCGTAGGGGCGACAGAA

ATAGCCAGTTCGGACGACTATTGCGCAAACGGGTACAAGGGTCCATATTGCGCCGTGTGC

ACCCAAGGGTACGGAAGAGGTGTCAGCAACAGTTGCCACTACTGTGACGATGCCACGGCC

CACCTGCTCACCGCGGCGGGCACATTCTTCTGCTTGGcggtggttctgctgctgcttttc

gcGGCGGTATTCCTCGTCGGAGGGCTCGACGCCATCGACATCGTTCGCCATTCCGTCGTT

CGGACAATTCCTTTCGCGAGCAACACATCCAGCAAGAATCCCAGTGTACCGGAGGCCAGC

CGGCAAGAACGGGCCGGTTCCGAGCGGGGTGGCAGAATGGACTCATTGGACGGCACCGTT

GCTCCTACGTATGAAGCGGCGGTAGCGCGTAAGCCAGacgctggcggcggcggtggcacc

aGTCTGGACGGAGTGTTCACACTGAACTATTCTGCGTCAAACGACGGCGAGCTTCCGTAC

AACCGGCGCCTTGGCGCCAATGCCTGGGTCGGGACTGGCGTTGACCTGTCAGGCGTGACT

GAAAGAAACTCTGCTTGGAATCTGGGGGTGCCTGCCCATCAAAATACTCGGTCATCTGCC

GTCGCTGGCACAAACGAAAGGGGATCACACGACAACAGGGAGCTAGAAGAGGCGGCGACC

AGTGGAAGCTGGCGGTCAAAGTGCTGCGGTCTCGGCAAAATGGTTCGACGATGGCTGCCA

AGGCTGCCACTGAACAAGCTCAAGATcctgctggtggtgtggcagATCCTCGCCGTTTTC

TCGAGCATCACCGGGGTGGAGTTTCCGGCCTCCTACTCGACATTTCTGTCTTGGATCAGC

GTCGTGAATCTCGACCTCGGGAACATCTTCTCGGCCTCGTGCGTCCTCCACTCCGTTAAC

TTCTACGTCAGGCTGCTGATTACGACGCTCGGGCCTCTTGTGTTGATTTGCGGTCTGATA

CTCACGTACCACTTGGCGAAGCGTCTAGCGGGCATCGGATCGGCCGGGGTAATTGCAAGG

AGGGCTGCATGGTCAAGGCATGTGGCGGCAGGGCTGCTTCTCTTGTTCTTGGTGTTCACC

TCGGTCTCTACGGCGGTCTTCAAGACCTTCGCTTGCGACGACGACGTTGTGATCGGGGAA

AGCTACCTCAGGGCGGACTACAGCATATCGTGTAACACGAGAGTGCACACCTTCTTCAAA

ATTTACGCGGGGTTCATGATACTGGTGTATCCGTTCGGCATCCCCCTCCTCTACGCCGCC

ATCCTCTGGAGCAAACGGGACTTGCTCAACCCTCGCCTCTCCCCCGACGCCATACACGAG

TCCGATGGAGATGGTGAGGCAGCTGCGAGAGCGACCTCACCGAGAGACAGCAGTAGGCCC

ACTTGCAAACGAAATATCACTATCGTGGGTCCGACCAAGAGTAAACTGACCCCTCAGGAC

CTGCACGAACTGGACAAAAAAGTGAAGGCACGCAGGGAGCATCCGGACCTAGTCCCCTCC

ATGTTCCTGTGGAAGGATTTCGGCCCCGActtgtactactacgaggtggtCGAGTGCGGT

CGGAGGGTTCTTTTGACAGGAGTGCTGATTTTCATATCGCCGTCCAGCGCGACACAAGCG

GCCATGGCATGCATCTTTGCCTTCGCGAGCCTGCTGGGTTTCGAGCTCATGCGGCCCCAT

CTGGACCCCGCAGACTCGTGGCTCTACCGACTGGGCTGCGTGATCATCTTCTTGAGCAAT

TTCCTGGCGCTAATGATAAAGGTGGACGCCGGAGAAGAGGGCGACCGTGCCGCCTTCGGA

GTCCTTCTAGTCGCCGTCAACGTGTTCCTGGTCATCGCCGTTATTGTGTCATCCTGGTTC

GCCACGCAGCAGTCGGTGGACGACTCCCGTGAGGAAGAAAACGCGCTCACGATAGCGAAG

ACCATGCTGACCGCCCAGCAACACACTGCTAGCAACGCCCGCCTCACGCGCAGTGACAGG

GCGCCGATGTCATCGGCGTCTTCCTCCGTCAGACCTCACCTCCCGCAGTCCGACACCGGC

GCAGTTTGGCGCGATTTTATGGAATCGCGTGGGCACAGAAATAGCGTGACGGACGCGATC

GGAAGCGAGGATTCCTTGCTGATTTTGACTCGCGATGGGAGGGTCAGCTCTGCCGTGGTA

GGGCCTTGGCAACACGACGGCGCCAGTGACTCGCCCCCGTTCAGAAATGGCATGGTTTGA

>g10745.t1

ATGGCCTGCAGGCAGCGCAGCGGCGACACTATAATGATGTGGTCGGGGGTCTTGGCGAGT

CTGCTCTTCGTCGGAGCCGAATCCGGCCGCTCAAAGCCGGACGTGATTCAGCTGCCCGTC

GGGTTCTTTCCGGAGGGAATCACGATCGCCAAGAAATCTACCGCCTATGTTGGCTCCCTC

GTCGATGGGTCGATTTGGAAGGGAGACATTAAAACCGGCAAAGGACGTGTCGTCATTTCG

GACGTCGGTGGCCAATCGATCGGCCTGGATCATGACGGCCGGTCGGGCTACCTCTTTGTT

GCTGGTGGGAGTGCAGCTCGAGTCTACGATGACGACGACTTCAGCCTCGTGGCAGAGTTT

GTGTTTGCCGGCGAGGGTGAGATATCGGTCATAAACGACGTGTACGTCACCAAGACCGCG

GCATACTTCACAGACTCCTTCCAGAACAAGCTGTTTCAGGTCCCGCTCGACGCGGATAGT

GGGGAGCTGGTGGATGTGGTGGTCACCGCCGCCAACACGATAACGCTTAGTGAGGAGTTC

GGCATCGTAGAGGGGGCGTTCAACGCGAATGGCATTGTGGCAAACGACGACGGCAGCATC

CTCATCGTGGCGAACACGAATGCTGGTCAAATCTTTACCGTCGATCCCGCGACCGGCACC

GCTTCTCTGATAGACCTGGGAGGCGTTCTTGTCTATGGGGATGGTCTGGTTCTGCGCGAC

AACACGCTTTGGGCTGTCGACAATGGTCAAAACGTCGGCGGGATACAGCAAGTCAGAGAG

ATCTCCCTCTCGGCAGACCTGACGTGCGGCTCAGTCGTGCCCCGCGCTCTAACGGATGAC

CTGTTCGACACGCCAACTACTGCGGCACGAAAGGGCAACTCGCTGTACGTCGTCAACGCC

AAGTTCGGCGTTGCCCCCGAGGACGTACCGACGACCGAGTACGAGATTATTCGGGTAGAC

CGGGACAGTCGCTTTTCGCTCTTCCTCGCCTGGGCTCGACTGCCGCCGTATCTGAAGGAG

ATCGCCTTCGAATTACATTACCCCGGGTGGGCGGCCTCGGTTATCGCACAGGTGGTTGAT

CTGCTCTGCGAGGTTCCTGGCGAAGCGGCAAGGGCAGGGGGCACGGAAGTTGTCGGGGCG

GACCGTGGCATGGAATCCGCGGCGGGATGTGAGCACGCCGGGCAGGTGGGCGAGGGGCCT

CGCTGGGCCTAA

>g10761.t1

ATGGGATCAGATGGGACTGCCAGTCCTACAGAGCGAGACGGCTCTAACGCCAAGAAGCGT

TTCAGCCAGACAGACGCAGCGAACTTGGAAGCCTTAACCAACCTCGAGGCGGGAGTGCTG

AAGCTGGAGAGGGAGGGACTCGTCTGCGCGGGTGGTCCGTACTCGCCCGGAGGAAACGAG

ATCGAGGCTAGGATGCCGCTGCGGTCGCGCTTGCAAGAGCTGGCGACGTTTGTTGCCTGG

TTCGCTCTCATCGCTTCTTGCACGATGCAGTACCCGATGGTCTGGGTGGCCTTTTGGAAT

CACATCGAGGCGCTGCGAGTTCACATGTACTCGTTCCACCGGAGAAGCTTCTCGCTGCCA

CTGTGGGGGCTGTGGGTGGCTCTCCTCCTGGGCTACCGCCGTATGATCTATCGCCGAATC

ACTCCGGACTTGGGCGCGCTCTTTCTCCACACAGAGGTCTTCGCTTGTGTGGTGCTGTGC

ATGACCGTGACGGTCAACTTTGCCACCTTCGTGCACACCCCTGGAGAGCCGCTGTTCGAC

ATCGGCTTCCTGGTCATCCCCGAGCAGGAGCTCAACTCGCCATGGCGGCCTGTCAGCGAC

ACGTTGACGGCGCTCCTGCCGGGCATCGCGCTCCTGAGGAGCCTGTTCATGGACCGCAAG

CAGCGCGTGACTCTGATAACCTCCTGGTTCCGGCTCGTGTCGATCGTGTACATGCTGCGG

TGTCTGACGATCGGGTTGACGTCGATGCCTGGGCCGGCCCCGCACTGCCGGGATAAGGTG

CTCTACAACCCGCCGACCAACTGGCACGAGATCGCGACGAGAATGGGCGTCATGGTTGGA

GATTTCAGCTCTTGCGGAGACTTGCTGTTCTCCGGCCACGCTGCTTTCACGACGTTGACG

ATGTTGGTTTTCGTAAAGTCTTGGAGGGGACACGCGACGTACCGCGTGTGGAAGGTGCTT

GGGGTGGTGTACCTGCTGACGATGTGCACGCTGGCGATAGCGGGGCGAAAACACTACACG

GTGGACATTACGCTAGGGATCCTGATCGCGAGCTTAACGTTCTTCCGCTTCGAGAATGGG

TGGAGAGCTTCGTTGAATGGGGGGGCGGCTAGCTCCAGCGCGACCGCAGCGGTAAAGGCC

GCGTACGATCCGCGCACAGAAAAGAAAAACGCGCCGTGGGTAGTCTGA

>g10816.t1

ATGGCTGGCACGCTAGAAGGAAAGGTGGCGCTCGTGACTGGGGCGTCGACAGGCATCGGG

AAGGCTACAGCGATCGCCTTCGCCAAGGAAGGGGCAGAGGTGGTATTGTCAGCCAGAACA

GAGGACAAGCTACAGGCCGTCGCCGAGGAGATCAACTCTGCGGGGGGGGAAGCCGTCGTT

GTGGTGGGCGACGTTTCAAAGGAAGACGATTGCAAGCGTATGGTGGATGCAGCGGTGGAG

AAGTTCGGAGGGTTGCACGTGGCCTTCAACAACGCGGGAACCATCGAGGCGGGCACATTC

GCCGACATCACCGATGAGCAGACGAGCAAGATGGTGGACGTGAACTTCAAGTCGCTCGTC

TTCTgcttcaagtaccagATTCCTGCTATGGCGAAGTGCGGGGACAAGGGCTCGATCATC

GTGAACACCTCAAGTGCTGGGTCGCGTATTCCCAGTCTTCCGGAGATGCACGGTATCGGC

GTGTACGCAGCCACGAAGGCGGGCGCCGACATGCTCATGAAGTACGCCGCGATCGAGGGT

GCTGAGTTTGGAGTGCGCGTTAACTCCGTGGCACCGGGGCACGTGGAAACACCCCTTCTG

GCGATCATACCTCGCGAACTCATCAACATGAGCGTTCAAAGCTCGCAGCTCATCAAACGC

GTCACCGAACCCGAGGAGGTTGCCAAGTTGGTGATGTTCCTGGCCTCCGACGACGCTGCG

ATGGTCACCGGCTCCGTGTATATCATGGATGGCGGCTGGTCCATTAAGGCCTAA

>g10818.t1

ATGTCTGCTGGAAAGCTTCAAGGGAAGGTCGCGCTCGTGACTGGAGCGTCGACAGGCATC

GGGAGGGCTTCGGCGATCGCCTTCGCCAAGGAGGGAGCCAAGGTTGTATTGTCAGCCAGG

ACAGAGGAGAAGCTGCAGGCCGTTGCCGATGAGATCACGTCTGCGGGGGGCGAAGCCCTC

GTTGTGGCAGGCGATGTTTCCAAGGAAGCCGACTGCAAGCGCATGGTGGATGCCGCGGTG

GAGAAGTTCGGAGGGCTTCACGTGGCcttcaacaacgcggggacCTTCGCGGTGGGCACG

TTCGCCGACATCACGGAGGAGTCGATGAGCAAGATGCTGGACGTCAACTTCAAGTCCCTC

GTCTTCTGCTTCAAGTACCAGATCCCTGCTATGGCGAAGAGCGGCGACAAGGGCTCTATT

GTCGTCAACACCTCGTGCACGGCGTCGCGCGTGTCCACCCTCCCTGCCATGAACGGCGGG

GGCGTGTACGCGGCCACTAAGGCGGGCGCCGACATGCTCATGAAGTATGCCGCGATCGAG

GGTGCTGCGTCTGGGGTGCGCGTTAACTCCGTCGCGCCTGGGCACGTGGAGACGCCCATC

TACGGCGACATGCCTCTCGAGATGCTGACCAACGTGGCTAAATCTACCCAGCTCATCGGA

CGTCCCATCCAATCGGACGAGATCGCCAAGGTGGTGACGTTCCTCGCCTCCGACGATGCT

GCGATGGTCACCGGCTCCGTTTACCTCATGGATGGCGGCTGGTCCATCAAGGCCTAA

>g10951.t1

ATGTTGAGAGCTTCTCGCGGCTCGCTCCGTTTAACcTTTGTTCCCGGTTgccaccccaac

atcgtggACTACGTGTCGTGCAGCTTCGACACGCTCGCCATCGTCATGGTGGCCGAGTCT

TGCGACCTTCAGGAGCTCCTGTCTTACAAGCTCCCCTTGGAGTCCATGGGTAGAGGTGAA

ACCATGAGGTTCTTCGCTGGGGGCGTGAAAGGCTTGATGTACCTCCACTCTCGAGGCTAC

ACCCACATGGATGTGAAACCGGCGAACTTCATGACCGACAAGGCGATGAGCAGGCTCAAA

GTCGGCGACCTGGGCTTCGCGGGACGGTCGCACGAGGCCGTTTCGAAGGGCGGAACGAAG

GAGTACATGGCGCCGGAAACCATGGCCCACTACCACGACCCCGACACCCCGCGCGCGACG

CTGGACCCGAGCATCGACGCGTGGTCCGTCGGGATAATGTTGCTGCAAGTCCTGTTTCTT

CAGGAGCTCCCCCGCACAGACCCCAACGAGCTGGCGATCCACGGTCTCATGCCGAAGCAG

GAGGCCTTTTCGTCATCCCTTGGGAACCTCGAGTTCCTGAGGCGCAAAGCGGAACAGACT

GGCTCGGCTAGCGACCAGGGGCGAATGGCCGCGGCCGAATCCGTTCACACCAAGCTGCTG

ACGGAGCATGGCATGCAGCAGGCCAAGATGCAAGACCTAACGACGACTCTCCTGTGTCGG

GGGAAGTGGGAAGAGCCGGGGGGGCTGCGGTTTGAAAGCGAGGTGACAATGCTCCTTCTG

AGCCTCCTTGATCCGAACCCGCGGCGGCGCCTCCCCCTCTCCGAGCTGTTGGGGCTGCTG

GAGTGCGACGCCCTGTGGGCGGGTGCCGACGACAACCTGGGGGAACTCCCGGAACCGGTG

CGGCGTTTTTTCGCGAAGATGGACGAGAAGAGCGTCCTGAGCCCCGCTCACGCGTCTCGC

GGGGCGTGGTGGCCGAGCAACCCGTGGTGGATGGCCCCGCCTCCGGTGTCGCTCATCCCG

GAGCTTCGGTAG

>g11041.t1

ATGGGGAGCACGGCCGGAGACGATGTCttggctgctggtactgctgcgtcCAGCTCTCCC

TACCAAGTGCCCACCATAGGCGAAGCCGTGGATGGCGGGGGGGATGGGGACGGCGAGTTC

GAAGGAGTGGATGAGATAAAGGGGTCCCTGTGTATGTCCTGTGGGGGCGACGGCACGACG

CGGATGCTGACCACCAAGATACCCTTGTTTCGAGAGGTCATCCTGAGCTCGTTTGAGTGC

GACGACTGCCACTGGAGGAACAACGAGGTGACATTCGGGGGGGAGCTGCAGGAGAAAGGG

TGCCGCTTCGACCTCAAGGTCAACAACGCGGAAGACCTCAACAGGCAGGTGATCAAGTCC

GACTTCGCGACGGTAAACTTCCAGGAATTGGAGTTCGAGATTCCGCCGCAGTCGCGGGGG

GAGGTGACCACGGTGGAGGGGCTGCTCAGGACGTCTGCCGAACGACTCGGCGAGGCCCAG

GACCTTCGCATGGAAAGATCGCCagagGTCGGAGCGCAGATTGCCGGGGTGATCGCGCGA

CTGGCCCTCATGAGCGCGGGGGTTGACTCGGAATTCCCCTTCACCATGGTGGTTGACGAC

CCTTCGGGCAACTCTTTCGTCGAGAACCCCTCAGCGCCGAACAAGGACCCCGCTTTGACg

ACGGCGTACTACGTCCGAACAGCGACGCAGGACATGTCGTTGGGTTTGCAGCCGTCGCAG

GAGGCGATCGAGTCGGGGACGGTGGACGCCACCTCAAAGGCGAGGCCGGCGCCGCACCAG

ATGGAGGGCTCGGCCGCGCTGATGGCCAGGACGACGACGATGCCATCTCAgACGGCGTAC

TACGCGAACAGCGAGCGCAGGATCATGTCTCGTTGGGTTTGGCAGCCGTCGCAGGAGGCG

ATCGAGTCGGGACGGTGGACGCACACCCAAAGCGAGGCCGCGCCGCACCAGATGGAGGGC

TCGCCGCGAAGCTGA

>g11088.t1

ATGGACGACTGGGGGTCGGCGTGGCAGGACGCTAAAGACAAGGGCGAGGCGTGGGGCGGG

CGCGGGAAGGGGGGACGGGGCGTCCAGAGGAACACCTGGCAGCCGAAAGACGTCTACGTT

GAGGGCGTGACGCTTGCGTATCAGGGAACCGAGCTGCTGCACAGGACCACCCTGAGGCTC

GGTCACGGCCGTCGGTATGGTCTTGTCGGCGCCAACGGGGTGGGGAAGACTACGCTGCTG

AGGCGTATCGCAGCCGGGGCTGTCCCTGGGTGGCCCCTGCACCTGCGGAGCTACCTGGTG

CAGCAGGAGGAGATGGCAGGGAGTCCTGACTGCAACGCCCTGGCGACGGTGATCGCCTCC

GACGCGCGACTAGCCGCTCTTCAGCAGGAAGCCGAACTTCTCGAAGAGGACGCAGAGGGT

CCTTCCACCGGCCCGACGCACGACGCCGCCGGCGAGGAAACCCCGCACCTTCCCGGCCGC

CGCGAGGGGGGGGATGATTCGGGGTCATCCCCTCGTCCACCGGTACCGCCTCCTTCTTCT

TCGCCCTTGGCGTCCATGGCGGCCTTGCGGCAGGAGATCGCGGAGCTTTCGTTGGAAGAG

AAGGTGGAGAGGCTGGAGGAGGTGTACGAGGGCCTGGATATGCTGGGCGCACACGACGCC

GAAGGACGCGCGACGAGCATCCTGAAGGGGCTAGGGTTCAGCGAGGCGCGCATGTCGCGC

CCTACCTCCGAGCTTAGCGGGGGCTGGATGACGAGAGTTTCTCTGGCCTGCGCCTTGTTC

TGCAAACCAGACCTCCTCCTGCTGGACGAGCCCACGAACCACCTCGACCTGGAGGGGGTG

CTCTGGCTGTCGAGGCAACTCGTGGATGGGATCGGGGATACCATGGTGCTCATCGTCTCG

CACGACGCGGCTTTCCTTGACGCCGTCTCGACCGACATGATCCACTTCCGCCTCAAGGCG

CTCACCTACTACCCAGGGAACTACTCCGCCTTCATGAAGACGAAGTCGGACCACGACGTA

GCCGCGAACCGCACCCAGGGCAACCTCGACAGGCAGCGGAAGCACATCGAAGAGTCCGTG

AAAAAGATGACCAAGGCGGCCAGCGGGAAGGGAGGAGACCAGAAAAAGTCGTCCCAGGTG

GCGTCTCGGACAAAGAAGCTGCAGCGTCACGGTTTGGAGAGAGACGCCAACGGGCACAGG

TGGAAGGCGCAGACGGTGGCGAACACGGGCAGCTCCATTCGCGCGGGCTGTGCGAACGAG

CTATCGTACAGGGGAGGGAAAACCGCCCGGAGAAGTTTGGCCGCTCACGTGGAGAAAGAA

ACGATGTTCCGTTTCCCGGACGTTGCCCCCCTCCCTTGCGCCATGGACGCGACCATCGTT

CAATGCCGGGGAGTCCACTTCTccttccccgcccctcccccctcacaaGCGGCGGCAGCG

CCGCCACCGGCCTCCGCCAGAGGAAATCGAACTCCCGGGGCCGCATTCCCCCTCCCCCAA

GCATCGCTAGCGTGTTCTACGAAGGGTGCCAGTAGTACCGCCCGTTCTGGTCAAAGCGCG

GCGGGCCGTAACAAACCCAATAACGGCGCGCGTTCTTCCCAAACGGTGACGCCCCCCAGC

AATCCCGCCGGTAGTGGTGGGGAACTCCTGGAAGGCGTTACGCTCGACCTCACCCGCAAG

AgccgggtggtgttggtggggcgAAACGGGTCGGGGAAGAGCACCCTCTTGAAGCTCATC

GCTGCGGCGACGGGAGTTGATGGAGAGGCGGGAGGCGGTGCGGCGGTGGACGCCCTCGTG

CCCACGAAGGGGAGCATCGTGCGGAATCACAACGCACGCGTCGGGCTGTTCACGCAGCAC

CACGCGGAGAGGCTACGGATGTCTGACAGCCCGCTTCAGCACATGCAGAGGGTGTTCACA

GGCGTGGAGGGCATTAACGAGGCAGAGTTGCGTCGGCAGCTAGGCTGTTGTGGCATTACG

GGATCCCTAGCTCTACAGGAGATGAGGAGTCTGTCTGGTGGGCAGAAGAGTAGGGTTGTG

CTCGCTGAGCTGATGACGCATCGTCCCCACCTCCTCCTTCTCGACGAGCCCACAAACCAT

CTGGACCTGGCGTCCATAGAAGCCCTCAAGAACGCGTTGGATGGCTACGGCGGCGGCGTC

ATCCTTGCGTCTCACGACCAGGCCCTGATTTGCGACATGCTAGACTCCCCCGACGACTCT

GCCGATGGAGCTGCGGGGGGCTTGCCTCGCGGCGAGCTCTGGGAGGTCAAGGCGCACCGC

GTGTTGCGGAGGGAAGGGGGTATTGCCGAGTACCTCGCCGAGCTAACCGTGCTGGCTGGG

AAGCGCGATGCAAAgagggcggcagcggcgcgCTGA

>g11119.t1

ATGTCAATATACCCGTCGCACACTACCACGCCCGTTCCAGCCCTCGTTGAGGCGGGCGAC

AGGACCTGGGTTGACGTAAGCGCCAATGGATACGACACGCGGACGTACGACGACGGCTGC

ACCCCGAAGGGCTGCGGACCCTACAACACCAGGGATGGCGACATCTGGGCCAACTCCCGG

TGGTCGTGCAAGGGGGAGATCCTCGACAGCGCTGATCAAGATGACGGTTGCTGCATCACG

TACTCCTTTGAGGTAGCCCAGGACCTTGTCAAAATGAGTATCGCCTTCCACAAGGGAGAT

GAGAACACCAGAAGGCTGGACGTGTACGACAACGGCATCTACCACAGCACAATCGCGTCC

AGCGGCAAGACCCTTGATTACCAGTTCTTCAACCTCTACACCGATGAAACCAAGACCCTC

AAGCTCTGCCTCGACGACCCTAAGTGGTACACTACCGTCTGGCTGAGCATCACCGAGGTG

AAGTTCTGGGTGGAGTAA

>g11128.t1

ATGTCTACCCTATCTATGTTGTCCTTGTGTTCACCCGCCGCCCCCTCATCAGACCTCGTC

GATGCCGGCGAGAGGACAACGGTAGGAGTGTCTGCCTCTGGGTACGATACTAGGACCTCC

TACCCTGGATGTAGCCCGGACGGATGCATTCCCGAAAACACGCGGGACGACAGCCGCACG

TCgaactctcgctggtcgtgcaagGCGGACATCCTCGACAACGACAAGGGCTGCTGGATC

AAGTACAAGTTTAAGGAAGCCCAGGACCTTGAGCAGATTCGAATCTCCTTCTACGAGGGC

AACGAAGACACCCGCACGCTGAAGTTATACTCGAACGGAAACTACCACAGCACGATCACG

TCCAGCGGCCAGACGACCTATTACCAGACGTTCGACCTCGTCACGGACGAGACCTCTGAG

CTCAAGATCTACCTCGACGACTACAAATCGAACAGCGACGTCTGGCTCAGCCTCAAGGAG

GTGAAGCTCATGGTGGAGTAA

>g11332.t1

ATGTCGGGGACCACGACAATAGAcaccgccgacgccgctgctgctggtgctgctatgtCG

GCCTCTAGGTACCAGCAGAGCCGGGAGCACCCGGTTGCGGCTGTGGCTAGGAGACTCTTT

CCCGCACACGCGTGGGAGCGAAGACTGGCGAGGAGAGAAGCCGCTGAGAAGGAGATCATC

CCGGAGACTGACATCACCAACTCGAACGTCCGGGTGTGGGTTGTGACGACAGCATGCTTG

CCGTGGATGACAGGCACGTCGATCAACCCGCTGCTCAGGGCGGCGTTCCTGGCCCGGGGA

AGGGACCCAAACATGGTCACGCTCATGGTACCCTTCTTGGCCCCCGAGGACCAGCCCAAG

gttTTCCCGGCGGGCGTAACGTTCGAAACCGCCGAGGAGCAAGAGGCCTGGGTGAGAAAT

TGGCTCCGAGAGGCCGGCTTGAGCCGGGAATCGGAGAGCTTGCGCATGATTTTCTACCCG

GGAAGGTACCACAAGGACTACGGAAGCATCTTTCCCATGGGGGACTTGACGCTCATGGTT

CCCCCCGAGGAGGCGGACATCTGCATCCTGGAAGAGCCGGAGCACCTCAACTGGTACCGC

GCGCCCGGGAAATCGTGGAGGAGAGCGTTCAAGCACGTCGTCGGCATCATGCACACCAAC

TACCTCGCCTACTCCTCGGGGTACTCGGTCTGGGCCCCGATGCTCACGTTCATGCTGCGG

CCCGCCGGCCCAGCGAGAAAGAGGCCACATTGGGGGTGGGAGGGACGGCATTCGCGACGC

GGACGGGAGATGGGTGGCACCGGGCGGATCATCGAGCTGTCGGCCTCACCTTTACCGGCA

CCGGCGATCGCGGCCGTGATCAGCGCGATGCACGCTCGAACGGGGGGCGCGTACTTCATC

GGCAAGAGCCTCTGGGCCAAGGGCTACGACCGCCTGATCGACCTCCTGGAGTACAACAAC

GACCGCGTGGGGAGGTCGTTCCACATGGACGTGTACGGCAGCGGGCCGGACCGCGAGGCG

ATCGAGAAGAGGGCGGCGGAGAAGGGGTGCGACCTGACGTTTTTCCCGGCGACCGACCAC

TCCGAGCTGGGGAAGTACAGCGTCTTCATTAACCCTTCGCTTAGCTGGGCCGCGGCGACG

GAGCGTTTAGGGGAAGCGACGGTGATGACCGTGGGGGAGAGAAATAGGCAGAAGCCGGTG

GCGGACGGGATACTAACGGTGGCGCACGCCGCTGCTGGTTCTGGTCCCGGCGGGGACCTG

GTTCGGCTGCTCGCGGGCGCGGATACGGCGGCCTGGCAGAACAAGTGGGTGCACGAGCAG

CGGGTGCAGGAGAAGGAGACGGAACGCTCGCGAAAACGTTCTGCCTCGACCGGGGCCGAG

AGCGGCGCCGACGAATCGACGGacatggccgccgccgccgccgccgcggtcgcAAGCGCA

TGA

>g11394.t1

ATGGCAACCTACTACGAGTCGATAGCGGGTGGTGATGATACATCTCGCGGGGGCAGCCTG

GATAAGCGTCATCagaatggtggtggtgctgctgttgctcgcgcCAGGGAAAACGCTTCA

CCACGCCCCACTGCTTCCCATACGCCGATCGTGCCTGCCGCCCCGGAGCCGCCGGTGCAG

GAATCCAATTCTGCTGCCCCCACCACCGCACCGCACGAAGGTAACCGGGGACACGCtgcg

ccggggggggcggggggggcgacagcGGTGGAAGAACCAGCCCGAGTCGACAAACGTGCG

CTTTCGGACTTGTCCGCCAAATGGGTGTCTCGGTCCCGTGGCACTTCGGGGCGGAGGGCG

GTGGTCGCCGACCCGGACTCGTCCATGGTGACAGACTCTTTCCCGTCTGGGCCTGTGAAC

TTTGTGACGATCTTCGGCCCAGGTAGAAGCGGCAAGTCCTTCTTCATGAACGCTCTGGCG

CGCAGGGACGGGATTTTCGGAGTGTCGCCTGCTGCGGAGCCCTGCACAAGCGGCGTCGAT

CTGTCGAAGATCGTGCTCTCGCTGGGCGAGTTCACGAGGACCGCCATGGGTGGTGGACCC

GAGAGCACGCCGTGCGTCGGCTTGGTCGACGTGGAAGGGTTGGGCCACAGACACCCCTCC

CACCACGTTAGGCTGGCTGTTCCTCCCATGTTGGTCTCCAAGgtgaTCATCTTCAACCTA

AAAGACGTGCAGAGGACAACGGCCTTAGACACCCTCGCGGTGCTGGAGCACGCCGCCCGG

TCGATCGCTGTGGGCGAGAGTCAAAATGTATACGGGCACCTCATCATCCTCGTTCGCGAC

ACGTTCGACAGGGCAGATGACATCCACCAACTCATTTTCGGCGAGGAGGAAGCAGACCCA

AACGCAAActacgaggacgaggaggaggaggacgaggagggggaggagcggaAAAGGCGG

AACTCAACTCGCAAGACCCTGAAGAACATCTTTCGAGAGATCAAAGTGTGGTGCCTGCCT

CAGCCTCACTGTGACATTAACGACAAGAAGCACTTCGCCCTGTCCGAACTGAAGCCCGAG

TTCCACGCCAAGCTGGACGAACTGAGGCGGTACATGGGTGGAGTGCTCGCAACCCCGCAC

ACCTTTGGCGGCCGCAACATCACCGGAGGGGCCACTCTCCAGCTTCTCATGACAACGCTT

TGCGATGCTGTGTCTACGCGCAAACCGGTGGAGCCTCTCAGCATGATGGAGAGCATCGAA

GCGCAGGCGGCAGATTCGTTGTCCAACAGAGGAGCCAAGGAGTTCGAGGGCTTGCTGCGA

AATTTCGATGCTGCCAGCGTGTGGTCCGTCGAAGCGATGGAGAACAAGCTTGCAGAAGCT

CGGGGCACGGTCTTGACTGGGTTCGAAGACGCAACCAAGGAGTTGCAAAAAGGCGTTACG

GCGGTCGCCAGGCAGGCTCTGGAGGGTCGGATCGACGACCAGGCGGACGCGTTCAGGACC

ACGATGGAGATGGACCGGATAAAGCTGGACCAGACCGTGAGGCTTCTGAACGAGAGGGCG

TATGCGCTGGTTGAGCAAGAGGCCACGTCTACCGAACTGCCCGTCGCCGACCCCGCGGCT

TTCTGGAGAGAGCTCTGCGACACCACCTTCGAATATCTCGAGTGGGTGCTGGACGGCGAA

GGCGAGGACAAGGGCATCCTCCGGCACTTGCCTGGATTCGTGAACCGCGAGGGGTTCGAA

AAGGCGGTCCAAGATAAGTACCAGCGCttcaaacagcgcaacagcgccaAGCTCGAGAAG

ATGGAAGCCGACAAGGTCATACAGCAGCTGGAAACTAGGGTGGCCGAAGAGAGAGAGCAG

AGGGAAGGGGCGGAAAAAGAAGCGAAATTCCGGGCCAACGAGGACAGCCCGTCCCCCAAC

ATCCACCCGCCGTTGCCCTCGCTTACAGTACGAGCTCGAAACGTTTGGCTCGATTGGCAG

AGGGTGAGCAAGGCGAGGCATTTCATCGACAAGGCGGCAAAGTCTCAGGGGTAG

>g11489.t1

ATGTGCCCCGACGGCTTGGTGTCAAACGGGACTGCTTCTGAGTGCTCGCTGGAGCAGGGC

TTCTGGAGAGTCGACGACAGTTCCCTGACGGTGTACGAGTGCCCCAACGCGGACGCTTGC

ATCGGTGGGTCCGACTCGACTTCCTACTGCGCGGACGGCTTCAAGGGGCCACTTTGTGCG

GTTTGCACGAATGACTATTACCCCAGCGGGAGCTACGAGTGCTTGGAATGCACGGAGGGC

AACGCTGCATCGGCACTCCTGACCATCATatTTTTCCTTGCCGTGCTGACGTTGTTTCTA

GTGGTGGTGCTCAAGGCTGCCGAAACTGTAGAGGAAACCGCGAGGGAAACGGAAGATGCT

GCAAACGAGTCCGCGGCGCAGAACGCAATGAATGTTGGGATGAGCAGCAAGCTCGTCGCG

TTCTTCAAGAAGTGGAAGGACACCCTCATCGTGCGCTTCAAGATCATCGTTGCTCATTTC

CAGATTGTCACCACTTTCCCGGCGGTGCTGGATGTGCAATGGCCGGAAGCCTTCAACAGG

TATGCGGACAAGGTGTCGGTGATCAGCCTGAACTTCTGGGGCATCCTGAGCCAGAGCTGC

TTCTTCCGGCCCGACTACCTGAAGCAGCTTGTCTTCACCACCTTGCTACCGCTCAGCATC

GTGGCCGTTCTCGTGATGTATTACGCCAAGACTATCAAGAAGGTAGGATCGAACGCGGCC

AAGAGGCGGGCTGCGTGGGACAGAACGGCCGGCGGGGTGCTGCTGACAGGGTTTTTCTTC

TACATCGTGACAAGCACACTGATTCTACAGGCGTTCGATTGCCAGGATTTCGAAGACGGG

TCTAGCTACATGAAGGCGGACTACCGTATCAGCTGCGACAGCGACAAGTACAACTTCATA

TACGGGTACGCTATCGCGATGGCCGTCGTCTTCCCTGCCGGCATCCCGCTGGCGTACCTC

ATCGTGCTTCACTTGGGGAAGGAGCGTATCAACCCCGACCCCCTCAACACTGAGCTGTCC

TACCAAAAGAGGGAAATGGACCCGACCATCCAGAAAACTAAGTTCCTGTGGGGTACGTAC

CGACCTCACgtgtactactacgaggtgtgggaatGCGTCCGAAAACTCTTCCTCACGGGA

CTATTGGTTTACTTCCAGGAAGGAACGTCCACGCAGGTGGTGATCGCGATGCTCCTTACG

CTGGCGGGCCTGCGCGTTCTATCAGGCTTGAAGCCCTACCGTCAGGGGGACGAGAACAAC

CTCGCGGAGGCGTCATTGTGGGTCACCTTCTTGACTCTGTTCGCTGGACTTCTGATCAAG

TCGCAAGTGGCGGAAGAAGATGGCTACGACACGGTAGCTTTGGGCATCATCCTGTCCTTC

ATGAATGCAGCTCTCCTCGGCTTGGCGGCGTTCCAGCTTCTTTGGACCACGAGCGAATAC

ATGAAGGAGAAATGGGACGGCGTCGTTGGAGCACGCTTCCAGTCCGACAAGAAAGAAGTG

CCGAGATCATGGAATAGGCCGCAGTCCGCTGGTCGCCCGGTAAGGTCCCCAACCTCCAAC

TACCAAAGCAACCAAAACAAAGCACGCGCCCCAGTGGCGTCACCAGTTTCGGGCGGGGGC

GAGGCCTACCAAGCAAGGGGACGCTCAGGGGTCGTTCGAGGGTGTCGCGGGACGGAAAGG

CCTAACACTTCACGGGACAGAAAGTCCTCGCGTTTCACGGGATGGATTGACTTCGCGGTT

CACGGGGCGGAAAGACAACGCGAATCACGGGACGGAAAGGCCTCGCGGTTCACGGGGCGG

AAAGGCCTCGCGGTTCAAGGTCTCGTGGTTCACGGGACGGTAAGGCCTCGCAGTTCACGA

GACGGAAAGGCCTCGCGGTTCACGGGACTGAAAGACCTCGCAGTTCACGGGGCGGAAAGG

CCTCGCGGTTCACGGGGTGGAAAGGCCTCGCGGTTCACGGGGCGGAAAGGCCTCGCGGTT

CACGGGACGGAAAGGCCTTCATTTTGGAACCCTTTCCTGCGGGATTGGTTGTCCAAGTTA

ATTTGTCTACAGTGGAAATTGATCGTTAACCCCTTTCGCACTGCTGCCTAA

>g11531.t1

ATGGCGACGTTAGGGAAAGCCGCTGCAAAAAGCGTGGGCCCGGCGCTACGATACGCCAAG

CATCTCAACCCGGGGATCAGCAGGATGTCGGATATGATCTTGGttgacGGCAAGGGCAGC

TATGTGACGACGAGCGAAGGTACACGGCTGCTGGACTTTTCCACCGGGATTGGCGTGGCC

AACGTGGGTCACTGCCACCCTAAGGTCGTCCGGGCGATCCAAGAGCAGGCCGGTCGAATC

ATCCATGCCCAGGTGAACATCGGATACCACGAAAGACTGCTTGAGCTCAATGGAGAGATG

CTCAAGATAATGCCAACCAAGAAAGGGTCGGAGTCTTCCCCTCTCGACAGCTTCTTCTAC

TGGAACAGCGGCTCGGAGGCGATCGAGGCGGCGCTCAAGATCGCCCGGCACGCGACCGGA

AAGCCGAACGTCATCGTCTTCCGCGGGGCCCACCACGGTCGCACCATCGGCACCATGGGA

ATCACCACCAGCAAGATAGCCTTCCGCGCAGGGTTCGGGCCGTTCGCCCCTGGGGGCACG

GTAACGAGATACCCCTACTGCGCGCGGTGCCTGGCGCGGCCCGCGCCGTCTGGgagcggt

ggcagtggcggcggcggcggtcaggAGTGCTGCGGGGGGCCGCTGCAAGAGATGGAGCAA

CTGCTGAAGCAGGAGACATCGCCCGACGAGACGGCGGCGGTGCTCATCGAACCCAtcctt

ggggaggggggatacatTGTTCCGCCCCCGGGTTTCATGGAAGGCGTTCGATCTTTTTGC

GACAGGCACAACATCCTTCTCATCGCCGACGAGGTTCAGTCCGGTGCTGGGCGAACCGGG

GAGTGGTGGGCGATGAACCACTCCGACGTTATCCCGGACTTGCTGGTTTTCGCGAAGGGC

ATCGGCAGCGGCATGCCCATCTCTGGGGTGGCGACGCGCTCAGAGCTCACCGCTCACCAG

ACGCCTGGGTCGATGGGTGGCACTTACGTGGCTAACGCCGTGGCATGCGCATCAGCGATC

GCCACGATCCAGGTCCTGCGCGACGAGAAGCTGCTGGCCAACGCGACCCTTCGCGGGCGG

CAGCTCATGGACGGCATCGTGCGGATGAGCGGAGGCACGGGGTCGGACCTCGGCTCGCGC

TTCCCTTTCAAGGACCTGCGGGGCCTGGGGCTCATGGTGGGGCTGGAGTTCGAGTCGCCG

CAAGGGTCCGGTGTGGCGGGCGCCGTGACGAAGGCGTGTTTGAAGAGGGGCATGATTATG

CTGACGTGTTCGACGTACGAGACGGTGCGGTTCATCCCGCCGCTGAACGTCACCGAGGCG

GAGATGGATGAGGCTCTGCGCATCTTCGAGGAGGCGGTCGCTGAGGTGTTTGTCTGA

>g11569.t1

ATGGGTAGCACGAAGGCTGCAGACGAAGAGCGTCTAGCTTCGGGCACGCCGCAACAGGAC

GATGGCACGacgactgatgctgctgctgctgcggcggcggcggcggacggaGCAGGTATA

CTCGAGAAAAGGTCGGACGACTGCAACACAAAGCGAGAAAAGAGAGGCGATGGGAGTAGC

GGTGGGATCCGCAACGCCGGTCCGAGCCCGGGCAAGGAGGGGGGCagcctcgaggaggag

gcggcagtGGGGTCGATCCACCACATCCTGAAGACCGCTTTCGAAGAGCTGTACGAGGAG

GATGCCTCGGATGAAcggggggagcagcagcagcagcagcagcggcagcactcgGCGACG

CTTCTTGCGAGCCCTCGTCCCGGTACGGGCGACAGTGCGTCCGGCCAGACGACGGCAGAT

ACGACCATCGCCAACGCTGAAGATGGGTCGACCTTGGGTGCGGTGGAACCCGGCGAAGAA

ACTTCCGTCGGACTGGCAACGGAGATCACGGCTGGGTCAACCGACCCGCAAGCCGAAACC

GAGAGCATTGAAACCCCCGCGGAGGCGTCTGACGCCGCCACCGATGAAGCGGGAGGGGTG

ACCGCGGGGAAGGAAATCGAGGCGGAGAACGATGAGCCGAGCGGTCCGAACGAGGGCAGC

CTTGCTGACGAAGCGGCGATTCAGGAACGGAACGGCTCCATTGAGGCTATGTTGGGCTCT

CAGCGAGAGCTGGCGAAGGGTCTGCGGAACTACACCCGGGGGAAGCGACAGGAGGCCAGG

GAATCGGACAGgGTGGCTGTCGACGTTTTGAAGAAGCAGGGCATCCTTCTCGGAGCGCAC

ATCCCGATGGGACCGAGCGGGCTGTCTCTGGACCGGTCGCAGATCGAGTCCTTCCAGGGC

TTCGCGACCTCGCTCTTGGACGCGTCGGAGGTTTCGCGGGCTAACAGGGAGGCCTCCCTT

CGCGCATCGGGCAACCTCCCCTGGAATAGGTTTCCGAAAAACGAGGACGAAACCAGCGTG

GTCACCCGGTTCGAGCCTCCCGGCTACCTCAAGAAGACCTCCATCCACATCACCAGGGAA

GCTCTGGCGCTGAAGCGTAGCACGGGCCGGCGAGGAGAACAGAGGCCAATCACAGAACAG

ACGACGGTTTCCGACGGCGACAGCGACGAGACCGTTACATTAGTCAATGGCGCCAccggc

agcgacggcagcggcgacggggacggcgacggcggtggcgaaaaggctTCGAGAAGTCGG

CGGGCGCTGGAGAACGACACAACAGACGACGACAGCGACAAGGaaggcgaagaagaagaa

gaagaagaagaagaaggagaagaaggacgGCGGAAAGCACGGAGGGTCTTCATCAAAGGA

GCCCAACGAGACGCGGACGCCGCCTTAATGACCCGAATGCAGAGGAAGCTTCACTTCCGT

CGGAACCCGCGGTGGGACGCGGAGAGCCGCTGGCACGAGAAGCTGCTCACCGCAGTGGCC

CCTTTTTCGGAGCCCCAGACGGGAGCGGCGACCCACGGAGGGGCCCACGGCGGCAGCAAC

CCCGGCCCGGCCAGTGCCGGGGGCCGGACGAAGGGCAAGAGCAAGACGGAGCTCCTGATG

TCGACACCCCCGAAGGGACGAGCCGACGGTGAAAACGGATACTTCTTGGCGACCCCGTCT

ATCGTTGAATACTTGGACTACGGAGTGGACGAAACCTACAGGGCGGAGCTGGTGCTGCGA

AACATAAGCTGCCTCAAGAGGGGATTCATGCTTCTGCCGCCCGCTACCGAGTACTTCAGC

ATCGCGAAGGTTGTCTTCCCATCTGACGAGGTAGACCCAGACTCCGGGACCACCGTCACG

GGGAGCGGGGACCTCGCCCCCGGGATAGCCGCGAAAGTTTCGATCGACTTCACGCCGGAC

AGCCTGGGGGAGTACCACGACAAGCTGTCCGTCGTCACCGAGGCGGGAACGTTCGAGGTT

CCGATCCACGCGCACCGGCGGCGGCCGTGCCTGTCGCTGCCCGAGTCGGGTCTCCTCGAC

TGCGGCGGGTGCTTGCTCGGGGAGGGGAAGACTATGCGCTTTTCCGTGCGGAACACCGGA

GGTCCTGTGCGCTTTCGCCTCTTCCCGCTGGAAACCCTCGACCGGAGGTGCACGAGTTCA

GACGGGGGGGGAGAACGGCCCAACCCGGAGGACTGCGACGgtgacgctgccgccgctgct

gctgcggattcTGCTGCTGGCGGAGGCGGGAGCGTATTCTCTTCCTCTTCTTGTTCAGCA

GGAGATGTTAGTATCGCCTCCCTTGCTACTGCTGGTGGTTCTAGTGGTGCCGACAATAAG

AAAAATCAGCCCGAGGGCGGTGGGCACGGCGACTGGTTGTCGTCATCTTCAAGAGCagac

catgctgctgctgctgctactgctgccgtcggTCGAGTGACCGACGGCAAGGACAGTACC

GACgacgatggtgttggtggtggcggcttCGAGCGCGGCAGCATTAACGGAGACCTGCAC

GACGACAAGctggagGATAACTGCGAGGAGCGCGAGCTGTGCGTGCGGGCCTTGTGCGAA

CAAGTGTCTCTGAGAATCTCGAACATTGACGGGATTCATGTGCCCaccgcagcggcggca

gcggggggagagaggggttcGCCAGAGGAGCCGCCGAGAGTTGCCCACCCGGCGACGATC

AGCCACCTCCTGTTCGAGCCGACCCTGCTGTCCAGTACGGGACGGCGGCAATTTACGCTC

CGGAACGACACAAGCGGAGATCTGCCGGTCAATTGGGCGATCTCCGAAgtccccctcttg

ctccccaaGGAGCCGTCGCGTCCGTCTGCAAAGAAGAGGGGCAGAGAAACCGCGCGACAC

GGGTCGACCGTGGGCGCCAGGTCGGATGCGACGACGGTTTCCGCGTCTGAATTCGGAGGA

ACGCTGCtagcggaggaggagaagaaacgAAATAGGAACAGCgaaaacagcaccagcaac

acctaCAACGACAGCAAAAACGGTGAAGTCGACCGCGAAGGCATTAATTGCGCCGCGGAG

CCGTTTATGGCACGCGAAGTGTATCCCACCGCGCCCCTTCCTGGCCAACaactagcagca

gcaacagcagccgcagcgggaAAATGGGAGTACGACATTCCAAATGAGTATCAACTCCGG

AAAATCGAAGACGAAGGTCGAAACATCGCAGGGCACGACGGCATGACAAGCGCCGGGCTC

TCGTCCTCTACACTAATATCCGCTGCTCGCGATGCTCGTAGCGTGGAGACGATGTCATCA

TCAGCGGTCACAGCGGCAGCCAGGGGGGCGTTCGCGATCGTCCCCGGGTTTGCCGTGGTC

CCGGCGCACGGCGAGGCTTCTTTCGAGGTGGCGTTTACTCCGACttcgttgggagaaaca

agGTTCGAGGCCGTAGCAAGCGTGATGGGGGTGCCGGCGGTTTCTCTGGTGTACCACAAA

CCGGACGGGATTCTTCCGGACGACGTTGGCGGTGGAACTGAGCCCGGCGTCCGAATCGGA

GCGAGCGACAGTCGCTTCAGGCTGTTTCTTCGGTCTTTGGACGCGGACGGAAAGGGCGTC

TTCAACCGGGTGGCCATCGCCGACGCCGTTAGAGGCCTCCTCCCGCTTCTCCGCGACACC

GCCTCCACGTTCGGAAACCCTTCACCCTTGCCAACCAGCCCAACCCAGGGAGGGAGAGGT

GGAGGGAGAGGGCCCCCCACTAGCGTAGGGGTTTGTCACTCCCCCttttcctccccctct

tcctccttgcCCCTTCCCCCGCCGGTGGTGCTCAACACCGACTTTGCCGTCGCAAGGGCT

GCTGCTTGGCACCCCAACCAAGAGGAAGCGGAGGGTGGCGACGACGCCTCCACAGGGGGA

ACTTCTCTGCCGGTGCCGTTGCGCGCAGGAGGCAGCGTCAGCGGTAGCTGTAACGGTGGC

ATTCCTcaggaaggagaaggagaatatATCGATGTTTCTGTGCTGGTCGAAGAGACTGCG

AAACGGCTGGACCCGGACGAAACGGTAGACGGTTACGCCGAAGAGCAAACCGTCACTTTC

GCGGGGGTTCTGGAGGTGCTCGGTCCTGAAATTCGAGCGCGAGTCACCCACGCCTTCGGT

GACAAATCCTTAGAAGAGCTGGCGGTGCGCCATCGTACTGACATGGCTGGCCTGCAGATC

GAGATGAGAGCGGCTGGCGAGGCCGTGAGGCTGTCTCTCGAGCCGGCCGTGCTGCAGGCG

CCAGGAGAGGTCCCCTACGGTATACCGCAAACCATGGAGTTCCACGTGTGCAATTCGTCC

TCGGCCGCGACTGAGATCGAGTTCAATCTGGAAGGTCTGACTGTGCTGGCCGCGAATGAC

GCCCTGGTGCACTCCAGCGTCGAcacggtagcagcggcagcagcagcagtatcgactCCT

AGCGGGAATGGAGCTGAAAAGGACGACAGCACTACTACCGGGTCGCATGCATCTGTACCT

CCGGCACGGCAAGCGTGCGACCTGCAAATTTTCCCATCGCGAGCGGTTGCGCCCCCCGGA

GGCATCGTGACCTGCAGGGCTACCTTGACATCGTACTGCTTGGGGTCGAACGTTGTGCAC

GTGCCAATAATTGCACcgacggcggcgccggcagAGTTGGGGGAGGAGCTGGGCATTCGA

ATCTGCTTCACTGGGGCCGGTGGAGCTGCTTGCATCGAGGCGGCTGAGCTGGACCTTGGT

CTGATCGCTGTGGGCACGGGAATGGCGAAAACCGTCAGGCTAACGAACGTGGGAGGCGTT

CCTCTGTCTTTCACCATGGTCCAATCAGgcACAGATGCGGAAGCATTGGCCGAGTCGCTT

ATTCGCGACTTTGCCTGGCAGAACCCACCGGATTCTCATGGCGACAACGACCATCAAGAC

CGCATCGGTGACGACATTTTCGAGATAAACTCAGctaccggggggggaggaggagcagga

gcaggagcagaaggAGAAGCTGGGCTCCAAACCAACAACGCGCTGGACGCGGCAGGCCCA

CTGGGGACCGGCGCCGACGGGAACAATACAGCCTTAGGGATGAGGAGGGGGTCGACGGAG

TCGATCGGAAGCGCGGATTCGTTTTCTGTTCCGAGAGACGGGTGCTGGGTCGGGTTTGAG

CCGGCGTTCGGAGACATCGCTCCGGGACGGTCCGCGACGATCACGGTGACCTGCGAGGCC

GGAAAGGTGCCGGAGCGGTTTCGGGCCGTGGCCAGAGTTCTGCTGCAGAGCTCTGACGGG

CGGGGCGTGTCGTTCGGGAGCGAGTACGTCGCCGTGCGGGCGGAAGTCCAAGAACCGACG

GTGTACATCAGCGGTGTAGACGTTGATGTCGGAACGGCGTACATAGGAGTGCCTCTGAAG

CGACGCGTCAGCATGGTCAACCTCAGCAATCTAGAGGCGAGGTTCAAATGGGACAGGCCG

GGGGGAGCATCGGGGTCCTTCGAGCTTGAGCTCGTACCAGACTGCGGCGTTCTGAAAGGG

AGAGAGGCGAGTACCAGACGCGTGCGAGATGTGGAGCTGCGCTACACGGCGAGGAGTGCA

GGCCCCGTGGAGGAGGTGTACGCCTGCCATATCTTCGGCATGGCGCAGCCTCTAGGATTC

GCCATCAAGGCCAAGAACAGGGGTCCAACACTCAGCTTTGGCCTTTTGGAGGACGGGGAG

AGGGCACCTCGCCCAATCCAGCGACCGAGCCTTCCGCAATACCTTGGGGAGGAGCCGCTT

CCCGACCCTGTTCCGGCACCATCGCTCGAGTTCGGCACCGTGGAGCTAAGGGGTCGCAAG

AAGCTCAGGGTTTTCGTCCGGAACCTATCCGCCATCGCGACGAACTTCACTGCCAAGGTC

CGCAAGCACTCTTGCACGCCGTGCCCGAAGAGGCTGAAGCTCGCGAACATGAGCTACTCC

GCCCTTCAGAGAGTGGTCGCCAACCCCAGAGCGCTGGCGGCCTacatcgatgacgacgac

gacgaagacgagcagcaacaacaacaacaacgacaacgacaacaccaccaccagctgaac

GTTGCTTCTGAAACCGCCGCCGCCTTGGCCACAGCAGATGCCACTGCAATAGGAAAGTCA

ACCGCCGCGGCCAACGCCCGTTCAGGAAAGCCAACCATTGCCTCTGCCgctgtgggggcg

ggagggggggtggggcagggaGTGGGGGTGGTTTCTTCCTCGGCCACCAGAGCCGGGGCT

GGGGCGGGAGTTGGAGCGGCTCGAGCGTCGGCTAAGTTATCGGGTGCGGAGCCGTCGATG

CTACGGTCTTCTCAGTGCTCCCAAGGAGTTGATTCGGGTGGGAGGACGCGGCCGGTACTG

GGTGCGGAGCATGAGGCTACCGAGGCGTTCTTCTCCGAGGGTGGGCGGGCGAGGACGAAG

GCCCTGTTTGACCAAAGAGAAGACTGCTTCATCCTCAAACAAGGTCTTGGGGCGGCGTTT

CTCGTGGAGCCGGTGGAGGGTCACCTCCCCCCGTGGGGTGTGGTGGAAGTGAGCATCACG

GTCTTCAACGACACCCCGGGCCGGTACAGCGATAAGTTCGAGCTGGCCTTCACTGGTGCG

CCGATAGCGTACCTACCGATGAAAGTGTTCGTGGAAGGGAGCCCCCTGTCTCTGAAGAGG

GAAGCGCTGGGGCTAGACATGTCTGGGCCGGAGCCCCTGCTAGGCTTCGGAGAGGTGTTC

GTTGGCGGAGGCAAAACATTCCGGGCGATAAAGGTGAAAAACGAGGGCACGTTACCAGCT

ATGCTGACCTGGTCCATGCAAGACGCCAACATCGACGCAAAGGAAGCGGCCAAGAAAGTC

GACGTGTCCCTTCAAGTCGTCAAGCGCACGGATGACGTCGGCGGATGGACGGCGGACTTT

TCTCTGAGCTACCGCCCGCCCAACTCCTTCCAGAGCCCGTTCATGGTGCAGCCCAGGCAG

GCTATCGTGGATCCTCGATCAGAAGGAGCCTTCAAGGTTGTGCTGCCGGAGCACACGAAG

GGGGAGATGGCGGCGAGGCTGGTCGCGGACGCCTACTGGCTGCCGCCCAGCGACGGCTCG

TCGTCCTCGCAGAACTCGTCGGGCGCGTCCCTGGCGTCTATAGCGTCGAGCGGCCCTGCT

CTCGACAATAGAATCGTCAAGTCGCAGTCCGCAACAAAGTCGGGCATGCCTCAAGGTGAC

GGGGCCGCGTCAGAAGTAGGTGGGGCCCTGTCTCCTTTGACCCCGGGTTCTCAACCGCCT

CCGGAGGGCAGCAGTTCTAAGGGAAACCCGACCAACTCGGCATCCGCTTTCCCTGTGGGA

GATGCGAGGGTGGTGTCCACGGCGGGGGATAGCAGCATTTCCAGCTCGGAGAATTGTATG

TCGCAATATGGGGGGATGGCACCTGGTGGAAGGATGGAGGGCGTGGCCAGCAGGGGTGGC

GGGGGTGGAGGTTTGGTTGGCGGTGGGGGCAAGAAGCGCAAGGGCAAGTCCACGACGCTG

GTGGCGACTATGCACGCGCGGTCGGCAGCGACTTCCATCATCAACAAAACGATAAACAGG

AAGCACGGGAGAACGACAAGCGGGGCCGTCACGCTCAAGGCGATGGCGTGGGGGATAGAG

CCGCGATTAGCCCTGGACAAAAAGACCCACGACGCGCTCCGTTCTGAGTCTGGGGGTAAT

GCCCTCCTGCCGCTGGCACCGACTTGCGATGGCGGCAGCGGCTTGACAGGTACTGCCGCG

GCAGCACGCGCTCAAGGGGGCGTCAGCAGTGGAAAGCAAGCCGCGGCAACGCAAGGTGTA

GGCAACGACTCCGGCGCGCCGGATGGCGTTGGCAAGCAGTTCTTGAAATTCGAGGCATGG

TCGACGAGGGCATGTGCCGCCTCGTCCCCCTGGGCAGCCCATCCCACCATGCGACGACAG

ATCAGCCTCAGCAACCCACTGACGGTGGCAGTGTCTTTCGTGGTGCAGACCCGGGGACCG

TTTGCTATCAACCCCTCGCCCACCAAGAAACGCCTGCGCTCTGGTGTTGGcgacggtagc

ggcggtggcggcagcaaaagcagcaccgGTGGCAAGTCAACCATGGTTGTTGCCCAGAGC

GCATCATCCCCGTGTCGGGACTCAGATGCGACTCTTGCAGCACCCAAgcggacagcaaca

gcaacagcagcagcagtaacctcAGGTGCCTCAGGGGctggagcaagagcaggagcaaga

gcaggGGCAGGACGAGGACCAGGAGAAGGGCCAGGAGTAGCCACAGCACCGGTCTACACG

ACGCCCCAGTCGTCGAGAGGCCACAAAACGGCGTCGACCGAAACGAAAACCAATGCCACC

GAAGTTGGTGCCGTCGGCGCACAGCGACAAACTGGAGCTCTCGTGGGAGGACGGGGTGCC

GAAACATCGCAACCAGACCAAGCCCGCGGTGGTGGCacagaaggaggaagaggaggaggc

acTGGGAGAAGGCGGGGGGGTGGAAGAGGTGTTGCGTCGGCCTCTCAGCAGCAAGCTGTG

AAGCTGTTGCCTGGGCAGAACTATCAGATGGAGCTCGTCTTCCTGCCAGCGACAGCGTCT

GAGGATATTGAGGAGGCTCTTCAGTCATCCTTCTGCCCTGGCTCAGAGGACATTCCCCGG

CTGATTCTCAGGATGGCTGGGGAGCTTTGTATCACGTTCTCCACGGGTCATGCTCAGACC

ATCTGCCTCAGAGGGGAGGTGCTCCGTCCGATCATCGTCGTGGCTCCACCGACGCACTCG

TTCGGGACAATCCACACTCAGCGCAGAGGAAACGCCACCCTGTATGTTGGAAACCCCACC

TACGCCGACGCCGAGTGGACCCTGTCGCACATCCCAGCACCCCCACCCAAGCACCGGCTC

TTCGAGAGCAACCGTGCCTccaaagcagcagctgcagctgtcgcttctgctggtgctgct

ggtgctggtgctggtgctgctatggCACCCCGTAAGAGGAGCTTCGCAGGGCAGCATACC

TGTGAATACATCGCCGGGAAAGGCACCACTGGGGATAGAGGGTCGTCACCTCTTGAACTT

GGTTGTACGGACGCTTCTTCAAGCCCCATGTGTTCACGAGAGACAAGCGGTGGCAGACGA

GCAAGCGGTGCAGCATCACagggcggtagcggtggctCGCGAGGGGGGCGGTTGAAGCTG

TTgccgccagcagcaccagcagcaccagcaggcgGACCTTTTGCACCTCCGCCAGTTTTC

GTCGACGACCCATCGGTGTTTGTGTTCAACGAGGTAGTTGGGGTGGCGCGTGGGGTTAAG

CTGCCCCTGTCGTCGTCTGCTTCGTGCTTGCCGGAAGACTGGAACCGACTAGAGGAGGCG

TGTTGGTCCCAGCAACCGACGCGCGTTACCTGGGGGTGCGTGGTCGACAGCAATCTCGGT

GGCCGATCTCGAGAAGGAGCCAGtgtacagcagcggcgacgcAATCACCAGAGCTCCTTG

CCGCGGCACGCTCATTTCCGTGAGCCCGGACAGCCCGAGAGCTCGCCATCAGCAACGGAC

GTTGGAGCGGCCGGGTGGGCCCAAGGTGGAATTTTCTGGCGAAAAGAGCCTCACGGACAG

CGCCAGGTGGCCGGGGCCACCCTGTCGCTGGAGCGGTTTACGGTGCAGAAGGAGGAGACC

GAGGAGGGGTACCGAGCCCCACGGCCCCTGGAGGTGAAATTCATGCCCAAGCGCAACGTC

CTCTACCGCAGCCGCTTCCGCCTGAACGTTCGGGGCGGGGAAGGCGCCGAGATTGTGCTC

GAAGGGTCCGGGACCTACCAGGAAGACTCCACGCCCGGGAAGCTACCACGAGTCTGA

>g11685.t1

ATGAGCAACTCCAAGGCCATCAAGGCAATTGTGGCCATAGCCACGTGCTCCGGGGCCTCG

GCCTTCGTTGCCCCCACCCACCTTGCCAGGACTCTCGCCGCTCCGTCGGTACCATCGTCG

CAAAGCGCGCGCCAGACGCCCGCAAACCGTGGCGTGGCCGTGGCTCCCCTGAACGGTTTC

TCGTTCTCGAGCATCACCGACATTTTCACACGCGCCGACCGCGGAACCGGAACCTGCCTC

CCGCAGGACGAGAAAGCCGACCAGACGGGCCGCAAGGAGGAGCTGGAGGCGGCGCGGAAG

TTATACGAATGGGACACAACGGGCATCCCCGTGGGCACCCCATTCCTCAAGGGCTACCCC

CCGGACGAAGAATTCCCCAACACGCTGTGGCTGGCGGGGTTCGTGAAGGCTGCCCTCCAA

ATCGTCGTCGGCACCCTTAGGGGGTCCATCGGCGCCAACGAGATCTTGGAGGCTCTGTCC

AGCCCTGAAAACGCGAAACGATTCGCTACCAGGCTGAAAGACCTGTTGAAGGAGAACGTC

GCCAGCGAAAGGGCCGATGACATTGAAGACTACGAGGACCTGCACGCGTTCCCTGTGAGG

GACCCCGATACGATGTTCGACTGGGAGGATGACGACACATTTGGGCGTCTCCGCCTGCAG

GGCGCCAACTGCGTCGTGATCAAGAAGGCCTCCGCGGCCACCCGCAAAAAGCTGACGGTG

TTGGACACTGATCCGTCCTACGCGGGCCTGAAGGACAAGGTGGACTCGCTTCTGGAGGCT

GGAAAGCTGTTCGTGGTCGACCACGAGCTGCTCGATGGCATGAAGACGTCGTCTTACGAC

AACTGGCAGAAGTACCTGACCCCCGGCATCGCCCTGTTCGAATATGTCGACGACGACCTG

CTGCCCATCAAGCCCATCGGCATCCAGCTGAGCCAAGGTAAGGAGGCCACTCCTATCTTC

ATGCCTGAGGACGGCTATAACTGGCAGATCGCCAAGGCGTGCTTCGAGTCCGCTGATTTC

ATCATCCACGAGGTTGTCTCCCACCTCGGCAACACCCACGTCGTCCTGGAAGGAGCCCTG

GTCGCCATGCACCGCCAGCTGCCCAAGGAGCACCCCCTGTACGACCTGCTCCACCCCCAC

TTGGAGGGAACCGCCTTCATCAACTACTACGCGCAAGACACCCTTATCGCCCCGGGAGGC

ACCGTGGACATCCTTACGGCTAACGACATCACCGAAACGTGGGACCTGTGCCTGGCTCAG

ACACTGGAGCGCATCAACTCAGACTTCTCCCCCGAGGCCGACTTCGAGCAACGTGGGATG

ACCAAGGATGATTTCCCCGGCCGGTACATGTACCGTGACATTGGGAGCCGGTACTGGGAG

GCGACGCACACCTGGGTGAAGGAGTACATGGACATATACTACGACAGCGACAAGGACTTA

GACGAGGACTACGAGCTGCAGGCATTCATCATGGAGATGACTGACATCGCCCAGATGAAG

TGGCTCGACGAGTACAAGACCACGACCGATAAGAAGGCCCTCATTACCAAGGTTTTCGCG

TCATTCATCTACACCGCCAGCACCCTGCACGCCGCCGTCAACTTCCCCCAGAAGCCTTCC

ATGTCCTTCGTGCCCTCCTGCCCGGCCTCGGTGTACGCCGAGCCCCCCACTGACAAGTCC

GAGAGGACCAAAGAGGACTACATGTCGTACCTGGTGCCCATGGAGATTGCACTCAGGCAC

GTCGCCGTCCTCACGCTGCTAGGGTCCATCCACCACACAGAGCTTGGACAGTACAGCGAC

GACAACTTCGACGATGACCGCGTGACCGGGAAGGATGGGCCGCTGAGCAGGTTCCACGAC

GCCATCGAGGATATTGGAGAGGATTTGACAAACACCAACCGCGACTTGGTGAACGCGTGG

GGCAAGAGGGGCAAGGACAAGAGGGAGGCCAAGAACTTCGGCTACAAGGAGCTCCTGCCC

GGAAATATCCCCCAGTCGATCAACATCTAA

>g11798.t1

ATGGCTTCTCTCATCAGCAAGACGGCATCGCAGGCGGACCCCTGCGAGACTGTCCCCGAG

AAGCCCGTTGCTAGCGTTGCGAACCGCACCTCCGACTGCTGCAGCGGAAGCACAAAGGGC

TGGGCGCAAGTCCCGGAGGAGGCGGGAGTGGAGGCTGGTGACCAACCTGCGCCAGGGTTC

GACGGTGCCGTCGGCCGCAACATGACTCGCATGGTTTCAGACACCGGGATGATCGGACAA

CCGGATCCTAGCGTCTACCCCCGCTACTGTGGCTTCTCTACGTACGCCCGTCTGCCGAGC

ATCCACGAGGTGTCCAACTTTGATGTGGCCGTGGTGGGCATCCCGTTCGACTCCGGGTGC

ACGTACAGGCCAGGGGCGCGGTTCGGCCCCGAGGCCATCCGTTGCGCGTCTCGCCTGATT

CGAAGGTACAACATGGGCACCGAGGTGTACCCGTTCAACGGCATGCAGGTCGTGGACTAC

GGCGACATCCCTTGCAACCCCTTCAACATACCCAAGGCAATCGACGAGATCAAGACCGGG

CTGGGGGCGGTTTTGTCGCGGTGTGAAGGCGTCGTCGTCATCGGCGGGGACCACACCATT

TCCTACCCCTGCCTTAAGGCTATCAGCGACAAATTCGGGCCGGTGTCGCTTGTACACTTC

GACAGTCACTTCGACACGTGGGACGAGTACTTTGGGGAGAAATGCACACACGGGACTCCT

TTTAAGCGGGCGATAGAGGATGGGCTGATCGACACATCAAGGTCCATGCACGTCGGCATC

CGAGGGTCGGTGAACGATCACGAGGACATCACTGCTGACGCCGAACTGGGCTTGAAAACT

ATTTTCTGCTCTGAGCTAGACGACAACGGTTTCGACTGGGTGGCGGAAAAGATCAGGACG

CGCGTGGGGACGGGCCCCGTGTACCTGAGCCTGGACATCGACGTGGCCGACCCCGCCTTT

GCCCCGGGAACGGGAACACCGGAGGTGGGTGGCTTCTCGTCGAGGGAGCTGATCCGAATC

CTGAGGTCCATGAAGGGGCTCAACATAGTTTCCGGGGACGTTGTCGAGGTGGCACCCGCC

TACGACCACGCTGATATTACCGCTCAGCTGGGGGCGAACATCGTGTTCGAGATGCTGTCC

CTCATGGCCGTCGCACGAAATCAAAAGAAGAACGCCTAG

>g11849.t1

ATGATGATAGTGTCCACGATAGTGTTCACGATAGTGTTCACGTTAGTGTTCACGTTAGTG

TTCACGTTAGTGTTCACGTTAGTGTTCACGTTAGTGTTCACGTTAGTGTTCACGTTAGTG

TTCACGTTAGTGTTCACGTTAGTGTTCACGTTAGTGTTCACGTTAGTGTTCACGTTAGTG

TTCACGTTAGTGTTCACTTTAGTCCTAACGGCAGTGAGTTATTTCGGACACGACGAGAAG

GCGTTTCGTAGAGCGGTAGGCTGGCAGCACTCCCGCCTCCGGGCGGCTGCTGAGGCCGCG

CCAGACGGAGCACCCTTGTCCGccgccggcagcagcggcggtaacggtatcggtaacggt

atcggtaacAGTAATAGTGTCGGCGGTAACGCTCACACTAACGGTAAtggcagcggtaac

ggtaatgaCGGCGGTATTGGTAGCGGTAATAGCGGAGGTAACGGTAACGTTAATGGCGGC

AgtattggcggcggcggcggcggcgctagcGGACGGGGCACGTTCTTGGTGTTTACGAGC

CACGGCCAGGGCCGGAGGGCCCGGGAAGCGTTGGAGGGCGCCCTTTCGCCCTCGTCCGTG

TCGACCGTCACCCACAACCGTCGGCAGGGCGGCTGCTTCATGCTGCACGCCACCGCGGCC

GAGGTGGACTCTTTGCTGCTCCTGgaccgagcagcagcagagggaggAAAACGAGCACCA

GGGCAAGGGCGAAGACGAGGGCTACAAGCAGAAGGAGATAGAAGAGatggagaagaagaa

ggagcaagagaaggggggggaggaggaggaggagaaggagaagaagaagcgcTCTTCGAA

GGCTTCGTTGCTTTGCCTCCGAGTCTCAAGCTGTCACCTTCTCTATTGGATCACggcagc

ggtagtggtagtagtggcagtagtgccggttacgacaacgacgacaattcAGCGGCATGG

TCAGCCACGACGGCGACGGAGCCGTCGACGACGATGCCCGGAAAGGCGTTGCACAAGGAC

GGGATTGTCGTGCTCCTGTCCCCGGGGTCCGCTGCCTCTCGGTTCGCCGGGAGCGAGGAC

GGTGGGAGCGAGGGCGGCAGTAGCGGGGCCGAGCAAGCCCTGGCCGAGAGCTGGCGGCGG

GAGTGGTCCGCCGCCGAGCTTGACTTGCACGGCCTCTCATTCTGGTCGCACACCGCGCAG

CGGTTGGGCCTGAGCGAcgaggaggcgggggtggaggggggagtggggggtgagggtggg

gcgggaggcggggaggaggggggcgaggggttgcTACTTCGGGAGTGGGGGTGGGCTGCT

CGGACGGTGCACTCGCTGGCGGAGAGGGAGGGTTTGTCCCCGGCGGAGGCGTGTGGTTGG

AACGacgtgatggtggcggcggagggCGACGGTCTCGTCACCGTGCGAGAAATCGGCCAC

CTTCTCCCAAAGCTCGGCCGGTCAGATCACGACAACACCGTCGACTCGCGAGACGCGCCA

GGAATCACCCGGACCCATCAATCGCCGGGAGGGGTCCCGACGAGGGGGCCCCCGACGGGG

GAGGTCCCGCCGGGTAGTAAGGGGGCCCCAGGAGTCCCCCCGCCCGTCCCGGAGAAGAGG

GAAAACAGGGAGCGCATGGCGTGCCTGATGGGGCTGATATCGTTCCTCGCCTCGAGGCCG

GAGGCTGCGCGGGTGTCGGCGCTGCCGCGGGGGGAGTTGTTTAACGAGGTGGCGACGCGG

ATCGTGCAGGGGGCGTCGGAGACGAACTCGCCTCTCTGGGACCGCGGGGTGGACGGCTCG

GGGCAGGTCGTGCAGGTAGTGGACTCGGGTCTTGCAGAGGGCTCGTGCTTTTTCAGGCAC

TCGTCTGGCAGCAGCGGAGTCGATCACGGCTATCTTAAGGAGGCGGACGGCACTCTCGGC

ACCAGAGATCTGCAGTACGACCTGTCGAGGCGTAAGGTCGTGCAATACATTGAGGGGCGG

AAAGGAGTAGACACTTACGGAGACGACAACGACGGCGGCCACGGGTCTCACGTATCGGGG

TCTGTGGCGGGAGACATCTACGCCGGCTGGTCGGGTCCCTCGGACTGCCCCGGGGGAGTG

GAAGGGGCGGGGTCGGAGGAGACGGTGCTGTCGTGCGTGGGGAAGTGCCTGGCCCCCTCG

GCGATGCAGGCGTTTTTGGACAACGATATGTTCGATCTGGACGCTTTTTGTCCCGAGTTC

GAGTGCGACGTATTCTGGGATGACGAGTGCCTCACCGACGACCGCTCGGAGGTTCTCCAA

AACGCCAGGGGCATGGCTCCGGGGGCACGCCTAGCGGTGCTCGACGTGGGGTCGTCGGAT

GGCCTGGACCAGGTGCTGGGGGGCGCGATGTGGGAGACTTCGGCCGGGACGGGGGCGAGA

GTGCACTCGGGCTCGTGGGGCTACCCGGACGAGCCCTGCGTGATAGACGAAGCTTCTGTG

TCGTTTGATCAGTGGTTGTACGAGAACCCGGAACATTTACTGGTGTTCGCGGCGGGCAAC

CGCGGCGATGACATGTCGGGCTGCAGCGTTGTCTCTCCCGCCCTCGGCAAAAACGCTTTG

GCGGTGGGGTCTAGCATGTCGGGGCCGAACCGACTGACGACGGGAAGCATGGACGACATA

TCGGGCTTCAGCAGCAAAGGGCCCACATCGGACAGGAGGATCAAGCCTGACATTTTGGCG

CCAGGGCATTTCATCTTCAGCGTTTCTAACGGCGGCGACGGTTCGTGCGAGCTTGGCATG

AACTCCGGCACGTCCATGTCTTGTCCCGTGGCGGCCGGCGCTGCGGCCCTCGTGAGGCAG

TACTTCGACGACGGCTACTACGCCGTCGATgtcgagacgaggggggggctgtGCTCCGAT

ACTtctagtagcagtagtggtacgtCAGTCACGCcggtgggggcggcagcgggACCGGGA

TTTTCGTTCGCATGCACCAGCTTTAGTCCGTCGGGAGCGCTCGTGAAGGCGATGCTCATC

CACGGTGCTAACGCGATGGGAGGCAACACGGACCCCGACGGCGATAGGGGCTTCGGCCGC

ATACACCTGGAATCGGCCATGCCGTTCGAGGGGGAGGATCTGTGGGCGCTCTACGTGGAG

GACGACGACGGCTCCACCTCAACCGTAGGAAAGaTTGAGGCGTTCGCGGTGGAGGACAGG

GGTTTCGTCGTCACGGAGgcggacgcggcggcggcggtggcggcgggtaGCGGAAGCGGT

CCCGGCGGGATAAGGGTTACCCTGGTGTGGATGGACCCGCCGGCGACGGCCGCATCGTCA

GTGCAGCTAGTACACGACCTCGACCTGATTTTGGTGGGCCCTGAGGGCGACCTGTGGACT

ATgTCCGGGAAGTGCGACGGCGACGACGAAAGCTCCTGCGACCTCGACGGGTCCAACAAC

GTCGAACGCATCATCGTGCCTGCTTCCGACATCACCGCGGGAACCTGGACCGCCCGGGTC

TCCGCCGCCAATGACCTTGTCGAGGGCGGGCCCCAGTCCTACGCCCTCGTCGTCTCCCTG

CCCATATCGGCGGGGTCTGGAGGCGGGCTCGGGGGTGCCGAGGGAGGCTctcgcggcggc

gacggcggcagcgactcCGCCTCCGATAGCAGCGCTGGCGATAGCTTCGCCGCAACCCTT

CTCTCGCCCACGCCGAGCCCCGTGAGCTCCCCGACAGGGGCCCCGGAGGCCCCCCAGGAG

CGGCCCAGCGAGGCTCCGAGGACAACGACGGAGTCGCCTGTGGAGGAGAGGATGACGGAG

AGTCCGGTGGTGCCTCCCACCGACGGCGATGACGACCGAGACAGCGGCCGAGACCAACCC

GTCGGTACCGTGGCGCCGATAGGGAGTCCGGCGCCGTCGGTAGCTCCTGCTGTTGGTCCC

ACGGGGACTCCGGAGGCTCCAGAGTTGTCTCCGTCGGTAGCCCCTGCTTTTGTTACGGAA

GCGCCCGCCGAGGGGTGGGCTCCGTCGGTGGCTCCGGTTGATGCGACCAGGAGGCCGGTG

GGGAGTGGGGCCCCGTCGGTATCTCCGGCGTGGATCCCGTCGGTAGCCCCAGCCTCGTCG

GTATCTCCGGCGTGGATCCCGTCGGTAGCCCCAGCCTCGTCGCCGCTGCAGCCGGAGGCG

ACCCTAACGCCGGTAATtgtcggtggcggcagcggcggcgttgGTGGGACGTTTCAACCG

GTAGTGTCTGAGGAGGAGGGGATAACCGCGCCAGGTAACTCGCGGACGGCCGTGCCAGCC

GCGCCTCCGTCGCTGCCGGCTTTGTCACCGCCGTTTTTCGGCGGgggtagcagcagtggc

ggcggtagcggtaatgGTTTGggaagcggtagcggcggcggcggcggtggtagtggtatc

AGTGGTGGTAGCGGGAGTTCGTCCTCGAGAGGGGTGGTAgcggatgatgatggtggtgcc

gGAGGTAGAGGTGGCGGTGAGGATGACGATCAGGAGGAGATAGGGGACGACGGCTTCGGG

AGGGAGAATGTCTACGATGACGATGACTCGCGAGAGGAGGGCGGGACGCCGTCCCCTGTA

AGGGGGGGTAGGTTTAACGGAAACGATGGGACGCCGTCTCCGGAGAGCGAGGCTGTCGCG

GCGGCTTCACGTTCTCCGCCGTCTCCATCTGCGGCTTCTGCTActgtcactgctgttgct

gctaccgccgctggtattgctgctgcggctaccgctggtggtggtggtggtattgctgct

gctactaccgctggtggtggtggtggtggtggtggtactgctgctttgctaccgctgctg

ccgatGTTGGGGCCTTTCGGGGACCACGTTCGCCATTGCCTCCTCGGATACGCCGTGAAT

GTCGCGAAGGTCCCTGACGCTACTCCATATGCGGCGGACGAGGTCCGAATTGGGCAGCTC

CCTTCGGACTTTTTCTCCCAATGGACCGGAGAGGTCAGcaaggacctctggcaccgtcgc

cactaa

>g11951.t1

ATGCGCCGTTCTCCGTCTCTGTGGTCGGGgggcctcctcctgctgctgttacaaCTACCA

AGCATCGCAGGTATCACCGTTGAATTGTCCGAGATCGAGCAGGTCGGCGATTCCAACGTT

TTCGGAGAGTATCGTGGTGATCACCACTACCTCTCCGATGAGGACCTTCTTGACACGGAC

TGCCCTGCTGCAGGAAAATGTATTGGACTGTTGACCAGGGATGGGTCTCTAGACCTAGAC

TCTCGATACGGGTGCACGGAGCCAACGGAACCGTCTTCATCAGACGATGACGGGCCCCCG

CAAAACTTTTGCGAAGTGGAGTACATCTTCGACTCGGCCACCATCGCATCTGTCCGCGTG

GCATTCCACCCCGACAGCACACGGACGCTCAACATTGAGACGAACGGGGACACGGACATC

GGACAATGGGAGGCCGCCCCTGAGACAACCGAGTTTCAAGAAATCCCCGGCCTCGAGGGC

CAGGAAGTGACATTTCTTCGCTTGCAGAGCGCTTTTGGATCCGGAGACTACCTCGGTATT

CTGGAGGTGGAGATCTTCGTCGTCATTGATCAGGTGCCCGTTGCCGGGACATCCGCCAGC

ACACGGGCCACCATCTCCGCTACGTCGACCACGGACACGTCCTCGGCCATGAACACTCTG

GACGGCGATGTCACCGATGCCTCTACGTGGAGCTGCTCGGCTGGAGAAGTGTGCGAAATC

ACGTACGATCTACAAGCTACAGAATCTCTGGAGCAGCTTCGCATAGCGTTCTCTGAAACA

CTATCGACAACCGGAGGCGAGCTGAGCGTCATGACGGCCGGCGAGTCCGGCGTTTTCTCC

ACGGTGCGAACCGGCATCAAGGCGGGTGGACGCCCTGTCTTCAGCGACGGCTTGCAGACA

TTCGGCGGCGTTCGGGCGTTGGCGCGCTACGTAAAGATCGAAGTGACCGCTCCCAGCGGC

GGAAACGTCGTCATCAGCCAGGTTGACTTCCGTGTCGGAGACGAGGCCCCCACTCGTCCC

GTGGAGGAAAAGAAGTGGCTAAAGCCCACGGGGCCTCTTCCTCTCGTGCATCAGGACACG

TCGTCCGAAAACGAGTTGAACTACGACAACAGGTCGCCTGCCGATGGGGGGTGCGACTCC

CCCTCTCACTTCGAGGGGTGCCACCCGTACTACGTTAAGGACGGAGACATGAGCTCTACT

TGGACGTGTGGTCCTCTAGCGGTAGGGACCGGTGTCAACTCCGGGACCGAAGATTGCGAG

ATGGGTATGGACCTCAACCACTTCCGCTACGTCCGCCAAATCCAAATTGCGTTCCCCATG

GGCGACGAGCAGCACGACGAGTTCAGTGTCGAGGCGCTCACTTCACTGGGATGGTTGACG

GTGGTGGCTTCCGCGATCAGCTCGGGCGATACTACCGACTACCAGACATTCAACGTCGGC

GTGCTTGCCAAGGAGCTAGTTGTGATCCCTAAATTCCGGCAATTGAACCAGTGGTTCAGC

ATCAGCGAGatcgtgatcttGGAGAAGAGGAAGAACGACTTCATCGAGGGAACCGTCCCG

GTGTTTTTCATTGATGAGAGATTTGCTGGCGACAGTGATGACGATGTGACTCAGGTCGAA

ACTCCCACGAGGTTCGAATTCGACATTCCCGAGAAAGACGACTCCATCGTAATGGACGTC

GGGGAGGGCACGATCGTGACAGCTCTGCGGATGCGGTTCCCTGCGGACAGGGAGTTCGTG

TTTGTGGTGGAGACTGAAGGTGTCCTGGAGGAATTCACCAGCGCTGGAGGTGACAACGTT

TGGGAGACGTTCACGCTGTCGAGCCCCTTCTCCGATTTCACTACCAGGATCGAAATCGTT

GCCGTCAGCGGGCCATCATTCACCAACAAACCGAATTATCCAGCGCTGCGAGTCGTCGAC

TTCCAGGTTGTTGGCGAGCTTAAGGACAACGGCCCGGGGAGCTTTAAGATGGTGTCCACA

ACGATCCCTCAGTGGCGTGGGATCCCGGACGTAATCGGAAACGGAGTTTCCGAGCAGCAG

GAGATCATGACGCTCATCTGCGAGACGAAGGGCGCCACGTTCGACGGGACGGACTGCGTC

GGAGAGCTCGACGATTCTACCGTTTATATCGAATTCGAACTAGGAGAGTACTTCCTGGAC

GGACCCATTTTCATGAAGAGCGGGGTAGAACTGGACGGGAAATACAGCGCCGAATTTTCG

TTCTACACGGAGTTCGTGCTGTACGATGGCGCCAACAACGGAAACACTGCGGAGGATGCC

ATCCTGATCTTCGACGACGTGCGCAGAATCGATATGCTGCGGAAGACGGACTGGAGCGGA

GACATCATGCCCAACACACTTGGAAACCTTTGTTTCGACGTCAGGAACTCCAAGGATATT

GTGTTCGATGATGTTGCCCCGCGGGAATGCAGGACAGGCGCAGCCCGGTTCACGGACTCC

AGCAACATCAATACGTTCCTCTTCTTGGAGTCGAGGCAAGAGACCGGAAACTACATGGAG

CTCACGCGCGTGGATGACTTCATCGTCAGGGGCTTCGGGGACGTGAACGGACTCCTCATC

GACACATGCAACAACGTTACCTGGGGTGGCTTCGACGGCTACAGTATTCCTGACCCTAGG

ATCCTGCCGCCCTTCGGGGGCGAACAAACGGCTAGCGTTGTCGTCACTGGCGACTCTTCC

GATGTAGTTTTCAAGGAGTGCTACATCGGCCCTGGTGCAGAGCCGCGCATCCTCATGGAG

TCGACTATGCCTCTTACGCTCGAGAACATCTTTACGTACGAGGAAGCGCTGAGCGGAGAC

TGCATCGTGCAAGTGCCAGAGGGCACAGCCGATGACCTCATTGTCCAAGTGGGTTCCGAG

CAAACCCTGTCCAAGTCTGGTGACTGCTGGGTCCTTGACTAG

>g11955.t1

ATGGGAGCCGCAGTATCTGCACCGCTGGACCTAGGGCAACGTTGTCTGGGCATCACAGTT

GAATTGTCTGAGATCCAGAAGGTCGACGATACTTTCGCTTCCGGAGCGTTCGATCGTACG

CCGACCTTCGGCACCGTTGTGGATCTCGTTGACACAGACTGCCCTGCCGCAGGCAAATGT

ACAGGACTGTTGACGAGGGATGGGTCTCTAGATCTCGACTCTCGATACGGGTGCTCTGAG

CCATCCGAGCCGTTCTCAGGCGATGACGACGGACCCCCTCAGAATGTTTGCGATGTGGAG

TACAGGTTCCGAACGGCCGGTGGCGACACGGCCACGATCGCGTCTGTCAGCATAGCGTTC

CACCCCGACAGCACCCGGACGCTCAACCTACTAGCAAACGCGGACACGGACATCGGAATA

TGGGAGGCCGTCCCTGACACAACCGATCTACAAGAAATACCTGGCCTCGACGGCCTGGAA

CTGTCGTCCCTTCTCCTGCGGGGCGTTTTCGGAGCCGGAGACTACCTCGGTATACTGGAG

ACGGAGATCTATGTCATCGTTGAGGATGTGGCCATCGTCGGGACATCCGCCAGCACAAGG

ACCACCATCTCCGCTACGGCTACCACGGACACGTCCTCGGCCATGAACACCGTGGACGGT

GATGCGACTGATTCTTCTGCGTGGATCTGTTCAGCTGGGGAAGTGTGCGAAATCACGTAC

GATCTACAGGCGACAGAATCTCTCGAGCAGCTTCGAATAGCGTTCTCTGAATCttcggcg

gtggggggggagctCAACGTCATGACGGCTGGCGAGCACGGCGTCTTCACTACCGTTCGA

ACCGGCATCGACGCGGGTGGGCGCCCTGTAGGCAGCGATGGTTTGCAGACATTCGGCGGC

GTTCGGGCGTTGGCGCGCTACGTGAAGATCGAAGTGACCCCTCCCAGCGATGGAAGCATC

GTCATCAACGAGGCGAGTGGTCAAGTTGAGTTTCGTGTCGGAGACGACGCCCCCACGCGT

TCCGTGGAGGAAAAGAAATGGCTCAAGCCCACGGGGCCTCTCCCTCTCGCGCACGACTAT

ATGTCGTCCGAGGACGAGCTCAACTACGACAGCAGGTCGCCTGTCGATGGCGGCTGCGAC

TCGCCCTCTCATTTCGAGGGGTGCCACCCCTACTACGTTAAGGACGGGGACATGAGCTCT

CGTTGGTCGTGTGGCCCTCTAGCGGTGGGGGCACACGGGACCGAAGAGTGCGAGCTGGGT

ATGGACCTCAACCACTTCCGCTACGTCCGCCAAATACAAATTGCGTTCGACATGGGCGAC

GAGCAGCACGACGAGTTCAGGGTCGAGGCACTCACTGCACAGGGATGGATGACGGTGGTG

GCTTCAGCGATCAGCTCCGGCGATACTACCGACTACCAGACATTCGACGTCGCCGTGCAC

GCCAGCGAGATCGTTGTGGTGCCCAAATTCAAGCAACTGAACCAGTGGTTCAGCATCAGC

GAGATCATTATCCTTGAAGAGAGAAGGAACGACTTCATCGCGGGAACCGTCCCGGTGTTT

GAATTTGCTGGGCCATTTGAGTTCGACAGTGATGACCAAGCGTCTCAGGTCGAAATTCCT

ACGAGGTTCTCATTCGACATTCCCAGGGCAGACGACAGGGTCAACGTCGACATGGAGGGT

GCGACGGTCACAGCTCTGCGGATGCGCTTCCCTGCGGACAGGCAGTTTGTGTTCGAGGTG

GATGCTAACAATGACCCCCAAATTTTCACCAGCGCCGGAGGTGGCAATGTTTGGGAGACG

TTCACGTTGTCGGAGGCCGTGGTGGTGCATGGTAGGTTCGAGATCACGGCCGTCAGCGGT

CCAACGTTCGCCAACAAACCGGATTACCCAGCGCTGCGGGTTGTCGACTTCCAGGTTGTT

GGCGAGCTGGAGAACAACGGCCCGGGATACTTTAAGATGATGACGACAACGATTCCTGAG

TGGCGCGGTGTCCCGGACATCATCGGTGACGGGGTCTCCGAGCAGGAAGAGATCATGACG

CTCATCTGCGAGACGAAGGGTGCCACGTTCGACGGCACGGACTGCGTCGGAGAACTCGAC

GATTCCACCGTGCATATCCAATTCGAACTAGGAGAATACTTCCTGGATGGACCCATTTTT

ATCAAGAGCGGGGTAACACTTCACGGGAAATATAGTGCCGAATTTTCTTTCTTCACGGAG

TTCGTCTTGTACGATGGCGCCAACAACGGAAACACGGCCGAGGAGGCCATGATGGTCATC

GACAACGTCGCGGGAGCAACGGAAAGTGCCGTTCCCTGCATGTCTGCCCTTTCCTGTTCG

GATGTTTAG

>g11958.t1

ATGCGCCGTGCTTCATCCCTGTGGTCGGGAGGTTTTCTGCTTCTCCTCTTGCAACTACCA

AGCATCGCAGGCATTACAGTTGAGCTGTCCGAGCTTCAGCAGATCGAAGACATCTACGCT

TACGGAGCGTATCAAGATCCGGCGCAGGCCTTCGGCAGCGGTGTGGACCTCCTTGACACG

GACTGCCCTGCTGCAGGAAAATGTACCGGGCTGTTGACCAGAGATGGGTCTCTAGACCTC

GACTCTCGATACGGGTGCACCGAGCCATCCGAGCTGTCTTCGGACGATGATGACGGACCC

CCTGAAAATGTTTGCGCTGCGGAGTACAGGTTTGCTACGGACGGTGGAACTACGGCCACC

ATCGCGTCTGTCCGCGTAGCATTCCACCCCAACAGCACCCGGACGCTCACCCTAGTAGTG

AATAGGGACACGGACATTGGACAATGGGAGGCCGCCCCTGAGACAACCGAGCTGCAGGAA

ATCTCTGGCCTCCAGGGCCTGGAACTGTCATCTCTTTTTTTGCGGGGGGTTTTCGGAGCC

GGAGACTACCTCGGTATACTGGAGACGGAGATCTACGTCGTCGTTGATGAAGTGCCCGTT

GTCGGGACATCCGCCAGCACACGGACCACCATCTGCGCTACGGCTACCACGGACACGTCC

TCGGCCATGAACACTCTGGACGGCGATGCCACCAATGCCTCTTCGTGGAGCTGCTCGGCT

GGGGAAGTTTGCGAAATCACGTACGATCTACAAGCTGCAGAATCTCTCGAACAACTTCGC

ATAGCGTTCTCCGAATCTTCGGCGGCCGGAGGGGTGCTGCACTTCATGACGGCCGGCGAG

TCCGGTGTTTTCTCCACGGTGCGAACCGGCATCGAGGCGGGTGGACGTCCTGTCGGTATC

GACGGCTTGCAGACATTCGGCGGCGTTCGGGCGTTGGCACGCTACGTCAAAATCGAGGTG

ACCCCTCCCAGCGGCGGAAGCATCATCATCAACGAGGTTGAGTTTCGTGTCGACGACGAC

GCCCCCACGCGTCccgtggaggaaaataaatcgCTGAAGCCCACGGGGCCACTTCCTCTT

GCGCACCGGGGTACATCGTTGGAGGTGGAGCTCGAATACGACAGCAGGTCGCCTGCCCAT

GGCGGCTGCGACTCGCCCTCTCACTTCGAGGGGTGCCACCCGTACTACGTTAAGGACGGA

GATACGAGCTCTCGTTGGGCGTGTGGTCCTCTAGCGATAGGAACCGGCGTCACCTCCAGT

GACAAAAGTTGCCGGCTGGGTATGGACCTCATCTACTTCCGCTACGTCCGACAAATTCAA

ATAGCGTTCGACATGGGCGACGAGCAGCACGATGAGTTCAGCGTCGAGGCGCTCACTATA

CAGGGATGGATGACGGTGGTGGCTTCAGCGATCAGCTCTGGTGATACTACCGACTACCAG

ACATTCGACGTCGCCGTGCATGCCAGGGAGATCGTTGTGGAGCCCAAATTCAAGCACTTG

AACCACTGGTTCAGCATCAGCGAGATCATTATCCTTGAAGAGAGAAGGAACGACTTCATC

GCGGGAACCGTCCCGGTGTTTGAATTTGCTGGGCCATTTGAGTTCGACAGTGATGACCAA

GCGTCTCAGGTCGAAATTCCTACGAGGTTCTCATTCGACATTCCCAGGGCAGACGACAGG

GTCAACGTCTACATGGAGGGTGCGACGGTCACAGCTCTGCGGATGCGCTTCCCTGCGGAC

AGGCAGTTTGTGTTCGAGGTGGATGCTAACAATGACCCCCAAATTTTCACCAGCGCCGGA

GGTGGCAATGTTTGGGAGACGTTCACGTTGTCGGAGGCCGTGGTGGTGCATGGCAGGTTC

GAGATCACGGCCGTCAGCGGTCCAACGTTTGCCAACAAACCGGATTACCCAGCGCTGCGG

GTTGTCGACTTCCAGGTTGTTGGCGAGCTGGAGAACAACGGCCCGGGATACTTTAAGATG

ATGACGACAACGATTCCTGAGTGGCGCGGTGTCCCGGACGTAATCGGAGACGGGATCTCC

GAGCAGGAAGAGATCATGACGCTCATCTGCGAGACGAAGGGTGCCACGTTCGACGGCACG

GACTGCGTCGGAGAACTCGACGAATCTACCGTGCATATCGAGTTCGAACTAGGAGAGTAC

TTCCTGGACGGACCCATATTCATCAAGAGCGGGGTAACACTTCAAGGAAAATATAGTTCC

GAGTTTTCTTTCTACACGGAGTTCGTACTGTACGATGGCGTCAACAACGAAAACACTGAT

GAGGATGCCATGATAGTCATCGACAACGTCACGGGAGCAACGGCAAGTATCATTCCCTGC

TTGTCTGCCATTTTTGTGTCCAAAATCGATATGCTACGAAAGACGGACTGGACCGGGGAC

ATCGTGCCCAACACACTTGGAAATCTTTGCTTGGCCGTCAGGAACTCCAAGGATATTTTC

TTCTTTGATGTTGCTCCGCGGGAATGCAGAACAGGCGCAGCCCGGTTCACGGACTCCAGC

AACATCAATACGTTCCTCTTTTTGGAGTCGAGGCAAGAGACCGGAAACTACATGGAGCTC

ACGCGGGTGGATAACTTCATCGTGGACGGGTTCGGGGACGTCGGTGGACTGCTCATCGAC

ACATGCAACAACGTTACCTGGGGAGGCCACGAAGGCGATGGCTACCCTTCTccaaaccct

cgcatccttccGCCCGTCGGGGGTGAACAAATGGCGAGCGTTGTTGTCACTGGCGATTCA

TCTGGTGTAGTGTTCAAGGAGTGCTACATCGGGCCTGGTGCAGAGCCGCGCATCCTCATG

GAGTCAACAAAGCCTCTTACGCTGGAGAACATATTTTCTTACGAGGAAGGTCAAAGCGGA

GACTGCATCGTGCAAGTGCCAGAGGGCACAACCGAGGATTTGATTGTTCAGGTCGATTCG

GAGCAAACCTTGTCCAAGTCTGGTGACTGCTGGGTCCTTGACTAG

>g12127.t1

ATGGACCGTCGAAGCAAGTCGGATCCGTTCGTTGTAGTGAAGTGGAAAACGGACACTGAC

GACACTTGGAGGGAAATAGGGCGAACGGAGATAATTTCGAACGACCAGAACCCGAAGTTC

ACGGCGCAGTTCACCTTCACCTATATTTTTGAGGAGGTCCAGAGCCTTAGGCTCGAGGTG

TACGACGCGGATACCGCGTACAAGAACAACGATGCGAGCACAATGGACCTTGCCAAGCAG

GACTACCAGGGCTACGTGGAGACGACGATGGCGGAAGTCCTAGGAACGAACGGACAGACG

GCGACGCTTCCGCTGCTAGACCCAAAGACCGGCCGGAAAGGGCAAGGGGCATTGTCCATC

CGTTTGGATGAGATTGAGCACCAGCACGACCTCGTCACGCTCAAGCTGCGCGCTAAGAAC

GTGGATAAGAAGGACCTGACGTCGAGCGATCCGTACCTTAAGATGGCGCGCGTGTTGGAG

TCGGGGGAGCAGACCTACGTCTTCAAGACCGAGGTGCAGAGCCGCACGCTCAACCCGGAA

TGGCGGACAATACAGGGTACGGTCCAGCAGATAGCCAACGGAGACCTCCTGCGGCCGCTG

CGGATCGAGTGCTGGGATTACGACCGAGCGGGCGGGCACGACTTCATCGGCAGCTGCAAC

GCTTCCATCGGCGACATGCTGAGATGGTCTACAGCGACACTTAATGACGTTAGGGGACCG

GCCCTGATCAACCCATCGAAGCAAGCCAAAAACCCCGCGTACGTGGACTCGGGCAAGCTT

CACGTCGACCTGTGCACGATCACGGAGCGCCCGTCCTTTTTGGATTATATCGCAGgcGGG

ACGGAGATTTCGTTCACAATGGCGATCGACTTCACAGCATCAAACGGGAACCCAGCCGAC

CCGCGATCTCTCCACTACGTGGACCCCACGGGCATGATtctcaacgactacgccaaggcC

ATGAGCGGTATCGGGCGCGTGCTGGAGTTTTacgATACGGACAAGCTTTTCCCGGTGATC

GGGTTCGGGGGCAAGCTGGGGCCGAACCAGCCGGCGAACCACGCATTCGCGGTGAACTTC

AATGAAGACCACCCGGAGGTGGAGGGCATGACGGGCGTGCTTGAGGCTTACTACCAAAGC

TTGAGGAAGGTGCAGCTGTCGGGTCCCACGCTGTTCCAGGGCATCGTTAGCCAGGCGGCG

GAGATAGCCAACAGCTCCAAACACTTCGACCCCGTGGGCCAGAAGTACCACGTTCTCTGT

GTCATGACGGATGGTATCATCAACGACATGGACGGGACCATCGCGGCCATCGTAGATGCG

GCCGACTCGCCGCTGTCCATAGTCATCGTCGGGGTGGGCCAGGGCGACTTCGCGGCCATG

GAGCAGCTGGACGGAGACAGGCAGCGCCTCACCTCCCCCTTCACCGGCAAGGTGGCCAGC

CGCGACATGGTTCAATTCGTGCCGTTTCGCGATTTTAACGGTTTCGGTTCCGCCGCGCAG

CACGCCCTCGCGAAGCACGTTCTCGCCGAGATCCCCGGTCAGTTCATACAGTACATGGAG

ACGAACAAGATTGCCCCGGCCAATAGGAGGCCGCCTGGAGCGGTTTTGCCGGTAAcgccc

gcgcagcagcagcagcagcagccgccgccgctaggGGTTCCTGCGGCTGTTCCTCAGGTG

GGCGGTGTGGGACAGGAAGCCTCGGCTTCGGTGCCACCACTACCAGGAGCGGGGTCGCAG

CAACCGGGAGATGCGGGCGATTTGCCGCCACCGTATACTGTCGAGTAA

>g12247.t1

ATGAAGACTTCGTCGGCCCGAAGCCTTACCCTGGTGTCCGGAGTAGTTTGCGCGTCTCTC

AGCGCTCTTAGAGGCGCGGAGGGGCAGGCGACCAGGAACACCCAGTACCAGGACGCGACG

TGCGAGGTGGAGAATGGACTCGTCATCCTGAACTTCGGGGTCGGCAGCTTCGAGGAACCC

TACACCCTGAACGCCTTCAACGAGTGCATCCCTGGCGACATGATGTCCTCCATCTCTGTG

TGCAACGTGGACAACAACGGAGAGGCCGGTTTGAACTTCTACTCGGACGAGACCTGCGAG

ACCGTCTCTATCGAGACAGCCGCGGTGCCGTCGTGCACGGAAACCGTGCCCGGAGCGGAA

TGCACCAGCAGCTACGCGTGCGCGACGATCACCGAGTGCACGAATGATGaaccaTCGGCC

ATCACACCCAACTCGTACAACATCAGGGCCATCGAGTACGAGGACGCCGAGTGCACCACG

CCCACCGGCGCCGCGGGAGAGACCGTCGTCCTCGCCTCCGACGCCCTGGGGTCGGTGGTG

GCCGACGGAACCTGCGCGGAGCCGATCGAGGGGATGGGGCACTACCTGAGCCAGTGCAAC

GACGACGGGAGCTTGCTCTCGCAGACCTTCTCGCCCGAAGACCACACGTGCGAGGGGGAC

GTGTGGGCGGAGACGAGCACTCCCTCGGACTGGTCCGCGGTGTGTGCCACGGTCGAGATC

GACGGCTTGTACTACAgCTACGTCTCCTGCACCATGGACGACGTCACCGTCGGAGGCACG

AcagacgccaccaccaccgccgccccgaCGGACATGGGCAACAACACCCCCACCCCGTCG

ATCGCCGGCGGTGGCGACAACGCGGTCGAGGTGACCATGACCATGGCCCCCACGGAGTTC

GGCACCACGATGATGCCCACGGCCGCGTCCGACCGAAGTATCGTCGACTCCGAGGAGGAC

AGCGAAGGAATCGACGGGGCTAACGGAGCCTCGGGAGCCGGCAACGGCTTTTCGGCCGTG

GCGACCCTCGTGTTGTCGTGCGCGGCGGGTCTCTTGACTTTCATCGCTGCCTAA

>g12300.t1

ATGTGCTTATCAGACGGGTCGCCCGACCCCACGCCGGTCGGCGTGGGTCGTCTACCATCT

GTGGAGCCCTTCTACCTCAAGAAGGTGTTCATTTGGGTGCCTGAGTACATGTGGGACGAG

ATCGAGCACATACCCTGTCCGCATTGTGGAGGGCGTGCGCAACCGGATGGGTGGAATAGT

GAGCCACGCCGGGTCTATCTGGAGGAGGATGTTTGCTACATGATCGGATTCAGGTATTAT

TGCAAGCGGTGCACGGACGCCAACCAGACGAAGGATAAAGAGGAGCGTACAACTGTCACG

TTCAGTCCGTGGGATGCTGACGTTTTGGCCAGGATGGACGACTTCGTGTCTTTGGAGTTC

CCGTTCGTCCTGACCAAGAAAGCCGCTATCTGTAAGAGCCTAGTTCATCGGTTGTCAGAC

GATCTCCTGGAAGGGAAGGGCTTCGCGGCGACTTCGAAGTCGCTTGAGAAGGCGTACTCA

GCAACGTACCTGAAGAATTACCGTTCGTACGTGTCGTTGATGAACAGCCGTAAAGCCCAG

CTGAAGGGGCTTTTTGGGAAGGATCCCGACGTCGGCCAGATCTCAATGTTTGGGTGCATT

GAAGACCCTGCGGGGTTCAACAGCAACCATCCATCGGCTCACTATCTGAGAGACATCTGG

AACAAGTGGTTCTACGAAACTCCAGTTGTACAGGATAACGGATGTACGTTGACACGTGAA

GAGTATCTACAGCGTCGAGCTCAGCTGGTAGACGGCCGTGTAATGGCAGGCGATGCTTCC

TTCAAGTACGCCAAGGTCATCCGGTTAGCTACAGGGCAAGATGGGACGCGTGCCCGGCCT

GTGTACGGCATATTCACGATCATGAACGAGTACGACCAGGTTGTTTTTTCGAAGGCCATG

AAGACTGCATCAGTCTATGATCTCAAGAACGACCTCAAGATTATGTTCGTCAAGCGTTTC

AAGGGCCATGGTTTCGACCTCCCGGTGATCTTCTTCACAGATGAATGTTGCGAGGATCGA

AGATTGCTGCTCACGATGTTCAAAGAGATACGATCGGAGACTGGCGTCCACCTTTACGAG

GCTGCTGATGGCGATGACCCAGGAGCTGAAGGGGAAAGTTTGCCAAAACTGGCGTTCCCC

TCGGGCACGTGCGGGCGGTATGTGTGGGATATGAGTCCCTCCGTCGTAGCTCAGTCAGTC

TCTACCCTTCTCCGCGGCTGCCGCCAGCAGGGGATGGTTTTGGGTTTGGACTGTGAGTGG

GAGTTATCGCTGGGCGGCGCACCGCCAAACCCTGTTTCAACTGTCCAACTCTCGCTCCCT

GGTGGCACTGCCTACTGCTTTCAACTTCAACGTGGAGAGAGGAAAACTACGAAGGAAAAT

TTTCCGATTGCCCTGAAACTTCTCCTGGAGGACCCTTCCATCCGAAAGGTCGGCGTCAAT

GTCAACTTGGACGCGACGTTTTTGGCGCGGGACTACGGCGTCACTGTCAAGAACACTGCG

GACGTACGGGCTCACGCAAGCGAGTGTTGGGTGGAGACACCATCCCGTTCGCTGGCGGGT

ATGGTGTCTTCTTTGCTTGGGAAAGAGTTGCCCAAAGACCCGACCGTGCGTTTGTCTCGC

TGGAGCCATCGGCTCGACGACAACCAGGTGCAGTATGCCTGCTTGGATGCGTTGGCGTCC

GTTCTTGTGTTTCTGGAGATCGAGAGACTGAAGGATCCAATACGCCGCCCGGCTCCAACA

ACCTTGCCGCCGGGAACACAGGTGCGACTTTACAGCAACAACCACGCcagttgtgttggt

gttggagaaGTACAGGACGACGAGACCGCAGCTAGAAGTTTGCATCGATGGCGTCCTCGA

TCGCGGCAAACGGGCGGCTACAAGCGCGTCGTTGTCAAAGTGAGCGAGGCGTTGAAGCCT

GCTGCTTTCACGGCACACAAGGGGGCACCTCCTGCCTCCACGGGTGTGGGTCGCGTGACG

ATGAAGGATGTctgcgacggcgacggcgacagACTTGTTCTGTGGGACGTTGCCCATGTT

CGTCTCGCAGCGGATTGGACAGCACCCCCTGCGACACCATCGCCTTCAGCAAACCCGAGC

ATGAGTGCCGCTTACGACGATGATGCCGACTTCTTCGCTGCCCCACGACCTTGCCGCGTG

GTGGGCCCGCCAGGTGACGTAGACTTGGACGAGGGCGAAGAGGACGGTGACGACGGGAGT

GACTGTGATGATGGATGCAGTAGCCGTGCTTGCAACCTAGGACCCGGATACGATATCGAC

AGCAATGTGGACATGGACCATATGGATGGCATGCGCATGCCCAAGGTTCGCCTGGATGTA

TTTCACGCGCTTCAACGTATCTCGCGGCTGGTGAAGAAGAACCACGGTGCTTTCAAGCCC

TTCATGGCTCGATTGAGAGACGCCTTTTTTATAGTTAATTCGGAAGACATCAAGGAGGTA

GAACGCTCCCTTGAAGCGCAAGGAATGAACCCGGAAGAAGTGGCACAGTACAGAGAGAAG

AATTGGACGTATTTCTTGCGGAATTGTCGGCGGTTGGTGCCGGAGCGAAAACGATTGTTG

AAGCGCTTCAATTCAGTGATTGACCAGTTCTGGGACGTGATCGACGCGAAGTCGGGAGAA

ATTCTCCTTCGGCCGAAAGCAATGGAGGCCGTTGACCTGCTCCGCAAACACATCGAAGCT

GACTGCCTCAGCGACCCTGACGCCATTGCGCTGTACTACACTACGGGGAAAAATGCAGCG

GGGATCACTACTCGCCGCTGTGTCAGAGGGACCAACAGCACTgagCGATATCATCGTTAC

CTCCGCAGGCTGCTATCGTCGTACTGCGCTTCTCCTGCCCTAGCACACTCCATTCTGCTT

GAGTTCAACTACCACTGGAATATTAGGATGGCCGTGAAGAACCGCGGCCTGCCGAAGGAG

GTCGGAGGCTTTTACGACCAGTTTGAGATAGAGATAATCCAACGCGACACGGCTAGCTGG

TGCCCAGACAACCCGCTGTTTTCTGATTGGGTCTCTGCACTTGACGTGGCAGACACTGGT

GAGAGGAGTGGCCTTTCTCAGAGTTCGTGGGGACCTGATGTTGCTGGCGGTCTTCCGGTA

GATATTGTCGGCGAGAGAGAAAAAGTCCAGAATGACGTACCCCTCGCCAGACTCACGCAC

TCCTCGAAGCAATACTCTCGAATGATGAAATCCGGACTGCCGTATACTAACGTGCACAAC

GGAGCAGAGAGGCGTAAATTTGCCAGCGAAGTCGACCGCCACCTCTTTGTCCAAGGTGAT

GAGATGGGGAGGATGCAGAGATACCAAAGCATAAACTTTGATAAGTGGGCTGTTGAGTGG

AATGACCACTGTGACAAGATCGAGTCTGGCCGGGTGGAGTGGGAACCCGTATATCGGAAA

AACTCCAAACAACTGCAGCTCTACCACGCATATCACAAAGCGAAGACCAACGCTATGCTT

ACGGAACGCCCGATTCATCAACAGCATGCTGAACTGCGTACTAACTTGCAGGAACCTGCA

GGGGGGGCGGCGTTTGCTGGTTTAGTCGGCACCGTAGTTCCCACTCCTGTTCCACGGAGT

TCTTCTGGGGCAGGcattggcggtggtggcggaatTAGTGAAGGAGGGGGTGCTGCGGGC

GGTGGGGACGTGGGGGTTGTCGCTACCCCACCGAAGCCGAAGAAGCCCAAGGGCCCACCG

CGGGGGCCTCAGCATTGCTTTCACTGCGGCCACCGTAGACAAGAAGGCGGGTACAAGGAG

GCGCACGCTACCgcgcgagagaggggggggcgacccCCATGCAATGTACCTCCAGGAGAG

TACCGGCCGGATACCTTCAGAACTGGGAAAGCTAGACCAGACGGACAGCGGTCGTGGTCA

CGGTGTGATTGTGGGAAGTGCACAGCAGGATCCATAAACGTTGCTTGA

>g12305.t1

ATGGCTCCCAAGCTGATCCTCACGTACTTTGGCGTCGAGGGCATCGCTGAGGAGATACGC

TGGGCATTGGAGCTATCAGGTCTCGAGTGGGAGGATAAGAGGCTCACGCGGGAAGAGTTT

GGCGTTCTCAAGCCCAGCCTACCGAACGGGCAGGTGCCTGTGCTCGTGATTGATGGCTAT

GTCCTCCCGCAGAGGTTGGCTATCCTTCGCTATGTGGCAAAACTTGGCGggctgttcccT

ACCGATGACTTCGAAGCGGCCAAGTGCGACGCTGCGATCGAGTTATCCACCGACTTTTAT

GCGCAAGGCGTGCCATATTTCTCAGAGAAGGACGAGACGAGGAAGCTGGAAATGCGCAAG

AATATGGTGGAGAACTTTTTTCCCACGTGGCTCAAGAATATCGAGAAGCACCTTTCCAAA

ACTGAAGGCGTCTACTTCGGTGACAAGATGACTGTGGCTGACATTACGGTCGCTCGCCTG

ATCAAGTCTCTCAAAGACGGCGCTCTGGACGGCGTTCCCACCGACATCGCCGACTCCTAC

CCTAAGGCCATCGCTCTCTACGACAGCATTGTCGGAGAGCCGAAGATCGCAGCGTTCATA

GCCAAGCATGCCAAGTAG

>g12309.t1

ATGACAAGCCCTGGGGTTGCTCGTATGGGTTTGTTTACCGTAAAGCAGAACGTGGCTGAC

GATGACCTGGGTTTGGACAAAACGTGGGATGCGGAGCCGGTGTCGGCGGGGACAGCGCTG

CGGGAGAGCGGGTTGACCACGATTCTCGAGGCCGCGCTGGCTAGCGCGAGGTCGAGGGAA

GGAGACGGGGCGGTAATTTTTGTCGGGATGGACACGCCCGAACTGCCTTGGTCCGAGGTG

ATGGCCGCGAAGAGCGCGGCAGAGAAGGATGGCAAGGCTTACATTTGCCCAGCCTCCGAC

GGGGGATACACTCTTCTGGGTCTGCCTCCCACGGCGGGAAGGGGAACATTCGAGGGCGTC

AAGTGGTCGGATGCCCTGACGTGCAAGAGCCAAACGTGCGCGCTGGCCTCTCGAGGGGTT

TTGACGAGAATTGGAGGCACGTACCACGACGTCGATGAGGCGGACGACGTGGCGGGGCTG

GAGAGGAGATCTCCCCGGGGTTTGGCGGAGTCGCCTTGCCCGCAAACGTTAGGCGTGGCC

AGGACCGCGCTCAGGCAGAGGCATTTGCCTCGCGTCTGGGTCGGCTAA

>g12639.t1

atggccaGCCTCGCATCTACCCCGAAGACCATCCTTGTGGCCGGTGGAGCTGGATATATC

GGCAGCCACACCGTTCTCCTGCTTCTGGAGGCCGGCTTCAATGTCGTCGTCGCTGACAAC

CTCATCAACTCCAGCGAGGAGAGCCTGAAGAGGGTGAGGATCCTGACGGGATGCGCGCCG

GAGAGGCTCGTCTTCCACAACGTCGACCTTTGCGACGCCGAGGCGCTACGCGTGGTGCTG

GAGGCCTGCCCTCCGTTCGACTCGTGCATTCACTTCGCTGGCCTtaagGTGGCCGTCGGT

AAGCGGGAATGCCTCACCGTGTTCGGAGACAAGTACGACACGAAGGATGGCACGGGGGTG

CGCGACTACATCCACGTTGTCGACCTTGCGGCGGGGCACCTGAAGGCGCTCGACTTCCTC

GAGGAGAAGGGCCACGGCTGGTTCACGCACAACCTCGGCACCGGGACGGGGTACTCCGTG

CTTGACATGGTGGCCGCCATGAAGAAGGCCTCGGGCAAAGACATCCCCTACAAGGTTGGA

ACTCCTAGGACTGGGGACCTGGCTTGCGTCTACGCCGACCCTACAAAGGCGGCGGAAGAG

CTCAAGTGGAAGGCCCAGTTCGGCCTGGAGGAAATGATGCGGGACATGTGGGCGTGGCAG

AGCAAGAACCCCGACGGGTTTTCGTCTGCGTAA

>g12640.t1

atgGGCGACCTCTCCACCACGAACGGCCAGGTACCCGAGGACCTGCGCGCGGCTTACGCC

GTGCACGGCCAGGAGCAAGTTTTCGAGTACGTCGACAGCGGGGTGGTCAAGGCGCCCGAG

GAGATCGACGCCCTCGTTGCCCAGCTCCGCTCGATCGACCTCCCTCGCCTGCATGATCTC

CACAAGTCCACTGACTCTCCTACTGCACCCGAtgctgcctcctcctcttctcccgaGGAG

GCCCCCATTGAGGAGATGAAGCCCATCGAGTCCTTCGGGAGCCTGGACTCCGCCCCGGCC

GAGGACAAGGACCGTTGGTACAAGACGGGGCTCCAAGCGGTGGCGGAGGggaaggtggcg

gtggtggtgctgagcGGCGGACAGGGCACCCGCCTCGGCTTCTCCGGGCCTAAGGGAATG

TACGACATTGGGCTGCCATCTGGCAAGACCCTCTTCCAGCTGCAGGCCGAGAGGTTGCTC

CGCGTTTGTGCGCTAGCCCGGGGCAGCGACAGTGTCGCCTCCAGCAGCGCACGTATCCCG

TGGTACATCATGACGAGCCCCCTCAACGACGCGGCCACCCGAGAGTTCTTCGCCTCCCAC

AAGTTCTTCGGCGTCCCGGAGGCGGACGTGTTCTTCTTCTCGCAGGGCACGCTCCCCTGC

ATGACGAGGGAGGGCAAGATCATCCTCGAGAGCGCGGGCCGCGTGGCGATGGCCCCCGAC

GGCAACGGGGGCATCTACCCGGCCCTGCAGCAGAAGGGCGCCCTGGAAGACATGCGCCTC

CGCGGAGTGGAACACCTTCACGTGTTTTCCATCGATAACGCCCTCGTGCGCGTCGCCGAT

CCCCACTTCTTGGGCTACTGCGTCGAGAACGGGGCCGACTGCGGGAACAAGTCGGTGTGG

AAGTCGGAACCCGAAGAAAAAGTTGGCGTCGTTGTGTCCCGGGGCGGCAAGCCTTGCGTG

GTCGAGTACTCAGAGATGAGCCAGGAGACCTGCGAAAGGAGGGATGCCGCGAGCGGTAGG

CTTGTGTTCGGAGCCGGGAACATCTGCAACCACTACTTCTCCCGGGGTTTCCTGGAGGAC

ACGGTGCtcccggggatgggggacatGTACCACGTCGCGCACAAGAAGATACCCGCGGCC

GACGGCCCCTCCGGGGAGACCCGCAAGCCTTCCGAGAACAACGGGATCAAGCTCGAGTCC

TTCATCTTCGACGTCTTCCCGCTCTCCAAGAAGATGGTCCTCTTTGAGGCCTCCCGCGCC

GACGAGTTCGCCCCCGTAAAGAACGCTCCGGGGACGGCGACGGACTCGCCTGACACCGCG

CGCGAGATGATCTCTGAGCAGGCGAAGCGCTGGGCGCTGGCGGCGGGTGCCAGCCGcccc

gggggggaggaggggggcgaaggaggcggtggagagggggggggctgtgcgaGGTTTCTC

CCCTGGTTTCCTACGGCGGGGAGGGCTTGCAGGACCGGGTTGGTGAGATGGAGAAGGCGG

TTTTCCGACTTGAGTGATGGATTGGCTCTTCATGATTGA

>g12728.t1

ATGGTTCAGATCATGAGCAACACCCTCGTCCTAGCCGCCGTCGCCAGTGCGTCGCTGCTT

TTTGCCCCTACGTCGGCGGCTCCGTACCCGTTCGGCAGCAAGTTCAAGGGGGACGGCACC

TACTACGGCGACGTCTCGCCCGGCGACGGCAACTGCGCCATCGAGCACCCCATCCCCTCC

ATGTACGACGGGATGATTCCGATGGCCGTTTCTCTTTACGACATGTACGACGACTCGACC

ATATGCGGGGCCTGCATCGAGGGTACCGGCTCCGGGAAGGGGGCAGGGCACAACCCCATC

AAGGGAAAGTTCAAAGGCTTCGTCAGCGACAGCTGCGCGGAGTGCGCCAAGGGCGACCTC

GACTTCGCGAAGAGCGGCGATGGTCGTTGGGACATCGAGTGGAAGTTCGTGGAATGTCCC

TCCGGCGGCAACCCTTCCTTCATTTTTGAGGGGAGCAACCAGTGGTACTGGAAGATCCAG

GCCCGTGAGACTAAGTCGCCCGTGACGCAGCTGTGGGTGGACGGCAAGAAGGCCAAGATG

ACCCAGGACAACTTCTTCACGGCCTCGGGGGGTCCCTTCTATGGCGAGCAGGTGGTGAAG

ACGAAGACCATGTTTGGAGACACGAAGACGACAAAGGTGGCCTTGTGA

>g12767.t1

ATGAGAAGCGCAGGAGCAACGCCGGCCTTGTTCGGCGCCTGCCTCGCGTGCGCAACAACA

GGAGGGCTTTGCTTCACGGCTGTCCCCTGCGTGGCTCCCCCCTCGAGATCAGCCGGTAGG

GTGTCCCGCGCCTCGCGGCCACCATCCACCAGGCTCGACGCAACGCGGCCCTCGTCCGGG

AagggcgacagcaacaacagcagcaggagcagcatcagCGGTGACGGCCGAAAATCAAAC

TTCGATCGATTATTGTCGATATCCGCTGTTGCCCCCGGGGTGTCCTCTGATTCCACCCGG

CGCCAGAGGCAGAAGTCTCGACGGAAGACCCGCCTCTCGTCGACGAAGGAGAACTCGGGC

AGATCGAGCACCGCCACCATCGACAAGTCGCCGGCGCCGTGGACGACGAGCCAGCAGTTC

GAAGCGGAGGTTGCCGCGAGGCGAGCGAGCGGCGCCAGATTCCCGCCAACATCAGGTCAT

GGTTTCGAGGATgaccgcagcagcggcagcagcagcagtgtcggccTCGAACTCTCCGAA

GCCCCCCCCCGAAGCGGCCTTCAAGAGGACGTGCCTAGGTTAACGAGGACAGCTATAGAC

GGcgacggtggcagcagcagaggcagcagcagcaggcgaccCCGCTCCGACTCCAATACC

GGTATGTCTGTAACAAagccaacacaaacaacagctgGTCACCCTAGTAATGTTGCCAGC

ACCACCGCCGCAGAGCGGCAGCGGCTGCTGGAcatgaccaccccccccctagagCTCCGC

GTCGCTGCGAAGACCCGCTCGGTTGGGGAGGGAGAGGCCACGGCGGGCAGCAacgtagtg

gaggggggggtgggggcagaggCGGAGGTGGATAGCGTGGGCGCTGATGTGTGGGCCTCG

AGGGGGATGCTGGCCGGCGCGGCCATGCTGTACGGGACGAACTTCGGGTGCGTGAAGCTG

CTGGAGGAGACTGTGCCCATGAGCCTGGCGGCGGCGTTGAGGTTCTCCGTGGCTTTCGTT

CCGTTCCTGCCATTCTTGACGAAGATCAAGCCTCAAGTTTTCAAGGCAGGCGCTGAGATC

GGCTTGCTCAACGCCGTCGGCTACTGGGCGCAGTCGGAGTCCCTCATGACCACGACGGCT

TCCAAGTCCGCCTTCATCTGCAGCCTCTCCGTGATCTTCGTCCCTCTCATGGACGCCGTG

CTCAACGCCCGAGACGGGAAGGAGGTCAAAGCAGCGAAGGCGATTGAAGCGGCGGAGAAG

AAGAgccaggggggggtgttcgcTAACCTCAATGGCCCCTGGTTTCCGGCGCTCCTGGCG

GCGGCTGGGGTAGCGTGCCTGGAGCTTATCGGGGTCGAGGGTGGTCCGAACTCGGGCGAC

CTGTGGGCGCTCGGTCAGCCTCTGTGCTTCGGCGTGGGCTTCTGGCTGACGGAGCGCTGC

TCTAGGAAGTACCCCGAAGAAACGTTCGGTATCGTGGCGGCGCAGCTGCTGACGGTGGCA

GTCCTGTCGATCGGCTGGTGCGCCCAGGCGGGGCAGATCCCGGTCTCCTTCGAAAGCCTC

CACGAGACCGTCCTGCCGTCTTCCGGGTCGCTGGCCGTGCCCCTGTCGCTCCTCTGGACG

GGGCTCGTGACCACCTCGTTGACCGTCTTCGGGGAGACGTTCGCCATGAAGCAGATCTCG

GCGGCGGAGTCGACGATAATCTTGTCGACCGAGCCTATCTGGGGCACGGCGTTCGCGGCG

GTGCTGCTGGGGGAGACGATCGGGTGGAACACGGGGCTCGGGGCGATGCTGATCGTCGCC

GCGTGCACCTGGAGCTCGGTGGGTCCTGCGATCCAGAGCAAGCTGCTGTCCCTCATTGCG

GCCACGGGCGCGGCCGAGACGGGTGCCGGGATGGACGTGGACGATTTGTCCGATACCGTG

GGGACAGTCTTGAAGGAGCTAGCCAAAGAGAACATCAACCTCTGA

>g12889.t1

ATGAGGCGTCTCCTTTCGCTGCGCACTTTCTCGAGGCATCTCTGCTTCAAATGGTCTTTG

TCAAGCGGGgataggctcttctctctgtcCTGCGTTTTCACGTCCCGCCTTCGCCTCGAC

GTGCAGCTTTGGAGGTGCGCGTGGCTTGGAgtcagcgctgctgctgtatcgtccTTTTTC

TCCTCGAACGCCGAAGCTTTTGTGCAGCCTGCTGCCGGGGGGGCACTAAGGATTGAGGCG

CAGAGCGGTGGGGATGGGAGGGCAGGAACAGTTGTGATGAGCGAGAGGCGGGGGCTGTGG

CCGCAGCGGTCGACCATTGACCTCAGGAGCGACACCGTCACTCAGCCGACGGGAGGCATG

CGCAAGGCCATGCAGAAGGCGGAGGTGGGCGACGACGTGCTGGGCGAAGACCCGACCATC

AAGAGGCTGGAAGCGGAGACGGCGGAACTTCTGGGCAAGGAAGCGGCGCTGTTCGTGCCC

TCAGGGACCATGGCTAACCTCATCGCCCACCTTGTGTGGTGCTCTCAGAGAGGCTCGGAG

ATGATCTTGGGCGACCAGTCTCACGCGTTCCTCTGGGAACAGGCAAACGCAGCGCAATTC

GGAGGCGTCGGGTACAGGTCCCTCCCGAACCTTCCCGACGGCACGATCGATCTCGGAGCG

ATCGCCGCGGCTGTGAGAGGCAACAACCCGTGCTTCCCCGTGTCTCGCCTGGTGTCTATC

GAGAACACGCAAGCGCAGTGCGGGGGAAAGGTGCTTTCGATCGAGTACCTCGACAATCTC

GCGAACCTCGCGCAGgAGGAAGGCCTGAGAGTGCATATGGACGGCGCGCGGATATGGAAC

GCGGCGGCTTCCTCGAACCTTCCGCTATCCCGGATCGTGGCCGGCGTGGACAGTGTGTCC

GTTTGCTACTCCAAGGGTCTGGGCGCGCCCGTGGGCTCCGCCATTGTTGGGACCGCCGAC

TTTATCAGCCGCGCGACTCGCGTGCGAAAGGCTCTCGGCGGAGGCATGCGTCAgtcgggg

gtggtggcggcggcagccttGGAGGGGCTCCGTAAGCAGCTGCCCCGGATAGGGGAAGAC

CACGCAAACGCCTGGCGGCTCGCGCAGGGTTTGTACGACCTGGGGATGCCAGACGCGGTT

GGGGTGGACCCTTCCTCGGTTCAGACGAACATCGTCCTGGCGACCATCGGGCCAGACCTA

ACGGACGAAGGCATGACGGCTTCTGTTGTTTGCGACCGACTCCGTGAGAAGGGCGTGCTG

GGCATGGCTATCATGCAAGGCGTCATCCGTTTCGTCACTCATTCTCAGGTAACCAGCGCG

GACATTCAACGGACTGTTTTGATGATGAAAGAGGTGCTTGAGGAAGCCGGCCTCAGGCCT

GAAGGAGTCAACCGCCCGCCGCGCGCCGCCCCCATCGAGCAGGAACCTGTCGTCGATGAG

CTCGAGTGGCGCTCGGACGACCAGCCCGCAGGTGCCGCggacccccccgccgccgccgcc

accaccaccaccaccgcagcccAGCGCCTCAGCCTTGCTGACGGCCCTGCCGGCCAGCAG

GCGCTGCGCGAGGTGTACACGGGGCCGGACTCCCTGCTCGACGGAGAATCCCCTTCCTCG

GCCGCCGGGGAAGAGACGGCTGCGGTCGAAacggggtcggcggcggcggcggcggctgag

tCGTCGTCGGTGGGTGACGCTgccgcgacggcggtggcgggcgCGGCCGAATTTGGTGGT

GAGGGCGAGACTGGCATGTCTTCGTCTCGTGGGGTTTCGATGGTCAGCGGCGGGAATGGT

GCGGGCActgggggcggcggggagggtttggaggatgaggaggagaagGATGACGCGCCC

CCGTTCTTGGAGCAGTTCGAGGAGGTCGAGCTTCAGGGCATGACCGTTAGCAGCGAGGGC

TTTGTTGCCATCATGACAAGCAAGGAGTCCCCGCGAGCCCTCAAGCTGGTCGTCACGCCC

GACGACCCAATGTCTGGGGGTTTGGACGTGGAGCAGGCGGAGACCTCGGAGGCGCGGACG

CTGCTGCAGCTCATGCAGGGCATTGACGTGGCCCGGCACCTTCCCGGCGACGCTCTGAGC

GACCTCATGGACCTCTCGCTCAACAGCGGCGTCCAGCTCAGCACCGTCTACGTCAGCCGC

GTCGACCCCTTCTCCGCCACACTCATGGCCAAGGTGCCCCAGCGAGACAGCGagaccctc

ccctcccccgccgccgccgcagccgccgcagccaccgccgctgccgctgccagcgATGCG

GCGTCAGCTAGAGCGGCCGCGGGAGCGGACGAAGCTTGGATAGACggcggagcggcggcg

gcagccgccgcggccgcggcggctacCACTGCAGACAGAGGGGGGTTTGATGGCTCCCGG

GAGGCCGGATCGGAGCGGGAGACgctggtgtcgtcgtcggtCCCGGGCACCACGATGGGC

TCCGGGTACGGCCGGCCGCGGCTGGGGGTGTCCAAGATTGCGGAAACGACGAACTCGTTC

GAGGCGCTCGGGTTGGCGCTGCGCTACCCGTCGACGAGGATTTTCGTGAGGGCGGACCTG

TTGgcgagcggtggcgggggcggggggggNNNNNNNNNNNNNNNNNNNNNNNNggcgggg

ggagggggaggggcggggaaaaAGGTGGAGTCTTCGGTGTCGTGCTTTGACTCGGCGGAT

GTGGCGGAGCTGTACCCTAACCTGATGAAGCTTGCCGATGCGAGGACGAGGCGGTCTGCG

TCGGACGACATGAACACGCAGTTCGAGGTGCAGAAGATTCGCCAGCAGCTGGAGAGCGCG

ATGAATGCCGGAGATTCGGACAGAatttcggccctccgggaggAGCTGAACTCGCTCGTC

GTTCCCGACTCGAACACGGCTCCGGTGTCCTAA

>g12895.t1

ATGGAACGCGAGGCATGGGTGCTACAGCGGTGGCTTGCTTTGTGCAGCAAACTCGGCTTC

GACGCCGCATTCACGGAGGAATGGGGTGGGAAAATTATGGATGGATACAGAGAAGAAGGC

CGGCACTACCATACCCTGGACCACGTTGCAGATATGCTGGAAGGCGCTGAGAGAGACTTC

CGCAACCTTGACCGGCCAGGTCTCGTCCAGCTGGCGATATTCTTTCACGACCTCGCCTAC

GACCCCAAGAGCGGGAGCAACGAGGAGGATAGCGACGTGTTATTCCGAAATTTCTCCGAA

GCGGTTGGATTGAGCCCGTCGGATGCGAGCACGGTATCGGGCTACATCATGGCCACGAAA

CGGCACAACGTTTCGGACTCCGAAGACCAGGACCTGCGCTCATTCATCGACCTCGACATG

GCGGTGGTTGGGAGAGAGCGCAGCGAATATTTCACGTACGCCTCTCAGATCCGCAACGAA

TACATCCACGTACCAGCCGACACGTACTGCCGAAAACGAGCGGAAATTCTGCGCGATTTC

CTCAAGACAGACTTCATTTTCGCCACTGACCAGTACAGACGAGATTTAGAGTCTGCAGCG

AGGGCAAACGTGAACGCCGAGGTGGCGTTGCTCAGCACAGGAATCATACCTGGGGATACG

TGA

>g12899.t1

ATGTTGAAGTCCGCATGTTCCTCTTCTTTGggtgcaggagcaggaggagcgatCGCCCCC

GTTGcttgccgtcgccgccgcagcggcagcagcagcagcagcagcagtgtcctaCGTGCT

GGCGCTGAGAAGGAGCCGGAGGTGACTGTGTTCGACGCGGAGGGGGGTGTCTCCTGGGAA

GATTACAAGAAGCAGAAGCCGGACGAATATAAGTTGGACAAGACGGACGAGATGCAGTGC

TGGGAAACGGACGAATGTCCGGTGGAAGACGAGCAGAAGTTCACCAAGGCCTGGCACATA

CACAGCGCCGAGAAAAACCCAACGACCGAAACGCACTTCGACAGCGAGGGCGAATGGGAG

GAGGAAAGTAAAGGCGCATGGTCGAAGAAGTGGTACGAAGATGTCGGGGCCGACGACCTT

CACGAGGAGGGCGGAGGGACCGAATCCCAAAGCGGCTCCAACATTTGA

>g12968.t1

ATGGTGCAATCTCGCACAGGAAAACGGGCCGGACTCGTCGTCGCCGCCTCGGCAGCGATC

GTCGGCCTCCTTGCTAGCGGCTCGCAGGCTGCTCCCGCTGTGACAGAAGAGAcctgcagc

aacaccaacagcaacgaaaCCGTCCTCTCGATCTTCGCCAGGGAAGCGTTCGACAGGTCC

AACAGAGTCGCCGTGGGCCCTACACGTACCACGGAGATCCTGTCGGGAGAGATTCTGGCG

GGTACGACCGCAGTGCCTGCTGGACTCGAAAATGCGGTTATCACCGTGTTGGATGACGAT

GGTACGGAGCAGGTTCTTGAGGTCCAGGTTGCTCCGGTGATTGATTTCGTGTCGTCTGGG

ATCCTCGAAAAGGTGAAGCAGCTTTGCtcttcctcgtcgtcgtctCCCGGCGGTGTCAGC

TGCGACCCCAACGAGACGGACCCTGCGTTCGGCACCACTCCGCTGCACCTGGCCGAGCTT

TGGGGTAGCCGCGACTTGGTGGAGTACCTCTTGTCTATCGGAGCCAACCCCGAGCTTTAT

GATTCGGCGGGTCGTCAGCCTCGGGACATGGCTTACAGCGACTTCACCGCCAGCAGCAAG

GAGGCAGGGGCAGCTCGCCACCCGGAAGGGGCTCACGCAGACGACAGGTGCGAGATTCCG

GAAGTGGTCATCCCTCTGTTCCCGGATGTGGCTACGGACAAGACCGCGAGGACGGCGGAG

GAAACCGCGGCCGTGAATGAGTGGCGAATCTCTTCCAACGCCGCTCTGGCCGAGGTGAGG

CGCTATGTCAGCGACGGCGTGCCAGTTATGACCCGCAACGTTGTCCCCTGGCTACTCGCT

GATTCGCAAGAGCTGGAGAGCGATGAAGGTATCTCAACACTTCAGTACCCGGATGCGGCC

ACCTTCAGCAAATCCTGGGGACACCGCGAGGTGGACGTCCAAAGCGTGTCTTACGCGAAG

AACTTTGAAGCCTCGAAAGAGCGAATGCTGCTCAACGAGTACGACGAGACCGTGgtggca

gctgctgcagctccgGCGGAGACGACCAGTGGTAAACGATACGCGCCAGCCCCGGACTTC

GTTTTCCAGAGGGACACCGAGGTCGTCGGGGAAGGACGCCAGCTGCTGGGGAAGTTCGTG

GAAGCCGCCATGCCGTCTTCCGGCTGCAATCCTATCGTGTGCCCGGCGCCGAGCGGCCTT

TCGGGCCTGCAGAGCATGCGACACTACCGGGGCGGTCCCTGGAGCGGCAACCCCTTCCAC

ATGCACTCCGACGCCCTGAACATGGTGGTGGCGGGCAGGAAGAGGTGGTACTGGGTGACA

CCTCGGGCCGCAATGTGGACTAGGCGGCACATCCAGGAGTACACTGGAGAGAACAAGGGC

AGGCCCTGGACGGATTTCGCAGCCCTCCAGGCAGAGACATCCGCGGGCGACGAGGCGGAG

CAGCTGATGGAGTGCGTTCAGCGCCCTGGTGACGTCATGTACATCGCCGGCGGTTGGGGG

CACACCTCGATGTTCATTGACGACAACACGTTCGGGTACACCCTTGGACTGATCAACCAG

AGAGACACGTTGTCGTCGGTGGTTGGGGTGGGCTGCGAAGAGTAA

>g12969.t1

ATGACACCAACAACGGAGGCGGCCTGCTCTTCGTCCTCAATACCATCGcgtaactactgt

ctgaagtgtaCCACGGAGATCCTGTCGGGGGAGATTCTGGCGGGTAAGACCGCAGTGCCT

GCTGGACTCGAAAATGCGGTTATCACCGTGTTGGATGACGATGGTACGGAGCAGGTTCTT

GAGGTCGAGGTTGCCCCGGTGATTGACTTCGTATCGTCTGGGATCCTCGAAAAGGTGAAG

CAGCTTTGCTCTGCCTCGACGTCGTCTCCCGGCGGTGTCAGCTGCGACCCCAACGAGACG

GACCCTGCGTTCGGCACCACTCCGCTGCACCTGGCCGAGCTTTGGGGTAGCCGCGACTTG

GTGGACTACCTCTTGTCTATCGGGGCCAACCCCGAGCTTTATGATTCGGCGGGTCGTCAG

CCTCGGGACATGGCTTACAGCGACTTCACCGCCAGCAGCAAGGAGGCAGGGGCAGCTCGC

CACCCGGAAGGGGCTCACCCAGACGACAGGTGTGAGATTCCGGAAGTGGTCATCCCGCTG

TTCCCGGATGTGGCTACGGACAAGACCGTGAGGACGGCGGAGGAAACCGCAGCCGTGAAT

GAGTGGCGAATCTCTTCCAACGCCGCTCTGGCCGAGGTGAGGCGCTATGTCAGCGACGGC

GTGCCAGTCATGACCCGCAACGTTGTCCCCTGGCTACTCGCTGATTCGCAAGAGCTGGAG

AGCGATGAAGGTATCTCAACACTTCAGTACCCGGATGCGGCCACCTTCAGCAAATCCTGG

GGACACCGCGAGGTGGACGTCCAAAGCGTGTCTTACGCGAAGAACTTTGAAGCCTCGAAA

GAGAGGATGCTGTTAAACGAGTACGACGAGACCGTGgtggcagctgctgcagctccgGCG

GAGACGACCAGCGGCAAACGGTACGCGCCCGCCCCGGACTTCGTTTTCCAGAGGGACACC

GAGGTCGTCGGAGAAGGCCGTGAGCTGCTGGGGAAGTTCGTGGAAGCCGCCATGCCGTCC

TCCGGCTGCAACCCTATCGTGTGCCCGGCGCCAAGCGGCCTCCCGGGCCTGCAGAGCATG

CGGCACTACCGAGGCGGTCCCTGGAGCGGAAACCCCTTCCACATGCACTCCGACGCACTG

AACATGGTGGTGGCAGGCAGGAAGAGGTGGTACTGGGTGACGCCTCGGGCGGCAATGTGG

ACTAGGCGGCACATCCAGGAGTACACTGGAGAGAACAAGGGCAGGCCCTGGACGGATTTC

GCAGCCCTCCAGGCAGAGACATCCGCGGGCGACGAGGCGGAGCAGCTGATGGAGTGCGTT

CAGCGCCCTGGTGACGTCATGTACATCGCCGGCGGTTGGGGGCACACCTCGATGTTCATT

GACGACAACACGTTCGGATACACTCTTGGACTGATCAACCAGAGGGACACGCTGTCCGCT

GTCGTTGGAGTGGGCTGCGAGGAGTAA

>g12970.t1

ATGGTTCAATCTCGCACAGGAAAACGAGCCGGGCTCGTCGTCGCTGCCTCTGCGGCGATC

GTCGGCCTCCTTGCTAGCGGCTCGCAGGCTGCTCCCGCTGTGACAGAAGAGAcctgcagc

aacaccaacagcaacgaaaCCGTCCTCTCGATCTTCGCCAGGGAAGCGTTCGACAGGTCC

AACAGAGTCGCCGTGGGACCCACCCGTACCACGGAGATCCTGTCGGGGGAGATTCTGGCG

GGTAAGACCGCTGTGCCTGCTGGACTCGAAAATGCGGTTATCACCGTGTTGGATGACGAT

GGTACGGAACAGGTTCTTGAAGTCGAGGTTGCCCCGGTGATTGATTTCGTGTCGTCTGGG

ATCCTCGAAAAGGTAAAACAGCTTTGCTCTTCCTCGCCGTCGTCTCCCGGCGGTGTCAGC

TGCGACCCCAACGAGACTGACCCCGCGTTCGGGACCACTCCGCTGCATCTGGCCGAGCTT

TGGGGCAGCCGCGATCTGGTGGAGTACCTATTGTCGATCGGAGCCAACCCTGAGCTTTAC

GATTCGGCGGGTCGTCAGCCTCGGGACATGGCTTACAGCGGATTCACGGCCAGCAGCAAG

AAGGAAGGGGCTGCTCGGCATCCGGAAGGGGCTCACCCAGACGATAGGTGCGAGATTCCG

GAAGTGGTCATCCCGCTGTTCCCCGATGTGGTTACGGACAAGACCGTGAGGACGGCGGAG

GAAACCGCAGCCGTGAATGAGTGGCGAAATGCTTCCAACGCCGCTCTGGCCGAGGTGAGG

CGCTATGTCAGCGACGGCATCCCAGTCATGACCCGCAACGTTGTTCCCTGGCTGCTCGCT

GATTCGCAAGAGGAGGAGAGCGATGAAGGTATCTCAACACTTCAGTACCCAGATGCAGCC

ACCTTCAGCAAATCCTGGGGACACCGCGAGGTGGACGTCCAAAGCGTGTCTTACGCGAAG

AACTTTGAAGCCTCGAAAGAGCGGATGCTGCTAAACGAGTACGACGAGACCGTGGTGGCA

GCTGCTGCATCTCCTGCGGAGACCACCACCGGCAAACGATACGCGCCCGCCCCGGACTTC

GTTTTCCAGAGGGACACCGAGGTCGTCGGAGAAGGCCGTGAGCTGCTGGGGAAGTTCGTG

GAAGCCGCCATGCCGTCCTCCGGCTGCAACCCTATCGTGTGCCCGGCGCCAAGCGGCCTC

TCGGGCCTGCAGAGCATGCGGCACTACCGGGGCGGTCCCTGGAGCGGAAACCCCTTCCAC

ATGCACTCCGACGCACTGAACATGGTGGTGGCGGGCAGGAAGAGGTGGTACTGGGTGACG

CCTCGGGCGGCAATGTGGACTAGGCGGCACATCCAGGAGTACACTGGAGAGAACAAGGGC

AGGCCCTGGACGGATTTCGCAGCCCTGCAGGCAGAGACATCCGCGGGCGACGAGGTGGAG

CAGCTGATGGAGTGCGTTCAGCGCCCTGGTGACGTCATGTACATCGCCGGCGGTTGGGGG

CACACCTCGATGTTCATTGACGACAACACGTTCGGATACACTCTTGGACTGATCAACCAG

AGGGACACGCTGTCCGCTATCGTTGGAGTGGGCTGCGAGGAgtaa

>g12972.t1

ATGGTTCAATCTCGCACAGGAAAACGAGCCGGGCTCGTCGTCGCCGCCTCGGCAGCGATC

GTCGGCCTTCTTGCTAGCGGCTCGCAGGCTGCCCCCGCTGTGACAGAAGAGAcctgcagc

aacaccaacaacaacgaaaccgtCCTCTCGATCTTCGCCAGGGAAGCGTTCGACAGGTCC

AACAGAGTCGCCGTGGGCCCTACACGCACCACGGAGATCCTGTCGGGGGAGATTCTGGCG

GGTAAGACCGCAGTGCCTGCTGGACTCGAAAATGCGGTTATCACCGTGTTGGATGACGAT

GGTACGGAACAGGTTCTTGAGGTCCAGGTTGCTCCGGTGATTGATTTCGTGTCGTCTGGG

ATCCTCGAAAAGGTGAAGCAGCTTTGCtcttcctcgtcgtcgtctCCCGGCGGTGTCAGC

TGCGACCCCAACGAGACTGACCCCGCGTTCGGCACCACTCCGCTGCACCTGGCCGAGCTT

TGGGGTAGCCGCGATCTGGTGGACTACCTCTTGTCTATCGGAGCCAACCCCGAGCTTTAT

GATTCGGCGGGTCGTCAGCCTGGCGACATGGCCTACAGCGACTTCACCGCCAGCAGCAAG

GAGGCAGGGGCAGCTCGCCACCCGGAAGGGGCTCACGCAGACGACAGGTGTGAGATTCCG

GAAGTGGTCATCCCGCTGTTCCCGGATGTGGCTACGGACAAGACCGTGAGGACGGCGGAG

GAAATCGCAGCCGTGAATGAGTGGCGAATCTCTTCCAACGCCGCTCTGGCCGAGGTGAGG

CGCTATGTCAGCGACGGCGTGCCAGTCATGACCCGCAACGTTGTTCCCTGGCTGCTCGCT

GATTCGCAAGAAGTGGAGAGCGATGAAGGTATCTCAGCGCTTCAGTACCCGGATGCCGCC

ACCTTCAGCAAATCCTGGGGACACCGCGAGGTGGACGTCCAAAGCGTGTCTTACGCGAAG

AACTTTGAAGCCTCGAAAGAGCGGATGCTGCTAAACGAGTACGACGAGACCGTGgtggca

gctgctgcagctccaGCGGAGACGACCAGCGGCAAACGATACGCGCCCGCCCCGGACTTC

GTTTTCCAGAGGGACACCGAGGTCGTCGGAGAAGGCCGTGAGCTGCTGGGGAAGTTCGTG

GAAGCCGCAATGCCGTCCTCCGGCTGCAACCCCATCGTGTGCCCGGCGCCAAGCGGCATC

CCGGGCCTGCAGAGCATGCGGCACTACCGAGGCGGTCCCTGGAGCGGCAACCCCTTCCAC

ATGCACTCCGACGCCCTGAACATGGTGGTGGCGGGCAGGAAGAGGTGGTACTGGGTGACG

CCTCGGGCGGCAATGTGGACCAGGCGGCACATCCAGGAGTACACTGGAGAGAACAAGGGC

AGGCCCTGGACGGATTTCGCAGCCCTCCAGGCAGAGGCATCCGCGGGCGACGAGGCGGAG

CAGCTGATGGAGTGCGTTCAGCGCCCTGGTGACGTCATGTACATCGCCGGCGGTTGGGGG

CACACGTCGATGTTCATTGACGACAACACGTTCGGATACACTCTGGGACTGATTAACCAG

AGGGACACGCTGTCCGCGGTCGTTGGAGTGGGCTGCGACAAGTAA

>g12973.t1

ATGGTTCAATCTCGCACAGGAAAACGAGCCGGGCTCGTCGTCGCTGCCTCTGCGGCGATC

GTCGGCCTCCTTGCTAGCGGCTCGCAGGCTGCTCCCGCTGTGACAGAAGAGAcctgcagc

aacaccaacaacaacgaaaccgtCCTCTCGATCTTCGCCAGGGAAGCGTTCGACAGGTCC

AACAGAGTCGCCGTGGGACCAACACGTAACACGGAGATCCTGTCGGGGGAGATTCTGGCG

GGTAAGACCGCTGTGCCTGCTGGACTCGAAAATGCGGTTATCACCGTGTTGGATGACGAT

GGTACGGAACAGGTTCTTGAAGTCGAGGTTGCCCCGGTGATTGATTTCGTGTCGTCTGGG

ATCCTCGAAAAGGTAAAACAGCTTTGCTCTTCCTCGCCGTCGTCTCCCGGCGGTGTCAGC

TGCGACCCCAACGAGACTGACCCCGCGTTCGGGACCACTCCGCTGCATCTGGCCGAGCTT

TGGGGCAGCCGCGATCTGGTGGAGTACCTATTGTCGATCGGAGCCAACCCTGAGCTTTAC

GATTCGGCGGGTCGTCAGCCTCGGGACATGGCTTACAGCGGATTCACGGCCAGCAGCAAG

AAGGAAGGGGCTGCTCGGCATCCGGAAGGGGCTCACCCAGACGATAGGTGCGAGATTCCG

GAAGTGGTCATCCCGCTGTTCCCCGATGTGGTTACGGACAAGACCGTGAGGACGGCGGAG

GAAACCGCAGCCGTGAATGAGTGGCGAAATGCTTCCAACGCCGCTCTGGCCGAGGTGAGG

CGCTATGTCAGCGACGGCATCCCAGTCATGACCCGCAACGTTGTTCCCTGGCTGCTCGCT

GATTCGCAAGAGGAGGAGAGCGATGAAGGTATCTCAACACTTCAGTACCCAGATGCAGCC

ACCTTCAGCAAATCCTGGGGACACCGCGAGGTGGACGTCCAAAGCGTGTCTTACGCGAAG

AACTTTGAAGCCTCGAAAGAGCGGATGCTGCTGAACGAGTACGACGAGACCGTGGTGGCA

GCTGCTGCATCTCCTGCGGAGACCACCACCGGCAAACGATACGCGCCCGCCCCGGACTTC

GTTTTCCAGAGGGACACCGAGGTCGTCGGGGAAGGTCGTGAGCTGCTGGGGAAGTTCGTG

GAAGCCGCCATGCCGTCCTCCGGCTGCAATCCTATCGTGTGCCCGGCGCCAAGCGGCCTC

TCGGGCCTGCAGAGCATGCGGCACTACCGGGGCGGTCCCTGGAGCGGTAACCCCTTCCAC

ATGCACTCCGACGCCCTGAACATGGTGGTGGCGGGCAGGAAGAGGTGGTACTGGGTGACG

CCTCGGGCGGCAATGTGGACCAGGCGGCACATCCAGGAGTACACTGGAGAGAACAAGGGC

AGGCCCTGGACGGATTTCGCAGCCCTCCAGGCAGAGGCATCCGCGGGCGACGAGGCGGAG

CAGCTGATGGAGTGCGTTCAGCGCCCTGGTGACGTCATGTACATCGCCGGCGGTTGGGGG

CACACGTCGATGTTCATTGACGACAACACGTTCGGATACACTCTGGGACTGATCAACCAG

AGGGACACGCTGTCCGCTGTCGTTGGAGTGGGCTGCGACGAGTAA

>g12974.t1

ATGGTGCAATCTCGCACAGGAAAACGAGCCGGGCTCGTCGTCGCCGCCTCGGCAGCGATC

GTCGGCCTCCTTGCTAGCGGCTCGCAGGCTGCTCCCGCTGTGACAGAAGAGAcctgcagc

aacaccaacaacaacgaaaccgtCCTCTCGATCTTCGCCAGGGAAGCGTTCGACAGGTCC

AACAGAGTCGCCGTGGGCCCTACACGTACCACGGAGATCCTGTCGGGGGAGATTCTGGCG

GGTAAGACCGCAGTGCCTGCTGGACTCGAAAATGCGGTTATCACCGTGTTGGATGACGAT

GGTACGGAGCAGGTTCTTGAGGTCGAGGTTGCCCCGGTGATTGATTTCGTATCGTCTGGG

ATCCTCGAAAAGGTGAAGCAGCTTTGCTCTGCCTCGACGTCGTCTCCCGGCGGTGTCAGC

TGCGACCCCAACGAGACGGACCCTGCGTTCGGCACCACTCCGCTGCACCTGGCCGAGCTT

TGGGGTAGCCGCGACTTGGTGGACTACCTCTTGTCTATCGGGGCCAACCCCGAGCTTTAT

GATTCGGCGGGTCGTCAGCCTCGGGACATGGCTTACAGCGACTTCACCGCCAGCAGCAAG

GAGGCAGGGGCAGCTCGCCACCCGGAAGGGGCTCACGCAGACGACAGGTGTGAGATTCCG

GAAGTGGTCATCCCGCTGTTCCCGGATGTAGCTACGGACAAGACCGTGAGGACGGCGGAG

GAAACCGCAGCCGTGAATGAGTGGCGAATCTCTTCCAACGCCGCTCTGGCCGAGGTGAGG

CGCTATGTCAGCGAAGGCGTGCCAGTCATGACCCGCAACGTTGTTCCCTGGCTGCTCGCT

GATTCGCAAGAAGTGGAGAGCAATGAAGGTATCTCAGCGCTTCAGTACCCGGATGCCGCC

ACCTTCAGCAAATCCTGGGGACACCGCGAGGTGGACGTCCAAAGCGTGTCTTACGCGAAG

AACTTTGAAGCCTCGAAAGAGAGGATGCTGCTAAACGAATACGACGAGACCGTGgtggca

gctgctgcagctccaGCGGAGACGACCAGCGGCAAACGATACGCGCCCGCCCCGGACTTC

GTTTTCCAGAGGGACACCGAGGTCGTCGGAGAAGGCCGTGAGCTGCTGGGGAAGTTCGTG

GAAGCCGCCATGCCGTCCTCCGGCTGCAACCCTATCGTGTGCCCGGCGCCAAGCGGCCTC

CCGGGCCTGCAGAGCATGCGGCACTACCGAGGCGGTCCCTGGAGCGGAAACCCCTTCCAC

ATGCACTCCGACGCACTGAACATGGTGGTGGCAGGCAGGAAGAGGTGGTACTGGGTGACG

CCTCGGGCGGCAATGTGGACTAGGCGGCACATCCAGGAGTACACTGGAGAGAACAAGGGC

AGGCCCTGGACGGATTTCGCGGCACTCCAGGCAGAGGCATCCGCGGGCGACGAGGCAGAG

CAGCTGATGGAGTGCGTTCAGCGCCCTGGTGATGTCATGTACATCGCCGGCGGTTGGGGG

CACACGTCGATGTTCATTGACGACAACACGTTCGGATACACGCTGGGACTGATTAACCAG

AGGGACACGCTGTCCGCTGTCGTTGGAGTGGGCTGCGACGAGTAA

>g12975.t1

ATGATGCACGGGGAACGGGAGGCTGCAGTCATTATTAGCTTTGACATCAGCATCGAGCAG

AAAAAGATCCTGTCGGGGGAGATTCTGGCGGGTAAGACCGCAGTGCCTGCTGGACTCGAA

AATGCGGTTATCACCGTGTTGGATGACGATGGTACGGAGCAGGTTCTTGAGGTCGAGGTT

GCCCCGGTGATTGATTTCGTATCGTCTGGGATCCTCGAAAAGGTGAAGCAGCTTTGCTCT

GCCTCGACGTCGTCTCCCGGCGGTGTCAGCTGCGACCCCAACGAGACGGACCCTGCGTTC

GGCACCACTCCGCTGCACCTGGCCGAGCTTTGGGGTAGCCGCGACTTGGTGGACTACCTC

TTGTCTATCGGGGCCAACCCCGAGCTTTATGATTCGGCGGGTCGTCAGCCTCGGGACATG

GCTTACAGCGACTTCACCGCCAGCAGCAAGGAGGCAGGGGCAGCTCGCCACCCGGAAGGG

GCTCACGCAGACGACAGGTGTGAGATTCCGGAAGTGGTCATCCCGCTGTTCCCGGATGTA

GCTACGGACAAGACCGTGAGGACGGCGGAGGAAACCGCAGCCGTGAATGAGTGGCGAATC

TCTTCCAACGCCGCTCTGGCCGAGGTGAGGCGCTATGTCAGCGACGGCGTGCCAGTCATG

ACCCGCAACGTTGTTCCCTGGCTGCTCGCTGATTCGCAAGAGGTAGAGAGCGATGAAGCT

ATTTCAACACTTCAGTACCCGGATGCGGCCACCTTCAGCAAATCCTGGGGACACCGCGAG

GTGGACGTCCAAAGCGTGTCTTACGCGAAGAACTTTGAAGCCTCGAAAGAGCGGATGCTG

CTAAACGAGTACGACGAGACCGTGgtggcagctgctgcagctccgGCGGAGACGACCAGC

GGCAAACGGTACGCGCCCGCCCCGGACTTCGTTTTCCAGAGGGACACCGAGGTCGTCGGA

GAAGGCCGTGAGCTGCTGGGGAAGTTCGTGGAAGCCGCCATGCCGTCCTCCGGCTGCAAC

CCTATCGTGTGCCCGGCGCCAAGCGGCCTCCCGGGCCTGCAGAGCATGCGGCACTACCGA

GGCGGTCCCTGGAGCGGAAACCCCTTCCACATGCACTCCGACGCACTGAACATGGTGGTG

GCAGGCAGGAAGAGGTGGTACTGGGTGACGCCTCGGGCGGCAATGTGGACTAGGCGGCAC

ATCCAGGAGTACACTGGAGAGAACAAGGGCAGGCCCTGGGCGGATTTCGCAGCCCTGCAG

GCAGAGGCATCCGCGGGCGACGAGGCGGAGCAGCTGATGGAGTGCGTTCAGCGCCCTGGT

GACGTCATGTACATCGCCGGCGGTTGGGGGCACACCTCGATGTTCATTGACGACAACACG

TTCGGATACACTCTTGGACTGATCAACCAGAGGGACACGCTGTCCGCTGTCGTTGGAGTG

GGCTGCGACGAGTAA

>g13237.t1

ATGATGCTCGCCAGGCACGCGGCAATGAGGCACGCCGGGCAGCGCGCGCTGACCCTTCTT

GCGCCtcgggcggcggcgacgacccGCTGTATCGCGCCGCCCTCCTCCAGCTCGCGAACC

TTATCTTCTTTCCGGGAGCAACGCAACGTCGCCTCCGGTaaggccgccaccgccaccgcg

ccAGTTGGAGCAAGAGCGGTGCAAACCGAGCACCGATCGCTTCGAGGACGCTCGTGTGGA

AGACACTCGTTAGGGAGGGGCGGGTGCGCCGCCGGCTTCCCGCTCGAAATTGGAAGCAGA

CGCGCGTTGTCGTCGGCTTCGCTAGCTAGCGACGGCGAGGACGTCTCGGTCGTCGAGGAC

GGGACGGAGAGTTTCCTCGTCGCTCAGCGGAAGCTGGTGGCGGGGGAGGTGTTGTTTGGT

TGCACCCCCGGCACGCTGTCCGCTGAGAGGTCGAGGCACAGCATCCAGGTCGCCGCGGGC

CTGCACCTGACCGTCTCGGACGACCTGCAGCTGATAAATCATGGCTGCACACCCAACTGC

CAGATGGAGCTGGTGGAAGGTTCTTCTACAACCGGCAAGGGTCCGTGCGTTCGCGCCGTC

GTGATAGCGCCCGAAATTCCAGAGGGAGAAATGTTGACAGTGGACTACAACGCGTTTGAG

GTGGACATGACGAGTTCCTTCGACTGCGCGTGCGGCGCCGTAAACTGCCGCGGCCGCATC

TCGGGCTTCTCGAGGCTCCCGCCCTCCGTCCAACAGGAGTACCTCCAGGGGCGCTGGTCG

ACGTCAGAGGTAGGCGCGGGCGGGTCGGGCGCCCCGCCGCTCCTGACGGAGGTCGTGAAA

GCGTGGGCGGCGGCTAACGTCCGCCGCGGGTGA

>g13502.t1

atgaacgcgcgtctgaaaggaGCGTACGGCTGCGGGTACTGCTCGGCGCACACCGCGGGG

CTCGGAACGATCTGGAGAGGGGATCGTGTTTTCCTGGCCAAGAACATCGCGGCCATGGCG

CCTGAGCCGGACCCAACGATATTCTCGCCGCAGGAACTTGCGGTGATCAACATCTGCAAG

AAGCTGGGCCCTCTTCCGTCGCGAGTGGCGTCCGAGGACATCAAGAGCTTGGCTTCGGTG

TTTTCGGAGAAGGCACAGGAGGCGGTCATCAACTCGGCCACGGTTATGGGCTTCCTCAAC

CGTTTCATGGACACTATCGGCATGACCTTGGAAATGGAGCCGCTCGAGCTGGCCATGTCA

GAGCTGGCGCAGTCGGGCTGGGAGGCGGGAAGCTCTTACGACCCCGTGGCGGATGCCGAG

CTTATGGACGAGGACAAGGTCGAGGCCGCTAAGAGGGAGGAACGGAAGTCGAACTCAGGC

TTCTTTACGTGTGAGTCCAAGTTGACGTTAGAGTTGAAGTTGACTTTAGAGTTGAAGTTG

ACTTCAGAGTTGAAGTCGGAGTTTAATATGCAGATGGAAGCTTCTCGCCTCGTTTTTTGC

CActag

>g13508.t1

ATGTGCTACATCTGCGCGACAAACGCTGGCAACAACATCCTCCGCGCCCACTACGCCTTC

ATGGCCTTCCGCAGCGGCGTGCCCATCCGCCGGCTAGCCACGATCGCTAGCAATGACGCC

GACAGCGACGTAGCGGCTGACAAGGCTGTCGGAGCGAAGGAAGTGGCCGCCATGGCCCTG

GCGGAGGCGGCTAGCGGGACGCCTTCGACCATGTCTCCGACCCTGATTGGGCTGCTGATG

CGGCAGTACTCGCCCGCGGCGGTGATCGAGCTGATCATCACGGTGTCGGTGGCTTTCATG

TTCCACCGCTGGACCTCCGTCTACATGCCGAGAGAGTACGAGCCCGAGGTCTTGAAGTTT

TTGCTGGAGTACGGACCGGCGCTGCGCATCGACCCCGCGCACCCCTGCTCCGAGGAACAG

AGCAAGTGGGAACAGCTCGCCAGTACTGCCCGGGGGACTGTCGGGCACGAATGA

>g13629.t1

ATGGGCATCGCGCCGGACGAGAACGCGATTCGAGAAATACTGAAGAGCTCGGGGCTCCGA

TGCCGCTTACGCGAGGTGGTGGCGACGCGTTCTTGTGTCGGCGACCTCCTCAATGCCGGC

TGTCAGATCATCCACTATGTCGGCCACGGGAACGGGCAGTGCCTGCCGTTCGAGTGTGAC

GACGATCAACACTGCGGAATCATGGAACCGCTCGAGGTGAACGATCTCAAGAACCTCTTT

GGATCAAAGCACGTGGAGACGAAGCTGGTTTTTATCAGCTCGTGCCGTTCGGAGTGGAGT

GGACAAGCCTTCGTCGATGCTGGGGTGCAACACGTCGTAGCGGTCAAGGCGGGGGAAGAC

GTGGAAGACGAAATGGCATACTTCTTCGCGAAAGTGTTTTACGGCGCACTGTTAGGCCCA

CGGAGACACGACGTGCAGGCCGCGTTCGACAGGgcttcagccgttgtctccaccGGGCGA

AGCGCTGGAAACTCCACGGGGCAGGGGGGACCTTTTATTCTGCTGCCTCGCGGCGGGAAC

CACGGCGTTTCCATCGTCGACCAAGTGCCCAGAGGAGAATTTATCGAGGAAACTCTACCC

CGGCCGAAGGTCCACCCGTATTTACCGGGCGCCCGAGAGCCCCACCTGGGCATACCGGCC

CTTCAGAAACTCTTGTGGCCGCTGCTTCGCGGTTCGGTAGCGTGCGTGACGGTGGTCGGC

GAGCGCGGATCGGGTAAGACCCAGAGGGTTATCCAGGCGTGCAAGTACTTGCAAGAGCGG

AGGAAGTTCGGTGCGATATACTTCGCGAGGTGCGACCAAACCATCGAGAGAACTGACGCC

TCTCCATCATGGGGTCGGGAGGCAGTGCGAGACCCCTGCCGGCTGGTCCTCCTCGTCCTG

GACGGCGTTGATGCGCTGGTCGAAAATCGGAATGCGCGACGCAGATTCTTGAAGTTTGCG

AGCTCCCTCTGCTCCGCGAACGGCTCCTACGGGCTCAAGCTACTCGTGACGAGCAACACC

AGGCTGCAAGACGACTCATGCACTTTCGATAGTGGTAGCGAACAGGTATTGCCTGTAGCA

CCTCTGGAGGGCACGGACCCCGCCCAGCTGTTTTTCAACGCGGTTCCACGGCCGTGTGTA

CGAACGGAACTCGGCGTTGTCCCGGGCTGCTCGCACGATGTATTGTTGGAGGCGATGCTA

GGGCACCTTGACGAGGCGAAGGGGCACCCGGGGACGCTGGTGCTCCTTGCTTCGAAAATC

GACGACATTCCCTTGAATTCACCTTCCTTCAAGGACGAGGCAACCAGGCTTTGGAAGTGG

TTCCACACGGCGCCGGGACAGCATACCCTTCCGAACGACGGTGACACGCGCGAAGCAAGT

ACCCAAGGCCACGGCCCCGGGggccacggccaccaccacacacccagGACCCAGCAACAC

AATCCGAGATGTCCgccccatcagcagcagcagcagcagcagcaacagcagcagctgcag

gtaTATCACGAGTACGAACCCCTCACGGGgtaccagcatcagcagcccGGGCAAGACACC

GCCAGTCTGCCGACCCCGAAGGACCCCAGCTGCCAAGCTGTTTGGGTAAAGGCTATCGCG

AAGGCCAATGAAAATGGCATGAGGCACGGTAGCGGGGACAAGTCAACGGCGGACGGCGCT

ATCTGGGGGCGGGCCGACCTTGCACCGTTAGAGTATCTATCCGACGGCCTGCGGGAATTC

CTATACCAGCAGCAGACGAGATCGAGCGACCACAGCCATGCCGAAGATTATGGGTACGAA

TGTCTTCTGGACGGGAGGGTGGATCTCGGGGATTGGGGGACTTATGACGCCATCAACGCG

CCGATGCCGGACGGTATCTACGAAGAGGAGATGCTTTTTGTCAAGAAACGGTTGCTGAGA

GAGCAAGAGAGGGTCATGGGTACGCGAGCAAGGAGCCTAAGGCGAGATGATAATCGCGAC

CACGCGATCTCCATCAAGGCCTTCGTGCGATTCTCCGAGTGGTGGGCGCCGGTGATGGCA

GCGCTGTCGCGACTGAGGGACTACTGGTGGTCGACGGATCTCGTGAGGGTGCACTGGTTC

TTGGGCAGGGAGGCGGCGCATAACATGTTGTTGAATACGACGGAGGGGACGTTCTTGCTG

AGGTTTAGTGAGTCTATCGCGGGGGTCCTCGTTGTCAACTACACGGAGCGAGCACCGAGC

ACGCCCGCGCGTGACACGCCGAGCCTGCGCGTAAAGAAGTGCCTGGTGCGAGTGGATCAA

GGGGGATATTTGAATCACGAATTTCGGGGGAAGCGCTTGGGATCCTACACCTCGCTACAC

GATCTCGTTGTGAGCTATGACCACATCCAGACTCTGTACCCCGACATTCAGAAAGAACTG

GCCTTCCCAGAAGTGCCCCGCTAA

>g13704.t1

ATGCGCATCCTTTTCTTGACCAACGCACACAACGGCATGAGCCAGGCGCTGTACCTCAAT

CTCACGGAGCAGGGCCACCAGGTTATGCTGCACGTTGCGCGCGGGGAAGACGACATGCTC

GCCGCTCACGATTCGTTCGGCCCCGACGCCATAGTGGCGCCCTTCCTCACCAAGCGCGTG

CCGGAGCGCTTGTGGAAGAACAACATGACCTGCCCCGTGCTCATCGTGCACCCTGGTGTC

CGCGGCGACAGGGGGGTCAGTTCCATCGACTGGGCGCTCAAAGattcTATGCCGGAGTGG

GGGGTGACTATTCTGCAGGCCGACGAAGAGATGGACGGCGGGGATGTGTGGAAGTCGTGC

AACTTCTCTGTCCCTCACTCGTCAACTCTCACAAAGTCTGCCTTCTACAACAAGCTGTGT

GTACCAGCCGCCCTGGCTGGAGTTCAGGCTGCCCTCGAAAGGCTCCAGCACGGTGTTCCG

CCACGGCCTTTGGACTACAGCCACACCGATGTGATCGGCACGCTGCGGCCGACCATGAAA

CTAGCGGACCGAGCCATTGACTGGAACACGATGTCGGCGGAAGAAGCAGCCCGGGTCGTG

CGTTTCAGCGACTCGAGCCCCGGCTGCCCGTACACATACCGTGGCACGAAGTACCGCCTC

TTTGGTGCCGTCCCTGAGagtggcgccggcggcggtgcaGAGGGCGCCAGAGAAACGGCA

GAGGTTTTGATGAGTGCGGGGATCCAACCCGGGGAGCCCGTCGGTAAGCGACACGGAGCT

GTGCTTTTCAAGTGCTCGGACGGGAGTGGACTCTGGATTTCGCACATGAGGCGCACCAGC

AACAAAATCACCAATGCGCTCGACCAGGATCAAGTTCCTTCCTTCAAACTCCCCTCGACA

TCCGTTCTGCCGAAGGACGTCGTGGATGATCTGCCTTGTCTTCCCGAGCCTCCTCTGTAC

GTGCCCTTCGGCGAGAGACCGTCCACATTCCAAGAGGTGTGGGTTTCCATCGACAAGGGG

GTCGCCTACGTGCACAGCGACTTCTACAACGGTGCCGCGAGCACCTCCCAGTGTGAGCGA

CTGACGGCGGCCATCCAGGAGGTGGGCAAATTGGACGAGGTCAAGGTGGTACTGCTCATG

GGCGGCGAGCACAGCTACGGGAACGGCATAAACCTCAACACCATCGAAGCCAGCGAAGAC

CCGGAGAAGGAATCCTGGTTGAACATCAACGCCATCAACGACTCCATCAAAGCAGGGTTT

TCAATATTGGACAAGGCCGTGGTTAGCGTTATGCGGGGCAACGCGGGGGCTGGCGGGGCA

ATGGCGGCCATGGCATCGGACCTCGTGTGGGCGCACGAAAACGTAATCCTGAACCCCCAC

TACAAGGCGATGGAGCTTACGGGTGGAGAGTACTGGACGTACTTCTTGCCCGCGAGGGTG

GGAAGAGATGTGGCGATGGAGCTCACCGGCGCCACGCGTCCAATCACCGCCAACCAGGCG

CACGCTATTGGCATGGTCGACGATTTATTGAAGCATTCTGCGAGCTCGAGCTTCCGCGAC

GAGCTGCACGCCATCCGCGATCGAGCCGAGGTTCTCGTGGCGAGTGGGCTGGCCGATACG

ATCATTGCGGAGAAGAAGAGAGACAGGAACGATGCCTGGTTCGAGAAGGTGGAGAATCAC

AGATCGTCCGAGCTGTTGCGGATGAGGGAGTGCTTCTCGAGCCCGTGGTACCATGACGCT

CGTCGCCGTTTCGTCTACAAGACCCCGACGCTGAGCACGACGCCGACACACATGGTGCGT

GAAGCGGCTATCCCGGCGCTGCCGCTCAACGGCAGAGTGGCGGCGAAGGTGATAAGGCAG

CAGATCAAAGACAGAATCGCGATCCTTGCTGGGGGAGGAGACGAGAGCAGCAGCGACGGT

ACCGACGGTGCAGGCAAAGTGCCTGGACTCGGAATCGTCATGGCGAACGGGCGTCCTGAT

TCCAGCATCTACGTAGACGCCAAGATACGGGCTGCCAAGGAGACGGGAATGAAAACCACG

CTTGTGGACATCCCTACAGAAGGCTTGTCTCCCACGGCCTTGGAAGCCCAGGTGTTGGCC

GAGGTGCACGCCCTGAACGAGCGAGATGACATTGACGGCATCGTGGTTCAGATGCCGCTG

CCGGCCGGAGTTAACTCGGACAAGGTGGCTAAGGCAATCGCGGTGCACAAAGACGCGGAT

GCTTGCCATCCTGCCAACGGTGCTAATGTGCTAAACGGAGAGGCCTGGTCCAGCGTTACG

GGGGCATCAGGGTCGCCAGAGGAATTCCGTCCGCTTTCGTGCACTCCGTACGGCATCGTG

GTGCTTCTAGCCATGCACGGAGTTCGCGTCGTGGGGCAGACGGCGGTCGTCATCGGCAAG

AGCAGGATGGTCGGGACGCCGATCGCGGCCATGCTTAGCAACGGTGGGGCGACCGTTACG

CAGTGCGACGTCCACACAGAAAGGACAACTCTTGAGCGCAAGGTACGCAACGCAGACATC

GTCGTGTCGGCCGCGGGAAGGGCTGGGCTTGTCAAGGGACACTGGATCCGCCCAGGAGCC

GTGGTCGTGGATGTTGCAATGAACACGAAAGGTGTCACACCTACCGGCAAGAGGAAGGTC

GTCGGGGACGTGGACTTCGCCATGGCTCAGTCGAAGGCTTCCTACATCACCCCCGTGCCT

GGAGGTGTCGGGCCGATGACAGTAGCTCTCCTCCTTCAGAACACCCTCAACCTCTTCGAA

GCACACCGCGGCAAAACGCCGTAA

>g13705.t1

ATGGTAAGGGAGGCTGCCATCCCGGCGTTATCGCTTAACGGCAGACTGGCGGCAAAGGTG

ATTAGGCAGAAAATCAAGGAACGAATTGCCGTCCTTGCCGATGAAGGAGCCGGGAATGGC

GACGGCACTGGCAGCGGTGGGAGAGTGCCCGGACTCGGTATCGTCATGGCGaacggGCGA

CCCGATTCCAGCATCTACGTCGACGCCAAGATACGGGCTGCCAAGGAGACGGGAATGAAA

ACCACCCTCGTGGATATATCTACGGAAGGGTTGTCACCCACCGCCTTGGAAGCTAAGGTA

TTGGCCGAGGTGCACGCCCTGAACGAGCGAGATGACATCGACGGTATCGTGGTTCAGATG

CCTCTCCAGGCCGGAGTGAACTCGGATAAGGTGGCCAAGGCAATCGCGGTGCACAAAGAC

GCGGACGCGTGCCATCCTGCCAACGGGGCCAATGTGCTGAATGGAGAAGCATGGTCGAGC

GATATGGCCTCATCAGGGTCGCCAGAGGACTTCCGTCCGCTATCGTGCACTCCGTacggc

atcgtggtgctgctggccATGCACGGGGTTCGCATCGTGGGACAGACAGCGGTCGTCATC

GGCAAGAGCAGGATGGTGGGGACGCCAATGGCGGCGATGCTTAGCAGCGGAGGGGCCACC

GTTACACAGTGCGATGTCCACACCGAGAAGACTACTCTTGAACGGAAGGTACGCAACGCA

GACATCGTCGTCTCGGCAGCTGGAAGGGCTGGGCTGGTCAAGGGACACTGGATCCGCCCA

GGAGCTGTTGTAGTGGATGTTGCAATGAACACTCAAGGCGTCACACCGACCGGCAAGAGG

AAGGTGGTCGGGGACGTAGACTTTGCCAAGGCCAGCTCTAAGGCCTCCTACATCACCCCT

GTGCCGGGGGGCGTCGGCCCGATGACAGTAGGTACGTGCACGGTATATGGGGGCGCAAAC

GATGCGCCTCCTCATCTACACGCACGAAGTCCGCGCCAAGAGGGGTTCTGA

>g13706.t1

ATGCGCATCCTGTTCTTGACGAACGCTCACAATGGCATGAGCCAGGCGCTATACCTCAAC

CTGACTGAGCAGGGCCACCAGGTTATGCTACATGTTGCGCGCGGAGAGGAAGACATGTTG

GCCGCCCACGATTCGTTCGGCCCAGACGCCATAGTGGCTCCCTTCCTCACCAAGCGCGTG

CCGGAGCGCCTGTGGAAGAACAACATGACCTGCCCCGTACTCATCGTGCACCCCGGGGTA

CGGGGAGACCGGGGCGTCAGCTCTATCGATTGGGCGCTCAAAGATTCTACGCCGGAGTGG

GGGGTTACGATTCTGCAGGCCGACGAGGAGATGGACGCCGGGGATGTCTGGAAATCGTGC

AACTTTTCTGTACCTCACTCGTCAACGCTCACGAAGTCTGCCTTCTACAATAAGCTGTGT

GTGCCAGCGGCTCTGGACGGGGTGAAGGCTGCTCTGGAACGGCTTCAGCACGGTGTTCCT

CCCAGGCCTTTGAACTACAGTCATTCCGATGTCATAGGTACCTTGCGGCCAACGATGAAG

CTAGCAGACCGCGCGGTCGACTGGACGACCATGTCTGCACACGAGGCGGCGCGGGTCGTC

CGGTTCAGTGACTCGAGCCCCGGGTGTCCTCACACCTTGCGCGGCATCAAGTACCGTCTT

TTTGGCGCGTTTCCTGAAGGGGGTGGTGGCTGTGGCGGCTGTGGGGGGTGTGGAGGTGGC

GGCAAacggtgcggcagcgcaactgATGAGGTGTTGTCGAGGGCGGGGGCTGAACCCGGG

GATCCCGTTGGTAAGCGAAACGGAGCTGTGCTTTTCAAGTGCTCGGACGGGAGCGGACTC

TGGATTTCGCATATGAGGCGCACCAGCAACAAAATCACCAATGCTCTCGACCAAGACCAA

GTTCCTTCCTTCAAGCTCCCATCGACGTCTGTCCTGCCTAAGGACGTGGTGGATGCTCTA

CCCTCTCTCCCTGATCCTCCTCTGTATGTGCCCTTCGGCGAGCGACCGTCCACCTTCCAA

GAGGTGTGGGTTTCCGTAGACAAGGGGGTCGCTTACGTGCACAGCGACTTCTACAACGGA

GCCGCGAGCACCTCCCAGTGCGAACGACTAACTGCGGCCATCCAAGAGGTCGGTAAACTG

GACGAGGTCAAGGTGGTACTGCTCATGGGCGGCGAGCATAGCTACGGAAACGGCATCAAC

CTCAACACTATCGAAGCCAGCGCAGACCCGGATATGGAATCGTGGTTGAACATCAACGCC

ATCAACGACTCCATCAAGGCAGGCTTCTCCATACTGGACAAGGCCGTGGTCAGCGTCATG

AGGGTCAACGCGGGCGCGGGCGGGGCCATGGCCGCCATGGCTTCGGACCTCGTGTGGGCA

CACGAAAACGTTATCCTGAACCCCCACTACAAGGCGATGGAGCTGACCGGCGGCGAGTAC

TGGACGTACTTCTTGCCGGAAAGGATAGGTAGGGACGCTGCGATGGAGCTCACCGGCGCG

ACGCGGCCGATCACGGCGAGCCAGGCCCACGCGATGGGCATGGTGGACGACTTATTGCAG

CACTCTCCGAGTTCTTCCTTCAACGAGGAGTTGTGTGCCATCCGAGATCAAGCCGAGGTA

CTCGTGGCGAGCGGTCTCGCTGACAAGATCATCGCGGAGAAGAAGCAAGAAAGGGACGCT

GCCTGGTTTGAGAAGGTGGAGAAACACAGGGAGTCCGAGCTGTTGAGGATGAGGGAGTGC

TTCTCGAGCCCGTGGTACCATGACGCCCGTCGCCGCTTCGTCTACAAGGTGATATAA

>g13710.t1

ATGGGCACGAGGACCATCGCCTCGAGCTCTACGCTCCTCCTGCTGTTCTCCAGCGTCTGC

CACGGGTTGGTGGAAACGTGCGAGGAGCTGCAGGCGGCGTTCGACCTCACCAAGACCCAG

GATGTCGTCGTCCAGATGCACCCCTTCCAAGAGATCGACTGCGCGGAGTTCACCACGATG

AGCATGGATTCCAACTCTCTCACCGTCGAATCCAGCGAGGGAGATGTCGGCAACTTCTTC

GGAACCTCGGACCTTAACCAGGTCCGGTTTGAGATCACGAACGGGGCGAAGCTGGTATGG

GAGACGAAGGCGAAGTTCATCGGGTCTGAAGGGCACGATGTCAACGGCGGAGCACTCTTC

ATCGGCGAGGGCTCGACCGCTCGCTTCCGGAGCAACCTGGAAATCGTCGACTACGGCATC

CGCAGCGATAACGACTTAGGCGACTACTCTTCGTACGTTCTCAGCGGAGGGtgtgtctac

accgacggctacTTTAGGGTGGACGGCGAGGCTACGTTCACGAACtgcgaggtggtgggg

ggcggggagagcccCCCGGGCCCCGGCGGCGCGCTGTATGTCGGGCCCGAGGGCTCCGTG

CTATTTAACGGCGCACTTGTCATATCGGATGTGTCCATCATCGATGACGACGGGGGCAAC

GGAGGAGCCATCTACAACGAAGGCAAGGTCAACATCAAAGCGGACGCCAAATTCGAAAAT

CTCAGCGCTAGGTCTGGCGGCGCAATTTTCAACGCGGTTGACGCGCAATTCCGCTTCAAG

AACAGCGCGAGGGCGATTTTCATAGACTGCACGGCCAACGACGGCACCGCTGGCGCGCTG

GACAACCGGGGCTTGTTCAAGTTTTCGGGCCCGGCTCTTTTCGTCGACACGTACACCCCT

GCTATATACATTTCGTCCGATGGAAACACCGTACTGTCGGAAAACTCCGTGTTTTGGGAC

AAGTACGACGAGGCCAACTCTCCCGCTATTCGGGTTGCCTCTGGAGGAACGCTAACGATT

CCCGACTCTGTTACTTTCGTTGAAAGCGACTCGCAGTGCAGCAGGGTTGACGATGACACT

TGCCCCTAA

>g13773.t1

ATGAAGTCCGCCGTGATCGCCATCGCATGCGCCGCCGGCGCCCAGGCCTTCGTGGCCCCT

AGcgCGTTCAACGGCGCCGCCCTCTCGACCCCGGCGAAGTCCTCCTCCGCCATGAAGATG

TCCTTCGAGTCCGAGATCGGCGCCCAGGCTCCCCTCGGCTTCTGGGACCCGCTCGGCCTC

CTCGCCGACGCCGACCAGGAGAGGTTCGAGCGCCTCCGGTACGTGGAGGTGAAGCACGGC

CGCATTGCGATGCTCGCCATCGCGGGGCACCTGACCCAGCAGAACACCCGCCTGCCCGGC

ATGCTCTCCAACTCGGCGAACCTCTCGTTCGCTGACATGCCCAACGGTGTGGCGGCTCTG

TCCAAGATCCCCCCGGCGGGCCTCGCACAGATCTTCGGGTTCATCGGCTTCCTCGAGCTG

GCGGTCATGAAGAACGTCGAGGGCTCCTTCCCGGGAGACTTCACCATCGGCGGAAACCCC

TTCGCTTCCTCGTGGGACGCCATGTCCTCGGAGACGCAGGCCTCCAAGCGCGCGATCGAG

CTCAACAACGGCCGCGCCGCGCAGATGGGCATCCTGGGAATGATGGTGCACGAGGAGCTC

TCCAACCAGCCCTACATCATCAACGACCTCCTCGGCGCGTCGTACTCCTTTAACTAA

>g13804.t1

ATGGCGTCGAGGGCCGGCAACAACCACCAGGGGAGAAGCTCGGACGCGGATGGCCCCGTG

GACACGAGAAACTGGAGCCTGTACGGGCGCACGCAGGCTGGCCGCCCGACCCCCTATCTA

GTGGGGCCCATCGTTGGCGCTGTGACTGAGACCACTGGCCGAGTCCTGGTGGAAGTGGAA

AAAGACATGGTCGTGAGGATGGATGCTTTTATGGATGGGACAAAAGTTTGCAGCGTAAAA

CAGAGAGTCACTGGCAAGAAGCCCACCGCTTTCCAGCTGAAGAATTTCCGCCCGGGGAAG

CGTATTCGCGTTTCCGTCAACGTTGGGGAGTTCGGGCAGGAGCGCGAGGCCCGGTTCACC

ACCACCTCTGCCAAGACACTCTGGAAAATCGCATCGGTGAGTTGTAACTGCCACACGCAC

ACGGAGGAGCTCGGTCTGTGGGACCGCCTGGCGCGCTCGGCTGGAGATGTGGACTGCGTG

CTTCACCTCGGGGATCAGATTTATGCGGACGAAGATTTCGGTGCCAAGCACGGTGGTAGC

GCGCGCTTCGAGTCCTCGTGGCAGGCTTGCCTGACGAAGATGGAAGGAGTGGACCGGAGC

GGGTGGGACAACCTCCGGCCGGCGCTTCTGGAGTGTTACAGAAAAACCTACCGCACgACT

TGGAACATGCCTGCGGTGGTGACGGTCCTGGCAAGCGCGGCGAACTACATGATCTGCGAC

GACCACGAATTCGTCGACGATCTCGgggacGAACCGGAGCACCGCGACCCGTCCACGGCG

GAGTTCTACGTGGCCCGGGTGGGCTACCAGGTGTACTGCGAGTACCAGAAGCAGCTCTTG

TTGGACCTCGACGtgACGGACCACAAACTGAGGCCGTACTACGCCTTCAGGCTGAGCCCC

AAGGTCGGCTTCTTCATGACCGACAACCGGATCGAGCGGAGCATTCACCGGCTGGAGGGT

ACCGCCGAGGAGTGGAAGGACCGGGACTTCCTTGGGCCGCGGCAGTGGGATAGGGTCCAG

CAAGCCTTCGCCAAGGATTTCGCCGGCTGCGACACCGTTCTGTTCGGCGCGCCCACCCCC

CTCGTCTTCATCAGCCAGGCGGCGACCGGCGTGGCGGAGAAGGTTATCGACGACGCTAGG

GGCACGTGGGGACACAAGCAGTTCAAAGTCGAGCAAGAGGCATTGGCGACGCTCCTGAGC

AACTGGCAATCCGCCAAGCCTAACCGCGCCGTGGTCACGCTGGCCGGCGACGTCCACATC

GGAGGCTTCACCGACTCGTGGCACAACAACGCGTCTGTGCCTTTGCACCAAATGACGGCA

TCGGCAGTCGGAAACACCCCCGAGAGGGACATCGACAAGCTCAAGGAGGTTCTCATGCGA

GGCGTCATGCACGCCGACGAGCGCCTCTTCAGCTTCAAGGGTGCCCTTCTTTGCAGCGAT

AGCTGCATCGTCAACATCCTACCGCGACATTTGCATCAGTCGCGATAG

>g13837.t1

ATGAGGGCCGGCAGCCTGCTCGGCTGTCTCCTGCTTGGCCTGCCACGCAATGGATCAGCG

TGGGAGGTCGAACTGAAGGAACTCGTCAACCCATCGCTTGTCAAGATTTCGGATAGCCCT

TCCACCGGCCGGGACGAGGCTTGCGCACCCGACGGGTGTATAGGCGTGCTCACGAGGGAT

GGATCACAGAATGTTGGCTCGAGGTACGGCTGCACCCAGCAAGAGCTCGGCGGCTTCCGC

GGCCCCTGCACAGTAAACTACAGGTTGAACGAGGAGCCATACAGCGGTGGAACCCAGTAC

ACAATCTCTTCGGTTCACGTAGCGTTCACGGCGGACAGCGAAACCACTCTCCAGGTTGTG

GCCGGTCGCGATACCATCGTGGAATCATGGACCGGGGAAAAGGGCGTGACCGGCCTGCAA

GAGATACCAGGCGTAGCGGGGTACTCCACAGACGAGTTGGACCTGGTGGCCGTCATGAGC

GAAGGAAGCTACATCGGAATCACAGAAACCGAGATCTACGTGGAAGTTGACGACGTCCCC

GTCGTCGGCATTTCTGCATCCGAGCGCGTAAGCCTTACGGCATCGGCGACGTTCAACCGC

GATGCTGCCATGGACACCCTTGATGGAAGCACCAGTTCAACGTCTTCGTGGTCTTGCTCC

CAAGGAAGCGTGTGCGAAATTACCTACGATATGCTGGAGGTTCAATCCACGGAGCAGCTC

CGCATTGCCTTTGTGGAAGCCTCTACGACGGTGGGAGCTACGTTCAACGTCTACACCGCC

GGTGCCGACGGAATATTCAGTGCCGTGCTCTCCGGAGTTACTGCTGGTGTGCGGGACTCC

TCTCTTGAAGGCCTCCAAACGTTTGGCGCAGTGAGGGCACTCGCGCGGTACGTCAAGATC

GAGGGAGTTCCTGGAAGCGGCGGCGAGTTTTCCATCAGCGAGGTGGAAATTCGCGTGAAC

GAGAACGCTCCGCTCTACCCCACGACCGTGGATAGCTCGTTGGTGCCCCTCGGGCGGCTC

CCTCTAGCAGGCGACGACGGTTCGAACACTGGTGTTCCCTATGACCCCAGGCCTGCCAGC

GAGGGTGGTTGCAGTGCTGGCCACCTGGAGGGGTGCAGCATCTACAACATTAAGGACGGA

GACAACTCGCCGGATTCTCGGTGGTCCTGTTCGCCCTCGGTGAGACACCCCCTCAGTGGC

ACCTGCGACGGTAACTTCGACCTCAACACCCACAGATATCTCCGTCAGATACAACTCGCA

TTCCACAACGGTGATACGCGCCACAACGAGTTCAGCATCCACGAAGCCATCCCGTCCGCC

ATTTCTTCCGGAGACACGACTGACTTCCAGACCTGGGACATTGCAGCGTGGACAGACGCC

ATCCAGCTCCGGCCTAAGTTCGGCAGCGTAGGAGAATGGATTAGCATCAAGGAGATGGTT

CTCCTGGAACAGCAGCAAGGCTCACGTTCAACTCCTATCGTGAACTACTACAACGGTGAA

GCTGGATTCAGCTTTGTGGAGAGCGAAGGTCGGATCTACGTGGGCGGAAGCGGGCAGACC

GCACGCATATGGGCAGTAAAGATGAAGTTTCCGTCTAACAAATCCTTCACGTTCGACATC

CGCTACTACGTCGACGACGAAACCGAAGTCTCCACGCGTTTTACCAGCGCCGGAGGAAAC

CGTCGGTGGGAGAAGTTCGTTCTGCCAGAGAGAGTTATCCTCGACCAATTCCGAATCGTG

GCAATCGAAGGCCCCGACAAGGAGGACTACCCCACGTTGCGCGTCAGCGACTGCCAAGTG

CTAGGCAAGCCCTACTCTCCGGGGTCCACGTGGGTGGTGTCGACCACAATCGAGGAATGG

TCGGTAGTCCCAGACTACATTGGCGACGGCAGCGGAGACCAAGAGGAGATCATGCAGGCC

ATTTGTGATGGCATCGGAGAAGGCTTCGACGGCACTGACTGCGATGGCGTTGCCACAGGC

AAAGACCAGACGCCGGAAGTGAACCTGAAGATGGGAGACTACTACCTGGACGGACCCGTG

TTCCTCAAGAGCGGCATCAACCTCAACGGTAGATGGAGCGAAGACGACTCTCCCTACGAG

ACGCAGTTCCACATGCACGGGAGTGGCACCGGGGCAGACGGGGTCATCAACGCCGACGGT

GTTTCGGGTGTCATAGTGGACAGCATACACGTCAAGGCGAACCTGGAGTCGTCAGGCCCT

GCTGGTACGGTGGGAAACGTCTGCCTTGACGTCAAAAACTCCCAGGACATCAATATCGAT

CTGATGGCCTGGACTGACTGCCCCGTGGCCAGCACTCGTTTCGTCGATTCCGAAGGCGTC

ATAGGTAACGAGTTCTATGTGGGTGGACCTGTCGAAGGCCCCGAGCTCCTTCTGGAGGGA

GTAACGGACTTCATTCTCACGGAGACGCGACTGGACGGTGCGACGATAACAGATTCCGCC

AACATATTGTTTGAGGGTCGTATTGACGATGGCGACTTGAACGGGTCCATCGATGTGCCG

GAGGGCGGAGATCAGTCGGCCAACCTCGTTGTATCCGGAACTACGTCCGGCGTGACTCTC

AAGGATATCAGCATCGGCTCGGGTGCAGAGCCCCGGATCGTGCTCGAGAACTCGAACCCT

TTCACCATCGACGGCGTTAGTATCAATGGTGGCACCGCTGGAGACTGCGTCATTCAATTC

CCTCAGGGGTCCGATGGCAATGACGTCCTTATACAGCTCAACGTGGAGACCGAGCTACTG

CTTGAGGACAACTGCTTCGTGCTGGCCTAA

>g14012.t1

ATGGCGACGACAACCATGCTTCGAACGGCGGCAATCGTGAGCCTCCTGGCGGCCACCAGG

CGAGCCGACGCGGCGGACACCGCTTCGCCGAACGACGCCATCGCCAAACCAGGGTGCGAC

TCGAGCACCACAGTGTCGCTCCGATATTCCTCAAGTTCAGAGCGTGTGTACGTCGAGTCG

GAGGACGGCAGTACTCGAGGTGGCTGCATCACGCTTACAGAGATCTGGGAGTGGTTGGAC

GGCGACGCGCCGTTATACGCCGTGGACCCGGACAGCGGCGATGTGAGCGACACCGCGACG

GGGACATGGCTTCTCACGGAGCACCTTTGGATTGAGGACGGCATAACTCTTGAGGTGCAC

GGTACCTCCGCCGGGGGTGACGCCGACGAGCTGCGCCTGCTGAGCACGAGTGATATGTAC

ATCAACCTGCGGGCCTACGGGGGTAGCCTGGATTTCGTGAGCACCAAGGTGTTTGCCTGG

GACACCGGCAGAAACGACGTGCGCGAAGACGAAGATGATGGCCGATCGTACATCAGCGCC

GTCTCCGAGATCATCTTAGACGACGACGAGAGTTGCGACGGCATGGCCAAGAGCAACATG

GGAGAGGCGCGGATGGACATCGAGGACAGCGAGATGGGATACCTTGGGTTTCACGACGGC

GAGAGTTACGGTCTCACATGGAAGGTACGAGGGTTCTGCAGCGACTTAAGTAACCCCGAG

CTGTTCGACGAGGTCAACGTCTACGGCAACATCTACGACTCGGACATTCACCACAACCAT

TTCGGGGTGTACACGTACGGCCACCAGCAGggagactggaggaggaacaagatgcacgac

aacacggcgtacggcttcgacccccacgacgacagcgacttcctgaccatccacgacaac

gagGTCTACAACAACGAGAACCacggcatcatcgcctccaagcgcTGCAACGACGTTTCC

ATCCAGGGGAACGAGGTGTACGGCGGCGGTGACGAGTCCGTCGGCATATTCCTTCACAGA

AGCGGCGACGACGCCACTGTCAAAGGGAACTACGTCCACGATAACGGCGACGCTGGAATG

GCGATGATGGAGAGTTTCAACGCCGACGTTTCCGACAACATCTTCGAGGACAACAAGTAC

GGCATCAGGATGTCCGTGGGGTGCGCCGACAACGTATTCTCTAAGAACGACATCACCGGT

TCCACTAGTTACAATGTTTACTCGTACGAGGGGTCGGATGAGCCGTGGGTGGTGGATTCC

GGGCGCATGAAGGATAACATCTTCACGGACAACACCATCATCGGTGGTCCGGAGAGTATC

AAACTCGCCTCGGCCGACGGCACCGAGTTCATCGACAACGATTTTGAAGACCCGGAAAAA

ATCCGCTTCGAGGACTGCTCGGGGACCGTCATGTCGGGCAACACCGGCCTGGACGACGTC

GAGCTAAGGATCACCGACGGCGCTTGCTTCGACGATGAGTCGGACTCTGCCTTTACCCCT

GTTTGCTga

>g14174.t1

ATGAGCAGGCAGAGCTTAGCATCATCAGCAGGCAGAGCTTGGCGTCCTATCCGCAACGTC

CTCTCCAGGCGCGAGTCCTCCATGACGAGCACGGGGGAGAGGGTGGCTGGGCGCAAGGTC

CCACGGGAAGcagcaacggcaacgacaATGACGACGGCTAGCCACGTGCTTCCCACGGTC

ACCAAGCCCCCCACAGATGCCGACTTGCCTGCCCCGCTCATCACCGAGTCCCGAGGTCTG

TTCGGAAAACGAGTGACCTACGACAACCCTTGGGCTCCCGGCGCAACTGCCAAGACCGTT

GGGGACCTGTTCAAAGTCATGACATCCAAGGAGGGGTTTGGCGAGTCTCCCACGGAGGAG

CAGGCGAAGGAAGCGCTCCCGATGGCTGAGGTGGAACTGGACGAGATCGCAAGGCACGGA

AAGGATGACCCCATGCGCGTGACGTGGATAGGACACGCCACTTTGCTTCTCCAGATTGAC

GGGTTGAACGTGTTGACAGACCCAATGTTCTCGGACAGGGCGTCCGCTAACCAAATGGTC

GGGCCCAAGCGGTTTGCCCCGCCTGCCATGTCCGTGGCCGAGCTGCCCCCCATCGACGTG

GTGCTGCTGAGCCACAACCACTACGACCACCTCGACGTCGGGTCGGTGAAGGCTGTGGGA

AACGACCCTCTCTGGATCGTGCCGTCTGGGCTGAAAGGGGTTCTGGCAAAGATGGGAGTG

AAGCACTGCATCGAGCTGTCGTGgtgggaggaggtggaggtggaaggGTTCGCGGGCGCC

GTGAGGTTCGCGAGCACGCCGGCCCAGCACTGGTCTACCAGACTCGTCTGGGATCGAAAT

ACAAGCTTGTGGTGCGGGTTTGCTGTTGCCGGAAGCCGAGAGCGCTTTTTCTTCGCGGGT

GACACGGCCTACTGCTCCGTTTTCGGGAAGATCGGGGAGCGGTACGGGCCGTTCGATCTC

GctGCACTCCCTATAGGGGCGCACGAGCCCGCGTGGTTCATGAAGGAGCAGCACTGCTCT

CCCGATGAAGCCGTCACGATCCACCAGGACTTGGGCGCGAAGCGATCTTTCGCGGTGCAC

TGGGGCACTTTTCCGTTGGCGGCTGACCGGTACGCCGACGCCCCGCGAGAGCTCGTGGAG

GCGTGTCGAACACGGGGCATCAAGAGTTCGGAGTTCACCATAGTCAGGCACGGCGAGAGC

ATAGTCAACGACGGCAGGGCTAGACGTTACAAGTGCCTCTTcggtggtgacgatgatggc

gaTATCGTTGATGATGCCGCTGCTGATACCGCTGGTGGTATCGTCGACGACCAAAGATGC

TACGAGTCgtag

>g14201.t1

ATGGGAACCTGCTCTTCGGCCCCGAAAGGAGTGAAATGGGAGCAGCGCTGGTCTTCCCCA

AGCTGCAAGAAAACTCTTCACAAGTTTTGCGACAAGAAGCTGAAGGGCGGCGACGCTATC

GTCACAGGCACCGTTCAAAAGGAATCAGGCAAGCATACCCACAAACACAAATACAAACAA

TATGTGTTAACTCTCCTTTACTGCACCGGCGTGGGTGCGCCGGCCGAGGGATCGAGCTCG

GGTAAGTTCGCGGGTCCTGTCAGCAAGGATGGGGAGTTCCGAAATTGGGCCGGCTCGGTG

CAGACAGCACCCGCTAGGGTGTTCACGCCGAAGAGCAGCAGCGAGGTGCAACAGCTTCTA

GCGCAGGTTCGTGCGCAGATGCCCATGCCAAAGGTGCGGTGCATGGGCTCCAGCCACTCG

TGGTCGCACCATTTTGCTGACGACGGCGCCTGGATGGTGGACACCAGTGGCATCAACCAC

CTCGAGTGGAGCGCCGACCAGCCTAAGCAGGTAACCATAGGCCCTGGCGTCACGGGCGGC

CAGCTGGCCACTTTCATGGACGCCAAGGAACGGGGTATCTGGCGCGGGCTGTTCTTGCCC

TCTGACGTTGTCTTGGAGTCTGTCACCTACGGAGGGGTCATCAGCGTGGGCTGCCATGGA

GCCGGACAAACGCAGACCGTTCCTGACTACGTGCTCAAGACCAGCATCGTCAAGTGGGAG

GGCACCAAGGTGGACCTTGTCCGGGCTGATGACCCGGCGGGCTTCGCCCTTAAGGTCTTG

AGCCTCGGCCTCCTGGGGGTGACGGTCGAGTATACGCTGCAGCTGGACGGGGATGTGAAC

GCAGTGAGAGTGACGGATTCAAAGGTGCCGGCGAGGATGTTGTTCCTCGACCGGAACGAG

CTGCCGAAGCCAGGAGGGAAGAACCCGCTCAGGGAGGCGTGGGCCGGCGGCTACGCCACG

GAAATGTTCTATTTCCCGGCCTCCAGCTTGAAGATCACCAACATCCTCAGCAACGAGTTC

GAAGACCAGAAGTGGGACCCTTACAGCGACATCTGCGCGGTGAAGACGTTCCAacgcacc

aaaaacaaagtgaaGAAGGACAACGATAGGTTTCTCGGAGCGAACCCCAAGACATCTACC

CGATTCGACAGGCACTTTAGCATCATGGACTACCTCCGGATCAACATCGGAGCGGAGAGG

GGATTGCCGCTGATCTCCAAGTTCGGTGACAAGCCGGCGTTGGTTGCGATGTACTCGAGA

ACGGCGGTGAGGGCGCTTGAACCACTGCGCAAAACAAGTGGGATGGTTTTCTACGAGACT

TCCCTCGCACAGGCTATTCACTGGCAGAGATATATCTCGGATGGCCCTCCCGTCTCGGAC

ACCGAGGTGTGCATCAAGTGTGACCCGGACTTCGCAAGTGCCTACAAGGCCATCCACGCC

GCGATCGGAATCGTGAAGGACTTCGCGGAGAAGGAAGACACCATGCCGCTCAACGTCGCC

ATGGAAATGCGCTTCACGAAGCACAGCACGAGCCCTATGTCTCCTGCCTACGGCCGCGAG

GGAGACGTTTTCATCTGGATCGAGGTGCTGTCCTCTGTCAACACCCCTCGGCTGAAGGAC

TTCACAACGAGGGTCGCGGACGCCTGGCTAGCCATCACGCTTAAGGATGGTTCGCAGGCC

GCTTACCCGCACTGGACTAAGTGGTCCGAGGCATACGTGGCCGACGCCGACGCCAAGATG

AAGCGTGCTTACGCTTCTCGCCTTCCGCTGCTGCGGCAGGTGTCGAGGGAGTTCGACCCT

CACGGCATTTTTGTGAACAACTATTTCGCAAAGCTTCTCGCGTCGTGA

>g14406.t1

ATGGCTCGCACCGCTTTTGTTGCTTTGGCTGTGGGGATGATCGGCGCCGCTAGCGCGCTG

AAGGTCAAGACTCCGTCCGAGGGCCTCACCGTCATCGCCGACAGGACGTACACCGTGGAG

TGGGAAGGCACCAGCGAAAACAACCGGTTCGAGATCGACCTGTACTACTGCGGCTCCTAC

TGCATGGAGGACGAGTGCGGTGAGTGGGTGACCGCCCTCTGCCCCTACGGCGAGGAGGGC

TGCCCCGACAACGAGGGCGACTACGACATCATCATGCCCGAGCCCATGTCGGGAACCTCC

GGGTCCGGGTACAAGGTGCGCGTGGTGGAcgtggacgacgaggaggacggcGACTGCTCC

GACGACTTCTACCTCATCGGCAGCGACGAGGCGCCCAAGGTGGGCGACTCGGACGGGCCT

TCGCTCGAGGTCACGTCTCCCGCGGAGGGCGACCTGGCGGAAGCGTGCAACGAGTACACG

ATCGAGTTCGACTACGACAACGGTGTCGGGTCAAGCGTGGACCGGTTCGCGATCGACCTC

TACAAGGCCGAGGGCCACGGCGACTGCGGAACGTGGGTCGCCAGCCTGTGCGACAAGGAG

AGCATCGGCTGCAAGGACTCAATGGGTGACTACGATGTCGAGATCCCGTGCGACACTGAG

CCGGGAGAGTACAAGGTCCGAGTGGGCCGCTTCGAGGACAGCTCCCTGTTCGGCTGCTCC

GGCGCCTTCGAGATCGTCAGCGACGGCTCCAAGGGAGGCGACTCGGACTCCGACTCGGAC

TCGGACTCCGACTCGGACTCCGACTCCGACTCGGACTCCGACTCCGACTCAGATGACAAC

GGCAACGGAGGGAAGGACTCCTCCGACGACATGAGCATGTCTTACTCGTTCTAA

>g14439.t1

ATGGGGGTGACCTGCAATCTCCTCAGCGGGGTCCCCTTTCTGCCTTCTGCCCGGGCTGAG

TGCGTCATGACGGCCGACCCGTGTTCTACTGAGGCCGGCAGTGAGGAGTGCTGTTTCGCG

GAGACTGGTGCCGTGTGCGATACAAACGCCGATGGGGGTTCGTGCTGTTTCCCGACCGTT

GGTGACGACCAAGCGGTCTGCTCCCAGAACGACGACTGCTGCGGTGGTCTCCTGTGCGTG

GCGAGTGTCTGTTCAGACACGATCGAATTCACGGTTGAGGACGTCCAATACACGATGGTG

ATGAGTGAAACTGAGTATGGGTCTGGTGAGTACGTCGCGAGCGAGGTTACAGTGCAGGAC

CCGGACAGTCCTCCAGTTGTGTTTACTCTGGACGGAGATGGGGACGTAACCGAAATTACC

TCTGAGGGGGAGACGCTGTACCCCCTGCCCGACACACCGACGAGACAACTTCAAACATAC

GGGAAGGGCGCGATATCTACGGTGATTGAAACAGCGAACGTGGACGGCATATATCGCAGT

ACGAGGAACCTTACTGAGGGCGGTAGGCTCGCGTGTGAAGCCGCCGCCAACATTCTCTGC

GGAATCGCGGGGACTGCGGCATGCCTAGCGGTGACCAATGTCTTCGGCGCTTTAGCCTGT

GGCGGTTTagcagctgctgcttgcttcgcAGCCGCCGGCGTCATCGAGGACGCCTGCGAA

GATATTGTTGACTGCACAGGTGACCCGCACATGCGGGGGCTTCAGGGCCAGAAGTTCGAC

TTTTCCGGCAAAGACGGTGGTTGGTACGCGATTATGCACGAGCAATCGTTCTTTGTGAAT

ATGCGCGTGACGGCGCCGATCGTCGACCTGGACGAAATTACCTACATTACCGGCCTGGGA

ATATCGGTAACGGACACCAATCACGAACGCCATACAGTAGTGATGACCGTCGACAACCCG

TTCGAGATGCAGCCCGAGTGTCCTGGtgttgataaggcatgcctagcAGACGGGGCGCTG

ACGGTGGAGTTGGACGGTATCAGCCTTACCGAGCCAGGAGAGGTTTCTCTTGGTGGGGGA

GTCTTCTTCGCGGCTGCCAACATTCCTGGTGAATGCCGCCCCTTCGGTTTCGAAAAGTAC

TGGCAGAAGAAGGCCGATGCTCAGGATGCCCTCATTGCCTCTAACGCACGCCGTCTAGGC

TTGGCACCCACCGGCCTATGGATCCTGGCGGACGAACACAAGACCAACCCCGCCGAGTGT

AAAACGTACGTAAGGACTGTGATGGAGGACGGCACCCTTTTCACGCACCAGTCGGAACAC

GCCTCCATCCAGATCCGGGCGGATGATCTGGCTATCCGCGTGAACCACGGGAAGATTCAC

CAGTTGGCCATGCGTGACCCGACCGACCAGTTCGACCTTCCCGACCACATCGCTTACCAG

ATGAACATCGCCTTCGAACGGATCAACGTTGGTGACTACCCCCAGGGTGTGATCGGAGCG

ACTGCTCATATTGCCAAAGATGAGTATGGTGAGGACATCATGAACGGTCCCGGAGCAATC

CCTGGCGAAGAGGAAGACTACCGCGTGTCGGGGCCCCTTGGCACTGACTTCATTGCCTCT

TCGTCATAA

>g14539.t1

ATGACGGCGTCGAAACCTAGCGCGGCCGACAAACTCCGGGAGCTGATCAACGGACCCGAG

GTCCTGACCATGCCGTGCTGCTACGACGGGCTTACGGCCCGCCTCGTGGAAGACGCCGGA

TTCCCGTTAACCGCGGCCGACAAACTCCGGGAGCTGATCAACGGACCCGAGGTCCTGACC

ATGCCGTGCTGCTACGACGGGCTTACGGCCCGCCTCGTGGAAGACGCCGGATTCCCGTTA

ACGTTCATGACCGGCTTCGGCGTTTCTGGGGCCCACGGCTTGCCGGACACACAGCTCCTC

TCATACGCCGAGATGCTGAGTGCCGCCACCAACATCTGCTCCACGCTCAAAGACATCCCC

TGCatcggggacggagacaccggTTACGGGAACGCTGTGAACGTCAAGCGCACGGTTAAG

GGGTACGCACAGGCTGGCATGGCTGGTATCATGATTGAGGATCAGATAGCCCCGAAGCGT

TGCGGCCACACTAAGGGGAAGAGCGtactggggagggaggaggcctACACCCGCATAAGG

GCGGCATGCGACGCCAGGGATGAAGGTGCAGATATTCTCATCGTGGCGCGCACGGACGCT

CTGGCGGGCCTAGGGCTAGAGGAGGCTCTGGAGCGGTGCCGGGAGTTCAGGAGGATCGGA

GCGGATGTTACATTTTTGGACGCCCCGCAGAGCGTGGAGGACATGGAGAGGTACTGCAAG

GAGGTGGACGGTCCCAAGCTGGCCATCATGATCGAGTTCGGAAAGACCCCCATCCTGCCT

CCGGCGGTGCTGGGCAAGATGGGCTATACCATGGTAGGCTACCCGCTGAGTCTGCTGAGC

ACGAGCATCAAGGCCATGAAGTCGGCGCTGGGGAGACTTAAGGCTGAAGAACCCCTGGAC

GACCTCTTGGAAAGTTTCGACGAGGTTAAGCGGGTGGTCGGCTTCCAGGACTACACCGAA

ACCGCCGCTcagtacgaaaaaaaagcagacgCCTAA

>g14544.t1

ATGAAGGATACGTACACCCCTTTCATCCTTTCCGCTGCGGGCAAGCTAGTGGTGCTGCTG

GGGACGGCCGCCATCCTCTCCGCTGGCATCTGGGGCGCCACTCAGGCGACACAAGGGTTT

GACGTGCTGGATCTCACCCCAGACGACCACTTCGCCCGGGACTACGCGGATGTGGCAAAG

ACGTACGAGCTGGAGGTCGACACTCAGTTCGTTCCGATGAACATCTACACCCGCGCTGTG

GACTATCCCGACATCGCCGTCCAGGCGCAGCTGCAAGAAACGGACCAGCTGATGATCGAG

GGGGAGTTTGTGGCGGGACCAGTGACGTCATGGCTGACTTCGTTTGTGGCATGGACCAGC

AACCACACGGAGTATAGCGCAAACGTTGGCACCTCCGGGGGACACGCAGTGTACGACGAC

CGAGACACGTTTTACCCGGCCCTGGCCGAGTTCACCGAGGAAGGAGACAACGTGCGTTTC

TTGTCCGATATCGTCTACGCGGACGACGGAGAGATAGAGATTAGCAGGACGGAGGTGTTT

CTGGTCGGCCTCACCGATTCCGAGAAGAACGTCGATGCGTTGATGGATGTCCGGGACGTG

GTGGGCGAATCGACGCTCGATCCCGAGCCGTTCGGATTTTCCTACCTGTTCATCTTCGTG

GAGCAGTTCGTTGTGATTTACAGAGAACTGATGATGAACTTCCTCTtggcgttggtggcg

gtggcaatCTTGAGCTTCCTGGTTCTGGGAAAGGTCGCCATCGTCATTCTCGTCTGTTTC

ACCGTGGTGATCATCGACGTGGACTTGCTCGGGTTTGTGTACCACTGGGGACTCGAGGTC

AACAGCATCACCGTCATCGAGCTGATCATGGCCGTTGGACTGGTGGTGGACTACATGGTT

CACATCGTGCACTACTTCCTACACCAGGACCCGGGCATCCTGAAGGATGCGCGTATAGCG

AACGCTCTGGGGGAGATCGGCCCCTCCGTGATGGTCGGGGCGGCGACAACATTCCTGGGC

ATTATGCCGATGGCGTTCGCCCAAAACGCCATCTTCCGAGTATTTTTCAAGATGTTTCTC

GTCATCATCACGTTTGGGTTCTGGCATGGCGTTGCCTTTATACCGGTGGCGCTTTCCCTT

TTACCCGACTCGATGCTCGCGTCTGAGCACCCCGCGAAGACTGGCGGTGTTCCTGCCAAG

GGAACCACCGGCTCAGCGGTCGTCGTGCAAGATTGA

>g14665.t1

ATGTCATCAGGGGAATGGTGGGAGGATATCTCTGTGTACGAGAGCGACTATGTCCCCGCC

ACGTTCACGCCGGGGTCTTCGGTGGAAACGAACCACGAGCTGCTGGCCGGGATGAGGGAG

AACGACTTCGACACCTCGGACTCGGCATACACCTACGGGCTGTTGACCTTGGCGATGGTA

TTCTTGGTGGCTGGGATCCTGGCGTGTATTCTTCTCCCGTGCTGCTACCTCGCCATCGGC

AAGTCCCGCCAAGAGATGCTTAGCGAGAGGTTGAACTTCGGGCGCAAGATGCACTACAAA

GGGAACTTAGTAATGTTGGTAGCTAGCGTGGCGGCCATAGCCACCGCGTGTGGCGCGATC

TGGGGACTGGAGAAGACGGACACTGGCTTGACCAACATCGAGCAGCGCACCCAAACAATC

GCCAGCGACTTGCAGAAGATTCTGAACCTTCCGAACGACACCTTGACCGTTCTAAACCAA

ATCACGGAGGACTTGGACACTCTTACCTCGGACGCCACTAGCTGCGTCGGCTCCGTCCTG

CCATCGAGCGTGGCCGATTTCGACACGGGTTTGACCGAGCTCACCGACAGCGTGCTGGAA

GAATTCGACCTGCTCAAATCGGAGAGCTTGAGCAATCTCGCGGATGACgtCCAAGGCGTG

TCGGACGCGGTCAACGATACGGAAGGGGTGCGGAAGGGCGTGGTGTGGCCGGCGGTGGCC

CTGGCGATGGCCTTCACTGCCATCTTCATCGTCATCGCGGTCGGCACGTACGGCTTCATC

GCGTGCGGGACCCACACGTGCGGGCCGGTGGGCTGCATCGTCAGCCCCTGCACGGTGTTC

CTGGCCATCATAATCTGGCTCTCAGCTGCCTCGGCGGTTACATTAAGCGCACTGTCCGGC

GACTTTTGCACCGACCCGGACACGAACGTGGAGACCCTGATCGCCAAGAGCTCGGACGAC

GAAACGCTCCGTAGCTTGCTCACCTACTACACCGGCGACTGCACCTCAGACAACTTCGCG

ATCGACGCGGTAATCGCGGCCCAAGAGGCGGCCATACCGGTCCTCCAGGAGGCGCTTCCC

CTCCTCTTCTCGGTCTCGGCCTCTTCCATTTGCAGTGGCATCGGGCCCTCCATCGACGCT

CTCAACATGTCCCTGTTCGAGTTGTGGTCACTCCTCGAAGAGGCGGGACAGCTGTCATCG

TGCGAGAACCTCAACGAACCCTACCAAGCGGCAGTGTACACGGAGCTGTGCTACGAGCTC

CCAAAGGGCCTGCTGGGCTTCTGGGTGTCCTGTGTGATTCTCACGGTGTTGCTTCTCGTT

CtgGCGGTCCAATGGAACCGGCTGCTCCGGCGGCAGCTAACAGAAGAGGAAGCCCAGCTT

GCGGAGGCAAGGGCAAGGAACAGACCTGCGTGGGTGCCCAAGATGCTCTCCAGACGGAGC

TTCAACTCCAACGCGTCCTCCAGCTCAAGACGCGGCATGACCGACACCCGTCGCGCGACG

CGCCCCGCCGCGAACCCTGTCCCTGATCgttgggggatgggggtggcggtgggtgcTCCG

GCGAGTGGGTCGCATGCTAATAATTCAGTCCAGCGGCCCGTGGAAATCGCTAACCCTATG

CGCCAGGCGCGTTGA

>g14681.t1

ATGTCTTCCGCAAGATGTGCGGCAGCGTGTATACTGGGCGGTATTGGGCTAACTTCAGCC

TTCACAGGGACCGCCTGGAACCTCGGCAGGCGggcacacgcagcagcaccgccgGCCGCC

AGCATCAGGGCGGCCTCCTCCGCCCTCATGGGGCAGAGGGCGCGCGCGGCTTCTCTCTCG

ACAGCCTCcaccgcgaggggggggggcggggcaacgTCCCCGCTCAACATGGTCGCGACG

GAGCCTGTCCTGAAGTCCGCTAGCAAGAGCGCAAGTCCGCGACTCGCTAAGGTGTTTGAC

GCCGTTGCCGAGTCGAAGCAGGCAGCCTTCATCCCGTACCTGACCGCGGGCTTCCCGAAG

AAAGCGGACACCGTTGACCTGCTGCTGGCGTTACagGAGGGCGGAGCCGACGTTATCGAG

CTCGGGGTGCCCTTCACTGACCCCCAGGCGGACGGGGCCACCATTCAGGGCACCaaccag

ATCGCGTTGAAGAACAACGTTAACATGAAGGACTGCCTGCAGTACGTGAGGGACGCGCGG

GCCAAGGGCCTGACTGCGCCGGTGATCCTGATGGGATACCTCAACCCTTTCCTGGCCTAC

GGTCTGGACAAGCTCATGAAAGAAGCCCAGGAGGCCGGCGTGACGGGTGCGCGGACCGGT

CTGCCCCCCGACCTCAAGTTCTTCGTGGAGCGCGTGCGAAAGGCGACGGACAAGCCCCTC

TCGGTGGGCTTCGGGGTGAGCTCTGTGGAGCAGGTGAAGGAGGTCGCCGGCATGGCGGAC

GGAGTCGTCGTGGGGTCGGCCTTCATGAACGCCATCAAGGAgGTGCCCGCGGATGCTCCC

GCTTCGGAGATCGCAGCGCACCTGAGCAAGTTCGCCGCAGGGCTCAAGGCTGGGGTAACC

CAGGCGGAGATGTCGTTCAAGCACACGGGCGAGGAGGGGCTGGCTTACAAGGGGAAGGAT

GTGTCGGGGGGCTACTTCGGAGACTTCGGTGGCCGTTACATCCCGGAAACCCTCGTGGAG

GCGCACAGGGAGCTCGAGGAGGCCTACGAGGCGGCAAAGGCCGACCCGGCCTTCCACGAG

GAGGTGGCCTGGTACCGCCGGGAGTACATCGGCGGCCCCACCCCCATGTACCACGCCAAG

CGCCTCACCGCAGAGATGGGCGGTGCCCagtacaccccgcgcGAGGAGCTAGCACACACG

GGTGCGCACAAGATCAACAACGCCGTGGGGCAAGCCCTCCTGGCCAAGCGCATCGGCAAG

AACCGCATCATCGCCGAAACGGGAGCAGGGCAGCACGGAGTCGCCACGGCCACCGTCTGC

GCACTCCTGGGCATGGAGTGCATCATCTACATGGGGGCCGTGGACTGCGAGAGGCAGAAG

CTTAACGTATTCCGAATGAAAATGCTCGGCGCTAAGGTGGTGCCTGTGGAATCCGGGAGC

CGCACCCTTAAGGATGCCATCAACGAGGCGATGCGGGACTGGGTGACGAACGTGGAGGAC

ACGCACTACCTCATCGGCTCCGCGATCGGCCCCCACCCTTTCCCCACGATCGTGCGAGAC

TTGCAGGCCGTCATCGGCAAGGAGGCCCGCTcgcagATCCTTGACATGGCTGGGAAGCTC

CCGGACTACGTGGTCGCCTGCGTCGGCGGGGGCTCTAACGCCATCGGCATGTTCCACCCC

TTCGTAGCGGATGTTGACAACGGGGACGTGAAGCTTGTGGGGGTCGAGGCCGGGGGCAAG

GGCATGGACGCTCTCAACTCCGCGACGCTCACCCAGGGCAGCCCGGGAGTGCTGCACGGG

ACTCGCACGTACCTCCTCCAGGACGAGGACGGTCAAATCCCGGAGACACACTCGATCTCC

GCCGGACTTGACTACCCCGGGGTGGGCCCCGAGCACTCGTGGCTGAAGGACAGCGGAAGG

GCAGATTACGCGGCGGTAACCGACGTCCAGGCGCTGGAGGCACTGCAGCTGCTGGCGCGC

ACAGAGGGTCTCATCTCTGCCTTGGAACCATCTCACGCCGTCTACCACGCCATGCAGATG

GCCAAGGGTCTGTCTCCGGACAAGATCGTTCTCGTAAACCTCTGCGGGCGAGGGGACAAG

GACATGCACACCGTCGCCAAGTACCTTGGCGAAGACGTTTGA

>g14715.t1

ATGGGGGAGGAAAAGGGAGCTCCGCCCCCAGGGTGTCGGCCGATATGGATCCAGATGAAG

AATTACGATTTGTACCAGCTACGGGAGGATGGGGCGGAGGCTGGCAGCATGACAGCTCTC

AAGCCGTTTGTCCTTTTGGATCGGGCGCGTCTGCTCGAAGAAGTCGGCAAGTTCGGCTTC

ATGTGCGATTGGAACGATTTCAAGGCCGACCTAGAGTCTTGTCCAACGGAAGAAGTGCTG

CTGATTGGAGACCCGAATAAGGTGTACGGGGAGCAGTGGTACTTCACCTACACAGCGGAA

GCCTTCGAGTCCCAGCTAGTCCTTCAGAAGGCAGGAGAGGTGGAGGAGCAGGCGCGATTA

GAAGCGGAAGCAGAGGCGGCACGGCTGGCGGAAGAGGAAGCCAACCGCAAACCCGTGTAC

GAGGATAAGCCATTCATCGCTCGAGGCTGGGAATCTGAAACAGCATCGGACACGGCTAGG

GAGGTGGAAGGTCTGACGATAAAGCCTGAGCGGCCGCTTGTGGTCATGTCCATGACGCGC

CCTAGGCGGAGCTTCGGTTGCCCGCCCAAGCTGGTGGACAGAGATGCGAGCGAGTGGCTT

ACGGACGACTACATGGCCAAGATGGGCATCCGGAAGTATCTCGCGACCGGCTTTACGGCC

TACGACCCGGGAAACCCTGATCGGAAGCTGATGGACTCGCCTTGCCAGGCCGCCGCCGAG

AGCTGCTCTGCCTCGTCTCAAACCACGTGGTTCCGCAGCGTGAATGCATCCGTGCAGTAC

TCCACCATGCAATGCGAGCCGAAGGAGGCGGCAAAAATCATGTCCAGCGAAGCCATGGTG

TCATTTCTACGCAAGATGGCACCGGGGGTGGAGCAATCTCTTCAGCAAAATGAAACGGTC

GACATATTCCAGGACGCATTCCATGGCGTCGGGGATGACGACTCTCTTTTGATCAAGGCA

AACGAGAACGAGAATGAGCTGAAGGAGCTGCGCACATTCACAGACCTTATACACTCAAAG

AACAAGGTCATGGCGGCGATTGACTGGCTCCCCAAGCGCAATGGCTGCGTGGCTGTGTCG

GCCGTGAAAAACGTCAACTTCGACGAAAGGGTCGAGCTCTCCGGTCAGGTGGACAATTCG

TACGTGCTTTTGTGGGATTTCGCGGACCTCATCCATCCAATGCTCAAGCTCGAGACCCCG

CACGAGATTTTCTGCTTCAGGTTTAACCCATCGATGCCTGGGATTGTTGTAGGTGGCGCA

ATCAACGGGCAGGTGTTGGTATGGGACATATCAAAGTCCTTGACGGCGATCGACCAGAAA

AAGCGAAAGCAGAGCGTGCGGCATATCGGGAAGCCGGGGTTAGGGGCGGGCGGCGATGAC

GACGAGCCTGTGGCGGCGCTGCCCCCCGTGCAGCCGTTAGCGGTGAGCCATATCGACATG

AGCCACCGCAGGCTCGTGGCAGATTTGGCCTGGCTGCCTACGTCGACTCAGGTTAACTCG

AAGGGTCAGCTCGTGGGGGACGAGCACATCACCGATCAGACGCACCAGTTTATCACAATC

GCCAGCGATGGTCAGTGCCTTATCTGGGACACGCGATATCAGGAGATAAGCGAAGGCCTG

CACCCGCATATCGCAAAGCCGCGACAAAACTACGACAAGAAAAGGGAGGAACACGGGCGC

CCACCACCGGTCCCGTGGACACCACTTTTTCGCCTACAACTCAACCGGTTGGAGGATGTG

GGAGAGCTAGGACTTTGTCGGATCGTGCTGGACTTGGGCGTTGCCGGCGACGGGGTGACT

TGCGACAAACCCGGCGATGGCGGCGAGGGTGTCGACATGCGCTCACAGCTTATCTGCACA

ACAGAAGAGGGGGAAATCCTACTCGCGGATTGGCGCGCGAGGGTagggaccaccaccaac

agagaGGAAGaaggggcggcgggcggggccGGAGGGAATGAAGACGGGGGAGATGTGGCG

CCGGAATTTGTAAAGTGGGTGTCGAGCGACCACACCAGGCCATGCGTAGCGCTGGACAAG

TCGCCGTTCTTCCCTGGAGTGCTACTCAGCGTGGGCGACTGGAGCTTCCAGATTTGGAAG

GTGGGACTTCGAAAACCCGTCTTCTCGTCGCCCATGGCGGCCAACTACCTCACCACAGGG

CGGTGGTCCCCCACTCGGCCGGGAATGCTCTTCCTCGGCAAGGTGGACGGTTCCATGGAT

GTTTGGGACTTCACCGACAGCAGTTACACGCCATCCGTGACGTTGATGAGCAGTCCTTCA

AGAATTACCAGCATGGAGTTTTTGAAGCCCAAGGGGGTGGGAGGAGCGGCCAAGGTGAAG

CAGCAGTTGCTCACCGTGGGAGACGCGGCTGGGAACCTCCACGTCTATGACGTGCCTCAG

AACCTGTGGCGACCGCTGACCAACGAACGGGCGCTCATGTCCAACTTCCTCGACAGGGAA

ACAAAGCGCGTGGAATATTCCACGGAACGATTCGAGTTTCGCACAGCTGAGTCCGATGCG

ATGGAGGCGGAGGAGCTCCAGAATGCCTTGAATCAGCCCAACCACCCTCCAGAGGGGGCt

gccggtggcgggctcagcgGAGGGGGGGACCCTGCGgctggagggggaggcggggcggaG

ACAGCAACCGCGACTGACGGAGCTGTTGCAGCCCCTACCAAAGGAGGAAAAAGGAGATCG

ACCGTCGATCCGGCGTTGGAACAAATGACAGAAGCAGAGAAAGCGCATCTTGCGGAAATG

GAGGAAAAGTTTATCGAGGAGTTGGGCTTGACCGACGCCGATCTCCCCGAGCGCTTCTTG

GCCTCCAAAAAGTTAGCAGAGGGAGATGGACCGGCGCTCAAGGCGTAA

>g14744.t1

ATGTCGCCTATCTATTTCCTGCACCACAAGCAGAACTACTTGAGGCAGGACGGAGGACGT

GCGCGCGGGCAGGAGCACCGCAGCCTCATTTCCAACAATATGGCGACCTTGCTCGATCTG

CACAACGACGTCAGGTGCCTACACAACGCAGACCCGCTGACTTACAGCAACACGGTGGCC

GCCTCTGCCACGTCTCACGCTCAAGTTCTGTCGAACTCCTGTGGGCAGCTTTTCCACAGC

GCGAGCGAAGACCGCAACGGGTACGGAGAGAACCTCTACTTGTGCGCCCAATCGGGCTCG

GGAAGTTGCTACACGCCCGAGGAGGCCATGGCTGCCCTATATGAAAGCGAAGTGCAGGTG

AGCAGCGTGACGAACTACGGTTTGCACGCGACTCAGATCCTGTGGAAGTCGACCACACAG

CTGGGCTGCGCGGAGGCGACTTGCACCCTGGACTCGTTCAACTACGTCTACCTCGTGTGC

CAGTACAACCCCCCCGGAAACGTCAACTTGTCCACCAGGCTTCAATCTGAAGTGGGCCTG

CCCACCGGAACCAACTGCTAA

>g14745.t1

ATGCGGAGCGCCGTGAGTGCCGTTTTCTTCCTGGCGATGTTTCCGTTCGGATCGATGGCA

GCGGGAAAAGCTCAAACATCTATCCGCCGGACGTCCAACGCGCCTGGTGCTGTTGAAGGG

AACGCCCAGCATAGCCACTTGAGGCAGGACGGGGGGCGTGCGCGCGGGCAGGAGCACCGC

AGCCTCATTTCCAGCAATATGGCAACCTTGCTCGGTTTGCACAACGAAGTCAGGTGCCTA

CACAACGCAGACCCGCTGACGTACAGCAACACGGTGGCCGCCTCTGCCACGTCTCACGCT

CAAGTTCTGTCGAACTCCTGTGGGCAGCTTTTCCACAGCGCGAGCGAAGACCGCAACGGC

TATGGAGAGAACCTGTACTTGTGCGCGCAATCGGGCTCGGGCAGTTGCTACACGCCCGAG

GATGCCATGGCTTCCCTATATGAAAGCGAAGTGCAGGTGAGCAGCGTGACGAACTATGGC

CTGCACGCGACTCAGATCCTGTGGAAGTCGACCACACAGCTGGGCTGCGCGGAGGCGACT

TGCACCCTGGATTCCTTCAACTACGTCTACCTCGTGTGCCAGTACAACCCCCCGTGA

>g15002.t1

ATGGACACGATGACGGAGCTAAAGCGCAAGCGAGAAGACGTTGACGACGGCAAACCTGAG

GACGGAGTAGCAGGCACCACCGACGTagccccttcctcctcctccccctcctctcagTCC

ACACCACCAgttgcaccagcaccagcaccactggTGTGTTCGTCAGAAGAAGAGGGCGAG

GTTAGTAGCCATGGCGATAGCCCTGCTGCCTCGACAAGTGGCACCTCGGGGGCGGCAGAC

AAAGCAGACAAGCCGGCCGCGAAGAACGCGGCGGCCGACCGCCAGACGATCCTGATATGC

GGCAAGGAAGTGGTGCTGAAGAACACCGGAGGGCGCAAGAGGCGAAAGCACCCCATGGAG

ACGCTGGTGATCTTGGCGTACAAGTCACGCCTGGCCATCTCGTGCAAGGCGAACGATATG

GTGGCCGCCCTCGAGATCCACCGCGAGATGAAGAGCAAGGGGGTCAAGCAGGATATCTCG

ACCCACCTGATGGTGATAGCGGTTTGTGGTGGCTCGCCAGGCGACAAGGACGAGCTCGAC

CCGGGCAATGGATCCACGAGCCCTTGTGCTGCATCTGCTGCCGAGAACAAGGCAGCTGTA

AGTGAGCTTGAGAAAGACGCGGATGGCTCTGCTGCCAGAGGTGCCGATCCCGGGTCTGCT

CAAGAGAACGCGTTCGCTGCTGCAGAAGCGGCGCTCCAGATATTTTCTGAAGCGTCGAAA

GACGGCACGGTCGCCCTCCCGGAGTCTGCCTACACCAGCGTGATTCGCGCCTGCTGCCTG

GACGGTCGAGCGGACAAGGCGAGAGAGCTCCTGGGCAGCCTGAAGCGATCCGGGGGGAAG

CCGCGCGTGAGGACGTACGCGCCGCTGCTGGAGGCGTACGCGGGCCTCGACGGGCGCCTG

GAGGACTGCGTGGCGGTATGGCGGGACGCGGTGTCAGGCCCCACGGGGGTGACGATGACG

GAGAGAGAGTACCTGCACCTCATCAAGGCTTGCACGAGGGCCAAGGACGAGGAACGCTTC

TTGGAGGTGATGACCGAGTACATGGACGACGTGCTGCAGCCCCGCAGCCGGCACAGCTGG

GACGTGCTCAAGGCCTGGTTTATCGCCGCCGGCGGAAGAGAAgtcacgaccacgaccacg

actgCCTCCGCGACTTCGACTGCCGCCGCGACTACGGCTGCAACTGCGACCGGGACCACa

accgcgactgctgctgtcaccACCACGgcagccgccaccaccgccgccgccgatagcACG

CCAAGAAAATGCGAGGCGGAGGGAGGCGAGGACGAAGAGATGACGCCGGTCCTCCCGCAA

GACGTCGATgatgacggtggcggtggcgatggccaCGGCGATAACGCGAAAGAAGAACGA

ACGCCACCAGAGGCGCGGCGAGAGACCGAGAgcgctcccgctgctgctgtttcggagCCT

CCGAACGCTCTTGAGAAGGACGGAGCGGTGGTCGCGGCAGCCCCTTCGCCTGTAGACGCA

GAGGTCCCCgccacaccgccgccgccgactgaCGCGGCGATTTCGAACGCCGAAGAACGA

GTGCGAGAGACcgagagcactgctgctgctgctgctgctgctgctgctgcttcggggCCT

GAAAAGGACGGAGCGGTGGGCGCAGCAGCACCGCCGGCAGACGCAGACGGCACCACCACG

CCGACGctaccgacgacgacgacggccgcGATTTCTTCGGTCTCCGTCGCAGACGGGGAC

GGGGACAGCGAGATGGCGTCCCCTACCCGACCACTGCCTGCAGCAGATAACTCGTTGGAG

GCAGCGCGTGGGGCGCCAGCgagcgctggtggtggtggtggtggtgcgtgtggtgACTGG

GTTGTGTCAGAATGCAAGGTCGCCGACGACGGGTCGTGTGGGAGTTGTGGAGAAGTGCTG

CGGTCGATCGACCTCTCGGAGGACGACGAGGAGCGGATGCTGAAGCAGATTGATGTCCTG

GTGTGCACGAACGAGCAGCGGACGAAGCAGTGGGAAGGGTTCTGCGCGTGGATCGAGCGG

CGCGGCAAGGAGCGGTACGACGTCATCATCGACGGAGCCAACGTGGGCTACTTCAAGAAG

AACGCGGCCGGTGCCGGAGAGCTGGCCGACCTGCGGCAGGTGGACTGGGCGGTCAAGAAG

TACGAGGAAGAGGGAAAGAGGCCTTTGGTGGTTCTGCACAGCCGACACCTCGTGGAGAAA

CGGCTTTCGGCAGATGCAAAGGCGATTGCCGCGCGGTGGAAGGAGGCGGGTATTCTTCAG

ACCTGTGCGCCCAAGAACAACGACGACTGGTACTGGCTCCACGCGGCCGTGTACACGGGA

GGGAAAGTGCTGGTGCTGACGAACGACGAGATGAGGGACCACCACTTCTCCATGCTGTCG

CACCGCTCGTTCCAGCGGTGGAAGGAGAGACATCAGGCTCGGTTTCACTTCGGCGTTTGG

AAGGACGACGTGAGAGAAGTCATCACGGACGAGCCTCCCCTGTATTCCAAGCGCACGCAG

AAGAGCGAGAGATCGTTTCACGTACCCCTCCAGGGCTCCGGGGACTGGCTCTGCGCCACG

CGGCAGCCGATTAAGACTGCCACAtcaagcggcagcagcgacagtagcggcagcagcagc

aaccagccagcagcagctactacgcCCGGTAGATAG

>g15178.t1

ATGTTCGCTCCGTTCCGTGTGTCAGTAACGTCATCGTCGGAGGACTCCCAGATCGCTGAG

GTCGCACATACAATTTCCAAGGCATTCCAAGACTTCAATATTTCTGTCAATGCCCCGGCT

GAGATAGTTGAGGAGGAATTCGGAAGCCCTGAGATCGTCGCTATGACCATACCGGGGATT

TGCAAGTCGTTGGTTGGGTACGCGGCCACCAACAGCGACGGCAAGATCGTCGGCGTTTCG

TTCGTTGACGTGAGCGCCAAAGGCTCAAAGGTGGCTATAGTGGGCCCCGTCGCAGCCTTG

TCTCCCGGCGCGGGGAAAACAACCTTCATGGCCGCGTGCGCGCATGCAGAGAAGCTCGGC

TTTTCGACCTTGATCCTGCTGCAAGTCGCCTCAAACTCGCGCTCGTTTGCCCTGTACGCC

AAGCTGGGCTTCGAGGCGAAACATACGTGCCAGTATATGGGCGGGTTCCTTTCGTCCTCG

GGGTGCCTGTCTCCTGCCACCGACACGGATGACCTGTCATTGACGGCTATGACCTTAGAA

GACGTGCCGGAATGCGCAGAGCTGTTTCTTAGGGCGCACGGGGCAGAAAACGGGTGGGAC

AGGCAGCAAGATATCGCTGAGATGGTGACGGCCAACCTTCCGTATGcgacGCTGGTGGCT

AGGGACGCTGACGGAAAGGTGGTCGGCTACAGCACAGGCTTCCTCCTTGTGGGTCACCAG

GTGTCGATTTCCCAGGACGTCTTCTGCGCCATGTACAAAGAATCATCGAAGCTGCACCAG

GAGCGTGGGCTCGCTTGCCCAAGGTTTCATTGCCCCGTGGAATTCCCCGGGCTGCTATCG

TGGGCACTGGCTCAAAGGCTGACCGTCTATCGAGCCGCCATCATCATGGCCAGGGGAACC

TACGAGCACGCTTGGCCCGGTTGCGGTTTGGTCGTAACACCGAGCGTGGCTGGATGCCCG

TACACCCTTCGCCTCCAAGCACCCACGACACGGCTACAGCACTCCCGTCAGGGTGATGTC

ATGTCACCGAGCCGGTTCCCTCAAATGGCATTCGTTCAAGTGACGGGAGGAATTGCGGAA

GTGATGAACGTCGACGATCTTATCAGGATTCCTAACACTCCTTGTTGGGCTTGGTGGTGG

TCGATTAAACTGGCATCTCTGGCTCGTCGCGGCTTTGAGGTGAACGCCGAGAAAGGAGTG

ATGTCCATCCCGCCGGAACCCCCGGCCCGAGTTCCTGTGGCAGTCACGCTAGCGTAA

>g15184.t1

ATGGCTGCAGCAGGCGCGAACAGAACGACAGGTCCCATCAAGCGTGGCTCGTGGTGGCCA

CGGCTCCACGCGAAGGCTAACAAAGCCGCCGACAATGTCTTCTACCGACTCGGATACTGG

GTGGCGGGGCACCCCAAGCGGACCCTGCTGATCAGCCTCGTGCTGGTCGTCCTGTGCTGT

TTCGGCTTCGTGAACTTCGAAGAGAGCGACGGCGGCGGTACGTTCGCCGTGATACTGATG

GAAAACCCGGCAGAAGGCGGTAGCGTGCTCACGAAACAGGCACTAGACGCGCTATGGGAG

TTGGACGCTAAGGTCTTGGCGGTAGAGACGGAGGGGAAGACGTACGCCGACCTGTGCACC

ACCGACACGGACGGTGTTACCTGTGCGCAGCCATCTCGCGGCGTGACTCGCTTTTGGGGC

AACAGCTTCGACACCTACGAGAGCTCGGTTTCGAGCGATGCGGACGTGCTGGCCGCCGTG

AACGTCGACCGCTACCCGGACGGCTCAGCGGTCGTCCTCAAGGCCGTTTTCGGCAACAGC

TTGACCTACGACGACTCCGGCGATGTCTCCGGCGCGACGGCCTTAACGCAGACCTACGCC

TTGCAGTCAGAGGACGATGAGGATGGCGAAGAGAACACCGAGACATACGAGGACATCTTG

GACTGGCAGCTGGAGTTCCAGGACCTTCTGGAGGAGGAGTCAGACGCCGACGACGTTTTC

AACATCCTGTATTTGACGGGCCGGTCGATCGACGATGCGTTGAACGAGAGCGTTTCGGGC

GAGATCCCGCTCTTCTTTACAACTTTCATCATCATGATCGCGTTTGTGATGATCGCTCTC

GGCCGGTGCTGCTCGGGGCCGGTCAAACGCCGGAGCTGGCTCGGTCTGGGCGGCGTGATG

GTCGTCTCCGCAGCCGGGCTGGCCGCGTACGGATTCAGCAGTGGCCTCGGCGTTCCATTC

ACGACCCTGTCGACTATCCTTCCGTTCATCCTCATCGGCATTGGCGTTGATGATATGTTC

GTTATCGTCGGTGCGTTCGACCACACCGACCCCGATCTGCCGGTGCAGGAAAGGGTCGCC

CTCGGACTGAAGCGCTGCGGTGTCTCCGTCTCCTACACGTCGATGACAAACTTCTTCGCC

TTCCTCCTGGGCAGCGCTACTTCTCTCCCGGCGGTAGAGTACTTTTGCATCTACGCCGGT

ATCGCCATCCTGTTCGACTTCTTCTTGCAGgtgacGGCATTCGTTGCCTTGCTGACGATG

GACGCCAACCGCCAGAAGGCTGGAAAAAtggactggtgctgctgcttcaagAGCAACAAG

TTCCTGGAAGAGGAGAGCGTGAGGCGTGGAGTTGTCCTGTCTTCCGAAGGGAACGGTGGG

ACCAATGCGGCGGCACAGCAGGAGACGTCTGGCCACAAACCCGAAGTGCATCAGCTGACC

GGAATCGGAAGGTTCATGAAGGAGAAGTACACGCCGTTCATCCTGTCCAAAACGGGCAAG

GCGTTGGCGCTTTTAGGAACCGCAGCCATCCTGGCTGCAGGAATATATGGCGTCACCCAG

GCGACGGAGGGATTCGACGTATTGGACCTTGCACCCGACGACCACTACGCACGTGACTAT

ACGGAACTGGCAAGGGAATATGAAGTGGAGATCAGCACGCAGTACGTACCCCTGGGCATC

TACACCCTGGACGTCGACTACCCGGACGTCGCCGTGCAGGCCCAGATGCAAGCTACGGAT

GCTTTGATGGAGGAACAGCAATTTTCGGAGGGTCCTGTTGATTCGTGGCTGACGTCCTTC

GCTACGTGGGCTGCCAATACAACCGAATACAGCGCTAACGTGGGCACCTCGGGAGGATAC

CCCGTGTACGAGGACCGAGATACGTTCTACACCGCCCTGGCGGCCTTCACAGATGACGAG

GACAACGTGCGCTTCGTGTCGAACATCGTCTACGACGACGACGGACAGATAGAGATAAGT

CGGTCGGAGCTTTTCCTTGTCGACCTGGTGGACACGACGAACAACGTGGACGCTCTACAC

GACACCCGCGACGTCGCCGACCAGTCCACGCTCGACCCCCAGCCGTTCGGGTTCTCGACG

GTCTTCTTGTTCACCGAGCAGTATCTGGTGCTCTACGACGAACTGATCATGAACTTCGTG

CTGGCTCTCGTGGCTGTGGCCGTGCTGAGTGTCTTCGTCTTGGGGAAGATCGCAATCGTT

GCCCTGATCTGTTTCACCGTGGTGATTATCGACGTGGACTTGCTCGGGTTTGTTTACCAT

TGGGGGCTGGACGTCAACAGCATCACTGTTATCGAGCTGATCATGGCTGTTGGACTGGTG

GTGGACTACATGGTGCACATCGTCCACTACTTCCTACACCAGGACTCTAACATCGCGAAG

GATCTCCGGATAGCGGACGCTCTCGGGGAGATTGGCCCTTCGGTCATGGTGGGAGCAGCG

ACGACGTTTATCGGAATCATGCCTCTAGCCTTTGCCAGCAACGCCGTCTTCCGAGTGTTC

TTCAGGATgttcctcatcatcatcagctttgGGTTCTACCACGGGGTTGTTTTCATCCCG

GTAGCGCTGTCGCTCATGCCCGACTGGCTAGTTTCAGCGAACCACCGACCAGGCCACGCG

ACAGCAGTCCATGCCAGAAGCAAGACGGACGGGTTAGCCATCCCTGTCAGCGTGGCATAA

>g15291.t1

ATGGATAACGCTGGTTTGAAGATGACGCCGGAGGCCATAGCGGCGAGCAAGGTGGGCATT

GGTTCCCTGTACGCAGCAGATGCCGCCTCTTCTCTCTGCGCGTCTTTCCTGGTGTCTCCG

TTCATCACCATCGTCGACCGCTCGATCATGCAAAACGCAAGCGGCGCGATGAAGATGGGC

GACTCTGTCAGggcggGAGCAAAGGAGCTGTTCACGAGGCCGCACGTGTTCCTTCGCCGG

CCGGAGTTTGCCATGGTCTGGGGCGTTTACGCGTGCACGTACCTCACCGCCAACGCCGTG

ATCACAACTTGCGAGCGAAAAAAGACGAACGCTGAGTGGCCCAAGTTCATCGGCACCACC

TTAGTCAACCTCACCACCTGCATTTCCAAGGATCGAGCTTTCACGCGGATGTTCGGGACG

TCGACGCCCAAGGCCGTACCCATCCCGACGTACCTCCTCTTCACGATCAGAGATgcggca

acggtggcggcaTCGTTCAACCTTCCCTCCCCGGTGTCGACGTATCTCCAGGATAAATAT

GGGATGTCCAAGAAGGTCGCCGACATCACCGCTCAGatcGGGTGCCCCGCCGGTGTCCAG

TTCCTGTCCACCCCGCTCCACCTGCTCGGCCTGGACCTGTACAACTCCCCCAAGAGCAAT

CTCGCCGCGCGCGGCAGCTTCATCAAGTCCGCCTACACACCGTCGGTGATCGCGCGGATC

TGCCGAATCGGCCCCGCATTTGGCGTCGTTTACGTCGCTCACGGTTACGCCGCCAGCAGT

TACGCCGCTATCGGTTACTACACGAACGGTAACGCCGCGAAAGTGTACACTGCTAACGGT

TACACTGCGAACGGTTACGCCGCGAACGGTTACGCCGCGAACGGTTGCACTGTTTCCATA

GCGGTTACACCGCTAATGGTCACGCCGCTAAACGGTCATGATTGCTACCGGTTGCGCATC

CAAACGGCGGAATGGATGCCCCCTTGTGGCTTGAGGGGCAACGTTTAA

>g15316.t1

atGAGTGGCGACACGCACACGGACACGAACAGCGACGTTGCCGGCGTCTCGGTGTTGCCT

GTAGGGGGGATGATGGCTGTGGAGGATGAGATGGACGAGGGCGACCCTGCAGCAGCTAGG

CAGCAGAAGCTCATCAAGGCAGGGCCGCTGGCGGTGCTTGTGGCGATCGTCATCTACGTC

GTCCTCGACTACACTATTTCCGGACTGGGCTTCGTCTCCGAAATCCTGGAGTGGGTTGAA

GACAACCCCGCACTGGGCGCCGTCGCCTTCGCCGCTGCGTATGTGTTCACTACAATTTTC

TTCATTCCGGACTTTCTGCTTACGATCGTCTCCGGGCTGGTGTTCGGCCGCGCGCTGGGC

ACGGGGTTGGGTGTGCTGGTGGGGTCCGTCGCCGTGGTGGCTGGCGCGACGACCGGTGCC

ATCTTGGCGTTCCTGTTGGGCCGATTCGTGCTACGGGAGCAGGCCCGCGGGCTGTTTAGC

ACGTTTAATATTCTGAAGGCCGTGGGTAGGGCCATTGAGACGGGGGGATGGAAGCTGGGG

CTGCTTTTGCGTTTGTCTCCGGTGGTGCCGTTCAGCGTCTTGAACTACGCCATGGGCGTC

ACCGCGGTCCAGTTCCGCGACTACGCGATTGGCTGCGTGGGAATAATCCCCGGCACCGTA

GCCTACGTGTTCATCGGCACCGCGGTGTCGAGACTCTTGAGAAACGGCTCAGAGGACGAG

tcggaggacgacgacgacagcggctCCAGCGTGCAGAACATCGTCTTCATCGTGGGGGGG

ATCGCCACGAGAATCGCAGTCGTGCTGATCAGCAGGAGGTCGAAGCGCGAGATTAACAAG

GTGCTGAAGGAGGACATGGAGGCGGAGGAGCGCATGGCTAACAACAACGGGGCGGAGATT

GAGGGCGGGACAATGGGAGccgaggaggagatggagggcaGGGAAATGGGAGCCACGGCG

GCGGTGGGCGATGTTCAGGAGCAGGGGACGCCCCGGCCATCTGCGTTGGCGTATCCCGTT

AGGTCACAAGCATag

>g15320.t1

atGAGTGGCGACACGCACACGGACACGAACAGCGACGTTGCCGGCGTCTCGGTGTTGCCT

GTAGGGGGGATGATGGCTGTGGAGGATGAGATGGACGAGGGCGACCCTGCAGCAGCTAGG

CAGCAGAAGCTCATCAAGGCAGGGCCGCTGGCGGTGCTTGTGGCGATCGTCATCTACGTC

GTCCTCGACTACACTATTTCCGGACTGGGCTTCGTCTCCGAAATCCTGGAGTGGGTTGAA

GACAACCCCGCACTGGGCGCCGTCGCCTTCGCCGCTGCGTATGTGTTCACTACAATTTTC

TTCATTCCGGACTTTCTGCTTACGATCGTCTCCGGGCTGGTGTTCGGCCGCGCGCTGGGC

ACGGGGTTGGGTGTGCTGGTGGGGTCCGTCGCCGTGGTGGCTGGCGCGACGACCGGTGCC

ATCTTGGCGTTCCTGTTGGGCCGATTCGTGCTACGGGAGCAGGCCCGCGGGCTGTTTAGC

ACGTTTAATATTCTGAAGGCCGTGGGTAGGGCCATTGAGACGGGGGGATGGAAGCTGGGG

CTGCTTTTGCGTTTGTCTCCGGTGGTGCCGTTCAGCGTCTTGAACTACGCCATGGGCGTC

ACCGCGGTCCAGTTCCGCGACTACGCGATTGGCTGCGTGGGAATAATCCCCGGCACCGTA

GCCTACGTGTTCATCGGCACCGCGGTGTCGAGACTCTTGAGAAACGGCTCAGAGGACGAG

tcggaggacgacgacgacagcggctCCAGCGTGCAGAACATCGTCTTCATCGTGGGGGGG

ATCGCCACGAGAATCGCAGTCGTGCTGATCAGCAGGAGGTCGAAGCGCGAGATTAACAAG

GTGCTGAAGGAGGACATGGAGGCGGAGGAGCGCATGGCTAACAACAACGGGGCGGAGATT

GAGGGCGGGACAATGGGAGccgaggaggagatggagggcaGGGAAATGGGAGCCACGGCG

GCGGTGGGCGATGTTCAGGAGCAGGGGACGCCCCGGCCATCTGCGTTGGCGTATCCCGTT

AGGTCACAAGCATag

>g15324.t1

ATGATGAGTGGCGACACGCACACGGACACGAACAGCGACGTTGCCGGCGTCTCGGTGTTG

CCTGTAGAGGGGATGATGGCTGTGGAGGATGAGATGGACGAGGGCGACCCTGCAGCAGCT

AGGCAGCAGAAGCTCATCAAGGCAGGGCCGCTGGCGGTGCTTGTGGCGATCGTCATCTAC

GTCGTCCTCGACTACACCATTTCCGGACTGGGCTTCGTCTCCGAAATCCTGGAGTGGGTT

GAAGACAACCCCGCACTGGGCGCCGTCGCCTTCGCCGCTGCGTATGTGTTCACTACAATT

TTCTTAATTCCGGACTTTCTGCTTACGATCGGCTCCGGCCTGGTGTTCGGCCGCGCGCTG

GGCACTGGGTTGGGTGTGCTGGTGGGGTCCGTTGCCGTGGTGGCTGGCGCGACGACCGGT

GCTATCTTGGCGTTCCTGTTGGGCCGATTCGTGCTACGGGAGCAGGCCCAGGGGCTGTTT

AGCACGTTTAATATTCTGAAGGCCGTGGGTAGGGCCATTGAGACGGGGGGATGGAAGCTG

GGGCTGCTTTTGCGTCTGTCTCCGGTGGTGCCGTTCAGCGTCTTGAACTACGCCATGGGC

GTCACCGCGGTCCAGTTCCGCGACTACGCGATTGGCTGCGTGGGAATAATCCCCGGCACC

GTAGCCTACGTGTTCATCGGCACCGCGGTGTCGAGACTCTTGAGAAACGGCTCAGAGGAC

GAGtcggaggacgacgacgacagcggctCCAGCGTGCAGAACATCTTCTTCATCGTGGGG

GGGATCGCCACGAGAATCGCAGTCGTGCTGATCAGCAGGAGGTCGAAGCGCGAGCTTAAC

AAGGTGCTGAAGGAGGACATGGAGGCGGAGGAGCGCATGGCTAACAACAACGGGGCGGAT

ATTGAGGGCGGGACAATGGGAGccgaggaggagatggagggcgGGGAAATGGGAGCCACG

GCGGCGGTGGGCGATGTTCAGGAGCAGGGGACGCCCCGGCCATCTGCGTTGGCGTATCCC

GTTAGGTCACAAGCATag

>g15406.t1

ATGGCCTTCATCAATGACCCTgcaccggcagcggcagcagcggcagcagcggcagaagcg

gcagcagcagcagttgcagcccAAGACCCTCAACAGAGAGGACCCATAGCCGGCGACGAA

AGCCCTAGTCCCGAAGTAGCTGTCGAAGCTGTCCATACCAGGCCAGCGGCATGCGTGACT

GGGGGAGGGCTGGAGCGCGATGCCGAGCACATGCGGCGAGCTTTGGTGCTGGCGGCGAAA

GGGCTGGGGCGGACCCGGCCTAACCCTGCCGTTGGTTGCGTCATCGTGGACACGGAGGGG

CAAGTGGTAGGAGAGGGCTTTCATCCGAAGGCTGGGGAGCCCCACGCCGAGGTGTGGGCG

ATCCGGCAGGCTGGAGAGCGAGCGAAAGGCGGTACTGCCTACGTGACGTTGGAGCCTTGC

AACCACTACGGCCGCACACCACCTTGCACCGCGGCGCTCCTCAACAGCGGGGTGTCTAGG

GTGGTAGCCGGCATGGTCGACCCCGACCCTCGCACGGCGGGAAGCGGCCTTCGCCGCCTC

GCAGACCAGGGACTTGATGTAACCGTGGGCGTGGAAGGCGCAGCATGCCAAGCAGTCAAC

TGTGCTTTCGTTCACCGCGTCTCGGAGAAGTCTTGCTATGGCGTACTGCACTGCTCGATG

GACGGGTCAGACTCGTTGGAGCTCGAGGATAACGCCTGCAAGACGGAGTTGACGCTTCCG

CCAGACCGCCACAGCTACAACGAATACGATGCTATCGTGGTGGAGGGTAGGTCTGGAGTT

GCCAAGCTAACGGCGTGCTTGCAGGCGCTACCCCCGACGGCGTTACGAGTGGTGCTTGTT

TCCTCGGGCGAAGGGATAGAGGAGGAGTTGGGGCCGGTAAAGGAGAGCGCACTCTGGACA

GATTCGGCCGGGCATACCATCATCGTCGCCGCCAAATGCTCTAGTGGAAAGGGCGACGGC

GATGCTCCAGGCCCCGTGAGAGCTATGCTGCGTGCAGCAGGCGTTACCATTGTAGACTTC

TCCGTCCATGAACAGCCTGCGTCGCGACCAGAGCCGCATTGTACCTCGGCAGAGGCAGAG

ACAGAGGCAGAGGCAGGCGCGGCAGAGGCAAGCGCAGCAGAGGCAAGCGCGGCGGCAGCA

GGGAaaggcgcggcggcggcagcgacaaaGGCAGAGGCAGGCAGGGCAGCGACAAAGGCA

GACGTGGCAGCGACAGCGGAAGTCGCGGCACAGGCAGGCACGGCAGCGACCGTGGCAGAG

GCAGACGCGGCAGCGGGAGACGCGGTAGCGGTAGACGCGGCAGCGGTagacgcggcagca

gcagacgcggcagcagcagacgcggcagcagcagacgcgGCAGTGGTAGAGGCAGGCGTg

gcagcgacagcagccgtGGCGAAGGAGCTCTACCGACGAGGCTTGCTGTCGGCTGCATGG

GAGGTCGGCCCGGCAACCGCAGCGCGAGCTCTCCAAGGCGGGCACATTCAGCGGGCCCTG

GTACGCAGACGGCGACCCGAAGGTGAAGAGAGTGGTGGCGAGCGTTTCGCTCAACCCACG

CCCCCGCAAGGAAgcagcggggggaaggggggggaggaggaggaggcggcgctAAATGCG

ATGGCGCGGCTGTGCGGGAAGGAGGGGGTTTGTGTgaggcgggacggggcggtgcCCAGG

GAGgcaggcgaaaaaaacttcACCGAGGTGTTGTTGCCCGTGGGCCCTTTTCGTCAGTCG

GCTTAG

>g15467.t1

ATGGTTGGAGTCGCTCTTTCTTTCCTTGATGGGCGCTCCCTGACCAGCTTCGAAGTGGCC

TCTAGAGGTACCAGACTCGCGATGCAAGGCCACCCGGACTTGTTCAAAGCACTACTGGAA

GGGAGACGTGCTCTCATAGCTTGGGATGAAGGAGACTTCCAGCCAGTTCAGGCGCGTCCG

GCCCGAGTGGCCCCGGCCCCGCGTTACCTCCACCGAGTGGCCCTCGCGGACGATGGCTGG

TACTACCTCTTCGGCGGCCACCGTCAGCTGGACGTGGTAGGGGACGTGTGGCGGTTTCGG

GTGGAGAAGTCCCGAGGTCGACGTACAAAAGGCGCTTTTCCCCCTGTCAGTGTACGGTGG

GAGCATGTGGACGGTGTCACCTCGACGGGCGACGGGGCGCCGGGCAGCgaaggcgatggc

ggcggcgatggcgggagCGCAACCGAGAGCGACAGCGACGGTAGCGACGAAGAGATGGAA

GAGGAAAGCTCGGACGCAGATGAAGCAGCCCTTCCGCCCCCAGTGGTGGCCACACCCATA

CCTCTGCCAGACGCCAACGCTTTCCCTTTCCTGGTGCCCATGATCGCCGAGGCTCAAGCC

GCAGGCCTtccgggggtggggtgggccgAGATTCAGGAACTGTTTTTCGAACAATCCCCG

CCCCCTGCCGACTTCGACGCCACCGGGAAGCCTACACGCCCTCGACCCAGGTGCGCCGCA

AGCTGGACGTCCGTTCCAGGCAGCAACAAGATTTACCTGTACGGGGGGCATGGCTCCGGA

AACGACTTTTTGGATGATCTGTGGTGCTTCCACGCGGGTGGCCGGGGGGAATGCCGCTGG

GAAAAATTAGAGGCCGAGGGGGGCCTAAGGCAGCGACACTCGAAGcaccaggaggaggag

aaagaggtgCATGCTCTTGACAGGTATCCTGTTCCTGAAGGCCGGTGGGGGCATACTATG

GTGGAGCACCGAGGCGCGCTGTACATGTTCGGAGGGAGCTCCCCCGGGCATGCGTACGCC

GGGTTGTGGCGCTTGGACACCTCCGTGTCGCCCTGCGTGTGGTCTCCTTTGAAGCCGGAA

GGAGAGAAGCCGCCGCCTCGCGGGGGGCACTCGGCGACCGTGGTGGGCGACACCCTTTAC

ATTTTCGGCGGAAACATTACAACGAGCGTGTTCAACGACCTTTGGGCTGTGGACCTACCT

TGCGGAAGATTTTGGCGTCAGATCCCCAACGCCCCCAACTTCCCGTTGCCTCGCATTGGC

CACAGCGCGGTCGCGATGGGCAACCGGATCCTTATCTACGGCGGACGGAACTTCAAAACA

GATAAGTACATATCGGGACTATCGTGCTTCGACACGGATCTCCAGACCTTCGCGCGCTTC

CCCCACGCCGAAGCGTTCTCCAAACGCGCTACTCGGCGGGGGGGTATGTGGCCCCACCTT

GCGATGACCGGGCATGCCGCCGTGCCTTTTGGGAGAGGCCTTTTGGTAGTGGGGGGTATG

GTCCCTCGTGACCACTGTACCATGACCCCctgggtgctggatgtcgtcagcgGGCgtcga

cgggggggcgggggatgtcCACGCAAGTCCCGTGTGACGGCCGCGACGGCTGGTTGCGGC

ACGAGTTGA

>g15468.t1

ATGCCCTCTCCCCCCAACCGGCTTGGCGGTTCCGACACTCCGGGGAGCTTTCCCCTCCTT

GGTAAAGTTCGCCGTGGGGAGTTAGGGCCGAAACATGGTGCGGACACAGACGATGGTTCT

AACGATTATCAGCAGGGTGTCCTCGGGCAGACCTCGTGCTGGCATGATGGTCCTCGCCGC

TTGAGGCTGGGCCTCAAGTTCCCGGTAGCTGTTGTGTCTCTGGCTGTCGCGCTGATCGCT

GCCGGCCCGATGAGCGTCCTGGCCCAGGAAATGGATGCTGAAGCTGCGGATGAGGACTCA

ggcggcggaagcagcagcgtgGGAGAAAGCATTCTGAGCTACCTTATCATCGTGGTGCTG

GTAGCAGCGTCGGGGCTGTTCTCTGGGCTAACGTTAGGGCTGCTGGGGCTAGACAAAATT

GGCTTGGAGATCATCAGCAATGGGGACGAGCCCAACATGGCTGCCTTCGCCAAGaaaATT

CAACCGGTGCGGGCCGATGGCAACCTGCTCCTCTGCACGCTGCTATTGGGCAACGTTGCT

GTGAACGCCCTGCTCTCGATCGTCATGGCACAGCTTACCTCGGGGCTGGTGGGGTTCGCT

CTCGCGACGACCGTCATCACCATCTTCGGCGAGATCATTCCACAGGCGGTGTGCTCCCGC

CATGCTTTGAGCATCGGCTCGAGGGTTGTGCCTTTGGTGAAATTGATCATCCTCATCCTC

TTCCCCATCACCAAGCCTCTTAGCCTTCTTCTCGACAGAATGCTGGGAGACGAAATCGGC

ACTATCCACTCGCGCAAGGAGCTGAACGAGCTGCTCAAGATTCACGTGAAGCACGGCGCG

ATCGACGTGGAGACGGGGAGGGAGGTGGCGGGCGCGATGAACTATAAGAACCATGTCGTG

CGAGACGCGATGACGCCGGTGAAGGATTGCTTCATGCTCAGCGTGTCTGAGAAGCTCAAC

TTTAAGACGCTGTCGGTGATTTTCAAGAGCGGCTTCAGCCGCATCCCGGTGTTCGCTAAG

GACCACAACGACGTGATTGGGCTTCTGTTCACGAAAGACCTCATCTTCATCGACCCCGAT

GACGAAACGCCTCTTAGGAATTTCGTGCAGATTTTCGGCAGGGCCGTtacgGTGGTCTGG

CCGGACTACACCCTGGGAGACGTTCTGAACGTATTCAAGAAGGGCAAAAGCCACTTGGGT

CTTGTTAGGGACGTCAACAACTCCGGGGAGGGAGATCCGTTCTACGAAGTGATTGGCATC

ATCACGCTCGAGGACATCATCGAAGAGATATTGGGGGACGAGATTGTCGACGAGACGGAC

GCCTTCGTGGACGTACGAAATCAGCTTCCGGTGGAAAGGGGCGAGTTCGACATGTCCCGC

CTGCAGCTGCTGAATGCGAATTCGAAGAAGTCTCTCCTCTCTGGGGACGAGGCCAAGGCG

GTGACGTCACATCTAATGGCCAACGTACCTCAGGCAAGCCCTCTCATAGATTTCGAGAGC

TTCAGTGCGGAGCACGTGGAGTCTATCATCGCTGCGTCGCCGGTCGTAGACATCGTCAGA

GAGCAGACGCTCGTAGACAAGGCGGAGCCTGCACCAGGGGAGGTGTTGTATCGCCGCGGA

CGCACAAGCACGATGTGCACCATCATACTCACGGGGAAGGCGACCGTTGTGGCCGGAAAA

GACGGTTTTCAATCGGATGCGGGTCCGTGGACGGTTCTCGCGGCGGACGCGCTGGTTGAG

GACGAGGGGACGTACAAGCCAGACTTCTCGGCGCACGCCGGTAGCGAGCACATGCGCTGT

GTACGCATCAGCAAAGCTTCTGTCGACACCATCACATCCTTGTCCCGCCAAGAGCAAGAA

GCGCACGAGATATCTTCGGAGGTTAAAGTAGATATTGTGGATGCCGCGGAAGCcaacggc

ggaggcggtggcagcagtaggagtgttgGCAACAGCAGCGCCTTGAGCGGAAGCgatcgc

aacagcagcagcagtcccggTCCGCCGGGCGTGGGTCCTTTAGGATGA

>g15482.t1

ATGACGTTAGTTAGCACTGCAGTCGTCACGGCCTCGGAAACGGCGGTGGGGTCTTCGGAC

GGGATTTTTTTCACCAGCGTGGGCCAGATCTCCTCGCTGCCTGCTGTGAGCGTGGCTCTG

CCAAAGAGTGGGGAAGCGGAGCTTATCTCGCACGATCCGTCTGCCCGTGACGACTTTAGT

GCATACCTCGAGGCGTACTGGATCTACTGGGGCGCATGCTTTGCGACCGTGATCACGTGG

GTGTTTGCTGTGTCTGCAGCGGCAGATGATGCGTGGGTTAGCTTCGACGGCAATAACGGC

TTTGAACTGGGGAATGATTGCGGCTTCTCTGATAGCATATGCGAACGCTATGATGCCGCG

ACAGCCTTCCAGTTCATGGCCACCATCATAGTCAGCGTCGCgcttgtggtggtggtcatc

GCCGCGTTTGCGCCTCCGCCCACGACAGGCACGCTCGGAGTGGCTTTGGGGATTCTGCTA

GCAGTGTACTCCTTCTTCCAGATGATCGCCTTCTCCCTCATGGCCGGTTTGGTCGACGAA

TTCGACGACTCCGACATCAAAACTGGCCCTACCCTCGGCGTGGCAGTGGTAGCTTGGCTC

TTCGGTCTAACCGGCACTATCATCATCCTGGTACTGCGCTCAAGCGTAACAAGTGACCAA

CCGGGCCCCTTCCGGAAGGGCAGAGGCGGCGCTGCCGGAGGAGATTCGACAGGACACAAC

ATCGGGACGGTGTAA

>g15655.t1

ATGAAGATCCTGGTGCCGCTCTACATCTATCCGATTCGAGATGGACAACTTGCGCCGGAG

TGGCAGATGATGATTGACGCGCAGAAAGCGGGGGTGCCGGTGGTAGCTGTGGCGAACCCG

GGGAGCGGGCCGGGCACGGAGGGGGACCGGCCGTCGTACGAGAGGGGGATGAAAGCTCTC

CGAGATTGCGgggtagagGTGATCGGTTACGTAGCCAGCGGGTACGGGAAGACGGACGAG

AAGGAGGTGAAAGGGCATATCGACCGATACAAGGAGTGGTACGGGGAATGGGTGACCGGT

ATCTTCCTGGACGAAACGGCACACGTAGCAGATGACCTGAACGACGGGGACAAGGCGATG

TGCGCGAGGTACCGGGGCTACCGCTTCCACATGAACGTTTGCTTTGGAGATTCGGCCTTG

gtGGTGATGAACACCGGCACGATAGTCACGGAGGCGACTCTCACCAACGCGGTGGGCAGC

GACGTCGTCTGGAACGTTCTGGAGAACAATCGCGAGTACATGGAGAAGAAGTTCGGCAAG

GTGACGTGGCTGTCTcccgaggagaagaagaagaagcggggtttccttggtttcggCGGC

GTTGGTATCGGAGGGAAGAACGGGGCGGGGACCGTGGTCGGGACGCCCCCTGCTGCTCCC

GACCACGTTAAGGAGAGAGCGGCGTTCATGGTCCACGGCGCGGCACGATTGACGGATGAT

gAGATTTGCCATTGGGTGAACCAGCTGCAAGACGGAGGCTGGACCCACGTGTACATGACG

GACAAGTGCTTTGATCCCACCTCGACAAACACGGCCCTACACAATCCGTGGGACGCCCTA

CCCACGTACTGGGGGGACCTCGTGAAGGCGGTGGGGTTGGCAAACGAAAAAGCAGAGGAG

GGGGAGAAACCGAACGGAGCGTTTTGTTGCTatgcctga

>g15804.t1

ATGATGATGAGGGCTTTCTACCTGAGCGCGGTTGCTCTCCTGGCTTCTAATGCGAGTTTT

GCACGGGCGGGATGTGACGACCTTCTGAacctctcccttcctctctcgGGAGACACGACC

CTGGAGCTATCGAGCGCCGCCATCGATTGCGCAATCCTCGCGGACAACGAGCTGGAGGAG

TTCATCGTCGAGGGTGGCACGCTCACGCTCACTGGCCCCAGCACTACCAAGTTCACGAAC

TTGAAGTTCACCGTGATGGACGGCGCAGGCCTGATCTTCGACTTCGACACCACGGAGTTC

GGCCCCAACCTCGGCTATGAGGTAGAAGACGACTACGGCGGTTTCATGGTGCAGGTCATG

TCGGGAGGCTCCGTCGAGTTCACCGGCGAGATGGCTGCGACGGGGCTGGAGAATATGCGA

AGCGTCTTCTGGAACGACGTTGGCGGGCGCATCGAGTTCGCGTCAGACGTGGTATTCAAC

GACTGCGACTCTAACGTCTTCAGGGACAACTTTGGCACGCTCAGGTTCCATGGCGATGTC

ACCTTCACTGACAATCGATTTCTCGCCATCAGCAACGACGGCGGCAGCGTCAGGATCGAT

GGCGATGCGTTGTTTGTGACCAACGGAAGATCGTTCGAAGGgcacagcggcggggcgttc

TCCAACTATAACGGGGGCACTGCCATTTTTCGCGGTTTTGCGACGTTCAGCGACAACAGA

TGCGACGAGTCTGGTGGCGGAGTTTACAACGGAGTCGGCTCCAAAATGACGTTCTACAAG

AAGGCTGGCTTCTACGATAACCGATGCTTGAACGGAGACGGGGGTGGCATCGACAACTAC

GGCGGCGACCTGACGTTCCGTGGAGCGGTGTTCGCCACCGGCAACCAAGCTGCGGAAACT

GGCGGCGGGGTCGCAACAGGCAACGGCGGCACCACGACGTTCTACGGCTGGACGACTATC

CAGTCCAACACGGCCGCTACCGAGGGCGGCGGAATGAGCCTGTACAAGCTCGGCCAGGGC

GACGGCGGCAACGTTGTGACGTTCAGGAAGTCAAACACGATACGCATCAGCGACAACAAC

ACCAACGCCGGGTGCAACAACATTTACGTGGAGGAGACCTCGGAGGTAGTCGGTTATGAC

ACCACCTCCGACACATGCGACGAGGCCCAGTGA

>g15806.t1

ATGAGGGCCTTCTACTTCTTCGGCGCGTTTGGTCTGTTAACGGCTGCATCCGTTGCACGG

GCCGAATGTGACGACCTGCTGAacctctcccttcctctctcgGGAGACACTACCCTCAAG

CTGTCAAGCGCCGCCGTCGACTGCACGATTCTCGCGGACAACGAGCTGGAGGAGTTTGTC

ATCGAGGGCGGGACCCTTACTCTCACTggcgccagcaccaccaaGTTCACGAACTTGAGG

TTCACCGTGATGGACGGCGCAGGCCTGATCTTCGACTTCGACACCACCGAGTTCGGCCCC

AACCTCGGTTTCGAACGGCAAGACAACTTCAACGGCTTCATGGTGCAGGTCATGTCGGGA

GGTTCCGTTGAGTTCACCGGCGAATTGGCTGCGACGGGGCTGGAGAACATGCGGAGTGTC

TTCTGGAACGACGTTGGCGGCAGCATCGAGTTCGCCTCAAACGTGGTTTTCAACGATTGC

GACTCGAACGTATTCAGGGACAACTTTGGCAGGCTCAGGTTCCGCAGCGATGTGACTTTC

ACCGAGAACCGTTTCCTTTCCATCAGCAACAGGGGCGGCACGGTTAGGATCGACGGCGAT

GCTTCGTTCGTGACGAACGGGAGATCGTTCGACGGgcacagcggcggggcgttcTCCAAC

ACGAACGGGGGTACTGCCATCTTCCGGGGTACCGCGGTGTTCAGCGACAACAGCTGCGAC

GAGTCGGGCGGTGGAGTGTTGAACGGAGCGGACTCGAAAATGACGTTCTACAAGAACGCT

GGTTTCTTCGACAACCGGTGCTGGAACGGGGACGGCGGTGGCATCGGCAACGCGGGCGGA

GACCTCAAGTTCCGCGGGACCGTGAGCATGAACGGAAACGAAGCGGCAGAGACTGGCGGT

GGAATCTCGACCGGCAACGGGGGTACAACAACCTTCTACCGCAAGACGACGATCACCTCC

AACCAAGCTGGTACCAATGGCGGGGGCATGAGCCTTTACGGGAGCGACGAGAGCAGCCTT

GTGACTTTCAGGAAGTCGGACAAGATCGAAATCGGCAGCAACCAGAGCAACACCGGGTGC

AACAGCATCTATGTGGCGGAGAACTCGGAGCTGGTCGGCTACGACACCACCTCTGACATA

TGCGACGGGGCCTGA

>g15807.t1

ATGATGAAGGCTTTCTACTTTGGCGCAGCTGGTTTGTTGGTTGCTGCGTCGGTTGCACGG

GCGGAATGCGAGGACTTGCAGAACCTCTCCCTTCCCCTGTCTAGAGACACGACCCTGGAG

CTATCAAGCGCCGCCATCGACTGCGCTATTGTCGACGTGGACCAGCTGGAGGAGTTTATC

ATCGAGGGTGGCACCTTAACGCTGGCCGGTCCCAGCACGACCACGTTCTCGAACATGAGG

TTCACCGTGATGGACGGTGCAAGCTTGATCTTCGACTTCCCCATGACGGAGTTCGGGCCC

AGCGTATCTGGGCGACAAGATGACTACGACAGCATCATGCTCAACGTCATGTCGGGCGGT

TCCGCCAAGTTCACCGGCGAGATGACCGCAGATCGAGTGGGGAACATGGAGAGCGTCTTC

GGGAATGCGGTTGACGGCAGCATAGAGTTCGCCTCTCGCGCGGTGTTCGACAACTGCAAC

GCTAACGTCTTCCTGCACAACTACGGCAGCCTCAGATTCCGCGGCGACGCAACCTTCATC

AGCAACGAATACATCGTCATCAGCAACGAAGGCGGAACTCTCAGGGTCGACGGTGATACT

GTTTTCGAGGGCAACGGGAGGTCGTTCGATGGACACAACGGCAGAGCGATCTCCAACACG

GACGGGGGCAAGGCAATTTTCCACGGTCACGCGACGTTCACCAACAACTGGTGCGACATT

GATGGCGGTGCACTGTTCAACGGGCCGGACTCGACAGTAACGTTCTACAAGAAGGCTGGC

TTCTACGATAACACTTGCCGGGGCGGTGACGGGGGTGCCGTCGACAACCACGGGGGCAAC

ATCAAGTTTCGTGGATCGGTGGTTGCCACCGGCAACCAAGCGAACAGCGATGGCGGTGGA

ATCTCGACCGGCGACGGTGGCACGACGACGTTCTACGGCTGGACGACGATCCAATCGAAC

AAGTCTTTCTTGGGTGCCGGCGGATTGAGCGTATACAAGTCAACAAGCCAGAGCGAGGGC

GGTAGCGTTGTGACGTTCAGGAGGTCGAACAAGATTCGCATCAGCGACAACGAGAGTGAA

CGAGGGTGCAACAACGTCTTCGTGGAGGAGACCTCGGAGCTGATCGGCTATGACGCTGAC

ACCACCTCCAACATATGCGACTAG

>g15881.t1

aTGGCGGAGGATGCCGGACTCGGTGGAGTTGATGAAATCGAGGAGTTGCGAGAGCAGGAC

CGGTTCTTGCCGATCGCCAACATCTCTCGCATCATGAAGAAGTCGCTTCCAGACAACGCC

AAGATTGCCAAAGACGCAAAGGAAACCGTGCAGGAGTGCGTGTCAGAATTCATCTGCTTC

ATCACCTCCGAGGCCAGCGACAAGTGCCAGCAGGAGAAACGCAAGACCATCAACGGCGAG

GACATGCTCTGGGCCATGAGCACGCTGGGCTTCGAGAAGTACGTCGACCCGCTCAAGATC

TACCTGACCAAGTACCGCGAGTCTGTGAAGGCGGAGAAGCCCGACAAGAAGGCTAAGCGG

GACGACGCGACGGGCGAAGCCATGGTGACCTCGTACTACGGGCAGCAGCCTCcgtacggg

ggcgggggggcgggagcaggtggaggcggcggcgggggcggcgggggtggagcGCATGAT

ACAGTATGGTAG

>g15999.t1

aTGGTTCGCATCGTCTCCGCTTCGACCGCGCTGCTACTGGCGGCTTCTTGTGCCCTCACC

CTCTCCGACGCAGCTTTGGAGTACAAGATCCGCGTGAGGAACTTGACCTACCTCCAGCCC

TTCTCTCCCATGATTATGTTCGCGCACACCGAGGAAATACAAGCGTTCCAAGTGGGTCAA

GAGGCATTTCCGGAGTTCAGGACACTCGCTGAAGACGGCGATCTGTCTGAGCTCCTGGCA

CTGCAAGACGACGAAGATATATTTCAGTTCGTATGCGGGTTCTCGGTCAACGAAGACGGT

TTGGCTCCCGCGGGTGAGGCGTGGTTCACCTTGTTCGTCGATGAGAAAATCTGCTCCGAC

GGAATATACTTCTCGATGGTCAGCATGCTTGTGAACACAAACGACGCATTTGTGGGAATA

GACACCATGCCTTTGGACAAGAAATATTCGACGACGGTCTACCCCCCTGCTTACGACGCC

GGAACCGAGGAGAACAACGAGCTGTGCTCTCACATTCCGGGCCCAGCGTGCGACGCCACA

TCCGGCAACCTGCCGACTGAATCGGGAGAAGGGTTCGTGCAGGTGCACCGTGGCTTCCAC

GGTGTTGGCGGAGAGCTGTCGGTTGATGACTACGACTGGAGGAACCCTGTCGCCGAGGTG

TTCATCTCGAAGCCTGCGCGGGTTCACTCGTAG

>g16002.t1

ATGGCACCTACTCGCATCGCCTCCGCTTCTACCGCGCTGCTACTGACAGCTTTGTCTGCT

CTCACCCCCTGCGAGGCCGCattgcagtaccagATCCGGGTGAGGAACTTGACCTACCTC

CAGCCCTTCTCTCCTGTCGCCCTCTTCGCGCACACCGAGGCACTTCAAGCCTTCCAAGTG

GGCCAGGAGTCCTCCCCTCAGTTCAGGGCATTAGCCGAAAACGGCGACCTCTCTGTTTTC

ATGGCGCTCACAGCCGATGAGGGCGTTGCACCTTTCTTGTGCGGCTTCACGTTTGGCGAG

CATGAATTGCCGCCCGGCGGAGAGCGGTCGTTCACCTTGCAGGTCGACGAGAAAACTTGC

TCGGACGGAGTTTTCTTCACAATGGCCAGCATGTTAGTGAACACAAACGATGCATTCGTG

GGCATAGACTCTTTGGCCTTGCAGAAGAAGCGATCGACAACCGTCTACCCCCCTGCCTAC

GACGCCGGAACCGAGGAGAACAACGAGCTCTGCTCTCACATCCCGGGCCCAGCGTGCGAC

GCCACACCCGGCAACCTGCCGACCGAATCAGGAGAGGGCTTCGTCCAGGTGCACCGTGGC

TTCCACGGGATCGGCGGAGAGCTGTCGGCTGATGAGTACGACTGGAGGAACCCTGTGGCC

GAAGTGTTCATCTCAAAGCCCACGAGGATTTAG

>g16004.t1

atggttcgcatcgcctccgcttcgaccgcgctgctgctggcggctTCTGCACTCACCGTC

TCCGATGCCACGTTGCAGTACAAGATCCGCGTGAGGAACTTGACCTACCTCCAGCCCTTC

TCTCCCGTCCTCCTGTTTGCGCACACCGAGGAAATCCAAGCGTACCAAGTGGGCCTCGAG

GCTCCCCCGGAGTTCAGGACACTAGCGGAGGACGGCGACGCCTCTGAGCTCCTGGCACTA

GCAGACGACGCAGACGTGGCGCCCTTTCTGTGCGGCGTCACGCTGGGCGAAGGCGGCTTG

GCCCCCGCGGGAGAGGAGTGGTTCACCTTGCTCGTCGACGAGAAACACTGCAAAGGCGAC

ATTTTTTTCTCCATGGCCAGCATGCTCGTCAACACAAACGATGCTTTCGTGGGCATAGAC

ACTATGCCTTTGGACAAGAAGCGATCTACAACGGTCTACCCCCCTGCCTACGACGCCGGT

ACCGAGGAGAACAACGAGCTCTGCTCGCACATCCCGGGCCCAGCGTGCGACGCCACATCT

GGCAATCAGCCGACGGAGTCGGGAGAGGGCTTCGTCCAGGTGCACCGTGGCTTCCACGGA

ATTGGCGGAGAACTTGCGGCTGATGCCTACGACTGGAGGAACCCCGTCGCCGAGGTGTTC

ATCTCGAAGCCTACGCGGGTCCACTCGTAG

>g16041.t1

ATGTGCACCATCTGCGTTTCAGCGCATCGATCGTCCTGTTTCCGGCAACCAGCCTCTTCT

CCGGCCCAGCGCCGGAAGAAGACGGGACGTGACGGCCCGCTCCAGCCGATGGCATCCAGG

CGAGGCAGACGAGTGACCTTCTCAAAGAGAGAGTATGTGCTCGGGACTGCCCAGGACTAC

GACAGGACAAGCTTCGAGGCAGAGGTGGAGGCTCCTCCACCaagcacggctgctgctgct

gcggcggctgctgcggcggctgccgctgctgctgagatCGCGATGgtcgcagcagcaacg

gcgcccactgctactactcctgcaGCAGGAACGCGGGATAGCAGAGCGAATTTCTGTGGC

ATCTGGAGACGAAGTCACGGGTTCAACTGGGCTGCACTGCTGGAGTTCTCTGGGGTAGAT

AAGGCCGCCATACCTGAACAGGCAGCAAAGATGCAGTCTTCTTCCGTGGTGCACCTGATA

GACCACGACGACGTATCCTTCCGTCTCGTCGTCCACAGCGATGACGCTGTCGGCGTCACC

ACCAGCCAAGACCAGCAATTCTTCATAGGGGGACAACCAAGACCTAACCCCAACTCCAGC

AACGCCACCTCGACCCTCTCCAACATGCGCTGGGCTAACAACAACCAGGCGCTCGTTCTG

ACGAGCGTTGACGCGGCGAGCGGAGACGAGATGACTGTTTCCCGCCATCTGGCGTCCCAA

GGGACCGTTATGGTGCAGCACTATCGCGCGCGTTGCGCTAAGTCGGGAGATACCGCGGAG

GCCGTAACGATTTTCCGCAGAATGGCGCAAGGATCTTAA

>g16517.t1

ATGTCCGACCCGCGGCCATCTGGCTCGACCGTGGCTGGGAGCGAACTGCCCTTGTCGCCA

CGGTCATCCCCTGCCCCGCACAATAAACGCCTCCGAGAAGAGGTAGACGTCTGCGGAGGA

AAGCGTGGGCGTGCGGCAGATAGTGCGGTGCCCTCTGCAGTGCCTTGGAATTCGCCGAGA

AAGTCCACACTCGGGAGCTCGGGTCGAAGCCACAGCCTAAACCGGgacaaccacccccac

ctgctCGTCGACGTCAAGACGTACAAGGACACGCTGTACGGTAGCCTTGCTCTTGAGCCC

GTGATCCTGCGGTTGATGGATACGCCGCAATTCCAGCGCCTGCATGGGCTGAAGCAGCTG

GGCACGAGCGACTATGTGTACCGTAGCTGCACGCACACCCGCTTCGAGCATTCCGTGGGA

GTGGCGCACCTCGCCCAGCGGTTCGCCGAGGGTCTCCGGAGACGACAGCCCGAGCTTGGG

ATTTCGGTGGTGGACGTGATGTGCGTTAAGATAGCCGGGTTGCTGCACGACCTCGGACAC

GGGCCGTTCAGCCACATGTGGGATGATGAGTTCGTCCGCCGCGCAGGGGTCGTGTGGAAG

CACGAGACGGGAAGCGTGAACATGCTGCGCTTCATGCTGAAGGACAACAACATCGACCTG

TCGGCCTACCAGCCGCCCCTGTTCGCGAAAGACTTGACATTTATCGAGGAAATGATTCTG

GGAACTCGCCAGGAGGCAAGAAGAGGACGAGGGGCTGATAAGGAGTTCTTGTACGACATT

GTCAACAACACAAGGTCGGGTTTGGACGTCGACAAGCTGGACTATTTCCAGAGGGACGCG

CGCAACTCCGTGGGCGTACGATCGGTCGACCTGGACCGGTTCATCGAGCTCGCCAGGGTC

CTGCCGGCAACTGACGAGAATGGCGAAGTCCATCGGATGGTTTGCTACCCCGACAAGCTC

TGGACAGAGGCGCTTCGTCTGTTCCAGACCCGAAATGAACTGCACCAGTCCGTCTACCAA

CACAAGGGCGGCAAGTCCGTCGAGCTCATGCTTGTAGACGCTTTTTTGCTAGCGGACCCG

CACTTCTCCGTGGCGGGCACCGTCGACGACGAACACCCCGACGGGCGTTGGAGGATGAGC

GAGGCGATGTTCGACTGCAAGGCCTATACGTGCCTCAAGGACAGCACCGTGGACTTGGTG

ATGAACGACCCCAATCCCAAGCTCGCACCGGCGAAGGCCATCATCGATCGCTTGCAGCGG

AGACAGCTCTACCCCTGCGTCGGGACGACGGAGTTCGACTTCACGGAGGCGGGAATCACG

GAAGAAGAGGCCAAACGCACCATGATTGACATGCTCATGCGCCCCTCCACGCCGAAGGCA

GCACCGCTCGGGAAGGTGCCCCGCACGCCGCCACAATGCGCCGAAAACGGCGCAGGGGAA

AGCGACACAGACATCGAGGACGACGAGGACAGCGGCTGCGGAGCGGAATCCGCGATTGCG

AACGGGGGCGGAAGTGGACGGGGGGCGTCGACGCCGCCAGCGGCGGGCGAGCAGGTGCAG

CTGGAGGAAAGTGATATCATCGTCGACTTCATGCACATTCATTGGGGGCTTAAGGGGGCG

AATCCTGTCAACCGGCTGAGATTTTTCTCGAAGTACGCCCCGACCGCGCCAGACACCCGA

GGAGTGCGCCTTGACGAGCAAAAGTTCTCTAGCTTCCTGCCGAAGACGTTCGAGACGAAA

ATGCTGAGGGTGTTCGTGCGGGACCCTGCCAAGGTGGACGCAGCAAGGAGAGCCCTGGAT

ATGTTCTGCTCCGAGTCTCGCTGTGCGAGCCCTTGTCCCCTGCTCTCGCAAGAGGACTAG

>g16679.t1

ATGGCGGAGACTGTCGGATTCATCGGGCTGGGTATCATGGGCCAGGGGATGGCATCCAAC

CTGGCCAAGGCCGGCGCCAAGGTGGTCGTGTGGAACCGCAGCGAGGGAAAGCGGTCGGCG

TTCGTCGCGGGGCACGAGGGGTGCTCCGCGATGTCCACCCCCAAAGAGGTGGTGGAGGCC

TGCGACCTCACGTACCTCATGCTCTCTGACCTCGAGGCCTCCAAGACTGTGTACGAGGCC

GAGGACGGCGTGCTGGCGGGCGTCTCCGCCGGGAAATGCCTGGTTGACTGCGCGACCCTC

ACGCCGGAGCACATGATCAGCCTTGCCGAGCGCGTTCAGGCCAGGGGTGGCACCTTCCTC

GAAGCCCCCGTGTCCGGGAGCAAGGGCCAGGCTGCCGGGGGCACTTTGATTTTCCTGTGC

GGCGGCGACGAGGACCTGTTCGAGCGCACGAAGGGGTCGATGGACGGCACCATGGGCAAG

GCCGCGCACTACTTGGGCGACGTGGGGACGGGTTCCAAGATGAAGCTCGTGGTGAACATG

ACCATGGGGACCATGATGAACAGCTTGACCGAGGGCATCGCGCTGGCGCAAGCCGCCGAC

CTGCCAGCCGACAAGCTCCTGACCGTGCTAGACCAGGGAGCCATGTCCAACCCTTTGTTC

AGAATGAAGGGTGCGGCCATCCTCGACGGGAAGTTTGACCCGGCCTTCCCGCTCAAGCAC

GCCCAGAAGGACATGCGCTTCGCCGTGGCATTGGGCGACTGCTTAGGGCTGGCGCTGCCT

GTGGCCTCGGCTGCCAACGAGCAGTTCAAGCGTGCGCGAACGGAGCACGGCGATGAGGAC

TTCGCCGCCGTTTTCGCGGCCTCTAAaaagtag

>g16777.t1

ATGGGGATGTCCAAGATTAAGCTGGTGGGCATGCTCAGCATGCTCACCGTGACCCGCGTG

ATGGGTCAAGCATCTTGTGTTCAGCTCAACACCCAGAAGTTCTCGTCGTGTCCTTGCGAA

GATGCCACCTTGGCGTTCACTCTGGAAGGAGAGACGACGGTGGCAGCCCATACGGTAAAT

CTCGCCGTGGTGCTCGACGGGTCAGGCAGCGTCAGCGCAGATGATTTTAGGACGGAGCAG

ATCTTCGCCGAGCACGTCGTCGCAGCCTTTGCGAAGAGAAACCTGTTCGACAATGGCGGT

ATGGCATCGTACGTGCAGTACGCAAAACGTCTGGCATCCTCAGGCACTTTTGCCTCCAAA

GCAGACTTTAACGCATTTGCCGATGCCGACACCCAAGCTAGGGGGGGCACGACCACGTCC

ATCGGCATTAAGGAAGGCACCAGGCTCCTGCGCAAAAACCCTGCAAGCGCATCGTTCATG

GTCGTGATCACCGACGGCCGAAGCACCAGCATCTCCGACACCAGAAACGCAGCAAACGCG

GCCAGGGATGCAGGCATAGTCGTGTTTGCTGTGGGAGTAGGGAGTCAAACCAACCCTGCC

GAACTGCTCGCGATCGGAGGTCACGGTTTCAACGTCTACGAAGTCGCAGAATTCGACCTG

CTCGACGCGGCCTTGGAAAAGATCCTCTCGTCCGGCAAAGGCCTCGTCATCCCGTGCGAA

TCAACAGGAGCCGAGATCACGATCGAGTTCGGCGTCCCGGTCGGCTCTACCTTTCCCAAA

TCCTTGTCGACAACCACGGGCAGGACGGTCACCTTTTCCGTGGGCGATCTTGAGGCCACC

CCCACGGACTTCGACGTTATGCTTAACGTGTGCAACGAAGCCCCCGGTAGCAGCATCATC

TTATCCGTGGACTATAGGGACGACCAGAGAAACAAACCCGATCTGACCGCTCTGAAAGGT

ATGGTCGTCGGCAACGACCTATGTGCCGCGGCTCCAACCCCGGCGCCTACTCCGAAGCCG

ACCAGTAAGACCAACGTGAACGCACCCAAGCGCACCGACAACCTCAACACGGGAAAGGCG

ATCGATACCAAGTACCTTGGCTGCTACGTCCCCAAAAGGAAGAACAAGCTCCTAGGCGGT

CCCGTGACAACTTGGGACGGGATGACGACGCAGGTGTGTGCCGAAATCTGTGGTTGCTTG

GACTGGCAGTTCTTCGCCACGGTAAACGGTTCAGAATGCCACTGCGGTAGTAGCGAGCCT

gccaagaaaaagaagaagggcACGTGCGATTACGAGTGCGGAGGAGAACCTACCACTGCT

TGCGGAGGCGTCGGCAGCATCAGCGTTTACCAGAACACGAAGTTCAAGAAGCCCTAA

>g16885.t1

ATGGAGGTCGCACTGACTCCAGGGTCCATCCCGGCCGGGGAACCCTCTAGATCTGCTTCG

GCCTTCGTCGAgaacggcggcgggggcggcggcggcgacgacgcaCAACACGCCAGCAAC

AAGCGCAAGGTCGACGAGGCAAACCTGGACGCTTTTCCTGCGCCCGCGCCCGTGCCCGCG

CCCGCACGCACGCCCTCGCCCACGCCTGTTACCTCGACCAcacccactgctactgctacc

gctaccgctaccgctgctgctatttcgcCGCATACCGCCAACGGTAACGGTACCGTTACG

CCGCCACCCACGCCCCCCACGACCACccccaccatccccaccaccacgaccgccaccacc

atccccgCCCGACAAGGACAGGGCTCCTGCCGGGAGACGGCCatcgctctcgacagcagc

agcagcagcggcagcagcgacgaggAAGATGAAACAGACGTTCCGGTGGGCAACATGGCC

TCCCCGTCGCCCTCTCCGAGGGTTTCCATTAAGCTTTCGTCCGCAGCCGTGCTCGCCGCG

GCAGCCGCCCCCCGCGGGGTGGGCCGGATAcccggcggcgccgccgccgtcgctgccgcc

gccgccgccgccgggggtgcCCGTGGTGCTGGCGGTTCGATCAGCAACGGCTGGGGGCAC

GGCGCGGAGGCGGCGGGCGGTGGTTATGGCAACGGCTACCAACGCGCCACGAATGGGTAT

GGGAACGGCGTGGCGAGTGCCGGAAAATACGCGAATGGTAACGGTGCCGGCGCGGCGGCG

TCGGCTTCGGGTTTTGgttcctcttcttctccttcatCTTCTCCATCGGGCCTTAAGAAG

AAGGCTGCCGCCGGGTATCAGAGAAAGCTAGGGCGCCCGGTGGGGAGCAAGAGCCGGTAC

GATGACGAGGACGCCCCTTACGTGGGCCCGAGCTCTCCTCAGactCCTAAGGCTCCACAG

GCGCTACCTCCGAATCCCGTGGACGCTTCCATACCCGACGCCCCGACCTTCTACCCGACC

CCCGAGGAGTTCCGAGATCCCCTCAGTTACATCGAGTCGATCCGACCAATGGCGGAGAGT

TACGGCATCGCCAAGATTGTGCCGCCCAAGGGCTGGAACCCTCCGCCCACGCCGATGAAC

ATGCACGCCAGAAAGCGCGTGCCTACCAAGAAGCAGGCTCTCCACAGTCTCATGAACTCG

GACGAGGTTTATGATGACGGCAGAGACTTCACGATCGTGGACTACAAGATCATGGCGGAC

CGGTTCTCAGAGTACTGGCGTAACCGGGGCGACCCCGAGCTGAAGCCGCGGAAGGCCCCC

CCAGTCGAAccgcagACCCCCAAGGTGGAAACCCCGCCAGGCGCGACGGAGGCAGAGTTT

CACGAGGCCGCCAGGGAGAATGTCCGTCTGCGTCGCTTGGAGCGGGAGTACTGGAATGTC

GTGGATGGCGgggtggaggaggcggagGTGGAGTACGCGAATGACCTCAGCATCTCCGAT

TTCTGGAGCGGCTTCCCAATGCCGCCCAACAACTTCATGGACGGCTCGTCCTACGACCGC

GCGGCGCCGTGCCGCTTCGACGACCCGGAGTACTACAAGACCTGCGGCTGGAACCTGAAC

AACCTGCCATTTTGGCCCGGGTCCGTGCTGCGCTTCTTTCGGACCCACATCAACGGGCTC

ACGGCCCCCTGGTTGTACCTAGGCATGCAGTTCGCGACGTTCGCGTGGCATAACGAAGAC

AACTACCTCTACTCGCTCAACTACCATCACTCAGGCGCCGCGAAGCAGTGGTACGGCATA

CCGGGCAAGCACGCCATGGGGTTCGAGGCGTGCCTCGCGAAGGAGCTCGGCGAGACCCTC

GACACGGTGTCGGAGCACCTGTACCGCATCACCAAGATGCTGTCCCCGGTCTACCTGCAG

CAGGCCAACgTACCGGTGTGCCGTCTTCAGCAACTCCCGGGGCAGTTCGTGGTGACGTTT

CCCAAGGCCTACCACGGCGGCTTCAGCTACGGCTTCAACTGCGGGGAGGCCGTCAATTTC

GCAGTGCCAGACTGGATTTCGTACAGCAGGGAAAGCACGGAGGCGTACCGAAGCGCTTCG

AGGATGGCCGCCCTCTCCCACGACAAGATGGTGGCCACGCTCACCATGTACCTGCCCGAC

CACGACGTGAAAGGGTGTGAGCTCGTCGTTCGAGAGCTGAAGCGCATCCACAAGGAAGAG

CTGGACCACCGCGTCAGCCTGGAGCTCAAGGGCGTGCAGGACCCAGCAAGGCAGGGGGTG

CCTCTGCCGCGATTCCGGCTGGGCCTGATCGAGAAGGACACTGAAGAGTACGACGAAAGG

GGGGTGCCTCTGCCGCGATTCCGGCTGGGCCTGATCGAGAAGGACACTGAAGAGTACGAC

GAAAGGGCGGCGGTGGCATCATTCAGGGCGAACATAAATCCCTTCTGA

>g16965.t1

ATGGCTCCCGTATTCAACTACTTCGGCATCCCCGCCCGCGGCGAGGCCACCCGCGTCGCT

CTGGCCGTGGCAGGCGTGGACTTCGAGGACAAGCACATCTCGTTCGAAGATTTCGGCGCC

TGCGGGTTCAAGACCCTTCCCGTCTACCAGATGGACGGCACCGACTACACCCAGTCCACC

GCCCTTCTGCGCTACGCCGGAAAGCTCGGCGGCACGTACCCGGAATGCCCTCTCGCCGCC

CTCAAGGTGGACGAGATCGTGATGATCGCGGAGGACGTGTTCATCAACCTCTTCTCCACC

ATGGGCGAGAAGGACGAGGCCAAGAAGTTGGAGAAGTGCGAGGCTCTGATGGCCGGCAAG

GTGAAGGAGCTGCTGGAGGACATCGCTCGCAAGGTGGAAGCCAACAAGTGCTCGACTTTC

TGCGTGGGAGATTCCCTCACCATCGCTGACATCACGATCCACGCCGTGTTCGCCACTGTC

CAGGCCGGCTTCCTGGCTGGCATCCCCACAACCATGGTGGAGGACATCTGCCCCTCCCTT

AAGGCCGTCGATGACGCCGTCATGGAACACCCCAAGGTGAAGGCGTACTACGCTTCCAAG

GCATAA

>g16966.t1

ATGGCTCCCGTGTTCCACTACCTCGCAATCCCCGCCCGCGGCGAGGCCACCCGCGTCGCT

CTGGCCGTGGCAGGCGTTGACTTCGAGGACAAGCGCATGTCCTTCGCGGAGTTCGGTGCC

TCCGAGTTCAAGACCTGCCCCGTCTACGAGCTGGACGGCAACAAGTTCACCCAGTCGACC

GCCCTTCTGCGCTACGCCGGGAAGCTCGGCGGCACCTATCCGGAAGACCCGCTCGAGGCT

CTCAAGGTGGACGAGATCGTGATGATCGCGGAGGACGTGTTCATCAACATCTTCTCCACC

GTGGGCATGGAGGACGAGGCCAAGCAGTTGGAGAAGCGGCAGGAATTGATGGCCGGAACG

GTGAAGGGGCTGCTGGAGGACATCGCTAGGAAGGCCGACGCCAACACTGCCTCGCCCTTC

TGCGTCGGAGACTCCCTCACCATCGCGGACATCCAGCTGCACGCCGTGTTCGCCACAGTC

CAGGCCAACTTCCTGACCGGCCTCCCCACGACCATGGTGGAGGACATCTGCCCCTCCCTT

AAGGCCGTCGAAGACGCGGTCTTGGAACACCCCAAGGTGAAGGCGTACTACGCTTCCAAG

GCGTAA

>g16977.t1

ATGGCTCCCGTGTTCAACTACTTCCCAATCCCCGCCCGCGGCGAGGCCACCCGCGTCGCT

CTGGCCGTGGCCGGCGTTGACTTCGAGGACAAGCACATTTCGTTCGAAGAATTCGGTGCC

TGCGGGTTCAAGACCCTCCCCGTCTACCAGATGGACGGCACCGACTACACCCAGTCGACG

GCCCTTCTCCGCTACGCCGGAAAGCTCGGCGGCACGTACCCGGAGTGCCCTCTCGCCGCT

CTCAAGGTGGACGAGATCGTGATGATCGCGGAGGACGTGTTCATCAACCTCTTCTCTACC

ATGGGAGAGAAGGACGAGGCAAAGAAGTTGGAGAAGTGCGAGGCTTTGATGGCCGGCAAG

GTTAAGGAGCTGCTGGAGGACATCGCTCGTAAGGTGGAAAACAACAAATGCTCGGCCTTC

TGCGTCGGAGACTCCCTCACCATCGCCGATATCACGCTCCACGCCGTGTTCGCCACCGTC

CAGGCTGGTTTCCTGGCCGGCATCCCCACAACCATGGTGGAGGACATCTGCCCCTCCCTT

AAGGATGTCGACGACGCCGTTATGGAACACCCCAAGGTGAAGGCGTACTACGCTTCCAAG

GCGTAA

>g16979.t1

ATGGCTCCCGTATTCAACTACTTCGGCATCCCCGCCCGTGGCGAGGCCACCCGCGTCGCT

CTGGCCGTGGCAGGCGTTGACTTCGAGGACAAGCACATCTCCTTCGAAGAGTTCGGTGCC

TGCGGGTTCAAGACCCTCCCGGTCTACCAGATGGACGGCACCGACTACACCCAGTCCACC

GCTCTTCTGCGCTACGCCGGGAAGCTCGGCGGTACGTACCCGGAGTGCCCTCTCGCCGCC

CTCAAGGTGGACGAGATCGTGATGATCGCTGAGGACGTGTTCATCAACCTCTTCTCCACC

ATGGGAGAGATGGACGAGGCCAAGAAGtTGGAGAAGTGCGAGGCTTTGATGGCGGGTAAG

GTGAAGCACCTTCTGGAGGACATCGCTCGCAAGGTGGAAAGCAACAAGTGCTCCGCCTAC

TGCGTCGGAGACTCCCTCACCATCGCCGACATCACGATCCACGCCGTCTTCGCCACTGTC

CAGGCTGGCTTCCTGGCCGGCATCCCCAAGACCATGGTGGAGGACATCTGCCCCTCCCTT

AAGGCCGTCGACGACGCCGTCATGGAACACCCCAAGGTGAAGGCGTACTACGCTTCCAAG

GCGTAA

>g17010.t1

ATGCCAGCTTCGATGTACGGGCAGCAGCTCTTCGGGCGCGAGGCCGAGCCGCACGGAGCG

ACGAGCCACGCTCAGAAGTTCAACCAAGATGTGGAGGCGACCAAGCGGTGGTGGAGCAGT

CCCCGCTTCCGTTACACGGCCCGACCCTTCAGCGTGCAGGAAGTCGTTTCCCTGCGTGGA

ACGATGCCCGAAACCCACCCTTCAAACATCCAGGCGCGCAAGCTGTGGGACCTGCTGGAG

AAGAAACAGGCCGAAGGGGCATTCTCTCACACTTTCGGTGCCCTGGACCCCGTCCagGTG

ATCGAAATGGCGCCCCACCTGGAGACGATTTACGTCAGCGGCTGGCAATGCTCCTCCACC

GCCAGCACCAGCAACGAGCCTGGGCCGGACTTCGCGGACTACCCGTACGACACCGTGCCC

AACAAAGTCGACCAGCTCTTCAGGGCGCAAAGGCACCACGACCGCAGACAAAATGACGAA

CGTTGCTCCATGAGTGAGGGCGAGCGCGGGTCTACTCCGGCGATCGACTACCTTCGTCCC

ATCGTTGCTGACGGGGATACCGGACACGGGGGGCTAACCGCCGTCATGAAGCTCACCAAA

CTCTTCATCGAGAAGGGCGCTGCCGGTATCCACTTCGAGGACCAgaagCCGGGCACGAAG

AAGTGTGGGCACATGGGCGGGAAGGTGCTGGTGTCCATGCAGGAGCACTGCGACCGCCTC

GTGGCGGCTAGACTTCAGGCCGACATCATGGGCACCGAGACCGTTATCGTGGCTCGCACG

GATGCCGAGGCCGCCTCTCTGCTCGACAACAACGTCGACCCTCGCGACCACCCCTTCATC

TTGGGCGCCACTGTCCAAGGCACGAGagcGCTGAACGATGTGGTTCGCGAGGCGCAGGCG

AGCGGCGCGACGTTCGCAACCATCGACAAGCTCAGCAAGGCCTGGATCGACAAGGCGTGC

CTCATGCGCTTCCCCGAGGCCGTTGAGGCGGCCATCCACTCCATGCCCGGTCTTCAAGGG

CGCGACCAGCGCCTCAAAGAGTGGCAGTCCAAGGCCTACACCTTGGGGCATGCGGACGCT

AGGGCCCTCAGCGTGCGCCTTCTCGGTGGCAAGCAGATCCACTTTGACTGGGACGCGCCG

CGCAGCCGCGAAGGCTACTTCAAGCTCCGTGGTGGCGTAGACTACTGTATCGCCAGGGGG

CGCGCCTACGCGCCGCACGCCGACCTTCTGTGGATGGAGACCGCCAAGCCGAACATCCCC

GAGGCTAGGGCTTTCGCCAAGGGCATCCACGCCGTCGCCCCGCACCAAAAGCTGGCCTAC

AACCTCTCCCCCAGCTTCAACTGGGACGCCGCCGGCATGTCAAAGCGCGAGATGGCATCG

TTTAACCGGGACCTCGGCCGCCTGGGGTTCGTGTGGCAGTTCATCACTCTGGCAGGCTTT

CACTCGGACGGGCTCATCGTGTCGCGCCTGGCTAAGGAGTACGGAAAGCACGGCGTGATC

AAGTATGTCGAGCTGGTGCAGAGGCCGGAGCGAGACGAACAAGTGGACCTGCTGACGCAC

CAGAGGTGGTCCGGGGCAAACCTCATGGACAAGCAGGTGAATACTGCCACCGGAGGCCTG

GCGTCGACTTCCGCCATGGGAGAAGGGGTCACAGAGTCTCAGTTCGTCAAGcacagcgga

ggaggaggaggaggcatgcGGAGGGGCGGGGCCCATTCCGTTGCGGTTTCTTCCGCTGCC

GGCGCCTCAGGATTCAGGGCCAAGCtctga

>g17168.t1

ATGTTCAAATCTTGGGCTATGGGATCTGTGCTGGCGCTGAGCGCCTTATCCACTGCTAGG

GCGGCCTGCGACGCCCTCGGGAACAACCTGGATGTTCCTTCGCCCTTGACCGaagacacc

accatcaccttgaGCGTGGGAGTCATCGACTGCCCAGCACTGGACGTAAACACGAACCGC

GTGGAAGTGGAGCAGGTTGTCGTCGAAGGCGGCACTCTCACCATCAAGAGCGACAACGCC

GTGCAGTTTATCAACGTGGCCTTCCTCGTTATGCCTGGGGCCGCCCTCGTGTTCGACATG

CCCGAAACCTTGTTCGGACCTAATGATAGGTCGGGGTTTGACTTTCCTCGGGGCACGCTG

CTCGAGGTAAGGGAGGGAACCAGTCTTACGTTCAACGGAGACCTCACAATCCAGGAGGTC

AGCAACGTCAACATCGTCATTGAGAACGCTGGGGACCTAGAGTTCAAGAAGACGATGCTA

ATCGAGAACAGCGAGAATGCGATCCCGGCAAACAGTGGCACTATCAAGTTCCGCGGCGAT

GCGACGTTCAGAGGAAATGATGGTCTCGGTATCGATAACTCCAGTGGTGGATTCGTGAGG

TTCTCAACGACCGCGACGTTCGACGACAACGGAAGCCAATTGGAAGGATTTGATGGATGC

AACATCCAGAATGACGCCACCTCCGAAATAAGCTTCCGAGGTGACGCGACGTTCATCAAC

ACCGGCTGTAGGCGCGCCGAAGGCTCGCCGGTGTACAACGCCGGACTCATAAACTTCTAC

GGGAAGGTTTTCTTCAATGATAACGACTCCGACGGCAACGGTGGTGCCGTTCTGAACGAC

GCTGGATCAATCAACTTCAGAAGCGCTGTGGAGTTCAACAACAACGAGGGAAACGAGAAC

GGTGGTGGTATTGCTGTCGACGGTGGAGACGTCACGTTCCGGAAGGCGGTAACGTTTAGT

GGCAACCGTGCTGAATCTGGTGGGGCCTTCGCGGTCCTAGTTCGCAGGGACTTAAGAACT

GGTGAGGTGGTCGAATCAGGCGTCGGCTCGCTTACCTTCGAGATGCCGTCCGCAGTGACA

TTCGAGAACAACAGCGTCTTCACCGGCGGCGGGGACAACGACTGCACCGTAGGCGAAGTT

GAGGAGGGGACTACGCTGGTTGGCTTCCCCGGTGATGACGTGTGTGTCTCGGCTACGTAA

>g17171.t1

ATGTTCAAATCTTGGGCTATGATGGGATCTACTTTGCTGGCGTTGGGCGCCTTGTCCACT

GTTAGGGCGGCCTGCGACGCCCTGGACAACTTCCTGAACGTCCCTTCGCCCTTGACCCGA

GACACCACCATTACCCTGGACGTGGGAGTCATCGAGTGCGAGCACCTGGATGTGGACTCG

AATAGCATGGAAGTGGAGCTGGTCGTCATCGAAGGCGGTACTCTCACCATCAAAAGCGAC

AACGCCGTGCAGTTTATCAACGTGGCATTCCTCATTATGCCTGGGGCCGCACTCGTGTTC

GACATGCCCGAAACCCGATTCGGGCCTAATGATAGATCGTCGTTTGACTTTCCTCGGGGA

AATCTGTTAGAGGTACGCGGCGGAACCAGCCTCACGTTCAACGGGGACCTCACAATCCAG

GATGTCAGCGACGTAAACATCGTCATTGACAACTCTGGAGACCTAGAGTTCAAGAAGACG

ATGCTTATCGAGAATAGCGAGAATGCGATCCCGGAAAACTCTGGTACCATAAAGTTCCGC

GGCGATGCTACGTTCAGAGGAAATGATGGCCTCGGTATCGATAACGTCAATGGTGGATTC

GTGAGGTTCTCAGAGACCGCGACGTTCGACAACAACGGACGTCAGTTGGAAGGCTTTGAT

GGCTGCAACATCCAGAATGACGCCACCTCCAAGATGAGCTTCCGAGGAGACGCAACGTTC

ATCAACACCAGATGCAGGGGCTCCGAAGGCTCGCCGGTATACAACGTCGGAATCATGAAC

TTCTACGGGAAGGCGTTCTTCGAAGACAACAACTCTCAGAACAATGGTGGCGCTCTTCTG

AACGACGCTGGATCAATAACCTTCCGGAGCGACGTGCAATTCAACAACAACGAGGCAGGC

GAATTCGGTGGTGGTATCGCTGTCGATGGTGGAGACGTCAGGTTCCAGAAGGCGGTGTCG

TTTAGCGGTAACAGAGCCCTTTCTGGTGGCGCCTTCGCTGTCCTAGTTCGCAGGGATCCC

AGAACTGGCGAGGTGGTCGATTCTGGCGTCGGTTCGCTTACCTTTGAGAAGCCGTCCGAC

GTGACATTCGAGGACAACACCGTCGATACCGAATTCAGGGACGACGACGACTGCACCGTG

GGCGAAGTTGAGGAGGGAACCACGCTGATTGGCTTCCCCGGTGATGACGTCTGCGTCGCA

GCTACGTAA

>g17172.t1

ATGACGCAGTCGAGGAATGTTTTCACCAACTCCCCCGCGCGCGAGCGCAAGACTCACAAG

GCCGGCTTTGTTGTGGGAGACAATGTCGGGTCGTGCGGGTTCGCGAACTGCAGTGGGCTG

GTCACGCGGCTTACCACGTCCGTCGCTGGGGCGGCCTGCGACGCCCTGGACAACTACCTG

AACGTTCCTTCGCCCTTGACCGgagacaccaccatcaccctgGACGTGGGAGTCATCGAG

TGCGAGCACCTGGATGTGGACTCGAACAATAGGGAAGTGGAGCTGGTCGTCGTCGAAGGA

GGAACTCTCACCATCGCAAGCGACAACGCCGTGCAGTTTATCAACGTGGCATTCCTCATA

ATGCCTGGGGCCGCCCTCGTGTTCGACATGCCCGAAACCCTCTTCGGACCTAACGATAGA

TCGTCGTTTGATTTTCCTCGGGGAAATCTGCTAGAGTTACGCAGCGGAGCCAGCCTCACG

TTCAACGGGGACCTCACCATCCAGGAGGTCAGCGACGTAAACGTCGTCATTGACAACTCC

GGGGACCTAGAGTTCAAGAAGAAGATGCTTATCGAGAATAGCGAGAATGCGATCCCGGGA

AACTCTGGTACCATAAAGTTCCGCGGCGATGCTACGTTCAGAGGAAACGATGGCATCGGT

ATCGATAACTTCAATGGTGGATTCGTGAGGTTCTCAGAGACCGCAACGTTCGACAACAAC

GGACGTCAATTGGAAGGCTTTGATGGCTGCAACATCCAGAATGATGCCACCTCCAAAATG

AGCTTCCGAGGAGACGCGACGTTCATCAACACCAGATGCAGGGGCTCCGAAGGCTCGCCG

GTGTACAACGTCGGGATCATGAACTTCTACGGGAAGGCGCTCTTCGAAGACAACAACTCT

TGGAACAACGGTGGCGCTTTTCTGAACGACGCTGGGTCAATAAGTTTCCGGAGCGACGTG

AAGTTCAACAACAACGAGGCAGGCGAATTCGGTGGTGGTATCGCTGTTGATGGTGGAGAC

GTCAGGCGAGTGATGCGGAACCAAATCGGCAGTTTTTCCAACGCGATTCTCTGGCGTGAC

AGGTTCCAGAAGGCGGTGTCGTTTAGCGGCAACAGAGCCCTTTCTGGTGGCGCCTTCGCT

GTCCTAATTCGCAGGGATCCCAGAACTGGTGAGGTGGTCGATTCTGGCGTCGGTTCGCTT

ACCTTCGAGAAGCCGTCCGCAGTGACATTCCAGGACAACAGCGTCTTCACCGGCGGCGGG

GACGACGACAACTGTACCGTGGGCGAAGTTGAGGAGGGACCCACGTTGATTGGCTTCCCC

GGTGATGACGTGTGCGTCGCAGCTACGTAA

>g17300.t1

ATGAGCGCGTGCGCGAGTCGTGGGGTGGGGAGTTGCGAGATGGACTCTAATGGCACGTGC

GAGATCAAGGCAAGAGGCAGAGCGGCGAAGATTGACATCACGTGTGACGCCTTGAAGGGC

AACCAAATCGACTGGGACTGTGTGTTTGGGCCGGAATCGTTGGAGGAAGTGTTTGATCGA

GGGGGGAAAATGACCACGTGCAAGGAGAGCACCAAGTCAGAGCCGTGCTCCAGGTTCATC

GTGCCCAAAGAAGAGGAACGCGCGGAGGTCAAGGTTCAGGCAGGGCCCGAAGGCTTCCCT

TTCCTCAAGCACGAGAAGTACATCTTCCGGTACAGCTTCCAGGCAAAAGAGGGCATGCGC

GTCGCCAACAGATTCACCCACCTCGGGCAGCTTAAGGGCTCCAAGAACCGGAGGATGCTC

AAGGGCGACCCCATCTACTCCCTCACCGCGAACGAGCACGGCCTGAACGTGCGCTTCAGC

AACCTCGAGTCCATCGAAGACTTCCACGACGGCATGGAGAAGCACTTGGACTGGGACAAG

GCTACCGGGGAGTGGGTGCACGTCAAAATTACTACCGTCTTTGGGGAGTCGATGGAGGTT

GAGCTATCGGGAGCGGTCAGCGGGAAAGCTGTTTGGCCGAAGCACCTGGAGCCGGTTGCC

TGGCACCGCGACTCCGAGATGGTCCGGATGAAGCTGGGGCtttaccactccaaaggaaac

GTGCACGACCAAGAAGTAATTTACAAGAACGTCTCCATCGAGGGGCCCAACGGCATCATC

AGGTCTGGCGACAAGGCCAACAGCGAGCCAAGCGACAAGGATTGCGCCAAGAAGGATGAC

AAGAAGAATGACAACAAGAAGAAGGACAACGATATTGCCGAGGGCACGTGCGAGGTCGAG

GCAAAAGGGAGAACGGCGAAGATTGACATCACGTGTGAAGCCTTGAAGGGCAACCAAATA

GACTGGGACTGTGTGTTTGGGCCGGAATCGTTGGAGGAAGTGTTTGATCGAGGGGGGGAA

ATGACCACGTGCAAGGAAAGCACCAAGTCAGAGCCGTGCTCCAGGTTCATCGTGCCCAAA

GAAGAGGAACGCGCGGAGGTCAAGGTTCAGGCAGGGCCCGAAGGGTTCCCTTTCCTCAAG

CACGAGAAATACATCTTCCGGTACAGCTTCCAGGCAAAAGAGGGCATGCGCGTCGCCAAC

AGATTCACCCACCTCGGGCAGCTTAAGGGCTCCAAGAACCGGAGGATGCTCAAGGGCGAC

CCCATCTACTCCCTCACCGCGAACGAGCATGGCCTGAACGTGCGCTTCAGCAACCTCGAG

TCCATCGAAGACTTCCACGACGGCATGGACAAGCACTTGGACTGGGACAAGGCTACCGGG

GAGTGGGTGCACGTCAAGGTTACTACCGTCTTTGGGGAGTCGATGgagGTTGAGCTATCG

GGAGCGGTCAGCGGGAAAGCTGTTTGGCCGAAGCACTTGGAGCCGGTGGCCTGGCACCGC

GACTCCGAGATGGTCCGCATGAAACTGGGGCtttaccactccaaaggaaacGTGCACGAC

CAAGAAGTAATTTACAAGAACGTCTCCATCGAGGGGCCCAACGGTATCATCAGGTCTGGT

GACAAGGCCAACAGCGAGCCTAGCAACAAGGATTGCGCCAAGAAGGACGACAAGAAGAAt

gacaacaagaagaagaaggacaACGATATTGCCGAGGTCAATGGGTACACCTACAAGGGC

TGCTTCCGGGACACCAAGGAGGACCGCGTATTCCCCTCCGAGTCAAAGAGCGACTCCATG

ACCACCAAGAAATGTGCGGAATACTGCAAGGGTTCCAAGGTGTTCGCGACCCAGCACGCC

AGGGAGTGCTGGTGCGGCGACTCCAACACCGACTTCGACAAGCACGGCAAGGCGGACGAC

TGTAACTACACCTGCTCGGGCTCCAAAAACGGCGAGATTTGCGGCGGGAAGTGGGCCATG

AGCGTCTACCAAGCGGAGGGCAAGACTAAGTCTGACGGTAAGGATGGCGCCCCCGACGGC

ACCACGTACCTCGGCTGCTTCAGAGACGACAAAGTTAATCGCGCCTTGGGCCTGGTCGAG

AAGAACGTCAGCGACAGCATGACCCTGAAGAAATGTCACAAGTTTTGCTCTAAGAAGAGC

GGCGCCACGCACATGGGGCTCGAGTTCAGCACCGAATGCTTCTGCGGGGGCTCGAAGGAC

AACCACGACGTCTACGGGGGTGGCAAGTGCGACATGCCGTGCGCTGGGGACTCTGGCTCG

GTTTGCGGGGGACGGTTGGCTCTGAGCGTTTACAAGATCTAA

>g17383.t1

atgaTGGATGATCCCGAAGCCACACCGGTGACTGCGGCAGAGCAAGAAGTGCTCGACAAC

GCATCGACGACTCTGGATACAAGCAGACACGATGGCGATTGGAACTACTACAAGCGGCAG

GCGTACGTCCAGAAGGACGTGATTGGCCAGCTCAAGTCCTTGCTGGAGAACTACGGCTAC

CACCTGCCCGcgGACCCAGCGTCGCTGAAGGCAGCTTTGTCGCACCACCTCGCCGACAAA

GCTCACGAGCCCCTCTTGCAAGGAGACGAAagcaggggggagaagggagactCGTCGGGT

gcgccttccaccccccccgcagCGCGGGGAATACTGACCCAACGCTCCTTGTTCATCACC

GATACGGAGATTTCCAACGCGGAGAAGTTCGAGCAGCTGACGAAAGAGCTGCCTCAGATC

CACGGTGCCGGCTGCGAGCTGCGCGTGAATGGCCTCGGGTTCTGCGTGCAGCGGGCTAAG

GGCAGCGCCGATGAGCCGACTGTTGGCGACAGCATCGTGTCCGTCGGCAAGTGCCTGATG

TGCCTTCCTCTCATCAACCGCCTCACCAAGGGGAAGGAAATGGAAAGCAAGACGATCCTC

CACGACGTCAGCGCCGTGTTCAAGCCTGGAACAACCACGCTCGTCCTCGGCCCTCCGGGG

TGCGGAAAGTCGACGCTGCTCAAGTCGATGGCGGGTCTGCTGAAGCACGACGCAGGACAC

GTCAACACGGGAAACGTGACGTACAATGGTCGCACCAAGGACTCCAAGGACTTTTCGCTG

CCCAAGCTGGTGCATTTCGTGGAACAGGTGGACAGGCACTTGCCGACGATGACGGTCGAG

GAGACGCTCAGGTTCGCCTTCGACTCCATGGCGGGCGGGACTCACCTCGGAAATCTTGAT

GGAAAGGACCTGAACTTGACCGACGAGCAGAAAGACCTGGTGGCATGGATGGACGCGAAG

CACTTCAAGGTGGAGATGATCATCCGGAACCTGGGGCTGTACAACGCTAAGGATACCATC

GTGGGGAACAACGACCTTCGAGGAGTCAGCGGTGGGGAGCGCAGGCGTGTCACCCTGgGT

GAGATGCTCTGTGGCCCTCAGGCGGCGTACCTCATGGACAGCATCAGCACCGGCCTCGAC

ACCAGCACGACCTTCGACATCATGCAGACTCTCAAGTCGGCATCGAACGTATTTCGCAAT

ACGGTGGTGGTCGCTCTTCTTCAGCCCCCTCCCGAGACGTTCGAGCTGTTCGACAACATC

TGCCTCATGGCGGAGGGCAGGATCATCTACCACGGTGGGCGCGAGGGCATCGTACCTTAC

TTCAACAGCCTCGGGCTGACCTGCCCCCCGCGAAAGGATGAGGCTGATTGGCTGGTCGAG

CTGACGGGAGACGCCGGGGAGCCCTACCGCGCCGACCCAGTGGAGAGGATGGCTCTGGGT

CTTGCCAAGGTGCCCGTGACAGCCGAGGAGTTCCATGCGAAGTGGCTGGAAAGCGACGGG

GGGAAAGCCATCGAGAAGGAGCTGGCGGGCAAGGGAGAGCTAGACGGCGCGGAGTGGGAC

GCGGTGCACAAGAACAGGTACCCCAAGTCCTGGTGGTACCACATGAAGTTGTGCCTGCAG

AAGAAGTATATGCTGTTGATCAGGGACACGGCGTACATCAGGAGCCAGGTCATGAGCGCG

CTTGTGATGGGACTGATCGTGGGATCGATCTTCTACGACCTCGACCTGGAAGATGCCAAC

GCCAAGTTTGGTCTCATCTTCTTCTCTCTGTTCTTCCTGGCGATGTCGGGAATGGCTCAG

ATTCCCACTGCCATCAGCCAGCGGGGAGTTTTCTACAAGCAATCGATGGCCGCGTTCTAC

CCCACCTCTTGCGAGGTTGTGTCGGACACGCTCGTCAACACCGGCCTAACGGTGTTGTGC

TCCGTCATCTTCGCTCCCGTGATTTATTTCATGGTCGGATTCAGCCCTGCTGGGAACGGA

TGGAGGTTCTTCACCTTCATGGTCATCGTCATCGCCACGAACTTGAACATCACCCAGTAC

TTCCGCTTCCTGGCGTCGTTCATGCCCAACTTCACAGCGGCGCAGGGTTTCGCCGGGCTG

AGTACCTTGCTCATGGTCCTGTTCTGCGGCTACCTCATCCCTGCCGACAACATCCCGGAG

TGGTGGATCTGGATTTTCTGGATCAATCCCATCGCATGGGCCTTCCGCGCGGCCGTCCTG

AACGAGTTCCAGTCTCCCGAGTACGATGACGTCTGCGTCAACGATGTGTCGGAGGGGCAG

GAATGCCAGACGCTCGGACAGACATTCATCGACGCCTACGGCTTTGCCGACGACAAGGCG

TACATCTGGGGCGGCATCGCCTTCATCTTCGCCGAGTTCGTGCTCTGCGCCGCCGCCACC

GGAGTCGCGTACAGCTTGTTCCATTGGGATGCCTCCGACAGCGCTCCCATCATGCCCACC

GCTACGGGCGAAGACGAGGAGGCGGACGAGACCAGGATGGCGGGTGCCGACCAGTTTAAC

GCTCCCGTCCCGATGCTGAAGCGACAGCCGTCCGAGCTGCAGAACGACCTTGccttcgaa

cccgtgacccttaCCTTCAGGTATGAAACGTACAACGTCGACGTTTTGATCCGAAATGAG

CGCCACTAG

>g17384.t1

ATGTCGTACAGCGTGCCGCACCCCTCGGGCGACGGCAACCTGGAACTCCTCAGTGGCATT

TCCGGGTTCTGCAAGCCTGGcgagATGACGGCCCTCATGGGTTCCTCGGGAGCGGGAAAG

ACGACGCTCCTGGACGTGCTCGCGGGTCGCAAGACGGGAGGAACCATCACGGGGGACATC

CGCCTCAACGGGCACCCCAAGCTCCAAAAGACTTTCACCAGGGTTGCCGGCTACGTCGAG

CAGCAGGACATGCACTCCACCGTCGTCACCGTCAAAGAGGCCCTGATGTTCAGCGCTACC

ATGCGGCTAGAGGAGGAAGTGGTCAACAAGGAGCGCCGTGAAGGGTTCGTCGACAGCATC

CTGTCGATGCTTGAACTGGACGTGATCGCCGATCGACTGATCGGTAGCGACGAGGACGGG

GGGCTTTCCCTCGAACAGCGCAAGAGGACCACGCTGGGCGTAGAGCTGGCAGCCAACCCG

AGCCTCGTTTTCCTGGACGAACCTACGTCGGGACTCGATGCTCGCTCGGCACAGGTGGTG

ATGCGAGCGATCCGGAAGGTGGCGGCCACGAATCGCGCGGTGATCTGCACCATCCATCAG

CCATCCACCTACCTGTTCGAGATGTTcgactctctcctcctcctcaagaAAGGCGGGCAG

GTTGTGTTCTTCGGCCCGCTCGGCGAGGGGTCCTCCAAGCTCATCCCCTACTTCCAGAAC

ATTCCCGGCACAACGCCTATCCGAGACAACGTCAACCCGGCCACGTGGATGCTGGAGGTC

ATCGGTGCGGGCACCTCGGGGAAGGTCAATCCTCAGATGTACGCCGACTACTACAACAAG

AGCAAGCTCAAGGAGGCCACAATGCGCCAGCTGAAGCAACTCATGGTTCCCGCTGAGGGG

TCGGAGCCGCTGCACTTCAAGTCGATCTACGCGGCCTCGACAGGCCTTCAGGGGCGGGCG

TGCATGGAGAGGGCGGTTATCCAGTACTGGCGCAACCCGGCCTACAACTTCATGCGGATG

CAGCTCTCCGTCCTTATCGCCGTCATTTTCGGCAGCAGCTTCATCGACGCCGATATTGAA

ACCGAAGCGGATGTCGCTTCGCGACTGGGCGTGATCTACATGTCGACCATGTTCATCGGT

GTGATCTGCATGCAAACCGTTATCCCCTCGGGAGCCAAGGAGCGCATAGTCTTCTACCGC

GAGCAGGCGGCCAACATGTACAGCGTGAGGTCGTACGCCATCGGCTACGCAGTCGCCGAG

ATGCCCTACGTCCTCTTCATCACGCTCGCATTCTGCTCCATCTTTTACTGGATCACGGGC

CTCGCAGACTCCGCCGAGCAGTTCTTCTTCTACTGGCTTTACTTCGTCCTCTGGACGGCA

TTCATGGTCTTCACCGGCATGCTGTTCGTCATGATTCTTCCGGACACGAAGGTGGCGCAG

ACCCTGGGGGGAGTTCTGTCGAGCATGTTCAGCCTGTTCGCGGGCTTCCTCATCAGCCCC

GCGAAGGTTCCAGGGTTTTGGCTGTGGGCGTATTACCTCAACCCCCTTCACTACGTCGTC

GAGGCGACAACACAGTACAGGGGCGACAACACGATAATCTCGACCGCGACGGGCGGCTCG

CTCACCGCCGAAGAATACGTCAATGGCTTCTTCGGGGGCGAGTACAAGTACTCCAACCGC

TGGTATGACGTTATGGGACTGATTCTGTTCATCGTCGCTGTTCGCCTGGGGTACCTATAC

GCCCTCAAGTTCGTCAGGCACCTCAACCGTTAG

>g17846.t1

ATGAGGGCGGCAACATGCTTCGCGCTGCTACCGGCTGCCTCAATCCTGATGGCTCTGGTT

CATCACGTACCCAAGGCGAGCGGCTACACGTACAACGTGTTGTCGGACGCAGAATCAGCA

TCATCAGACGACGACTCGGCGTCatcgtccttctcctcctctgccggCGTCAACTCCGTC

AACAACGACGTGGTCTACAGCCTGCCCGACGCGCTCTCGAAGGCAAAGCCGGGGGACACG

ATCGTGCTCGCGAACGGGACATATATTGACCGGTTGGAGAGCTATGCCAGCGGAGAGGAG

GGGAGCCCGATCACGATTGTCGGAGAGCGGGGTGCGGTGCTGCAGTCTATTTCCCCCTCC

GTTCACATCGAGCACTCGTGGATTACACTCGAGGGGTTTACCGTGGACGGTTTTCACGGT

TCGGCGTTCGAGGCGGACTCCTTCGTGAACAAGTGCGTCGAGGTCATCGGCAAGGGGGAA

AACGAAACCGAAGCCATCGAAGGGTTCGTCATGAAGAACATGGCCATCCAAAACTGCGGG

GGAGAGTGCATTCGTCTCAAGAACTTTGTCGTGAACGCCAACATAACCAACAACACCATC

CAGCACTGCGGCATGTACGACTTTGAGTTCGGCGGTAACTCCACCACCAGAGGGAAAAAT

GGAGAAGGCATTTACATTGGGACATCGTCCaaccagTGGACATACGGGGAGGACCGATGC

AACGGCAATCTTGTGAGCAACAACCGTATCGCGACGTACGGTAACGAGTGCGTGGAGATG

AAGGAAGGGTCGTCCGGCAACATCGTCGAGAGCAACGTCTGCTCCAACCAGCGTGACGTC

AACTCCGGCTGCTTGGGGTCCAGGGGTGACTCCAACACCTTCAGGTTTAACAACGTTTCT

GACTGCGAGGGAGCCGGTGTGCGACTAGGAGGCTGGGAGAAGGAAGGGCACCAGTACGGC

CAGAACAATAGCGTATACGGTAACAACATTTACCACGTGGACAATGGCGCAATCAGCGTG

CAAACGTCGCCGCAGCAAGCCATCTGCGACAACGCGTGCGAGGACGGCTCGTGCAACTTC

ACGGGGGATGACGACAGCCTCGGCGACCTGGATGGAACGTGGGACACGGCTTGCCCTAGC

GAGGCATTTGAACAAGACGGTGGTGGAGAGGACGATACCACCGACCGAGAAGAAGAGGAG

ACGTCTACCGACGACGAGGAGACGTCTACCGGCGAAGAGCAGACCTCTACCGGCGAAGAA

AAGACGACGTCTACCGGCGAAGAAGAGACAACGTCTACCGGGGAAGAGGAGACGCCTACC

GACGAAGAGGAGACGACGTCTACCGACGACGAGTACTCATTGGGCGATGATGCTACCGAC

GATAACGCCGGCGATGACGTCGCCAACGACGATGTCGACGAAGACGGAGACGAAGACAac

aatgacgacgacgacgaaacaGACGATGCAAGCAGCCGTGATGAATCTCGAAGGCGTCGG

CTGCGGGGTGCGTCCAACTAG

>g17924.t1

ATGCCTGCCATGGCCCGGTTGACGAGCCGtgctctcctcctcctgtcctGCATGCCTGTG

GCTTCATCAACGTCAGAAGAAGTGTCTTCGAAAATAAGCCCGCCGCCACCATATCCTTCC

TTGAGGCTCGTTGAGCAAACTCTTCTAACAACCGCAAAGCTGGACTTCAGCGGTCACGAT

CAAGCTTTCGAAGCAGCCACCAGATGGAACGGAGAGAGACTGCAAGCCCACCAAGGCGAC

AGCAAGCCGCGTGCGCCGCACCTGGCCTGCGCAGAGTACGGCCACGGTCGCGAGGCCGCT

TCTCTCCTGAAAGCATTTCTCTCCCCTGAAGCTGTGAAGCCCGTGTACCACTCCAGCGAG

CACGGGGCTTGCTTCTTGGCGACGGCCTCGGACGCCCAGGCGGCTGCAATGTCCACGGAG

CCGGCTAGGTTCCAGCTGACGAGCGTTGGCGCTTTCCCATCCGCGCTTAAAATGGCCCCT

GGTCTCCTTGACCACGATGGCAGCAGCACCCGTGAGACTTCTTCTGCTGCAGAGGAGGGG

AGCAATGAGTCGGGTCGGCTGACCACGAGCCACGGCTACTTGATGCGAGTCAACGACATC

GCAGGGCTTAGCGTCGAGCTCTCGCCTGGCACCTTGCCCGCGCACTCTTTCGAGGCCGAC

GCGTTTGTGACAGACTTGCTGGAGGGCCTCATGTCCGAGTCCGTCGACCTTCACGCCGGT

AACTTCTGGTCCGATCCCTCCATGTCGGGGGGTGAGCATCTCGCCAGGCCGGAAGGCGCT

TTGCGTGGGCGGGAGTGGAGCAGAGCTGCGGCGGTGGTGCACGAGCTGAGCGAAGCGGCA

GGGACGACCCCCGGGGACGTTTGCTCGTGGGGCGGCCTTGTCGCGCACCATGCCGCCAAC

GATGTGCTGCTGGTCTCAGGCTTGGACCACCTCTTGCACggtggaaggggggcggggggg

cagcggGAGGAAGCGACCGAGCTGCacgtggcgtgcttcatgggcCTTGTTTCTTTCCTG

GCGGGTAGAGTTGAGGTGCTGCGGGTCTCGCCGTGGCAAAAGAAGGCCACCCTCAACGCC

TCCGCGCGGGCGAACATACAGAGTGGTACGGTCACGGCTACCCCGCTCACAGATGCAGGG

CTGGACGGTACAGGCGAGATTatccagATCCTAGACTCTGGGCTCGACGAGACGTCGTGC

TACTTTGAAGACGGTGACGGGCTGGAGGTGACGCACGGGTACTTCTTCGACCAGATTGGC

TTGATACCCGACTCATCCTCGCAGGAGAGCTCTATCGCGGCGTACGCCTTCTTTGAAGGT

GGAGATTTcttcatcgacaccaccaggcGTAAGATCGTTCAGTACATCAGCATGGTTAAG

CCCGACTCAACCCCGGGCTCATCCACCTCCACGTTCATCACCGGCGAAGGCGACCGCGGG

TACTGGATACCCGCGGACGCCTTCTCGGCAGATGACAGGGCGGGACACGGCACCCACACG

GCGGGGTCGGCGGCGGGGTCCACCCTGAACTCGCCCGCGGAACTGGTCACGTGCGCCGGC

ACGGACGTGTTGAGCTGCGCCGGAGGGTGCATCGACGATAACTCGATTTCCATCACGGAC

GACCTTCTTCCGCTCTACGAGCAGATATTCACCTCCATCGACATCGACAGGATCTGCCCG

CTCTTCGACTGCGACGAAACCGTCTCTGAGCTGTGTTTGAGCGAAGACGTGGCTCAGACC

TTGACGGACAATGGAGGCATGGCTCAAGGCGCCAAGCTGTCGATCTTCGACGTGTTCTAC

GAAGAAACTGGGCTGAGCGATTTCCCCGGGAACGGGCTGTGGGAGCCGTGTATGGACGCT

GGGTGCAAGCTGCACTCGAACTCTTACGGCGGCGACCTGTTGTGTTCCTCGTCTTCGATT

GATGCCGAGTACGACGACTTCATGTACAATaaCCCGGAGAACCTGCTGATATTCGCAGCC

GGAAACGACGGTGACGCCAACGATGGGCGCTCTGTTTGCACCATAAGCAGCCCGGCGATT

GCGAAGAACATCCTCGCGGTCGGTGCTACTTCGTCGGGGGCATCGCGCTACACCTCTACC

GCTCTAGACGGCACTCCCGCTGACGGGACTAACGGCTTCGCCGATATCGACACCGTCGCT

GCTTTCAGCTCCTACGGTCCCACGCAGGACAACCGCATCAAACCGGAAATCGTCGCTCCA

GGAGACTTGatctACTCTGCGGCCAGCGACGGCAGCGACGAGCACTCGTGCCGGCTCTAC

GCGTACCTTGGCACGTCCATGTCGTGTCCGATCGTGGCGGGCGCATCGGCGATGATACGA

CAGTACTTCGTGGATTCGAGCTTCTACACCGCCGATGTGACGGCAAGGGGCTTCTGCGGG

CAAGGGTTCCCCTGCGAGGGTTTCTCGCCGTCTTCCGCCACCGTGAAGGCGATGCTGATC

AACAGCGCGAACCTCATGGGCGGCAGCTCAGAGCCCGACGGCTTCCGCGGCTTCGGTCGG

GTCCACCTGGAACAAGGGATCACcctggagggagagggggacatGGCCCTCTTCGTGGCC

GACGCCGCCAACACCTCCATCTCCGAGCTGACGCAGCACGAATACCTCTTCGACGTCGAC

GGCGACGCGGGGCTGGACCTCCGCGCCACGCTCTCCTGGATAGACCCCGCCGCCACTGCG

TTGTCCGCGAAACAGCTGGTCCACGACCTGGACCTCGCGGTTCGCTCGCCGAGCGGAACG

ACCTACACCATGTGGGCCTCGGGAGACGCCGACGAGGACAACGTGAACGAGAGGGTTATC

GTTGACGCGGAGGACGTCGTGAGCGGGACGTGGgcggtgtgggtgtgggcgcaGGCTCTC

AGCACCGACTCCCAGAGTTACTCCTTGGTCGTGAACGGGGCTATCAGtccggggacgggg

gacgggacgggtgcGGGCAGTACGGGGCCGTTTATCTCCTCCGACCCTCCGTCCCTATCG

CCTGACAGCTCGCTGCCTGAAGGCGACGACGAGGGTCTGGCTACCCCTTCGCCCGCTTCC

TCTAATAACGCGGGGACTTCCGGCGCGGTGCAAAGGTCgaccccggcggcggcggtgacc

tTTTTGTCGGCACTTTTCTGCTCTATCGCGTCGACCCTcgtagtcgctgctgctgcgttc

cGCGAGTGA

>g17925.t1

ATGTGGAATTATCCATGCCACCAGCTGCGGGGCctcctggtgttgctgctgttcgtagCC

CCAGCTGCACAGGGGGCCTCGCGATGGCCGCGACCCTCGCTCTCTCTAGTGAAGCAGGTT

TTGGTGAGCACAGCGCACCAGGAATTCGACAGTGATGCCGCATTCGAGGCAGCAGCAGAA

TGGCACGGGGAGAGGCTATCGCGGAGCCCATCCCGCCAGGCACCCCATCTGGCCTGCACG

GACTACAGTAAAGGAAGCCGAGCCCTGCGAAAGCTTGAGGCATTCCTACCCAGCGGGTCT

GTCCGCAAAGTCTCCAATCATAAGCTCCACGGTGCGTGTTTCATCGTCACGGCTGCTGCG

CCGACCGCAGCCGCCATGAAAGACCATCTCCGGGATTATGACCTGACTTCTTTCGGGCCC

ATTCCCGCAACACTGAAGCTCGCGCCGGAATTGCTGGACCATGACGGCCACCCGCTCGAC

GAGGAAGGACGCTTGGCCACGACGCATGGGAAGAGGATGCGCTTCGACAGCGTTGGAGGG

CTCGACGTGGCGCTGTCCCCAGGCGCCTTGGCCTCGGGTGAAGGGGCGGACACTTTTATC

GTCGAATTGAAGGAAGGCCTCATGTCGGGGTCGATCGACCTTCATCGCAATAACTTTTGG

TCAGATGCGCATGGAGATCACTCGTCCCGCGCTGCCGGCGCGGTGCGGACCAGGGAGTGG

AAGAGGGCTGCTTGGGTGGTGCACGGGCTGAGCAACGCGGATGGGGAAGGCCCCGCCCCG

GGAGACGTCTGCTCCTGGGGCGGCCTCAGAGTTCATTACGCGGGCAGTGACGTTCTTTAT

ATCACAGGTATGGACCACCTCCTGCACCAGGGCAGAGGCGCTGGAGGCAGCACAGGGGCG

GAGTCAGACGAGCTCCAGATGGCGTGTTTTATGGGCCTCGTGTCGTATTTGGCCGGGCTT

CCGGAAGTGCAGCGAGTCTCTCCGTTCCACGAATCGCGGCTGCTGAATGCCGTCGCGGGA

GCCATCGTGCAGAGCGGCAACATCGTCGACAGGCCGCTAACTGACGCTGGCTTGGACGGC

ACGGGCGAGGTTATtcagATCGTTGACTCGGGCTTAGACGAGACCTCTTGCTTCTTCGAA

GACGAGAGTGGAGAGGAGGTGGAACACGGCTACTACTTCAATGACATCGAAGTGGTCGGA

GTGGAATCGTCTGCACTTAGTACTGATTTTAGAGGAGGCTCTTTCCCCATTGATCTCACC

CGCCGAAAGATCGTCCAGTACACCCGATTGGTGAGCGCAACGAGCACGGTAACACAGTCC

AGCAGTGTCGGCGGTCAGGCCTTCACGGAATCCGAATATTATTATTTCAGCGGCTTCGGC

CAAGACATGTTAGACGGACACGGGACGCACACGGCCGGCTCTGCGGCGGGGGCCGTGCTG

AACAGCCCCGCCGAAACGGACACCTGCTCCAGTGACGAAAACCTCGGGTGCATCGGAACG

TGCTTGAGCGCGTTAGAGGAAGCAAGCCTTCTGCCTGACGGTACAGCGATGTGGGACACG

CTGTGCCCGCAGTTCGGTTGCGACGCTACTGCTGGAGACACGTGCCTCGGGGCGGACGTG

GGCGAGACTCTCACGGAGAACGGGGGCGTTGCGCAGGGAGCGAAGATCTCGGTCTTCGAT

GCCTCCGTCGACGGGCTCCTGGTGTGGGCGTCCCTGGCGCTGAACGGGCTGTGGGAGTCG

ACGGACGGCACGGATAGCTTTGTGCACTCGAACTCGTGGGGTAGCGACAACGACTGCAAC

GTCGACTCCCAGAGCGTGGCGTACGACGAGTACATGTACGAGAACCCAGAGCACCTTCTG

CTGTTTGCCGCGGGCAACATGGGCCTACCCGACGACCCCGATCGCAAGACGTGCACCATC

AACAACCCCGGCGGAGGGAAGAATGTTCTCACGGTCGGCGCGTCCTCGTCCGGCCCTACG

CGCATCTCCTTCTCCAACGACGACGGCGGCCAAAGGTACTCAAGCAGCGAGCCCGGTGGT

ATCGACGTCGTCGCTTTTTTCAGCTCCTACGGCTTGATGAGGGACGGTCGCATCAAGCCC

GACGTGGTGGCCCCCGGCGATCAGGTACTCTCCGCGGGCAGCGACGGATCAGACGGCCAC

AGCTGCCAGCTGTCAGCCCAGTCGGGGACTTCCATGTCGACGCCTCTCGTTGCAGGCTCT

GCAGCTTTGATTCGGCAGTACTTCAAGAATGAGAGCTTCTACGCAGCAGACGTGAACTCG

AGGGGGCTCTGCGGTGAAATCTTCAACGCGACCAACACCTTCGCGTGTGAAGCGTTCTCT

CCGTCCTCAGCAACGCTCAAGGCGATGTTTGTCAACAGTGCTGACCTCATGGGCGAGAGC

TCGGAGCCAGACGGAGTCCGCGGCTTCGGGCGGGTCCACCTGGAAGCAGGCATGCCCTTG

GGCGGTCAGGACAACCAGGCTCTCTTCGTTGCGGATGCCTCCGTGACCACTCTGGACGAA

TACACCATCGACGAGTACCACTTTGAGATGGCGTCGGGTGCGGAGCTAGAGTTGCGGGCG

ACGCTGGCCTGGATAGACCCTCCCGCTTCGGCCGAGTCTTCCACCCAACTCATCAACGAC

CTCGACCTCACCCTCGTGGGCCCCGACGGTACCTTGTACAGAATGTTCAGTGACGGGGCG

GACGACAGGAACGTGATCGAGCGCGTCATCGTGCCCGCGGACACCGTCTCCGGAGGCAGC

GGGAATTGGACGGTCGCCGTGTCGAGCTTCGGCCTCACTACGGGCACCCAGGACTACTCA

CTCGTGGTGACGGGGCCGATCGACGAAGGGTCGGGCGCGAAGACGACGCGCGAAACTAGC

GGGAGTGCGGGCGGGAAGGGGCCCGGTGTCGCCCTGCTTTTCTCGGCCGCGGTTGCTGTC

GCGCTGTTTGTGGCGCAAATGGTGTAA

>g17981.t1

ATGAGCATCCTGGCATTGGGCGTCGTTCTGCTGCTAGACCAAGCGACGAATGTGGAAGCT

TGCTCGTGTTTCATTAACTCGGGGGGGCTGTGCGACGATGTGGACGCTTCAGGTGTTGTG

CTGCATGCAACCGCGCTTTCCAGGTCGCAGCAAGTGGACATCAACGACGATGTGACGTAC

ACAGTGGCAGTAAACACCCTCTACAAGGTGGAACCCGACGTGACGTACGGCCAGCAGCTG

GACATCGTCACTGGTGGGAACAGCGCCAGCTGCGGGGTGACACTGGAGCTTGGCCAAGAA

TACCTTCTCGGTCTACACCGGAGTGGAGCCAACATTTTCAAACCGGAACAGGAGGGTCAG

CTAAGGGTTGGTCTCTGCGACCTGGTGCGGCTCTGGAGCTCAGTCACCGATGAAGAGGTG

GCGCTTCTAGAAGCCGGCTGCGGCGACGACCCATGCGATGGAAGATGCAGCGAGTTTCAG

GAGTGTCTGTTCTACTCTGACAGTTCCACGGCGGAGTACTACTGCGCCGACGTGTGCGAC

CCGAGCCCATGCGATGAAGGAGAGATATGTATCTTGGAGGATGTGGACTGTTTTCGGGCC

CCCTGCCCGCCCCTTGCTGAGTGTACCGGTTCCGGCGATTGA

>g18050.t1

ATGGACCACAAACGCGCCAAGACGTCACCGAAGGAGCCCATTGTGTGCGACAATTACATT

GGAGGAGAGTTTACGCCGCCTTTGTCTGGGTGCCATGAGGATGTCGTGTCCCCCTCAGAC

GGGACCGTGCtcgccagAGTTGCGATGGATGATGGGcaaggggtggcggtggcggcggca

cgcGCGAAGGAGGCGTTTGCGTCCTGGAGCGGCATGACGGTGAAGAGCCGCGCGGCCGTG

ATGTTCCGCTTCCACGCCCTCCTGGAGAAGCACGCGGACGAGCTGGCGGACATCGTGGTC

GCCGAGAACGGCAAGAACAAGGCGGAGGCGTTAGCGTCAGTGGCGAAGGGGAACGAAACC

GTCGAGTGGGCATGCTCCATGCCCCAACTCATGCAGGGAAAAATCTTGCAAGTCAGCAGG

GGCATCGAGTGCCGCGATCTGAGGGAGCCCCTGGGTGTCGTTGCCTGCTGCGTGCCCTTC

AATTTCCCGATCATGGTGGTGAGGAGGCCtgtgggGTGGACGGTGCCGATCGCCCTCACA

GCCGGCAACTGCGTCATCGTCAAGCCCTCGGAGAAGGTCCCGCTCACCATGCGCAGGGTC

GCTCAGCTGCTCACAGAGGCAGGAGTGCCTCCTGGGGTTTTCCAGATCATAAATGGCGGA

AGGTTAGCGGTGGAGGCCTTGTGCGACTGCGAAGACATTAAGGCCTTGACTTTCGTCGGG

AGCAGCGGCGTCGCCAAAGCAGTCGCTACGCGCTGCCACGCTGTGAACAAGCGGGTGCTG

GCGCTGGGAGGGGCGGAGAACCACCTCGTGGCTCTGCCGGACTGCGACTACGAAGCGGCC

TCGCACGACATCGTGGCGTCCTTCGCGGGGTGCGCGGGGCAGCGATGCATGGcagCGAGC

GTGCTGGTGCTGGTCGGCGACACCGGAGACCTCCTGGAAAGGGTCTGTGCGAAATCTCGG

GCGCTGGCAGCCGGCACCGGAGCGTTGTGCCTGCCCAATTTGTGA

>g18089.t1

ATGAACCGAGGAATCAATTTGGAATCGGCGGCGAGTGGACAGTCCTCTGGCCTCAAAGCC

GAGGTGCACCAGCTCAGCGGCATCGGAAGGTTCATGAAGGAGACGTACACGCCGTTCCTA

CTGTCCACCAAGGGCAAGGCTTTGGTGTTGCTGGGGTCTGCCGCCCTCTTTGCTGCCGGC

GTGTACGGCGTTACCCAGGCGACCCAAGGGTTTGACGTGCTCGACCTTGCGCCAGACGAC

CACTACGCGCGCGATTACACGGAGTTGGCGCGCGTGTACGAGCTGGATATCGATGCCCAG

TTCATCCCGCTCAACATCTACACCCTCACTGTGGACTACCCCGATGTCGAAGTGCAGgcg

CAGATCCAAGCTACGGACACCTTGATGGAAGAGCAGCGACATGTCGCCGGGCCGATCGAC

TCGTGGATTTCCTCTTTCGTCGGCTGGGCCTCCAACACCACCGAGTACAGTGCTAACGTG

GGCACCTCTGGGGGGTACTCCGTGTACGAAGACCGAGAAACGTTCTACCCGGCCCTGACT

GCTTTCACGGAGGATGCGGACAATGTGCGCTTCCTGTCCGACATCAGATACAACGACCAG

GGAGAAATTGAGATAAGCCGGTCGGGTCTGTACCTCGTCGACATGGTGGACACGGACACC

AGCGTCGACGCGCTCGAAGACACTCGAGACGTCATCGAGCTGTCCACCCTCGACCCCGAG

CCATTCGGGTTCTCGGGCACGTTCGTCTTCACCGAGCAGTTCCTGGTCATCTACGAGGAG

CTGCTCGTCAACTTTGCTCTGGCCCTTGTGGCCGTGGCCGTGCTGAGCGTGCTGATCCTG

GGGAGGATCGGCATCGTCGCCCTGGTCTGCTTCACCGTGGTGATAATCGACGTGGAGCTG

CTTGGGTTCGTTTATCACTGGGGGCTGGACGTCAACAGCATCACTGTTATCGAGCTGATC

ATGGCCGTCGGGCTGGTGGTGGACTACATGGTGCACATCGTGCACTACTTCCTACACCAG

GACCCCAACACCCCTAAGGATGCGCGTATAGCGGACGCTCTGGGGGAGATCGGTCCCTCC

GTGATGGTGGGCGCGGCGACAACATTTTTGGGCATCATGCCGCTCGCTTTCGCTAGCAAC

GTGGTCTTCAGGGTGTTTTTCAAGATgttcctcatcatcatcaccttcGGGTTCTTCCAC

GGCGTGGTTTTCGTGCCAGTAGCGTTGTCGCTTATGCCCGACGGCCTGGCGCGGTCTatt

gagagggaggaggggcacGGACAGAATTcgggcggcggcaacagcagcagcaagATAAGC

CGGTCGGGTCTGTACCTCGTCGACATGGTGGACACGGACACCAGCGTCGACGCGCTCGAA

GACACTCGAGACGTCATCGAGCTGTCCACCCTCGACCCCGAGCCATTCGGGTTCTCGGGC

ACGTTCGTCTTCACCGAGCAGTTCCTGGTCATCTACGAGGAGCTGCTCGTCAACTTTGCT

CTGGCCCTTGTGGCCGTGGCCGTGCTGAGCGTGCTGATCCTGGGGAGGATCGGCATCGTC

GCCCTGGTCTGCTTCACCGTGGTGATAATCGACGTGGAGCTGCTTGGGTTCGTTTATCAC

TGGGGGCTGGACGTCAACAGCATCACTGTTATCGAGCTGATCATGGCCGTCGGGCTGGTG

GTGGACTACATGGTGCACATCGTGCACTACTTCCTACACCAGGACCCCAACACCCCTAAG

GATGCGCGTATAGCGGACGCTCTGGGGGAGATCGGTCCCTCCGTGATGGTGGGCGCGGCG

ACAACATTTTTGGGCATCATGCCGCTCGCTTTCGCTAGCAACGTGGTCTTCAGGGTGTTT

TTCAAGATgttcctcatcatcatcaccttcGGGTTCTTCCACGGCGTGGTTTTCGTGCCA

GTAGCGTTGTCGCTTATGCCCGACGGCCTGGCGCGGTCTattgagagggaggaggggcac

GGACAGAATTcgggcggcggcaacagcagcagcaaggtgcGTCGTCGAGTTGTTGGGCCG

TTTTGA

>g18143.t1

ATGATCCAGCTGTTGCTCGCCCTGGCTCTTCCAGTCATCTGCTCCAGTGCCCTCGTGGAC

GCGAACACCAAGACAGCTATCTCTACGTGGGCGTGGGCTCACGACGAAAAAACGCTGGAC

GCTGGGGGCTGCGACCCGGACGGGTGCACCTCCGAACTGACCCTTGACTCCGACACCCAC

CCTTCATCGCGGTGGTCGTGCAAGTACGACCTCGCAAACAAGCCGTGCAGGCTCTTCTAC

AGCTTCGACAGCCCCCAGGATATCGTGACGCTCGATCTGGCGTTCTTCAAGGGGGACGAG

CGCACACGGACCTTCTTCGTCAAGACCTACAACGCCCACCGGGACTACCCCGGCACGTCT

GCCGTGTTCACCTCGAGCGGGAGCACGCTGGGGTACGAGAGCTTCGAGCTCAACACCAAC

CAGACCTGGTACATGTACCTGGCGCCTTCGACCGCGGGAGACAACGACAAGGATTGGATC

AGCATCACCGAGACGCGGTTCATGGTCGAGGAGTAA

>g18241.t1

ATGCGGCTGCCTAAACACCGCAGGTCGCGATCTTGGAGCAAAGATCGGGCTCGCAATTTC

GGTCGCCGCTCTCGGAAATTGACCGTCTGCGCGTTCGCCGGCGGCGCCCTGTTGACCATC

TTCGTGTGGACGCAGCTGCAGTCTCACGGTGTGCCGGGCCCGTGGGCGAGATCGGATGAG

CCCAAGAGTGCGGGTGATGTGGAGAGGAGGCGAGCAGGCGTGGCCCCCCAGGGAGACAGG

GTAGGGGGGATCCCTCCTAGCGGTGATGCGATCGAAGGGGTAACAGGTGGGGGTCAGTGG

GAGGGTGTGGGATCTGCGGGTCCATTTAGCGGGGAGGGTTTGGGAGTTGGAACGAAGacc

ggaagggggtgggggcggggcggggcggcgggggggccaGGGATCGCGATCGGTGCAGGC

GGGCACTTCATTTTGGGGCTGTCGACGGAGGCGTTTGGCTCAACTCCTGTGGACGAgATG

ATGGCGCAGTACGGCACGGAAGAGTGCGACTCTTCTTTCGGGAACGGTTTGGTGGACACC

TGGCGGGGAACGGGTACGGACTGCTGTGCTGACGAAGGAAGGGGAGGTTCCGATCCATCC

TCCATCCGCTGCCATTTGATCCAGCAGGATCACCACCACGGAAGCGGAGATAACCTGGTG

CAGATGCGTAACGTGCAGCTGGACCTGAGCGACTTCGAGGATCCAGGCGTTGCCCAGCGG

GTGATGCAGGCGTACAAGAACTCCAAGCACGAGAAGCAAGCCTATGTCCAGCTAAGACCG

GGCTCCGTGAAGGGGACGTGCGCCAAGCAGCGTGGCTCGTGGAAGGAGGAGCACTTTCCA

GGTTGGACCGCTGACTGGACAACGAAGGTTTTCCAGAATGTGGAGGCCTTGGAGTGCGAC

GAGTGGGTCGACCATCCCGTCATGGTGATGCAGAGAGACACCTTCGCAAATCTGTTCCAC

GACTCGGAAGATTTCGTGAACGCGTTTTTGGCGATGGCGATCCTGAGACAACGCCCGAAG

GATGTTCAGGTGTTTCTGACCGACCTCTACCCGCAGGGACCATTTTGGTCAATGTGGGAT

AAGGTGTTCGGCGCGGGGCATCCAACGCTTACATCGTGGGAGCTGGGCAAGCGATACGGA

GGGAAGAAGGTGTGTTTCCGTGACTTGACCGTCGGTATCTACGGCCCCGCGGCACCGACA

ACTCTGGCCAGGATGGTCACCCCCTGCTTCCATACCGCGCTCGTCAGAGCCTACTCGGAC

TTCGTCATTAGaggactTGGTCTCCAAGGCTTCACGCGCTACGCATCTCCGCCTTCGAAA

AAGGTTGTTGTCACCTGGGTGGCGCGAAGGTCCTCCGTACAGTGGCCGGAGCGGGCGTTC

TGCAGCGAGGATGGGAGGGACTCGTTCTTCACGTGCGAGTACTTCTCGCACTTAGACACA

AGGCAGCTGCAACGCCGGGTCAAGAACGAAGAAGAGGTGGTGCGAAGCCTCAAGACCCTA

GAAGGACAAACGTTTGGAAACGGAGCTGTCGTGGAGGTGCGCGACATGGACTACAACCTG

CTCAGCTTCGAGGACCAGATCAAGAACGACTTAGACACGGACATAATGATCGGCCCGCAC

GGAGCAGGCCTCTTCCACGTCATATTCACGCCCGACAGAGCAGCCCTCATCGAGCTCCAG

ATAGACCACACCACGGAGAGAAAACACTTCAACAACCTCGCGAGATGGTCCGGGCACGCC

TACGTTTCGCGCGGCGGCCCGAACCCCGTAAACACGGACGACGCAACGGCTATGGTGCGA

AAAGCCGTTACCGATATGGATCTGTCCAACCATTGA

>g18455.t1

ATGGTCGGCCATGGCCGAGGCCGAGTTCAGCCATGGCCGATCATGGCTGCGGTCATGGCT

GCGGTCATGGCTGCGGTCATGGCTGCTGTCATGGCTGCGGTCATGGCTGCGGTTATGACC

ACCCATGGTTTGTGCAATAACTTGTCGTTCATGGCCGCGCTCATGGACGCGCCGCTTGTA

TTCCTAGGGATCGCAGGACTACCTACCTCCTGGTGGGATGTGCTCGTCTTGGACCTCGGT

TTGGGTCGCCTGGTCTACCAAGGCATAATCGCGACCTTCGTCCGCATTTGGGGGGGTATT

CTGGCGCTCGTTCGGCCCACTATCCCCGGAACTGCACCCAACTGGCTTCGCGAAAATTGC

CCTATAGTGGGGTGGTTCTTCTTCTCTGATGATCCCTCGGTTACTGTGGTGAACCATTGC

TGTGCGCCGGAAAACTCTGATTTTTTCCTGTACGTGAACGGAATGTGTAGCACCAAGCTT

CTGGCTCTCGACACGGGCAGGGTGCTGGCAGACATGTTTCACCGGGATATCACGGTGGTA

CACAACCCCACGGATTCTGTCATGGTGGACCTATTCGAGTGTGTCATCGGGAAGCTGTGG

ACGGGGAAAAGCTGGTCGACGATCGTCCCCTGTACTTTGGCGCTGAACGAGATCATAGCT

GCTCTGAAAAACCCAAACAAGACCAAGGTTGTTCTGCTCTCGCACTCACAGGGAACCATC

ATCGCTAGCGACCTCCTGTGCTACCTGTGGAATGCTGTGGAGTGCGGCGAAATTCCCGAG

GACGCAATGAAGAAACTGGAAATGTACAGCTTCGCAAATGCAGCGCAAACGATGGTACAG

AAGAATGGAGTCCCCTACATGGAGTCGATCTGCAATCATCGTGACAACGTGGGCATGCTT

GGCGCCAACGCCCCTGACCTGGTGAAAAAATCATGGAACATCCACCTCGGAGGCAAGGTG

ATTTACCCCAACAAGATTAGGTGGGGGCATATGATCTCGTCGCACTACCTCAAGCACCTC

AAGGCTGGAGACTACATCGAATCTAAGCTGCACGTGTACATGAACAAGGACCGGGACACG

TCGGCGGGCGCCGCGAGGTAG

>g18690.t1

ATGTTCGTCAGCGTAATTACCCCCCGGCCCTTGCATGTTCGGCTATCCGACCTCCTCGCC

TCCGCAGATGTCGGGTACATCGTGTGGGACACTGTCGATTCGCGGAACAATCCCACGGCA

AGTGTCGAGCTCAAGCAAAGTCCCTTCAAGTTtcctgatgtggccgtctgcaCGGACACC

AACAGCGGGTGTACGTTTGAGAGCGGAAACTGCCTTCAGGGCGaggattttgttttggtg

cgACAGTCGGGCTTCATGGAAGACGACGACGATTTTGATCTGGCGGACTATCCGGACGTG

GCGAGAGTCAGGGAGTCGTTCCCCCATTGCGCCTTGATGCCGCTATCACAGCTGACTGTA

AACGAGACAGGGGTGAACAACGGCGATATCGAGAGTTTCAGCGCGGACATGTTTCTTCTT

TGGTTTGATGACCCGGACGGGGTTCTCGAAACAGGGGACTACAGCATCTTCAAACAGTAC

GTTAGTGTTCATTTCATTGACATCGCGCAAGATGTGGACGATATTGAAGGGACCGTCACG

AGTGCCAAGATTCCCTACGAGCGGATCACCCTCACAAATGGGGCGTCGTTCACTGCAACG

AGCAGTCATATGGTGATAAGCTTGTCAGAGTTCTCCGGGATCACGAAGAACGGCGCCAGA

CAGGAGCGTGAGCGGACTTATTCGCAGACCACAACAACCGGAAAGGAACAATGGTTCTGG

ACGGACGAATCGTTCCCGACTCCGATGGCTTTCCTGCAGCTTGAGGTCGCCATCAGCAAG

TTCGAGTTCCTCAGAGTCAAGGAGGTCGATCCTGTCGACGTGTGGGCGATTATCGGCGCC

ATTGGGGGCGTTTGGCagttcgTCGTGGTGGCGTTCggggtgttcttcatatttTCGGAG

AAACAGCCACCGGACAAGAAGATGCGCGACTTCAACGACACGTTTGTCACGCCAGCCGCC

AAGATCAACAAACGCTTgtccagcatcaccaccacctcatTAAGGCCATCACAACAGGAC

ATTGAAATTGACGCAGGAGACGAGGACCTGCCGCCCGGGTGGGTAAAGAAGCAGCGCAAA

TCCGGTAGCGTGTACTACTTCAACACGATGACAGGCTCCACAAGGGCCACCCCCCCCAAC

GAAAGCGGAGAACCAACCGAAAGCGCGCCCGCTGCACCCCGACCTTCGAACGGAGCCCGC

ATGCTGTTCCGgGTGGCACAAGACCCCCTAGACTCGGGGACCGGCGGTGTGCAAAGCTCT

ACAAACGGAACGACTGATGGCGGGAGCCTGCCGCCGGGCTGGCAACAGAGGACAAACGAG

GACGGCAGAAGCTATTATGTGGACACGGAGAACCGAACGACGCAGTGGAATCCGCCTTCG

TGGCGGGTTAGTTCCAGCAGCGGCAAGGTTCAGCCCGTCGACGCTCCCTTGAGGCGCGTA

CGCCGGGGCTCGGCCACGGACATACCTCAGCCTACCGCGCCTCCCACCATCATTCCTCCG

ATCACCACGTCCGCAACGGGGGGCATCGTTAACGAGCCCCTGCCTCCGAACTGGGAAACT

CGCACCACTGGAAATGGGAAGACATACTACGCCAACACCGTTACAAAGAAGACCCAGGCG

TCGGAGGCGCTGCCTCCGCCGGGGCGTCGTCTCCCTCGTCCGCTGTTGCACCCGCTCCAG

ACGCTCGAGTTCTCCGGTTGGGCCGACGCTAGCGTTTTCGGCCGTGGGACGAGTCCTGTC

GCGGCTGCGCTGTCGCGCTCGGAGAGGTGTTAA

>g18733.t1

ATGGGAAAGCCGGATATCATGTCGGACAAGAAGGACAAGAGGGTGGTACCAAGCGAACCC

GAGACGGATTCGGGGGCGGAGGAGTCAGAGACGGAAACGGAGTCGGAGGGGGGTGATGAT

GAAGAGGATACGGGTTTAACAGAAAACCAGAACCGTCTCCTGTATATGGTGTCGTTGTAC

ACGCACAAGGCTGACACGCGCGGCGACAGAGATCACTGGCTGCGAAAGCCAGCCCTGGTA

GTGCTGCTATACGAAGGCGTCGTCGCCAACGTCCTTGATTTCGACTACGCTCCCCAGTCT

GAGCTCATCGAAAACCGCCGAATATGGATGAACGTCAGTCAGGAGGGGAAGTCGGATATC

GAATTCCTTCGAGAGGAGGAACTCGTCAGTGGCCTACACGTTTCGTCGAGAAGCTACAAG

CCCAACACCTGCTACCAGATATCTCCGAAGGGGAAGGAGCTGGTGAAGCGCATCACGCGA

AAGGAGAAGGAAGCCGTGCACGAGTTTGTGTACGCACGAGGGACTCGCGAACTACTCCAC

GTTCGGTGGGACGGAAACGAATACTGGCTGGAGAGTGCATCAGGCTACCGACGGAAGTCG

ACGATAACAGAGACAGAGGACGTTTCGTACGTGTCGTCGGCGTACGTGCCGCAGTGCCTA

CGGTACGGGGGGCGGCCCACGCTGTCAAACGCACATCGCGCGCACGAATCTGGTGTCAAC

ACGGACAACATCAGAGACGAGTTGGATGAGGTCATCACCCTCAACTCTGTGTCGATTATC

GTGGCCGAGTACATACCTTTCGGGGcgaaccagATCGTGAACCTCAACAACAGCGTGGGC

TCGACGGAGAGATGCCAGGGAGGGTTTATTTCCCCTGCCATCGATGACGACTCCACCGGA

ACAGACCTGGAGATGTCGCCCGAGCTCACGAGCGTCGACATTCTTGATTACACGCTCACG

AACCACATCAACTTTGAGGCGGAAATTAACTTCGCGGAGGAGAGCGGAGTGGTCCAAGTG

GAAACTTTCGGTGTCAGCCTGAACGCCGAGGGCACTTGCTTCTACGGCATGCAGGTGGAA

GCGGTTATGGACAGGATCAAGGACAACATCTCCTTGGATCACATGGCTCGATTACTCGTC

GACGTGCAGCAAGATTCGTCCGGCATAGTCGACTCTATCATCTCGCAGCACCAGCGGGAG

CTCCTGAATCTGATTTTCCTCGGCGACGCCCCTAACCGGAACAagGTGAACCTGATAATG

GCGAACGAGATAACGCCCCACTTGACGGCGGAGGAGTACATGGACAAGGGCGAGTACGAG

AACGAACTGAAGCAGGTTATCGGGGATACAAAGGCGGCGTACGACATCAGCGAGCACGAC

ACGCTCATTTTTGGAGGGCATGGGCTGCTTGTCGCGGGACCTAACTCTCGCCACCACGAG

CCGCTGCTTTGTGCGTACCTGCAGTTCATCACGATCGACATTTTTTTACAGAACTTCTTC

TCCCGGTTGTGGATTCTCACCGACGACATGAAGGTGACTTACGACATAATCGTTACATCC

GACACCGACCCGACGGCGCTGGATCGCATCAGGTATCGAATCTGCGCAATGTCCAACGAC

GTCATCGTGCTGGAAGAGATATTAGGCTACGTATTGGAGGCTCTCGACGTTATTGAGGTG

CCGCCGGAGCCACCAGAGCAGGCGGGACGATCGCTGTACGAACGGCTGGAGATATCCGGC

ATGCGCTCCCAGCTGTTGAGAAGGTCCGCCGACCTCAAGAAAAATGTGTGCGGATCTCAG

AAGTATTTGGACGTGCTCCGTGAGATGAGTGCTGTGGTGAGCGAGAACAAGATGTTCCGG

CTGAACGAGAGCGTGGAGATGAACACCAAGAAGCTGTGCAACCTGAACGAGAGCAGCGAC

AAGTCGGCAAAGTCTCTCCAGATCATGCTGGTCATGTTTGCGGGAATGCTGGCGTTCGAC

GTCCTGGACCGTTTGACAGGGGACTGGTCGGTCACCAACTCCGACTGGTTTCAGGGTTTT

TACGCGGCGGTCATTCAGAGCACGCCAATTTTGTGGTTCCTCATCTCCCTCTTGGTTTGG

TTCTTGGTGGCTTTGCTGGTTTGGAGAATATTCCAACGAATGCAGCACAGGGCTCAGGGG

CTCACGACGATTCGTACGCGATTCTTCCGAAAGGTGTTCCAAGATAAGCTACAGACGTGG

ATGGCGTCTAAGGCTATATCACTCGAAGAGCGGAACTTCGACCTCAGTAACgatctgGTA

CGGATATCATACGAGGAACGTCACCCGGGAGAGTGGGGGGGTTACAAGCcgaaggtggtg

gtggagtaTGACGAGAGAAACAATTACCTGATGGCTATCGAGGTGACCTACAACCGGCGA

GAGGCGGATAAGAACGTGGCGTACAACTACAGAGAACTCAAGGACAAGATACAGAACGAG

CTTGATTCTGCGCAGgTGTGGGATCGATCTGGCGAAGATCACTCATCGCAGGACCTTGCA

GCGGACAAGCGAGCGGCGATCGAGCGACGTCTAAACGAGGAAGACGAAGACGAGCAAGAC

GAGAAATAA

>g18734.t1

ATGGTGGACCGCTTTGTACAATCGTTATCCCTTCTTATCCTGCTATCGGTCAGCGGGCTG

AATGGAGGGCTCTCCACAAAGATGAGCTTCACGGTGGCGGCAGGAGGGGCAGCTGGGATT

GCTGGGGATGCAGCTATCGAGCAGGGTGGAAGCGCGTGGCGCGCGTCGTACTTGCCGCTG

ATGCTGGCAAGGCCTGGGCTTCGATGTTCTGGGACGTGCGCAGAAGACACGGGCGATCCG

AACCGACAGGAGGGCGGTGCGCACCACTTGAAATTTAGCTCGAAGCGTGCCCGAGGAGAT

GGGGATGTGGgagtcgacggcggcggcggcggcggcggccatggCACAAGGAGCAAGAGG

AGCACGTCACACTCGGAGACGGCCCTCTGGTCGGCTGCACGAATGCGAGGTGGAGAGGTC

GATCGGAATCCTGACGTGTCCCAGGAACTCGCCGAGTCAGCCAAGAGCCCTCGAAGCCCC

CACGATATCGCGAAGGCGGAGGCTATTTCTTCCTCGCCGTCTCCGTGGCCGCACGCCGGC

GTGTCAAAGGGGCCACGGTCGTTCAGGAGGTATAGCGACGCCGAGGTCGTGATCGCGCCG

AACACCGACTTCGATGTGGAGGGAGGCTCGCCCGCGACGGCAGCGTCGAAAGCTCGGGAC

ACGACAACGACGCACTTTGTGTCGGAGTGCTCTTTGCCGACAGCCCGAGGGAACTTTCGG

CTCAGGGCGTACCGCCACGAAGGACACGGACGCTCACTCGAGCCGGTGGTCATGGTGGCG

GAAGGCCATACGGACTTGGAAGGCGTGCCCGTCCGCGTGCACGACCAGTGCCTCACGTCG

GAGGTGTTGGGATCGCTTCGATGTGACTGCAAGCAGCAGCTCGAGCTCGCGCTGGACTAC

ATCGCCGAACACGGAGGATGTGTGATCTACATGCAGCAGGAGGGGAGAGGCATCGGGCTG

GCCAATAAAGTGGCGGCTTACACGCTTCAGGACGGCGGCTTGGATACCGTGGACGCCAAT

CGACACCTGGGATTCGACGATGACCTCCGGTCCTACGAAGCCGTGGAGTACATCCTCTCC

GACATGGGTATTAAGAGCGTGAAGTTGATGACAAACAACCCGTTCAAGCTCAAGTGCCTC

AAGGCCATGGGGATCAAGATTCTCAGCCGCATACCCATGCTGGTGGCCCCCAACGCGCAC

AGCCTGGCCTACCTTCGGGCCAAGGCGCACCGCATGAGCCACTTCCTCCACCAGTTGGAC

GACGTTGAGATTATGGGCGAGCCTCCGTTGAATGGTGCTGCGGCGCCGGTGGCCCTCCCT

CTTGATGAGGTTGGGGCGATCAGGGGTGGGGAAAAAATCCCCGATACCGAGGTGGCGCGC

GAAGAAGAGGTGGAGGGCGAGGGGGACGAGGACGAAGAGTACGCCTTCGGGAAGGACTCT

GTCCTGGCGGCCATTCAGGCCGTGCGAGAGGGGAAATGCGTCGTCGTGACGGACGACGAA

GGGAGGGAGAACGAGGGCGATCTTGTCTTCGCGGCGGAAAAGGCCACGGAAGACCTGCTG

GCATTCACAATCCGCCATTCGAGCGGAGTAATTTGCGCTTCGATGGAAGAGGAGCGTCTT

GAGGAGTTACGGTTACCGCAAATGGTGGAAGACAACGAGGACCCGAAGATGACAGCTTTC

ACCGTCTCTGTCGACGCTAAGCTAGGCACCACCACGGGTATCAGCGCGGGCGACCGCGCC

ATCACGTTGCGCTCCCTAGCGGATTCAAAGGCTAGTCCGCACGACTTCAACCGGCCTGGC

CACATATTCCCGCTACGATACACGGCTGGAGGCGTGCTTAAAAGAGGGGGGCATACAGAA

GCGGCTGTTGACCTCGCCAAATTGGCGGGATTATCACCGGTGGGGGTTCTTTGCGAGATT

ACCACTAAGGACGGGAAGCACATGGCAAGGGTGCCGGAACTTCGAGAGTTCTGCACGGAG

CACGGACTGGTCCTGACCTCCATCCAGGACCTTCGTTGCTTGATACGGGAAAGACAGCGG

GCAGAAACGCTCGGCCGCAACGGCCCTGCCTCGCGTGTGCTCACACGGTAG

>g18910.t1

ATGACCGCGGAACGGATGCCAGGCGGTGGTGATCCTGCCGGGGCGGCGTTCGACACGTCG

TGGCTCCACGAGGCCTCTGACCCCTCCGGCGGTGATCGTGTTCGCGGCCGCAGCGGCAAA

CGCCGCACGCGTCGATGTACCCTAAGTCCGTCGATAGGCAGCAAGAGCTTCATGTCCGGT

GTCGATCTCGACGACCCCGAAAACTTCCCAAATATCCCTGTTGCTGGCGGTGTCACCACG

AACGGCGGCAGCATGAGCGTGGGAAACATTGCAGCAGAGGACGGAAGCGAGGCGCCCGCT

ATCTCTCCCACGCGACAAACTCTCTCCCCGAGTCCTGGCAGCGATGCACGAGACTCCCGT

CTGCCCGACCcgagcgtcgttgccaacctgTTCGATGACGAGGAAACGACACCGGAGCCG

AAGCTCCCGCCACCGCTCGGGTTACTGGGCTCCCTCGGGAACCAGAACCAGGCACGCTCG

ATGGCGACGGAGAGGGACCGCTCCGGTAAGAAGAGTCAAGGACAGGGTTCTGCAAAAGGC

GTGAAAGGCAGCAGCCGTGATGCCCACACCTCGGTGGGGGTGGAGAACCGTCAACCCGGC

CTGGACCTCAGGCCTCGTGTTGCCGCGGAGGGGGCTGCAACGGCCACGGTGGTCACGAGT

AGCCCCCCGTCGGTGGTGTCGACGAGCGCAGCAAGCAGGGCGCCCAGCAGCACCGCGGCG

ACGAAGATGCgccttgctactactactactacaactactacgacCGTCACACCTGGCGGT

GGTAAGGGGGAGGAAAAGAGGCCGCTGCCATCGCCACCGGCATCGGCGTCGGCCGCGGCA

CCGGCACCTGAACCAGcaccggctgcggctgcggctgcggctgcggctgcggctgcggct

gcggcaccGCCGCCGATCGCGGCTAAGCAGAAGAAGTCCCGCAACTCTCGACGTAGGTCT

ATCCTGATGCCCGCCGAGGCGCAGTCTTTCGTGGAGAGTGATCTTGGCGGCAGCGGTGTT

GAGGTCGCACCGCCGCAGGGGCAAAGGTTCCCCGGCGGGGACGCcggcgctcctcctcct

ccaacagcagcagcagcagcaacgacgacgacagacCGCGAGGGAAAGCCTAAGCCgagc

agcggcaggagcggTGGCGGCAAGACAGCGGCGGAGGAGTTTCCGCTGACCTCGGTCAAC

GCCACGTCCTGCCTGTCGCGCCCGCTGGCGACGCAGCGGGGCTGGCGCTCGGGCACCGGG

GACGAGACGAAGTCGCTGGTTCGAGCGTACTACGCGGCCGCTAAGGGGTCGCAGCGGGCG

AGGCGCGTCGCGGCGCGCCTGTTCTGCCTGACGGGCTTCTGCCTCCTGCCTGTGAGCCCT

GACGATGCTCGGGTCTTGTGCGAGGCCGCCGGGGATAGCTTGGAGTGGGGGCTGGAGGAG

ACGGAAAAGCCGCGGTTGGTGACCCGGCCCTGCGTCCGCGAGACCAGGGATCAGAAGCGG

GAGCTGGTTCGCCGCATCAAGCCGCTGTTCGAGCTGATGGATAAGGCCACCCGGGACGAG

AAACGCGCGGCGGAGGCAAATACCGGAGTGGTCTCGAAGAAGGCGGCTGCGGACGGCTAC

TGCTACGAGGACCGCAAGACGAAAGCGCCGGTCGACCCCGACGCGTACAAGGCTATTTAC

ACCGTCCACATCCATGCCTTGAGAGCAGCAAGAGTTAAGGAGTTCGCGCTGCTTAGGGCC

AACGCTTCTTCTCCACCCGAGGTGGGGGTCGCCTCTTCGACGTCGGGTTTATCTCTGCCC

GAAGCGGTTACCGGCTCAATTTCGGGCCCCGGGAAGAAGAAAGCCTCCGTGGTAACAAGC

ACGCCACCCCTCGCTGtaccggcggggggggcagtagCTCGATCTCCGGAAAAGgccgcc

gcagcggcggcggcgttgaccGCGCAACCTCTCCCTCTTGAAACGAAACGGTTCGAGGCG

GACCGCGCCCTTGAAACGGCGAAAACGGGAAAGGTTCCCGATGAAGATGATGCTGTCGCC

TCTTCGATTGAAACCCAAGCGGGAGAAGGGCGATTGTCGAACGCAGTCCCGGGCGCTCCG

GGCCAGGGCGCAGTAGCACACTCGGGGGATGGCGCGGGTTCAATCGTGGCAGGGAAGGGC

atcgcggtggggggggtgggcgcgGGTGGAGAGGGGACGCCTAAAGCTGCGACCCCTTCG

CGAATGCCGGCGGGAACTGAGGAGTCTGCGGGCGGAAAAGGGTTTGCAACTCCGGCTGTG

GCTGgcggggtcgggggtggggggacaaACGGGACGGAGGCGGACGGGGAAGTGAAGACG

CCGGCGGGTGTGCAGTCTCCTTCGGGGGGAGCTGAGAGGAACGCTGCTGTAGCCGAAGGC

GTGGAGAGGTGTGGTGGGTTGGGGGGTGACCCACTGGTGGTGAGCAGTGGCACCGTTTGG

CCCGCTGAAGATTCGCTCTCAAGCGCTACTGGTGGGACTAGCCTCGATTCGCCGGTTGTT

GACAGCGAAGGCACTGCTGAAGCGCAGCCGGCGCCTGCTTCTCCCTCGGGACTCGAGCTA

CGCGGGGTCGTGGTGGTCGAGACGGATGTGCCACGTGTAGCAAGCGTTCAAGAAGACGAT

GGCCACCAGCCGGACTCGGAAAGCAGTGATTTTTCTGGCGGGAACCTCCCTATCCGGACA

TCGCCGCCTCCGTCGCTGCTTGCGGAAGAACCTACGGCGTCCCCTTCGACAATTGCTGCC

GGCCCTAAATATGTCTCTCCCAAGCACGCTCGGGCCGAATTGCGCAGTGATGGAGTCTGT

GGTGGCGACGGCTCGCCGAGCAGGGATAGCCAAATCCAGGAAATTAGCGCTGTGGGAGCT

GGTGGTGCTCAAGCCGCCACAAGCCCGTTGCTTCCGTCGCGAGTCAGCACCAATACGACC

CCTAGAGGTTTGGTCAGTGGGGGTGGAAACAGGGGAAGAGTTGGCGAGGCACCCTGGACC

CCGCTGAGTCTGTCACCTCTGGCTTTCCCGGAGAGGTCGGAGGAGGTTGACGGGGAAGAG

TCGGGCCGCCAGGTCGTTGGTGATGGCGACACGGTGCAGGCGTTGGCGTTGGAGCAGCAG

AATTCCGCTCCGATGGACGGCTCCAACACGCGCGGGGGGGAGGCGCCCTCCGAGGCTGTG

CTCAGCCCAGGAGAGGCTGCAGAGGAGGCTGAGGAGCTTGCCTCCCTCGAGGAGCGTCtg

tggtccgggtgggatgCTTTGCTGGCGGAATACCATGCCGCGGCTGCCCTGGTTAAGTCT

AGACGCAACCGCCACGGTGTGGTGGGCTCTGTTTCCTCGGTGCCGCCGCATGAAAACTCG

CCGGTGCCCCGTCCGTTTGAAGCTTCTGCGGCGCCGCCGCCTGTCGCCACGCCTAACGGG

GCGATGGTAGCGACGCGTTCGGTTTCAGAGGGCCTGGAACATGCTCTTGCGTCGCGTGTG

AGCGCGGCGTCTTCGCCTCCCATCGGTAGCGTGATTGAGACGGGAGGGTTTCCACGCGGG

GGTGGCGTTAGAGCATCATCGTggctgccgcaacagcagcagcagcagcgaccgcaGCCC

GCGGAACGCGATGATTACATTGGCGTGCTCTCTCCGACGTCCCCACTCCTTCCCCTGCTC

GACCTTCGCCTTTCGGAGGAAGAGGAGCTGTTCGAGTCTCGGGAGTTGCGCAGGGCTGTC

GAGGGCACCCGCGCGTCAAGAGCGACGACCAGGAGCACTAGGAGGAAGAGCCCGGCGCAG

CAATTCTCTGCCGAGAAGAGGGATAGCGGGCtaaatcagcaacagcagcagcgacagcgg

cagcaacagcggcagcagccggaCGGGAGGGCGATGGGATCGGCGGTAGCGGAGGCTGCC

GCCGGGGAGAGCGTGGCgcgcgaagcggggggggcaggggaggaggagggcagcACGTGT

AGCCTTTGTTACAGGAAGGAGTCTGACAGAATCTTGCGCCCTTGTCAGCACGTGGCTTGT

GGTACGTGCGTGGAGAAGCTCAGGGTTCAGGCCGAGCAATCGGGAGAGGCCTTGTCTTGT

CCCTGGGATAGGCAGCCGATAGACGAGATAGATGTATTCACCAAATTTTAG

>g18934.t1

ATGGCGAAAGTGGTTTTCTCAGAAGGGCGAATGCTTGGAAGTGTCGCCGGCGGCATGGTT

GTCGATGCCGACTACGGCGAGACGGACaactcggcagcagcagcagcagcatcaggagga

ggaggagcaccgTCGTCTTCGTTCACCGTTCTGGTGAACACCTTTAAAAGGCCCCGGCAG

CTGGCCGAGGCCGTGCGGCACTACGCGGCGTGCGAAGGAGTGGAGAGCGTCCGAGTGGTC

TGGTCGgagccttccccgcctcccgacGCCACCACGAACCCGGGACTGTTTGACCACCCG

AGGCCCGTGAAGATCCAACGGCACCCGACCACGAGCATCAACAACAGgTTCATTCCGCCT

TCGGACCTATCGACGGAggcggtttttgtggtggacGATGACATATCCGTCCCCTGCGAG

CACCTGCTGGCCGCTTTCGGGACTTGGCGCCGCCACCCGGACACCCTCGTGGGCTTCTTC

CCCCGCTCCCACTCCCACAGCGAacaccccaaccaccaccagcttaGGGGGCAGCAGGGG

GACGAGaagggaggaggcggtggggtcTGGGAGTACCTCTACTTTTGGAGGGTGCTGTGG

ACGATGGAGTACAGCATCGTCTTGACCAAGGCCGCCTTTGTGCACTCCAAGTACCTCGAG

CTCTACAGCGGGGTTTCTAGGGATGGCGGAGGCGGTAGTGGCGATGTCGGGGGCGGAGGT

GGCGCACCAGGGGGGCGCGAGTGGGGCGAGGGAATGGTCAAGGCCATGGCGAAGACCCGT

GCCTACGTGGACTCCCACCGGAACTGCGAGGACATCGCCATGCAGATGGCGGTAACATCG

GTGTCGGGTCTCCCACCGGTGGCGGCGTTTGCCCCCGTGGTGGACATCGGGCTGTTCGGA

GGCATCAgcaccggggaggggggcgggaagtGGTGGACAGCCCCGCACGCCAAGACACGC

TCAAGGTGCCTGGCGGATCTGAACGAGATCATCTGTGCCGGAGCCACCGCTgatgccgct

gtcgccgccgccggcgccggcgctggCCAGGAGCCGTCGAAATCATCCTGCGAAACGTTG

ATCGGAACAAACCTGTTCGCCGTCACCCACCCCGCGCACGCCTCCAGAGGCAGAAGAGGA

ACTCCTGTCGACCCTGGAGACGGTAGTGGTATCGGTGAAGGTGGTGTCGCTGGAAGAGGC

AGGCGAGGAAACCGTGGTAACCGCgacagaggtggtggtggtggtggtggtggtggtggt

ggtggcaactaTAACGGTGATTTGTCGCGGCTGTCCCGCGGCGGCCTCGCTGTGAGGGCG

CCCACCCTGGCGGAGTTCGTGAGCGCGGACATGCTGCTCGTGCCGGCGAGGTTCTGGAGG

TGGCTGGCGGTTGAAGATGCTTTCGGCCGACCgacggaggagcagcagcagcagcaaaag

aaggagtag

>g18996.t1

CTGTTCTCCTGTTCAAGCTCTTCTGACAGGCGCGGCATGAGCAGCAGTGAGGGCAAACCT

GGCCCCGGCATGATCAGCGGGTTCGGGCGAAGACAAAAGCCAGTGGACAGCGGCAACACC

GCCCTGGGGAAGGGGTTCGATCTCATGGAAGACGACCCGGGGTCGCGGAAAAAGGTCGTC

ATCGATGGCTACGACGACCATGGGTTTCAGCTGACGGATGGCGTCGACGTGAGGGGGTCG

ATGATATGCCTGCCCAactcgtacgtgctgtgggAACCGAAGCGATCGGCGGACATCACG

GTCGAGAGCCTCCTCCTGCTGGAGCTTGTCATCCCCAAGATAGACCTGTTGATTATTGGA

GTGGGCAAGAGAATGACAGAGAGACTCTCTCCGGACTTGGTGCAGCACCTCAAGTCCAAG

GGGATCAGTGTAGAGCAGATGGACACGGTAAACGCGTGTTCCACCTTCAACGTGCTTAAC

GCGGAGGACCGAAGGGTGGCAGTGGCTCTGATTCAGCTCTCTCCCGAGGGCGAACCGTCC

GAGTAG

>g19174.t1

ATGACCGGCTTCCTCGGCCAAAAGTTCGACTTTACCGGCAAAGACGGCGGGTGGTATGTC

CTTGTATCGGACCCCCTTTCGATGCACCTTAACATGCGCGTCTCGGCTCCAATCCCCTCC

TTGCCCGAGATTACGTACATTACTGGGCTCTCGCTCCTCACCACCGATGCCGACGGAGTT

GACCACACTGTCGTCATATCGGTCAAAACCCCGCACAGTATGGACAGTGCCTGCCCTGAA

GGTATCAACCCCTGCCTAGCAGACGGTTCGCTCAACGTGGTAGCAGACGGCAATGAGGTC

TTAATGGCGCCCGGCACAGTAACGTTGGGTGCTGGTGTGCAGGTCACCGCGGTCAACATT

CCCGGAGAGTGCAGGTCCTTCGGGTTCGAGACGTACTGGGAGAGGAAGAAGGAGGAACAC

GCGCAATCCGGTCGCAGGCTGAAGCGCGAGACGACGCTGAGCATTGGAGAATGGATCCTT

GCCGACCCTACGGCTACCAACATGGCGGAGTGTACCGCGTACGTCATCCGTGCAATGTCG

GAAGAAGGCGCGATATTCGCACACCAGTCCGAACACGCCTCCTTCCAGATAGTTACACCC

ACGGAAACCATCCGCCTCAGCCATGGCAGGCTGCATCAGCTCCCGATGCGGGACCCTACC

GACCGGTTCGACCTCCCGGACCACTTGACGTGGCAAATGAACATGGCCATTGACCACACC

GACATTAGCGACGACGCCCAAGGCATCCTTGGCGAGACGCTCGTCCCTACTGTTGACGAG

AGCGGCAACGCCGTCATGCACGGGATGGAATGCATCCGCGGGGAAGAGGCCGACTGTAAG

TTGGTGCAGCCTTTGTTTCCGATTGCTGAACGTCGCTAA

>g19376.t1

ATGGTAAACATCATGCTTGGCGGCACAAACGTGCAGCAGCGATACGAACTGGGTAGTGTC

GGCGCAGGCAACTTCTCGGGGCAAGGGTATGGGGGAGCTCCACAGCAAGgacagggggga

gggtgggggcagGGGCTGGCGAATCACGATCGAGCGGGAAATGCTTCCAAGTGCATCTTC

TGGTCGGCAATCGCCATCGGGGCGCTTTTGAGGGGTCGTTCGCTGGAGTTTGTGACGGGG

TATGTGAAGCTAGCCCGAGACTCTCTGGCGGAGTGTTTTGACGGAACATGCGTCGATACG

GCTAGGGCCTACCTTCTCATGGCCTTCTTGTACAGATTTATGGGGGATCAGGCTAAGCAC

GAGGAATACACCGCCTTCGCAAGCAGCATCGTAGATACACTGCCTTCCGAGCTGGTCCCC

TCGGCGAGTCAACACCTGACGACAGACTTGGAAAACCTGTTCCACGTTTTGCGTGACGAG

TTTGAAGTGATAAATTACGGCCGCCGTTCCTTGGACTCTCAAGCCAAGCCCAAGGCAAAA

TCAGAGGATGCCGTCAAACTCCCTGCGGTGCGCCGCGTCGTTCATGAGACGGACCTCTGC

AATATTATCTTCACCGCCGACCGGCGCATGCACCAGGACATCGTCGAGGGCATGGGGAGC

GACGGCTACTCGGCCAACATCGAGACAGAGGGAGAGGAGACCCCTGACGAGGCCGTGGAC

GGAGAGTTGGAGAGGTTCCAAGAGCAGGTCGAATGGGCGACGATGGGGAGGGTCGGGGAG

TGGGCCGATGGTGGGGTCGAGGCGGGTCTGCGGTCTGGTTTCAGTGCCCCTGATTGGGTG

AGGAGCGGAGATGGTGGCATGATACCACCGCTTCGAGCGGAGACCATGACGTTTGTCGGC

GACGTGTTGCCGGAGCTCGAGCGTGTCTCGAGCGTGGTCGAGAGgtcggGAACGTGCTCA

GGAGTGGGTGGAGTTTTCTACCATGGCGCGAAGGCCTACGTGCACATCATCAAGGGCGAG

CGGAAGTGCGCCTTTGAGTCGATCAGGGTCTTCACGAGGGTGATGGCGGAATTTCCAGGG

ATATGTCGCTTCAGGCCGCACCTGATGCACGGCGCGCTGTTGGCTGCAGCGTCGTCGATG

TGCCCGGTCATGTACGAACCAATAAGAGAGGTCTACAATTCGGCCCTACCTGACAATAGC

CCTCCTGCTCCGCCTCTGGAAGAATGGAGCGGGGCAGCCTGCATCTGCGACCACGTGTTA

TGCAGGTCCTTCGAGATCTCAACCAACTACGTCGGCTTCGGACCCAGAAACCAGAGCGTC

CCCGGCGAAAGAGCCACGGGTGTTCCTACACCCCCTGATCTCTTTGCCGCTCGAGCCTCC

CCCGTTAAGGAAGGAACGCACGGATCGTCCTATTGTCTAGGGTGCGGAGCGCGCGCCGCT

CCATGGTTGGCCTCGGCAAGGGGGGCGGAAGTGGGGCCGCCGCTTGCTGCCAACCAGATG

CGACTGTCTTCCCACTCTCATGCAGGAGCTTCCTCGCAGCATACGGTTAATCTCGCAACA

GGAGTAATCGAACCCGCTGAAGTAACGGCAGCGGTTATACCTCTTGGCTCCATGCAGACG

CTAGGCGACGGAGGGAATCCCCGTGATATCCGTGAGGAGGCTGGAGCCGACTGTGGGTTA

AGGGTGCAAGAGCTGGAGCAAATGAGCATCGATTTTCTCAGCGGTTTGGTTTCCGAGGAT

CAACACGAAAGCCAGGCATACATCATGGATGAGCCCGCGGCAGCATGTGCCGACCCTGCC

GTCGTTCCCGCCGGTGACAGTATCTGGTGCCATGGGGGGATTGGGAACGTAGAGGCACCG

GCTATAGTAGACGACGTGGCCGTTGAGAACGACCCGACATCGTGTGGTGACCTCCGCTTG

TTTGACGGCGAGCCAGAAGATGCTGGCCTTTGTTTGACGGAAAACGGATCGATGGACGCA

GGTGCTGGCCTTTGTTTGACGGAAGACGGATCGATGGACGCAGGTGCTGGTCTTGGTTTG

ACGGACGACTACTTGATGGATGCGGCGGAGGCACTGATGAGCTCGCCCCCGCTTGATAGT

TGA

>g19931.t1

ATGATTCCACTCTTCCAGTTTCCGAGCACCGCAGCCGGTAATATGTGGGTCGACGTTCGG

GCCGCCGCAATGTCCGGAGCCGTGATTACGGGTATCATGAACCCGAACAGCGGTCCTGTC

GTCGACAGCGGCACCACGTCCGCCTACCAAAACTTCCTCACAGACGAGATGGGCATTGGC

GATGGCGCGGTGGCTTCGGACGTTCTGAAACCTACGGTAATCTGCTACATTGCCACCGAC

CGAGGTAATCAAGTCACCAGGCCCATCGCTAACGTTATTGCTGAGATCGCATTGTACGAT

GAGCTCTATCCTGGCGTTTGCTCTGGTATCTTCTTTGACGAGGCATCCTCGGCACCGCTT

AcggacgaaaacggcgacctCAACACCGTGGGGCAGCTCTTCTTCGATTACAATGAGGCC

GCACTATCGGCGGGGAAAACCGTAACGTTCAACCCCGGCGGTGCGGCTGACCCTCTGTAC

TACAGTTGGGAAGGTTCGCCTGCCGTGATGAGCTTCGAGGACTACTATTCCCGCGCCGAA

GCCGTGTGCCCCGCCGACCCCGACTTCGAACTAGAGTGCTACCCAGGCGTCAACACCGTG

GGTGTACCCAGGACCCCAACAGGTTCTACCCCTGAGCAGAGCGTGATTCTCGTCCACACC

GCCTCAGAGCTGGAGACGGACACTTCGCTCGTACAGGCCATGGTGGAGAAAGCGGCGTGC

TCCGGCTGGGGTCACCTGTACTTCACCGGCTCGACTTTAGACGGCAACCCTTGGAACGAA

AACACCCTTTTCTGGGACGAGCTAGTCACCGAGGTGTCAAAAGACATCGACATCACCTGC

GCGGACTCAGCTGAATCGCAGATGAGGATTATGGTGCCTCTCCTTCCGAATCCCGTACCA

AGCTCCACTGATGTAACGGCCATGGTTGCGCCTGTTTTGGACGTGCTGTCGAAGATCGAC

TTCGGTGACGTTGTGTCTGTTGTCCTCGATGCGAGCGACGCTACCATATTGGCGTCTGCT

GAGATTCTGCGTGATGCTGGAGCGAGGATATTGTGCTACTTGGATGCTGCTGGCAAGACG

GAAACGTTTACACTGTCCAGCATGTTCAACAACGTGTACGTGTCAGAGGTCTGCGGTGGC

TTCTTCCTGGACAATTTCAACACAGCGATCATCAGTGAAGTTGAAACTCAAAACTTCTAC

ATCAACGCTCAGTTCAAGATACGCGGCTGCACAGTCGTGTTCAAGCCTGTGGACAGCTCG

GCAACTGAAAACGCGTTCCTGTACGAAAACGACTGGGAGTCGGCTTCGATTCAGACTGGG

CGCGTTATTGTCGCCACTGTGGTAGACGGTGCGACCTCTACGACCATTCCCCCAACTACG

GGACCTCGGAGCAATACCGCCGTGATGATGACCGGCTACAGCGGCAGCCCTGCAGAAGTA

GGTCAAGTGTCTCAGCCGTTGTGA

>g19948.t1

ATGCGCACCGCCTTCGATTCCGAAGAGTCACAAGCGTCGACGCTTATCATCGTATCATCC

GTGATCGGCCTGGTCTTTGCCCTTTACCAATTTACTCTCATCGCACGAGTCAAGCTTCAG

TCCGGCGATGAGGCCTCCACTAGCCTGACGAACAACGTCAGTTCGGAGCGCACGAAGCGT

TTGATTGAGATTTACGAGGCCATCCGCAACGGGGCGGACGCCTTCCTGTCCGCGGAGTAC

ACCatctgctgcatcttcatcgtCCTGTTCGGGGCGGTTGTCTTCTTCTTGGTCGGCATC

GGCCAGGACTCTTGGGTTGAGGGCGCCTTCACCACGGGCGCCTTTGTGCTCGGAGCGGTC

ACCTCCATCCTCGCCGGGTACATCGGCATGAAGGTGGCGGTTTACTCGAACGTCCGCACG

ACGATCGGGGCGCAGCGAGCGGGCTGGTCCGACGCGTTCAACGTGGCTTTCCGCGCCGGC

TCCGTCATGGGCTTCGCGCTCACCGGCATGGCCGTGCTCGTACTTTACATCGCGCTCTGG

GGCTACCGCCAGTACTTCGGGGATGACGAGTGGAAGGTGATGATGAACGCCATCTCCGGC

TACGGTCTGGGTGGTTCTTCGATCGCCATGTTCGGCCGCGTCGGCGGAGGCATCTACACT

AAGGCTGCTGACGTGGGCGCCGACTTGGTCGGCAAGGTGGTGCACGGCATCCCCGAGGAT

GACCCGCGTAACCCTGccaccatcgccgacaacgTTGGCGACAACGTGGGTGACGTTGCG

GGCATGGGCGCCGACCTCTTCGGGTCCTACGCTGAGGCCACCTGCGCCGCGCTCGTCATT

GCCGCCACCTGCCCCGACCTCGTCGAGGCCGGCTGGAGCGCGATTGTCTTCCCCATGGAG

GTGTCGGCGGCCGGCCTGCTGGTGTGCCTCGTGGCTTCTTTCCTCTCGACCGACTTCTTC

CCcgtgaaggaggagaaggacatCGAGTGGTCCCTCAAGATGCAGCTGGTTGTGACCACC

GTGGTGATGGTACCCGTCACGTACGTCCTCGCCGTCGCCTTCCTGCCTGACAGCTTCGAC

ATCGCCGCGATGGATGGCAAGACAATCCACACCGGTGCCACCGATGCGTTCGTGTGCGTG

GTGAGCGGCACCGTGGGCGGCCTCATCATCGGCCTGGTCACCGAGTACTACACCTCCTTC

TCCTACGCACCCGTCCGCGAGGTGGCGATGGCGTGCAAGACCGGTTCGGCGACGAACATC

ATCTACGGTCTCGCTCTGGGGTACAAGTCGGCGATCATCCCCGTCTTCGTGCTTGCCGCC

ATCATCTTCACGTCCTTCCAGCTCTGCGGAACTTACGGTGTCGCCCTTGCCGCGATCGGC

ATGCTGTCGACGCTTGCCACTGGCCTGACGATCGACGCTTACGGCCCCGTCTGCGACAAC

GCCGGAGGCATCGCCGAGATGGCCGAGCTTCCCCCTGAGGTGCGCGAGAAGACCGACGCC

CTTGACGCCGCGGgaaacaccaccgccgccgtcggcaAGGGCTTCGCCATCGGCAGCGCG

GCCCTCGTGTCCCTGGCCCTCTACGGCGCCTTCGTCACCCGTCTGTCCGACGGCCCCAAC

CAGACCATGTCTGGCGGCGTGAACATCCTGCAGCCTCTGACCTTCAGCTTCCTCGTGATC

GGCGCCATGCTTCCCTACTGGTTCTCAGCGATGACCATGAAGGCCGTGGGCACCGCCGCC

ATGGAAATGGTACAGGAGGTGGAGATTCAGTTCGACGAGAACCCGCGCCTGCTGGACGAG

GGCACGACCGAACGCCCCGACTACCAGAGGTGCATCGCCATCTCCACGCGCGCGGCCCTC

AAGTACATGGTGCCCCCCGGCGCCCTTGTGATGCTCGCCCCTCTCATCACCGGCACCTTC

TTCGGTGTCATGGCGGTGGCTGGTCTGCTCACCGGTGGCATGGCTTCCGGAGTGCAGGTT

GCGATCTCCATGTCCAACACCGGTGGCGCATGGGACAACGCCAAGAAGTACATCGAGCGA

GCCACCCCCGACTCAGAGCTCCAGGGCAAGGGTTCCGACATCCACAAGGCGGCTGTGGTC

GGTGACACCGTGGGAGACCCGCTCAAGGACACGTCGGGCCCGTCCCTCAACATCCTCATG

AAGCTCATGGCCATCATCTCTTTGGTCTTCGCGGAGTACTTCCAGGCCATCAACAACGGC

CAGGGGATCTTCAACATCGCTAGCTAA

>g20187.t1

ATGGCTATGGACGACCGAAATTCGAAGCGAGCACGGCACCGTGGAGGGGGAGACTACGCT

GGCTTTGCATCGGACTCGatgcgcggtggtggtggcggtcccGGCAAGCCTCTGGCAGGG

TGCCACCTCGAGGTGGGGGTGGCAACTACCCACTTGTTTCCGGGGCAGGCGGTGAGCGTC

CAGGTGAAGCTGCTCAACGACGAGGACGTGGTGCAGAAAATTACGAGGGACATCAATGTC

ACGGTCACCGGCGAAAACAATaagGCGATTAGCGCGCGCCCCGGCGTGTTTAAGCTCACC

CCGCAGCGGCCCACTTTGCTAGAGGGCACGGTCGCGTTCCACCTTGAGCTAGGGGATGGG

TACGTGCGCTCGGGCACCTCCATCAAGGTGCACGTGGAAACCTCCGGAGgcggcgcgggg

gaggggggagagcagTTGAAGGCCAGCAGCGAGCCTTTGATCGTGTGCCAGTCGGCGCTG

ACCGTGACCGGACAGCCTCCGGATAGCTGGTACAAGGAcgagggcggcaaaaacaaCTGC

ATCGACATCGCTGTGCAGGTGCGGGAAAAGGCTAGCGGTAGGTCACGACCGGGGGTGCCG

CTTAACCTCGCGCTCGTTTACGAGATGGGAACGGAGGTCCACTCCCAGGATATTCTGCAG

GTGCAGACCGACGTCCCGCTGGCCACGAACGAATCAGGGCGCGCGCATATCAAGtttaag

ATCACGGAGGTCAGCCAGCGGCACCAAGGCCAGAAGTTCCGCGTGAGGGTCACTCCGGAC

GTGAAGGCGAACCCCAACGTTGGTGACGTCGCTCCCGTGGACACGGTTCCTATCAATGTT

CTGAGCAAACGCAAGAACCGGAAAATCAAGGCTGAGAAGCAGTCCGTGGCAGGGTACCCC

CAGGGCGCTGGTGCCATccagcaggcggcggcggcggtggcagcggcagcggctaaTTCC

ATGCAGGGTCCACCGCTAAAGACGCCTGCTGGGGCCACGGGCACCCTGCGCAACATGTTC

AAGTGGACCGATGCCGTCCTTCAACTGCTGCAGGTGACCACGCTTTGCTGA

>g20197.t1

GGCGGTGATTCGACCAGAAAAGAGTCGGAGTCTGAAATGATTGAGGACGTGCACGATGCC

GTCGCCGATATGGTGCAAATAATCAAAATCTACCAGAGCAGGAACAAAGTGTCGCAGGTT

CTGATGTCGACGCTGTTTAAACGTCGTCAGGAAGAGGCGGAGGCCGTCATCGACAGAGCC

ATTTGGCGCCTAAACTTAAACTTGCACGTAGAAGTCGGGCAAAAACTGCATGAGGTGGGA

GAAGACGTTAAAGAGGGATTACGCCTTCAGGCCGCCCAAGCGTCCAAGGAGACTGTGGTG

ACGGAGCCAATTTCAGAGTCCCTAGCGGAAGCCCGTCGCGTGCGGCGGCAGCGAAAACTG

CATACAATCCAAATCCCTGAAGACCATGTGATGGTAACTGACGAGCTGCTTGGAAAGGGC

GGGTTCGGTGCGGTCTACATTGCCGACTACAACGGCCGAAACGCCGCTGCTAAGGTTCTG

AATGTCACCCACGACCTTGGGGGTCCCGGCGGCTTAAACAGCGACGACGATGATATTGGG

GAGAGAAAACAAGGTACGTTCAAGAGCGAGCGGGTCAGACTCCAACGCAGAGCGTTCTTG

CGGGAGCTAGACGCGATGATGCGCCTCAGGAGCCCTCACACGGTGAATGTTTACGGCGCG

ATGATGTCTCGCGGAGACCGCCTTGTTCTCGTCATGGAGCTCCTAGCGGGCGGCGATCTG

CTCACGTTCTTGCGAAATTCCGTCGAGCCGCTTCCGGAAGACATCGCCAGACGTATCATC

GGGGACGTTTGCGCGGGAATGGCGTTCTTGCATGCCAGGGAAGCCGTCCATGGGGACCTC

AAGTCCGCGAACGTAATGCTGGATGGAGACGGCCGGGCGAAGATCGGAGATTTCGGGACC

TCGAAATGGGTGCAACACACTAACTCCACGGGCCTGACCACTTACACGACCAGCGAGAGT

CAGGGGGCCAAGATGAGCATCGCATGGGCCGCCCCTGAGGTGCTAGATACCACGGGAAAC

TCCTACGCAAGCGACGTGTACAGCTTCGGTATTGTCACATGGGAGGTTATCACCAGACAA

CTTCCTTGGGCGGATCAACCTCTCGCTCGAGACATATTTCTGCGCGTAGTCATGAAGGGA

GAGCGTCCGGTTATCCCCGCCGACTGTCCTCCGGACATCGCACAGGTTATTCGGGCGTGC

TGGGCCGAGTTACCCGCAGATCGCCCCACGTTTGATGAGCTCGTTAAAGGCATGAAGTCG

ATTGGTTGA

>g20225.t1

ATGATGACCATCATCAACAAGGTTATCTCCGCTGTGGCGTTTGCTTCGGCGACGGTGCTG

CTGCACCAAACGACACCAGCCGACGCATTTTGCGCTTGCTTGGCTGTCTACTACTCCCCT

TCCCAGTTTGCCGAGATGGGAGCTGTTGTGCTGCGCGCCGACGTTTTATCCAGgtcgGAG

GTCGACGACGACGCAACCTACGTGCTCGACATAACCACCCTCTACGCGGGGGAACCCGAT

GTCGAGTACGGCGATACCATATCTTTCGTGACGGCAGCCAGCGTTCACGACTGTGGAGTG

GACCTCAAAATCGGGGGCGACTACCTTGTCGGCCTGACCCGCCGGACGCCAAGCAGCAGC

TCCTCCGATCCGGATGACAGCGCGCAGTTCAAGCTGCACGCGTGCGACCTAAACAAGCTG

TGGAGCTCTGTTTTCGAAGCGGAGCACGAAGATCTAGCTCGACTGCTGGatggcgacgac

gacgatgacgggtcgGACGTCGACGACGACGCAACCTACGTGCTCGACATAACCACCCTC

TACGCGGGGGAACCCGATGTCGAGTACGGCGATACCATATCTTTCGTGACGGCAGCCAGC

GTTGACGACTGTGGAGTGGACCTCAAAATCGGGGGCGACTACCTTGTCGGCCTGACCCGC

CGGACGCCAAGCAGCAGCTCCTCCGATCCGGATGACAGCGCGCAGTTCAAGCTGCACGCG

TGCGACCTAAACAAGCTGTGGAGCTCTGTTTTCGAAGCGGAGCACGAAGATCTAGCTCGA

CTGCTGGatggcgacgacgacgatgacgggtGCTGA

>g20257.t1

ATGGCGACCCTGAAGGTTGTCGTTACCGGTGGCGCCGGGCAGATCGCGTACTCCCTCGTC

CCCCTCATCGCCCGCGGTCTGGTCTTCGGGCCCGGCGTCCGAGTCCACCTGCGGCTGCTC

GACATCCCTCCGGCCGCCAACGCCCTCGAAGGCGTGGCCATGGAGGTGCAAGACAGCCTT

TTTTCGACAGTCTTGGACGGCGTTCTTGCAACCACCGACGAGGCGCAGGCCTTCGACGGG

GCACAGGTGGCCATCCTTCTGGGGGGTTTCCCGAGGAGGCCTGGCATGGAGCGGGGGGAT

CTGATCGGAAAAAATGCCAGCATAATGAAGAGAATGGGTGAAGCTCTCGAGCGCTACGCC

TGCCACAACTGCAAGGTGGTTGTTGTCGCGAACCCGGCTCCCACGAACTGCTTGGTGCTT

TCCTCGCACGCTCCATCACTGTCCAGGAGAAATATCAGCTGCCTCAGCCGGCTAGACCAC

GACCGCATGGTGGGCATGCTGCTCCACGAGGCTAACCGCAGCCTTTCCGCCCCAGTCAGC

GCCGGCGGCGGAGACGCAGGAGCGCGTGGGTGGCGACGGCTGGGGCCGGCGGACGTTCGA

GGGGCTTGCGTCTGGGGGAACCACAGCAACTCGCAGGTGCCGGACGCGAGCGCCGTGGAA

TTTTTAATCGACGGCAGTTGGGTGCCGGCTGTGTCGGTCATTGGCGACAGCGGCTGGCTG

GATCTGTCGACCTCCACCCCCCAGGGCGGAAGAGTCCTGGAGGGGGAGGTTTCCGGACTG

GCGGAGGCGGTTCGAGGAAGGGGTGCCGCGGTTCTGGGAGCGCGCAAGCTCAGCAGCGCC

ATGTCCGCCGCGAACGCGATTGCCGGCCACCTCTCTGATTGGCTCGCCCCTGCAGCAGTC

TCAGTTTCGCCTTCGCAGGCGGTGTCGATGGGGGTGACATCGGACGGCAACCCCTTCGGG

GTGCCGGAGGGGTTGTTCTGCTCGTTCCCCGTCCACTGTGGCGGGGACGGAGAGTGGGCG

TTCGCGGAGGGCTACCGACTGCCCGAGGAGGCGGAACGTCAGCTGGAGGCGTCAATAGCG

GAGCTGAGGGAAGAAAAGAGCATTGTGTCGGAGATGCTTGGAGAAGGGGCGGCGACCGAA

GGGTCTCAAACGGCTTCTTATATTGCCTCTTTTGTAGCCCCTGGTCACTCAAGCGCGGCC

CCAGTCTCCAGCCTCTGA

>g20376.t1

ATGGGGTATCGGTTGCTCGCGGCGACAACCCTTGTTTGTAGGGTTGCGGCCGGGGGTACT

GCTGTGACAGCGCATCCGAGTCTACGTGGGTTAGCTGAGGGGCTCGAGATCGAGAGCTGC

CCTCGCTGCGACATGATATTTTCGAAGGTGGAAATCTCTCCATCTGGCGAGCGCGTGCAG

CACGTGGAAATGCAAGAGCGAGGGGAACCTAAGAGCGGAACAGCGTTGATGTATCACGGC

TCGCAAGGTGCCCTTATGCACACGTGCAACTTCTTGAAGCGGTGGTTCGGCAGCGATTCT

TGCCACCTAGAGGGCGAAGACTACACAGGACATCACCACACATCGCCAAACATTGGCCTC

ACATTCGACCCACGGCTAGGCGAAAGCGACGCGAAATGCCCTTGTGACGGGGTAGACAGG

GTTGAGATACGTGTTTCAAGCGATAACAAGCACAGGCTGCCAGTGGACTTAGCTTGCAGA

TGGCATCACAACAGCGGGATCGCTAAAGAGCAAGGGGAGGGCTGTCGGACCGTAGCTGGC

AGGCCAGTGGAAAACTTCCACGACATCTGGCTCTGTATGGTGGAAGCGGACTGCGACGTG

ACAGATGATCGAGTACAGTTTGCCATTCTACGGGACCCTCGAGCAGAGGCCGTGAGCACG

TTTCTCtacgagcagcagcacgcgGACGCCATGAAGAATCACGGAAGCGTCGGTGCTGGC

CTGGACACGGTTGACGAGTTCGTGCTGGAGACTCTTCCCGTCCTTTGTCAGTGGGTTGCC

CTCCGGTACATCCTCTTTGCCGGCATTCTCAGCCAGCAAAGCACCATATTTTGGTATGAC

GACGCCATGGAGGATGTCTCGAAATGGCACCGCGACTGGCTGGCCTCTGTCGGCCTGCAT

CTACCGTCAGCCGTCGTGGAGGCGATGGTAGATGTCGACTTACGCGGCGAGTTCGACTTT

GACACACGCGGCAAAACGAATCTCCTTGAAGACGGTACTAAACTTGCTGAAGGGGAACCA

GGAACGATGCCAACTTGGCAAGATGGCCTGCGTCCAGAAACGGTGATGGAAATGGACCGA

ATCGTGAAGCAATGGCTACCGCCAGTGATCCTTGCGAAGATAGCAGATGTACCGAAACCG

GGTACCCAGTAG

>g20452.t1

ATGGTGGACAGGTACTTGATCCTCGACACCCTCGGCGTTGGCTACAGCGGAAAggttAAG

CGAGGAGTGGACACATCGACTGGCGAAACGGTGGCCCTCAAGGTCATCGATAAGACGGCG

CCCTACGAACCCAGCAGCGAGAAGAAGCTCAAGCGCCTGCAGTCGGAGATAACGGCGATG

AAAATGTGCGGCACGCATCCTCACACTGTGACTCTCCACGACGCGAGGCTGAACGCCCTC

TATCCGAAGAGGGACGGGTCGCAAGAGccggCCCACGTACTGGTGCTCGAGCTCTGCGAG

AAGGGCGAGCTGTTCGATCTTCTCTACAAGGGGGGTCTCGTCCCGTACGATGTCTGCAAG

GAGTACTTCCGCCAGCTCATGGTCGGCATCAGCTACTGCCACAGCAAGGGCGTCTTTCAC

CGCGACCTGAAACCCGAGAACCTTCTTCTCGATGCCAACTTCCAGCTCAAGATTGCCGAC

TTTGGCTTTGCGGCGATCGGAGAATCTCCGATGTTGTGCCAGAGTATCGTGGGATCAAAG

ACCTACATGGCTCCGGAGGTGCTGGGCCGGCGGTCCAACACCTTCACGCACCACGGTGCT

GGATACGACGGGGCGTTGGCGGATGTGTGGAGTGCCGGCGTGATCCTTTTCACGATGCTG

GCGGGGCACCCTCCCATGGAGCAGGCGTCAGAAACGGACTGGTGGTTCCGAGCCCTTAAG

CTCGGGCGGCAGGATCTTTTCTGGAAGAGCCACGAGGGCAACACCCGCCCCTTCCCGGAG

GAGGCGAAGGTTCTTATCAGCGCCATGCTCATGGTCAACCCCGCGGAGAGAATCACGGTA

TGGAACATCATGCACCACACGTACGTagcaccgcccccgcccgcggCGGCCGCCAACGCT

CGCCGCTCCGGCGGGATGTTCTCGAGgcagagcaacagcagccagccgGTGCCCTCGATG

GACCCCAGCGCCGCCCTCTTCATGGAGATGCAGCGACGCTACGAGATGTCGATGGCCTCC

AAGCGACTGTGCACCgtcgagagcagcagcagggggaccGCCGACTCGGCGGGGGCGTCG

GGTTCCGGGTCTCCCACCTCCGCCACGACCCCGGCGAACCCTCACATGGCCATGGGGATG

CTGCACGGGCCTTACGGGGGGCCGTTCTCGCAGGCGACGCACAGGGCTCTGGACGACGAG

GACAACTCGCCGCCCGAGATGAGCGCGGAGGAGGCGGCGAGGGTCACCAACGGGTTCATG

GCCAACGGAGATGCGAGAGACGTTGTCAAGGCTCTGGAGACGGAGCTGTTCGACATGGGG

GCCGagATCGAGGGCGTAGGAAAGGACGGTACTCGAGCCTTCCGATCTTCGTTCAAGGTC

ACTGCGGCTATCCCAGCCGTCGACAAAATCGGAGGGAGCGAGGTGGCTCTGGTGGCCCTG

GTGTACCGCGTGGGGCGCGAAGGGAGGCAAAACGCCAACAACGAAATGCCGGGCAGGTTT

AGGAAACCCGCAGAGGCCgacttggtggtggtgctgaagAGGAGGAGAGGACACTACCAC

AGGTACCGCAGAATTGAAGACAGCCTGAATTTCTCTCGACACGTCGTGACGGGAATGAAG

GCGGCGGAGATGGCTTCGTCTTCAGTGAACATGTCCCCCACTCCGTAA

>g20678.t1

ATGGGTTTCGTGCGAGGCAGCGTCGTTGGCGTTGCGGCCCTGAGCTGCGCGCTGGCACTC

CTCTCCGGAAGCGTCCTCGCCAGCAGCGACTCCCCCTCGTCCAGCTGCATCGCCTCCTTC

ATCTCGGATGGAGACTGTGACCTGGTCAACGACACCGCGGAGTGCGGCTTCGACGGAGGC

GACTGCTGCGAATGCACTTGCGTCAGCACCGAACAGTTCACTTGTGGCGAAAACGGCGGG

TTCTCGTGCCTCGACCCGAGCGCGCCCTGCGTCGATGACGATGATGTGACCACCCTGCCG

AGCTCCGAACGCTCCGACCAAGTCTGCCAGGAGAGCTTCATCGCCGACGGTGACTGCGAC

CCGAGCAACAACTTCGAGGAATGCGGTGGTTTCGACGGCGGCGATTGCTGCGAATGCACC

TGCGTCAGTACTTCGGACTTCACATGCGGGGACGACTTCAACGGCGGCTTCGAGTGCCTG

GACCCGAGCGTTCCGTGCTTCATTAGCGGCGATGACGATTTCGCTGGCGGCGACGACGAC

ACAATGTTCGACGACGACATGTCGTCGAGCTACGACTTCGAATTTTCGCAGAGCTTCCCG

GACAGCTTCACCGACTCCTCCGCCCCATCCTCCCCCTCGTCATCATCGTGCTTGGATGCG

TTTATCGCGGACGGTGAATGCGACATGGAGAACAACAGCGACGAATGCGGTTACGACGGC

GGGGACTGCTGCGAATGCACCTGCGTCAGCGGCTTGTTCGCCATCTGCGGCGAGAACTCC

GAGTTCAAGTGCGTCGACCCGAGCGCTGTGTGCTTCAACGGCAACGTCGAGGCCGGCACA

AAGACCAACGTCTTCGTGTCGACCAACGCCTACGACACCCGCCCCGGTCAGGCCTCCGGT

CAAAGCGGCTGCCTGGTTAACGGGTGCGCGCCCGAGCTTACCCGGGACGGAATCAGTGAC

GACGTCGAGTCGAGGTGGTCCTGCAACCCGAGCATCGTCTCCGACGGAGGGCTGTGCGAG

ATCGAGTTCGTGTTTGAAGCTCCCCAGGACCTCATGAGCGTCCAGGTGGCTTTCTGGAGG

GGTGAAGAGCGCACGCGAACGCTGGAGGCTGCCATCGATGGCGAGGTCCTCGGCAGTTTC

GACTCCTACCCGGGGTCAGTCTTCAACGCGTTCGACATCAAGGGGAGCGACGTACACACA

CTGACTCTCACATCCACCGGCATCACCCAAAACGAATGGATCAGCCTGATCGAGGTCCGC

CTCATGGTCGCGCCCTAA

>g20950.t1

ATGGAAAGCCGCggCAGGACCATTGCCTCGGGCGCTCCGGTGCTCCTCCTTTTGTTATTC

GCGAGCGTCTGCCACGGACTGGTGGAAACGTGCGAAGATCTACAGGCGGCTTTCGATCTC

ACCAAGACCCAGGATGTCGTCGTTCAGATACCCCGCTCGGTCAGCATCAAATGCGCCAAG

TTCACCACTATGAGCATGGGCTCGAACACTCTCAGGGTGGAGCCTCCCGAAGGATCTACT

CCTATATTTTTCCAGCGTGTCGCTCTGTCCGAGATTCGGTTCGAGGTTGTGAACGGAGCA

TCGCTGGTCTGGGAGCATGATGTGGCATTCCGCGGATCGGAAACGCAGAACGTCAACGGG

GGCGGGTTGTTCGTCGGAGAGGGCTCTACCGTCCGCTTCTACGGCAGTCTGGACATGACC

GACGTAGGCGTCCGCAGTTTGACCGAAGAGGGAACCGATTTCTCCCGGGAAGAACTCAGC

GGAGGATGTGTCTACGCCGACGGCTACTTCAGCGTGGACGGGGAGGCAATCTTCACGCGC

TGCGAAgtggggggtggcggggagagCCCCCCTGGCCCCGGCGGCGCGGTGTACGTCGGG

GGGCAGGGCTCGGTCCTGTTCAACGGCGGGGTGAAGATCTCAGAAGTGGACATCATCGAC

GACGACGGGAACAACGGAGGAGGCATTTACAACCTGGGCAAGGTCACCATCAGAGGAGAC

TCGGTGTTCGACGACCTTCGGGCGGAGGCCGGTGGGGCCATCTTCAATGCGGTCGGTGGC

CAGTTCAGCTTCAGGGACGGGGCGACCGCCCTGTTCAAAGACTGCTTGGCGTTCGATAGC

ATCGGAGGCGCCTTGTACAACAAAGGTTCCTTCAAGTTTTCTGGGCCAGCTCTTTTCGTC

AACACGGATTCCCCCTCTGTCTACGTTTCGTCCGCGGGGAAGACCGTGTTTTCGGAACGC

TCGGTGTTCTGGGACAACGAGGACAACGCGCTCGGTATCGACGCCCCTAACCGCGCGGTC

GTCGTTGCCCCGGGAGGCGAGGTTGTCGTTCCCATCTCCGTTGTGTTCTACGGCAGCGAC

GACTCTGAGTGCGGGACGGTGTTCTTCGAGGAAGACGAGACGTGCCTTTGA

>g20951.t1

ATGTTCGCGCAAACGGCCCGCAGGTCCTTCGCATCTAGCGCTACGCTCCTGTTGCTGTTC

TCCAGCGTCTGCTAtggattggtcgaaacgtgcgAGCAGCTGCAGGCGGCGTTCGACTTG

ACCAAAACCCAAGATGTCGTCATCGAAATGCACCCCTTCCAAGACATCGACTGCGTCACG

GCCACCACGATGAGCATGGACTCCAACACTCTCACCGTGCAGTCTAGTGAGGACCTCGAA

AGATTCGGCGGAAACTCAGATCTGTTCCAGGTTCGGTTTGAGGTGACGAACGGCGCACAA

TTGATCTGGGAAACGAATGTAGAGTTCCACGGGCCGACTAGGCAGAACGTTAACGGAGGA

GCCGTGTTCGTGGGAGAGGGCTCGAGCGTCCGCTTCTTGAACGACCTATTGATGGACGAC

GTCAGCGTCCGCAGCGTGCCCGAGGATTACTCCGATTTTGCCCAATACGTTCTAAACGGG

GGTTGTGTCTACAACGACGGCGACTTCAGGGTGGACGGTGACGCAACGTTCACCAGGTGC

GAAGTGACGGGTGGCGGGGAAAGCCCACCGGGCGATGGCGGTGTGCTGTACGTCGGCAAA

CAGGGCTCCGCGCAGTTTAACGGGGAGTTGGAGATGAAGTACGTGTCCATtaccgacgac

ggcggcggcaacggaggAGGCATCTACAACGCGGGCAAAGTAAACATCAAAGGAAACTCC

AGGTTCGAATCTCTCCGGGCTGCGGACGGTGGAGCCCTCTACAACGCGGGCGGTTCCCAG

TTCAGGTTCACGGATCAGGCGACCGCCCTGTTCATAGACTGCACGCTGCGCGACGGCATC

GGTAGCGCGTTGTACAACGCAGGGAGCTTCAAGTTTTCTGGTCCCGCTCTTTTCCTCGAC

CAggaaacgccagccatctacgtTTCTTCCACTGGCGAGACCGCGCTTTCGGAGAACTCG

CTGTTCTGGGGTGACGGACCCCGCGAGGAGGTCATTGGCGTTGCCTCAGGGGGTGTGCTC

GATATCCCTAGCTCCGTTGTGTTCGTCTAG

>g20952.t1

ATGGGAAGCCGCGGGTCCTTTGCATTTAGCGCTACGCTCCTCTTGCTGTTCTCAAGCGTC

TGCCATGGACTGGTCGAAACGTGCGAGCAGCTGCAGGCGGCGTTCGACTTGACCAAGACC

CAAGATGTCGTCATCGAAATGCACCCCAGCCAACGTATCAACTGCGTAACTGCCACCACG

ATGAGCATGGACTCCAACACTCTCACCGTGCAGTCTAGTGAGGACCTCAATACATACCGC

GGGAAATCGGGTCTGTTCCAGATTCGGTTTGAGGTGACGAACGGCGCGCAGTTGATCTGG

GAAACGAACGTGATTTTCCACGGGCCGACGGACCAGGACGTTAACGGAGGAGCCATACTC

GTGGGAGAGGGCTCGAGCGTCCGCTTCTTGAACGACCTACTGATGGACGAGGTCGGCATC

GTTAGCGTTCCCGAGGAAGACTCCGATTTTGCCCAATATGTTCTAAACGGGGGTTGTGTC

TACAACGACGGCGCCTTCAGGGTGGACGGCGACGCAACCTTCACCAACTGCGATAACTCG

GGTGGCGGGGAGAGCCCACCAGGCGATGGCGGTGTGCTGTACATCGGCAAACAGGGCTCC

GCGCAATTTAACGGGGCGTTGCAGATGAAGTACACTTCCATTACcgacgatggcggcggc

aacggaggAGGCATCTACAACGCGGGCAAGGTCGACATCAAAGGAAACGCCAGGTTCGAA

TCTCTCCGGGCTGCGGACGGTGGAGCCCTCTACAACGCGGGCGGTGCCCAGTTCCGGTTC

ACGGATCAGGCGACCGCCCTGTTCATAGACTGCACGCTGCGCGACGGCATCGGTAGCGCG

TTGTACAACGCAGGGAGCTTCAAGTTTTCTGGTCGCGCTCTTTTCCTCGACCAggaaacg

ccagccatctacgtTTCTTCCACTGGCGAGACCGCGCTTTCGGAGAACTCGGTGTTCTGG

GGTGACGGACCCCGCGAGGAGGTCATTGGCGTTGCCTCCGGGGGTGTGCTCGATATCCCT

AGCTCCGTTGTGTTCGTCTAG

>g20957.t1

ATGCCCGCTCTTCTATTCGCGCAAACGACCCGCAGATCCTTCGCATCTAGCGCTACCCTC

CTCTTGCTGTTCTCAAGCATCTGCCATGGACTGGTCGAAACGTGCGAGCAGCTGCAGGCG

GCATTCGACTTGACCAAGACCCAAGATGTCGTCATCGAAATGCACCCCTTCCAAAGCCTC

TACTGCGAAAATGTCACCACGATGAGCATGGACTCCAACACTCTCTCCGTGCAGTCTAGT

GAGGACCTCGAAGGATTCCGCGGAAACTCGGGTCTGTTCCAGATTCGGTTTGAGGTGACG

AACGGCGCGCAGTTGATCTGGGAAACGAATGTAGAGTTCAACGGGCCGGTGGACCAGGAC

GTTAACGGAGGAGCCATATTCGTGGGAGAGGGCTCCAGCGTCCGCTTCTTGAACGATCTA

TTCATGAAATATGTCGGCATCGCTAGCGTGCCCGAGGAGGACTCCGAGTTTGCCCAATAC

ACTCTAAACGGAGGTTGTGTCTACAACGACGGCGACTTCAGGGTGGACGGCGACGCAACG

TTCACCAACTGCGATACCTCAGGCGGCGGGGAGAGCCCACCGGGCGATGGCAGTGTGCTG

TACGTCGGCAAACAGGGCTCCGCGCAGTTTAACGGGGAGCTGGAGATGAAGTACATTTCC

AATACCGTCGACGTCGGGGGCAACGGAGGAGGCATCTACAACGCGGGCAAGGTCGACATC

AAAGGAAACGCCGAGTTCGAATCTCTCCGGGCTGCGGACGGCGGAGCCCTCTACAATGCG

GGCGGTGCCCAGTTCAGGTTCACGAATCAGGCGACCGCCCTGTTCATAGACTGCACATCG

GACGACGGCAACGGGAGCGCATTGTACAACGCAGGGAGCTTCGAGTTTTCTGGTCCAGCT

CTTTTCCTGGAACAGGAAACGCCAACCATCTACGTTTCTTCTACTGGCGAGACCGCGCTT

TCGGAGAACTCGGTGTTCTGGGGTGACGGACCCCGCGAGGAGGTTATTCGCGTTGTCTCA

GGGGGTGTGGTCGACATCCCTAGCTCCGTTGTGTTCGTCTAG

>g20958.t1

ATGCTCCCCCTTGTGTTCGCGCAAACGGTCTACAGGTCCTTCGCACCTAGCGCTACGCTC

CTCTTGCTGTTCTCAAGCGTCTGCCATGGGCTGGTGGAAACGTGCGAGGAGCTGCAGGCG

GCGTTCAACCTGACCAAGACCCAAGATGTCGTCGTCGAAATACGACCCTTCCAAACCATC

AGCTGCGTCACTGCCACGACGATGCGCATGGACTTCAACACTCTCACCGTGCAGTCTAGT

GAGGACCTCGAAAACGTCGACGAACGCCTAAGTCTGTACCGGGTTCGGTTTGAGGTGACG

AACGGCGCGCAGTTGATCTGGAAAATCAAGGTGGATTTCAACGGGCCGACGAGGCAGGAC

GTCAACGGAGGGGCTGTGTTCGTGGGAGAGGGCTCGACCGTCCGCTTCTTGAACGACCTA

GAGATGGACGGCATCAGCGTCCTCAGCGTGCCCGAGGAGGACTCCGATTTTGCCCAATAC

ACTCTAAACGGAGGTTGTGTATACAACGACGGCGACTTCAGGGTGGACGGCGACGCAACG

TTCACCAACTGCGGTATCACGGGTGGCGGGGAGAGCCCACCGGGCAATGGCGGTGTGCTG

TACGTCGGCGAACAGGGCTCCGCGCAGTTTAACGGAGGGTTGGAGATGAAGGACGTGTCA

ATTGTCGACGACGGCGGGGGCAACGGAGGAGGCATCTACAACGCGGGCGAGGTCGACATC

AAAGGAAACGCCGAGTTCGAATCTCTCTGGGCTGCGGACGGTGGAGCCCTCTACAACGCG

GGCGGTGCCCAGTTCAGGTTCACGGATCAGGCGACCGCCCTGTTCAGAGACTGCACATCC

GACGACGGCAACGGGAGCGCATTGTACAACGCAGGGAGCTTCGAGTTTTCTGGTTCAGCT

CTTTTCCTGGAACAGGAAACGCCAACCATCTACGTTTCTTCCACTGGCGAGACCGCGCTT

TCGGAGAACTCGGTGTTCTGGGGTGACAGACCCCGCGACGAGGTCATTCGCGTTATCTCA

GGGGGTGTGGTCGACATCCCTAGCTCCGTTGTGTTCGTCTAG

>g20971.t1

ATGATCGCGTCAAGCTTGCCGCTCCTCTTGCTGCTGTCGAGAGTCTGCCACGGGCTGGTC

GAAACGTGCGAGGAGCTGCAGGCGGCCTTCGATCTCACGAAGACCCAGGATGTCGTCATT

GAAATGTATCCCTATGCCACCATCGCCTGCGACGTCTTCACCACGATGACCGTGGACTCC

AACAGCCTCACCGTTAAGCCTAGCGAGGACCTGAGCAGTATTTACACACACCTGGAACTC

GAGAAAATCCGGTTCAACGTGACGAACGGTGCTACGCTTTCCTGGGAACCGAACGTAGAG

TTTATCGGGTCGGGCCATCAGGACGTCGACGGAGGTGCTCTGTACGTCGGGGAGAGCTCA

AGTGCTCACTTTCTGAACCGCTTGGAGATGGAGGATGTCAAGGTTGCCAGCGAGACAGAC

AACGCCGATTTCGCCACATACACGCGCAGCGGGGGGTGCCTGTACATCGACGGCTACCTC

AGGGTGGACGGGGCGGCGACGTTCACCGGTTGCGATAACGCTGGCGGCGGGGAGAGCAGC

CCGGGCCCTGGCGGTGCGATGTACGTCGGGCGGGAGGGCTCGATGTTGTTCAAGGACACG

CTAGCGATTTCGGACACTCATCTGACCAACGAGGGCGGAGATGACGGTGGAGGCATTTAC

AACGAGGGCAAAGTCAACATCAAGGGGGCCGCGACATTCACCAACGTGTGGGCTTACGAC

GGCGGCGCCATCTACAACACGGTGCACGGCACGGTCAACTTCAGGAACAAGGAGGCGACC

CAGTTCATCGACTGCTCCGAACCCGAGCGCATAGCTGGCGCGGTGTTCAACGAAGGATAC

ATGAAGTTCACCGGGCCAGCCCTTTTCTACGACAGCGGATACCCCGCTATCACCATTTCG

GCCGATGGCGAGACCGTGCTCTCGGACCCCTCCGTGTTTTGGGCCACCTTCGGTGACAAC

GAACCTTTTGTTCTGGTTGAGTCCGGAGGCCAGCTGGTGATTCCCGAGTCCACGTTGTTC

GTAAACAACGACGAGGCTGACTGCGCGACGGTGTACTACGAGGAAACCGACACATGCCTT

TGA

>g21023.t1

ATGATTCTGGACGCCCCCACGATGGCGAATGATCAACTCCAAGGAACACTTCGTGTGTTG

CTGCCTGGTACAGACTTGACCGCTTCATCTTGCGATTGCGAGCACCACGACCATGATGAC

CACCTTGACCTCTCTGACTGCAGCAGCGAGGCCAGCAGCAGCGAACAAACACCTGACAGC

AAGCGCCGCCAGCCAGTCCTCCTGGCGAAAACCCAGCTGGGGATCGCGCAAGCCGTTCGC

ATATTCGGGGGGGTGATAATTGGCACGGGTGCCGAGGAAATTTCTCGCAGTTGGGGACAA

TTATCGGATCCCACCTTCTCGACTCCAGATTCGGGCGGAGAGAGCGACCCTGGCTATGAG

CCTTTCGTGGAGTCGTTCGACCTCTCGGACCCCTCTTCCCACCAGCTATCAGGGGAAGCC

CCCTTCCCTGACCCCGAGTGCAGCAAGGGTCTGGAAGGGGACGATATAGGCAGGGTGAGT

GATAGCAGCAGCGACCGCACATTGCCGGAAAGGCCCGTCGGGGGTTTAAAGCGAAAGTTG

CACTCGTTTGTTGAGGACGATTCGCCGGGTTTAGTCGTCACAGGGGACGCCACCCCTCGC

GCCCCGGGGGGCCGGGCAGCCGCTCGCGCGAAGGCTGAGGGGCAAGGGTGCACGAGCTTC

GTGTCCGAGTGTGGCCTGCCGACGGACAAGGGGAGGTTCAGGCTGCGGGCGTACCGCTAC

CACGGCAGCGACAAGTCTCATGAGCCTGTGGTGATGGTCGCGGGGAAGGTTCGCGGGAGA

GAGGGCGTGCCGGTGCGAGTTCATGACCAGTGCCAGACTTCAGAGGTGTTTGGCTCTAAG

AAATGCGACTGCAGGGAGCAGCTGGACATGTCCCTGAGGTACATCCAAGAgaacgggggc

ggggcggtgaTTTATCTTGCCCAGGAAGGACGCGGCATCGGCCTCGCCAACAAGGTCGCG

GCGTACGCTCTACAGGACGGCGGGCTCGACACGGTGGACGCTAACCGACAACTCGGGTTC

GAGGATGACCAGCGCACGTACGACTGCGTCGACTTCATCCTCCGAGACATGGGCATCAAG

AGCGTCCAACTTATGACCAACAACCCGTTCAAGATTGAGTGGCTGAGGGCTCTGGGGATC

AAGGTGGACGGACGCATACCTGTGGAGGTGACTTCGAACGAGCACAACCACGGATACCTT

GAAGCCAAGGCCGCTCGCATGAGCCACCTCATCAATAACttgtag

>g21247.t1

ATGACGAGGCAGGAGAGCGCCTCCGTGTTCCAGCAAGGCTTCCGCCCAAGGCGTCGCGGC

TACCCGACCTCGAGGAAGGCAGACGCCCTCGCAGCATATTCGGCGACTCCACAAGTCGCA

CTGGGCGAACGGTTCTTCACCCCCGTCCGGCACCGCGAAGAAGACAAGCCACCACGACTC

AGCCCTCCACTCGTGGGGGCATTTCGGGACAAGGCGGAGTCGAGGCCTTACAAGGACGGC

GCCTTGATTTCTTCCTACACCGATCGCTTAGAGCAGCAGCTGGCTCGTGTGCGGGCTAAA

ATGTGCAAGCAAGCCTTGACCATAAGCGAGCTCGCCGGGATGTTTCGGACGGAGAAGGAA

TATCGCATAAGGTCCCAGAAGGAGTCTTTTCGGCTACGTGAGCAACTGGAGAGGGCCAAC

GCACGGGCAAGCGCTGCCGAAGTGCGCGCACGGGCCGCAGAGGCGGTGGCGGCCGGTGTC

CCGTACCAAGATGTGGGTCGTCCTGACCTGAGGTTAGCGGTCGGCACGCCGAGAAAAGTG

ACCACTCCGCAAGACGACGACGGCACACCGCTGGACGCCCCAAACTTCGTCGGCCGCTTC

TGGCAGCTGCAAAAGGGGCAGTGCCACAGGGACTTTGTGGCGAAGGTGCGCACCGAGTGT

GCGCTCAAGATGCGCGTAGCTGAGCACGCCCTGGCCCCGCGAAGAAGGATGCTCGGCCCC

TTGGAGAAGCTCGGGGAGGGGGGCTTCGGAGGCGTGTACAAGGCGACCTGCAGAGAAGCG

AACGCTGTGTTCGCCATCAAGAAGCCCAAGACGGAAGCGGACAAGCAGGCGCTCATATAC

GAGATGCTGGTGTACACCTGGGTCGCTCCGCACCCCAACATCATCGGCGTGCACGCGATG

GACGACCGAGGGCCTTGTCCGACTTTGGCCCTAGAGTGCGGTGACTGTGACCTTTTCGCG

GAAATCAACAGCCAACGCCTGTCCCTGCCTGAATTACTCGACCTTGTAGGTGATGTGATC

GAAGGCGTGGATCACCTCCACAAGCACAAAGTTGTTTTGGTCGACAACAAGTTCGAAAAC

GTCATCGTGAAAAGCAATGGCTTCCGCAGGACCGCGAGCCTCGCAGACTTTGGGCTGGTA
[truncated: 226,308 more chars]
