# Supplementary material for: Identification of differential gene expression related to reproduction in the sporophytes of Saccharina japonica
Source: Front Plant Sci. 2024 Aug 6;15:1417582. doi: 10.3389/fpls.2024.1417582 (PMC11333212; doi:10.3389/fpls.2024.1417582)
Supplement: Supplementary file 2 [file DataSheet_2.docx]

**Supplementary Fig.2**

**The sequence of downregulated DEGs**

>g113.t1

ATGTCCACCCCCGACGCATCGACGCCCTCGGCGCCGCCTCCGGCAGAGGCCATACCCGTC

ACTTCAGGGCCGGTGACAACATTAAAGCCAGGGGCGAAAGTAAGCAACGAGTGGGAGCTT

GATGTCTACTCGAGGCCTGTTGTCGGTGCTGACGGGAAGAAGCTGTGGGAGCTCCTTATC

TGCGACTCGACGGGCAACATGCGGCACGTGTCGCCTATCCCGAGTAACATGGTCAACTCC

CGCGAGGTGCGGAAGACGATCGAGGGGGTGATCGAGGCAGCACCAGGGGGCTCCAAGCCC

ACTGTGATTCGATTCTTCCGTAACGCTATGTTCAACATGATCGACATCGCGCTGAAGGAG

GTGGAGGTGGCCGTGAAGCCTTGTCGAACGACCTACGCGATGTACCAGTGGCTAGAGCAA

CGAGAGCGCGACGTGTACCCGAACATGCCTGGCTACAAGCCGACCATGAAGCAGCCGGCG

TTCTTCGACATTCGGACTCCGACCCCTCTCCCGGACGCTCTGCGAGGAGAGCAGTACGCC

TTCGTAGGCATGCCGGTCTCCGAGTTTCGCCAGGGGAACATCAACGAAGACAACGTTGGA

GTCGGCAGGCTGTGTCCGCTCGATGACTCCCTTCCGGATGATGCCATCATCCCCGGCCTT

GCGATGTTCACGACGAGAGCGGAGCCCCTCGCGACATGGATGACGGGCCTCGAGGTCGCC

TATTTTAAGGCTGACCTCAAGAACCGGGAACTCGCTCTTGAGTGCGGCATCAACACGCAG

TACCTTGTCGCACGTGTGATCGGAGACCAACGCAAGGAAGCGCAAAACTTCGAGGAGACG

AAACGATCTCTGGGGGGTTTCCACTTCGTGGCGGTGCAGAACGACGCTGACGCCGATGAC

GTCGCGGGCTTCTGGTTACTGAAGGAGGTCAACATCTAG

>g214.t1

ATGTCGGCCATCGGGGACGGCAACTCCATGATTCCCACGGATAACGTGGTGATGGGCGTG

CCGAAGAAGGGGAGGCTGTACGACCGCTGCATGAAGCTGCTGGCGGGCGCTGGCATGGAC

CACCGGCGGCCCGAGCGCGTGGACATCGCTCAATGCAGCGACCTCCCGGTGACCCTGGTG

TTCCTACCGGCCCACGACATCGCCATGTTTGTGGGGGAGGGGAACGTGGACATTGGCATC

ACAGGGCTAGACGTGGTCAAGGAAACTTCGGACGAGAGCGAGATCGAGGTGGCCATGGAG

CTAGGGTTCGGCAAGTGCAAGCTGTGCGTGCAGGCGCCGGTGTCTGCTGGGATAAAGGAC

GTCAGGACGCTCTCGGGCAAGCGTATTGTTACGTCGTTCCCGGCTGTGTGCAAGGCATTT

TTCGACCAGTACGACACACCGGACAACCCTACCAATATCAAGTACGTGTCTGGGTCTGTC

GAGGCGGCGTGTGGCTTGGGATTAGCGGACGCGGTGGTTGACCTTGTGGAGACGGGAACG

ACGATGAAGGCGGCGGGCCTGGAAGTGGTGGCGGACGTGATGGAATCGCAGTGCCTGCTC

ATCGTGGGGAAGCAGACGAAGCACCGGGAGTTGGTAGACCTCATCGTGCGACGCATCGAA

GGCTACATCACGGCAGAAAAgCACGTCATGGTGTCGTACAACGTTACGAAGACGAACCTC

GAAGCGACGAAGAAGGTGGCTCCCGGCCAGCGGTCGCCCACCGTGTCCCAGCTCGACTTG

GACGAATGGGTGGCGGTGCAGTCTCTCATCTTGAAGAAGGGGTCGAGCCGCATAATGGAC

GAACTCCGCGCCTGCGGCGCAATGGACATCCTCCTCTTCTCGCTCCACAACACACTCATC

AGCTCGGCCTGA

>g537.t1

ATGTCCAAGATATCGCCCTCATTCGTGGGTTTGGCACTTGTGTTGGCCGTTGCGCAGCGG

TCTTGCTACTGTACCTCGACGCTTGTCCTGCCCGACACAGCAGCTTCTGCCTCGGTCGGC

CACCCTGCGAGTACCACGAGAACATCATTTGTTTCAACGCCATTCTTGGCGAGACTTCAC

AAGCGAACACTGGCAATGACGCAGGCCGACAGTGGAGCCACTTCTCCCGTGCAGACAACA

CACAGTAGCGGCGACAGCACTGCAGAGGtggGATCGTTGATGACGGTAGTGACGGAGGAT

GACGAAGTGAAGCAGCTGCGCAATCCGGACAACGTCAGCGCGGCTGCAATCCAAGACGGG

CTGGACCTGTACCGGAAGTTGATGGCGTGCGAAGACGCGATCCTGAAGCAGCCGGTGCAG

CAAGCGATGCAGGTGCTGCGCGATAGCTTGCGGCTATACGGGCCGGAGCAACTGATCGGG

AGCTACAACGGAGGGAAGGATGCTGTCGTCATTATGCACCTCCATAGAGCGGCCGTGGCT

CAGTACTCTTTGGACAAGGGGCAGGCGTACAGGACGAAGCTGATCTACTTCGAGAACGAC

CGAGAGTTCCCCGAGGTGGAAGAGTTGGTTCTGGAGAACGTTAAGGAGTACGACCTGGAG

CTGGCCCGGTATCAGACGGGCTTCGTCGACGGGCTGAAGCAGCGCATGGAGGACGGAGGA

AACGATAAGTGCTACGGGTTCGTGTTGGGCACACGTCAGGGGGATCCCAACTGCGGAGCG

CAGACTAGCTTCACGCCCAGTAGcgACTGGATGCCTCCGTTCATGCGGGTCAACCCCATC

CTCGACTGGAACTACGGCCAGGTGTGGGGGTTCCTGCGCGTGTTCGAGCTGCCCTACTGC

CCGCTCTACGACAAGGGCTACACCTCTTTAGGTAATCTGGACAACACATTCCCCAACCCG

AACCTGAGAAGACGCTCAGAGACCAAAAACGCGAAGGATGGGGAGACTGGTGGCGGTGGc

caggaggggggagcgggcgaCGAGGAGTACCTGCCTGCGTACATGCTCTCGGATTGGTCG

CTCGAGCGAGCAGGACGCGGAAAGCGGACGATAGATACCTTGGCATGCGACCTTAGGGAC

CTGGAGAGCAAGCGGCGGAAGGTGCGAGAAGCTCGGTCTGCCGGCCTAGTGGTGATTGGA

GACGAGATTTTGAAGGGGAAGTGCCTAGACAGCAACACCGCCTTTGCCACGCAGAAACTG

TGGGAGAAGGGCATCCCGGTGAAGCGGGTAGCGGTTGTGGCTGATGACGAAGATGCCATC

ATGGACGAGGTGCGACGGCAAGTCCAAGAGTTCGACCTTGTCATCACCAGTGGCGGCATC

GGGCCTACCCACGACGACATTACCATCTACTCCGTCGCCCGAGCCCTCAACCAGAATGTT

CGAGAGAACAAGGACATGCTGAACACGCTGGCGCACAATTTTGGAGTGGACAGTCCGGAC

CAGCTCACAGACGCGCAGCAAAAGATGGCGATGCTGCCGGAGCTGTCGAGGCTGCGGGTG

GCGCCGGTGGACGGGCAGAAGGGTTGGCCCATACTCCAGACCGAGAACGTCTTCATCCTC

CCGGGGGTGCCTCAGTTCTTCCAGACCAAAATGCAGACCATCGTGGACCACTTCTTGGAT

ACGCGCTCCATGCACGTGAAGAAGATCGTTCTATCAGCAGACGAATTTTTGATCGTGGAC

TTCCTCAACGACGCTGTGGCAGCTCACCCAAAGGTGACCTTCGGCAGCTACCCCTTCTTC

TCCAACCCTGCCTACAAGACGGTGGTGACGATGGAAGGGGAGCTTCAGGAAGACGTTTTA

GCGGCAACCGAAGCCCTGGAGAAGTTGCTACCCAAGGATTTCGTCGTGAAAGTGGTAGCA

GACAACGACCTTAAGGACGTCTGA

>g564.t1

ATGAAGGCAACCTGCGTCTTCGCCCTGGCGGCCTCGGCCGCCACCGTGTCCGCCTTCATG

CCGGCGGTCCCTGCTTCCGTGTCTACGCGCGCAAGCTCTTCGCTGGCCTCTGGGTCTGCC

CAGTTCGACGAAATTTTGTCGCAGCCTGAGGCGGACGCAGACGCCCCGCCGCCGATGCCG

ATCGAGCTGACCGGGCCTCAGTACGTCAAGACGCTGCCGGGCTCGAGCGCGCCGCTCAAG

TACTTCGACCCCCTCGAGATCTCGTCCAAGTTCCGCGCCATTGACGTGAAGAAGTTCCGC

GAGAGCGAGATCAAGCACGCTCGCATTGCGATGCTCGCCTTCGCGGGCATGTTCGTCCAG

GAGCTAACCCACCCCCTGTTCATGAACGGGGGCAAGGACCTCGGGCCGGCCATCTACCAC

TTCCAGGCGGTCGAGGGCTACTTCGCGCTCATGCCCGCGATTTTGCTTATCATCGTCGGC

ATCTTGGAGGGGAACAACATTTACGTCGGGTGGGTGAAGCAGCCCTTGAAGCTCGGCGTT

GCGGACCTTAAGAAGGACTACGAACCGGGAAACTTCGGGTTTGACCCCCTCGGCCTTATG

CCCAAGGACGAGGCAGGGCGAAACGCCATGATGACCAAGGAGCTCAACAACGGGCGGCTA

GCGATGCTCGCGACGATCGGTGTATGGGCGCAGGAGCTCGTGGATGGCAAGACCATCCTG

GGCCACCTTTTCGGATAA

>g587.t1

ATGATGACTGCCCAGCGCACCCGTTCCGTGGGCTTGATGGCCCTGAGCTGCTCGATGATC

GCCTTCCTTCCGGGCGGTTTCGTCTCCGGTGCTGGCGTCGCTGGCTCGACGGGCTGCAAG

TTCCCGGAAGCGGTGGGGAACGGCGCCTGCGACATGGCGAACAACAATGCGGAGTGCGGT

TACGACGGGGGCGACTGCTGCGAGTGCACGTGCGTCGTCGACTCTCTCTACCCCTGCGAC

GACGAGCTTCTGTTCGAGTGCCTCGACCCGAACGCGCCGTGCCGCGATGGCGCGAACCTC

TTCCACGTCGTGGAGGACGAGTTCATGGCCTTCGCCGGGAGCTACGAGTTCGGTCCGTGC

ATCTTGGGCCCGATTGGGGACGGCCAGTGCGACCTGGAGAACAACCACGAAGAATGCGGT

TACGACGGCGGGGACTGCTGCGAGTGCACCTGCGTCAGCACCACGTCCCACGACTGCGGC

GACGCCGGGTTCACCTGCGTCGACCCTACCTCCGGCTGCGCTCTCGTCGAGGCCGGCGCC

GTCACCACCGTCAGCGCCTCCGCCAACGCTTACGACGCTCGCCCCGGCGCCGCCAGCATG

GGCTCCGGCTGCATGGCAGACGGGTGTTCGCCCGCTCTTACCCGCGACGGGATCACGGCC

GACGGCGAGTCGAGGTGGTCCTGCTCGCCGACCATTGTCGCCGGCGGGGGGCTGTGCGAG

ATCGAGTTCACGTTCGATACCCCTCAGGACATCAAGGACGTCCAGGTGGCGTTCTGGGAC

GGGGACGAGCTCACGCGCACGCTCGAGGCGACCATCAACGGAGAAGTGTACGGCATGTTC

GACTCGTACCCGGGATCAGTCTTCAACTCGCTCGGCATCGAGGGGAACGGTGTTCACACC

CTGGCGCTCACGTCCGTCGGCGCGACTGCCCAGGAATGGATCAGCGTACTCGAGGTGCGC

TTCATGGTCGAGGCCTAG

>g655.t1

ATGCGGCAGGTGAGAACGCTTGAGAAGGCTCTCGTTAATGCGTACGACCCGCGCAGAGGA

TTACATGCCCGCGCTCGACCGGAAATCAAATTGGACCGCGAAGATTTCATCGACCTGGCT

ACGGTCAGCAAGGAAGTGCCCGGCAAAACTCCTGCTCAGGTGCTCTCTTTCTACTACAGG

TACCTTGCGACTGCCAAGCCTCTCACGGACGTTGTTCACGGTCCTGAGGCAGCTGAAGCT

CGAAAACAGGAGCCTATCTCTCCAATTTCCTCCAGACCCGTGGGCGACGCGATCATCCCT

GGTTCTGGACGATTGACTGGGGGAAACGCCAGCACCGTTAACTCAAAGACACTCGCCAGG

CAAGTGTCGCCTGGGCTGTGCAAAGCTAGTCCAGCTAGCAATTCTGGTAATGCGAGCGAG

TCGGCGCCCAAACCTAACTCTCGAGTCATTCGGCTGTCTGCGCCAAAACCGATCGAATCC

GCTGTGGCGACTACCAAAACAAACGATACCGCCGCTGTCGATAGCGTACCGAACGACGAG

GGATCTACAAAGCGGGACCCACCCCAAAGCAACAGTGACGCCGTCGGTTCCGCTGGAGAA

ACCGTTTTCACTTCCCGCTCGCCGGCTGTGTTCACGCATGCCACAATCAAAGCTACGTCC

GGCGGTACGAACCGCTCTGCCGACGCCGATCGTGTAACGGGTAGACCGGCGAACAGGCAG

GGTGGAGGAGGTGGGGACCTTCACGGCACTAGCGCCTATTTCGCTCAGCAGCGGCGGTAC

GCAGTGCGACAGAGAGAGGTCTCGAGgaccctccctcctctccctcctgatgctgagatt

ttggagGTAGACGACAGCGACGACGCTGACGGGATCGATGAAGCTCAGCGCTCGGCGTTA

TCGGCATCCAGTGGGGCATTTGGTAACAAGGAGCCGACGCAACCATCCGGTATGGCGACG

GCTTCTCGGCACAATGCACCGGGGTCTCAACCGAGCGGAGTCCTGCGGATGGCGCCACGT

TCTATCACCCGCAACAGCAAAATAATCAGAGGGAAGATAAACAGAGCATCTGACGGCAAG

GGGTATGGCTACCGCAGGGAACCATCCGGTGTTCCGAGTAGCCAGAGAGCGCCAAAGTGG

GAGCACCCGCCGCGGCCTAGTTCAACCCGGCTTGAAGAGCAGGCGGGGCGCCTGCATGAC

CGACCCAACGACGGCCAGGCCGAGCGGAGCGGCACACCGAGCAGCGAAGTTAGTGGTCCT

GGCTACCCCGACCGGAGAACAGCCCACGTGGGAGCAGAGCCGGCGTCTAGAGACGATACG

TTCGGGTCGATGGCCCCTTGCTGGCGGTTACTAGAGCGTGCTCAGGATTACATGGACAAG

GATCAGCTTGAGTACATGCGGGGCCTCGTCATTGCCCACGTCAGGAAGCCCATGCCTAGG

GACCAGCTGCTGGAGAGGGCTGAGGGCTGTCTCCACGAACAAGACAAAATATTCACCGCC

TTTGTAAAGATGTTCGACTGCATCGACCAACCCCCCGAGGTTCCCCTTCACCCGACGAAG

AAAGCACTCGACCCTCGGCCGCACTCGACGCCGCACAGTATACTGCGTTCGCCAGACGCG

AACCCCATCCACGCTAGCCCTCACGCACGGCTCTCGCCTCGCAACCAACCGCCTCTTGCT

TCCAAGGACCAAGGAGGCGTTCCCAAATCGTTGCGTCCCATCAACGACGGTGGGGAAGGT

AGCATTGCCATGAGCACCCCGTCTGTGCCGTACCCCGGCGTGCATATGTCGGTAAGTGGT

CGAGTGGAGGCCAGACGGTCGACCCGTCCCGATGACCACAACGGTGCTGGCAGTTGGCGA

GCCAGCGGTGTTGGAAGCACTGAGAGGAACGATGCCGTGGGCATCAGAACTACCCTGGCG

GCGTATGCGGAGAGTCGGCCTGCCACGGGCCACGGTGGTGGAGCAAGGGTGCGAACCGCT

TGGTCGCAGCAGCTCTCGGGAAGGGGCGCAATCAAAACAGGCCctttcgccacagcagca

gcacctgtacCCACCGCAGCCGGaaatatgaggaagacaggcaGTAGAAGAGCGGCGTGG

GGGGCTGGCAAGGGAAGCATGTGGAGCTAG

>g845.t1

ATGAAGTTTACCCTTGCTACCTCGTGGGTTCTCGGCGTGCTTGCCACGCGTGTGCAGTCC

TTTGTATCCAGCGGAGGCTGTGCCGTCGTTCGGCATGCAGGTTCTCCCGGAATCGCATCA

AACGCGGCGTTTGCGCACCCACGTCACAGCCGATGCGCCGGCAAGACACTGACTCCTGGC

GCCGCTCGCAGTGGAACCAATCTGGGGATGTCGGCCGTCGTGATCCCGGCCTCATTCCAG

CTGCCGATAGGCATCGCCACGGTCGGCCTCGCAGTGTCCGGCGTTGGAAATGTTGGGGCG

GGGTTTCCACTGACTTTGGTGGGATTGGCGCTGGCGTTTCAGGCTACGAGGGTTTCGTTC

GAGTTCGACAATGAGTGCATGGAGGTTAAAATCGGCAACGACGCTGGTGAACTGGAGAGC

TCGGGCGAAAACGCGTTCGTCGGCGGCGAGTCGAGGTGGGCGTACGACACCTGGACCAAC

TGGGAGGTTTACCCCAGCGAGAAGCTGCCCATCCTCATGTACTTCAAGGAGACGCAGACT

AAGCCCGAGGGACAGATCcACCGAGTTGAACCTTCTCTCTCCAGCCACGTCGACCCTCGC

GAAGCTCGCGCATTGGCCACACACAATGACAACTACACCGCCGCCGCGGGGCACGACTAC

CAAACGGCAAGAGACCCGCCGCACGTCGAAACCACGCCGCAAAAGACCACGTTAGATGGG

TGCCAAAAGACGGCGAACAAATGCTAG

>g898.t1

ATGGCTAAGGTGTCCACCTTTgctatggcggcggcggcttgcgCCGGACTGAGCCAGGCT

TTCGTGACTCCGGTTGCCAACGTGGCCGGCGCCACCTCGAGCTTGCGACAGGCCTCGTCC

GCGTTGAGCATGTCGACGCCGGACAACTCCTACCACAAGAAAGATTTCTGGATCGCCCCC

TCCATCCTCTCGGCAGACATGGCGAAGCTGGGCGAGGAGGTTGACAACGCGATGGCAGCG

GGAGCGGACATCGTCCACTTCGACGTGATGGACAACCACTACGTGCCTAACCTTACCATC

GGCCCCCTGGTGTGCAAGGGTCTTCGGGACCACGGCGTGACGAACCCCATCGACGTCCAC

CTCATGGTGTCCCCCGTCGACCGAATCGTGGGAGACTTCGCGGAGGCCGGCGCGTCCTTC

ATCACCTTCCACCCCGAGGCCTCGCCGCACGTCGACCGCACGCTGCAGCTCATCAAGTCG

CACGGTTGCAAGGCTGGACTCGTGTTCAACCCCGCGACGACCCTTGACCATGCTAGGTAC

GTTCTCGACAAGGTCGACATCATCTTGCTGATGTCGGTGAACCCCGGCTTCGGCGGACAG

AGCTTCATCCCCAGCACCCTCGACAAGCTCAGGGAGGCGCGCAAGATGATCGACGAGTCT

GGCTACGACATCCGTCTCGAGGTTGACGGTGGGTGCGGAGTGGCCAACATCAAGGAAATC

GCGGAGGCGGGCGCGGACATGTTCGTAGCGGGGTCGGCCATCTTTAACAACCCCCGGACG

CAGGACAACTACAAGGAGACCATCGACAAGATGAGGGCCGAGCTGGCCTCCGCGACGATC

GCGTAG

>g904.t1

ATGATCAAGACTTGCGCTCTCCTCGCCGTGGCATCGGGTGTGGCAACTGCCAGCCCATGG

GCCGGGCAGCCCTCGGCGAACGCGGCGTTCGTGGGCGCTCCTGTGGGACGATCAAGCCCG

TGGGGTTTGGGAACGCGGTGGGTCACCAGCACGCCGACTTCGACCACGGCACGCGTCGGC

GCTGTGTCGCGATCCGCGGGCACCCGCCTGAACGCACTCAACATGAAGCTGGGCGAGGAC

GAGAAGgtgTCGGGCAGGACCGCCCTGCACACCGACGAGCAGAAGTTCGACCTCATGTTC

GAGCAGCTCAACGACCTCAAGAACGGGAAGAGCGTGATGAAGCCCATCTACAACCACGTC

AACGGCACCCTGGACACCCCCGAGGAGATCAAGCCCACCCCCATCGTCATCATCGAGGGC

CTCCACCCCTTCGTCGACGAGCGCGTGCGCGACCTGCTCGACTTCACGATCTACCTGGAC

ATCTCCGACGAGATCAAGTTCGCGTGGAAGATCCAGAGGGACATGATGGAGCGCGGACAC

TCGCTCGAGTCCATCAAGGCCTCCAtcgagGCCCGCAAGCCCGACTTCGACGCGTTCATC

GCCCCCCAGCGGAGCGAGGCGGACGTGGTGATCCAGGTGCTCCCCACCCAGCTCATCCCT

GACGACAAGGAGGGCAAGATCCTCCGCACGCGCATGGTGCAGAGGGAGGGGCACGAGTTC

TTCGACCCGGCCTTCCTCTTCGACGAGGGGTCCACCATCTCCTGGGTGCCTTGCGGGCGC

AAGCTGACGTGCTCCTTCCCCGGCATCAAGTTCGCGTACGGCCCGGACACGTACTACGAC

AACGAGGTCTCCGTCATCGAGATGGACGGGCAGTTCGACAACCTCcagGAGCTGATCTAC

GTCGAGTCGCACCTGTCGAACATCGGCACCAAGTTCTACGGCGAGCTGACCCAGAACATC

CTGAAGCAGGCGGACTCCCCCGGGTCGAACAACGGTACCGGGTTCTTGCAGACCCTGGTG

GCCCTCAAGCTTAGGGAAGTGTACGAGAGGATCACCAAGAAGACGGTGGAGGTGAAGGCA

CCCGCCATCGCACCCGCgcagtaa

>g909.t1

ATGGACGGACGTAATGTAACAGGCGCGTGGTCTAAGACGATGATCGGGTACGGGCCGGAG

GAGGAAGGTTTCGCGCTCGAGTTGACGTACAACTACGGCATCGACAGCTACAAGAACGGA

GACGACCTGCAGCACATCTGCCTCCAGCGTGACGTCGAGGCGACCAAGGCGAAGGCGCAG

GCTGAggGTTACccttcggtggcggcggcggacggcGGAGGCGTTATCATATTCGGCCCG

GACGGTTACAACTACAAGGTCGTCGCGCCCATGGAGGGGCGCAAGGAGCGCTTCGTTTCC

GTCGGCCTCCGCGTGTCAGACCTCGCGTCTTCCACCGCCTACTGGTGCGGCGTGCTCGGC

ATGTCCAAGTTCCCGCAGCCGGCCCCGTCGAGTGAGGCgggagagggtggcggcggcggt

ggtggtgcagcGGCCGCGCCTGGGCTGCGGGAAACGGTTGGCTACGGCGAGGAGCAGGTG

AAGCTCGACCTCATCCAGCTCTCCGGGGTGGCGAAGGGCACCCCGATCGACCACGGGCTT

GCCTCCGGCCGCATCGCCTTCGCGtgcgacctcgtgccccccatcCACGCCGAGGCCGTG

GCGGCGGGCAGTGGTACCGTGATAACGCCCCCGCTGACCCTGCCCACCCCGGGCAAGGCG

GACGTGGTCGTGACAATCCTGGGAGACCCTGACGGCTACGAGATCTGCTTTGTCGAGGCG

GTAGCGTTTTACCAGCTGGCCGAGCCCAAGTACGACGTGATCGACTTCGAGTCGCGAGCT

TCCAGGGGCGGGGACGGCGCGGCACCGCCGAAGAGCGAGAAGCTGCAGCACACGGCGGgg

gtgacggcggcggtgacgacgccggaggaggtggcggaggcggtggctgggggcggggat

ggggtggTCCTCCTGGACTTCGGCGCGGGGTGA

>g973.t1

ATGCGATTCGGGTACGTGCTTCTGGGGCTGGTGATGCTGGTGTCCGTGCATACTTCAAGC

GGCCAAGAATGCCGTAACGGCTTTGTGGGATTCGAAACGGGAGACATCTGCTGCGCTGAG

AgctgcgggacatgcggaggaTCCGGTTGCGGCGGGCTGGACGGCGGACCGGCGAATTGT

TGCGCAGGAGCAATCACTTCGGCTGGCATGCTATGCAGCGAAACCATGGAGGCACCGTGC

ATCATCGATGACGTAGCAATGTGCACTAACGGCCTTCCGGGAATCCAGAATGAAGACGTC

TGCTGCGATGCCGGCTGCGGCGTGTGCGGAGGAACCGGCTGCGGCGGACTACCCGGCGGA

GCGGCCAACTGCTGCATAGGCACTATCACGACGATGGACATGCTGTGCAGTGTCACAGGG

TCAGCGCCATGTGTCGTGGATGACGCGGTTCTTTGCAGCAACGGCGTGTTTGGCGTGGAG

AACGACGTTGTCTGCTGCGAGGCTCagtgcgggacatgcggaggaTTTGGTTGCTCGTCG

CGGCCCGGAGGCTCGGACGCCTGCTGCACCAGCCGGATCGAGGAAAGTGATATTTTCTGC

GACGACACCCCGGTGGCTCCGTGCATCCTCTAA

>g976.t1

ATGTGCACTAACGGCCTTCCGGGAATCCAGAATGAAGACGTCTGCTGCGATGCTGACTGC

GGCGAGTGCGGAGGAACCGGCTGCGGCGGACGACCCGGTGGAGCGGCCAACTGCTGCATA

GGCACTATCACGACGATGGACATGTTGTGCAGCGTCACAGGGTCAGCGCCATGTGTCGTC

GATGACGCGGTGCTTTGCAGCAACGGCGTGTTTGGCGTAGAGAACGACGCCGTCTGCTGC

GAGGCTCAGTGCGGAACATGCGGAGGATTTGGTTGCTCGTCGCGGCCCGGAGGCTCGGAC

GCCTGCTGCACCAGCCGGATCGAGGAAAGTGATATTTTCTGCGACGACACCCCGGTGGCT

CCGTGCATCCTCTAA

>g978.t1

ATGTTTGAGCTTTGTAGCTTACCCTCAAGCGCGCTGGCCTTAACGGCTCACGTTGTCGGC

CGGACCCATCCCCGCTGCGATGCATCCTCACGTTGTGCTAAAGGCCAAGAATGCCGTAAC

GGCTTTGTGGGATTCGAAACGGGAGATATCTGCTGCGCTGAGAGttgcgggacatgcgga

ggaaCCGGTTGCGGCGGGCTGGACGGCGGACCGGCGAATTGTTGCGCAGGAGCAATCACT

TCGGCTGGCATGCTGTGCAGCGAAACCGGCGTGGCTCCCTGCATCATCGATGACGTAGCA

ATGTGCACTAATGGCCTTCCGGGAATCCAGAATGAAGACGTCTGCTGCGATGCTGACTGC

GGCGAGTGCGGAGGAACCGGCTGCGGCGGACGACCCGGTGGAGCGGCCAACTGCTGCATA

GGCACTATCACGACGATGGACATGCTGTGCAGTGTCACAGGGTCAGCGCCATGTGTCGTC

GATGACGCGGTGCTTTGCAGCAACGGCGTGTTTGGCGTAGAGAACGACGTCGTCTGCTGC

GAGGCTCagtgcgggacatgcggaggaTTTGGTTGCTCGTCGCGGCCCGGAGGCTCGGAc

tcctgctgcaccagccggATCGAGGAAAGTGATATTTTCTGCGACGACACCCCGGTGGCT

CCGTGCATCCTCTAA

>g1050.t1

ATGAAGTCCAGAAGgtcgggtggtggcggtgaagggGATCCCAGCGATAAAAACCGCAAG

ACGGCAGAGCATAGGAGCTCAGCACGCtgttctcgtgcccagtcgCTGAAGGTGCGGTGC

TCGATACGGGCGAGTAGATGGGGGTCATCGTCGGGTGTGACGTCCGGCTCCGAGAGCGGA

ACTGGCGACGATGGGGTCGGCAGGGAAGGGTTAGTCGAAGGCGCCGGCCGTGTTTGGGTA

GGTgtcggaggcgtcggtggcgcaggttgcgcGGGGACTGTGGCTGAAGGCGGTGTCGGT

TGGAGgggcctcggccgggccgtggttgggggcgctgacgtgcgacggGTCTTCGAGAGG

AAGTGGGAGTTGGGTCTCGTCACCGAGGCCCAGCTCAAGGATCGCATTTGCCCCACAGAG

ATGATCAGGGCTGCTGTTGGCGAGTTCATCGGCACgctcctgtttttgttcagtgTCATC

ACCATCGCTCGGCTCGCCGCCGAAGGTGCCGATTCTTCGGCGACTGTGATCCTGATCGCC

TTCGGATTCGGGCTCTCCATCTTCGTCCTCGTGTACATCATGGCGGACGTCTCCGGTGCC

AACCTGAACCCTGCAGTGTCCCTGGGCCTGCTCCTCGGCAAGCGTATCTCCATCGAACGC

TTCGTCATTTACGTCATCGCTCAGATTCTCGGCGCACTCGCGGGCGCAGGACTTGCGACG

ATCTTCCTCGACAGCACCAGCGGCGGCTTCAACGCCCTCGCTGATGGCATCGATgctgaa

gacGCGTTCGCCGGTGAGGTGCTGTGCACTTTCCTTCTGGTGTTGACCGTCTTCTCGGCC

ACTGACGGACAGGTCGGCCGCAAGTTCAAGCACACGGGGGCCCTCCTTCCCCTTAGCATC

GGGATGGCCGTCTTGCTGGACCACCTTATCATGATCCCCGTGGACGGCTGCTCCATCAAC

CCGGCCCGATCGCTGGCGACCGCTATCACCAACAACAAGTGGGACGACCACTGGGTGTTC

TGGGTGGGCCCTCTTCTTGGCGGTGCCCTGGCCACCGTTGTGTGGGAAGGCATCCTTCGC

CCGGAGCAGCCCGTGTCACCTAAGGACAAGCCCATCGTCGAAGCGGGGACCGTctaa

>g1181.t1

ATGGCCTTCGTTAAGTTTCTGACTGTGGCGGCACTCGCCACGGTCTTGCTCCTGGACCTG

GCCGTACAGGCTGGCGCTTGCAGATGCTTGCGTAGGAGCCTGTGCCAGAAATACGAGGAA

GCAGACGTCGTGGTGCGTGCAACCGCGCTGTCAAGGACGGGGCCCGTGGAACGCAGGACA

ACGTACATTTTGGACCCCACAACCATCTACAAGGGGGTAGGGCTAGTCAACCCAGGCCAG

GAAATAAGCTTCGACACCAACGCCAGCGGCGCCTCGTGCGGGGTCAGCCTGAATCTGGTC

AGGGAGCACCTCATCGGTCTCCACCATAACGGCGGCGGCTCTTTCTCGGCTAGCACGTGC

GGCCTATTGAGTGATTGGGAGTTCGTCACCGAAGAGGACAACCGCTCCCTTGAGACTGGG

TGCGAGGAGGAAGACCCGTGCTATGGACAGTGTGGCGAATTTCAGGAGTGCCTGAAATAC

GACGACAATCTCCCGGACCCGTACTACTGCTCCGATGTGTGCGACCCGAGCCCGTGTGTT

GATGGACGCCCCTGCACGTTGGTGTTCCTCGGGGTGTGTGCGCGTGGCCGATGCCCGGCC

GAGGCCCACTGCTACTGA

>g1335.t1

ATGTACTCCGTGAACAATGGCGCTAACCGCTCTCCGTCAAGAGTCAAGTGCGCGAAAACC

TCGAACTTCGTGAAGCAGCAGGACACGGCGAGCATGACACCAATCAAGCCAAACAACACG

ACCAAGGCGCAGAACAAGGAGACGAGCGAGCAGAACGGGAACGGGACCGTCAAGCACACT

TTCGACAAAAAAGGAGACTGGAGCCTGATGACGGCgtcgagGTGTTGCTCCGTGGAGGTT

GGGTGTGGCACTGGGGACGTAATCTTGAGCCTAGCGCCCGATTTCAAGCAGAGCCTAGGG

CTGGACATCAACGATGGCTTTCTCGCCTACGCCTCGTCGCAGACCCCTGAGAATCTGAAG

CACAAGGTCTCCTTCACGAAGGGAAGCGCGACCGACCTGATCGACATCGTGAACTCTCAC

CCGGTGACGGAGAACTTCTCCGGGCCGACGGTGGTGACGTGCGTGAACAACACGCTCGGA

GTGTTCCCGGATGCGATCAAGCCCCGCACATACTCTCAGgtCCTCGGAGAAAGCGGCTTC

CACGTCTTGGACGTGACGGAGATCGGGGTCGGTGTCCTTTGCACGTGCTCGGGAAGCAAG

AACGTACCGCGCCCCGCCGGCACGACGACTTTCCTGCCGCACCCCGCCGCCATCTCCTCG

GACACCCTTACCGCCGAAGTCGAGGCGCAGAGCATGAACCACTACGGCGacagCTTCACG

CAGTACTTTTACCGAGCGCTGTGGGGCGGGCGCCACCAGCACCTTGGCTTGTACGAAGAC

CCCGCCACCGTGGCGCTGCCCGCGTCAAAGAGGGTCATGCAgGCGTGCGAGGACAGCACG

GTGGTGCTCTTCTCCCTCGCCCGACCGACGATGGGAACGACGGCCGTCGAGCTCGGCTCC

GGCTACGGCTCGGGCGCGCGATACCTGGCGCTCAACTTCGGAGCGACCGTGGAGTGCATC

GACCTGTCCCCCGAGGCCAACGACCTGAACCACCGCTTAACCGAGGAGGCCGGCCTATCG

AACCTCGTCAAGGTTGGGGCGCCGGCGACGTTTTTCGGCACTGGGCTCCCCGGGGGCTCG

TTCGGGTTCTGCTTCTCCCAGGACTCTTTCTGCCACGCGGGCAAGCAGACCCCGCGCGCT

CTGGAGGAGGCCGCTAGGCTGCTGGTGCCCGGGGGCATCCTGGCGTGCACGAACATTTTG

CGCACGGAGGAGGCGACCGCGGAGGAGCTGGACGAGGTCCTGGTGCGGTTGCAGATCAAC

TACCTGGAGACGCTCGAGTCGTTCGTCGAGCACGCGCGCGCGGCGGGGCTGGAGCTGGTG

GAGTCTCTGGACAAGACCACCTCGATGGCTAGGCATGTCCAGACCGTCCTCGAGGTGGCG

GACTCTCGCAAGGCGGACATGCTGAAGCACACTTCAGCCGAATACCTGGAAGAGCTTTCC

GAGGACCTGTTGAGGTGGTACTCCGCATCCAACCGGGGTGTGCTTAGGTGGGCGTTCTTC

GTCTTCCGCAAGCGCACCCCCTGA

>g1376.t1

ATGATAtccggcggcaacagcagcgggggTGAGGACCCATGCTCGTACGATGGCAAGCGT

AAGGCAGAGATGACGGCCGAGGAGCGAGCCAAGCATGACCGCGAGCGCAACCGCGTGCAC

GCTCGGAACACGCGGGCGCGGAAGAAGCAGTACATGGAGGAGCTCAAGGAGCGCGTCGAG

AACATGCACGCGCAAAGGGCGGCAGTGGAGCGCAAGAAAGAAGACGCTTCGAGAGAAGAG

CAGGAGCAGATCGCCCGCTGGTCGAACACGCTGACGAAGGTGTTGGACCTGAGATGCGAT

GGCGTTACGGACGAGGCGGTGTGGAAGGAGGTCCTGTCGGAAGACTTTCGGCTCTCTCTT

CCGATCACGCCTTACCGACCGTTCGACCCGGCCGACGTGGAAGAAGGCAGGAGGCGCGTC

ATGTACGGAGTGGAGGGCATGATCTCGGACACCGAGTCTCtcacgatgttggaggtgttg

AAGAGTGTTGCAGTGTTCGAGTGTGTTGTGGGGTATTCGGGCGTTGGAGGGGTATTTGGA

GTGTTGGGGGTGTTGGGAGTCGAGGGAGTCGAGGGGAGTGGAGGCAATTGA

>g1396.t1

ATGGTGTCTGTTGGCCTGGCGCCGCCGGACTTCAAGCTCATGAACCAGAGCAACAAGCCG

GTGTCCCTTAGCAGCTTCAAGAACAAGAAGAGCGTTGTGGTGTTCTTCTACCCCAAGGAC

AGCACCCCCGGGTGCACCAAGGAGGCGCAGACCTTCCAGGCCGACCTCGCCCAGTTCAAG

AAGCTCGGGGCGGAGGTGATCGGCATCAGCAGTGACGCTGACCACAGCGCGTTCGTGAAG

GAGAATGGCCTGAGCATGACGCTCTTGTCAGACATCGGGGGCAAGGTGCGCAAAGAGTGG

AAGGTGAAGGGCGCCCTGTTCGGCAAAATCGACGGCCGTGTCACGTACGTTTTGGACAAG

AAGGGCGTGGTGACTAGCATGTACGACAACCTCCTCGACGGCGCCTCTCACAGCAGGGAG

GCGCTCAAGGCTCTGGGAGCCTAA

>g1397.t1

ATGTTTCGCGCTCTCACTCTCGTTACAGCGCTGGCACCTGCCCATGCTTTCGTGACACCG

AGCGCTGCCCGAGCCATGGGCGTCAGCAATTATGAGCAGCAAACGGTCCGAATGTCCGCG

GAGGCACCGTTAGACAGGGCACAGTTCCTGGCTGCTTCTGCGGCTGTGGCCACGTCGTCG

GTCATGCTTCCCCTGGTAGCTCTCGCAGAGGATGAGGAGAGCCAATCAGCGAATGCCGTT

GAAGACGTGAAGCTGGTGACCACCAAGCTCGGCGGACAGCTCGAACCTTTCGCGGACGTA

TCGAAGGGCTACCGGCTTGCGAAGCCGCTGGGGTGGAACCGCTACGATGGAACTTCTGGC

GAGTACGCGGTGAAGATGGTTGACCTCGTCGACCCCACCACTGTCATCTTGCTCACCAAC

TCGCCGGTCAAGTCGGACACGCAGTTGTCTACGCTAGGTTCTCTGCGCCAGGTCGGGGAG

AAGCTGAGCAAGGGAAGGGACTTGGAGATTATCATGTCACGGGAGCGCCTCACGGAAGGC

ATCCGACTCTATGACTTTGAGTTTAAAGGTGGGGGGCGCCGCGAGCTTAAAACCCTGGCA

GTGAACAAGAACAAGCTGTGGAACCTCACCATGGGGTGCCCAGAAAAGTCGTGGAAGAAG

CAGGAGGAGGTGTTCAAGACTCTCGTCGACTCGTTCTTGCCTAGGCTTTAA

>g1493.t1

ATGATGGGTCGTGGAGCtttggcagcagcggtggcggtctTCGCTGCAGGCAGAGGCGCA

TCCGCGAGTTGCGCCTTTCGATTCGAGGGGCACCTCAATATCAGCGAGCTTCCGACATGG

ACAATACCCGCGACAGGGGCTTCCGGCTCCGGATACCGGGTGCTGAACGACGAGTACCCC

CTCGTTTTCCTCGtaTCAGAAGAGACGGAGGAAACGGCGGCGGTTAAGAACTGCACCGGA

ACTTCCTGGCGGTATCTGCAGGCCGATGGGTCCTACACAGCCGAGCTTTCCTACCCGGAC

GCGGATTCTCGCGGCGACACCTCCAAGACGACGTTCGAGGGGCTGTTTAACGCCTCCATG

GAAAACCTTGTGGTGGACTCCATCCCTGCGAGCAAGGCCGTGGCGGTTTCGGATTCGATT

TACGGGCGAACGGTCGAGGTCGTTCTCTCCTGCGAGATCGAGGACGACCAGGAGGTGGAC

GACGCAGATTCCTGCGTCACGTTCGAGGTCAACTTTACGAACACCAGCCCCGTTCCGGAA

GGTACGGCGGCCCTCTACCGCTTTGAAGGCCCCGCGGTAGCTCAAGTGGTGCTGCCCCCG

TCGGGTAACTTTTCCCCTGGGGACATCCAGCTCAACATGAACAACTCCATCGTGGAGCTT

GACGGCTCGGAGATTCTCTACGGGTGCAACGGAGACGTGgcgGAATACCTCGGAGAGGAC

GGCGAGTGGTACTTCTGGTTTACTGGCAGCAACGGCGATTTGTTCAACCTGACCCACGTG

TTCTACGCCACGGAGCAGGACCACCTCAACATCGCCACGATTCAGCGGAACTGGAGCCCC

AACATGGAGGCGGCGCTGTACAGCCGAGTGGTGAGGCTGGAGATGTACTGCAACAACATC

GAGGAACGCGAGTACGGGTACATCTTCTTCCGCGTGGGAGACGAGGAGGACTCGTTCGGC

CTCGAGTGCGACGAAGTCCCCTGCCTctgcgaggaggagggggacaaCTGCGATAGGTCG

GGAGCGCAGCGCCGCGCGGGAATGGGCAGCCAGGGCGTGCAGCAGTGGGTCGTGGCAATG

TGCACCCTGGTAGCGATGGGAGCCGTCTCCACGTTCATGAGCAAGTCGCCATCGTAG

>g1591.t1

ATGATGGTGGAACCGGAGGCTTCCGCATCTGTCGGGTCGAGCGCTGGGCGGGGGCAGTTC

ATAGCGTCGGTGGCGGCGTCAATCGGTCTCGCTCTGTCCGTCGGGCCTGACGCTGCGACT

GCGGCTGTtaatttcgagacggagcggtacGGGGACAAGGAGCTGAAGATCGCCACGGTT

AACAAGCTTCGGCAGCAGATTAGAAACTCGCTGCTAGAAGACCCGAAGCTGGCGGCAGAC

ATGGTGAAGCTGGCCATCGCGGACGCTCTTGGCTTCGACGCCACCACGCAGACGGGCGGA

CCCGATGGGTCAGTCTTGCTCGAGATGgacagGGATCTTGTGAAGGGGCTCAAGCCCGCG

GTAGACAACGCCCTGAAGATCAAGAAGAACCTGCAGCGCACGAACGAGATGACTCTGGCG

GATGTTGTGGCGATGGGAGGGGCAGAGGCCATCCACGCCTGCGGGGGACCCCCGATCCTC

GTGCAGCTAGGAAGGTACGACGAAAAGAAGCAGGGCAACCCCTCCCCAGACATCCCTGGT

TACAACTTCGATGCACCCACGGGGGCAGGCGTAAAGGCCGCTTTCAAGCGCGCAGGTCTC

GGGCCCAGGGAGATGGTGTTGCTCCTCGGGGCAATCGGAAGCGTCTCGGAAGCGACTGCG

GCTCttggagacggaggGGGAAACGGAGAGGAGGACGATCTGGAAGACCTGGCGTGGCAA

AACAGCATCCCCAACACCTTCGGCAAGGAATCGGACAAGCTCGGAAGGCCGCTGTCTAAT

GGCTTCGGTCCGGGTTTCCTACAGAGGGTGGCTGCTGGCGGTAAAGACCTCGCGGGGACG

GGGGCCGTGGGAAAGGCTCTCCTCGAGGACGAGGAAATCAAGAGCTACGTTAGAAAATAC

GCGGGGAATTCGAAGGCATTCACCCAAGACCTGTCGGAGGCGTACACGAAGATGACCCTG

CTAGGGGAGCGATACGAGACGCGCAACGCCCTCTAG

>g1594.t1

ATGAAGACTCCAGCCGCAGCAAccacgtctctgctgctgctgggaggcTCTACAGGAGCG

TCTGCCTTCGTGCATAGCGCTGCTGGGCGCTTCTGCTCGGTGACGCAGGCGTCGGCGACT

TCGAAGGCCGGGAGGCAAGTACCATCGACGTCGCCGTCGCTGGAATGCTCCTTGTTTCGa

cggggagagaggaggagcgAGGCGATGAGAGGAACGGTTGTTCAAGGGCAGAGGAGTACG

AAGTCGCAGGCGTTACGGATGGGGTTGTTGGACGACCTGTTGAAGTCGAAGGGAGACCCG

GTCATGGCGGCCAACGCCGACGTCCTGTTGTCCTACGAATCTCGCGTCAAAAAAATCAAT

GACCTAGAGGACGAGATGGAGGGCATGTCCGACGATCAGCTCAGGGCGAAAACGCAGGAG

CTCCGCGAAAGGCTTTCAGGTGGGGCGGGTTTCGACGATGTGTTGGAGGAGGCATTTGCT

GTAGTTCGAGAGGCGGCCTGGAGAGTCTTGGAACTACGTCACTACGATGTTCAGCTTTTG

GGCGGGCTAGCCCTGCACGAGGGCAAGCTCGCGGAGATGGCCACCGGGGAAGGAAAAACG

CTCGTGGCCACGCTGCCTTGCTACCTCAACGCCCTGGCCGGTAAGGGCACCGTGCTGGTC

GTCACGGCCAACGACTACCTCGCGCGAAGGGACGCCGAAACCATGGGACAGGTTCACCGT

TTTTTGGGACTGAGCGTGGGGCTGATCCAGTCGAACATGCCCGAAGCACAGAGGAAGGAG

GCGTACTCCGCGGACGTTACGTACGCGACCAATCAGGAGCTCGGGTTCGACTACCTCCGG

GACCACCTAACGGTCACCAAAGACGGAGCGGTCCAAGCAAAGGAGTACTACTTCTGCCTT

GTTGACGAAGCTGACTCCATCCTCATCGACGAGGCTAGGACGCCGCTCATCATCTCTAGG

AGCGTCGATGCGCCCGCCCAAAAGTTCGCCACCTCCTGGAAGATCGCAGGCGTTCTCGAG

AAGGGGCTGCACTACACCGTATCAGAGAAAGACCAGTCGGTCGTGTTGACGGACAAGGGA

TACGACGACTGTGACCGCATCTTGGGGAAGTCCATATTTGACCCCAGAGACCCGTGGGCG

CCGTACGTGATCAACTCCATCAAGGCCAAGGAACTCTTCACCAAGGACAAGGAGTACATC

GTTCGTGACAAGGACGTGCTCATCGTGGACACCTTCAGCGGCAGAGTGCTCGAGGGGAGG

AGGTACAGCGACGGGCTTCACCAGTGCATCGAGGCCAAAGAAGGCATTACCGTGTCGAAG

CAGAGTCAGGTGATGGCGCAGGTGACGTACCAGGCCCTGTTCCGGAGCTTCCCCAAGCTG

TGCGGGATGACGGGTACCGCCATGACCGACGCAAACGAGCTCGGTTCTACCTACGGCCTG

GAAGTGGTTCAGATACCGACAGCCCTCCCGATCGCGAGGAGGGACTACCCCGATGTGGTC

TTCCGCAACCGAGTGGGCGCGAACGCCGCCATGCTTACGGAGGTCGAGAGGCTGCACAAG

GACGGACGCCCGGTGCTCATCGGAACGACCAATGTCCGGATGTCGGACCAGACCGCTAAG

GACCTTGAGGAGAGGGGGGTGCCTTGCCAGACGCTTAACGCCAACCCAGACTTGGTGGAG

CGCGAGAGCGAGATCGTGGGCCAGGCGGGGCGGCTGGGGGTGGTGACCGTGGCCACAAAC

ATGGCGGGGAGGGGAACGGACATCCTGTTGGGTGGAAATGCCGCAGTGATGGCGAGGATT

AGAGTGAGGGACGCCCTGGCCAAGGAGCTTCTTGCAGAGGAGGATTTGGCGGTGGTGCCG

AAGGTGGGAGAATCGTTTTTCCCCTGTGAACTCCCTGAAGAAGCGACTCTGGCTCTAGCG

GAGGCCGTCTCTGAGTGCGCCAAAGACTCTTCTCTCCCCAAAAGCGACGAAGGGAAGATG

GGGAGGGAAGCGCTGGAGGAGTTGGTTTCGGCGGCCTGTGAGGCGGGGCCTGTGGAGGGT

AACGGGGTTGTCGCGGTCAGGGAGGCTGCGCAGGCGGTCAAGAAGTGCTTCAGTGACGGC

TTGGCCGAGGAGAAAGAGAAGGTGATCGACCTTGGCGGGCTTTACGTGGTGGGCACGGCT

CGGCACGAGAGCCGCCGCGTGGACAACCAGCTGAGGGGCAGGGCGGGGAGGCAGGGCGAC

CCCGGGGCAACTCGCTTCTTCCTTTCCCTCGATGACGACATCTTCAGGGTGTTTGGAGGG

GACCAGGTGACGAAGATCATGGACAGCTTCCGGCTGAGCGACGACATCCCCATCGAAAAC

AAGCAGGTGTCTGCTACCTTGGATAAGGTGCAGAGGGCAACGGAGGACTACTTCGCCGGC

ATTCGAAGAACGGTGTTCTCTTTCGATGAGGTGATGAACGACCAGCGGCTAGCGCTGTAC

CAAGCAAGAGACTCCGTGATCGATCAAGACAAGGACGGATTGAGAGAGCTTGCCCTGGAG

TACTCGGCCAAGACTTGCACCGAGATCGTTCAAGGGAACGCCGGTGAGGATGGGTCACCC

AAAACCGTTCTGTCTGCGAAGCTGCAGCAGTTTTTCCCGATGGCGGAGGCGTCGGCTATG

TCGGAGGAGTCTCTCAACGGGGCTTTCGGGTCAGGGGGCAAGGAGGGTCTGCTGAAGTAC

TGCCTGGACCAGGCGGCAGCCGCCACGGAGAAGAAGCTGTCGGACATCGACTCGATCAGG

ACGGGGCTAGCCAGCGAGTCGACCCGGTTCTTGACGTTGACTCAGATGGACGACCTTTGG

TGCAGCCACCTGGAAAACATGAATCTGCTGAAGGAAAGCGTGTCCATGGAGGTGTTCAGG

GGAAGGAACCCGCTCGAAGAGTTCGGCGTGCAGGGTAAGGACATGTTCCGCGATCTCTTG

GACAACGTACGAAGAAACACCGTCTACTCCCTTCAGATGTATAACCCCAGCCCTAAGACT

GCCGAATAG

>g1777.t1

ATGCAATTCGGGTACGTTCTTCTGGGGCTGGTGGCGCTGACGTCCGTGCATATTTCGAGC

GGCCAAGTATGCCGTAACGGCTTTGTGGGATTCGAAACCGGAGACGCCTGCTGCGCTGAG

AGCTGTGGGACATGTGGAGGAGGCGGTTGCGCAAGACTGGACGGCGGACCGGGCAATTGC

TGCGTAGGAGCAATTACTGCAGCTGGCATGCTGTGCAGCGAAACCGACGCGCCTCCCTGC

ATCATCGATGACGTAGCAATGTGTACTAACGGCCTTCCCGGAATCCAGAATGAAGACGTC

TGCTGCGATGCTGACTGCGGCGTGTGCGGAGGAACCGGCTGCGGCGGACTACCCGGCGGA

GCGGAAAACTGCTGCATAGGCACTATCACGAGGATGGACATGGCGTGCAGTGCCACGGGA

TCAGCGCCATGCGTCGTCGATGACGCGGTGCTTTGCAGCAACGGCGTGTTTGGCATAGAG

AACGACGGCGTTTGCTGCGAGGCTCGgtgcgggacatgcggaggaTTTGGTTGCTCGTCG

CGGCCCGGAGGATCGGACTCCTGCTGCACCGGCCGGATCGATGATAGTGAAGTCTTCTGC

AACGACACCCCGGTGGCGCCGTGCATCGTCTAA

>g1785.t1

ATGCGATTCGCATACGTGCTTCTCGGACTGGTGACGCTGGCGTCCGTGCATACTTCGAGC

GGCCAAAGCACATGCGATAACGGCTTGACTGGAATCCAGACCGAAGACATCTGCTGCGCT

GAGAgctgcgggacatgcggaggaaCCGGTTGCGGTGCGCGGGACGGCGGAGCGGCTGAT

TGCTGCATAGGCACAATCACGAGGGGTGGCATGCTGTGCAGCGaaaccatggaggcgccg

tgcatcatcgacgATGTAGAAATGTGCAGCAACGGCCTTACAGGAATCCAGAATGGCGAC

GTCTGCTGCGATGTTGGCTGTGGCGAGTGCGGGGGAGGCGGTTGCGCCGGGCGGCCGGGT

GGTTCGGACAACTGCTGCACCAGCCGGATCAGGGAGTTTGACATGATGTGCAGCGACACC

GAGGCCGCGCCCTGCATCATCGATAACGACTCCATGTGCGACAACGGCCTTCCTGGAGTA

CAGAGTGCCGACGTCTGCTGCGAAGCATCCTGCGGGACATGCGGTGGAGGCGGTTGCGCC

AGACGACCGGGCGGTGCGTTGGCCTGCTGCTCCAGCCGGATCAGGGAAAGTGGAGATATG

TGCGGcaccaccatggaggcgccgtgcatcatcgatgCTCCGACACCGGCGCCGACGGAA

ATGCCCACGCCAATGCCCACGCTAGCGCCGACGGACATGCCCACGCCAACGCCGACGGAA

ATGCCAGTAACTATGTGCAGCAACGGTCTCGCTGGTGTTCAAACGGGCACTATCTGCTGC

GAGGCGCAGTGCGGAGGATGCGGAGGAGCCGGTTGCGGCGGGCTACCCGGCGGACAGGAT

TCCTGCTGCACAGGACCAATCGGGTCCAGTGGCATGATGTGCAGTGTCACTCAggcggcg

ccgtgcatcgtcgACAATTAG

>g1786.t1

ATGCGGTTCGGGTACGTGCTGCTCGGGCTGGCGACGCTGGCGGCCGTGCATACGTCGAGT

GGCCAAACCACGTGCGATAACGGCTTGATTGGAATCCAGACCGAAGACATCTGCTGCCCC

TTGAGCTGTGGCACATGCGGAGGAAGCGGTTGCGGTGCGCGGGACGGCGGAGCGTCAAGT

TGCTGCATAGGCACCATCACGAGGGGTGGCATGCTGTGCAGCGAGACCATGGCTCCGCCG

TGCATCGTCGACGATGTAGCCATGTGCGACAACGGCATTCCTGGAGTACAGGATGACAAC

ATCTGCTGCGCTGCTtcctgcgggacatgcggaggaTTCGGTTGCGCCAGACGACCTGGC

GGTTCGTCCGCCTGCTGCACCAGCCGGGTCGAGGAAAGTGGCGATATGTGCGGcaccacc

atggcggcaccgtgcATCATCGATGCTCCGACACCGGCGCCAACGGCACCGCCCACACCG

GAGCCCACGGCACCGCCCACACCGGAGCCAACGGCACCGCCCACACCGGAGCCAACGGCA

CCGCCCACCCCGGAGCCAACGGCACCGCCCACGCCCATGCCCACGCCAACGCCAATGGCC

ATGCCAGTAAATATGTGCAGCAACGGTTTGGCGGGCTTTCAAACGGGCACTGTCTGCTGC

GAGGCCCAGTGCGGAGCATGCGGAGGAGCCGGTTGCGGCGGGCTACCCGGCGGACAGGGT

TCCTGCTGCGTAGGCCCAATCGCGTCCAGTGGCGCCATGTGCAGCGTGACTATGGCGGCG

CCGTGCGTCGTCGACAATGGTTCCGAGATTGATGGTCTGGGAGACTCCTACGGTACTCCC

AACAGTGAACACATTCATtgGCTCGGGGTCTCCGATCGATGCCCCCTCTGCAAGGTCAAA

GTATCGCGGGTGCTATTCGACATAAAATCGCCCAAGGAATACAAGGTGCACCTGCACGCG

CGAGGCAAGAGCAACAagagtaacagcaacagcagtgacggcggcggggggtcgTCGCGT

GCAGCGCcaccttcttcctcttcctcctcgtccctGTGGTCCCCGTCTCCATCAGTCTCA

GCCTcgtcctcggcggcggcggtggtggtaggtgCTTCGGCAAGCGCGTCCTCTAGGGAG

CACCGCGATGGCGGATCGGGCGGggcaggcgggaggggggcggagttCAGGTCGACGGTG

TATAGGCGGGGTTTGGCCGCGGAGCCGCCTGAACCGAAGGCCAGGCGATCGAAATCCGTG

GCTCAGCTCCGACCGTGGATTGCCAGAGAGGTTCACGCGGCAGCGGGTGGCCGTAGAGGG

GCGGGGGTAAGGGACACGCGATTGGTGGCGGACGTTGTGGAGGCGTTGCTGAAGGACAAC

GACGTGGACGAACAGGAGGGCTACTGCACTGTCAAGAAGGAGGATTTTCTGTTCGAGTAC

ACCGGACCGTTCTTGCACGAGCTTTGGTGCTTCAGGTGGAGCAAGGCGGGTATATCCGCC

TACGACCGAGACGCCGTGTACCACCCGAGGCGTCAGGGGTGCTAG

>g2133.t1

ATGAGCATGCTGGCCTTCCGCGTGCCGGCCAGCTGTCGTTTGGCATTGACCCCATTGCGC

ACGTTTGCGTCCAGCACGCCGACCAAGAAGAAGACAATCCAGCAGCTGGCCAAGGAAATA

AGCCTCAAGAATGTTCCGGTTTTAGTCAGGGCTGACCTCAACTTGCCTCGGAGCAAAGAA

GACGGCAGTATTGTCGACGACACTCGAGCACGCGCGGTCTTGCCGACTGTCAAGTTCTTG

CTCAAGGAAGGTGCTAAGGTCATCCTATGCAGCCATGCTGGTCGACCCAAGGGCGAGGTG

GTGGAGTCTATGAGGATAGGGAACATTGGAGGTTGTCTAGGAGAACTGCTGGATGAACCG

GTGGCGTGTCCCGCTGACTGTATCGGGGAAAAAGTGGAAGCGCAGGTTACAGCAGCGCAG

TGCGTGCTTTTACTGGAAAACGTTCGGTTCCACAAAGGGGAGACAAAGAACGACCCTGCT

TTCGCAGAGCAGTTGGCGGCACACGCGAAAATCTTCGTCAATGATGCATTTGGAGCTGCT

CATCGCTCCCATGCTTCCACGGTGGGCGTGACCAAGTTCATGGACCACAGCGTAGCAGGG

TTTCTGATGGAGAAGGAGCTTGATTATCTCAAGGGCGCTATGGACAGTCCTTCCCGCCCG

TTTATCGCTATGATCGGGGGAGCCAAGGTCTCCACGAAGATCCCGGTTCTGGAATCATTA

CTGGACAAGTGCGACAGCATTCTTCTCGGCGGCGGGATGATTTTCACCTTCTATCGCGCT

CTTGGCATGTCAACTGGTGCTTCCCTGGTCGAGGAAGACTTCGTCGATATGGCTCGAGGA

TTGATCAAAAAGGCCGAGGCGAAGGGAGTGAAGCTCTTGCTTCCAAAGGACGTCGTCGTC

GCAGACAAGTTCGCCGCAGATGCTGCCACGAGAACGGTTTCTGCGAGCGAAATCCCTGAC

GGTTGGATGGGCCTCGACATTGGCCCAGACGCCGTGGACGAGTTCAAGCAGGAGATATCG

CTGGCTGACACCATCGTCTGGAATGGACCGCTCGGGGTCTTCGAATGGGATGCGTTCGCG

AACGGAACGAACGAACTTGCGCACGCCTTGGCTGCTCGCACGGCTGCTGGTGGCACCACT

ATCGTGGGAGGTGGCGATTGTGTTGCTGCGGTAGAAAAAGCCGGCGTTGCTAGTCAGATC

AGTCACATTTCAACAGGCGGCGGGGCTAGTTTAGAACTTCTTGAGGGCAAAGAATTGCCT

GGTATCGCAGCCCTAGACGACGCATAA

>g2551.t1

ATGCAAATCGGGTACGTGCTTCTGGGGCTGGTGACGCTGACGTCCGTGCATATTTCGAGC

GGCCAAGAATGCAGGAACGGCTTAGTGGGATTCGAGAATGGAAACATCTGCTGTGCTGAG

AgctgcgggacatgcggaggaTCCGGTTGTGGTGGACTGGACGGCGGACCGGACAACTGT

TGCACAGGGGCAATCACTGCGGCTGGCATGGTGTGCAGCGAAACCGGCGCGGCACCGTGC

ATCATCGACGACGCAGCCCCAGAATGCCGTAACGGCTTTGTGGGATTCGAAACGGGAGAC

ATCTGCTGCGCTGAGAGttgcgggacatgcggaggaGCCGGTTGTGGTGGACTGGACGGC

GGACCGCCGAATTGTTGCGCAGGAGCAATCACTTCGGCTGGAATGCTGTGCAGCGAAACC

GGCGAGGCACCGTGCATCATCGATGACGTAGCAATGTGCACTAACGGCCTTCCGGGAATC

CAGAATGAAGACGCCTGCTGCGATACCGACTGCGGCGTGTGCGGAGGAACCGGCTGCGGC

GGACTCCCCGGCGGAGCGGAAAACTGCTGCATAGGCACCATCACGACCATGGACATGTTG

TGCAGTGCGACAGGGTCAGCGCCATGCATCGTCGATGACGCGGTGCTTTGCAGCAACGGC

GTGTTTGGCATAGAGAACGACGGCGTCTGCTGCGAGGCTCGgtgcgggacatgcggagga

TTTGGTTGCTCACTGCAGCCCGGCGGCTCGGAATCATGCTGCATCGGCATGGTCGAGGAA

AGTGGAATTTTCTGCGACGACACCCCGGTGGCGCCGTGCATCGTCTAA

>g2642.t1

ATGGCGAACATCATGGGACCCCTCGATGCGGCGACGGCTGCTGTCGTCCGCGACTACCAC

GTCGAGCCTCGCATGGAGTACCGGACGATCTCCGGCGTGAACGGCCCGCTCGTGATCTTG

GAGAACGTGAAGCTGCCCAAGTACGCTGAGATCGTGGACCTCACGCTGTCGACGGGAGAG

AAGCGCCAgggccagATCCTGGAGGTGTTCGGCAGCCGTGCGGTCGTACAGGTGTTCGAG

GGAACCTCTGGTATCGACAACAGGAACACCCGCGTCTCCTTCACCGGCGATGTGTTGAAG

ATGGCCATCTCAGAGGAGATGCTTGGGAGGGCCTTCAACGGGTCCGGAAAGTGCATCGAC

AACGCACCCCCCGTACTGGCGGAAGACTACCTGGACATCATGGGACAGCCCATCAACCCT

TCCTGCCGAGACTACCCCAAGGCCATGATCCAGACGGGTATCTCTGCGATTGACGTGATG

AACTCCGTCGCCCGTGGGCAGAAGATCCCCCTCTTCTCGGCCGCCGGTCTCCCTCACAAC

GAAGTAGCCGCGCAGATTTGCCGAcagGCGTCTCTGGTGAAGCAGAAGGATGTGTTCGAC

AGCCACGACGACAACTTCGCCATCGTCTTCGGAGCCATGGGTGTCAACATGGAAACCGCG

CGATTCTTCCGCAACGACTTCGAGGAGAGCGGTGCGATGCAGCGGACGGCGCTGTTCCTC

AACCTCGCGAACGATCCCACGATCGAGCGCATCATCACCCCGCGTTTGACCCTGACCACG

GCCGAGTACCTGGCTTACGAGCGCGACCTCCACGTGCTTGTCATCCTTACCGACATGTCT

TCGTACGCCGACGCCCTTCGCGAgGTTTCCGCCGCCCGTGAAGAAGTCCCCGGTCGCCGT

GGTTACCCCGGTTACATGTACACCGATTTGTCGACCATTTACGAGCGGGCCGGGCGTGTC

AACGGCCGCAACGGGTCGATCACTCAGCTGCCCATCCTGACGATGCCCAACGACGACATC

ACGCACCCTATCCCCGACCTGACGGGTTACATCACGGAGGGGCAGATCTACCTGGACAGG

CAGCTGCACACGAAGGAGATCTTCCCCCCCATCAACGTGCTCCCCTCGCTGTCGCGACTG

ATGAAATCGGCTATCGGGGAGGGCATGACCCGCGAGGACCACTGGAACGTTTCCAACCAG

CTGTACGCGATGTACGCTCAGGGCAAGGACGTGATGGCGATGAAGGCAGTGGTGGGTGAG

GAGGCGCTCAGCCTGGAGGACCACCTGTTCCTCAACTTCGTCGAGAAGTTCGAGGCAAAA

TTCGTGTCCCAGGGCCCGTACCAGGCACGTACGATTTTCGAGTCGCTCGACCTGGCGTGG

TCGCTCCTGCGGTCTTTCCCCAAAGAGCTGCTCAAGAAGATCACCAAGAAGCAGTTGGAG

AGCTGGTACTCTCGCCGTTCCGCGGCCAAGCAGGCCGGCGCGTCCGACCTTGTGTCCGCG

GGGGACTCCAAGGACGACGAGTAG

>g3560.t1

ATGAAGACCGTCGAAGCCACGGTTACGCTCATGGCTCTCGCCGCCCTTGCACCGGCCATG

GCGTTTGCTCCCTCAGCCGCGACCGGGACGGCGATTCGACGCAGCCGCGCGTTCTCGCAG

AACGCTCAGACGACTTGCGCCCCCCGGGCGGCAGAGAAATCCTCTTCAACGGGTTTCCAG

ACCGTCGGAGGCGTTATGGGATCGTCCGCCCTGTTCGGGACGGGCGCCTCGTACACGGAG

GTGATCGCGGAGTCCGAGGGGGAggaaggcggtggcggcggagacggcgacgaggagggg

agcggggagaaGAAGGTTAAGAAGAGCAAGGGAGGCGGGGACAAGGAAaaggttaAGCTC

GACGCCGAGGTAACGTTTTTTGAGGGCGGTCCGGACCCTTCCGAGCTGGCGGCCCCTGCG

GTGTCCATCCTGACGGTCATCGGGCTCGTCCCCTTCTCGGCAGCGGTGGCGCGCCAGGTC

TGGGTGAAGTACACCCTCACCTCGCGACGCATCAAGATTGTGAGCGGATGGGGTGGGAAG

GACACGACCGAGGTCGTTTACCCGGACATCGTGGACATGAAGTACGTGTGGCGTTTCTTC

GGGAGGTGCGGAGACCTGGTGCTTACGTTGCGGGATGGGTCTAAGCTGGAGGTACGGTCG

CTGCCGGACTTCGAGCGCAACTACAACTACATCTTCGAACGCACTTCGgccagctgccaG

GCCAACAGCGAGGAAGTCAAACCGGTTTAA

>g3570.t1

ATGAAGTTCACtatcgctgctgttggtgtgatCGCCGCCGCCTCGTGCGCGAGCGCGTTC

GTGGTGCCCACCACCCCCGTGGGCGGCCTCACCCAGACCGCTCGCTCTACCGTGACCCGC

GTCAACGCCGGTGGCAAGTACGACGGCAAGCTGTGGGACGACGCCGCCAAGAACGACGTC

AAGTCCCTCTACAACGCGGACGAGCCCAGGAGCGAGACCAACTTCGACCCCTTTGAGAAG

GACGCCACCGGCAACAAGTGCGACGCCAGCGGCTGGTACCCGGGAGAGTCCAGGTACAAG

GACCCCATCCGCCCTAACGTCAGCTTCGCGGAGTACCTTAAGCAGAAGGCCGAGAAGGAG

GCCGCTGAGGGAAGCGCATAA

>g3584.t1

ATGATGTGGTCGGGGGTCTTGGCGAGCCTGCTCTTCGTCGGAGCCGAATCCAGCAGCTCA

AAGCCGGACGTGATTCAGCTGCCCGTCGGGTTCTTTCCGGAGGGAATCACGATTGCCAAG

AAATCCACCGCCTACGTTGGCTCCCTCGTCGATGGGTCGATTTGGAAGGGAGACATTGGC

ACTGGAGAAGGAGGCGTCGTCATTTCGGGCGTCGGTGGCCCATCGATCGGCCTGGATCAC

GACGGCCGGTCGGGCTATCTCTTCGTTGCTGGTGGGAGTGCAGCTCGAgtctacgacgac

gacgactacagCCTGGTCGCAGAGTTCGTGTTTGCCGGCGAGGGTGAGATATCGCTCATA

AACGACGTGTACGTCACCGAGACCGCGGCATACTTCACAGACTCCTTCCAGAACAAGCTT

TACCAGGTCCCGCTCGACGCGGATAGTGGGGAGCTGGTGGATGTGGTGGTCACCGCCGCG

AACACCATCACCCTTAGTGAGAACTTCCGCATCGTAGAGGGGGCGGTCAACGCGAATGGC

ATCGTGGCAAGCGACGACGGCAGCATCCTCATCGTGGCGAACACGAATGCCGGCCGAATC

TTCAACGTCGATCCCACAACCGGCACCGCTTCCCTGTTGGACCTGGGAGGCGCTCTTGTC

TACGGGGACGGTTTGGTCCTGCGCGACAACACGCTTTGGGTTGTCGACAATGGTCGAAAT

GTCGGCGGGATGCAGCAAATCACGGAGATCTCCCTCTCGGCGGACCTGACGTGCGGCGCT

GTTGTGCCTCGCGCTCTGACGAACGACCTGTTCGACACCCCAACTACTGCGGCACGAAAG

GGCAACGCGCTGTACGTCGTCAACGCCAAGTTCGGCGTTGCCCCGGAAGACGTACCGACG

ACCGAGTACGAGATTATTCGGGTGGATCGGGACAGCGTGGGGTACATTTGCCATTCGGCT

TAG

>g3712.t1

ATGTCTTCCTTCCGTGCTGGTCTGTGTGTGGTCGCGGCCATTGTGCTGTCATGCACTTCC

TCAACCGCTTTCGTCGCGCCTACTGCGTCTCGGGCGAGCGCAATGACTACTACGAGGTCG

TCGGCGTTGTCCCGATCTGCGGTGTCCTCCAGGTCAGCTGTGAGGCAATCTCGGGCCATG

CGCATGGAGGAGGGCAAAGGGTACTTGGACGACCCGCTCCTCAAGCCCCTGGAGGACGAT

GACTACGTTGCGTTCGGCCTCGCCTGCTGCTTTGTCATGAACGACAACCTCAAGCTCGAC

GAGTACTACGTGTACGAGCCCCTCACGGCGGCGACGTTGGAGACCATCGCCAGCTCGGCC

AGCCTGGAGACGTCGTACGTGCGCGTGACCGCCTTCAAGGCGAAGGACATCTTCATCGGC

CCCCCCTCGAGGCCTACCGGCATCCATGTGGAGAAGCTGAAGATCCTCGACGGCGAGGAG

GACGCGCACATCTGCGAGAACGTGGTGGAGAGGACGCTGGCGGCGGCACGTACCTACAAG

AGGCGGGTTGAGGCACAGATGTGCGGTTTCGGGGACGTACTGGACGGTTTCAACTTCAGC

GTCGAGCGTAAGCGCATCCTCAACCACAAGTACGAGCCCAGCTTCGACGACAACGTCAAG

CAGGACAAGAGCATTGACGTCTACGGGCGGGAGGAGGACGAGGCCGTTAGCAAGCAGGTC

GCGGACCTCGAGAGCATCTAA

>g3756.t1

ATGCTGTCTTCTCTCAGGGTCTCCGCCAGAcgagctgtcgctgctgctcccTCGGCGGCG

GGCAAACTCCGACagggtgctgctgcttccgttCCTGGCATCGAACAAACCGCGTGCCGA

GCTTTTGCGACGGTACCTGGGCCCCGCCTCTTCGACTACGAGACGGTCACCTCGGTGCTC

AAGCAGAGCGACGCAGTAGAGGCGGTAGAAGCCGCGTTTGGCATGCTCGCCAAGGGAAAG

GTGGATGTGCCCATCCCCATGCACATCGGCATTGACGAGTCCGCTAACGCTGGGCCGGGG

GATTGCCACATCAAGGGCGGGTACATCTCTGGCACGTCGACTTGGACCGTCAAGCTCGCC

AACGTGTCCTTCTACAAGAACATCGAGAAGGGTCTCCCGGTCGGATCCGGCATCTTCGTT

GTCTGCGACGCCACCAATGGTGCCCCTCTCGCCATCTTCCAGGAGAACCGTTTCCTCACC

GACCTCCGCACCGGCGCGGCGGGCGCGGTGTCCGTCAAGTACTTCGCTGCGCGGCACCAC

AAGAAGGTCGGCTTTATCGGGGCCGGCGTCATCGCCAAGGCCATGGCCCGAGGCTCGGCC

TGCGTGCACGAGTTCGACGAGGGGTACGCGTACGGCCTGGACACCAAGTCCACGCAGAAG

TTCGCGGACGAGCTCGAGGCGGAGCTTGGGTACAAGGTGCACGTCTGCGCCACCGCGGAA

GAGGCCGTGCGCAACTCCGACGTGGTGTTCACCCAGACCCCGGGGAGCGAGACGGTGCTC

GAGCTCGACTGGCTCCGACCGCACGCGACCATCATCGCGAGCGGGTCCGATCAGCCGACG

AAGAACGAGATTCCCGCGGAGGTCCAGAAGGCCAGCAAGATGGTCTGCGACCTCGTGCGC

CAGTGCAAGCAGGTCGGGGAGCTGCGCAGCGCCATCAAGGCCGGTGTGATGACGGAGGAG

GACGTGCACGCGGAGATCGGGGACGTGGTGAACGGAGACAAGCcggggcgggtgggggag

gagCTCATCCTCGTTGACCTCACGGGCACCGGCGCGCAGGATGCCGCCATCGGGCAAGTG

GCGTGGGACATTCTGTCCAAGCAGTAA

>g3775.t1

ATGGTTGGCAAGCAGGTCCTAGCGGGCCTATCGGACGTCCAGCTCAAGTTCATGAACTCG

GCTGGCCCGGAGGAGAGGGACAGGTTACTTCTTCAGTGCTTGGAGTACGCGAACGGAGAC

ATGCAACAGGCGACGAAGGTCTGCAGCACTTTCTGCAACTTCCGGCTGACGCAAGGCTGG

GGCCTGGTGCTGTCTGCCGCCGAGCTGGAGGGGCCTTTGAGAAGCAGGGTCCACACCTTG

ACCGAACAGACGGACAGGTTCGGGCGAGGCATCATTACCTTTTCTCCGGGCAGACTGGAC

ATGAGAAACGCGTCGCCCGAGGCGTACCACAAGATGCTGTGCTACGTTCTGCAAGAGGTT

TTGAAGAAGCAGGACTTCCAGAAGAAGGGTATCGTGCTCTTGGTGGATGCGAGGGGCGTG

GGCTTCGGTCTCCTGAGGCATTTTGTCTTGGCCGACTACAAGCGGGGCTTGGGGATGCTG

AGCGGGGCGTTTCCCGCGAAGTTGAAAGCCATACGCATCCTCCACCCCAACCGCGCTCTT

GCCTTGGCGCTGTCCATCGCCCTGCCTCTGCTGTCGCCCAAGATGAGAGCCAGGGTGGAG

GTTGTGGCGGGCGACAGGGCGAGGGACGGTAGCTTCTCCAGAAACCTGGCAGATCCTTCT

GCGTTACCGATGGAGCTCAGCATCGGTGGCAGCTGGGCGGGGTCTCAGTTTTTCTGGGAC

AGATGGATCGCGGAAAGGCTCGGGAGGTCAAAGTGA

>g3799.t1

ATGGCAGGGGAGGACTTCAAGGGTACGACACGCACCGCCGATGGGTTGGAGTGCACACTC

GGTCGCCTGGTCTTCGATATCTTCGAGCCTGTAAAGATGCCCGTTCTCATAACGTCCATC

GAGAACAACCTGGGACCGCGACCGGCAGGGGCTTCGTCTGCCGACGAGTCTGCCTACGAA

ACCGTCACGCATTTCGTAGCGCGCGACGGCAGTGGCTTGAGGGGGATTGTCGTCACGAAA

GGCATTGTAGCTGAAGATCCCTCCGACCCGAAGTGCTTGGTGGTAAAATTTACGGTGGGT

ACAATCGAGCCGAAGGGTGATCAGGATCTCGACTCCTGGAGTAAAGTGATCGGCTTGAAA

GACCGAACGGGCAAGGACTCGAAAGGACCGTTGTCCTCGATTAAAGGAATAGCGAAGGGT

CTCATGCTTAAGTTGGCGTTCGGGTTTCGCGGACCAGCCGATAAGCTTGGCAGTCGTGGA

GAGCTCTCGTACGAGATGAAGCGTAGCCCGTCGAGCGTGCTCAACATTCTTTACATTGAC

GATGAAATGCGGGTCACGCGCGGCGGCCAAGGTGCCCTTGTCGTGGTCACTCGCCGGTGA

>g3883.t1

ATGCGCCAGGGGATGCACCCGGTCCAGAAGCGGATCGCGGAGATGCACGGCAGCCAGTGT

GGCTTTTGCACCCCAGGCATCGTCATGGCGCTGTACGCTCTCCTTCGATCCAACCCTTCC

GCCACCGCGGAGGAGATAGAGGAAGGCCTCGACGGCAACCTCTGCCGCTGTACTGGTTAT

CGGCCTATTCTTGACGCGGCGAAAAGCCTTGGTGTCAATGGTGGAGCCCCGGGAGGCTGT

TGTAGAGGTGGTGGCAGCGGGGGTGGGTGTCCCTGCTACGATGACAAGgcagcagcagca

gccaaagtGGTGGGGGGAGAAATGGACGATATCGTCTCGGAGTCTCCATCTACTACATCA

ACGCCTGTCACTGACGGGTCGAGCGTTCAACCCAGTTCTCCtattgatgatgctgctgct

gctgctgctgtgggtccCATCTCCAACGGCTCTCACGGTGACGGTGACAAGGACCGTTGC

AGCAAGACGGACTGCACCGGTCGTAGGGGGAGGGAATTGAGGTTCAGCGAGAGGTACACG

GACGCAAGCGAGCCTATCTTCCCGGCCTCCCTCATGCTTAAGCGGCCCTCCCCCGTCACC

ATCGCGGGAGATACTGTCACGTGGCACTTCCCCACCACCCTGGACGACCTGGTGGAGCTC

AAGGCGAAGCATCCCCAAGCGAGGATCGTGGCGGGGAACACGGAGGTCGGAATCGAGGTG

AAGTTCAAGGGAATGCACTACCCAGTGCTTATTTCCCCTTCCCGAGTGCCCGAACTCCAC

GCCATCACCGCAAACCCCGACGGAAGCGTGTTTATCGGCGGGGCTGCTCCGTTGTCAGCT

GTGGAGCACGCGCTGTCGGAATTGGACAGGATTGGGAAGGATGTGGAAGGAGCCGGAGGG

GAGCTGGGAGTAGGGTCGGTAGCGTTTGGGGCGGGAGCAGCTCGTGCGTGTGTTGATATG

CTGCGATGGTTTGCGTCGACGCAGATTAGGAACGTCGCGTGTCTTGCAGGAAATCTGGCC

ACGGCCAGCCCTATCGCCGATATGAATCCTCTGCTGGGCGCGTGCGGTGCCGACGTCATC

CTCAAGTCCGCTAAGGGCGCCAGGCGTACCGTGAAAGTTCGCGACTTTTTCCTGGGGTAC

CGGAAGGTTGCCATGGAGCCTCACGAGATCATCCTTGGCGTGACCTTGCCCAACGCTTAC

TATGTCGCGGCAGCCGCTCCACCATCACCCTCATCGCCATTGTCGGCATCATCGTTGCAG

TTCGAATTTGTGAGGCCGTTCAAGCAGGCTAGGCGTCGTGAGGACGACATATCGATCGTC

ACGGCCGGGCTTCGCGTCAGGCTGGAGCCGCGGCAGGGCAAGTGGGTGGTGCTTGAAGCC

GGGATGTGCTTCGGCGGGATGGCGCCGACGACTGTGGCGGCTCCACTGACCGAGGCCTAC

CTGGCCGACAAGGAGTGGTCAGCGAAAGTGATCGAGGGAGCTTACCAGACCCTAGCGGAA

GACCTACCCTTGCCTCACAATGTTCCAGGCGGGCAGTCCGAGTATCGCCGAGCGTTGCCA

CCGTCTTTCCTGTTCAAGTTTTTCGTGGACGTTTCCATGAAGCTGGAGGCGTTGTCGACG

AGGTCGGCGGGAAAATTGCCTCCGCCTCCGGTGATTGGAGATACAGACCGATCCGCGGCA

ACGAATTTCATCACGAATCCCAAGCCTGCAAGCAGGGGACAGCAGGAATACACCCCCCGT

AACGGTGGCATGCAGAAGGCGCGGCCCGTCCCTCACGTGCCCATTCCGGAatcaggagaa

ggggggggaggggagttgaAGGTGGGAGGCGTTAACGACGTGGGGAAGGCGATGCCGCAC

AAGTCTTCCGCCCTCCAGGTTACGGGGGAAGCCGTCTACGCGGACGACATGCCATCGCCC

GTCGGAACGCTTTACGCCGGCCTTGTTCTCTCTACGAAACCCCACGCCAAGCTGCTGGGA

GTAAACCCCTCGGAGGCTCTGAAGCTGGAAGGCGTGCTGCGTTACGTGGGATCGGGGGAC

GTTAGCATGGAAAGAAACGCCATCGGACCGATCGTTCGCGATGAAGAACTCTTCGCTGTC

AGCGAGGTCCACTGTATAGGACAGGTCATCGGCGCCGTGCTGGCAGAGACTCCTGTCCTC

GCCGAAAAGGCGGCGAGACTGGTGGCGGTTCGGTACGAGGAGCTGGCCTCCATTATGACG

ATCGACGAAGCCATCGCCGCCGACAGTTTCTACGAGGGCCGAGGAAACGGTCTTGCCGAC

GGTGACGTCCAGGGGGCGCTGGCCAGCGCTGACGTCGTCGTTGAGGGCGAGCTAAAGATT

GGTGCTCAAGAACATTTCTACCTCGAGACGAACGCTACCCTGGCCGTGCCCGGCGAGGAC

GGCTGCTTAGAGATATTCGCTTCCACGCAAAACCCGGACAACGTACAGACCGTGTGCGCT

AGCGTATGCGGGGTTGCTTTCAATAAGGTCCACTGTATAGGACAGGTCATCGGCGCCGTG

CTGGCAGAGACTCCTGTCCTCGCCGAAAAGGCGGCGAGACTGGTGGCGGTTCGGTACGAG

GAGCTGGCCTCCATTATGACGATCGACGAAGCCATCGCCGCCGACAGTTTCTACGAGGGC

CGAGGAAACGGTCTTGCCGACGGTGACGTCCAGGGGGCGCTGGCCAGCGCTGACGTCGTC

GTTGAGGGCGAGCTAAAGATTGGTGCTCAAGAACATTTCTACCTCGAGACGAACGCTACC

CTGGCCGTGCCCGGCGAGGACGGCTGCTTAGAGATATTCGCTTCCACGCAAAACCCGGAC

AACGTACAGACCGTGTGCGCTAGCGTATGCGGGGTTGCTTTCAATAAGGTGGTCTGCAGG

TCTAAACGCATGGGAGGTGCGTTCGGGGGCAAGGGGACCCGGTCCGTGTTCCTCTCGTGC

ATCGCCGCTCTGTCCGCCCACGTGACGGGAAGGCCGGTGCGGCTTAGCCTGGACAGAGAC

GTGGACATGCAGATAACCGGTCAGAGACATGCCTTCCTGGCCAGATACAGAGCCGGGGTC

ACCAAGGGTGGCAAGCTCTGCGGCATGGACGTGAAGCTGTACAGCAACGCTGGGGCCAGC

CTCGACGTATCTTCCGCGGTTATGAACAGATCTCTCCTCCACATCGACAACTGTTACAGG

TGGCCTACCATGAAGGTGGAGGGCTTCGTGTGCAAGACCAACCAGGCGTCCCACACCGCC

TTCAGGGGCTTCGGCGCGCCTCAGGCGATGCTTGTCACGGAGACCGTCATGGATCACTTG

GCCACAAGCGTTGGCATAGACGCCTTCAAACTCCGCACGGACAACATGTACAAGGAGGGC

GATGTCACCTTCTTTGGGCAGGCCTTAAACGCGTGGAATGTTCGGAACGCTTGGTCGGAC

ATGCAACGGTGGGCCGAGATAGAGCGCCGTCGCAAGGAGGTGGACGCCTTCAATGCCGGA

AGCCGCTGGAGGAAGCGAGGCCTTTCGGTCATTCCGACCAAGTTCGGCATCTGTTTTAGT

GCTAGTTACTTGAATCAAGGTGGAGCGCTCGTGCACGTGTACCAGGACGGCACGGTCCTA

GTCACCCACGGCGGAACGGAGATGGGGCAGGGGTTACACACCAAGGTCTGCCAGGTTGTG

GCTAGTGCTTTCGGGATCGAGGCGGAGAAGGTGCATGTTGCCGAGACGGCGACGGACAAA

GTCGCCAACTCTTCGGCCACCGGCGCCTCGGTTTCGACCGACCTGTACGGCATGGCCGCG

CTGGATGCCTGCGAGCAGATCACGGAGAGGCTCGAGCCCATCGCCGCGCAGCTGCCTCCC

GGTTCTTCCTTCGCGTCGTTGGTCACgGCGGCGTATTTCCAGAGGGTTAACCTCTCTGCC

CAGGGCTTCTACAAGAACCCCGGCAAGTGCGTCTTCGATTTCGCCATGGACACCAAGAAC

AACGCGGAAAGAGGCCTTCCTTACAACTATTTCACCCAGGGTGTAGCGGCGAGCGAGGTT

GAGGTCGACTGCCTTACAGGGGACGTGAAGGTGGTGAGGGCAGACATACTCATGGACATC

GGAGCCAGCATCAACCCAGCCATCGACATCGGCCAGATCGAGGGTGCATTCGTCCAGGGG

TACGGATGGTGCACGATGGAAGAGACCACGTGGGGGGACGCGGAGCACCCCTGGGCTAAG

CCAGGCCAGCTCCTTACTCGCGGGCCTGGTACGTACAAGATACCTTCGTTTAACGACGTA

CCGTCCGACATGCGGGTGAAGCTCATGGACAGAAAGAACCCCTTCGCCGTACACTCGTCC

AAGGCCGTGGGAGAACCTCCGTTTttcatggcgtcgtcggcatttTTCGCGATCAAGGAT

GCGGTGGCTTCGGCCAGGAGGGACCACGGAGAAGCGGGGTACTTCGTGCTCAACAGCCCC

GCTTCTTCGGAGAGGATCCGCACGGCCTGTCTCGACGGTTTTACGGAACGTTCCGTGGAC

cgttttggtgctggtggtgctgtcggCGACGATGAAGTCGAGGTCAAGGACTTCCAGGCG

AAGGGGTCTTGGTGA

>g4144.t1

ATGTGTGCGGATTTCTGGAGCGCGGTTCAGAGTTCTATCGGGGTTACTCGCTTGCCCAAC

CTGTACAAGGTCGTCATTATTTACATTGTTCAGCCGCAGCACTCGCCTATCGATCGAGTG

CGATCGCGGGTTATCGTCCCTAACCAGGATCACGAGTCCGGCCCGCAGCAACCACTCTGT

GGAACGCGGGCTAACAAATACGGGCAGCTCGGGAGGGGCGACAAATATGACACTGGCGAC

GACCCCGATGAGATGGGAAACAACCTCATCAAAGTCAACCTCGGCTCAGGAGAGGTCGCA

GTCGGCATTGCTCTTGCTGAAGAGCACAGTTGCGTTGTGCTCAAGTCCGGCGGCACGAAG

TGCTTCGGAAAAAACGACGACGGGCAGCTCGGTCTCGGTGACACGAAGCACCGTGGGATT

TACGCCAGCGAGCTCGGCAACAGCCTGCCTGAGGTGGACTTCGGCAGCGGTCGCACAGCA

AAGTCGATCACGGCCGGGTGCTCCCACACGTGCGCCCTCCTTGACAATGGATCGGTGAAG

TGCTTCGGGTACAACAACTACGGGCAGCTGGGCGAGGGCTCGACCAAGGATGTCGGTGAT

AAATCCGGCCAAATGGGCAACAACCTCGAGGCCGTGCCTCTGGGCAGCTCGAAGGTGGTT

GCCATCGCCGCCGGCTGCGACTTCACGTGCGCAGTTACGAAAGACGGGGACGTCAAGTGC

TGGGGGCGTAACACGTGGGGGCAGCTCGGCactggcgacgacgacgaccgccTGGATGGC

TCAAACGAATCGCTCAAGACCGTGAACCTCGGTGGCTCCTCCGCGATCGCCATCGCTGCC

GGCGAGGCCCACGTCTGCGTCCTGACCAAAGACAAGTCTGTCAAGTGCTGGGGCCGCAAC

AACCGTGGCCAGCTCGGCCTCGGTGACGACACCGACCGCGGCGACGACTCATCCCTTCTC

GGTTCCAACCTCAAAGCCGTCCGCCTCGGCAGCGGGACCGCTGCCGCGATCGACTGCGGC

AACGAACACACCTGCGTTCTTCTCAAGGACGGCGACATCAAGTGCTTCGGCGAAAATGGA

AACGGCGAGCTCGGGATCGGGTCGACGTCCGACGTCGGCGCGGACTACTCGGACATGGGC

AACAACCTCGATGCCGTCGACCTCGGCAAGAAAGCCACTTCCCTCGCTGTTGGCGGATCA

TCGGCGTGCGCTGTCCTGTCCGACGGCTCCATCAAGTGCTGGGGCCGCGGACGCAGTGGA

CAGCTCGGCCAGGGTGACGACGAGAGTGTTGGCGACAAATCTGGTGTTCTGAAAGCCTTG

GACCCGGTTGACGTCGGCTCCGGCTAA

>g4257.t1

ATGATCACCTTGCTGGAGGTGGACGAGAGGGGCACGGTCTCTCGACGGCTGGacgaggtg

gtgatggtgaagtACGTTCGGAGGGAGATCAGGACCCTGACCGACGACGAACGGGAGGAG

CTGCTTGACTCGATGTTTGAGCTGTGGGCGGTTCGTGTGAAGGATGGAAACGGAAGGGAG

ATCTACGGAGACCAGTACTCCGACATCTGGGCCATCAACCGCCTGCACTACAAGGCGGCC

AGCCCCCAACTGTGCGATCACTTCCACGATGGTCTGGGGTTCTTGACGAGCCACGCACTT

TTGACTAATACGTTCGAGGCCAGTCTGCAGCAAGTGAACCCGAAGCTTACGGTTCCGTAC

TGGGACTTCACCATCGAAAGTTCTTCCGGTGCTACCTCCTTCGAGCCCACCGTGGACAGC

AAAGGGGGAGAATCGCTCGAGGGAGGCGGCAGAACGCCTCTTCTGCAGCCCTCGTGGTTC

GGCACGACCGACCCCGACGACAACATGGTGAAGGACGGGCGTTGGAGGTACACCAAAATC

CCACGGATGGCGATAAACAACCCGAGCGGCATCGAACCCGACGTCTACGGTAGGCTCCGG

GCGCCTTGGAACATCAACAACCGAGAATACCTGGCGAGAGGGATGGGCAAGATGTGTGGC

GTCGACGTCGAGGAGGACTACCCTTGGCCCACGTGCGAGGCGCACTACAAGCTCGTCACC

TCTTACTCGGActtctactcgtgggtgtgggctagCCTGTACGACCCTCACGGCCCCGTA

CACATCTGGATCGGCGGGATGCTGGACTGCACGAACACGTACGCGAAGATCCGCGAGCTT

GTGGGCGAGGAAACGGCGAGAAAACTGATAAAGCTGGGTTTTGTTCACCGCAAGGAACTG

TTCCGCAGCGGGTTTTTCACGTGCGAGGGCAAGGTGGACGTTTCGGCGCGGGAGGACGAG

GTCTTTGCTAGCGGGGTCTGCGGGTGCCACGGCTACGACCTTACCCAGGGCGATGACTAT

TTGACGATATTCTACGAGATGTTCATGATGAAGGAGTTTCTAGTGGACGCGGATGATGAC

GCTAGGCGAGAGGTTGTCTCCATCCTCTGTAGCGGCGCTATCAGTGACGGGGACCATCTT

CAGgcgAGCTCGTCGCTGGACCCTACCTTCTGGCCGATGCACCCGACCATGGAGCGGCTG

TGGATGTTCTCGGTCCTGACCGGGTCCGTCACGGACATCTCCTGGCCGGACGAAGACATC

ACCTTCACCGGCACCGACGGGAAGACCCACACAGAGCCCATCTGCGCCTACGCGGCGGAC

TGCGACGGCCACCGAGGCAAGGACGTCTTCCCGTTCGGCCTGCTCGACTCCGACGCGACC

GACTTCACCGTGCAGGCGGGTATTAAGGGGAACGCTCCCCGGGGTGGACACGCGCTCACC

AACCGGGAAACCCTGGCGGCGCTCGACCCTAAGGTGAACGCGCTTCCGTACATCTATGAC

ACGTTTAAGTGGACCCACTGTGAGGCGGACGGTGTCAACATGGACGATGCGTGGGCGGCG

GGTCGGGACGCGGAAGAAAATACTCGAGCTCCGGCTGGATACCCCGGTCGGAAAGAGCGG

CCCGTGTAA

>g4292.t1

ATGGGTCGCACTGCTTTCGTTGCTCTGGCGGCCGGTATGCTCGGCGTAGCCAGCTCGCTG

GAGGTTATCTCCCCTTCCCAGGGCTTGACGGTCGTCTCTGAAAGGTCCTACACGGTGGAG

TGGACCGGAACGGGATCTAACAGCCGCTTCACGATCGACCTTTTCTTCTGCGGAGACATG

TGCTCGCAGGACGAATGCGGTGAGTGGGTGACCGCCCTGTGCCCTTACGGCGAGGACGGC

TGCCCCGACAACGAGGGCGACTACGACGTCTTCATGCCCCAGCCCATGGCGGACACCTCC

GGCTCCGGCTACAAGGTCCGCGTCGAGAACGTCGCGGATGAGACCGAATACGACTGCTCC

GACGAATTTATGCTCATGGCGTCGGAAGAGGTGGACGAACCGGGGATGCCGGGCGGGCCC

ACCTTGGAGGTCACCTCCCCGTCTACGGGTGACGTGGCGATTGCCGGAGACGAGTACACC

GTAGAGTTTGACTTCGACAACGGCTACGGGTCCCCGGTCGGGCGGTTTGCGATCGACCTG

TTCATGGCGGGCGGCAACGGCGACTGCGGCACGTTCTTCTCCACCATTTGCGACAAGCCC

TCCATCGGGTGCAGGGACACGATGGGCGACTACGACGTGACTATCCCGGAGAACGCACCG

GACGGCGACTACCGTATCCGTGTTGGCGACTTCAGTGACGATGAAGTCTTCGGCTGCTCT

GACATGTTCGAGGTCGTGGGTGGGCAAGATCCCATTGAAGGCAGCATGTCTTTCGACTTC

GACTTCGACTTCTAA

>g4468.t1

ATGCCTCGGTCACTAACATGCGCTCTCGCGGCTACTGCCGTAGTTTCGTCAGTCACAGCC

TTCGTTCCATTTGGAGGTGCGATTGCAACACCggcgccgagcgcggcgactcACCGAGCG

GCGCAAAGGACCCGACCACGCGCCCAGCCCACGTGCATGGCGATCAGGGCCGTTGAGAGC

TACAACGCCGGTTTCGAGCTGGAGGACAAGACGGCGGCCACCCTGGACGGGTACCTGAGA

CATCCGGACATGATCGCGGAGGTCTGCAAACAGTTCGACTGTACCACTGCCGAGTACACG

GGAGACATCTTCCAGCCGGTACCCTACAGCGCCACCACGCCCCAAGGAATGCCGATGGAG

ATCGAGGACAAGTACTTCCTGAAAGAGGAGTTCGGCATCGCCAACATCCCCCCGGTTGTG

ATGTTCAACGCCAAGATCTTCAAGCCGTCGCGTCTGTGCGCGGTCTACCGGATCGAGGGC

ATGTCGGAGGCTGCGCGAGAGGTCCTGAAAGAGCGAGTGCTTCGCCTCAGGGAAAACGAG

AAGCGCAAAAAGGACGAGGCGGAGCTGGAGAGCGAGTGGGATGACATCGGAAGCGCTTTC

ACGGAGGTGTACAACATGCCCGTTGGCTCGGACGACGATGATGTCGACCTCGACAGCATG

GTCGTGTAG

>g4595.t1

atggcgaTGCTTTCGTTCTTTCTGCGGATCGCTGCGAGGGAGCTAGGAGTGCCTTTCGAG

TTCCACGCTATGGAGCTGGGAAAGGACAACCGCGAGGAGTGGTTCTTGAAGCTCAACCCG

CTCGGCAAAGTGCCCACGATTGTCTGCGGCGATGATGTGGTATACGAGTCTTTGGTGGTT

AACGAGTACCTGGCGGACGCCTTCCCCCCGGGGGGAGAGCTTGGCGCGTCCCCTCTCCTG

CCCGCGTCGCCCGCGGGAAGGGCCATGGCGCGCGTGATAGCGCAGCGCAGCAACGATTTG

GTGACGGCCTACTTCACCTACCTGAGCAACAATGACGAGGAGCAAGAGGAAGGGAAGCGC

GAAAAGTTCGTGAAGGAGCTGAAAGCACTCGACGGCTGGGCGGCCGACGCCGCGGGGGAA

GGAGAGGAGTGCGGCTGGCTGTGCGGTGCGGCGGGCGAAGGGTGCATGACGCTAGCGGAC

ATCTCGTACTTCCCGTTCCTCGAGCGCATAGATGCCACACTGAAGCCCTTCAAGGGGTGG

AGTCTGAGCGAAATAGAGGCACCTGCCCTTGTCGCCTGGATGGAAAAGTGCAGGGCGAAG

GATTCGGTGGCGGCCACCCTCAAGGACCCTGCCCTCTGGGCCGagctgtacaaaaagttt

ctGGGGGCGGACTACTTCGTGAGGGCTGGGGTCGCCAAGAAGTAG

>g5207.t1

ATGCTCACCGTGGCTGCTCCTGCCATGAGGCTCCGCGACTACCAGCTTCGCATTGCACGG

GGATGCGGGAAGGCGAACACCATCGTGGTGCTGCCCACGGGGTCTGGCAAGACCCTCATC

GCAGCCGAGGTGATCAAGCGAATTGGCGCCCCAGCCCTCTTCCTGGTCCCGACGTGCCTC

TTGGTCGATCAGCAGGCGGAGGCACTGCGTAGCTGGACGGGTCTCGACGTTGCAAAGTAC

AGGGGTGGCgtgtcgctgccgccgtcatTCGACGTGTTGGTTGCAACCCCCGAGGCTTTC

AGGATCGCCCAGCGTGACGCAGCGAGCGAGAACTCGGGCTCGTTCCGGCTGCAGTGGCAG

ATGTtccgggtggtggtgttcgaTGAGGTGCACCACGTACTCAAGGAACACCCGTACCGC

AAGCTGGCGCTAAGTCTTCATCGTTGGTCGCTTTCACCATCACATTCGAGCagctcgcca

ccgccaccactgccatCATCGATGCAGCCACGCGCCCGTCACTCcccacctcctccacca

ccaccaccacagccgagCGGGCTGCGGGTGCTAAGCCTCACCGCCTCCTACACCTACGCT

GTCGGGGACAAGAAGGTCGAAGCCTCGCTGCGCTCCATGTGCAGCGAGCTCCTGGTGACA

AACACGGAGACAGCAACCCCGCAAGAGCTGAAAGATGGTGGTTACCACGCCGTCGGGGCT

ACGGCCGAAGTCGTCCTCGATCCCATCGGAACGCGTGCGATTCCGCTTCCGAGGGGAGTG

GTTCCGGTGGCCGCTCGCAAGCCGCACGAGATGGGGTCCACCTTCTTCCGCCGGGTTAGG

GAAGGTAGCAATACCGCCTTCACTGGCCTGCTCATGGCTTGCGTTCGCGCAATGGAAAAC

GCCGTTTCCCTGTCGGAACTCCCGTCGTTCTCCTCACCGCTACCTCCTTCCGGAAATCTC

GCGCCCAGGGAGTGGGGCACCCATGCTCACAAGCTCGCACGTGGCGGTCGCAGTTCCAAG

AAAATTGCCTCCACTCAAAAATCCAGGGCTCACTCTACGCGCGGCCAGCCCGCGCAGCTA

GCGCGTCCGGACGCCGTTCGCTTTCCGATGCTCGCCGAGCTAGAGCATTGGTACGAGGCG

GTCAAAACCTTGGTTGTCACctgggaggaggcggaggacgaAGCTGCCATGATTCTTGAC

ATGGGCGGTTGCAGGGCACAGAGCAGTtctgcgagtcggcgaggggaAGACACGTGGCCG

GGCTCCGTACGGCAGGTGGTTTCGGCGTTTTGGGAAGAGGTTCCAGACAGGTTTCCCCGC

TACGAGCACCTTAAGGACGTGCTGATGGAAAAGTATGTTCAccatggcggcggtggcggt

ggcagtggcggcggcgacggcggcggcggcggcggcggcggcgacagaaaCAGCTTCCGC

GGCGTCGTGTTCGTGCAGAAGCGCGTCACAACCCACGTGCTGGCCCACGTGCTCTCCATC

GACCCCGATCTGGCGCCGTTGTTCTCGACGGCCTGCCTGTACGCCTCCTCGTCCCCCGCG

ACGGCCTCCCTTTCCGTTACCAAGAGCGCGGCGCAGGCTCACCTGAGTGACTTTCGCGAA

GGCCGCGTTAACCTCCTCCTGGCTACCGTCGTCGCTGAGGAGGGCATGGACATCCCCGCC

GCAAACTGCACCATTCGCTTCGACGCGATGGAGCACGCCGTTTCCCTCGTCCAGGGGCGG

GGTCGCGCGAGGCAGGCCGGGAGCAGCTTCGTCGTCCTCCGCGAGCGCTCGGACCGCACC

ACCGCCGACCTCGAGGCCGTCGAACAGCAACAGCTGCGCCTCGTGCGGAACTTCAAGCCT

CCCGTGGGGGGCACGGATAAGGCGGCCCACGAGATTCTCTTGGCGGCGCAGCGTTCCCGC

GAGATCGGAGCGCGTGGCGTCTTGGTAGACTTCGGAGTGAGGTTGAGCCTGCATGTGGGT

GCGGCTCCTGCAGCAGGCACGGCGAGCCCCACGCCCGGGGCGCTGAGTGCGGCGGACCTT

TTCTCGAAAAGGACGAAGGCTGTCCTTGAGGACAGCTTCAAGAAGGAGGCGGGGCTGTGG

GTGTGCACCATGAAGTACGAGTCGCCTCTTCGCGACCTGCACGCCGCCGGGAAGTCTCCC

GGAAAGAAGATCGCCAGGCGTCTGGCGGCTGAAAGGCTTGTCGCGCAGCTCCTCGCCGCG

GTCCCGGCGTGA

>g5223.t1

ctgtccgaTGATCTGTGCAAACCCTCGCAGGTGACCTTCGAGTCCTGCACACGGCGAACT

ATCCCGAGCTTCGCCACCTCCCGCAACGTGGTCGTTTTTCCGTGCGTCGGGGACCGGCCG

GAGCCTTCCAAGATGAGCGGCGGTGACTTGGATGGCGACATCTACTCCGTCATCTGGGAC

AAGAAACTCCTgccgcctgagcagcagcagcggtcggggggggggggggagcggaacTAT

GAGCCGATGGGGTACGCTCCGCCAGAGAAGCCCGTCAGGGCGGAGACGGGAGGCGTCGCG

ATTGAGGCGAGcagagtgggggggGAAATCACCGAGTTCTTCGTGAAGTACCTCGTGAAC

GACAACCTCGGCATGATCGCGAATGCACACCTCGTGCACGCAGACCTGTCTCCCATCGGG

GCGAAGAGCAGCGAGTGCCTCAAGCTGGCGGCCTTGCACTCGACGGCGGTAGACTTCGGC

AAGTCGGGCGTCCCCGCTGTCATCCCGAagGAGCTGCGTGTCTCGGAGTACCCGGACTTC

ATGCGCAAAAAGCACGGCAAATCCTACATGTCCCAGAAGCTGATCGGAAGGCTGTTCCGG

ATGACACCGGCAGATCGGGACCGTCAGGAGTACGACCGCACGGGGCTACACCCGACGGAG

GCGTTTCTAGGCGGTTTCGAGCTGGACGAGGCGTTACTGGTGCACGGGTGGCAGGACTAC

GTTCCCGAAGCGGAGGTGGAGCGCTTCCGAGCCAAGGGTCTGAAGAGGGTCAAGCGGGAG

GATGCACAGGGCAGGCTCAACGTTGAAATGGCAAACGTGAAGGCCTACTTCCGTATGCTG

TTCCAGGGGGACAGTCCAAGCCAGGAGGGGGACGGCTTCGAAGAGGCAACTCAAGgcaag

gaggaggaaggagaacaggacgaggaggaggaaggagacgaggacgaggaggaaggagag

gaggacgaggcggaaggagaagaggacgaggagggagaagaggacgAGGATGGTAGCCTC

CCGGAAGGAATGGAGCTCTTCAAGAAGGCATCGGCCTGGTACTACGTCTCCCACTCCGAC

AAAATGAACCCATACATGGGCGACGCTCGCAAGAAATGGAAGGCCAATGCTGGCCACAAC

AACGGAAGCGTGCGGCAGCAGCCGGAGCTCATGTACCTGAGCTTTCCGTGGGTCTGCGCG

TACCAGCAGCTGTGCCAGATCAAGAGGATGGAGACTAGTCGTGGCACGTGA

>g5241.t1

ATGATTGGGCGAACAGCCGCAAGCTTGGCGCTGCTAGCGCTGGCGGTGCCCTCGCAGCGC

ACGGCGCACGCGTGCACGGACGACGAGCCGAGGGCCACCATCCGCTTCAACGTTGCCTCG

GACCGGCTTTACCTGGAGGGGTCGGGGTGCATCACCCCCTCGGACATCTACGCGCAGAAG

GTGGCAGGCGATTCGAATCTGTCTATCAAGGCTGTTACTGAGGATGGGGAAACGGCCGCC

AACGAGACTGGGTACTGGATTTTCACGTCTGACGTTTACGTGTACACGGGGGTCAAGCTC

GAGCTTCACAAGGAATCCGGCTGCTCGGCGATGCGGCTCAAGTCAGACGACACCAAGTTC

ATAGAGCTCAGGGCGCACGGGGGGTGGCTGTCGATGCTCAACACTGAGTTGTCTTCTTGG

GACGAAGACGAAGGGGGCTACCAGACGAACTACTCGGCGCCGAGGTCGTACGTGACGGCC

ATCAGCGAAACGTTGGAAGACCCTGCGGAGGACTGCTTGGGCCACGCTAAGAACGATGTT

GGGGAAGCTAGGATGGACATCATCAACAGCGAAGTTCACCACCTCGGGTACCACGGTACG

GAGGCCTATGGCATCACCTACAAGGTGCGAGGGTTCTGCAAGGACCTGTCTAACGAGCAC

ATCTTCGACTCGGTGAACGTAAGGGGCGACATCCGGTTCTCCAACCTGCACCACAACTAC

TTCGGCATGTACTCGTACGGCCACCAGGATGGCCTGTGGGAGTACAACCTCATGCACCAC

AACGCTAAGTACGGCTTTGACCCCCACGATGACTCGGACAACCTTCGCATCCACAACAAC

GTGGTCTGGGAGAACGgtgacCACGGCATCATCGCGTCTAAGCGATGTAACGACGTGAGC

ATCCAGAACAACGTGGTTTACGACAACCTGAACGCGGGCATCATGCTCCACCGGTCCAGC

GACAGCGCCATCGTCCGAAACAACACAATCTCAGGCAGCGGAGACGCTTGCATCGCCATC

TTCGAGTCCTTCGACGGTATTTACATGTACGAGGGCAGCGACTCGCCGGAAGCGAGTGAC

GGGCTTCCCTCCTTCAACATTATCGCCGACAACTACATCGAGGATGCTCTCGAGGGCCTT

AAGATGGGAGACACGGTTGGGAACGAGTTCAGCGGTAACgtgtTTGTTGACACGGCGGAC

CTGCGATTCCGAAACTCCTCCAACGTTACCTGGacgAACAATGTCATACCGGAGGGAGTA

TCGATCGAAGTGTCCGAATCCTGTTTTTCGTCAGATTCCGATATCGTTCAAGTCGGCGGT

ACCGTGTGCTAA

>g5245.t1

ATGATCAACCATGTGGTGTACGACGTGTTCATCAAGAACGACGAGGATGACAAAAACGCC

CAGGGCCTTCGCGGTTGCTCGCGCTTCAACGAGGACCTGGAATCTTTCACCAAGAAGGGA

CTCGAGTTCAGCTACGTCAAGAAGGGCGGCGAGAAGAAGCAGGAGTACGACTTCACCAAG

GACTACCACGTCATTGCCAACATCGTCTCCAGCCCCAGGTCTTTCTTTTCGGCTGAGGAC

AACTGGTTCGGCTTCGTCCTGCTCTCCAACGACTCCAAGGAGatggtggtggtgttccgt

GGAACGGAGACGGAGGCTGAGTGGACGGAGAACGCAACGATGACGATGACTCAGCTGGAG

GGCACGGAACAGGAGCACGGCCTGGGACTCATCTTCAACCGCAAGCAATTGATGTGCCAC

CTAGGCTTCCAGCAGCTGTACCACCAGCAGTCCGTTCAGGGCCTGGAGTCCCCCAAGCAG

ACCGTCCACCGCATGGTGGAGGAGAACAAGGCCACCCTGGAGAAGGTCACCATCGTGGGA

CACAGCCTAGGCGCTGCTATGACCCAGCTGTGCGCGGTGGACCTGACGTACTCCAAGGCT

CTGGGAGACATCCCCATCCTCGCCATCGCCTTTGCTCCGCCCAGGATCGGCAACAAGCAC

CTCGCGCAGTGGGTGGAGAAGCAGGAGAACCTGCGCATCTTGAGGGTCAGGAACCCCATC

GATGACGTTACCAACCGCTTCCAGCAGCTGTACCACCAGCAGTCCGTTCAGGGCCTGGAG

TCCCCCAAGCAGACCGTCCACCGCATGGTGGAGGAGAACAAGGCCACCCTGGAGAAGGTC

ACCATCGTGGGACACAGCCTAGGCGCTGCTATGACCCAGCTGTGCGCGGTGGACCTGACG

TACTCCAAGGCTCTGGGAGACATCCCCATCCTCGCCATCGCCTTTGCTCCGCCCAGGATC

GGCAACAAGCACCTCGCGCAGTGGGTGGAGAAGCAGGAGAACCTGCGCATCTTGAGGGTC

AGGAACCCCATCGATGACGTGACCAACCTGCCTCCTAACTGGCTGTGGACCATCCTCACC

GGCGGGTACATGCCCTTGGGCACCGAGTGCAACCTCATCAACACGCACCTGCACGACCAG

GGCCTCGTCAAGGGAGACTCCGGAAACAGCCCCAACCACAACCTGGAGATGTACCTTCAC

AACATCGACCCCACCAGAGACCCTGTGCTCATGAACAAGGTGGGCGACGTGGTGCCCGAC

GCGTACGGGATCAAGAACCACATCTCCCCCCAATGGCACAGCCAGACGTGGCCCCGTACc

atctacaacagcaacaagtcCTAA

>g5348.t1

ATGCGCTCTTTCGCTGTTCTAGCCACCATCTTTGCTGCCACCGCCGGCACTACATCAGCC

CTCGTTGATGCGAGCAAGAGGACATGGGTCGACGTCAAGGCCACTGGGTACGACGAGCGG

GACTACGGGAATGGTTGCACGCCGAACGGATGCGAACCTGATAACACTCGGGACGGCAGC

ACATCGGGAAACTCCCGCTGGTCTTGCCGAGGAGACATCCTTGACAGCTCTGATGACGAC

GAGGGATGCTGCATCAGGTACCACTTCGAAGAGCCCCAGGACATTGAGAAAATTCAAATC

GCTTTTCACCAGGGTACATCCAGGACCCGGACCTTGGACGTGTACGACAACGACAACTAC

CACAGCACTATCTCGTCCAGTGGCTCGACGAACGGATTCCAGACGTACGACCTCTACACC

GACGAGACCGAAGAGCTCAAGCTTTGTCTCTTCGACCCCAAGTGGAACACTGATGTTTGG

CTGAGCATCACCGAGGTCAAGATCCTGGTGGATTAG

>g5350.t1

ATGCACTCTTTCTCTATCCTAGCCACCATCTGCGCTGCCACCGTTGGCACTACCTCAACC

CTGGTGGATGCGAGCAAGAGGACCTGGGTCGACGTCAAGGCCACTGGGTATGACGAGCGG

GACTACGGCAACGGCTGCACCCCGAACGGCTGCAAACCCGATAACACTCGCGATGGCAGT

ACATCCGGaaactctcgctggtcgtgccAGGGGGACATCCTCGACAGCTCTGATGACGAC

GAGGGGTGCTGCATCAGGTACCACTTCGAGGAGCCCCAGGACATTGAGAATATTCAAATC

GCCTTCCACCAGGGTACTTCCAGGACCCGGACCTTGGACCTGTACGACAACGACAAGTAC

CACAGCACTATCTCCTCCAGCGGCTCTACGAACGGATTCCAGACGTACGACCTCTACACC

GACGAGACCGAAGAGCTTAAGCTTTGCCTCTTCGACCCTAAGTGGAACACTGACGTCTGG

CTTAGCATCACCGAGGTCAAGATCCTGGTGGACTAA

>g5371.t1

atggcggcggtggcgagttTCCCCGCTGCGGAGGACGGCCGCGGATCGCCTTCCCCTACC

AAGCGGTTCGAAAGCTCATCaccacagcatcagcagcagaagcagcagcagcagcaacaa

caggatcTATTAGGGTCCCACCGCGATGGACACGAAGAGCAGTCTCTCGAGTCTCCTTCC

TCGAGGTCACCGTCGACCTCTACTTTGCCCAGGATGGTGCCTGTTGTGTCTCGGCCGCTC

CCTCCTCGAGATGCGtcacccacagcacccagccaACGGCAGTGGACGGGGCCTGGGAAG

ACAAACACCGTGGTCAACGGTCTCACGTTTCGGTCGGATTTCGACTCGGGCAACCTCATG

AAggtggtgcccccccccgccgagcaGGAGGGAGCGGATGGGGTATATCAGCTGTGGACC

GCTAGAGACTGCGAAGGCGGGCCGAACACCAAGCGCAACTCGAGCTGGTTCTACTTCGGC

GTGGCTGGTGGGTCGAGGGATCAGATAATCTCTATGAGGCTCATGAACTTGAACAACCAG

AACGCGCTATACAAGCACGGCATGACACCGGTGTTCCGGCTCTCGGGAAACCCGCTGTGG

ACGCGCCTCAAGCAGAAGGTTACGTTCGAGGAAGAAGGACGGCATTTGAAACTCCAATTT

CAGTTCCGTTTCACTAGGGACTCGGAGGAAGTGCTGTTCGCGTTTTGCTTCCCACACAGC

TATGAGGATTGCAAACGTGATTTGGAGCGCTGCGAAGACCAGGCTAGAAGAGGCGGCATG

GCCGTGCAGGCGTTGTTGACTCCAAAGGCTGGCCCAACGGGCTATTACCACCGAGAACTC

CTTATTCGGACACCTGAGGGGAGAAGGGTGGACCTCATAACGGTCACGGACTGTCACGGC

ATCCTGGACGAGCGGGAGTCTCGAATTGATCCCCACCTGTTCCCGGAACTCCCTTGGAGA

CGCAGACCGTTCAAGTTTGAGGGCAAAGGCATTGTCTTCGTCTCCGCGAGGGTTCACCCG

GGGGAGACTCCGGCTAGTTTCGTCTTCCAGGGCATACTAAGGTTTCTCCTGGATTCGATT

GACCCTCGCGCTGCGGAGCTTCGGCGGAGATTTGTTTTCAAGTTGGTTCCGCTCATGAAC

CCCGACGGAGTGGCGGCGGGTCACTTCAGGCAGGACTCTTACGGGAACAACCTGAACCGG

CACTACGCCGACCCGCACCCGGAGAAGCACGCCTCGGTGTACGCTGCCAAAGCGGTGGTC

ATGCACTACGCCGCGAGACCGGTCGGGAGGGGGCGGCTCACGTTGTACCTCGACCTACAT

GCACACGCTTCCTCCAGAGGGTGCTTTATGTACGGTAACCACTTACCCATGCTTGAGGAT

CAGGTGGAGAACCAGTTGCTGCCGTTGCTCATGTCGCTCAATACGCCGCACTTCGACTTT

CCGTCGTGCAACTTCACGCTGAAGCACATGTCGAGGGTAGACAGCGGAGACGGCGGTCTC

AGCGCCGAGGGAACCGGGCGAGTGTTCTACGGCAAGAACGCGGGGGTGTTGCGGTCGTAC

ACGCTGGAGTGCAACTACAATACGGGCAAGGCTAACTGCAATCACATTCCGCCCGCcagt

ggggggaaggggcgaggCTTCGCCAGCCCTGAGAGGAAGGCGACTCCCGCGCCGCGATAC

AATCCCGACGTGTGGAGGGACGTCGGTGCAGGTTTCCTAAAGGCGTTGTTGGATGCCGAG

GGTGCGTCGGTCTGGTCGCGCCTGCCCCAGTCCAAGCTCAAGTCCCTGGACAGGCTTCTC

ACCCCACCCATCCCCGCTCCACCCCTGACTGTTCGCCAAACATCGGGTACGCTTGGGCCT

GATGCTTCACCAAAACACCACAACCTTATCTTCTGGCAACAAAAGGCACGGAGATTCCTG

ACGAACGAGCTCAGAAGCCAGCCGTCATACCGAGAACAGTCGTTACTACTTAGGAAGGGA

GCCTCAGGCACGCCCCCGACCCTGACACCTCCACCCTCCACGGAATTGCGCAGCACCGCG

TCCTCACCTCCGCCGTTGTCGCGCACAAACGGAACTGCTCATCCACCTCTGTCACGAAGG

AAATCAGAATGCGCGATGGGTGTCGCCCGGCTCGTAACCGACAGCGCTACACCGTCGCCG

CCGGAAGCCGCTATGTGGAGAGATTCCCGGCCACCACCGCTTGTGTTGCCACGACGCGCG

GGGAGAACACCGCCCGctcaggggagggggaaggcggtATGGAAGACcggttgtggcggt

ggtggtagcggtggtggtggcggcggcggcaatagtggtggtggtggtggcgatgcagTA

ACGGCAGTTCTGGCGGAGGCATCAACCAAAAACAGTGCGTCGGCTAGGCAGAACTTGGGG

GGCAGCCGAACGCGATTAGGGAGTGCCAAGACGAACGCTGGTGGTGCCGTCTCAACGTTG

ACTTGCGTGGAAGGTGCTAGCAGCACCCGCAGCGGCCGCCGAGGCCGCGCTGCGTGCGCG

AAGAGGGGGACGCGTCGACAATCACCGCACCATCCCGACCGCTCCCTACCGCGAGACACG

AGAAAGCATAGCACGCGCCACAAAAGAGCGGTGATGGGTGTCCAGGGCAGCCGGTGCGGC

GGCAGCCGTGAAAGCCATGGCAGGGCTGCCTCGTCTCATTCCGACCACAGAAAGATCGTC

GCCAAAACGAGCGCCCGTCTTTCGTGCCGCAGCAGGTCCAGACGAAGAAGGGCGCCTTCT

TCCACAGGCACCCCTCTGGAAGCCCCCGTCCGTGTACTCGAGCCGCTGCCAGCCACGGCC

ACAGCGGGCGCAGCAAGCGCGGTGTCAACTCCGCCGGCAAGGGGACGAAGAATACGGCAG

CCGCAGCCGACCATGCTGTCCTTGAGCACTCGCCACAATAAGCGCTCGGGGTCGCTGCAA

CCAGCGCCTGGGTGGGAGCTGGAGGCTTCAGGCTCTGCGTTGTGA

>g5408.t1

ATGAGGCCGGCATTGTCGATCACTGCCGCCCGCAAAAGTCCACTATCCTTACTCATCTTC

CACCGTCATGGCGACAGGTCTCCCCTGAAGGGCCACACCCCCGACGAGGCGCTGACCCTG

TCTGAaggtgggggtagtggtggcagcggtggcggtggcggcaacagTAGCGACCCCGgc

aggggggtgcggggggtttcggcggtggcggtggggacaAGGGCAACGGCGGAGGCGTTT

TGGAAGCGGGAGCTGGTGCCGCTCGAAGATGTCCGACGGCTGGATGTGTTGTTTCCCGTG

TGGAGCTGGCCTGTtggggaggacgaggaggtgGTTTTGCCGCCCGACGAAGTGTCGGCA

CCCTTCGGGTGCCTGACATCTCTGGGGCTGCAGCAGCTTCGGGACCGGGGAAGGCAGCTC

CGCCAACGGTACTCTATTGATGACGTCACCAGGACTCAGATGGAGGTATTTTCGACCAAT

TACCGACGAACCCAGCTCTCTGCGCAGGGTTTCCTGGACGGGTTTCGAGATGGAGAAGGC

GGCGTTCCCGTCGTGGTCAGGCCTCGAAGCGAGGACTTTCTCAACCAGTGGGAGAGCCAA

GGGACCGACATGTACAAGCTCATGATGTCCGTTGAGTCCGAACCTTCTTTTCGGGAGACG

GAGGAAGCCGTTGGAGGGCCGTTGAAACGGCGGCTTCACGCGCTGGACCCGGCGCTTTTC

CCGTTGCCTCTCGGGGAGAGATTCAGGTGGATGATGGCGGCCGACTACTTCATGAGTGCA

CGCGCGCGCGGCCTCAGCATCCCCCCGGAACTGGACAGTCTGGGCGCGGCCACCATTCGG

CACCTTACGTGGCGCTTCGGCCGTTTCTACCGCGACGAGAGGATGATGAGGATTATGGTC

GGTCCCCTGCTTGCGTACATACTAGAGTGCGCAGCGAAGAGTGATatcgacggcgacggc

gagACCGGGACGGGAAGTACTGTGTCCGTCGAGCGAGGCGGGTCGTTGTCGCGCGTGGTG

TCCTCCAGCTGCCACGATGTGACCATCCTGGcgctcctttacgccatggagGCACATCTC

CTCGACGATGAGGACTACTGGCCCCCGTACGGGTCCACGATAGCGTTCGCGGTTTCGAGA

TCCCGGCAGGCGTTGCGACCGGGGCAGGGGGAACAAGGCCTGGTCTTGTCAATCACAGTT

GACGAGGAGCCTCTTCGATCACGCCTCtttgagggggcggggggggctgtcCCCCTGTCC

GATTTCCGAGCCGCCATGAAGCATTTTCTGCCGCGATAg

>g5598.t1

ATGGTgctgtcatcattgtcatcggtGGCGAGTGCTTCCACTCGCCAATGCCTGGTCGCT

GGACGCAGGAGGGTTCGTGGAGGAGCTGTGCTCGGTGGCGCCGTCCCGCTCCATTGGCGG

CGATTTCTCAGCGATGCTTCTGGGAACGGGCCttcgccccctccaccccctcgtgCTCCC

GGCTCGACCCAAACGGCGGGTGAGCGGGCCTGGAcgtggtcgacgccttctccGCTTCGG

CCGGGACGAGGAGCAGCGGAGATACCCATAatccccagAACGAACCTCACGGTGGAAGAG

GTGGTGAAGGCGATAGAGGGGCAGGGGGCGGTGGACGTCCGCGCGATCGACCTCCGGGGA

AAGGGAGCCGGGATGGGAGACTTCATGATCTTCTCGACAGCCACGACGCCCCTGCATATG

CGGCGTCTGGCGAACATGGTCGTGCACGCGCTAAAGAAGCGCAAGCTGGTGAACGCCATG

GGTATCACGGGGGCGGAAGGGATAGACTGCGACGACTGGATGCTGGTGGACTGCGACAAC

CTGATCGTGCATTTCATGACGGAGGAGGCGCGACAGGAGCTCGACCTGGACACGCACTGG

GCGAACGTGGCGAGCGGCTCGGCGTTGAGCTcgaagcgggggcggggggagaaggggcaC

GTGGAGGACCCCGTCGATTGGGAGGAGGATTTCGGGCAGGAGGCGGTGAAGGCGGCGGAG

CGACGGGCTAAATGA

>g5728.t1

ATGAAGTCTGCTGATTTCCTCACGCTCGCGACGTGCCTTTCGCTGGTGCATGGGGCATGG

TCGTTCACCCCGAACGCTCCGTCGTTTGGCGGCAGAAGAGGTGCCCAGCAGCATAGCCCC

GCAAAGCTGCACCACACTGAGTCGAGAGGTCAACAGCAGATTCTccaagcaggcagcagc

agcaccaactggTTTACTCAGTTTTTGAAGCCcggagcgcaaaaaaaaacgaacgggaAT

GGCGCGGAAACTGCGAAACTGAAGCAGCGGCTTCTCGACACCGTGCGATCCACCAGGCGC

GGTATTTCCACAAGCGAAGAGCAACGGCAGGACATCGATGAGTTGATCGCCGCCCTGGAG

CCTTTGAATCCTAACGCCGAGTTGGTCTCGAGCGAGAGCCTCAGCGCGCTGTGGATCTTG

GAGTGGACGACGGAGCGGGAGATCCTGTTCTTGATGGACAAGGGCTTGCTGGGGCTGCCG

TCGGGACCGGTGCAGCAGGCGATAGACGTCGACGCGAGGACCTTGTCCAACACGATGCTC

TTCGGCCCCGACTCGGTGTTTGAGGTGGCGTCGTCGATCGATCCGGAGGACTCAGGGCCG

CGCGTGAACTTCGAGTTCGAAGCTTGCAAGCTCAAATACCGAGGCTTTACGGTGCCGTTA

CCGCCAGTGGGCAAGGGCTGGTTTGAGAGCGTTTATCTTGACGAAGATTTCCGGGTGACA

CGAGATGTGCGAGGCGATGTAACCGTGCTGGTCAAAGGCCAGAGCTGA

>g5811.t1

ATGTCGCGGAGGCTTGCGGTTACCCTTGTGTGCGGCTTGGCTTTCCTGGCGGGAAGCGGC

GTGTCGTGGAGTCGGAGCTCTGGCAGTCGGAGCACGGCTGCGACAACTGCTGGTGGGTTT

TACCAAGTCGGAAGTCACACCGGCATTCCTCTTGCCGAAATTAGGCCTGGCGAGCCATCG

TCCGGTGTTATTGGTGAGGATGCTGGTGCTGTCGCGGCCCTCGAATTCACGGCGCTCAAC

TTCTACCACGTTCGGGATGGGAAGCCTGGCCAGGACTACCCTTGGCTGAAGGACGTTAAG

CTCATCGAGCCGCACCGCGACACGACCCTCGCAGTCGTGGGGGCACGAGAGGGGTTCGAT

TATCGCTGGGAAGTGCGTGcggctggcagcagcagcagcagcaacaacagcagcggctcg

GGCGGAGTAGAAGTGCAGGCTACTGCCACAGGGACGGTGACCATTGTGGTCCTCACGCAG

CTAGACGAGCATGTTGTGGTGCTGGAAGAGGTCGATGGGGATGGCAAGGTGACGAACCGG

CTGGAGGAGACGGTGATGGTGAAGTACGTGCGCAGGGAGATCAGGACGCTGACCGAGGAC

GAGAGGGAAGAGCTGCTGGATGCGATGTATCAACTGTGGGCGGTGCGGGTCGACGGTGGC

AACGGGAAGGAGCTTTACGGTGAAGACTACGCGGACATCTACGCCATCAACCGGCTGCAC

TTCAAGGCCACTACCAACGGCACCTGCGATCACttccacgacggaatgGGCTTCCTGGTG

AGCCACTCGCTCATCACCAACACTTTCGAGTTCAGCCTGCAGCGTGTGAACCCCAAGCTG

ACCGTCCCCTACTGGGACTTCACGATCGAGTCCACTGCTGCCGCGGATATTGCGTACGAC

CCGAAACTGCCTTTCACCAGGACGGAGTTGTTGAGCCCCTCGTGGTTCGGAACCGCTGAC

CTCAAAGACAATATGCTCAAGGACGGCCGGTGGGCGTACACCGAAATCCCCAGCGTAATA

AACGGCAACCCGGGCTTGCTTGAATCGGACGTCTACGGCAAGCTACGTTCACCgtggaac

accaacaacagcccGTACATCTCCCGCGCAGTGGGCGAGCAGTGTCAAGCCAGTGTCGAC

GGATACCTAACCTGGCCAGACTGCCAGGCGCACTACGAGCTCGTCACCACCAACCATGAG

TTCTACTCATTTGTGTGGGGTAGCATGGGCGACCCGCATGGGCCGGTTCACTTCTGGCTG

GGAGGCAATATCGACTGTGACACGACGTACAACAAGATTGCTACCCTTGTGGGCGCCGAG

ATCGCCGAAGTGTTCGCGTTTTTGGCAAACGGCCACCGTAAAGGACTGTTCTGCGAAAAG

GTGTGGGGCTGCAAGAGGACCGCGTCTGTGGACGAGAAGCCCGCCGAGCTCATGTCGAGC

GGTACCTGTGGCTGCCAGGGCTACGACCTCACCCAGGGGGACGACTATAAAATTATCATG

GAGTATCTCTCTTACGCCGAGTTTTACATCGGGGGTTTCAGCGAGGACATTCAGCGGCAG

GTCATCGAGATCATCTGCTCCGGGGTGGTCAACTACGGCGAGCACTCACAGTCGAGCTCC

TCGCTAGACCCGATGTTCTGGCCGATGCACGGTACGATGGAGCGGATGTACCAGTTCGCG

GTGGTGACAGGCCAAGTGACGGAGTTCACCTGGCCGGACAAAGACATGACGGTTACCCTC

CCCGACGGTACGAGCTACACCCAGTACGTGAGCAGCTACTACGAGGAGTGTAACGGCCAC

CACGGGAGTCACATCTTCCCGTTCGGCTTGTTGGGCTCAGACATCGACGGCTTCAAGGTC

AAGACGGGAATCCGCAGCAACCCCGCCACTGGGAACAACCTCACCAACCGCGAGGTGCTC

GAGGCATTCGACCCTCGATCCAACTCCATGAACTACGTGTACGACACGTTCAAGTGGGAC

CACTGTGTGGCGGAGGGGTACAACTTCGAAGATGCCTGGGGTGAACCCGCGACGTCGGCC

AGGAAAGAGTTTTTTGAGAGGCAAGAACCTCGGTCCGCCGTGTACACAAGCTTCAAGAGG

AAGATGGCTGTGCTCATGAAAGAGGAAGGAGATCGACTGAAGGAGGAggcgcaggggggg

gagagcgggggaAAGGCATCGAGCGGGAAATGA

>g5812.t1

ATGGGACAGATGCTTCAACTGTGGGTCGTACGGGTGGACGGGGGCGACGGAAAGGAGATT

TACGGGGAAGACTACACCGACGTGTACGCCTTGAACCGCATGCATCACAGGGCCTCTGCG

AGCGGAATCTACAACAAATTTCACGAAGGAATGGGCTTCCTGGTGAGCCACGCGCTCCTG

ACCAACACCTTCGAGTTCAGCCTGCAGCGCGTGAACCCCAAGTTGACCGTCCCTTACTGG

GACTTCACGATCGAGGCCACCGCTGCTGCAGGTCTGGCCTACGACTCGAAAAATCCGTTC

ACCCGGACGGAGCTGTTGAGCCCCTCGTGGTTCGGAACCATGGACCTCAACGACCATATG

GTTAAGGACGGACGGTGGGCGTACACGGAGATCCCCTTAGCCAGGAAAGACGTCGAGGCA

GACGTCTACGGCAAGCTACGCGCTCCGTGGAGTACCAGCGACAAAGCGTACATGTCCCGC

GGATTGGGCGAGATGTGTCAAATCAGCTCCAACGTACACCTGCCTTGGCCGGACTGCGAG

TCTCACTACGACCTCACCACCGGCAACGCCGActtctactcgtgggtgtggaaTAGCATG

AGCACCCCGCACGCGCCCGTTCATCTATGGCTGGGAGGCATCCTCGATTGCGACACGATG

TACAACAAGATTGGCGACCTTGTGGGGCCCGATATCGCCGAAGCTTTGGCGTTTTTGGCG

AACGGCCACCGCAAGGGTCTGTTCATCGAACACGTTTGGGGCTGCACTGGAACCGCTGCT

GTAGACGAGAAGCCTGACGAGGTGATGTCGAGCGGTACCTGCGGCTGCCACGGCTACGAC

CTCACCCAGGGGGACGACTACAAAACTATCATGGATTTGCTCGCTTACATGGAGTTTTAT

ATCGGGGGCTACGGCGAGGACATTCAGCGTCAGGTCATCGAGACCCTTTGCTCCGGGGTG

CTCGACTACGGCGAGAACTCACAGGTgactggggAGCCGCCCTACTCACCCTCACCACCC

CTCGCCGTGTCGAGCTCAACGCTAGATCCGATGTTCTGGCCGACTCACGGTACGATAGAG

CGCCTGTGGCAGTACGCGGTGGTGACGGGCCAAATTACGGAGTTCACTTGGCCGGACGAA

GACACGAACGTCACCCTCCCCGACGGCACGAGCTACACCCAGTACCTCTGTGGCAGCAAG

ACGTGCGACGGCCACCACGGGAGCGACGTGTTCCCCTTCGGCCTGTCTAGCTTTGAGGTT

GATGGCTTCGAGGTTAAGACGGGCATCCGCAGCAACCGTGTCATCGGGAACAAGCTCACC

AACCGCGAGGTGATGCAGGCCTTCGACCCTCGATCGAACTCCTTGACGTACGTGTACGAC

ACGTTCAAGTGGGACCACTGCGGGCCGGAAGGGTACGACTTTGACGACGCCCGGGGGGAG

ACTGTGCCGCCCTCCCCCCAGAAGAAGTTTTTCGAGAGAGACAGACCCCAGTCCCCCTCG

TACACGAGCCTGAAGAGGGAGATGGCCGACCTCATCAAAGAGAAGGGGCATTCGTTTaag

gagaagaaggggggggtaggagagACGTAA

>g5815.t1

ATGGCTGCTCCCGACGAGGGCCCGTCCTCTCATCACAAAAAATATGGCTCGGTCGACGAC

ACGGAGCAACCCACCTCTCACAGCAGCTTGCTCCCCAGATCGCCGAGGTGCAGCATCACT

ACGAACAGACATACTGATACCGATACCGCgggggaacagcagcagcgtccgccACAACAG

AACAAGAACGGTCTTCGAGACTCTGAGTCTGGGAGACGTGTTTCGCGGAGGCTTGCTGTT

GCCGTAATATGCGGCTTGGCCTTGCTGGCGAGAAGCGGCGTGTCGTGGAGTCAGAGCTCG

GGCAGTCGGAGCACGGCTGCAACAGCAGGTGGGTTTGTCGAAGTCGGAGCACACACcggc

gttgctgctgccgaCATGATGCCTATTCACGACCCCGCgtcttctgctactgctactact

gctgctgctgctgcaaccctGGAGTTCACGGCACTCAATTTGTACCACGTTCGGGACGGA

AAGCCTGGCCAGGACTATCCTTGGCTGGCAGATGTCAAGCTCGTCGAGCCGCACCGCGAT

ACGACCCTCGCAGTCGTGGGGGCGAGAGACGGGTTCGACTATCGCTGGGAAGTGCgcgcg

ggcagcagcagcagcagcagcccaggtGGATTGGGGGAAGTGCAGGCTACGGCGACAGGG

GCGGTGACCATTGTGATGCTCACCCAGCTCGATGAGAATGTTGTGGTGTTGGAGGAGGTG

GACGGGGACGGCAAGGTGACGAACCGGCTGGAGGAGACGGTGATGGTGAAGTACGTGCGC

AGGGAGATACGGACTTTGACGGACGATGAGAGGGAGGAGGTGCTGGACGCGGTGAGCGAA

CGTATCGAAAACAAGTAA

>g5984.t1

ATGTTCAAGACTATCGCCCTCGCGGCCCTGGCGGCTCTCATGGGCGCCGATGCGTTCGTA

TCACCGGTGGCCACCAGCTTTACCGGCAACGCTGTTGCCATGCGgACCAACGCCGCGGCA

AAGGCCTCCACCAAGCCGATGAAGATGTCTGCGGCGGACGATGCGTTCGTGCCCGACATG

GACCGCCGTACGGCCATGAACCTCATCCTCGCGGGCTCCATCGGCGTGAACGTGCTTGGC

TTGGCCGTGCCGTACATCGCTTTCTTCGTGCCccctggtggcggtggcggcggggggggc

gttatcGCGAAGGACGCCATCGGCAGTGAGGTGACCACCGAGAGCTGGTTGGCCACGCAC

GGGGTGGGGTCCAGGGAGCTCGCGGAGGGCATCAAGGGTGACGCAACGTACTTGGTGGTG

ACGGAGGACAAGAAGATCCAGGACTACGGGATCGTGGCTGTATGCACTCACCTGGGATGC

GTGGTGCCGTGGAACAAGGCGGAGAACAAGTTCATCTGCCCTTGCCACGGGTCCCAATAC

GACGCCACCGGAAAGGTTGTGAGGGGTCCCGCACCTCTGTCGCTCGCTCTCTCGCACGTC

GAGGAGACGGACGGCAAGGTCATCTTCAAGCAGTGGTCGGAGACAGACTTCCGTAACGGC

GAGAAGCCGTGGTGGTCGTAG

>g6083.t1

ATGTCGGGGTGttggacagGCGCCTACACGCCTCTGGAAGCGGCCGGGATCCTCATGGCT

ATGTTCGGAGCTCAGAGCATCATGTTGACCGGCAGGGCCGGCTTGATGGCGGTTGCTGGC

GAGAGAGTAGCCGCGAGGTTAAGGAATCTGGCGTTCGGCTCCATGGTCGTCCAGAAGACG

GAATTTTTCGACCGAAATAgGACGGGAGACCTGGTGAACAGGCTGGCTAGCGACGTTTTT

CTTGTGCAGGGCAGCGTCACCTCCCACGCCGCGCAGGGTCTGCGCAACCTTCTGATGGTG

GTGGGATGCACGGGGATGCTGACCTATCTGTCCCCGCAGCTGGCGGTCGTGAGCGTCGCG

GTTTTCCCGCCCGTGGCAGGCATTGGCGTGTGGTTCGGCAGGCGGATGAAACGGCAGCAG

AAGGGGGTGCAGGAGGCCCTGGCGGCCAGCAGTAGCGTGGCTGAGGAGGTGCTGTCCAAC

ATCCGAACCGTGAGGCAATTCAGCGCCGAGCTCCGCGAGGGAGGGCGGTACTCTTCGAAG

GTGGAAGACTCGTACCATCTGGCGGCGAAGGTCGGGATCACCAACTCGTTTTTCCAGGGC

TCCATGCACTTCGGCGGGCACGCTTCTCTCTGTGCTGTCATGGCTCTCGGCGGGCAGCAG

GACGTCCACTTCAGCTACCCAACACGCCCGCATGCCTCCGTGCTCGGGGGCTTGACGCTT

GAGGTGGAAGCGGGCTCGTCTCTGGCGATAGTGGGCGCCAGCGGGTGCGGCAAGTCGACG

GTGCTGCGACTGCTGACGCGCCTGTACGACCCGCAGTCGGGAACGATAGAACTAGACGGC

GTCCCGCTGAACACACTGGAGCCCAGAGGCCTCAGGGGCAGGATAGGGGTGGTTGAGCAG

GAGCCCGTGCTCTTCGGTGGGAGCGTTGCAGACAACATACGCTACGGACGACCGAGGGCA

AGTCAGGAGCAGGTGGCGGAGGCTGCGGCGGTGGCAAACGCGTCGGCATTCATAGAGGGG

TTTCCGGATGGATACGACACGcaggtcggggaggggggcgtccaGATGTCGGGAGGGCAG

AAGCAGCGCATCGCCATCGCGAGAGCGGTGCTGAAGAACCCAGCAATCATGCTCCTCGAC

GAAGCCACCAGCGCCCTGGATAGCGAGAGCGAGCACCTGGTGCAGGCGGCGCTGGAGCGC

GTCACCGAGGGACGGACGTCCTTGATAGTCGCGCACCGCCTCTCCACCGTGCGGAGCATG

GCCGACAAAATCTGCGTGCTCAACAAGGGGGAGGTGGTGGAGGTGGGAAGCTACGACGAG

TTGGCGAGCAAACCAGATGGGCACTTCCGACGTCTGGTCCAGTACCAGATGCTAGCGtaa

>g6113.t1

CGATGCGACGCGcgaagcggcggcggtaggggcTTATTACCCAACACTCGCCACCGTGGG

GGGTCGAGACCATCCCAGACTTCCATGATGGCCGAGAAGAACCGGAGAGCCCGGAGAATT

ATCGAGAGCCGCAAGAAGAAAGCGGCAACCGCAAACGCTCCCACCAAGCCCACCAGGTCA

TCGTCAGACGCTAAGTGGCTGCAGGTGGTGTCGGGAAAGTACAAGGGCAACGACAAGTTC

TTCACTCTTGCCACATACGGAGGAAAATTCTTCGCCGTGAGCGAGGCCTGCGGCCGCTGT

AAATTCCCAATGATTAACGgcagagttaaggttctccTGGGCGATGGGAAGGCGGAGGAA

GTGACGGAGGGGTCGGAGGAGGACCCCGAGGCAGATGTGGGGATCGGCTGTCCCCTGTGC

GGAGCGATGTTCGACATGAGAACAGGAGCCATCGCTGGGGAGCAGCCTAAGGGCCTGGCG

CAGACCTTCGTGTCCAAGATAGTGTCCCAAAGCAACGTCGAGAGCGTTACGACCTATCAA

GCGCAAGAGCTCTCTTCCGGGGCTATCGTCGTGCGCGTTGACTGA

>g6429.t1

ATGGCCTTCGTTAAGTTTCTGACTGTGGCGGCACTCGCCACGGTCTTGCTCCTGGACCTG

GCCGTACAGGCTGGCGCTTGCAGATGCTTGCGTAGGAGCCTGTGCCAGAAATACGAGGAA

GCAGACGTCGTGGTGCGTGCAACCGCGCTGTCAAGGACGGGGCCCGTGGAACGCAGGACA

ACGTACATTTTGGACCCCACAACCATCTACAAGGGGGTAGGGCTAGTCAACCCAGGCCAG

GAAATAAGCTTCGACACCAACGCCAGCGGCGCCTCGTGCGGGGTCAGCCTGAATCTGGTC

AGGGAGCACCTCATCGGTCTCCACCATAACGGCGGCGGCTCTTTCTCGGCTAGCACGTGC

GGCCTATTGAGTGATTGGGAGTTCGTCACCGAAGAGGACAACCGCTCCCTTGAGACTGGG

TGCGAGGAGGAAGACCCGTGCTATGGACAGTGTGGCGAATTTCAGGAGTGCCTGAAATAC

GACGACAATCTCCCGGACCCGTACTACTGCTCCGATGTGTGCGACCCGAGCCCGTGTGTT

GATGGACGCCCCTGCACGTTGGTGTTCCTCGGGGTGTGTGCGCGTGGCCGATGCCCGGCC

GAGGCCCACTGCTACTGA

>g6514.t1

ATGTTGCGGTCAGCAGGACGAGAGGTCTTCAGGCAGTGTCGCCGAGGGCCAACAACGGCA

CCTCTTGCAATCCGACTATTCTCGGCTGAGGCTGGGGGGAAGGCATCTGGGCTCGCGGAG

CTCATCACCAAAGATGAATACGTGAACTTTCCGAGAGAGAAGAGCGTATTTTACTTCACG

GCGAAGTGGTGTCCGCCCTGCCGCCGAATTGGACCGTTTTTTGTTGAGCTGAGCGAGGAA

ACGCCCTCGGTGTCCTTCGCCAAGATAGACATCGAGGACAACGAAGCAGCCGCTAAAATG

GCCGGGATCACGTCCGTGCCCACCTTCAAGTTCTTCAAGGCAGGGAAGGAGATCGAAGCA

ATCACAGGTGCTGACGCAACTTTGTTGGAACAGGCGGTGGAAAAGCTCGCGGAGTCTTGA

>g6550.t1

ATGAAGACCATCAAGGAGCAAGTCGGAGGGCGCCCTCTCAGCGCGGTTACGCCGGAGAAG

AAACTCATGGACCCCTCCAGCAGGCCCGTAGTGGATGGGAAGGCCCAGCAGCTGTGGGTT

GCAAGCGTGTCCCCGGAGGCGACGATGAGTTGCATTGTCGAGGCGTGGAAGGTGCTGCCG

CAGGAAACAAGGGAGGAGCTGAAGCGGCTGCTCATAGATTCGGAGAAAAACATTGTCCCG

GGGGGGGACACCGCAGGGGGCGTCGCAAGCACCCTTCGCCCCCTGAACGGCTACGCGTAC

TCCAAGCCAGAGCCGCTGGCGCGCGCTCCCGAGAACGTGTTCGTGTTCAGAGGGAAAAAG

CAAGAGGTCCTCGGCTGGCGCAACGTGTTCGACTTGCCCATCGAGTACGATGTCGAGCTG

TGTCCGGGACTGCTGTCGGCGGAGTCGCCTACCCTCTCCTGCCGCTTTCTGCCCGAGGGG

GGACCTCGCCGCCGCTTCATCGTGATCGACGAGGCTGTGGAGAAGCTTTACGGCGCCAAG

ATGTCGGCCAGCTTCAACAACCATGGCGTGTCCGTGCACAGAGTTGTGCTACCCGGAGAA

GAGGCTAACAAGCGCATGGAAGCCGTCGACAAGATCCTGGAGGAGCTGTGCCGCTTCGGC

CTCCGTCGCCGCGAGCCCATTCTGGCCATCGGCGGCGGGGTGCTGCTGGACATTGTCGGC

ATGGCTGCCTCGCTCTACCGGAGGGGGGTGCCTTTCGTGCGCGTGCCGACGACTCTGCTC

TCGCTCGTCGATGCCTCGGTGGGGGTGAAGAACGGGGTGGACTACTGCTCGTGCTCCATG

GGGCCGCAGAAGAACCGTGTGGGGACGTTCTATGCGCCCGTGGGTGCCCTCCTGGACAAG

TCCTTCATCGCCACccagGACGAGCGCAACATCATCAACGGCCTCGGAGAGATCATGAAG

CTGGCGCTCGTAAGGTCCACGGAGCTGTTCGGCTTGCTCGAACAGCACGGCCCCCGCCTT

GTCCTACAGCGCTTCCAGGGCGTTGATGGTGTGGCGGACCGTGTCATCGAGCTGTCGGTG

CAGATCATGCTGGAGGAGCTGGGCCCCAACCTATGGGAGCACAGGCTGGAGCGcTGCGTG

GACTACGGGCACACTTTCAGCAAGATCATCGAGATGCTGCCGGGTGCCGACATCATGCAC

GGTGAGGCGGTCAACGTCGACGGCTTTCTTTGCCTCGTCATCGCCAAGCGCCGCGGGATG

ATCTCTGAGGCGGTGCTCAACCGGGTGTTTGGGGTGATGAAGAGCATCGGCCTACCTACC

ATACACGAGGGCGTGGAGCTCGCGCTTATGGGACTGGCGGATGCCGTGGAACACCGCCAC

GGAAAGCAGCGCGTGCCGCTCCTGAAGGACGGGATCGGCCACTCAGTCTGCGTGAACGAC

ATCGATGCCAACGAGCTTACGGGCGCCATAGCCGAAGCCTGGGCCCTACACGGCGATGAC

GACTCGGACGACATGACCATCACCATGTAG

>g7367.t1

ATGGAACCGTCGCGACCAGTATCGCCTGGGGAACAACAGCGGCATAACCGGCACTCACGT

GAAGGGCGTAGCAGCCTAAGGAAACGACTGGGCCGTAACCCCAACGCTAGGCATGTCCAA

TGCTGTTCCCGACACCCCGTGGTCTTAGGTGCATGTCTCTTGTTCAGCGTAATCCTCACC

GACCCGATGAGCACGCTGTTTTCGAGGCGATCAGGGGGGTGCATGGGAGTAGCCGCAGTA

CCTTGCGATTCCACGAATGCCACCGCGGCGACGACGGGAGCTCCGAAACTGGTAGCTGTC

ACTCCTTCCGCGATATTGAATATCACGAACATGTTCGCCTGCGAGGACGGAGATTTCGAG

GTATTCTGGTCAGGCGCTGTCAACGTGCCTGAAACTATCGTCATCGGGAACCGCACCAAA

GTCAGGATTATTGGAGACAAGGGGATGGCCAGCAGTACGTCCATAGACACAACAAGCGCC

AGCAGTGGtaccgaggaggggggggacagCAGCTCGAGCGGCGCGGATAGGCTTCAAAAC

CTGACGACGAGCAGACTCTCCCTCCCGAGCGGGCTCTCGTCTGAGGCGGTGGGTGTTGGA

CCCCCCGACAAAGATAAGCTTAACGATACCACCACCGTGTTGTCCGTTCCACTTTTTCGC

GTCGATGGCGGCACGCTTATCATGACCGACTTTATCGTTCGCGATGGGTATGCGGTCGAC

CAAGAAAACGACGACAACTCGAAGGGAGCCGGCATCTTCGCGGTGGATGCCGAGATAAAC

GCCACAAGATGCGTATTCAGGAACAACTTCGCCGTGGGCGCGGGCGGCGGCATCTACGCG

CAGGATTCGACTGTGGTCGCGGTCGACTCCGTGTTTGAAGCGTGCAGGGCTGGGTTTAAA

TCGATTTCTGGAGAGGAGGACGCGGAAGGCGAGGGCGGCGGCATCTCGGCCAAAAACTCG

TACGTCCTGGTCGACGGCTGCCTGTTCGAGGGCAATTACGCTGGCAACAAAGGAGGCGCC

ATTAATCACCAGGACAAGCAAATTTCTGTAATCGGTTCGGTCTTCTACGACAACCTTGCC

GGGAGCAAAAGCGAGGAAGACAAGGATCCGAAAGGACAAGGCGCCGACCTCTCCTTGGCC

GAATGCGAGGCATCCTTCGGCGGGGAATCGCCGTGCTTTGTAAGCGATACGGTGTTCGAA

AATGCATACGCAGGGAGCAAGGGGGGCTCGGTGTCCCTGTCCGGCGGCGACGACCCTTGG

ACCGTAGAGTTTCACCGATGCAGCGTGGAGAACAGTTCGACTGGGTTTGATTTCAAGGAC

GACccgcaaggggaggggggcgcgttTTCGGTCGGCGAGAAGGTGACTCTCGTCTTGTCG

GGCTGCCTCCTCAAGGACAACTCTTGCGGCAAGAAGGGCGGCGTGTTGGTTATGTCGTCG

GGGGATAGCTTATCTGAGCCTGGCGCGACCGTTATCATGCGCAACTCGTCCTTCGAGTCG

AACGCGGCAGACCGCGACAGCGGAGGAGTCGCGGAAATGGCTAAGTTTTCCATGGCCATC

GTTGAGGGCGACGACAACGTGTTTGACGGGAACGAGTGCTACGGAGATGGTGCCGTTTTT

GCGGCGTCGACCGACACGTCGATCACCATCGAGGGAGGCTTGTTCATCAACAATCACGTC

GAAGAGAGTGGGGCTGTGATTTGGTCCAAAGGAAACCTGCTCATCTTGGACGGCAACTTC

ACCCGCAACCATAGCCCGGAAAACGGCGGCGTTCTCTTCGCTTCCGAGGGTAGCAATTTC

ACCTTGGCAGGCGGTTTTTTCCAGGAGAACGAGGCTCAAGATGGCGGCGTTCTCAATGCG

ATCGAGGGTTCGATTGTTCACGTCGAAGGTGGCGTGTATTCGGGAAACGTGGCGTTGAGC

GACGGTGGAGTGTTTTCTCTCCTCGATGGGGCGAATATCCAGATAACGGGTGGAAACTTC

AGCGGCAACAAGGCCGACTTCGGTGGGTTTATGTACAGGACTGGGCAGGGTGACACCTCG

TGCGAAGGGACCTCCATACTGGCCCACGAATCGGTCGACGGTGGCGCCATCTACGCTATC

GACGGGGCGACCGTGCATTGGGCGTGCGACATACGAGACAACTCGGCGATATCAGCGGCT

GCCATCCACGCTCGGGCGAACACGACGGTTTATTTGCGGGACATGTCGCTAGTGGACAAC

CGCGTGAGTCGCGGCAGCGTGATTGTCCTCATCAGCAGCCAGCTCGAGACGTTTCAGGTG

GAATTCAGCGACTCCTTGGGCTCTGTTGATCTGGCGGCGGTCCAAGTGGACGAAGGGTCG

AGCTACATGGCCGAAGATACCTCCTTCATGGGGTTCGCGGCAGAGGGGGTTGTGTTCAGC

GAAGGGGAGCTGTTCTTGGACAACTGCGACTTCAGCGGTAGCACCTCTTCAAAACTCGTC

TACTCGGAGCCGAACTCTACGGTGGTCATCAGGAACGCCGTTCTCGGGGACCTAAACTAT

ATGAGCCTGGCCGCGAGGAGCTCCATGGTTGGAGAGATACCGAACGCCAACTCCTTCGTC

AACGCCAACCTCACCTGCGGAGTAGATGTTTTGGAGGACTCGGGAGGGACGGGGGCAGCA

TCGTCATCTGGCGCCGTGCCTTCTGTGTGCAGTGAAGGGTCTGCTTGCCTGAACGGAGAC

CTAGGCGTGTACTGCGAATGCTACTTCCCACAGAGGACTCTCGACAGCACCAAGGAGGAG

AGGTGTGTCAGCGGTGACGCGTCTGAGCTTGTCTTGCTCATGGACACTGAGCCGGGACAG

ACATTCCTCCCGGAACTCGTGGAGGGAGACCTTCTCCTCAGGTACGAGGCAAAAGCCGAC

ATTGCCGAGGCGTCTGCGAGCGCAAGCGACACCAGCGGGAACGGGGCAGGCGGTGGCGTG

GTGTGGACCGTGCAAGCGTTGACGGGTCGGTTGGACTGGACGATATTTCCGTCGACTGGT

TTGCTCCTGCCGGGACAAAGCATAACGCTGAGGGTTGTCACCCAGCCCAGGGAGGTTTTC

GACGGCACCGCCAACGAGACGTTCGGTGCAATCGACTTTCGTCTGGATTCCCACACGTCT

GACGCCGTCACCACCAGCGACGAAGACAATATCGGCGACAATGACGACAGTGGAGACTTG

CTTGCAGCGCAGGGAGTGGCTGCCACGGTTATGGTACAGGTCACGTACTACCATTGTGCA

GCCGGGAGCTTCTGGGACCAGACCTTCGACCGCGACAAATACTCTAATTCGAGCAGTGGT

GATTGCAAGATTTGCACGGAGGAGACGGACGGAAATCCAGAGGGCGTAGACTGTTCCAAC

GCGGGAGCAACGATCGATGCCCTGCCGATCAAGGCAGGATACTGGCGGGCCACGCGCAGC

CAGGTTTTCATCCGCGAGTGCTCCAACGAGAATGCCTGCACGGGGGGAGCGGTTGTCGAG

AGCGTGGAAGACTACTGCGGTGAAGGCTACGAAGGACCACAGTGCGCTGTGTGCTCCCCC

GGGTTCGGGAGAGGGGCGGCGAATGAGTGCCATACCTGCTCGGAAGGCTTCAAGATCGGC

ATGTACTTCCTGGTGGTAGTTTCAGCATTGGTAACCATCGTCATCGTGGCCCTCATCGCG

ATTTACCTGATTGGAGGCAAGGACGCGGTTTCGTCGACAGTTGCAAGCACGAAGGAGACC

GTTCTCACGGTGAAACGGCGGAGCGTCGGGATGACCAATTcatcgggcggcggcggcggc

ggcggcggccgcggcagcatcGGCTTCGGCCCTCTCCACTCCCGGCGCGGCAGCTCCTCG

GGGATGTTCGACCGCTCCAAGCGCGTCGGAGGGAAGTCGTCGGTGGCACACGAGGCAGAC

ATCACCTCTCTCGGCGGCggcatcgacagcagcagtggcggagcCATGGGAGGGAGCGAT

TCACGAACAGCCCCTCCCCACgacgttggcggcggcggtcgtgGGGGTGTCGGAATTCAG

AGACAATCCCGACTAAAACGCGGAATGGTTGGGCTGTCGGcggttttcggtttctcgcGC

CGAGGCAAGACCACCCCCGACGGTTcaagtgttgagttgacgtcgTCATCGTGGTTtgga

accaagcgcgaaggatCAGCCGGTGGAACCGGTACCGATCCCGGCGCCGCtagcctcggg

gggggggtgaccagtGCCTATGGAGGGGCTGGCGCCGACTCACGCGCGCGCAAACAGGTC

CCCGGGGAACGGAGCGATAGACCCACCACCGCCAGGAGGATCGGGCAAACTTTGGCTGTG

CTGCCCCTGTCGAAGGTCAAGATTGTGATCGTGGTCTGGCAAATATCAAGCGCGTTTGCC

GACGTCACGAACGCAGGTTTCCCACCAATCTACGACAAGTTTCTCTCGGTCATCGGCATC

TTCTCCATGGACCTGGGATGGATTCTCTCAGCGGCGTGCCTGGCAACAGACATCACGTTT

TACGACAAACTGCTGATTGTGACCATCGGGCCGATGTGCCTCCTGGGGCTTCTGGGAATC

ACCTTTTACGTGGGATCGAGATCGCCAAAACCCCGGGCCGCCGATCAGCCGGTCGTCAAA

TACCAGAGCTGGGCAAGCACCTCAGCGGATGCCAGCACGCGGGTTCCTCCCGGGGTGGAT

AGCACCTTCAGCACCCGCACCATCTCAGCAATTAACCCGACTCTGTCAGGTGATATGTTC

CGTTCAGATCCTGGAGTGGTGTCGGATCAAGGTCAAGCGGCGGAGGCCACAGGAGGTACG

GGCAGTGATGCTCCCCCTGCCAAGTCGTCCGAGGTGCCACCCGGCCGCCAGGCTAGCTGG

ATGATACGTGTGGATGAAGAGAGCACTGCGGGGTCGGGGCGAAATGCTACAGCCCCCACG

GGGCCAATACGAGAACCCATCCTCGGCTCAGAACAATCGCTGCGGAACCGCTGGCAAGCC

GGGCCAGGTACCGGAGCGAACGCCGAACAGAACCAATTATGGGGGCTGTTCGCACGACAC

ACCACGATGACCCTCATCATCCTTTACCTCGTCTACAGCCAGGTTTCCACTGTGGTGTTC

CAGACGTTCTCTTGCGAAGATTATCACGAAATAAACAAGAGCTACCTGAGGGCCGACAAC

CGCATCGAGTGTTACACAACCACCCACACCGGGTACAGGATATACGCCGCCTTCATGATC

TGCCTCTACCCCCTGGGTATCCCGGCGGCCTTCGCATTCCTGCTCGTACGCCAAAGGTCG

GCTATCAACCCGCCGACCGACACCAGGCTCAAAACTACCAGAGGGAAAGAACACGTCGTG

AACGAGAAGATCAAACAGCGCGGGTTGGATCCGACGGTCACCCCCACTGCCTTCTTGTGG

AGCGCCTACTACCCCAACCGCTACTATTTCGAGGTGTTCGAGTGCATGAGGCGTCTGCTG

CTGACGGGTGTTCTGGTGTTCGTGCCGGATCAATCGGGACAGGTAGTCTACGGCTGCATA

TTCGCTTTCATCAGCCTATGGGCGTTTGAACTCCAGAGGCCACACATAGACGGGTTGGAG

ATCCAGCTTTACCGAACGGGGTGTCTGGTCATCTTCTTCACAAACTTCTTGGCGCTGACT

ATCCAGGCCGAGCTGGCCCAGCCCGACTCGAGCAGTACCGCTGTCTACTCCGTGGTTCTG

ATCATGGTGCACATCCTGTTCATCCTGTCGATATGTTGGAACAGCTGGGCGACGATGAAG

GCCACTTTCAGCCGGAGACACGTTCAGAGTATGGTTCTTGGCGTCGACTTGGTCGACGAG

GAAACAGCGGACAACATTCTAGGCCCTGAGAAAGACAAGGGGGACAAATCGTCCGGGGGC

GGAGTGGCAGACTTCAAGACCAACGACGACGAAATGCTTCCGGCCTGGCAGACCGAAGGG

ATCGTGCAGCCCGCGTCTTTCGACAATCCAACTAAGTGA

>g7441.t1

ATGGCTCGCACTGCTTTTGTTGCTCTGGTGGTCGGCATGCTTGGCGCGGCCAACTCTCTC

GAGGTCATCACCCCTTCCGAAGGCCTGACGGTTGTGGCCGACAGGACCTACACCGTGGAG

TGGACCGGCACTGACTCGGACAACCGGTACGAGATCGACCTCTACTACTGCGGCTCCTAC

TGCATGGAGGACGACTGCGGTGAGTGGGTGACCGCCCTCTGCCCGTACGGCGAGACCGGC

TGCCCCGACAACGAGGGCGACTACGACATCGTCATGCCCGAGCCCATGGACGGCACCTCC

GGGTCCGGGTACAAGGTCCGCGTGATGGACATCGAGGACGAGTCCAACATGGACTGCTCC

GATGACTTCATCCTCCTGGCGTCCGGCGAGGCTCCCTCGGTGGGCGACTCGGACGGACCC

CGCCTGGAGGTGACGTCTCCCGAGGCGGGCGACATGGCCTACGCCGGCGAGGAATACACG

GTCGAGTTCGACTACGACAACGGTGTTGGGTCTAAGGTGGACCGGTTCAGCATCGACCTC

TACAGggccgacggcggcagcggcgactgcGGGTCGTTCGTGACCTCCGTCTGCGACAAA

GAGAGCATCGGCTGCAAGGACTCAATGGGCGACTACGACGTCGACATCCCCGAGGACACC

CCCTCCGGCCAGTACCAGATCCGAGTGGGCCGCTTCGAGGACCAGGAGCTGTTCGGCTGC

TCAGGGACGTTCGAGATCGTTGGCAATGGCGATGACACTGACTCACCCGATGACATGAGC

ATGTCCTACGCCTTCTAA

>g7757.t1

ATGGCACCGAAATATGTCGCATGCACGGTAGCGCTCCTCATGGGCGGGGCCTCCGCCTTC

GTGGTGCCGACGCCCGCGCTCAGGAACGCGGTCGCGTCCACACGCTCGGCGTCCAGCCCC

ATGCGCATGAGTGCCGGAGATGAGGAGCCCTGGTTCGCTGAGGCCGTGGCGGTCAACTTG

GTGGACGTTGACGAACTCTCGGCTGCGTCCGACCGGGGAACCGGCAAGCAGGACTTCCTC

GAGGCGGACCCGTACTTTGACCAGTCCAACATCCCCCTCAACACGTACAAGGCCAAGGAC

CCGATGATCGGGAAGGTCATCTCGGTGAAGCGCATCGTTGGCCCCAACGCCACGGGAGAG

ACGTGCGACATCGTAATCAACCACGGGGGCAAGATGCCCTACTGGGAGGGCCAGTCGTAC

GGCGTCGTACCCCCCGGTGTGAGCTGGAAGACCGGAAAGCCTAACGGCGTCCGGCTGTAT

TCCATCGCGTCTTCCCGTTACGGTGACGACATGACGGGAACCACGACCACCCTCTGCGTG

CGCCGCGCCACCTACTGGGACGACGAGATGGGCGCCGACGACCCGGCCAAGAAGGGTGTC

TGCTCCAACTACCTCTGCGACGCCACCCCGGGGACCAAGCTGAAGCTCACCGGCCCCTCC

GGAAAGGTCATGCTCATGCCCGAGGACCAGCCGAAGGCCGACCTCATCATGATCGCCACC

GGCACCGGAATCGCCCCCTACAGGGCCTTCGTGCGCCGCCTCTTTTCGGAGCAAACCCCC

GCCCGCGACGCGTACAAGGGGCAGGCGTGGCTCTTCCTTGGAGTGGCCAACTCGGACTCG

CTCCTCTACGATGCGGAGTGGCAGACGGTGCTCAAGGAGTACCCGGAGAACTTCCGGCTG

GACTACGCTCTCTCCCGCGAGTCGAACAACAAGGCGGGCGGAAAGATGTACATTCAGGAC

AAGGTGGAGGAGTACGGTGACGAGGTGTTCCAGAAGCTCAGCGATGGCGCCCACATCTAC

TTCTGCGGGCTGAAGGGCATGATGCCCGGCATCCTGAAGATGCTCGAGGGCGTCGCCGGG

AAGAAGAAGATCGACTGGGGTGACTTCCTCAAGGACCTCAAGCACAAGGGCCAGTGGCAC

GTGGAGGTGTACTAG

>g7761.t1

ATGAAGTTCGCGTGCATTGCGTTGTGTGTCCAGGCTGCCGGCGCGTTCATCGCGCCGGCC

GTGACCCAGTCCGGCGTCTGTGCGTCCAAGTCATGGGCCGCCAACACCGGCTCCGCGCTG

CAGATGTCCGCCGGGGCCGAGGTGGTGGTGAAGAAGCTCTCCGTGGGCGACCTCAAGGAC

GAGGACCTCAAGGGCAAGAAGGTGCTGGTGCGCTGCGACCTCAACGTGCCCCTTGACGGC

AAGACCATCGGGGACGACACCCGCATCCGCGCCTCCATCCCTACCGTGGAGTACCTCCTC

AGCAAGGGCGCCCGCGTGGCCCTGTCCTCGCACTTGGGCCGACCTAAGGACGGACCGGAG

GACAAGTTCTCCCTTGGCCCCGTGGCCGAGCGCCTGACCGAGCTCCTTGGAAAGGAGTGC

AAGATGGCGCCCGACTGCATCGGCGACGAGGTGGCCGCGATCGCCAACGGGCTCGGAGAT

GGGGAGGTGATGCTCCTCGAGAACGTGCGGTTCTACCCCGCGGAGACCAAGAACGACCCC

GCCTTCGCGGAGAAGCTCGCCGCTCCGTTCGACCTGTTCGTGAACGACGCTTTCGGCACC

GCCCACCGCGCCCACGGCTCCACCGAGGGTGTGACCAAGTTCCTCTCCCCTTCCGTCGCC

GGCTTCCTTCTCCAGAAGGAGCTCGACTACCTTGAGGGAGCCGTCAAGGTCCCCGCCAGG

CCCTTCGCCGCCATCGTCGGAGGCTCCAAGGTTTCCTCCAAGATTGGCGTGATCGAGTCT

CTCCTCGCCAAGTGCGACAAGCTCATCATCGGCGGTGGCATGGTGTTCACCTTCCTCAAG

GCCCGCGGACAGGGCGTCGGCTCTTCGCTCGTGGAGGAGGACAAGCTTGAGCTGGCCAGG

GAGCTGGAGGTGAAGGCCAAGGCCGCTGGTGTGCAGATCATCCTCCCCTCCGACCTCATC

GTGGCTGACGCCTTCGCGGCCGACGCTAAGACCCAGGTGGTGCAGGCGGACGCCATCCCC

GACGGGTGGATGGGCCTCGACAACGGACCCGAGGCGACCAAGGAGATCCAGGCCGCGCTC

AAGGAGTGCAAGACCGTGGTGTGGAACGGGCCCATGggtgtgttcgagtacgagGCGTTC

GCCCGGGGAACGTTCGCGATCGCCGAGACCCTCGCGGAGATCACCGGCGAGGGCTGCACC

ACCATCGTGGGTGGCGGTGACTCCGTCGCTGCCGTAGAGAAGGCTGGCCTCGCGCCGAAG

ATGTCCCACATCTCCACCGGCGGCGGAGCCTCGCTCGAGCTGCTCGAGGGCAAGGTCCTC

CCCGGTGTGGCCGCGCTCAACGACGCTTAA

>g7883.t1

ATGGCGCGTGGACAAGGAAAGAGGGAGCGCAGAGGCCAGCAGGCCAAGATGCTCCAGGCA

GTGCTGCTCGGCGCCGCCGTGGGCACCACGTCCGCTTTCGTACCGTCCGTGCGACCAAGC

TTCGGCTCCACCCCGGCAACCTCGGCCGTTTCCTCGAGCACCGCGAGCGCTCGATCGCTG

AGCTACTGGGCGCAAGCCAGGGCTCCCGTTAGAAGCACCAGGTCGCGGGCGGTGGAGGCC

CACGGGTTGCAGATGGTCTCGGCCGGCATCGAGAAGGGAATGTTCACGACCTCGAGCCCG

GAGGACCGCCGCGTGACGCCCGAGACCAGGGACGGCAAGGCCTACTTCAAGGTGACGTAC

GTAGTACTGGAGTCGCAGTACCAGAGCTCCCTGACGAAGGCGTGCCAGTCGATCAACGAC

AGCAGGGACGACGTGTGCGTGGAGTGCGTCGGCTACCTGCTGGAGGAGCTGCGCGACGCT

AAGAACGTTGCCGCCTTCAAGAAGGATGTTGAGACTTCCAACATCTTCATCGGATCGCTC

ATCTTCGTTCAGGAGCTAGCGGAGGAGGTCCAGAAGGTGGTGGAGCCCCTAAGGGACCAG

CTGGACGCTGTTGTCGTCTTCCCCTCCATGCCGGAGGTCATGCGCCTCAACAAGGTCGGC

TCGTTCACCATGCAGAACCTCGGCCAGAGCAAGTCGGTCGTGTCCGACTTcatgaaaaag

aagaagaagGAGGACGGCTCTTCCTTCGAAGAAGGCATGCTCAAGCTCCTCCGCACGCTG

CCGAAGGTGCTCAAGTACCTGCCTTCCGACAAGGCGAAGGACGCGAAGAACTTCATGATG

TCCTTCCAGTACTGGCTGGGCGGATCTCCCGAGAACGTGGAGAGCTTGCTCCTCACCCTG

GCTACGTCGTACGTGCCGGAGGTCATGGAGAAAGAGAGCCTTAAGAAAGTGGAAATCGCG

GAGCCTGTGCTGCTGCCTGACAAGGGCATCTGGCACCCCGTCGCCGACAAGGTCTTCGAG

AACGCCCCCGAGTACCTCGCGTGGTACGAcaagGAGCACGCGCCCGCCGCTGGCATCAAG

GCAGACGCTCCCGTGGTGGGCCTGGTTCTCCAGAAGAGCCACATCAACACCAAGGACGAG

TGCCACTACGTTGCTCTCGTCGCTGAGCTTGAGGCTCGCGGTGCTAAGGTGATGACCCTC

TACACGGGCGGACTGGACTTCTCCGGGCCCGTGGAGGAGTACTTCATCTCCGGCGGCAAG

TCTATCGTGGACACCTGCATCAACCTCACCGGATTCGCCCTCGTCGGCGGCCCTGCCAGC

CAGGACCACGCGAAGGCGGTGTCCACCCTCCAGAAGCTGAACGTGCCGTACCTGTGCACG

GTGCCGCTCGTCTTCCAGTCCTTCGAGGAGTGGCAGGCGTCCGAGCTTGGTCTTCATCCC

ATCCAAGTCGCCCTCCAGGTCTCCCTCCCCGAGATCGACGGCGCCATGGAGCCGATCATT

TACGCGGGCCGCGAGGGCGCCACTGGGCGCTCCGTGCCCCTGGCCGACCGCGTCCAGCTT

GTCGCTGACCGGGCCCTCAAGTGGGCTACGCTCCGGTCGAAGAAGAACAAGGAGAAGAAC

CTGGCGGTGACCATCTTCAGCTTCCCCCCTGACAAGGGAAACGTCGGTACCGCCGCCTAC

CTCGACGTGTTCGGCTCCATCCACACCGTGCTCACCAAGCTCGGGAAGGAGggGTACGAC

GTGGGAGACCTCCCCGAAAACCCTAGGGACTTGATGGAGAGGATCCTCAACGACCCGGAG

GCCAGGATCGACTCCCCCGAGATGAACGTGCAGTACCGCATGTCCGTCCAGGAGTACGAG

GAGCTGACGCCCTACGCCGGCGACCTCGTCGAGAACTGGGGCAAGGCGCCCGGGCACCTG

AACTCGGACGGCCAGAACCTCCTCGTCTTCGGGGCCAAGTTCGGCAACATCTTCATCGGT

GTTCAGCCCACCTTCGGATACGAGGGTGACCCGATGCGTCTCCTTTTCTCCAAGTCAGCG

TCTCCCCACCACGGTTTCGCGGCGTACTACACCTACCTGGAGAAGATCTTCAAGGCCGAC

GCCGTCCTCCACTTCGGCACGCACGGCTCGCTTGAGTTCATGCCCGGAAAACAGGTGGGC

ATGTCCGGAGCTTGCTACCCCGACAGGTTGATCAACTCTATCCCCAACCTCTACTACTAC

GCGGCCAACAACCCGTCCGAGGCGACCATCGCCAAGCGCCGCTCCTACGCCGCGACGATC

TCGTACCTCACGCCCCCCGCCGAGAACGCGGGTCTCTACAAGGGACTCAAGGAGCTGGGA

GAGCTGGTGGCCTCCTACCAGGGCCTCCGCGACAACGGCGCCCGCGGACCGTCGATCGTG

AGCGCGTCGATCGCGTGCGCCCGCACCTGCAACCTGGACAAGGACATCCTGGACCTGCCC

GGCGACGACGCGGACTGCAAGCTGATGACCCTCGATGAGCGCGACATGGTGATCGGCAAG

ATCTACCGCCGCCTGATGGAGATCGAGTCCCGCCTGCTCCCCTGCGGCCTGCACACCGTC

GGCGTGCCTCCCACCGCCTCTGAGGCAATCGCCACGCTCGTCAACATCGCGTCGCTCGAC

CGACCCGAGGACGGGATCAAGGCCCTGCCCCGCATCGCCGCCGAGTCGGTCGGACGCGAC

ATCGGAGAGGTGTACGTCAGCGCCGACAAGGGTAACCTGGAGGACGTGCAGTTGCTGCAG

CGGATCACCGAGGGATGCCGCGACTCCGTGCGCGCTTGCGTGGAGAGGTCGACTAACTCG

GAGGGGCGCATCGTGGAAGTTAACGCTTTCGCGGACTTCTTCAGCAAGAGCTTCGGTGGA

GGAAGCCCGATGAAGGCGGCCCTGGAGAAGAACGGCTTCAAGGGCTGCAAGGACGCCGAC

CTCGAGCCGGTCTTCACCTACCTCGAGTTCTGCCTCAAGCAGGTGGTGGCTGACAACGAG

CTCGGCGGCCTCATCGGCGCCCTGAACGGAGAGTACATCACCCCCGGCCCCGGCGGGGAC

CCGATCCGCAACCCTGACGTGCTCCCCACGGGCAAGAACATGCACGCCCTCGACCCCCAG

TCCATCCCGACGAAGGCTGCCGTCGACTGCgccatggtggtggtggaccgTCTGCTGGAG

AGGCTCTCGGCCGACCAGGGAGGGGTGTACCCGGAGACGGTTGCGTTCACCCTGTGGGGC

ACGGACAACATCAAGACTTACGGCGAATCGCTCGCCCAGGTGTTGTGCCTCGCTGGCGTC

AGGCCCGTGGCGGACTCGATCGGCCGCGTGAACAAGCTCGAGCTGATCCCCCTCGAGGAG

CTTGGCCGACCTCGTATCGATGTCGTGGTTTCGTGCTCCGGAGTGTTCCGCGACCTGTTC

ATCAACCAGATGAACCTGCTTGACCGCGGCATCAAGATGGCCGCCGAGGCGGACGAGCCG

GAGGACAAAAACTTCGTCCGCAAGCACGCCGTTGAGCAGGCCAAGGAGTTCGGCCTCTCC

GTCCGGGACGCCGCCACCCGCGTCTTCTCCAACGCCGCGGGGTCGTACTCGGCCAACGTT

GGCCTGGCCATCGAGAACGGTGGCTGGGAGGGCgaggagcagctgcagcagcagttcgtc

aACCGTAAGGGTTTCGCTTTCAACGCCGACAAGCCCGGCATGATGGAGCAGCAGGTGGAC

AAGTTCAAGATGGCGCTTAAGACGGTGGACGTGACCTTCCAGAACCTCGACTCCTCGGAG

ATCTCCCTGACCGACGTGAGCCACTACTACGACTCCGACCCGACCAAGGTGGTTTCCAGC

CTCCGCGACGACAAGAAAAAACCGGCTTCCTTCATGGcggacaccaccaccgctaacgct

cagGTGCGTACCCTCTCGGAGACCGTGCGCCTCGACGCCCGCACGAAGCTGCTCAACCCT

AAGTTCTACGAGGGCATGCTCGCCTCGGGATACGAGGGGACTCGCGAGATCACGAAGCGC

CTCCGCAACACCATGGGATGGGCGGCGACCGCTGGCGAGGTGGACAACTTCATCTTCGAA

GACGCGAACGACGTGTTCATCAAGGACGAGGAGATGAGGGACCGACTCATGAACCTCAAC

CCCAACGCTTTCCGGGACACGGTGACCACGTTCCTGGAGGCGAACGGGCGAGGGTACTGG

GAAACATCCGAGGAGAACATCGAGCTCTTGCAGGACTTGTACCAGGAAGTGGAGGACAAG

ATCGAGGGCGTTTAA

>g7997.t1

ATGACGCAGGCCAGCACGGAGGTTGTCCGGGGGGCGTGGTGGAAGGAGTACCTTACTTCG

GAAGAGGGAAACGCTTGGTTTGAGGCATGGAACCCACTAATGCAGGCAAGAATTGACGAG

CTGGTGCAGATTGCGGAGTCCCGAGAAGACACGACGGAGTACTACCGAGTGATGATGGTC

GGAGACAGCACCATGGAGCACCAGTACGGCGCCATCTGCTCCTTTCTCGGCGAGAGGGAA

GGGCGCCGCTTCGACCCTGAGGAGGCTCAAGAACGCACGCCCGGGTGCTGCATCGACACG

GCGgaagggggcggtggcggccttTGCTTCGACTACACCTGGTTCCGATTCTTCGACCCG

AAGACCGCAGCGAGGCAGGAGGTGGACGCGTACTACTTCGGGAGCGGACTGCACTTGTTT

CACATGGTACCCAGTGCCCCCCTGGAGCCCATGAAGATTTTGAtGTGGTTGCACTACGAG

GACCTGCTAGAGGCAACCATCGTGGCTCTCCGAGAAACTAATCCGGACGTCAGGATCGTT

TTCATGACCAACCACATCGCCTCCGAGCAGCTGTTCACCCATGAACGTGCTACCGTCCGA

GACGCCTACCGACTGGGAGAGGGGAACAGCACCGTCCGCGCGGTGTGCGAAGATCAGGTG

GAAGAAGTCGCCGTGGCAGAGTATGTCGACCTGCATTACTACGACTGGCGCGGCAAGCCG

GACATCGAGGAGTACATCCAAGGGGTGCCCGAGGGGGAGTTCACGGTGAAAACCTACTGC

GAGGAGGCCCTCTCGGACGGTCATGGCTGCCGCCAGCTCGTAAGGCGAGCGAAGCCGGTG

CTGGCGAGACTGGGCGTGCCTATCGTGGACGCCGCCCAGATCGTGGATGGACAGGCTTGG

GCTTCGAGGCGAGGAGACGGATGCCACTACCACCCCCTCGTACCGACGGAGGTATTCGCT

TTGCTGGGAGCGCTCGTctcgccccccccgctaccGCTGCTCCCTGCGAGCGAGGTAATC

GCAAGCAGGGGGGATGAAAGCGGCGATGGCCCACGTTTGGTCTCGCTTGGTGCGGCGTGT

GGTGAGCCCTGA

>g8075.t1

ATGACCGCGGAGGACGCCTCCGAGGCGGTGCGTCAGGGCGTCGACGGCATCTGGATCTCC

AACCACGGTGCTCGTCAGCTCGACACCACCCCTGCCACCATCGAGGTCCTCCccgagGTG

GTACAGGCGGTGAGCGGACGGTGCGAGGTGTACCTGGACGGAGGTATCTGCCGCGGCACC

GACGTGTTTAAGGCCATCGCTCTCGGCGCTAAGGCCGTCTTCATCGGCCGCCCCGTGCTT

TGGGGCCTGGCGCACAGCGGAGAGGAGGGAGTGTCGAAGGTCCTCAAGCTGTTGAACGAC

GAGCTGATCATGGCCTTGCAGCTGACCGGCTGCACCCGCATCAGCGCCGCCACCCGCGCG

ATGGTGACCCACCAGATCTCCTACTACTCCAAGCTCTGA

>g8519.t1

ATGAAGTCTGTAGCCGCCGCCGCGTTCCTCGCCCTTGCTGCCACGGCGAATGCATTCGTC

GTGTCTCCGTCTGCGGCCGGCGTGACTGCCGTGCCAGCGACTTCGCTTGCGAAATCGCCG

TTCATGTCACGTCGCGTGGCTGGCGCACCCACATCTACCGGAGCCCTCAAGATGTCCACG

CCCTCCACCTCGATGAACGACGGACAGCAGCCCACTCTGGAGCAGTGGCTGACCTTCGCG

GAGCCCAAGCTGCAGTCTACCATGATCGCGATGTTCGCGGCGTGCAAGGAGATCGCGTAC

AAAATCCGTACCGCGTCGTGCGACAAAATGGCGTGTTTCAACGAATTCGGGGACGAGCAG

CTTGCGATCGATGTGCTCGCCAACAACGTGATTTTCGAGAACCTGAAGGCGTGCGGTTGC

TGCGCAACCGCCTCCTCGGAGGAGACGCCTGTGGAAGACCCGATGGGTGGCGAGGGATAC

TCCGTGGCGTTCGACCCTCTGGATGGATCGTCCATCATCGACACGAACTTCGCCGTCGGC

ACCATCTTCGGCGTGTGGCCCGGAAACCGACTGGTTGGCATCAGCGGACGGGAACTTGCG

GGAGCTGGAATGGCCGTGTACGGCCCCCGCACCACCATGACGGTCGCACTGGAAGGCATG

GAAGGCTCGCACGAGTTCCTTCTCATCGACGACTTCTCTGGGCGCCACGGACAGTGGGTC

AAGACCAACAGCTTCACCACAATTGACGACGGCAAGCTGTTCGCGCCGGGAAACCTGCGG

GGAACCCAGGACAACGAGGGATACAACGAGCTCTTCAACTTCTACCTTGACAACAAGTAC

CAGCTGCGATACACTGGGGGAATGGTGCCTGACGTGAACCAGATCATGGTGAAGGGCAAG

GGTATCTTCGTGAACCCGGCATCTGCGGCAGCTCCCGCTAAGCTTAGGGTGCTGTACGAG

GTCGGCCCCATCGCTTACCTGATCGAGAAGGCGGGCGGCAAATCGTCTGACGGCGAGAAC

TCTGCTCTTGACATCAAGATCCCCCACACCGAGGTGCGCTCGCAGGTGGCTTACGGATCT

GTGGGAGAGGTGGACCGTTTCGAGTCGATGGTGGGCGTGAAGGCGATGGCGGGGATGGCG

TAG

>g8520.t1

ATGCGCCAGCTATCTAACCAACTTGCCGATGCAGAGCAAGTCGCCCTCGAAGCCGAGGCC

ACTCTCGCGACTCGCACAACGGGCGCCGTCCGAAAAGCGGCTGGTCACGAAGTAAGCATG

CTGTCCGACATGCACGGACGCCATCACAACTACCTCCGCATCTCACTTACAGAGAGGTGT

AACTTGCGATGTGTGTATTGCATGCCTGAAGACGGCGTCGATTTGCAACCCCAAACCAAG

ATGCTGAACCAGCAGGAGATTTTGAGGTTGGCCAGTATGTTCGTCGATGCCGGAGTCGAC

AAGATCCGACTAACGGGGGGCGAGCCGCTAGTGCGCCAAGATCTGCCTCAAATCGTAAAG

GCGCTCAGCTCGTTGGACGGGGTGCGCAACGTTGGCGTCACGACGAATGGAATCAATCTT

CGGCGGAAGATCCCTGCTCTCCGTGAGGCAGGCCTCACCCACATCAATGTGAGCCTCGAC

ACCTTGCGGCCGGACCGCTTCGCCGCCATCACCCGGAGGAAAGGCCTTGAGGCGGTTTTG

GCGTCCTTGGACGCAGCGCTGGCCCACGGGTACGGAGGGAGGCTGAAGATCAACTGCGTC

GTCATGAACGGAGTCAACACGGACGAGTTGGCAGACTTTTTGGATTTCACACGTGACCAG

GAACTCGACGTTCGCTTCATCGAGTGGATGCCTTTCGATGACAACCGCTGGAAGGACTCC

AAGTTtttctcataccagTCTATGTTGGACGTGATTCGGGAGCGATACCCCGACCTGGAG

AGGACTGTCGATTCTGCAAATGACACCACCAAGTGGCATCGCGTTCCTGGTTACCAGGGC

AGAGTCGGGTTCATCACGTCCATGAGCGAGCACTTTTGTGGCACTTGCAATCGCCTCCGC

ATTACTTCTGATGGCAACCTCAAGGTCTGCCTTTTCGGAGACGAAAGCCTCAGTCTTCGG

GATGCTCTTCGAGGAGGCCTCTCTGATGCAGAGATATCGGTCCTGGTGAGGGCCGCAGTG

CTAGGGAAACGTTCTGCTCTAGGAGGTCACGAAGACATGTACGGCATTGCCGGCGCCAAG

AACCGGCCGATGACCACAATTGGAGGATGA

>g8761.t1

ATGGAACAAAGAGTGCAGCTTGAAGGTAACGCTGGAGACGATGACGGGTACGAAGGGGCA

AAAAACATCACGGATGTCGAGAATAAACAGTCTACCGAGAGAGGGCTTGCGGTTGGAGGT

GAAATGGCGCGAGGTGGAAAGCGCAGCGTTTACGAGAGCGTAACGATGCAAGTGGTGGAG

GAGCTGCAAGATTCGGCGAGGGGATTCTTCGCGAAGATCTCGGCGGCATTTGAATCAGCA

GGTATTTACATTTCAAACCGAGCACAGGTGGACACGAAAATTGCTCGAGCCGCAGTGGAC

TACGCATCTAGTCAAGCAGCTGCAGGAACAAGCAGGCTTCTTCTGACAGCAACGAGTGCA

GCAAGTCCAGCTTTGCTTCTGATGGGAGTCAACACAACGGAGACGCAGGCCGCCTTGAAC

GACTCGCTTCAGTTGTTGTCGATGGGGATAGTGCCCGACTCGGTCGGTGTCTCGCCGAAG

CTTTTGCCGTCGTTCAAGGACTTCGACGATGATGGCAAAAGGCTTGACGCCGCATATAAA

CGGCAGCGCCGGCGCCGATCGAAGCCGCTGTCAGTCAAGATCCTCGGAGCGCTGCCGTCT

ACATTGAACAAAGCTGCAGACCTCGCGTACGAAGTGGAACAAGAGGTCAAGTACGAACGA

GCTGGGAACCGCGCGAACAAGCTCCTGAAGCCTGTTGGTCTTTCCCTCCCAGAGGCTAAA

GGCAGTGGTCTCCTCTTGGAACGTGGATCCGACCCGGCTGAGCCAAAACTTTTAGAGGCA

CCATCCGCCGATGACTCGCCCATCGCTGCAAATAGGTTGCCCACGTCGGCTGCCGAGTCA

GACATCGTAGAGGGTATGCTTGAAGTGGGGGTATCCACGACAGCAAGGGGTGGTCCGTTG

AAAGAGGCAGTGATGAAACAAGAGCCAACGCCACCGGCACCGGAAACTTTTGACGTCCTC

GACTTTTTCCGGCGAAAATTCGGTACTCCCGAGGGCGACCGGGTTTCTCCCGGTGCAGAA

GGCAAAGCCGATGGGGCCCAATCGAAAGTCGAGCTGAAATTGGCCGCGGCACTGACGTCG

GAGTCGTATGCTTCCATCAGCGAGGTGACGACGTGCTTGGCATCTGTCGACAGCGAAGTG

CTCACGCAAGAGAGCAGTCTGCAGGCATCGAACGCGGTGCTGTGCTCTTCAGAGCCCGTC

ATCGCTGGTCTTGAAGAGAGTTCCAAGAGAGACATGTTTCTACTGAAGGAGCTCCGGACT

GTAGCAAGCAATGTGCAGCAAGTGTACGTTCTCGCTGAGATGGCAGAAGCCGTCGAGGCA

GCGGAGGTGCACCTTAGGCGACTTGAAGAGTTGTTGGCAAATGTCGTCGACTTATCGGCG

GCATTGAGAACGTACGCAACAAAAGCGAATGTCAGGTTGGATCGACTGGTCGTCTTGAGG

ACAAAGTTGAAGAGATTACTGGACTTGGGCGGTGTCGCAGAAATGCAGAGGCTGGTAGAG

CGAGATTCTTCGTACGGGAGCCAAGAACTGCAGCTGTCATTTGACGGCGAAAAAAATGCC

TGCCACCGAAATAGAAGGGCAGTGGGGGAATTGTTGGGCGTCGTTTGGCGAGAGTCAGCC

GACCGGGACAAAGACGATGCGTCCAAATTGTTTGAGGGTGCTCTGGCAGCTGCATTTCGT

CTGTGGGAAGCGTGTGAAGCAGAGGGCACGAGTGAACCAGCCAATGTGATAGAGAGTATT

ACGCAAGAGTTAACTGCAGTGGAGAACGTAGTAGCTTCCACTGCGCCCACCCCAAACGCA

AGAGGAGCCTTATTCACTGCGAAGCCGAGAGATAAAGAGCGAAGGGGTGTGACTGAGGTC

GCTGAAGCAGAGCGGGAGGCCCTAGGTGCCGAAACGGAGTTTGCACGAATAGAGATGAAC

GAGCAAGCCTCCACGACGGAAAACAGCGACCAGCGAGAACGCACAGCGCAGATGGATGGA

GGAATCACAACGTCAACTACGAGTATCGATGGCGACGAGCAAGTGCAAGATTCCCCAATT

GGTGCGGCCCCGACGATATCCCAGCCGGACATCGCTCCCGTCACTGTCGCGCCGGAGGCC

ACCGTGGAAGATAAAAAAGTGGGTGTTGACCGGGAAAAGGTGTCCAAGGTTTCCAAAATC

AACGTTGGCGCTGTCGATGCCAACATCGTGAGTGATGCTACCGTTGTTAGGGGCTCGGTC

GCAAGATCGCTCAATACACGGAATATGAGGCGAGCAAGCGAGGAGGAAAGGCCACGTTCT

GGTTCCTCCCCATCAGGAACGTCAACGTCCCACGAGTCTAGTATTGAGGTCGTCGTGGAC

GACACCAGTTCATTTGTAGATATAGAGGCCGAAGTAGAAGACATCTCTGACCAAACGAAA

GTTGGCTTGAAAGTCTTGGACGTCTTTGCTTTGTTGCTTGAAAAGGTCCTCTTTGTTGGT

CTCCCGATGGTTCTATCTGGCGGAGCTCTTGTCTGGGAAAGAGTGGACAACGCAATGAAC

GGCGCAGAGGGGCGCAAAGGGTGGAAGCTCCTCAGCAAACTAAAGCCTGACCCGGGTGGG

AATGACGAATCTAGAGAATAG

>g8897.t1

ATGGGTGATCCCGCAGTTGAGGGACCCCGTCGGTCAGATAGTGTGAACGCCTTCTATGAG

CATAACCTACGCCGCTCCCGGCCGGTCGTAGTCTACCTTACGGGAGCCGATGCATGCATG

CTGTGGGACGAGTTGAAGCCCCAAGAGGTGGAAGCAAACGCTGCGGAAACTTCTGTGCTT

AGCAACATTGGCTCTGCGGCGTCGGCAGCTGCAGGTGGTAACACACCACTGGCTATCGTC

ACGTTCGGCTGGGACCACCACGGATCCCCGTTTGCTGCTCCTGCATTCCGCGAGTGTAGC

ACCAATATCCTAGAACTGCCGCAGGTCATCGGCTACAAGTTTTTCTTCGACGAGAACACT

GCCGTTGACTCTCCCGAGGCCCTCGCGGCACGACTGGTGGGACTGGAACGCCTATTCGAG

GAAATCCGGGAAACAAGCAAACAGGCGGAGGACAGGACGGGCACATCATCGCCAGTGGTC

ACACCTGCTCTATCATCGGGAGTCGATGCACCATCGTCCATGCCCTCTGCATCGTCAGGG

ACCGCACTGGCCGCTCGCGAAATGTGCGTGACACGCGTCTCCACGGAGGGCTGCCCCAAC

GCTGATGCCAATATTGACAATCTTCACCCGGTCATGTCGGTCTTAGTCGCAAGAGCACAA

TCACGGGCCAACCCTTTGACAGGACGTGCGGATTTCGCGCCCGAAAACTTCGACCAAGCG

CTGGCGTTCACCGACTTCATCTTCGCGACCGGCAGTTGTGACCACACAGGCCTCTCGGAG

GAGACGGATCGAGCCATCACGATCCCCAATCGGTACCAAGACGCCATCAACTCCCCGCCG

AGCGAGCAGTGGATAGAAGCTATCACCGAGGACATGGATAGCCTGAAGGGGCATGAGGTG

AAATTCCTGCAATGCAAGATCACGTCCAATGTGTACGTCAAAGTGGCCCCGGGACAAAAG

GTCACGGACCCGGAGACCGGCGTACCCACGGTGTACAAGCTGCAACGCAACCTGTACGGC

CCAGGGCAACCACCCGTCCTATGGCACGGTTCTATCGACGCGGCACTGGTGACCCTGGGC

TTCACGCCCACGAACTATGACCCTTGCGTGTACACCCACGGAAGCAACGACACACGTGTG

ACCCTCAGACTGTACGTGGACGGCATACTGCTCagtggaggggaggaggaagcaCTGGAG

AAGCTAAAGACGGCGGTCATGGACCACTTAGCCATGATCAACATGGACGAGGTCAGCCGT

GTCCTAAGTATGACCGTCACACGGGATGAGAAGGGCACACTGACCATCGACCAGAAGGAA

TACGCACTCGCCACCTTGGAGAGATTCGGAAGCCTGGACCCAAACCCCGTCCACACGCCG

GGGTACGAACTGAAACTGTCCACCCAACAGCCGGAGAAGAAGCTGTTAGGCGCAACAGGA

ATCAAGCTGTACCAGGCCATCGTGGGTAGCTTGCTCTACACAGGCCCCGACTGTTTTCGC

CACTATGAGTTCCTGCTGGCTGGCAACATCGTGTACGTGCCGGACGATCCAAGCTTACAC

ATTGTGCTCGCCGGGCTGCCGGTGATCTTCGTGCAAGGGCGAGAGGATGGGTGGGGAAAG

ATTAACTGCGACAATATGGTCCAACACGCTCAACAGGTCGCACACCGGCTCGACCAAGGC

CTCAAACCGGGGGGGAAGCCTCCTTTCGACTATGGAAAACTCACGCTTCGCCACTGGATC

CGGTTCGTGCGCGACCGCGTGTGGCAGGCCGCAGCGGAAATTCGTCAGGCGGGTGATGAG

GCTAGGTTGACAGGGTACGAACCTGAAGAATCCGAGGAATCCCAGCCGTATCCAGAGATG

GTCGTCGGCGGATGA

>g8927.t1

ATGAAGACCCCGTCTTCCATCGCCGCACTGCTCCTCGCTGTAGCACCATGCGGTGCCTTC

GTTCCCGCCATGGTCGGGCGCCGGGGGGCCGTCCATGCAGGAGgacgtcccgtcccgtct

accTCTCCGCTTAGGTCGTCGCCGGCCGACGCAGCGATCGACGCTTACCCCTCGGACGTT

TCGACTGAGGACGGAACTggcgccgcgccccccccggcgGACGAGTCAGACTTCATGGCC

GTCGAGCTTTTGCGGACGATTTCCGCCGGTGTCGGGGAAGGCTACATGCCGTACTCAGAG

ACGGACAAGAGCGCTATCGAGGACCTGGTGGTGGAGCTGGAGTCTTCCGGGGAAGGCACG

GATGTTGAGTTCCCTAGGGACTTGGAGAAGCTGGATGGCCGCTGGAGACTGGTGTTCACC

AACAACCTGGTCGGGCTTGGGAAACTGTCTCCCCTCGCCCTCAAAGACGTCTACCAGGTG

GTGGACTACTCTTCCAGCCTCGTGACCAACACTGTCTACGCCGTCATGTCCCCGCCGTTG

TTCGCGGAAACATGGGGAAGGCTGGGCCAGCGGGCCGCGGACCTGGCGAAGACGGTGGAA

GACCAGATCACGTTTCCGGTAGACTTCACCATACAGCACGACTTTACCGTGTCGTCGCAG

TCTAAGCCAGCGCAGATCGAGCTGGTACAGAAGGAACTGAAGATAATGAACGCGGAGGAT

GCCGGCAAGCGATCCCTTGCCCTTCCTGCTCTCAAGCCTCTGGCGAAGGCAGCGGCCGGT

CGTTTCGACACCACGTTTTTGAGCAATGGCGTGCGCGTCAGCCGCGGGCGTTTCGGCGAA

CTTAGGGTTTTCGAACGAGAGGCGTGA

>g8931.t1

ATGTGGCTGGCACACGCAACCTTGGTGCTGTCCTTGTCCGccatcccccctcccgtctca

CCGCTGCTGAACGGCATCCAGCCCCACGCTAACCCGGTAGCAGGAACAGCAAGCGAGGGC

GGCTCTCATCTGGGACTCACCACGTACGCAGAACGACCGacagcatcaagcagcagcacc

agcaccagtcgGGGTGGATGGTGGCTACAACACCACAGCTGTGCTGGGCGCGAACGACCC

ATCGCCGGAACCCTTTGGCCGCGAGGGAGCCCTGTGCTACACGTGACGGGAAAGGAGGGA

CGCCAGGAAGCACGTACCTGCAGATATAGCAAATCAACACAGCAAGATGTTGAGCTAACC

GCACAGCAGGGACAAGGACAACAGCAGGTGTACAATCCATTAGAGCAGCCgttagaggag

gaggaggaggaggaaggagaggaggaaggggacgaagaggaagaggaagaagaagaagaa

gaggaagaagagcaagagaaggggggagaggaaagGGGTAGGAGCGATCGGGCGAGTAAG

ACCCACTTGAAAAATGTCGTGGCTCGGTTGCAGGGCGACATTCAGGCGGACGCGACGCCC

CAGATGCGATCTCGCGTCAAGCTGGATAGTCTGTTCGCGGAGCTCCGGGCCGgcgagccc

cctgcccccgtcGCCGTCGCTCCTCAAAACTCGACTATGTCGGTGGCGCCATCAGCAACG

AAAACGCCGCCCTCATACCGCAAAGCACCCGCACCGAGAGCACCCCAAGGTATGCGcgac

acagccgccgccgccaccgccgccgccaccgccgaaacGCCTCCGAAGAAGCCGCTCCAC

CAAACCGACACCACCGGAGGTGGGTTGACTCCCCCACCAAGCGACGATCTGGAGTCCCTC

TCCGACATGCTAGACCGGCTTCAGGCCACAGCTAAGGCGAGAGACCTGGCGGAGGCCGGC

GAGCGCGCGGCAGCGATGAGGTCCGGGGGGCCCGTCAGCGAAGGGCTTTCCTCGGTCACC

ATGTCGAAACACAAGGACATGATGACGCGGTTCGCGAGCATCCGCGAGGGGAGGGTGAGC

TGGGCCATCCTCCCCCTGGTCATGCGCGCCCGCAAGGCCGGGATACCGCTGAGCACGGGG

GTGTACAACGCTGCCATCGGCGCGTACGTGAGCACCCCGAGGAAGTACACCGACGCTCTG

CGGGTGCTGGACCTGCTGAGACACTCGGGGGATCCCAGCGTGCGGCCGGATTTGACGTCG

TACAATACGGCCATGTGGGTGTGCGGGGAAGCGGGGCAGTGGAGGGTCGTGTACGAGATG

CTGGCGCAGATGAAGATCGAGGGCTTAGAGCCAAATACCGGTTCTTACAACTGCGCTCTG

AACGGCCTGGCGAAACGTCGAGAGTGGTACCGGGCGCGGCGACTGTTCCGCAAGATGGTG

GGGGAGGGTCTGTCTCCGAATGCCAAaagctacaacgggctggtggaggcggcggggatG

GGAAGCCTCTCTCCTCGCCAAAACATGATCCAgATCGTGTACGATATGGAACAGGCCGGC

GTGGAGCCAACCGCGTACACGTACACCATCCTGCTGGACTGTTTGGCACAGCGGGGCAAG

GCGTTCGACGGCTTCCAGGTCATGGCTCGTCTGGTGGACGCGCGCGCGCCTCTCCTCCCG

CTAGGGTACCGCGCCGGGCTGCTGTTCTGCAAAGAGTTTGGGGACTGGCGCCGCGCGATG

GTTCTTATGGAGGACATGCGCGTGGCTAAGCGCCGCCCCTCCGGGGTGATGTATTTACTG

GCGGTGCAGGCATGTTCGAAGGGGGGGCAGTGGGAGCGCGCCTTGTCTCTGATGGTGGAG

CGACGGACCACGGTGGCGATCGAGGCCAAGGAGCaattggCTCTCACAGATGGGCAGGTG

CCGGACCGGGCGGGGGGTTCGGTTTCGACGCAGTCGAAGACGCGGGCGGCTGAGCTGGAC

ATGAAGACCCTCGAAGCGGTGCTGAGCGCGGTGTCGGAAGCGGGGCAATTCGAAGTGGCA

ATCAGGCTGGTGCAGCAAATGCGTTCGGCGGGAGACACGCCAAGCAAGAAATGCTACCTC

TACACACTGCGCGCCGCGTCTAAGTGGGGTCGCTGGGACGTGATCGAAGGCCTCATGAAA

GACATGCGCGCGCTGCGCGTGGGACTCGTCGATCTCGAAACCTCCGGTGGCGTtatggag

gaggggggaggggaagggataAGGTTATTGAGCTCGGACTGTTACCCAGCTTTGGTGGAG

GCGTACGCTCAGGCTTCGATGTGGGAAAGAGCGATAGAGGCCTACCAGGAGGGGTTTGTG

GTGGGCCGGGAAGGGGTGGAGGCGGTGAACTACCGGGTTTTCGAGTGCGTGCTGAAGGCG

TGCGTGGGCGCAAAGGACGGCTACACAGCCCTCGAGGTGATCAGGAGGCAAGCCGCGGAA

CCAAAGCGAGCTCAGGGATCACTGTCATCCCCGTCGAAAGGCGGGGAGACCGGAGAAGTC

AGCGGGGGACCCCCGGATCGGCGGTGCTGGTGCCTGGCGGCGGAGGCGTTGGGGAGGGCA

GGCATGGTGGAGGAGGGCCATGACATCCTGAAGGCGATGGTCAACTCCGGGATACCGGTG

CGAGAGTCGACGAAGCAGGACGTGCCATCTCTGATGCCACTCCCGACCTGCGAAGACGGT

TTCGACTGGCATCAACGAAAGCGACGGAGGCGTCCGCGACGATTGGGGGCTAGCCGGCTG

TCTCCCCGAGGCTTGACGGTCAAGAGGCGGGAGGCGGCGATCCGGCATTCCTTGGATACT

TCCGGAGAGGTCGTCGTCGCGGGAGAAAGCGGAGACGGTGCCGATCCTTCCAAGAACTTT

CTGGCCGGTGCCGAAGTTTCCAAGAATGTTCTGGCCGGTACCGAGCTCTCCAAGGGCGTC

GTCAACGGGGAAGggtgtgcggcggcggcggttgctgcCGCGGGGGGGCAAGCGAGCAGC

GGGGGCAAGCGGTCGCTGGGGCTGGGGTGGCTCCTGGTTAGCGATTGGAGGTACAAAAAC

TCGGCGATGGAGCGCGAGGCTAGAGGCGGGGGACGCAGCGCGCTggagcggaagcggcgg

cgcaTGGCCGATTTTTCCCTGAAGGCAATTGAGATGCGGGAAAAGTTGAAGAgcgtcggt

ggtggcggtggttccGTCGGTAAAGGAAACGCTGTCGGAGGGGGACTCACCGACGGCGAG

gacgctgctgcgccggcggcgGGGGAAACGGCTCACCCTCAGATAGAACTGCGGCCGGGG

GGAAGGCGAGGCAGGAACGAAGCGCTGACGTTGAGGATGCGCGTCCGACGCACGCCCGGC

GTTTTGGGAAGGGGAGACGGCGGTATTATTGACCGGTCGCGGCGACGAGATGGTCGGCGC

CCGAACAGGACAGATCGGCGCGTTGCTGGGAACGGCGCCAATCGAAGGGAGggccgtggt

ggcggtgttggcggcagtgGGGGTAAAAGGCGAGGTGGCGGGCGGTGA

>g9224.t1

ATGGCAAACACCCACTTGGCCGCGCTGTTCGCCCTGCTGATGGCGATTGTTTCCGTAGAA

GGCTTCGGCTTGAGCGCGCCGCGACCGACCGCCCACACCCGGCGGACATTCGCCGCGGCA

TCGtcatcgtcctcgtcgtcgtcagGGATCCGACGACGAAATTCGGCAACCTCGACCGCG

ATGTTTTTCGATTTCGGGAAGAAAAAGACGAGCAGCGTAAACGCGAGGCCCGTCGCCGGG

GGGACCGTGAAGGGGGGCAAGAAGTCGAAGGTAGTCCAGGAGGTTAGTCCCGCGTTGCAG

CGACAGCGAGACGCCGCGCAAAAGAAGGTCTTGGCTCGGAAGGTTGTGAGGAAGACTGAG

GGTGTCTTCACTCCGCCGCAGTTTGAAGCCTTCAAGAAAGCGCGAAAAGAGGTGCCCTCC

GGCAGCGGGGACGCGTACTGGCAGAAGATAGCCGCCAAGGTGCCCGGACAGAACGCGGCG

GCCTGCAAGCGCGTGGCGGAGTCGATGCTGTTCGAGGCGGCGGTGGGCTCGAAGAGCAAC

TACTTCGGCGGTTACATCGAGGTCAACGAGACCCCGGACTACGTCGACCCGGAGGCGGAC

ATCATGTTCAAGATCAACAAATTGTTCGGCAGGGCACAGAAGAGCGCGGACAAATAG

>g9323.t1

ACAGGTTCACTGTACTCCGACCTTAGTTCTACCGGCAGGGTAGCGTTCATCGATAATGTC

GCCACCGATAAAGGGGGCGCCATGTACATTGCTCGGTATGGTCTGCTTCAAGTCGGCAAT

GCCATCTTTCGATCGAATCAGGCCGAATCGGGAGGTGCAGTTTTCCTCGTCTTCGGGTCA

GGCGGACAATCACACTTCACTGCTTCTTTCTTCGGGGGCAACAAGGCTAAAGATGGGGGC

GCGGTTTACTTGTACACCGGTACAGGGGTTGACATCTTCAACAAATGCGTTTTCCAAGAC

AATTCTGCAGGGTTTTCTGGAGGAGCAATCTTCCACACCGGACTCCTGCTAGTTTCCAAG

AGCAGTTTTGTGGCAAACGTTGTCGGCAAAGACGGGCCGGCCATTATGAGCGTTGGGCTG

ATGAAAGTACTGTCCAACGTGTCTTTCTTGGAAAATACGTACCGGTGCCCTGCCGGGCAG

TACGGTTATATCCTTGACACGAATCAGAGCACATTTTGCACTTCTTGCTTTGAAGAAGTG

TGTGCAAGATGTGTCAACGGGGGCGAGGGCGTACCAAATGGAGATCAAGTCCAAATCGAC

GGCATACCTCTCTGCGCAGCGCCACCCTCCGGAGTTACCGCGTTGTCAAGAGGCACAACC

CTCGAAACTCTTACCCTGGAGAAGGGCTACTACCGAACATCCAACCTCAGTCACGACGTC

CGGAGGTGCTCCAATGAAGAGGCCTGCGTTGGTGGCAACGACGCAGACAATTACTGCGCC

TCTGGCTACGAGGGCCCGTATTGTGCGATCTGTGCTCAAGGGTTCGCGCCTGGGGTTGCC

TTCAGCTGCAGGAAATGCTCCGGAAGTACCATGCAGTCGGCGATAGGGCTTGCAGTGGCC

GTCGCTTTGCTTGTGCTGCCGTTGGCGGCTCTGATGTTTTATTATCTGGGGAGTTTGGTG

AAGGAAGgagagcaggaagaggaggatcAGGATGAGGATGTGGAGGTGGCTGGCAGGGCT

TGGAAGCAAAAATGCTGGTCTTGGCAAAGGTTGGTGGTCAAGATGCTTCCGCTCACGGCG

ATCAAAATTGTCGTCACCGTCTGGCAGATCATTTCTCAGTTTGCCGCCATCGCTGGAGAC

GTCTATCCGGACATCTACACCAAGTTCGTTTCCAAGCTGAACCCGATCAATCTGGACCTC

GACTTCGTTTTGTCATATTCGTGCATAGTAGACACCGATTTCTACGATCGTCTGTTTTTC

GCCACGATTGCACCGCCTCTGGTGCTGTTAATGCTGGCCCTAAGTTACTTCATCGCAAAG

AGGCGAAACCGTAGCTCTGAGTCCGCAATGAGCGTGGTGCGGCACAAGCACCAGTCTGCA

GCACTTTATCTCGCATTCCTAGTGTATTCCCCGGTATCGTACAAGGTATTCCAGGCATTC

TCTTGCGACGAGCTAGGCGGTGGAGGCACATACCTTCGGGCGGACTACAGCCTTAGCTGC

CTAACCTCTAGTCACAGCTCCTACGAGGTGTACGCACTCGTCATGGTGTGTATTTACCCT

GTCGGCATACCCGCAGTATTCGCCTGGTTGCTTGCTCGTCACCGACATGACCTCGTCAAG

CCGGACCGGGCAACCATGCCGCACCTGCAGCCGCTGAACGGAATCTGGGGCGCGTACAAG

CCGTCTCGATACTACTTCGAGGTGGTAGAGTGCGGTCGCCGGATCATCCTGACCGGCATA

GCGGCATTTGTTCTTCCCAACAGCACGGCTCAAATCTCCATCGTTCTTTTggttgctttc

gtttttttgtttatatcgGAGTCCGTGTCTCCGTTTGAGAAGGAAATCGACACGATGCTT

TACCGTTGGGGCAACGGTATCATCGTGGGCAGCGTGTACATAGCTCTCCTTTTGAAGATG

GAGGTCGGCCGTGAATCGACAGAAGCCATCTCGGCATTTTCGGCCGTGCTTATCATCGCA

AACGTGGCCATGCTCGCCACGGTGATGCTTCAGACTGCGCTTTTGGTGAAAGAGTGGCGT

AGGATGGAGAAAGCGGCCAAGCCAGCTGAATCGCCTGTTCCCCGCCGGACGACACCATCG

CTATATTCCAGATACCTAGGTCGAGGAGACGAGGAGGGGGCAGGCTGGTAG

>g9415.t1

ATGATGACTGCCTTGCAGGTTCTCATCGTGTTCCTGTGTGTTCAGCAAGTATTTGGCCAG

CTGGATCAACTTCGTGCGGCGGCTCCTCAGAATTCCGAGCACGACGGCCATGGCGGGGAA

GTTGATCAAACTATTTTTCTCGCCGATGCCTTTAACAAGGCTGTCGCGGAAGGTCACATA

CCTAAGGGGTACACAGATCTCGgggccgtcgtcatcgccacagtcgagcatgagcTGCCG

TCTTTCGGTGTCGGCCTACCCTCGGTAGTTCGTGATCTTCAAGCACAAGACGCCGAGGAT

CTGTGCGTGACTCTCGAACTCACTCTCCCTGGATGCGACGACGCAACTCTGCCCCCACAA

ACCGTAACGGTGTGGTTTCTCCCGCCGGGCAACGCGGCTGATGACCTATCTGGGTGGCTG

GGTGTCGTTAATATTGAGTCGGTTGTATTTGAAGGTGGGAAGGCCACAGCAACCTTTGAC

GAGGAAAATCTCTATGATTCTGCCGGCGCCAAGACCGATTTCTGTGAAGACTGTGTCGTG

ACAGCAGTTGGGTCGAACCGAGATTGCGCCGATACCACGATGACAGCGTCCAACGTGGAG

GTGTCTGTGAAAGGTGACCCTCACATGGTTGGCCTGCTGGGCCAGAAGATCGACTGGGTC

GGGGAGGACAACATGTGGTACTGCCTCCTTTCTGACGGGCCCGACTTCCAGATAAATGTA

CGCCTGAGTGCTCCCATGAAGGACGAGTTCCCAGACAGGCAGCTAGTGTCCGCTATCAGC

CTGCTCACCAACGACGGCCACTCACTGCTGATCGAGGTCAAGGACCCTTACGTCACCGAG

ACTGATGGGTGTCCCCAGCATTCGTCAGCCCCGTGcctaggggaggggggcgtacgcaTC

CTCGTTGACGGAGAGGTATCCAGCCCTGTGCAAAGCCCAGGCGACAGGGCGCACCTGCCG

GGAGGTGTGGTATTTTCCGCTGCTAACCTCTTGCCCGAGTGCCGCCCGTTCGGCGGAGAC

CGTATCTGGGCTGCACACTTCGAGAACATGATGGTCGGTCGTCGCTCATTGCGCACCGTT

ACCCCTGCCATACCGTTCAAAGAGTGGATTCTCGCAGGCGACAGCCTCGCGGCCCCGACG

TGGTGCGCCAAGTTTTTGGAGGAAGGTGGAGGTACGGGGCTTCTCTCCGCGTCTACGAAA

CACATGACTGCTCGCATCGAGACGCAGACAGCCACGATCAGGGTCAACGTCGGCGTCAAC

TATCAAGACCTCGTGACCGGACCGAATGGAACCGTTCTGGTTCCTCAGCTGGAGTTCTGG

CAGACTGACCTCGTTTTTGACACGCTCTATTACACTGACGCAGTGACGGGACTGCTGGGT

GACACCTCTCGGTTAGTGCTCGACGACGAGGGATTGCCGGTGACGACCGGGCTTGGCGCC

CTTTACGCTCCGGTAGAAACCTACCTCGTGGACGGGCCGTTTGGTAGGGTCGTCTAG

>g9531.t1

ATGGTTCAAGCAGGAGTATCCATGCTCGCCTTCCTGGCTGCCGCTGCCGGGCCTGCCATG

GTGGCCGGCTTCGCGCTGTCCCCGAGCACGATGAGCGCTCGCCGAGGCGGCCTAAGCATG

GCTGCGGACAAGCAGAGGGTGCTGGTGATCGGGGGCACTCGGTTCAGCGGGCTGTACCTC

ACGAAGGAGCTGCACTCCAGGGGCCACGAGGTGGTCCTGTACAACCGCGGGAAGACGGCG

AACAAGCAGCTGCCCTGCGAGTCCGACGCCGAATACGCCAAGCGCTCGGCGGACGTCAAG

ACCATTATCGGCGACCGCAAGGACCCTGAGGTGTGCCAGTCTACCCTGGGAGGGGAGAAG

TTCGACGCCGTGTTCGACATGAACGCTCGGGCGATGACCGACACGAAGGCCGTTGCCGAC

GTGTTCAAGGGGAAGGTTGACCACTACGTCTTCATGAGCTCCGCCGGGGTGTACATGAAG

TCGGAACTCATGCCGCACCGTGAGGAGGACGCGGTGGACCCCCAGAGCAGGCACAAGGGC

AAGTACGAGAGCGAGGCCTACCTGGCCGAGATCGGCATGCCCTTCACGTCCATCCGACCC

ACCTACATCTACGGCCCGCTCAACTACAACCCCCTCGAGGAGTACTTCTTCGAGCGCCTC

GACCAGGACCGCACCATCATCGTGCCTGGGCACGGACAGCACCTGACCGGCCTCGGGCAC

GTGAAGGACCTTGCACGGGCGATGGCTAACGTGCTCGGAAAGGAGGCGGCCAAGGGACAG

GTCTACAACGttcagGACAACCGCGCGATCTCGTTCGATGGCATGGCGAGGGCTTGCGCC

GAGGCGATGGGGAAAGACCCGGCAGCCGTGGACATCAAGCACTTCGACCCGGCTGCCTTC

GACTTCGGCAAGAAGAAAGCCTTCCCCATGAGGCCGGGACACTTCTTCGCCAGCTGCGAG

AAGGCGATGACCGACCTCGACTGGGCTCCGGAGTTCAACACCGTGGATGGCCTCAGGGAT

TCTTACGAAAACGACTTCGTCCACAAGAAGGCGGCGGGAGGCCTGAAGAACGACTTTGAG

TGCGACGACCAGGTCATCAACGACCAGACCATCGACAACCTGCTCTCCAGAAATTAA

>g9646.t1

ATGAAAGGGATCGTCGCTTCTATGGCTCTCTCGGCCACAACGGCCTCGCTTGCGTCAGCG

TTCGTGGCATCTCCTCTCAGCCTGCGAGCCTTCACCAAGCCTACCTGCTCCGCGCGTGCG

TGCCGCGTCCAGCCTGTCCGCATGACGGCGGACGAGAACGACGATGGAGAACAGAAGGTG

TACTACGCGTCCGAAGTAACCGAAGGAAACGAGAAGACGACGTTCAACAAGGAGCTGGGG

GAGCAGCCCTTGCCGGAGGACACCACCGCGGGCGAGTCGGACAGGTATGGCCCCGTGGAC

TACTCTGGCTTTATTGATGGCGAGGGGTTCGACGGGGGCGACGGCCAAGTCGGATGCGTC

GGAGATGGCTCGAACGCAATGCAGGAGTTTGACAACAAGGCGGTCGGCCTTGCGGCGGAC

ATGAAGGCCAAACTCAAGAGCTCCACGATGGTGACAGAGTCCAAGACTCGCCAAAGAAAC

GCGTGGGGCGCCACGAACACTGGATACGCCGACAAGCTGAAAGAAGAGGGCATGGTCAAG

ATCAACCTTCAGGGGGAGGACATCAGCAAGATTCGCCGCCAGCAGTTCGAGaactGGCGT

AACCAGCAGGAGATCACGTCCAAGCAGCGTGCCGATATCAACGAGATGGAGCGCGTCACC

GCGAAGTCAAAACAGGATGACACCAGCAACAAGTGGAAGAGGGGCAAGGCCAGCTACTTC

GACGAATTGAACAAGCAGCAAGAGGAGGGCAAGGACGAAGACTGGAACAAGTACAGTGCT

CCGGTCACCTCGGGGAAGAAGGACGGAACCGAGTGGGAGGAGACAGGCCTTACCGGCAAC

GAAAAAATCGAGGACACGGTGCAATGCGTATCCATGAACAGTCGGCCCGGGCACGCAACT

ATCCAGGTAAAGAACACCGTGATGACGTTCGAGCCGTTCCACTGCACCTTCGTGGGCGAC

ACGCAAGGGTTCAAGGTGTCTCCCGTGGAGGGGACGCTGGAGCGCAGGGGTGGCGACGTC

ACCGAATTGGAGGTGTCTTACAAGGGCAATGGCCCCGGCGACAACCGGATCGGCACCCTG

GTCGTGGAGACGGAAGAAGACAAGTGGGTCTACAAGGTGAGACAAGGAAGCGTTTTCGTC

TGA

>g9653.t1

ATGGTTCTCTACCGTTCTGTGTTTTTGGCTGTGCTGTGTTCAGTTGCTGTAAATGCGTTT

GTGGCTCCGTGCGGCAGGCCGAGACTAGCAGAAGCCACCCCCAGGTCTCAATCCAGGCGA

CTAACTGGTTTGGAAATGGTGTACGGCAAGGTTTTTGTCGCGGGTGGGTCGAACGGGGTT

GGACGCGTCGTGATCGACAAGCTGGTGGAGCAGGGATCTGAAGTTGTGGCTCTCGTTCGC

AGGGAGGACGCGAAGGCGGAGCTGGATGCTATCAAGGGCGTGTCTGCCGTGGTCTGCGAT

GCGCTAGACCTGAAGGGCGTGGAGGCTGTTCTGGATGGCTGCGACGCAGCAATCACCACT

CTGGGAGGAGTCCCGGAGGGAGACGAGGCGAAACGAGTTGACTATGCGGGAAATCGTAAC

GTCATCGAGTCGGCAGGGATTTTGGGAATCACTCGAGTAGTCATGGTGACCAGCGTGGGT

TGTGGAAGCAGCCGCGAGGCTATTAGTGACCAAGTATACCAGGTGCTCGAGAAGGCGCTG

AAAGCGAAAACCCTGGCGGAGAACATGCTGCTCAAGTACTACACCAACTCGGAGTGGACG

ATCATCCGCCCCGGTGGGCTGAAGAGCGACGCTGCCACTGGAACAGCGATCCTTACAGAA

AACACCAAGGCTGCGGGGGTGATCAACAGGGCAGACGTCGCGGATCTAGCGGTGAAAGCT

TTGGGCTCGCCGCAAACCATTAGGAAGATTCTTACAGCAGTCGACCCAGGTACCACGAGC

GAGTACGACTCTACGGAGAAACTTGTGGCGTTCGACCTTTAA

>g9911.t1

ATGCCTTCCTCTTCCTTGGCTGTGGCCACAGCGGCGCTCTTGTCTCTGTGTTCCCGGACA

CAAGCATTCGTTGGCGTTCCTTCTgcctctgcagctgctgctgttgcgcgagGACAGCAG

TCGGCGTTGAGCATGGCCGCGAAGCCAGACCAACTGCCGGCTCAAGCCAAGCGCTACTAC

GTGCGCCCGGACAGAATACTGGACGTGCTTACGTCTGCTCCCCAGCTTTTGCTCAGGCTT

GGCAGCGGAGCACTGGTGGATGGATACCGATTCAAGGTGTCTAAgcaggaggagggagac

gagggCGAGTATGCTGTCGTCCGCGCTCTGGGGCTCAAGGTAACCGAGAGGGGAAACACG

TGGGACAGAGCGCAGCCCCGAAAACCCATCGAGATCTACGAGTTTGAGGGCTGTCCGTTT

TGCCGCAAAGTGAGGGAAGCGGTCAACATCCTCGACCTGGACGTGGTGTTCTACCCTTGC

CCTCAGGACGGGCCCACGTTCCGACCGAAGGCGAACAAGTTGGGGGGCTCAAAGCAGTTC

CCGTACATGGTGGACCCCAACTCCAAGACCTCCATGTATGAGAGCGACGACATCATCAAC

TATCTCTTCGAAACGTACGGCGAGGGGTCCAAGgttccGTTCCAGCTGTCGCTCGGACCT

CTAACCACAATCACCGCGGGTCTCGGGATGCTCCCGCGAGCTCTCAAGGGGTCAAAGTAC

ACACCGGCCAAGATGCCCAAGAAGCCTCTTGAGCTGTGGGGATACGAGTCCTCGCCTTTC

ACGAAGGTTGTCCGTGAAAAGCTGTGCGAGTTGGAGATTCCCCACAAGTTCGTTGCGGCC

GCACGAGGAAGCCCCAAGCGCCAAAGGTTGTTCGAGATGGCTGGGGCTGGCCAGACGCCG

TACCTGATCGACCCCAACACCGGCGCCAAGGGCTACGAGAGCTCTGAGATCGTCGACTAC

CTCGACGAAACCTACGCCCTCTGA

>g9946.t1

ATGAGTTCTCTCATGAAGAAAGCTGGGCTGATATTCGACGAGCATAAGACGAAGCCATTC

CTGAAGATGAGCATAACGAGGCTCCAGCTGCTCGTCAACAAGAAGTCAAACCACATCAAG

GTTAGCAAGAAAGAGATAGCCCGGCTTCTCGCGGACGGCAAAGAGGAAAAAGCGCGCATC

AAAGTGGAGCAGGTGATTCGCGAGGACTTTACGATAGAGGCGTACGACATCCTGGAGCTC

CACTGCGAGCTGGTGGCCGAACGAATGCGGCTAGTGGCGTCGCAAAAGGACGTCCCCCCC

GACATGGACCAAGCTATTTCCACGCTCATCTGGGCCGCCGATCGAGCAGAGGTGGCCGAG

CTGTCGACGGTGAAGGCTCAGTTTCAGAAAAAGTACGGCTACGACTTCGTGAGAGCAGCG

GAGCTGAACGATGGCGGCTGCGTCAACCCGAAGGTGGTCGAGAAGCTCGACTGCCAGCCC

CCTTCAGCCTTCGTCGTCACGGAATACCTTCTAGGGATCGCGGAGGAGTACGGCGTGGAG

TACACCCCGGCCGTCGCGGTCCGCCGGAAAAGCACCGAGGCCGCCGAAATCCCCGAGATG

CCTCAAGCCGCCTTTGAGGCGCccgcgcaccaccaccacccaccccctggCTCTAGcgga

ggcggtggaggtggcggcggcggcggcggcggcggtgggggtagcGGAATTTCGTCATCC

CCTCCCGTAACCGCGTGGGCTATCCCCTCGGATAGCATTCCTGAAACCACCGCCAAATTG

TCGCCGGAAGATTACTCTCacggcagaggaggaggaggaggagggggcggtggagcgggg

gggggcggggctggcatGCCCAGCCATTACCCGCCAAACGAGGGGGAGCCTGACCCCTAT

CAGGAGTCGGGCAGGGTTCATTCGTTGCCCGGGGGCTTCGAGGCCGCTGTTCAGGGCTGG

AACCCTTCCGAATCGGGCAGGATTAAAtacgacggggggggcgggggcggcggggagtac

GACTCGAACACTCCACCCCCTCCTTATACGCGTACACCCTCTCAATACGACCACAACGAC

ATTCCGTCTCCACCGCACACGAGACCCGTCATCTTCGATATTCCTGCGGCACCCGGCCAC

GAAGACCCTCCCGAAAAGGGGCCGGACACGGGAGAAACCAGCCCACCGGCAAGTTCGAGC

GGGCAAACCGAAATGGACGACCTCGCCGCAAGATTCATGAGCCTTCGAAAGTGA

>g10009.t1

ATGAGCACGACGACGAGCAGCCTTTCCGAAGAAGTTGGTGCTTCTCCTCGATGGGAGGGG

TTCTGGTCCTGCGGCCTCAAAAAGGGCGATAAGTGGGACACCGGGACTGTCTCTCCTGCT

CTCCAACAGCTTCTAGACCAagGTGTTCTCCCCAAGGGCCGGGCGCTTGTGCCCGGCTGC

GGCCGTGGGTACGACGCGATCGCTTTCGGCAAGAGCGGGTACGACTCGTTAGGCCTCGAT

CTCTCCCCGACGGGGGTGGAGCAGGCCAAAGATCTGCTCGCCGAGGAGACGGAGAAGCCC

AGCGGCAAGGTGACGTTCCGTTCAGGCGACTTTTTCAAGTTTTCCTGCTCGGAGGAGGGG

AAATTCGACGTCATCCTCGACTACACCTTTCTCTGCGCGCTCGACCCGAGCATTCGGAAC

GACTGGGCTGACCACATGGTATCGCTGCTTAGCCCCGGGGGAGAGCTGGTGACATTGATC

TTCCCGATCGTGGAGAAGGAGGACGGACCGCCATTTGCGGTGAGCGAAGCAATTGTCGCT

GGCCTCTTAGAAACTCGAGGGCTCGAGGCCGTGCGCCTTGAAAAGCTCCCGCCTAACCTC

TGCCacgaagggagggaagggaagacCGCTCTCGGGCGTTGGCGATTCTCAAAGGCGGAC

TAA

>g10026.t1

ATGGAGAATCTGTCCAAACCTCGAGGACGGTGGACTCTCCGCCGCAACAGCCTCGACCAA

aacccccaacaacaacaacaacaaaaggagaGGAATCGCGAGGAGGTTAACCGTTGCCGG

CAGGATTCCTCAAGCCTCCCGCCAGGATGGTGCCCAAAGCAGACCCTCTCGCCGTCTCCC

AGACCAGAGAGGAAGGTCTCGACCCCAAGGCGGCATTGGGCGGCACGCACGGCTATCGGc

gtaacagcggcagcggcagcagcagcgatggtcgCGTGGACCCTCCTCCTTAGCTCTGGG

CAGGAAGCGTCTATCAACAGCACGAACACAGCAGCGGCCACGCAAGAAGACGGTCGAAAA

ATGTGGAcagctgttgttggtgatgctTCGGAAGGGGAGGATGTGCGGCGCGCATCGAAG

ACGAGCAGCAAGGGCCGAGTTGCCGTGTGCTTCTTCGGCTTGGCGAGGTCGCTCCGCTGG

ACCGTGCCGAGTATCGAGCGGCGCTTGCTCGGCGTGCTTCGGGAGGACGGGTTCGAGGTG

GACATTTTCCTGCACACCTACAGCCTACTCGAGGTGGACAACACGAGAGCGAGGGAGCAA

GGGATACAGTACGCCCGCTTCCAGAACGACTTTCGTGTTCTAAACTCGACGAGGTCGTTG

ATTACCAACCAGGACGACCTGGACCTCGTGGTGCCCAACCCCATTTCCCTGACGAAGTAT

GACTGGGTGTACTCGCCCCGAGTAGCCGCCGATGTCGTCAGGAATGTGTTCCGGGCTTAC

TGGTCGCAGGCGATGGTGTGGAGCCTTATGGCGGAGTACGCTGTAGAGGCCGACATCACC

TACGATGCCGTCGTGCTTGCTCGCCCGGACGTCTGGTTCCACAAGGACGTCGATTTGGGG

ACGCGGGTGCTCCCCCTGCCAGAGCGTGCGGTTTTCATCCCGAGCTTTGACACGGAGATG

TGGGACGGCGGGCAGATTAACGACCGGTTTGCGTACGGGTCGATGGCGGCTATGAAGGTA

TATATGAACCGTATCGCGACGTTCACGGACCCGGAGGCCGCCAAGTACACCAACCTGCGT

CTCAACTCCGAGTGGGTGCTGGGCCATCACCTCAAGGTCCACAACATAGCGgtaaacaCT

TTCGACTTTTCTCTGACAAGGATTCGGCTAACTGGAGACATCCCGAAAATGGACCAAGAA

CGGGTGTCCGAGGCCTGTATGTCGGGCGCCGACCCGATCGCCTGCGAGATGGTACGCCTG

GGCTACATGATGCCCGTGCAGGGCGGTAAGTACTGA

>g10148.t1

ATGAAGTCCTCCGCAGCAGCATTCGCGGCGTCCTGCGCGGCCATGCTGGCATCGAGCGGA

GCGTTCCTCGGACCTTCGTTGAACCTGGTGGCTAAGCCTCGAGCAGCCTCACAATACGCA

CAGCAGTCATCATGCAGGACAAGGTTGCGTCTGCTGGGGACGAACGTTGAGAGGAAGCGC

AGTCGTTGCTTGTTTAGCCACTTTGCCTTCCTGTTCGCGCAAGACGTGCCGTGCGAGGGG

CTAGTGGTAGCCGCGGGGCCGGGGCGAACGCACCCGTTGACGGGAACGCTGATCCCCATG

TGCGTGTCCGAGGGGGACACCGTGCTCTTCTCCAGGTGGAGCGGTCGCAAGGTGAAGTAC

TGCGGAGAGGACCACATGTTCATCATGGACGACGACCTCGTGCTGGTCTACCGAGGGCAG

GAGttgacGGAGGAGAGCCTTAAGATGGTTCGCGACCAGGTCCTGGTGGTGACAGAAAAG

GGAGAATCTGAAACCGACGCCGGAATCGTCATCGCGGCGGCGGCGGCGGAGAAGGACACG

GCTAGCCAGGGCCGAGTGGTGGCAGTCGGGGAGGGTCGCACGACCAGCGTGGGCACCATC

GCGCCGTGCCCCTTCACGCCCGGAGACAGCGTGAAGTTCTTGTCGTATGCCCCCGTCGAG

ATCAAGATCAAGGGACAGTACTACGCCGTGGTTCGAATGGTGGACTGCCTCGCCAGATGG

GAGGGAGACCTTgtctaa

>g10175.t1

ATGAAGGTCTTCGCCACTTGCGCGCTCGCCCTCGCGGTTGGCGACTTCGCCAGTGCCTTC

ACGGCGCCCTTTGTTGCCCGGGGTGTAGCTCCCGGAGTCGCTCAGCGTGCGAGTTCGACG

TCCGTGTGCATGTCCGCAAACGACGAGGCCATGTCTCGCAAGAACGCGATCGCGTCGTTC

TCCGCGGGCGCAGCGGCCGTGCTCCTTGGGTCCGTGCCTGTGTTCGCCGCGGACGAGGCT

GCCGCGCCTGCCGCCACGGCCACCGCCAGTGACGGACCGCCGACGGACTGGGGACTGACC

AAGCAGTACTACCCGGACGCGGCCAAGATGGTGAAGCACATGCGGTACTGCACCAACATG

GAGAAGGGGGACCCCAACATGTCGGAGACTGCTCTCAACTGCAAGAAGGAGATGGTGGAG

TTCGTGGCGAACTACCGGCGTTCGGCGAACATCAACGGCAAGCTGTCGTACTCCAACCTG

TACACGTCCATCAGCGTGCTGGCGGGCCACTACGCCTCGTACGGGCCCAAGTTCCCCGTG

CCGGAAAAGCGCCGGAAGCGCCTGTTGCAGGAGTACACCGACATCGAGCGAGCCATCAAG

CGCCAGCGCTAA

>g10191.t1

ATGACCCACGCGACGACTGCCGGGGGCGCAGGCCCACAATCTTTAGCAAGACGGCACGTG

GGGAGTGCGTTAGGCCACTCCCGACGGCAGCACTGCTGGACGTTAGACCTCGACGACTTG

GAGCGGAAGGCGGAGGAATCGGGAGCCAAGGTCCTCCTCGTGTCGCACATGCGTGGTAAG

GTGTGCGACATGGACAGGGTGGTAGAAATTTGCGAACGGCACGGATTGATGCTGGTGGAA

GACTGTGCCCACGCTTGCGGGGTCAAGTGGCGCGGTAGGCAGCTGGGATACCATGGCAAG

GTTGCCGCCTACTCGACTCAGTCGGACAAGGTCATCAACTCTGGGGAGGGCGGCTTCGTC

ACCACGGATGATGACGAGATTGCCGCACGGGCCATCTACCTGGCCGGGGCCTACGAGCGA

CGATACGCCAAGCACCTGGTGCGCCCGTCGGACGCCCTGTGCGAGGCGGCCATGCTGTGC

GTGCCTAACCTCTCCGTGAGAATGAGTGAGGTCACCGCGGCCGTGCTGAGGCCCCTGCTC

AAGACTTTGCCGGAGAGGGTCGTCCAGTACAACCGAAGGTATCGAATGGTGGTGGAGACG

TTGGAGGCGGAAGCGCCGGGGTTGATCCAGGTTCCGAAGCTGGACGAGCGCATTGATGGC

GTCGGCGATCACCTCAACTTTCGTTTGGTAGAGGCCTCCGAGGAAGAAAACCTCTCATTT

CAGGGCAAGTGCAAAGAGCTCGGGGTGCCTGTGAACTGGCTGCGCTCCAAAGTGAACGCA

AGGTGGCACGTGAACTGGCGAGGCTTTGGAGGGCCGGTGCAGGACCTGCCGATGACGGAC

AGCGCCCTCGCGTTGGCCTACGACCTCAAGCTTCCGCCGCACTTCGAGGACGAGGACTTT

GTGCACCTCGCGAGAATCATAGCGTACGCGGCGCTCACAACCGTCGGTGGACAGCTGAAG

CGCCCGCTGAACGAGGAGAAGTAG

>g10213.t1

ATGAAGTCCGCTGTCATGGCTGTTGCTTGCGCCGCCGGCGCCCAGGCTTTCGTTGCCCCC

AGCGCCTTCAACGGTGCCGCCCTGACCACCTCGGCTAAGTCCTCTTCCGCCATGAAGATG

TCCTTCGAGTCCGAGATCGGCGCACAGGCACCCCTCGGCTTCTGGGACCCGCTTGGCCTC

CTGGCCGACGCCGACCAGGAGCGATTCGAGCGCCTCCGGTACGTGGAGGTGAAGCACGGC

CGCATTGCGATGCTCGCCATCGCCGGCCACCTCACCCAGCAGAACGCCCGCCTGCCCGGC

ATGCTCTCCAACTCCGCCAACCTCTCGTTCGCGGACATGCCCAACGGTGTGGCGGCTCTG

TCCAAGATCCCCCCCGCGGGCCTCGCCCAGATCTTCGGGTTCATCGGCTTCCTTGAGCTG

GCGGTGATGAAGAACGTGGAGGGCTCCTTCCCCGGAGACTTCACGATCGGTGGCAACCCC

TTCGCTTCGTCGTGGGATGCCATGTCCGAGGAGACCCAGAACTCCAAGCGCGCGATCGAG

CTCAACAACGGCCGCGCCGCGCAGATGGGCATCCTCGCCCTCATGGtgcacgaggagctc

aacaaCAAGCCGTACATCATCAACGACCTCCTCGGCGCCTCGTACAACTTCAACTAA

>g10858.t1

ATGCCGCAGGCACTGGTGTGGCATGTCTCGGGCAGTGCCATGAAACAGTCAAACTTGGTG

AACGCTTGGGTATACTCTGTCGTAGTGGAAGGGTGGAGCGACCTCGCGGTAGTGGTGGAC

GACGAACAGCTACAGGAACTTGACTGCTCCCTGCCGAGTCGGGGACTGGAATCGGGAGGA

TGGGGCTGCATATTCAGCAGCATGCCGCACTTGTGCAGTTTCAAGTCCGTCGAGAAGTGG

CAAGCGTACATGGATTCGGCAGGCGTTTCGGCTGAAGACAGAACAGAGGCGGACAGGCTC

GGCAGTTCGCTGGAGAAGATCCGGCAAGCGCACACCTCTGAATCGTTCGAGGGGTTAAAT

GTCGACCAACGCGGCGCTAAGGCGGTGATGACTACGTTATTGTGGCGTTACATTACTCCC

TGGCTTCGAAGGGACGTGGACGTGGTGACCCATGTCAGGGAGACGTTCCAAACGTCACCC

TTTATAGCGATACACGTCCGGCGCGGGGACAAGCTTGCATCCAATCACCAAGAGAATAAC

CACAGTGTGAAGAGTTATTTGGAGGAAGCGGTGAGAGTCCTCAACGAGGATCAAaacaga

actacagtagatgccaTCAAAGGTATATGGGTCGCCTCGGACGACACGAACGTGGTGGAC

GAGGTCCGCGAAAAAGCCGGCGCGTACTTCCCAAGCGTCCTCGTCGAGGACATCATCTAT

GTCGCCGGTGGAGTTCCCAGCGGGGTTCAAATCGATAAAGTGACTACACACTCGGGCTCA

CAGGGGTACGGGTCGTTAGTTTACCTGATGGCGGACATCGAGCAGCTGGTGGCGGCCGAC

GTCTTCGTGGGATCTTGCTCCTCCAACATCGGACGGCTTGTGATGGTGCTCAGAGATGGC

CTCGGCAAAGACAAAAAGACGGCCATTAGCCTAGACATTCCGTGGACCCCCAAGAAGCAG

CGTAGGCTTGTGCTGGACTGA

>g10922.t1

ATGACGGTCGTATTGGATATGGACGAGTGCCTGCTGCACAGCAAGTTCCACGGGCCTGGA

GCGGCCTCGGAGGCGTACAGACAGTCGGAGGACAGACCGGATGCGGTAAACGAGGTCAAC

AGTTTCTGGGTTGCGCTTGACGACGGGGACACGGCGCAGgtGAACAAAAGGCCTGGGCTC

GACCCATTCCTTGAGGCGTTGGCGCGAGACTACAACACGATCGTCTTCACAGCTGCGATG

CCGGATTACGCGGGGCCCGTGCTCGACTACATCGACCCCAAGGGCACGCTATTCCACCGA

CGACTCTACCGAAGCAGCTGCAGACAGGTCAAGGGAGCCTTCCTGAAGGACCTCTCTGTG

CTGGGCATCGAGTCGACGGACATGTCTCGGACTGTCCTCGTCGACAACAACCCGCTGTCG

TTCATCTGCCAGCCGACGAACGGCGTCCTCGTTGCGTCGTTCTACGACGACCCGAACGAC

ACCGCCCTGACGAGCGTCATGCAGCTCATCCGGCATCTCGACCGAGCGGGAGACGTGCGG

CCAATCCTCAAAGATATGTTCCGTCTAGACGTCCTTCTCGGCGACTACCGAACGGCGCTG

TTCGACGAcaacgaggaagaggaggaggacgaggaggacgaggaagaggcgTTTCTCGAA

AGCAGCTTGgaggaggggacaggggagTGCCGCAGTAGCAGTAATAGCGCGGGTGCCGAT

TTGCTCGTCGTTGTcgcggagaaggaggaggcggtggaggaGAAGTGCCTGTTCCAGGGG

GACGAGGACGTGGattgctcggggggggggttgagagacGACGATGACGAGTCTGTCAAG

ACGGAAGCTTGCTCGGAAGGGTCGGGCGAATCATCCTTAGGAGAGGAATCGTTAACGTCC

AGGGAAGAGATGGAGCTTGAGCTCGAGTTCTag

>g10955.t1

ATGGCGGAGTTTGACCGGCAGACGGTCGGGGCTCGAGGGGGTGGCTTCGGGCTTGACGCT

GAACTTGCTCGTCAAAGGGAAGCGAAGTACGACCACGGCGCCGAGGACGAAGCAAGGGGG

TGGATCGAGACGATCACAAAGGAGCCATGTGCCGGCCCGTTCGGAGAAGCCTTGCGGAAC

GGCGTTCTGCTGTGCAAACTTGTGAACTGCATAGCCCCCGGGAGTGTGAAGCGAGTGAAC

GAATCGAGAATGCCCTTCAAGCAGATGGAGAACATCAGCAACTTCTTGAAGGCATGCCGC

ACTTTGGGAGTTGCAGAGCACTCGCTCTTCGAGACAGTCGACCTCTTCGAGAACAAGGAC

CTGGGCCTTGTGGTCCGATGTGTGTTTGCGCTAGGGTCGTCTGTACAACAAACCTGCCCG

GATTTCCCAGGACCTCATCTTGGAGCGAAGCTTCACAAGGCAAACAAGCGGGTGTTCAAC

gatcagcagaagcagcaggcaCGGGTCAACGCGTCGTTTTCGAAGATATCCATGGGGTCG

GCTCAATGCATGGAGCGTACACAGATTGCCAAGACGGGAATAACTTTTGGTGCTGAGAAT

GCTGGCAAAGGAGACACGAGCTTCATGGGCATGATGGCAAAGGGTTCACATGGAATTCAG

GAACGCCTGCCTGTGGATCAATCAAAGTCGATAACGTTTGGAGCTGAAAAGGTTGGCACG

GGCGACACGACGGTCATGACAAAGTCTTCTGCTGGGTCACACGGAATCATGGAGCGCGGT

ACGACTTCCAGCCCAAACAGCATCACTTTCGGCGCGGAAAACGGCAAGATGGTACAGCCT

GCTCTGGGCTCTAATGGAGGAGCCACGGAGAGCGACGTGGTTTCGAAGGTCGACAAGGCG

ACAATGGAGGGTTTGAGCAGTCAGGTGGACAACGTCACCATCGAGACCAAGGGCAGCCAG

AGCGAGTCGACAAACGCAGACAAGACAGCAGAGAGCTGCCCTGCCAACGTCGCGGACGTG

TCGGTCGTGGTCTCCTCTACTTCCGTCGACGACGTCAACAAAAGCTGGTTCCAGGAGGAA

GGAGACTCACAATTCCAGCGAACCATCTCCAAGAGTTCGCTATAG

>g10977.t1

ATGCGTAGATCTCTCGCGCCGCTGCTACTAGCCGGCCTCTGTCTGCCGAGTGTTCAGTGC

TTCACAGGGCTCACAACAGCGGCCGTGCGACGAGCAGGCGTTCCCGTCGTCGGACAGAGC

AGCAATGCTCCCGCAACGCTCGTCAGCAGCAGACGTGGGAGCGCTCGCTGCCCGAAGGAG

GGTAGATGtggcacggctgctgctgtcatgatgGTCTCAAAGTCGGCTGCGTTGGCAGAG

GAGACTAGGGTGGACCCGTACGAGATCGAGGCGGATCTCGTGGAGGAGGACAAGGCCGTa

taCCTGGGCCTGTTTGTGCTGCAGCTGCTACCGTCTCTTCTGATTGGTTTCAAGTACGCC

TCTTTCGTGTACTTCGGACTGCTGGCCGTAAGCACCGTCTACCTCGGAGCGAAGAGGCAA

GACATCCCAGATGAGCGGTCGCCCATCACGACGAAGCAAGCGTTTGGGGCGCCAGTGGCT

GCTTCTGCGTCTCTGTTCGGCATCTTCCTGATCCTCAAGTACACCGATGTGTCGGTGGGc

gtggcataccagCTGGTGACCACTCTGCTGGCGGTTGGCAGCGCGGTGTCCATCCTCCCC

CCTGTCATCCGCAAAGTCTTGCCGGAGAAGATCACGGCTTTGCCGGTGTCAGCCCCGCTA

GACGCTGTCTTGGCCAAGGCCTTCCCTAAGACGTGGGAGGACGACGACCAGCCGCTGCTG

GACTTCGCGGAGCTGACGGTTTTGCTCTCGTCGATCACCGCAGCATTTGTATATTTGAAC

CCCGACGTTGGGCTAAGCGCCAAGTTCCTCATCCCCAATGTCTTCGCATGGTGCATCGGA

ATGCAGAGCATCGGACTTATCAGCATCTCCAGCTTCACGACGGCAGCCGTTCTCCTGGGA

GGGCTGTTCTGCTACGACATTTTCTGGGTTTTCGGTACCGAGGTCATGATGACCGTCGCC

ACCAAAATCGAGGCGCCTGTAAAGTTCCTCTTCCCGAGCACCGTGGACCCGGCTAAGCAG

TACCCTTTCTCCGTGCTAGGACTCGGGGACATTGTCGTTCCCGCTACCTTCTGCACCCTC

ATGAGGTCCTTCGACAAGCAGCTGGAGGGGGCTAGACAAGCGGAGGCAGCGGCCGCCCGG

GCACTCGCTGACCAAGTTTCTAGCAAGAACCCCGTAGCCCTGTGGCTTGACTCGTTCCTG

GGAACTCCAGTCGCCAAGGCGGAGGGAAGGGTTGCTATGGTGCCTGCTGTGGCCGCTGTG

TCCGGGGGTGCGGCAGTGGGGGCTGACGGTGGGGCAGGGAGGTCGTACTTCCAGAGCAGC

GTGGCAGCGTATGGGCTGGGGCTTGGTTTGTGCTTCGTGATGACCGTCGCCACCAAAATC

GAGGCGCCTGTAAAGTTCCTCTTCCCGAGCACCGTGGACCCGGCTAAGCAGTACCCTTTC

TCCGTGCTAGGACTCGGGGACATTGTCGTTCCCGCTACCTTCTGCACCCTCATGAGGTCC

TTCGACAAGCAGCTGGAGGGGGCTAGACAAGCGGAGGCAGCGGCCGCCCGGGCACTCGCT

GACCAAGTTTCTAGCAAGAACCCCGTAGCCCTGTGGCTCGACTCTTTCCTAGGAACTCCA

GTCGCCAAGGCGGAGGGAAGGGTTGCTATGGTGCCTGCTGTGGCCGCTGTGTCCGGGGGT

GCGGCAGTGGGGGCTGACGGTGGGGCAGGGAGGTCGTACTTCCAGAGCAGCGTGGCAGCG

TATGGGCTGGGGCTTGGTTTGTGCTTCGTGGTGAACTATCTCTCCAAGTCGGGGCAACCA

GCGCTCTTGTATTTGAACCCGATGCTCCTCGGAAGCGCGTTCGCGACGGCGCTTGTCAAC

GGCCAAGGGGAGTTGCAACAGCTGTTGGCGTTTCAGACAAGCGCGGAGGAGGCGCCAGTG

GCGCCGAAATCCACGGATGCGTGA

>g11241.t1

ATGAAGGGTACCGCAGGATCCGCCCTTGGCGTGGTCGTCATCGCCTTCGGGCTTTTACCG

GGAGCTATCGGGGAATCGCTGACACGAGAGCATGCTGAACACTACCCAGCCGGTGGTGTA

GAATCGAGCGCCTTAGACGGCAGTGCTCTGCGTCGTCTCCAGCCTGTATCCGATGATGCC

TTCGACGTCAACGGCACGCCCGCGGAAAGAGCTGCCAACGCGCTGATGGAGCGGACCGAT

TGGGCGGAGACCGAGTTCTTGGCATCGGAAGACGTGTTCCACGAAAACCTTGGAGATTTT

TCCTCGGCCGCCACGTTCCACAAGTCGCTTCCACACGACGAACTCGGACAGGTGTTCAGC

TTCGATTTCGAAGAACTCATCGAGTGTGTTGCTTCAGGAGATTTCGACACGTGCCAGGAG

GTGCCGGCCGGAAACGTCGAAGGCTTCCTGGTCAACCCTCTTGGTGGTCTCGCCAATGAC

ATGGCAGGACCCGCGGGGCATGCTTTAACCATCCCTGCCGCTCCAGCACTCGACTCCGAG

GACCTGGCTGCTCAAATTGCGGAGGTGTACTGGATGGCCCTGACAAGGGAGGTGCCGTTT

TCCCAGTACGGCGAAGACCCGACAACTGTGGAGGCCGCAGCCAGCTTGGCCGCCATGCCG

GGTTTCGCGGATCTCAACTTTGTGGCGGTCGGGCCGGATGGCAGCCCCGACCCACTGACG

CAGCTTTTCCGGTCTTCGGCCGTCGGTGTAGAGATAGGACCCCATGTTTCACAGCTACTG

GTGAATAACTTCACGATTGACTCCATCACTGTGGAGGCTAAGCAAAATACGTTTGCGCCC

GGAGAAGACTACATGGCCGAGTTCGACGAATGGCTATCCATACAGAACGGTGAATTTCCT

GTAGAACCGGAGATCCTCGACCCTGTGCCGCGGTATATCCGCAACGGACGCGACCTTTCG

ACAATGGCAGCAACCGACACCATCAATACGGAGGCGTACCGATCCGCCCTGATCCTTATC

GAGCAAGACGCCATCAGCCGTTTCGGTATCAACGGCCCGTACGTGGCAGACGGTCGTCAG

AGCGGTTTCGTCAACTACGGCATCTCCCATACTATGAGACTTGTTGGAAGCGGCGAGCTG

TCCATGAGGTCCTCATGGTACCAGAAGTGGAACGTTCACCTGTATGCCCGTCCGGAGGCC

ATCGGTGGAACCATCCACAACGTCCTCAATGGCGATCTCGACATAACGTTCGCCGACTCG

ATTATCAACAACGACGAGTTGTTCAGTAAGGTGGAGGCTAGGAACATGGAGATCACCGGG

ATACCATCCACCTTCCTTCTACCCCAGACCGTCAGGGAGGGGTCACCAACTCACCCATCG

TACCCCTCTGGACACGCCGTCCAAAACGGTGCCTTTTCAACGATACTCAAGGCTCTTGTC

GGGCTGGAGAGGGGATTGGAGTGCTTCAACGACCCCGTGGTCCCCTCTGATGATGGATTG

ACCCTTCTGCCCTTCGACGGCTGCCTGACGTTCGAGGGAGAAATCAACAAATATGCCGCC

AACGTCGCTCTTGGCAGGAATTGGATGGGTGTTCACTGGAGGATGGACGCTCAGGAAGGG

TTGCTGCTCGGAGAAACGGTCGCCGTGCGGATCCTGATGCAGGCGAGAGCACTGCCGCGC

ATTGTTGCTACTCGTGCTAGCTGTGGCAGAGTGATGGTTCAGGGGGAAATCGTGGGATAC

TGTGGATTGGCGGCGTCGTTGGAAGCGGAAGTGCGAAGGGTGGTGCTGGTGATAAGAGGT

TGGGAGATGGGCAAGCGCAAGGGTGTGGTGGTGCTCGAGGTGGCGGTGCAATAG

>g11441.t1

ATGAGCGCCGTTGTCTCAATAGGTGCAGCGCGCACTCTCGCCCTTGTGGCATTAGTGGGA

GTATCGCTGTGCCATCACTCAGTTGCGTGGGGTCCGGTTTTACAAAATGTTGGTGGTGTG

CAACGCAATAGGCAACAGCCAGTCGGTACGAGTAAAGCCTCGATAAGGCCATGTTTCGGC

CGGACCAGCGCAGGACAGATCACGTCCTCGTCACCCCCTCTCGAAGCAGCCTCTTCCGAC

CAGGAACAGGAAAACCCTACCCCAGCCAAGGCAGCGGTACCCAAGGGCGATGATGGGGTA

GAGCAGGCGACGGGTGCGCCACCAtcggaacaacagcagccaccGAAGAAGCCACAGCAG

CCGTGGTGGGAAGATGAGAGGCGCACTCAGGGGCTGCCTACGCTCACGGCCTCGACCCAG

TGGCGCATGTTTCTGACCCTGAAGGATCCGGCGCTGGGAGGGGATGCAGGGGAGACCACG

GAGGTGGCCGTCCGCGTACGGTTCAGCGAAGACAGAGGCTTTGAGCCCCCTCAAGGCTCC

CTGGAAATAATCGAAGACAACCCGTACTTTGGCCAGCAAGCGGGAAAGGCGCGATGGACG

CTGTCCGAGGAGGAGGACAAAAGAGGCGCGGGACTGTGGGTTTGGGGCTTGTTCGAGGAC

CCGCTGTACCCGTTCCTGTTGTTGCAGGTCGACAACCTTGAGATCGACCTCAAGGGGGGC

GCGAAGATCGCTCCCGGTAGGCTGTACGTGAAGGTGGATCACAAGCGAGACGACGAGCTC

GGGCCGACACTCAACAGTGGAACTGTTGGAGTGAAGCTGATTGAGCGTGTTCGAGTGGAC

CCGCTCGGAATGGCGGAGGCCGATATCTCAAGCGTCGTACCCTGCGGCTCGGTGCGATTT

CGCCCAGTGTAg

>g11480.t1

ATGTGCGTACGACGTTTAAGCGACGGCACAGGGGATTTCATCGGCGCTACAGGCTCGACC

GACTTGGAAACATACGTGGGTGTCGCGCCGCCTGCTTCAGCGATCAGCGACGATACGGGC

AACGTCACGGATGCCCACTCGATCGAGTCAGGCACCCCCTTAACCGACGAGTGCACACAG

GACTGGATTCTGGTGTCCACAGAGGCCGACGCCGACAGCCTGGTCTTTGAGGCCGAGAGA

GCTCTAAACACGGGTGACACGCAAGACCACGCGTTTCTCGACGACAGTGCGGATGGAGCT

CTGGCCACGCGAATCCTTACGGCCTGGGGTGATCAAGATACGGTCAGCTACCATGCCGGC

AACATCGCCAAGGGGCAAGTGGTTCTGTACGGCGGCGCGGACAATTCCAAACTCAACCCT

ATCGTCCAGCTCACGTCCAACCCCGCGGTCTCTTATTTGGACGTCAGGATGCACGACTTC

CCCATCCCGGCCACTCGGACGTGGTACGAACTCAACTGCATACCGGCATCGGATCTGCCT

GCATTCGACAAGTACCACGCGATCGGGTTTGAAGCAATTGTTAACAGCGAAAACGCGAAA

CATGTGCACCATCTCGTGGTCCGGGCGTATACCGTCGACCATTGCGCCGAccggtgcggc

ggcggcggggacgacGATTACTACTCTACGACGTCCACAACACCCACGACGTCCACTCCT

TCTACAATCTGCGAAAACGTAACGTTCGCGGATATTTTTGTCTGGGCTCCCGGATCGACG

GACACAGTCTTCCCCGACGACGTGGGTCTTCTACTCGGTAACGCCTCCGGGAGGTTTCTG

TCTCTGGAGATTGATGTGCACTATAACAACCCCGACGAGGTCGAGGGTTTGATCGATGAC

TCCGGCGTGCGCGTGTACTACACGGAGGAGCTCCGCCCGATCAACATGGGGGTGTTAGAG

CTGGGCGATCCAAATCTCGAGCTCTTGGGACAACCCTTGCCGACGGGAAAGTCTTGCTTC

TCCTTCCAGTGCCCGGGCGCTTGCTCTGAGGAGTACTTTCAGGATGACGAAGTGACCGTC

TTTTCGCACGGCCTCCACATGCACGAGAACGGACAGCGGCTCTTGACTCGGCAGTACCGT

AACAACGGCGACGGCGAGGAGGTCATGGTCCACTCTGCCGAGGTCGAGTACTACTCCTTC

TTGCAGGCGGGCTTCCACATTGTCACCAGTAACGACAGCGAGACTATTCAGAAAGGGGAT

CGGTTTGAGACGAGCTGCTACTACGACACCGACCTCTCCTCGGTGGACTCGGACAACGTC

ACCTTTGGCTTCGGGTCTGAAAACGAGATGTGCATCCACTTCGTCTTGTACTACCCTGAC

CAGGATGTCCCATTCTCTGGGATTTGCGGTTTGGATTGGTGCGGCGGTACCATGGACGAC

ACGGAGGTTTTCGCCGCCGATTCCGACTTCGAGCGATCCTTTGGGATAGTCGACACCTTC

TCGCCTGCAATCAACCTCTTCCACGGAACGGGCCGATACACGGTAGCTTGGTTGCCGGCG

TCGGCGTGCGAACGAGCTGCCCCTTTAAGGGCGTTAACCAGGACGGGATGA

>g11595.t1

atggTGCTCGAAAGCGAACTCCacgtagtgttgctgctgttcatcGTCTGGTCGTCAGAG

CTGGCCTGCAAGCGCGTGAATGTCTCCCCCATCATTGGCCAGATAGCCGCGGGGCTAGTC

ATAGGGCCCGCTCTCCTCGACCTCTTCGCCCACGTGGAGGTGTTTCGCCTGCTGGGCAAG

CTGGGCGTGATGATCGTCGTCATCGAATCTGGGCTCACCGTGGACGTTCAAGTGCTGCGA

AAGGTTGGAACCCGGGCTTTCCTGGTCGGGATAACTGGCGTGATCCTGCCCACGGTCATC

AGCTTCGCGCTGTACGCGGGGCTCCTCGACGCAGACTGGAAGACGGCCCTCTCGGTTGGA

GCGGCGCTGTCTCCCACCAGCCTCGGCTTCAGCGCGCAGCTGCTCGGGGAGGTTGGAGAG

CTGACGACCCCGATCGGCCAGCTGATCTGCACGGCCGCGGTGATTGATGACGTGCTGTCC

CTCCTCTTGCTTGCTGAGGTGCAGGCGCTCGGAGATGAAGACCCCAAGATGAAGCGATTC

TCTCAGTGGGGGTTGAGGCTCTTTTTCGCGGCCACGGTCGCCTTCAGCGTTCCCTCCATC

TACCAGGACGGCGGACTGCTCCGGGTGGGGCCCTTCTGGAAAGGGCTCGTCCTCACCGTC

GCCGCGATCGTGGGAAAGCTCGCCGTGGGGATCTTCGCGGGCCCGCCCCTGACCCTCGCG

GGTTTCCTAAAGCTTGGCTGGGCGATGAACGGGAGGGGGGAGTTCTCGTTCCTGATCGCT

CAGGAGGCGAGCGACGAGGGGGTGCTGTCGGCGGAGGACTACTCGGCGGTGGTGTGGGCG

CTGCTCCTCAGCTCCCTCGCCACGCCCATCGCGTTCAGGAGGGTCTTGTTTGCCGATCGC

CGGGCGCAGGAGAAGGGAGTCGCCGCCACCAACGAGGCacagggtgGTCTCACGCACCAC

GGAGGCGGTTCGCTGCAGGGCGGCAGAGAGCATGCATGGGCCAGCAGGGGCGATTAG

>g11881.t1

ATGATGGTATCTCCCAAGAAGCGTCTCAACCGCTCAACCCCTTCAGCCCTCGCCAGGGCA

TGTGTGCCAGCGACGGACCCAGCCTCGGTGGACAGAAACCCTGTCGTGTTGTCGGCCAGG

ACGGAAGAAGACCTACAGGCCGTCGCGGAGGAAATCACGTCTGCGGGGGGCGAAGCCGTG

GTCGTGGTGGGCAACGTTTCAAAGGAAGCTGACTGCAAACGCATGGTGGATACGGCGGTG

GAAACGTTCGGTGGTCTGCACGTGGCCTTCAACAACGCTGGCAACGCCTACACGAGCACC

TTCGCTGACGTCGAGGATGAGGGGCTGAGCACGATAATAGACGTGAACTTCAAGTCTCTC

GCCTTTTGCttcaagtaccagATTCCTGCTATGGCAAAGTGTGGGGATAAGGGCTCGATC

ATCATCAACACTTCGGCGCTGGGCTCGCGTGTTGCCGCGTCAATGCCTACCATGGGTCTG

TACGCGGCTACAAAGGCAGGCGCCGACATGCTCATGAAGTACGCCGCAATTGAGGGCGCC

GAGGTTGGAGTGCGGGTAAACTCGGTCGCGCCGGGGGCCGTGGACACGCCCCTCCTCCGC

AACACCGTACCTGCGGAACACCGTCAGCCTATGCTTGAAAATGTACACCTCTTGAAACGC

TTCATCGAAGCGGAGCAGGTGGCTAAAGTGGTCACCTTCTTGGCTTGCGACGACGCTGCC

ATGGTAACCGGGTCCGTTTATCCCGTTGATGCTGGCTACACTATCAAGGCCTGA

>g11898.t1

ATGAAGACCTTCGTGGGTTTGACAGCCCTTCTGGCGGCGTTTCCTCCCTGCCTCGGATAC

GATGAGCCCCCCCAGCcaacacaccccctcgtcaGCGGGAGCGTGTGCAGGGTTAGAGAC

TCCCTCGACTTCTTGGACCCGAAGGAGCGCGCGAAGGTGACCCTCGACAAGCGACTGGCC

ATTGCCAAGGACGAGTTTGAGGTTGGACCTACGTGCCACATTTCAAACGGCGACGAGGAA

AACGTAGAGGACTTCGCCGGAAACTTCCACAAGACCCTCCCGCACAACAAATTCGGAGAG

GTGAAAAAGAAGGCCTACCGGAAGCTGCTGGAGTGTGTGCGCACGAGCGACATCAATGTT

TGCGAGGACGTGCCCTCTGGGGCTGGCCGGAAGGGCGGCGCCAAGCTCACCAACCCGCTC

GGGGGCACTGCGCACCAGGTCGATGGTGCTGACAGCGACAATGTCTTCATCACCACGCCG

GACAGCCTCCTCTCGGAAAGGCTGGCCGCCCAGCAGACAGAGGTGTACTGGATGGCTCTG

GCGAGGGACATCCCCTTTTCCCAGTTCGCGACCAACGATCTCATCGCGCTTGCCGCAGAG

AACCTTCAGAAAAATTCGGCGTTTGAGGGTCTTAACATCCCCAGAAGCAAAGGTGGAAAG

ATCAACCCCGTCACCGATCTGTTCCGGACGACCTGGCCGGGTGTGACCACCGGTCCCGTC

GTGTCTCAGTTCCTGCTGTCCGACTTCATGATCGATTCGATTGTGGTTGAGCCAACGGCG

GCCCCCCTCGTCGCAAAGATGGACTACATGACGTCTTTCGACGACTGGCTGGACGTGCAG

AACGGCGATTCCGACGTGAAGACTCTGTTCGACAACGAAAACCCCCGCTTCATCCGAAAC

GGCCGAGACCTGGCCACTATCGCCTTCAACGACGTCCTCTACACGGAGGCGTTCCGTGCC

GCACTTATCTTGTTCACCCAAGGCGCACTTGGGGGCTCTGTGGGACCGTACGCGGAGGCC

GAGCGCCAGGAGGGGTTTGCCACCTTCGGCGCGCCGCACATCCTCACGGCCATGGCCTCG

GCCAGCTCTTCTACGCGCCATGCGTGGTACGCTAAGTGGCAAGTCCACCGCATGCTGAGG

CCCGAGGCGTACGGCGGCCTGGTGCACAACACGCTCATGAAAGATGTCATTACTGCGCTG

CCGGACTCCATCTTGAAGAACACGGATCTGCTCAGCCGCGTTGCGGAGCACAACAAGTAC

ATGAACGACCGTGGCGAGAAGACCTTCCTGCTCCCCATGGCGGTCGCCGAGGGTTCTCCT

ACTCACCCCGCCTACCCCAGCGGACATGCCATCAACGTCGGCTCTTACATTACCACGCTT

AAGGCTTTCCTCGGTCTGGAGATGGGCAAGAGGTGCTTCCCCAAACCCGTCATCTCGAAC

GACGAGGGGACAGCCAGGATCTCATACGTGCCGACAAAGCACGAAATCGTGGGCAAGTGC

ACCAACGAGTACGGCAAGGAAGTCGACGGCCTGACGTACGAGGGAGAGCTTAACAAGGTA

AGCTCCAACGTGCTCTTGGGACGGTCGCACATCGGTGTTCACTGGAGGATGGACGGCGTA

TACGGAGCGCTCATGGGAGAGACTAGCGCGGTCCGCCGCCTGCAGCAGGAAATCCCGGGC

CTCCCGGAAGCCCGACTTGTGGAGAACCCAAGCCGTAAGGACATCCCCCCGGCGATGTAC

AAGTTCCGCCTTTACAGCGGCAAGGTGCTCGAGCTTTACGGCGCAAACTTGTTCAAGCTG

GACGGCCAGCTCTGCAAGGGAGCGTTCACCGGCGACGACTTCTGCGACCCGATTCGAGTT

GACGCGTTCGACTCGTTTGAAGACATCGTCGAGGAACACGCCACCCAGTCGCTCCACATC

GAGCTGTGA

>g12143.t1

ATGCTCCCCGTGCACCACTTACCGCTGTACCCGcaagcGGGGCTTGGACTAGCGGCGCTG

GCGACGGGGGTGGCAACCGGGGCGGCTCCTGCTaacgcggcggcgggggcggcggtcgaG

GTGATGAAGCTGAGGTTCGGCGACAAGCTAAGGAGGGCGTCCAAGATGCTGGACGAGCTA

CAGATGGACATCTCCAACGACGACTGGGACCTGGTCTCCACGTACCCGAACGCGTTCCGT

TCCCTGGTCCCGGTGTTCACGAAGTACACGGATGCTGCCTTCCCGGGAGATGACCCGGTG

GACACCACGTCGAGGGTGGCGCTAagGTACGAGGTGGGTCGATTTTTCGGAGCGGTGGAG

CGGCTGAAACGCGCCTCGGAGGAACAGAACAACCGCGAGGCGCAGAAAGCCTTCGCGGCG

ATGTCCGTCGCCTACGACCGGTATCTCAAGGCGGGCAACCTGTACGAGAGCTACGACACG

GTGACATCGACGGAAGGCTTTTACGCGGGGGTGGACAACAGAAACCTTAAGTTCCTCCCT

CCCTCGACGGACCCTCCCAAGATCAAAGACAACGTTCTGTTGGTGGCGGGGCCCGACAAG

GGTAAAACTGGCTACATGATTGGAGTGGAGGGCGCAAACTCTTCGATGAAGGCGATCATC

AAGCTCGAGACTAGCGCCTCTCTCGGGATTCGCGAGATCAAGGTTGTGCCGCTTGACTAC

GTGGCGAAGACAGTGGAGCAGGGAGGTATGGCCCGACCGGCCGTCAAGAAGATGAAGTCC

ATTTCTTAA

>g12226.t1

ATGGCTTTTAAGGCGTTCGTTGTGCTGGTGGCTCTGTGCCTGGTTTCGCTGGCGTCTGGC

TTCGTCGCACCGACCGCTTTCGCAAGGAATGTGGCCGTAAAGCAGGCCGTGCAGATGAGC

GCACAGACCAGCTTGCCCGCCGCCGtgccggcagcggcagcggccgccgTCCTCTCGGTG

TGCTCGCCCCTCGCGTCCTACGCGGCGGAGCAGTCGAGCGCCCTGGCGCAGCTCCCCACC

GAGCTGATCGCGGACAACGGAACCGCGACCCTCGCGATCGTCGTGGGCATGTTCATCCCG

TGCTCGTTCCTTATCACCCTTTACTTCGGCACCGAGCAGAAGAAGGCGGGCGTGATCGAG

GGGCAGGGGATGGACAAGAAGTAA

>g12281.t1

ATGTGGTTCTTCAACCGTGTGCCCCACTACCACGGAAAGGGAAAGGTGGTTGTGCTGTCG

GGGTGTGACCGAGGGATCGGAAACGCGACTGCCAAGAAGCTCAGCTCGCTCGGGTACACC

GTGATCGCGGGGTGCCTTACCGATGAAGGCGCCAGCAAACTGCAAGAGGAGGTCACCGGT

ATCAGGGCCGAGAAATGCGACATCACCAAGACGGAAGACGTTGCGCGCCTGGTGTCTATC

GTGGAGACGGAATACAGCGGGAAACTGCACGCCCTCGTCAATAACGCCGGGACTGTGAGC

ACCGGGCAGGCCCTGATCGTCTCTCTAGAGGCAGCGGAGAAGGTTGTGACGGTGAACCTT

CTCGGCACCATGAGGATGACCAAGATGTTCCTCCACCTCCTGGTGGATAACCCTGGGTCG

AGGATCGTGTTCGTGTCATCTATCGCGGGAATCTGCCCGACGTGGGGACTATCGTCGTAC

ACCGCGTCCAAGTTCGGGATCGAGGGTTACGCCCGCGTTCTGCGCGACGAGCTCCACGTG

TTCGACGTCCAAGTGAGCGTCATCAACCCGGGAGCTACCGACACCAACATGTTCAACACC

TTCGTGCAGTCTACCCGGGACAATTTCGAGGCATGCCCGGAAGAGACGAAGAAGATGTTT

GGGCTGGACTACGGCGAAAGGTGCGCCAAAACCTACGAGAAGGACGTACCGCCGGGGGCG

GACGACGTCTCAGTGCCCATGGACTGCATCTGCAAGGCCGTGGGGTCGATGAGTTCCGGG

GACAGGTACTATAGCGGATTCTCGGCGCAGACCGTCTTCCGCCTCGGCTACATTTTCCCC

GCTTTTTCTAGCTTTGTGGGTCGTTTCACGGCAATCCCTCCCCATCCAGTGAAAAATGTC

GAGTGA

>g12284.t1

ATGTGGTTCTTCAACAACAGCGTGCCCCACTACCACGGAAAGGGAAAAGTGGTTGTACTG

TCGGGATGTGACCACGGCATCGGGAACGCTACCGCCAAGAAACTCAGCTCGCTCGGGTAC

ACCGTGATCGCGGGATGCCTCACCGACGAAGGCGCCCGCAAACTGCAAGAGGAGGTCACC

GGTATCAGGGCTGAGAAATGCGACATCACCAAGGCGGAAGACGTTGCGCGGCTGGTGTCT

ATCGTGGAGACGGAGTACAGCGGGAAACTGCACGCACTCGTGAACAACGCCGGGATTGTG

AGCAGTGGACAGGCCCTCATCGTCCCCCTAGAGGAATCGGAGAAGGTCGTGACGGTGAAC

CTCCTCGGCACCATGAGGATGACCAAGATGTTCCTCCCGCTTCTGGTGGGCAACACTGGG

GCGAGGATCGTCTTCATGTCGTCCGTCTGCGGACTCTGCCCTCTGTGGGGAGGGTCGTCG

TACAGCGCGTCCAAGTTCGGGATCGAGGGGTACGCCCGCGTTCTGCGCGACGAGCTCCAC

GTGTTCGGCGTCCAAGTGAGCATCATCAACCCTGGAACTACGGACACCAACATGTTGAAA

TCCTACGTTCGAACCGCCAGGGATAATTTCGAGGCATGCCCGGTCGGGATCAAGAAGATG

TTTGGACTGAACTACGGGACAAAGTGTGCCGAGCACTACACGAAGATCATGCCGAAGGTG

TCGGACGACATCTGCTTGCCAACGGACTGCATCTGCAAGGCCGTGGGGTCGATGAGTTCT

GGGGACAGGTACTATAGCGGATTCTTGGCCAAAACCGTCTTCCGCCTCGGCTACATTTTC

CCCGCTTTTTCTAGCTTTGTGAGTCGTTTCACGGCAATCCTTCCCGACCCGGAAAAATAA

>g12294.t1

ATGGCGGCCATCCTGGCAAGCCTGGTCAGCAACCTGGTGGTCCAGATGAGTGTTTTCTCC

TACGCGGGCTTCATGGTGGAGTACCTGGGCGTCGTTGATGACAAGGACAAAGCCGGTGGA

CGAGGTTACGCCGccgaccatcccccccccccgacgggaGGCATGGGATACCGTACGGCC

GCTGGGCACTACAACGAGGTGGGCTGCCCCAAGGCGGGCGGCGCGGGCCAGGCTTTCGGA

CGAAACTGCCCCGCCTACCCGGTTCAAAAACGCCTCGAGATGACCCCGGACCCGTTCCTC

GTGGCGCAGAAGCTGCTCGCCCGCCGCGAATCGGGCCCCGATAACGCCTATTTCAAGCCG

GCGGATGCCCAGCTCAACATCCTTGCGGCGGCATGGATTCAGGCAATGACGCATGACTGG

AtggaccacactgctgccgacGAGGCCATCATATCTGGAGGCGAGGCGCACGGTTGCCCC

ATGGGCAGCTTCAAATACAACAAAACGGGTTCCGTTCCTTCCACGAACGGACAGCCAGCC

CACCCCAACCGCAGGACTCACTGGTGGGATGTTTCCTTCGTCTACGGTAACGATTCCGAC

ACCGTGGGCAGGACCCGCACTGGCGACGGAGGCCGCATCcatgcgggggaagggggggtg

atgGACCACGCCGAGAACGGTGTCATCAGCACCGGCGACAACAAGAACTCTTGGGTCGGG

GTTTCCTTGCTGCAGGCCCTCTTTTCGATGGAGCACAACTCTATCGCGGATGAGATCGCC

TTGGCTCACCCGGCCTGGGACGGCAAGTTCCTCCACGACAACACCATCCTCGGAAAGGCC

GGAGTCGCCATCATTTCCGGCCTGGCGGGCCTAAAAGAGCCCGAAAACCACGGGACTCCC

TACAAGCTGACGGAGGAGTTCGCGACCGTGTACAGGCTGCACCCGATGCTGCCCGACTCG

ATGGAGGTGGAGAATGAAACCGTCAAGATGGAGGATCTCTGCGCCGTAAAGGGAGAAAAT

ACCCTTCGCCGGATTGGCGCTGGGCCCTTCTGGGAGACGGCTACCCGCGATCCGTGCGGC

GCGTTGGTGCTATTCAACTACCCCAACTTCTTCAGAACGgtcccacccaccaccaacgcG

GGCGTCCCCCTGCCCGATGCGCCGGTCGACCTCGCCGTGCTGGACATCTTTAGGGACCGC

TCCCGAGGGGTGCCGCGGTACAACGACATGCGCCGCGCCATGAACATGAACCCCATCAAG

AAGTGGAGCGACCTGACCAAGGACACGGAGGCCCAGGCGGCACTGCAGGATGTGTACGGT

GACGATGTTGAGCTGTGCGACACGCTCGTCGGAAATCTGGCCGAGGACAAGATCGACGGT

TTCGCCATCAGcgagaCGTCTTTCCACATCTTCATCGTCATGGCCAGCCGCCGTATCGAG

TCGGACCGATTCTTCACCAAGGACTTCACCCCGACGGTGTACTCGCAGGAAGGCTACGAC

CGCGTTGGCAACACGGAGGGGATTCACGATCTCCTCGATCGCCACTTCCCGCACATCGCC

AACATGATCCCCCCGGGCCAGTCGGCCTTCAAGCCGTGGGGTGTGTAG

>g12402.t1

ATGCAGGTCTGGTCCAACCCGCAGGCCGTGCAGGACTACAAAGACCTGTTGAACGGCGAC

GCGCCCGAAGAGAGGTTCGACGGGCCGTCGGTAATCATAGGCAGGGGCAGGCTGGGCACG

GCGCTCGCTACGATGGGCATGGGTGAGGACGTTGTGCTGGGCAGGGGCGAGGCGATACCG

GCGACCCTCGCGGCGACGTCGGGAAGGGGGGAGTCCGAAGGAGACGATGGCGTGTTGACG

GAGTTCCCGATATACGTGTGCGTTCCCGaggaggaggtggcggcggtgatcGACCTGTGC

CCCCCCGAGAAGCGGGAGGACCTGGTCTTCATGCAGAACGGCTGCCTGGAGCCGCTGCTG

AAGTCGAGGGGACTTTGCCGCGGCGAGCAGACGCAGGCGACGCTCTACTTCCACATTAAg

aagGTGGGCGCTAAGCCGAGCGAGTACCTGTCGAACATCGGCAttgatgggaggggggag

gcgaagCTCGCGGGGGAGTCAGCCGTGTGCGGCAAGTGGAGGGGGGCTCTGGCCATGCGG

CTCGAGCTGTCGACGTACGATCTCCACTGCGCGAGGgTGTACTACTTGGACTGGCGGAGG

GTTATGTTCGAGAAGCTGATCTTCGAGTGCGCCATCAACCTTGTGGGCGCCTTGCACAAG

TCCCAGAGCGTAGGCTTCATTATTGAGTACCACGACGAAGAGGTGTGTGACATGCTGTTC

GAGATGAAGAGCGCCCTTCGCGGCACCCTGGCCGTGTCTCTCATGAACGGTTACCCCGAG

CGGCTTATCGCGTACGGAGCGTCGGTGGGTGACGACAAGACCACCGGCATGGACCCGGAA

ACGTTCCTGTGGTACAACAAGTTCTTCTACGACATGTCGTACATGGCGATGTCGAACAAG

TTCGACGACCCGTGCCCGATGCACacggagtacctcaactacggGAAAGAAAAGGGGGTC

TTCACCTTTTAG

>g12412.t1

ATgtacaccctcgtcgaggccgTCCAGAGCGGGGACCCCAGCGCGGTGGAGGTGGTAGTG

GGCGTCATCGGGAAGGAGCAGGCGGTCCTGCAGCTGTCGGCGTTGCGCACCGACGGCGAC

CGCAAGCTGGAGACGGTCATCATGCTGGCGGCCCGCAGCGGAGAGTTGGAGATGTTCCGC

GCCGTCCTTCGGTCCCTCGAGCGAACGCTGTCCGAGCGGCAGATCGCCGAAGTGCTCGAG

GCCAAGGGATCGGGCGGGAGCTCGCTGctcacggcggcggcggagggaggCAGTGAGGCC

ATCCTCGAGGAGGTCTCCAGTATATTGGGAGGCATGGACCATTCTGGGGCTGGGAAAATG

ACGGAGCAAGAGGCTCGCTGCCTGATGAAGATGGCCACCAAGAGCGGGAAAACGGGGGTG

TTTCATAAAGCCATGGAGGCGGTGCTGCTGACGAGCAAGCGGTCCGGGTTGTATGCGATC

GGCGCCAGCGAAGACCCGAACGAAACGAGCGTCCTTGTCGAGGCGgccaggggggggagc

ggggagatGATTGACGTCGTTGTGGAAGCCATGAACGACCTGCTTACGGAAGACAAGGCG

GTCGCCGCGGTGACTTCGTGTGCTGGACCTCGGCGCATGTCGGCGCTGATGTCTGCGGCG

GAGAGCCGGAGCGTGGAGGCGGTGACGGCTGTGATGAAGGCCGTTTCGGCTATCCGCGTC

GGCACCGACAGTGCTCGCAACATGGTGATCGACATGGTGACGGCGAAGGATGTCCACGGC

ATGACGGCGCTATCCTACGCGACGAACAGCGGCGTGATGGagatcatcgtcatcgtctCG

GCGTCGATGCGCCGCTATCTTCCCGCGGGCCAGgtgGAACGCGAGCTGCTGGATGTGATT

TCGTACCTGCTCCAACACGGCGCGAAGCCCGGGACGCTTGGCCTCATTCGGCTCTCGACC

AGCGTCCAGTATCCCCGCTTGAAGGAAAGCTTGTTGGCAGCGGTGTCCTCGGCGAAAAAC

CCGTTTATCCCGGGGATGAACCTCTCGGTCGCCCTCTCAGTGGCCGCCAGAAGAGCCGTC

GAGGGAGAGAAGAGGGCCCTGCTTTCGATGCAGGCCGCCGTGGACGAGCTGCTTCTCGAG

GTCCTCGAGCACCTGCCGCAGACCGTGAGGGGTTTCGAGGGAGAGATGAAGGCGTGTTCG

GCGGTGTTCGAGCCCGAAACCGTCATCTCGAGCCACAAGGGCATGATGGGGCCTCTGAGC

GTGGCCCtccagaagcggcagcagatggAAACCTACTGCACGATCCCCTTGGTCCTGGAC

TTCATGTCGCGGAAGTTCACGAGGGGTCTGCCCAGCCTGAGAGACACGGAGGGGGTGCTG

GAGAACAACAAGGAGCTGTCGAACGCCGGGCAGACCCTGTACGGCGATGGCTTGCTCGCG

GAGGGTAGGATCCTCTTTCTGGGGATGGCGCTGCAGAGGGGCTACGGGGAGGGCAAGCTG

TCCAGCATCACCTACCTCCCGGGGGCTAGGTTTATCATCGCTGGACTCCTCTCGCGACCC

GACAGCTACTACAAAGTTCCTGCTTTGCGCATGGTGCTAGATTTGGTGACGTACCTGTGC

ATGATGGCGGTGTTCGGAGGCTTCGTGCTTTTGAACGACGAAGACATTTTCAACTGGTCG

GAGATCGTCTTCGGTGTTTACATCTGCgGGGCAATCCTGACAGAGTTCCGAGAGATCGGG

GAAGACCCTTGGGAGTACGTCCGCGACCGCTGGAACGTTCTCGACGTGACGTCCCTCTGT

CTCATGTTCGTGAGCCTGGGGTTTCGCGCCTTCGACCACGTCGACTCGATCGTGGCCACG

TCTCTGTACGCTCTGAGCGCACCACTGGCCTTCACGAGGATCCTGTTCTTCGCCCAGATA

CTGCCCTCCCAGGGGCCCATGatccagGTTATATTTTCAATGACCGGCGAGCTCGTCAAG

TTCGGAGCGGTGATGATCGTGGTCATGGGCGGCTTCGTGATGTCCTTTTACAGTCTCTTC

AGAGACTCCATGTCCTACGGAGAGGTCTGGCAGAATGTGTTCAAGGCCATGCTCGGAGAG

ACCGACTTCTTCGACGAGTTCTCCGGCACCCAGTTCGACACTGTGGCTACGGTGCTCCTG

GCGACGTACCTGGTCATCATGACCATCATGATGCTCAACCTCCTTGTCGCGGTGCTGAGT

ACCGCTCACGCCAGGGTGGACCAGAACGCCGATCAAGAGTACAAGGTTTCCAAGGCGCGC

TTGATCCAGCACTACGCGTCCGTGGTCGAGACAGACCGCCTGCCGGCGCCGTTCAACCTG

GTGCAGTGGGTCCTCTGCGCCCCGATGTTTGTGATCGACTGGTGTTTCTCCACCACGCTC

CACAAGGACAGCAAGCGCTTCTTCGGGCGCACCGTGTTCTGGGCGATGCTGGGGCCCATC

GCCGTGGCGGCTGGGTGGCTGCTCTGGGTGGCATCGGTGCTGAAGGCCGCGACCGTGGTC

TGGCGAACGTCTGCCGGGAATACGCTAGGGGGGAAAGTCGGGAGGGTGTTCCTCGCCGTG

TCCTGCTGCATCGTGGGAGCGCCGGTCTGGCTGCTCGTTTTGTGGGTCAAGGGTGGGTTG

GCCGGGGTGCGGGCCGTGGTGAAACGCGTCAGTGCCGGGCACGGGGGGAAGGTATGCTGC

GGAACGAGCTGTGTAAAAACTTTGAACAACAAGGCGGCGAGGCGTTTGTCGTTTCCGGAC

GAGGCCAGCGCGGCCACGAGAGGTTTGGGAGACCAGCCTGAAGACGTTGTCGTGACCATG

CTGGAGGAGGCCGAGGGAGGCCTCGCTGTTGGCGATCTACAGAAATATCTGGACGACCCG

ATGAGCGACCCCGACGTCCGGCGCGATGAAGAGACGCGGGCCACGACCGTGGAGCACGTG

AAGCTGCTGAGGAACAGGCTGGAGGCGACGACAAAGGAGCACATCATCGAGCTACGGTCC

TACCTCGTCGAGGCGACGGAGGACCTTTCCCGACAGGTGgaggacgcggcggcggcgggt

accGGGGGCAACACCGGCGGGGACAAAGGGAACGAGTTCTGGCCGAGCGAGCTCGAGGAT

GTCTTGAACTCCAGGTTTAGCGAGCTTGATCGAAAATTGGACGCCAGGACGACGGTCCTG

GAGGAGAAAATTGGGTCGCTGATCGACGAGCGGTTAAGCGCTCTCGTGCAGAAGCTGGAC

GTAGTCATCGGAAAGCCGGCCGAGTAG

>g12503.t1

ATGTTGACGGAATTATTGGCCTGCAGGTACGCGCTCAACATCGGgtacaacatcaccaac

aagAAAGCGCTTAACGCCATCGCCCTGCCGTGGAGCATCAGCGTAGCCCAGCTGGTCGTG

GGCTCCATCTTCGTCCTCCCCTTGTGGTTCCTCAAGCTCCGCGAGGCACCCGGCCTGACC

ATGGACAACGTGAAGGACCTGTCCCCCATCGCGCTCTGCCACATGCTCAGCCACGTCTGC

GCCGTCATCGGCCTCGGAGCCGGCGCGGTTAGCTTCGTGCACATCGTCAAGGCCGCGGAG

CCTCTCTTCACCGCTCTGTTCAGCGCCATCTTCCTTAAGCAGATCTTCGCGCCGGTGGTG

TACCTGACCCTCGTGCCCGTGGTGGCCGGTGTTGGGCTTGCCTCCCTCAAGGAGCTTGAC

TTCAAGTGGGCTGCTCTCCTCGGCGCCATGGGCTCCAACCTCGCCGCCTCCACCAGGGCC

ATCCTGTCCAAGCGCTCCATGGGCATGGACATGGGCAAGAACATGAACCCCGCCAACCTG

TACGCGGTGCTCACGATCATGGCCTCCGCCATGCTGCTGCCCCTGTCCGCCATGGTCGAG

GGCCCCAAGATCAAGGACCTCTGGGCGGCGACGGTGGACACCACCGAAAAGGGGAACGAC

ATCATCTACAACACCGTCGCCAGCGGCGTGTTCTTCTACCTCTACAACGAAGTGGCGTTC

CGCTGCCTGGGCGTCGTGCACCCGGTGACCCACGCGGTGGGTAACACGTTCAAGCGCGTC

TTCCTGATCGCCACCTCGATCATCGTGTTCAAGTCCAAGCTTACCCCCATGGCCGCCTTC

GGTTCGTTCATGGCCATCGCGGGTGTGCTGATGTACAGCCTCGCCAAGGAGTGGACGACC

AAGAACGCGGCGGCTGCGCAGGCCTCCaagtaa

>g12515.t1

ATGCGGACCTCGATACCATGGGGaagggcagcagcggcagcgacaaccCTCGCGGTAGTC

ACCGGACCTCTCGCAGCGCTGGTCCCGGCCCCCGGCGTCGCGGAGGCGTTCGTCACCCCC

GACCTCTTTTtcgcggcctcctcctcctcctcctccgcgacggcgacggcggcaacagca

gcaaagccCCATCACGGCTGGTCGTCGACCACAGGACCGGCGGTGATGTTCGCGGGCAGA

CCAGCGGTAGCCACTTCCGCCGCGCTTCCGCTTAGTGGTGGAGGTGGGCGAAGTAGCGGC

TGGACGCACTACGCCGTcccagccgccgccaccgccgccagagGCGGTGTCTACCTTAGC

GCCGGGGACAACCCTACCGAGGGACACCTCGCTTTGGCGGCTCGCAATACCGCCTCTTtc

gtgggagggtggggggcgggggtgaagtGTGAGGCAGGCAGGGGTAGACTGTGCAACCCT

GAACCCCTCCGATCAAGCGTTTCCGGAGGCGCTGGGGACGATATGCTGCCGGAGAGTGTT

GAGCTCCCAGCGAGGGGCGTGGATGGCGGTGCCCGCGCgaacgagggggagggggaggag

gaggaggacgacgggaGCTGGAGCGACGCCGAGGTGGAGCGGCGGGTGGCGGAGCGCAGG

GTGTCGAAGGACGACCCCTATGcggtggacgaggaggaggaagaagaagacctCGACGCC

GAGGTTTACGAAGAGATCGTCGCGAATATGACGAAGCCTCTGGGCATTACCATCGAGGCG

TCGACGGACCAGGCCGAGCTGATCTACATCACGAGCGTGGGACAGCAGGCGGAAGACGTC

GGGCTGGCGGTGGGCGACCTGCTTACCGGCGTGAGTGCCGTGTTCGGCAACGACGTGTGG

TCGGTCAAAGGAAAAAACATCGAGAAAGTTCGCTCCCTGGTCCGGTGCAGgacggaGCCC

TTTATCCTAGTGCGCGTGGAACGCGGGCACGTGTCCCTGGAGGAAAGGTGCTCGGAAGGC

TTCGGGATCGTGGAAgacgcggactgctggcggctcACGGAAGACGGGGAGTGCGAGGTG

GAGTACACCAACTTGTGGTCCAAGGTTTATAAAGAGGAGAGGGAAGTgctgacggggggg

gagggggccgcgggggcggcggcgggcggcgggcaAAAGAAGAAGACGAACGAGCCTCCA

CCTCCAAGGGACATTCCGGGGCTGGGCTCTTCGTTTTCAGGAGATCAGTGGGGGGGCAGC

GActactga

>g12592.t1

ATGACGATGTTCAGCTTTGCCCAGATGGTGTGCTTGGTAGCCATGTGCCTTGTGGCATCG

ACCTCCGCTTTCGTAGTGTCGGCACCGGCGGCTCGTGCTTCGGCCCTCGCgacgtcagca

gcagcatcaccgtcTACATCCCGAGCGGCACTCAGGTGCCGCCGCGCCGCTTCCGTGCTG

CGCATGGCTGAGGAGGAAGCCGTTGAGGAGGTGACGGAGGAAGCGGCCGACATCGCGGGG

GACGCCACAAAGAGCGTCCAGTCTTTTTTCGCGCCCAACGAGAACGTGCGCCTGGGCTCC

AGCCGCGACCAGGACGGCAAGTCCAACGTATGGGCCGTCGAGCCCAAGATGAAGGTCGAG

GGGACGGACGTGGAGAGCCCCGAGGGAAacaagctggtggtggtggggggcaTCATCGGA

GTGCTGGTGGTAGCGATGGCGGCAACGATTGCCCTCCTACCCGCCGCGGACAGCCTCTAA

>g12692.t1

ATGACGTCGTCATCGGAGATGGACGTCGTGGACGGAGACATCGTGGTTGTCGTCGGCGCG

TCGGGTGGTGTCGGTCGCTTGGTTACTCAGAGCTTGGCGGCCACCGGCAAGTACAAGGTG

AAGGGGCTGGTGCGCAACCTCGAAAAGGCGAAGGAAGCACTGGCGGACGGGGAGGGTCTG

GAGCTTGAGCTGGTGCAGGGGAACATTGTGGACGAGGAAAGCCTGAACGCGGCGatgaag

AATGCGGCGTGTGTGGTGGCGTGCACGGGAACCACCGCGTTTCCGTCGGCCCGATGGGGC

AGCGGCAACACTCCTGACGCCGTGGACAACGTTGCCGTCGGGAACATGCTGCGGGCGGCG

GCATCCCCTTCCAACCATCCAGAtgGCGGCAAGCTGAAACGCttcgtgctgctgtcctcg

gtcGGGGTGGAGAGGGCGGACAAGTTCCCGTTCGTCATCCTGAACGCATTCGGGGTGCTG

GACGCCAAGGCCAAGGGGGAGGAGGCCGTCAGGAGGGCCGCAGAGGAAGGGGGGTTCAGC

TTCTCCATCGTGAGGCCGGGCCAGATTAAGGGGGATCCGTTTTCGAGCTACTCTGCAAGT

GGAGTGAAATCCACGGCAGCGGGCACCGAGGGGGCAAGCGGGAAAGCGCCCAAACGCATG

GTGTCGTTGCGGCAGGGCGACGAGGAAGCCGGAGACGTGAACCCTAGTTCAGTGGCGGCG

GTATTTACTCAGGCTGTTGGGCAGCCTGGAGCGGCCGGCAAGAGCTTCACCGTCGTGAAC

GTTCTCGGGACTGACCCAACCCAGGGAGAATGGGACACGATTTTTTCCGAGCTTTGA

>g12722.t1

ATGGCTGTTATAGGAAGCCTCGCCTTAGGGCTCTCGCTTGTCGCCATTGGCCAGCCTGTC

AACGCGTTCGTGGCTCCTTCGTTTGCGGGGACGAGTGCGCTCCGGCAGCTGGACTTGCAG

GTTTCACGACCGGCGGCACGTTTGGCTTCGTCGGCACCGCAGCTCGCTCGTTCTTCTTTC

GCCCCGGCGGCATCAtgcacagcagcaccggcgCGCTTGCAGCAGGGTGGCAGCGGAGGA

CTCTCTATGAAGGTTATCAACGTGGGCGTGATCGGCGCAGGAAGGATCGGGTTGGTCCAC

TTGGAGGCGTTGGCTAGCTGCGCCAATGCGAAgcccatcatcatcagcaaccCCACCGTT

TCCAAAGCGGAGGCCGCCGCGGCGAAGTTCAACGTGCCGTATTCGTCGGCGGACGCTATG

GACGTCATCACCCACCCCGAGGTCGACGCCGTCTGGATCTGCTCTCCTTCTCAGTTCCAC

GCTGACCAGATCAAGGCGTGCGCGGCGAACGGTAAGCACGTGTTCTGCGAGAAGCCCATC

GCGACGGACGTGCCCGAGACGATCGAGGCCATCAACGCCTGCAAGCTGGCCGGCGTCAAG

CTCATGACCGCCCTGCAGAGGCGGTTCGACCCCAACTTCGCCCGCGTGAGGATGGCCATC

GCGGACGGGGAGGTCGGCGAGGTCATACAGGTTAAGCTTACGTCTCGCgacccttccccg

cccccggtGGAGTACGTGCGCGGAGGTGGCGGCATCTTTAAGGACATGGCGGTGCACGAC

CTCGACATGGCCCGGTTCCTCATGGGCTCGGAACCTACGCGCATCCTGGCCTCCGGGTCA

TGCCAAATCGATCACGCGATCGACGAGCTGGACGGAGCAGAGGCGTTCGACACGGCGATG

ATCATGGTGGAGTTCGAGGGAGGGCGGACGGCCGTAATCGACGTGTGCCGCCAGGCGCCG

TACGGATACGACCAGCGGGCGGAGGCGTTGGGCAAGAAGGGGATGATCCAGACCGACAAC

ATGTACCCAAACACCGCACGAGTTTACCTCAAGGGTTTCACCGGAAACGCCGACATGCCG

TACGACTTCTTCATGAGCCGTTACAAAGAGGCGTACATCCAGGAGACGCTTGCGTTCTGC

GAGTGCCTGGTCAACGACAAACCGTCACCGTGCTCCGGAGAGGACGGGCTGATGGCGCTT

ATCATGGCCATCGCCGCGGGAATATCCGCAGAGGAGAACCGCTGGGTTAGCTTCAACGAA

ATCCCCGAGGTGCGCGCGTGCGAGACGCCGGACAGCTGCGAGATCGTGGACCCGATCGAG

TTCGCCAGGACGGGCGAACTCTCGGAGGAGGGCGGAGAAAGCTTCTTCCAGAAAGCCAAA

GAAATGCTGGGCATGGCCAAGGCGTAA

>g12762.t1

ATGGGCTCAGGGTCATCGCGTCAGGCCAAATCGCTGAGCCAAGATCTCCGTGGGAAGCAC

TTTGTGGTGACAGGCGCCAACACCGGGATAGGGTATGTCACCGCCCGCGAGCTGGCGAAG

ATGGGGGCCAAGGTGACGTTAGCGTGCAGGAGCGCCGACAGGGGCCAGCAGGCGGCCGAC

AAGCTCCGCGGGGAGGCGCTGGCAAAGCCCGTTGAGGAGGGCGTGGATCTGCTAAAAGGG

CTGGAGGACGTCGACGTGGAGGTGCAGATCTTGGACCTCGGATCTTTGCAGTCGGTGGTA

GCCTTCGCCGAAAGGTTCAAGGCGTCCGGGCGCAAAGCCAACGTCCTTATCAATAACGCC

GGGATCATGGCCATCCCGGAGAGGCGTGAAACGGTCGACGGCCTCGAGATGCAGATCGGC

GTGAACCACTTCGGCGGGCACCTCTTGACGAGGCTGATGGAGCCCATGATCACTGACGGC

GGCCGAGTCGTGTTCCTGTCGTCTCTGGGGCACGACAAGCCGCCCGGCTTTGCCCAGACG

ACCATGGACTGGGACAACATCAACTACGATAAGCCTGATACCTACGACAGAGCGATGGCG

TACGGGCGGTCGAAGCTGGCGAACGTCCTGGACGCGAAGGAGTTCGCGAAGCGCCTGGCC

GGCCGCGGAATCAACACCTACGCCGTTCACCCGGGTGTGGTCAATACTGATCTCATGCGC

AACATGACGGACGACAGCTGGACATCGCGCATTTCAAGGTGGGCGGCCCCTCTCATGAAG

TACGTATTGTCGACTCCATTGACTGGATCTTTGACATCCCTTCGATGTGCCGTGGACCCG

GCTCTGGCCGCCCCGGAGTGTTCGGGAAAATACTGGGCCAACATGAAGGAAACGACACCG

TCTGCCATAGCGTCGGACCCCGCCAACCCGCCTCGCATGTGGGCCTACACGGAAGACGTC

CTGGAAGCGAAGCTCGGGAAGAAGGTGGACACGGTGCTTGCTCCGTAA

>g12778.t1

ATGTCAGTTTCTGCTGACGATgacgaggacagcagcagtgggccgTTCACGCGCAAGTCG

CTCCTGGAAGGCGTCACCAAGGCTGCCGGGGTACTCGGTGCGGGAACGTTCGTGCAGAAA

GGCTTCTTTGCGGGCGTACCGTACCACGGCACACCGGATCTGACTGGCAAGACGGCGGTG

ATTACGGGCGGGAACACAGGGCTGGGGAAAGAGACGGCCGTGAAGCTCGCAGAGCTCGGA

GCCGACGTGACGATCGCCTGTCGAAACCCAGAGAAGGCGTTTGCGGCGCTGGAAGAAATC

AAGGCCAAGGCGCCCGGCGCCAAGGTAACCGCGATGCCGTTGGATCTCGCGTCGCTCGCC

AGCGTGGACTCTTTCGCCAAGCGCTACTCCTCCTCGGCCGGCGCACTGGACATCTTGGTC

AACAACGCGGGGGTGATGGCCATCCCGGAGCGACAGGCAACCAAGGACGGCTTCGAAATG

CAGTTCGGGACCAACCATCTGGGTCATTTCCGCCTCACTAGCCGGCTGATGCCGAGCCTG

CTCAAGAGCCCCGACGCTCGAGTGGTGTCCGTTTCCAGCTCGGCCCATCAGTTCGCTTCG

ACTGTGGAGTGGGACGACCTTAACGCCGAGAGAGAAGGAGCTTACCAACCCTGGAAGGCA

TACGGCTTGAGCAAGCTCAGCAACATCTTCTTCGCGAAGGAGCTCCAGAAGAGGCTGGAC

AGCAAAGGCGCCTCCGTAACTTGCACGGCGCTCCACCCGGGCGCATGCCGCACCGAGCTC

GGCCGCTACCTCTTCGACCCCTCCCAACCGATCAACCCTCTGGCCTACCCCCTCTTAGCG

GCCGCTACCCTCGTCACAAGAACACCACAAGAGGGAGCGCAGACGCAGATCGCTTGCGCC

GCGGACCCGGCGCTAGGGGCTGGGCGCGGGGCCGGGGGGCAGTACTACGTGGGCCCCAAG

ATATCGGAGCTTCCCACTGCGCTTGCGAGGAACTCCGAGGCGGCGGGGCGGATGTGGGCG

GCGAGCGAAGGGCTAGTCGGGAAGTTCGACGTGTGA

>g12850.t1

ATGAACTCCGGTTGGTGTGACGACCCCGATCACAGGGCAGCTCCTGCATTCCTTGGCCTC

GGGGAATTCTGCTTCACGACAGACGGATGCGAGTGCGAAGTTGGGGCGTGCGACGTCACG

TGTGGAGGAGTATTTTGCCCTCCCGGGACCAACCTGTGCGACGACCCCCACATGCAAGGC

CTCCGCGGCCAGACGATTGACTGGTCTGGGGTCGACGGGGCCTGGTATAGCATGGTGAGG

GATGGCGACGCCGATCTCCATGTCAACATCCGACTCACTGCACCTCTGCCCGAGGAGTTT

CCGGACCGGCAGCTGATCACGGGCCTCAGCGTCTTGTCAGAGGGGCATTCTCTTACAATC

GAGGTGAAGAACCCGTATCGTATCGACACTCGTGAAGCCTGCCCCCGAAGGATCTCGCCC

TGCCTGGCGAACGGCGGACTGCGTGCTGTCGTGGACGGGAAGGAAGTGGATGACCTCCTT

CGCTTCTCCAGAGACGAACACGCCGTGGACGGCATCACCGTATCGGCGGCGAACCTACCG

GCAGAGTGCCGTCAGTTCGGCGGGGACAAGATCTGGGCCCGCATGTACGAGGAAATGCTG

CAAGGAACCCGCGAGCTGGCCCCGGAGGAAGCATTCGAAGACTGGATCCTACGCTTCGAC

GACATGGCGGCGCCCGGCTGGTGCACGAAGTACATCGCGGAGCACGGTCTCGCCGATGTG

CAGTCCATCCATGCCGTCTTTAAGATAGTGACGCCCACCGTCACCGTGCGCCTCAACGTC

GGTGCAAACTCCCAAGGAGGTGGTGACCTAGACTGGGATGGTCGCGTCTTGCCCGAgttg

gagttctggcagatggacgtggggctGCACGGCCTGTCCCTCGAAAATGAGTCGCTGTCC

GGAATCCTGGGCGAGACGGCCCGCCCGGTGCTTGACAAGGACGGtcgcgaggtgatggaa

gggTACGAGGCGTTCCGCGGGACCGTCGAAGACTACCGTGTGTCCGGCCCTCTGGGCACC

GATTTCGCCCTTCTCAACAAACTCTAA

>g13033.t1

ATGGTCGGACGTTCCTGCCTATTGGCGGCTGCTCTTTCGGTCGGGGATCTAGCATGGGGG

TTTCACTTTGTAGCACCATCGAGCGGGGTTCGGGCGCAGTCAGAGCTTAGCAACAGCCGG

TGGGCAACGTCCCCAGCGGCACCGAGGCACTCCAACCTCGTCACTCTGCAGGCGGTGGCA

CAGGATGCGACGAGCAGGAAGGCGCCGATCCTGGTGACGGGAAACAACGTGGAAGTGACC

GAGCCGCTGAAGGAATACATCGAGAAAAAGATGGTCAATGTCCTcgacaagGTGGGGTCG

AGCGTGTCGAAGGTGGACGTGCACCTGAGCGTGAACAAGAACCCGCGCGTCAGCGAGAAC

CACAACACGGAAGTGACGGTCTTCTCCAAGAACCACGTCATTAGGGCCACCGAGACCAGC

GACAACATGTACGCCTGCGTCGACCTGGTCACCGACCGCATCCGGAGAAAGCTGCGCAGG

TTTAAGGAGCGAAAGGTTGACGAGACGCGCAGCAGGTCTGGCGTCGGAAACTTGTCGGAA

CAGGTGGCGGAGAAGGACCAGTCCAGCGCCGCGGCGGCCGCAGAGGCGgCGGGAGAGGAC

CCTTTCGAGGACAAGTACGGGGAGCCTGAGGTGGTTGTGGACATGTCGCTCGTGAAGACC

AAGTCCTTCCCCATGCCGCCCATCAACGTCGATGAGGCGGTCATGTGCCTCGACTACATT

GACCACGACTTCTATCTGTTCCGTAACGCAGAGACAAACGAAGTGAACGTAGTCTACAAG

CGCAACAGCGGAGGCGTGGGGCTCATCCAACCGGAGGCCGACAAATaa

>g13035.t1

ATGGCAGACGCGGGGGTTGCCGAGACTGAAGCAGTCGAAGACAAGAGGAAGAAGGTGGTC

GTGATTGGGGCGGGGTGGGCCGGGCTCGCCGCAGCTTATGAGCTGTCGAAGCAGAACGAG

GATTTCGACGTGACTTTACTTGAAGGAGGCAAGTCTGTTGGGGGGTTGGTGGCAGGATGG

CTCACCCCAGGAGGCAGGCCGGTGGAGGCAGGGGTGCACGGTTTTTGGTACCCGTACCGG

AATATCTTCCAGCTGATCGAGAAGGATCTAGGCATCGACCCTTTCACCCCCTGGACACAG

TCCGCGCAGTACAGCCCCGAAGGCCTCGAGGTAATATCTCCGATTTTCCAGAACATGCCC

CGACTGCCGTCGCCGCTGGGGACATTCTTGTACCCCAGCTTTCTGAGACTGCCATGGGCT

GACCGCCTCTCAGCGCTGGGAGTGGTCGCCAGCGTGGTGGACTGGGACAACACCCCGGAG

GCCTGGCGAAAGTACGATAAGTACACAGCCAGGGAGCTGTTCCGGAAGATGGGATGTTCG

GAGAGACTTTACAAGGATGCTTTCGAGCCTATGTTGCTGGTCGGTCTCTTCGCGCCGGGG

GAGCAGTGCAGCGCggcaggGGCGTTGGGCATGCTTTACTTCTTCATCCTCGCGCACCAG

GCGGACTTCGACGTCAAGTGGTGCCGGGGAACAACGGGCGAGATGATCTTCAAGCCGTGG

GTGCAGAGGATAGAGCAGAACGGCGCGAAGGTGCTTGTGGAAAGGCGGGTAACCGACATC

GAGGTTTGTCCTGAGACGGGAAGCGTTACTTCCGTGTCGTGCGGGGACGAAACGTTTCCG

GCGGACGCTGTGGTGTCGGCGGTCGGCATCAACGGCGTCAAGGGCATCGTCAGAGCCGCG

TCCGGGCTGTCCCGGCTGCCGTTCTTCTCTAGGATGATGAACCTCAGGTCTGTAGACGCT

TTGGCGGTGAGGCTCTACCTGGACAGGAGGGTGAGGGTGCCTTACCAAAGCAACGCCTGC

TTTGGCTTCGACAAGACGACCGGTTGGACTTTCTTCGACCTGAGCACAATGCACGACTCC

CTCAGCCAGAGCGCTGGAACGGTGCTAGAGGCCGACTTCTACCACGCCGACCAGTTGCTA

CCGCAGCCGGACGAAGACCTGGTAGCCAAgGTTCAACGCGATATCGCAGTGTGCGTGCCA

GCGGTGGGGAGAGCGAAGGTGGAGGACTACAGCGTGGTAAGGATCGCACAGGGGGTGACC

CACTTCTCCCCGGGATCGTACGACAGCATGCCGACTTGTAAAACCCCGATACCCAACATG

TTCATGAGCGGGGACTGGGTTATCAGCGACCACGGCAGCTTCTCACAAGAGAAAGCCTTC

GTGACGGGGGTAGAAGCGGTCAACCAAGTGGTGAAGCGACTGGGGGTCGGGCGTCCTGCT

TCGATTATCCCTCTCGAGGAGGACGAGCCTCACATCCAGGCGTTGAGGGCAGTCAACGGC

AGGGCGAAGCGAGCGATCAAGGCTTTGCCCGGCTCAGGATGGCTGCTCCCATGA

>g13207.t1

ATGAAGCTCGCCGTGATCGCCGTCGCTATGTCTGCGCTCAGCCCTTCGGCTGATGCGTTC

GCTCCCTCGTCCGCTTTCAACGGAGCGCGAATGGCATCCGCCTCGACCACCCGCGCATCG

AGCTCCGCCATGGAGATGTCCGCCGCGGACGACTTCAAGAAGGCTGCCACCGGTGCGCTC

GCCGTGTTCGCTGGCCTGTCCATTTTTGCGGCACCGCCGGCGGAGGCGATCACGCGGGAC

ACGCTCGACTCGCTCTCGTACACTCAGGTGAAGGGGACTGGTCTGGCCAACCGATGCCCG

GATGTGGTGGGAGACGAGACCATCAGCGTCAGCGGCAACGCGAAGATCGTTGACATGTGC

ATCGAGCCCAAGAACTTCCAGGTCTTGTCAAGGGTGGCACGGGGGGGCAACATGGTGCCG

CAGATGATTGACACGAAGCTCATGACGCGTGAGACGTACACCCTCTACGGCATCGAGGGA

GACTTCGGGAACAAGGACGGCAAGCTGACCTTCACGGAGAAGGACGGCATCGACTACGCG

GCGACTACGATCCAGTCGAACTACGAGCGTATCCCGTTCCTTTTCACCGTCAAGGACCTC

GTCGCCAAGGCTTCCTCGGGCGGCAACCAGGTGAAGCCCGGGTTCCAGATGGGCGGCGGC

TTCCGCACGCCATCCTACCGCACGGGTCTCTTCCTTGACCCCAAGGGCCGCGGTGGGTCG

ACGGGTTACGACATGGCCGTGGCGCTGCCCGGACTCCAGTCGGGTGTGGAGGGAGACAAG

GAGATGTTCAAGGAGAACAACAAGGTGTTCGATGTGACCGACGGAAACATCGAGTTCGAG

GTTAACCAGGTGGACAAGGAGGCCGGGGAGATCGGAGGTGTGTTTGTGTCGAAGCAGAAG

GGCGACACCGACATGGGCTCCAAGGTGCCCAAGGACATCCTCGTCAAGGGTATCTTCTAC

GCTCGCATCCAGTAG

>g13371.t1

ATGTGTCAGGTCGTGGTTGATTGCACAACGGTGGTGTTTGGGGAGACCACGGGAACAGGC

ATCGGACCTTCCCTTGTGGCTGCTGGCGGCGGGCGTGTCAGCTGCAGGAGATGGGTCATG

AACGCGGCGGAACGTGCTGACGAAGACGGTCTAACTCGCCGCGAGGTGTTGAAGAACATC

GGGatagccgccaccgccgcggcctgTGCTACTACCGGGACATCACTGGTGCAAGGCGGG

CCTGCCGCAGTTCTGGCCGCCTCCGATGATCTCCCGGCGGAGGCGTACACCACGATAGGG

GGAGACATGAAAACCTGCCGTGTTTTGAATGGCATGTGGCAGCTTTCGGGCGCTCACGGG

TTCCTCCCCGAAGAAAAACCCGCCCTCCAGGCGATGGGACGGCTTGTGGACAAGGGTTAC

ACTACCTTCGACCTTGCCGACCACTATGGCCCGGCGGAGGACTACGTTGGCGCCTTCGAG

AAGCAACAGCAGGCGCGGGCTGCAAAGGGCCAGTTCTTCACGAAGTGGGTGCCGCGGCCT

ACCCGGATGGACCGAGCCACCGTCGACGCCGGGGTGGGACAGTCCCTCGCAAGGATGAAG

ACGGAGCCCCTCGACCTAATGCAGTTTCACTGGTGGTCCTACGACGCCCCTTACTACCTG

GACGCCCTCAACCACCTTCAAGACATGAAAGCGGAGGGCAAGATCAGGCACGTCGGCCTG

ACGAACTTCGACACGAAACACCTGCAATATGTCACGGACAAGGGGGTCCAAATCGCATCT

AACCAGGTCCAGTACAGCATCCTCGACCAGCGCCCTGGGCAGCGGATGGCCGGACTATGC

GAAGAGAGGGGGGTGAAGCTTCTCTGCTACGGGACGGTGCTAGGGGGGTTGCTGTCTGAG

AAATGGATAGGCCGCCCCCAGCCCACGCGGGCGGACTTCACCACGGTGTCCGAGATGAAG

TACTTCAACATGATCCGGCAATGGGGGGGTTGGCCGTTGTTCCAAGAGCTACTGTCCGCA

GTCAAGCAGGTTGCGGATAAGCACGACGTCAGCATCCCCAACGTAGGGGTTCGATGGGTC

CTTGACAGGCCGGGAGTAGCCGGGGCCATCGTGGGAACTCGGTTGGGGCTGAAAGATCAC

TCAGATGACAACAAGAGAGTTTTTGCACTGAGGCTCGACGATGAAGACCGAAGCAGGATA

GCGGCGGTGACAGAGCAGTCGCGCGACCTGATGAAGGTCATCGGCGACGTCGGAGACGAG

TACCGGGGCTAA

>g14116.t1

ATGAGAGCACTGCGACTAGTATGGGGCGGAGTGGTCCTGGCTCTTCTGGACTACTTCGGC

ACCGGTTGTCTGGCTGAGGCGCAGGGCACTCCAGCCCTTGGCGTCTCAGACACTACCGCC

GGGGAGTGCTGGACATTCACCCACCAAGTTAAAGCCGGTGGGTCCACCGTGAAGGCAATG

CTTAGAAACATGTGGGGGCCACAGTATTTCACCTACGGCGATCGCGAGTGGCACAGGGGG

GATGCCTACGCGCAATCGATTGCGGAAGACCTTGTGTATGGGCGCAACCATAGTGTCGTC

GCAGGCGGGTATGTGGAGTCACTGCGACGCTCGAGTGACGTCGGATCGAAGTGCAAGTGG

TTCACCGTGTTCCGGCACCCGATTTCACGCTTGGTCTCGGCATACTACTTCTGTAAAGTG

ACTGGGACCTGTGCGTCCGAGCTAGTGAACGCGAACCAAGTGGATATAACTGCGTTCGCA

AAGCATTGGGGAAACCGCGCGTTGAGGCAGTTCGTGCTGAGCTTCGTGTCAATCGACGAC

GTCATAGACTACTCACGAACAGATGCGGTGCTAAACCACTTACCGCCAACCGTAAAGAGA

CCGAATTCAATCCCCGGTGGGTTCTTTGTCCAAATGTACCTGGAAGAACGCACCCACGCG

CCCAACAGCGATGATAGCCATCCGGACGCAGTCTTGTACGCGATGTTACAGCCGGTACAG

GACATGCTCCGCGATCAGTATTCAGCTATCGGTATTTTAGAGGAGTATGACACCACGCTT

TCAATTTTCAACGCCGCGCTCGACATGCCTGGGGTCGACTGGCACGAGGCGTTCAAGAGC

GTGGGTCACGTCAACGTCAACGACGAATACAAGGACGAGAGGGTGGCCACGCTGGCGGAG

GCGTGGACCAACTCCGAAGTTAAACATTACCTGCAGCTCGATATATTGTTGTACGAGCAC

GCCCTCGCTATCTTCCAACAACAGGCGAGCGCTTACAATATCTAG

>g14210.t1

ATGCGCTTCTTCGCTATCCTGGCCACCATCTGCGCTGCCACCGTCGGCACTGCGTCAGCC

CTTGTTGAGGCGGGCGACAGGACCTGGGTGGACGTGGACGCCAACGGGTACGACACGAGG

AAGTCTGGGAACGGCTGCAGCCCGAGTGGTTGCGTACCCTACAACACCCGCGACAACGAT

GTGTGGGCCAACTCTCGCTGGTCTTGCAAGGGGGACATCCTCGACAGCTCTGACAAGGAC

GATGGGTGCTGCATCACGTACTCCTTCGAGGAACCCCAGGACGTCGTCAGCATGAACATC

GCCTTCCACAAGGGGGATGAGAACACCAGAACCCTGGACGTATTTGACAACGGCAACCAC

CACAGCACCATCACATCCAGCGGCAAGACCCTCGACTACCAATACTTCAACCTCTACACC

GACGAAACCAAGACACTCAAGCTTTGCCTCGACGACCCTAAGTGGTACAATACCGTCTGG

CTGAGCATCACCGAGCGGGTAGTGACCCCGCCGGAAACATTACCGCCAGTGCCCGACTCG

GTGAGCGGGGTGGTGAGGAAGGCACCCTCCCGGGGGTACCTCGCACTGTCCGCTGAAAGG

CGGGGGTTCATGGTGCCCCTCGTCGAGGCGGATGACAGGACTTGGGTCGACGTGGATGCC

AACGGGTACGACACGCGCAAGTCTGGGAATGGCTGCAGCCCGAGCGGTTGCGTACCCTAC

AACATCCGGGACAACGATGTCTGGGCCAACTCTCGCTGGTCTTGCAAGGGGGAAATCCAC

GACAGCTCTGACCAGGACGACGGGTGCTGCATTACTTACTCCTTCGAGGAACCTCAAGAC

CTCGTCAGCATGAGCATCGCCTTTCACAAGGGGGATGAGAACACCAGGACGCTGGACGTG

TTCGACAACGGCAACCACCACAGCACCATCACATCCAGCGGCAAGACCCTCGATTACCAG

TACTTTAACCTCTACACCGACGAGACCAAgaccctcaagctttgcctcgaTGACCCTAAG

TGGTACACTACCGTCTGA

>g14213.t1

ATGCGCTCTTTTGCTATCCTCGCTACCATCTGCGCTGCCATCGTCGGCACGACTTCAGCC

CTCGTCGAGGCGGGCGACAGGACCTGGGTCGACGTGGACGCCAACGGTTACGACACACGC

AAGTCTGGGAATGGCTGCAGCCCGAGCGGTTGCGTACCCTACAACACCCGGGACAACGAT

GTCTGGGCCAACTCCCGCTGGTCTTGCAAGGGGGAGATCCTCGACAGCTCCGATCAGGAC

GACGGCTGCTGCATCACGTACTCCTTCGAGGAACCCCAGGACATTGTCAGCATGAGCATC

GCCTTCCACAAGGGGGACGAGAACACCAGAACGCTGGACGTATTCGACAACGgcaagcac

cacagcaccatcaCATCCAGCGGCAAGACCCTCGACTACCAGTACTTCAACCTCTACACC

GACGAGACAAAgaccctcaagctttgcctcgaCGACCCTAAGTGGTACACTACCGTCTGG

CTGAGCATCACCGAGGTCCAGTTCTGGGTGAAGTAA

>g14243.t1

ATGAAGACCTCCAACCTAGCGCTGATGCCGTCGTCTGTGGCTGTCCTTTTGCTGGCCATG

ACCACTGTGGAAGTCCGAGGCTTCGCTGTACAACATGCTATCTCGACGTCAGGATGCGGC

GCAACCTCCTCACGACATGCAGGAAGGGCGGCGGCATCTTGCAGGGTTTCCATGACCGCG

ccggggggcagcagcagctcagagcTGTCCCGCACCGCGTGGGTCAGCAGGGTTCTTGGG

GCGGCCTTCACGGGAGCAGCCGTAGCAACACAGGGCCCTGCCGGAGCCTTGGCCGAGCTG

GGAGTCGGAGAGGGGGGATTGCCGGACGGCGCTCGGCAGTTCTCCAACCTTGTCAAGGTC

CAGAAGGACTGGGTTGCCCTGGGGAAGACGGTCAAGTCGCAAGGAGCGGAGGTTTCCGCG

GGGGAGTGGAAAAACGTTGCTCTTTTCTTGCGCAAGGTTTACCAGTTGGGCGGGGACCTT

GAGTTCCTTGCCGGGACGTTCTCGTCGGACAagaagaaaacggccatggctctCGTTCGT

GGCATCCAGAAAGAAGTGAAGGCGGCGGACAAGCCGGCGCGAGAGAAAGacgtggaggcg

tttttgtctGCCCAGGCCTCCGTGGAGAAGAAGTTCGAGGACTTCATGGAGCTCTTCAAC

GACGTCCCATCCGAGCTTTGA

>g14398.t1

ATGACTCGCACTGCTTGCATGGctctggcgacggcggcggcggtggtgctcgGCGCGGCA

GCGGTCAACTCGCTGGAGGTGACGACTCCTTCCGAAGGGCAGCCGGTTGTGGCCGACAGA

ACCTTCACCGTTGAATGGACAGGGTCTAACCGGAACAGCCAGTACGAGATCGACCTCTTC

TACTGCGGCTCCTCTTGCGCAGAGGACGACTGCGGCGACTGGGTGGCCGCTCTGTGCACC

AACGAGAGCGGCTGCAGCGACCTGCAGGGCAACTACGACGTCGTCATGCCCGAGCCCCTC

GACGGCGTGTCCGGGTCCGGGTACAAGGTCCGCGTGATGGACATCGCCGACGAGTCGAGC

ACGGACTGCTCCGCCGAGTTCATCCTCGTGGCCTCCAACGAAGCGCCCTCGGTGGGCGAC

GCAGACGGTCCCCACCTGGAAGTGATCTCCCCCCAGGACGGTGACATGGCGTTCGCCGGC

GAAGAGTACACGATCGAGTTCGACTACGACGACGGCTTTGGATCGTCGGCTGACCGGTTC

AGCATCGACCTCTACAGggccgacggcggcagcggcgactgcGGAACGTACGTGACCTCC

ATCTGCGACAAGCCGAGCATCGGCTGCAGGGACTCCGGGGGGGACTACGACGTCACCATC

CCCGAGGACACCGCCTCCGGCGAGTACCAAGTCCGAGTGGGCCGCTACGAGAACGAGGAG

CTTTTCGGCTGCTCCGGCACTTTCCTGGTCTTGTCCGAGGGCGGGGACGAGCTGAGCATG

TCCTACAGCTTCGGCGCCGGGGACGAGGACGAGCTGGGCATGTCCTTCAGCTTCGGCTTC

GACTCCGACGACGACGATCTCTTCTTCTAA

>g15140.t1

ATGCTCTCCCAGTCCTCCATGAAGGCTTCTATCCTGGCCCTGCTTGTCGTCTTGCCCTGC

GACACGCACGCGTTTGTCCCACAAATGcccacagcaacagcgcagACGATGCGGGGCTTG

TCAACAAGAGCGAGTATGCATgctgacagcaacaacaacagcagagggacAGCGCGATCG

AGAATCGAGCATGCtgttgccgtcgccgtcgcATCGTCTGTCGTCATCTTCGGGGCCGCC

AGTGGCCCAGCCTTAGCAGAGGAGCTCCCGCCTGGCACCAATCCCTTCACGTCTATCTGC

ATGGGGTTCGGCTGCGGCGAGTTTCAAGGGCTGGATTACCCCGGCGCGCCAGCCCCCACC

GACGAGGAATCGATCACCTTCAAAAACTTCCTGGGTTCGCTGGACAAGGGGCTGGTGGCG

AAGGTGGACTTCTTGAACGGGGGAGATAAAGCCTACGCCTTCTTGAAACCCGCGGGGGAC

GCGAGCGGGGAGCCCACGCGCATCAGAATCGGGGAGGGCTACCCGGACGAGCAGACGAAC

GGGTGGAGCTCTCCGCTGTGGGTGGTCCGCGCATTGAACGACCGTAATGTACCATACCAT

TTTGAATACAACCTTGGCAAGGGGCGGGCAACGCAAACCGTACCGAAGTGA

>g15166.t1

ATGCTTCGAACCACCTGTGTAGTGCTGCTGGCGGTACGACGCTCCGCAGCCTTCTCTCCT

AACCTGTCCAGCGTCTTCGCGGTTGGTGACATGGGGAACAGGGCGGGGAACATGGCCACT

GCAACCCGGCCTTCGACTGCGTTGTTCTCCGCGTCAGGGAAGGTGTCGGCAGAGGAGGTC

TTGAAGGCCCCAAAGTGGCCCGAGAAGTGGCCCTTCTACGATGACGACTTCAACCGCATG

GACGAAACCAGCGATGGCGACTTCTACTCGCAGCCCAGGCTTGTCTACCACATCGACGAT

GCCGCCGTGAAGGCTTTGACGAAGTACTACTCGAAGGCGCTGCCGAAGGGGGCGGATGTT

CTGGACATCTGCTCGTCGTGGGTGAGCCACTTCCCCAAGGATTGGCAGCACGGCAAGCGG

ACAGGGCTGGGCatgaacgagtacgagctgagCAAGAATGAACAGCTGGAGGAGTACAAG

GTGACCGACCTTAACGTAAACGCCAAGTTCCCCTTCGAGGACAACTCGTTCGATGTCGTC

ACCTGCGTCGTCTCGGTGGATTACCTGAACAAGCCCCTGGAGGTGTTCAACGAGATCCAC

AGGGTGCTGCGACCAGGCGGCAAGGCCATCATGTCCATGTCCAACAGGTGCTTCCCGACG

AAAGCCATCCAGATTTGGAACCAGACGAACGACATGGAGCACATCTTTGTAGTAGGGTCA

TACTTCCACTACGCGGGAGGCTTCGACCCGCCAGCGTCGCACGACATATCGCCCAACCCC

GGCCGATCTGACCCCATGTACATTGTTGAAGGCCGAAAAAAGGCGTGA

>g15408.t1

ATGAAGGCCACCGTAGCTTCTTTCACCGCGCTCCTCGCGAGCGCCTCGGCTTTTATGGCG

CCTATGCCTCTTTCGCGCACGGTTGCCCCCTCGAGGTCATCTGTGTGCATGATGGCGAAC

AGCAAGGCGATCCCCTTTATGCCGCAGCCGGAGGGGTTGGACGGAAGCATGGTCGGGGAT

ATCGGATTCGACCCTCTTAACCTTTCtggcatcgacatcgactttaGCGAGTTTATCGTG

CCCGGGGCCGCAGTGATGCGCGAGGAGGGAGTGGATGCGACCAAGTCGCCCGTGGACACG

CTCTACTGGATGCGTGAGGCTGAGCTTAAGCACGGCCGCGTCGCGCAGCTCGCAGTGGTG

GGCTGGATCTTGGTGGACCAGGGGGTTCGGTTCCCCGGGGCACAGTACGCTGCTATCAGC

CAGTCCGTCGGTGCGCACGACCCCATGGTGGCCGCGGGCAACATGACCCTTATGCTCCTG

GGCGCGTTCCTCCTTGAGATGGTCGGGGGAGCTGCGATCTTCGGAGCAGCAAGTGGATCC

GGCCGCGCCCCGGGAGACTTCGGGATGGACCCGCTGAACCTCACCTCCAACCCCAGCAAG

AAGGCGCGATTCGAATTGAGCGAAATCCAGCACTGCCGCCTGGCCATGATGGCCATCAGC

GGCATCGCAACGCAGTCCGTCCTCAACGGCGGCGCTTTCCCCTACACGGGATAA

>g15784.t1

ATGGCAGCGCCAGGAGGAAAGGTTCTCACGTACGACACGATCGCCGAGTGCGTGAAGAAA

TGCGAGTACGCTGTCCGTGGCGAGATCTACCTGGCGGCTACCGAACGCATTAAGGCAGGA

AAGGAGGTCATCTTCACCAACGTGGGCAACCCTCACGGCTTGGGCCAGAAGCCGCTCACT

TTCCTCCGCCAGGTCATGGCTCTCGTCATGGCTCCGTTTTTGCTGGAAGACCCTCGCGTG

TACGACATGTTCCCCGGCGACGCTATCGCCAGGGCGCGTACGTACCTCGAGCACGTGAAG

GGAGGCATCGGAGGATACAGCGACTCCAAGGGGAACCCTTACGTCCGTCAGGAGGTATGC

GACTTTATCCAGCGCCGAGACGGCCACCGGGCCGACCCGGACAACATCTTCCTCACGAAC

GGCGCGAGCGAGGCTGTGCGGTTGGTGTTGCGCACGACCATCCGCGGCCCGAGCGACGGC

GTCATGGTCCCCGTCCCGCAGTACCCCCTGTACTCGGCCTCGGTGGCGCTGTACAACGGT

ACCTTCGTCGGGTACAACCTCTGCGAGGCCAGGGGGTGGGGCCTGGACCTTGCGTCGATG

GAGAACGCTTTGGCCGAGGCGAGGAGGAGCGGCATCACGGTTCGCGCGATGGTTTTCATC

AACCCGGGCAACCCTACGGGCAACTGCCTCACGGTGCCGGACCTCCAGCAGCTCGTGCGC

TTCGCGTACAACAACGGCCTGGTGCTGATGGCGGACGAAGTGTACCAGGAGAACATCTAC

CAGGACAAGACCCCGTTCACCTCCTGCAAAAAGGTGCTGGCTGAGATGGGACGGCCCTTC

GCGGAAACCGTGGAGCTGGTCTCCTTCCACACTGTGTCCAAGGGTGTCTACGGCGAGTGC

GGCCTGCGGGGAGGGTACATGGAGCTCACCAACATCGACGCCAGGGTGTCGGATGAGATG

TACAAGCTGTGCTCCATCAACCTGTCGCCCAACGTGACGGGGCAGGTGGCGCTCGGCTTG

ATGTGCAACCCCCCGAGGCCCGGGTCGGAGTCGTACGCCAACAACATGAGGGAGAAGGAT

GTCCTGCTGCAGTCTCTCATCCGACGGGCGAGGTCGATCACGGACGCCTTCAACAGCTTG

GACGGCGTGACTTGCGAGGAGACGGAGGGGGCCCTGTACTCCTTCCCCAAGATCGTCCTC

CCGAGGGCGGCGATGGAGGCCGCCAAGGCAGCCGGAAAGGCTCCCGACGTGTTCTACTGC

CTCGAGCTGCTCAAGGAGACCGGCCTCTCTTGCGTGCCGGGGTCTGGCTTTGGGCAGGCG

GAGGGGACGTACCACTTCCGCACCACCATCCTCCCGCCGGAAGACAAGTTCCAGAACGTG

GTCGACGGTTTCAAGTCGTTCCACGAGGGTTTCATGGCCAAGTACggagacggtggcggc

ggcggacgctCGTCTTGGCGTGCTCGCCTCTGA

>g15787.t1

ATGTTGCGCTTGACCATCATCGCAGCCGCCATGGCGTGCTGCAGCTCGTTCCTAACGCCG

ACGGTGACGTTGCAATCATCGCACCGCATCGGAAGCAGCAGGGCCGGCCTATACATGTCA

TCCTCGTCATCAAGCCCAACAGAGACATCGTCAGCTTTGCCCGAACCTACTTCCAGGACA

GCCTCCGAGGCCCCTTTCTCGGGACAGATGGATCGCCGTACCCTACTGAGGGCCGTCCCC

GCAGCCTTTGCAGCGGGAGTGATTGGCGCTGCGGTGGTTGCGGCGCCGGAGACAGCATCA

GCACGAGCCACGCCGAAGGCCGCACCGGCTGGCTCTAAGGTTGTTGTTCTTGGAGGAAAC

GGCTTTGTCGGCAGCAAGGTCTGCGAGATGCTCGTCGAAGCCGGCGCGTCAGTCTCAAGC

GTGAGCCGCAGCGGCTCGAAGCCCGACAAGTGGGCCGCCGGGCAGAGCTGGGTCGACAAG

GTTTCTTGGACCAAGGGCGACCCTACGGCCGGAGATATCTCCTCGGCATTCAGCGGAGCT

TCGGCTGTCGTGTCGTGTGTGGGGGTTATCGGAGGCAGCGACGATGAAATGGAGAGGGGT

AACGGAGACGTCAACGTGGCGGCCGCGTCTCAGGCAGCGAAGGCGAAAGCAGGACGCTTC

GTGTACGTGTCGGTCTCCCACCTTGTGCCGGAGGCCTTCGGCGGGGTTGCCTTCAAGGGA

TACTTCGACGGCAAGAAACGAGCGGAGGAGGCCATCGCGGCGTCCTTCCCGTCCACTGGA

GTCCTGATCAAGCCTACCTTCATCTATGGTGGGGATTCTTTCGGGCTGACGCCTCCTAGG

GTGTCGGATGGGTACGGGTCGGGCATCGACGCGCTGCTGTCCTCGGGCGCTATCCGAGCA

GTCGCCGGCATCTCCCCTGGCCTTATCAAGGTTGCGTTGTCGCCACCTGTATCGAGGGAT

AGCGTGGCGCTAGCATGCGTTGCGGGGGCGTTCGGTAGATTGGAAGGGTCCACCTTCGAT

GGAGCAGACGAGATCAACGCGGCGGCCAGCAAAGCTTGA

>g15933.t1

ATGGCGACCAGCGCCCCCCCCTCGATGTCGGAGAAGCCACCGGCGCCGTCTTACACCGCC

GTGCCGCCTTCGCCTGCGTCGATCACCCCTGTCACGCGCTGGCGCTGTTCCATCAACAAG

CCGGTTCTTCTGTCGACCGAAGCCCTGAACAGTCACCAGGCGCAAATAAGGGAGCAGCGG

CGGCTGAAAGAGGAGCAGGGGCAGCGGCGGGCATCGCTCGAGCTCCTCGAGATCCAATCG

CTGAACCGGGCGCTGGACCAGCAGCACCGTCAGGACCAGCGCGAGGTTGCCAGGTTAGCG

CACGAGCGAGCAGAGTTGCTCCGCACTAACATGACTCTGAGGTCCGAGCTCGACGAAGCG

AGGGCGTCGCTCAAGTACCTCCGGTTGTCGAAAGCTCTGGATAAGCAGGCTGCCCAGTCC

GCCAGCAGTAGGAACGCTCGCCACGAAGCAGAGGAAGAGCTGTACCGAGCGGATGCCTAC

TGCAGCACACCCACCGCCACCGAGACCGTCGTTGTGCGTGGTGGTGTTCGGGACTCTGAG

ACAGACTGCAACGACAGCAGCGTTGGCAGCGTCGAGAACGCTTCGTCAACAGAGACTGAG

ACGAGCGAAGACTACGCCTGTCTCCTGCCCGAGCTCGTCGAGCACGTGCACTCGTCCCTA

ACAGGGACAGTTAGCCGATGGGTCAAGGGCATGCAGGCCGCGTGCTGCCCGGAGATCAAG

CAGGCCCTGGTGCTGCCATGGCTGCTGCACAAGCTGTTCTATCTGTGTTCCGAGTTGATC

GAGGAGAAAAGGCAAGAGGTGGCGACTCTTTTCTTGGCTGGGGTAGTCTGCGAGGCAGGC

AAGGAACAGGCGACGATGGGCGTTGAGGCGTCCGAGTCCATGCACCGTCATCTGCGCCGT

CACCAGCTGACCCTGTTCCCTCTGTCGGGAGACAGCCTGCGGGTCGCCGTGGACAAGATC

ATCATGGCCCTCGCCTACAGCACCTCGGGATCAGCGCCCCCGGCCGACAGGTGCGTGGCA

cccccaatcctcagggagtgcaaCGCATACTCCTCTGCCTGCACTCCCGCCACTGACACC

ACCTGTCGCAACGCCCTCGTGGCCTGCGCCTGCGTCCACATCGACCACCTGCCATTGCCA

CAACCAAACGCCACTAACGGTGCCGACGGGGGAAGGAAAAGCTGGGAAACCTGCTTTTCC

CACGCCCACGTGCGAGATGGCGGCCACTCCGCCATCAGGCGGCCCAGCTTCTGCATTTTT

GTGGGTGGCATCGTCACTGACAACTCGTCGGTGTCCACTATCCAACCCAGCACCTCCAAC

CGTGAATCCCAACTAGTAATCTTCCGCGCGGATAACAATGCCGGATCGTCTGGGCCCCGT

TCTCCTAACAAGCGGAAGTGA

>g15940.t1

ATGGCATCCGCACCTGCAGCTATAAGCGGCGCCACCACCGAGGCAACCGGTCTCGCTGCC

TCCCACGGCATCGGCAGCGTCAGCACCAGAGACAGATCAGTCGGAGCGTTACCCATGATT

CACGGCGAGTTCTTGGGGCGTGCTCTTGCGTCAACTGGCGCAGGTCTACCGAAGAGCATC

AACGGGCCGACAACAGGAGGATTGCACGCTGCAGCAGTCGCCCTGCAAGAGCCACAGCAG

CGCGAGGTCGGAGCATCCCCTCCGACGCTGGCAGTGCCTGTTCTGGCCGGGCGCAAACGG

TCGCAGCCAACAGGGGGCTGGAGTGCACGTGGCGGAGTCGATGAATCTCGCGAGAAACGT

GTTAAGGATGCGCCTGAGGCGGCGCAAAGCAGAAAGTCGTCTGGACTGAGatcggcagct

gcagcagcagcggcagcagcagcagcagagacagcagcggtagcagcggcggcagcagca

gtagtgtccacagcagtggcagctctgcccccccctcctcaggtCGATGGCGCCGACATC

CTTCCTGGAGACGATGCCAGCGCAGACAGTCAAGCGCTTGGTGACGAGgcatcgctgccg

ccgccgggggaGCCGCAGGCGGAGCCTGCGGAGCTGGAAGGTGCCGTTATTGATGACGGG

TCTGCTTCTTCCCCGAAAGCAACGGGTACCGAGCTCGCGGTGGACTCGCCCATCATTGTG

GAGGAGAAGGAGCTGCCCGTGTCGTGTCTGGGCGACTTCAAGTTCACGTTCGCGGAGGTG

AACAAGATGTGCAAGCGCTACCCCACCCTCAACACTCTCGGAGCCGAGACCGCCATGCCC

TTGCTTGGCTGGCTCACTCGGGAGTTGGACATGGGCCTGGGCGACATGCGGAATCTTGTA

CTGCGGCACCCTCGGCTTATGGCTTATCGCGTGACTTCTCACGTGGCCCCCAAGACCAAG

TGGCTCCGGGAACGCTTGGGCCTGGGACAGCCGCAGCTGCGAAAGCTCATCACGACCTAC

CCAGCGGTGCTGAGCCGCAGCGTCGAGGAAAACTTGGAGCCCAAGTTCAAATGGTTGGAG

GAGCGCCTCGAGGCAACGCCGGAGGAAGTGCAGGTGCTCATCAAGCGCTTTCCCCTTATC

TTCGGGTACAGCACCTCCCAGAACCTGGAGCCTACGGTGGCGTTCTTCACGGAAGACATG

AGCGGCGGGCTGGAAGAGATCAAGGCCGCGGTCATGTCCTGCCCTTCCATCCTCAGCCGC

AGCCTGGACAAGCGCATCATGCCAAGGGCCCAACAGATGCGAGATAAGGAGATCGAGCCC

CGCTTTGGCCCGCACAAGTGGGTAGTATCCACCTACACCGACGCCCAGTTCAACCGCTGG

CTCGATGGGAGAGGCACGTAA

>g16117.t1

ATGGCGGGGAACCGACTAGACTTTCAGAGGAGGCAACATGTGCATGAGAGTATCACTGCG

GACAGCTTGACCTTCGCCGCCCCCGGAGGAGTTGGAGCGTCTTCGTTCGCAGCGCCAATG

ATGTCCGAGCCTATGGGGTTCCGAGATATCGAACAAAACGAGGCTACAAGAGCGGCGGAG

CGCCTCTCACGCGGCAGCAGTCGCCGTGGACACTCGATTTCGTTTGGGGGCGGAGTGGAG

GAGCTTGCACACGAGCCCGTAGCGGTGGTCCTTGTGCGGAGTAGGCCGGGGTCTGCGGGA

GGGCCCACCATACTGCCGGGGTTGCGAGATTCTCGTGCACCAATCCGTGATCAGGACCTG

TCGAATCAAccCCAGCCTCAAGGTGACCGATGGAATGCGGAAGTCAGTGCTGACCTCCAC

GGTGGTGCCGGctaccccaacaccaacacctcaaTCAGTAGTCGCGCATATCGCCAGACA

ACGGAGCAGCCGAGCGGACCGTGGAACTCGCTCGGACACCGCAGAGTACACTCCGCCCCC

AACATCATGGAAGGGATGAACACCAAACCCTCCAGTTCGGGTTCATCTCGCTGGCGCAGT

TCTGCCCTCCCGTGGCTGGCGTCGTCTGATGCCATATCACCCGGCTTGCGTGAACATGCT

ATACCGGAGGGCCGCCGCTTCGAGGCCCTGGAGCCTCCGGTCCAGTCTGCCGGTGTCGTG

CCCGATCGCCCGCGATCATGGCGTGCAAGGACTGCCGCGCCGCCGTGGGGTGCGGGTCCT

ACATCTATTAACGATTTTGGACAAAGGGACCCCAGCAATCTGGACTGGGCGCCGAAGCTG

AAAGCGTCTTACTTCGTCGATGTTCGCGGTCAGGATGGAGACGACAGCGTCTCCGCGTCG

GTTAGCGGCATCATCCCCCAGATAGCCGCCATAGGCAGCATGGCCGCGGATACGCGCCCG

TTCGAGGAGATCAAGAGGATGCGTCTCAGCCAAGCTCTCGAGAACGCACCGAGCGACGGA

ACCATGTTGGGCTTGACGCCGTCGCCGACGTACTCGGCGTCCGGCATGGCGCACTTCGAG

AACGTCCGCGTCGCACCGCGCTTCTCCCCCGGGAAAGGAGTTAAGGTGCCCGGGTTCGCG

TCCTCTCTCAACTGCAGACCTACCTTCGGCGGTGGTAATGGTGCTGGTatcggaggaggg

ggaggaggaggaggagtaaggGACGGAGTTCGGGACGGGGGCTTTGGCTGGCAGCCCCTG

TCCCCGGCGTTGACTGAACCGCCTACGCCGCCGCCCCGGCCTGACTGGAAGGCGCCGCCA

GACGCCGAGGCATGCTCGCCGCGAGTGGCAGGCGAAGGCCAAATGCAACCACCGGCGACG

GAATCGGAGCAGGGCGTGTTGCCCATGGAGCAGAGGCCGCTGGACGAGGTGGACGCCGTG

CAGGCGCTTATGTCCGTTGGCCGAGGAACCAGGGCTCAGGCTGAGGCGATGCCGCCCTTC

GATCAGTCGAGCGACTGCGGTCCGGGCGCCGCCGACAGCCACTCGGACGCTGTGGACATG

TCTGAGGCTTCCTACCCCCCCGTGCCTTCGCTGGAGCGGCTGAAGCCGTTCGAGGGAggg

gatgatgacgacgacggccTTGACAAGGACCTGTGGCTGGTCGTCGAGAAGGGCAGTTTA

TGTGCGTCTGCAGACTGCGTCTACAGGGATGCGGCTGCGCGAATCGCATCTCGGAGGCAC

TACCACTCGAaatgcgcacacacgcacaacggTGGAGTGAAGAAAGGAATggtcttccac

caccaccaacttgaAAAGGTTCAAAAGCACCAGCGGTCGCACAAGCGAGAAGCCACAAAG

AGCGCTCCCTACAAGCGCACGACTCCGGAAGACCACCAGGCAATGGAGGGGGCAGGCGTC

GAGGGATACCGTCCAGAGGACTGGGGCCAGGAGGAGACGCGGAAACTAGATGAGCTCGTG

AACCAACTCGTCGCCCCCGTTACGCATGCGTGGCCGAGCATCGCCGGCTTCTTCCCAACG

AAGTCGGGCATACAGTGCCTTCTGCATTGGCGCTTCACGCTGAACGGAAATGGCATTATT

CGGGGAAACGGGACGTGGGGCGCCGAAGAAGACGCTCGACTGCGCAAGCTTGCGCCTGTA

TTCTCGTCTCCTGCAACCGGCCCCCGCTGGGCCAAGATCGCGGAGGTGATGCCAGGGCGA

ACGGCTAAGCAGTGTCGTGAGCGCTACAACAATCACGTCGACCCGGCGATCAAAAAGGAC

AAAATCTGGACGGCGGAAGAGGATGCGCTGGTCATGCAGCTGCACGCCGAGCACCACAAC

CAGTTCGCAAAAATCGCGAGGCGCATCCCCGGCCGATGCTACGACGACGTCAAGAATAGG

TTCAACCTGCTCGTGAAGCGCCGGCAGCTGACAGGCAAGCCTTCTAACTCGGCATTACCG

AGTACTGCAGCCAGCGACAATGCCAGGTTTTTGCCGGCGCCAGGCGATCCCCGACAGGCA

GCACCGCTCACACCGAGGAGCGGGGTCAAACGATCTACGCCAGAAATgtcgggcggtggt

ggtggtggtggtggtggtggtggagaccGCGTGTGTTAA

>g16121.t1

ATGGCTCCTACGTATGGTGTCCTCGTGGCTGCCTCTCTTGCGTGTGCAGCTTTGCAGGCT

AGAGCGTTTGTGGCCCCAAATAGCTACCGCCCATCCTCGGTTGGACGGTCCTCGGCGACA

GCTGCGCCACACCTGACTTCGTCGACGAGGAGACAGCAGTTCCGCGTTCCATCAGGTGCG

ACATGTGTTAGGGCTGTCGCCGAGGAAAACGCTGTGGCAGAAGCGTTCGAGCAGACGCAG

CGTGAAGACCTTCGGAACCTCGCAATCGTGGCTCATGTTGATCACGGCAAAACCACCTTG

GTGGATGCCATGCTCCAGCAAAGCTCAGTCTTCCGCGAGAACGAGCAGATGGGGATCAGA

GTGATGGACAGCAACGACCAGGAACGCGAGCGTGGGATCACCATCTTGGCGAAGAACTGC

GCAATTAGGTATCAGGGCACTAAGTTCAACCTGGTCGACACACCTGGGCACGCGGACTTC

GGGGGAGAAGTGGAACGCATCCTCAACATGGTGGACGGCATTTTGCTCGTGGTGGACTCG

GTAGAGGGCCCCAAGCCTCAAACTAGGTTTGTGTTGAAGAAGGCCTTGGAGCTCGGCTTG

CAGGCCGTTGTGGTCGTCAACAAGATCGACCGCCCCGCGGCTCGCCCGGAGTACGTGGTG

GATAAAACGTTCGACTTGTTCTGCGATCTGCAGGCCAACGACGAGCAGTCTGACTTCCAG

ATCGTCTACACGTCAGCAATCCAAGGGGTCGCTGGAGACGAGCCAGAGGCGATGCAGGAA

AACATGGAGCCTTTGTTCAAGGCCATCATGGGGATGCCGAAGCCCGTAGTAAAGGAAACG

GCCCCTCTGCAGGTGCTCGTCGCCAACATCGACTACGACGACTTCAAGGGGAAGTTGGGT

GTTGGGCGTATCCACTCAGGTTCTTTGCGAAAGGGGCAGACGGTTGCTCTCGCGCGCCCG

GATGGCCCCGTGAAAACGGGCAAGATTTCGGAACTTTTCGTCTTCGACAACCTCGGGCGC

ACGGGCGTGGACGAGGCGCACGCAGGCGACATCGTCATGGTGGCTGGGCTTTCTGAcatc

ggcatcggggacacaaTCGTGGACCCCGCTAACCCCCAACCGATGGTCCCGATTGCCGTG

GAAGAACCAACAGTTCGGATGACCTTCGGGGTTAACAAGTCACCTTTGGCTGGTCGGGAG

GGCAAGTTCCTCACGACGCGGATGATCCGGGACCGCCTCATGAAGGAGTTGGACCGCAAC

GTGGCTCTTCGTGTCGACGAGACGGCCTCGTCAGACGTCTACGAGGTCAGCGGACGGGGA

CAGCTACACCTTACCGTCTTGATCGAGAACATGCGACGAGAGGGCTTCGAGTTGCTGGTT

GGTCCTCCGACAGTCATCACCAAGACGGTGGATGGCAAGACGCACGAGCCGTTTGAAAAC

GTGGAGGTGCAAGTGCCCGACGAGTACACGGGGGCAGTGGTGGATTTACTCTCTCGCCGA

AAGGGTGAGATGCTCAACATGTCGCCGGTTGACGGGGGAGACTCGCGCATGACCAACATC

GAATACCTCGTCCCTACTCGGGGGATGATCGGTCTCCGTAACAGCATGCTGACGGCAACC

CGAGGCACCGCGGTGAtggacaccatcttcgattcGTACAAGCCCTACGCAGGCGACATC

GAAGCGCGCGACAAGGGGTCTTTGGTCGCGCACGAAAACGGGGTGGCTACCCCGTTTGGC

ATCGTTGGAGCCCAGGGCCGCGGCGTCATGATGGTATCGCCTAAAGACGAAATCTACCGC

GACATGATCATCGGCATGCACCAGCGCCCCGGCGATCTCAGGGTCAACATTTGCAAGGCG

AAAGCTTTGAACAACATCCGCTCGGCCACGAAGAGTATCAGCGAAGGCATCCAGGCGTCG

ATGGAGGTAAACCTCGACATGGCGGTGGAATACATTCAGGCGGACGAGCTTGTCGAGGTC

ACCCCATCAAAAATCCGCATGACGAAGAATGCTCAAATGgccgggaaaaaataa

>g16391.t1

ATGTTTGGGTCGTGTTGCTTGTCCAGAACTCTGTGGGGGGTCGGCTTTGGCAACTGCGCA

ACGTGCCGGTCAAACGGGGACCCTACCTGGACATGTCCCGTCGCCGCGAGCACTTATCTC

GATATTGGCGGTCCTGATGCTCAATTCCTCGAgttggacggggggggcgGGACGAGATGC

GGCCACCTGGACGAAACCATTTTCCAAGATGAGCTGATGACCGGATTCGTCGACAACGTA

ATGTTCCTGTCGAAGATGACCGCGGCCGTTCTCGACGACCTGGATTACACCGTCGACCCA

AGCGCGGTGGATGCATACACTCTGCCTTCCGAGCGCGGCGCTTCGATCATCGCGGGGTCG

GGGCCTTCGGTTATGATGAACGACACCGCCGTGGAGGCGATGATCTACGCTATGGACGAG

AACGACAATGTTGTCGATGAAATTGAGGGTGTTATGATGGTATTTTAA

>g16465.t1

ATGAAGTCCGCTGTGATGGCTGTAGCTTGCGCCGCTGGCGCCCAGGCCTTCGTCGCCCCC

AGcgCCTTCAACGGTGCCGCCCTGACCACCTCGGCCAAGGCCTCTTCCGCCATGAAGATG

TCCTTCGAGTCCGAGATCGGCGCCCAGGCCCCCCTCGGCTTCTGGGACCCGCTCGGCCTC

CTGGCCGACGCCGACCAGGAGCGCTTCGAGCGCCTCCGCTACGTGGAGGTGAAGCACGGC

CGCATTGCGATGCTCGCCATCGCCGGCCACTTGACCCAGCAGAACACCCGCCTGCCCGGC

ATGCTCTCCAACTCGGCCAACCTCTCGTTCGCTGACATGCCCAACGGTGTGGCGGCTCTG

TCCAAGATCCCCCCGGCGGGCCTCGCCCAGATCTTCGCGTTCGTGGGCTTCCTCGAGCTG

GCGGTGATGAAGAACGTGGAGGGCTCCTTCCCCGGAGACTTCACCAACGGTGGCAACCCC

TTCGCGTCTTCGTGGGACAACATGTCCGAGGAGACCCAGGCCTCCAAGCGCGCGATCGAG

CTCAACAACGGCCGCGCCGCGCAGATGGGCATCCTCGCCCTCATGgtacacgaggagctc

aacaaCCAGCCCTACATCATCAACAACCTCGTCGGCGCCTCGTACACCTTCAACTGA

>g16481.t1

ATGTTCCGCTGCTCGTCTTCGACGCTCCTGGCGCTGGGACTGTTCTCTCTCGACAGCGCG

ATGGCCGCGATCGACGTGGGAGGGAGCCACGCTTGCACCACCGTCGGCAAATCTCTCAAG

TGCTGGGGTCAAAACAAATGGGGGCAGCTCGGGAGGGGGGACCAAAATGTCACCGGCGAT

GACCCCGAAGAGATGGGAGACAGGCTCATCACGGTCAACCTCGGCACGGGAGAGACCGCA

GTGGGCATGGCTCTCGGAGAAGAGCACAGCTGTGTCATGCTCGACTCCGGCGGCACGAAG

TGCTTCGGGCAAAACGACGACGGACAGCTCGGCCTCGGGGACACGGAGCCTCGTGGCAGA

TTGCCAAGCGAGCTCGGCGATGCCCTGCCCGAGGTAGACTTCGGCACTGGCCTCTCCGCG

ACGGCGATGACGACCGGGTGCTCCCACACGTGCGGCCTTCTTTCGGACGGCTCCGTGAAG

TGCTTCGGGTACAACAACTACGGGCAGTTGGGCCAGGGCTCGACCGACAATGTCGGCGAC

GAACCCGGTCACATGGGCGACGACCTCCCGGCCGTCCCCCTTGATGGCGAGAGCGCCGTC

GCTATCGCTGCGGGGTGCGACTTCACTTGCGCAATCTTGGAAGGCGGTGCCGTCAAGTGC

TGGGGCCGCAACACCTGGGGCCAGCTCGGCACCGGCGACCGTGCTGATCGCCTGGACGGG

GACATCGCGGGGCTCGTTACCGTCAACCTTGACGGGTCTTCCGCGAAGTCCATCGCCGCC

GGGGAGACCCACGTCTGCGTCCTCAACCAGGACACCTCCCTCAAGTGCTGGGGGCGCAAC

AACAAAGGCCAGCTCGGTCTCGGTGACACCATTGACCGTGGAGATGACCCTTTGCTGCTT

GGCGCAAACCTGACTGCCGTCATTTTGGGCGACACAGACATCCCTACCGCGATCGACCTC

GGCATCGAATACACCTGCGTCCTGCTCCAGGACGGCGCCGTCAAGTGCTTCGGCGAAAAC

GCAGACGGCCAGCTCGGCATCGGGTCGCGGACTGACATCGGCGGGGACCCCATGCAGATG

GGCGACAACCTCGTAGCCGTCGACCTCGGCGGCTCTGCCATGGACTTGGCTGTCGGTGAT

GCGTCGGCGTGCGCTGTCCTGTCCGATGACTCCATCAAGTGCTGGGGCCGAGGAAACAAT

GGACAGCTCGGGCAGGGTGACGATGAGAACATCGGCGACGTGGCCGGTGTCCTGGCGGAG

TTGCCCCCGGTCGACGTCGGCACCGACGCCTccatcacccccccgccccttcctcccCGC

GATGTTTCGTTGACATCGAGTTCTCCGTCGGCCGCCCCCACTACCCCGATAACCATTGCC

CAGTCGACAGCCCCGACGATCGACCCTACCGCAACCTTGGCCCCTGGCGCCACCAATGCG

CCGACGATGTTCGGCGACACTGCCTCCCCTTCCGCCGAATTGGAGATAAGGAGCAACAGT

GCCTCGGGAATCCACTTCTCGGCCGGCCTCCTGGCCACGGTCGTTCTTGGTGCCGCTGGC

TTCATCGGCAACTTTTTGCCGTGA

>g16498.t1

ATGAGGTCTGCCGTGGTACTACTGGGCTTCCTCCTCGCCTCGGCGCAGGCGTTCCACATC

CCCGTGCAGACCCGCACCGGCCTCTCGGGCAGCTCCAAGCCCTCCTTCTTCGGGCAGGCC

GTAGCACAGCGCGCGCCGGCAGCCCGGGCCACCCTCGTTGCCTCGCTCGAGGACATCGAG

AGGAAGGTCATCGAGGCCGAGAAGGCCAAGACGGCGGCCGCGCAGAAGGCTAGTGCCCCC

GAGCCCAAGGCGAAGGCTGCGCCGAAGGCTAAGGCGGCCCCtgccccggccccggccccc

AAGCAGACGACGGCTCCGGTGAAGACCGTGGCTCCCGCGCCCGCTCCGGCCGTCgTTGCG

CCCGCTCCGACCCCGAAGGCGGTTGCCCCGGCGGCGAAGTCCGGTGGTGGTATTGGGACT

GACGAGGTGGCGGAGGGAGTGGCGCTAGGAGCGCTGCCGTGGATCGTTGCCCCcgtcgct

gccgtcgccgccctGCGCCCGTTGCTGGCGAAGGCGGCCGAAGTCCGTGACGAGGAGCGC

AAGCTGAAGGCGGCCGAGGACGCCAAGAAGGCCATCGCCGCGGCTCGCGCGGAGAGGATG

GAAGGCCTCACCGACAGCGACCAGGGTGCCGTCAAGGccctcgccgccgtcgccgccgtc

tgCTTCGGAGGCCTCCTGGTGCAGCCCCTCTTCGCGCCCGAGGACCTCGCCAAGACCGCC

GCGCCCCCAGCCATCGTCCAGAaggcccccgcccctgcccctgcccccgccccggccccg

gctccggctccggccCCTAAGAAGGAAGAGGCTGCCCCGGCGCCGGCcccggctccggct

ccggctccggctccggccCCCGCCCCGGCACCAGCACCGGCCCCAGCACCGGCCCCGGCg

ccggctccggctccggcaCCGGCCCCCGCACCGGCTCCCGCACCGGCACccgcaccggca

ccggcacccgcACCGGCTCCGGCtccagcccccgcccccgcccctaagCCTGCCGCTAAG

CAGAGCAAGAAGGAGGAGGCTGCTGCTCCCGCGTACGGCGGGCCGGCTAGCGGGCGAGTG

CTAGAGGCGGAGACGTTGAGCCCGGACGTTCTGAACATGCTCAAGTCTTCCAAGACGGCG

AAGAAGTGA

>g16600.t1

ATGGCCGCACCTACGGGCGATGACTTCTCGGGCAGGGAGTGGAAGGACGGCGACGCTCAC

GAGATTCTGCCCAACGTGTTCCTCGGTTCCATGGAAGCGGCAAAGGACAAAGACAGCCTA

AAGTCCCACGGCGTGACTCATATCCTGACTGTCAACGGCAAGGACCCCGCGTTCAAAGAC

GACTTCGAGTACAAGGTGATCAACTTTGGGGGCGACAACGGCGACCTCACGCCCCATTTG

GAAGACAGCGTGGCGTTCGTTAACTCTGGCGCGAAGAGCGGCGGGGTGCTGATCCACTGC

ACTCACGGGACGGGCCGAAGCGCAGCCGTGGCGATCGCCACCGCAATGAAAGCGAACGGG

GATGGAACTCCAGGAGGCGGTGTTGGGGATTTCGCAGAAGCGTTCAGGCTGGTGAAGGCG

CGCCGCCCCGGCACGGACCCTCCGATGCCTTTTCAGTTGGAGTTAGACGCGTGGGAGAAA

TCCATGTCGACTCAAAAATGA

>g16685.t1

ATGGCTTTTCAAcaacgacgaagacgacgaacaacaacaacaacgcccttCACGAGGGCC

TTGAGCGCCGCTTGCTTGGCGGGAGCGACGGCGTTCTCGGCCGCCTTCATCGCTCCGCCG

GGCGCCGCAGGAGGCGGGCTCCACCACCGAGTCAGCCGAGTCAACCACGGCGTCGACGCC

GGACGAGTTCGCCGCTTCTGCAGCGGCCCGAGCTTGAGGGCGAACCGCCTCGCCATGGTG

TCGGCGCTCGTGGAGGCCCCTCCTTCCTTGCCGCCACCCATGGGGGCGGCGACGACGAGc

gggcgacggcagcggcgacgaACACCGCCACTCACCGTCGAGGAGGAGCGAGCGCTGCTG

ACGAAAATCCACGCGGCGCGCTTGCTGCGAGCGATGCACAAGAACCTGGCCgccgccgga

ggggggggggggggcttggtcaACTTGGAAGAGTGGGCGCGGGAGGCCGGCTTGGGGGTG

GAGGAGCTGACGAGCGCGCTGCAGGGGGGCTTGGACGCGAAGCGAGCGCTGGTGGAGCGC

AACATGCCGATGGTGATGCAGCTCATCgagcagcagtaccggtggCGACTGCGCGGGGGG

CAGGTGTCGACGGCGGACCTCCTCCAGGAGGGCGCCTACGCCCTCGGGCTCGCCGCCGAC

CGCTTCGACCCCGCGATGCCGAATCGCTTCCTCACCTACGCACTGTTCCTCGTGCGAGAC

AAGCTGGACACCGCCCTGGCCGGCGGCAACACGGCCATCAGCGTGCCCGTGGCGGCCCTC

AAGGAGCTCCACCGATCTCGGCGAGAGCTGACGGCCGAGCTAGGCCGCAGCCCCTCGGAG

GCAGAGGTGGCGCACTTTTTCGCAAACGGAGTGGTGGCTGGCTCTCCTCCGgcaatggca

gcggcagcgagaaGTGGTGGCGCGAGACACGCGCCGACCGCGGAGGGACGCAGCACGGTG

GGCGAGCCGGCGGCGGCTGGGGGGGCTGTACGCGGGAAGATTGAGGTGCAGCCTCGGATC

CGCGAGCGGCGGTTGAACCTGCTGTCGGCGGTGAACAAGGTGAAGAGCATCGACACGCTC

ATCAGGGACAGCGAGGGCAACACCATCTCCCTCGTGGACACCCTCGTGGGCCACCACGGG

GACCCGCTACGCATGCCCGGGAACGGGGACATGTCGGAACTTCTCCCGAAGGTGCTGACG

CGGGAGCAGGCGAACCTGGTGCGAATGGCGTGCGGGCTGGAGGACGGCCCACCCCTGTCC

ATGAAGGAGTGCTCGCAGAGGCTGTCTCTGAGCGTGGGGCGAACGAAGACCCTGTTCGAG

AACAGTCTCAAGAAGTTGAGAATAGCGGCCACCGCCGACAACCCGGCGTTGGTTATGTAC

AAGTGA

>g16694.t1

ATGCGGTGGGTTTTGCTACGCATGTCAGCAGACATTTTCAACATCAATGTGCCAGCAGCA

AGCGCGTGCCTGTCGGCCGACAAGATGACCGTGCCAGCATTCCTGGCTCACACCTCTCTC

CCGAAAGTCCCCAGCCGTGTTGCTCCTGCGTTTTGGCACCATCACGTTCCCAGCGCTACC

GCTGGACATACATATACGACCAGCCTTCCCACAAGCAGAAGAAGAGTCTGGGCGCTGAGG

CTAGGCGACGATGTTGACGCCGCGGCGGAAGAAGATGCTGCCGTGGTCGGGGTGAGGTGG

GAGGAGCCTACCCTGGAGGAGACGTTCGATGCAATCGACGACGTGATGATGGACAGGTTA

CCTGGCGAGGGGCGAGACAAGGTCAAGGTGTTCGGAGCCTGGCTTGACGCGGACGTGGCC

GGAAGAAAAGCGGGGCAAGGCAACCGAGCAGCGGGGCTGGTCGCGCGTGCTGAGCGGGGA

GACCAGCTGACGAACCAGGAGAAAGCTGAGGTCATCTACGAGGACGCTATCTCCGTCATG

CGGGGGGGCGACTACGACGCGTCGTTCACCCTCCTCGAGAAGGCCGTTTCTCTCGCGGGA

GCAGACTCGCGGAGAGGCGGCGAGTTCAAGCTGTGGGCAGTGCAGGCATTGCAGGGCGTG

GGGAGGAACAAGCAGGCGGTGACGCTGCTCGAAAGCCTCAAGGCTCACCGAGACTTGGAC

GTCCGAAAGGTCAGTCAAGAGCTGCTGTACATCGCCAAAGCCCCTCAGCTGAAGCTCGGC

GAAGATGACTTCGTCCAATTTCCTGACGTGTCGCACCTGACGGAAACTTTTGACAAGACC

AAGTGGAAGGAGATAGGGAAGCCTAAAGTGGTTCCTTGGAAAACAGAGAAGAAAGACGAT

CTGGGTTACTACGTGGGGAGTGACACCGGCCAGGCGCCTGGTAACGACCCCGTTATGCTC

GTGGCTTGCGTGGGCGCTATTTTGGCGCTGTCGTTTGTCTTCTTGGGGTGA

>g16727.t1

ATGATGCGCTCTACATTTATCTGCGCGTTAATCGCAGCGGCCAATGCGCCGGAAGGAGTA

GCCTTCGTGCCCTCGGGCGTGTTTGGTGTTACCGCGCCGAGCCAGGCTCGTCTTTCCTCC

AGCAGTTCGCCACGAACTCATCGGACAACCACAACGCGCATGGCCGCGGGCCCCGAGAGC

AGCGAGGGCCAGACTCGGGGGGAGCTGGTGCAGACGCTGTGGAAGACGGCATTGGCGGCA

GGGGCTACGCAGACCTTGCTCGCGTCTCCTAGCTTTGCGGAGGACACCACGGTTACAGCA

AGCGGGCTCAAGATCAAGAAGCTGGTTAACGGCAAGGGCCCTCTGGTGGAGGTCGGCGAC

CTGGTGGGCGTTCGCTTCAAAGGGAACTACGGCACCTACGAGTTCGACAACATCTTCGCT

ACCGCCGAGCCTTACTACATGCGCGCGGGAGTGGGCACCCTCGTAGCGGGTGTGgaggag

gcgttgttgttgatgaaggTGGGCGACAAGTGGCTGCTCACGTGCCCCCCCGCGCTCGCG

TTCGGAAACAAAGGCCGCCCCCCTAGCCCCGGCAGGCCGAGGATCCCCCCCGGGGCGGAG

GTGGAGTACACGTTGGAGCTGGTGGCGTTGCCCGGGAAGGAGGAGGACCTCGTCCTCACG

CGCGACGACGCCTTCACCGGCGACGCCTTCGATTCCGCCTAG

>g16729.t1

ATGCCGCCACCGATACCGCACCGAGGAAGCACAGCCATGTCGTCCGTCCCGAGACGAAAC

AGAGAGAGAGAGTTTGCCCTGGTGCCCCAAGACCCTGTGGGCTCACTCGTCCTCAAGGAT

GGCACCACGCATTCAGGGTACAGCTTCGGAGCGCCGACCTCGATGGCCGGTGAGGTAGTG

TTCAACACCGGCATGGTTGGGTACCCGGAGGCCCTGACAGACCCGAGCTACCGAGGCCAG

ATTCTGGTTCTGACCTACCCCCTGGTGGGGAACTATGGTGTGCCGAACGAGGCGGACATT

GATGACCTGGGCCTGCCGGCGCACttcgagAGCTCGTCGATCCACATCAAGGGCTTGATC

GTGTCCTCCTACTCGGCGGACCACTCCCACTGGAACAGCTTCCAGAGCCTCGGAGACTGG

CTCAAGAAGCACAACGTGCCGGCGCTGTACGGCCTCGACACCCGCTCCCTGACCAAGCGC

ATCAGGGAGCACGGCGCTGTCTTGGGGAAGATAGAGCTTCCGGGACAGGAAGTGGCGTTC

GACGACCCAAACTTGCGCAACCTGGTAGAGGAGGTTTGCGTGAAAAAGGTCCGCGTTTTT

AACCCGGGGATGGAGACGAACATCATCGCCTTCGACTGCGGCATGAAGTCGAACATCATC

AGATACTTCGTGCACGAGCAGAAAGTGACCTTCACGGTCGTCCCGTTCAACTACAACCTC

GAGGAGAACCCGGAAAAGGTGGTCTACCACGGGGTGTTCGTCAGCAACGGCCCCGGCGAC

CCTGTCATGTGCACCCCCACGATCAACTCGCTGAAGTGGATCATGAAACAGGCCGAGGCG

GGCAACAAACCGATACCCGTTTTCGGGATTTGTCTCGGGAACCAGCTTCTGGCGCTGGCG

GCGGGAGCGAAGACCTTCAAGATGAAGTTCGGCAACCGAGGCATGAACCAGCCGTGCATC

GACATGCGCACGGCCAAGTGCTACATCACGCCGCAGAACCACGGGTTCGCTGTGGACCCC

GCCACGCTTCCGGATGGGTGGAAGACCCTGTTCAGTAACGCCAACGACAACACTAACGAG

GGCGTTATCCACCAAACCAAGCCTTGGTCCTCCGTGCAGTTCCACCCCGAGGCTGCTGGC

GGACCCATGGACACGGCCTTCCTCTTCGAAAGCTTCATCCACGCCGTGAAGGGATTTCCG

CCCAAGCTAACCCTGCTGGAGCCCCACATCTTCGAGATGAGGTCGATCAAGAAGGTGCTG

CTTCTGGGATCAGGCGGGCTGAGCATCGGCCAGGCCGGAGAGTTCGACTACAGCGGGTCT

CAGGCAATCAAGGCTTTGAAGGAGGAAGGCATCGAGGTGGTGCTGGTCAACCCCAACATC

GCCACGGTGCAGACGTCCAAAAACCTGGGCGGAGCTTCTCCGGACAGGAcgtactttcta

cccgtcacgCCCGAGATCGTGGAGAGCGTTATCATCAAGGAGCGCCCGGATGGCGTCGTG

GTGTCGATGGGAGGGCAAACGGCTCTCAACTGCGGTGTTGCCCTGCACAACGCTGGGACC

TTCCAGAAGCACAACGTTCGCGTCCTCGGGACTCAGATCCCGGTGATCATGGCGACGGAG

GACAGGGACGTGTTCGCGCAGAAGCTGAGGGAGATCGATGAGAAGCTGGCGATGAGCATC

TGCGCCAATTCGGTGGAGGAGGGGCTTGCTGCGGCCGAGAAGATCGGCTACCCGGTGCTG

ATTCGAGCGGCGTACGCGCTGGGGGGACTCGGGTCGGGGTTCGCCGAAAACGAGGAGGAG

TTCAGGGCTCAGGCGGAAAAGGGCCTCGCCTACTCCGACCAGCTCATCGTTGACCAGGAC

CTGCGAGGATGGAAGGAGGTGGAGTACGAGGTGGTGAGGGACAACAGGGACAACTGCATC

ACCGTCTGCAACATGGAGAACTTCGACCCTCTCGGCGTCCACACCGGTGACTCCATCGTC

ATCGCCCCCAGCCAGACGCTCTCCAACAGGGAGTACTTCCAGCTTCGCAGGACCGCCCTC

AAGGTGGTTCGACACCTCGGCGTGGTCGGAGAGTGTAACATCCAGTACGCGCTCAACCCG

GAGAGCGAGGAGTACTGCATCATCGAGGTTAATGCGCGGCTGAGCCGATCGTCGGCCCTG

GCCTCCAAGGCTACGGGCTACCCGCTGGCCTACGTGGCCGCTAAGCTTGCCCTCGGAATC

GACCTCGTCTCCATCCGCAACAGCGTCACCAAGTGCACAACTGCGTGCTTTGAGCCGTCC

CTCGACTACTGCGTGGTCAAGATGCCTCGCTGGGACCTGAAGAAGTTCTCCAAGGTGTCG

ACGAAGCTGGGCAGCTCCATGACTAGCGTCGGGGAGGTCATGTCCATTGGAAGAACCTTC

GAGGAGGCTATCCAGAAGGCTGTCAGGATGGTCAACCCTGACTTGGACGGTCTGGAAGGC

AAGTGGGACAAGGATTCCAACCTCTCGAAGGACGATCAACTCAAGATACCGACTGACTCG

AGGCTTTACGCCGTGCAGGCTGCGCTGGAGGACGGCCTCAGTGTGGACCATGTCCACAAC

CTGTCTCGAATCGACCGCTGGTTCTTGTCCAAGCTCAAGAACATTGCGGTCATGAAGAAG

GCGGCGAAGCTGACGGGTGGCCTTGAGAGCCTGGGGCGTCCCGGACTGTTGTCGCTCAAG

ACCTCCGGTTTCAGCGACCGCCAGATTGCTCGCTACACGAGCACCTCTGAGAACCAGGTA

CGCCGTCGAAGGCAAGCGGCTGGGGTGAGGCCGTTCGTGAAGCAGGTGGACACTTTAGCC

GCCGAGTTCCCCGCGAGCACGAACTACCTGTACATGACGTACGCGGGGAACGAGGACGAC

ATCGAGAGGGAGAACATGGGCATCATGGTCCTGGGATGCGGGGCGTACTGCATCGGCTCT

AGCGTGGAGTTCGACTGGTGCGCGGTGAGCTGTGTGAGGCAGCTGCGCTCGCTGGGGTAC

AAGGCCATCGTCGTCAACTACAACCCGGAGACgGTGAGCACGGACTACGACGAGTCTGAC

CGCCTGTACTTCGAAGAGCTGTCCTTCGAGCGCGTATTGGACATCTACGAGTGGGAGATG

GCGGGAGGAGTGGTGGTCAGCGTGGGTGGCCAGATCCCCAACAACCTCGCCATGCCATTG

CACCAGGCCGGAGTGAACATCCTCGGCACTCGCCCCGAGGACATTGACCGCGCGGAGGAC

CGCGACAAGTTTAGCGCCATGCTTGACTCCATCGGCGTCGATCAGCCGAAGTGGGCTCTC

CTCAGCACGCCCAAGGAGGCCTTGGCTTTCGCCGACCGAGTGGGTTTCCCCGTCCTGGTC

AGGCCTTCTTTTGTCTTGTCGGGGGCCGCCATGGCCGTGGCCAGCAACCACACCGAGCTC

GAGaaATTCTTGTTTGCCGCTGGCGAGCTGGCTCAAGACAAGCCGGTCGTCGTGAGCAAG

TTCATCCTGAACGCGAAGGAGATCGAGTTCGATGGCGTGGCTCACGACGGAAAGATCCTC

AACTATGCCATCAGCGAGCACGTCGAGAACGCCGGCGTTCACTCGGGAGACGCGACGCTG

TTGCTGCCGGCGCAGAAGCTGTGGGTGGAGACCATACGCCGGGTGAAGCGGGTGGCCTCG

GCTATCTGCAAGGCGCTCAACATCTCGGGCCCCTTCAACATCCAGCTCATGGCCAAGGAG

CAGGACATCAAAGGTGGGAGGGTGATCGAGTGCAACCTGCGGGCATCGCGCACGTTCCCG

TTCATCTCGAAGACCCTGGACTGCAACTTCATCACGCTGGCGACTCGGGTCATGCTCGGC

GTCAAGACCGCCCCGTACAACATCAGCCTGCTGGACATCGACTACGTCGCCGTTAAGGCC

CCGATGTTCAGCTTCACGCGCCTCAGGGGCGCCGACCCCACCCTCGGAGTCGAGATGTCC

TCCACGGGGGAGGTGGCCGCGTTCGGCAGGGATTCTTGCGAGGCGTTCATTCAGGCCCTC

GTTGCCACCGGCTTCAAGCTTCCCCAAACCAAGAACGAGATTCTGATCTCTTTCGCTGTG

GACTCGATGCGGGCGGAGTTCCTGCCTTGGGCAAAGGAATTCATCAAGATGGGGTACACC

TTTGTTGGGACCCCGGGGACCGCGGAGTACTACTGCAAACACGATGTTCCCACCAAGACG

CTAGCTAAACCCCAGGACTGCAGAGGGTCGGGCAACACGACGGCGCTTGACGGCACGCCC

CTTCCCAACGTGGTGCAGTACATCAAGGACGGAAAGGTCGACCTCGTCATCAACATCCCA

GAGGGCACGAAGAAGACGGAGGAGATCAGTGCCGGCTACCTTATCCGTCGCTCTTCGGTG

GATTTCGGCGTTAGCCTCATCACCAACGTCAAGTGCGCGTCCCTGCTGGCGGACAGTCTC

GTCAGAAAAGCCGCCAAACCGCCCGGGCCGTACATCCCCGTATGCATCGAGGAGTACTAC

GGAGCAAAGACTAGCGCGGCGTGA

>g16757.t1

ATGACCGAGGCTGTGAGAGGGTTGTCAAGCGATGGCAGCAAACGCCCCCCTCCCTTGCCG

GAGAGTGTGCGGGAGGTCCTAGAAGGCCTCGACACGGGCGACCTGATCCTGTTCGACCGC

CCGTGCTCCAAGATGGGGAGCGTCATTGGGGCGATAATTTGCTCAACTGCCAAGGTTGTT

GGCGGCTCCCCTTTCGACCACATCGGCGTCGTGGTGCGGGACGAGGGCGGAAACAATGTT

CTTGTGGAAGCTTCCTTCAGCGGCGTCGCCGTGCGCCCTCTTTGCGAGCGCGTGAGGAGG

AGCTCGGCGTCTCAGATCTGCGTTCGCCGGCTTCAGACGAACCGCACGGAGGAAATGCGG

GCGGAGGCTTGCCGATTCGTCTCCGAAGTGGCCGACGTTCCCTACACTAACACCGCGCCG

GGGCTGCTGCAGATGGCGTTTTCGTCTTTTCGGTACCATCCCGCGAAGGAGCGGAGGCGA

AGAATGCACGCGGAGACCATCGGGCTGTCGGCGTCTATTTCGTCCCTGGAAAAGGAGCTA

GCCATTTGTCAGTCGACAACTGAGAGGACCCAGGTCCTGCACGACCGACTTCGTCGGGCT

CTCACACAGCGGCGCAAGGCTCAGGTCTTCCTGGATGACGCTGCGGATCAAGAACGGCCg

gccaccccaccaccatcaccaccacaaccaccaccaccgccaacaacagcgGCTCCTCTT

GAGAATGGCAGCCTACCTCCATCTTCCCCTGTTCCCTCCTCCCCGTCGCCACCGGGGTCG

CCACCTCCGTCATACCCGGCGGGCTTAACGACCTCTTACGAAGGCGGGCTGTTTTGCTCC

GAGCTTGTGGCCGCCCTTTACCAGAGGCTGGGGCTGTTCGACGCGCCGTATCCGGCGAGG

CACGACTACGTCCCCGCTGATTTCACGCCTCCCATTGGCGCCAAAAGGgatggcggagga

ggaggaggaggaggaggaggaggaggaggcggcgacgCGACCAAGgACACGATCGATGCC

GAGTACCTTACTACCGACGGCGAAACGCGACCAAGCGATACCTTCGCTACAGCCTCCCAG

CCGCTGCTCCTGGACGATGAGATACGGGTAGCGCTGCTCAGAGGCGCAACGCTAGAACCG

GGGCTGTGGATTCGAGGCGGACCTGGGTCAGCTCCGTgtccaccgccgcggccgccgccg

aagAGCCCCGTCGACACGGAAGACGGAGAGCGGGGagtggggggctgggggggtagTCCA

GGCTCATCTCCTCGGCCAGCCCCTTCGAGTGGGGCGGCTGGGACATCCCAGCCTTCCCAG

CGCGTGGTGCTAGGGTACGCGAAGGAGCTAGGGGAAGAGCGGGGGGTACAAcctggcggc

cgcggcggcagaTGGCTGCCCCGGCCACAGAGGTTTCGGCTTTCACCGTTGAACCCCACA

GCATATCTCCCTCCCCCCGCGAGGTTAAACTCCAAGCATTATATCCCTTGTTTCGCGAGT

GTTGCGCCCCTTGGCGTACGCCCTTCCCTATCACAGTTGGCGTGGTTTCTGCATGATTTC

TCCCAgagcgccgctgccaccgcgaCCGCCACCCACGCCACATTTATGAAGGGAGTTGTT

GGGCTAACGGATGATCTGACTATTCAGCCACGGAAGGTTTGGCCACCAGCGAGGGATGAC

AACGAAGCCCGTCCAGAGCGTAAGCACtggctttcagcagcgacaacgacaacaactact

GCCGTGGTGGCGGTCGCAGCTGAGAGGCTGCGCACGACACCGGTGGATGGTGGCCTCCCC

CTCTCGCCCTGCACCGTGCGCCAAGAGTGTGCCGAATATGGCGTCTTGGGGTGCACCCCT

ACTTCTGCggctatcgccgccgccgcagccaccgccgccgccgccgccgccgcgactgCC

TTATCGGCTGTCAACAAATTTAATCCCGCCGGACGAGGTGGTCGCCCAGGCGCCGTCGCG

ATGAagcgaggagggggtggagcAGCACTAGCGTTGCTGACCTTCGGCGCCGCCTGCGGC

CTTGCGTCGGAGCTGGGACCGACCGGCGACGGACGCGCGGGGTTATTTCGACGTGGTGCC

TTTGTTTCTGCCCCCGCGCAGAGGTGA

>g16901.t1

ATGGTGGGAGGAAAGCGTAGCATTCGCTTTGGAGCGGCCGGCCTCGCGCTGCTGGGAAGC

GGAGCTTTCGCGCTGCCTTCCACGAAGGTTGCCTTCTTGCAGCACGGGGTGGTGGAGGGA

GAGGATGCCGTCATGCACCCCCCATCGACGCTCAGCTCTAAGCTGGCCTCGCTGACACCT

CAGCTTCTGTCGTCCGCAATCTCAAGGGTTATGAGCCTGGACGCGCGTGCGGAAGTCGAC

ACAAGCCTTCCAGCGGGCGACATTTTCAACAGGCCCGACGGGAACGTGTTGGTGTTTGTC

GATGGCCTCCGAGCTTCAGGCTCCAAGTCAACGCCGTTTATGACGGAGTGGTTGCAGCAG

CCGACGGCCGCGTATGCGCTGAGGACGCCGTCGGACAACACCTGGACTTCCGGTCTTGAG

AGCACCCTCGGTGGTGGCTTCGACGTCGTCGACGCCGTGGGCAGCAAGGGGGGCGTCGTG

TGCGCGGCCGTCGACCCGGCCCTCGCCAGCGCGGAGGCGTGCGCGAACGCCGACTTCGCG

TTCCCCGCGGACGCCGCCACGGCGGCTCGGTGGGCGGAGGGCGTGAGCATGGGGCCCGGC

GTGATCGTCGACTCCTCGTCCGGGCTTCGGACGCTGGTGTTGTCTGACGTCGCCGCAGGG

CAGAACGCCGTGCTGGACCTGACCGTCGAGGCTGACCTGCGCCTAGCGGAGGAGCTTAGC

TTCCTCTCGGCTCTTCCCGCGGCGCTTGCCGGGCGATTCGCCAACAGGGGAGACTCTTCG

CCTCCCCTCGTAATCGTGTACCTTTCATCCCTCAAGgGTATCAAGAAGGCGTACGGTGAT

AGGTCTGTGAAGGGCTCCTTGGCGTCCAAGGCACTTGACGGCGCTCTGGCCAAGGCTTTC

GGGGCCATGTCCACGGGCGCCGGACGCCGCTTGACCTCGCAGCTTGTCGTCGGCCCGGCG

CTGGCACAAACGCTCGCCATCGGCGGCCGCCGCTTACAGGAggagagcaccagcagcagc

gtcggcagCAGCAACGTGACCCTGATCGAGATCACCCAGTTCCAGCTCAACATGTGGACG

GGCGTGGGCCTGGCCCTCCTCGGCTTTTTGGCCATCTACGCTACCTTCACCATGGACGTG

CAGCCCGACAGCCTCCTGTACGCCAAGTTCATCACCGACACCAGCGGGGGTGGACTGAAG

ACCGACTAA

>g16912.t1

ATGAAAAAAAGTTCCTTCCAAAACACCCAGCATGCTAATGCGGCCCACTCTCCGGTGGGA

ACAGCGGAGTTCGGCGAGCTGATCGTGCAGCTCTCGCATCCTCGACTCACCATCAAGGCG

GTCGGGGTGGAGTACGACAAACGCTTCGCGGATCGGGCGCAGAAGCGAGTAGCAGATGCC

GGGCTCGAACTCAAGattaaGATCATCCACGGCAATGTACTGGATATAGATGCAGATGAG

GCCACCGTAGTGTTCATCTACCTCGTCCCGGGGGGAATGGCGGCAGTGAAGGACACTATG

GTCTCGCTCCTTCGCACGGGAGCTAGGGTTGCAACATATGTTTTCTCGTTGCCAGGACTG

ACTCCTGCGAAGGTTACGAAGCTCAGGGCCACCAGCATATACCTCTACACTGCGTCTTCT

TTGGAACCCGCTCAGCCGGAATCGCCATGCGCTTTAAGAACGGCGGCCTAA

>g17011.t1

ATGGCACCAGCACTGGCCACGATTGCGGCCTTGTTGGCCGCCCTCACCCCCGCTTCCGCC

TTCGTCGGCGTCAACGTTGGAAGCTCGTTCAAGGTGGAAAACGTGGCAGCTGTTGTTCTC

GCTGGAGGCGTTGGCAAGCGGATGGAGGCGGACAGGCCCAAGCAATTTTTGGAGCTCCAG

GGGGAAACGGTGCTGGAGCACAGCCTCAAGCTCTTCCTCTCGCTCAAGGGAGTTTCACAG

CTTGTCCTGGTGCTTGAAGAGCGGTACCGCCCGATGCTGGAGGACCTTCAGGGCCGGGAG

CCAAGGCTTGTCTTTGCCGACCCCGGCACGGAACGGCAGGACTCGGTGTACAACGCCCTG

CAGAAGGTCGACGTGGACGCCTCTTTGGTGTGCATCCACGACGCTGCTCGTCCGTTGGTC

ACCAAGGACTCCGTATACAAGGTGATCGCGGATGCCGATGAGCATGGCGCCGCTGTCCTG

GGAGTGCTCATGAAAGCTACCGTCAAGGAGAGTGAAGACGGGGAGTTTGTGCTACGGACC

ATCGAGCGGTCCCGCCTCTGGGACATACAAACCCCGCAGGTAGTTAAGCCTGATATTCTG

CGAAGGGGATTCGAGGAGGTGAAGAAGAACGGGTGGGAGGTGACGGACGACGTGTCCATC

GTGGAGCAGCTCAACTTGCCGGTGAAGATCACCGAGGGAGAGTACACCAACCTGAAGATC

ACCACACCCGAAGACCTTGTCGTAGCCTCACAAATCTTGGAAGCGCGGCGAATATCGGAC

TTGGAAAGCGGCGCCGCGGACACCGaccaggacgaggaggaggagaacttGTCGGTGACG

CGAGCAAACGAGGTCTGGACGGATAAGCCCCACGCTGTCGGCGTTGATGAGGAGGAGGAC

ATCGAGTCCGTCTCTGCTCTCCGGGCGGACGAGAAGTGGGTGGACAGTCCCGTCGGGAAC

GCCCCCAAGGGGGACGGAGATGTGGACATCCTCCTCGACCTTTCCAAGATGCGCGCCGGC

GAAtag

>g17018.t1

ATGCGGTCAGCGTACGGCAAGCCCAGGCCCGTGTCGAGGGTGATGCGCATGGCCAACTCG

CAGCGCTTGGCGGACCCGGTCGGCCAGATGCttgacaagACGGATGTGTTCATCTTCGAC

TGCGACGGCGTTATCTGGAAGGGAGACAGCCTCATCGAGGGCATCCCGTCGGTCTTGGAC

CGCCTCAGGGCAGCTGGGAAGCGGATATTCTTCGTCACGAACAACTCTACCAAGAGCCGC

AAGGGCTACAAGAAGAAATTCGACTCCCTGGGATTGAACGTTGAGCCCGAGGAGATCTTC

TCGTCGAGCTTCGCCGCTGCAGCCTACCTCGAGCAGACCAATTTCAAGGACACGGGCAAG

AAGGTGTACGTGATGGGTGACGTGGGGATCGAGGAGGAGCTGGACCTCATCGGTGTGCCT

CACTTTGGTGGACCCAAGGATGGAGACAGGGTGGTGGAGCTCAAGCCGGGGTACGCCCTA

CCGCACGACGAGAACGTTGGAGCGGTGGTTGTTGGCTTTGACAGGATGATCAACTACTAC

AAGATCCAGTACGCCCAGCTGTGCATCAACGAGAACCCGGGGTGCGAGTTCATCGCCACC

AACCTCGATGCCGTCACCCACCTCACCGACGCCCAGGAGTGGGCGGGGAACGGGGCGATG

GTGGGCGCAATCAAGGGCTGCACCGGAGTGGAGCCGACGATCGTGGGCAAGCCGAGCCCT

CTGATGATCGACTACATCGTCGACAAGTACAGCGTGGAGAGGTCGCGCATCTGCATGGTG

GGGGACCGCTTGGACACGGACGTCCTGTTCGGCTCCAACAACGGCTTGATGAGCATCCTT

ACCTTGAGCGGTGTGACGACGGAGGCGAAGCTTCTGAGCGACGACAACAAGATCAACCCG

GACTACTACGTTGACAGCATCAACGATTTCTTCCCTTAG

>g17019.t1

ATGCGGTCAGCGTACGGCAAGCCCAGGCCCGTGTCGAGGGTGATGCGCATGGCCAACTCG

CAGCGCTTGGCGGACCCGGTCGGCCAGATGCttgacaagACGGATGTGTTCATCTTCGAC

TGCGACGGCGTTATCTGGAAGGGAGACAGCCTCATCGAGGGCATCCCGTCGGTCTTGGAC

CGCCTCAGGGCAGCTGGGAAGCGGATATTCTTCGTCACGAACAACTCTACCAAGAGCCGC

AAGGGCTACAAGAAGAAATTCGACTCCCTGGGATTGAACGTTGAGCCCGAGGAGATCTTC

TCGTCGAGCTTCGCCGCTGCAGCCTACCTCGAGCAGACCAATTTCAAGGACACGGGCAAG

AAGGTGTACGTGATGGGTGACGTGGGGATCGAGGAGGAGCTGGACCTCATCGGTGTGCCT

CACTTTGGTGGACCCAAGGATGGAGACAGGGTGGTGGAGCTCAAGCCGGGGTACGCCCTA

CCGCACGACGAGAACGTTGGAGCGGTGGTTGTTGGCTTTGACAGGATGATCAACTACTAC

AAGATCCAGTACGCCCAGCTGTGCATCAACGAGAACCCGGGGTGCGAGTTCATCGCCACC

AACCTCGATGCCGTCACCCACCTCACCGACGCCCAGGAGTGGGCGGGGAACGGGGCGATG

GTGGGCGCAATCAAGGGCTGCACCGGAGTGGAGCCGACGATCGTGGGCAAGCCGAGCCCT

CTGATGATCGACTACATCGTCGACAAGTACAGCGTGGAGAGGTCGCGCATCTGCATGGTG

GGGGACCGCTTGGACACGGACGTCCTGTTCGGCTCCAACAACGGCTTGATGAGCATCCTT

ACCTTGAGCGGTGTGACGACGGAGGCGAAGCTTCTGAGCGACGACAACAAGATCAACCCG

GACTACTACGTTGACAGCATCAACGATTTCTTCCCTTAG

>g17039.t1

ATGGTTCTTCGCAAGACGACGGCAACATGCGTCGCCGTCTGCCTCTGGACGCAACAAGCC

CACGGCTTTGGGTTCGGCCTGCGACCCTCTGCCAGTCTCGCGCTGTCACCGTCTTCCTCG

AGGAGCTTCACCGACGCCGTCGGCAGTAACGGAGTTGGtatcggtggtggcggtggtggt

ggcgacggcgcgGGAGCACGCGGAATGACCATGTACTCGTCGTTCAAAAAGAAGGGCGGG

AAGAAgaaggggaaggcggggggaggggggaagaaaacctcgggtggcggtggcaagggc

ggggatggggggccTCCTTCTCCTAACGTGGACACTTCCAGAAGGGATTTCGTGTACCAA

ATGAACCACCTGACCAAGTCGTACGGAAAGGGGGTTAGCATCCGCCCTGTCCTGAAGAAC

GTGAACCTCTCCTTCTACCCTGGGGCTAAGATCGGAGTCTTGGGTGCCAACGGCTCGGGA

AAGTCAACCCTCATGAAGATCATGGCGGGTTTGGACGATGAGTTCGAGGGCGAGTCGCGT

CTAAGCGACTGGGCGAACGTGGGCTACCTTGAACAAGAGCCCAAGCTAGACGACGGGGAC

ACAGTGGAATCCAACATAGAGGCGGCGGTTGTGCCCACTCGCACCCTCCTCAAGGAGTAC

GAGCAGGTTTCCGCTGATCTGTCGGGCGTCGGTGCGGACATGGAGAAACTGTCGAACGAG

ATGGACCGACTCCAGAACGCCATCGAGGCCGTGAACGGGTGGGAGCTGGACCGAGTGCTG

CAGCGAGCGATGGACGCTCTGAGATGCCCGGACGGTGACTCTTTGGTGGCCAACCTGTCA

GgaggagagaggaggcggGTGGCCCTGTGCAAGCTGCTCCTGAGGCGGCCCGACCTCCTC

CTATTGGACGAGCCGACCAATCACCTCGACGCGGAGAGCGTGGCGTGGATGGAGGACTTC

CTGAAGGGTTTCCCCGGCACCGTGGTGGCCATCACGCACGACCGGTACTTCCTGGACAAC

GTTGCCGGGTGGATTCTGGAGCTGGACCGAGGGGAGGGCTTCCCTTTCGAGGGGAACTAC

ACGGGTTTCCTAGAGAAAAAGATGGCCAGGCTTGAATCGGAGGCCAAGGTGGACAACAAG

AGGAAGAAGGCGCTGGGCAAGGAGCTAGAGTGGGTGAGGATGAACCCGAAGGCACGACAG

GCCAAGTCTAAGGCTCGCCTGGCGCGCTACGACAGCCTGAGCCAGGCAGACGAGGCTGCG

GACGAGGCTGCGAGGGCTTCGCTGGAGAGCATCTTCATCCCGCCCGGTCGACCCCTGGGA

ACGACCGTGGTGGAGGCgacgAACCTCGGCAAGGCGAAGGGAAAACGCCTTCTGTACGAC

GGGGTGAACTTCAGTCTTCCCCGGGGTGGGGTTGTTGGAATCATCGGGGCCAACGGAGCC

GGAAAGTCGACCCTCCTGAACATGATCGCCGGACTGGACTCTCCCGACATGGGTGAGCTG

GTCGTGGGAGAGACGGTGGACGTCATGTACGTGGACCAGAACAGGGAGGGCCTCGATGAC

CCGGAGCTGTCCGTCTTTGAGGCCGTCACGGAGGGAGCGGAGGAGATTAACCTGGGGCCG

CGAACGATCAACAGccgggCGTATTTGAGCTGGTTTAACTTCAAGGGTGGAGACCAGCAG

AAGAAGGTCAACCTCCTCTCTGGTGGCGAGCGAAACCGACTTAACTTGGCTCGCACGCTC

AAGCAGGGAGGAAACCTGCTGCTCCTCGACGAGCCGACCAACGACCTCGACGTTGACACG

CTCAGAGCTCTGGAAGAGGCTATCGACGGTTACCCGGGGTGCGCCGTGATCGTGAGCCAC

GACCGCTACTTCCTGGACAGGGTGGCCACGCACACGCTGGCGTTCGAAGACGACGGAGGC

GTGGTGTGGTTCGAGGGCAGCTTTGCCGAGTACGAGCAAGATCTCCGGAGAAGGGCCGGG

GGCAACGAGCCGAAGAGACCCAAGTTCAGGCCGCTGCCGACTGTCTGA

>g17365.t1

ATGGCCTACCTCTCCCTGATCGCTGTTCTCCTCTTGGGCGCCCCTCTGGCGGATGCCTTC

ACCATGTCCGTTAGGCCTGCCGCCTCTTCCACAGGAGGCGGGGTGCAGAGGAAGGACTTC

TTGAAGCAGATCGCAGGAGCAACCACCGCGGCCGTTGCTGGGGTGGCTTGCTCCCCCTTG

GCGTCTGTGGCTGCATCAACGCCGACGGTCGGAAGCATGGCACCCGACTTCACGCTCCCG

AGCAACACCGGCAAGGACATCAGCTTGAAGGACTTGCTCAAGAAGGCCAAGCACACCGTG

CTGTACTTCTACCCAGGGGACTTCACTTCGAGCTGCACCATCGAAGCACAGGGATTCCAG

AAGGACTTCGCCAAGTATGGCGAGAAGGACGCGCAGATCGTGGGCGTGAGCGTGGACTCA

ATCGAGAAGCACCTCAACTTTGAGAAGTCGTACGACCTCCAGTTCCCCCTTCTGTCGGAC

ATCGGAGCGAACGTGGCCGACCTCTACGGCTCTAAGCTGGACATCCCCTTCATGGGCAAG

TTCGCCAACCGCCTGACCTTCATCATCGGCTCCGACGGGAAGATAGAGAAGGTGTACACT

GACGTCGAGGGCAAGGTGGCGAAGCACTCCGCTGACGTACTCGCCACTCTTGCGACCCTG

TAG

>g17485.t1

atgacgaAGATGCTCTCGACTAACCCGAACACGCACGGCTTCAAGTGGCATCGATCGATG

CTTCGCTGTAAGGACCCCACTGCCAGCGTGAAGTACTACGAGGAACGCTTCGGAATGAAG

CTAGTGGACGTGTACCACAGCCCCACCCTAGGCAAAAGCACCTACTACCTGGCCTCTGTC

AGAGAGGGGGAGGAGTGGCCTGAGCCCGGCACAGCACAGGCCCATGAGAGGTTGTTCGAC

ATGGATCACTCCTGTGTCGAGCTTGAGCACGAACATGGCGCAGAGAACAATGCCGACCTG

CGGTATTCCAGTGGAAACGACGAGCCGCACCGAGGTTTTGGGCACCTGGCTCTCCTTACC

GACGACGTGTACAAGGCCAGCGAGGAGCTTGAGAAGGCTGGAGTGTCTTTCAAGAAGAAG

CCAGATGAGGGACGGATGAAAGGTTTAGCATTTGCGTACGACCCCGACGGATACTGGATC

GAGCTGGTGTCGAGGAACAAGGAAGCTGGTCACCCCGAGACATACAACCTGGGGCAGACA

ATGCTTCGCATTAAAGACGTCGACAAGTCCTTGGACTTCTACACCGGCGAAGGCGGGATG

GGGATGACCAAGGTTTGTGAGCTCCACTTCGAAAGCTTCAGCCTGTACTTCCTGCAGTCC

TTGTCTTCCGAAGAAGTTTCTTCGTTGCCTGCCGCTGACTCGCCCTTGGCGTACGAGAGA

ATGGGCCGATCGTGGGCTCCAGTGTTGGAGCTGACGCACAACCACGGGACGGAGAGCGAT

CCGGAGTTTTCGTATCACGATGGAAACACGGAACCGAAGGGGTTCGGGTTTTTGGGGTTC

ATCGTTGATGACTTCGATGGAGCGTCCAAGTGCCTCAGAGCAAGGGGGGCTAAGGAGATC

CCGGAACCGTCGATCTATGAGGGGAAGTTGGCTCGCTTTGCTGACCCGGACGGTTACCAC

GTGCAGCTCGCACTCCGTAAATCTGTGCTCGAGTAA

>g17491.t1

ATGAAGTTCAACGGACCGGTCCTGGCGGCTATGGTCGGTTTGTGCGCAGGAGTACAAGCC

TTCAACGTGAGCAACCTTACGCCGCTCTACCTAACTCTACCCCACCCTGGGGTCACCCGC

GGCCAGGTCCTTACTCTCCCATTGTCCCTTAGCGGTGGTCGCAGGCGAGCCCAGCTTGAT

CTGAAAAAACGATCGATCACGGTATCTTTCGTCCAAGAGACTCCTGGCGGCTACATATGT

GGCGTTGACGTCGTGAACTTCAAGACACCCTCAACCCACCGGATAGTGATGTCGAAGGAT

CAGCTGGAGGATTACCGCGAGCGGTCAGGAAAACAGGTGGAAGTTTCGGACCTGATGACG

TACGTCATCAAGTACATGATGGACAAGGGAATAAAGCTGGAAAACACCGAGGGCATGATC

GAAGCATCCGTGTTCCCTGTAAATTACTTCACTATTAAGCAGCTCACTTACTTCCACGAT

GACGTGGATGACAAGATCGTGGAGCTCTGCAAACAAGCCCCGGGCTTGTCGGAGTTTTGA

>g17631.t1

ATGCAGTCATCTCCGGCGGTTGAGGAGCAGGTTGCAACTCCCACCTTTGGCGACACTGGG

GGCGCTATGGTGGTGCTCAACAAGTGTGTCGTGTCACAAGGAGACACAGAGTTGATGTCA

AATGTTGATCTGATGGTAATGCCGGGAGATCGCGTGGGGTTGGTGGGGTCTAACGGGGCG

GGGAAGTCTACTTTGCTGCAGTGCATCGCCGGCTTCCGACCGATGGACGAGGGAACGTGC

ATCGTTAAAAACGGTGCAAGGATGGGGTACTTGCAGCAGAAGGGCGTCAGCGGATCGACC

AAGACGGTGTACGAGGAGGCGTGTTCTGAGATGGACACCATTAATAGGGCTCGCGATGCA

ATGGAGGCGGCCGAGGCCGACATTGAGGCGAACCCTATGGACCAAAAGGCGCTGGACAGG

CTGATGCAGGCGCAGTCCACGTTTGAGGCTGTGGGGGGTATGACGCAAGACAGGCTCGTG

GCACAGATTTTGGGCGGGTTGGGGTTCAGTGCTCCGGATCAGCAGCGGCTGTGCTCGACC

TTCAGCGGCGGGTGGCAGATGCGCATCGCTCTGGCGAAGCTTCTCCTGTCGGAGCCcgat

ctgctgctgctcgacGAGCCCACCAACCATCTAGACGCTTCGGCGAAGACTTGGCTTGGG

AGGTTCTTGTCGACGTACCAAGGCACCCTCGTCACGGTTTCGCACGACGAGGCACTTTTG

GAAGGCGTGAAGCTGTCCACCGTCGCCGAGGTGGCGAACCAGAGGGTGGAGGTCTTCAGG

GGCTGCGGATATAAGAAGTTCCTCTTCGAGAGGGACGAGCGGATGAAGGCTGCGAAGGCC

AAGTATGAGGCGGAACAGAAGGAGATGGCTCGTCTCCAGGGCTTCATCGACCGCTTCGGA

GCGCAGGCTACCAAGGCGTCCGCAGCCCAGTCCCGCGTGAAGATGCTGGCGAAGATGGAG

GCGAACGCTGCACCCTTGCCCGAGGGGAAGTCGACGTTCAAGGCCAAGATCAAGCTGCCT

ACGCCTCCCGCATGCCACACGAAACAGATCGTTCTGGAGGGCGCTAGCTTCGGCTGGGGA

GATGCTCCGCCCACGGCTACCGGCGTTAACCTCAAGCTGGAGAAGGGTCAAAAGCTGGCT

ATCCTTGGGCCCAATGGTGCGGGCAAGAGTACCCTGCTGAAAGCACTGGCGGGAGTGCTG

CCGCTGTCCGAGGGCGAAAGAAACGAAGGGGAGGGGCTCAAGCTCGGGGTTTTCACGCAG

GACTTGGCCCAAGACTTGCCGCAAGATGCCGTGGCCTTGGAGTTGGTGCTGGACAGGGTG

CGCGACCATGACTCCACCGTCTCCAACGAGCAAGCGCGCAACATCCTGGGATCCTTGGGG

CTGACAGGGCCGAAGGCGCTCCGCAAAATCGGCGTTCTCTCTGGGGGAGAGAAGGCAAGG

GTGGCTCTGGCAATCTTCGTCATGATCCCCTACAACCTCCTCATGCTTGACGAGCCGTCC

AACCATCTCGATGCTGAAACGCTCGACTCCTTGGTGGACGCTATCAAGGGATGGAAGggt

acggtggtggtggtgtcccaCAACAAAGACTTCGTTACCCGCCTTGCTCCGACGCACACG

GcggtagtggaggggggggtggtcaaGTACCTCGACAGGCCACCGAGGGCATCCGACTGG

GAGCACGACGCCGAAGGGCAAGGATGGAAgggggaggaggcggtggcggctgaGCTCACC

CCCGCTCAGAAGGCGGAGCAGAAGGCGCAGAGGGTGGTGGCTGACAAGCTGAGGAAGAAG

AAGGTTAACGCTCCTACAAGAATAGTCAAGATCCAGGCGTTGCTTGAGGAGCTGGAGAAC

AAAATAAAGACATTAGAAACTGACCTGTATGCCGAAGGGGCCAGCGCGGCAACCGTGGGA

AAGATAGTCAATGATAAGGAGGCCACCGAAGCTAGGGTCGTCAAGCTCTACAAGGAGtgG

GAAGAGTTGGACGAGTTGCTTGCAGAGGGATGA

>g17636.t1

ATGCTGGCCAAGAATGTGGCGAGCTTCAACATGGTACCTAATCCTGTCTCCAGGAATGGT

GATGTTGGGAATACGATCAGGACATCCTCTCCAGTGACGCACCTTCCGAAGACGCAACAA

CGGTCTTCACCTCTCAATGTTGCAGATGACACAACAGACACGACAGGGCTGCCGGACGTA

GCCCGCGCCGTCTTTGCAGAGGACAAGCGACCCGTGCTCTTGTACGATGGTGTGTGCAAC

ATGTGCAACGGCTTTGTCAACAGGTTTCTCGATGTAGACAAGGAGGAGAAGTTCCGGTTT

TCAGCCCTGCAAAGCGAAGCTGGTCGTTCTCTCCTTGCTCTGTCGGGGAGATCGCCCGAT

GACATTTCCAGTATCGTACTTGTCGAGGCGAATGGTGAGGCCCATATCCAGTCAGACGCT

CTGCTGAGAATGGGGCGTATCATTGGAGGACCTGTGGGGCTGGTGCTTTTTCCTGGGATT

GCTGTGCCCAAGTTTGTTCGCAACAAGATGTACGACGTGGTGGCCGACAATCGCTACAGC

TTCCTGGGAAAGAGGGAGGACTGCCGGTGTTCAGACGACCGATACGCGGACCGCTTTATA

TAG

>g17639.t1

atGACCGTTGCTTCCAAGGCCGTCGGCCTCTCCTTTGCGCTAGGACTCATTGCAAGACAG

GCCATGGCGGTGGATTACATTACGTGCGAGACGCTCCGCACTGCCATCGAGGCTGCTTCC

GCGGCGGCGACGTTCACTATCGGTGCTGACTTAGAAGAATGTACCGGATCCATCGTTATC

AGCGGAACAACCACGATCGACGGTGGCGGTCACACGATCACCATTTCCCCCACTTTCCTT

ACGGACTCCAACGCTCTCGGCTCGGGGTTGTTTGTAGTTGAGGCGGGGGGCACGCTGGAG

CTTAACGACGTGACCATAACGACCACCTCCGATGTCGAAAGCGAGGGGGTTCGAGGGATT

TACAATGAGGGGACTCTGACGGTGAACAACTGTGTGTTTTCGAAGCTCAACACGAACGTC

GCCGACGATGCCTTCGTTGACAGGGGAGCCGCGATTTACACCAGCTCCGACGGAAAGGAC

GTGACGATCACGGGCTCCACCTTTTCGTCTAACGTGGTGACCCATCAAGGCGGTGCCATC

TGTGCTTACAACTCCGACATCCTCACAGTGACTGGATGCGTGTTCACGAGCAACGAGGCT

GGTCTAGACGAGGCGGAACCTGGTTCTGGAGGCGACATCTACGCGGCCCGAGACGTGTCC

CTCACAGTGGAAGCGTCGAATTTCACCCAGTCCACTGCATACTACGGCGGAGCTGCCATC

GAGTGTTGCGGCGCGATTATCACTGACAGTCAGTTCACTGCCACAGAGTCGGCTATGACC

GAGGAGCACTACGGAGCCTTGCTCGTAGGCCGACCCGACGAAGCAGCGTGCCCGAGGGAG

CTCAAGATCACACACTGTGACTTCACGAGGTGCACAGTGGATGTCGGAAACGGAGCCGGC

GGATCGATGGCCATCTTCGACACCACTGCCGAAGTCGTGGGATGCACCTTTGAGAGCAGC

CAAGGGACGGCTGTGCTCTTCCAATCTTCCGTCCCGGACCCTTACCAGCTCACGATCACC

AGCAGCTATTTCTACTCCAACTCCAAGCCCGCGAAGGAGTTCAGCCTCAGCTCCTACACA

CAGGGAACCGCGCTTGCCATCCAGAACACCAATTTGGGGGATGGAGAGGAGCCGACCAAG

ATGGGAGAATTCACTAACGTTTTCTGCTTCGAGAACGAGCCGTACGAGTGCGAATTTGTC

TACCACGATGCGATTCTGGACCACTTGGGTGAATTCAAGTGCCAGATCTGCGACCAAGAT

GGAGACAGGCAGGACCCCGGCACGGACGACTTCTTgagcgcCGCTCGCGACAGTAGCGGG

TCTGGCAACAGCGACGGTATCGTGATCGGCCTGTCCCTTGCTCTCGGACTTACGCTCGTC

GTCATCGGGGCATTGGCGGTGTGGAAGTTCAGGGTTCGCAAGAATGCGCGCCGTCTTATG

GAGGATATGGGCGAGGGGGACCTGTGA

>g17668.t1

ATGGGTATAATGATACCTACGCCTCCAGACAACGTTGCCTTTTGCATCGCATTGAGCGTC

TTGCTCTTGTCTGTCGTGTTGCTACCAATAGTTGAGATATGTTTACAGCGGATAAGAGCC

GACATGGCATGGAAATCGATCGAGAAGCAGCAATCTGTCGATGTAACAAAGTACAACATG

ATTTCGGAAGCTATGGGCGACCGTGGGAGATGGGACACAGCACGTGTTGTCACGTTGATG

TTGGCCGCCTTCCATGTGTCCACTTGGGGATTGGAGCTGTCCTTAGACCTTGCGCTCAAC

ACGGACGGTCCCGTAGACCTTCTTAATCGCCCACCCCCAGTGCTGCAACGTACCGAGGTT

GTCGACCCCGCACACAATTTGACAGACTGGATAGTGCTGCCTCATGAACAACCTCGTGAA

GGAGGCGCCCTGAACAATTTCAAGGGGACGTTGGACGAGGGAAGCGCGAAGTCGTCTTAT

CGGATCGGCGACAGCTTTATAAAGGGGAAAACGTTTTTCGCCTCGTGGTCGAAGGAACCC

GCGCGGATTGAATCCGGCCTCTTTTACGACAGACTCAATGGCCGTGCCTCAGTCCAAGGG

CTAAATTGTTCGGAATCTTTGAGGAAAGCTGCCCTGTATGTAGGGGGTGTGTCTGATGCA

ACGAAAAAATGGGGGTGCGTAACGGAGTGTGAGGCTGGGCCTAAGTTGGTTGAAGGTAAT

TCGACCGTTCCCGCGAGCCCACCGACCATCATTTTAAGGAGTTCTGAGGGCATTGTGCAC

ATAATTGTGGAAGAGCAGAGCAGTTATCCGAGCTTCTTGTACTCGGTATGGACACCGGGC

GAGATAACTGCCGAGGTGACTTATCTAGACCACGTCTTTTACGTTTCAAGTACGACTCGC

CTCGCGGAGGCGATCGTGTCGGGTGTTGTGAATGGGGTATTGACCGGAGGTGGTTGTGTT

GATTTGCTTTCGAAATTCAGCATCAGCAACACAACCTACGATCTCGGAGGCGCTGAGCGT

GCGTTTCCTTTCGGCGAGCACCCGGGCTCGTCCTCTGTAGAGACATTGGACCAGGTAGAA

CCTATTGTAGCGGGTGTCCTTGTGAGTAATCTTGGCTCAGTGTCTGGAGCTCTGTTAATC

TTagtcaccgccgccgcgttAATTGGGTGCTTGGTTTTCCGCTCCAGTAGAACGCTTGAT

GTGTACAATCGAGACAAATTGATCCGGGCTGTCTCATTGCCTAGTGGAGAAGGTGCGGAT

GGCAAGCCCGTAGCTCTCAAAATCCATGTACGCCGAGATGCGAATGACGTGTTTGGTATC

GTCATCTCTGACGATGGTGTGTACAGAGGGTGCATCGGCTTTCGCAAGAGACTGGCGTCC

AAAGCGGCAAAGGTGGCACAGCGTTCGAGTAATAGTAGGACCGTGTCGCTGCCTGAGTCG

TTGCCGGCGCTTTCTCGGGAAATAACGCGCGATGGCATTCGCCCTACTTTGGAGAGTAGA

CGACCTACGCTTCGTGATTTGAACATTCGTACTCCGGTTCAGCCCCGTGCTACAGCCCAT

CGAGAACCGGTGGTCGTGGAGCTGATTGCTTCCCCGGTCCCTGGCCCTCGTTCAGAAAGA

CATGGATCGCGCGCCTTGGCTTTACGGAGCCTGAGCCGGAACAACTCCTCTTCCCCAAAC

GGAGGCCCTCTGCTGGAGGTCGAACCGCTGGGACGTGCGACCCGTGAGCATTCACGGGCC

CCTGCCGCCGATGTAGAGGCGCAAATGACCATGGCAGCAGAGTAG

>g17861.t1

ATGTGCTCCGGCTCGTGCCCCATAGAGGCCAGCCAGAGGCCATCCGGAGAAAAGGCTAGC

AGACCCACCCCGACCCTATGGAGGGATGGCAACAGGCCGGACTCTTCTCCCGCGGTACCG

CAAACGACCCCTGCGGGAGAAAAGGGGGCCGGGGGGCAGAGGTTTAGCGCGGAGCGGGTA

ACCGACATCGAGGTTTGTCCTGAGACGGGAAGCGTTACTTCCGTGTCGTGCGGGGACGAA

ACGTTTCCGGCGGACGCTGTGGTGTCGGCGGTCGGCATCAACGGCGTCAAGGGCATCGTC

AGAGCCGCGCCCGGGCTGTCCCGGCTGCCGTTCTTCTCTAGGATGATGAACCTCAGGTCT

GTAGACGCTTTGGCGGTGAGGCTCTACCTGGACAGGAGGGTGAGGGTGCCTTACCAAAGC

AACGCCTGCTTTGGCTTCGACAAGACGACCGGTTGGACTTTCTTCGACCTGAGCACAATG

CACGACTCCCTCAGCCAGAGCGCTGGAACGGTGCTAGAGGCCGACTTCTACCACGCCGAC

CAGTTGCTACCGCAGTCGGACGAAGACCTGGTAGCCAAgGTTCAACGCGATATCGCAGTG

TGCGTGCCAGCGGTGGGGAGAGCGAAGGTGGAGGACTACAGCGTGGTAAGGATCGCACAG

GGGGTGACCCACTTCTCCCCGGGATCGTACGACAGCATGCCGACTTGTAAAACCCCGATA

CCCAACATGTTCATGAGCGGGGACTGGGTTATCAGCGACCACGGCAGCTTCTCACAAGGC

GAGGATCACTCCATGCAAAAGACGTATTAG

>g17994.t1

ATGAGCGCAGCGCAAGACGGCGCATGCTCGCGGTCCGAAGCCCTGAGCACCTTCGGAGCG

GCCCTCCTCGGTGCGGTGGCCGTCGGCGGGGGCGTCCCGTCCGCGGCTCTAGCGGCATCG

ACGGCCGATGTCAACAAGAAGTTGACCGCGTACGACCTCCCTCCCGTGGTGGACGCTCCG

GACGGGTTCAACACGCTCCTCGAGGGCTACGGGAAGGACGGTGCCCTGAAGAACTCGCTC

GGGTCCAGCAACCGAGACCCCATCCTCGTCACCTGGAACTACCCTAAGGGTTGGATCGTC

GAGAGgcccaacaccgacaccaacaaggAGGCTGGCACCATCTCGACGGGGGATTACGGC

CGGGGGGACTCGGCGGCCCTCTTCGTCGCCCCCAAGTCCCTCCTCGGGGGCTCCAACCTG

TCGAGCAAGGAGACCATGGAGGGAGTCGTGAAGAAAGCTCTGTCGCAGAAGGGTGACAAC

CAGTTCCAAAGCTTCAAGTTGAAGAGCGTCAAGGAGGGAGTGACCGACTTTGCCGGAAGC

AAGTACTACATCGCCGACTTCCAGTACGAGCTGCTGACCGGAGCGGGTTTCACTGTGGAG

AGGAAGGGGATGGCATCTGTCGCACAGATCGGGAACACGGTTAACGCGGTAGTGGGGGCA

ACAACGGCCAACCGCGCTAAAACGGTCAAGCCGCAGCTGGTGGAGATTGCCAACAGTTTC

CGCGTTTACGCTGGAAAATTCGAATAG

>g18017.t1

ATGACCAGACTTATCGCTATCGTGGCTTGCGCCGCGCCTTTGGCTCTGGCTGCTACTCAG

GACGCGTCTTTGCGAGGGGTCAAGgtAGCGATGGGAACTGACGAGTCCGTGCACGTCCAA

CGACACCTCACGGGGGACGACGACGGTCGCGGGAtgggccccacccccaccaccacctac

gACGGGTGGGACGACCCGTCCACGGGGGACCAGGGCCTTGTAGCCGACACAGAGGAGGAG

GGCACCTACAAGCACATCGGCTGCTTCTCGGACTCGAAGGAGGACCGAGTTCTGGGCCAC

ATGATATGGGCGGATGACATGACCGCTAAGATGTGCCACATCCACTGCAACGTACGAGGT

GCGTTCTACATGGCAACCCAGTACGGGACAGAGTGCTGGTGCTCTCGCGCGCAGGACCTC

GACTACGCTTCCGTTGACGTCGGCGTGTGCGACATGGAATGCTCAGGCGACGCGGACGAG

TCTTGCGGCGGTTACAACGCGTTCGACCTGTACGAGCTCGTGTGGCCGGCAGAGCCCACG

GACCCGGAGTACATGGATTGCTTCGCCGACAAAAAGGAAGACCGCGTCATGTCTAAAATG

CACGTTGCCCCGGACATGACCCAAGCGGCCTGCCGGAGCCACTGCGACGGCTACATGTAC

TACGCCACACAGTACGGAAACGAATGCTGGTGCGGAAGTTCGGAGGTGTTCAGTGACTAC

GACAAGCACGGCGAGGGAACGTGCCACATGCCTTGCTCGGGCGACGCTTCGGTGGCATGC

GGTGGTTACAACGCGTTCAGCCTGTACCGCTTCATAGACGTCCAGCCGGGAGGCGCACCC

GAACCCTCCACCCCCTCGCCGGTCCCCGAGGCCACCCAGTCCCCCGTGAAGGCACCCACG

CCGTCTCCTGAGAAGGCACCCACACCATCCCCGGAGAAGGCACCCACGCCGTCCCCCGAG

AAGGCACCCACGCCATCCCCCGAGAAGGCACCCACTCCGTCCCCCGAGCCCGAACCGACC

CCCTCGCCCGTTGCCAAGCCCACGCCGTCCCCTGAGAAGGCACCCACTCCGTCCCCCGAG

CCCGAACCGACCCCCTCGCCTGTTCCCCAGCCCACACCTTCCCCTGAGAAGGCACCCACT

CCGTCCCCTGAGCCCGCACCGACGCCCGCCCCTGTCGAGGCACCCACCCCGGCACCCGTC

GACAGCGATgacggtggtggaggcggtggtggtgcggaGGGGTCGTTCGACGACACCGTG

TACAACGGGGATGGTACCTACTACGGCGCCACGACCGGCGGCAACTGCGCTTTCGGAACG

AACGTCCCTTCGATGTACAACGGCATGATCCCAGTCGCGCTCAACGAACCCCAGTACGGC

GACTCTCTCATGTGCGGCGCATGCATCGAGGGGGAGGGATCCGGGGTTGGCTCCGGCGGC

AACCCGATTTCTGGCAAATTCAAGGCGTACGTCTCCGACAAGTGCCCCGAGTGCAAGAAC

GGCGACCTCGACTTCTCAGAGTCCGGCGACGGCCGctgggaaatttcttggaaGTTTGTA

CCCTGCTCTGGTGGAAACGAGGAGCCTAGCTTTATGTTCGAGGGCAGCCACGAGTACTAC

TGGAAGCTGCAGCCTCGCGGGACGAAGTCGCCGGTGGTAGAGCTCTCCGTTAACGGGGCG

AAGGGTGAGAGAACGGACGACAACTTCTTCATCGTGACGGAGGGAGTTCCGTTCTACGGC

GAACAACACGTGGTGACCAAGACTGTGGGCGGCACAACCCACGAGATGGAAGTTGCGATC

TGA

>g18210.t1

ATGCGCATACCCACAGATGACAGCTATGACGTGCCCGTGGACCCCAAGCAGCAGGACAAG

GCTACCACGTTCAAGCTCAACAGCTTCAGGCGACCCCACATGCGCGCATTTCACTTCGCG

TGGTTCGGTTTCTTCATGGCCTTCGTGTCCTGGTTCGCCTTCGCGCCCCTCATGAAGGAG

ATCAAGAAGGACCTCGGCATGACCACAAACGAGGTCTACAACGCCAACATCGCTTCCGTG

TCCTCCACAGTATTATCGCGGTTCATCGTTGGACCCCTGTGCGATACCTTTGGCGCGCGC

ATCATCTCCACCACGCTGCTCATCATGGGAAGCATCCCGACGTTCTTTGGAGGTTTGGTG

AACAGCGCCGAGGATGTGGCAATTATCCGTTTCTTCATTGGCGTGATGGGCGCAACTTTC

GTGTGCACTCAGTTTTGGTCTTCGCAGATCTTCGTGAAGGAATTTGTCGGCACTGCTAAC

GCCACCACCGGAGGCTGGGGCAACCTCGGCGGCGGCGTGACCCAGATCTTCATGGTGGGA

ATCTGGAAAGCTTTCCAGATAAGTTACAGCAGCGAGACGTCGTGGCGACTTTCTTTCATC

GTCCCGGCCGCCATCGTGCTCTGCGTCGCTATGGGGCAGCTGTTCCTCGCCGACGACTGC

CCCAAGGGAAACTACAAGGAGCTGGAGGCGCACGGCGCCATGACCCGAAAGTCCTCCGCT

GTTTCCTTCAGAAAGGGATACGTCAACAACAACTCGTGGCTACTGTTCTGCCAGTACGCG

GCTTGCTTCGGCGTGGAGCTGACGGTCAACAACGTGGCGGCTACGTACTTTTCGGACGAG

TTCGACCTCTCAACGTCCAAGGCAGGGATTGTGGCGTCGCTGTTCGGCCTGATGAACCTG

TTCGCTCGTTCGTTGGGCGGCATCTGGTCTGACTTCCTCTACCGTAAGTTCGGCAGCGGA

GTCACCGGCATGCGTGGACGATACTTCGCCCAGTGGTCGGCCCTTGTGTGGGAGGCCGTC

TTCTTGTTCGTCTTCACCCAAATGAACACAATTGGCCCCGCCATCGTGGTCCTCATCCTC

TTCTCCGTTGGCGTCCAGATGGCCGAGGGGTGCACGTACGGCATCGTGCCTTACGTCTGT

CCGGAGGCGACGGGAGCCGTGTCGGGGATCGTGGGCGCAGGTGGAAACTTCGGGGCCGTG

ATGTGGGGCCTTATCTTCCGCTTCGGCCCTTCGGATCCAAGGACGGTGTTTCGTATAATG

TCAGGGCTTGTCTTAGCCTTGTCGTGCCTCACCCCCCTGCTCAAGATCCGCGGATACGCC

ACGGTGTTCGGCGCTCCTAAGGGCGAACCTGACTCGATCGCGGATATCTAA

>g18322.t1

ATGACGTGCGTGAGCGTGTGCGTCCTCGGCTTGGCGATGGCAATGACGCCGCTGACCCAG

GCGTTCGTCGCCCCCGGCAGCGGCTTCTCCGCGGTGCACGGGCGCGATGTCGCATCTATC

GCTTGCTCGAAGACGTCGCCGCGCACTAGCTGCAGGATGACGGCAGAGGGGGAGGAGCCG

CCAACGGAAGAAGGGGAGAAGAAGGGGAAGACGAAGCCGCCGGCGTGGATGTTCAACGAC

CAGGGCGTCGCCTACGCGCCGTGGATGGTGGATGCCTTCGACCCCGAGaACCTGGCGAAG

GTAGCGGCTTCGATCGAAGCTCGCAAGGAGAAGGAGGCCAACATGGTGCCGGAGGCGCTC

GGCGCGCTCGCCCGCGACCCTCAACAGATGGAACTCTCCGGAGCAGGCCTGTACGCGAAG

AGGGTGGCGGACGACATGGTGGAGCTGACGTGGAAAACCGGCAACGAGAAGGGCAACGTC

GGGTTTATAGTGAGCAGGAGGGCGGCCAAGACGGAAGGCTGGGAAGAAATCGCGTCGTTC

ACCGACTTCCCGCCGCTTAACTCCAAGGGAGAGGGAGGTGGGACTTACAACTACATGGAC

GAGGGCGTCTCGGAGGGCACGTGGGTGTACAGGGTGTCCGACATGAGCAAGTCCGGAACC

AGGAGCGACTTGTGTCAGACGCTCATCGAGTTGCAGAGCAGCTCCGACGCTTTGCAGACA

AAGATCGGATTGGCTGGCCTCGTCGTCGTCCTTGCTGTCGGCGTTGCCGCCGGCACCTTC

ATCGACCCCTTCGCCCAGTAA

>g18732.t1

ATGTCCGGCGAAGAAGAGGTGGACGCAGATCTGCCGCAGCTGTGGGCGTCATCTCTTTCC

ACCAAGAAGGAGCTCGTGCCCGCCATAGCGGAGGCGGCTGCCAAAGCGCTCGCGCAATTC

CCGGAAGGCGCATCGATAGACCTTGCGCTGATTCACGTTTCTAGCATATACGGGAACGCG

GAGAGGCTGGAAACGGTGGTTCCGGAACTCCGGAAAGCCGTGCCAGGACTGGAGTCCGTC

GTAGGCTGTAGCTCAGCTGGAGCGGTTGGTATGCAGGGCAAGGACCGCGCAGTGGAGATT

GAAAACAGGACGTGCTTCGGGCTTACCCTAGCCAGCCTGCCGGGGGTGAAGGTTAAGCCT

TTCTACCTCGCCGCCATGGATGTGCCGGACCCGCTCGACCCCTCGTCGGAATGGAAAAAA

GCCGTGCACCTGCGCGACGAGGACATCCCCGCGATTGGGGCGGACGGGGagacaggagag

gggggggggggggggggcggagttggAGCACCCATATTTTTGTCGTACGCGACCACGCAA

TCCATCGATGCGCTCGGGGACTACATGGCGGGAATGGACGCTGCGTTCCCTCAGTCGCAG

AAAGTCGCGGCCATTGCGTCAACGCGCACACACGAGGCTAACCGCCGCACAACGACCCTC

ACCAACGGTAGCCTGACAAGGTCTTGTGTGTTTTTCGGAGAGGGTGGGACGAGCGGGACT

GTCATGGAGCAGAACTCGTTCTACAAGGAGGGTATGGTCGGTGTGTCTCTCGTGGGGGAC

ATCCGAATGCGCTCCTTCATTACTCAGGGGGCGAGGCGTGTCGGACCGACATTCCACGCG

GACAAGGTGGACGGGCCAGTGGTGAAAAGCCTTCGCGTCGCGGACAGAGGAGTCGACGGC

GAGGGACAGGACGATTGGATAAGCCCGGCGCTGCCGCCGCTAGCAATGATAAAGCAGGTC

CAGAAGAAACTGTCGGACGAAGACAAGCAGCTCGTGCAAAACAACATGCTCGTGGGCATC

GCACCTGAGCTTATCGGCAACACCCCGGAGGAGATGCGAGCGATCAGCACTGGAAAGGGA

GACCAGTTCGTCGTGCAGGGAGTCCTCAGAACGAGCATGCGGGACGGTGCGATCACCATC

GGCAGCTCGATCGACCCAGGCACGCGACTGCAACTCTTCGTGCGAGATAGGATCGCCGCA

GGGGACGAATTTAACGCCGCCCTCGTGGCCTACAAACGCCGTCAGCTCATGGAAACCCTG

GCCTCCACGTCGACGGACGAAACAAGCGAAGCGAGCGGCGGGGTAGACCACCCCGACGAG

GAGCAGCCCGAAAACCAGAATGTTAACGTGGTGGACAGCTCGATCGCCGCGCCTCCTGGC

GGAGCTCCGAAAGAGACGGCGGCGTTCCGAGCGGCGGGGGCGTTgatgttccctgggttg

gacaGGGGACGGAATCTGTGGGACGAGGACAACTTCCAAAGCTCGAAGGTTTTCAAGACG

GTGCCAGTACCGCTAGGAGGCTTTTTTACGAACGGAGTGGCAGGTTGTATATCGGAAGGA

TCCCGCACAACCCTCTTCGGCTCGAGCACGTCGGTGGTGGTCTTCTCCCCCATCTCAGCC

CGGCGCACAACCGCAGAGCTCCCGGAGGGCTCGGCGGGCAACGCGCAGGACGCAGGCAAG

CTTCGCGTGTGCGGGACCGGGCCCCGGTCAGCGGTTGTCGTCGTCCGTGCGGGCGGTGGT

CCAGTGGCAGGTGTGGGTGAGGACGGGGAAGACATTCTCAGCGACGATAGCGACGACTTT

GTCGTGATGCGCAGAGACGTCAACGCCGGCCGGGCGGTCATGTCGGGCCCTGTGCTCTAC

AGCGTCGCTGAGAGCGTGGCGCAGCCCAGGAACTCCCTGGAGGCATTGGTCTGGGAGAAG

GAAGCGGAGGTGGACCGCTTTCGCGACCGCTGGCCAATGAGCATGCTCGTCAGCCGGTCG

CGGCTGTTcaacctcgaggaggagaacAAGCCCAGAGATGTCCTTGCTGCGCTGCGAAAG

GGGAAAGGCGCGAGCGGTGTGTCCATCTTGGCAGAGGTTAAGCGGAAGGCACCTGTCACG

GGGCCGATGCGACGCGGGGAGATGGACGTGGTGGAGTACGCTCGAGCGATCGAGGGGGCT

GGGGTGGCTGCGATTGCCGTCAACACCGACCGAAACTTCTTCGGCTGCACCTACGAAGAC

CTCACTAGCATAAGGAATGCGGTCAAGGTCCCGGTGATGTGCAGCGATGTCGTCGTCTAC

CCCTACCAGATCTACCAGGCCAGATTAGCCGGGGCCGATGCTCTCAAGCTCATCGCACCG

GCTCTGCCACCAAAGGACCTGATGTATTTCCACAAGATAGCTTCTGCTCTCGGGATGCAG

TGCATCGTGGCGGTGAGCTCTGTGAAGCAGATGATTGCGGCGCTACGGCTGCCGGGCATC

CGAGCGGTGTCGATCAACAACCGGAACATGGCCACGTGGGCCCTCGACACATCAAGGGTC

GACCGAATACTTGGGGACATTGAGGTCCAAGCAGAGCTAGAAGGGAAGGACATCACCGTG

TTGGTGGAGGGCGGTCTCAAGACCAGGGAAGACATTGACCGTGTGAAAGCCGCTGGCGTG

TCGTACGTTGTGGTGGGCGAGGCGTTTCTCCGAGAAGAAGACCCGGCAAAGGCAGCCCAA

TCGTTTTTGCTTTAA

>g18740.t1

ATGTTCCCAAATGCCCAGCGGATGCTGAGCTATGGAAGGCTGCTCTTCTTCGCCGCCGCG

CTTGCGCCTGTTGTGAGATGTCTCCTGCAAGTACCATACGGGAACTGGTGCACTAAGCTA

CCCGCTCGGCACGGTGGTATTGTCAGTGCTGTTCTGGCGCGCAAGCAAACGTTCTACAGG

GTAGTCCATCGTCCAACCGTACGATGCCACGCACGTTCAACCCGGCGTCCAACCGACGCA

AGAGAAGCTTTAGCTTTGCCTTGTCCTCCTTTTATCGCTGTCTTACGCGACGTGTGCCTG

GAGGATGCATCGGGGGTAGCGCGTGCTTTACGTGCAGGCGGGTTCAATATGGTGTCAATT

ACTGCTGACACCGCCGGGTTCGTGCAAGTGCTGCAGTCGATTACCAACGACGACTTCCTT

GAGGGCATGGTAGTGGGTGTATCTTCTGTTACGTCTCCAGAGCAGGTGGAGTTGGCCTAC

GCCAGCCAGGCCGAATTCGTGAGTAGTACCTGTTGCGATCCTGCAGTGGTAAGGCGCACC

AAGGAGCTCGGCCTCATCAGCATTCCAGGAGTTTCAACGGCTGCCCAGGCCATAGCGGCC

GTTGAAGCAGGGGGGGATATTCTCAAGATCTTCCCTGCCACGAGCCTCTCCTCGGCTGCC

ATCCGAAACATTGCAGGGTGTATACCCTCCGATGTGCCTTTTGTTGTGGCGGGCGGAATA

GAAGTCGAGCAATTGGATGCCCACTGCGACGCCGGGGCGTCCGGCTTTGCTGTAGGTCGA

ACATTGTTCAAGCCCGGCATGAGCCTAGTTGATCTTAAAGCGAAAGCTCGTAAGTTTGTA

CGTCGCGGGAGTGGTTTGCGGTGGAGCCACTATGGCCGACCGAGAGAACCTGCATGA

>g18763.t1

ATGGTGCAACTGGCACGTACGATAGCCGTGCTTGTGGGAGGGGCAGCATGCGCGTCGGCA

TTCACAGGCCCGATGGCCGTGACCAGCTTTACCGGCGTGCGTGTGCCTGAGAGCGTGGAC

AGCGTGGCAGCTGCACCCTCGGCGCGTCGCTCGGTCGTAACGCCAAGGATGGGCGGGAAG

GAAAACGCTATTAGGCAGCGTATCACCACCGTCAACAACACGAAGAAGATCACGACGGCA

ATGCGGCTGGTCGCTGCCGCCAAGGTGCGTCGTGCGCAGGAGGCCGTGGTCAAGACCCGT

CCTTTCTCTGAGACCCTTCAGAGCATCTTCGGGGCCCTCATTGCTCAGCTCGGAAAGGAG

GACGTCAACCTCCCCCTTCTCAACGCGAGGGAGGTCAAGAACGTGTTGCTAGTGGGGCTC

TCAGGCGACCGTGGCCTGTGCGGGgcgtacaactcgtacgccatcAAGAAGACGGAGGCG

AGGGGAAAGGAGCTCGCGTCTCAGGGATACAACGTGGAGTACATCACCATCGGCAAGAAG

ATCTCGCAGTACTTCCGACGCAGGGAGGAGATCTACACCGTGAAGCGGGCGTTCGACTGC

GGACAGGCCCCCAACGCGGCCGAGGCTTCGGCCATTGCCCAGGAGCTCCTGACGGACTAC

CTCTCGGGCGAGACGGACAGCGTTGAGATCGTGTACACGAAGTTCGTTTCTCTCATCGCG

TCGGAGCCTTCGATCCGCACTTTGCTGCCCCTGTCCATCCGCGGCATTGAGGACGAGAAG

GACGAGATCTTCCAGCTCACCAGCGACGGAGGAAAGTTCAAGGTGGAGAGGGAAGAGGTC

GGATCCACGGAGCCCGCTGAGCTGCCGCAGGACCTTATCTTCGAGCAGGACCCCATCCAG

ATCCTCAACTCCATCCTTCCTCTGTACCTCGACGGGCAGGTGCTGCGCATGCTTCAGGAG

TCGGTTGCGGCAGAACTGGCTGCGCGAATGGCTTCCATGGCGGCTGCCTCGGACAACGCC

GCTGACCTCTCCAAGCGGCTGTCTCTTACTTACAACCGCGCTCGTCAGGCAAGCGTCACC

CAGGAGCTCCTGGAGATCGTTGCTGGGGCGGACGCGGCGCAGTCGGGATCCAAGTAG

>g18774.t1

ATGGACTCCTCCACGAGTATGCGATCGATGAAGGTAAAGGCAGGGGCCCTTGCCATGCTG

ATGGCATCCAGCGAAGCCTTCATGGTGTCGAACAACATCCGGCTCGGCACAAGCGCACCA

TCGGCGTTCGCTGGGCAATCAACAGCAGCGATCAGCGCGCAGTGCGCCGTGAGGCCCATG

CCGGTTGCACCAGCGATGGCAATGAAGGGGGACGGGTTCAACCCCGCAAACTCTCTCGCA

GCGATGCTGGCAGCCGCCACCATCGCGTTTTCTTCGCCTGCTTTCGCTGTTGACGTACCC

CCGCCATCCCCCTTCGCGAACGCTGGCGGCCAGACTCCTCCTGCGATGATGCAGAAGCAG

TCGGTTTCCACCGTCGAAGACATCCGCTACTCGGACTTCGTGAATGCTGTGGAGAAGGAC

GAGATCGAGAAGGTGTCGTTCTCGTACGATGGCAAGAAGCTTATTGCCGTGGACACTGAC

GGTGTACGCGTGAAGCTCGACTCCATCCCGAACGACCCAGAGCTTCTGACGATCCTGACG

AAGCACAAGGTGGACGTGACCGTCATGCCCAACCAGACCAACCAGGGCGCTGGCGGCTTT

GGCCAGCTTGGTTCCCTCATCTTCCCTGCCCTTCTGTTCGGCAGCTTGCTCTTCCTGTCC

CGGAGGGGTGGTGGgcagggaggaggaggtggtatGGGGGGCGGCGGCATGCCAGGCGGG

GGAAACCCGATGGAGATGACCAAGTCCAAGGGGAAGCTGGAGGTGAACCCGGATACTGGT

GTCACCTTCGACCAGGTCGCTGGGTGTGACGCAGCCAAGTTCGAGCTGGAGGAGGTGGTG

GACTTCCTCAAGAACCCCGATAAGTACACCAAGGTTGGTGCCAAGATCCCCCGTGGCGTG

ATCCTGGAAGGTCCTCCCGGGACCGGTAAGACGCTCATCGCGCGGGCCGTGGCCGGCGAG

GCGGGCGTCCCCTTCATCGCCACGTCTGGGTCCGAGTTCGTGGAGATGTTCGTCGGCGTG

GGTGCCGCGCGTGTGCGCGACTTGTTCGACAAGGCGAAGGAGAACTCGCCGTGCATCATC

TTCATCGACGAGATCGACGCCGTTGGCCGGCAACGTGGCAGCGGCATGGCTGGCGGCAAC

GACGAGCGCGAGCAGACCCTCAACCAGATGCTGGTGGAGATGGACGGCTTCGTCGGGAAC

CCTGGCGTGATCGTCATGGCCGCCACTAACCGTATCGACATCCTCGACGACGCCCTGCTC

CGCCCCGGGCGCTTCGACCGCAGGGTGCTCGTCGACCTGCCGAACAACACCGGGCGCGTC

GCCATCCTCAAGGTGCACGCACGCGGGAAGCCCCTGGCCCCCGACGTGGACATCGAGGGT

ATCGCTCGCCGTACCCCCGGCTTCTCCGGCGCGCAGCTGAAGAACTTGTTGAACGAGGCC

GCGATTTTCGCAGCACGGAAGCAGCGCCCCATCCCCAGCATCGAGTGGGAGGACGTCGAC

GCCGCCGTGGACCGTCTGCTGGTCGGCCTCGAGAAGAAGGGCGCCCGCGTGAACGAGGAC

ATGCGCAACATCGTGGCCTTCCACGAGTCGGGACACGCCATCGTGGGAGCTCTGATGCCC

GACTACGACACGGTGCAGAAGGTGACGATCGTGCCCCGCACCAACGGAGCGGGTGGGTTG

ACTTTCTTCTCGCCTTCCGAGGAGCGCTTGGAGTGCGGCCTGTACTCGAAGGTGTACATG

GAGTCTCAGCTGGCGGTGGCGCTCGGGGGGCGCTTGGCTGAGGAGATCATCTATGGAGAG

GACCAGGTAACCACCGGCGCGTCGAACGACTTCCAGCAGGTGGCCAACATCGCCTTCCAG

ATGGTGACACAGTGGGGAATGAGCGAGGAGATCGGCCCCTTCGTGGTCAACATGGGGATG

CAGGGGCAGGAGGGCGACCAGTGGGGGCCGACCATGAACGTTAGGGTCAACATGGAGGTG

GAGCGATTGGTGAACCAGGCCTACTTCCGCGCGAAGAAGATCCTCACGGAGAACCGGGCT

CTGCTTGACATCCTCGCGGAGAAGTTGTTGGAGCAGGACACGGTGACCTCCGAGGAGCTG

TCGTTGATGATCGCGCAGAACGCGGTCGAGACGGCACCGTACGAGACGTACGACGGCCCC

GCCGACAAGACGCAGCTTCCCTTCCAGAAGCCGGTGTCTGACTACTTCTAG

>g19087.t1

ATGAAGTCCGCTTGTGCTATGGTTTTGGCGTGCGCCGCGGGCGCCTCGGCCTTCGTGGCT

CCCAGcGCGTTCAACGGTGCCGCTTTGTCCGCCGCCAAGCCCGCGTCGTCGTCGATGAAG

ATGTCCTTTGAGTCGGAGATCGGAGCCCAGGCGCCCATCGGCTACTGGGATCCCCTTGGG

CTCCTCACCAAGGACCCCTCGCAGGAGCGGTTCGACCGCCTCCGGTACGTGGAGGTGAAG

CACGGCCGCATTGCGATGCTCGCCATCCTCGGCCACATCACGGCACAGAACTTCCGCTTC

CCCGGCATGCTCGCCCCCTCGGCCGGCCTCTCCTTCGCCGACTGCCCCAACGGGATCGCC

GCGCTGTCGAAGATCCCCCTGTACGGCCTCGTGCAGATCTTCCTCTTCATCGGATTCCTT

GAGACCAAGGTGATGTTGCAGAAGGAGGGATCCTTCCCCGGGGACATGTCGCTCATCAAC

CCCTTCGAGAAGGAGTGGGAGGCATGGACGGAGGAGGAGCAGAACACCAAGCGCGCGATC

GAGCTCAACAACGGCCGCGCCGCGCAGATGGGAATCCTCGGACTCATGGTGCACGAGCAG

ATCAACAACACGCCGTACGTCATCAACGAAATCCTCGGCTCGCACGTTGCCTTCAACTAA

>g19128.t1

ATGTATGAGGGGTGGCAGATCGCGCCTTTCCGCAACCCGCTGCAGTCATCTCCAGCTGGC

CAGAGGGTCCCAGTTGAGGAGTACAACAACGCTTGGCTAAGGAGTGTCGCATATCAAACG

CGCTACACGTTCCAGGCGCTCGGGCTAGGCCTCTTTACCATGGGCGTGTACGGCGCCAAC

ACTTGGACCAAAGCACTGGTTGGTGGCAGCATGGCGGTGGCGCTGCTTGGCCCGAAGGAG

CCTCGGCTTGGCCTGACCCGCCTCGTCGACGGCGAGAAGAGACCCGCCCTGCCGCTCTCG

CTGTCCCTTGCGGTGGTGTTCACCATCAACGTGGTCGCCAACCTGGCCGCCAACATTAGG

CTCTTCGCCCCCTCCATGGAGGAACAGGTGCCCTTCTTTTCCGATGGCATCGTGGTGTCT

GGCTGGGTCTGCGCCATAATACCGACTCTCATTGTCGGAGCGGGCGGGGCTATGAAAACC

TACATTGCGGAGACGTCGTTCAATCAGTGGTACCATCTCGTCGCCCTGGTGTCCCTGCTG

GCCGGCCTGTCGTCGAGCCTCCAGCTTTACCAGCACCTTGTGGACTTCATCAAGTCGTAG

>g19146.t1

ATGCTCCGCTCCTCCACCAAGCTCGCTGCAGCAGGATGCCGTGCGGGGCGCGGTGTCCGG

GATAGCCTTGCCTCTTCTCCCCTGGCGTCTCAGCAGCCTATGGCGAAACGCGGGCAGCTC

CTAGGCTTGTGTCGCGCAAGGGGGTTATCCCACCCGGCGCTTCGACAGCAACAGCATTTG

TCGGCGACGCACAGCTCGTCGATGGCTACCTTTTGCGGGCGCCACCGGAGCGTGGTATGT

GGCGCCTCCGTGTCTCGAGGAGTTCTCCTGGCCGACTCGAGGAGGCGGCCGGCCGCGCGA

GAGGCGGCGCCGGGGAGCATCATCAACCCTTCTATGATTGTACGAGGAGTCCAGACCGGA

CCGTTCAGCGGGCCCTTTACTCCGGGGTCGAAGCCGCCCATAAAGCCTTCCGACTCATCG

GAAGTCGCTAAGAAACCGACGCTCATGAACCGTTACATCGCTCCGCCAGATGACGGGTTG

ACGCGCTACCCCACTCTCCGCTCGCCTCAGGGTGTGCTGCGAGGGATGGACTACATGGGT

ACAGCCGTGTTCGCGCTATCGGGAACCGTGACCGCGGGACAAGTGGGCATGGACCTCATG

GGGTGCGTTATCGTGGGAACCATCACCGCTACGGGAGGGGGCACGGTGCGCGACCTTCTG

CTGGGCAACACCCCGGTGTTTTGGATGCACGAGACGGAGTACCTGTGGATATGCCTCTTG

ACCAGCCTCGGGGTGTTCTTCCTGTGGTCCTACCTCGCCAAAATAGGAGTGCGTGACGAC

ATGGCCCTCCTTAACTGGGCGGATGCAATGGGGATAGGCGCTTTCTGCTGCATCGGAGCG

CAGGCCGGCGTTCGCAAGGGACTGTCGAAGATTGTCTGCGTGGCGTGCGGCATGCTCACG

TCTACCTTCGGCGGCGTGATTCGCGACGTGCTCTGCTCGCGCCCTCCAAGGATCCTGTTC

TCCCATGCGGAGATCTATGCCTCCACTGCGGTGCTGGGCTCCACCGTGTACATCTTCAGC

CGGGCCGCGGGCTTGCCTCCAGTGGGACGAATCGCCTGTGGctttgccgccgccgcgggt

CTTCGGGTGCTGGCCTTCACCACGGACATCAGGCTGCCCAGCTGGGTGCAGCCTTCCAGT

GCGGCCGTCGATAACTTGGAAAAACTGGAGGCCGAAAGCGAGGCCAACAAGATCAAGATT

TCCTCCTGA

>g19147.t1

ATGACGGCGCTGATTGCAGTCGCTCGCCGCGCAGGGCTTGTCCGCAGTGTATTACCCAGC

GGTGGCAGCTCCAACACTGGCTTGCTTTCGAGAGCGTTATTGGGAAGCCCGACTTCATCG

CTGCGAGGAGCATCAACATCATTTCGagaccactgctgccgctctaTCTCGGCGTCTCCC

CACCGGCAGCAGGGGAGCGGAGGCCCGATGTTTTCAGTGCTTGGTAGTCGATTACCGTTT

TCTGGCGAGGAGCTCGCTTCCCCGTCGGCCGGCCACCACAGCGCTGCTGGTAGTAGTAGA

AGTTGCAGCAGCTCGAGCGGTCCAACCAAGCCGCTGCTCAAGCCGATGAGGTCGGTCCTG

TACACGCCAGGATCATCGAGGCACCTCTACAAGATCAGGGATATCGCCTGCGATGCGTCG

CTCATCGACTTGGAGGATGGCGTTGTGCCGGAGGCAAAGGACAACGCTCGCGAGCGGGTG

TGCTCGGAGGTGGCGAAGGGAGGCTATGGACgcaaggcggtggtggtgaggaTTAACTCG

CTCGACTCGCCCTGGGGGGAGGACGACATCAAGGAGGTGGCCAAGCTTACCTTGGATGGG

GTCGTCATACCCAAGGTTGAAAGTGCTGCGGACGTTCGTAGGGTGGCAGACCTTCTTCGG

CAGCATGGGGGCAAAGACACGGACCTGTGGTGCATGATCGAAACCCCCCTGGGAGTGACG

CGGGCGAACGAGATCGCTTCGGCGCACGAGACCGTGTCGTGTCTCGTGCTGGGCACCTCG

GACCTGTCCAGGGACCTCCAGGCTAACCACACGCCTGACCGCACCCCTCTCCTCTACAGc

ctgtCCCGATGCGTCCTGGCGGCTCGTACGTATGGCCTGAGATGTCTCGACGGGGTCCAC

CTGGACATGACGGACGCGGAAGGTTTCGAGCGGTCCTGCCGGCAGGGGAGGGATATGGGC

ATGGACGGGAAGACGCTTATCCACCCCAGCACGGTCGCGGTTGCAAACTGGTTCTTCGGC

CCCAACGCCAGCGAGGTGGAGCAAGCTTACAGAGTCGTAGAGGCGCACGAAGACGCGGCG

GCGAGGGGCGAAGGGTGCGCCGTCCTAGACGGCAGCCTGGTAGAGCGCCTTCACGCCGAG

AACGCCCGACGAGTTATCGAGCTGCACGAGTCCATCACCGCGCTAGAAGCGGGCCAGGGG

GGGGCGCAGTAG

>g19148.t1

atggTGTTCGGATTTGGAAGCGACTCGGACgacgaggagaagaagaagaagaagcaacaa

gaggaggaggaggagaagaagaagaagaagcaacaagaggaggaggaggaggcggccaAG

AAGGCCAGGCCTGGCAGGCACGGTCGCAAGAAAAAcgacggcgaggaggaggaggaggag

gaagaggagggggcggagggcggTGGCCGCAGGCCTGGACGCCACGGACGCCACGGCAAG

GCGGACGCCGAAGAAGAGGAGGGCAAGCGCCCCGGCAGGCACGGCCGCAaagacgaggag

gaggccgAAGAGCCCGCTGAGGGCAGGCCCGGCCGCCCCGGCCGCCATGGCAAGGCAGAC

GCCgaagaagccgccgccgccaggccTGGCAGGCACGGCCGCAAGAAACAagacggagag

gaggaggaggaggaggaggctgctGAGGGCGGAGGCAGGCACGGGCGCCACGGTCGCCAC

GGCAAGGATGACGCCGAAGAGGCCGAGGGCGGCCGCCGCCCCGGCAGGCACGGCCGcaaa

gatgaggaggaggaagcagagCCCGCCGAGGGCAGGCCCGGCCGCCCCGGCCGCCATGGG

AAGGCAGACGACGAAGAAACCCCCGCCGGTAGGCCTGGCAGGCACGGCCgcaaggacgag

gaggaggaggagcccgCTGAGGGCGGCGGGAAGCAAGGACGCCCCGGTCGCCACGGCAAG

CCTGATGCCGAGGAGGCGGAGGGTGGGGGTCGCCCTGGCAGGCACGGCCGCAAGGACGAC

GAGGAGGCAGgacacggcggtggcggtggtgaagaCATGCAGGTCGACCCGAACGACCCC

GACGCGGAGCGGAAGAAGATCAACGCGCAGAGGAGGAGGGACGAGGCCGAGCGCGAGAGG

CTGCAGAAGATCGCCGGCAACCAACCTCAGCAACCTCAGCAACctcagcagccccagcag

cctcagcaacaccaccagcaacccCAGCAACCTCAGCAACCTCAGCAGCATGGCGGAGGG

GGTCaccacagcggcggcggtggtggcggcggggggaccGTGACTGTCAAGGCCGGCGAG

TGCATCCAGGATGCGCTCGACAACGCTGGGCCCGGCACGACGATCCTCCTCGAGGCTGGA

ACCTACAACGAGGCTCTCAAGTCTAGGGTGGACGGCACGAAGGAACAGCCAATCACCATC

AAGGGTCCCAGCAGCGGCGACACCGCGATCGTCAAGGGGAGCGACAAGTCGGGTACCTGC

GTCGAAATCAACCACGACTTCTACGTGCTCGATGACTTCACTATCTGCGGGCAGATCAAG

GACGACTGCAACAAGAACAACGTGAAGGAATGCTTCCGCGAGAAGTGCCTCGCCATCAGG

GGCAACCGCGAGCACCGCAACTTGAAGCTGGACAACGGTGACCACTTCCTGTCTTCCGTC

GAGGGCGTGGTGGTGAAGAACATGAAGATCAACAACTGCGGAGGCGAGGGCATCCGGCTG

CGCTACTTCGTGACCCACTGCACCCTCTTCCACAACCGGATCACATCGACCGGTTGCTTC

GACTTCAAGTTCGGCGGAGACCAGGGCATCAAGAACGGAGAGGGCATTTACATCGGAACC

TCGAGCAAGCAGTGGGAGAAGAACATCACGAACGAGCAGGACATCTGCCGTTACAACCGC

GTCTACGAGAACTACATCGACACCAGCgGCAACGAGTGCGTGGACGTGAAGGAGGGCGCG

ATGGACACCATCATCGAGACCAACTACTGCACGGGTCAGCTCGACGAAGACTCGGCGGGC

ATCGACAGCCGTGCCAGCACGACCATCATCCGGTACAACAAGGTCGTCGGCTGCAAGGGC

GCCGGGGTGCGCCTCGGCGGGCACAAGGTCGGCCCTGACCAGTACGGCATCGCCTGCCAG

GTGTACTGCAACATCCTGGAGAACAACGAGTACGGCGGTATCAAGGTCATGACCGAGCCG

CAACAGATTTGCCAGAACACCATCACTGGGCACGGCTCGAAGTACAAGGCGCTCCGTGGA

GAATGCGCCGACAAGTTCGAGGAGTGCAGCGAATGCCCCTTCGACGTTGACCCCCCTTCG

CAAGACATCGAGACCACTAGGTGGTTCCAGAACTGCAAGCCCTGA

>g19305.t1

ATGACGACACGTGGAACCTTCGTGATCTTCGGCGTTGTAAGCGCCATGCGCGTCAGTTCA

GGGTTCCTGCTGGCGGCTGGGTTGACGGTGGCGAGCAGGCAAGCAAGCGCGAGCCCAGCG

TGCGCCGCTCAGGCCAGCGTAACCATGTCTGCGGAGGCGGGAACTGCGAAGCGCGTGCTC

GTGACCGGGGCCGGCGGGCGAACTGGAGGGATCGTGTTTGACAAGCTCTTGAAGAAGGAG

GGCTACGCAACCAGGGGCATGGTTCGAAGTCAGAAGAGCGCGAACAAGCTTAAGAAGAAA

TCGGACGCTGAGGTGCTGGACACGAACGTGTTCATCGCGGACGTGACAACCCCGGGGTCT

CTGGCGGCGGCTATGGAGGGCATGGACGAGGTCGTCCTGTGCACCAGCGCCGTCCCGAAG

ATCTACCCGTTTTCCATCGTAAAGGTGCTCTTCAAGAAGCTCTTGCGCAAGGAGCCGGGA

AGGCCGAAGTTTTATTTCGGGGAGAGGGGAACTCCGGaagagGTGGACTGGATGGGGGCG

AAGGCCCTCATCGACGCGGCAAAGACTGCCGGGGTGAAACACTTCGTGTACGTGGGGTCC

ATGGGCGGCACGCAGCCGGATAACTTCCTGAATACCATCGGGAAGCAAGATGACGGCACC

GGCGGGGACATCTTGCTCTGGAAAAGGAAGGCAGAGGAATACCTCATCGCGTCAGGCCTG

ACCTACACCATCATCCACCCGGGAGGTTTGCTGGATGCGGCAGGCGGCAAGCGGCGCCTA

GTACTGGGCGTGGACGACACCTTGCTCGAGAGAAAGTCGAGGAGCATCCCTCGTGCGGAC

GTCGCTCAGCTTTCTGTGGAGTGCTTGGGCCTGGAGTCCGCGAAAAACCGTTCATTTGAC

GTCGCCAGCGACGCGGAAGGAGAGGGAGAGGTGACCACCGATCTTGGTGCCCTCGTGGAG

GGCTTGACGGGCAACACGGACTACACCAAGGCGCCCCAGCCGTCATCCATGAACTGA

>g19370.t1

ATGCTGTTCGACGCCATGGCCACGGTCTGGGCTGCAGCAGCAATCTTCCTCGTCCCAGGA

GCATTGGCAGGGCCATGCCCAAATGGCACAGTTTCAGTGAATCTGACTTCGAGCGGCGGC

ATGCAGGGTTTGATCGACGCGATGGATTGCAGTGGTGCAGGCGTTTATGACGTCACCGTG

CACGGCCGCCTGCAGATAGGGGAGATACTTAAGGTGTCCGAACGGAAGGAGGTAACTGCT

ACTGGTTATGCTGGCACCGCGACGGGCAGCGCCAGTGATACAAACTCTGCACTGGGCAGC

ACCAGTGATGCGTACGCCGTACTGGATGCTGGAAATACAACCGGCATATTCTTGGTGTCG

GAAGGTTCTACCCTGACCATCAACAATTTGATACTCGAGGGGGGCTATTCAGGGGATGGT

GCGGCAATCGCGGTCACTTCTTCCAGCACCTTGAAGGTGTTTGACTGCGATTTCACGAAC

AACACGGCTTCAATGTCCGGAGGTGCCATTTCTGCAGATGACCACTCGAACATCTACATC

GATGGCCACACATACTTTGGCGACAACTCGGCATATTATGGGGGTGCCATTTCTGCAAGT

CATTCACGTGTCTACGTCAACGGCTCAACAACGTTCAGGCGAAACTTGGCTGATTCCGGG

GGTGCCATATTTGCCAACAACTCGGATACCTACATCAACGGCAACACAGAGTGTGACAGA

ACCTTCCCCCACGGCTACGGCGGAATACACGCTGTGGGGGGTAGCATTCTTTGTTGTGCG

GGGGCTGGGGGCACCGTGTTCCTCGAGAATGAAGCATTGGACGACGGAGGGGCCCTTTCC

CTCACGTCACCCACCAAAGTTAACGTAGCAAGCGTAATCTTCATTTCGAACACCGCGTAC

CGTGGGGGTGCCCTCTACACGATCTCCACCAAATTTGGCGTTGAGGCGGTGTACCAGAGC

TGCGTGTTTTCGAACAACTACGCCACAGACGGGGGAGGCGCCTATTTCTATGGCGACCCG

GCGTTTTACCTCGTCAATGCttccatttttttcgaaaaccaTGCAGGTACTTCGGGTGGG

GCAGTCTACCTCGCTGGGAGCCTGAACCTGTTTGGGTCTGAGTTCTACGACAACAGAGCC

GGTAGAGACGGTGTCGCTATTTCAAGCGGTTGCGCATCGTCGTTGGTGGAGTTTGAGGAT

GTCACTTTTCAGGACAACGGTATGCTCTGCCCGCTAGGGCAGTATGAGCCTGTGGAATTG

GACTCGGATGCCGCAGCTTTCGCCAAAGTGTGTGAAGGTTGCTCGTCCGATGAAAGAACG

GTCCCTGTCGGTGTTCTGGACACACAGTGGCAGCTTCCCTCTTGCGAGGAGCTACCGACG

GGCACCGTTGCCGTGACCGACGGCACTACAAGTTGGACTCTTACCCTGGAAGAAGGGTAT

TATCGCGACTCCGCTACGAGTGACGATATCCTGGAGTGCTACAGACATGAAGCCTGCGTA

GGAGGCGACGACGTTGAGAACTACTGCGCCGACGGCTACGAGGGTGCATATTGTTCGGTC

TGCTCGATAGGATATTCATCCGGGATCGCTCACAGCTGCAGAAAATGTTCTGGGTACGAG

AATTCATCGGCGGCGGGAACTACGATCGCCGTGGTCATAGGTGTTTGTCTGGCGGCAGCT

GAGATTTTGAGGCGCTTGGGGAATGTGGCAGAACACGAGGAAAGGGAACGTCTCGATACA

CCTCAAGACTTCGTCAAGCGATCGTGTACTCGTATCCAGAACTTAATATTGAAGGCGCTC

CCGCGGACGGCCATCAAGATCGTCGTTGTTGTGTGGCAAATCATTTCTCAGTTCGCTGCT

GTTGCCGAAGATGTGTATCCAGAGGAGTACGAAATGCTCGTATCGGACCTCAGTTTCGTC

AACCTTGACCTCAGCCTCATCACGTCCTCCTTCTGCCTACTTTCTACAAATTTCCACGGT

CGACTCCTGCTGGCCACCATCCTACCGCTGTTGGTGCTCGTGATATTGGCGCTCGCGTAC

CATGTCGCCAAAGGCAAATACCCCATCTCCTCACAGCAACGTCTGACAGTGTGGAGCAGG

CACCTCGTCAATAATATCGCGGTCATCCTCACGCTCACAGGTGGGTTTATGTTCGTTTCG

GAGTCACTGTCCCCCTTTGAAAATTACTGGGATATGGCCCTGTATCGAGGAGGTAACGCG

GTTGTCATGTCTAGCATGTTCGTTGCCCTCCTTAATACAGCTAACGCGTCCGAGGATGAG

TCGCTATCAGTGCTCGGCTGGGTGTTAATCCTCGTGAATGTCGTGATGATCGTTGCAGTA

GTGGCCGAAGCTGTTGCGCTGGTTTGGAAATTTCGTAGCCAACTCGAAAGTGATGCAGAG

TAG

>g19409.t1

ATGTCATCCACACTTTGGACGATCGCCCTGCTGACTGCCTCCATTTCCCCGTCCTGCCAC

GGCTTCGTGGCAACCCCGCCACCGACTTCCTCCACATCAAGGAGATCCGCACAAAACTTT

CGACAAGGCGGCGCAAAATGCACCCCCCCTCATGTTGCACCAAACGTGGCTGCCGCAAGT

AGACAGCGTCTACCCGGCCATGCATCGTCGCTTCAGCAGAACACAGGCAGGAAACCCTTC

AAGCAGTACTCTGTAGGCGAAGATGTGGGAATTTCTGCGGCTACGGAGGGTTCAGTGGCG

GCGTCCAGCGTGTGGGCTGAAGAAAAGTTTCCCGGCTGCTCTACCGTCGTCTCACCGGCG

ACGAGACTGTCGGGTCTGCTTGCTGAGGTGTGGGAGACGCTGGCCGACCCTTCCGCCGTG

CAGGAAGGCAAACCGCGCGTGTTGGCATTCCCAGACTGCGCCGCGGTCGCCAGCCCCCGA

GGCCTCCAAAACTTGTACGAGCACCTGGAGGTGTGCCAGGACGCCTGCGAACAGTTCGGG

ACGGCGGTCTCCATCGTCCCCCACCCCAAAGGCGGAAGCTCTGGCGCGCCCGCTCCTGCA

CTTGTGGTGCGAGTCATTGccggtgccggggggggggacgacttCGATTACGACCCCGAC

TGGGATGGGGACTGGGACATCGACCGCTCTCTCCtggacgatgacgatgacgagggGGGT

ATGGACTTGGGCGGGGGGTGGGACGGGGTGCAGGTGGACGCGAAGGCGCTAAGCGTCGTC

CCCGCTTCGGATGAGGAGGTGACGGAGCTGACCAAGGATTGGGTGCAGGCGGTGATCACG

GGCATGGGGGTGTGCCCCTTCAGCGTGGACGCCTCCAGGGCCGGCCTCCCGATAGGACAG

GTCCGCTACCCCGTCACGAGGGAGACCACGGCGGAGTCCATCTACAGGGAATACTGGCGC

GAGGTGGAGCTGCTTGTGTCGGAGACGGACGAGCGGTCCCTCAGCACGACCCTCCTCATC

ACACCCGAGTTCTCGCTCAGCAACGCGGAGGCTTTCGAGGTCATCGGCCAGACGCTGACC

CAGCCGCTGGAAGCGCTCCACTTGGAGGACGACATCCAGCTGGTCTTCTTCCACCCCCAG

TACGCCTTCAGGGATGGACGAGACCGCATTGGAAGCGGGGACGCTGCGAACTTCGCCCGC

AGGTCTCCTTTCCCGATGATCAACATCCTCAGGACTAACCAGGTTCGCCTGGCCCAAAAG

TCTATCCCCACGGGATTGGTCTACCAGCAGAACGAGGAGGTGCTGACCGAAGTCGGAGCC

GGCGAGCTGCAGAAAATGCTGGAGCAGAGAGACTGGGCGGGGCTAGAGGGCAAGAAGGTC

GACCGCAAGCGCATCGACTACTATGAAACGGCGCGGAAGCTGCAAGGCATGGACGGACAG

ccagcggctgcggcggcggcggcgaccgtcggaggaggaggagctgtcGTCGACCAAGGA

AACGCCGATTTTTCGGCCGTGGCGAGGAGCGGAGCGCGAGAATCGCTGGCGACAGCGGCG

GGCTCTATGTCCACCTCCTTCGACCAAGGCTTCTCCGGGAAGGAGGAGTCGAATCCCGCA

GCACCCGGGACACCGAGTGCCTCAAGTATTCCTCAAGCGGTGGGGGCTGCTCCGGCGGCG

GCTGGGGACGGTAGGCGTGTCTTGGAAGCGGGGGAGGGGTCGGCGGCCCCTGTGGCGAGC

TCTTCTGCCGGGATTGCGAACGAGGATCTGAGGGACGTGGCTAGTATGCTCGTGAAGAGG

CTGTCGAGCGGGGTGCCCCTGACACCGGGCGGGTTTAGTGGGTTTAGCGACGCGGTGGAC

CGCATTATAGAGAGCATGTCGGCCGAAGGCGGGCAATCGACGGTTACCATCACCAAGGCG

CCAGCGCCAGGAGAATCTACGGGCTACTCCACGCAGGCGTGGTTCGTCGCCGCGAGCACC

CGCTGCCGCACGTACGCAGATGCGCCTATTTTGCCACCGGTGGCCAAGATGCCACCTGTG

GCCAAGGTGCCGCCGGTGGCCAAAAAGGCGGTGTCCAAGCACGAGGAAGACAACGCCACG

GTGGGCAGCTGGCTTCGATTCACGGACCGTGAGATGACGCAGCTGAAACGAAAAGCTCCC

TCCGTTCGGAAGGACGAGCGCAGGCAGGAGATGCTTGCAAGGAGACTGGACGACGAGACG

GACCAAGACCCGGGTGTCATAGCCGTGCTGCGGGGGCCGCGGCTCGGCATGACGGCCGAG

GAATGCCGGAAGGTCGTGCTGAAGCACCCTTGCCTGCTCACCATGAGCGGGGAGGGGCCC

TCCGAAAGGCTGGCGTGGATGATAACCAACCTTGCGGTGAGCCAGGACCAGCTACGCGCG

ATAGTCCTGAAGCAGCCCAGGCTGCTGTCGAACAACATCGGGGTGAACATGGCGCCGGCG

GTGCATTTCCTGACCGAGGAGCTGGGACTGTCGATCGAGATGGTTGCGGCCATCATACGA

AAGTTCCCGGAGgtCTTGAATCTGAGCGTTGAACGGAAGCTCGCCGTGAAGGTTCGGTGG

TTCGAGGAGACCCTGGGTATGGACAAGCCGGACATCGCGAGGATGGTCTTCACCCTGCCC

GCCCTCTTCGGCTACAGCATCGAAGACAACATGGAGCCTAAGATCGCCTGGCTGCAGGAG

GCCTTCGGAACGTCCCACCAAATCGCCCTTCAGATGATGGTCAAGTGGCCAGGCCTCTTC

ACGTGCAACGTCGAGCGCAACCTCAAACCCACCCTGGAGTTCTTCCTCGAAGACCTCCag

gggggaaaggaggaggTGCGCGACATTGTCACCAGCAACCCTCGATTGCTGGGTGCGAGC

ATCGAGAAGCGGCTGCGGCCTCGCGTCGCCAGGATGCTGAGCCGAGGAGTGGTGGCCACC

TTTTCGGAGCATCGGTGGGTGCTGGCCATCCGGACCGACGCGTTTTTCGACCAGTGGGTG

GAGCAGTTGGAGCCTTcgcagcaggcggcggcggaggaggaggagcaggaggaggacgag

gccgGTTGTcggtcttctgctgctgctggtggtggtggcggtggctctactggtggtggt

agtgccgCTACGGCAAGGAGGGTAGTAAGAGCGGCAGACGTCCTCGTTCCCGCTGCTATG

GAATGA

>g19461.t1

ATGTTGGCGAGAAGGACCGAGGAGGTGGTAGCGCTAGAGATTGGCCATAGGTCTCGAAGA

GGACCCACATCCGGTGACGGCGATAAGGCGTCGGTTTGGGTTGAGGGCTTACGTCTCGTG

ATGCGCTCTCTTGCCATATGCGGCGCTTCTTCCCTGCTCTTTGCTGGCGCTCGCGCTTTC

ACGGTCTCTCCGACAGgggTCTCTCGCCTGTTGTCTGTCTCTAACGCGCACCGCGACGCC

AGAGGCTCGTTGTCCTCGGTTCCTTCCTTGTCCGCTAGAGGCCGGCAACGCCGACTGAGA

ATGGAAGCCTCCAGTGGCAGCggtggagaagaaaaaaaaagtgATGGAGGACACAGCCCC

CGTAAGCCGACTGGGCACGGGGCGGGCCCTGCGAAGGTCAGGTCTTCAGAAGATGAGGCG

TTGGCCGCGCGGATACAGGAGCACCAGCAAGCGGCGCCTCGTCTTTCGCACGCCGAGGAG

GTGCGAAACCTTATGGAGTACAGCACGGGCTTCGGCGTCCTCAGCACTAACTCTAGGACG

CTCGAAGGGTACCCCGGCGGCTCCGTCGTGGGATTTTCCCTTGATGACAAAGGCCGGCCT

CTATTCGCGTTCAGCTCCATGAGCTCGCACACGGGGGACCTAGCCGCCGACTCGCGCGTC

AGCCTGACTGTCACGTCGGCCACGTTCAAGGGGGCGGCTGACGGTCGTGTGAGCCTGGTC

GGAGACGTAAACAAGGTACCGGAAGAAGACCTTCCCAGCGTTCGCGAGATGTACAAGAAG

AAGCACCCCAACGCGTACTGGGTGGACTTTGGCGATTTCAGTCTGATGCGCATGGACACC

ATCAAGGCGATGCGCTTCGTCGGAGGCTTCGCGATGGCGGGGGACATTCAGCCTGACGAG

TATCTGTCCACATCGCCCGACGCCGTGGCGCAGTTTTCAGCCCCTATCCTGCAGCACATG

AACGACGACCACTCGGAAACGACCAAGGCCATGATCGAGCACTACGTCACGGGCGGGGTA

GAGATAGCATCGGCCCAGATTACTGCTGTGGACAGGCTTGGCATGTACGTGCTCGTTGGC

ATGAACGACGGCCAGTCGGGCAAGCTCAGACTCCCCTTCCCCCGGCCGGCCGAAGACCGT

AAGGATGTGAAAACCCTCATCGTCGAGATGACGAACGTGGCTATGGCTTAG

>g19734.t1

ATGGCAACCTCTGGCCCGCAAGTTAGGGGCACAGGCCCAAGCCCAAGACAGAGGCGCCAG

CAGGCCCTAACGCCCTCCCTACCGTCATCATGCGGCGCGTCGCGCGTACGACGGGCGAAG

TCGAGCTTGGGGGCCGCTTCGGGGCTGGCGGCCGTGGCAATTGTGGGGCTCGCCGGGTTT

CCGCTAGCGAGTGCGTTTTTCACGGCGGCGCCGCGTGTCTCAGGGAGTTGGGCGGCAGGG

GGCGTTTGTgtcggaagggggggcgggacaGGGGTAAAGGCGTGCCGAGCGCGACCGGAT

GGGGTGGCGATGATGAGCTACCAGGGAGGAGATGAAGACGCGAACAAGCCGCAGTTCATA

CGGTCTCCGGAGATGGACACGAGCGTGAAGGAGATTCAGCAGCCTCCCGACAGAGAAGCG

GCGATGAAGATGGTTGCTGCCAGAGAGGCGGAAGAGATTTCGAGGGGAGAGCTCGGCGCC

TACTACTCCGCCGATGTGCCGGACATGCCCAAGCCGCCATACCAATTCCAGGTGCCCGGT

GAGTTCGGAAACGACACCCTTTGCACCGACCCCGCTTGGCACACTGCGCTTGCCGCGCTC

ACGTGGCTCGGATTGCCTTACGAAGTCGAGCACGAAGAGGACGAGGATGAACCCATGCTG

CTCATGGGGGGTGAGGTGTACGACGACCCGATGCAGCTGATCGCCAGCCTGCCCGAGAGA

GTCCTCCTGTCCGACTTTGTGGAATCTATGCACCAGGTTACCGAGGTCGTCTCGATGGTG

GAGCCGGCATGGATGGCCCTGCACTTCAACGAGGGCAAAAACGTGGCCCAGCTGGAGAAG

AAGCTCAACGACATCCTGCAGAAGGTCGAGAACATCTTCGTGAAGAACTATTCCAAGGGG

TGCATGTTGGAAGGCGCAGACCTCACCGTGGGCGACCTTATGCTAGGAGTCACGACGTTC

CACATGGTTACGGCCTTTGGACTGGAGAAGCAGTGGAAAGTGCCCGACGAGCTGGTGAAG

CTGAAAAAGCACATGGCGATGATGCACGGCATGCAGACCTTGACGGCGGTCATGCCCTCG

GAGAGCGATTTGGCGCAAAAGTACGCGCTCTCAAAATAA

>g20059.t1

ATGCGCCTGTCGTGTACACTCGCCGCCGGCGCGCTGGCCGCGGCCGCGATCAACCCAACA

TGCGCCTTCCACGTCAGGGGACCTGTCCCAACGACGTCCTCTTCCTTCGCTACCGGGACC

TCGATCAACGTGAGGCACGTGCCTGCTGCGCGCAGCACCAGGCAGCCCGTGCTCGAGGCG

TCCCTGTCCATGCCGGGGATGGACTGGGCCCGCGCACGCGCGGAGCGCGTGAAGTCGGCT

CTCGTCCTTAATTCGGCGGGGGATGCTGCGGTTTCCGTGCCCGGCGGGGCTCCCGTGAAG

GAGAAGACCTCGACCGCGAAGGTCGGCTTCTACCTCTTCGTATGGTACAGCTTGACCATC

GGGTACAACATCTATAACAAGAAGACCCTCAACATGCTCAACATCCCCTGGATTCTGAGC

ACGATCCAGCTCGCCGTGGGTGCTATCTACGTGAGCCTGATCTGGTTGACGGGCATAAGG

AAGGCGCCCAAGCTTACGGGCGAGAACCTCAAGGCCGTGGCGCCTCTCGCGCTGCTGCAC

ACGACCTCCCACATCGCTGCCGTGGTCGGACTCTCGGCCGGAGCCATCGGCTTCGTCCAG

ATCGTTAAGGCCGGTGAGCCGCTGTTCACTGCGCTCTTCAGTGCCCTTTTCCTGGGCCAA

ATCTTCACCCTGCCCGTGTACGCGGCTCTCTTGCCCGTTGTCGGTGGTGTGGCCATCGCC

TCCCTCAAGGAACTCTCCTTCACGTGGCTGGCGTTCGGTGGCGCTATGATCTCCAACGTG

GCGGCTGCCTCGCGGGGTGTGCTGGCCAAGGCGTCGATGGACCAGCCGAAGGGCGAGAAC

ATGGACGCCGGTAACCTGTACGGCGTGATGACCATCATGGCCACCCTCATGCTGGCGCCG

TTCGCCGCGGCGGTGGAAGGCCCCCAACTGAAGGCCCTCTACGACGCGTCCATGGCCGCG

GGGAACACGAAGGCCGCGCTGATCAAGGGCAGCTTGTTGAGCGGGCTCTTCTTCTACATG

TACAACGAGGTGGCCTTCTACTGCCTGAACGCCATCCACCCCGTGACGCACGCCGTCGCC

AACACCGTGAAGCGCGTGTTCCTCATCGGCGTGAGCATCCTCGTGTTCGGCCACAAGCTG

ACCCCCCTCGGCAGCATAGGCTCCGCTGTGGCCATCGGCGGTGTGCTTCTGTACTCGCTC

GCGAAGCAGAAGTTCCCCAACAAAAAGTAA

>g20061.t1

ATGTGCGATGATCCCCACATGCAAGGCCTCCGCGGACAGAAGATCGATTGGTCTGGAGTC

GACGGTGGCTGGTACAGCATGATCAGGGATGACGACGTTCACCTCCAGCTCAACGTCCGT

TTGACCGCCCCTCTGCCCGAGGACTTTCCCGACCGCCAGCTGATCACGGGCGTCAGCGTT

TTGTCGGAGGGGCACACTCTCGTTATCGAGGTGAAGAACCCTAACGATATCAACACAGGC

GGCTGTCCCGATGGGATGTCGCCCTGCCTTTCCGATGGCGGACTGCTCGTTGTTGCCGAT

GGGCAGGAGGTAAAAGGCCTGCTCGGTTTTTCCAGGGACGAGTATGTGGTGGATGGCATC

ACTTTGTCGGCGGCCAACCTACCGGCAGAGTGCCGTCAGTTTGGTGGAGACAAGATTTGG

GCCCGCATGTACAACGAAATGCTGCAGGGCACCCGCCAGCTGACCCTCGAGGAACGCGTT

GAGGATTGGATCCTACGTTTCACGGACATGGCAGCACCCAGCTGGTGCGCGCAGTACATC

GAGAAGCACGACCTGGCCGATTTGCAGTCCATTCATTCTGTATTCAAAATCGTGACACCA

GCCGTCACTGTGCGCCTGAACGTCGGCACGAGCTCCCAAGGCGATGGGGAGTTGGACTGG

GATGGCCGCGTCTTGCCCGACTTAGAGTTCTGGCAAATGGACGTGGGAATTCACGGCCTG

TCGCTCGAGAATCAGTCGCTGTCCGGAATCCTGGGTGAGACGGCCCGCCCGGTGCTGGAT

GAGGATGGtcgcgaggtgatggaagggTACGAGGCGTTCCGTGGCACAGTGGAGGATTAC

CGCGTGTCCGGTCCTTTGGCCACCGACTTCGCCCTTTTAAACAAGGTGTAG

>g20091.t1

ATGGCAAGGATTGACGTCGTGGATGTGGACGATGCAGGCGGCTCTgACCGTCCTGCGGTA

GGAGCATCGCTGCGCAACTCCCGTGCCTCTGACAACTCCGGCGTCATGGTGTCATCCTCC

AGCAACAGCACGAATGCATCGCCGTTTGGCTCTAGTGGATGCGAGTTCGACCTTGGGAGC

CCGGGAAGCATTTCGCGCCCCCAGACCCAGCCAACCGCAGATGCCCGAGGTTCCGACGCC

CGGCAACAGTTTCCGCCGATGCAGTCAACCGAGGCGCGTACGCccgagaaggaggaagag

gagaagagtgCCGAGACCTTGGTTGGGGCAGCGGCGGCTCCGGTACTAGTTCAAGTCTCA

GGAGAGCAATCAGCACCACCTTTATCCCCCGCCGAGTTGACGATCGGTATCGATTGGCCA

TTGACATCAACCTCCTTGGGCACAGCGATGACCACCGAGCAAGCAGCGGCGTCAGTTGcc

gtggtgggggtggaggcatCGGCTTCTCTTCTGAATTCCCCGCCGCATCAAGAGAAGGAC

CCACAGAATAACCCAGTGATGGGGGCGCGGGTGGTTGGGGCGTTGGCACATTTGAGGGCC

GAAGAGGGTTATCAAAGGGGACACAGACCGATTTTCGAATTGCCGCAACCCGAGCCCCAG

TCCAATCCGGAGCCGCCAACGCCGGTGCGAGAGCCTGCTGAAGCCCGTACATCAAACGAG

GCCCAACCGTCACGACTTTTGCCGACGGCTGAGGACGGTGCGGAAGCGAAGGGGCAAGAT

TGTAGTTCTACCACCAATGTGACCGTCGCCATGGGAATGGGGGCGAGAGCAGAGAGGCAG

GTGAGCCAACCGATCGGAGGGACCGTGACTGTAAACGGCGAAGCAAAGGAGGACGCATGG

CGTGAAAGGAGGGCTATCTCTGAGGCACCTCCCAGCTCAATAAATAACGCCTACCTAGCG

AAGGCGGCAGCAAAGGAACGGGGGAAGCAACCAACGACGACGGCTGCGACGACAAAAGCA

CAACCAAAGGTGGAAGAATTGTTCCATAAATGGAGGTCTAACTCCGAGGCGCCCCAGGCA

AACCCTACAAGATCGGTGATGGGCGTTGTTGATCGcctggcggcggtagtggcggagGCA

AAGGAAAAACGAAAGAAGAATCGCGATGCGTCCGGTGTCGGAAACATCTCGGCCAGCGCC

GCCGGCAGACGAATGGGCCCCCGCGAAGGTAGCGTTCGACGGGGGTTGGTGTCAAGACTG

TCGATCGGAAGACGGGGACCATTTCAACCAGATCAACCTACAGGGGAAAAGGACGTGGAA

GTGAAGATGCCGATTGATGTGGAGGCAGAAAATGAGGTCTGGGAGCGCGTGATGCAGAGA

CATAATGAGCTAGAGGCcgaagagaaggggggggacTCTCGGGTCGCCATGCCAGTGAAA

GGCATGAAGACCCTTGTAGAGGCGGTCTACAGCGGCGATCCCGGCGCGGTGGGAGCGGTG

GTGGGCATCCTCGGGCATAGTCAGGCGGCCAAACAGCTATCGGCCTTGCTCATTAACGAC

AGCTACCAGCTGGAGACCGTCGTCATGTCGGCGGCCCGTGGGGGAGACATAAAGATGTTC

CACGCCGTTCTTCGATCCCTCCGGCAGACGCTGACCGAGTCGCAGATCTCTGATGTGCTC

AAGGCCAGGGGGGGCGACGGCTGTTCGCTGTTGACGGCGGCAGCCGAGGGAGGAAATGAG

ACAGTTTTTGTGGATGCCTGTGTCCTAATGGGGGGAAAGGACACTGTTTGGGGGGAGCTC

TCAGAGGGCGAAGCTCGCTGTCTCATGAAGATGGCGGCCAGAAGCGGGAACTTGGGAATG

TTTAGCAAGGCCAAAGAGGCGGTGCTGTCGATGGGCAAGCGGGCCGGCTCGCTTGCGCTG

GACTCCAGTGACGACCCGAACCAAACCAGCATCGTGGTTGACGCGGCGAGGAGCGGGAGT

GGGGATATGATCGATGCGGTTGTTGCCGTCATGTCCGAACTAATGCCAGAACAGAAGGTG

GTTACCGAAGTCATGTCGTCCGCCGGCCCGCGCCGAATGTCGTTGCTTATGGTGGCGGCC

GAGACAGGGAGTGTGGAAGCAGTGGCTGCCGTCATGAAGGCCTTGCTAACTATCGTCGCT

GGTGCCAAGATCATTATGTTGGACGTGTACAGCGCCACCCTGGCCGGGTGCTGCGCCAAC

CTTTCCATGCCGGTGGAAAGCAAGCTTCTTGAGGTGGTTTCGTACTTGCTACAGCACGGC

GCGAAGCCTGGAGCGCTTGACCTCACCCGGCTCTCGACCAGCGTCTCGTTCCCCCGCTTG

AAGGCGAGTCTGCTGGGAGCGGTGTCTTCAGCGAACAACCCGTTCATTCCAGGCATGAAT

CTCTCCGTCGGCCTTGCTGTGGCTGCAAAAATAGCCGTTGAGGGGGAAAAGCGCGCTCTG

CTTTCGCTGCAGGCTGCGGTGGACGAGCTACTGGGTGAGGTCCTTGAGAACCTGCCGCAG

ACCGTGAGGGGTTTTGAAGGACAAATGAAGGCGTGTTCAGCGGTGTTCGAGCCTGAAACC

gtcatatttcgtcacaagggcACGGCGGGACCCCTAAGCGTGGCCCTCCAAAAGCGCCAA

CAGATGAATACCTACTGCACGATCCCCTTGGTGCTGGATTTCATGTGGCGGAAGTTCACG

AAGGGCCTTCCCAGCCTCAGGGACACGGAGGGGGTGTTAGATAATGACAAAGAGTTCTCT

AACCCCGGGCAGACCCTATACGgtgaGGATATGCTCGCCGACAGCATGGCGTTGCAGAGC

GGCTACGGGGCCGGCAAACTATCGAACTTCAGCTACTTCCCGGGGGCACGGTTCGTCATC

GTCGGACTCCTGTCGCGACCCGACAGCTACTACAAAGTGCCCGCTCTGCGGATGGTGCTA

GATTTGGTTACATACCTGTGCATGATGAGCTTTTTTGCGGGCTTCGTTCTTTTGAACGAC

GAGGAGGAGTTCGACTGGACGGAGATCATCTTCGCTTTCTACGTCGTTGGGGCAATCGTG

TCAGAGCTCCTAGACGTTGCTGAGAACGCACGAGAGTACTTCCTCGACCGATGGAACGTT

CTGGACGTGGGTTGCTTATGCCTCATGTTCTTCGGGCTCTGTGTTCGAGCTTTTGATCCC

GCCGACTCGATCTTGGCAAGATCAATGTACGCTCTGAGCGCACCGCTGGCGTTCACAAGG

ATCCTGTTCTTCGCCCAGATACTCCCTTCCCAAGGACCCATGATCCAGGTTTTATTTTCA

ATGACGGGTGAGCTCGTTAAGTTCGGCGTGGTCATGCTTGTGGTGATGGGTGGCTTCGTG

GTGTCCTTCCACAGCATATTCCAGCACAACGTAACCTTCGGCCAGGTTTGGCTGAACGCG

GTCAAGGCCATGCTTGGAGAAACCCAGTTTTTCGATGACTTTTCGGGAAGTGTGTTTGAC

ACCATAGCGACGGTACTTCTGGTGGCGTACCTGCTGATCATGACCATCATGATGCTCAAC

CTTCTGATCGCGGTGCTGAGCACAGCTCACGCCAGGATTGATATGAACACCGATCAAGAG

TACAAGATTTCAAAGGCACGGATGATCCAGCACTACGTGTACGTCGCCAAGATCGACCGC

CTTCCTTCGCCCTTCAACCTCCTGCAGTCGGTGTTCAGCTTTCCGACGTTGATAGTCGAC

TGGTGTTTCCACACCACGATCCACGCTGTCACCAAACGCTTCGTTGGACGCGCCGTATTC

TGGGTGATGCTTGGTCCCATCGCGGTAGTAACAGGGTGGCTGCTATGGATATCGTCGCTG

CCCAAGACGGTCACGGTGGTTTGGCGAAATAACGCCGGAAAAGGGATGTTTGTAAAGATC

GCATTGGTATCCTTGGCGACGTTAATCAACACGATTATTGCCCCTTTCTGGCTACTCGTT

CTGTGGGTCAAAGGCGGTTTCGCGGGGATGCGGAGAGTGATCGTTCGAATGAGGAATGGG

GGCAAGCGCGTGTGCTGTCGCGGAGCGGATAGGGTAAAGACGGCGCGCGCGTCACGAGAG

TCGTCTCTGCAGGACCTCGATGGAGGCGCCACCGGACATTTGGAAGATGTACTCGAAAAC

GTCGTGAACGTCACGCTAAACAAGACGGAGGGGGGCCTCGGGGTTGGCGAGCTGcagaaa

tatctcgacgaccCTATGAGTGATCCACAGGTGCGACGTGATGAACAAACGCGAGCCACG

ACAGTGGAGCATGTGAAGTTACTGAGAAACAGGCTGGAGGCGACGACAACGGAGCACATC

GAGGACCTACAGTCCTACCTCGTCCGAGCGGCAGAGGACCTTTCCTGGCAAGTTGGTCAC

GCAGCGGCGGTCACGGGGGGTGACAGTGGCAGAATATCGGGGAGTGGGTCACAACCGAGC

GATCTAAAACGCAAGTTCAACGCCAGGTTCAGCGAGCTTGAAAGAGAACTAGACGGGAGG

ACGGCCAAGGTGGAGGAGAGGATAGGGGCGATGATCGATAAGCGATTGGGCGTTTTAGCA

CATAAGCTGGAAACGGTCCTCAGGACTGCGGATGTCTGGATAATCGTCGGCGCACGCTTC

GTCATGCGAGTTCGGCGTCGAAAGCTCGTTCGCCGGGCACGTGGGCAGGGGAGGGTTCTG

ATCCCAGCTCCCGGGGGGCGCGCTGCGAAAATGGCTACCGCGATgacgtctgctgctgcg

gctgtgctTGCTCTGACGGCACCGTCGGcaacggccgcggcggcggcggcactggtgctg

gcggcggtagcgatagcgctggtggcggtggcggcggcagctcgAACCATGATCCCGCCC

CTTCCAGGGGTTCCGGCTCCCCCAGTGACGGTCGGCGCTGGGGGTCCGTCTCCCCGCGCC

GCAGGCAACAGCTCAATGAACGCCTCCCCCAGTTGCTCCCAACCGACATCTCGAGAGTAC

GATCTGCGGCCGGACGGAAGCAGGATCTTGCAGCAGTCCGACCCgtggttgctgccgctg

ttcaaATAA

>g20122.t1

ATGGCTTCTCGCTCTCTCACCGTGCTGCTTGCATCGTTCCTCGCATCGAGCGCTGCTTTT

GTCCATGTTGGCATCTTTCCCAGGAGCAGCAGCTCCGGGCCTCTAAGCCTGAAGAGTGGC

AGGCATCACTCCTGCCGATCGCCGATCAGACTGCCCGTCAGATGCGCGGCGGAAGGCGTG

GCGGATGGGGAGAGCGAGTTTGACAAGGAGGAGGAGATTGTTGACGAGAGCCTTGAGGGG

GTGGCGAAGATGCGGGCGAGCGAGATAAAGGCGGAGCTGGAGATGCGAGGGGTTAGCTAC

GAAGGCATCTTCGAAAAGACCGAGCTGGTGGAAAAGCTGATAGAGGCGCGGAGCTTAGGG

CGAGCTAACCCTTCCCTCATCGACGACTTCAACAAGGAGAACCTCGAGCGAAAGATGGAC

CCCGAGAGGGCGGCGGCGTTTGCGGACGACCTGCTGGACGAGGACAAGCTCAAGGACCTC

ACAGCAGCCGACGGCACTCTCCCCGGAGGAATGACTCCGGAGATGCTGTCCAAGCTCGTC

TCGAACCCCGAGCTTATGGTGTTGATGCAGAACTCTAAGTTGCAAGAGGTCATGAAGAAG

GTCATGGCCGGCGGACCTGAGGCTATGGAAAGTCTCCAGAAAGACCCGGAGACAGCGGAG

TTGTTGCAAAAGCTCGAAAAGGCAATGGAATCCATgaagtaa

>g20623.t1

ATGTGGCACGAACGCTGGGTTGACGCCGTgcaagcgacagcaacagcaaacatcATCAAC

AGCTGCACCGAAGTGGCGTCAGCTTTGACGGCGGGCGCGGTGGTGGTCGGGCCGAACCTC

CAAGTAGGACTGCATTTTTCTGCCCTGCAGAGCAGCGCAGCGAAACGGCAGCAGAGCGGT

CGGCGAGagcaggcagcagcatcaaGGAGGCGAGAAGGggcaggaggagaggaggaggag

gaggcaggaggaggggggggcgaggaggcggaggaggcgggaggaggagaaggggggcgg

gggacggcagcagcagcagaagcagaagcagcgctAGCACCACCTTTCGCGACGAGCTTA

AGACGAGGTACTGCCTCCCGCGAGTCCGCTTCGGTTTTCCACACCGCGGGTACGGTCGGG

GATTACCGGCATGTAattgaggcggcggcggtggcggcggggggcggggggggggcgggg

ggcggttcAAGCGGCGACGCGTTGGGTTTCCAGAAAAATCAGCGTGTTTTTTCGTCGGTG

CCTACCGACGGCGCtgaccagcagaagcagcagcagcagaaacagcagcagcagcagaaa

cagcagcgatCGTCGTGGGACGATGAGGAGCTGCTCAAGTCGCTGGCGGCGAAAAAGGCC

ACCCCGCTGTCTCTGAGGGATATACACGCGTTCTGCCAGTCGACGAGCATGTCCTCGAGG

ATAGCGCAGGCGCGGTTCTTGCATCGAGAGGTCCCCATTCGGTTCGCCAAGAGAGCGCTG

GAGCTCCGGCATCTACCCTACGGCCTGTCCGAAACGGCACCGGTTATCGAGGCGGCTGGG

TGGTACGCTCGCATCATGCGGCAGCTGGTGGAGTACCCCGTGCCGGAGGGGAGCGACGGG

GACGAGAATTTCTCGGGGTTTTTATCGTCGCTGCTGATGAACCACACGTCCGTGCCGCAG

GCGCTGAGTCGCGGTGTGCTGGAGCTGCGGCAGAGGGGGAAGGTCGGCGTCATCCAGCGG

CACCGCATCGACCGCATCCTCGACAACTTCTTCATCTCTCGGATCAGCCTCCGCTTCCTG

TTGGACACGTACATCAGTAGCAAGAACAACAAGCCGGGGTTCTCCGGGATTATCCAGTCG

AAGTGCTCGCCTGTGCTGGtcgcctggacggccgccgctGACGTCGACCGCCTATGCCGG

TTCCACATGGGCACAGCCCCCCCGATCGAGGTGTTCGGTCGAGAGGAGGACACCTTTACC

GCCGTGCCAAGCCACCTCTACTACATGATGAGGGAGGTGCTCAAGAACAGCTGCAGAGCC

ACGGTAGAGCACGGTCGCCGTACGAGGCCGGGCGAGAAGCTGCCCCCGGTGAAgATAATC

GTTGCTCGCGGCAAGGAGGACATGACCATCAAGATTGTCGACGAGGGCGGCGGCATTCGT

CGCTCGGACCTGCAGCACGTCTGGTCCTACATGTACTCCACCGCGCCGACACCAACCGCC

GCTCAGCTGAATGGAGTGCCACTGGACCACATGTCGGGCATGAGGCTGAAGGAAGAAGCG

TCGCTGCTGAGTGCCCCCAAGGCCGACCGAGGGACGACCTTTGCGTTCGCCGGGTACGGG

ATGGGCCTGCCCCTGGGCCGGCTCTACGCTAGGTACTTCGGCGGCAGCCTCAAGCTGAGG

CCCATGGAAGGCTACGGGACGGACGCGTACATACACCTGCACAGGCTGAAGACGAACTCT

GAGGAGCTCCTTCCCATCTCCCTGGAGGAGACGCTGCGCGACATGGCGGACGATGGACGC

GTAAACGCCAATTCTTCAAGGTCCTGGAACACTCCCTCAAACGACGATGCCGAGGCGAGA

AATATCATCCGAAACCTTGGCAGGGGGCTGTAA

>g20939.t1

ATGAAGTCTGCCGTCGCTGCTCTCGCCCTTGGTGCCGTCGGCGCAAATGCCTTCGTTACC

CCCCACGCCGTCGTCAGCCGCGTCTCAACTCAGTCTGCACGGCAGCTGTCGATGGCGGCG

GACCCGGAAGCGTCCATCGCGCTCCCGTTCACCCCGCGGCCCGCCATGCTGACTGGCGAG

CTCGCGGGCGACGTCGGATTCGACCCCTTGAACTTCTCTGAGGAGGGTGACCTCAAGAAG

TACCGTCGGGCGGAGCTCAAGCACGGCCGCGTTGCCATGCTCGGTGTGGTGGGCGCCATC

TGGCAAGAGTACAGCGTGCTGCCCGGCCTCGGCTACACGCCGACGAAGAACTTGTTCCAG

GCCGTCGCGGACGCACCGTGGCTCGCAATCCTTCAGATCGTCGTCTTCGTGGGCATCTTC

GACCTCCAGAGCACGAAGTACGACATTGAGCAGGGCCGCGTGCCCGGCGACATCGGGTTC

GACCCCCTCAAGCTTTCCAAGGACGGGATCAACGAGAAGTGGGCGCTTTCGGAGCTCAAG

CACGGACGCCTGGCGATGTGGGCGATGGCGGCAATCCTCGTGCAGCAGCTGCTTGTGCCG

GACCAGTCTCCCCTAGAGCAGGCCTACGAGTGGTCTACCCAGTTCCACTGA

>g20989.t1

ATGGCCGCCGCGGGTAAGACGATCACTCTCGCTctcgccctcctccccttccgggGCGCG

TGGGGCGCGTTGCCCGCCTTCCCGCTGAGCACCAAGGAGGTCAAGATCCGCATGGGCTAC

TACACCGAGGGGGGCAAGTACCTCGGCGGGGTGCCCAAGTACGACGAGTCGGAAGGCCGC

TACCAGTGCTCGGACCTCAACTACGAGCTGGGCACGTACAACACCGCGTCCGGGGGGAAC

GCGACGGTCTGCATGAGCTGGGCCACGTCGAACCCGGACCAGAACAACACCTCGACCGAG

GACCAGCCGGCCGTGGACAGGTGCCTGTGCCAGGAGGCCATCACCTCCAACGCGGAGTAC

TGCGCGGAGTGGAGCTGCGGAATCCAAGACGCGGGCAACGTCCTCACCTGCCCGGGTGGC

GTCACCCCCTGCTCTCTCGACTACGAACCGTCGGAGACGACCGCGATATGCGCCTGCGGG

ACGGCGGAAGAATCCGGGAAATTTTGCGCTGCATGGACGTGTCGAGGGTGGAACTCCGAC

GGGGTTGAAGAATCCGAGGAGTTCCAATGCGTGAGGGATTCGCCTTCTGGGGAGTACTGT

GACGGCTGGACGAGCGTGATCGAGAGGCCTAAGGAGATCCAGGTGTCCACCTGCGACTGC

ATCGGGCAGTGGAACGGCGACAGCGTCTGCACGTACTGGGATTGCAAAGAGCGGTCCATG

CCGGTCTGCTCCCACGCCAACGATCCGCACATGAGCTGGTGCAACATCGGCATCGCGGTG

GGGCTCTGGGGCTTGCTCGGATCCATCGGGGCGTTTTTTGTGGCGTCGGGTCTCATGCGT

CTCGTGGAAGACTCGTGCGTTAACGTGTTTGTTCTTGGAGCCTTCTTGATGGTGGTGTTT

TCCGTGCCGGTCGTGATATGGGGCGGTCAGGATGCCGCCGTGTACGTTGGTATATGGTGG

GGGGCCATCATTCTTGTGGGCCTTGTATACGGCTACTGGACGAAGCCCGTGTGA

>g21252.t1

ATGCAATCCCTCCTGTACACGTTGGTGTTGGCCCTTTGTGCCATCGCCGGAAGCAGGGCG

TTTGTCATGTCCCCGCCAGCACGAGCTGGCCTATCGACGACAGCGTCCCTTCGGGAGGGA

TCCTTCGTTTCAAGCACTTCGCCCTTTGCCTCGCTGACGGTCGCGCAAGGAGGGGGAAGC

CTTGCCGTATCCCGACCCAGGACAAGGCGGGGAGGCGAGCTCCACATGGGAAAAGTGTCC

AAGTTCGGCATCTTTTCCCCCGCCGTCGTAGGCGCGAAGATAGCCCTTGGGGAGAGTAGG

CTCAACAAGATCAGGGGAAAGGTCATTGCCTTGCACTCCCAAGTGATCACGGAGTACTGC

CGATGGGTCGGGGCTCCGTCGAAGGTGCGTGGGCTGCTTATCCGGAAGGCTAAGCGCAAC

GGAGACGACCTCGGCTTCCTGTGGTGA

>g21278.t1

ATGGCGCGTGTCCGCAGAATCACAACAGCAGGAACGATTGCCTTGTCAAGAGCTCAAGCT

ACGCTTGCTCTTGTTGGCGCTGGTATAACAGCACCTTGGCCTGCGAATGGCAGTGCTACA

AGCGGATCATTTTCAAGAACATGTTCGCAGCAGCCGCGATGCATCGGGAAGCTCCCTTTG

TCGTCCGCTGCTGCTAGCTCTTCAGACTCCACCAATCCCGAGCAAGAGaCGCCACCTTTG

TCCGACTCTGTCCAAAGGCTGCGCGAACTTCTTGACGAGGCAGGAGGCTCTGCGGTGGCT

CTCACCGGCGCGGGTATGTCGACTGACAGCGGGATTCCTGACTACCGTGGACCCCGCGGC

TCCTACTCGCGAGGACACAAGCCGATGACGCACGACGAGTTCTTATCGTCCGAAGCCAAC

CGCAAGAGGTACTGGGCCAGGTCCACCTTCGGGTGGAGCAGTTTCAGCAAAGCGAGGCCG

AACGCTGCTCACGTCGCTCTCGCCGCGCTTGAAGCGGCGGGCAAGGTCGGCGGTGTTATC

ACGCAGAACGTGGATGGCCTTCATCAAAAAGCTGGGAGCCGGAACGTAGTGGACTTGCAC

GGAAGGAACGACAAGGTTGAGTGCATGTCGTGCCAATCCAAGTCCAGCCGAGATGTATAC

CAGGCCCACCTCGCTCAGCTAAACGCCGCCTGGATGGATAGGTATTCCCCGGAACCGACA

CCGGGGGAGGCGCCATCGGGCGGGGACCCCAGCGTGGAATTGACCCCCGACGGCGATGCG

AATGTGGAGCCCGGGGAGTACCTCAGGGAGTTTGTGGTTCCCGCTTGTGTCAAGTGCGGT

GGCGTATTAAAGCCGACCGTTGTTTTCTTCGGGGACAACATCCCCCGGCCTCGGGTGGAG

GAGACCTACCGCATGGTGGACGAGTCAAGCTTGCTCGTTGCCGCGGGCTCATCGTTGCAG

GTGTACTCCGCGTTTCGCCTGGTGAAGAGAGCGGCCGATGCGGGCAAAACGGTTGTTGTC

GTGAACCTGGGAGACACGCGCGCGGAACGGTCGGGCCTCGACGTGTTGAAGGTGGAAGGG

GGTGTCAGCAGCGTGTTGCCGTTACTTCTGTAG

>g21291.t1

ATGCTCCAAGAGGGTTTTAGGTTGGCGGGGGTAGTCGCGCGCACGGCACCGCCACGTGTT

TTAGTGGCTGTGTCGTGCGGAGCGGGAGGCCAGCGCCTCGTGATTTTCGGGGGCTACAAC

GCGGACGGGCTCTTGAGCGACGTCTGGGAGTTCCACCTGGAGGCGAGGGAATGGGCGAGG

GTTAACGTCGCGGCCGGGCCGGCGCCGCCAGAACGAGCCTACTTCCGAGGCTGTGATGGA

CCGCCAAGCCGGCGCGGACGAAGCAACAGCCGGGAGAGTCCTTCGCCTACATTTTCGGCT

GTACACGGTGGCGAGTTACCTTCCGGCGACCTTCTTGAGGACCTCTGGCTCTTCAATCCC

ACCACCGCGTCTTGGAGTGAAAAGCGCGATTCCGCGACCGGGCCGCGCCCCTGCGCGCGG

TCTTCGCACTGCCTCGCCTACTCAAAGAGTGCCGCCGAACTGTCCCGGGACCGTTCTTCG

GGCGGTTCTGCGGGCGGTTCTTCCGTGCCGGTGGAGGCGGATGGGTCTCTGGTGCTATTC

GGGGGGCTCGGGCGCGACACGGACGAGGACAGCGACGCAGAAGATGTCGCGCCGCTGAAT

GATCTCTGGGTTTGGAGACCAGCTTCTGCcgcagGATCGACCGGGGGAGgtggagcggcg

gcggcaacgtcgGCGGGGGCTGGAGAAGGCGAGGGTGGCCGCCCGCGCGTCTGGTCCCTG

GTGATGCTCAACGGGGTGGGACCTTCGCCGCGGTCTCTGGCTGTCCTGACACCAAGGCCG

TGCGGCGGCGACCTGTTTCTTTTCGGCGGGTACGGCTTAGTCGAGCTTCCGTCGCCGCCT

TGCGATAGCGACGGGGTTGCCTCCGACGCGGAGGACGACAGTGGCGAAGAAGAGAGCGCC

GACATCATCATGGCGTACATCGATGACCTGTGGAGGTTGGACTTGGCCGGGGCGGGCAat

ggtagcggtagcagcggtggtggtggtggtggtagtagctcGATTGCGTCGAGCACGAGG

TGGGTAGACGAGGAAGAGATGGGGTCCGTGGGGTGCTCGATAGTGGAGGGCCGAAATGGG

CACACGCTGACCTGGTGTGGCGATACGCTGGTCCTGTTCGGAGGCTTCGTGGGTGACGGG

TTTGATGCCGGCCTGCACATTGCCAGGCCGCCGCGGCCACCTACTTCGCTTGAATGA

>g21314.t1

ATGTTAACATCGTTCTTCTCCGGCCCTCCTCCACCGGCTCAGGGGAAGACGGAGCGCGTA

GGCCGCATGCTCGAGATGCACGCCAACAACCGCGAGGAGATCAAGGAGGCTAGGGCCGGA

GACATCGTCGCCCTCTGCGGACTTAAGGACACGACCACGGGAGAGACGCTCTGCGTCAAG

GACGACCCGATCATCCTGGAGAAGATGGACTTCCCCGAGCCCGTCATCAAGGTGGCGGTG

GAGCCCAAGACCAAGGCCGACCAGGAGAAGATGTCGACCGCTCTGGCCAGGCTCGCCGCC

GAGGACCCGTCTTTCCGTTTCTCGCGTGACACGGAGACCGGCCAGACCGTCATCGAGGGA

ATGGGTGAGCTGCACCTGGAGATCATCGTGGACCGCATGAAGAGGGAGTACAACGTCGAG

GCTAACGTCGGCGCCCCCGAGGTTGCGTACCGTGAGGCCATCACGAGGAAGGCCGAGGTG

GACTACACGCACAAGAAGCAGTCGGGCGGGTCCGGTCAGTTCGCGCGCATCAAGGTCAAG

TTCGAGCCCGTGGAGATGGACGAGGAGGGCGCCACGTCCGACTTCGAGTTCGTCTCCGAG

ATCAAGGGTGGCGTCGTGCCCAAGGAGTACATCCCCGGAGTGCAGAAGggTGTGGAGTCC

GTGCTCTCCAACGGTGTCCTCGCGGGCTTCCCCGTGCTGGGCATGAAGGCGATCCTCGTC

GACGGTGCCTACCACGACGTCGACTCCTCCGTGATGGCCTTCGAGATCGCGGGGCGCGCC

GCGTGCAGAGAGGGGCTCCGGAAGGCCGGCGCTCGCCTGATGGAGCCCATCATGCAGGTG

GACATCATCACGCCCGAGGAGTACATGGGGGACGTGATCGGCGACATCAACGCAAGGCGC

GGCAGCATCGTGGAGCTCACGGACCGAAACGGCCTCAAGCAAGTAGAGTCCACCGTACCC

CTCGCGAACATGTTCCAGTACGTGTCTACTCTCCGGTCAGCAACCAAGGGACGCGCCAAC

TACTCGATGcagttgctcaagtacgacttCGTGCCGCCGGTGAGTCCTTCAAGGGGGAGG

GACGTCGTGGCCAAGTTCGGGGCAAACCGGAGCACCGACGAGGAGGAATAG

>g21316.t1

ATGGCCGAGGCCCCCACCACGACCGGGCGCAAGATCGCCCTGGACATGTACCGCAACATC

GGGATCATGGCCCACATCGATGCGGgtaagacgacgacgacggagcGCATCCTCTTCTAC

ACCGGAAAGTCCTACAAGATCggagaggtgCACGAGGGCGCGGCGACGATGGACTGGAtg

gagcaagagcaggagcgaGGCATCACCATCACCTCCGCCGCGACCACCTGCGCGTGGAAG

GACCACCGCATCAACATCATCGACACCCCCGGACACGTGGACTTCACCCTGGAGGTGGAG

CGTTCCCTCCGGGTGTTGGACGGCGCCGTGGCCGTGTTCGacggggtggcgggggtggag

CCCCAGTCTGAGACCGTGTGGCGGCAGgcGGACAAGTACGGCGTTCCCCGTATGTGTTTC

ATCAACAAGATGGACCGCACCGGCGCTAACTTCTACCGCGCCGTAGACATGATCAAGTCT

AACCTGAAGGCGGTGCCGGCGGTGTTGCAGCTCCCCATCGGAAACGAGATGGACTTCGTG

GGCGTGATCGACCTCGTCTCGATGACGGCGGTGACGTGGACCGGAGAGGAGCTTGGTGCC

AACTTCGACGTGGTCCCGCTCGCCGAGTCGGACGCGGTCGACGCCGCCCTTAAGGAGTCG

GCGCAGAAGTACCACGAGGAGCTGGTCGAGCTTGCGGTGGAGCAGGATGAGGACGCGCTG

ATGGCCTACCTTGATGGCGAGGAGCCGTCCGTGGAGACCCTGaagaaatgcctccgtatc

ggCACCCTGGCGAACACCTTCGTTCCCGTCCTCACCGGGACCGCGTTCAAGAACAAGGGC

GTGCAGCCCCTCCTGGACGCCGTGATCGACTTCATGCCCTCCCCCGTGGAGGTCGAGGAG

ATCAAGGGTGTCTCGATTGAATAG

>g21506.t1

ATGCGGGTCCGACAAAGGTCCAGCACAATGCCCTCGGCTATGGCTGCGGCGACGACGGTA

GCATCAAAAACCTTGCTCGTGGGCATCGTGGCAGCTTCCGGTCTGTGCGCTGCCACCGCG

TTCGGATTTGGCTCGGCGCTGTCTCCGTCCGCGGCAGTGATGGCGTCAGCCGCGGGCGGC

GAGCCCTCGAGGACAAGCGGTGTTAGCCGAAGACACGCTACGACGATGACGGAGAAGCCA

TCGCGGGTGTTCGTGGCGGGGGCTTCCGGTCGCTTGGGGCAGCGGGTCGTAAGGGAGCTG

TTGCTGGATGGTGTCGGGGTTACCGCTGCTGTGAGACCTTCGAGCTTGGAGAAGGCCAAC

AGCCTCTTCTCGGACAAGAGTTTTATGTCCGGAGACCTCTTGGGGAAGTTGGACGTGGTG

GGGGTAGAGCCGGAGAGCGACGATGAGTGGCGCAAGGCGATGCAGAGCTGCCAGGCCGTC

GTGTGCGCCCTGGGTGCGTCGGAATCGGAACCTTTCAACGTCAAAGGTCCTTCCCagGTG

GATGGAAAGCTGTCACAGAGGGTGGTACTGGCAGCCAAGGAGACGGCGTCGGTGCGGCAC

TTCGTACTCGTCACTGCCCTCGGAACTGGCAAGTTCGGGTGGCCGGCGTCGGCGTTGAAT

TTGTTTTGGGGCATCCTGTCGTGGAAGCGGAAGACCGAGAAGGCTCTCATGGACAGCGGA

CTCCCCTACACCATCCTCCGTCCCGGGGGCATGGAGAAGCCTCAGGACGATTTCGAGGAG

ACCCATAACGTGAGGGTAGCGTCGAAGGACACCTTGTTTGGAGGCGTTGTTTCCAGGTTA

CAGGTTGCCAAGCTGACCGCCGCAGCCGTCGCCTCCCCGGACACCAGCACTAACaagGTT

TTGGAGATAGTGGCGGAGGACCTGGCGCCGAAGGTGGCCTACAGTGAACTCGTCGAGAAG

GCTAGGGATGATCAGCCCGACGAGGTCTGGAAAAACAAGCTCAGTCCTGACCAGTACTAC

GTCCTCAGGATGGGAGGAACCGAGCCTTCCTTCACGTCCCGTCTGAACGGCGAGAAGCGA

GACGGGGATTTCTTGTGCGCCGGTTGCGGTCAGGAGCTGTTCCGGTCGAACAGCAAGTAC

AACAGCGGCACGGGATGgccgtCTTTTTTCGAGCCCACCTCTGAGGCGAGCGTGAGGGTC

ATTCGCGAAGGGGGCCTCTTCCCTCGGagagaaGTCAGGTGTTCGAACTGCGACGGGCAT

CTTGGGCACGTCTTCCCCGACGGTCCCCAGCCTACGGGCATGCGCTACTGCATGAACGGC

GTCGCCATGGGGTTCAGATCtaaggaggagaaagaggctgctgctgctcaagaAAACGCC

TGA

>g21613.t1

ATGCCTCGGAATATTCAGAAGCAGGCGCCTGTATGCCTTCCCGCGAGGGGACGGGAGGGT

GGCGCCGTTTTTGTGTTCTTGAACGATGACACGGGGGTCAACCGCAAGCTTAGGGCCGTC

CTTCTGCACAACATGAAGCCGATCCTTTGCATCGGGGAGAGCAAGTCGGAGTACGACGCC

GGGCTCGTCAAGTCGATCTGCGCGATCCAGCTGGCCAAGGACCTCGTGGGGGTCAGTGCG

GAAGAGATGAAGAGCATTGTCATCGCCTACGAGCCTATCTGGGCCATCGGCACTGGCCTT

AGCGCCACCCCTGAGATTGCGCAGAGCGTGCACGCGTACATCCGGTCATGGGTCGCCAAG

GCTTACGGCCAAGAGATCGCGGACACTGTGCGCATACAGTACGGCGGCAGCGTCACCCCG

GAAACCGTGGACGAGCTCATGGCGCAGCCCGACATCGACGGAGCGCTCGTGGGCGGCGCG

TCTCTGGACGCGGAGAAGTTTGACCGCATCATCCACTACAAGCAGTAG

>g21614.t1

ATGGTGCCCGCGACAACTTCCCAGCGGCTGCCCGCGCGCGACAAGCAACGCCGGCCCGAA

ATTCGACTGCCGATATTGCACTTCGATGTCGGGCGCTTTGGGAACGGAGGCGAACTACGA

GATGATCTGTGGCACGGACGGCCGAAGGCagagtcggcggcggcggcggcggaggctgaG

GCGTTCGAGAGAGGTCGGGGTTGGACTAAAGAAGACGGCCTGCGATGCCGTTTGCTCCGA

ATAGCAAGcACACGCGGTGCCTCGTCGCAAGGGCTTTGCATGGCGCGAGTCCCCCTCATC

GCGGGAAACTGGAAGGAAAACCCGTTGACCCTTGAGGAGGCGACGTCGCTGGGGAGCGCC

GTGGCCGCGGCTACCGCCAAGGTGGAGGGCGTTGAGGTGGCCGTGATCCCCCCCTACCCA

TTCATCTACCCCATCACCGAGGGAAGGGGAAACATGAAGCTCGGCGGGCAAAACTGCTAC

TTCGAGGAGAAGGGCGCTTACACCGGAGCTGTGTCGACGGGCCATGCTCGAGAGCATGGG

GTGCGAGTACGTCCTCTGCGGGCACTCCGAGCGCCGCACGTGAGTGATTACCATGTATGG

TACCTTGCGATAGCCCTTGCGACAGCGCCTGCGATAGTATTCGCAATAGCTCTCCTTGTG

TTCTTGAACGATGACACGGGGGTCAACCGCAAACTTAGGGCCGTCCTTCTGCACAACATG

AAGCCGATCCTTTGCATCGGGGAGAGCAAGTCGGAGTACGACGCCGGGCTCGTCAAGTCG

CTCGGCGGGCAAAACTGCTACTTCGAGGAGAAGGGCGCTTACACCGGAGCTGTGTCGACG

GCCATGCTCGAGAGCATGGGGTGCGAGTACGTCCTCTGCGGGCACTCCGAGCGCCGCACT

GTGTTCTTGAACGATGACACGGGGGTCAACCGCAAACTTAGGGCCGTCCTTCTGCACAAC

ATGAAGCCGATCCTTTGCATCGGGGAGAGCAAGTCGGAGTACGACGCCGGGCTCGTCAAG

TCGCTCGCCAACGGAACAACagcgcagtactcgtag

>g21791.t1

ATGCCgggaaacaagaagaagaagggagGGAACAAGGCCAAAGGAGCAGCTCCTCGGAGC

AATGCCGCCAACAAGGACGACCTTGCCGGGCTGGATGACATCTTGAGTAACCCGGAGCAA

ATGGCGGCGGCGCAACGAGAGGCCGTAGAGAACGGGACCGGGTACAAGCCGCGGTCGGGG

AAGCAAAACGCTCAGATCGGGCTCAAGGGTTTCACTCAGGCAACCCAAAACCCCGCGATG

TTCACGGACGCCATGAGCATGCTCGGAGACCCCGGAGcagtgaaggaggcggaggcgaTG

ATGAACGACCCAGAGTTCAAGTCGGAAATTTCTCAGTACATGGAGACGCTGAAGAACAAC

TCGGCCTTCCGGGACGCCATGGCGGAGGCGCAACGCAAATACCAGGCGCTCTTGGCCGAC

CCCGAGAAGATGAAGGAAGCAACGGATAAGTACAACGCCATGATGAAGGCACAGCTGGAC

GCGGCGGCTGCTACGGCGGagggtgcgggtgcgggtgcggcAGGGGGTGCGCCGGCGAGT

GTCGAGGCGGgggcaacggcggcagcggaaggcgcggcggcggcagaaaaGGGGGCGgAG

GCGGGATCTATagcaaaggaggagggggcagcGGTGGCACCGGCAGAGGCTTCCGCAGAG

ACCGTTGCGGCTGAGGCACCGGCGGCGGGAGCAGCGGCGGCATCTTCTTGA

>g21796.t1

ATGGACTTTTGCGGAGGGCGAAAGTTTGCGTTGGCGGCCACGACGGCCGCGTGCCTGCTG

GCCGCTGAGCCATGCTCGGCTTTCATCGCGCCCTCGCCGATCacctctggtgctgctgct

gtcaacagGGCTACTGGAAGGAGCTCTCTGAACATGGTGGCTGCGCCGCCCAGCACTCTG

GTCACCACCAAGTCTGAGGAGACCTTCGCGGAGGCCAAGGGAATCATGCCTGGAGGCGTG

AGCTCTCCGGTGCGGGCGTTCAAGTCGGTCGGCGGCAACCCGATCGTGTTCGAAAGGGTC

AAGGGCGCTTACGCCTGGGATGTCGATGGGAACAAGTACACCGACTTCGTAGGAACGTGG

GGCCCTGCTATCGTGGGACACGCCGACGACGAGGTGCTGGACGCCATCAAGGAAACCTTG

GAGAAGGGCACCTCGTTCGGCGCCCCTTCCCTGCACGAGAACACCCTGGCTAAGATGGTC

ATCGCCGCCGTGCCCTCGGTAGAGATGGTCCGGTTCACCAACTCGGGAACTGAGGCGTGC

ATGGGCATGCTTCGTCTCGTCCGTGCCTTCACCAGTCGCGAGAAGATCATCAAGTTCGAT

GGCTGCTACCACGGCCACGCCGACGGCTTCCTCGTGCAGGCCGGCTCTGGCGTCGCCACC

CTCGGCCTCCCCGACTCCCCTGGCGTGCCCCAGGGCGCCACCCAGAACACCCTGGTGGCC

ACGTACAACGACCTCGCGAACGTGGAGGAGCTGATCAAGGGCGGTGACATCGCTGCGGTC

ATCCTGGAGCCCATTGTCGGCAACTCTGGCTTCATCAAGCCCACCCAGGAGTTCCTCCAG

GGCTGCCGGGACCTGTGCGACAAGTACGACACCCTGCTGGTGTTCGACGAGGTCATGACC

GGCTTCCGCGTCGCCTACGGTGGTGCCCAGGACTACTTCGGTGTCCTCCCCGACATCACC

ACCATGGGCAAGGTGATCGGAGGTGGCCTTCCCGTGGGCGCTTACGGCGGACGCAAGGAC

ATCATGGAGATGGTGGCGCCCTCGGGCCCTATGTACCAGGCTGGCACCCTCTCGGGCAAC

CCCCTGGCCATGACCGCGGGTATCAAGACCCTGGAGATCCTCCAGCGCCCTGGTTCGTAC

GAGAAGCTGGAGGCTCTGTCGAAGCGCATGGTCGAGGGTGTGTTGGCAGCTGCTGAGGAG

GCCGGACACGAAGCGTGCGGCGGTTACATCCGCGGCATGTTCGGATTCTTCTTCAACAAG

GGCCCCGTCAACAACTTCGACGACGCCTCCAAGTCTGACGGAGAGAAGTTCGCACGGTGG

CACCGCGGCATGCTCGAGAGGGGCTTCTACCTCGCACCGTCCGTGTACGAGGCCGGCTTC

ATGTCCCTCGCCCACACTGAGGAGGACGTCGACCGCACCATCGCGGCGGCCAGGGAGGTC

ATGGCTACGCTCTAA

>g21830.t1

ATGTGGTCGGGGATCTTCGCGAGTTTGCTCTTCGTCGGAGCCGAATCTGGCAGCTCAAGG

CTGGATGTGATCCAGTTGCCTCTGGGCTTTTCTCCGGAGGGGATCACGCTGGGCAGGGAA

TGGACGGCCTATGTTGGCTCCCGCGCCCATGGATCGATTTGGAAGGGAGACCTGCGCACT

GGCGAAGGAGAGGTCGTTGTTTCGGAAGTGGGTAGCCGAGCGCTTGGTCTGGATCACGAC

CGCCGGTCGGGATACCTCTTTGCTTGTTTCGCGAGTTCAGCTCGCGTTTACGACGACAAT

TACAGCCTCGTGGCAGAGTTCGTGTTTGCTGGCGAGGGTGAAGTATCGACGATAAACGAT

GTCTATGTCACCAAGACCGCGGCATACTTCACAGACTCCTCCCGGGACAAGATATACAAG

GTCCCGCTAGACGCGGATAGTGGGGAGATAGTGGATCTGGCAGTCACCGACGCAAACACG

ATCACGCTCAGTGAGGACTTCGGCTTCGTGGAAGGGGAGATCAACGGGAATGGCATTGTG

GCCAACGACGACGGTAGCGTTCTGATCGTGGTAAACAGCCACGCTGGTCAAATCTTCACC

GTCGATCCTGAAACCGGTACCGCCGCCCTCATAGACCTTGGAGGCGTTCTTGTCCACGCA

GACGGCTTGGTTCTGCGCCAAAACACGCTTTGGGCTGTCGATAATGGTCGTCGCAGCGTC

TCCGGGCTGCAGCAAATCAGCGAGATCTCCCTTTCGGCAGACCTGACGTGCGGCTCTGTT

GTGCCCCGCGGCCTGACAAGCGCCCTCTTCGACACCCCTACCACTGCGGGGCGAAAGGGA

AACTCGCTTTACGCCGTCAACGCAAAGTTCGACGTTGCCGCGGAGGACGTGCCAGCGACC

GAGTATGAAATTGTCCGGGTGGATCGCGACAGTGGGGAGTACATGTGCACTTCGGCTTAG

>g21995.t1

atgCTGAACGAGAGGCAAAAGCAGGTGCTGGTGGACAAGATCAACGAGGACGTGGACATC

CCGTGGACCTCGGAAAACAGAGAGGAGCGCATGATCGACAAAATCGTCGACAGCGTCGCG

CCTAAGGTCGAGCCGGCCCTTCAAGCGATCCTCCCGGCCGTGTACGTGGCGTGCATCAAG

CTAGCCCTGGACGAGTCGGTGCCTCTCGACGACAGGAAGGACCAGATCTCCGACATGCTG

CGCAACGAGCTTTCTGATCCGCTCACGAGGGAGCTCAACGAACGCATCGACATGAAGCTC

CTTCCCGAGAACCTCGAGGGTGTGGTGCTCAAGGTGGTTTCCAACAAGGTCATCAGCGCG

TTCGTGGAGTGGACTGTCGGAGAGGTCACCGAGCAGCTCGCGTGA

>g21996.t1

ATGTTGAGCGAGCGTCAAATTGAGGTGGTGGTGGACAAGGTCAACGCAGACCTGGACATT

CCGTGGGTCTCGGAGAGGAGGGAAGAGGCCATCATCAAGAAGCTCGTCAACAAGATCGCG

CCGAAGGTGGAGCCCGCTCTGCTCGCGATCATGCCCACCGTCTACGTCACGTGCATCAAG

CTGGCGCTCAACGAGAGCCTCTCCGTCAAGGAGCGACGGAGTCAGATCTCAGACCTGCTG

CGCGCGGAGCTGTCCGTGCCTCTCTCGAGGGAGCTGAACGAGCGCGTGGACTGCTCGTTA

GTTCCCGAAAAGATCGAGGGCAAGGTGCTCAAGGTGGTGGCTAACAAGGTCATCGACGAG

TTCGTCGAGTGGACTGTTGGGGAAGTCTCGGACAAGCTCGCGTGA

>g21999.t1

ATGAAGGCCTCAATGTTCATAGCGACAGCATCAGCCACCACAGCGGCCCTGTGCTCTGGG

GCGACAGGCTTCGTGTTTGCGCCGACGCTTGCTGTAAAGCGAGCCGTCGGAGGCCAGGCG

TCCTATGCaaccacagctcctcgcgcgagggCCTCTTCTGCCTTGATGATGGCTGGCGAA

GAGTTTGACGACAAGGCTAACATGGAGTCGGCGAAGACCCGGATGGAGAAATCGACGGAC

AGCGTGGCCGACAGCCTCGAGACCCTTCGAACCGGCAGGGCAAGCCCGAAGGTGCTCGAC

AGGGTGGTGGTGGACTGCTATGGAGCAGAGACGCCGCTCAACCAGGTCGCTTCCATCAAG

ACCACTAGCGCTACCCAGCTCCTGGTGGAGTCGTACGACCCGACTATCCTCGCCGACATC

GCGAGCGCCATCCAAGAGGCGGACATCGGCCTCACCCCCAACAACGATGGCAGCGTAATC

CGGTTGAACATGCCGCCCGTGACGGAGGACCGCAGAAAGGAACTCGCTAAGGAGGCCAAG

GGTTTGGGCGAGGAGGGGAAGGTTGCGATCCGCAACATCAGGAGAGAGGCCGTGGATTCG

GTAAAGAAGGCTGAGAAGGCGAAGAAGTTGGGGAAGGATCAGTCGAAGGACGCGCAAGAC

GCCATCCAAAAACTTACCGACAAGTACGCGAAGACGATCGACGAAAAAGTGGCGGCGAAG

GAGAAGGACATTCTCAAAGTTTAA

>g22136.t1

ATGAAGTCCGCTTGCTGCATGGCTATCGCTTGCGCCGCTGGCGCTCAGGCCTTCGTGGCG

CCGAGCGCCTTCAACGGTGCCGCCCTGACGACCTCCGCCAAGTCGTCCTCGGCCATGAAG

ATGAGCTTCGAGTCCGAGATCGGCGCGCAGCCCCCGATCGGCTTCTGGGACCCTCTCGGG

CTCGTGGCGGACGCGGACCAGGAGCGGTTCGACCGCCTCCGCTACGTGGAGATCAAGCAC

GGGCGCATTGCTATGCTCGCCATCGTGGGGCACATCACCCAGCAGAACACCCGCCTGCCC

GGCATGCTCTCCTTCAAGGAGAACCTCGCGTTCGCGGACGTGCCCAACGGGCTCGCCGCC

TTCTCCAAAATCCCCCCCCTGGGCACCCTCCAGATCATCCTGGCCATCGGATGCCACGAG

CTCTTCGTGGTGAAGCAGGTGGAGGGCTCCTTCCCCGGGGACTGCACGACCGGCGGCAAC

ATCTTCCAGTCGGCATGGGACAACATGTCCGAGGAGACCCAGGCCTCCAAGCGCGCGATC

GAGCTCAACAACGGACGCGCCGCGCAAATGGGCATCCTCGCCATGATGGTGCACGAGCAG

CTGTCCAACCAGCCCTACATCATCAACGACCTCGCCGGCGCCGCGTACCAGTTCAACTGA

>g22332.t1

ATGGAGAGGGAAAGCCCAGCCCGCCCTCCTCCCACCTTTTCCGAGGCGATCGATGAGATC

GGCATGGGGCCGTTTCAAAGTCGGCTTTTGATGGTCGGCGGCATGGCTTGGCTGGTCGAT

GCGATAGAGGTTCTGGTGATAGCCTTTGTGCTCGAGGACGTGGCGGTGACTTTCGACCTG

GGCAGCTTCGAGAAAGGCCTTGTCGGGAGTTCCAGCTTCTTCGgcatgaTATTCGGCGCG

GTGTTTTGGTCTGTCTATGCCGACAAGCACGGTCGCCGCAGCGCCTTCGTTGCCTCCCTC

GCCTGCGTCTTCCTCGCCGGGCTAGCCTCCGccttctccccctccgcccTATGGCTTGTG

GTTTTCCGCATGCTGGTGGGATTCGGGGTAGGGGGGAGCCTTCCGGTAACGACCATACTC

GTGTCCGAGTTCTTGCCGACGAATCACCGCGGACAGGTTATCTGTCGCTTGAGTGGCCAG

TTCTGGGGCGCCGGTCTCGTATGCGCGTCCGTCTTGGGGCTGGTTCTCAACAGGGCTATC

GGATCATCCCCCGGGCGGGAAGAAGACATGTGGAGGTGGTTCCTGGGACTGGCGTCCGTT

CCAGGGGTGATGGTTCTGGCGGCTTACTTCTTTCTCCCCGAGAGCCCGCGGTTTCTCAGC

GTGGTGGGGAGGCACGACGAAGCCGTTACGATCGTTGAGAGTGTGGCGAGATCGAACGGC

AAGACCGATGTCCTGGGCATGAACCTGTCGGAACTGAACAAGAGCGACGGAGGCGGTGTC

GGTCTCACCATCAAGGCCCCGTCGGCCGATTCGGTGGAAAACAATAATAGCACTGACGGA

TGGGACGTGCGTCAGCTGTTTAAGACGCCGACTCTACGAAGGGCGACGCTTTCATGCTAC

TACGGCTTGACCTTTCTGCTCCCGTCCTACTACAGAGACATTTTCGGTGCCACGGATGAC

TTCGTGTACATATTCAGCGCTGTGCTTGGAGTGGTCATCTCTCTATCAGAAGGTCGACTC

GGTCGAGTGGGCACCATAAAGTGGTCCTCCTTTGTCACGGCGGTCCTGATGCTCGTCATG

GCCCTATCCTACGACACGCCGGCGGTGTTCCTTccggtgtcggtgttggcttACTTCGTT

AAGGCAATGCCAGCGATGGTCAAGTACGTGATCACGCCGGAGCTGTTTTCCACCCAGCAT

CGCGCGGTGGCCATGGGTACCGCTACCATCTGGGCGCGAATTGGAGCAACGCTGGCCCCG

GTGGTGGCTTCTGTCCTGTTCGACAAGGGGAGGGTGTTGCCTCTCGCCTTCTTCGGCCCT

TCAACGCTGCTGGCCTCCGTGTTTTGCTATATGATCCCGTTCGAGACAGCAGGCAGGAAG

CTGGACGACGAGGAGCACAAGGCCGAGCACGCAGCAATCGAGCCGGCAGCGTGA

>g22431.t1

ATGTCCGAGCAAATGAGCCAGATGCGAAAAGACCTGGAAGAGAACGAGGAGGCCAAGGCG

TATATGCAGGCGTTACGGGGGGCGGGCATCAGGGACTacgcggaggcggagggggagatG

CGGCTGCTCGACATCGAGGACGGAGAGGCTGAGGGAGCCGGGGGCGAGGCGGACAGGCTG

CCCATGGCGTACGACCCCGAGGCGCTGAAGGATTACTTCGCCCGTCGGCCACAGGTGGCA

ACGAGACGTACGCTTCAACTTGCTGGCGCCTTCTCCGGGTTCGTGGCGAGTTACCTCATC

GACACCGTCACGGGGAAGCTCAAGGAAAACGAGATAAAAAGGGCGATACAGCTGCGCGAG

ACCATCACCTCGCTGGGGCCGTTTTACATCAAGCTCGGCCAGGCCCTGTCCATCCGGCCC

GACATCCTCTCTCCGGGGGCCATGGTGGAGCTGCAGAGGCTGTGTGACAAAGTCCCTTCC

TTTGACAGCACCATCGCGTTTCTGACGATGGAGCTGGAGTACGGCCGACCCGTGGAAGAA

ATCTTCGTAGACATCACGCCGGAACCTCTCGCCGCTGCTTCGCTCGGACAGGTGTACAAG

GCAACGCTTCGATCGAATGGAAAACCGGTCGCGGTGAAGGTGCAGCGCCCGGGAGTGCTC

GAGACTGTCAGCCTCGACCTCCATCTCGCCAGACAGCTGGGCTACCAAATGAGAAAGATC

CCGTTCTTGGCGGCGAGGACGGACCTTGTGGCTCTCTTGGACGAATTCGCGAGCAGATTT

TTCGATGAGCTGGACTACGTGAAGGAATGCGCCAACGGGGTGACCATAAGGGAACAGATG

CAGCACATCAAGCAGGTTGTGGTTCCGTTCAACTACCCGGAGTTCACCACCAGGCGCGTG

TTTGTGTCGGAGTGGATCGACGGAGAGAAGTTGAGCCAGAGCCAGGCCGACGACGTCCAG

GACCTTGTCAACGTCGGAGTCACCGCCTACCTGACGCAGCTTTTAGACACGGGGTTCTTC

CACGCCGACCCCCACCCCGGAAACCTGATCAGAACGCTGGACGGCAAGCTGGCCATCCTG

GACTTCGGGCTGATGACGGAGATCACGGAAAACCAGAAGTTCGGGATGATCGAGGCGATA

TCGCACCTCGTACATCGCGACTACGAGGGCATCGGCGACGACTTCAAGCGTCTCGATTTC

ATCCCAGAAGAGGTGAATACACAGTTGGACGTGTGA

>g22924.t1

ATGCGGGAAAGGCTACTGAAATTAATGTGGATTGCGCAAAAAATCACGGATTTCGAAACG

GAGGACTTCGCCTTTGCCCTAGGCGGCCCTGAACCAGACAACCTCGTCAACAACTTCCAG

CTCCGCGCGGTGAACCTCAACCAGCTTCCCGCTCTGGAGGGACAGGGCATCTCCATGGCC

CTCGTCAACCTCGGACCCTGCGCCATCAACCTGCCCCACGTCCACCCCCGTGCCACCGAG

ATGCTGTACACGATCGAGGGCAACGACCTCCGGGTAGCGTTCGTGGAGGAGAACGGTGGC

GAGGGTGCCGTGGTCAACGACCTCTTCCAGGGCGACGTGGCCTTCTTCCCCCAAGGCCTC

ATCCACTACCAGCAGAACCTCGGCTGCGAGCCGGTGACATTCCTGGCTGCGCTTAACTCG

GAGGACCCCGGGGTGGTGACCATCACGACTCGCTTCttcgagctgcccagcgaggcCATC

CAGGCGTCGCTGAACATCGATGACCCCACTCTCAAGGCCCTCATAGAGTCACTGCCGGAG

GCTCCCGCCATGGCTCAGCGTCAGTGCCTGGAGATGTGCGCCCTTGAGAACGATGACGGT

GACTTCAGCCTCAACTTCGACAACTAG

>g22931.t1

ATGAAGACGTACTTCCACGCTGCTGTCGGCATCCTGGCTACGGGCCTCGTTGCCCCTGCG

GGCTCCATTACGGCACAGGAGAAAATCTCGGCCTTCGACGCGGATGACTTCACCTTCCCA

CTCGGCGGTCCCGAACCAGACGACCTTGTCAACGGGTTCCAGGTCCGTGTGGTGAACATC

AACCAGCTTGCCGCTCTGGAGGGGCAGGGCATCTCCATGGCCGTGGTCAACCTCGAGCCC

TGCACCATCAACCTGCCCCACGTTCACCCCCGTGCTACGGAGATGCTCTACGTGATCAGG

GGCGAGAGGCTGCGAGTAGCGTTCGTGGAGGAGAACGGCGGCGAGGGTGCCGTGGTCAAC

GACCTCCAGGACGGTGACGTGGCCTTCTTCCCCCAAGGCCTCATCCACTACCAGCAAAAC

CTCGACTGCGAACCGGCGACGTTCGTGGCCGCGCTTAATTCGGAGGACCCCGGGTCGGTG

ACTCTCACGACTCGCTTTTTCGAGCTCCCCACCGAGGCTATACAGGGGTCGCTGAACATC

GGTGACCCCACTCTCATAGCTTTGATGGAGGGACTTCCGGAGGCACCCGCTTTGGCTCGG

CTGCAGTGTCTGGAGCGCTGCGAGTCCAGCGACGACGGTGACTTCAGCCTCAACTTCGGC

GACGATCTACGGTTTTAG

>g23235.t1

ATGGAAGCTCTTCAAGCCTTGCCTCGGCATAAGCTTCGCGAGGAGCTGCTCAGGTGCTGC

CACTCAACCGCATGGGCGGACGCTGTTATTGCGAGGATGCCTTTCGCGTCCGAAGAAGAA

GCTCGGAAGGCACTGGATGACTGCTGGGGAAAGCTCACGCTGTCAGACTATCTAGAGGCC

GTCGCAGCGCACCCAGCTATCGGAGACAAGGAGGCGCTCAGAGAAAAGTTCGCCCCCTCG

agatccgggtgggagtcggcggagcaggcggcggcggcaggggcgagTGAGACAGTATTA

GACGAGCTATCCGAATTGAACCACGCATACAGGGAAAAGAACGGGTTCGTGTTTCTCGTG

TGCGCCTCGGGCAAGCCTGCGGACGCAATCCTGACCTCGTTGAAGGAGCGCTTGCGGAAT

GACACCGAAGCAGAGATGAAAAACGCTGTGGAGGAGCAGCGATTGATCACGCAGCTCCGA

TTCACCCGGCTGCTGGTGGAACCTGATCCACCTCTGTTGGCGACGGGAGAGGGGCGCGGC

GATGCTGGAGGGCAGAGGCAAATATCCCGACGTTCGCGCCTTTGA

>g23453.t1

ATGAGGACTACTGCTTTCGTATGTGCCGTGGGGGCTTGCGCGGGTGGAGCGGAAGCGTTC

GTAGGATCGTCGATGCGGCCCGCGTCAATGCGCCGCGCGAGCCCGAGCAGCGTTCGCATG

GCCTCGGACGTAACCGTCCCCGACCTGCTCAAGCAGACTGAGCAGCTCAAGCTTCTTTCC

ACGGCATCGAAGCTGGGACTCCTCTCGAAGCTCGAGAAGGCCGGCCTGACTCTCAAGGAC

GTCGAGAAGCTTCTCCCCCTTGTGGACGAGAACGACCTCATCGGTCTCGCCAAGGGCTTC

GGCCCTGACCTCCTCAAGATCGCGCCCGCCGCTCTCAaggccgcccccgccgcccttcCC

CTTCTGGCCACCGCCCTCACGGTCCCTGGGGAACTCCTCTTCGTGGGCGCCGCGGGCTCT

TTCGCCGCCGGCGCGGGATTGGTTTACCTCCTGCCCGACGACTCTCTGACCGGCGTGGCC

CTGGAGACGTTCCTCGCCGTCCCCCTTCTCTTCGTGCTCCCCGCTGTCCTCGGTGGTGGC

GGACTCGCTCTCTCCGCACTCAAGAGCGGCAAGCTGGTCAGCCTGCTCACCTCCTTGCCG

GCACCGCGCGTCCAGGTCGCCGACGCACCCGTAGAGATCTCCGTGAAGTCGGCTCCCGTC

GCGGCGGCCAGGAAGGCGTCGGGGGGACGGCCGGCGGCGGCTAGCGCGGGCAGGCCAGCA

GCCGCCCCGAAGGCTGCCAAGGCTTCCAAGGCGAAGGCCGCCCCTCGCCCCGCACCCAAG

AAGGCCGCCCCTAAGCCGGCGCCCAAGCCCAAGGCTATGGGAAACATCAGCAACCTGCCC

AAGGCTCTGTAA

>g23470.t1

ATGGAAACTCTGTACCGACTGTCTACCGCAGAAGGAGTACACCTGTATGGCAGCGAGTAC

AAGGGAATCGACTTTTTTGTTCAAATGCACCTTGACGGAGCCGGCATGAAAGACTGCGAC

CACTGGCACGACGACGCCGGAATCATGACCCATCACGTGGCCTACACCCTCCAGTTCGAG

CAGGCGATGCAACTCGTGGACCCTTCCGTGTCTATCCCGTACTGGGAGTACACCATCGAA

CACGCCATGGGCTTGGAGAACTACGGGCAATCTGAAGTCTTCGCAGCAGATTGGTTCGGC

GAGTTCTCCCCTGACAACGAGCTGCACACGGTCACCAACGGCCGTTGGGCTTACCTGTCT

GTCATGGAGGATGCCTGGGACTACGTTCACAACCCGTACGGCCTGCTTCGGTCACCCTGG

AACCTGGACCCAACTCCTTACGTTACCCGGCACAACACCACAAGCGGCGAGTCGCCAACC

GCCATGGTTTCTTGCGAGGTGTACTCAAGCTGCTTCGAGTCTTCTTCTATGAAGGTCATC

AACTCCTGCCTCAATGGACAAACCCATGGGCCCGTTCACATTCTCATTGGCGGAGAGTGG

GGTGCTTCGGAAGAGGCCTTCATCCAGAAAGTCGGATACGCCGAGTCTGTTCCGCTGGTG

TCCAAATACCTCTGGCGCAAGGGATACTTGAGAATTCCCGAAATCTGCACAGCAGGAGAG

GGTGACTGCATCACGACATGCCCGTCTCATCTATACGAGGCCAAGGGCATGACACCGTAC

GACGTGCTCATGGATGTCCATGCTATGATGTGGCTGGCACACACCACGCAAGGAGCACTT

GTTTACAACAAGAAAGCAGACAAATGGCAGATCAAAGGCCACGAAGACGACGGAGAGTTC

GAGAACGCATTCTGGAGCAAAATGCTTTCTTCGATGTGCAACCCTGGGCACGTTGGCGAG

ATGTTCACCTCTGCGGCTCCGTACGACCCTTTGTTCTGGGTGGTACACCCAACAGCCGAT

AGACTTTTGGGCTGGAGGCGGATGCTCGGGCGCGAAGGAGTAGACGGGTATGACTTCGAC

GAGACGTGGGGCTACAGGCACGCGACCGTCGTGGGCGAAACAGGCTCCGTTTGCAGCTGG

AAGAAGGTGGTGGCTGGCACAAACGACATGCCGACCTGCATCATTCAAACTTGCGGAGGG

CACAACGAGGACGACTTGCTTCCCTTTATCGTCGAGATCAACGGCCAGCGCGGACGGTAC

ACCAACGCAGAATGGATGGAGCTCATCTACCCCACCAACGTCGATCTGCCCTACATGTAC

GACAGCTTCGGATGGCAGCATTGCAGCTCGGAAGGCTACACCTTCGGGACTTGA

>g23544.t1

ATGGCTCCCACGTATAGAATGGCAACCGTGCTCCTGGTTGCGCTTTTGGCCCTGCCGGTG

CAGGTCTTGAGCTTCGCGACCGTGCCTACGCTGGCCGGAAAAGGCAGACATCAGCGGTTC

CTCCAGAGTGATGTCATCACACCGATAGCAACGCCGAACGAGCCTGGATTTGcagcacga

ggtcgCGAGGGGGCCATGAAGACCGGCTTCGCAACCGctcgtggtggtgatgttggcggt

ggtgcCTGCAAGGCCTTGATCCCCACCTTGACGAAGCGGGGCCTCATCCCCTCTTGGCGA

CGCAGGCGTTCTGGTTGCTCGGGGCTGCGTGTGCTGGGGGACGGCGGAGGGTGCGATAGC

GAAGAGGAGTGCGAGATTGTTTACGACGATGACGAAGGGTCTCGCCTTCTAACGCTGGGC

CTCGTGGGAGGCGACGGGCTGGAGTACGAGTTTTCGAGGGCTGCCTGGCTAGACAATTAC

AAAGTTCCCGGGCAGTTCGTGGGGGTCCGCCTCGACAGCGGTGGGGACGGCGGGGTCGCG

GGCTGCCTCGTGGCCGTCGCGATGGACCCGACGTCCGTGCGACAAAGCGAAGGAAAAATC

CAGGTTTTGGTAACGGAGGAGGCCACCTTTCGTCTCAAAGGGGATACTCGAGGGCCTGCG

GAGGAGGCTAAGGGGCGCTCGTTGGCAGCGCTGGAGGTGGGGGACAAGGTTAAGATAAGC

GACTTCATGGGGAGAGGGTTTTCCTCGCTGTTCGTGGGCTGGATAGGGCTGCAGTCGGCC

CTGCAGGAAAAACGGGATATCGTTATAGTGGCGTCGGGTGCCAGGGGCTTGGGTTCCATC

AAGCCCGTGTTGGATTGGCCTCCGATCCAAGCCCACGCTGGGATAAAGCAGGTTAGCGTT

TACTTGGAAGACGAAAGCCCCGTTTCCGCGGCGTTTCTGAACGATTTCCTGGAATGGAGG

TCGTCGGGCATTCGCGTGACGCAGTGCTTTACCTCGGAAACACCGGGGACCCCTTCAAAC

GTGGAGGCGGCGATATTCTCGGAAGGCGAGGGGTTGAGAGCAGCGGTTGGGGGCGACCCG

GCGGCCGCGTCCTTCCTCATTGCAGGTTTGCCGGGCAAGCGGGCGATGGAGCTGACCGAT

CGGTTAACAGACCAAGGGGTGGATGCTAGCCGCCTCCTGTTTAATGAGTTCTTTTGA

>g23606.t1

ATGTGGAGTCTCATAGCCGCATCAAACATAACCGATAACTCGAAGGTTCTGTCTTTGTGG

GAAGATACATTCACGGGCGTATTCATGTTTCGGGCACGCTGGCTGGTTCACGCGTCGAGC

CTTCCTGCAGACGCTTTGTCGCGGCTACGAGCAACAAGTCAAGCCTCTGCAGCCGACGGT

AGCGCGACCGAAAGCGAGACGCCCTTAGGCAGAGATAAATACGGGGCTAGCGAGGTTTTC

CTCTCCACAAGAGTGAAGGATCTCGAGGTTCATTCTATTGTGAAGCCGATCAACCTGTGC

GTCAGCACCAGCACGACCACGGAATTATCTCCTGCCAGGAAAGACAAGATGGCGCCTTGT

CTTGCACATGCCTATTGCGACGCTTCGGGCGAATTCCACCGTCTGGAGAACACGGATCCC

ATCTTCAAGCGCGCTCGAGTCCGGCAAGCGAATGCCGTTGCGCTCGCCTCTAGAGACGAC

CAGCAGCATGAAGCTGCTAAGAGCGAGAAGCCACTTGTGCACAGCAACGGGTGGTTGCTA

CCGAAGAAGATGGCGTTCGAATGGAGGGGTGGGCGGACTGCTGTTGAGGGGGCAGAGAGT

GCGGAGATCGTGCCACCACCATCAAACAAAACGGGTAGTTATGCAAGCTCTAGCTTGCCT

AGCGTAGACAATCGAAACGCATCTGACGACCGTGGGCCCGAAGCGCCTCCTAGCTCCGCG

GGTGGACAGCGGGAAGAGAGCAAAGAGGACGAAGCCCAGGACACCATCGACAGCCAATCC

AAGTGTCAGAGAGACTCAAGACCAACCCTTTCCGACAGGGAAGGGTGTACGCAGAGAAAG

TCCCCCCGACAGCGCAATTCCCAGGGGTCGCCGACCCCTGATGCCAAGGTGGAGGATTTC

TTAGCAGCAAAACCCGAAGACAACCtctgccctcccgcccccactgcCGCTCCGACTTTC

CAGAGACGCCGTTGTGTACCTTCCAAAATGCGTCAAGGATCACTCCCTCCCCGACACCGA

CGCTTGTCGATGCCGCCCACGGCAGAAAAGCCTATCATCTCTCACGTATTTGAGGCCCCT

ACCGAACCTAGCGCGAAGTTACCCGTGGCGGTCAAGGTAGTGCGTCCTTCTAGGACGCGC

ATGTCGTCGGGGTTAAGGCCGAAATATCCCCTAGACCTACCAAGCAGCTCTTCCGTGGAA

GATGCTGCCAAGGCCTCTCCTCAGCTTTCAATTCCGCCAAAAAGAAAGCGGGTTGTTGCA

AACAAGACGACGCAAGCAGCAAACCGATCAGACTACCCACGCCTGCGCACTCTAGTGGGG

AAAGACTACCAGACAGACATCCCTGAGATGGTGTTGCCAGACGAGAGAAACCGGCCACGC

TCAGGGCCGGGTGCCAAAATGGTGTGGCGAAGCGTTCAAGACTGGGACCCGCAGTCCCGC

GAAATGTTGGCCAGTTACCTGCACGTAGCCAAGGGCGTTGTCGAGGAAAAGCAGGTTCGC

CCCGGTGTTGCCGTTCACGTGCGACTTGGTGGCAACAATCGAAGCAGTGGTGTGGACAAG

GATGATAACACGGACATTGCCAATAGCTACCCCGTGTGGGCGATTTCGTCCGGCCGCGAG

ACCTGTGGACTAACAAGAGTTGGGTGTTCGGACCTCGCTCAAACAGACATTCCACGGTCT

TCCATCCAGAGGGTGCAGAGTCAAGAGCAGGCTCTATCTGCTTTGGTCAACGCGAGGTGT

GTTCTGGACAATTTCGAGCCAGCCCTGCAAATGCTCGCCGGTGACCAGGGATTGCCAGAG

AGCATCGAGCCCTGGACGCTCCGACAGGTGAGAACCCTCGAAAAAGCGCTGGAAAAGGAG

TACGACCCAAACAGGAGGTTGCATGGCTGGGCTCGACCGGGAGTGGAGATGGACCGCGAA

GAGTTCATTGACCTGGCGAAGGTCAGCAAAGAAGTCAGCGGCAAAACTCCAGCTCAGGTG

CTCTCCTTTTACTACAGGTATCTTGCGGCTGCAGAACCTCTCACGGACGTTGTCTACGGG

CCTCAGGCAGCAAAAGCTCGAAAGCAGGAACCCATCTCCCCTGTCTCCTCTTCCCATGAG

GCGGACGTGATCCTTCCCACCAATGGCCGACCCGCTCATGGTAAGGTCAAAACCATCAAC

CCGAAGCTTCCTGCCAGGAAAGAGTCGCTTGGGCTGTCCAAAGTCGGTACTCCTAGCGGT

TCTGGAACGGCGATCGACTCAGCGCCAAAACCAAAACTACGAGTTATCCGGCTGTCCGCC

CCGAAACCCGCCGAACCCGTTGTGGTGAGCACCAAGCCAAACGACGGCCCCACTGCTGTC

AACAGCTTACGTAATGGTGATGTGCCGACAAAGcaggtcccccctcccccaacctgcGAC

GCGGTTTGTTCCGCTCCAGACGGCACTGCCACTTCCCATTCGCCGGCTGAGCCTAAGCAT

GCCACCAACGAAAAGTATCGTGATGACGAGCCGCACGCACAAGCCGATCGTGTAACGAGT

AGAGAGATCGCCGGGGCTGTTCGTGTAACGGGTAGATCAATGGAAAGGCGGGGTGGAGGA

GGTGTTGGCCTTGACAGCGCAAGCGAATACTTGGCTCAGCAGGGGCAACGCGCAGTGCAA

GGGAGGGAATTCGGAAGGACCCTTCCTGCTCTTCCCATTGACGCTGAGGTGGTGAAGGTG

AGACGTTGTTAA

>g23697.t1

ATGGAACAACAACACCGCGACGGCGACGGAGAAGATGGTGCCCGCTGGCGTACCGGCTCC

TACAGGTTTTTGCGAGCAGGCGCCAAAGAAGGACGACGAAAGTCTCGTGAGCTCTACGCC

GATCCGGATCCTACTGTCAAGTGGATAATGGAAGGCCGATCGAAACAAAACGGGGTGGAC

GAGCAGAGCCAGGCGATACCCGACAGGGACTACCTGAACGGATTGGTTGAAGCTTCAGTA

GCAACCACGGACCACCAGGAGGGAAACATTGCTTTTGCCAACACCTACCAGACGGGATCT

ACGTCTATGGCAGGCATTTTGTATCGATACGCAGTACGGCACAACCTGAAGTTAGCGCGG

GTGGGCCAAGAATCAGCAGTCGATCCCAAGTGGTTTGCTACCCAGGTGCAGCAGGGGAAA

TCCGAGCGTGTGGACATTATGCACTACCACATCTCCAATAAAGGGCACTTGAAGGGACTT

TGGCCTGGTGCACGCGAGAACTACCGAGGCATAATGCGAGAGCCTGACAACGTCAACTTA

CTCACGATGATGAGAGAGCCACGAGAGCACTTCATCAGctactactactatttcgtCGAA

CCGGCGACCAAGCTATCTCTTGAGGACTTCCTGATGAAATCACACGGCGGCGACGACTTC

GCACTGCTGAAAAACCCGCTCGCGTCGGAGGTCGGGGTGACTACATTTCACAATATGGAA

CAGCTGTTCGTCAACGACTTTTCGTCCTTCAGGATGATACTCCTGACAGATCGGTTCGAT

GAGGGGCTGATGGTTCTACGGCGCCTTCTAGGCTGGAGCATGATCGACATGACCTACATG

GCGATGGACGAGACGAAGGCGGGGGACATAGCGGTCGTGGACAGGCCATCCTTCCACGAT

CTCCCTGAGAAGGTGCGGAACAAAATCGATTCGCTCACGGAACTGGATCAGATGGTGTAC

AGTGCAGCCCAAGTGCTGTATGACAAGCGACTGCGGCCTATGTCCGACGTGATAGGTGCA

GACGTGGAGGAGTTTCAGAAGCTGCAGAAGGTCGTCAGCGGTTACCTGGATGCCAACCAG

TCGAGCAAGGCAAATGCTATGTATCGAGCGGAGGATGTTTACATCGGAGCACCGCCGATG

AACCAGTTCTGA

>g23713.t1

ATGCATTCCCAGTCAACGTGGACGATGGCAACACCAACCTTGCCGCTCGTTGTATCCGTC

ATTATGCTGGGGCTGGCGTGGTTTGGTCAAGTGTCTGCATTTGTACCAACGCCAGCAACA

ACGTCGTGGATCCAACGAGAAGTACGTAGCACGGCAGGCGTGAATCGCGTGTGCCAGCAG

CAGGAACGATGCGGAGCGTCAGCCGTGAGCACCGTCACGATGGCCGGAGACGGCGTGGCT

GAAGCGAAATCGAAGATTCTGCAGCTCGCAGCTGTCATGGATAGGGGAGGCATGGCGAAC

CCAGGCGTTTCCAGCGCTTACTGTGGCACCAAGGACGACATGCGGCGGTTGGTGGACACG

CTCGCTGAGCTCGACCCCTTGGACAAGCCGCTGACGGCGACGGAGCTTAGCGGCCGCTGG

GAGCTGGCGTACACCACAGTCGAGCTCTTCCGCGCCTCTCCCTTCTTCCAGATGGTGGAG

GCGGGCTACGACAACCCAGAAAAGTCCAACCTGTTCTTCAAGCTGCACCAGCTGCAGACG

GGTTCGTGGGGCGCCAGCTCTATCGGCCGCATCACGCAGACCTTAGACCTTATTGACTCG

GTCCCACGTAGCGTAGAGAGCTCCGGGGGCGAGGCGTCCGCGGcagcagagggggagggg

aagggggaggtggagggggacGGGAGTGCGACCGCCCTGGAGACTACATCGGGAGGCGGC

AGCGGTTCCTTGGAGTCCGAGGTGGACTTGATAATTCTTCCTCTCACTTCTGTGCCGCTG

GTGGGCTTCTGGAAGCTCCTACCAACGTTTGGGGGATGTATTGCGACAAGGGCGAGCTGT

GTCCTCTCGGGGGACAACAACGAGGTTGTGGAGCTCACTGTGGAGTCCACGAAGCTCAAG

GCGGTGGAAGACGTCCCGTTGCTTCCGCTTGCGGGAGCGTTCTTCGAGGGCAAGGACTTT

CCCACCGGAGACGCGGCTAAGATGGTGATGGGGGACGTGCCAGTGACTAAGCAGACGGTC

GCGTACGTTGACGGTGGCATGCGCGTCATGCGCGACAAGGCGGGGGAGCTGTACATTTAC

GTCAGGCCGTTGGAGAGCTACTAA

>g23738.t1

ATGATGCGAGCTTGCCTATTCTTCGCCGCCGTGCTTGCGGTCGGCGACCGCTGCCTCGCG

TTCACGACGGTACCTTCAGCTGCTTCGATTCGGGCGCTCGGCTCAACACAGGTAAGCGAT

GACTCGGGTCCGTCGTCGACGGCGGTGAGCATCAGGTCCTCGGGAGGCATGCGAATGGCG

CAGTCGGAAGAGGAGAAGGACCGCGCTCTGGCGAACATGGCGCGCGCGGCGCGGCAGGTC

GGGCCGAACGACCGAGTGGTGGAGCTCAGGAAGccGTTGGGGCTCGTTCTGGAGGAGGAT

GAGCGGGGAAATGTATACATCGTTGAGATCCTGGACGGAGGAAACGCCTCCAGAAAGAAA

ACCATCAATGTTGGGGACAAAATCAGTTTCGTGTCGGCTACCTTCGGCGACCAAATTTGG

AGCGCTAAGGGCGTGGGGCTGAGCCGTGTCCAGTCAGCTATCAAGctcCGGAGTGGACCG

TTTGTAAAGTTGGTCCTTGAAACGAGTAAGGAGGGGGCTAAGAAGATGCAGCGAGACGCG

GCCAACTTGAAGAAGAAAATGGAGAGCCAGGAGAGCGAAGCCGAAAAAAGGGACCGTCTT

CTTCTCGAGCTCTCGGACGACACCAAGACTCAGAAGAAGAAGCCGTGGTACGGCTTGTTT

TAA

>g23875.t1

ATGCCCGTCAGCATCAACTTCAACCGCAGAGACTACCTCAACGGATTGGTGGCCATGTCA

GCCGTTACCAGGGACAGCCAATTGAGCAATCTTGCTCTGATGAGAACACACAAGACCGGA

TCGACAACCCTCGCGGTCCTGCTGTACCGATACGGCAGAAGACACGGCCTCGAGGTGGCG

CAGTTCGCGGGTGCACACTCGACGATTCCGATCGCGGAAGCAGCTAGGAAGACGCAAGAG

AGCCAGAAGTTTGTGGATATCATGCACTACCACATAGGCGACGCCGGGCCGTTAGCGGGC

ACGTGGGACGAGGCGAAGGGCATGTACAAACAGGTCCTCCGGGACCCTGAGAACATCAAC

TATATCACCATCTTCAGGGAGCCGCGGGAGCATCTGCTGAGCTACTACACATTTTTCATC

GAGCCGAAGACTCGGATGCCGGTCGAGGACTTTCTGAGCCAGCAGAACCCCGACCCGGCA

CTTCTCCAGCGCATCGACAACTCCCTTTGCCAAGAGTTTGGCCTCTACACGTCGGGAGAT

CTGGAACATTTCATTTCGTATTCGCTCCCGCAGTTCAAAATGGTTCTCCTCACGGAAAGG

TTTGACGAGAGCCTGATGATAATGCGGCACATGTTCGGCTGGCACCTGATCGACATGACC

TACGTCTATCTCAACCAGACCGCTGGCCTGAAGAAGAGGGGAGAGGTCCTTAAAGACAGG

TCGCCTTTCGACAGCCTCCCTTCTGATGTGCGGGAGAAGATCGACAACCTCACGCGCATG

GACCAGGCGTTGTACGCGGCTGGAGAGGCTGAGTTCGAAAAGAACAAGGCGCCCATCGCG

AAAGAGGTGGATGCTGACATGGTGGTCTTCGAACAGCTGCAGAATGTGGTCAACGAGTAC

CTCAACGCAAATCGCAGGAGCCCAGTAAACCCGATGTACCGGGCCCGAGCGTTCTATGAA

GACCCGCCGCCGATGCACGACTTCTGA

>g23917.t1

ATGAAGACCGCCGGTGCCATTGCTGCCTTTGTTACCCTGGGCTGTGCCCACGGTTTCGTG

GTACCTGTGCCTGGAGCAACAGCAATGCGGCCGCAAACACAGCGAGCTGCGGAGCGCATC

AACGAGTCGGTCGAGCTGGAGAAGCCGAAGGTTGTGACCATGGAATCGGTGGAAGCTGGC

GACAAGAAGGTCTACTGCCGGTGCTGGAAGTCCGGGACGTTCCCCCTGTGTGACGGCAAG

CACATGGACCACAACAAGGCCACTGGGGACAATGTCGGTCCGCTCATCGTGTCTGGTCCC

AAGTAG

>g24368.t1

ATGCAGTCAGCCTTCCTCGCCGCCGTGCTCTCTCTCCTCTCGGTGGCCTCCACCGATGCC

TTCGTGGCGGGTGCTGCCACGCTTCCTGCGCGGACATCTGCCTCGCGATGCGGAGCCGCC

TCGGTGGCGATGAGCGCGGAGCCGAGGTCACGGAAGGCCCTTCTCCAGCAAGGTGTGGCA

GTCCTGGCAGGACTTGCGGTGGCCACACCGCCCGCGTCGGCCGGCCTGTTCGGCGGAGAG

AAGAAGTGGGCGGGGGTGATCGACCCCCGAAGCTCTGTGAAGGACGCGGACAAGCTGGCG

ACGGATGCCGTGCAAAAAGACATCGCGGCGCTCAAGAAGTACCAGGCTGCCACCAAGGAC

ATTTCCGCCCTTTTGgAGAGCAACCCCCAAGCGGATGTGCTGACGAAGGTGAAGTCCACG

TTTAACATGGCCGAGTTCCGCGCTACGTTGAACGGCATCAACGACGTCTTCGACGAGGAC

ACGCAGAGGGGGACCGACCTCATTGTCCGAAACATGCTCCAGGACGCCCTCGAGCTAGCG

AGCGCCTCCAAGATGAAGCCCGATGTCCCTCGCTCCGAGCGCAAGATCGAGATCTTGCAG

AAGAAGTTGATGAAGCTGGAGCAGGCGTTTGTGAGCCTCAACGCCTTCCTCTAG

>g24654.t1

ATGAAGTGCATCGCTATGGCTGCTGCTTGCATGGCGGGCGCTCAGGCGTTCGTGACCCCG

AGCGCGTTtaacggcgcctccatggccaCCCGGGCGAAGACCTCTTCCGCGATGAAGATG

TCGTTCGAGAACGAGCTGGGCGTGCAGGCCCCGCTTGGCTACTGGGACCCTCTCTACTGG

CTGGAGAACGCGGACCAAGAGCGGTTCGACCGCCTTCGGTACGTCGAGATCAAGCACGGC

CGCATCTGTATGCTCGCCATCCTCGGCCACATCGTCCAGGAGAACGTGCGCCTCCCGGGC

ATGCTCTCCATCTCTAACGACCAGTCCTTCGCGGACATGCCTAACGGCCTTGGCGCCCTC

AGCAAGATGGGCCCCATTGGCGTCGCTCAGATTGTGATCTTCATTGGCTTCCTCGAGGTC

TTCGTGATGAAGCAGAAGGAGGGATCCTTCCCCGGCGACATGTCGCGCGTGAACCCGTTC

GAGAAGCAGTGGGATTCATTTGACGAGGAGACCAAGCTCAGGAAGCGCGCCATCGAACTC

AACAACGGCCGCGCAGCACAGATGGGCATCTTCGGCTTGATCGTGCACGAGGTCATCAAC

AACCATCCGTATGTCATCAACGACATCCTCGGCACCTCGTACACCTGGCCTTGA

>g24658.t1

ATGTCGACCGAGGCGGACCAGCGGTCGCCGGTTTTGTCTTCGAGTAGCCCAGAGGTGCTG

AAGGAAGGCAGCTTAGAGATACCCGTCAAGAAGCGAGTCATTTCGGGAGTGCAGCCCACC

GGCAACCTACACTTCGGCAACTACTTGGGAGCTATCAAGCAGTGGGTCGATAACCAGGAC

AAGTACGACAACTTCTTCTTTGTGGTGGACCTGCACGCCATCACCGTACCGCAAGACCCC

AAGCTGCTCAAGGCGTCAGTCACAAATTCTGTAGCCACATACCTCGCGGCTGGTATCGAC

CCGGAAAAGTCCAAGGTCTTTGTGCAGTCCCACGTCTCCGCACACGCGGAGCTGACGTGG

CTGCTGACGTGCTCGACACCGGTGAACTGGCTGGAACGGATGATTCAGTTCAAGGAGAAG

AAGGTCAAGCAGGGGGAGAACACGGGGGCAGGGCTTTTAACGTACCCAGTCCTCATGGCC

GCGGATATCCTGCTTTACCGGCCGGATCTTGTACCTGTGGGAGAGGATCAACGACAGCAC

CTCGAGCTTACGAGAGACCTCGCTCGCAGGTTTAATGACAAATTTTGCAAGCGAAAAAGG

AAGACCTTCAAGGATCCCCAGGCTCTCATTGTGAAGGACGGCGCCCGCGTGATGTCACTG

ACGGACGGTACGTCGAAGATGAGCAAGTCGGACCCTGTGGAGGGTAGCCGCATCAATCTC

ACCGACTCGCCGGACGTTATCAACAAAAAGcTCCGGAAGTGCAAAACAGACATGTACCAA

GGCCTTGAATGGGATAACCCTGACCGACCCGAGTGCACTAACCTTCTCACCATCTACCAA

GCGGTTTCCGGGAAAACTCGCGATGAGGTCGCCCTGGAAGTGCAGGACATGACCTGGGGG

ACGTTCAAGCCTCTCTTGGCCGACGCCACCGTGGAACACCTCAGGCCCTTCCAGGCGAGG

TACAAGGAGATCCTTGAAGACCGTTCGTACCTCAACAAGGTGTTGCGAGATGGGGCGGAG

GCCGCAGACGAGGTGGCTTCCGAGACGTTAGGGTGGGCCAAGGAGGCCATGGGCATCCCT

TCGCTCAAGGACTTCCAGGATTAG

>g24694.t1

ATGGGTCTCGTTTCCTTCTTGGCGGGGAGGCTTGAAGTTTTGCGAGTCGCGCCGTGGCAC

ACGAAGGTCCTTTTCAACGCGGCTGCTCGTGCGAACATCCAGACTGCTACGGTCACGTCT

ACTCCTCTTACAGACGCAGGGCTGGACGGCACGGGCGAGGTCATCCAGGTCCTAGACACT

GGGCTGGATGAGACTTCGTGTTTTTTCGAAGACGGTGATGGACTGGAGGTTACGCACGGG

TACTACTTCGAACAATTGGGTCTCTTTCGGAATTTCTTCTACCCTTCCTCGGGGTCGTCC

TCTGCCGCGACGGTCGCGGACTTCATAGGTGGAgatttctcgttcgatatcaaTCGGCGC

AAGATCGTCCAGTACATCAATATGGTCAAGCCAGACTCGCCCACTAGCTCGTTGTCGCCA

CTACCTCCAACAAGCCAAGGCCGctctgtttcctgggttgggggCGATTGGTTTGAACAA

GATGACGCAGCCGGACACGGTACCCACACCGCAGGATCGGCGGCGGGTGCGACATTGACC

AGGCCTGCCGATCCTGTAACATGCAGCGGTACGGACCGTTTGAGCTGCGTCGGTGGGTGT

ATCGACGACGACGCCAGTTATTCCATCGATGACTTGGTACCTGCGTACTACCAGCTGATC

GGAATAATCGACATCGACCGGATCTGCCCGGCGTTCGGTTGCGACGATGCCACCAGTGAC

GTGTGCCTGAGAGACGACGTCAGCGAGACTCTGACCGATCATGGAGGCATGGCCCAAGGC

GCCAAGCTGGCGATCTTCGATATTTTTTTGTCCTTCATTGGGCTAGGGGATACTGCCGGA

AACGGATTGTGGGAGGCGTGCATGGACGCCGGATGCAAGCTGCATTCGAACTCGTACGGC

GCCGACAGCCTGTGCACCCTGTCTGCGATGGAGGTGGAGTACGACGATTTCATGTACAGG

AACCCGGAGAATCTGATTATATTTTCCGCTGGCAACCTCGGTGACATCAACGATGGCCGC

ACAGTTTGTACCATAGGAAGCCCGGGAATTGGTAAGAATATGCTCACAGTCGGGGCTACT

TCGTCGGGACAAACGCGAATCACGGTCACCGGGAAAGACGGCACCATCGTGGACGGCACA

AACGGGTCCGCTGATATCGACACGGTGTCTTTCTTTAGCTCCTACGGCCCCACCCGGGAT

GATCGCATCAAACCCGAAATCGTCGCGCCAGGCGATATGATATACTCTGCGGCCGGTGAC

GGTACAGACGATCATTCCTGCAGGCTATATGCGTACCAAGGCACGTCCATGTCCTGTCCA

ATCGTGGCAGGTGCCTCGGCAATGATACGGCAATACTTCGTTGATGCAAGCTTCTACGCC

ACCGATGTGTCGGCGAGAGGTTTCTGCGACCAAGCCTTTCTGTGCGAGGGATTCTCCCCT

TCCTCGGCAACCGTAAAGGCGATGTTGATCAACAGCGCAAACCTTATGGGCGGAAGTTCG

GAGCCAGACGGACTTCGCGGCTTCGGACGCGTCCACCTAGAACAGGGGATGCCGCTGGAC

GGGGAGGGGAGCCTGGTCCTTTTTGTCGCCGACGCCGCAAGTACCGCCATACCAGAACTC

ACGGTACAGGAATATGTTTTTGAGTTAGACGGCGCGGCGGGCTTGGATTTCCGCGTCACG

CTCTCCTGGATAGACCCCGCTGCCACCTCGCTATCTGCCAGGCAGCTTGTCCACGACCTT

GACCTGGCCGTGAGCGCACCCAGTGGAACCAGGTACACGATGTGGGAGTCCGGTGAACCG

GACAAAGTGAACGTGAACGAAAGGGTGATTGTTGCCGGTTCGGAGGTCGAAAGCGGTACA

TGGACAGTGTCCGTGTGGGCCAAGTCGCTCACCACCGATTTCCAGAGCTACGCGCTTGTC

GTCAACGGGGCCATAAGTTCCGTGGTTGAGAATTTTGGGGGTGCAGACAGTTCAAGCTCG

ACCCCTTCTGCCTCCTCGAGCGCGCTTTCGAAATCGATTTTGAACCAATCGTCGGCAGCA

GCGAGAGCGTCATTTTTGGCGGTGCTCTGCAGCACTATCGCCTCTACCATTGCAAACGCT

GCGTTGCGCACGTGA

>g25020.t1

ATGAAGACTGTCTGCGTTGCTCTGGCTCTTGTGGGAAGCGCTTCGGCGTTTGTGACCCCC

ATGGCGACCGTGCGGGCGACCTCGACCAGCGCGTTGTCCATGTCCACCAGCGAGCCGATG

TCCCGATCGCAGGCGATCAGCACTGTCTTCGCCTCGGGAGCTGCggccgttgccgccgct

gccCTCCCCAGCGCCGCCTTCGCTGACGGCGCCAAGTCCCTCGCCACCCAGGCCCGGTCC

AGGGGAGTATACGGCGCGCGCATCGAAGCGCTTAAGCCTGCCGTGGAGAAGGGAGACTCG

GCCGCGGTCTACAGCGAAAGGAACGCTTTCACTCTGTTCAACTCGGGAGTGTACTCGACC

GACAAGACGAAGCGCCTCGAGGCAAACAAGCTTGCCGAGGCTGTCGTAGCCGCGGCCTCC

ACGGGCGACGCGGGTGCGCTCAAGAGCTCGTACGCGGCATACATGAAGTTCACGGAGAAG

AAGAGCGGCTACTCCGGGGCTGGTGACGGACAGGGCTTTGGGTCTGAGTTCGACTACAAG

AACAGGACGCCGGCGGGCaccgtgtaccagGACGTCGGAACGCTGTGGTCGAGCCTCTGC

GATGTTCGCGCTCTCGTTCGCTGTGGCTGCAGCCCATAG

>g25030.t1

ATGGTCGCTACCCAGATGTTCCTCGGTGCTGCCGCCACCCTCATCGCGTCCTCGACCGCC

TTCATGGCGCCGATGGCCGTGCGATCCGTTGCGCCCGCGTCCTCGTCGAGCTTGACGATG

CAGAGCGAAGGCTCCGCATATGTCGCCACTCTCCCCGGCGCTCCGTTCTCCGACGGCAAG

ATCTTCGACCCGCTCGAGTTGTCCGACGGCGCCGAGCCCGGAGACATCAAGAAGTGGCGC

GAGGCTGAGATCAAGCACGGCCGTGTGGCTATGCTGGCGTCCCTCGGCGTCATCGTCGCT

GAGCAATTCCACCCTCTGTTCATGGGCCCCGACTACATCGGCCCTGCCGTGGACCACTTC

CAGGAGATCACCGCGCAGTACCCCGAGTTCTGGGTGTTCTCCCTCCTCGGCATGGCCCTG

GTGGAGTACAAGTCCATCACCACCGCCTTCGCTGAGCCCAGCCCAATCACCGGCGAGGGT

GGACTCAGGGACGACTACACCCCCGGAGACCTCGGCTTCGACCCCCTCGGCCTCAAGCCC

AAGACCGAAGAGGCACTAGCCACGATGCAGACCAAGGAGCTCAACAACGGGCGCCTGGCC

ATGATCGGCATCGCCGGCATGTTGGTGCAGGAGCTCGTTAACCCGGTCAACATCCTCGGC

TAA

>g25033.t1

ATGGTCGCTACCCAGATGTTCCTCGGTGCTGCCGCCACCCTCATTGCGTCCTCGACCGCC

TTCATGGCGCCGATGGCCGTTCGATCTGCTGCGCCCGCGTCCTCGTCGAGCCTGTCGATG

CAGAGCGATGGCTCCATGTACGCCGCCACCCTTCCCGGGGCTCCGTTCTCCGACGGCAAG

GTTTTCGACCCTCTCGGCTTGTCCGACGGCGCCGCGCCCGGTGACATCAAGAAGTGGCGC

GAGGCCGAGATCAAGCACGGCCGTGTGGCCATGCTGGCTTCCCTGGGTGTCATCGTCGCC

GAGCAATTCCACCCTCTGTTCATGGGCCCCGACTACATCGGCCCCGCCGTGGACCACTTC

CAGGAGATCACCGCGCAGTACCCCGAGTTCTGGGTGTTCTCTCTCCTCGGCATGGCCCTG

GTGGAGTACAAGTCCATCACCACCGCCTTCGCTGAGCCCAGCCCCGTCACCGGCGAGGGT

GGACTCAGGGACGACTACACCCCGGGAGACCTCGGCTTCGACCCCCTCGGCCTCAAGCCC

AAGACCGACGAGGCACTCGCCACGATGCAGACCAAGGAGCTCAACAACGGGCGCCTGGCC

ATGATCGGCATCGCCGGCATGTTGGTACAGGAACTCGTCAGCCCGGCCAACATCCTCGGC

TAA

>g25037.t1

ATGGTCGCTACCCAGATGTTCCTCGGTGCTGCCGCCACCCTCGTCGCGTCCTCGACCGCC

TTCATGGCGCCGATGGCCGTGCGATCCGCGGCGCCCGCGTCCTCGTCTCGCCTGTCGATG

CAGAGCGATGGCTCCATGTACGCCGCCACCCTTCCCGGGGCTCCGTTCTCCGACGGCAAG

GTTTTCGACCCCCTTGGCTTGTCCGACGGCGCCGCGCCCGGCGACATCAAGAAGTGGCGC

GAGGCCGAGATCAAGCACGGCCGTGTGGCCATGTTGGCTTCCCTGGGTGTCATCGTCGCC

GAGCAATTCCACCCTCTGTTCATGGGCCCCGACTACATCGGCCCCGCCGTGGACCACTTC

CAGGAGATCACCGCGCAGTACCCcgagTTCTGGGTGTTCTCTCTCCTCGGCATGGCCCTG

GTGGAGTACAAGTCCATCACCACCGCCTTCGATGAGCCCAGCCCCGTCACCGGCGAGGGT

GGACTCAGGGACGACTACACCCCAGGAGACCTCGGTTTCGACCCCCTCGGCCTCAAGCCC

AAGACCGACGAGGCACTCGCCACGATGCAGACGAAGGAGCTCAACAACGGGCGCCTGGCC

ATGATCGGCATCGCCGGCATGTTGGTGCAAGAGCTCGTCAGCCCGGTCAACATCCTCGGC

TAA

>g25039.t1

ATGGTCGCTACCCAGATGTTCCTCGGCGCTGCCGCCACCCTCATCGCGTCCTCGACCGCC

TTCATGGCGCCGATGGCCGTGCGATCTGCGGCGCCCGCGTCCTCGTCTCGCCTGTCGATG

CAGAGCGATGGCTCCATGTACGCTGCCACCCTTCCCGGGGCTCCGTTTTCCGACGGCAAG

GTTTTCGACCCCCTCGGCTTGTCCGACGGCGCCGCGCCCGGCGACATCAAGAAGTGGCGC

GAGGCCGAGATCAAGCACGGCCGTGTGGCCATGTTGGCTTCCCTGGGTGTCATCGTCGCT

GAGCAATTCCACCCTCTGTTCATGGGCCCCGACTACATCGGCCCCGCCGTGGACCACTTC

CAGGAGATCACCGCGCAGTACCCCGAGTTCTGGGTGTTCTCCCTCCTCGGCATGGCCCTG

GTGGAGTACAAGTCCATCACCACCGCCTTCGCTGAGCCCAGCCCCGTAACCGGCGAGGGT

GGACTCAGGGACGACTACACCCCGGGAGACCTCGGCTTCGACCCCCTTGGCCTCAAGCCC

AAGACCGACGAGGCACTCGCCACGATGCAGACCAAGGAGCTCAACAACGGGCGCCTGGCC

ATGATCGGCATCGCCGGCATGTTGGTGCAGGAGCTCGTCAGCCCGGTCAACATCCTCGGC

TAA

>g25040.t1

ATGGTCGCTACCCAGATGTTCCTCGGCGCTGCCGCCACCCTCATCGCGTCCTCGACCGCC

TTCATGGCGCCGATGGCCGTGCGATCTGCGGCGCCCGAGTCCTCGTCGAGCCTGTCGATG

CAGAGCGATGGCTCCGCGTACGCTGCCACCCTTCCCGGGGCTCCGTTCTCCGACGGCAAG

GTTTTCGACCCCCTCAGCTTGTCGGACGGCGCCGCGCCCGGCGACATCAAGAAGTGGCGC

GAGGCCGAGATCAAGCACGGCCGTGTGGCCATGCTGGCCTCCCTGGGTGTCATCGTCGCT

GAGCAATTCCACCCTCTGTTCATGGGCCCCGACTACATCGGCCCCGCCGTGGACCACTTC

CAGGAGATCACCGCGCAGTACcccgagTTCTGGGTATTCTCCCTCCTCGGCATGGCCCTG

GTGGAGTACAAGTCCATCACCACCGCCTTCGCTGAGCCCAGCCCCGTCACCGGCGAGGGT

GGACTCAGGGAGGACTACACCCCCGGAGACCTCGGCTTCGACCCCCTCGGCCTCAAGCCC

AAGACCGACGAGGCACTCGCCACGATGCAGACCAAGGAGCTCAACAACGGGCGCCTGGCC

ATGATCGGCATCGCCGGCATGTTGGTGCAGGAGCTCGTCAACCCGGTCAACATCCTCGGC

TAA

>g25080.t1

ATGGATGGCGTTACGGGACTGGGGTTTGCGGGACTGCTGGTGACGACGGGGGCGCAGTTC

TCACCGCCTCCGGTGGAAATCCAACACAGATTAACGTGGAGGACGGACGACACCATCGAC

GTCATTGAGAAACGCCTCAAACAGTACAGCGAAACGGCGGATGGCATCCGAGAGTCTTAC

AAGGATGTCCCCTGCAAGGTCGTCGATTGCTCGAAGAGTGACCTAGAGACCTTCGCCGAG

ATCTGCGACTTCGTGGAAGAGGTGGCGGCGAAGAGGGAGGACGGCCTTGGGCCCGAGGGG

ATGAAGGCGCTCAAGGAAATGGGGGACGCCAGAATGTCAGACGTCGGCGAGCTTACGGAG

AGGGACGAACAGAAGGCCTTCCAGGAGAGGCCTACCCTGCTTGCCGCGGCTCAGCGATGC

AATCGGTATAGGGCTACCGATTACATGCCCGTCTACGTTAGAGAAGATCGTCAAGGGGCT

GTTTCCCGAGCCTTCGCGATGGAGCTGAGCCTGTTCCCCGGCACGGCTGTGGAACTCCGG

CCCAGCATTCCCGGGGCGGGGCCGCTCGGAGAGGAGGGGCCGGGGTACGTGCTGGCCCCC

TTCGCGTCTAGCGTGGAGGAGCGAACTCGAGTAATGAGCGGTCTGGTCGAGGGGCTGGTG

GAGACGGGGGCTATTCCGAAGCAAGCTTTGAGGAACGAGCTGCAGGATGTGAGGTCAGTG

ACTGGAAAGCTGAGCGCTAGCGGGGAGGTGTTGATTCAGCTCGAGCGAGCGGCGATGATC

CACTTCGGCGTGCCGTCTTTCGGGGTCCACCTCAACGGATATGTCAGAGCGACCGACGAC

AGTCCGATGAAAGTATGGATCGGCGTGAGGAGCGTGAGCAAGGCCACCTACCCGGGCATG

TGGGACCAGATGGTCGCGGGGGGGCAGCCGGCAGGAATGGGGTTTAAGGAGAACATGCGG

AAGGAGTGCGAGGAGGAGGCCAGCCTTCCCTCCAGCCTGTCGAGCAAGATCAAGTCTGCC

GGACAGGTATCGTACCGATACGGAACGAGGAAAGGTCTCAGCACGAAGTTCTTGTGCGTT

TTCGACCTGGAGGTCCCGGAGCACTTCGTACCGTACAACGGGGATGGCGAGGTGGAAGAG

TTCCTGCTCATGCCGGTGGAAGAGGCGTTGGAAAGCATCAAGACGGACCTCGCAAAGTGG

AAACCCAACTGCGCTTTGGTCATGATCGACTTCGCCCTGAGGCACGGCTTCCTCGACCCG

GACCACGAGGTAAGCGAAGACACCGCttag

>g25119.t1

ATGAACACCACGGGACCAGACGGGTGCAAGTCGGGGTCTAGGAAGCAGCCGCCATTTTTC

AAACATGACGAAGTCATCGCCCCGTTCGTAGCTATCATCATGGGTCTACAGCACGCGTTG

GCTATGGTGGGAGGCATCATCACGGTCCCGCTCATCGTCGCCGGAACTTTCGACGCCAAC

CTGGGCGCTGAGCAGACGCAGTACCTGATATCGGCAGCGCTGATCGTGTCGGGCGTGACC

TCATGGATCCAAGTGAGCCAGATCAAGATACCCCGGACAGGTATCACGATCGGCACCGGA

CTGATCTCGGTCATGGGCACCTCCTTCACTTTCTTGCCTGTCGCCCGAGACGCCATCGCG

CAGATGAAGGGCAAGTCGGACTTCTTGGACGACGACGGCGAGTTCGACGGCGAGAAAGCT

TACGGCGCGGTCCTCGGAACGTTCCTCGTGGTCTCGTGGGTGGAGATCCTCCTCGCCTTC

ATCAAGCCGCAGGTGCTGCGGAAGGTTTTCCCCCCGATTGTGACGGGCGTAACGGTTTTC

CTGATTGGGGCCGCCCTTATCGGCACCGGTTTCAAGTACTGGGGAGGCGGTGTTTTCTGC

GGGGATAACGTCAGCAGCGCCGACCCCCCTCTGTGCGGGGGCAATGGCGAGGTCCTGCTG

CCGTTCGGCTCCGCCGAGTACGTCGGCCTCGGCTTTTCCGTGTTCGTCATGCTGGTGATG

CTGGAAGTTTTTGGGTCTCCGTTTGCCCGCAGCGTCAACGTGGTGGTGGCCCTCCTGTTT

GGCTACTTGGTCGCCGCCGTGTCGGACAAGGACGGCTTGGACTACGTCGTCAGCAGCAAG

ATCGACGCCGCCGAGCATGTCACCTTCCTGTGGGTCTTCACCTACCCGCTATCGGTGTAT

CCCCCCCTCATCCTGCCGGCCATCCTGGCCTTCGTCGTCACGACGGTCGAGACCATCGGc

gacgtcaccaccaccgccgatgtGTCCAAGCTCCCGGTAGAGGGCCCCGAGCACTTccag

GTGCAGCATGAAATGGGCTACAATTAA

>g25182.t1

ATGGAATCCTTTGACACTCTAACTTCGGCGGAGGTGTCGGCTGTCTTGAAGGCGACGCCC

TCCACGATAGACCCCGATACGGATGCCATCATCTCCAGGACTCAGGACTACTACAACAAA

GAgcacatttttcagttctacaTCCAGGTGTATGGAGGAGAGTTCCTGCATGTGGGCCTG

TACTCCATGTTGGAAGGCGACGACGCCAACTTGCAGGGAGTTCCAAGGATTTCCAAGGCC

TGTTCCATCTCGACGCGGGAGCTTCTCTCCCGATTTTTCCCCAGCGACAGCGATTTTGTG

CCCGAAAAGTGCACCGTCATGGACATGGGCGCTGGGTTCGGAGGAACGGCACGAGTGGCC

GCCAAGGAGTTTGGCTGCAAGGTAATTTGCATCGAAATTTCAAAGAAAGAGAACGACTTC

AATGCGTCCCTGACCAAAACGGCTGGACTGGAAGACAAAGTCATCATCCCTGGGGAGAAG

TCATTTTTCGAAACGGGCGTGCCGGACTCAAGCTGCGAAGTCGTCTTCGCCCAGGATGCG

CTGCATCACGGCGGGTCACAGAGGCACAGGATTGTGGAGGAGGCGGCCCGCGTGCTGAAG

CCGGGGGGGAGAATGGTGTTCGCGGACGTCATGATCTCGGACGATGCCACCCCTGAGGAG

GTGGAGAAGGTGTCTAAGCGTGAAGGCACACTGGAAAATCTGGGCTCGGTGAAGTTCTAC

AAGAAATGGGGCAAGGTTTACGGACTTGAGTTCGTGGATTACGTCGACTATCCTACCGAC

CTCTCGGATCACTACGAGACGGTTTCTGAAGTGATGCTATCTTTTCGCGGTGAGCAGCAG

GGCAAACACGATGAGTTTATCGATCATACGGTGAAGGATGTTCGCACGTGGGCCTCGGGA

GCGAGGAGCGGTCTCATCCGCTGGGGCTTCATGGTGTTCAAGAAGTCGGTGAGCCGTGGC

GAGAAAATTGCTGCGGGAGCTCTTGTACCCGGGCAAGCCGTCCTACATCGACAATAG

>g25224.t1

ATGGCATTGCAATTTCCGGGACGACCCGTGATAGTCCAATGCACGTACATCTCTTGGTGC

GCCTGCTTCGGGACACTCGCTCGCCTCTACACAGACTCCATCAACCCTTCAAACCTCGCG

CTGCAGGGCTCCTTCCTGAGCAATTCGTTGGGCAGCTTCGCCCTCGGCGCCCTCGTCGCT

TCGGACCTTGACGAGGAGAGCATGCCCGGGCTCTACACCGGGCTCACCGTGGGCCTCTGC

GGCTCGTACACCACCTACTCGGGCTGGAACTTAAGGATCGCGAGAGCAGCGCTGCGAGAC

GCCCCCGGCCCCGGAGGCGCAATCGTCGCTATCGTCGCGATCGTCAAGTCGCTGGCGTTT

TTCGCAGCTTGTTTTGTGGCGGGAAGGGACCTCGTGAAAGGCCTGGCGAGTCGCGGGCGC

AGGCTGCGCTGGCAAGGAAACGCCCTAGGGAGCAACAATGCTTCTTCTCTCGGGCGTGCG

ATGGGGCCGATCGGCGCAGTCTACGCGCTGCTCGCCGTTCTGCTTGTTGTGGATGACAGT

CGGACCAGAAGGATCCGGTGGCTGGCGTGCATGTTTGCCCCTTTCGGGGCTCTCGTGAGG

TTTTCCCTGTCAAGGAAATACAACAACAGATGGCGAGAAGGAGCATTTCCAGTGGGTACC

TTCCTAGCCAACGTTTGTGGATCACTGATCATGGGGATCGTATATTATGGCGCCAGACAG

AAAGACATGGCGGGATGGAGTGGTGTCGCTTTGGCGGCCTTCCAGGGCGGCTTCTTGGGC

AGCCTGACGACCATGTCCTCGTTCATGTCGGAGGTCGTTGGCCACCGAGAAAAGCACAGC

CCGGCGGTGAGCTATTCTTACCTGGCGGCTACGGCAATTGCTGCACAGGTGGTGGTCCTC

GCCGTCGGCGCGGCACTTGGGGACTGA

>g25270.t1

ATGAAGGTTCTCTCCACCGTAGGCGCGATGACCGCGTGCGCCTCGTGCGCCAGCGCGTTC

GTTACCCCGTCTTTTTCGCGCACCGTGATCTCTACCCCCAGGACTTCGTCGTCTGTACGC

ATGTCCGAACCCGCggccgacgccgctgctgtggaaCCTGAGGTGCCGGTTGCGCCCCCG

GCGCCCAAGATGTCGGCTTCCATCCCGTTCTTGAAGCAGCCGGAAAACCTTGAGGGCATG

GTGGGTGATATCGGCTTCGACCCGTTCGGGTTTGCCACTATCTTCCCTGCGAAGTTCATG

AGGGAGGCCGAGCTCAAGCATGGCCGCGTCGCGATGCTCGCGGTGGTGGGGTGGATTGTG

TCGGAGGTTGTGCACGTGCCGGGGGCAGCGTACATGTCGGAGAACCCCGTTGacgcgatg

gcggcggtgggcCCCGGGCCCATGTTGCAGATCTTCACCTTCTGCGGGTGGCTGGAGTGG

AACTTCCACAACGGCAAGATGACGATGGACAACATGCACGACAACGGGGAGACGCCCGGA

GAGTTCGGCTTCGACCCGCTCAGGCTGTCCACCAAGTCCGCGGCCGTGCGCGAGAAGTAC

CAGTTGCAAGAGATCAAGAACGGCCGCCTGGCCATGTCCGCCATCGGCGGCCTCGTGCAC

CAGTCGCTTCTTCTGGGCGGCGGCGTACCCTTCCACGcgtaa

>g25926.t1

ATGGTCGATAAAAAATCCAGCTCAGGCTTGGACGTGAGGTCAGCGATGATGCGACATCCG

GAAGGAATAGCGGGAACATCGCTTGGGAAGGGCGTCAAGGGGGGGACAGCACGGCATCAG

GAACAACTAGAGGAGAGGCCGCTTGCGGGGGGCGTTCAAGAGGGGTTCGCGATGAAGCCC

GCCCCTGCTTACGGCGACGCCGTTGGGCATGCTGGGAACAGCGGCAGGAAAGAAAAACTC

CGGAACCATCTGCGGGATCTCAAGCGCCGCCTGCCGTGCACCACGACCGCTAGCGCTTCG

CCGGCCGGTTCACCGACAATGAAGCAAGGAAAAGGAGATGGCatacccatgccccccccc

accgcgatTGTCAACACCGTAGGCAACAGCCACACTGGGGCAGCGAACAATGAGCGAGGG

GACCAACAATCAGACCCCGACCTCAGTAAGCCGACGGTCAGCCCAAACACCTTAGTAGTG

GAGCGGACGTTTCACCAAGGTGGGGAACAACTAATTGGGGAAACAAAGGCACTGCAGCAC

TCGCAACGACAAGGGTTAGGGCTTCGGCGCGGTGAGGGGACCCACACGGGCACGGGAGGA

GTACGTCAGCTCGGGCCCAAAAATGCGGAGGCTGGTCTGGGCGAGACTGTTCATAACGTA

AAGGATGGAATGAGCACACTAGTAATGCACGCGAGCGGTGGTGCTAGTGGGGAGTGCGTT

GACGAAGGAAGCGGCTGCGAAGGGAGCGGTGACGGAATCAGGAGCACAGCGAGCCGGCCA

TCTGAGCTGTTTGATAGCTTGGACCTCTCCGTTTCGGACCCCCGCAACGGCGGCATATGC

ACAAACATATTCACCCGGCGAGAGAACGCGAGTGGAACGAAGGTAGCGGCGAGAGGTCGA

GTGGAGGGTTTCATGCTGCCCGTCGAGGCCACCCCTGGTGCTGATTCCGCCTCTCACGTG

CTGCAACCCCAGGACACCCAGTACCTCGGAAACCCAAAGGCATCCGGCGAGACAGATGAG

GGACGTGCGGCTGAtgccgatgccgctgctgctcccacCTTCAAAAGTAGAGCACGTCCG

GTCGAAGCGGCTCCCAAACAGAGAGACTGTTTACCGGACGTCGGCTGTGCTACGAAAGAC

ACACCCTTGAACTCGCAGCCTTCAGAGCCACGAGCAGAACGTGACCGTGCACTAACCCTG

AGGGAAAGACGACTTGATCGGACGCGCAAAGGGTCCGCGGCTGAGCTACTACCACCAGAA

CGCGCTTTTGATAGTCCGCCAACAGCAACCCACCGGGATCACAATGACAATGGAAGAAAA

CGAGGAAATCGCGAAACTCGAGGCAATCGCAAGCTTCGGGGACAGCCCGGTGTTTCTAGG

CTAAGAGCACCGGGCTGGGGGCGTGACGTCGCCATCAACTCAATCCCACCAGCTTCGCGC

TCCTCAGTGGCTGCAGACAAGGTTGACGTCTCCTTGGCCGCGGGTCATTCTGGCACCAAG

GCACCCGCTTCGACGATGTTTCTCCCCAAGGACCTTCTGACACCAGTCACCGAAGTGGCT

ACAGTGGACGAGCAGTCAGTGGTCTCTTGTTCTACTCATCGTTCAGCGCGGAATACACCT

GCTTCCTCGGTGGCATCCGGACACAATATCAACCATAGCAGGTTTCGTAGTCCAAGCCCC

AGCGGAGATCACCGACAGAGGCACGGGAGCGGGGCCAATCGGCTCCAGTCGCCCGCGCAA

CCAAGCGACAGGAGCAGACGATACCAACAAGGTGTCAGAAAACGAGGGAGTTGGGGCATT

GGTAGCGTTGGCAGTGCCCATTCGGCTCCCGGTGAGGGCACGAGGAGAGCGCCAGGGCTC

GAACTTGCGTCTGGTGTACGGTCGTTTTCCTCACACCGGTCTCCTCAAAGGGGTGCAGAT

GACGGGGTGGATGGGAGGCTAACAAGCCTATCTAGTGGACGCACACGGAGAGGGTCGGGG

GGGCAAGCTGGGGGTGGCTACATTGCCGCCATGAGCCGGCGCGCTAGGTCTATGGCGCGT

TGTGAGGCGGCGAGGCGTTTGGCCGCGGTAGCAAGAGCGACCGACAAGGCTAGACCATCA

ACGCCTATTTCTGAGCGGGCGCGTTGGGGCAAGGGGCGGGAGACTCAGGTGGAGATGGAA

CGGAGGAAGCTCCTGCAACGGCGATCCGAGTACGCGGATGAGCTGCAGAGAAAAGCCAAG

GAAGACGCTGAAGCGCTTGCAGCAGACTACGGCGAGCCCTCCACCACCCTGCACACAGAG

CGTCGCCATATGGTCGCCGCTCTTAGTCCCGCCAGCGGTGGAGGCTTCACCTACGATAGC

CCCATCCACGACGACAACTTGCCCATGGGATGTAACATGGAGATCATCCCACATGCGGTC

GCAGTTAACTCCTATTGCGCCACCGCGGCCCTCAAGGCCGTCGGTGAACCTCAGGACAAG

TTTCACCGCCTTGCGACGGGTGGGCTAGCACCTCCACCCGGCCTGCACCGGCACGACTGC

GATTCAGATTGTGTGGTAAGCGGCGGTAATGGCAGCGGAAGCTCGGCAGGAAAAAGCGAC

GGCGACAGCGTCGCCGGACGACGGCATGATTCGGGAAAACGTTCAGTCGCACAGGAAGAG

ACTGGATCGGGGAGGGCGCcggctacggtagagagggAGGAGCAACAACTCATGGCGTCC

ATAGCTCGGCTGGATGCGCTCCTGAAAGACGACAGGGCTCCGGCCAACAAATCGCATCCC

TCAAgcagaaaaaataaaactcctGCATCAACTTCAAGCGCTAGGACTGGAGATGTTGCG

CACAGGGAAACATCCCGGAGTGACGCGCGAGAGAGGAAACCAGCGCGGTCGGGAGGGATC

ACCCAGACGAGAGTGAGGAGAGGCAGTGGAAAGGAGACACAGATGTGTGGCTCGGCGCGA

GCTGcgccggcggtgccggcgggtAGTAGGTCGAGCGCTACTTCTGCGGTAGAGTCGGCG

GCGGTACCGCCGACAACGGTGACACCTGTGGCTGGTACTTCCGCAGCGATATCGTACCCC

GTTGTGTTTCCCCCGCTAGCGAGGCGAGAGAGAGTGTGCTCGGACGAGACGCGTGAACGA

GGACACTATCTACCCGGTGTCAACAGCAATGGTGATCTTAATATGAATAGTGACAACGCC

AAAGGGGACCTCCGCGGAAGCTCCCGTGAACACGACCGACAACGAGAACCACTACGGGCG

TATGACGGGCCGGTTTCACCTCTGGGAGTCCAAAACACAGACGAAGGACGGGGTTGGAGA

ATAAGGGATCACCGCCCGGAGGTACACGACTGCTACCGACCGCGCGATGTGGTTCGACCG

TCGTTCGCGCCTGCCCGTCGGGCTGCCGAAAACGTTCAGTACGGTAGCGGTGCCCGTGTT

GAAAGCAATCAATACTGTAACGGGGCACGTGTTGAAAACGATGAATACGGTGACGGCGCA

CATATTGAAAACGATCAGTACGGTAACGGTGCACGTGTTCAAAATGACCGATACGGCAAA

AGCGCATCGGCTCCGCGACATCCGAATCGGAGGGATGAAGGATGGACGGAAACGGTCCCA

TGTGAGCCAGCGCGATATCGAGGCGCGATATATCGCGATGATCGTGCCGAGTTGACGGAC

GAGTACCCGTACTACGATTATTGCGATccgagggggaggggcgacAGAGCTAGATCTTGC

CATCGACGCATCGCATCCAGCCCACAAAACGTGCGGGAAGCGCGGGTGGAGGGCAGGGGC

TGGGAGGAAGAACCACGGGAAGACGACCACTTTACAACTGATCGGGATAGAGATGGCCGA

ACAGACTACGACAGGCGGGATGGAGGGCGATATCTGCAAGCACACGACGAACAAGGGTAC

TCTCCGGTATGCAACACCAACGCAACAACGAGTTGA

>g25984.t1

ATGGAGCAGGCAACCGCCAACAAGGATATCTCTATCCTCTTCGACTTCGATGGCACCATC

GGAGACACGGAGACCCCGGCTATGGAGGTGGCGTTCTGGGAGCTCGCCCCTTACCTCCCC

GATACCACCCCCGACAAGCTGGACAACTTGATGCCCGAGTTCGTGAGGGACAACGCCGGC

AAGGCTTTCGAGTTCATGGTGGAGACGGTCGACGAGGAGCGCAAGGCCGCGGGGATGGAC

AGCGTCGAGGAGATGTTTGCCGAACGCAACTCGTTCGGTGCCTCTGTCGCGGCACAATCA

TTTTCTGCAGCGGTACTGGCGGCCTTGACCGCGGCGACGGTGCCATTCTGCATCTCTACT

ACCAGCCCCAAGCCGAGGGTGCCAGCGTCCATCACGGCCTGCGGCCTTGACGAGTACTTC

CCCCCCGACAAGGTGCACAGCGGAGAGAGCGACTTCGACCCCCCTCGCTTCAAGCCCAAC

CCGTCGGTCTACCTCAAGGCCGCCGAAACCGAGGGCAAGGAGCCCGTGAACTGCATCGCG

GTTGAGGACAGCGGTTCTGGCGTGGGTTCAGCTTCGAACGCCGGCGTCGGACTCACCGTG

GGGTACGTCGGGGCGTCTCACATTCCCGAATACAAGAAGGACACGCACGCCGAGATGCTC

ATGGCCGGGGGGCGCGCGGAGAACGGGAAGGGCGCGGAGATCGTGATCTCGGACATGAAG

GACCTGCTGAAGATCATCGACTTCTTCGCGGGCGCGAAGACAGCCGGAAAGTTGGCGCCC

TTCGATTTCCCGACGGCCATGGTTGCCTCCATGCAGCAGCCGGTGTGGGTCCACGGCAAG

AAGGCCTAA

>g26196.t1

ATGGAGATATCTCTTGTGCATCTTCCTTTTCTGCTGTCCTTCCAGGTTGCTGTCATCGTA

TCCATCATCTTCTTCGTCGGAATCGGGATAAGCACCATCTTCTTGGTCAAGGGCAAGGTG

GACAATTTCTTCGTGGGAGGAAGAGACTTGCCACTCTTCGTCGTCGTGCTCACGCTGGCG

TCCCAGAGTATCGACTCCAACGCCACGCTCGGAAATGCCGATCTCGCCTACAAGTTCCAC

TACTGGGACGGAGCTGTCCTCCCAATGGGGCTCGGCCTCAGCCTGGTCATCAACGGCCTG

TTCATCGCCCGCCACATCAACAAGGCCCAGTGCCTCACCCTCCCGGACTTCTACGGCAAG

GCCTGGGGCCCCGCAGTTGAGATCGTGGTGTCCCTGCTCACGTGCATCTCTTTCATCTGC

CTTCTCGCAGGAAACCTGGTTGGTCTATCGATCATCATCCGCTTCCTGTTCGGCGGAGAG

CTGGCCACGAGCGTCTTCATTGCCGGTATCGTCACCATGATCTACACCGGCGCTGGGGGG

CTCCTTTCCGTCGCCTACACGGACGTCGCCCAGGCCTCCCTCGGACTGCTGGGTCTGCTT

GCTGCCGCGGGATGGATGCTCATCAACAGGGACCCCACTCACCCGCAGCCGAGCGTTGGC

TTCCCCGGGTACCTCTACCCCGATGACGCCACTTGCGAGACGTACCAGGGAGTGCCTGCC

GTGGAAACGGAGGGAGCGTGCATGTACAACGAGGATTTCTGGGGGTCCGCCGGAGTGGAC

AACGGTGCATACCCCTTCGGAGACAAGAAGgtgttCAACGACGGTATGATGGACATCGAC

GCGTACGGCCCTTTCCCCAACGCCATCCTCTTTAACTGGGCCACCATCTTCGTGCTCGGG

TTCGGCAACCTCGCCGCGCTTGACTTCCAGGCCCGGTGCATGGCTGCCAAGACGCCCAAG

ATCGCCACCATGGGTAACCTCATCGCGGCAGGCCTAACCTTCATCGTGGGAAACACCTTC

ACCTTCATGGGTGGCTACACGCGTCTCAACTACGGCCCCGACAGCCGGTTCGCCTCTTTC

ACGGTGGACACGTGCTCCAAGTTCCTCGACCTCCCCGCCTGCGCCGCATGGGAGCCGGAC

GCAACCGCGTTCCTCAAGCTCACCACCACTCAGCTGCCGACATTCCTGGGGTGCTGGGTG

CTGATCGGCATCTGCGCTGCCTCCATGTCCACGTCCGACGGTGCTATCCTCGCTATCTCC

ACCGTACTCTCGCACAACATCGCTCGCAAGGCCATCCCTGGAGGCGAGCGCTTCACCGAC

AAAAAACTCCTCAACATCGTGCGGCTCACCATCATTCCCATCACCCTCATCGCCTGCATC

GTGGCCAGCGCGTACAACGAGACCGGGTACCTGCTGATTGTTGCGTTCGACGTTGTGCTG

GCGGGCTGCATCGCGCCCCTGTTCGCCGCAATCTACTTCAAGAAGACGGTCACCCCCGGA

GGTGCCCTGGCTGCTGTTCTGTTTGGCTCTATCCTGCGGGCTGTCCTAGAGTTCGCTTTG

CCTAAGGATGGTTCGCTTGTCATCCCACGCGGAGAGTTCAACTTCGACTACGGTCCCGGG

CAGATCGGGCCTCTGCCTACCTTCATCGACGCCCCTGCCACCGAGCAATGGGACCCCGAG

TCGTGCGAGCAGCCTCGCCTCGAGGACTGGACGGGTCTGGACTCTCTCCTGTCCCCCATC

GTCAGCGTCATCGTCATGTTCACATGGTCCTTCATCGAGAGGAAAATGGACCGCACCCTC

TGGACGTGCCTACCGTCTCACTGGCTCGACCCCACCTTCACGCTCAACAAGACTCTCTCC

AAGTcTTCGGTCATGGAGACCACTGGCAGTGTGCCCGCGGTGCCTGTCAGCTAG

>g26300.t1

ATGGACGACCTCAAGTACGCGCTGGGAGCGGTGTGCCTGTTCAGCTTCGCCGCTGCTGTG

GGGTCGGGGGCCGCCATCGGGGGCAATGTGGGCGCGTCTTTCACGTACTTCTTTGCCCTT

CTTCCCATCGTCTTCCTGGCCATTGGGTCCACGGCCCCGGGCATCATCACGGCCGTCATC

AACAACGTCAGGAGGAAGTCGCAgGATGACTTCGAGGAGCGACGCGTACGCCACGAGGCT

TCGCACTTCCTGTCCGGCTACCTGTGCGGGCTGCCGATCAAGAGCTACCGCGCCGACGGG

GGCACTACGCTCGTCGAGTTCTACGACTCCGCTGAGGGCGACATGGCAGGGAGGGCGCTC

AAGTTCACCGCGGACGAAGTTGACATGGACTTCAAGGTACCCACCATCTTCCGAAGCTTC

AACTCCCACGACTCCATGAGGAGAGGGACTAGCTGGAGCATGCTGTCGCTGGGCGCTTCG

GGAGCGGGGCTCCCNNNNNNNNNNNNNNNNNNNNNNNNCCACCCTCCCCCTTCGGAATTT

GCTgtcgccacccccaccgccgctctTGCACGGGCCCAACCCGCCCCCGGTGTTCGCTTC

AGGTGGGCGACACTCATGGCACACCGGCTGCTGTCTAGTCACGAGCCCGAGCTTAACTCG

GTGATGGCGGCTTTCCGCGAGGGGAAGCCGGTGTCGGAGTGCGTAGCCGCCTTGGAGGGC

GCGAGGAGCTGA

>g26744.t1

ATGGAACCTGCCTACGAACACCTTTTGCCGCGCAACTGGGACAGCGTTGTGCAAGCATGG

CTTGATGAGGACATTCCTGGCTTCGACATCGGTGGGTACGTTGTCGGCAGCAAGGAAGAG

ACGGCAGTCCTCTATGGCAAAACTGATGGCATCATGGCGGGGAGGCCATTCTTTGAACGC

GTTTTCACTCTCCTTGGCTGCAAGGTGGAATGGCTCATGCGCGACGGCGCGCAAATAGAC

ACGAGTTCGGTCGAGAATGGCAGAGTGCCGGTTGCGAAAGTGACGGGGGGATGTCGCGAT

ATCCTCCAAGGCGAACGGACAGCGCTCAACGTGATTTCTCGTTGTAGCGGTGTCGCCACC

GCAACATCCAAGGCTGTGCAGCAGGCCAAGGCCAAGGGTTGGAAGGGGTACGTCGCAGGC

ACGAGGAAAACCACCCCAGGTTTTCGTCTTGTGGAGAAGTACGCCCTCGTGGTCGGGGGC

GGTGCTACTCACCGATACGACCTGAGCCAAATGGTGATGTTAAAGGACAACCATATTGCT

AGCGCTGGGGGTATCACAAGTGCCGTGGAGACCGCCAGGCGGGCAGCTGGGTTCTCTATG

AAAATCGAGGTTGAAAGCAGCACTTACGAGCAAGCTTCTGAAGCAGCTAGAGCCGGGGCA

GACATCGTTATGCTTGACAATTTTGAGCCAGACAACCTGAAGTCTGTGTCAAGCAGGCTA

AAGGAGGAGTTCCCTAACGTGACCCTGGAAGCCAGCGGGGGGATAACCGCAGAGACTTTG

CACCTCTTCTTCGTCGACAGTGTCGATGTCATCAGCCAAGGGGCGCTCACCCATGGGTAC

GATTGCCTCGATTTCTCCTTGAAAATCATCGCACAGGGCTCGTAA

>g26812.t1

ATGGCACCCATTTCTCCGACCACCTTCGTTGCGATGCTGGCTCTCCTGCTGAGCCCCGCG

TCGGCCTTCGTCGCCCCTACCGGGTTGGCTGGGCTGCGGCACCAGCCCGGGTCGTCGTCA

TCTTCGTCCCGCTTCGCTTCTTCCACAACGGCAAACGCTCGCTCAAGCCTTCGTCCGTCG

CGGACGCCGTTCGCTTCCGTTGGACTCAGCATGGTCAGCGAGATCGCCGAAGAAAAGGCG

GCCAAGCTGCGTGAGACAGCAGCCAGGTTTCGCGAACAGGCGGCCGAGCTAGAGGCCGCG

AGGGAGGTGGAGCGCCGGGCTGGCGCGGACAGATCCTTCAACACCTTCGACTCCAACAAA

GACGGGGCCGTAGACATCGCCGAGTTGAAGGCCGGCCTGGAGGGCCCGCTGCGCAGGAGC

TACGTGAAGCAGCTGACCGCTCGGATGGGCCGCAAGCCCGACAAAGACGAGATTGAAGCC

CGCATCGCGCAGCTCCCGGGCGGCTCCCTGTTCCCCGACAACCTGGCGCGGAAGCTGATC

TCCACGTACGACGAGAACGGCGACGGCATCCTCCAGAAGTCCGAGTTCGTCCCCTCGGAC

GAGCTGAGGACGAGGGTCGAGAGCATGTTCCGCGAGCAGCGGGACCAGGAGCGCCTGGCG

CGGATGGAAGAGCGGCAGCAGGAGATGGAGGACAGGCTCAAGTCGCAGGCTGGGGCGGTG

GCCGcgtcgggcgggggggcggcaggCATGAACGACGGCGCGCCGACGGCCGCGGACAAG

GTCCTGTCCGCGCTTCCCTACGTGCTGCCGCTGGCCGACAGCCTGGCGTACGCGGGGCAC

GTGTTCGCGGCCTTTCCCGACCAGATGGCGTGGGCACAGCCCCTCGCCGGCGCGCTTCTG

GCCCTGCGGTCCCTCCCGTTCGCCACCCTGGTGGCCTTCTTCGGCCTGTCGACCCTGTCC

AGCCTTCCGCAGGTGAACAAGCTGGTGCGATTCAACATGCAGCAGGCCATCAACCTGGAC

ATCGCGCTGATCTTGCCCGGCGTGCTCGGGGCTCTGACCTCGGCCTCGCTCGGCCAGGAT

GCGTACAAACTGGTGCCGTTCACGCAGGCCGGGTCGGACGTGGTTTTCATAGcgatgttg

gtggcggtggcgtacTCGGTGGGCGCGTCGGCCGGTGGGGTTTTCCCGAACAAGCTCCCG

CTTTTGGGGCGGATGAACCGCGAGAACCCCGACCGGGAGCAGGAGGAATaa

>g26823.t1

ATGAAGTCCTTTGCCGTAGCAGCCGCTTCCTTGTGCGCCCTGGCGCCGGCGTCGGCCTTT

GTGCCCGGAGCGTTGCCCCTTGCCGGCGTGCGCAACGCACCCGCGAAGCTCTCTATGGCG

GCCACACCCGACAGGTCCGTCGCGATCCCGATCGACCCTTACCCGGAGGGCCTTGACGGC

GAGATGGTGGGCGACGTGGGCTTCGACCCGGCTGGGTTCGCGAACAACCCTCCCTCGTGG

GTATCTGGCGACGCGCCGGGCACCGCGGGCCGGGTTAAGTGGTACCGCGAGGCCGAGCTC

GCGCACGGCCGCGTTGCCATgctcgccgcggcgggctggaTTTTCCCTGAGATCTACCAC

TTCCCTGGAAACAACGTGCTTGGCGCAGACCGGTTCGCGGAGACTAACCCCCTCCTTGCC

TACGGCAACATCCCGAACGCCGGAGGCATCCAGATCGTGGTCACCATTCTGATCCTCGAA

ACCATCAGGCTCAACCGGAGCATCCGTACCAACGACGGCCCCGCCGGGGACATCGGGCTC

GGGCAGGGGGAGGGCCGCTGGAACCCGTTCAACTTTAACTACACGGAGGAGGAGTACGCG

GAGAAGCAGCTCCAGGAGATGAAGCACGGCCGCGCCGCCATGATCGGGATCACGGGCATG

TACTTCCAGACTCTTGTGCAGGGGAACGGAATCCTTCACCAGCTGGGCGAGGCGTTCTCC

GTGCCGTCCGACGTCGCGAAGGCTGGCTACTTCTTCCCTACCGAGGGCCTTTAG

>g26901.t1

ATGGCGGTGGTATTGCTCATTGGCGCCAGCGGCGCCATTGTGGGGTTCCTGTACACCGAG

GAGCACCGAAGGCTCGAGGAGTCAGAGGCGCCGCCTGAGTGGACGTACTTGCAGGGACAG

GAGTTGGAAACGCCCTACGGCCCAACCGACTGGGGGGAGTCTTTCGAGGACTGCTACGGT

GACATGCAGAGCCCCATCAACTTGTCGGAAGCGCACCAAGTTATCAAACACGCCGGCGAG

TACGATTTGGAATTCCACGCAGAACTGTGCTCGAGCGAGGAGCTCAACTTCTCTCCGGGA

GAGCACCAATGGATGGTGAACTTCGCCGACTGCACGGACAGATCATCTCTGACGTTTGAC

GGCGACCACTACCAGCTCCTCAACGTACACATCCACTCCGTTTCCGAGCACGAGAACGGA

GGTGCGTGCCACGACGCCGAGATCCACATGGTGCACGTGCGCGAGGGCACGGACGACGAA

CTCCTCGTTGTAGGCGTTCTTCTCGACGCGTCGATGTTCGGCTACAACAACGAGCTGACG

CCGATGTGGGAAGTTCTCGCgaagggcgaggaggagggcgaCGAAGAACACGAATTCTCC

TTGTCTCCCTACGAGATGCTCCCGGCGAGCCGCGCGTACTCGCACTACATGGGCTCTTTG

ACCACCCCTCCGTGCACCGAGGGCGTTAAGTGGATCGTCATGACCGACCCCACGCTTCTT

GGCCTTGGCCAGCTCACCACCTTCCGCGCCGCCGTCGGCTCTCACATGATGGTTGACTCG

TTGGGCAACACGAACCGACCTGTGCAGCCTCTCAACGGCCGCGAGGTCCACTACGTATCA

GTCGCATAA

>g27335.t1

ATGCGATTCAGGTACGTGTTTCTGGGGCTGGTGACGCTGGCGGCCATCCACACGTCCAGC

GGCCAATGCAGCAACGACATTGCGGGGGTCCAGAATGGCGATATCTGCTGCGATGCTGGC

TGCGGGACATGCGGTGGAAGCGGTTGCGGCGGACGACCGGGCGGAGCGGATAACTGCTGC

ACCAGCCGCATCAGGAACAGTGGCGATATGTGCAGCGACACTGACGCCGCGCCGTGCATC

GTCGATACCCAATCCGTGTGCGACAACGGCCTATCCGGAGTACAAACtggcgacatctgc

tgcgatgccGGCTGTGGGACATGCGGGGGGTCCGGTTGCGGCGGACGACCTGGCGGCTCG

TCCAACTGCTGCACGAGTCGGATCTCGGAAAGTGGCATGATGTGCGACACCACCATGGCG

GCGCCCTGCATCGTCAATGGTGTGTCTCGACAAAGCGTTGCTCGACCAGATACCTCCGGG

GTTCGTGCCGATACCCTTTCCGAATCCGGGACCATTTCCAGTACCATCTCCGATAAGCGT

TCCAACGCTAGCCCCAACGGCTGCGCCAACGGCTGCACCAACGGCTGCACCAACGGCAAT

GCCAACGGCAATGCCAACAGCAATACCAACGGCAATGCCAACTTCAGCGCCAACGACGGC

ACCTTCCACGGCAATACCCACGGCATCGCCCACGGCACCACCTACGTCGACGCCCATGGC

AACGCCGACAGAAGCACCGGTACCTACTTGCAGCAACGGTCTCCCCGGGATTGA

>g27373.t1

ATGGATGTCTTGCTCGATCTGCACAACGAAGCCAGGTGCCTACACAACGCAGACGCTCTA

ACGTATGACGACGGAGTGGCCTCCTCTGCCATGGCCCACGCTAAAACTCTGGCAAGTAGT

TGTGGAGACCTTTACCACAGTGACAGCGACGACCGCAACGACTATGGCGAGAACCTTTAC

CTGTGCTCGTGGAATGGCACGACATCTTGTTACACGCCCGAGGCGGCTATGGCCTCGCTA

TACGAAAGCGAAGTACAGGTGGACACCGTGGCCCAGTACGGGGAACATGCGACACAGATC

CTGTGGAAGTCGACCACAGAGATGGGATGTGCGGTGGAGACTTGCCATCTGGATGGTTAT

CTGTACGCATACGTTGTGTGCCAGTACAATCCAGCCGGCAACATTGTGGGCAAGCTTGAA

GAAGAAGTGGAACTGCCGTCTGTAGACTTCTGTTAA

>g27451.t1

ATGGCAGCGGACCAGGATGACGAAGGGTACCTGACCTGGCTGTCGAAGAAGGTCGAGCGA

GCGCAGAGACCCCCCTTTGTAAAGATTGCGAGAGCTCGGCTGACGAGGGATTTCGCTGTG

CTATTGATGcgcacctcgtaccagGTGTGCGACGAGCTTGACTTCGTGCCGATGGACGAA

TTCCAGAAACAGTTCTTCCTCCTCCGTCAAGACGAGTGGTTGGAGTACAAACAAAACTTC

CCCCGAATAAAGCAAGGTGACTTGACCGACCCGGACTACTTCGACTTCATCAGCTTCGCG

CAGTACGCCAGCGTGGCCATGGCCATCCGAAACGGTCGAGACATTTTCGAGGAGAAAGTG

GGAGCGGAAGGGGAGACAAGGACGGTGCAGCGAGACCCGAACTTCAAGGATAACGGTCTG

CTGCCGGACGAGCACGCGAGGAGGGTGGGGGATAGAATTTTGGCCTACTGTACCGAGACG

TTCGGGACGACAAAATTAGCGGCGGTGGTTGGCCAGGGACTGTCGGTGCCTGAGCTACAG

GAGGGGTGCGAGCGGATCATGAATCTGCTACGCCTCAACTTTTACTTGTTAGACTACACT

GTGGAGTCGGACCTGAGTCGCGGCATGATCACGGTTAAGGCAACAGCCCCGGCGACAATC

TGGGGGCAGCAGGTGCTGATGCGGAGAAGAGACAAACCTACCAACGATTTTGAGGCGAAA

GCTGTGCTCGCCTACCTTAGGGCGTGCGGAATTGGTGGCGCCTCATACTCCACTGCGTTC

AACGGCCTGGATGCCATACACACCTTCCGGCTGTGA

>g27457.t1

ATGGCCAGACTCAATATAAGACTGTGCTTTAGGCTCCTCCCTGCGGCCGCTGTGGCGGTA

ACATCAACAGCGGCTAGAGGGACCCAGCGCGCGatataccaccaccatcgccggcCTACC

GCTGCCTTTGGCTGGACGTCTCGAGGTGTTTCCTCGGGGGACGCATCGGCTGAAAGAAGC

GCGCGTATACAAAAGCTGCAAGGCACAGCCAATAGCACCCAAGATGTCCAGCTCCAAAAG

CTGGCAGACGCTGCACCATCGCTGCTGGCGAACGTCGTTGCTGTCGCGAGCGATGTGGAT

GGCACACTCACGACGCCGGCCGTGACTATTACGCTCCGCACGAAGCAGGCCATCAAGGCG

GTGATGGACTCCGGACTCGTGTTTTTTCCGGCGACGGGTAAGACCCGCGCCGGCATGTAC

AAAATATTCGGAGAAGAAATGAGCTCGCATCTCAAGGCGAACAATGTCCCGGGTGTGTTC

ATCCAAGGCTTGCTGGTCTACTCCGGTTCCGGAGACGAAGTGCTGTACGAGCGGCTGCTG

GATGCTGATATTGTCGCACAGGTGGCGGCTTTCTGCGAGGAAAAGGGGGTTTCTCTCATA

GCCTACAGCGGTGACAACATTGTGTGCTCGAAGAAGGACGCCCAGACGGACaagattgct

ctgtactatgagCCCATGCCGTCTGCTGTTGGTCCTCTCGACGAGGCGATGGCTGCAGGC

CTTCGGGTACACAAGCTGATCCTGATGGACACCAAAGAGGCCATTGATCGAGTCCGACCA

GACGTGGAGCGATTGATCGGCGATCGGGCATCATTTACACAGGCCCTCCCCGACATGTTG

GAAGTACTACCGCCAGGAGCATCCAAGGGCCACGGGGTAGAAGTGCTACTGAAACACCTT

GGAATAGACCCCCTTCATTTGATGGCACTAGGTGACGCCGAAAATGACGTTGAAATGTTG

GGTCTCGCCGGCGTCGGGGTGTGCGTTGGAAACGCTTCCCCCCCAGCTCGAGCGGCGGCC

CGCTTTGTGGCATTGACAAACGCGGAGGATGGATCCGCTGTTGCCATGGAGCACTTGCTG

AGCACCAAGTCGGCAGGAGTGTAA

>g27561.t1

ATGAAGACCTCGTCTACCCCCTGTCTAGTGCTCATTGCTGCCCTCGTACGCGAGGTAATT

TGCCACAAGACCCCCCCGCGGGCAGACGTCCCGGTGGTGGGATTGGCCTCCGTTGCACCG

ATCGGAAGCAACGCGTCAAGGCGTTTGGCTCCGTCATCCTCCACGCGTTCTAGTAGCGAG

CCACGGCCAAGACCGTCAGGCCAGGCGGACCTGATACAGGCCCTTGAAACCGACAAAGAA

GTGACAGTCTTGGGATCGGTGGAAGTCGGAGAGCTGGCGTGCCACACCCTGGTGGTGGCT

GAAGACGCATCAACGCTGTCCTTCATCACGCACGAGAACGAGTCAATAGTGGCCGAGCGA

TCGTCCTACACAACCACCAGCGGGGATGCGTTCACGGACGGCATAATACGCAAGGTCTGG

ACCGGGACCGTTATCGACCCCCCACAAGCCGAGGATGAGGCGGTTCCGCGTACGGTCAGC

ATGACGTGGGGCGAGGTGTGCGACACCGAGACGTTTCTCCTCAAGGTCACGAAACGGAAC

AACGATGGAACATCCACCATCCTCAAGACCGTTCCGTGCGACGAAGGAACCACGACGCAC

ACCTGCCTGGTCGAGGTGCTCGTTGACTTCGACGAATCGAGCGATGCGGTAGCCCCTCAT

GTGGCAATCCCGACAACTGACACCTCGTCCAGGTATGTCGATCATGCCTCCGCCTTCTCG

CAGATCGATGTCATGATTCTATACGACGACCCGGCCTTGGCGACGCTGGGCGGAATCACC

TCCAATCAGATGGAGACCATCATTGCGGAGAGCATCGTGGAGAGCAACCAAGCATTGACC

AACTCGGAGGTGTCGGCCTACTTGAACCCCGTCCACATCGGCCCGCTGCCGTTTGAGCCG

TTGACATCCAACGACCAGACCACGCTCTTGCAGCTGCAATTTAGCGACGACGTGGCGGAT

CTTCGTGACGCGACGGGAGCCGACCTGGTGCAGTTGATCGGCGACTTTGACGATTCCTGC

GGACGAGGGTACCTTTTCGTGGATGACCCAGACTACGCTTTCTCGGTGGTTCACGCCACA

TGCATCGACAACTTCTCCCACACGCACGAGCTCGGGCACAACTTGGGGTGCTCCCACAAC

CGGGAGGACAGCACCGAAGACACTGATTACGCTCACGGGCTCCGCTACTGCGACGGGGAC

AACTCGTACCGGACGTTGTTGAGCTACTCCTGTGGATCTCCCCGAGTAAATTACTTTTCT

AACCCCGACGTGGAGTACCTCGGCAAGCCGACGGGTACCGCCACAGAGAATAACGCCCAA

GCCATCAAAGACAACATGGTTGCGGTGTCGAATTATAAGTCCCTTGCGTCTGAGAcggca

tactcgtacctggggtGTTTTGCAGACATCATCGAGGAACGCGTTCTGTCGGGCGATTTC

GTCCTGACCCACCAAGCACTAACGGTGGAGTGCTTTTGTGGGTCTCCCAGCGATAATCCT

GATGATCTCGCCGATGCCACGTGCGACCACGAGTGCGCCGGGGACCCATCGCAGATCTGT

GGGGGCTCGAAAGCTATAAGCGTGTACCAGTACTTCGCCAGCACGGGGGAAAACGTGGGC

TGCTGGAAGGACGCCGACGTCGATCGCATTATGGACACCGTGCTGGTAGAATCCTCCATG

ACGAACGATTGCTGGTGCGGTGGCGCCTCAACCGACTACACGACCCACGGAGAGTCCACC

GAGTGCGATGTCGTATGCGTGGGAGATATCGACACGTTTTGCGGGGGTAACTTTTCAATG

ACAGTGTGGGAGCACTAA

>g27624.t1

ATGTCCGGGACGAGCGTGGAGTTGATGCTTGCACACCTCAGAGTCGCAGCAACACCTGGT

ACAACGCCTGTCTCGCACCCACGCTCGCCGTTCGAACCCGAAATCGCGGCAACCTTATCG

AAGGGGGGCGGCgatggcggaggtggcggcgatggcggtggtggtggtggtggtggtggt

ggtcgtggtcgacgtggtggtggtggtggtggtggtggtggtggtcggagcTTTATTTCT

TCTCGCACTCCGCCTCAAACCAGCGAAGcgcgcagcagcatcagcatcagcagcagcagc

agcagcagccgcgcagAAATCTCTCGGAAATGTATGCGGCGGCTCACCGTGTCTCTTGCC

GTCGCGGCGCTGCTGTGCTTCGCGGCGTTGCTTTCGGGCCTCGCGGGGCTCTGGAAATGG

CAAGATGTGTTTCCGTACAGGGGAGCCACTTTTAACGCGAGCAAGAACTGCCGGCAGGTT

CATACGGGCCCGGAGGAACCCTGCAGCAACATTTTGGAGTCGACCTGCTTCGATCGCAGC

CGGTGCCTCGGGAGCGGGGCGGAGGGGAACTTGCTCTCGGTCTATGTGCATGACGAGACG

TGCTCGATGAAGAGCAGCTCGGACGTGGTGGTGAGCTTTAAGGGCGAGGCGGTTTCGAGC

ATGTGGTCGCGAGCGGCCGGAGCGTTTCGTCAAGCCGCCGAGTCaagGGGGGTGCTCGCG

GCGACGCCGGAAGAGGCGTGCATCGTTGTGTACGTGATTCCAACGAAGGGAGAATGCGTG

TCGCAAACGCCCACGTGGGGGAAGGGGCAAAACCACCTGCTGGTGGACATGAACGACCAA

ACTCGCGAGCAGCGCTCCAGGCTGGACCACAGGGCAATGTTCGCGGAAGCCAACATGCGG

CCGTGCTACTTCCGCTACGGCTACGACATCGCGATACCCTTGCTGGCCAACTCGATTTTT

CACCACTTGACGGGAATCGCTCCCCGGGATCGCAAGTACTTCGCCACCTTCAAGGGGACC

TTATACCTGAGCGGAAACGGTTTCCTGGAGCGGACTGCCGTGCGCAACCTCGGCGACGCT

GCGGCCAACGACGCCAGCGTTGTCGTTGTGGAGAAGTGTCAGACCCGGCACAAGGAGCAC

CTGCAGTCGCGAAACGTGGACCTCTGCGAAGGCTTGCAGCGGGAGTACGACAAGGCCGAC

TACAGCGACCTCATGAACACGACGTTCGCGCTGGTACCTGCCGGGCGGTCGCCGGCGACG

TACCGGCTGGGCGAGGCGCTCTCGGCGGGCGCCATCCCCGTCTTCATCCACCAGAACTTC

GTGAAGCCGTTCCCCGGGAAAATTCCCTGGAGcacgttctcgttctcgtttcCGGCGGAG

GAGGCGCCGCGCATCATCGACACGCTCAGGGCggttcccgtcgaaaaactcgcgAAGATG

CAGGCTACCGCGTTGAAGGTGTTCGACAGTTACTTTGGGCGCGATATGAACGGTACCGTG

CACACGACGCTGGACATTTTAGAAGAACGGCTGTCATTCCGCGGGAGAAGCTGA

>g27689.t1

ATGAAGACGGCTTTGGCTTttatggcggcggcggtggccgccgAGGCTTTCGTGCCTGCT

TCCAGCTTTGCCGGTAGCGCTATGGTGGCAGGTCGCGTGTCGAGGTCATCCACCTGCCCT

CTGTCCATGGCAGAGAACAACGCTGCCTTGTCCAAGATGGACGAGATGGAGAAGGAAGTC

GCGCCTCCCGGGGGAGGGTTCTGGTTCGGTTCCGCGGGTTTCTGGGGAGAGGAGGACTAC

CGTGGGTTCGTGGACTCGTACAAGCCCGACAACCTCATCAACGGAGTGTACCCGATCATC

GACCGCGTCCGCGACACCAAAATCCTCACCACCACCTCCGAGTCGGGTCTTCTGTCCGAG

CTTGAGGACGCAGGCCTGACCCTCAGTGAAGCGGAGAAGCTCCTCCCAACATTGGAGGAG

GCGGGCCTCCTTACCTTCGCGGCCGACAACCTGGACGTTCTCATCAACTTGTTCGGCTTC

CTCGCGATCGAGCCCGCGAAACTCGCCATCCCAGCGgTGGTGAGCGTGGTGAAGGCCTTG

AAAGGAGCGGGTCTGCTCAAGATCGGAGGCGACTCCGGCGCAccggccgctgctgttgcc

tccGCGCCTTCCAAGCCCGGACGGGCGAAGGCTTCCAAGGCCCCGAAGGTCGTCGCCATC

AAGGCCGTGAAGGCCCCTAAGGCGGCTGCCCCTAAGCCTGCTCCCAAGTCGAAGCCCGCC

AAGGCTGCCCCTAAGGCTGCTGTCGCTAAGGCTGCccctaaggctgctgctgctaaggcC

GTGAAGGCTGCCGCCCCGAAAGCCGCGCCCAAGGCGAAGCCGGCTCCCAAGGAGGCACCC

AAGCCCAAGGCGGTAGTGAAGAAGGCAGCAGCAAGCCCAGCGAAGGGGAACATCTCCAAC

CTCCCCAAGGCTCTCTGA

>g27793.t1

ATGACGGCGTTGCACGGTGGTTCTCCTTCACATAACAGCCCAAATGAGGCAACCACGCTA

CGAGAGACAGCCAAGAATCAGGCGCACagggccaacagcagcagcagcagcagcagcagc

agcttcagcagcagcagcagtgtgctacCCTTATCCTTTGTAGTCGCTTTCTTGTGGGCC

ATGCTTTGGGCGGGGCCCGGTCGGGCTGTGGGAACGCCAACATTGGACGGCTATCGGGTG

GTCCAAACCATCAACATTGTCGTAATATTTACCATCTCCGGCATGACGCTCAAGACAGAG

GACACCGAGAAGGCCACAAGCAAGGAAGGGATACTGGGCTTTGTCTACGGAGTCGTGGCC

ATTCTCGGCATCACGGCATGCATCGGCTTTGTTGCCGTGCAAATCCCGTTCGACGTCAAG

GAGTTCTCGTACGGCCTGGCGGTTTTCTGCGTCGTCCCGACTACATTGTCCGCTGCAGTC

ACCCTGGTCATGAATGTCACAGACAGCCGTCAAGGCATCTTTCTGCTCGACGAGAGCGGA

GGCGAACAGCACAGCATCACACAGGCGGCGTCGGACGAGCCGGAACGTTTGGCGACGACG

GTCGGTGATATGATGAGCGATGGTTTCCAACTGTCCGGCGATGGGGCGGGAGGCAACGAT

GCATTGGCGCTTTTGCTGACAGTGACATCGAACTTGCTAGGGGTTGCAACGGTCCCTTTT

TTCGTCAAGGCTGTGCTATGCGTGGGAAACGCATCAGCAATCGACAGCGTCGATCTCTTC

AAAAAGCTGATCATCTCCGTTGTGGTTCCGCTACTGGGAGGGAAACTATTTCGAGAGACT

GTGCCGGGGGCGAAGGGCTGGGTGCAGCGATGGAAGACGCCGCTGAAATTGACGAACGTT

TTCATGTTCGCCATGATCGTGTGGCAAATCCTCAGCAGGGCTCACGACGAAGTAACAGCC

CTGGGCTTGGGGCAAGCCGTCCCCTTAGTCGTGGTATCGATCCTGTTGCACGTCATGTGA

>g27831.t1

ATGCCTTCCTTCCAAGCGACGGCGCTTGCCGTGTCTTGTGCGGTGCTGGCAGCCTCGCCT

AGCTCGCTAGTCTCGGGTTTTGTCGTAGCCCCTAGTTCCTCTTCCAGAGGCCTTGCTGGG

GCTCGTGTGGTCGCGTCTGAGGGCGTGGCCAAGGCCGCTGTTGGGCGCAGGTCTCAGGCG

CTGATGAGCGACACGTTGCTCGACAAGGACAAAGAGGAGGTGACGGAGTACTTCAACAAC

AACGGCTTCGAGCGATGGAACAAGATCTACTCGGAGTCGGACGAGGTCAACGACGTGCAG

CGGGATATCCGCACCGGGCACGGCCAGACCATCGACAAGGTCTTGCGATGGGTGGAAGAG

GACGGGAGCGGCAAGAGCTTCTTCTGCGACGCGGGCTGCGGAGTGGGTTCCCTGACGATT

CCGCTTGTGAAGCTTGGAGCAAAGGTGGCCTCGTCCGACATTTCGGCGGCCATGACCAAG

GAATGCGAAGCAAGGGCGAAGGCCGAGCTAGGCAAGGACGCCAAGCGGATACAGTTCACG

ACGTCCGACCTGGAAAACCTCACGGGAAAGTATGACACCGTCCTCTGCATCGACGTCATG

ATCCACTACCCCACCGACAAGATGTCCGACATGGTGAGCCAGCTGTGCAGCCTGTCCAAG

GGCCGAGTGATCCTGTCCTTCGCGCCCAACACGTGGTACTACTCGATCCTCAAGAAGATC

GGCGAGCTGTTCCCCGGCCCTTCAAAGACCACGCGGGCGTACCTCCACACGGAGGAGGCC

GTGAGGAAGGCCCTGGGAGACGCGGGGTTCGCCGTGAAGAGGACGGAGATGACCTCCACC

AACTTTTACTTCTCCCGCATGCTCGAGGCCGTGCGCGAGTAA

>g27996.t1

GGTCGAGAGGCGTCGAAGCAGTCGCACGTGTCCTCGATGATTCATGGCTCGGTGCTGGCG

CGCACTTCGCTGGAGGACGCCATCACCTGCGACGTGGCGAACAAGATGGCCACCACCTTC

CTCAGCCCAACGGAGGTGAGCTCGCTGATGTGCGAAATGTTCACAGACGAGCCCCTTCTG

GACTTCGTCGTGGCCGAGGACCTTCTGGCGCACGCCATGCAGGACACCTCTCTACCCGAC

GTCCTGACGGCCATGCTCTTCCACAAGGGCTTCACCGCCCTCACCACCTACCGTCTCATG

AACTGGCTGTGGCGCAGGGACAGGCGAAATATGGCGCGGTACCTGCAGTCCATCTGTTCT

GAGgtaTGTGCCGCGGATATTCACCCGGCGGCTAGGATAGGCAAGGGCATCCTCATGGCC

GGGGGCTGTGACATAGTTATCGGGGAGACGGCCGTGGTTGGAGACAGCGTGTGCGTCTTG

CATGGCGTTACCTTGGGGGGCACGGGGAACCAGACGGGGGACAGGCACCCCAAGGTCGGC

AAGGGATGCCACATCGGCGCTGGCTCGTCTATCCTCGGAAACATTCGCATAGGAGAGGGC

TCGCGCATCGAGGCCGCCTCCGTCGTCGTGAAACCCGTCGCACCGTACACGGTCAACAGG

GGTGTTCCATCAAAAACGGTGGCGTTTGTCGTGTGCGACCCTCACCAAATCGCGGGGGCT

GGGGTTGGGGATGAGGTGGAGAACGATGGAGATGGAGATGGAGAGGGTTTGCAGGgcgag

gaggcgggggcgggttcAAAGAAGCTCCCGAGGTTGTTCACGAACTACGGGTCGTTATAC

ATTGGCGTGTGA

>g28001.t1

ATGGTAGCgtatagcagcaacagcagcaccagcagcggcagcagcgccagcaacaGTAGA

GGTAAGCCTCACCTGACGGTCAGGCTGGTCCTCGAGCTCATGTCCGCCGACTGCTGCGCC

TCTCAGCGCTGGGGGACTGCCCCGGACGCAGCTTTGGCcacccttgctgctgctgttgag

acaCACGAGGAGTCTCTTCGAGCCGCCCTGCAGAGGTGCTTGCTGGCTGCGTCGCGCCTC

CACTTGAACGACTCAGCGGGCTCCGGCAGAAGACGAATGCAAGCGATGTCCTCAGCATCA

AGCAGCATGTCTCCTGCGATGTCAACCACGAGACACGTCAGCAGCTGCTGTGACGAGCAA

TCAATAGGGTCTGCGGATTCGTGGGATGAggaacaagagcagcagcagtgggaagATGAC

TGCGATTACCTGAGGGAGGACAGGGGTCGCCTGGGAGCGGCTGTGAGATCCAGAGATCTG

TCTCTTGACGACGAGCGTTTGTCTGCCCTTGCAGACCATCCAGAGGAATGTTGCGGCGGG

TCATCGTGcAGGGAATCGAGCGCGTTTAGCGAGGTGGAAGACTGCCAGGAGCTGACCGGC

GTGGTGGCGGCCATTTACGGCTACAAGGCCAGCGGCGGGGACCTGGTGTTCTTGGAGAAG

GCATACGATCTGGCGAGGGGCGTGGCCGCGTCGAAGCAGGACCTCTCCGTCGCCTTCAAC

GCGTGCCGAGTCGCGCTTGCCGTTGGGTATGTCTACACTTTCAAAAGCGTCTCCTTGGAC

AGCGCTCGCTCGTTCGCCCTCCACCACAGCCTCCCCAAGCTAGCGCTCTCGCTCAAGCAC

TACTGGTCGCGCGGAACCACCGAGCTGAACAAGCAGGCCAACGAGCTGAAATGGCAGGAG

TGCGTACTGTCCCTGCTCGAGGTGGGATCGAGTGTTTCCCTCTTAGCCAACGAGGAGGAC

GTCTTCTGCGCGCTGGATGCTGACATCCGAGTGGGCGTCGTAGAGGCGATTGGGTTGTAC

TGCGTCTTGAACCCGCGTGTTCTGTCCCAGTACCAGGGCGTAGGACGCACGTGCAAGGAG

AGGTACCTTGCCATTCTTGGCTTCGACCAACGAGAGCAGACCAAGGGCAAGGTCCTCTAC

TCCACCTCCAACTCGAACAACATATCTGCCGTAACGGAGCAAAATTGGTTCGTGGAGTCC

GAATGA

>g28299.t1

ATGCGTTGCCAGGCCCTCGAAACGTTGCTAGTCTTGTGGGTAGCTACCCTAGGTGATGCC

TTCACAGGCGCACCGTTCTCGTTCAACGTGCGAAGCGGGAGCGGCGCTTCGTCACTGAGT

AAGACTCATGTTTCTATGTCTACCTCGTCACAATCAGGCTTCGGAACGCGGGGGGATATG

ATCAAGGCAGCCGGGGTATCAGTTGCTGGCGTGCTTGGCTCAGCGATGCTTCCTGCGGGA

GCCCTCGCAGCGGCTGACCAACAGCGTAAAGTTTTCACAACAGCTGGCGGCGTAAAGTTC

ATCGTCTTGAAAGAGGGCAGCGGGCCCGTCGCGAGGGACGGTGACTTCTGCGTTGTGGAT

TTCACAGGGTTTCTCCCGGACGGCAAGGTTTTTGACGCGAGCAATGCGCCGGGCCGGAAA

CCTATCGCCTTCAAGCTGGGCGCGCGGCAGGTCATCAAGGGATGGGAGGAAGTGCTGCGC

CTCATGAATGCTGGCGCGGAGGTGCAGATGGTTGTGCCGGCCAACATGGCCTTCGGTGAC

AAGGGCATCTGCATCGAGAACGGAGACTGCCTCGTCAAGCCAGGGATGGAGGTGCGCTAC

GACCTTCAGCTCAAGCGCGTGGCCCCCACCCCTTAA

>g28453.t1

ATGGAGAGGGAGCCCCGTCCTTCTTCTCGGTCGGTGCAGCGGCCTTTTTCTCCGCGGTGG

GGCACTTGCCCACCTTCCAGCCGGAACAGCGGTCTCCCTCCGGCACTCCACTCAAAGCGC

TGCTCCACCGCGATGCGCCGCGATGTAGCCGCGAAGCGCCGGGAagccaacggcggcggc

ggcggcggcggcggcggcggcgggCCCACATCCGCGAGGTCTCGCAGAGTATCCGCTGTC

GTCATGAGCGAGAGCTCGGCGGCAGCCTCGGTGGCTGTGTACAAGAAGTTCATCGGGGGC

AAGAGGTGGGACGCGGTGGACGACGCGGAGGCGAGGGCGACGGGCGTGTTTGAGGAGATC

TGCAACGTATACGGCGAGGAAAATGCTGTCAAGATGGTGAAGAACTCGCCGAGCTGCCTC

GGGTACGACTCGTCCTTGTTCAGGGCGACGTTCGAGGCTTTCTCGGAGGTGTTCACTCCC

GAGGACACCAAGGGGATGGTCACGCGCAACCCTAACCTCCTGGCCGTCCGGCCGACCGGC

TTCGGCGGCGCCTGCAACGCCAAGTCGGACACCATGCAGATGAGCTACGTCATCGCGTTC

AcgagGCCCCTGGGTCCCGTGCTCCTGGGGGGGCTGTTCTTCCTCCTCGCCATTCCGTGT

ATCGAGATTGCCACCGGCATTCCTCGAGAACAGTTCCTCTCTTCGATCATCTCGTAG

>g28531.t1

ATGGCGTACAGAGGGATGCGGACAAGGCTCTTGGGTGTCGTAGTCGGAGCGGCGACGGTG

GAGGGGATGTCTCTCGTAGGCAGGAGCAGCTGCTTGAGGATGATGTCCACCTCTTCCTCT

CTCCGAATGATGGCGACAGGCTCTGTGGTAGACGCGGATAGGTACGCGGAATTCATCAAG

GATAAGGCGAAGTTCCCGAACCCTCCTAAGAGGCTGCCGACGCTGCTGAAGGTTCTTGAG

CACAAGGGGCTGTCTCCCGTGGACCCCTCCGAGCGAGCGGGCCTTAACCCGTTCTTGGTG

CCGCTGGCAAAGGACGAGCACGGGCTGGTCACGGGACTGCTGCGATGGCCGACCTCTCCG

GAGACCCTGGAGATGCCGGTGGTCCGAAACGGGGAGACGGGGCTGGAGCTGCTGGCGAAT

AACGCCGAGCACTTCATTAGGAGAGCCGTGGCGGAGGAGGATTTCGCTGGGGGGGCAGAC

GCTGAGGCAACACACGTTGTCAACGAGCTGATTCGGTTGGGGAACGAGGGCCTGCCTGAG

GGAGACCCCGTGTACGAGAAAGGCTCGGTCGAGAAGCTCGGACGCGGGATAATGAAGTAC

CACGCGCTAAGGATCGGCCCGTTCGCGGACGTTTACGAGTGGCTTACGTCCAGCCACTTG

GAGAAGGGGGACTACACCGCAGCGCTCGCGTCGgccgagcgggcgaacgagatcttcgtg

gggtgggggaggcccTACGGGCACTACGCTAGGGTTCTGGCCGGCTTGAACAGGAGAGAG

ATGGAGGCGAGAGACGCGGCCAAGGTATCGCTCCGGTGTCCGTGCTGGACTATCTCCCCC

AACGCCGCGGACTTGGAAGCGATCGTCAAAATCGCAGGGCACGAGGACGTGGCGAGCGTC

AGGGATATGTACGCCGGGATGGCGGCGGACGAGCAGGCCGACAAGATCACGGAAGGCAAA

GCCCCCATGCAGGTGGCGCTTGACCGAGCAGCACATCTGATGGACGCCGTTGCCTTCGGC

CACAAGGACTGGGATGGCGTTCGCGAGGAGCTCGCCGAGAGGTACGATCAAGGAGGAATC

CCCGAAATCGCCCAGTTCGTGCGGATGGAATAG

>g28606.t1

ATGGGGGGCGAAGATTCTAAAGATCAGACGAAAGCTAAAAGGGAGCCGTGGGAGTTTGGC

CGTTTCGTGAGAACGCTGCTTTTCTTCCAGAGCCCGCGCCGGCCGCAGCTCCCCTTCTCA

CGCCGATCCCGCGCCGCCAGAAAATCCCGCCGCGAAGCGCGAGCGAACGGCGGCAAGTCG

GCCTCCGTGCCGGACTCAGTGGAGGGGAGCGGCTTACCGGGGCACTTgactagggggggg

gtggtgttggtcacGGGGGCGACGGGTGGGGTTGGCCGGAGGGTAGTGAAGGAGCTGAGG

AGCAGAGGGGTGCCCGTCAGAGCGatgGCGAGAAACCGTTTGAAAGCGTTGGCGATGCTG

ACGGATGGGTTGGAGCCGTCAGAGGGGTCTGGCCTTGACCTCGTTGTGGGGGATATCCGG

GACAGGTCGTCGCTCACGCCGTCGCTGTTTAAGGACGTTGCTGCGGTGGTGTCGTGCACA

GCCGCGATCGTGCGCCCGAAAGAGGGAGACGGCCCGGACAGGGCTAAGTACTTCCAGGGC

ATCACATTCTACGAGCCAGAGGTGGCGGACGTGCCGAAAGAGACGGAGTTCGAAGGGCTT

TCCAACCTGGTGGAGGCGGTGAGCAGGTACTCCGACGTCAGCGGCAAGACGCTTTACAGC

TGCCTACCTTCCTTCCAGGAGGGGTGGAAGCAGTGGGGAGCGCTTGACGACGTGGTAATG

GGGGGCGTCAGCGAAAGCGTCCTCCGCGTCGTCCCGGGTGCGGGAGAAGCTGCAGTGGAC

GGTggggtcgcagcagcagcagcggtattcAGCGGGGAAGTGAAGACGTCTAACTCTGGC

GGCTTCGTCTCCATTCGCACCAAAAACTTGTCCCCCGCGCTGGATTTGTCACGCTACAAC

GCCCTCAGGCTGAGAGTCAAGGGAGATGGAAACCGTTACAAGTTCAGCATCTATGACTCC

CCTGGGTGGAACAGCAAGTCCTGGTGCGACTCATTCGACACAAAGAAGGGAGAGTGGATG

GACGTCGACATTCCTTTTAACGCCCTTACgtataactttcgcacgtcgtctGTGAAGGAC

CCCCCGGCGTTCTCTTCCAGCACCATCAACTCCTTGCAACTCACGCTCTCCAAATTCGAG

CTAGACGGCGATCTCAACCCGAACTTCTCCGCAGGGCCTTTCGAGCTCACCATTTCTTCC

GTCAAGGCGGTGACCATGAGCGGGGAGGAAGACGAGACCCCTCGCTTCGTGCACCTCAGC

TCCGCGGGAGTCACAAGACCTGGAAGGCCGGGGCTAGACATCGAGGCCGAGCCTCCCGCG

GTGCGCATGAACGACATGCTGTCGTACCTGCTGACGTACAAGCTCAAGGGCGAAGACGTG

GTGCGCGACAGTGGCATACCCGCGACGATCATCAGGCCCTGCGCTCTCACCGAAGAGCCG

GCCGGGGCGCCGATGATCGTATCTCAGGGAGACAACATCAAGGGGAAGATAAGCAGAGAT

GACATCGCGGAGCTGGTTGTGGCGTCTCTGCTGTCCCCGCTGGCCAAGTCGCTGACCTTC

GAGGTGAAGTCTGACCTGGCATTCAGCACTCTGTGGGAAGGGCCGGCCGAGGGAGACCAA

CCTCGGGATTACGGGGACATATTCCGGCCTTTGGAGAAAGGGCTAACCGGCAAGGAATGG

ATGGGCGATCAGTCGCCTGAAGAGGTGGCGGGGGTTAACACACCTTAA

>g28835.t1

ATGTCGCTCCCCAGAGCGATGACAGCAGCGGCGGTCCTCCTGCTCGTGTCCGCTGCCACC

GTTGTCCATGGCCAAACCCAGCGCTTCTGCGAGGGTGAGCTCGGGCAAACGGACTGGGCG

TGCGAAACGCTCGCGCCCATTTCCCCGGATGTATTCGAGGAGTTCTTGCCTCTGCCGGGC

ACCCCCGACCCTGCAAACCCCGCGACGTACAAGCAGATCGCGGGCGAGCCTGCGTTCGAC

GAGGTTACCGGCCTGAGGAACGATAAGGCAGCGGTGTTCGCGGTGACGGAGGGGATATAC

TTCTGCATGCTGGCGGAGAGCAACGGCGAGGCTGTGCTCTTTGACGCCCCGGAAGGGGGC

TTTGTATCGAGCTCTACGCCGTTCCCCACCAGCTCATGGCTTCCGGAGTCAGTTGCGGCT

CTGCTGGCGGAGGGAACGAAGCTCAAGTACATCGTCTACACACACACCCACTGGGACCAC

ATCGGTGCCGCGGGCCTGATGGCGGACTACTTTGCGGCCGACGAACCCCAGGTGATATCT

TCCCGCCGCCCACGGTGGGTactgaagaacaaaaacaagagcgaccccaacactgcgttC

GGCAACAACCGCGGCGTGCCTCTGCCCGAGTGGTACCGGGAGGACGTCCTTGAGGTGGGC

GGCCTTAGGTTCGTCTTGGACAAAGCTCACATCCACCAGGCGGGGGACTACTTGATCTTC

CTCGACAAGACCGACCCAATTAACGCCGATgcaggtATCGCAACCAGCATCGTAATGGTA

GCCGACGCCATCTTCCCAGGCTGGGCTCCCTTCTTCGGTATTTCTGGCGCCCAAGACGCC

CAAGGCTACCTCGAGGGGCTCAACCAAGTTGTCGCCATCGACTTCGACGTGCTCATGGCT

GGTCATCTCACTCGCCTGGGAACGAGGGAAGACGTTCAGGTCAAGGTCGACTTCTTCGCC

GATGTCCTTGCGGGTGCCCAGCTAGGCCTTGCATCCGTCTCTGCTGCCGACGTCACGGCC

GGCATCGGCCTCAGCGACCCGAACAACCCTAATGCGGGCAACTCTTGGCTGATCTTCAAC

GAGGTATTTGACCGCGTCCTCGACGTATGCTACGACTACGTCGTGGACTCCTCTGCTCGG

GGGCGGGACTGGCTCACTGAGCTCGGGGGGGTCGACATTACGCTCCGCTCTCATACGGAC

GACAACACGCACAAGGGGGGAGACGACTTCCCTGATCTGGTTCAGACCACTCACAAAGGT

GCCCGCCACTCCTTACGTGCCGAGGCACTTTTGTAG

>g28864.t1

ATGAGCAGGAGGCGAGGGGCATCGCTCGTCGCCTTGGTTCTGGCTGTGACGGCCGGCTCC

GGGACTGCCCTGTTGGCTCCTGTCGGACAGaCAAGCATGGGCCGATCGAGCGCCATCACG

GGCCGGGGAGCCGTCGCTAAAGCTCCCCTGGGGGTAAGACCTGTCGAGCTCGGAGGAGCg

aacggcggcgccggcggcgctgGCGGTGGCGCCTTCAGACAGCAGAGGCAGGGGAGGGCG

GTGGTGAGGCCGGACTCCGTTGTCCTGAAGCAGTCGTTTTGGGACAGCCtagcgggcgga

gggggggcggggggggcgaagaaGAAGCCGGCGGCGGGGTCGAAGGGGCAGAGCTTCAAG

TCGCCTCTTCCGAAAGGGTTTCCACCAGCCAGTACGCTTTTGAGACCGACGGTGGACGAC

AGCCGCGGACATTTCCTCGACGTTTCCGTGGAGAAGTTCAAGCCCAATGGAGCTCTTAAC

CTTGTGGGGAAGGGGCAGGTTTTCGGCAACGCGGAGCTGGACCTTTACGGGTGCACCAAC

GCGCTTAAGACCGCGGCGCACGACCCGCGAATCCGAGGAGTGCTGATGGAGCTGAACGCG

GAGGACATGAGCATGGCGACCATCATGGAGattcGGCGGGCGATGGACTACTTCACGCAG

AGCGGAAAGCCTCTCTGGGGGTTCACGGAGAATGCCGTGGACATGACCCTCCTTTGCCTC

ATGGGTGGCTGCACAAGGattcGGCGGGCGATGGACTACTTCACGCAGAGCGGAAAGCCT

CTCTGGGGGTTCACGGAGAATGCCGTGGACATGACCCTCCTTTGCCTCATGGGTGGCTGC

ACAAGGAGGTTGGCAGCGCCGGGGGTGTACGTAAACCTGATCGGATTTTCCTTCAACTCG

GAGTTCTACAGAGGCTTTTTCGACAAGGTCGGCATCGAGCCTCAAGTCAGGCGCATCGGA

GAGTTTAAGTCTTTCGGAGACGCCTACGCTAGGAACTCCATGAGCGCGGCCCAAAAGGAA

GTCACGGAGAGCCTGCTCTCGGCCGTCAGCGGGTTCAAGGCGGGGCTGCTGGCCcgagac

gcggggggggggaagacgatGGAAGACGTCGAGAAGCTCTACAACGGCGATgAGCCGGTG

ACGGTGGAGGCGCTTCAGGAGTTCGGCCTCCTGGACGGCGTGTTCTATGAAGACCAGATG

CTGGAGTTGCTCAACAACGAGATGAACAAGCGGTACAGGAAGGAAGTGGCGGGCAAGCCG

TGGAACCGCGCGAGCAGTGGCACGAAGGCAACCAcgaagggcaagaagagtacgAGCACC

gtcaagaagaaaaagaagagcaaGCTGATTAACCCGTCGAAGACCATCATCCCGGTCACC

ACCTACCTCCGGAGAACAAGGaggaACGAAGTGGAGGGTGTGCCGGGGAGAACGGGCGAC

AAAACCATCGCCGTCATCAACGCAGAGGGAGCGATCGTAAACGCGGCTGGGCCGGGAAGC

GGCGCCAATCTTCAGGTTGACAACTTCCGGGAGCAGGCGAACGCTATCCTCAAGGACAAC

GATGTGGACGGAGTGGTGGTGCGCATCAGCTCGCCGGGCGGGGACGCTCTCGCTTCGGAC

CTCATGTGGAGAGAGGTTCGGCGGTTGCGTGAATCGGGCAAGGTGGTGGTCGCCTCCGTC

TGCAACGTGGCCGCTTCGGGCGGCTACTACATCGCCATGGGATGCGAGACCATCGTGTGC

GACGAGCTCAGCATCACCGGAAGCATCGGCGTGGTGCAGGCAAAGTTCGCTGCGGGGGAA

TTCCTCGACAAGATTGGACTGAAGATCGAACGAGTGAGCAAGGGCAAGTACGCTGAGCTG

TTCGCCGCGGAGAGAGGGTTCACCCCTGCAGAGGACGCGTACTGGAGCAAGAACGCTATG

AACAGCTACGAGGACTTTGTGGCGAAAGCTGCCATGTCTCGAAGGATGCCCCTCGAGGAC

ATGAAGCGGAGGGCGCAGGGGAGAGTCTGGACAGGGTCGCAGGCCAAAGCTTTGGGTCTT

GTCGATGAACTGGGGGGACTGGACAGGGCCGTGGAGACCGGAAGGGTTACTGGTCGTGAC

ACGGTCATGACAGCACCATCGGAGCCCCCCAACTCCGTTGTCGCCTGCTACAACTGCGGC

GAGGACGACCACTACAAGCCGCCCCTTGTAGTGGCCGTCATTACCGCAGTGATGGCAGGT

GTCAACGGAGTGTGCCGCGACATGGTGGACTTGAAAAAGCTTGCGACTAAGAAGATGGTG

GCGGCGGAGAGAGCAGCCGCTGCGGCGATGAAGAAGAAGGAGCGCGAGGAAGGAAAGGGG

GCTAAAAAGGACGCACCAGCAGCTAAAGAGGAGGTTGGTGCGGAGCTTAAGGGTGACGTC

GACAGCACCAAAAACGCCTCCATGGCAATTGTGAAAACCGAGAAAACGGAGGAGGCAAAG

GACGGTGACGACGAATCGGAGATTGTCCTGTCTGACGAGGACATCCCCTTCGGACCACTG

GGAATTTTCCCGGAACTTTACTCGGATCTTCAGGCGCAGGTGGTCCAGGAAGCGAAGGAT

GAGATTCGGGCGCAATCGGAGAAGGCCTCGAAGGGGGGCAAGGAGAGGCAGACGCGGGTG

GTCCAGGAAGCGAAGGATGAGATTCGGGCGCAATCGGAGAAGGCCACGAAGGGGGGCAAG

GAGAGGCAGACGCGGGTCAAGAACATACAGCCCAGCTCGGGAGTTTTCTCGTTGTTGAGC

TCGTCCAGTCgacaggagggggggttggatgATATAGTTAGACGCTTGGGGCTGGCGCCA

TCGCCTCAAGCTGACAAGCCGTCTTCGTCGAATTTGCTCAaggggtTGGGGTCCTTGATG

TCCCCGGAAGCTGCCAGGCCCATGGCCGTCATGGACGACGACCTCGCGCGATCGCTACCG

GGAGTGAGCGGAGTACCTCAAGACCTTAAACGACTTGGGGTGGGTCCCCTGCTGCACTCC

TTCCTGCTCCGATCTCCCGGGCTCGCAGAATCGCTGGTGGAGACCCTTGGCAAGGTCGGG

CTTTCCTCGCTGCTGCTCGACTGA

>g29038.t1

atggagaggGGGGGTGACCGTGAGCACGATTTCGTGTTGCCGCGGTTGGCGCGATGGTTG

GAGGCCATCTGTATGACCGCCTGCTCCCTCGGCCTGCTGCTCGTGGTGGTGAAGTGCTTG

ACAAGGCTCATCGTCGGCCCTGGGTCTGGCTCTGGTTGCGGTCTAAGGACTGAGTGGTTT

TGGGCGTCTATCGGGGAGAGGGGTGATGTCGAGTCCGCCTTACAAGAGGAGAAcacgggc

ggggaggggggggacaaagGGAAGAAGAACGGCGGGTACTCGGCCTCGTTTGCGGACATC

CTGGGGCTGTGCGCGGAGGATTGGCAGCTCATCCTGGTCGCATTTGTTACTCTGCTGCTG

GCCGCTGTCTCTCAGGTGCTGATTCCGCACTTCACGGGAAACATGATCGACAACGTGGTG

GAGTCAGGAGACAAGGCGGCCTTCCGAAGGTCTGCCCTTTACCTCGTTCTAGCCGCCATC

GCCTGCGGCGTGTTCTCGGGCATAAGGGGGGGCATTTTCACCGTTGTCGGGGCGAGGGTC

AACGTGAGAGTGCGGCAGCGTCTGTTCGAGTCGCTGGTTCGACAGGAGATCGGGTTTTTC

GACACGACGAAGACGGGCGACTTGACGAGTCGGCTAGCCTCAGACTGCACGAAGGTTGGG

GATCAGGTCACGCTTAACGTCAACGTTTTCCTGCGCAacatggtgatggtagtggtgacC

CTTCTCTTCATGTTCTATCTCTCATGGCGACTCTCCTTGGTTGCCTTCATCTCCGTGCCG

GCGATAGTCGTAATCTCCAAGTGGTATGGACTCTACATCCGAAAGCTTGCCAAGCTTTCC

CAGGACAAGCTGGCGGAGGCCGGCTCTGTGGCAGAGGAAAGCCTGGGCAGCATGAGCACC

GTCAGATCTTTCGCAGCCGAGGGGAGAGAAAGCAGAGAGTACGCCAACACGCTCCGGgaT

TTCTACTCGCTGTCGTTGAAGGCGGCTTACGCTTACGGGCTGTACGCGTGCAGCTCCGTC

CTCCTGCCGAACCTCGTGACGGCCATAGTTCTGTTCTACGGCGGTACCTTGGTGATGCAG

GGACAGATCACGGGAGGGAAGGTCGTGAGCTTTATGATCTACCTCACGTCCCTCTCGGAC

GGCATCAACGATATGGCCTACATTTTTTCGTCCATgaCCCAGGCCGTAGGAGCGGCGGAC

AAAGTTTTCGAGTTGATGAGGCGGGAGCCAAAGGGGGccgcgccgccaccaccacccaca

cctGCCCAgcgaacggcagcagcagcatcagcagcagcaacattaccatcgtggtttggg

gggggggtaggggcgggaAACCTAGGAGGTGAAGCGCCAGAAAGCTGCCTAGGGGAGGTG

GAGCTTCGAAACGTGGACTTCGAGTACCCTTCAAGGCCCGGAAAGCAGATTCTGGACGGG

ATTTCGTTCAAAGCTGCCCCCGGTCAGGTGCTAGCGCTTGTTGGTCCTTCTGGAGGCGGC

AAGAGCTCGTGCATCGCCCTCCTTGAGAACCTGTACCAGCCTTCAACGGGACAGGTGATG

TTGGATGGCATTCCCGTGCACGAGTATAACCACCACTGGCTGCACAAAAGCATCTCCATC

GTGGGCCAGGAGCCGACGCTTTACGCCCGCAGCATTCGGGAAAACATAATTTACGGCCTT

GAGGACCAAGAGCCCAGCATGGCCGAGGTCGAGGAAGCGGCGAAGTTAGCCAATGCCCAC

GTTTTCATCTCCCAGCTGCCGGACAAGTACGAGACGCAggccggggagaggggggtgcag

ATTTCCGGCGGACAGAAGCAAAGGATTGCCATCGCGAGAGCCTTGGTTAGAAAACCGCGG

GTACTCCTGCTTGACGAGGCTACCAGCGCGCTGGATGCCGAGAGCGAACATCTGGTACAG

CAGGCGATCGACCAGATGATAGCAAGGGGAGGCATGACGGTCATCCTCATCGCGCACCGC

CTCAGCACGGTCAAACGGGCCGACAAGATCTGCGTCATTcggggggggaagGTCGCCGAG

GAAGGCACCCACGAAGAGCTGGTTTCGGTGAAAGGAGGTGTCTACTCCGCCCTGGTCAAA

CGACAGCTCGATATCGGAGACAGTGGAGGCAAAGAAGACATCGGGGTTAGCACCCCAGCA

TCAGTCAGCTTCAAGAACAGGAAGGCATTATCTTAA

>g29089.t1

ATGAAGAGTATCGCGGGATCTGCGGGTGCCGTGGCCATTATCGCCTTCGGGCTTTTTCCC

GAAGCAAGCGGGGAATTGCTGCGACAAGAGCCGGCTGACCACGGCCTAACTGGCGGTGTG

GGTGACACAGGGGCGACATCGACGAAGGCCCTCGACAACAGTCTTTCGCGTCGTCTCCAA

AACAGAGCACAACCGCCCCCGGTCGTCAACGACGATGCCCTCGATGTTAATGGCACGCCC

GCAGAAAGGGCTGCCAACGCGCTAACCCAGCGGATTGAATGGGCGGAGACCGAGTTCACG

GCATCGGAAGGCACGCTTCATGAAAACAATGGAGACCTTTCCTCCGCCGCCACGTTCCAC

AAGTCGCTGCCACACGACAGCCTAGGACAGGTGGACAGCGAGGCCTTTGATGCCCTCACT

GAGTGCATTGCTCAAGGAGATTTTGACACTTGCGAGCAGGTGCCGGCCGGAGACGAGGAC

GGCTTCCTGGTCAACCCCCTCGGGGGTCTTGCCAATGACATGGCAGGGCCCGCCGGGCAC

GCCCTTACCATCCCGAGCGCTTCAGCGCTCAACTCCGAGGACCTTGCTGCTCAAATGGCG

GAACTGTACTGGATGGCCTTGACGAGGGACGTACCTTTCTCCCAGTACGGCGAAGACGAG

GCAACTGTGGAGGCAGCAGACAACTTGGCCACCATGCCGGGTTTTGCTGACATGAACATG

GTCGCCGTCGGCTCGGATGGCAGAGCCGACCCGCAGACGCAGCTGTTCCGTACTTCTGCC

CTCGGTGTCGAGACCGGACCTTTTGTTTCCCAGCTACTCGTGAAAGACTTCACAATTGAC

TCCATAACTGTGAAGGCTCAGCAGACGACGTTTGCAACCGGAGTGGACTACATGGCCGAC

TACGACGAATGGCTGTTCATACAGAACGGTGGCAAGCCTGAATTGCCGGAGGAGCTAGAC

CCCGTGCCGCGGTACATCCGCAACTCCCGCGACCTTTCGAGAATGGCAGCAACCGACACC

ATCAACACGGAGGCGTACCGGGCCGCCTTGATTCTCATCGAGGAAGGAGCTATCAGCCGT

CCTGGCTTCAACGGCCCGTACGGGGAAAGCGGTCGTCAGGTCGGTTTCGTCAACTACGGC

GTGTCCCACGTTATGAGGCTTGTCGGAACTGGCGAGCTGGCGCAGAGGTCCTCATGGTAC

CAGAAGTGGAACGTGCATATGTTCGCCCGCCCGGAGGCCGTCGGCGGAACCATCCACAAT

GTCCTAAACGGCGATCTCGACGTAGAGTTCGCCGACTCGCTTCTCAACAACAGACTGTTG

CTAAGGAGGGTGGCGGACCGGAACAGGGAGATCACCGGGCagcgacgcaggcgcccgcgc

ACCTACCTTCTCCCCCAGAGCGTCAATGAGGGGTCGCCCACTCACCCATCGTACCCGTCT

GGGCACGCCGTCCAGAACGGTGCCTTCTCAACGGTACTCAAGGCTCTTGTCGGGCTGGAG

AGGGGGTCCGAGTGCTTCAATGACCCCGTGTTCCCCGACGATGAAGGGTTGACCCTTCTG

CCCTACACCGGCGATGAGTGCCTGACGTACGAGGGAGAAATCAACAAAATGGCCACCAAC

GTCGCTTTCGGCAGGAATATGATTGGTATTCACTGGAGGATGGACGGCCAGGAAGGATTG

AACCTCGGCGAGATGGTCGGCGTGCGGATTCTGCAGCAGGAGGCCGTGGCATTCCCGGAG

AACGAGCCCTACGAGTTCCGTCTCATGTCAGGCCAGACGATCAGGCTCGAAACCGACGGG

TCTTTCTTCATCGACAACAGGAGGTGCAGCGGGGAGGCGTTCATGGGGGCTGATCTGTGC

TAA

>g29105.t1

ATGAAGGCTGTCTTTGCTATCTGCGCTGCGATGCTGGCGGGCGCTCAGGCGTTCGTACCG

CCGACCGCAGGCTTCGGCAGCGCCCGCGCGGCCCTCCCGTCCTCCGTGGCCCGCACCGCC

TCCACCTCTACCCCCCAGATGGCGGCATCCAACTTCGGCGTCTTCGACAGCGCTCAGACC

GAGTTCGCGGAGGAGTTCCCCGAGTACTCCAAGTACGGCTGGGGCCCTACCGCCAAGGCG

GAGAGGTGGAACGGCCGCCACGCCATGTTCGGGTGGGCGGCGATCATCGCCACGGGGTAC

TGCCAGGCGCACAACCTCATCCCCAACGCCGATGTCGCTCTGGACCCCAAGGTCTGGGGA

ACGCTGGCCTACACCACGGGCACCGAGACCATCACGAACGAGAGGGCCATCATCATGATC

GGGCACGTGCACGCGCTGGCGGTGTCCGTCTGCGCGACGGTCGCGCCGCTGTCTTTCCAG

GACAAGCTGTTCCTGGACGCGGACGAGGGCGAGAAGGACGGACCGGCGCCGGGCCTCATC

CCGTCGTTCGTTCCCGGGCTGACCCCCGAGGCGGAGATCTTCAACGGCCGCATGGCCATG

ATGGGACTCGTGGTAACGGCTACCGTGGCGCTCACGACGGGACAGTCTTTCCTTGAGGTC

GTTAACGCGGGCCTCGGCGGTCTGCTCatgtaa

>g29166.t1

ATGGGCGGCACAGAGGTTGCTCTCACGAGCATCCGGATCCTCACCTACGCTGTGCTGGGC

TACGGTATGTTCTACCTGACCATGCACTTCAGCGACTGGTACGAGATCAACGTGTTGGAC

GACCAGCTGGAGTTCACGTTCGGCGATGACATCCGGATAAGGAGCTCCACCAACGAGCGC

TTCCTGTTCCTCGCGATGCCCATCGTGGTGGCCGCTGCGCTTGGCGGTCTGTACGAGGCA

GTCTGGAAGAGAAGGGAGATCATCGCCAGCAAGCTCGGCACCCGTGGGGAGAGCGAGCAG

CCCGATGAGGCGGAGCGTCCCGGTCTTTTGGCCCTCCTTCACGGGGTGATGCACTTCAAG

TTCAGGCCTCTGGGACAGTACGCTCCGTGGGTGACAGTGGGAGAGGCCATCGCCCTGTCC

ATCTGGTGTCTCGGCATGCTAGTCGAGATTTGGCAAGGTGTCGCGAGCAAGAACTACTAT

GAGCGGTACGCCCCGTGCGAAGACTACCCCACGAAATGGTGCAGCCTACGTTCGGGGGAA

GTGGGGCCAAGTTTTGACCAGTACCGGCTGAGGATGATCGCCATCTACATGGGATTCATA

AGCTCCTCCCACTTCGCCGTCATCCTGGTCCCGGTCTCCCGCGACAGCAAGATATGGTCC

GCCGTTGGGGTGCCCTTCGAGCGAGCCGTGCTCTACCACGCCGTCGCGGGGCACCTCGCC

TTCTCCTCGGTGTTCATCCACGGTTTCCTTTTCCTTGCCTACTGGGTCTGGACCGAGGGC

TGGCGCCATGCCGTGCGCGAGTCCATCCACGTCAAGGCGGGTGACGGGAGCGTCGACATC

CCGATGGGGTGGATGGCCGCCCTGTGCGCGCTGCCGATGTGGATCACCTCGATAAACTAC

GTGCGCCGCCGCTGGTACAGCCTGTTCAAGCTGAGCCACTGGCTCTTCATCGGCGTCTTC

GTCTTCGGGTCCATGCACTGGGCCCGTAATACGCTCTACTTCCTGGGTGGCTTGACGCTG

TACACCATGCACGTGCTGTCGCGGCTTGAGGCCTGGAAGAGGTGGCGCTACTGGAACCGG

TGGCTGAGCCCCGCCACCAAGGCCAGCCCCACCAGCCTCGTCAACGTGCTCACAACTGGG

GACTACACCCGCCTCGTCCTCCGCAACCCCAAGACCCTGTCTGCAAGGGGGGGCGCGTTC

GTCTACCTCAGCGTTCCGGCGGCGCTTGGCACCGACGAGGCCCACGCCATGTCCGTGGCC

CTGCGCGgcgcccccccgtcctccctgcCCGAGAAAACCCGAGGTTTTCCAGAGGAAGAA

GTGTTCACGGTGTACGTCAAGGAGCTTGGCCCGTGGACCAGAGCTCTTCGCCTCGCTAGC

GACGGCCCGGCGGCCACAGCCTCGCCCCAGTCGCTGCTGGTGGACGTGGACGGGTTCTAC

AACACAGTGGAGTCGCTCGGCGCGATGATGAAGGGGGGCGCGCCGCGCATCGTCGTGGTC

GCGGGAGGGTCCGGTTTGACGTCCATCATGGGATTCATTCAGGACTGGTGTGTTGCCGCC

GGCGAGGGGGTTGCCGTGCCCGAAGTGCATCTGGTTTGGTGCTGCCGATACATGGCCGAG

ATGGAGCTGGTGGGAGAGGCGATCCCTTCGATGCTTGCAACAGCGGGCGAGGCCAAGAAC

AGCCACTTCACCATGTCCCTCTACTGCAGCAAGCCCAACAGCAAGAGCGGGCCCCTGAGC

GTGACGTGGCCCCTGGACAACATGGCCTAcgccaacagcacaacagccacGCACGTCATC

GGCGAGACATCCATCGCGAACAGCCTCCACCACAGCGTCCGCGTCATCATCGCAGGTTTA

GCGGGGTACTCCGGGTACCTCCTGTCCGTCTACGAGCTAGAGCGTCGCAAATTCGACAAC

ACTTTCCACGAGGGTGGCATTCAACTTGTCCTTGTGGTAATCTGCATCATCAGTGTCCTC

CTGGTGTACGGGTGGATCTGCGCTCTCGTAGGGTGGATCCGGGGTCCGGGGCACTCGAAG

GTCGGCAGAAAGATGTCTACTGACGAATCTAAGGCCGATCTGGAGTCGTCTTCCATGGGC

GGTAGTACCACCTACGAGGTCAAGCACGGCCGCGTGCCCACCAGCGCGTTGTTCACCCAG

GAGTCCGATCTGGCCGAGAAGAACGGTGTGAACAGGGTCAAGGTCCTCACGAGCGGCCCC

AACTCTCTCGTGGACCAAGTTCTGTCGGACTCTCGCGGCATTAACTGGCAGCTGTTCGAG

TCGGAGGCCTTCTCCTTCGCGTTCTGA

>g29188.t1

ATGAAGGTTGCTTTCGTGGTCGCCTCCGGGGCGGCAATTGTTGGCACCAGCGATGCGTTC

TTGGGGCCAGCCATCGCAGGCGTCACCGCGCCGTctgccatgcagcagcagcacaggtcc

TCACGATCTCGGGCGATGTCGATGGCGGCAACGACGGAGAAGCCGGCTGTGAAGGCATCG

CCGGTTCCCGGCGGCGTGATGACGCTCTCCCGATACATGCTCGACCAGGCCAGGGTGAAC

ACGGACTACCAGGACCTTGAGCAACTGATGGGGTCCATCCAGTACGCGTGCAAGACCATC

GCCAACTTGGTGAGCCGGGCGGGCATCTCGGACCTCACTGGCCTCCAGGGGGACGGAGGT

TCAGTGAACATCCAGGGGGAGGAGCAGAAGAAGTTGGACGTGATCTCCAACGACGTGCTC

AAGAACGCCCTCCGATCGACGGGCAAGCTTGGCGTGATCGCCTCCGAGGAGGAAGACCAC

CCCGTGCTCGTGGAGGAGGCGTTCAACAGCAAGTTCGTGGCTGTGTTCGACCCCCTTGAC

GGTTCCTCCAACATCGACGCCGCTATCAGCACCGGCACCATCTTCGGCATCTTCGAGGAG

ACCGAGGAGTGTATCGTGGACGACGAGATCGACAtggacgaggccgcccaggtgtGCCTC

CTCAACACCCTCCAGCCCGGAGGGAGCCTTGTTGCGGCGGgtTACTGCATGTACTCGTCC

TCGACGATCCTGGTGTTCACCATGGGCAACGGCGTTAACGGCTTCACCCTCGACCCCCAG

ATCGGCGAGTTCGTCATGACCCACCCCAACATCCAGGTCCCCAAGCGGGGCAAGaTTTAC

TCGTTCAACGAGGCGAACGCCCCCGAATGGCCGGAAAACCTCCAGAACTACGTCCAGGCT

CTCAAGACGGGCACCGGCGAGACCGGGTCGAAGTACTCCTCCCGCTACATCGGGTCCATG

GTTGGTGACGTCCACAGGACGCTCTTGTACGGCGGAATCTTCGGTTACCCCGGAGACCTC

TCCAACCCCACCGGCAAGCTGAGGCTTCTGTACGAAGCCGCCCCGATGGCGTTCATCTGC

GAGCAGGCGGGCGGCATGGCGACGACGGGCGAGCAGCGCGTGATGGACATCAACCCGGAG

AAGGTCCACCAGCGCGTGCCGACCTACCTGGGGTCCGTAGACGACGTGACCGAGCTCTGC

AACGCATTGAAGAAGTAG

>g29696.t1

ATGAAGACCGCCGTACTTTCTCTGGTCGCTCTTACCTGCTCGTGCGCGGATGCGTTCACC

GTCTCCACCGCGTCGTTGAGGCCGCAAGCGAGCAcccgcgccaccgccgccgcgtcaTCG

ACCCCGCTCCGGATGGGCTTGCTGAGGGAGGCGGACTTCATCCCCTCGGAGGAGTGGCTC

GGGGAGTACATGGGGCAGCAGGGCCTGCGCTACGCGTTGAACAAGACGCCCGACGAGGTC

CAAGCGGAGGGGGACTACACGCTGCTGCAGAAGCTGTTCGGGCAGACCTCGAACAACAAG

GCCCGCATGGACTTGAAAGCGGAAGTCTTGGCCAAGGCCAACACCCCGACAAACCCTTCT

CTTTACAAGTCGTGGACGGCTAAGTACGGTTACGGGCGGTTCTTCCCCGTGTACGTGGAC

AAGTCCGGAAACCCCGACGGTGAACCCGCCGCGCCGGCAGCCAAGGCGATGGCGAAGAAG

TCGAAGGCCGCTCCCGCCCCGGCGGCTAAGAAGGGACCCATGGCCGCGGCTAAGAAGGCT

GCGCCCGCCGCGAAGAAGGGCCCGGTAGCGACAGCCAAGAAGGCTGCGCCTGCCGCTAAG

AAGGTGGTCGCGAAGCCGGCGCCTAAGGCTGCCCCGAAGAAGGCCGCCCCGAAGGCCCAG

GGCAACATCAACAACTTGCCCAAGGCACTCTAA

>g29699.t1

ATGAAGGTCTCCTGCGTTGTGGCGTTGGCTCTGGCGGCCGTGGCCGAAGGCTTCCTGACT

AGCTCCGTCGGTCTTCGCGCTGGAGTCAGCGGTTGTTGCGCGAGAGGAAACATGCGTATG

TCAGCCGTGGACGACGGGTTTGCGGCGGCGTTTTCCAAGGTGGAGCAGGCCCTCGAGCTG

GCCCCGTTGGACCACGCCGAGGCCATGGGGCTGGAATCGTGGGAGGTCGGGGCGAACAGC

GGGACCATCGCCGGCTACAAGGGGAGGAAGGTGGACTGGGTCTCTAGCTGCTCTTTCAAG

GAGGAGGACGGCAGCACCCGCCAGTGCCTCCGGGCATGGGTAATGCCGATGTTCGACGTG

CCCCACCTGTCCATCTCTCTGGGAAGCGGCCCTAACGGCCTGACGGCGGAGCTGGACCTG

GTGGCGAAGGACGACGTCATGTACGCGAAGGCTTACCGTGAGAGTTACTACGGTGGGGAG

ACGTCGGAATGGTGGGAGGCCGTAGTCGGAAACGAGCACGCCACCCCGACACAGGTATCC

TTTGACTTGAAGGAACGGGCGTTGGCTTCTCCCGTGCACGTATCGGTGACGATGCCCGAC

ACGGCAGAAAACGCCGCCTTGCTTGCGAAAGCCGCCGAGgacctcgccgcgcgctggctg

gGTGAGATGGAATCGATGAGTCTTCAAGAGGCGGCGAAGCAAGCAGCGGAGGCCGAGCTC

AAGCAGCGGGAAGCCGACAGGGAGTTCGAGGCGCAGCGGCAGGCGGAGGCTATATTACGC

GAGGAGGAGCGGTGGGTGTGGgcgatataa

>g29734.t1

ATGCAACCACAAGGGTCGAGCGTTCTCCGGCCGTACCAGGACGATGTGATCCAGCGAGCC

GTTGATCTCTACGGGCAGGGGACCCGTCGGCTGTTAGTACCGTTGGCAACGGGCCTCGGC

AAGACCGTAATCTTCACGCACCTGCCTCAGGCGTTTCCAGAGCTGTGCCGAAGAGGAATC

GTGGTGCTGGTCCATCGCAACGAGCTTGTGCAGCAGGCGGTGGCTTCGTTCTCGAAGTTT

TTGCCGCATTTCTCCGTCGGCGTGGAAAAGGGGGCGGAAAAGGGGCACCCCGGCCTTGAC

GTGGTGGTTGCATCGGTTCAGACGCTTGGAAGGCGCGGCTCCGGCACAGCGAGACTGTGG

CAGTATGCTTCCTACGGTGGGGTGGTCATCGTCGACGAAGCACACCACGTCAAGATGGGT

GGCATGTACGACACCGTGCTCAATGCGTTCGGGGTGGGGAGCGAGTGCAAGGGTGACACG

AGAGACGGCCGTCTCTTGGCCGGGTTTACCGCTACTCCTCGAAGAGCGGACGGGCTGTCC

CTACGGCCGTTCTTCGACTTCAGCGTAGGCGAACGAGACCTGTCCTGGGGCATTACCAGC

GGCTATCTTGTCGACATCCATGCTTACGCGGTGAAAACGGCTACCGACATTGCCGCTGTG

CCCGTCAGGATgcagGAATTTTCTACCGGTGCTTTGTCGGCCGCGGTGAACAACGACCTC

CGCAacgcggccgcggcccgtgcGATGCTGGATTATGGGGCGAAGGCTTCGGCGACTGGA

AAGGGAAAGAGAAATCCGAGGGGCATCGCTTTTTGCGCGGACGTTGCCCATGCGCACAGC

CTGGCTCGCGTCCTGAACGAGCATGGCATCACCGCGCTGGCGGTGGACGGGAAGATGAAG

AAGGAGAAGCGAGCGCGAGTCTTGGACGCTTTCCGCTCCGGAGAGGTTTCGACTCTGACC

AACTGCGGCGTGTTGACCGAAGGCTTCGACGCCCCCTTTTGCGACACTATTGTCATGTGT

CGCCCTACAAAGTCTAACCCTCTCTACATGCAGATGCTCGGGCGGGGCACTCGTCCGTGG

CTGCCGTCTACGccccaagGATTGGTGGGTGGCGCGCTCCCTTCGACAGCCAAGGAGAGA

AAACATTACATACAAGAGTCGCCCAAGCCCCGCATGCATGTGATTGATCTGGTCGACAAT

TGCGGCAAACACGAGGTGATGAATATTCCGGCGGCCCTTGGGCTTTCCTCGGGCTTTGGA

AGCGGTGCGGCGGGAGAGGCCTTCTCCGTGGAGCGTGTCGCATCCCTCGCTGCGGAAGCC

CGCACGCTGCTGGGCGAAGACCGGCTATCAGAGGGGGAAGACTCTCCCCTGAGGAGAGCT

AGGAACCCTAGAGACTTGGCTCTGTTGGTGAAGCGCTACCGCCTTCTTGAGGGAAAGAAC

ACCGCGCAAAGCCTGGTTTCGACACCAACCAGCATGCTAGAAGAGGGCGACGGCTGGGAC

GGCGAAAAACCGCTCGGGCCTTCCGGCAATTTGGCTATATCCACCCTTTCTGTCGGAGAG

CGGTTGTCGAAGGACGCTTTCTTGCGAGTAATCGTGGAAGCGATAATCACGCCCGAGGAA

GAGAGCGAGGACGATTTCGAGTCGGAAGATGACGACGGCcggtacgacgacgacgactgc

gATGGCGCGGTCGAAGACAATGCCAGCGCCGGCCTATCGCGCCACCCAAAGGTCAAGAAG

GCGGCACGGGGTTTCGGTCGCAGCCGGCAGAATGCCGCGCCACGTCGCCATCGTCAAGCG

ATGTCAGCTGTTACCGTCCACTTGCGCCACCAGCCATCGAAGGCGCTGTTTGTGTGGAGA

ACGGCGGGTGTGAAAGACACGTGCGGCGCGGAGTTGCACGAGGGGGAGGCGTATTCCCTG

GTAGCAACGGTGCAGAGCGTGGCAGACGTGGCAGCGGGGAAGGTAGGAGTGACGCGATGC

CGTCTCACTCCGATAACGGATGCTCGTGGTAcgcagtag

>g29758.t1

aTGCCCTCTTCCGCGACGATGTTTCGCTTGGCCACCGCGGCCCTCCTGGCCCTCTGCCCC

ACCCCGTTCTACAACGGCGTTCGCGcacaggaagaggaggagcccGAGCTCGACGACGAG

GTCAGGCCCGCTCCTCCCTCCATCGGCGCCGACGTGCCGCTGGCCTACTTCGGTCCCGCC

CCGTCGAGCGTCGACAAGCGCCTGGTCGGCCCCGTGCAGCTGCTCCGGTCAGGCGTGATC

GACGAGGACTTGGGTACCATCGAGCTGCCTCTCTACACCGGGTACTACACGGACGGTTCT

AGCCACTACTACATCCTCACCGACACCACCGACGAGTCCAACGCGGCCGCGCTCGGACTC

AACCACTCGCCCAAGCTCGCCTTCTCCGGCGGAGGAGCCACGACCGCCTCCCTCGACTTC

GAGAAGGTCATCCTCGTCAACCGCGAGGGCAAgGTGGACTTCTCTCCGGAAAACTTCGTT

GTCGCTGGCGATGAGCCCACTCCCTTCCCTCCGACCTCCTTCGGGAACGGCGCCGTGGGA

GACGAGCTATACAGCCCTCTCGTGATCGTGGACAACATCGGCGGGTCTCTGTGGAACGCG

CCCATCGTGGCCAGCAACGTCACCACGGAGTTCCTCAACCAGTTCTGCGACATGGACGTC

ATCCCGGAGGATATGGCGGAGGAAGCTTTCGAgtACCTGCACCCGAAGGTGGTCACCATC

TGCCCCCGCGACCAGACGGTCAAGATCAACCTCGTGCCCGGGTTCTCCTTCGCGAAGCCC

ATCCTGTACCTCACCATGGACTCTTCCGTGGACCTCGCGGCCGCGTTCGAGGACGTCACC

TTCGCGCCGAGGCTGTCCGGGGTCACGGTCGGCGGGGACGACAGCGCCTTCTCGGCGGTG

GAGCGCCTCTTCGCCACGATTAACGGGTACTCCAACTCGGACCTGCCCGCCAACGCGCCC

AACGGCACCGCCAACCACCCTCACCGCCAGGGGTTCTACAGCGTGCTGAGGGACGAGGCG

GCGCCGCTGAACGTGCTCGGCGGGATCCCGACCGTTGCGACGGACTACTCGCCCCTGTGG

GACCTCAACATCGGGGAGTGGACGCAGGAGGCCGTGGACGGGGGCATCCGCACGCGCCTG

TTCGACGAGTTCCAGATCTTGGGCCTGGTGCAGCAGGGGTGGATCACGGGGGCCGGCGGC

GCGCCGTACGCGTCGAGCGGCATCATCGTCAACTGTCCCATCGCTATGCGTTTcctgtag

>g30408.t1

ATGAGGGGTTTTGCCGCGTGGACATCAGGCCTGGCGGTTCTCGCAGTGACCGGAATCACA

CAAGGTTTCGTCATCCACGCCGCGCCTGCCGTGCGATCAGCTGCTAGTGCCACGGCGTGG

GGGGGGAGCGCCAGGCGGGCAAGACCAGCCGCGCGGCCTTTAGTTGGCATGCAGATGCAC

AAGGTCACCATCGAGCACGAGGGAAAGTCTACCGTTTTGGAGGTGGACGAAAACACGAGC

ATACTCGAGGCTGCCCTAGACAACGACATTGAGCTCCCCCACGACTGCAAGCTCGGGGTG

TGCCTGACCTGCCCCTCGCTTGTCGTCAGCGGGGACGTGGACCAGAGCGATGGGACCCTC

GATGACAGCGTCATGGAGCAGGGATATGCACTGACGTGCTGCTCGTATGCGAGGTCGGAC

GTGACTATCCGGTCCGTGGAGGAGGACCAACTTGTGGGCGCCCAATTCAGCGATAGAGAG

TAA

>g30420.t1

ATGAGTGCTGAAGAACCATTAGTGCTCCAGACTTCGGTGCCTCTGGAGTACCAGGACCAG

GATTCTTTCTTCGGTGGGGAGCCTCCTCTTAGCGAGGCcgtcggatacgTTGTCGTCCTT

ATCTTCGGGGTATTCTTCTCTTTCTTCACAACCTTCCTGGTGTGGCTTGACGCTAAGTTC

AATGGGGTCAACATCACCTCTGAGCacctctcggcgttgtgcagGTACGCGTCAGGGGCG

ACAATCCAGGTGCTCTTGTTCGGCATCCTCGCCATAGAGGTGAAGCGGAAAGCGCCCACC

TGCCACACCTTCCTGGAGATCATCCAGGCCCGCTGGGGCGACACCGCGCACaaggtcttc

ctcttcttcgcccTGCTCACCAACATCATCGTCACGTCCATGCTCCTGCTCGGCGGCGCC

GCCACCGTGAATGCTCTCACCGGCGTCGACCTCAACGTGGCCTCCTTCCTGATCCCGTGG

GGCGTGATCGCGTACACCATGCACGGGGGGCTGAAGGCGACGTTTTTGGCTTCGTACACC

CACACCACCGTCCTGTTCGTCGCCCTGCTCATTTTCGTGTTCGTCATCTACGCCGGAAAC

GACAAGTACATCGgcagCCCCGGCGCAATGTACGACAAGCTCCAGGCCCTCACCAAAATC

GACGACTGCTCCTACGGCTCGGAGGGACAGTTCACCCTCTCGCCCGGCTGTGGACCGGTG

AAGGGGAACGAGGAAGGGTCGTACCTCACCATGCTGTCTACCGAGGGCTTCGTCTTCGGC

ATCATCAACATCGTCGGCAACTTCGGCACCGTGTTCGTGGACCAGTCGTACTGGCAGTCC

GCCATCGCTGCCAAGCCGCAGTCGTCCCACAAGGGCTACCTGCTCGGCGGCATGTGCTGG

TTCACGATCCCCTTCGCCCTGGCCACCTCCCTCGGCCTCGCGGCCTTGGCGATGCAGCTC

CCCCTCACCGGCGACGAGGCCGCGGCGGGCCTCGTGCCCCCGGCGACGGCCCTCTTCGTG

CTCGGCAAGGGCGGGGCCACGCTGATCCTCATCATGCTCTTCATGGCGGTCACATCCACG

GGCTCGGCCGAGCTCATCGCCGTGTCGTCCCTGGTCTCCTACGACATCTACCGCGCGTAC

ATCAACCCTCAGGCGACGGGCGAGCAGATCCTCAAGGTCTCTCGGTACACCGTGTTTGTC

TTCGGCGCGCTGATGGGCGTGCTGTCCATAGGGCTCAACGAGATCGGGCTCAACCTGGGC

TGGGTGTACCTGTTCATGGGCATCGTCATCGGCTCCGCCGTGGTGCCCGTGTCGATGGTG

CTCACCTGGAAGAAGGCCACGGCCAAGGCAGCCATGGCCGGGGCCATCTCCGGGCAGATC

TGCGCTATCATCGCGTGGCTGgtGACTACCTCCGCTCTGTACGGAGACATCAACCTGGAC

AACACGGGGAAGAACTACCCGATGCTCACGGGGAACCTGGTGGCCATCTGCTTCTCGGGC

ATCGTGTGCATCGTCGTCTCCCTGGCCAACCCGGACGACTACGACTGGGAGTCCACCCGG

AACATCTCCATGGTCGAGACGTACGACAACGCGTGGCACACCGACGCCGACTACAACGAG

GCCGACCTGAGCAAGGCCAAGGCCTGGATCATGAAGATCGGCCTCGCGTTCACCCTGGTC

ATCGTGCTCGCGTGGCCCCTTCTGTCGCTCCCGGCGGGCGTCTTCTCGGAGGGCTACTTC

AACTTCTGGGTGGCCCTGTCCATCCTGTGGGGCATCGTGTCGACGCTGTTCGTCGTGTTC

CTGCCCCTCTGGGAGTCGAAGGATTCGATCATGCAGGTCTTCAACGGCATGTGCGGCGGC

GAGGGCATCCctgccgcggcggcggtggccgacAAGGCGGACCCCAAGGTCGCCAACGGG

AGCTACCCCCAGGCCCAGacgtga

>g30679.t1

ATGAACTCCATCAAGGGGGCTTTCGCTAGACATCCCGTGCTCTTCAACAGCATCGTGGGC

TACAGCGTGTTCGCTTCCGGGGATGTGATGGCCCAGCGGCTGGACCTGACGTCTGAGGCA

GGGGCATCCGCAGAGTGGAAATGGGACCACAAGCGGTCGCTGTCCATCGGGCTGCTCGGC

ATCGTGCAGAACGGCTTTCTGCTGAGGATCTGGTACCGCACCCTCGACAAGTTCGTTGCC

CCCAAGACCGATCTCATGAGCGTACTGAAGAAGATCGCGTGTGACGAGGCCGTGTTTGCC

CCGCAGCTGGCCTGCTCCTACCTCGCCACATCGGCCTACATCCAGAGCCCCGGTGATTGG

AACGCCGTTAGCGACAACGTCCAGAACAAGGTGGTGACAACGTGGCAGAACGACCTCAAG

CTCTGGCCGATGGCCAACCTGATTGGGTTCTCCCTCGTGCCGAGGCCTATGCGCCCCCTG

TACGCCAGCGGGGTGCAGTTGATCTGGCAGTGCTACCTGAGCAGCACGAGCTTCGCTGGG

TCGGCCGGGGACGAAGCTACTGGGGCTGCGGTGACGGCGGTTACCACTGCCATGCAGACG

GAGATCACCAATCCTCACCtgccctaa

>g30685.t1

GTAATCCTCCTGTCCTTCGAAGGCGACGACGGCGAAGCATCCAAGTTCCGAGGGTGGGTA

GCGGCGGGGGTAAGCGCCGTCCAACTCATCTTTGTCCCGGCTCTGAGCGGAGCGAGCGAC

ACCGTGGGTCGACGGGCGGTGATAGCGGGAGCGCTGGCCTTGCACGGGGCATGCGTGTTT

GCTTTCGCGACGTCGCCCAGCTCGGTCGTGTGGGCGACCGCGTGCGACCTGGTGACGAGC

CTCGGCATGGTGATCATCCCCCTCAGCCAGGCCATCATGATCGATATCTCCCCCGATGGA

GGTACGGGTGCGACGCACGGCCTGGGCATAGCCTTCGCCGCCTTCTCGTTAGCTACCAGC

GTCGGCGACATCGTCGGGGGATCTCTCAGCGAGCACCACCGAATGGAGGCGTGTCTCCTT

TGTGGGTCACTTTCCGTGGCCGCTCTCCTGTCGCTCGTGCTCTTCGGCTGGGAAGAGACC

GCACCGGAGGGGGTAGCGGAAAATAcaggcggcggcgaaggcgaaggcgaaggcgaaggc

ggCATAGAGCAGCTATTGGCAGACGCGACGGACGATAACGGAACGCGGAGGGGGGGACAA

GGAGGGGGGTCGGTAGGAAGGGGCAAGAGGAaaggggagaggaggaagagaaggaggccA

GCGCTATCGAATCCGCTCTCGATCATCAAAGTGATCTTTGGAAACAGGGTGCTGCTGCAG

GTGGCGTTTAGCTATTTCTTGTTCGTCGTGTCCCTCAACGTGTTCGCCGCCGGCTACAAC

TACCTCGACTTCCGCTTCCACTGGAGTCCTCTAGAGATTTCCTACTtcttcgccaccttc

aacgcCCTTATGGCCATCGCCGGGGGCTTGGGCATTCGCCTCATCGTTCCCAAGCGCCTC

TCCGAGGAGAACGGTGCGCTGTTCGGCATTTTGATACAGGGCTGCGCGTTCACCGTGTAC

GGTCTGTGCGTTCGCGGGTGGATGTTGTACCCCGCTCTGGCGTTTGGGGCGATCCAGAAC

ATCACCGAGCCTTGCCTCCAGGCCATAATGGCCACGTTCGTAACGGTCGATCGCCAGGGG

AGCCTACAGGGCTGCGCGTTCACCGTGTACGGTCTATGCGTTCGCGGGTGGATGTTGTAC

CCCGCCCTGGCGTTTGGGGCCATCCAGAACATCACCGAGCCTTGCCTCCAGGCCATCATG

GCCACGTTCGTAACGGTCGATCGCCAGGGAAGCCTACAGGGGGCAGTGATGAGCTTGCGC

GTCATCGGCGAGGGTGTCGCTGGTCCGCTGTTCACCCAGATATTCTCGGCGGCGTCGTCG

GTGGGGTTTGAGGAGGCGCCTTTCTTCGTGGCGGCGATTATCAGCGCTGTCGGCTTGACC

GTCGCTTGGCGGCCTCTGCGCAAGTTGGACAAGACGAGAAATACACCCGGTGGACGCCCA

GCCCTAGCAGGCAACGGAAGCCGCACGGACGAAGAGGGCCGCGACGCGACGGACGAAGGT

GCAAACGTCAGCAAGCCGggggacgatgacgacgacgacggcggcttCGACGGCATCGGT

GTGCTCAAGAGTGATCCGTCGAGGCAGGAGCAAGAAACGGgaggggcggcggagggggag

ggggaggtggcggGGGACGAGGACGAGCTGACACGGCCGTTGCTTGCGTCGCCGCCGGCC

CCGGCATGGGAGCGGTGCTAA

>g30857.t1

ATGAGGTGCGACTTCTGCGACGATTACGTGCAAACCCTGGAGACCGGTTTCCTCCCCGAC

ATGGTCACTTTCACTCCAGACGGAAGGCGTATCCTTACCGCCAACGAGGGAGAGCCGCTG

GACTACACCAAGCCAGAGAACGATCCTGTGGGCTCAGTGTCGATCTTCAAGCGTATGTGG

TCTACGGAGACGTACAAAGAATCTTGCGAGGTGGGCTTCGAAGATTTCGACGAACCATAC

AACACGAAGAAGCTGGTCAGGAACGGCTTGCGCGTTAGCGGCGTGGACTTCACTACCTTC

TCCATGGACATGGAGCCGGAGTACATCGCGGTCGGGAACTACAGCAATAACGCCTTCGTC

ACTCTGCAGGAAAACAATGGTGTGGTGAAGATCGACATAAAGGGCTGTAAGATCAAGAAC

ATCTTCCCGCTCGGCTACAAGGAGTGGGGCAATGGTGTGCAGTTCGACGCGTCAGACAAG

GACGGTGCCATCAACCTCCAAAACTGGCCGACAGTCAAGGGCATGTACATGCCCGACGCC

ATCGCAACCTTCAAGACAGGCCGAAAGCGttACTTCGTTACGGCCAACGAGGGCGACGGG

CGCGAGTACGGGGAGGAGGACACCCCGAGCTTCTACACCGACGAAATCCGCGTCGAAGAC

TTGGCCGTGGAACTCGGGCTGAACGCCACTTTGCCGCCTTACGACGAGACCAACCTCGGC

AGACTGAAGGTGACGACGGCGGCTCCCTTCGCTGGCGACATCAACGAGCTCTTCGCATTC

GGTGGCAGATCTATCTCCATATTCGAAAGCAAATCGGGCGAGCTTGTGTGGGACAGCGGT

GATTTCATCGCGAGGTACATCGCCGACCCGGCTAACGGGTTCAGCGAGCTCTTCAACTCC

CAGGGAGATGCCGGCTCGTTCGACGAGCGCAGCGATGACAAGGGAGCGGAGCCAGAGGGG

CTTGCGATAACCGAGATCGACGGAAGGTCGGCCGCCATAGAGGAGGACGGCGAGGAGGTG

GAGCTGTGGGAGGGGATACAGGAACACCCCGACGATGGGGAAGAAGGGGAGGAGGCTCCT

GACTGGGCGGAGGCGCCGGCAGAGCCCGGGAAGTCGTCGTACACGAACTCGAACGAGGTG

GATCTTGCCCCCGAGTCGGGTGCCATCATCCCCGCCGAAGACTCTCCCTCCGGAAAGGCG

CTGCTCGTCGCTGCCTACGAAGCCAGCAACACCCTCGCCGTCTTCGAGATTACCACCGGG

GGGCTATAA

>g30888.t1

ATGATGGCGACAGGCAGCAACTCGCACTCGGACAAGAACACGGCCACCGTCTCGCCGATG

GAGCACGACAACGACGAAGAAGCCAGCCCCGAGGCAACCAGGAAGCAAAAGCTCATCAAG

GCAGGGCTGCTGGCTGTGCTGGCGGCCATCGTCGTCTACGTTGTCCTCGACTACACCATT

CCCGGACTGGGCTTCGTCGCCGACATCCTCAAGAGCTTCCTGGAGTGGGTTGAGGACAAC

CCCGCGCTGggcgccatcgccttcgccGCTGTGTACGTGTTCACCACGGTTTGTTTCATT

CCTGGCTCGCTGCTTACGCTCGGCTCCGGGCTGGTGTTCGGCCGCGCGCTGGGCACTGGG

TTGGGTGTGCTGGTGGGGTCCGTCGCTGTGCTGGCTGGCGCCACGATCGGTGCCATCTTG

GCGTTCCTGTTGGGCCGATTCGTGCTACGGGAGCAGGCCCAGGGGCTGTTCAACAAGTTC

AAGATCCTCAAGGCCGTGGACAGGGCCATCGAGACGCAGGGGCTGAAGCTGGTGCTGCTT

CTCCGTCTGTCTCCCGTGGTGCCGTTCAGCGTCTTCAACTATGTCATGGGTGTTACCGCG

GTCCACTTCCGCGACTACGCGCTCGGCTGCCTCGGGATGATCCCCGGCACCGTAGCCTAC

GTGTTCATCGGCACCACGGCGTCAAGCCTCTTGGGAGACGACTCGGAGGAGGAGTCGGAG

GACGACGGCAGCAGCTCCAGCGTGCagctcatcgtcatcatcgtgggGGCGATCGCGACG

ATCATCGCGGTCGTGCTGATCAGCATCTACGCGAAGCGCGCGCTCAACAAGGTGCTGGAG

GAGGAcatggaggcggaggcggaggaggagcggAGGGCCGGGGGCGGGGTGCAGATGGTG

GACGGGGATAAGATGACCGCCACGGGTGATGTTGAGGCCCAGGACGGGACACGGCCAGCC

GTGGTGGAACACGCTGTTAGAATGAACGCGTAG

>g30972.t1

ATGAAGGTTTCAGCTCTTGCGCTTGTCGCGACGACTGCGACAGCCACTTCGGCATCCCAG

CAGCCTTGCTTTGTTGCGCCAGTTGGAGCTCGATGCCACTTCAGGCAATCTGCGCCGACT

TCTGCTGTAGCGGCGAGGAGAACAAGCGGCTTGCAGATGAAGCTGTGGGACTTCGGGCTC

AACCGTGAGGCCATCACGGGACCGTCTCAGGTGGACGCCAACTGGAAGGCCCAGCAAGAG

ATGCTCAAGCGTAGAAACAACAAGGATACCATGGACAAGTACAAGTCAGGGGTGAACGAC

AAGAGGCAGCAGGCCGATGCCAAGGCGAACAAGTGGGCGTGGCAAAACAAGAGGAGCTCG

GAGGACCCTCTGGTGGAGTGGAAGAAGATGAAGGCGGCCGGAAAGGTCGACGACATCTAT

GACGGCGATGAGCCGGAGGGAGGCATCCCCATCCCCATGGCGAGCTTCGGTGTGGGCGGA

AGCTTCGGTGTCGGCGGCAAGTGGGACGAGGGTGGCCGGTTCGACCTCCGCCTCCCGTAC

GCCGACCAGGGCTACGTTGACCCGGACGAGGCCCCCATGAAGAAAATCAGCAGCATTTTC

GGGTGGAATAAGAAGAAGGCGGAGGAACCCGAGCCCGTGGCAACGAAATCGAAGAAAATG

TCTGCCAAAAAGGTTGTGCCTAAGAAGGGAGCAAAGGCGGCAAAGGTGGCTGAGGTCAAG

CCCAAGAAGAAGGGATGGTGGTAG

>g31022.t1

ATGCGTCTTTCAGCAGGAATCGCAGTTGGGGTGGGGTATGCTTCGCTCGCGCAGGTATCT

TCGTTTGCGTTCGTCCCGTCAGCGACATCAGTGAGCACATCTTCTGCAGGACGCGccgct

acacagcagcagcagGCAAGGGGACACCGCAACTTATGCTCTAGGCCGGGAGGGCTGTCG

GTGGCAATGTCCGCGGAAGATGACGAAAGCTtggggggcgagggcgagggggtggaggcg

gaCGACACGTCGAGCCAGGTGGCGACAAAGCGCAAGAAGGGAAGCGAGTTGGACGACATT

ATGAGCGAGGTTGCCGGCCGGGCGAACGACGAGCCGGTCGCCGTCCCGCGAGTGATGCCG

GTAACGACTGAGCCTATTATCGTGCCACGCGGGGCTGGCATCGCGCCTATGTCACCTGGC

CCCATCAAGGCGGATCCCATCCCCGGCATGGACGCCCTCGACGTTCCGGGCAAGATCGTC

TTAGATCCGGAGACCCTGGCCTCGCAGCAAAAGGGGCTGGAGAAGATCGCGCAGCAGCTG

AGGAGGGAGCGCCTAGACAAGGAGGCGGTAGACGCGATCACCTTTGGCTTCTGCCCGAGG

GCAGAACTGTGGAATGGAAGGGCCGCCATGTTCGGCGTGACTGTGGGCATGCTCACCGAG

CTGTGGACGGGGCAAAGCATCCCGCAACAGGTAGAAACCTTCGCGCAGCTTCTCGGGTTG

CTGCCCTTGGACTACGACACGTACTTCCAATAA

>g31039.t1

ATGGAGAAAAGCAAGGGGGAGTTTGAAGGAAGCATCGAGAGAGCACAACAGGAAAAAGTC

CGTGAGAATGTCGTGGTCAGGGGCAATAAGGAAGATGCCGTCAGCGAGCTGGAGGCGGGT

CGGCGGACGATCGTCAGAGACCCCGACGATCccgtgcagcaggcagacattctGGACAAC

CTGATGATCTCGAGGGAAGAGGACATCGAGGAGGAGCTGAGGGCGGCGTACATGCCGGTG

AGGGCCGCGGCAGCGAGCGCGGCGGTAACCACCGATGGAGACCGCTCATTGGACGATGGC

GGCGTCCAAggaggggggccgggggggaaaGGCAAGTTCGATGGCGGTTACGCAACGACC

ATGCAAGGGGCGGAGGCTTTTTTCAACAAGCCGTCGCCGTTTCAGAAGGCGGCTGAGAAG

GAGCGCGAAGCGGCTGAGGCTGCCTTGGAGAGGGATGACCGCGTCGCAAACGCCATCGAC

GGCGCAGAAACTGCCGTAGAGACTACGGGAGAGGTTGTTGAGGATCCCTTGACAGATGAA

GCATCCCAACGGATAACGGGAAGCGTCGGCGGGCGCTGGGAGAAACCAGAGGAAGGGGTA

GAGCTTGACAAACACAAGCCTGGAGTGGGGTCATGGGGGGTGTTCGAGCGGCCCAAGGAC

ATGAGCAAGGCTTACGGGGGAGGGCGAGACCCAACGTTGAAGAGGATGGACCCTGAGGAG

ACTCGAAGGCGCGACGAAGAAACGAAGGCCATTTTGAGGAGCTACCGCGGATCTGTGGGA

GAGGACTTGcagagggagaagaagaaggaggtggaGATTCAGGCGGCACTGGATCAGTCG

AAGCGAGCCATGAGGTTCGGGGATACTTTTGGCGCAGTGTCTGCCCTGGAGTCCGTGAAG

CAGGAATGCTCCACCCACGGACCGTTAGGGGCTGTGGTTTTCCTTGAGCTTGCCATGGCG

TTGGAAGCTACGGGGAGATCGTACCAGGCCCAGGACATCTACAAGGTCCTTCGGCGGTCT

AAGAACCGCGAGGTGCGGGCGCAGGCGAAGAGGCTGAACGAGGGGCTGGAAGCGATGGAC

ATGCTCAAGTTCGGCCGCCCTGGGGAGGTTAACGAGCAGTCGGAGATTAGCAAAATGTGG

ACCACCAGCTTCACCCCGGTGTCGGGCGACACGGAGAAAAAGTACAGCTCCGTGTTTTTC

GAGAAGGATGCCGATGGGAGACCGAAGGGGGACAACGTGTTCGCAGTCGATGACGCCAAG

CAAGTGCTGATGTTCGCGTCCAAGTACCCGGGAGGCATATCGTCGAAGCGCATCATAAAC

GCCTTCGAATTCTTGAGGAAACGGTCGGAGGAGGCCGCTCTAGCCGCACGGAGGGAAGCC

TCGGCGCTGCGAAAACAAGCCGAAGCGGCAGAACCACAGCAGACCGAGGAGGACCCTGCC

TCTGgcgaagggaaggagggagaaaatcCTGTTAAAGATTCTATAAATTTGGAGCGGAAA

CCGGCTCCGTGGGTGAGAAATGCCGAGAGCACACTTCCCCAGGGCGGTGCTGGACTTGTG

TCGCTGATGGGAGACATGTCGAGCCTGGAGCAGAGGATACAGGGAGGCTGGAATATGGTC

ATCAAGGCCGGCGGAAGCAAAGTGGAAGTGTTTGAGCGGGACAGCTCGCTGATTCTCTCC

CCTGGTGGGTCGGTTAAGGCTGTAGAGCCGGCCGGGCCTCTCTCGGTCTCAAAGGAAGGG

CAGTGGGAGTTCGATGCGGATGACCGAGTGGTGAAGCTTGATTACGCCAGCTCCAAGTGG

GTCGGACTCCTGGACGTGGGTGGTGGTCAGGAGGGCTTGGGCGTCTTAGCTCTTGACGAC

CAATTGATGGTTGCCTTTGAATCGGCCCGGGCTGGAGCTGGAGCGCAAGGGGGGTCGGGG

ACGGCTAAGATGGGCGCCGTCAAGTACAGGTTGCTGGTACGGCAATGA

>g31120.t1

TGCACAACTGCGTGCTTTGAGCCGTCCCTCGACTACTGCGTGGTCAAGATGCCTCGCTGG

GACCTGAAGAAGTTCTCCAAGGTGTCGACGAAGCTGGGCAGCTCCATGACTAGCGTCGGG

GAGGTCATGTCCATTGGAAGAACCTTCGAGGAGGCTATCCAGAAGGCTGTCAGGATGGTC

AACCCTGACTTGGACGGTCTGGAAGGCAAGTGGGACAAGGATTCCAACCTCTCGAAGGAC

GATCAACTCAAGATACCGACTGACTCGAGGCTTTACGCCGTGCAGGCTGCGCTGGAGGAC

GGCCTCAGTGTGGACCATGTCCACAACCTGTCTCGAATCGACCGCTGGTTCTTGTCCAAG

CTCAAGAACATTGCGGTCATGAAGAAGGCGGCGAAGCTGACGGGTGGCCTTGAGAGCCTG

GGGCGTCCCGGACTGTTGTCGCTCAAGACCTCCGGTTTCAGCGACCGCCAGATTGCTCGC

TACACGAGCACCTCTGAGAACCAGGTACGCCGTCGAAGGCAGGCGGCTGGGGTGAGGCCG

TTCGTGAAGCAGGTGGACACTTTAGCCGCCGAGTTCCCCGCGAGCACGAACTACCTGTAC

ATGACGTACGCGGGGAACGAGGACGACATCGAGAGGGAGAACATGGGCATCATGGTCCTG

GGATGCGGGGCGTACTGCATCGGCTCTAGCGTGGAGTTCGACTGGTGCGCGGTGAGCTGT

GTGAGGCAGCTGCGCTCGCTGGGGTACAAGGCCATCGTCGTCAACTACAACCCGGAGACg

GTGAGCACGGACTACGACGAGTCTGACCGCCTGTACTTCGAAGAGCTGTCCTTCGAGCGC

GTATTGGACATCTACGAGTGGGAGATggcgggaggggtggtggtCAGCGTGGGTGGCCAG

ATCCCCAACAACCTCGCCATGCCATTGCACCAGGCCGGAGTGAACATCCTCGGCACTCGC

CCCGAGGACATCGACCGCGCGGAGGACCGCGACAAGTTTAGCGCCATGCTTGACTCCATC

GGCGTCGATCAGCCGAAGTGGGCTCTCCTCAGCACGCCCAAGGAGGCCTTGGCTTTCGCC

GACCGTGTGGGTTTCCCCGTCCTGGTCAGGCCTTCTTTTGTCTTGTCGGGGGCCGCCATG

GCCGTGGCCAGCAACCACACCGAGCTCGAGGTGATGCCCGCTATGATACTATTGTAG

>g31336.t1

ATGGTGGCCGAGAGCTGGAGATGGCAGGGTGGAACATCGGGCATGATACCGGGTACTGCG

GCCGCGATGGGAAAGGACGGGTACGTTTTACTGGCAGGAACGGCACAAGACAAACAGAAA

ACGTTCGCAGCCGTCAAGCTCGATGGAGATGGGACCTTGCTGtgggaatggcagGGTGGA

CCCCGAGGGGACGCTACGGTTCATACCGCGGCGTTCACAGATGATGGATCTGTGATATTG

GCGGGATCAACAGCCAGTGTTTGGGGCGAAGACAACATCGGTCCGGGTGACTTCGCTGCA

TCCAAACTAGATGCGGACGGAACCTTGGTCTGGACGTGGCAGGACGGAACCCCAGAACTT

GACTGtatttctgctgctgtcgtcggagAAGACGAGTCGGTAGTCTTGGCCGGGTACTCG

TCCGGAAATTGGAACGGTATTGTGATGGGAGGTGCCGACTGTGCCGCCGTTAAGCTTGAC

GCCGATGGGAAGGAACTGTGGAGGTGGCAGAGTGAAACCGTAGGTTTCGATGTTTGCACG

GCCGCGGCTATGACCCCAGATGGATCAATAGTGCTGGCGGGAATGGCCAAGGGCGTATGG

AGCGGCACACATGTCGGTGAGTATTCCGAGTTTGCTGCGGTGAAGCTGGATGCCAACGGC

AACGAGATCTGGAGGTGGCAGGATGGCCCAGCAGGTGGATGCGTTTTGAGAGACGCAGCT

GGGGGAGATGGCGAATCTGTTGTTCTGGTTGGAACGGCCGACGGAGTATGGGGAACACGG

AGCGACATCAGCCACGGCGTTATTGCAGTCAAGCTGGATGTAAAGGGGATTGAGGTCTGG

CGCTGGCAGGCTACGTCTGCTGAAGCCGTATCTGTCAGTCCTGGAGCTATTGCGGTGGGG

CACAACGGATTAGTTGTCGTGACGGGATTGATGGCAGGTTCCTGGAGAAATGACACGGTT

GGTGGGTCCGACTTCCCGGCTGTGATGCTGGAAGTGGGTACGTCGTTCGCGGTTCCATCT

CGTCCTGGACTAAACTCCGGTGCAGAGTCACTCAGCACTCCGGAGCCAACACCCACCGAC

TACCAAATCACGTACGCGCGTACCCTCGTGGTGGCCAGGAATGCTCTCCGTGTTGTGGAA

GGAGCGATATCAAAATGGTCATCGGCACCGACATCAACGACACAAGGACCACCAGTCTCC

TCCGCCACGGTCTCACCAATATACGAGCTATCCCTGGACGAAGATACGTTCATCATACTG

GCTCCAATGGTCGCCGATCTATCTTCCGCACCTACACTCAACCAAGCACGGGCCGCCATG

TTTACGCCGGGTCCCACCCCAACATCAAACCCGGCGCCAACATCACCGGCAACAGCAACA

CGAGCTCACGATGACATTCGTTTGCCAGTTCCCGGATCCCACTTGGACGCAGATGCGCTC

TTCACTCAAGCCCGATTGCTCATGGATTCAGCTACCACGCCTACGCCCACCGTTGGATCC

ACTTTTGCAGTATTtccggcagcagcaacgcaagCTCCGAGCTCCACGAATTCACCAGTC

GCCACACCTGAACCAACGATTCCGAGCTCCACGTTTTCACCTCTTCCGACAGGAGCAACG

CAAGCTCCGAGCTCCGCGGTTGCACCAGTTTCCACACCCGAGCCAACGATTACGACATCC

ACTTTTGCACCACTTCCGGTAGGAGCAACGCGAGCTCCGGGCTCCACGCTTTTACCCGCT

TTCACACCGGAACCGACGGTTCCGATGTCCACGTTCGCTCCACTTCCGGTAGGAGCAACG

CGAGCTCCGAGCCTCACGCTTTTACCCGTTTCCACACCGGAACTGACGGTTCCGATGTCC

ACTTTTGCTCCACTTCCGGCATCCGCAACGCGAGCTCCGGGCTCCACGCTTCCACCAGTT

TCCACATTCGAACCGACGGTTCCGATATCCACCTTCCCTCCAGTGGCAACAATGACACCA

ACCCCAGGATTGCAGGACCCAACATCGGGAAGCTCGTTTTCGCCGACAGCAATAGGCGCA

GTGTCCGCTGCAGCGGCCCTTGCGGTGCTTGCGGCGGGTGTGTGCCTGCGCAGAAATAGG

AACTCTAAGAAGAAAGGCAACGCGCAGATGGTCCCCGATTCGGGAAACACCACCCCCGGC

AATGCTTCCAACGGGATATGGAAATATATTTCCCACTCCTTAGCCTCTGGCCTTTCCAAA

GGGAACACGGGCCTTCCCTTGTATACCCAGAACTCTTCTGACAGAAGGGATGATTCAGCG

GCGTCAGGTGGAGAGACTAGATCTTCGCCAAGCAATGACATTCCGGACAGCAGATCCGAG

CCCCCTCCGGGCTTCCACAATGCAGCGGTCGTCGATGCCCTCCCTGTCCTTCCGCGGGCC

GAGAAAGGAACCGTGCCACTCAACAGGGGACTCCCGTTAGTGGTCGGCCTTACGGAGACC

GCATCAGATAACGAGGTTGCGAAAGCTGTGCTGGAAGCGGCGCGCAACCTGGCGACTAGG

TCCCACTTTCCCGGCGTCAGCGAAGCAGCAGCGCTTGTGTCAATCTTAGTGAACCTGGTC

TCGGATGATCATGCCTGCTTCGCAGAGGTGGAGTCGAGGGTAAAACGTTGCCGCCTCGTG

ATTATGCTTCTTCAGCGAGCGTCCACTGTACTTGGAAAGGGCAACGACATCGATGGGGTC

ATGGAGCGGATGCTGATCGAAGACGTGCAAACGGCCGTCGGGAACATGGTGGAACTCATC

AAAATATACCGGAGCAAGGGCAGACTATCACAGGTTATTGTCTCGACGATGTTCAGGCGA

CGCATGGAGGAGACAGAGGCGGTCATCGACCGAGCCATATCTGATTTGAAACTGGGTTTG

CACGTGCACGCCGAGGTCAAGCAGGAAGAAGACCGCGAGTATGTCCGTAGCGGGACCAAA

TACGAGGGTACGTCAGACAAGACTTTAGCAGAGTCGATGGCATCCGCCCGTCGTCTACGA

CGCCAGCGAAACTTGAACCAGATCGAGATCCCTGAGGAGCACGTGACAATATCCAAGGAG

CTCCTGGGGAAGGGCGGTTGCGGCGCGGTGTACATCGCCGACTACAACGGACGAAATGCC

GCCGCTAAGGTGATAAGCATCGACATCGCCCTTTGTGACCTAAGCCGCCGACACGGCCAC

AACTTCAGAAGAGGCGGGGGCACCGATGACACCGAGGCGAGCCGCCAGTGCACGTCCTTT

CTTTTGGAGCTGCAAACGATGATTCGGCTCCGAAGTCCGCATACGGTGAACGTCTACGGG

GCTATCACATCACGCAAAGACCGGCTCGTTCTCGTCATGGAGCTGCTCACGGGCGGTGAC

CTGAGCTCGTTTTTAAGACACAACGAAGACCGGCTTCCGCAGGCGCGGGCCAGGGAAATC

ATAGAGGACGTTTGCGCCGGTATGGCGTTCTTGCACAGTAAGAACGCCGTTCACGGTGAC

CTGAAGTCCCCAAACATACTGCTCGATGGAGAAGGGAGAGCTAAGATCGCGGACTTTGGG

ACCTCCAGATGGGCGCAGCACGGCGACACTACGGGTCTCGCCACGTACAAAACTAGCGCC

GGAAACGTCATGCAGATGATGAGCTTCGCGTGGGCTGCTCCCGAGGTGCTTGAGTCCAAG

GGAAGCTCTTACGCCAGCGACGTGTACAGCTTCGGTATCATAACGTGGGAAGTGCTTTCC

GGGAAGGTTCCTTGGGCCGACGAAGTCCTTCCTCGCGATATATACCGACGGGTGGTCTTC

CACGGGGCTCGCCCTCAGATTCCTGCAGATGCTCCTGCCGAGCTAGCAAGGATCATGCAT

GAATGCTGGGCTGGACCACCCAAGGAACGGCCCGCCTCGAGCGAGATACTGGATAGACTT

AAGTCTCACAGGACCGAAGGGTAG

>g31507.t1

ATGCGTGCGATGGCCTGGTTTGCGAAGAGTGCTGtcctcgtgtcgtgcatggcttgGACT

TCGGTGGTCCACGTCGCTTCGGCCTCGGGAGCATCGGGAAAAGGTCAGAACAATTATCCT

TCCCTGAGGCTGATTGAAGATACTATTTTGACCACCGCAAAGCTTGGTTTCAGAGGCCGC

GACCGAGCGTTCGAAGCGGCCACAAGGTGGGACCAAGAGAGGCTCCGGGCCGGTCAACAC

GGCTCGCGCGCGCCGCACCTGGTTTGCGCAGAGTACGGCCACGGTCGTAAGGTCGCCTCC

GGCCTCCAAACATTCCTCTCCCCCGACGCCGTGAAGCCTGTACACCACTCCAGCGAACAT

GGCGCATGCTTCCTCGTCACAGCTTCGGAGGCCCAAGCCGTCGAGCTTTCTGCGGGGCGG

GCTGGGTGTGACTTGGTGAGCGTTGGTCAATTTCCCTCTGCGCTGAAAATCGCTCCTGAC

GTTCTCGaacacggcagcagtgtgggAGCACGGGAGGGGAGCGGTGGGTCCGGCCGGCTG

ATCACGACTCACGGGTCCAAGTTGCGCATGGACAACGTTCAAGGTCTCATCGTGGAGCTT

ACGCCCGGCACATTGCCCGCGCACTCTTCGGAGGCTGAGGCATTCATCGGGGACTTAGGG

GAAGATCTCATGTCCAACTCGGTGGACCTTCACTCCGGTAACTTCTGGTCGGATCCCGCC

ATGTTGGGGGGTGAACACCTCGCTATCCCGGAAGGCGCTTTGCGTGGGCGGGAGTGGATC

AGAGCTGCGACAGTCGTCCACGAGCTGAGCGCGGCGGCAGATACTACTCCTGGCGACATT

TGTTCGTGGGACAGTGTTGCCATGCACCATGCTGCCGACGATGTACTGCTTGTCTCAGGC

TTGGACCACCTCCTGTAcgatgggaggggggtggggggtcaggGAGAGGAAGCCGAAGAG

CTGCacgtggcgtgcttcatgggtCTCGTTTCCGTCTTGGCGGGGAGGCTCGAGGTGTTG

CGAGTCGCGCCGTGGCACGCGAAGGCTCTTCAGAACGTGGCCGCTCGTGCGAACATCCAG

ACTGCTACGGCGACGTCCACTCCTCTCACAGATGCAGGGCTCGACGGCACGGGCGAGGTT

ATCCAGATCTTAGACACTGGGCTGGACGAGACGTCGTGCTATTTCGAACACGGTGATGGG

CTGGAGGTGACGCACGGGTACTACTTCGAAAGGTTTGGCATGGTGTACGCTGACAACACG

ATCGACTCCACCTCTGCCACAACCACCCTAAAGCCGTACTACATCTTCGAAGGGGGAGAT

TTCACATTCAACAATGACAGGCGCAAGATCGTCCAGTACATCAATATGGTCAAACCCGAC

ACTGAATCGTCCGCTTCGGTATCGACAACCAGCCAAGGAGGCAGGGTGTACTGGTTTTCG

GCTGACCCCTTTGACCAAGATTACGTGGCCGGGCACGGCACCCACACTGCAGGGTCAGCG

GCGGGGAAAACGTTGACCGTCCCCGCGACGCCAGTAAATTGCAGCGACACGTACGTTTTA

AGCTGCGCGGGGGGTTGTATTGATGGCAACTCGAGCAGCTCCGACGACGACTTAGTGTCG

TCGTACCACCAGATTTACAGAACAACTGACATCGACCGGATCTGCCCGATGTTCGGCTGC

GACGAGACGTACACGGAAGTGTGCCTGGGGGACGAAGTAAACGACACCCTATCAGATCAT

GGAGGCATGGCCCAAGGCGCCAAGCTAACTGTCTTCGATATTTATTTCGGACCTTACGTA

CTAAGCGATTACGCCGGCAACGGACTGTGGGAGGCGTGCATGGACGCTGGCTGCAAGATT

CACTCCAGCTCATTTGGCGGCGATGGCATGTGCACCCTCACTTCCATGGAGGTTGAGTAC

GACAACTTCATGTATGAGAACCCGGAGAATCTGCTGATATTCGCCGCTAGCAACTACGGC

GATGTTGACGATGGACGCACAGTTTGTACCATGGGGAGCCCAGGCATCGGGAAGAATGTT

CTTACAGTCGGGGCGACTTCGTCCGGGGAAGCCCGATTGACTACCACATCGGAAAATGGT

ACTGCGGCGGACAGCACTAACGGATCCGGTGACATCGATACCGTGGCGTTTTTTAGCTCC

TACGGGCCTACCCAGGATGGTCGCATCAAGCCAGACGTCGTCGCACCAGGCGATATGATA

TATTCCGCGGCCAGCGACGGTACAGACGAGCGTTCCTGCAGGCTGTACGCGTACAGAGGG

ACGTCCATGTCGTGTCCAATCGCGGCAGGCGCGTCGGCAATGATTCGGCAGTACTTTGTC

GACTCAAGCTTCTACGCCGCGGACGTGTCGGCAAGAGGTTTCTGTGACCAGGGGTTCTTG

TGCGAgggattttccccttcctcgGCAACCGTAAAGGCGTTATTGATCAACAGCGCGAAC

CTTATGAACGGGAGCTCGGAACCAGACGGATTCCGCGGCTTCGGGCGGATCCACCTTGAA

CAGGGGATGCCGCTGGACGGGCAGGGAAGCCTGGTCCTTTTCGTGGCCGACGCTGCCAAC

ACCTCCATACCAGAGCTCACAAAACAGAAATACCTGTTCAACGTAGACAGCGGAGCCGGC

TTGGATTTCCGCGTTACGCTATCGTGGATAGATCCCGGTGCCACCTCGCTCTCTGTCAAA

CAGCTGGTCCATGACCTAAACCTGGCCGTGTTCTCACCCAGCGGAACTAGGTACACGATG

TGGAAATCGGGTAAGGCTGATAACGCAAATGTGAACGAGAGGGTGATTGTGGACGCCGGT

GATGTCGAAAGCGGGACGTGGTCGGTGTGGGTGGCAGCCAAAGCTCTCACTACCGACGTC

CAGAGCTATTCGCTGGTTGTCAACGGGGCCATCAGTCCAGTGACACAAGGTTAG

>g31662.t1

ATGGAGGCACATACCATGCGCTCGGCATGGATGGTGTCCCTGCTCGCGCTGAGCACGTGC

GGGATCGTCCGCTATGCGGCGGCTTTCGGGCTAGGGGTCCGGCCTGCCGCACagctgttg

cagcagcacgagcggGCGTCGTGTTCGGCTGCGCAGACGCGCATGTCGGAGGAGACTGAA

CAAGAAGGAGCTATGGGCAGAGGGGCATTCCTGCGCAATTCCGCCGTTTTTCTCGCCGCG

GGCGGTTTGGCGACAGTGGCCAGCCCAGGCCCCGCGAGCGCTATCGGAGACCTGTTTGAG

TTTAAGGACCAAGCGCGCTTCGCGCAGCACACCACGATACAAGTGCCAGACATGGCTGCC

GCGCTCAAGTTTTACACCCAGGGTTTCGGCATGAAGGTGCTTCGCACGCGCGCGGGGCCT

CAGTTCAATACCACCGTGGTTGGGTTCGGCCCCGAGGCGCTTCAGGTGCCGCCAACCTTC

TTGTTTGGCGTCAGCTCTATGAATGCCTACGGCGGCCACTTCACGCTCGAGCTCAACGCG

CAGAAAGAGgtcggggcgggaggggaggaagacgtcgattttttttacgACCCGGGAAAC

GGCGTGCAGTACGTGCAGGTGGCGGTTGACTCGTACCGGATATCTCAAGTGATCAAGTCG

GGGGGAATCATCGAGTCTGGCTACGGGCACCTCCAGGTGCTCGCGCCGGGGGGTCTACGA

TTCAAGCTCATGATGGGCGATCGAAGAGACCCGCCGATGTTCGTCGCGGTGAAGGTAAAG

GACATCAAGCAGTCGATAAAGTGGTACACCGACGTTGCGGGCATGACCAAGTTCCCGTTC

CCGAAAGCCCGGGCGCCGGGTTCGCCGTTCGAGCCCGAGCAACCGAAAGACAGCGTCTTC

ATGGCTTACGAGGGGGAGGCGTTTGGGGTGGTGCTGGTCCCAGCCGCCAAAGGCGAAACC

TTGAATCCAGGCAGCGTCCTATCCCTCACTGTCCTGGCTGAAGACGTCGACAAGGTTGCG

GAGGACCTCGGGGGAAGTGTAGTCGTCGATAGGGCTAGGGGGGGGACGCGGTCAACGGCG

GTCACCGATCCGGATGGGTACCCCGTAAAGTTTGTCGAGTACAGTGACTGGCAGAAGGAG

CTTCCCAGTTTTTAG

>g31914.t1

ATGGTGAACTCTCTCGCTGTTGCGGCTGCCTTCTGCGCTTCGGCGTCTGCTTTCATGGCG

CCCACGCCGCTCGCGCGCACGGCTGCTCCTCAGCAGAGTGGGatGACCATGCAGGCAGCG

AAGTCCAAGTCTCTGCCGTTCATGCCGCAGCCCGCTGCGCTTGACGGCACGATGGCCGGC

GACGTCGGATTCGACCCCATCGGGTTCTCGAGCTTCATCCCCCTCGACTTCCTGCGAGAG

GCGGAGCTCAAGCACGGCCGCATCTGCCAGCTGGCCGTTGTCGGCTTCGCTTCGACCGAC

TTGGGCCTCCACCTCCCCGGTgccatgcacgacGTATCGTCGATCGCCGCGCACGATGCC

GCTGTGACCTCCGGAGCGATGCCGCAGATCCTGCTGTGGGTGTCGGCGTTCGAGGCGATC

TCGACCGTCGCAACGGTCCAGATGCTCGAGGGATCCGGCCGCGCGCCGGGAGACTTCGGC

TTCGACCCCGATGGGCTCTACAGCAAGCCCAACAAGGAGGCGAAGAGGGCGAGCATGGAG

CTCAAGGAGATCACCCACTGCCGTCTTGCGATGTTGGCGTTCTCCGGCATGGTAACCCAG

GCCGTACTCACCAACAGCGGCTTCCCGTACACCGGTTAA

>g31921.t1

ATGCGCTCCTCTTTCTGCGTCCTGGCGTGTCTGATCGTCGGGCGCGTGGCGAGCCACCCT

CTCTGCTTCATCGACGATAAGCCGACGGACTTCGACCAGGAGCTGACCTTTTGCCCGGAG

GCGCAGGACGGCGCGTGCTGCACCGACATCGAGGAGGACAACGTGTCGGCGGCCGTGGAC

GCAGTGGGCGAACTTACCACGGAGTGCCGCGAGCTGTATAAGAAGGTTGTGTGCGGTGTA

TGCCACTCCTACAGCGCCCACCTCTACGAGCGGCTGGGAGCCGAGCTGGGGGCTCTGGAC

GGGATGACGATGAAGCAGGACTTCTGCGACGAGCTGGTGGACGCGTGTGCGGATCAGATT

CCGGGCATCGCGCGAACGTACCCCGGAGGCCTCAGCTACTGCGAGAAGCACGTCGGCTCC

ACCGGCGATCAGTTCTGGTCTTTCCCTTACGAAGAACCCGAGATTTTCGAGCCGGGGCTG

ACCTTTGTGTTCCCGGACctggacaacgacgacgacttcCCGCGCAACACGATCAGCATG

CGCCAGACCCCCGACGGCGAGTCGTTCTGGCTGATGGGCCAGCAGGGGGAGGTCAAGATA

GTGCAGGCCGACGAGCTGGGCGAGATCAGCCAAGTCGTCGACATCTCCAACAACGGAGGC

CTCCTGGACATCGCGTTCGACCCCAACTGGGGCGTGAGTGGTTTCCCCGACATCTTCTAC

CTCAGCTACACCTGCCAGCTTAACGATGGGGAGAACCCGAGAAACCGAATCTCCAAATTC

GAGTACTTCCCGGGAGACCCGTCCGCCACCCTCGATTCCGAGGAGGTTCTGCTCACCACC

GCTCCGAAGTTCAAGCATCCACTCCGCTGGCTGagCTCCTCCCACGACCTCTACTGGACC

ACAGGAGACGGCGGACCGCAGACCGACACCTTCAACGCCGGACAGGACACGACGAACATG

CTGGGCTCCATGATGCGCATTACCGTCCCCTCCGACGGCACCGGCTACACCATCCCTTCG

GGAAACCTTGGATcgctccCCGATGTCTTGCCCGAGATCTGCGCGAGCGGGCTCCGCAAC

CCCTTCCGGTGCTCTTTCGACCGCGCTACCGACGTGCTCTACTGCGGCGACGTCGGACAC

ACTAACGTGGAAGAGATCGACATCATCGAGTGTGGTAATAACTATGGTTGGTCGAGGTTC

GAGGGCAGCCGTTGCCAAGAAGCTCAGGAAGACCGCGACGGAGACTGCCAGGGGGCCGAT

CGGAGCGGTTTCGAGTGgccgtacttcgagtactgccACCCCGACTACTTCTCCGAGGGA

GACGAGGACCAGTTCACGGGCAACCAGAACATCTGCGGAGACCGATTCATCACCGGCCAC

GCCGTCATCGGTAAGGGGATGATTTGTGATTGGAACATCTACTACCTCGTCGAAGTGGAT

GGGGAGCTAGTCCTGGGAACCATCGTGAGCGATTCGAGCGTGGCCATCATCTCCTTCTCG

GAAGACATCAACGGCGAGCTGATCATGATCACGAGAAACTTCCAGATCTACCACATGCCC

TGCGGTGACCTGTGCGCCTCCACCTGTCTCCCCCAGAGCGAGGACAACGTCGTCGTCGCG

GACCAAGGATGCTTCAACGACGACTCGGGGGACCGCGTGTTGTCTCTCGCCGAGAGCGAC

TGTGGCGAGGGCGAGAGGGCGATGAACGCTCAGATCTGCGCCTCGTACTGCGACACGCTG

GGTGCCAAGTACGCCGGAGTGCAATTCTCCTTCGAGGTTTACGAGATTTCGTCCACCACC

CGGCCGCCGGTCACGCCTGCGCCTTTCGTCATGACTGGAGACACGCCGGCTCCCACTCTC

GCCGAGGCAGAGACCCCCGCCCCTGTTGTGGTTGGCCCGGGAACCACGCCTGCACCCATG

GACCCGAGCATCACGCCTGCACCCGTGGACCCGGGGACCACGCCTGCACCCATGGAGGTC

GTCTCCGGTCCCGGAACCTTGATAGGCTGTTTCACGGACACGAACGCGGCGCGCATCATG

ACCAAGACTGCCACGCAGTCCCCGATGGGCTCGGAGGTCTGCCAAGCGATCTGCTCCGAC

TCCTTGTTCTACGGTACTCAGTTCTCCAACGAGTGCTGGTGCGGAGGACAGAGGACGGAC

TACGACGTCCACGGCCCGAGCACGGGCTGCACCTCCCCCTGCGCCGGCAACACCGGCGAG

ATCTGCGGCGGTTCCTTCGCCATGACCGTCTACGAGAACAACGGCGACGCCGGCGAGCCG

GTCACCCCGTCTCCCGTGGTGGACGCTACGCCGGCGCCCGTCGCCGTCCCTACGCTTGCG

CCGGTTGTGGACTCGACGCCGGCGCCCGTCGCCGTTCCTACGCCTGCGCCGGTTGTAGGC

TCGACGCCGGCGCCGATCGCCGTCGCCGTCCCGACGCCCGCGCCGGTCTCCGTTCTGACC

CCTGCGCCTGTCGCCGTTGTGGACGAGCCCACGCCTTCTCCGATCAGGATCCTCACCTCT

TCCAACTCTATGGAGACCCCGGCCCCGGTTGACGTGGCCTCGCCGGCGGACGAGGACTCG

TTCCTGGGGTGCTTCGCCGACCTGAAGGAGAGCAGGATCATGGCCAACGGGCCGAACTCT

GCTACCCCCATGAGCGCAGAGATCTGTGCGGACTTCTGCGGTACTGTCATCTACGGAACC

CAATTCGGGCAGGAGTGCTGGTGCGGCGATGAGAACACAGACTACGACGCGAACGGCCTC

GCCACCTGCGACTTCGCCTGCTCCGGCAACGCCGGCGAGATCTGCGGGGGAAGGAACGCC

ATGAGCGTCTACTCCGGCGGGAGCGGCACCGCGCCGAACCCCCAGCCGCCGACCCCCGAC

GCGCCGACGGCCACATTCATCGGCTGCTTCGCGGACGTGATGTCTGACCGGATCATGATG

GACCAGATCTCCTCTGCCCTCATGACTACCGAGgCTTGCGCGGTGCTCTGCGACGGGAAC

CCCTTCTTCGGCACCCAGTACGGCCTAGAGTGCTGGTGCGGAGCCGACTCCGATTACGAC

AGACACGGGCCCGCCGTGTGCGACTTCGACTGCACTGGCGACTCAAGCGAGACGTGCGGG

GGCAGGAACGCCATGAGCGTTTACTCCAGCTAG

>g31936.t1

ATGTCTCGACTCCTGGTGCTGGGCATGTTGCCCCTGGCGGCGTTATCGGCGAGCAAGGGC

GCTATGGGCGCGGACGCGTTGAGCGTAGCTACTAAGGCGAAACCCAGTCACGGAATAAAA

CCGGCGAGCTTCGCCCCGCGAAACAAGGACGAGCAGAACAACGGCGGCCAGCGTCTCCGC

GGAGTCGGGTGGTGGAGCGCCGCTAACGCCCAAAATGAGGACACGGGTCTGCTTCAAGCG

CGCGACCTTCAAGCGACGGGCTGTGGCGACGGCATCGCCGGCGTCCGGTCCTCGGGCGGC

GACGGCTTCATCTGCTGCGTCGAAGAGTGCGGGGTCTGCGGAGGGGAAGGGTGCTCCTCT

GCCGCAGGGGATGGCTTCGACGGCAGCCACTGCTGCGTATCGTCGATCGCCTCCTCTGGA

AGGCTCTGCTCCGAGACGGGCGAGGCGCCTTGCATCATGGAAGAGGGTCTGCTTCAAGCG

CGCGACCTTCAAGCGACGGGCTGTGGCGACGGCATCGCCGGCGTCCGGTCCTCGGGCGGC

GACGGCTTCATCTGCTGCGTCGAAGAGTGCGGGGTCTGCGGAGGGGAAGGGTGCTCCTCT

GCCGCAGGGGATGGCTTCGACGGCAGCCACTGCTGCGTATCGTCGATCGCCTCCTCTGGA

AGGCTCTGCTCCGAGACGGGCGAGGCGCCTTGCATCATGGAAGAGGCCGAAGGATGCGGC

AATGGTATCGCCGGCGTTCGGTCCGAGGATGGTACCGGTGACGTCTGCTGTTCGGAAGAG

TGCGGGGTGTGCGGCGGGGCGGGATGCGCTTCCGCGGCCGGCGAGGGATTCGACAACACC

CACTGCTGCTTGTCGGCTATCCTCGCGGCGGGCCAGACGTGCGACGAGACCGGGGCTGCC

CCGTGCCTGCTCGGTGGTGTGCCGGGAGACAAGGATGGCGATGGCGGTATCGACGGCTTC

GCCTTCCGCGGCTGCTACCTCGTCGAAGGCTCCAACCCGTTCAGCGAGATCACCGAGGAT

GGAGCCATGACGCCTGAGCTATGCTTCGACACCTGCGCCCGTAAGTCGTCCCTGTACATG

GGCCTCCTCAAGGGCAACGAGTGCGGTTGCGGCGAGGACCCCAGCTTCCTTgccaccgac

caggcggacggTGTCTGCGACGAGCCCTGCCCCGGAGACGAGGTCCAGACGTGCGGAGGC

GCCCTGGCGTACGACCTGTTCGAGCTGATGGACGATGACACCACCGATGACAACGTTGAC

GAAGACTCCACGTGCGACGGTGGCATTCCGGGGGTCCAGTCCGCGAACGGGAACGTTTGC

TGCCAGACGGCGTGCGGTATCTGCGACGGTTCATCGTGCGTCGGCTTCGGGGATTGCTGC

GAAGAGAAGATCCAGGGCTCTGGCGTGATGTGCTCTGATTCGGGAAGCGCCCCCTGCATA

CTTGACACCGATACGGACGACGCTGAACGTACCAGCCCAGACGGCAACGACATGACCGGG

TTCTTGCACGTTGGGTGCTACGCGGAGACCAGCTTCGACCCCTTCACGTTCGTGACTCAG

ATCAACTTCATGACTATCAGGCTGTGCAGCGAGAGGTGTGGCTCGATGGGTGCCGACTAC

GCCGGTCTGATGGACGGCGACGTGTGCGGCTGCGGCAGCGAGGCTGGCTACATCGAGGCG

GATAAGGAGGACGGCGAGTGCCTCGTCGAGTGCGCTGGAGACGACGGCCGCACCGAGATC

TGCGGCGGCGAAACCTCGTTCGTcctgatccagatcatctactcgGGCATGGCCATCGTC

ACCAACGCCGGCAACGCTATCGCTGCGCCTACTCCCACTCCTTCACCCGAGCTCATCGCC

CAGCTTAACTCCGACGCCGCGGGTGCCGTGATCGCTGCCCCAGCCGATGCCCCGGCCGGC

TCCGACGCTTCCGTCCTGACCGCATACACGCACCTGGGGTGCTTCGGCTTCGAGGTGGAG

AACAACCCGTTCTTTTCGCTCGTGCAGATGGATGGCATGACCTACCACACGTGTAGCGAG

GCCTGTGGCGCGCTTGGCGCCGTGTACATCGGCGCCTTCGACGGCGACATGTGCGGCTGC

GGAAGCAAGGAGGGCTACCTCCTCGAGCAGAAGCCGGCGGGGACGTGCGACGCGCTTTGC

GTGGGAGAAGCGACGGGAGACGCGGAAACGTGCGGAGGGTCTGTCTCCTTCGACCTGCTG

TTGATCAACTACCTGGCTACCGAGGATGACTACTTCgGCCCCGACTCCGCCACGATCACC

CCCGGCATTTCTACTTCGGGTGGGCTTGAAGAGGGCAGCGTTCCCCAGTCGGACGGTGAC

ACTACCGACGACGTCGACTCGACCACCGACTTCGAGACGGGGCAGGTGCAGACCGTCGTG

GCTCCCATGGCCGGCTACGAGTACACCGGGTGCTTCGAGATGGAGCCGACCCTCGACCCT

TTCTCGAGCCTCGTCCAGATGGACAGCATGACCATTGACACGTGCACCGCGTTCTGCGGA

GCCTCGGGCGCGACGTACGCTGCTGTGATGAACGGCGACATGTGCGGATGCGGCAGCGAC

GATGGCTACCTCGCCTCTGACAAGGAGGCCGGCCTGTGCCTCGTCGAGTGTGCGGGGGAC

GGTGCGCAGGTCGAGATGTGCGGGGGCTCCTCGTCGTTCGGTCTGTTCGAGATCATCTAC

GTCGCGGAAGACGGCACGAGTGACGCCAGCGACTCCTCGGCTGCGCCCGCATCGGCATAT

GGACTCGTCTTCGCTAACGAGGAGATGACCGGCTACATCTACGAGGGCTGCTACCAGATC

GATGCCTCCTTCGACCCCTTCTCGTCGCTCTCCCAGATCGACGCCATGACTCCCAGGTCT

TGCAACGAAATGTGCGGCTCCCTGGGTGCTGACTACGCTGGCGCCATGGACGGAGACCTG

TGCGGCTGCGGCAACGCTGACAACTACCTCGAGGCGGAGAAGGAGGACGGCGAGTGCTTC

ATGGAGTGCACTGGGGACGACGGCCGCACGGAGATGTGCGGAGGCTCCGCCGCCTTCGAC

CTCTACCGTATTTTCTACAGCGAAAGCGATGACGCCGCTATGCCTGCGGCAGCGCCCGCA

GTATTCAGCGGCATGCCGCGGTATGTCGCGGAGTGCCCCGGGGTTACCATCTGCCGTGGG

ACGTACCGCGGTGGTGCCCATGGTAAGATCCGCGGTAACGTGCATCGCAAACCCCTCGGC

AAGACCCACGGAAAGACCCACGACATGACCCGCGGAAATACCTGA

>g32329.t1

atgTCGGGAACAGAAGCCACGCCTTTGATACGAGGGCGTGCCATAGGCTCCAGCCCGCGA

GCACCCTCGGAGAAGGGGCAGGAGGGTGGTGAGCGTGGCCAGGGGTCTTTGCAGTCATCG

CAGGCGCAGACGCATCAACCTAGGAGTTGGACCTCGAATCGACTGTTTTGGACGTTGCCG

GGCTTCGGGTTGGCAGTAGCCGCGGGATTCATCTCCGCAACGCAGACGATCGAGTTCTTG

CGACAGCCttccctgcagcagcagcagcagcagcctcagcaaggagcagcagcagcagca

ggagaaggagtGGTTTCGAGCGTCGGCGACCTGGCTGCTAGGACCGTCCAGGAAAGCAGT

GGGCCGTCGGCTCAGCTCGACATATTCTCTGGTGCCGAGGGGCTTGTTAGCAATGCGCAA

CAAgaacaactacgagtacagggCAGAATTTCCAGCCCTACAACAGATGCAgattctact

gctgctactgaggTGAACAGGCCTGATAGCCCTGACAGCAGTCGGCACGGATTAGAAAGC

GAGGGCCTAGCGGCGGACGGGGTCGTCGATGCTGGGgttggagggagaggagccgacGGG

AGCGAGGCGATGCGCTCGGACGGACAGGGGACGCGATCTGGGGGGGGCTGGCGATCGAAT

GGCATCAGCAAGAATTCAGGGGCGGGTAGCGACCGAAAGCCGAacgtgtttttcattttg

atcgATGACATGGGCCACGGGGATATCGGATACCAGTCCACGGATCTGTCCTCGATGACG

CCCAACCTGGACGCCTTGATGTCGGGGGGAAGAAAGCTGAGCAACTACTACAGCATGTCG

CTCTGCACGCCGGCGAGGGCAAGCCTTATGACGGGACGCTACCCGATCCGCTACGGGATG

ACTTACGGCGTTATCGTACCGGGGGCACCATGGGGCCTTCCCCTCTCGGAAAAGGTGCTG

CCCCAGTACATGAACGACGCCGGTTACGAATCGCACATGGTTGGGAAGTGGCACCTTGGC

AGTTACAACGACGAGTCGCTCCCATCTCAGCGGGGTTTCACCTCCTATTTGGGATACCTC

AACGGGGAGGACACGTTCTACACACACAAGaaCCTGGAGTCGATCCTTGACGGCGAGGCT

TTCTACGATTTCGGGTACGGAAACGAGACGGGCTACTACGACGTCACGAGGATGGAGGAA

GGCGAGCCTTGCACCGGCGTCGGAAGCTTGCCCGGAGCGTTGTGGGAAgacgtggaggag

gaggaggagggtgaccCGGCTGGAGTTTGCTACACGGGCACCTACGCCACGGATAGTTTC

GTTAGGCGCGCTCGCaagGTGATCAAGGAGAAGACACCTTGGGACGAGGAGCCTCTCTTC

CTCTACCTGGCCCATCAGTCGGTACACGCTCCTATCGGCCCCGCACCGCAAGACGGGTTC

ACGGAGGAAGAGCAAGATCTACTGGACGCCGTGCACGaaggcactactcgtactgacgCA

GTTTTGCTGTACTTGGACAAGAGGATAGGGGAGCTAATGGACTTTCTCGAGGACGAGGGG

TGGCTGGAAAATTCTGTGATCGTGGTGGCGAGCGACAACGGAGGCTGTCCTTTCGCGGGA

GGCTGCAACTATCCGCTCAAGGGCGTCAAGCAGTCCGTCTTCGAGGGGGGGACGAAGGTG

CCGGGTATGATCTGGTCGAAGAGCCACCTCCCGGAGTCTGTTTGGGGCACGTCGTACGAC

AACATGATGCACGTCACCGACTGGTTGCCCACCCTGGTGTCGGCAGCGGGGGGCGAGGTA

ACCGGCAGcgcTGGTACTCTGGATGGTGTCGACCACTGGGCCTCGCTGACAGGCGTTGCC

gtggaggggacaggggacagcggAGACGGTCCCCGCACAGAAATACTTTACAACTGGGAC

CCGTACCTGCTTTCGTCGAGGGATGAGCTGACGGAAAACCTTGACCTCGTGCAGGGGGCC

TTCAGGCAGGGAAGCTGGAAGCTTTTGGTTAACGTTTGGTGCTCGGGGTACTACTCCTTC

GACAAAACCATTATCCACGACGATCCAAATACCGACCCAGACGTGACGTGCAGCACAACA

TGCCAGTGCGAAGACTGCATGGACATATGCCAGGGGGACGAGAGCTTCGCGTTCGGCGAC

GTCCTCGTCAATCTGGACGACGACCCGACGGAGGCACACAACGTCCTCGAACAGTACCCT

GAGATCGCGGCCCGCTTGAGAGCACGCGCTGCGGAAGTGGCGTTCTCCGAGTACAGGAGC

TCGGTCTACTCGCCGGTTAATACCGACAGTTACTTTTTCTGGAAGGAAAAGGGATGGTGG

ATGGCCCCGTGGTTCGGAAAGCTCCCCAACTGA

>g32346.t1

ATGGTGCGGCAGTTCAAGCATGACCATGCTGTCTGCCTGCCCGGCGCTCGTGTCATTGCT

ACACCTCCCTACGTGAACACGCTGCACTCCGGGCGCATCTTGGGCCCCAGTTTCTCCGTG

CGGTCGATGGATGCTGTTGCCTCTGTGGAAGGAGGGTATACAGGGGTTTTGGAGACGACT

TGCGGGGCATTGATAATTGCGGTGGGAGACACCTTGTACGAGCTGAGCGGCGTGGACTTC

ATCGAGAGAGAGATCGACGTGATCGATATGTCGCCAAACGAATGCACCGGGGTGGAAATT

GATGAGCCCGCTGACTGCTACAGCGGCGGATGGTCCTCGAGTTTGGACGGCAGCATCGAC

CTGACTGTCACCATTTGCTACTGCAAGCACGTGGACGGCAGTGCTACCGCGTACAGGTCC

AACGTGGGGACACAAGTGAAGGGAGCGAGCTCCATCAAAGTGCCCAGCGACGAGCTACAC

ATCTGCTTCGACTTCGAGTACGTGTCCCAGATGGCTGCTTTCGAGGTGGCGGGCTCGACG

CACTACTACGACTTCGAGCTTGCGATTCCTGGAGATGACACCTCCGCCGACTTATCCATC

GAGTCTGCCCCCCCATCATCAGCTGGGGCGTTTATGTGCGGGGAAACGGAGCCTACGCCT

TCCCCTGTAGAACCGCCAACTGCGGAGCCCACCTCGCCCCCAGTTCCGGAACCTACTCCA

GCCCCTTTGAAGCCCACCGCACCACCCGTAGACCCCACCACTCCTGCTCCCACAGTCGCC

ACCCCTGCCCCGGTGATGCCCGAACCTACTCCACCCCCGCAGCCCCCCCCAACGCCGGCC

CCTGCACCACCTCCAACCCCCAGCCCAGTAGCACCAGAACCAGAGCCGACTTCTTCTCCC

GTTGTGCCAGAACCAGATCCAACATCAGCCCCAGTAGCAGACCCAACCTCAGCGCCAGTG

AAGCCTGTACCGGTGCCAGAGTTAACGCCGGCCCCTGTAGCCCCAACCCCGGAACCAACA

GCCGAGCCGACCCCTCGGCCGGTAACGCCGAAGCCGACCGTGGCGCCTGTCTCTCCCAGC

CCGACCACGGCCCCCATGGCGCCCCCCGTTGCGAGCGCTTCTCCGGCCCCATCTGTggcc

agcaacaccagcagtccGGCTGCCACGGTGGCTACGCTGGGCCCTATGGCGTTACCGACG

GCCGCCCCCGTCGCTGTGCCCTTTACCGATGCCCCGATGGAGGCGGAGGCGAGGCCTACA

TTAGGACCTTCTGCCGCGGAGAATGCGACTACAAGTCCGGCGCCGAGCACCACCGTGGGC

TCGTCTTCGTCAGACGGGGAAGGCCAGGAGTGCGAGCTTCCGTTGGACGCCCCGTGCACT

GAAAATCCTTGGACTGGGAACCTGAACATGGGGATCGGGACGTTCTACGACCCTGTTTGC

AATACCACTTCCACTGAGGAAGAGAAAGAGCCGGGGTGCCAAGGCGACTCGGAAGTCTGC

CGGCTTTGCCTGTTCAACACGGTGCTTTTCAGGGAGAGCAATCCGGGCGAGGATCTGCCG

AACTACACCGTTTGTCCGTGCTGCGTGTTGAGTGCCTACAAACAAGAAACAGCTGGCGAT

GCGGACAGCTCGATATGCGGGACCGCGTCCCCCGCTCCCAGCGTAGGGACGACTGCGCCA

TCACCAGGGGCGCTTGGCTTCGCCGACCGAGTTGCTCAAACACAGGACTTTCAAGACAGG

CGGCTAAGCACTGGGGGGAGCAACGGAGTCGCTGTAGCGGCGCTGGGGCTACTCGCTGTG

CTCACTTTCTTACCCGTTTGCTCCAAGTAG
